# Supplementary material for: Sensortoolkit—A Python Library for Standardizing the Ingestion, Analysis, and Reporting of Air Sensor Data for Performance Evaluation
Source: Sensors (Basel). 2025 Sep 10;25(18):5645. doi: 10.3390/s25185645 (PMC12473223; doi:10.3390/s25185645)
Supplement: Supplementary file 1 [file sensors-25-05645-s001.zip › TableS1_sensortoolkitDataFormattingSchemeParameters.pdf]

Table S1. Sensortoolkit Data Formatting Scheme Parameters

Parameter Classification	Parameter Name	SDFS Label	Units	Notes
PM	PM_1 (particulate matter < 1 micrometer in aerosol diameter)	PM1	μg m^{-3}	
	$PM_{2.5}$ (particulate matter < 2.5 micrometers in aerosol diameter)	PM25	μg m^{-3}	Criteria Pollutant
	PM_{10} (particulate matter < 10 micrometers in aerosol diameter)	PM10	μg m^{-3}	Criteria Pollutant
Gases	O_3 (Ozone)	O3	ppbv	Criteria Pollutant
	NO (Nitric Oxide)	NO	ppbv	
	NO_2 (Nitrogen Dioxide)	NO2	ppbv	Criteria Pollutant
	NO_x (Nitrogen Oxides)	NOx	ppbv	
	SO_2 (Sulfur Dioxide)	SO2	ppbv	Criteria Pollutant
	SO_x (Sulfur Dioxides)	SOx	ppbv	
	CO (Carbon Monoxide)	CO	ppbv	Criteria Pollutant
Met	Relative Humidity	RH	Percent	
	Temperature	Temp	Degrees Celsius	
	Dew point[1]	DP	Degrees Celsius	
	Wind Speed	WS	m/s	
	Wind Direction	WD	Radians	
	Pressure	Press	hPa	
Ancillary	Parameter Units	[param name]_Unit		Parameter Metadata
	Parameter Quality Control Code	[param name]_QC	n/a	Parameter Metadata
Eval	Parameter data normalized by corresponding reference	[param name]_Normalized	n/a	Calculated during sensor evaluation analysis

[1]If internal Temp and RH measured, but not DP, DP is calculated via the `Dewpoint()` module and is labeled `'DP_calculated'`
