# Supplementary material for: Sensortoolkit—A Python Library for Standardizing the Ingestion, Analysis, and Reporting of Air Sensor Data for Performance Evaluation
Source: Sensors (Basel). 2025 Sep 10;25(18):5645. doi: 10.3390/s25185645 (PMC12473223; doi:10.3390/s25185645)
Supplement: Supplementary file 1 [file sensors-25-05645-s001.zip › Supplemental1_CaseStudy_PM25_Testing_Report.pdf]

Testing Report - $PM_{2.5}$ Base Testing

PurpleAir

This report reflects out-of-the-box performance

PurpleAir
U.S. Environmental Protection Agency
Office of Research and Development
Clements.Andrea@epa.gov
919-541-1364
May 2019

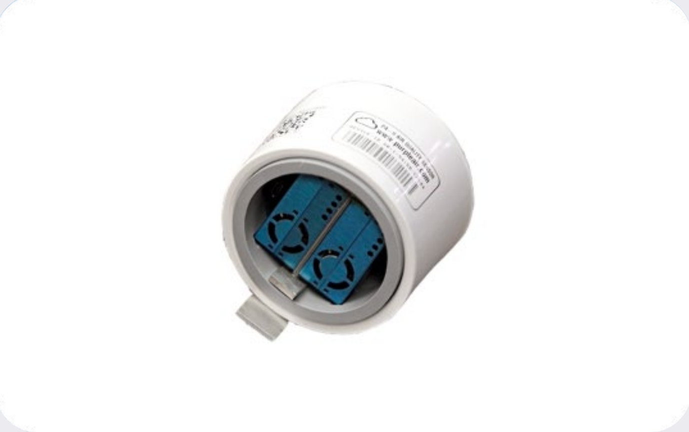

Deployment Details

Testing Organization and Site Information	
Testing organization (Name, Organization, Contact website / email / phone number)	U.S. Environmental Protection Agency - Office of Research and Development<br>Air Sensor Toolbox | U.S. EPA Website<br>Clements.Andrea@epa.gov<br>919-541-1364
Testing location (City, State, Latitude and Longitude)	Durango Complex<br>Phoenix, AZ<br>33, 112
AQS site ID	04-013-9812
Sampling timeframe (MM-DD-YY)	05-01-19 to 05-30-19
Sensor data source	Manual download from SD card
Reference data source	Download from Maricopa County

Sensor Information			
Manufacturer, model	PurpleAir		
Device firmware version	v3.00		
Sampling time interval	1-minute, 20-seconds		
Sensor serial numbers	PA01	PA03	PA05
Issues encountered during deployment?	☐	No Issues	

FRM/FEM Information	
Manufacturer, model, designation	Teledyne Advanced Pollution Instrumentation T640x
Sampling time interval	1 minute
Date of calibration	As required by 40 CFR Part 58 and the Air Monitoring Network Plan maintained by MCAQD
Date of flowrate verification check	Monthly as required by 40 CFR Part 58 Appendix A
Description, date(s) of maintenance activities	Span Check May 2019

Time Series Plots

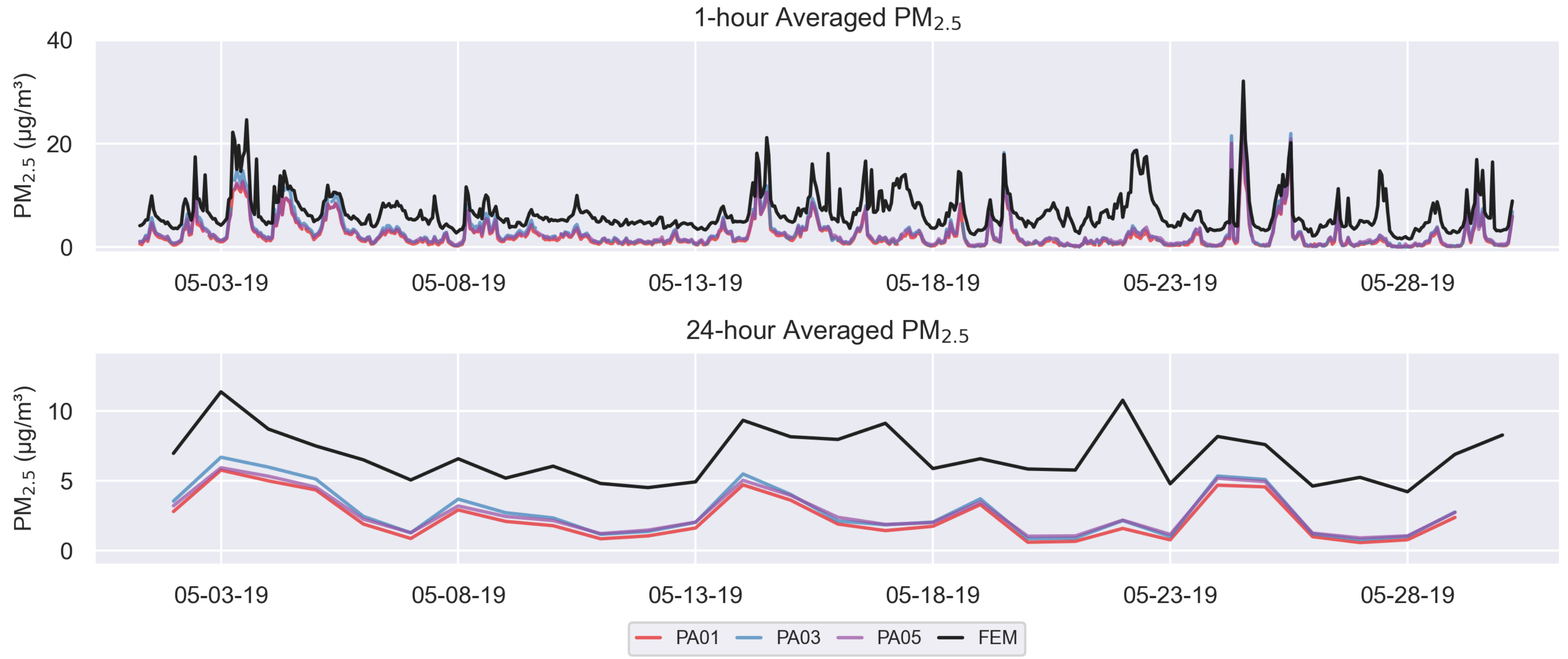

Scatter Plots: Comparison to FRM/FEM

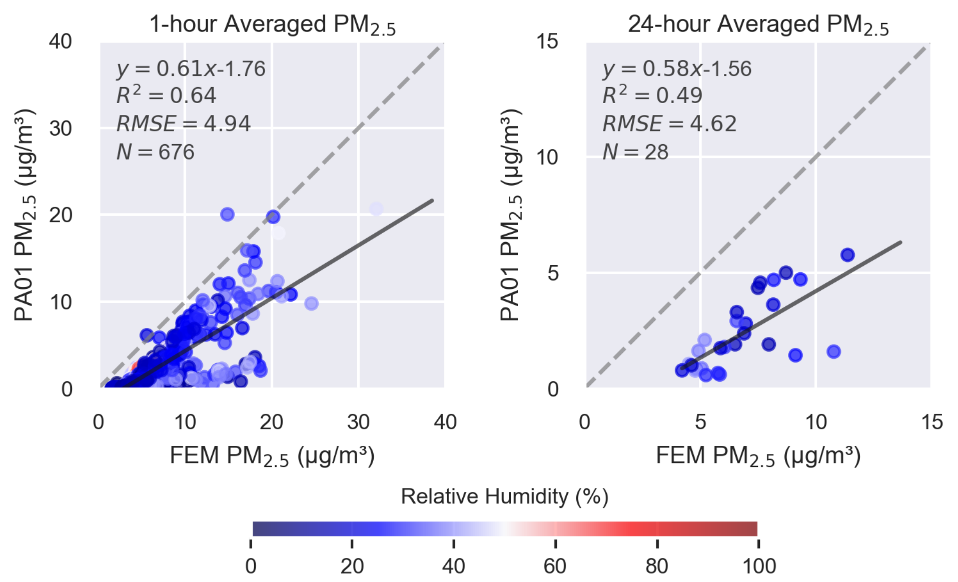

Range of FRM/FEM concentrations over duration of base test (µg/m³)	[1-hr] 1.6-32.0, avg: 6.7,<br>[24-hr] 4.2-11.4, avg: 6.8
Number of 24-hr periods in FRM/FEM monitor measurements with a goal concentration ≥ 25 µg/m³	0

Performance Metrics★

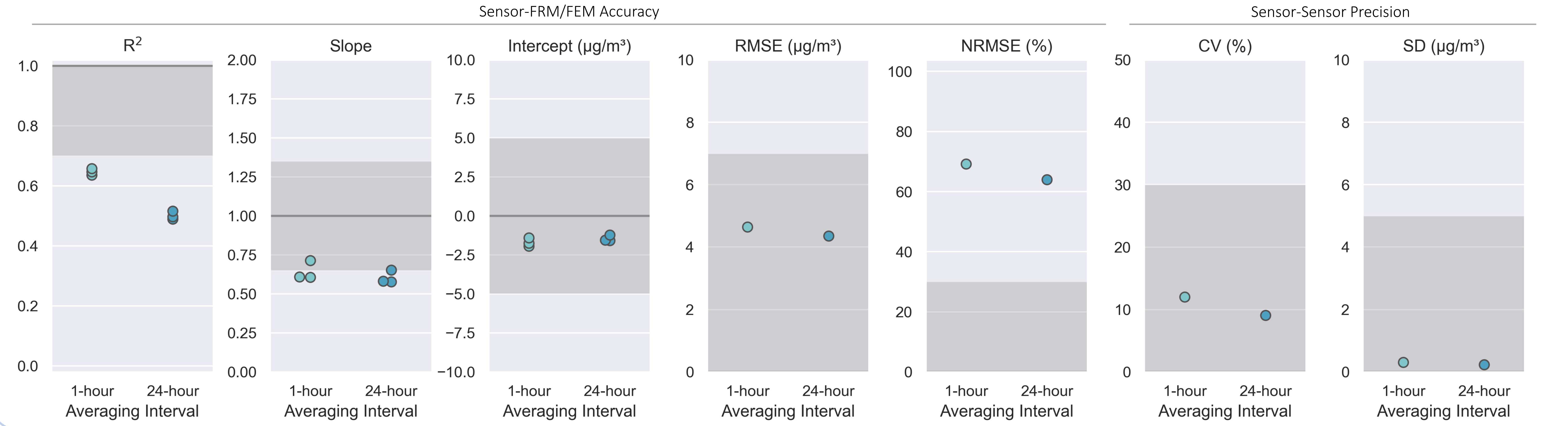

Meteorological Conditions During Deployment

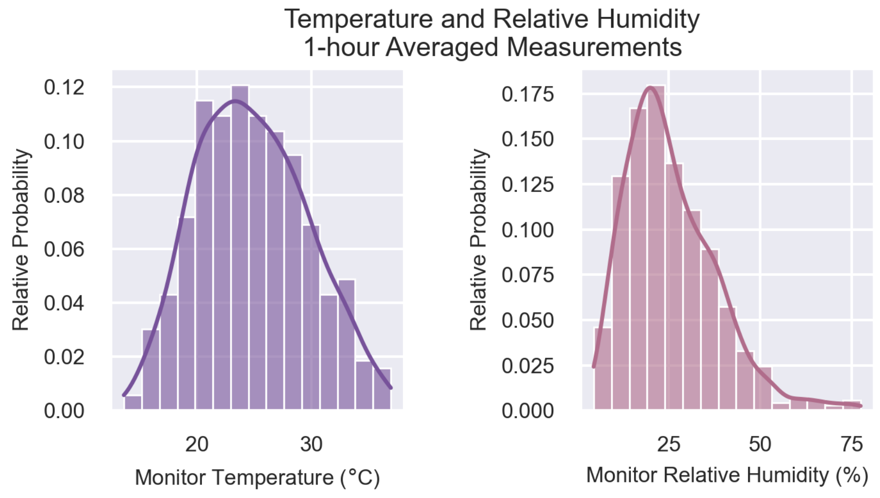

Number of 24-hr periods outside sensor manufacture-listed temperature operational range (no operational range specified)	-
Number of 24-hr periods outside sensor manufacture-listed relative humidity operational range (no operational range specified)	-

Meteorological Influence

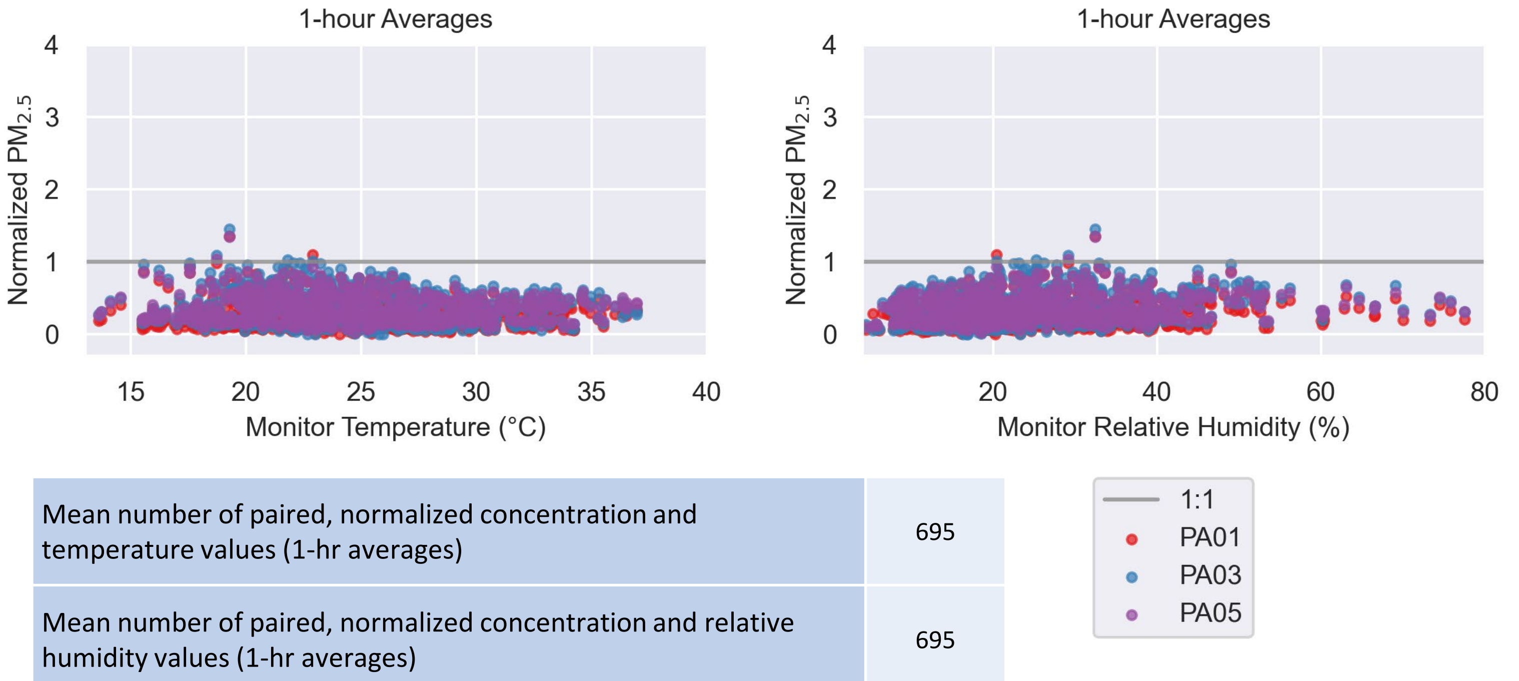

Mean number of paired, normalized concentration and temperature values (1-hr averages)	695
Mean number of paired, normalized concentration and relative humidity values (1-hr averages)	695

★For evaluations with greater than three sensors, grouping individual sensor metrics into boxplots is recommended for displaying results. Note that this recommendation does not apply to metrics computed as a single value for all sensors over the whole evaluation group, such as RMSE, NRMSE, CV, and standard deviation.

Testing Report - $PM_{2.5}$ Base Testing

PurpleAir

This report reflects out-of-the-box performance

PurpleAir
U.S. Environmental Protection Agency
Office of Research and Development
Clements.Andrea@epa.gov
919-541-1364
May 2019

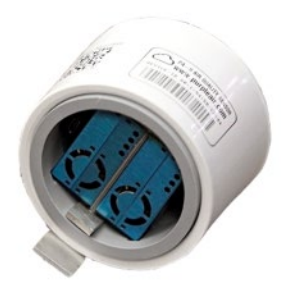

Tabular Statistics

Sensor-FRM/FEM Correlation

	Bias and Linearity						Data Quality			
	R^2		Slope		Intercept ($\mu g/m^3$)		Uptime (%)		Number of paired sensor and FRM/FEM concentration values	
	1-Hour ○○○	24-Hour ○○○	1-Hour ●○○	24-Hour ●○○	1-Hour ●●●	24-Hour ●●●	1-Hour ●●●	24-Hour ●●●	1-Hour	24-Hour
Metric Target Range	≥ 0.70	≥ 0.70	1.0 ± 0.35	1.0 ± 0.35	-5 ≤ b ≤ 5	-5 ≤ b ≤ 5	75%*	75%*	-	-
Sensor PA01	0.64	0.49	0.61	0.58	-1.76	-1.56	97	97	676	28
Sensor PA03	0.66	0.50	0.71	0.65	-1.95	-1.59	95	97	658	28
Sensor PA05	0.65	0.52	0.61	0.58	-1.41	-1.23	95	97	660	28
Mean	0.65	0.50	0.64	0.60	-1.71	-1.46	96	97	665	28

	Error			
	RMSE ($\mu g/m^3$)		NRMSE (%)	
	1-Hour ★	24-Hour ★	1-Hour ☆	24-Hour ☆
Metric Target Range	≤ 7.0	≤ 7.0	≤ 30.0	≤ 30.0
Deployment Value	4.6	4.4	69.2	64.0

Device-specific metrics (computed for each sensor in evaluation)
○○○ Metric value for none of devices tested falls within the target range
●○○ Metric value for one of devices tested falls within the target range
●●○ Metric value for two of devices tested falls within the target range
●●● Metric value for three of devices tested falls within the target range

Single-valued metrics (computed via entire evaluation dataset)
☆ Indicates that the metric value is not within the target range
★ Indicates that the metric value is within the target range

Sensor-Sensor Precision

	Precision (between collocated sensors)				Data Quality	
	CV (%)		SD ($\mu g/m^3$)		Number of concurrent sensor concentration pairs	
	1-Hour ★	24-Hour ★	1-Hour ★	24-Hour ★	1-Hour	24-Hour
Metric Target Range	≤ 30.0	≤ 30.0	≤ 5.0	≤ 5.0	-	-
Deployment Value	12.0	9.1	0.3	0.2	608	28

*This value is only a recommendation for ensuring data quality and is not included in the list of target values discussed in Section 4 of the Performance Testing Protocols, Metrics, and Target Values for Fine Particulate Matter Air Sensors document.

Testing Report - $PM_{2.5}$ Base Testing

PurpleAir

This report reflects out-of-the-box performance

PurpleAir
U.S. Environmental Protection Agency
Office of Research and Development
Clements.Andrea@epa.gov
919-541-1364
May 2019

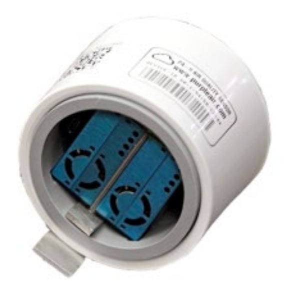

Sensor-FRM/FEM Scatter Plots

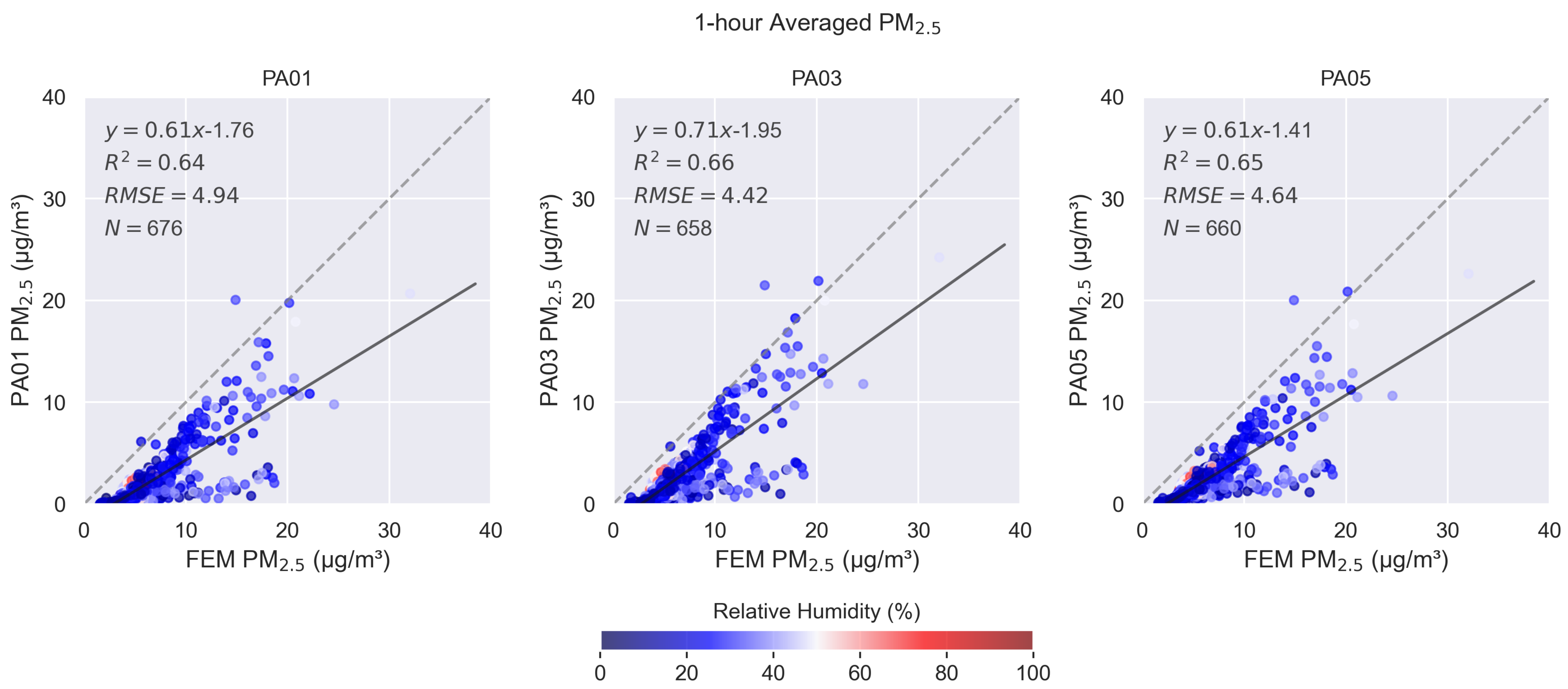

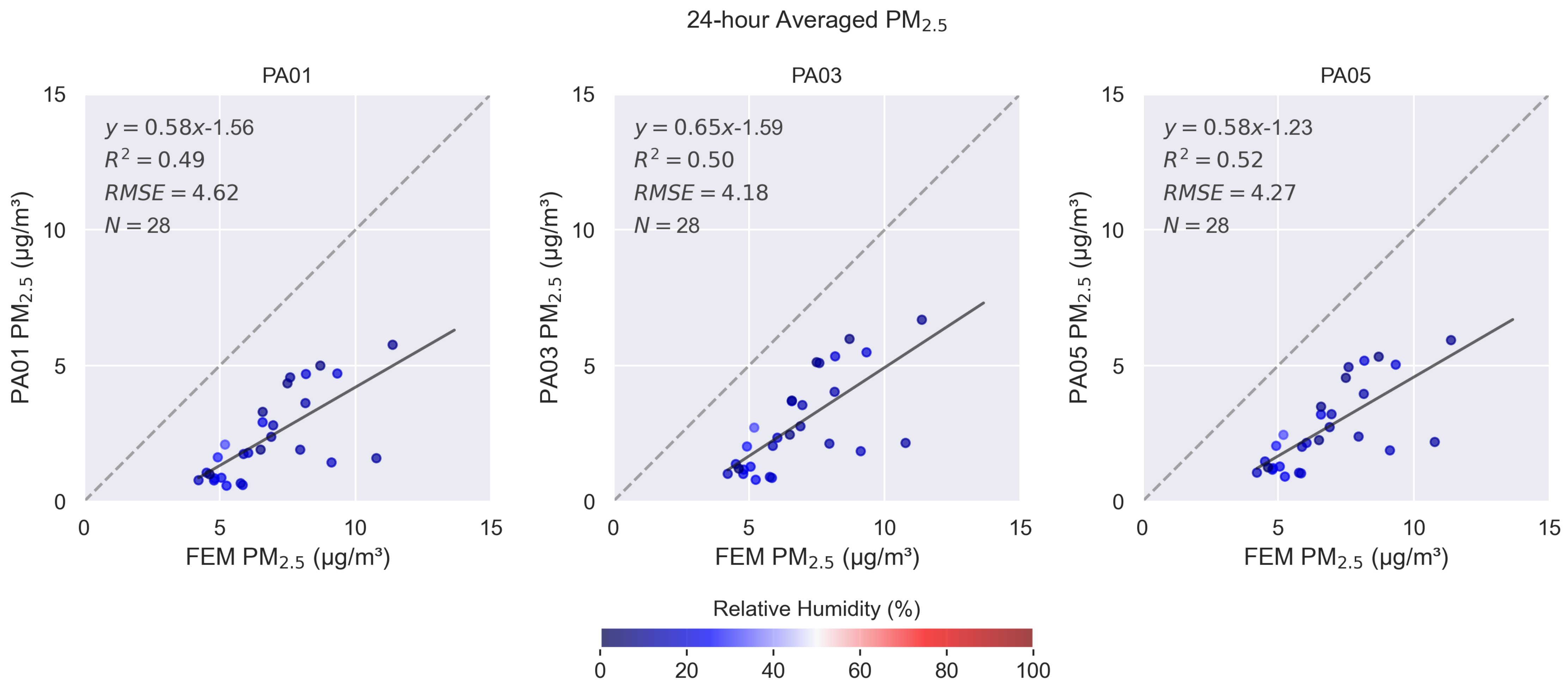

PurpleAir
U.S. Environmental Protection Agency
Office of Research and Development
Clements.Andrea@epa.gov
919-541-1364
May 2019

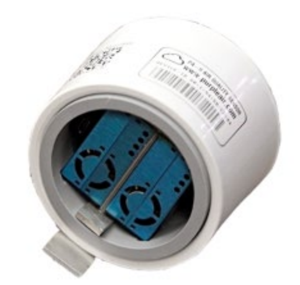

Supplemental Information

Additional documentation may be attached or linked to digital versions alongside this report. Such documentation may include field reports and observations during the testing period, maintenance logs for sensors and FRM/FEM monitors, standard operating procedures, and other documentation relevant to this testing report (see below for examples).

Supplemental Documentation	Attached	Description & URL or file path to documentation
Field observations	☐	
Maintenance logs	☐	Maintenance logs maintained by Maricopa County Air Quality
Standard operating procedure(s)	☐	U.S. EPA Office of Research and Development SOP available upon request
Photos of equipment setup and testing	☑	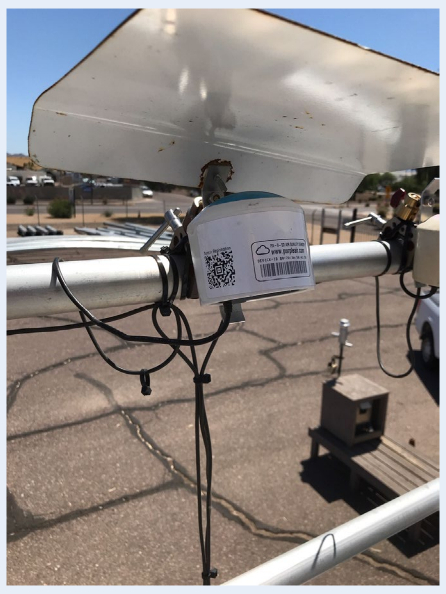 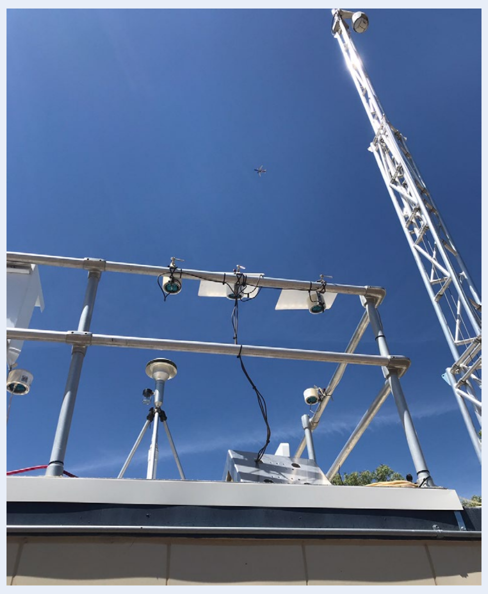
Product specifications sheet(s)	☑	PurpleAir: PA-II-SD Product website
Product manual(s)	☐	N/A
Deployment issues	☐	
Data storage and transmission method	☑	Sensor data stored locally on the sensor SD card. Card was swapped weekly and manually downloaded by Maricopa County Air Quality Department Staff and securely stored by the Quality Assurance Program. FEM data was connected to an on-site data logger and was reviewed and securely stored by the Quality Assurance Program. Copies of all data were emailed to U.S. EPA Office of Research and Development.
Data correction approach	☑	No data correction was applied to the sensor data. This report reflects out-of-the-box performance.
Data analysis/correction scripts and version	☑	Averaging and processing of data, calculation of performance metrics, and generation of figures and other supplementary material for analysis were obtained using Python 3.8.20 with the packages sensortoolkit v1.1.0, pandas 1.3.5, NumPy 1.24.4, Matplotlib 3.7.5, statsmodels 0.13.5, and seaborn 0.11.2. All packages are available from the Python Package Index (PyPI) at https://pypi.org/. The integrated development environment (IDE) Spyder 5.5.5 was used for scripting and data visualization. Version control for the Python base, packages, and IDE were all managed by conda 24.4.0.
Air Monitoring Station QAPP	☑	U.S. EPA Office of Research and Development's research study QAPP available upon request. Maricopa County Air Quality Department's Network Monitoring Plan available upon request.
Summary of FRM/FEM monitor QC checks	☑	Because this is a regulatory air monitoring site, FEM QC checks were conducted as required by 40 CFR Part 58 and the Air Monitoring Network Plan maintained by Maricopa County Air Quality Department.
Manufacturer website for FRM/FEM monitor	☑	Teledyne API: Model T640 Product website
FRM/FEM monitor manual	☑	Teledyne API: Model T640 Product Manual
FRM/FEM monitor specifications sheet(s)	☑	Teledyne API: Model T640 Specification Sheet
Other documents	☐	
