# Supplementary material for: Sensortoolkit—A Python Library for Standardizing the Ingestion, Analysis, and Reporting of Air Sensor Data for Performance Evaluation
Source: Sensors (Basel). 2025 Sep 10;25(18):5645. doi: 10.3390/s25185645 (PMC12473223; doi:10.3390/s25185645)
Supplement: Supplementary file 1 [file sensors-25-05645-s001.zip › Supplemental3_EPA_Ozone_Test_Reports2022.pdf]

O_3 Base Testing Reports

Long-Term Performance Project & RTP, NC Evaluations

Initial Base Testing
U.S. Environmental Protection Agency
Office of Research and Development
PI: Clements.Andrea@epa.gov
919-541-1363

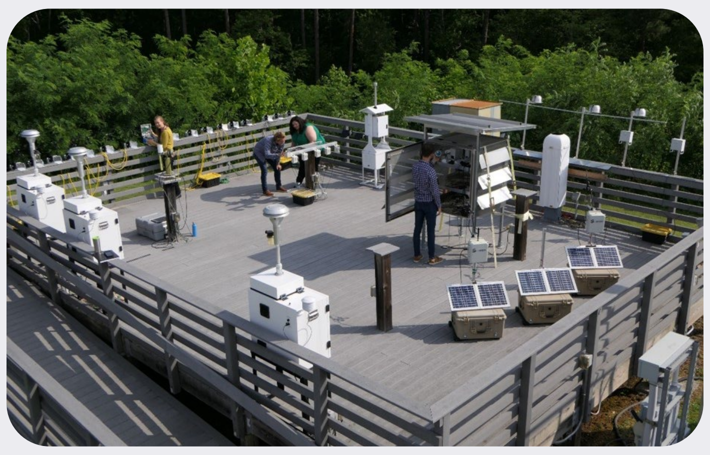

Disclaimer

This document has been reviewed by the U.S. Environmental Protection Agency (EPA), Office of Research and Development, and approved for publication. Any mention of trade names, products, or services does not imply an endorsement by the U.S. Government or the U.S. Environmental Protection Agency. The EPA does not endorse any commercial products, services, or enterprises. Links to websites outside the EPA website are provided for the convenience of the user. Inclusion of information about a website, an organization, a product or a service does not represent endorsement or approval by EPA, nor does it represent EPA opinion, policy or guidance unless specifically indicated. EPA does not exercise any editorial control over the information that may be found at any non-EPA website.

Aeroqual Ltd. provided sensor technologies as part of a Cooperative Research and Development Agreement (CRADA, #934-16) with the EPA. The views expressed in this document are solely those of the authors and EPA does not endorse any products or commercial services mentioned in this publication.

Table of Contents

Base Testing Reports (organized by testing site)

Long-Term Performance Project (LTPP)

Phoenix, Arizona
- Aeroqual AQY
- SENSIT RAMP

Denver, Colorado
- Aeroqual AQY
- SENSIT RAMP

Wilmington, Delaware
- Aeroqual AQY
- SENSIT RAMP

Decatur, Georgia
- Aeroqual AQY
- SENSIT RAMP

Research Triangle Park, North Carolina
- Aeroqual AQY
- SENSIT RAMP

Edmond, Oklahoma
- Aeroqual AQY
- SENSIT RAMP

Milwaukee, Wisconsin
- Aeroqual AQY
- SENSIT RAMP

Additional Ozone Sensor Evaluations

Research Triangle Park, North Carolina
- Apis APM01
- Myriad Sensors PocketLab Air
- Vaisala AQT420

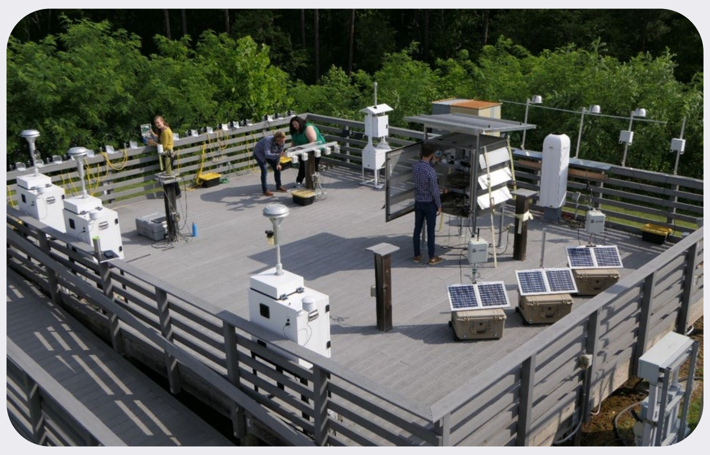

List of Acronyms and Abbreviations

AIRS	Ambient Air Innovation Research Site
AQS	Air Quality System
AZ	Arizona
CFR	Code of Federal Regulations
CO (state)	Colorado
CO (pollutant)	Carbon monoxide
CRADA	Cooperative Research and Development Agreement
CV	Coefficient of variation
DE	Delaware
DMAS	Denver Municipal Animal Shelter
EDT	Eastern Daylight Time
EPA	United States Environmental Protection Agency
FEM	Federal Equivalent Method
FRM	Federal Reference Method
GA	Georgia
IDE	Integrated Development Environment
MCAQD	Maricopa County Air Quality Department
MDL	Minimum Detection Limit
NC	North Carolina
NCore	National Core (Multipollutant Network)
NO	Nitrogen monoxide
NO_2	Nitrogen dioxide
NO_x	Oxides of nitrogen
NO_y	Reactive nitrogen compounds
NRMSE	Normalized root mean squared error
O_3	Ozone
OAQPS	Office of Air Quality Planning and Standards
OEM	Original Equipment Manufacturer
OK	Oklahoma
ORD	Office of Research and Development
PI	Principal Investigator
PM_{10}	Particulate matter with aerodynamic diameter less than 10 microns
$PM_{2.5}$	Particulate matter with aerodynamic diameter less than 2.5 microns
QA	Quality assurance
QAPP	Quality Assurance Project Plan
QC	Quality control
RH	Relative humidity
RMSE	Root mean square error
RTP	Research Triangle Park
SD	Standard deviation
SLAMS	State or Local Air Monitoring Stations Network
SO_2	Sulfur dioxide
SOP	Standard Operating Procedure
SSID	Service set identifier
T	Temperature
UI	User Interface
USB	Universal Serial Bus
WDNR	Wisconsin Department of Natural Resources
WI	Wisconsin

Testing Report - O_3 Base Testing

Aeroqual AQY

This report reflects out-of-the-box performance

Initial Base Testing - Phoenix, AZ

U.S. Environmental Protection Agency
Office of Research and Development
PI: Clements.Andrea@epa.gov
919-541-1363

September 2019—October 2019

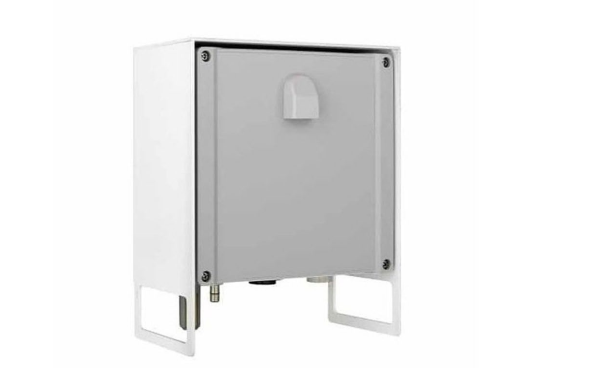

Image Attribution: Aeroqual Limited (Auckland, New Zealand)

Deployment Details

Testing Organization and Site Information	
Testing organization (Name, Organization type, Contact website)	U.S. Environmental Protection Agency - Office of Research and Development<br>Federal Government<br>Air Sensor Toolbox | U.S. EPA Website
Testing location (City, State, Latitude and Longitude)	West Phoenix<br>Phoenix, AZ<br>33.48385, -112.14257
AQS site ID	04 - 013 - 0019
Sampling timeframe (MM-DD-YY)	09-15-19 to 10-16-19
Sensor data source	Aeroqual Cloud
Reference data source	AQS API download

Sensor Information			
Manufacturer, model	Aeroqual AQY		
Device firmware version	1.14.2		
Sampling time interval	1-minute		
Sensor serial numbers	AQ Y_01		
Issues encountered during deployment?	☐	No Issues	

FRM/FEM Information	
Manufacturer, model, designation	Teledyne API T400 FEM
Sampling time interval	1-hour averaging
Date of calibration	As required by 40 CFR Part 58 and the Air Monitoring Network Plan maintained by MCAQD
Date of one-point QC check	Every two weeks as required by 40 CFR Part 58 Appendix A 3.1.1
Description, date(s) of maintenance activities	N/A

Time Series Plot: 1-hour averaged O_3

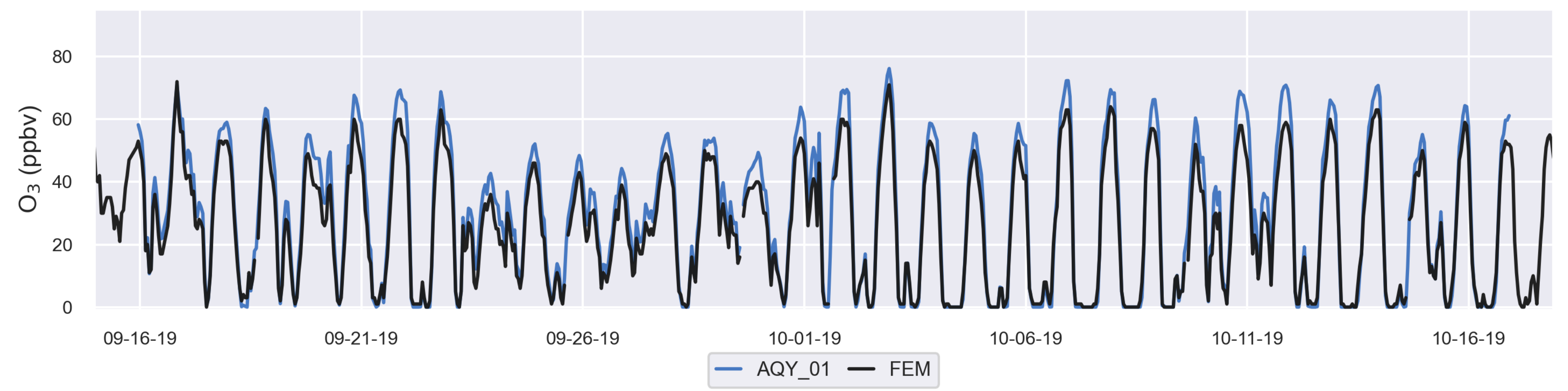

Range and average of FRM/FEM concentrations over duration of base test (ppbv)	[1-hr] 0.0-72.0, avg: 25.5,<br>[Rolling 8-hr] 0.0-60.2, avg: 25.7
Number of 1-hr periods in FRM/FEM monitor measurements with a goal concentration ≥ 60 ppbv	17

Scatter Plot: Comparison to FRM/FEM

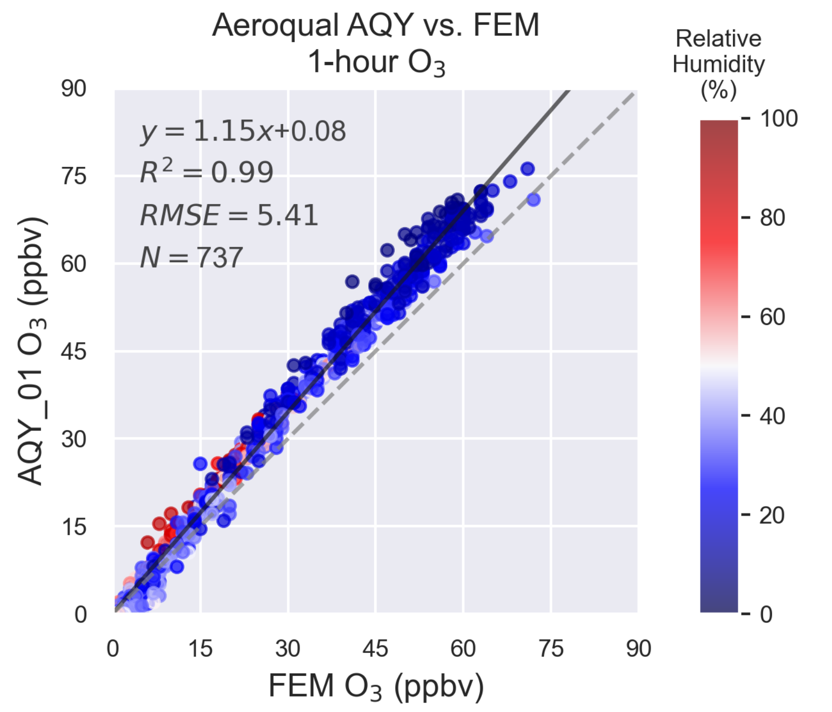

Performance Metrics

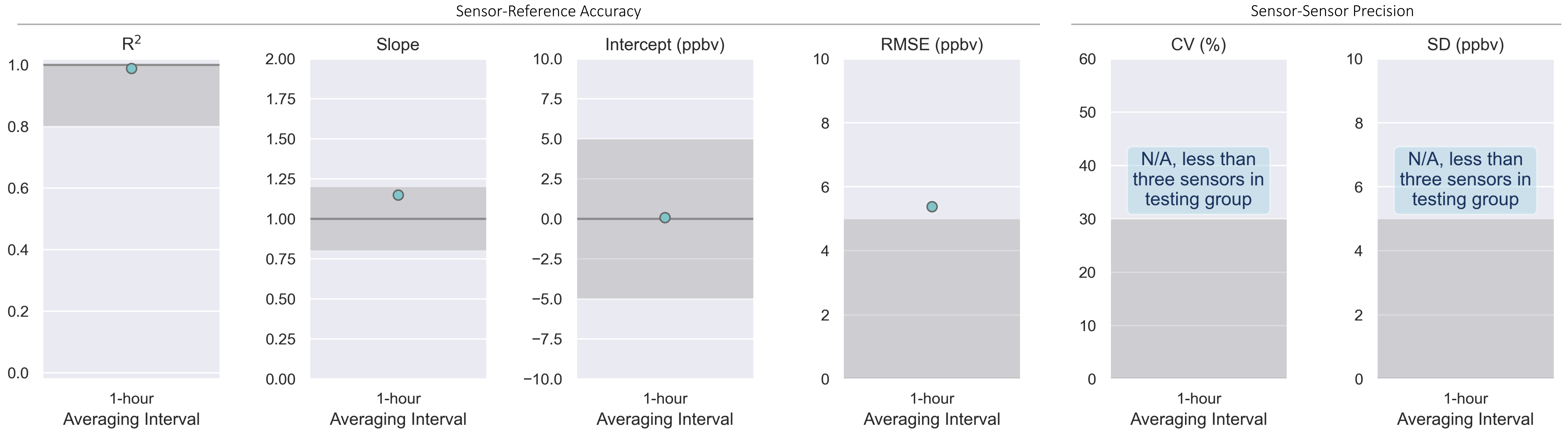

Meteorological Conditions During Deployment

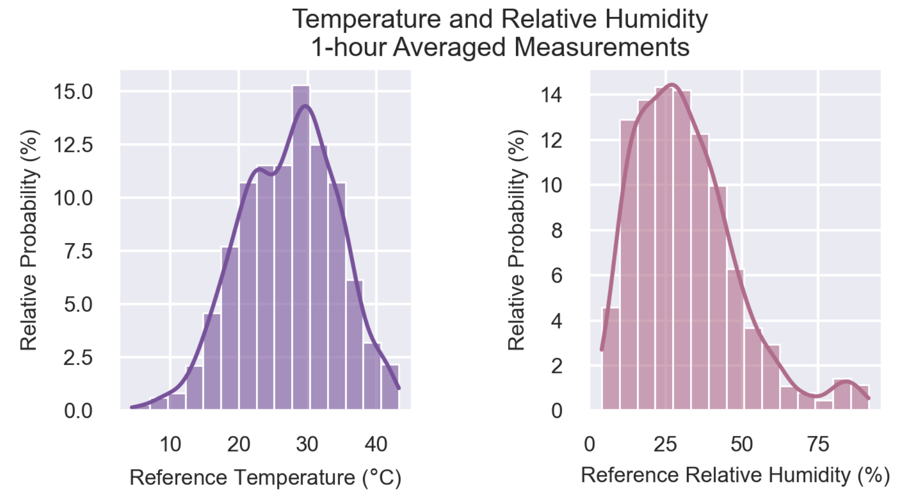

Number of 1-hr periods outside sensor manufacturer-listed temperature operational range (-10 to 40 °C)	0
Number of 1-hr periods outside sensor manufacturer-listed relative humidity operational range (no operational range specified)	-

Meteorological Influence

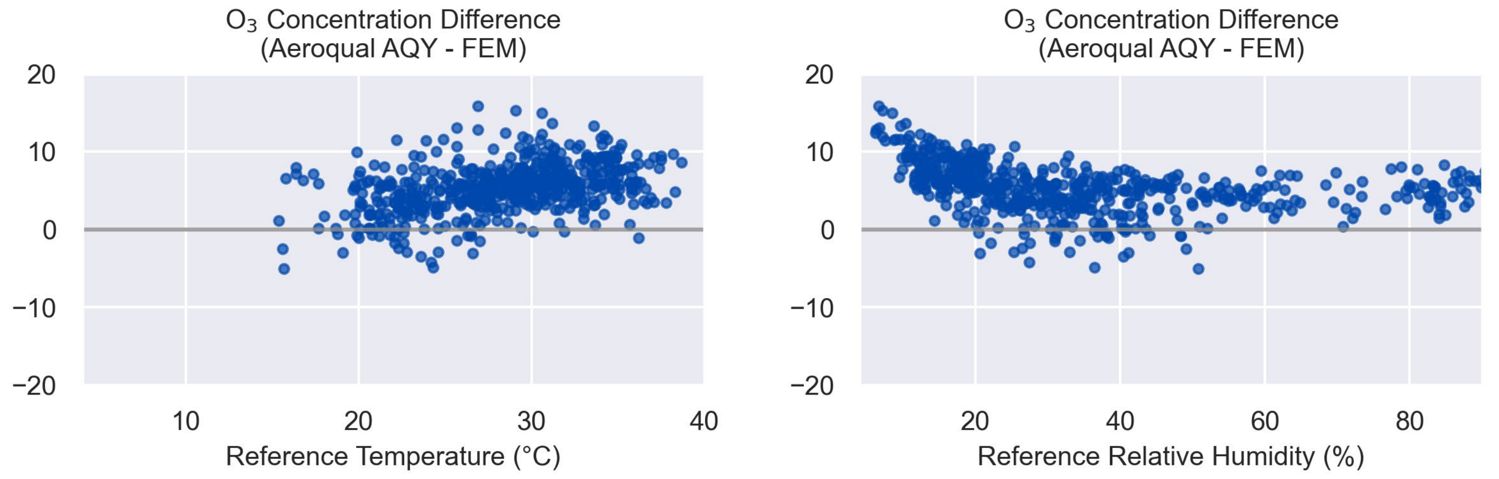

Mean number of paired, normalized concentration and temperature values (1-hr averages)	744
Mean number of paired, normalized concentration and relative humidity values (1-hr averages)	744

FEM data <5 ppbv (Federal MDL for the T400) has been removed (Meteorological Influence section only)

Testing Report - O_3 Base Testing

Aeroqual AQY

This report reflects out-of-the-box performance

Initial Base Testing - Phoenix, AZ

U.S. Environmental Protection Agency
Office of Research and Development
PI: Clements.Andrea@epa.gov
919-541-1363

September 2019—October 2019

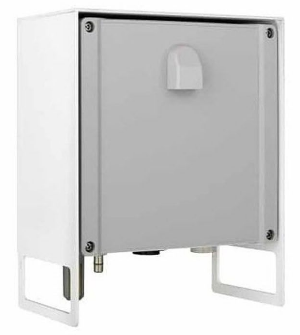

Image Attribution: Aeroqual Limited (Auckland, New Zealand)

Tabular Statistics

Sensor-FRM/FEM Correlation

	Bias and Linearity			Data Quality	
	R^2	Slope	Intercept (ppbv)	Uptime (%)	Number of paired sensor and reference concentration values*
	1-Hour ●	1-Hour ●	1-Hour ●	1-Hour ●	1-Hour
Metric Target Range	≥ 0.80	1.0 ± 0.20	-5 ≤ b ≤ 5	75%*	-
Sensor AQY_01	0.99	1.15	0.08	100	737

	Error
	RMSE (ppbv)
	1-Hour ☆
Metric Target Range	≤ 5.0
Deployment Value	5.4

Device-specific metrics (computed for each sensor in evaluation)
- ○ Metric value for none of devices tested falls within the target range
- ● Metric value for one of devices tested falls within the target range

Sensor-Sensor Precision[1]

	Precision (between collocated sensors)		Data Quality
	CV (%)	SD (ppbv)	Number of paired sensor and reference concentration values*
	1-Hour ☆	1-Hour ☆	1-Hour
Metric Target Range	≤ 30.0	≤ 5.0	-
Deployment Value	-	-	-

[1]Precision statistics are computed for evaluations with at least three collocated sensor units. Metric values are left blank for evaluations with two or fewer sensor units.

Single-valued metrics (computed via entire evaluation dataset)
- ☆ Indicates that the metric value is not within the target range
- ★ Indicates that the metric value is within the target range

*This value is only a recommendation for ensuring data quality and is not included in the list of target values discussed in Section 4 of the Performance Testing Protocols, Metrics, and Target Values for Ozone Air Sensors document.

Testing Report - O_3 Base Testing

Aeroqual AQY

This report reflects out-of-the-box performance

Initial Base Testing - Phoenix, AZ
U.S. Environmental Protection Agency
Office of Research and Development
PI: Clements.Andrea@epa.gov
919-541-1363
September 2019—October 2019

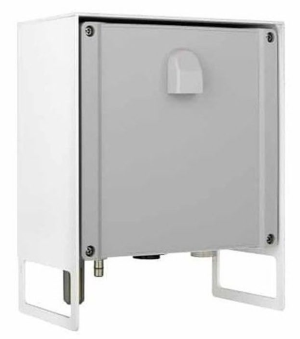

Image Attribution: Aeroqual Limited (Auckland, New Zealand)

Supplemental Information

Abbreviations used in Supplemental Information

FRM	Federal Reference Method
FEM	Federal Equivalent Method
SOP	Standard Operating Procedure
QAPP	Quality Assurance Project Plan
QC	Quality Control

Supplemental Documentation	Attached	Description & URL or file path to documentation
Field observations and sensor data flags	☑	See AZ-AQY-Page 5 of this testing report
Maintenance logs	☐	No logs recorded during testing
Standard operating procedure(s)	☐	U.S. EPA Office Of Research and Development SOP available upon request
Photos of equipment setup and testing	☑	See AZ-AQY-Page 4 of this testing report
Product specifications sheet(s)	☑	See Appendix C, “Spec_Sheet_Aeroqual_AQY.pdf”*
Product manual(s)	☑	See Appendix C, “Manual_Aeroqual_AQY.pdf”*
Data storage and transmission method	☑	See AZ-AQY-Page 5 of this testing report
Data correction approach	☑	See AZ-AQY-Page 5 of this testing report
Issues encountered	☑	See AZ-AQY-Page 5 of this testing report
Data analysis/correction scripts and version	☑	Averaging and processing of data, calculation of performance metrics, and generation of figures and other supplementary material for analysis were obtained using Python 3.9.7 with the packages sensortoolkit v0.8.3b2, pandas 1.3.5, NumPy 1.21.2, Matplotlib 3.5.0, statsmodels 0.13.0, and seaborn 0.11.2. All packages are available from the Python Package Index (PyPI) at https://pypi.org/. The integrated development environment (IDE) Spyder 5.1.5 was used for scripting and data visualization. Version control for the Python base, packages, and IDE were all managed by conda 4.11.0.
Air Monitoring Station QAPP	☐	U.S. EPA Office Of Research and Development QAPP available upon request
Summary of FRM/FEM monitor QC checks	☑	See AZ-AQY-Page 6 of this testing report
Manufacturer website for FRM/FEM monitor	☑	Teledyne API: Model T400 Product website
FRM/FEM monitor manual	☑	See Appendix B, “Spec_Sheet_TeledyneAPI_T400.pdf”
FRM/FEM monitor specifications sheet(s)	☑	See Appendix B, “Manual_TeledyneAPI_T400.pdf”
Other documents	☑	Manufacturer notice of AQY sales on hold

*As of 3/18/2021, the manufacturer of the AQY has placed sales of a similar unit on hold. Documentation for the AQY is currently unavailable from the manufacturer’s website.

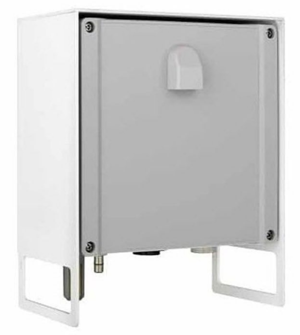

Image Attribution: Aeroqual Limited (Auckland, New Zealand)

Supplemental Information: Photos of Testing Site and Equipment Setup

Site Description:
The West Phoenix Monitoring Station has been operational since 1984. The spatial scale for the West Phoenix site is Neighborhood. It is located in an area of stable, high-density residential properties. This State or Local Air Monitoring Stations (SLAMS) location monitors for CO, NO_2, O_3, PM_{10}, and $PM_{2.5}$. In addition, this is a quality assurance (QA) collocation site for $PM_{2.5}$ where the Maricopa County Air Quality Department (MCAQD) operates one filter-based $PM_{2.5}$ FRM sampler along with one continuous $PM_{2.5}$ FEM analyzer as per 40 CFR Part 58 Appendix A 3.1.1. Resources detailing air quality monitoring QA programs and procedures are detailed on EPA's Ambient Monitoring Technology Information Center website (https://www.epa.gov/amtic/ambient-air-monitoring-quality-assurance, *last accessed 5/11/2022*). Meteorological monitors operating at this site measure ambient temperature (T), barometric pressure, delta T (temperature inversion), and wind speed/direction.

Figure 1: Aeroqual AQY sensor (indicated by red arrow) attached to metal railing atop the sampling shelter at the monitoring site.

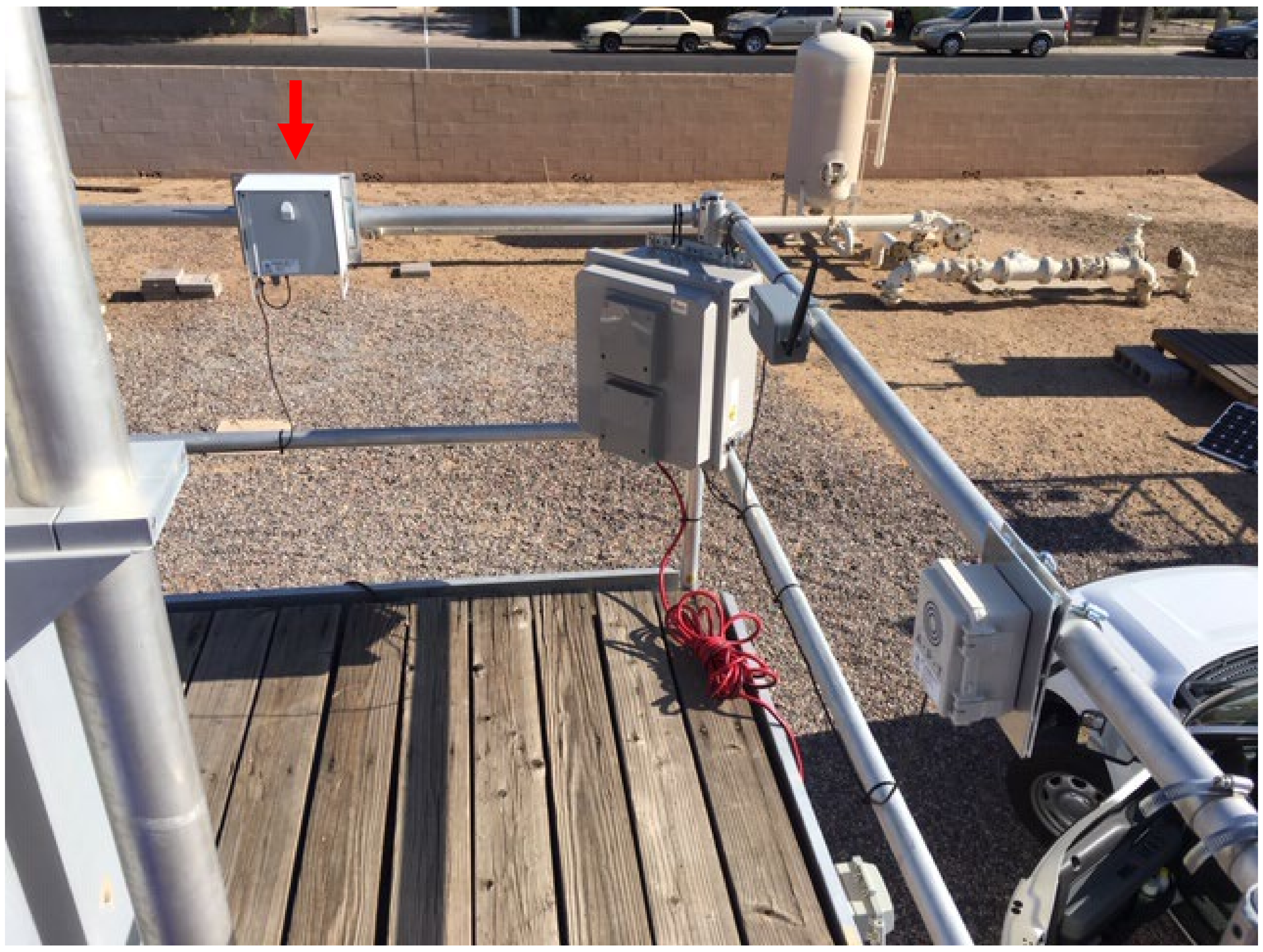

Figure 2: West Phoenix Monitoring Station

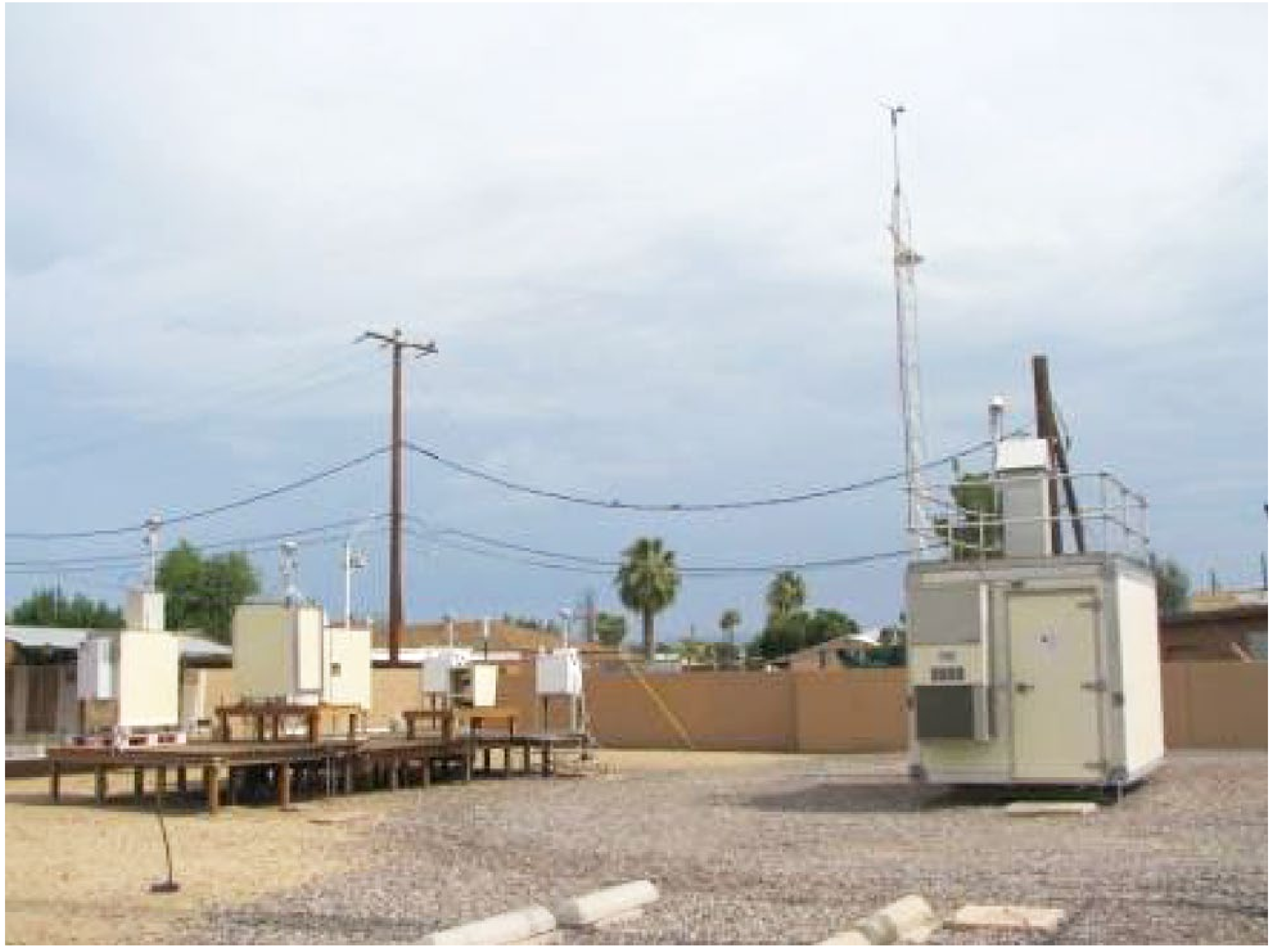

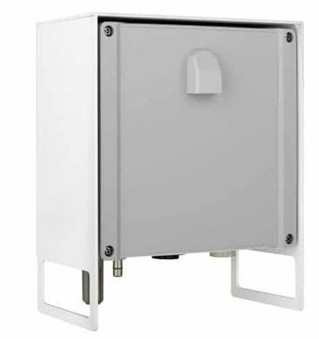

Image Attribution: Aeroqual Limited (Auckland, New Zealand)

Supplemental Information: Data Storage, Correction Approach, and Issues Encountered

Data Storage and Transmission Method

As part of CRADA #934-16 between Aeroqual and US EPA, Aeroqual supported data streaming. SIM cards were installed, and data flowed to the Aeroqual Cloud. The 1-minute raw data was acquired weekly using the Aeroqual Cloud (*last accessed 5/11/22*) user interface (UI). The AQY has an internal data storage USB flash drive as a data backup, however access requires software proprietary to Aeroqual.

Data Correction Approach

This evaluation report reflects “out-of-the-box” performance of the AQY. The manufacturer provides a procedure by which local collocation (sensor operated along side an FRM/FEM) data can be collected, a gain (slope) and offset (intercept) determined, and parameters entered into the Aeroqual Cloud user interface to be applied to all subsequently collected data. This procedure and feature was **not** used prior to this evaluation. Prospective consumers may get different performance from this device if they utilize this feature.

After acquisition, the raw data was processed using the *sensortoolkit* python code library (v0.8.3b2). A continuous data set at the recorded sampling frequency was written to a .csv file. 1-hour averaged data sets were generated using a 75% completeness threshold and saved as separate .csv files. Outliers were **not** removed from data sets in order to assess “out-of-the-box” sensor performance.

The duration of the warm-up period required for sensor measurements to equilibrate was determined from field data to be 10 minutes. Warm up periods were considered to occur following any power outage to sensor units, either due to routine field visits or unscheduled site power outages. Data recorded during warm up periods has been removed from data sets.

Issues Encountered

Pre-deployment observations

- *Timestamp inaccuracies*: During pre-deployment, the AQY devices did not properly sync timestamps with the onboard Real-Time Clock. Connecting the units to the internet by cellular or Wi-Fi allowed the unit to sync with internet time and resulted in proper timestamps.
- *Unit connectivity error:* The AQY unit experienced initial startup connectivity issues from August 12, 2019, when the sensor arrived at the monitoring site, through August 15, 2019, when the issue was resolved.

Field observations and sensor data flags

The following table contains data flags describing events that were encountered during the testing period. A brief power loss to the sensor occurred on 9/29/2019 and lasted approximately 10 minutes. This event occurred when the field technician was not at the monitoring site, indicating that the outage was likely not attributed to operator error.

Start Time (UTC)	End Time (UTC)	Sensor Serial ID	Parameters Impacted	Flag
2019-09-29 14:30:00+00:00	2019-09-29 14:41:00+00:00	AQY_01	ALL	9-Data Loss - Power Connection Error

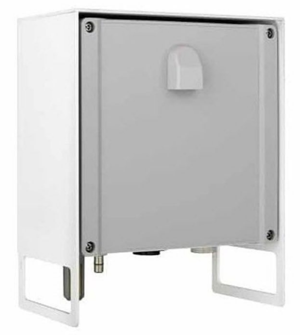

Image Attribution: Aeroqual Limited (Auckland, New Zealand)

Supplemental Information: Description of FRM/FEM QC Checks and Data Flags

Description of Data Flags

AQS

The U.S. EPA's Air Quality System (AQS) is the Agency's primary ambient air monitoring data archive. A comprehensive list of data flags that are recorded alongside AQS data sets, referred to by U.S. EPA as 'qualifiers', can be found at the following link: https://aqs.epa.gov/aqsweb/documents/codetables/qualifiers.html

AirNow-Tech

AirNow-Tech is an additional ambient air monitoring data service maintained by U.S. EPA and is commonly used by monitoring agencies to upload and validate monitoring data. Data which have yet to be QC'd for inclusion in AQS as well as monitoring data sets which are not planned for inclusion in AQS are typically available for near real-time download from AirNow-Tech.

Invalidation of reference data: AQS qualifiers are organized by qualifier type, which indicates whether data logged alongside qualifier flags should be invalidated (set null). Qualifiers with type "Null Data Qualifier" are invalidated, and includes data logged during periods that coincide with QC checks (e.g., "BF-Precision/Zero/Span", "BJ- Operator Error", "BL - QA Audit", "AZ - QC Audit") among other events such as power outages. Data logged alongside qualifiers with type "Quality Assurance Qualifiers" are not invalidated and are included in this analysis (e.g., concentrations less than the federal MDL for the reference monitor "MD – Value less than MDL", QA reviewed values "Validated Value").

Data Flags Recorded During Testing

FRM/FEM Monitor	Timestamp (UTC)	Flag
Teledyne Advanced Pollution Instrumentation T400 (Data acquired via AQS)	2019-09-18 15:00:00+0000	AX - Precision Check
	2019-09-25 15:00:00+0000	BF - Precision/Zero/Span
	2019-09-29 14:00:00+0000	AV - Power Failure
	2019-10-01 14:00:00+0000	BL - QA Audit
	2019-10-01 15:00:00+0000	AX - Precision Check
	2019-10-09 15:00:00+0000	BF - Precision/Zero/Span
	2019-10-14 15:00:00+0000	AX - Precision Check

Meteorological Instrument	Timestamp (UTC)	Flag
MetOne Temperature Monitor (Data acquired via AirNow-Tech)	2019-09-29 14:00:00+0000	9 - Invalid
	2019-10-01 19:00:00+0000 to 2019-10-01 20:00:00+0000	9 - Invalid
RM Young Relative Humidity Monitor (Data acquired via AirNow-Tech)	2019-09-29 14:00:00+0000	9 - Invalid

Testing Report - O_3 Base Testing

SENSIT RAMP

This report reflects out-of-the-box performance

Initial Base Testing - Phoenix, AZ
U.S. Environmental Protection Agency
Office of Research and Development
PI: Clements.Andrea@epa.gov
919-541-1363
October 2019—November 2019

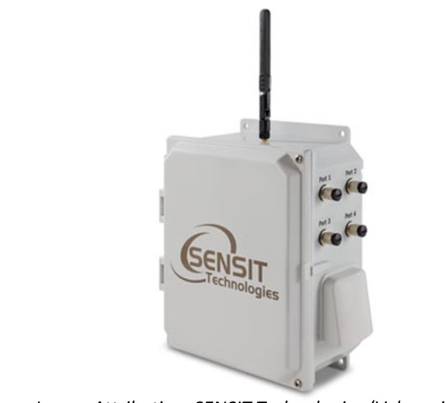

Image Attribution: SENSIT Technologies (Valparaiso, IN)

Deployment Details

Testing Organization and Site Information	
Testing organization (Name, Organization type, Contact website)	U.S. Environmental Protection Agency - Office of Research and Development<br>Federal Government<br>Air Sensor Toolbox | U.S. EPA Website
Testing location (City, State, Latitude and Longitude)	West Phoenix<br>Phoenix, AZ<br>33.48385, -112.14257
AQS site ID	04 - 013 - 0019
Sampling timeframe (MM-DD-YY)	10-11-19 to 11-10-19
Sensor data source	Onboard MicroSD card
Reference data source	AQS API download

Sensor Information			
Manufacturer, model	SENSIT RAMP		
Device firmware version	190308_AQ_v9.30		
Sampling time interval	15-seconds		
Sensor serial numbers	RAM_01		
Issues encountered during deployment?	☐	No Issues	

FRM/FEM Information	
Manufacturer, model, designation	Teledyne API T400 FEM
Sampling time interval	1-hour averaging
Date of calibration	As required by 40 CFR Part 58 and the Air Monitoring Network Plan maintained by MCAQD
Date of one-point QC check	Every two weeks as required by 40 CFR Part 58 Appendix A 3.1.1
Description, date(s) of maintenance activities	N/A

Time Series Plot: 1-hour averaged O_3

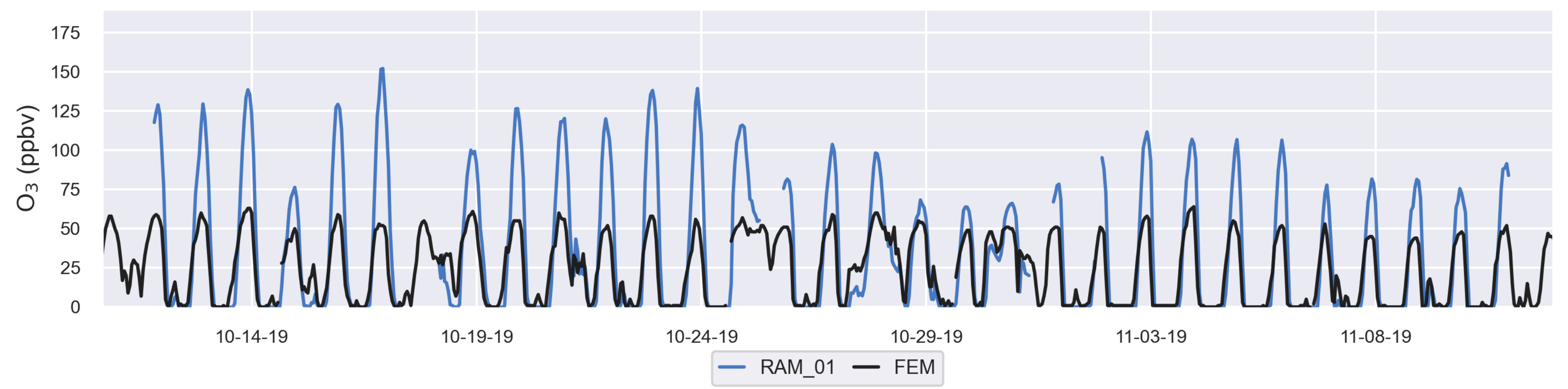

Range and average of FRM/FEM concentrations over duration of base test (ppbv)	[1-hr] 0.0-64.0, avg: 23.1,<br>[Rolling 8-hr] 0.0-57.1, avg: 23.2
Number of 1-hr periods in FRM/FEM monitor measurements with a goal concentration ≥ 60 ppbv	7

Scatter Plot: Comparison to FRM/FEM

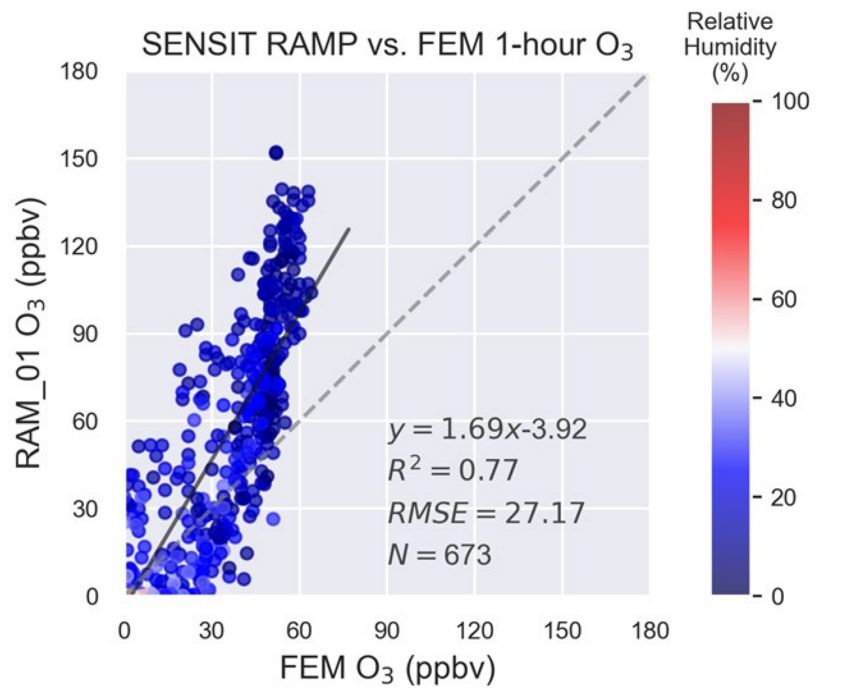

Performance Metrics

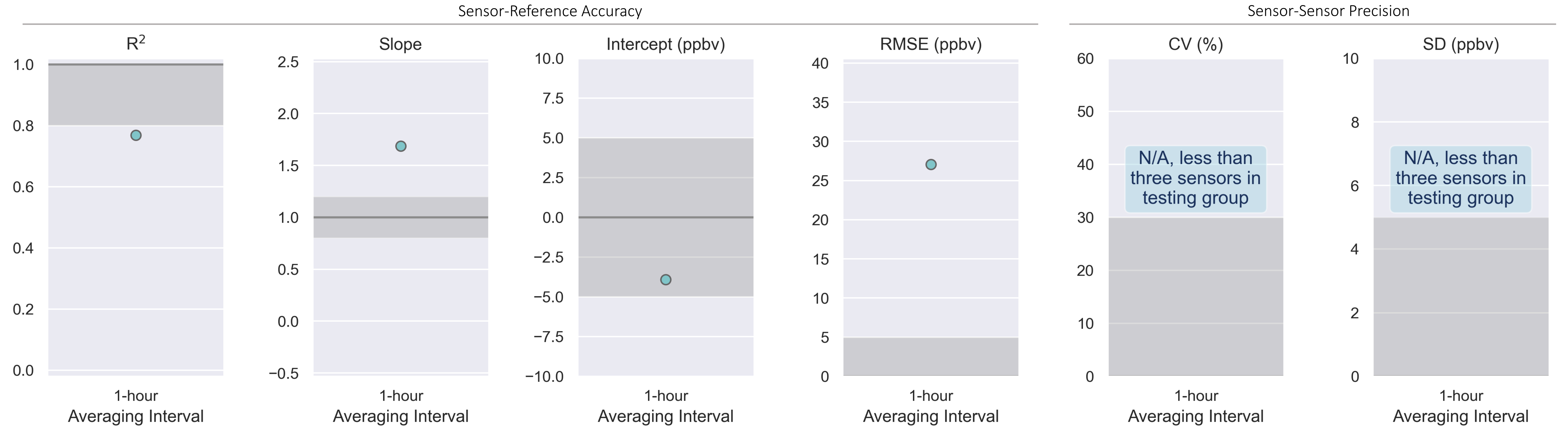

Meteorological Conditions During Deployment

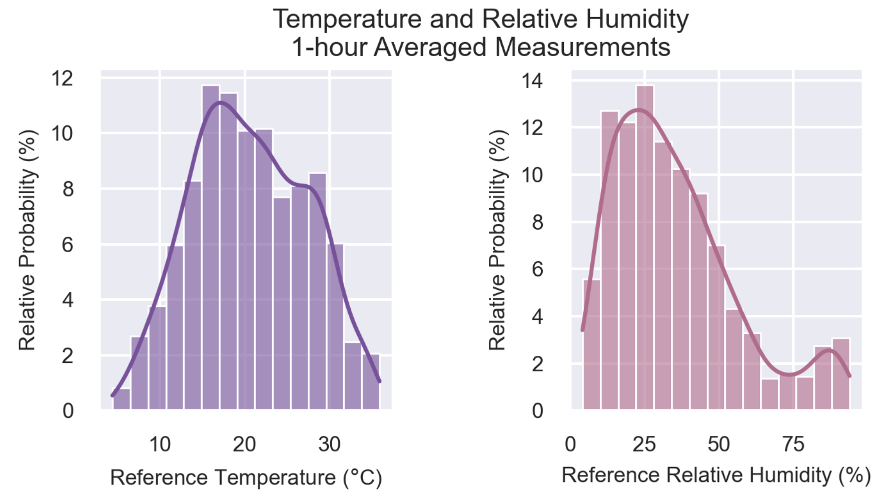

Number of 1-hr periods outside sensor manufacturer-listed temperature operational range (-20 to 50 °C)	0
Number of 1-hr periods outside sensor manufacturer-listed relative humidity operational range (no operational range specified)	-

Meteorological Influence

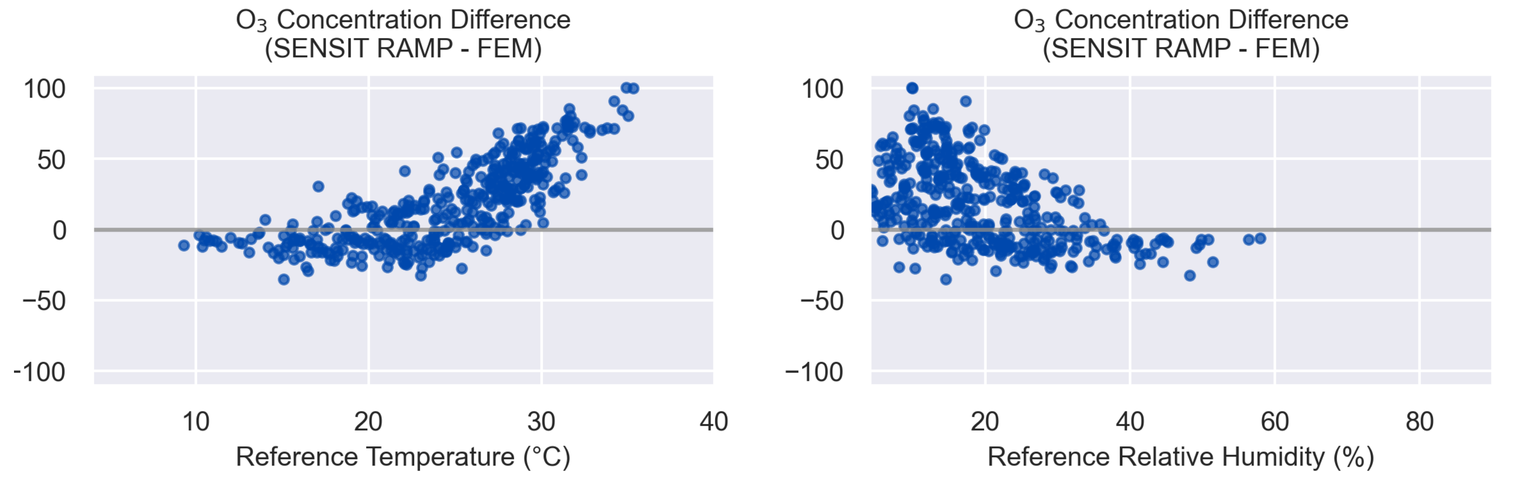

Mean number of paired, normalized concentration and temperature values (1-hr averages)	743
Mean number of paired, normalized concentration and relative humidity values (1-hr averages)	743

FEM data <5 ppbv (Federal MDL for the T400) has been removed (Meteorological Influence section only)

Testing Report - O_3 Base Testing

SENSIT RAMP

This report reflects out-of-the-box performance

Initial Base Testing - Phoenix, AZ

U.S. Environmental Protection Agency
Office of Research and Development
PI: Clements.Andrea@epa.gov
919-541-1363

October 2019—November 2019

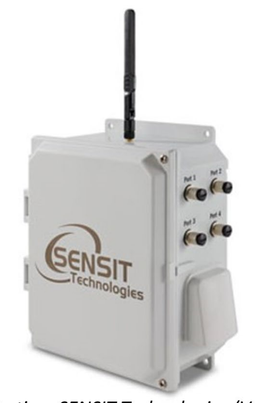

Image Attribution: SENSIT Technologies (Valparaiso, IN)

Tabular Statistics

Sensor-FRM/FEM Correlation

	Bias and Linearity			Data Quality	
	R^2	Slope	Intercept (ppbv)	Uptime (%)	Number of paired sensor and reference concentration values*
	1-Hour ○	1-Hour ○	1-Hour ●	1-Hour ●	1-Hour
Metric Target Range	≥ 0.80	1.0 ± 0.20	-5 ≤ b ≤ 5	75%*	-
Sensor RAM_01	0.77	1.69	-3.92	95	673

	Error
	RMSE (ppbv)
	1-Hour ☆
Metric Target Range	≤ 5.0
Deployment Value	27.1

Device-specific metrics (computed for each sensor in evaluation)
- ○ Metric value for none of devices tested falls within the target range
- ● Metric value for one of devices tested falls within the target range

Sensor-Sensor Precision[1]

	Precision (between collocated sensors)		Data Quality
	CV (%)	SD (ppbv)	Number of paired sensor and reference concentration values*
	1-Hour ☆	1-Hour ☆	1-Hour
Metric Target Range	≤ 30.0	≤ 5.0	-
Deployment Value	-	-	-

[1]Precision statistics are computed for evaluations with at least three collocated sensor units. Metric values are left blank for evaluations with two or fewer sensor units.

Single-valued metrics (computed via entire evaluation dataset)
- ☆ Indicates that the metric value is not within the target range
- ★ Indicates that the metric value is within the target range

*This value is only a recommendation for ensuring data quality and is not included in the list of target values discussed in Section 4 of the Performance Testing Protocols, Metrics, and Target Values for Fine Particulate Matter Air Sensors document.

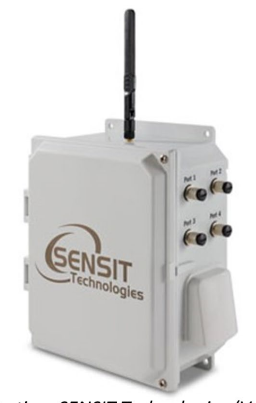

Image Attribution: SENSIT Technologies (Valparaiso, IN)

Supplemental Information

Abbreviations used in Supplemental Information

FRM	Federal Reference Method
FEM	Federal Equivalent Method
SOP	Standard Operating Procedure
QAPP	Quality Assurance Project Plan
QC	Quality Control

Supplemental Documentation	Attached	Description & URL or file path to documentation
Field observations and sensor data flags	☑	See AZ-RAM-Page 5 of this testing report
Maintenance logs	☐	No logs recorded during testing
Standard operating procedure(s)	☐	U.S. EPA Office Of Research and Development SOP available upon request
Photos of equipment setup and testing	☑	See AZ-RAM-Page 4 of this testing report
Product specifications sheet(s)	☑	See Appendix C, “Spec_Sheet_SENSIT_RAMP.pdf”
Product manual(s)	☑	See Appendix C, “Manual_SENSIT_RAMP.pdf”
Data storage and transmission method	☑	See AZ-RAM-Page 5 of this testing report
Data correction approach	☑	See AZ-RAM-Page 5 of this testing report
Issues encountered	☑	See AZ-RAM-Page 5 of this testing report
Data analysis/correction scripts and version	☑	Averaging and processing of data, calculation of performance metrics, and generation of figures and other supplementary material for analysis were obtained using Python 3.9.7 with the packages sensortoolkit v0.8.3b2, pandas 1.3.5, NumPy 1.21.2, Matplotlib 3.5.0, statsmodels 0.13.0, and seaborn 0.11.2. All packages are available from the Python Package Index (PyPI) at https://pypi.org/. The integrated development environment (IDE) Spyder 5.1.5 was used for scripting and data visualization. Version control for the Python base, packages, and IDE were all managed by conda 4.11.0.
Air Monitoring Station QAPP	☐	U.S. EPA Office Of Research and Development QAPP available upon request
Summary of FRM/FEM monitor QC checks	☑	See AZ-RAM-Page 6 of this testing report
Manufacturer website for FRM/FEM monitor	☑	Teledyne API: Model T400 Product website
FRM/FEM monitor manual	☑	See Appendix B, “Spec_Sheet_TeledyneAPI_T400.pdf”
FRM/FEM monitor specifications sheet(s)	☑	See Appendix B, “Manual_TeledyneAPI_T400.pdf”
Other documents	☐	

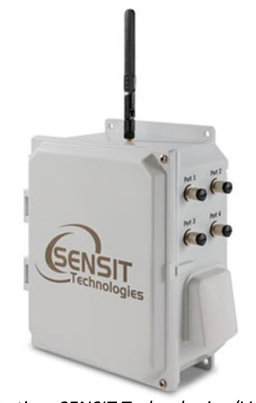

Image Attribution: SENSIT Technologies (Valparaiso, IN)

Supplemental Information: Photos of Testing Site and Equipment Setup

Site Description:

The West Phoenix Monitoring Station has been operational since 1984. The spatial scale for the West Phoenix site is Neighborhood. It is located in an area of stable, high-density residential properties. This State or Local Air Monitoring Stations (SLAMS) location monitors for CO, NO_2, O_3, PM_{10}, and $PM_{2.5}$. In addition, this is a quality assurance (QA) collocation site for $PM_{2.5}$ where the Maricopa County Air Quality Department (MCAQD) operates one filter-based $PM_{2.5}$ FRM sampler along with one continuous $PM_{2.5}$ FEM analyzer as per 40 CFR Part 58 Appendix A 3.1.1. Resources detailing air quality monitoring QA programs and procedures are detailed on EPA's Ambient Monitoring Technology Information Center website (https://www.epa.gov/amtic/ambient-air-monitoring-quality-assurance, *last accessed 5/11/2022*). Meteorological monitors operating at this site measure ambient temperature (T), barometric pressure, delta T (temperature inversion), and wind speed/direction.

Figure 1: SENSIT RAMP sensor (indicated by red arrow) attached to metal railing atop the sampling shelter at the monitoring site.

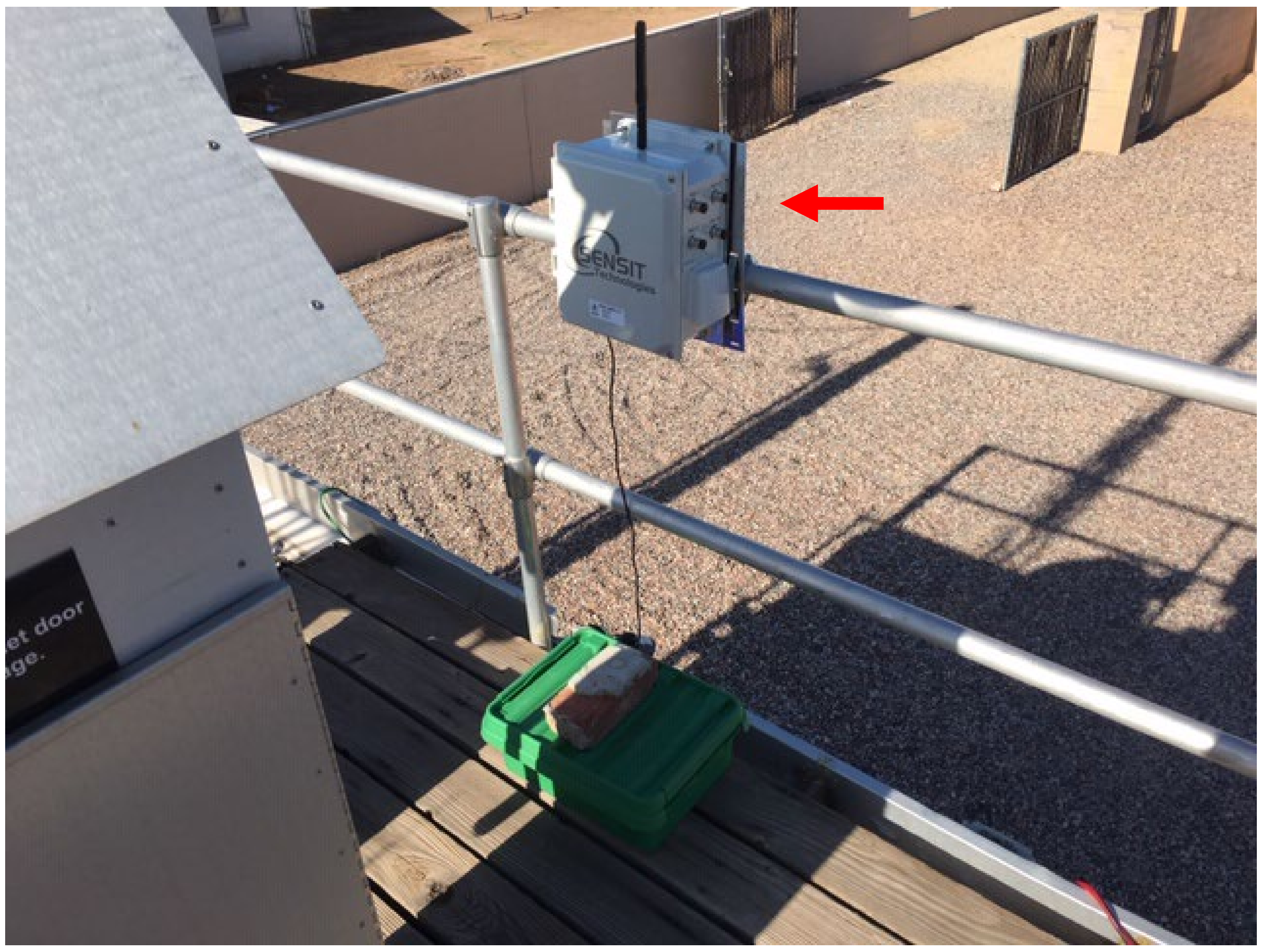

Figure 2: West Phoenix Monitoring Station

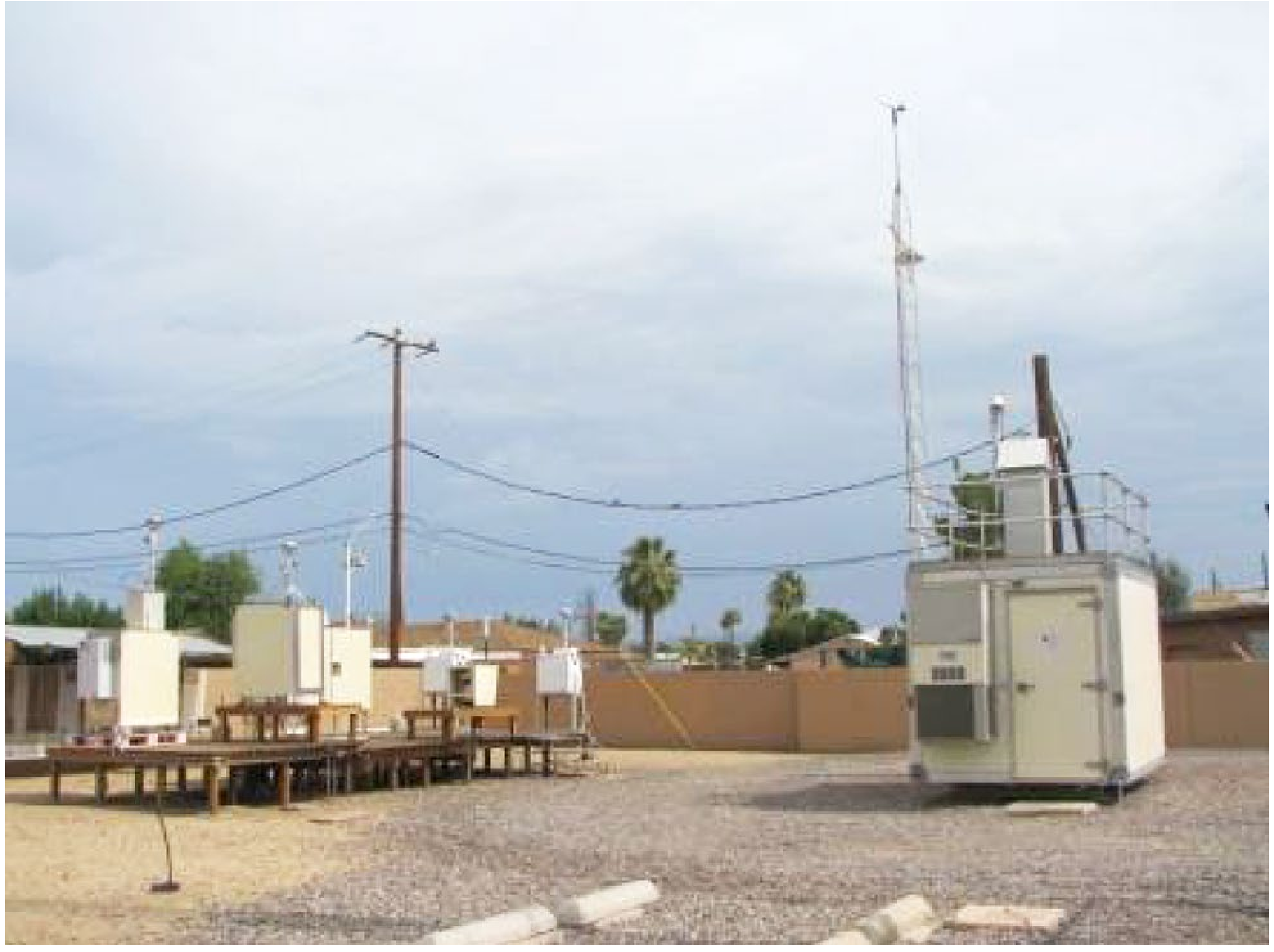

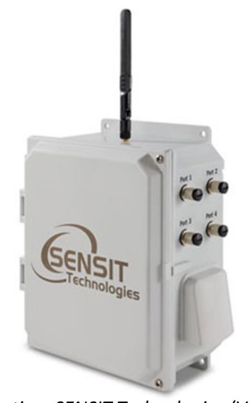

Image Attribution: SENSIT Technologies (Valparaiso, IN)

Data Storage and Transmission Method

The SENSIT RAMP was configured to record data at a 15-second sampling interval. Data are stored as daily text files (.txt format) on an onboard MicroSD card. Data files were obtained weekly via SD cards. Each field site operator was provided two labeled MicroSD cards for sensor units that they used to swap out each week. Data from the collected card was then read and processed off-site.

Data Correction Approach

SENSIT RAMP units were pre-configured by the manufacturer with a linear correction (i.e., concentration gain = 1.0 and offset = -5.0 ppbv). These presets reflect out-of-the box performance and were not modified by EPA prior or during testing.

After acquisition, the raw data was processed using the *sensortoolkit* python code library (v0.8.3b2). A continuous data set at the recorded sampling frequency was written to a .csv file. 1-hour averaged data sets were generated using a 75% completeness threshold and saved as separate .csv files. Outliers were **not** removed from data sets in order to assess "out-of-the-box" sensor performance.

The duration of the warm-up period required for sensor measurements to equilibrate was determined during bench-top testing (additional detail in pre-deployment observations) to be approximately 2 hours. Data recorded during warm up periods has been removed from data sets.

Issues Encountered

<u>Pre-deployment observations</u>

- *Changing logging interval*: SENSIT RAMP units were received without documentation or manuals. After communicating the need to change default settings (logging interval and time zone) with the manufacturer, a draft user's manual and a USB cable were supplied. With the use of this USB cable, instrument settings could be changed, and real-time data could be logged using a serial communication software (CoolTerm, v.1.5.0). Because the sensor did not record data at the top of every minute, the RAMP was configured to record data at 15-second intervals so that the data could be averaged more closely to complete minutes.
- *Gas Sensor Warmup*: Prior to deployment, RAMP units were collocated in a bench-top evaluation to verify operational status and determine the extent of data invalidity (i.e., determine equilibration period) after an initial start-up event. The recorded response for parameters measured by the RAMP suggests that the gas sensors (CO, NO, NO_2, O_3) required approximately a 2-hour equilibration period, while the remaining sensors (temperature, relative humidity, particulate matter) did not require any equilibration period.

<u>Field observations and sensor data flags</u>

The following table contains data flags describing events that were encountered during the testing period. A sampling interval abnormality was reported on 10/25/2019, during which the unit deviated from its configured 15-second sampling interval, and instead logged an interval lasting approximately 8 hours. The cause of this anomalous interval is unknown. Following this event, the sensor logged samples at regular 15-second intervals.

Start Time (UTC)	End Time (UTC)	Sensor Serial ID	Parameters Impacted	Flag
2019-10-11 07:53:00-07:00	2019-10-11 07:53:00-07:00	RAM_01	ALL	3.0-Intentional shutdown for data collection
2019-10-11 07:53:00-07:00	2019-10-11 08:01:00-07:00	RAM_01	ALL	5.0-Operator working near device (scheduled site visit)
2019-10-17 13:18:00-07:00	2019-10-17 13:25:00-07:00	RAM_01	ALL	5.0-Operator working near device (scheduled site visit)
2019-10-17 13:18:00-07:00	2019-10-17 13:18:00-07:00	RAM_01	ALL	3.0-Intentional shutdown for data collection
2019-10-25 07:40:00-07:00	2019-10-25 07:47:00-07:00	RAM_01	ALL	6.0-Sampling interval abnormality
2019-10-25 07:50:00-07:00	2019-10-25 07:52:00-07:00	RAM_01	ALL	5.0-Operator working near device (scheduled site visit)
2019-10-31 09:14:00-07:00	2019-10-31 09:14:00-07:00	RAM_01	ALL	3.0-Intentional shutdown for data collection
2019-10-31 09:14:00-07:00	2019-10-31 09:20:00-07:00	RAM_01	ALL	5.0-Operator working near device (scheduled site visit)

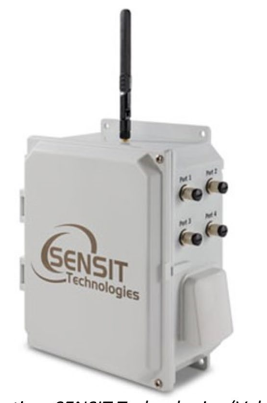

Image Attribution: SENSIT Technologies (Valparaiso, IN)

Description of Data Flags

AQS

The U.S. EPA's Air Quality System (AQS) is the Agency's primary ambient air monitoring data archive. A comprehensive list of data flags that are recorded alongside AQS data sets, referred to by U.S. EPA as 'qualifiers', can be found at the following link: https://aqs.epa.gov/aqsweb/documents/codetables/qualifiers.html

AirNow-Tech

AirNow-Tech is an additional ambient air monitoring data service maintained by U.S. EPA and is commonly used by monitoring agencies to upload and validate monitoring data. Data which have yet to be QC'd for inclusion in AQS as well as monitoring data sets which are not planned for inclusion in AQS are typically available for near real-time download from AirNow-Tech.

Invalidation of reference data: AQS qualifiers are organized by qualifier type, which indicates whether data logged alongside qualifier flags should be invalidated (set null). Qualifiers with type "Null Data Qualifier" are invalidated, and includes data logged during periods that coincide with QC checks (e.g., "BF-Precision/Zero/Span", "BJ- Operator Error", "BL - QA Audit", "AZ - QC Audit") among other events such as power outages. Data logged alongside qualifiers with type "Quality Assurance Qualifiers" are not invalidated and are included in this analysis (e.g., concentrations less than the federal MDL for the reference monitor "MD – Value less than MDL", QA reviewed values "Validated Value").

Data Flags Recorded During Testing

FRM/FEM Monitor	Timestamp (UTC)	Flag
Teledyne Advanced Pollution Instrumentation T400	2019-10-14 15:00:00+0000	AX - Precision Check
	2019-10-24 14:00:00+0000	BF - Precision/Zero/Span
	2019-10-24 15:00:00+0000	BC - Multi-point Calibration
	2019-10-29 15:00:00+0000	AX - Precision Check
	2019-11-06 14:00:00+0000	BF - Precision/Zero/Span

Meteorological Instrument	Timestamp (UTC)	Flag
MetOne Temperature Monitor (Data acquired via AirNow-Tech)	2019-10-17 15:00:00+0000 to 2019-10-17 16:00:00+0000	9 - Invalid

Testing Report - O_3 Base Testing

Aeroqual AQY

This report reflects out-of-the-box performance

Initial Base Testing - Denver, CO
U.S. Environmental Protection Agency
Office of Research and Development
PI: Clements.Andrea@epa.gov
919-541-1363
August 2019—September 2019

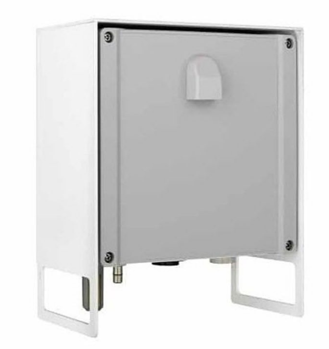

Image Attribution: Aeroqual Limited (Auckland, New Zealand)

Deployment Details

Testing Organization and Site Information	
Testing organization (Name, Organization type, Contact website)	U.S. Environmental Protection Agency - Office of Research and Development<br>Federal Government<br>Air Sensor Toolbox | U.S. EPA Website
Testing location (City, State, Latitude and Longitude)	La Casa<br>Denver, CO<br>39.779429, -105.005174
AQS site ID	08 - 031 - 0026
Sampling timeframe (MM-DD-YY)	08-17-19 to 09-16-19
Sensor data source	Aeroqual Cloud
Reference data source	AQS API download

Sensor Information			
Manufacturer, model	Aeroqual AQY		
Device firmware version	1.14.2		
Sampling time interval	1-minute		
Sensor serial numbers	AQY_01		
Issues encountered during deployment?	☐	No Issues	

FRM/FEM Information	
Manufacturer, model, designation	Teledyne API 400E FEM
Sampling time interval	1-hour averaging
Date of calibration	As required by 40 CFR Part 58 and the CO Regional Monitoring Site QAPP maintained by CDPHE
Date of one-point QC check	Every two weeks as required by 40 CFR Part 58 Appendix A 3.1.1
Description, date(s) of maintenance activities	N/A

Time Series Plot: 1-hour averaged O_3

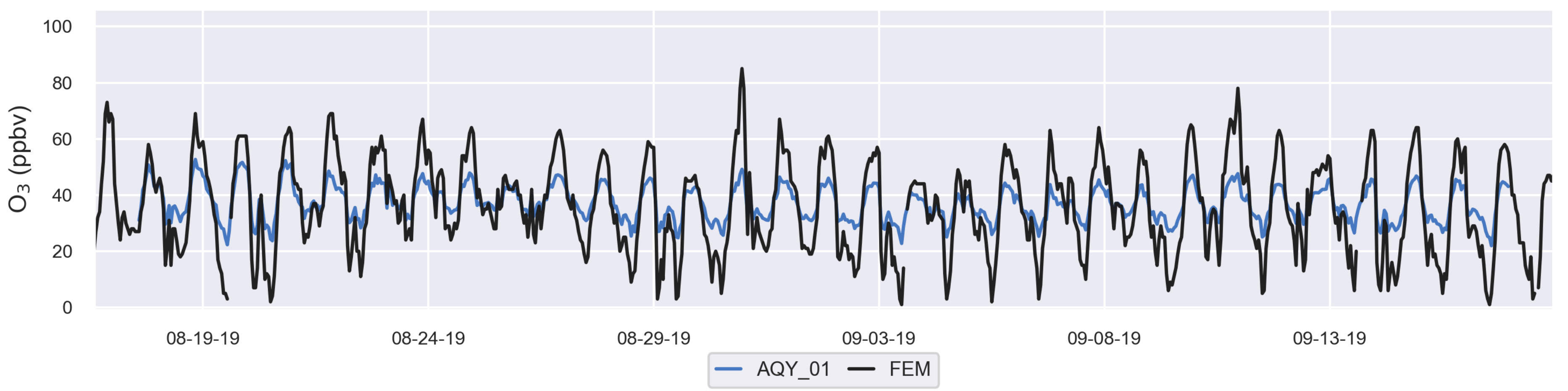

Range and average of FRM/FEM concentrations over duration of base test (ppbv)	[1-hr] 1.0-85.0, avg: 36.0,<br>[Rolling 8-hr] 10.5-66.1, avg: 36.1
Number of 1-hr periods in FRM/FEM monitor measurements with a goal concentration ≥ 60 ppbv	52

Scatter Plot: Comparison to FRM/FEM

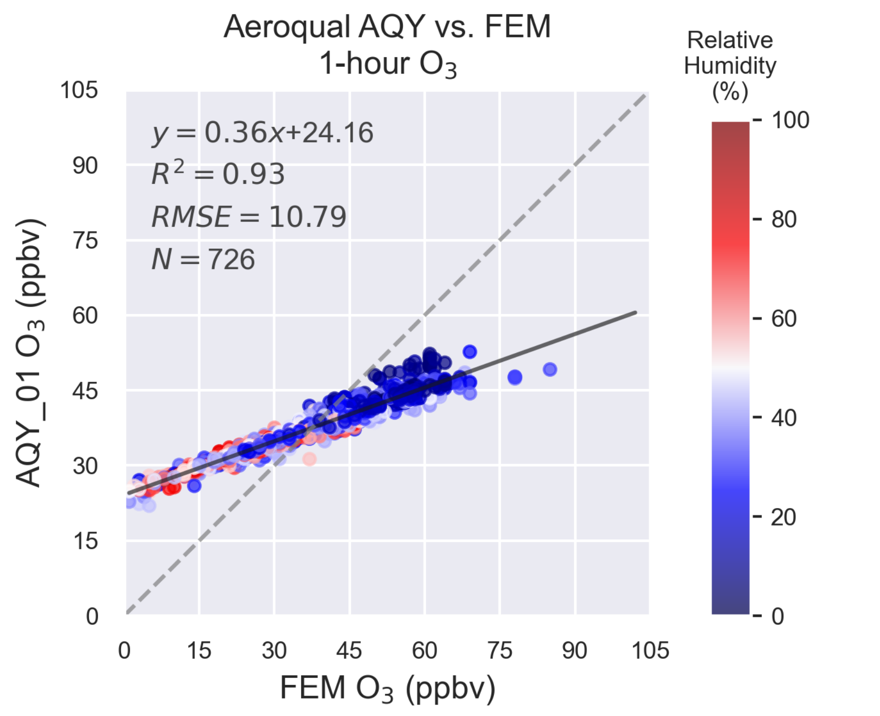

Performance Metrics

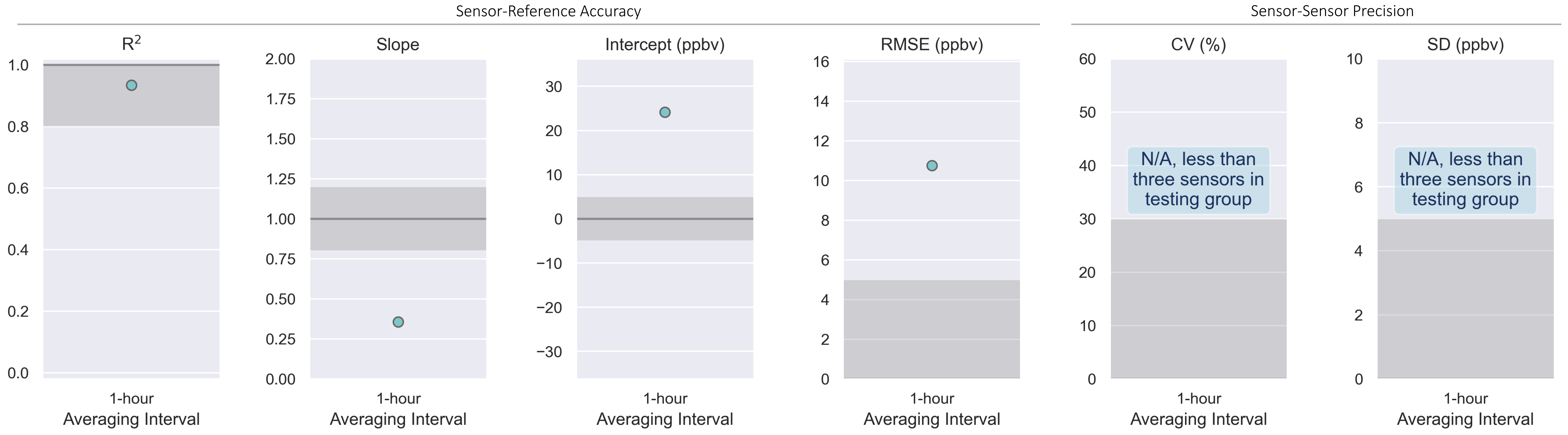

Meteorological Conditions During Deployment

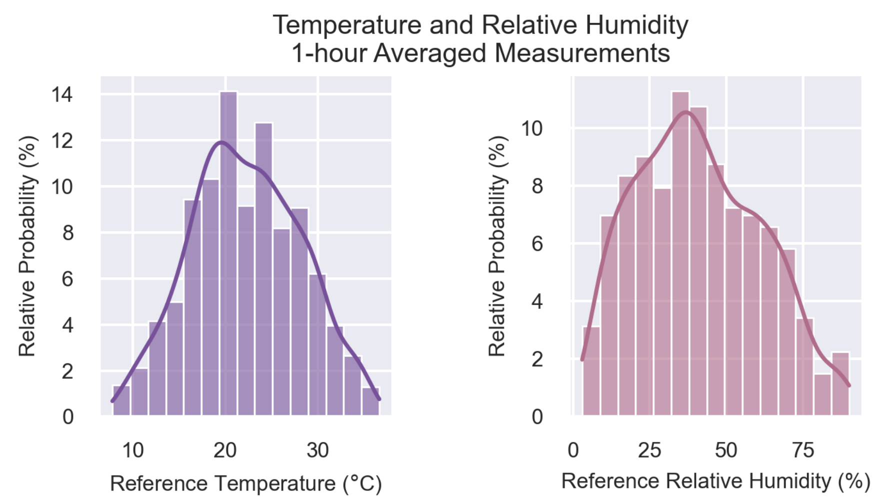

Number of 1-hr periods outside sensor manufacturer-listed temperature operational range (-10 to 40 °C)	0
Number of 1-hr periods outside sensor manufacturer-listed relative humidity operational range (no operational range specified)	-

Meteorological Influence

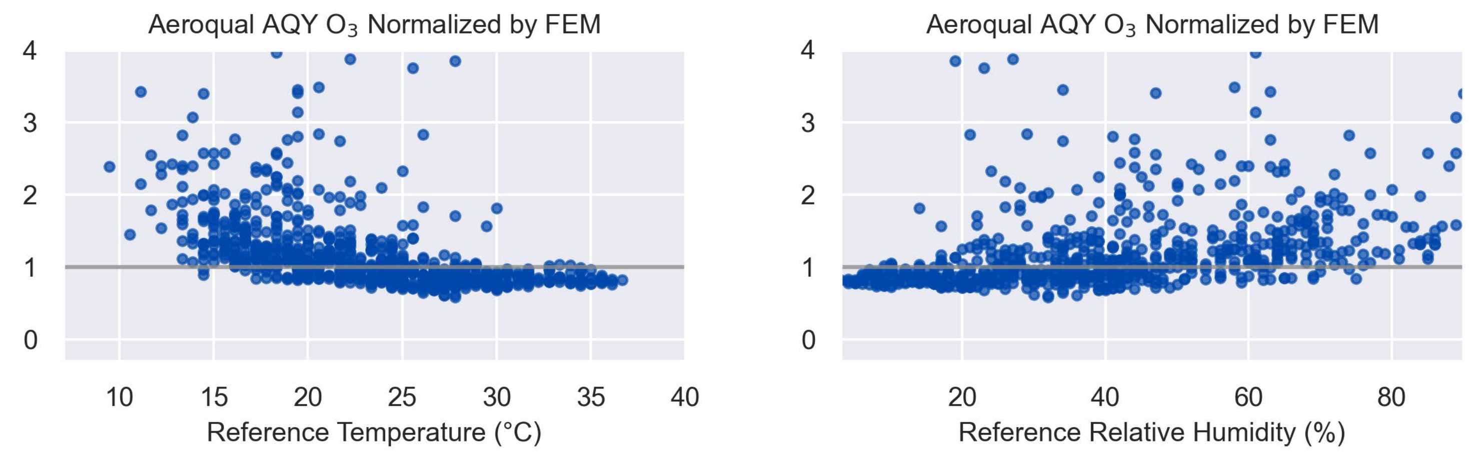

Mean number of paired, normalized concentration and temperature values (1-hr averages)	742
Mean number of paired, normalized concentration and relative humidity values (1-hr averages)	742

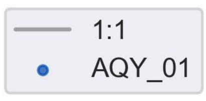

FEM data <5 ppbv (Federal MDL for the 400E) has been remove (Meteorological Influence section only)

Testing Report - O_3 Base Testing

Aeroqual AQY

This report reflects out-of-the-box performance

Initial Base Testing - Denver, CO
U.S. Environmental Protection Agency
Office of Research and Development
PI: Clements.Andrea@epa.gov
919-541-1363
August 2019—September 2019

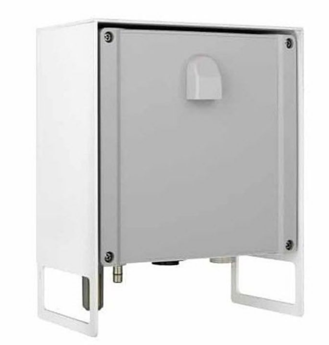

Image Attribution: Aeroqual Limited (Auckland, New Zealand)

Tabular Statistics

Sensor-FRM/FEM Correlation

	Bias and Linearity			Data Quality	
	R^2	Slope	Intercept (ppbv)	Uptime (%)	Number of paired sensor and reference concentration values*
	1-Hour ●	1-Hour ○	1-Hour ○	1-Hour ●	1-Hour
Metric Target Range	≥ 0.80	1.0 ± 0.20	-5 ≤ b ≤ 5	75%*	-
Sensor AQY_01	0.93	0.36	24.16	100	726

	Error
	RMSE (ppbv)
	1-Hour ☆
Metric Target Range	≤ 5.0
Deployment Value	10.8

Device-specific metrics (computed for each sensor in evaluation)
○ Metric value for none of devices tested falls within the target range
● Metric value for one of devices tested falls within the target range

Sensor-Sensor Precision[1]

	Precision (between collocated sensors)		Data Quality
	CV (%)	SD (ppbv)	Number of paired sensor and reference concentration values*
	1-Hour ☆	1-Hour ☆	1-Hour
Metric Target Range	≤ 30.0	≤ 5.0	-
Deployment Value	-	-	-

[1]Precision statistics are computed for evaluations with at least three collocated sensor units. Metric values are left blank for evaluations with two or fewer sensor units.

Single-valued metrics (computed via entire evaluation dataset)
☆ Indicates that the metric value is not within the target range
★ Indicates that the metric value is within the target range

*This value is only a recommendation for ensuring data quality and is not included in the list of target values discussed in Section 4 of the Performance Testing Protocols, Metrics, and Target Values for Ozone Air Sensors document.

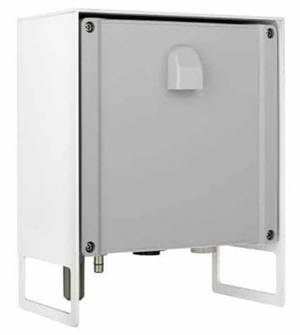

Image Attribution: Aeroqual Limited (Auckland, New Zealand)

Supplemental Information

Abbreviations used in Supplemental Information

FRM	Federal Reference Method
FEM	Federal Equivalent Method
SOP	Standard Operating Procedure
QAPP	Quality Assurance Project Plan
QC	Quality Control

Supplemental Documentation	Attached	Description & URL or file path to documentation
Field observations and sensor data flags	☑	See CO-AQY-Page 5 of this testing report
Maintenance logs	☐	No logs recorded during testing
Standard operating procedure(s)	☐	U.S. EPA Office Of Research and Development SOP available upon request
Photos of equipment setup and testing	☑	See CO-AQY-Page 4 of this testing report
Product specifications sheet(s)	☑	See Appendix C, “Spec_Sheet_Aeroqual_AQY.pdf”*
Product manual(s)	☑	See Appendix C, “Manual_Aeroqual_AQY.pdf”*
Data storage and transmission method	☑	See CO-AQY-Page 5 of this testing report
Data correction approach	☑	See CO-AQY-Page 5 of this testing report
Issues encountered	☑	See CO-AQY-Page 5 of this testing report. No issues were encountered during testing; however, various issues were faced during the pre-deployment phase.
Data analysis/correction scripts and version	☑	Averaging and processing of data, calculation of performance metrics, and generation of figures and other supplementary material for analysis were obtained using Python 3.9.7 with the packages sensortoolkit v0.8.3b2, pandas 1.3.5, NumPy 1.21.2, Matplotlib 3.5.0, statsmodels 0.13.0, and seaborn 0.11.2. All packages are available from the Python Package Index (PyPI) at https://pypi.org. The integrated development environment (IDE) Spyder 5.1.5 was used for scripting and data visualization. Version control for the Python base, packages, and IDE were all managed by conda 4.11.0.
Air Monitoring Station QAPP	☐	U.S. EPA Office Of Research and Development QAPP available upon request
Summary of FRM/FEM monitor QC checks	☑	See CO-AQY-Page 6 of this testing report
Manufacturer website for FRM/FEM monitor	☐	Discontinued model, not available on manufacturer website
FRM/FEM monitor manual	☑	See Appendix B, “Spec_Sheet_TeledyneAPI_400E.pdf”
FRM/FEM monitor specifications sheet(s)	☑	See Appendix B, “Manual_TeledyneAPI_400E.pdf”
Other documents	☑	Manufacturer notice of AQY sales on hold

*As of 3/18/2021, the manufacturer of the AQY has placed sales of a similar unit on hold. Documentation for the AQY is currently unavailable from the manufacturer’s website.

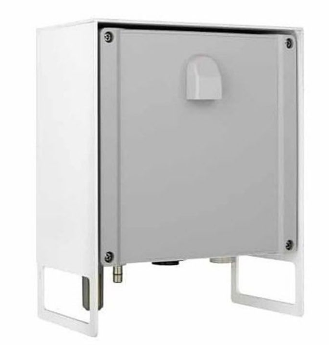

Image Attribution: Aeroqual Limited (Auckland, New Zealand)

Supplemental Information: Photos of Testing Site and Equipment Setup

Site Description:

The La Casa site was established in January of 2013 as a replacement for the Denver Municipal Animal Shelter (DMAS) site when a land use change forced the relocation of the site. The La Casa location has been established as the NCore site for the Denver Metropolitan area. Measurements include trace gas/precursor-level CO analyzer, and a NO_y analyzer, in addition to the trace level SO_2, O_3, meteorology, and particulate monitors. The site represents a population-oriented neighborhood scale monitoring area.

Figure 1: Aeroqual AQY sensor (indicated by red arrow) attached to metal railing atop the sampling shelter at the monitoring site.

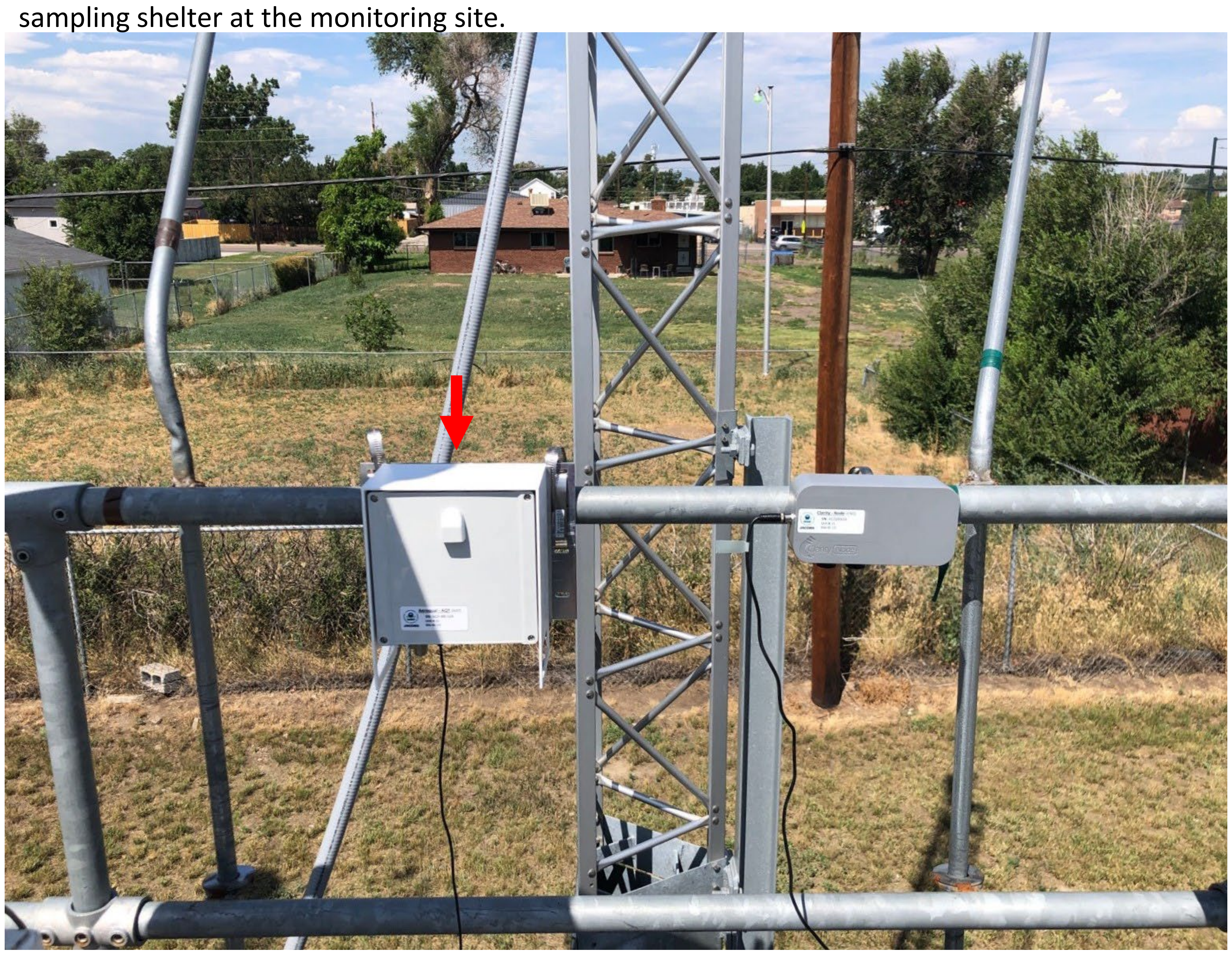

Figure 2: La Casa Monitoring Station sampling shelter, side view

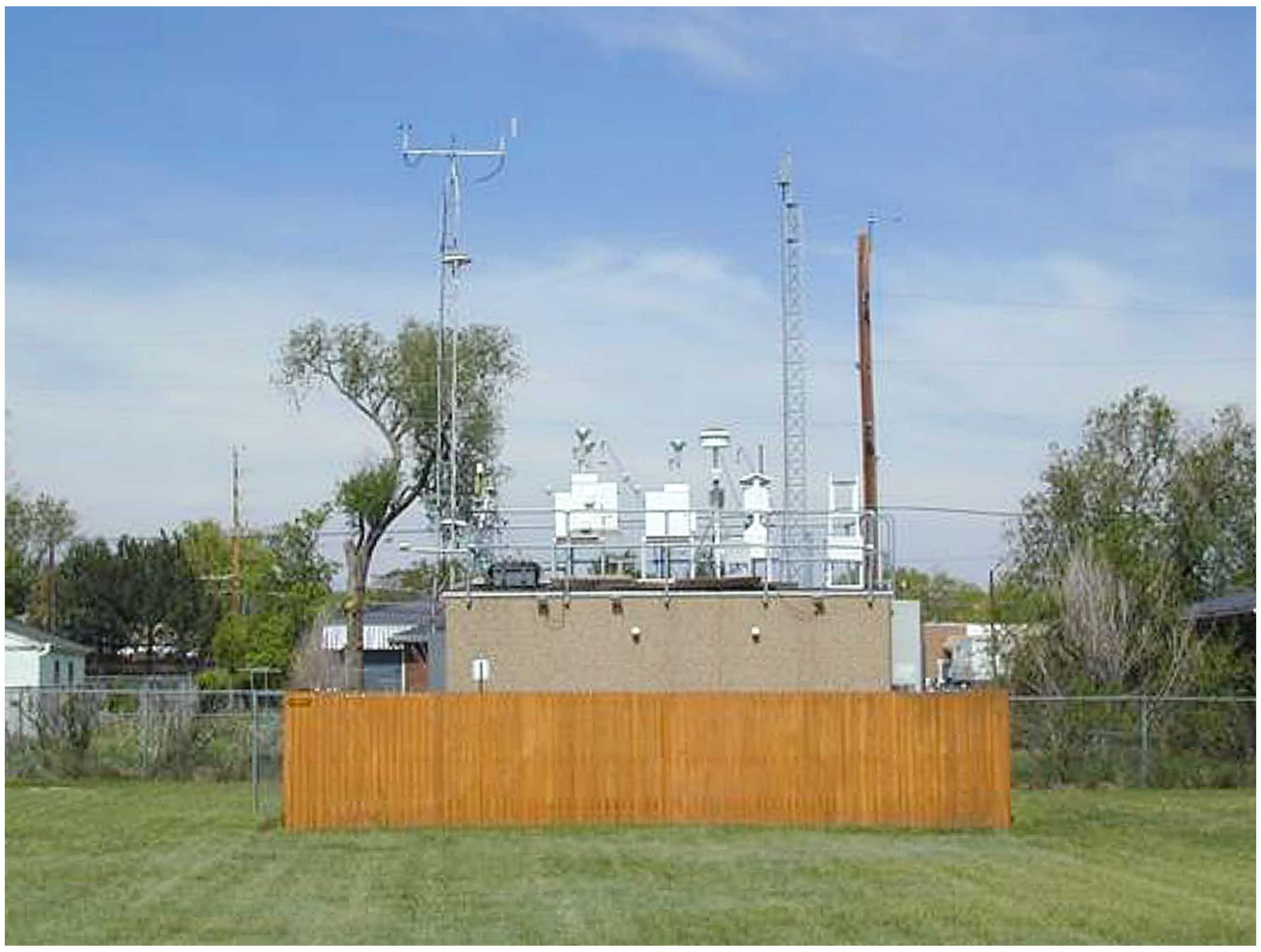

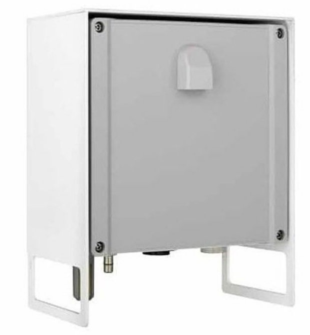

Image Attribution: Aeroqual Limited (Auckland, New Zealand)

Supplemental Information: Data Storage, Correction Approach, and Issues Encountered

Data Storage and Transmission Method

As part of CRADA #934-16 between Aeroqual and US EPA, Aeroqual supported data streaming. SIM cards were installed and data flowed to the Aeroqual Cloud. The 1-minute raw data was acquired weekly using the Aeroqual Cloud (*last accessed 5/11/22*) user interface (UI). The AQY has an internal data storage USB flash drive as a data backup, however access requires software proprietary to Aeroqual.

Data Correction Approach

This evaluation report reflects "out-of-the-box" performance of the AQY. The manufacturer provides a procedure by which local collocation (sensor operated along side an FRM/FEM) data can be collected, a gain (slope) and offset (intercept) determined, and parameters entered into the Aeroqual Cloud user interface to be applied to all subsequently collected data. This procedure and feature was **not** used prior to this evaluation. Prospective consumers may get different performance from this device if they utilize this feature.

After acquisition, the raw data was processed using the *sensortoolkit* python code library (v0.8.3b2). A continuous data set at the recorded sampling frequency was written to a .csv file. 1-hour averaged data sets were generated using a 75% completeness threshold and saved as separate .csv files. Outliers were **not** removed from data sets in order to assess "out-of-the-box" sensor performance.

The duration of the warm-up period required for sensor measurements to equilibrate was determined from field data to be 10 minutes. Warm up periods were considered to occur following any power outage to sensor units, either due to routine field visits or unscheduled site power outages. Data recorded during warm up periods has been removed from data sets.

Issues Encountered

Pre-deployment observations

- *Timestamp inaccuracies*: During pre-deployment, the AQY devices did not properly sync timestamps with the onboard Real-Time Clock. Connecting the units to the internet by cellular or Wi-Fi allowed the unit to sync with internet time and resulted in proper timestamps.

Field observations and sensor data flags

The Aeroqual AQY was deployed at the La Casa monitoring site on 7/31/2019. The Aeroqual unit operated nominally during the testing period and did not require replacement or repair.

Description of Data Flags

AQS

The U.S. EPA's Air Quality System (AQS) is the Agency's primary ambient air monitoring data archive. A comprehensive list of data flags that are recorded alongside AQS data sets, referred to by U.S. EPA as 'qualifiers', can be found at the following link: https://aqs.epa.gov/aqsweb/documents/codetables/qualifiers.html

Invalidation of reference data: AQS qualifiers are organized by qualifier type, which indicates whether data logged alongside qualifier flags should be invalidated (set null). Qualifiers with type "Null Data Qualifier" are invalidated, and includes data logged during periods that coincide with QC checks (e.g., "BF-Precision/Zero/Span", "BJ- Operator Error", "BL - QA Audit", "AZ - QC Audit") among other events such as power outages. Data logged alongside qualifiers with type "Quality Assurance Qualifiers" are not invalidated and are included in this analysis (e.g., concentrations less than the federal MDL for the reference monitor "MD – Value less than MDL", QA reviewed values "Validated Value").

Data Flags Recorded During Testing

FRM/FEM Monitor	Timestamp (UTC)	Flag
Teledyne Advanced Pollution Instrumentation 400E (Acquired via AQS)	2019-08-19 14:00:00+0000	AX - Precision Check
	2019-09-03 14:00:00+0000	AX - Precision Check
	2019-09-13 15:00:00+0000 to 2019-09-13 16:00:00+0000	AZ - QC Audit

Meteorological Instrument	Timestamp (UTC)	Flag
MetOne Temperature Monitor (Acquired via AQS)	2019-09-13 17:00:00+0000 to 2019-09-13 18:00:00+0000	AZ - QC Audit
Climatronics Relative Humidity Monitor (Acquired via AQS)	2019-09-13 17:00:00+0000 to 2019-09-13 18:00:00+0000	AZ - QC Audit

Testing Report - O_3 Base Testing

SENSIT RAMP

This report reflects out-of-the-box performance

Initial Base Testing - Denver, CO

U.S. Environmental Protection Agency
Office of Research and Development
PI: Clements.Andrea@epa.gov
919-541-1363
August 2019—September 2019

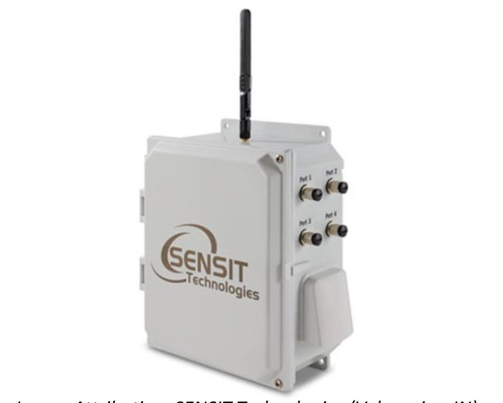

Image Attribution: SENSIT Technologies (Valparaiso, IN)

Deployment Details

Testing Organization and Site Information	
Testing organization (Name, Organization type, Contact website)	U.S. Environmental Protection Agency - Office of Research and Development<br>Federal Government<br>Air Sensor Toolbox | U.S. EPA Website
Testing location (City, State, Latitude and Longitude)	La Casa<br>Denver, CO<br>39.779429, -105.005174
AQS site ID	08 - 031 - 0026
Sampling timeframe (MM-DD-YY)	08-14-19 to 09-11-19
Sensor data source	Onboard MicroSD card
Reference data source	AQS API download

Sensor Information			
Manufacturer, model	SENSIT RAMP		
Device firmware version	190313_AQ_v9.30		
Sampling time interval	15-seconds		
Sensor serial numbers	RAM_01		
Issues encountered during deployment?	☐	No Issues	

FRM/FEM Information	
Manufacturer, model, designation	Teledyne API 400E FEM
Sampling time interval	1-hour averaging
Date of calibration	As required by 40 CFR Part 58 and the CO Regional Monitoring Site QAPP maintained by CDPHE
Date of one-point QC check	Every two weeks as required by 40 CFR Part 58 Appendix A 3.1.1
Description, date(s) of maintenance activities	N/A

Time Series Plot: 1-hour averaged O_3

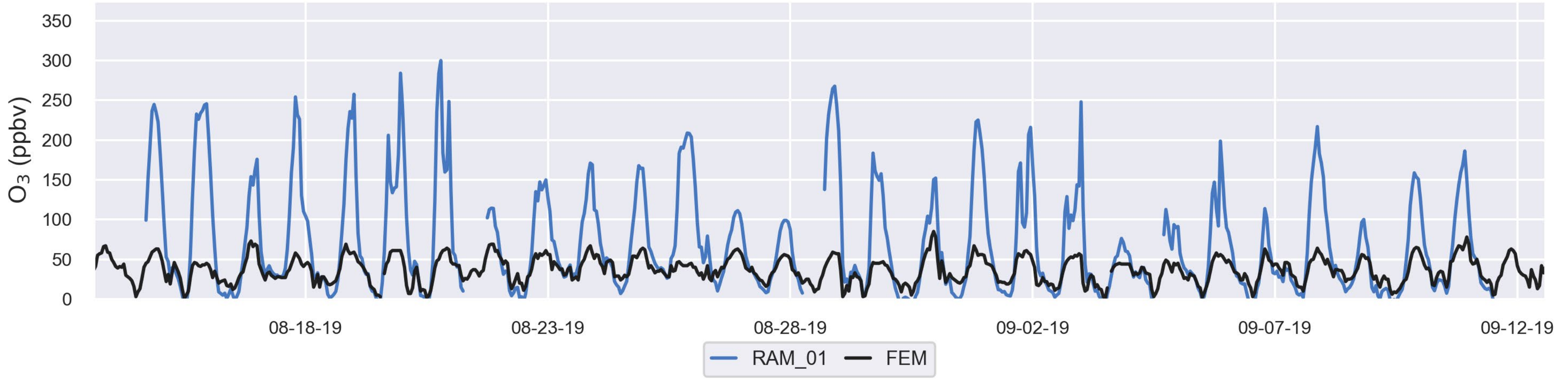

Range and average of FRM/FEM concentrations over duration of base test (ppbv)	[1-hr] 1.0-85.0, avg: 36.3,<br>[Rolling 8-hr] 12.2-66.1, avg: 36.5
Number of 1-hr periods in FRM/FEM monitor measurements with a goal concentration ≥ 60 ppbv	52

Scatter Plot: Comparison to FRM/FEM

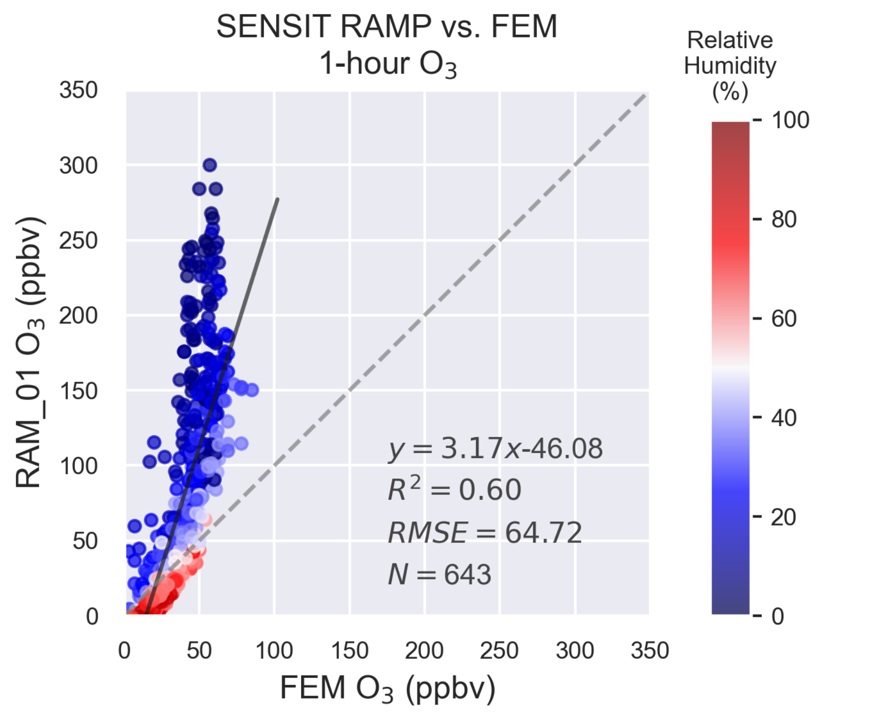

Performance Metrics

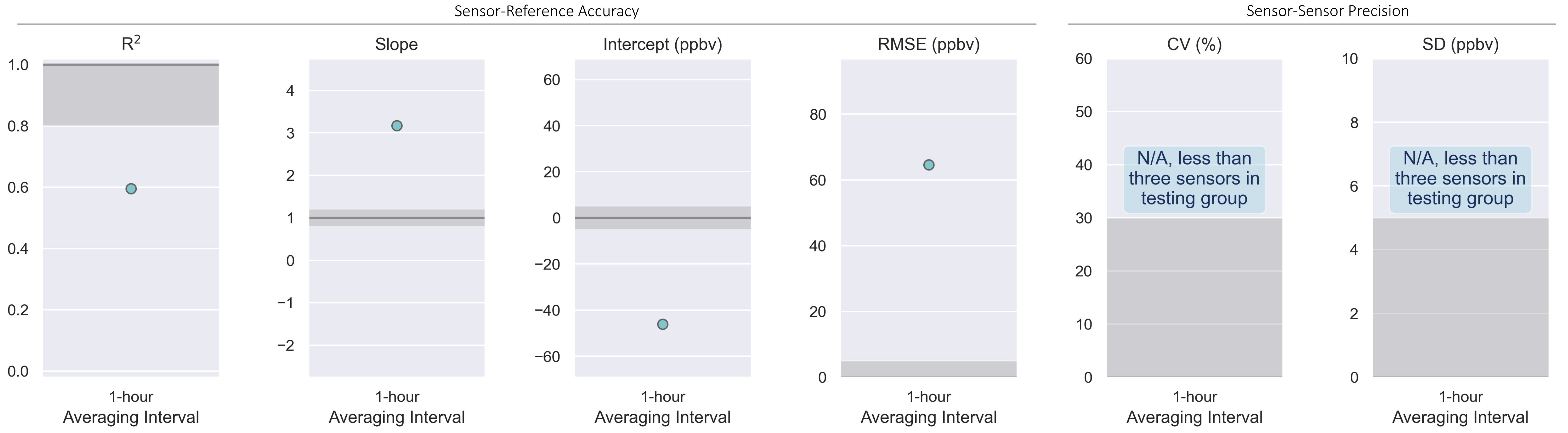

Meteorological Conditions During Deployment

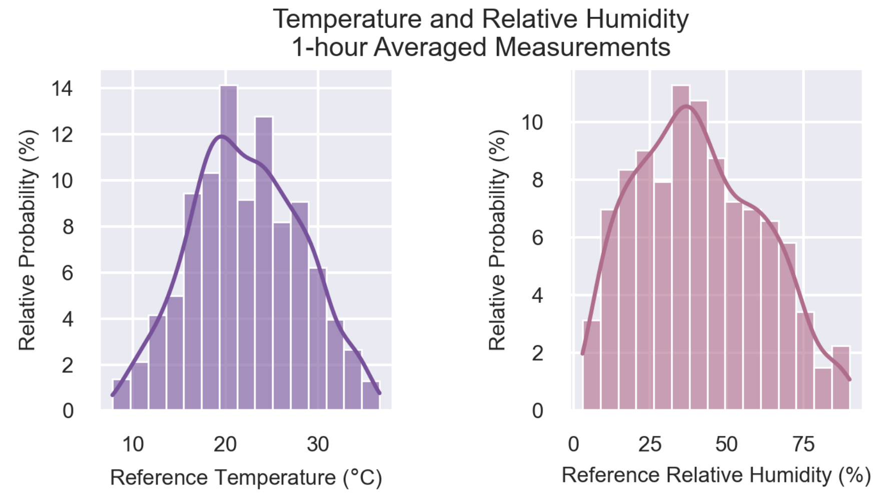

Number of 1-hr periods outside sensor manufacturer-listed temperature operational range (-20 to 50 °C)	0
Number of 1-hr periods outside sensor manufacturer-listed relative humidity operational range (no operational range specified)	-

Meteorological Influence

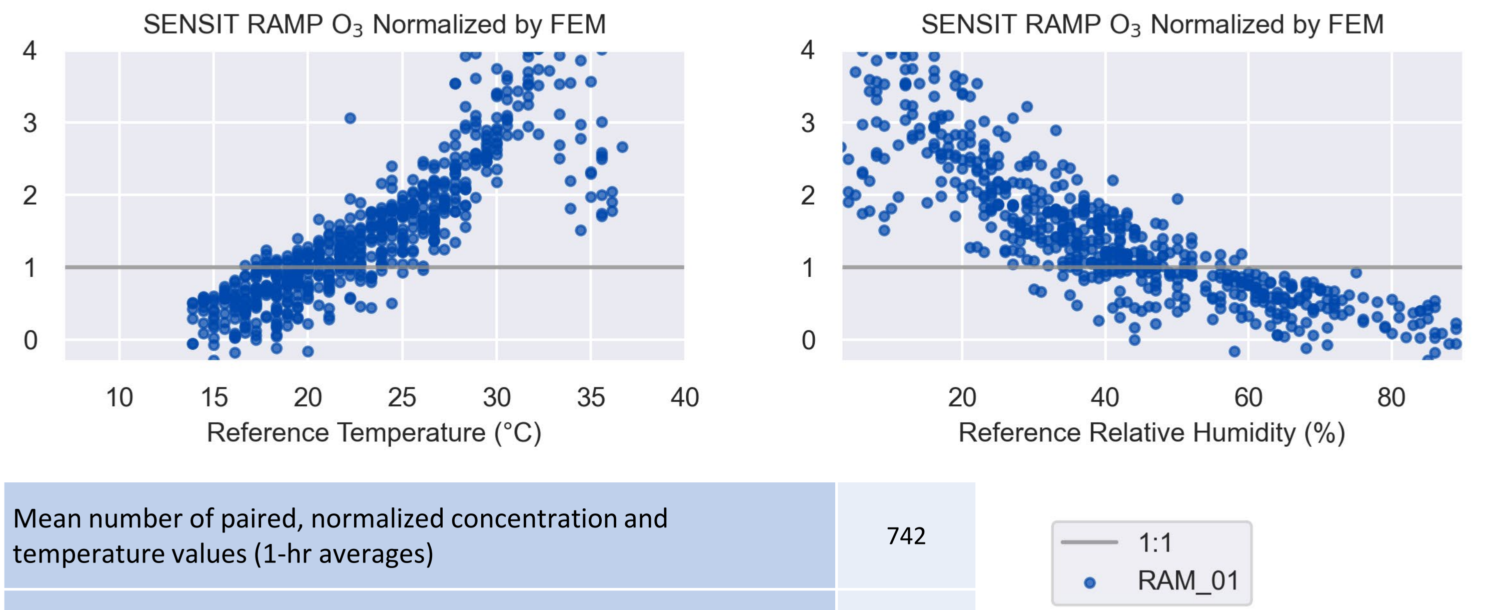

Mean number of paired, normalized concentration and temperature values (1-hr averages)	742
Mean number of paired, normalized concentration and relative humidity values (1-hr averages)	742

FEM data <5 ppbv (Federal MDL for the 400E) has been remove (Meteorological Influence section only)

Testing Report - O_3 Base Testing

SENSIT RAMP

This report reflects out-of-the-box performance

Initial Base Testing - Denver, CO

U.S. Environmental Protection Agency
Office of Research and Development
PI: Clements.Andrea@epa.gov
919-541-1363

August 2019—September 2019

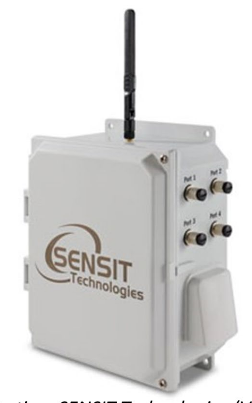

Image Attribution: SENSIT Technologies (Valparaiso, IN)

Tabular Statistics

Sensor-FRM/FEM Correlation

	Bias and Linearity			Data Quality	
	R^2	Slope	Intercept (ppbv)	Uptime (%)	Number of paired sensor and reference concentration values*
	1-Hour ○	1-Hour ○	1-Hour ○	1-Hour ●	1-Hour
Metric Target Range	≥ 0.80	1.0 ± 0.20	-5 ≤ b ≤ 5	75%*	-
Sensor RAM_01	0.60	3.17	-46.14	97	651

	Error
	RMSE (ppbv)
	1-Hour ☆
Metric Target Range	≤ 5.0
Deployment Value	64.2

Device-specific metrics (computed for each sensor in evaluation)
- ○ Metric value for none of devices tested falls within the target range
- ● Metric value for one of devices tested falls within the target range

Sensor-Sensor Precision[1]

	Precision (between collocated sensors)		Data Quality
	CV (%)	SD (ppbv)	Number of paired sensor and reference concentration values*
	1-Hour ☆	1-Hour ☆	1-Hour
Metric Target Range	≤ 30.0	≤ 5.0	-
Deployment Value	-	-	-

[1]Precision statistics are computed for evaluations with at least three collocated sensor units. Metric values are left blank for evaluations with two or fewer sensor units.

Single-valued metrics (computed via entire evaluation dataset)
- ☆ Indicates that the metric value is not within the target range
- ★ Indicates that the metric value is within the target range

*This value is only a recommendation for ensuring data quality and is not included in the list of target values discussed in Section 4 of the Performance Testing Protocols, Metrics, and Target Values for Ozone Air Sensors document.

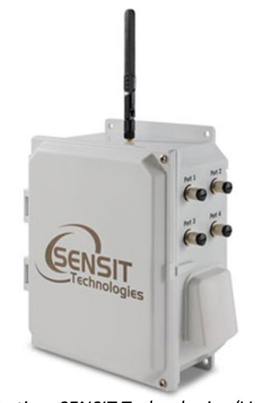

Image Attribution: SENSIT Technologies (Valparaiso, IN)

Supplemental Information

Abbreviations used in Supplemental Information

FRM	Federal Reference Method
FEM	Federal Equivalent Method
SOP	Standard Operating Procedure
QAPP	Quality Assurance Project Plan
QC	Quality Control

Supplemental Documentation	Attached	Description & URL or file path to documentation
Field observations and sensor data flags	☑	See CO-RAM-Page 5 of this testing report
Maintenance logs	☐	No logs recorded during testing
Standard operating procedure(s)	☐	U.S. EPA Office Of Research and Development SOP available upon request
Photos of equipment setup and testing	☑	See CO-RAM-Page 4 of this testing report
Product specifications sheet(s)	☑	See Appendix C, “Spec_Sheet_SENSIT_RAMP.pdf”
Product manual(s)	☑	See Appendix C, “Manual_SENSIT_RAMP.pdf”
Data storage and transmission method	☑	See CO-RAM-Page 5 of this testing report
Data correction approach	☑	See CO-RAM-Page 5 of this testing report
Issues encountered	☑	See CO-RAM-Page 6 of this testing report. No issues were encountered during testing; however, various issues were faced during the pre-deployment phase.
Data analysis/correction scripts and version	☑	Averaging and processing of data, calculation of performance metrics, and generation of figures and other supplementary material for analysis were obtained using Python 3.9.7 with the packages sensortoolkit v0.8.3b2, pandas 1.3.5, NumPy 1.21.2, Matplotlib 3.5.0, statsmodels 0.13.0, and seaborn 0.11.2. All packages are available from the Python Package Index (PyPI) at https://pypi.org. The integrated development environment (IDE) Spyder 5.1.5 was used for scripting and data visualization. Version control for the Python base, packages, and IDE were all managed by conda 4.11.0.
Air Monitoring Station QAPP	☐	U.S. EPA Office Of Research and Development QAPP available upon request
Summary of FRM/FEM monitor QC checks	☑	See CO-RAM-Page 6 of this testing report
Manufacturer website for FRM/FEM monitor	☐	Discontinued model, not available on manufacturer website
FRM/FEM monitor manual	☑	See Appendix B, “Spec_Sheet_TeledyneAPI_400E.pdf”
FRM/FEM monitor specifications sheet(s)	☑	See Appendix B, “Manual_TeledyneAPI_400E.pdf”
Other documents	☐	

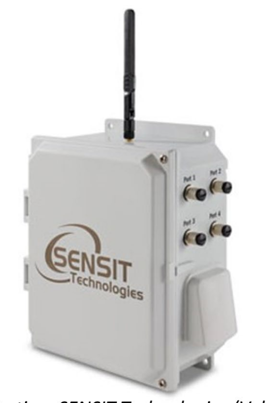

Image Attribution: SENSIT Technologies (Valparaiso, IN)

Supplemental Information: Photos of Testing Site and Equipment Setup

Site Description:
The La Casa site was established in January of 2013 as a replacement for the Denver Municipal Animal Shelter (DMAS) site when a land use change forced the relocation of the site. The La Casa location has been established as the NCore site for the Denver Metropolitan area. Measurements include trace gas/precursor-level CO analyzer, and a NO_y analyzer, in addition to the trace level SO_2, O_3, meteorology, and particulate monitors. The site represents a population-oriented neighborhood scale monitoring area.

Figure 1: SENSIT RAMP sensor (indicated by red arrow) attached to metal railing atop the sampling shelter at the monitoring site.

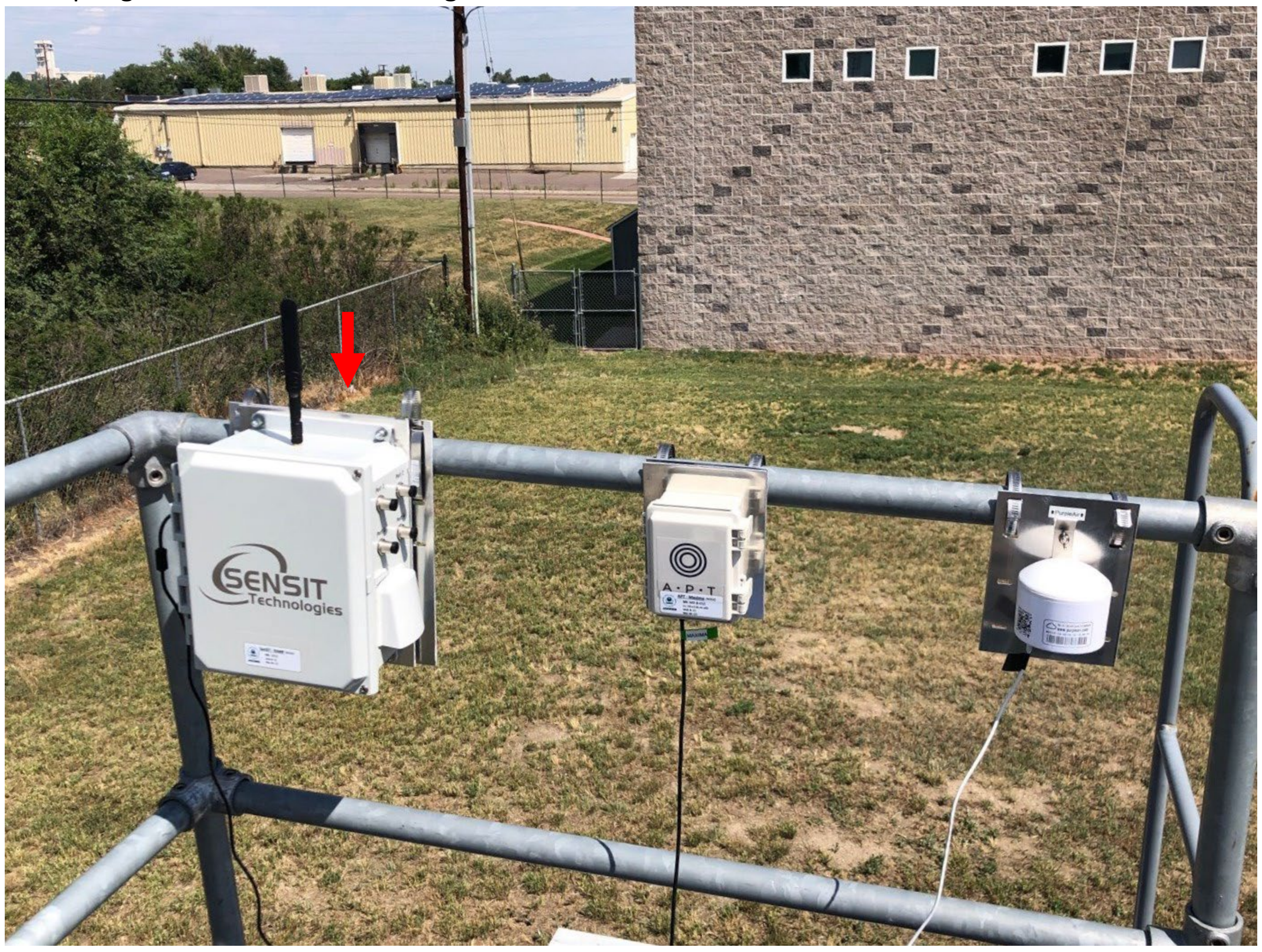

Figure 2: La Casa Monitoring Station sampling shelter, side view

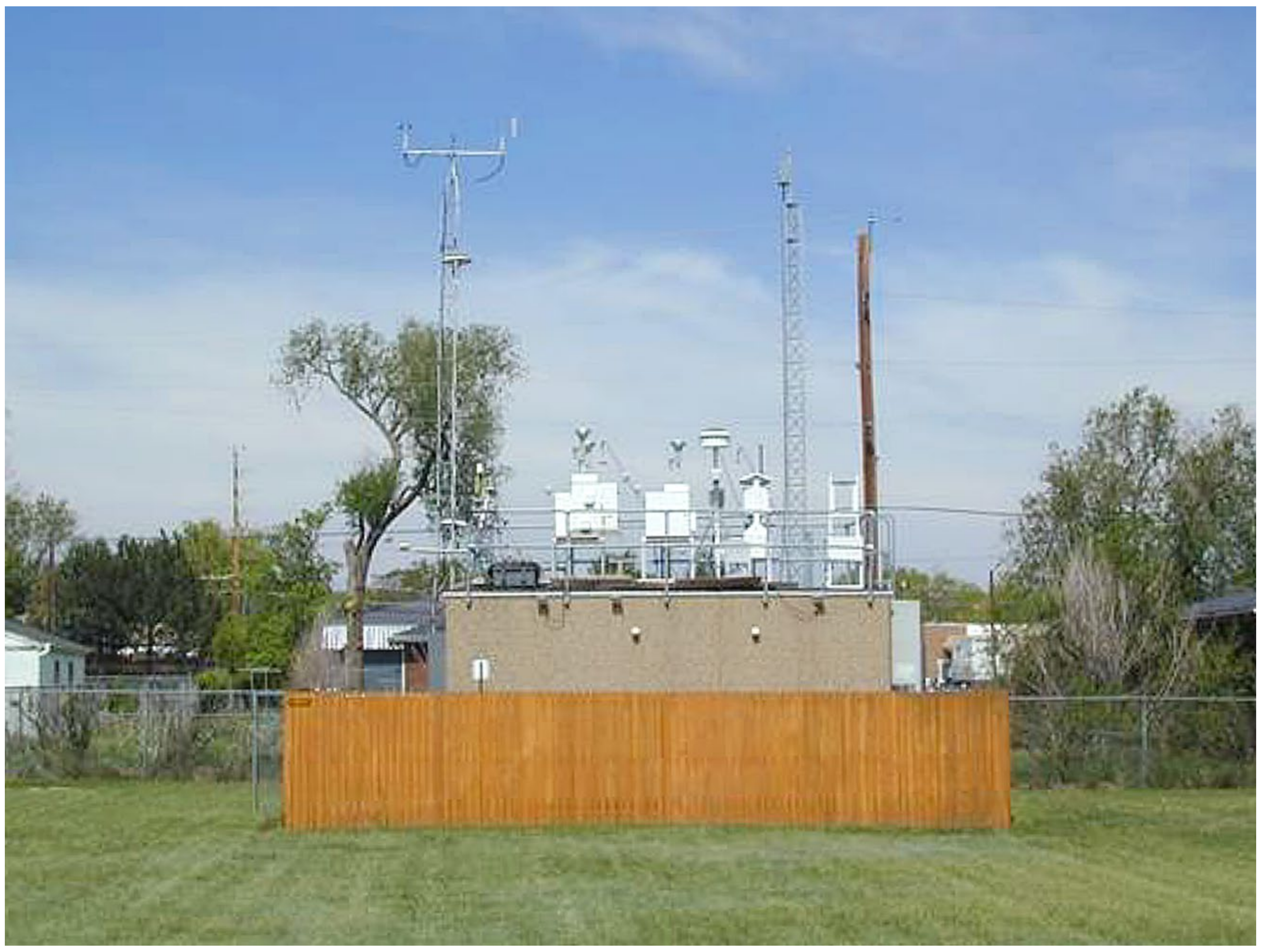

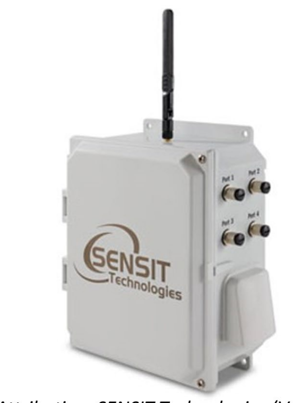

Image Attribution: SENSIT Technologies (Valparaiso, IN)

Supplemental Information: Data Storage, Correction Approach, and Issues Encountered

Data Storage and Transmission Method

The SENSIT RAMP was configured to record data at a 15-second sampling interval. Data are stored as daily text files (.txt format) on an onboard MicroSD card. Data files were obtained weekly via SD cards. Each field site operator was provided two labeled MicroSD cards for sensor units that they used to swap out each week. Data from the collected card was then read and processed off-site.

Data Correction Approach

SENSIT RAMP units were pre-configured by the manufacturer with a linear correction (i.e., concentration gain = 1.0 and offset = 0.0 ppbv). These presets reflect out-of-the box performance and were not modified by EPA prior or during testing.

After acquisition, the raw data was processed using the *sensortoolkit* python code library (v0.8.3b2). A continuous data set at the recorded sampling frequency was written to a .csv file. 1-hour averaged data sets were generated using a 75% completeness threshold and saved as separate .csv files. Outliers were **not** removed from data sets in order to assess "out-of-the-box" sensor performance.

The duration of the warm-up period required for sensor measurements to equilibrate was determined during bench-top testing (additional detail in pre-deployment observations) to be approximately 2 hours. Data recorded during warm up periods has been removed from data sets.

Issues Encountered

<u>Pre-deployment observations</u>

- *Changing logging interval*: SENSIT RAMP units were received without documentation or manuals. After communicating the need to change default settings (logging interval and time zone) with the manufacturer, a draft user's manual and a USB cable were supplied. With the use of this USB cable, instrument settings could be changed, and real-time data could be logged using a serial communication software (CoolTerm, v.1.5.0). Because the sensor did not record data at the top of every minute, the RAMP was configured to record data at 15-second intervals so that the data could be averaged more closely to complete minutes.
- *Gas sensor warmup*: Prior to deployment, RAMP units were collocated in a bench-top evaluation to verify operational status and determine the extent of data invalidity (i.e., determine equilibration period) after an initial start-up event. The recorded response for parameters measured by the RAMP suggests that the gas sensors (CO, NO, NO_2, O_3) required approximately a 2-hour equilibration period, while the remaining sensors (temperature, relative humidity, particulate matter) did not require any equilibration period.

<u>Field observations and sensor data flags</u>

The SENSIT RAMP was deployed at the La Casa monitoring site on 7/31/2019. The RAMP unit operated nominally during the testing period and did not require replacement or repair.

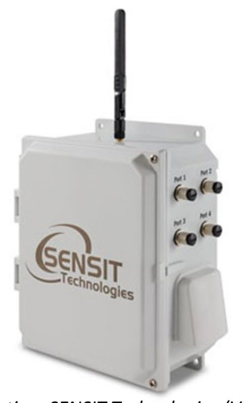

Image Attribution: SENSIT Technologies (Valparaiso, IN)

Description of Data Flags

AQS

The U.S. EPA's Air Quality System (AQS) is the Agency's primary ambient air monitoring data archive. A comprehensive list of data flags that are recorded alongside AQS data sets, referred to by U.S. EPA as 'qualifiers', can be found at the following link: https://aqs.epa.gov/aqsweb/documents/codetables/qualifiers.html

Invalidation of reference data: AQS qualifiers are organized by qualifier type, which indicates whether data logged alongside qualifier flags should be invalidated (set null). Qualifiers with type "Null Data Qualifier" are invalidated, and includes data logged during periods that coincide with QC checks (e.g., "BF-Precision/Zero/Span", "BJ- Operator Error", "BL - QA Audit", "AZ - QC Audit") among other events such as power outages. Data logged alongside qualifiers with type "Quality Assurance Qualifiers" are not invalidated and are included in this analysis (e.g., concentrations less than the federal MDL for the reference monitor "MD – Value less than MDL", QA reviewed values "Validated Value").

Data Flags Recorded During Testing

FRM/FEM Monitor	Timestamp (UTC)	Flag
Teledyne Advanced Pollution Instrumentation 400E (Acquired via AQS)	2019-08-19 14:00:00+0000	AX - Precision Check
	2019-09-03 14:00:00+0000	AX - Precision Check
	2019-09-13 15:00:00+0000 to 2019-09-13 16:00:00+0000	AZ - QC Audit

Meteorological Instrument	Timestamp (UTC)	Flag
MetOne Temperature Monitor (Acquired via AQS)	2019-09-13 17:00:00+0000 to 2019-09-13 18:00:00+0000	AZ - QC Audit
Climatronics Relative Humidity Monitor (Acquired via AQS)	2019-09-13 17:00:00+0000 to 2019-09-13 18:00:00+0000	AZ - QC Audit

Testing Report - O_3 Base Testing

Aeroqual AQY

This report reflects out-of-the-box performance

Initial Base Testing - Wilmington, DE

U.S. Environmental Protection Agency
Office of Research and Development
PI: Clements.Andrea@epa.gov
919-541-1363

July 2019—August 2019

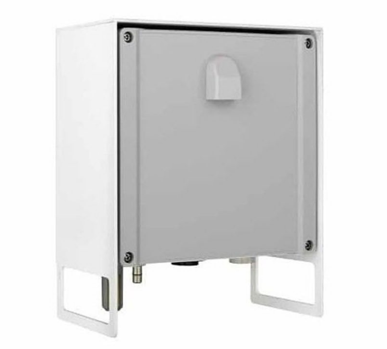

Image Attribution: Aeroqual Limited (Auckland, New Zealand)

Deployment Details

Testing Organization and Site Information	
Testing organization (Name, Organization type, Contact website)	U.S. Environmental Protection Agency - Office of Research and Development<br>Federal Government<br>Air Sensor Toolbox | U.S. EPA Website
Testing location (City, State, Latitude and Longitude)	MLK - Corner Of MLK Blvd And Justison St<br>Wilmington, DE<br>39.7395, -75.5575
AQS site ID	10 - 003 - 2004
Sampling timeframe (MM-DD-YY)	07-26-19 to 08-25-19
Sensor data source	Aeroqual Cloud
Reference data source	AQS API download

Sensor Information			
Manufacturer, model	Aeroqual AQY		
Device firmware version	1.14.2		
Sampling time interval	1-minute		
Sensor serial numbers	AQY_01		
Issues encountered during deployment?	☐	No Issues	

FRM/FEM Information	
Manufacturer, model, designation	Thermo Scientific Model 49i FEM
Sampling time interval	1-hour averaging
Date of calibration	As required by 40 CFR Part 58 and the DE Ambient Air Quality Monitoring Program QAPP maintained by DNREC
Date of one-point QC check	Every two weeks as required by 40 CFR Part 58 Appendix A 3.1.1
Description, date(s) of maintenance activities	N/A

Time Series Plot: 1-hour averaged O_3

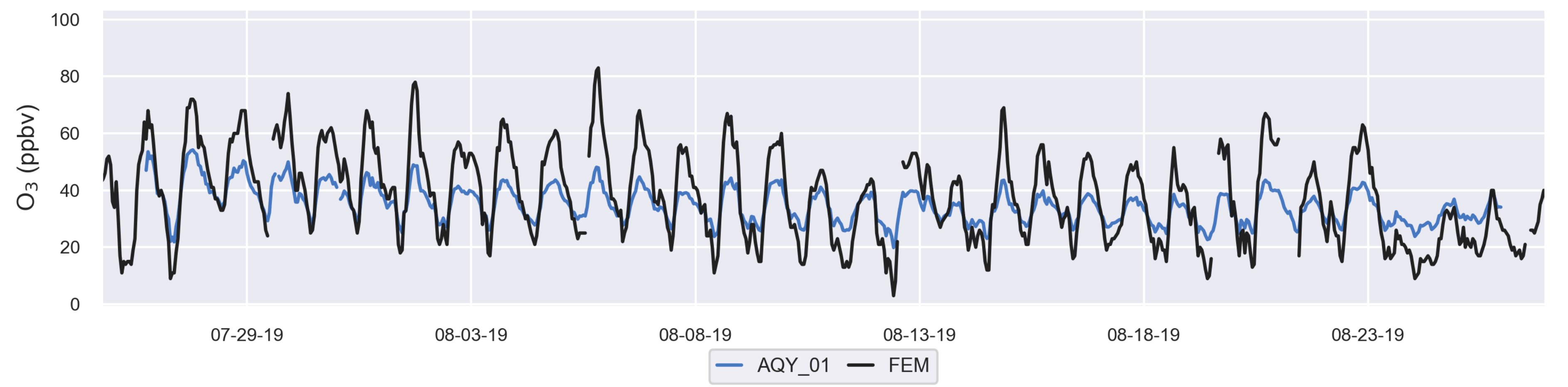

Range and average of FRM/FEM concentrations over duration of base test (ppbv)	[1-hr] 3.0-83.0, avg: 38.4,<br>[Rolling 8-hr] 12.9-70.5, avg: 38.4
Number of 1-hr periods in FRM/FEM monitor measurements with a goal concentration ≥ 60 ppbv	59

Scatter Plot: Comparison to FRM/FEM

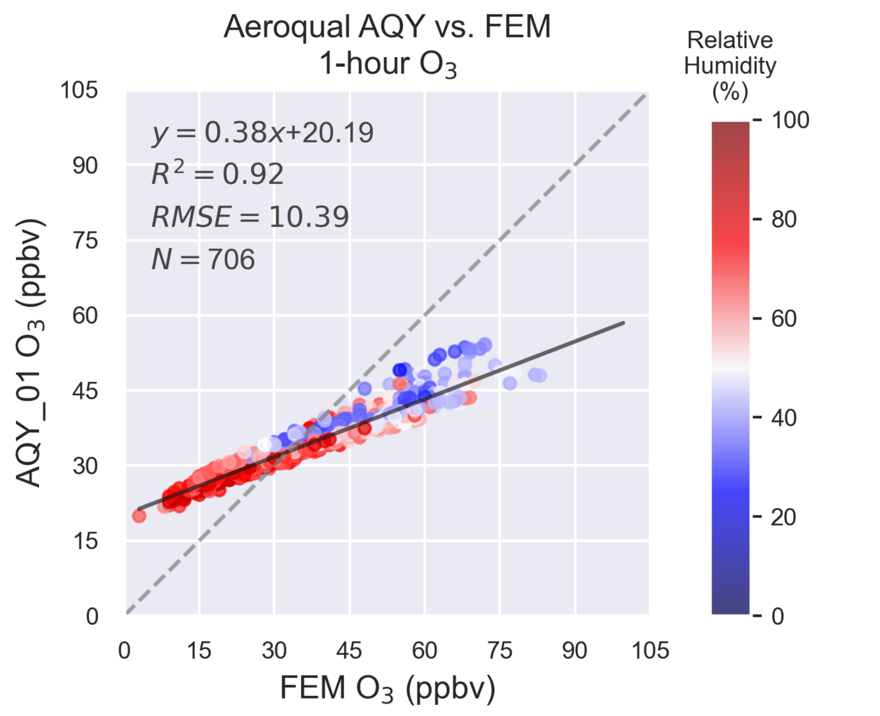

Performance Metrics

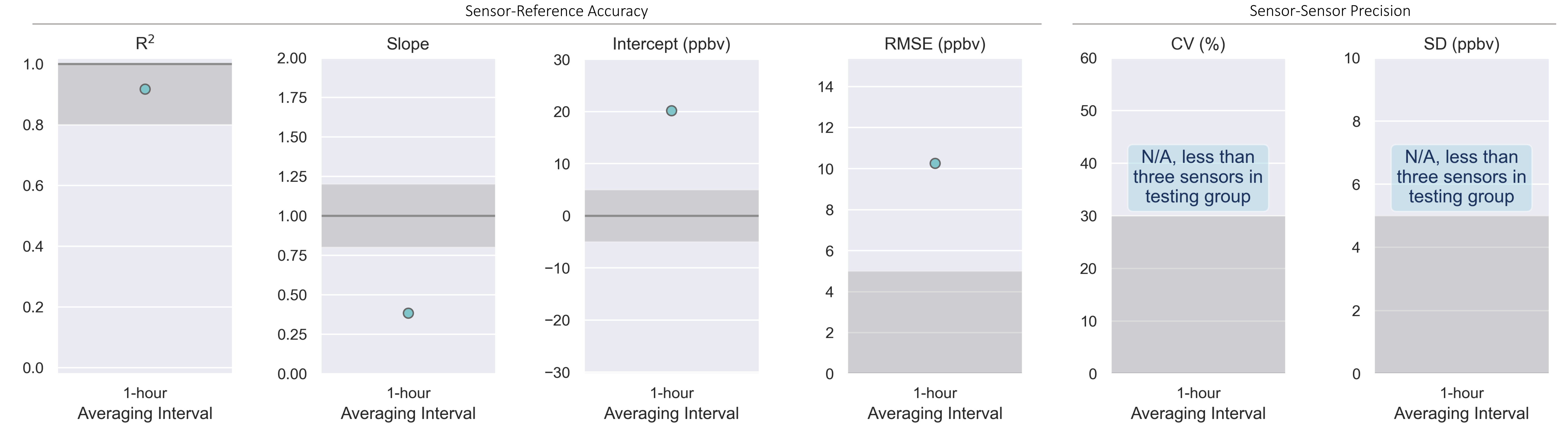

Meteorological Conditions During Deployment

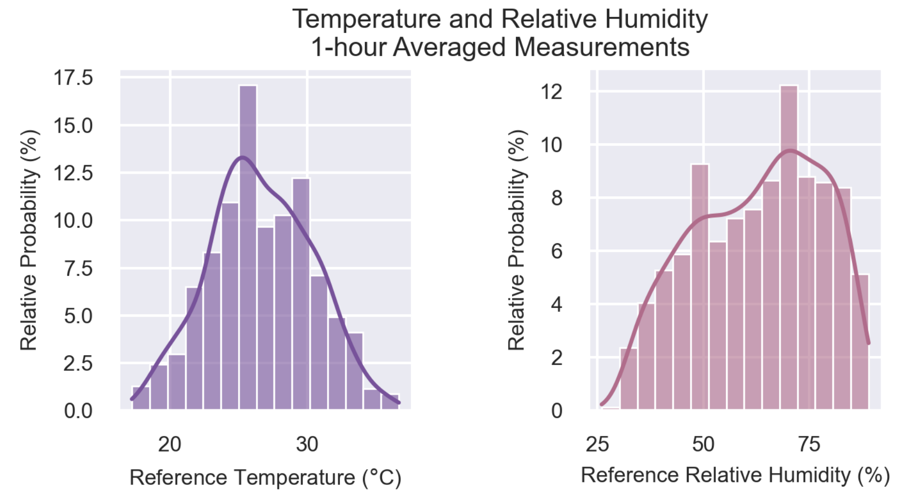

Number of 1-hr periods outside sensor manufacturer-listed temperature operational range (-10 to 40 °C)	0
Number of 1-hr periods outside sensor manufacturer-listed relative humidity operational range (no operational range specified)	-

Meteorological Influence

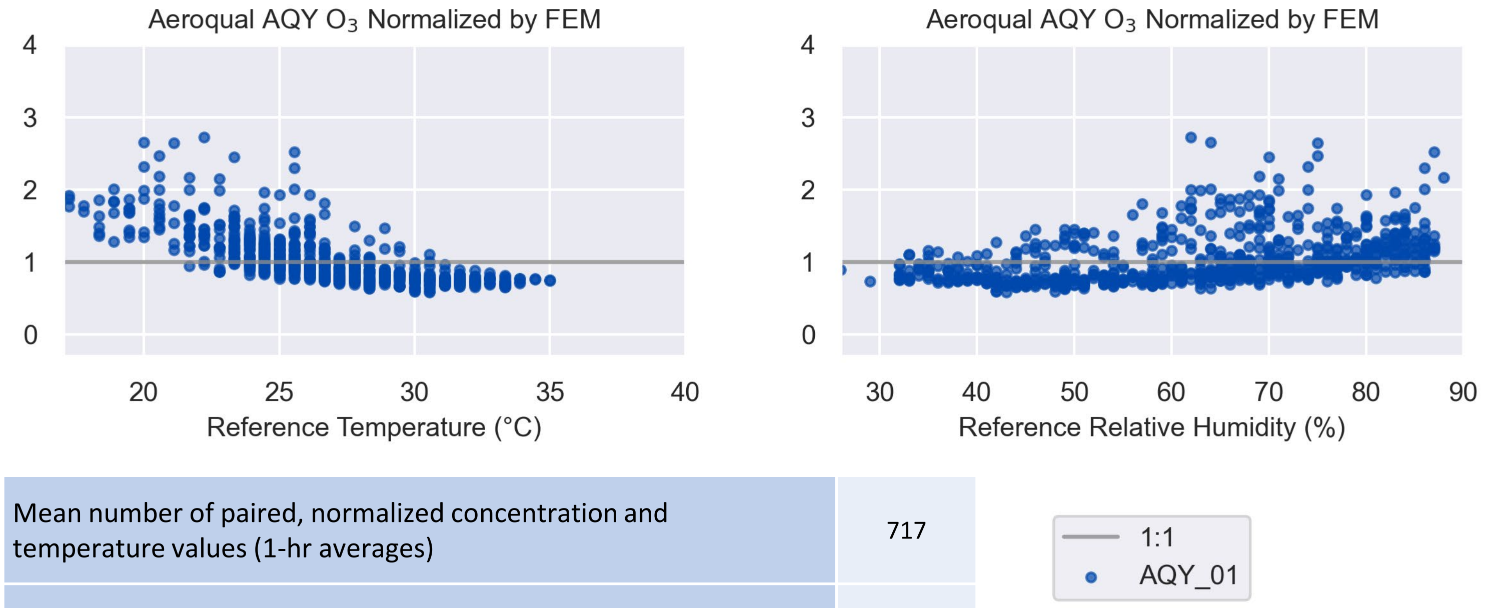

Mean number of paired, normalized concentration and temperature values (1-hr averages)	717
Mean number of paired, normalized concentration and relative humidity values (1-hr averages)	717

FEM data <5 ppbv (Federal MDL for the 49i) has been remove (Meteorological Influence section only)

Testing Report - O_3 Base Testing

Aeroqual AQY

This report reflects out-of-the-box performance

Initial Base Testing - Wilmington, DE

U.S. Environmental Protection Agency
Office of Research and Development
PI: Clements.Andrea@epa.gov
919-541-1363

July 2019—August 2019

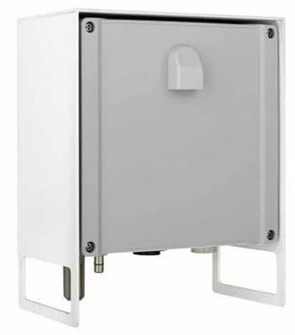

Image Attribution: Aeroqual Limited (Auckland, New Zealand)

Tabular Statistics

Sensor-FRM/FEM Correlation

	Bias and Linearity			Data Quality	
	R^2	Slope	Intercept (ppbv)	Uptime (%)	Number of paired sensor and reference concentration values*
	1-Hour ●	1-Hour ○	1-Hour ○	1-Hour ●	1-Hour
Metric Target Range	≥ 0.80	1.0 ± 0.20	-5 ≤ b ≤ 5	75%*	-
Sensor AQY_01	0.92	0.38	20.19	100	706

	Error
	RMSE (ppbv)
	1-Hour ☆
Metric Target Range	≤ 5.0
Deployment Value	10.3

Device-specific metrics (computed for each sensor in evaluation)
- ○ Metric value for none of devices tested falls within the target range
- ● Metric value for one of devices tested falls within the target range

Sensor-Sensor Precision[1]

	Precision (between collocated sensors)		Data Quality
	CV (%)	SD (ppbv)	Number of paired sensor and reference concentration values*
	1-Hour ☆	1-Hour ☆	1-Hour
Metric Target Range	≤ 30.0	≤ 5.0	-
Deployment Value	-	-	-

[1]Precision statistics are computed for evaluations with at least three collocated sensor units. Metric values are left blank for evaluations with two or fewer sensor units.

Single-valued metrics (computed via entire evaluation dataset)
- ☆ Indicates that the metric value is not within the target range
- ★ Indicates that the metric value is within the target range

*This value is only a recommendation for ensuring data quality and is not included in the list of target values discussed in Section 4 of the Performance Testing Protocols, Metrics, and Target Values for Ozone Air Sensors document.

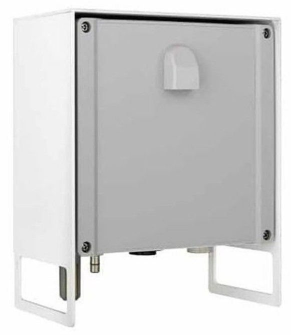

Image Attribution: Aeroqual Limited (Auckland, New Zealand)

Supplemental Information

Abbreviations used in Supplemental Information

FRM	Federal Reference Method
FEM	Federal Equivalent Method
SOP	Standard Operating Procedure
QAPP	Quality Assurance Project Plan
QC	Quality Control

Supplemental Documentation	Attached	Description & URL or file path to documentation
Field observations and sensor data flags	☑	See DE-AQY-Page 5 of this testing report
Maintenance logs	☐	No logs recorded during testing
Standard operating procedure(s)	☐	U.S. EPA Office Of Research and Development SOP available upon request
Photos of equipment setup and testing	☑	See DE-AQY-Page 4 of this testing report
Product specifications sheet(s)	☑	See Appendix C, "Spec_Sheet_Aeroqual_AQY.pdf"*
Product manual(s)	☑	See Appendix C, "Manual_Aeroqual_AQY.pdf"*
Data storage and transmission method	☑	See DE-AQY-Page 5 of this testing report
Data correction approach	☑	See DE-AQY-Page 5 of this testing report
Issues encountered	☑	See DE-AQY-Page 5 of this testing report
Data analysis/correction scripts and version	☑	Averaging and processing of data, calculation of performance metrics, and generation of figures and other supplementary material for analysis were obtained using Python 3.9.7 with the packages sensortoolkit v0.8.3b2, pandas 1.3.5, NumPy 1.21.2, Matplotlib 3.5.0, statsmodels 0.13.0, and seaborn 0.11.2. All packages are available from the Python Package Index (PyPI) at https://pypi.org. The integrated development environment (IDE) Spyder 5.1.5 was used for scripting and data visualization. Version control for the Python base, packages, and IDE were all managed by conda 4.11.0.
Air Monitoring Station QAPP	☐	U.S. EPA Office Of Research and Development QAPP available upon request
Summary of FRM/FEM monitor QC checks	☑	See DE-AQY-Page 6 of this testing report
Manufacturer website for FRM/FEM monitor	☑	ThermoFisher Scientific: Model 49i Product website
FRM/FEM monitor manual	☑	See Appendix B, "Spec_Sheet_Thermo_49i.pdf"
FRM/FEM monitor specifications sheet(s)	☑	See Appendix B, "Manual_Thermo_49i.pdf"
Other documents	☑	Manufacturer notice of AQY sales on hold

*As of 3/18/2021, the manufacturer of the AQY has placed sales of a similar unit on hold. Documentation for the AQY is currently unavailable from the manufacturer's website.

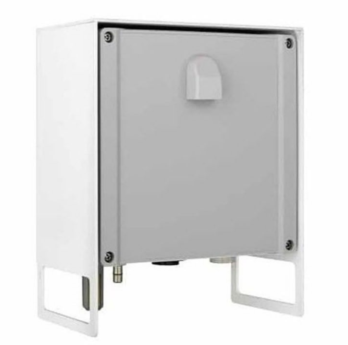

Image Attribution: Aeroqual Limited (Auckland, New Zealand)

Supplemental Information: Photos of Testing Site and Equipment Setup

Site Description:

The DE site is located in Wilmington, Delaware at the intersection of Justison St. and MLK Blvd and has been operated at this site since 1999. This site is the state NCore site and represents urban population exposure to multiple pollution sources. This site meets all EPA *40 CFR Part 58 App D and E* siting criteria.

Figure 1: Aeroqual AQY sensor (indicated by red arrow) attached to metal railing atop the sampling shelter at the monitoring site.

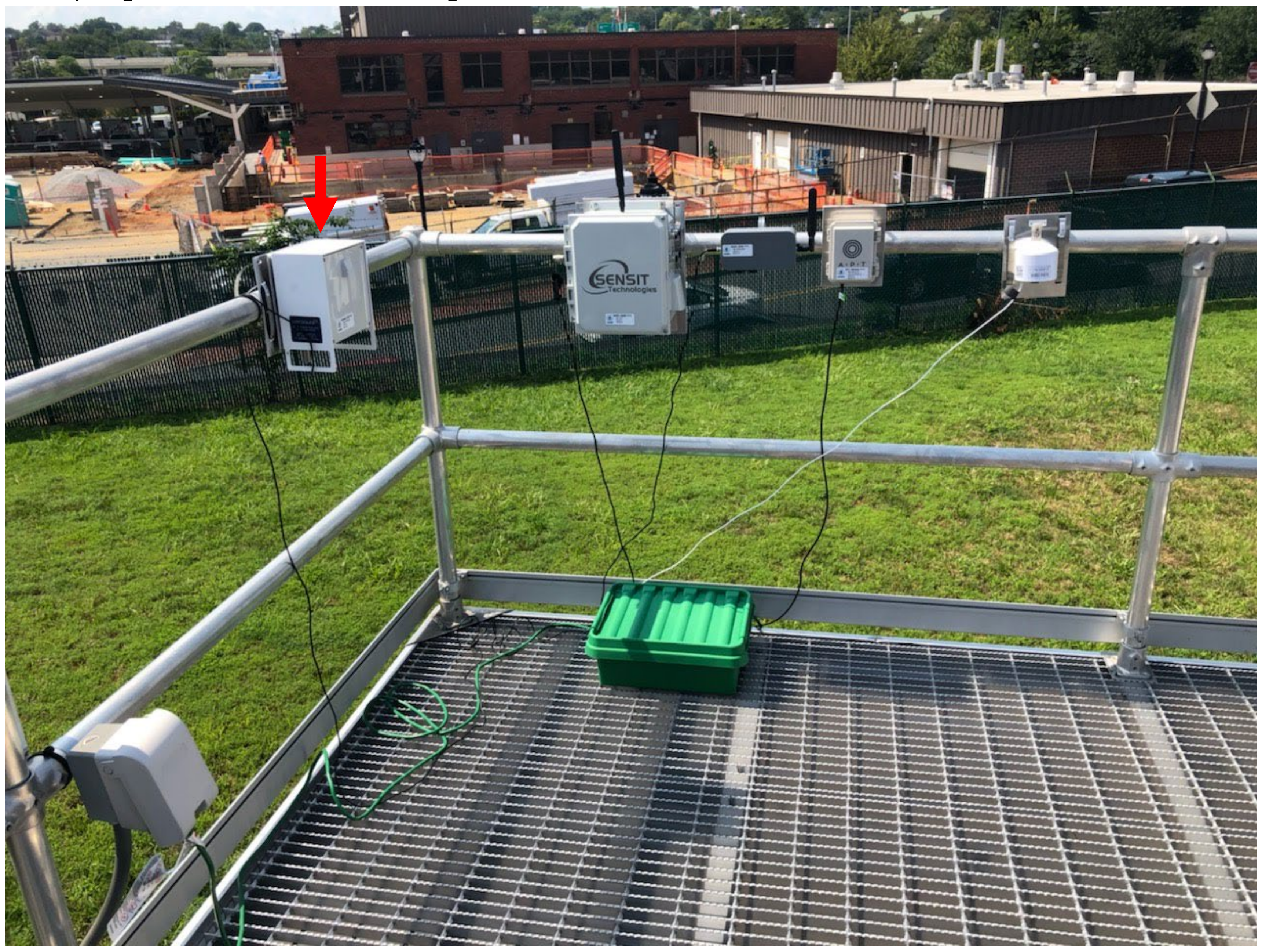

Figure 2: MLK Monitoring Station

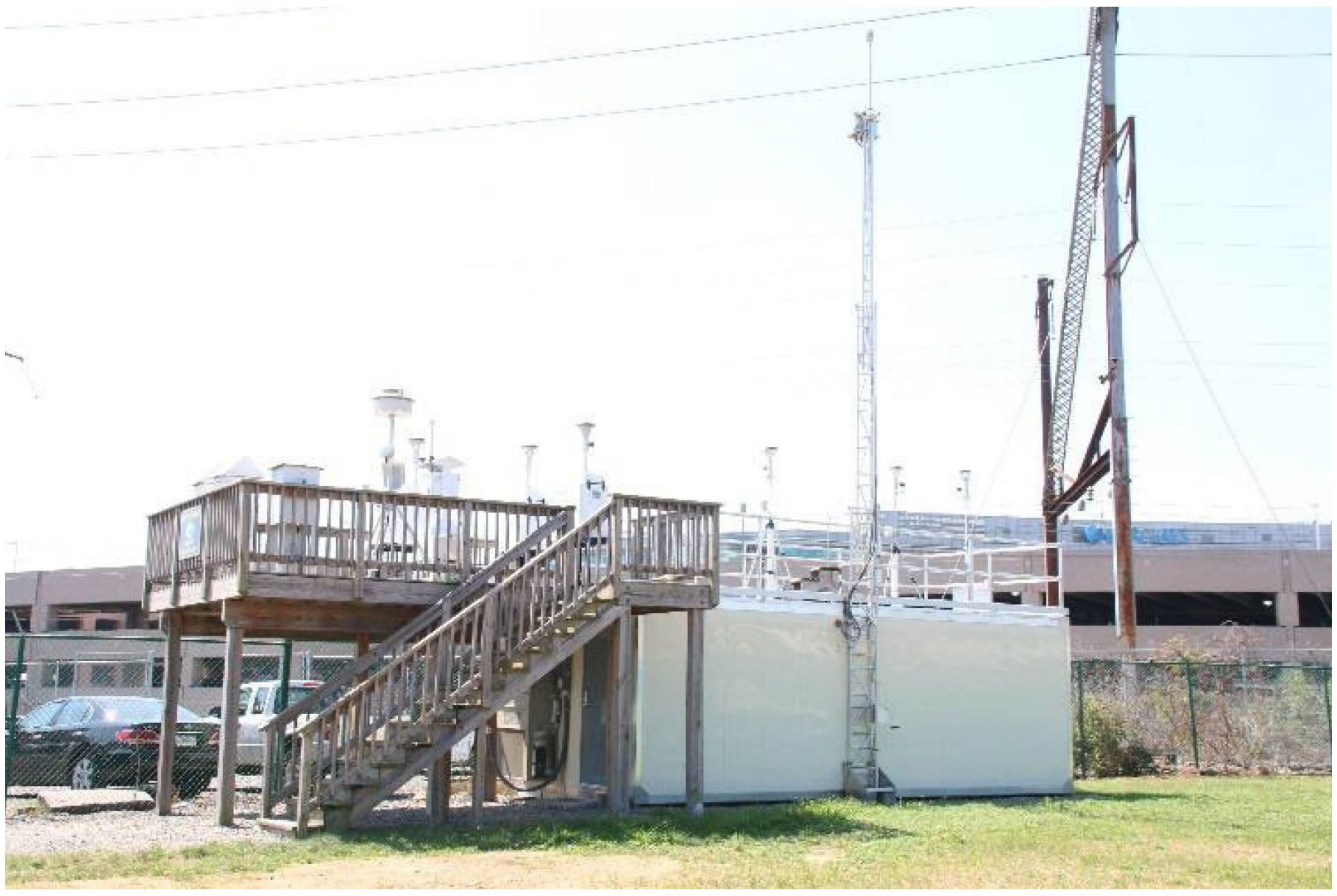

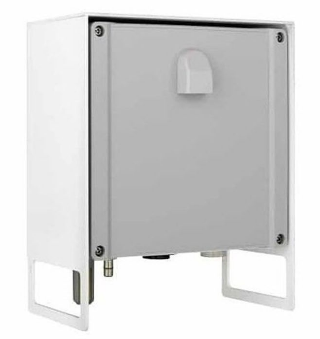

Image Attribution: Aeroqual Limited (Auckland, New Zealand)

Supplemental Information: Data Storage, Correction Approach, and Issues Encountered

Data Storage and Transmission Method

As part of CRADA #934-16 between Aeroqual and US EPA, Aeroqual supported data streaming. SIM cards were installed and data flowed to the Aeroqual Cloud. The 1-minute raw data was acquired weekly using the Aeroqual Cloud (*last accessed 5/11/22*) user interface (UI). The AQY has an internal data storage USB flash drive as a data backup, however access requires software proprietary to Aeroqual.

Data Correction Approach

This evaluation report reflects "out-of-the-box" performance of the AQY. The manufacturer provides a procedure by which local collocation (sensor operated along side an FRM/FEM) data can be collected, a gain (slope) and offset (intercept) determined, and parameters entered into the Aeroqual Cloud user interface to be applied to all subsequently collected data. This procedure and feature was **not** used prior to this evaluation. Prospective consumers may get different performance from this device if they utilize this feature.

After acquisition, the raw data was processed using the *sensortoolkit* python code library (v0.8.3b2). A continuous data set at the recorded sampling frequency was written to a .csv file. 1-hour averaged data sets were generated using a 75% completeness threshold and saved as separate .csv files. Outliers were **not** removed from data sets in order to assess "out-of-the-box" sensor performance.

The duration of the warm-up period required for sensor measurements to equilibrate was determined from field data to be 10 minutes. Warm up periods were considered to occur following any power outage to sensor units, either due to routine field visits or unscheduled site power outages. Data recorded during warm up periods has been removed from data sets.

Issues Encountered

Pre-deployment observations

- *Timestamp inaccuracies*: During pre-deployment, the AQY devices did not properly sync timestamps with the onboard Real-Time Clock. Connecting the units to the internet by cellular or Wi-Fi allowed the unit to sync with internet time and resulted in proper timestamps.

Field observations and sensor data flags

The following table contains data flags describing events that were encountered during the testing period. During the testing period, two power outages were reported on 7/29/2019 and 7/31/2019. Both outages were relatively brief, with each lasting ~15 minutes and 3 minutes for the 7/29/2019 and 7/31/2019 events, respectively. The field technician was not reported at the site during either outage, indicating that each outage was likely not attributed to operator error. A sampling interval abnormality was reported on 7/31/2019, during which the unit deviated from its configured 1-minute sampling interval, and instead logged an interval lasting 53 minutes. Following this event, the sensor logged samples at regular 1-minute intervals.

Start Time (UTC)	End Time (UTC)	Sensor Serial ID	Parameters Impacted	Flag
2019-07-29 15:41:00+00:00	2019-07-29 15:55:00+00:00	AQY_01	ALL	9.0-Data Loss - Power Connection Error
2019-07-31 00:08:00+00:00	2019-07-31 01:01:00+00:00	AQY_01	ALL	6.0-Sampling interval abnormality
2019-07-31 17:36:00+00:00	2019-07-31 17:39:00+00:00	AQY_01	ALL	9.0-Data Loss - Power Connection Error
2019-07-31 17:39:00+00:00	2019-07-31 17:39:00+00:00	AQY_01	O3	13.0-Data Incomplete - Gas Data

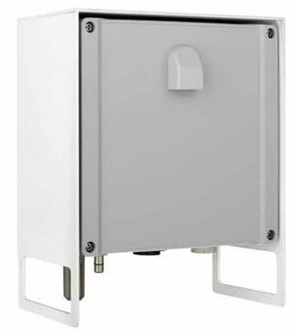

Image Attribution: Aeroqual Limited (Auckland, New Zealand)

Supplemental Information: Description of FRM/FEM QC Checks and Data Flags

Description of Data Flags

AQS

The U.S. EPA's Air Quality System (AQS) is the Agency's primary ambient air monitoring data archive. A comprehensive list of data flags that are recorded alongside AQS data sets, referred to by U.S. EPA as 'qualifiers', can be found at the following link: https://aqs.epa.gov/aqsweb/documents/codetables/qualifiers.html

Invalidation of reference data: AQS qualifiers are organized by qualifier type, which indicates whether data logged alongside qualifier flags should be invalidated (set null). Qualifiers with type "Null Data Qualifier" are invalidated, and includes data logged during periods that coincide with QC checks (e.g., "BF-Precision/Zero/Span", "BJ- Operator Error", "BL - QA Audit", "AZ - QC Audit") among other events such as power outages. Data logged alongside qualifiers with type "Quality Assurance Qualifiers" are not invalidated and are included in this analysis (e.g., concentrations less than the federal MDL for the reference monitor "MD – Value less than MDL", QA reviewed values "Validated Value").

Data Flags Recorded During Testing

FRM/FEM Monitor	Timestamp (UTC)	Flag
Thermo Scientific Model 49i (Acquired via AQS)	2019-07-29 12:00:00+0000 to 2019-07-29 13:00:00+0000	AI - Insufficient Data (cannot calculate)
	2019-08-05 14:00:00+0000	AI - Insufficient Data (cannot calculate)
	2019-08-12 13:00:00+0000 to 2019-08-12 14:00:00+0000	AI - Insufficient Data (cannot calculate)
	2019-08-19 13:00:00+0000 to 2019-08-19 15:00:00+0000	AI - Insufficient Data (cannot calculate)
	2019-08-19 14:00:00+0000	AQ - Collection Error
	2019-08-21 01:00:00+0000 to 2019-08-21 09:00:00+0000	AM - Miscellaneous Void
	2019-08-21 10:00:00+0000	AI - Insufficient Data (cannot calculate)

Meteorological Instrument	Timestamp (UTC)	Flag
Vaisala WXT-520 Temperature Monitor (Acquired via AQS)	2019-08-21 01:00:00+0000 to 2019-08-21 09:00:00+0000	AM - Miscellaneous Void.
	2019-08-21 10:00:00+0000	AI - Insufficient Data (cannot calculate).
Vaisala WXT-520 Relative Humidity Monitor (Acquired via AQS)	2019-08-21 01:00:00+0000 to 2019-08-21 09:00:00+0000	AM - Miscellaneous Void.
	2019-08-21 10:00:00+0000	AI - Insufficient Data (cannot calculate).

Testing Report - O_3 Base Testing

SENSIT RAMP

This report reflects out-of-the-box performance

Initial Base Testing - Wilmington, DE

U.S. Environmental Protection Agency
Office of Research and Development
PI: Clements.Andrea@epa.gov
919-541-1363
July 2019—August 2019

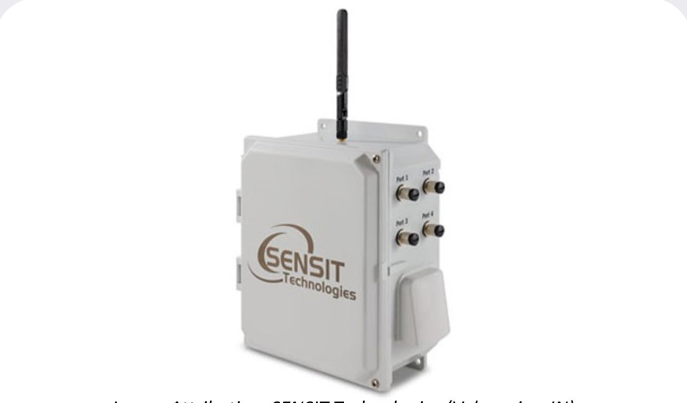

Image Attribution: SENSIT Technologies (Valparaiso, IN)

Deployment Details

Testing Organization and Site Information	
Testing organization (Name, Organization type, Contact website)	U.S. Environmental Protection Agency - Office of Research and Development<br>Federal Government<br>Air Sensor Toolbox | U.S. EPA Website
Testing location (City, State, Latitude and Longitude)	MLK - Corner Of MLK Blvd And Justison St<br>Wilmington, DE<br>39.7395, -75.5575
AQS site ID	10 - 003 - 2004
Sampling timeframe (MM-DD-YY)	07-30-19 to 08-29-19
Sensor data source	Onboard MicroSD card
Reference data source	AQS API download

Sensor Information			
Manufacturer, model	SENSIT RAMP		
Device firmware version	200203_AQ_v9.36_ALT		
Sampling time interval	15-seconds		
Sensor serial numbers	RAM_01		
Issues encountered during deployment?	☐	No Issues	

FRM/FEM Information	
Manufacturer, model, designation	Thermo Scientific Model 49i FEM
Sampling time interval	1-hour averaging
Date of calibration	As required by 40 CFR Part 58 and the DE Ambient Air Quality Monitoring Program QAPP maintained by DNREC
Date of one-point QC check	Every two weeks as required by 40 CFR Part 58 Appendix A 3.1.1
Description, date(s) of maintenance activities	N/A

Time Series Plot: 1-hour averaged O_3

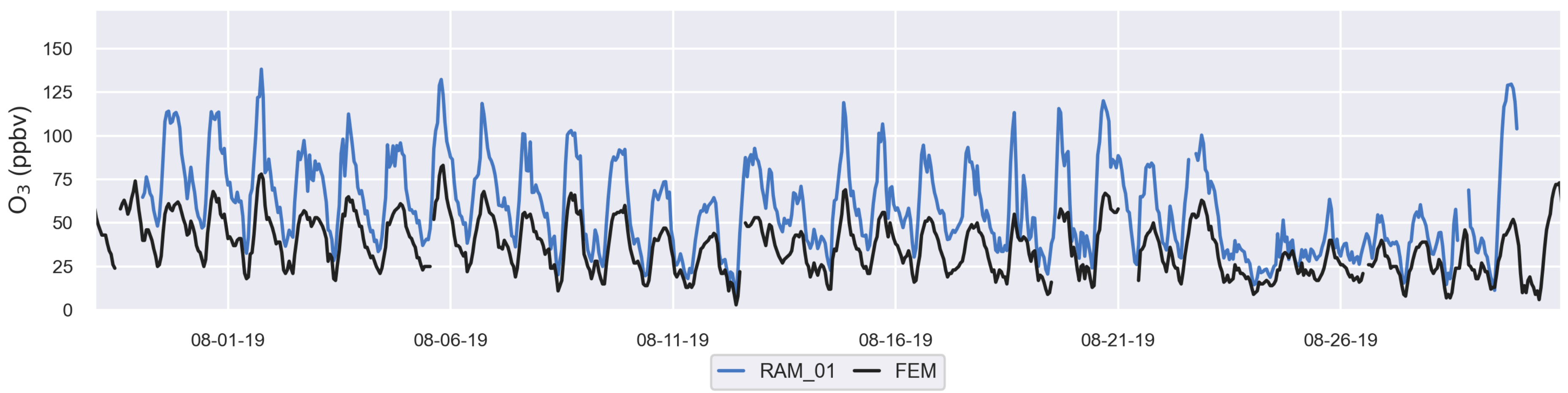

Range and average of FRM/FEM concentrations over duration of base test (ppbv)	[1-hr] 3.0-83.0, avg: 35.8,<br>[Rolling 8-hr] 12.9-70.5, avg: 35.9
Number of 1-hr periods in FRM/FEM monitor measurements with a goal concentration ≥ 60 ppbv	40

Scatter Plot: Comparison to FRM/FEM

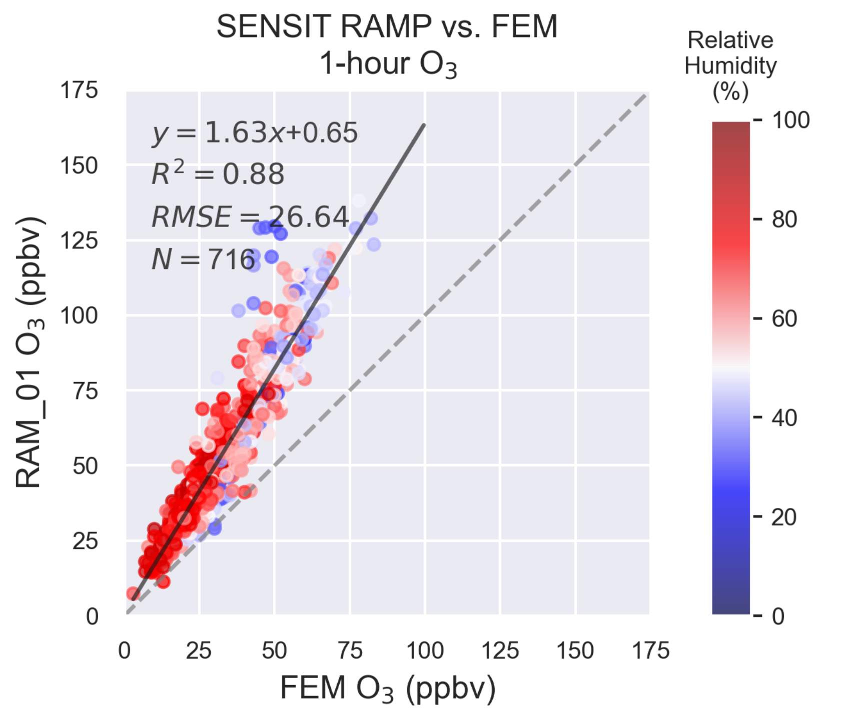

Performance Metrics

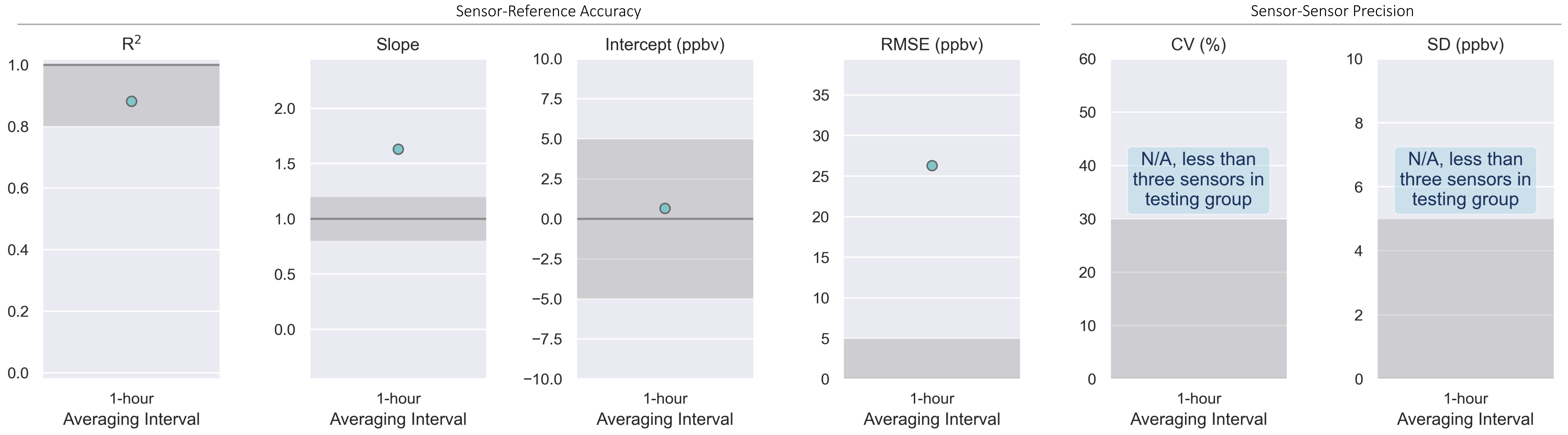

Meteorological Conditions During Deployment

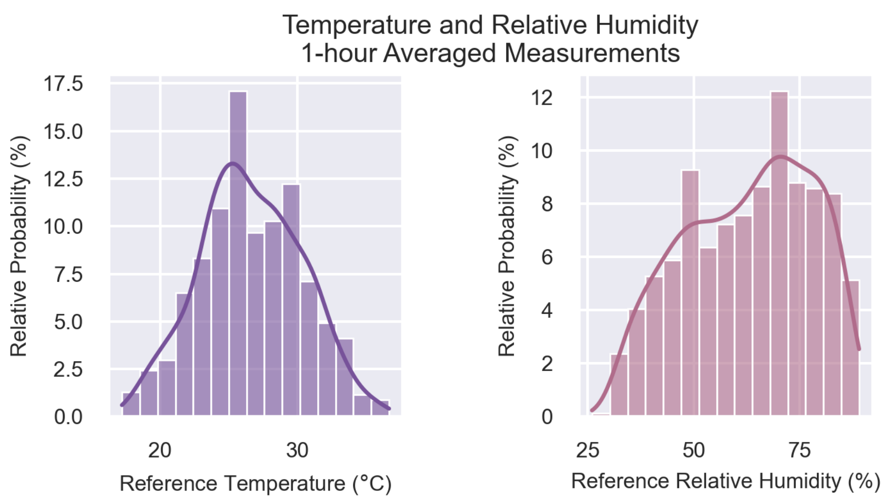

Number of 1-hr periods outside sensor manufacturer-listed temperature operational range (-20 to 50 °C)	0
Number of 1-hr periods outside sensor manufacturer-listed relative humidity operational range (no operational range specified)	-

Meteorological Influence

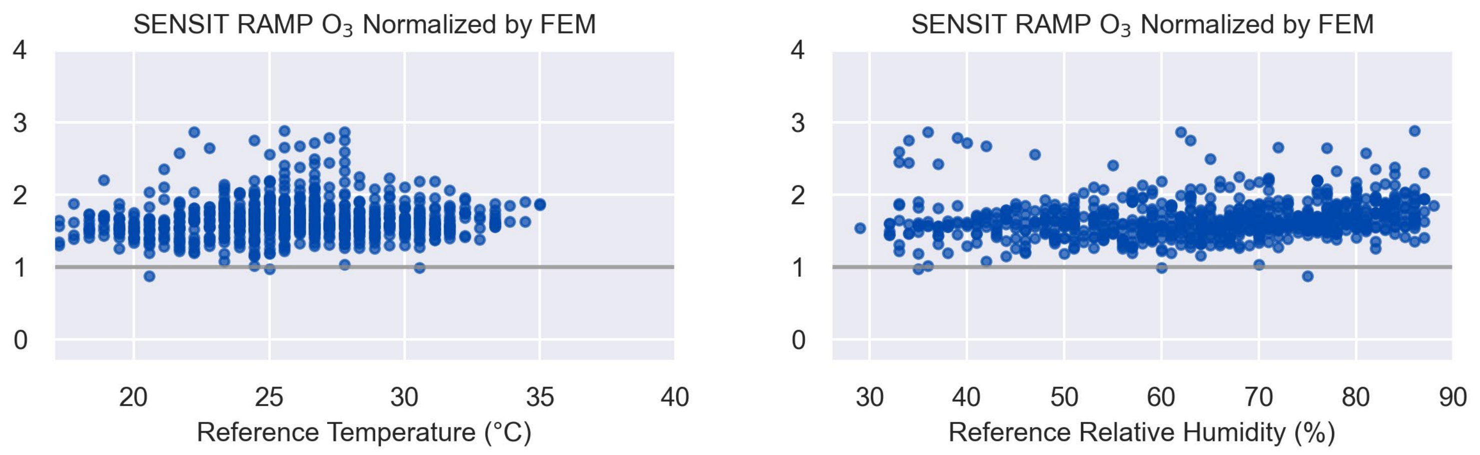

Mean number of paired, normalized concentration and temperature values (1-hr averages)	734
Mean number of paired, normalized concentration and relative humidity values (1-hr averages)	734

1:1
RAM_01

FEM data <5 ppbv (Federal MDL for the 49i) has been remove (Meteorological Influence section only)

Testing Report - O_3 Base Testing

SENSIT RAMP

This report reflects out-of-the-box performance

Initial Base Testing - Wilmington, DE
U.S. Environmental Protection Agency
Office of Research and Development
PI: Clements.Andrea@epa.gov
919-541-1363
July 2019—August 2019

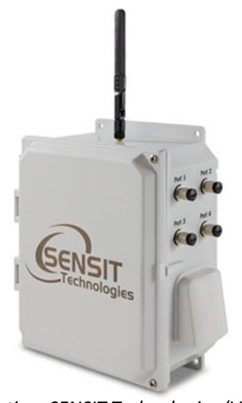

Image Attribution: SENSIT Technologies (Valparaiso, IN)

Tabular Statistics

Sensor-FRM/FEM Correlation

	Bias and Linearity			Data Quality	
	R^2	Slope	Intercept (ppbv)	Uptime (%)	Number of paired sensor and reference concentration values*
	1-Hour ●	1-Hour ○	1-Hour ●	1-Hour ●	1-Hour
Metric Target Range	≥ 0.80	1.0 ± 0.20	-5 ≤ b ≤ 5	75%*	-
Sensor RAM_01	0.88	1.63	0.65	100	716

	Error
	RMSE (ppbv)
	1-Hour ☆
Metric Target Range	≤ 5.0
Deployment Value	26.3

Device-specific metrics (computed for each sensor in evaluation)
- ○ Metric value for none of devices tested falls within the target range
- ● Metric value for one of devices tested falls within the target range

Sensor-Sensor Precision[1]

	Precision (between collocated sensors)		Data Quality
	CV (%)	SD (ppbv)	Number of paired sensor and reference concentration values*
	1-Hour ☆	1-Hour ☆	1-Hour
Metric Target Range	≤ 30.0	≤ 5.0	-
Deployment Value	-	-	-

[1]Precision statistics are computed for evaluations with at least three collocated sensor units. Metric values are left blank for evaluations with two or fewer sensor units.

Single-valued metrics (computed via entire evaluation dataset)
- ☆ Indicates that the metric value is not within the target range
- ★ Indicates that the metric value is within the target range

*This value is only a recommendation for ensuring data quality and is not included in the list of target values discussed in Section 4 of the Performance Testing Protocols, Metrics, and Target Values for Ozone Air Sensors document.

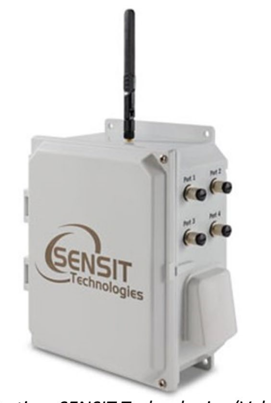

Image Attribution: SENSIT Technologies (Valparaiso, IN)

Supplemental Information

Abbreviations used in Supplemental Information

FRM	Federal Reference Method
FEM	Federal Equivalent Method
SOP	Standard Operating Procedure
QAPP	Quality Assurance Project Plan
QC	Quality Control

Supplemental Documentation	Attached	Description & URL or file path to documentation
Field observations and sensor data flags	☑	See DE-RAM-Page 5 of this testing report
Maintenance logs	☐	No logs recorded during testing
Standard operating procedure(s)	☐	U.S. EPA Office Of Research and Development SOP available upon request
Photos of equipment setup and testing	☑	See DE-RAM-Page 4 of this testing report
Product specifications sheet(s)	☑	See Appendix C, “Spec_Sheet_SENSIT_RAMP.pdf”
Product manual(s)	☑	See Appendix C, “Manual_SENSIT_RAMP.pdf”
Data storage and transmission method	☑	See DE-RAM-Page 5 of this testing report
Data correction approach	☑	See DE-RAM-Page 5 of this testing report
Issues encountered	☑	See DE-RAM-Page 5 of this testing report
Data analysis/correction scripts and version	☑	Averaging and processing of data, calculation of performance metrics, and generation of figures and other supplementary material for analysis were obtained using Python 3.9.7 with the packages sensortoolkit v0.8.3b2, pandas 1.3.5, NumPy 1.21.2, Matplotlib 3.5.0, statsmodels 0.13.0, and seaborn 0.11.2. All packages are available from the Python Package Index (PyPI) at https://pypi.org. The integrated development environment (IDE) Spyder 5.1.5 was used for scripting and data visualization. Version control for the Python base, packages, and IDE were all managed by conda 4.11.0.
Air Monitoring Station QAPP	☐	U.S. EPA Office Of Research and Development QAPP available upon request
Summary of FRM/FEM monitor QC checks	☑	See DE-RAM-Page 6 of this testing report
Manufacturer website for FRM/FEM monitor	☑	ThermoFisher Scientific: Model 49i Product website
FRM/FEM monitor manual	☑	See Appendix B, “Spec_Sheet_Thermo_49i.pdf”
FRM/FEM monitor specifications sheet(s)	☑	See Appendix B, “Manual_Thermo_49i.pdf”
Other documents	☐	

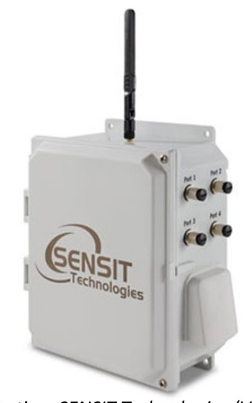

Image Attribution: SENSIT Technologies (Valparaiso, IN)

Supplemental Information: Photos of Testing Site and Equipment Setup

Site Description:
The DE site is located in Wilmington, Delaware at the intersection of Justison St. and MLK Blvd and has been operated at this site since 1999. This site is the state NCore site and represents urban population exposure to multiple pollution sources. This site meets all EPA *40 CFR Part 58 App D and E* siting criteria.

Figure 1: SENSIT RAMP sensor (indicated by red arrow) attached to metal railing atop the sampling shelter at the monitoring site.

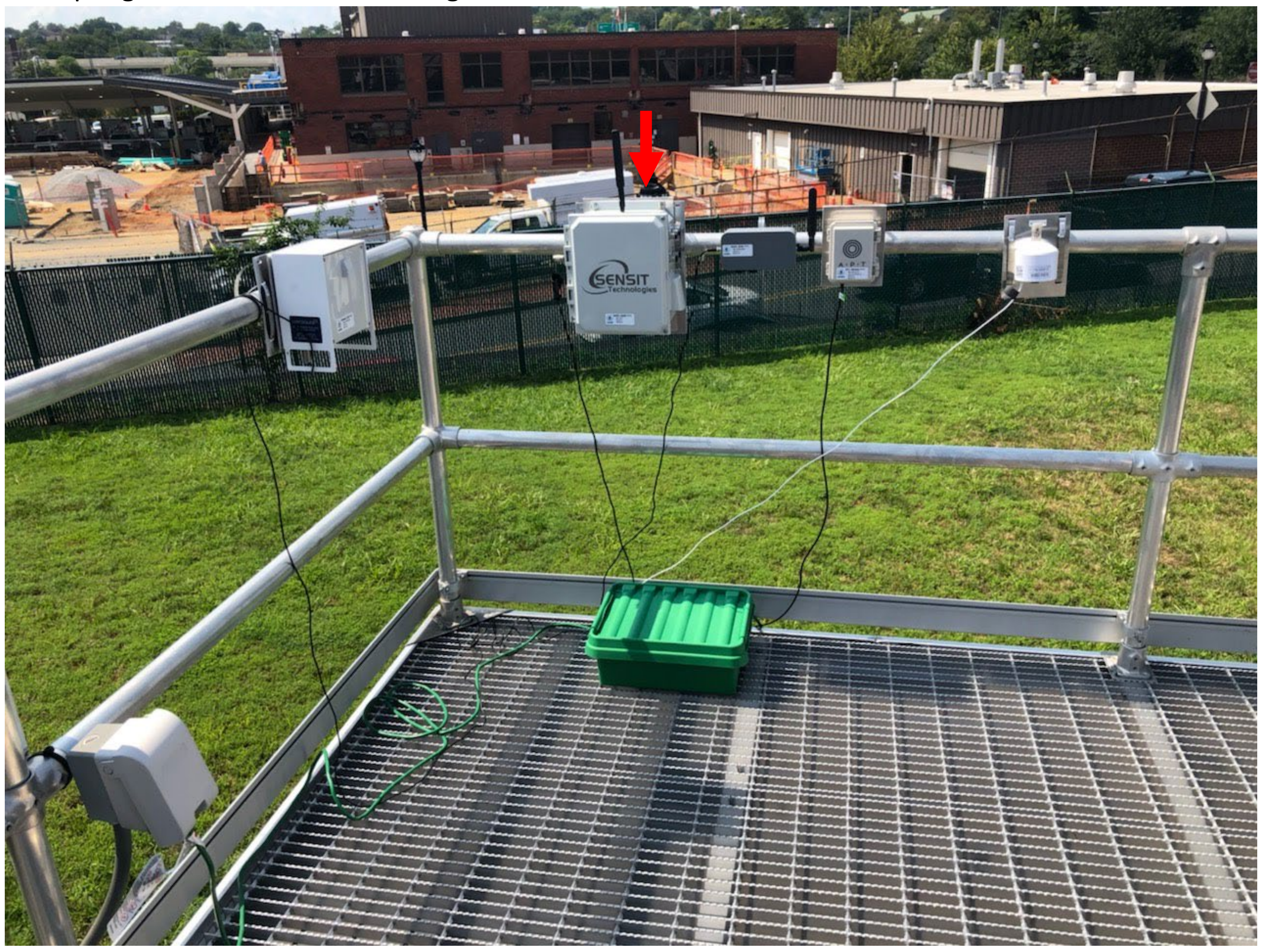

Figure 2: MLK Monitoring Station

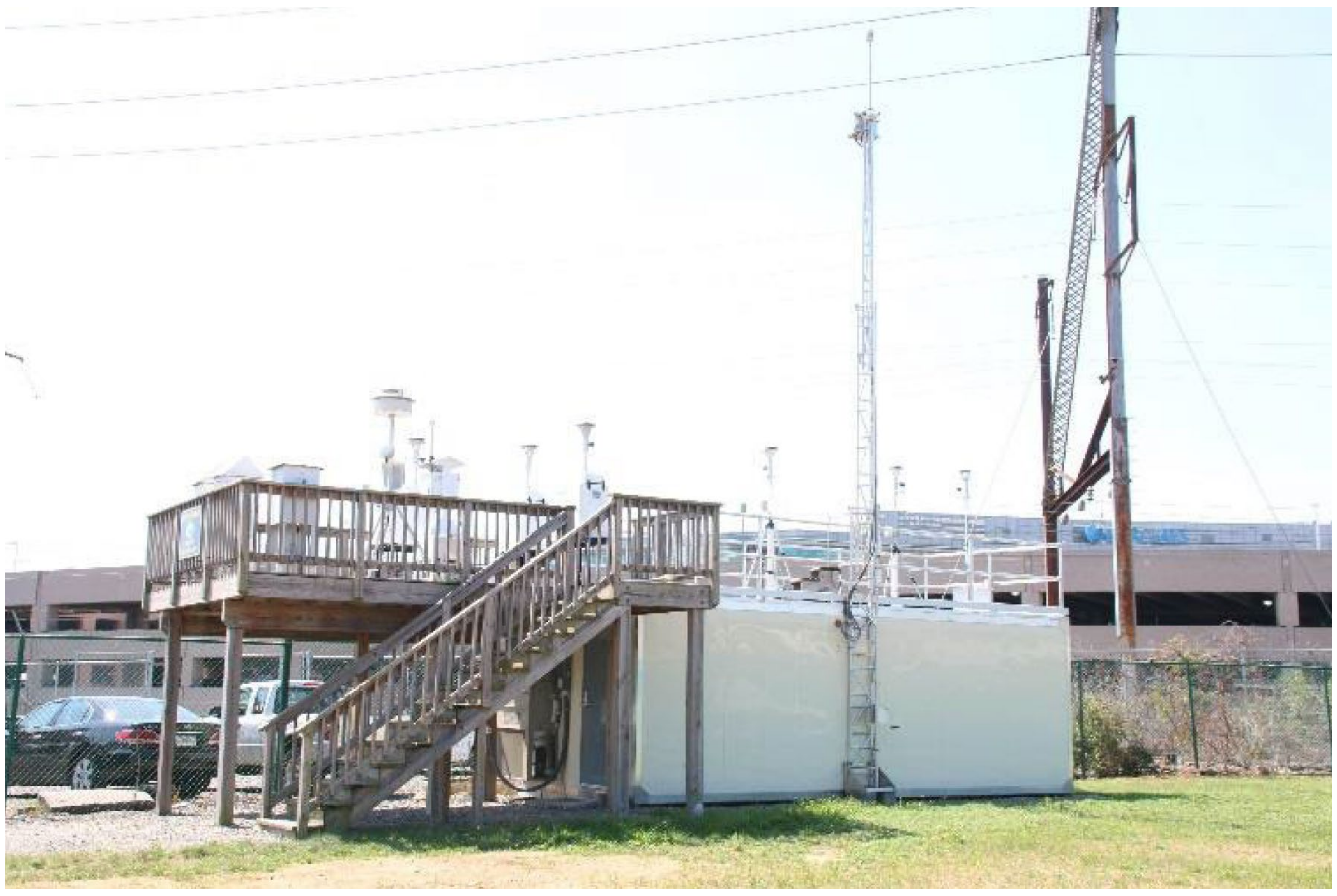

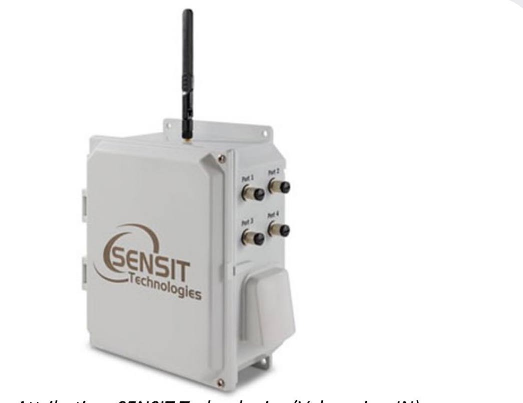

Image Attribution: SENSIT Technologies (Valparaiso, IN)

Data Storage and Transmission Method

The SENSIT RAMP was configured to record data at a 15-second sampling interval. Data are stored as daily text files (.txt format) on an onboard MicroSD card. Data files were obtained weekly via SD cards. Each field site operator was provided two labeled MicroSD cards for sensor units that they used to swap out each week. Data from the collected card was then read and processed off-site.

Data Correction Approach

SENSIT RAMP units were pre-configured by the manufacturer with a linear correction (i.e., concentration gain = 1.0 and offset = 2.0 ppbv). These presets reflect out-of-the box performance and were not modified by EPA prior or during testing.

After acquisition, the raw data was processed using the *sensortoolkit* python code library (v0.8.3b2). A continuous data set at the recorded sampling frequency was written to a .csv file. 1-hour averaged data sets were generated using a 75% completeness threshold and saved as separate .csv files. Outliers were **not** removed from data sets in order to assess "out-of-the-box" sensor performance.

The duration of the warm-up period required for sensor measurements to equilibrate was determined during bench-top testing (additional detail in pre-deployment observations) to be approximately 2 hours. Data recorded during warm up periods has been removed from data sets.

Issues Encountered

<u>Pre-deployment observations</u>

- *Changing logging interval*: SENSIT RAMP units were received without documentation or manuals. After communicating the need to change default settings (logging interval and time zone) with the manufacturer, a draft user's manual and a USB cable were supplied. With the use of this USB cable, instrument settings could be changed, and real-time data could be logged using a serial communication software (CoolTerm, v.1.5.0). Because the sensor did not record data at the top of every minute, the RAMP was configured to record data at 15-second intervals so that the data could be averaged more closely to complete minutes.
- *Gas Sensor Warmup*: Prior to deployment, RAMP units were collocated in a bench-top evaluation to verify operational status and determine the extent of data invalidity (i.e., determine equilibration period) after an initial start-up event. The recorded response for parameters measured by the RAMP suggests that the gas sensors (CO, NO, NO_2, O_3) required approximately a 2-hour equilibration period, while the remaining sensors (temperature, relative humidity, particulate matter) did not require any equilibration period.

<u>Field observations and sensor data flags</u>

- The RAM_01 sensor unit recorded zeros for the CO_2, temperature, and relative humidity measurements during the testing period. This failure mode was due to failure of the COZIR sensor. The failure persisted until 3/11/2020, when the sensor unit was removed and a replacement unit was installed.

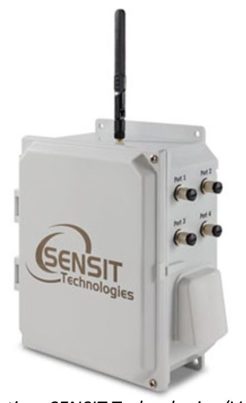

Image Attribution: SENSIT Technologies (Valparaiso, IN)

Description of Data Flags

AQS

The U.S. EPA’s Air Quality System (AQS) is the Agency’s primary ambient air monitoring data archive. A comprehensive list of data flags that are recorded alongside AQS data sets, referred to by U.S. EPA as ‘qualifiers’, can be found at the following link: https://aqs.epa.gov/aqsweb/documents/codetables/qualifiers.html

Invalidation of reference data: AQS qualifiers are organized by qualifier type, which indicates whether data logged alongside qualifier flags should be invalidated (set null). Qualifiers with type “Null Data Qualifier” are invalidated, and includes data logged during periods that coincide with QC checks (e.g., "BF-Precision/Zero/Span", "BJ- Operator Error", "BL - QA Audit“, “AZ - QC Audit”) among other events such as power outages. Data logged alongside qualifiers with type “Quality Assurance Qualifiers” are not invalidated and are included in this analysis (e.g., concentrations less than the federal MDL for the reference monitor “MD – Value less than MDL”, QA reviewed values "Validated Value“).

Data Flags Recorded During Testing

FRM/FEM Monitor	Timestamp (UTC)	Flag
Thermo Scientific Model 49i (Acquired via AQS)	2019-08-05 14:00:00+0000	AI - Insufficient Data (cannot calculate)
	2019-08-12 13:00:00+0000 to 2019-08-12 14:00:00+0000	AI - Insufficient Data (cannot calculate)
	2019-08-19 13:00:00+0000 to 2019-08-19 15:00:00+0000	AI - Insufficient Data (cannot calculate)
	2019-08-19 14:00:00+0000	AQ - Collection Error
	2019-08-21 01:00:00+0000 to 2019-08-21 09:00:00+0000	AM - Miscellaneous Void
	2019-08-21 10:00:00+0000	AI - Insufficient Data (cannot calculate)
	2019-08-26 13:00:00+0000 to 2019-08-26 14:00:00+0000	AI - Insufficient Data (cannot calculate)

Meteorological Instrument	Timestamp (UTC)	Flag
Vaisala WXT-520 Temperature Monitor (Acquired via AQS)	2019-08-21 01:00:00+0000 to 2019-08-21 09:00:00+0000	AM - Miscellaneous Void
	2019-08-21 10:00:00+0000	AI - Insufficient Data (cannot calculate)
Vaisala WXT-520 Relative Humidity Monitor (Acquired via AQS)	2019-08-21 01:00:00+0000 to 2019-08-21 09:00:00+0000	AM - Miscellaneous Void
	2019-08-21 10:00:00+0000	AI - Insufficient Data (cannot calculate)

Testing Report - O_3 Base Testing

Aeroqual AQY

This report reflects out-of-the-box performance

Initial Base Testing - Decatur, GA

U.S. Environmental Protection Agency
Office of Research and Development
PI: Clements.Andrea@epa.gov
919-541-1363
September 2019—October 2019

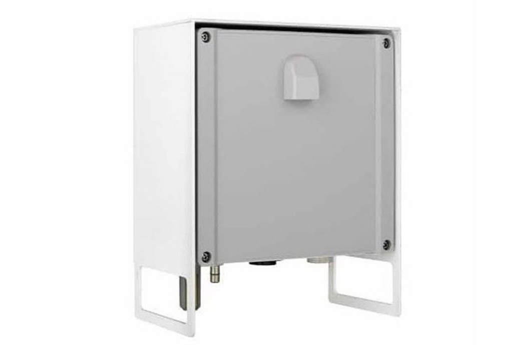
Image Attribution: Aeroqual Limited (Auckland, New Zealand)

Deployment Details

Testing Organization and Site Information	
Testing organization (Name, Organization type, Contact website)	U.S. Environmental Protection Agency - Office of Research and Development<br>Federal Government<br>Air Sensor Toolbox | U.S. EPA Website
Testing location (City, State, Latitude and Longitude)	South Dekalb<br>Decatur, GA<br>33.6877, -84.2905
AQS site ID	13 - 089 - 0002
Sampling timeframe (MM-DD-YY)	09-11-19 to 10-11-19
Sensor data source	Aeroqual Cloud
Reference data source	AQS API download

Sensor Information			
Manufacturer, model	Aeroqual AQY		
Device firmware version	1.14.2		
Sampling time interval	1-minute		
Sensor serial numbers	AQY_01		
Issues encountered during deployment?	☐	No Issues	

FRM/FEM Information	
Manufacturer, model, designation	Thermo Scientific Model 49i FEM
Sampling time interval	1-hour averaging
Date of calibration	As required by 40 CFR Part 58 and the Ambient Air Monitoring Plan maintained by GA DNR
Date of one-point QC check	Every two weeks as required by 40 CFR Part 58 Appendix A 3.1.1
Description, date(s) of maintenance activities	See GA-AQY-Page 6

Time Series Plot: 1-hour averaged O_3

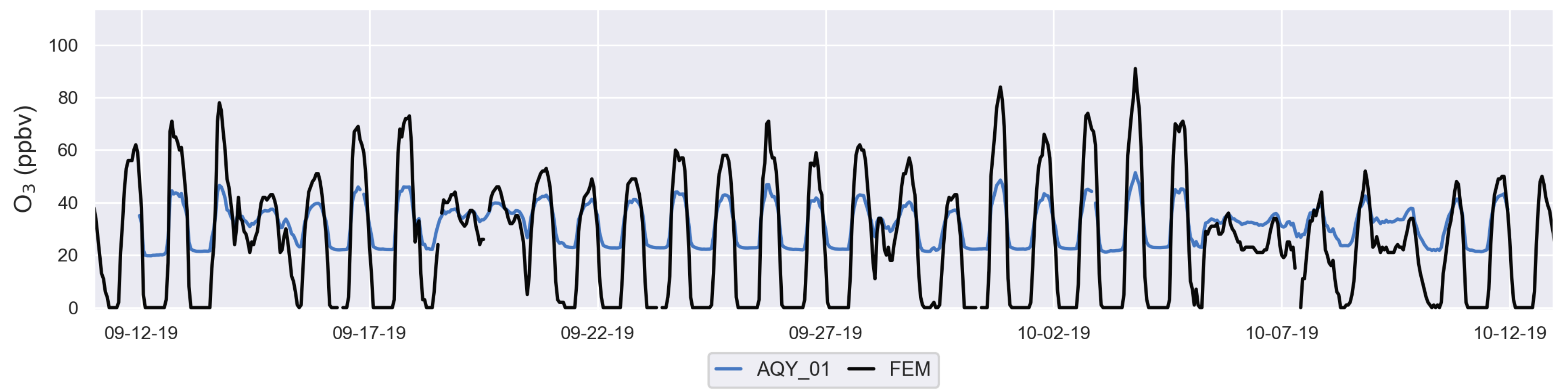

Range and average of FRM/FEM concentrations over duration of base test (ppbv)	[1-hr] 0.0-91.0, avg: 25.3,<br>[Rolling 8-hr] 0.0-73.5, avg: 26.0
Number of 1-hr periods in FRM/FEM monitor measurements with a goal concentration ≥ 60 ppbv	55

Scatter Plot: Comparison to FRM/FEM

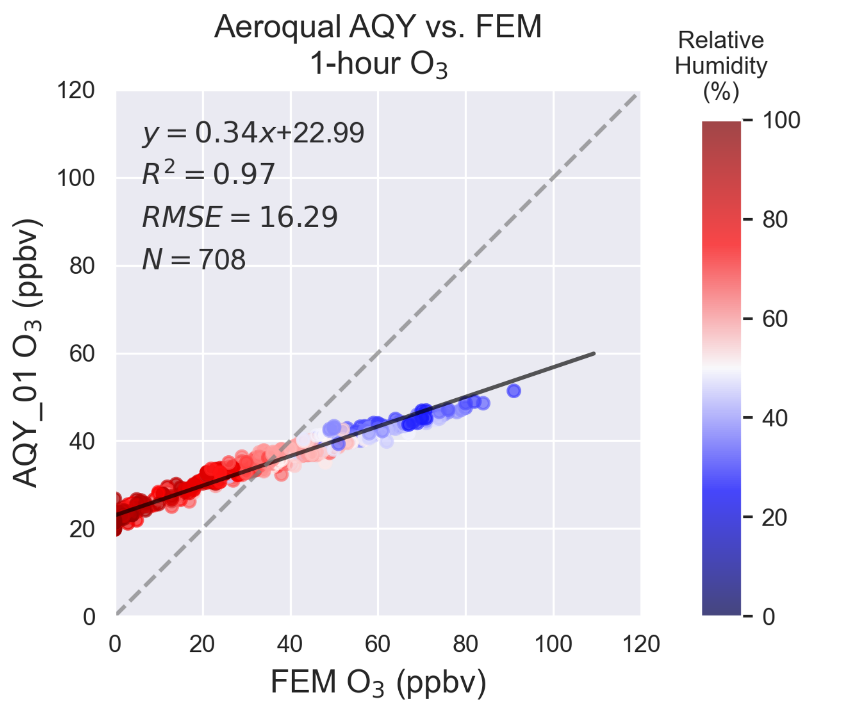

Performance Metrics

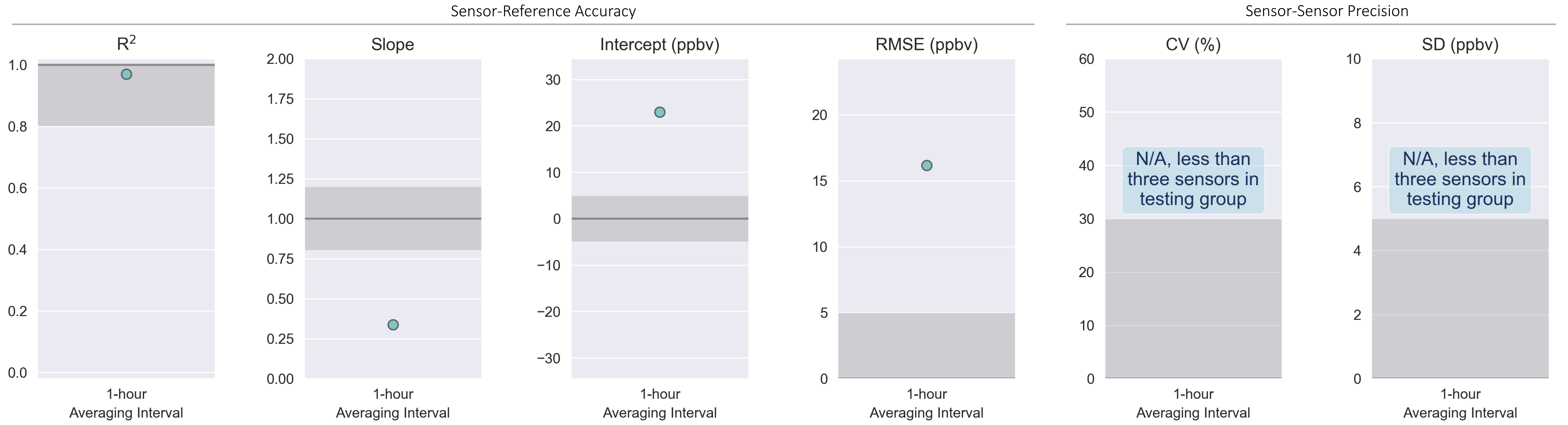

Meteorological Conditions During Deployment

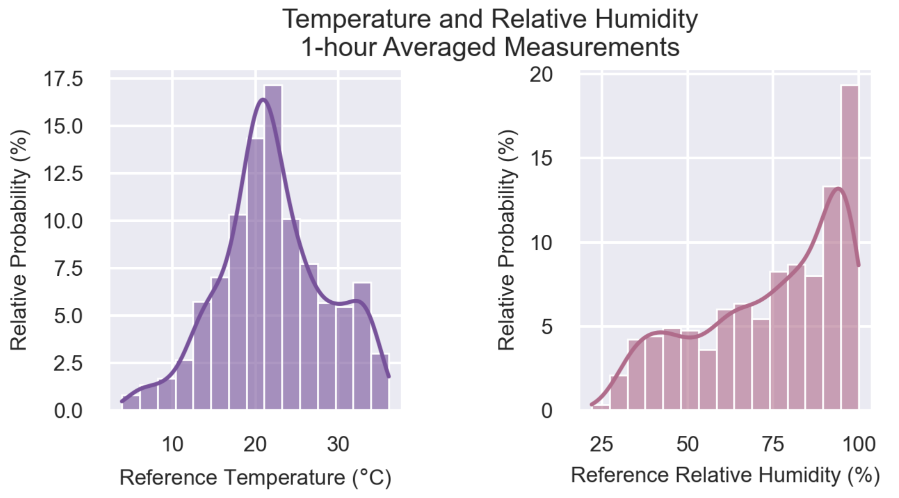

Number of 1-hr periods outside sensor manufacturer-listed temperature operational range (-10 to 40 °C)	0
Number of 1-hr periods outside sensor manufacturer-listed relative humidity operational range (no operational range specified)	-

Meteorological Influence

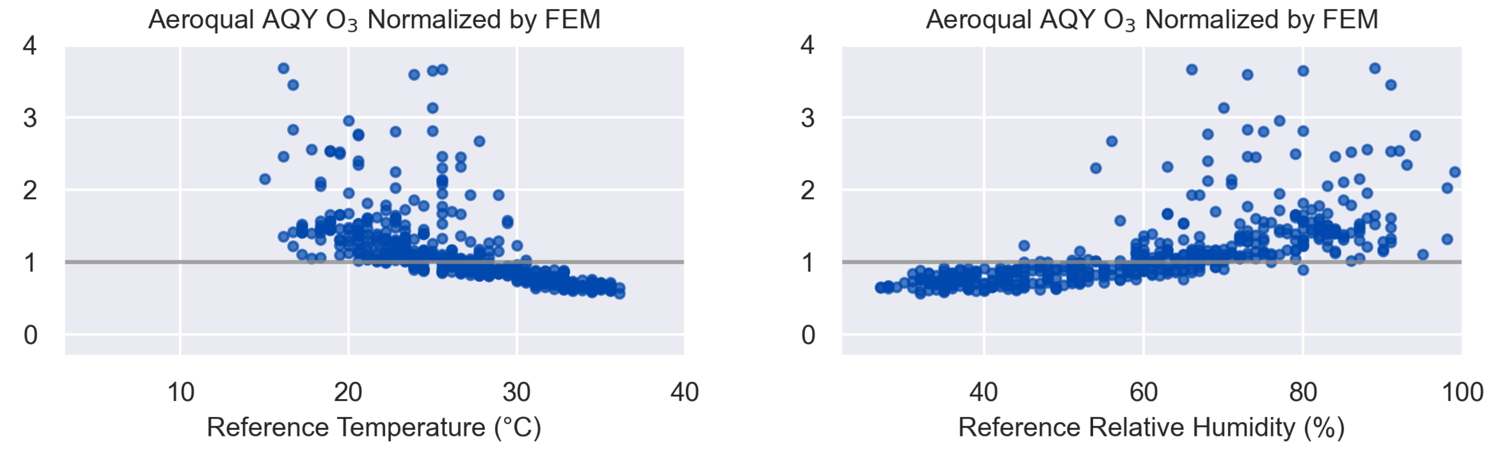

Mean number of paired, normalized concentration and temperature values (1-hr averages)	742
Mean number of paired, normalized concentration and relative humidity values (1-hr averages)	742

FEM data <5 ppbv (Federal MDL for the 49i) has been remove (Meteorological Influence section only)

Testing Report - O_3 Base Testing

Aeroqual AQY

This report reflects out-of-the-box performance

Initial Base Testing - Decatur, GA
U.S. Environmental Protection Agency
Office of Research and Development
PI: Clements.Andrea@epa.gov
919-541-1363
September 2019—October 2019

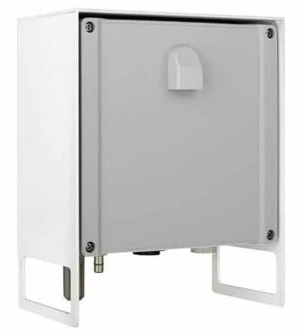
Image Attribution: Aeroqual Limited (Auckland, New Zealand)

Tabular Statistics

Sensor-FRM/FEM Correlation

	Bias and Linearity			Data Quality	
	R^2	Slope	Intercept (ppbv)	Uptime (%)	Number of paired sensor and reference concentration values*
	1-Hour ●	1-Hour ○	1-Hour ○	1-Hour ●	1-Hour
Metric Target Range	≥ 0.80	1.0 ± 0.20	-5 ≤ b ≤ 5	75%*	-
Sensor AQY_01	0.97	0.34	22.99	100	708

	Error
	RMSE (ppbv)
	1-Hour ☆
Metric Target Range	≤ 5.0
Deployment Value	16.2

Device-specific metrics (computed for each sensor in evaluation)
○ Metric value for none of devices tested falls within the target range
● Metric value for one of devices tested falls within the target range

Sensor-Sensor Precision[1]

	Precision (between collocated sensors)		Data Quality
	CV (%)	SD (ppbv)	Number of paired sensor and reference concentration values*
	1-Hour ☆	1-Hour ☆	1-Hour
Metric Target Range	≤ 30.0	≤ 5.0	-
Deployment Value	-	-	-

[1]Precision statistics are computed for evaluations with at least three collocated sensor units. Metric values are left blank for evaluations with two or fewer sensor units.

Single-valued metrics (computed via entire evaluation dataset)
☆ Indicates that the metric value is not within the target range
★ Indicates that the metric value is within the target range

*This value is only a recommendation for ensuring data quality and is not included in the list of target values discussed in Section 4 of the Performance Testing Protocols, Metrics, and Target Values for Ozone Air Sensors document.

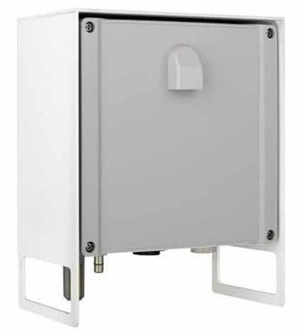

Image Attribution: Aeroqual Limited (Auckland, New Zealand)

Supplemental Information

Abbreviations used in Supplemental Information

FRM	Federal Reference Method
FEM	Federal Equivalent Method
SOP	Standard Operating Procedure
QAPP	Quality Assurance Project Plan
QC	Quality Control

Supplemental Documentation	Attached	Description & URL or file path to documentation
Field observations and sensor data flags	☑	See GA-AQY-Page 5 of this testing report
Maintenance logs	☐	No logs recorded during testing
Standard operating procedure(s)	☐	U.S. EPA Office Of Research and Development SOP available upon request
Photos of equipment setup and testing	☑	See GA-AQY-Page 4 of this testing report
Product specifications sheet(s)	☑	See Appendix C, "Spec_Sheet_Aeroqual_AQY.pdf"*
Product manual(s)	☑	See Appendix C, "Manual_Aeroqual_AQY.pdf"*
Data storage and transmission method	☑	See GA-AQY-Page 5 of this testing report
Data correction approach	☑	See GA-AQY-Page 5 of this testing report
Issues encountered	☑	See GA-AQY-Page 5 of this testing report
Data analysis/correction scripts and version	☑	Averaging and processing of data, calculation of performance metrics, and generation of figures and other supplementary material for analysis were obtained using Python 3.9.7 with the packages sensortoolkit v0.8.3b2, pandas 1.3.5, NumPy 1.21.2, Matplotlib 3.5.0, statsmodels 0.13.0, and seaborn 0.11.2. All packages are available from the Python Package Index (PyPI) at https://pypi.org. The integrated development environment (IDE) Spyder 5.1.5 was used for scripting and data visualization. Version control for the Python base, packages, and IDE were all managed by conda 4.11.0.
Air Monitoring Station QAPP	☐	U.S. EPA Office Of Research and Development QAPP available upon request
Summary of FRM/FEM monitor QC checks	☑	See GA-AQY-Page 6 of this testing report
Manufacturer website for FRM/FEM monitor	☑	ThermoFisher Scientific: Model 49i Product website
FRM/FEM monitor manual	☑	See Appendix B, "Spec_Sheet_Thermo_49i.pdf"
FRM/FEM monitor specifications sheet(s)	☑	See Appendix B, "Manual_Thermo_49i.pdf"
Other documents	☑	Manufacturer notice of AQY sales on hold

*As of 3/18/2021, the manufacturer of the AQY has placed sales of a similar unit on hold. Documentation for the AQY is currently unavailable from the manufacturer's website.

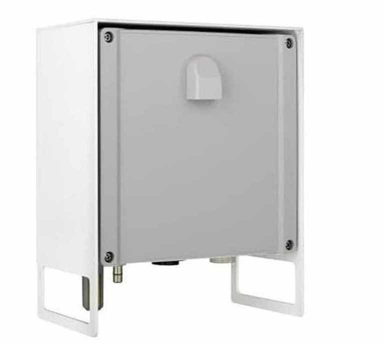
Image Attribution: Aeroqual Limited (Auckland, New Zealand)

Supplemental Information: Photos of Testing Site and Equipment Setup

Site Description:
The South Dekalb monitoring station was established as O3 site located in Decatur, Georgia.

Figure 1: Aeroqual AQY sensor (indicated by red arrow) attached to metal railing atop the sampling shelter at the monitoring site.

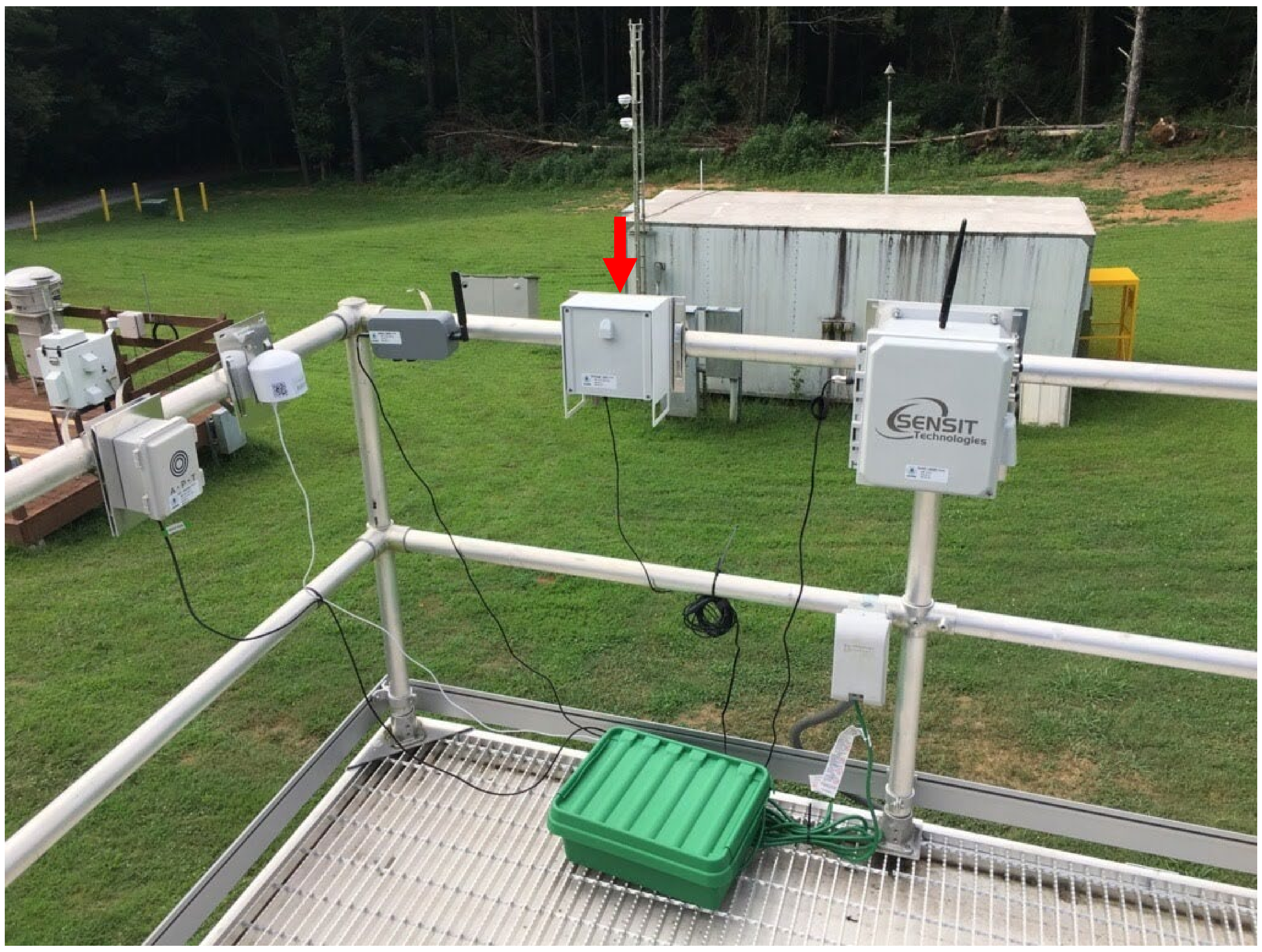

Figure 2: South Dekalb Monitoring Station

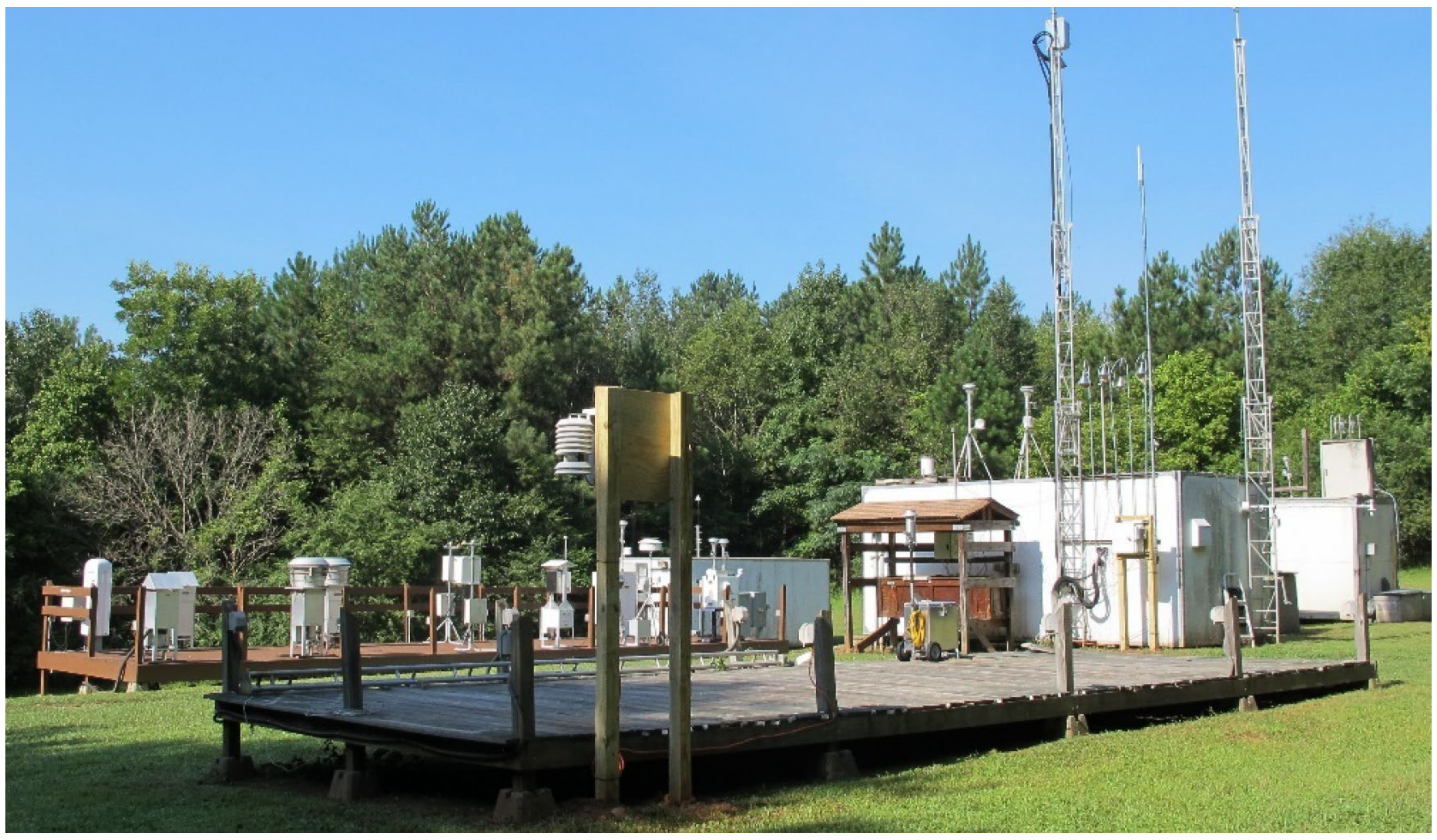

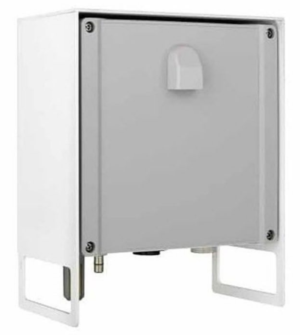

Image Attribution: Aeroqual Limited (Auckland, New Zealand)

Supplemental Information: Data Storage, Correction Approach, and Issues Encountered

Data Storage and Transmission Method

As part of CRADA #934-16 between Aeroqual and US EPA, Aeroqual supported data streaming. SIM cards were installed and data flowed to the Aeroqual Cloud. The 1-minute raw data was acquired weekly using the Aeroqual Cloud (*last accessed 5/11/22*) user interface (UI). The AQY has an internal data storage USB flash drive as a data backup, however access requires software proprietary to Aeroqual.

Data Correction Approach

This evaluation report reflects "out-of-the-box" performance of the AQY. The manufacturer provides a procedure by which local collocation (sensor operated along side an FRM/FEM) data can be collected, a gain (slope) and offset (intercept) determined, and parameters entered into the Aeroqual Cloud user interface to be applied to all subsequently collected data. This procedure and feature was **not** used prior to this evaluation. Prospective consumers may get different performance from this device if they utilize this feature.

After acquisition, the raw data was processed using the *sensortoolkit* python code library (v0.8.3b2). A continuous data set at the recorded sampling frequency was written to a .csv file. 1-hour averaged data sets were generated using a 75% completeness threshold and saved as separate .csv files. Outliers were **not** removed from data sets in order to assess "out-of-the-box" sensor performance.

The duration of the warm-up period required for sensor measurements to equilibrate was determined from field data to be 10 minutes. Warm up periods were considered to occur following any power outage to sensor units, either due to routine field visits or unscheduled site power outages. Data recorded during warm up periods has been removed from data sets.

Issues Encountered

Pre-deployment observations

- *Timestamp inaccuracies*: During pre-deployment, the AQY devices did not properly sync timestamps with the onboard Real-Time Clock. Connecting the units to the internet by cellular or Wi-Fi allowed the unit to sync with internet time and resulted in proper timestamps.

Field observations and sensor data flags

The AQY unit was deployed at the South Dekalb monitoring site on 8/1/2019. Due to a miscommunication, the sensor unit was temporarily removed from the site on 8/28/2019 and was reinstalled in the previous configuration on 9/11/2019.

The following table contains data flags describing events that were encountered during the testing period. On 9/11/2019, a brief power outage was reported and lasted less than 10 minutes. The field technician was not at the site when the outage occurred, indicating that the cause of the outage was likely not attributed to operator error. The AQY unit was shut down temporarily during multiple occasions during the testing period, with the duration of shut down ranging from approximately 10-15 minutes. On 10/9/2019 during a routine site visit, the field technician noticed that the AQY unit's USB cable required reseating, as it had loosened since the prior site visit.

Start Time (UTC)	End Time (UTC)	Sensor Serial ID	Parameters Impacted	Flag
2019-09-11 21:08:00+00:00	2019-09-11 21:08:00+00:00	AQY_01	ALL	0-Sensor deployment
2019-09-16 19:38:00+00:00	2019-09-16 19:45:00+00:00	AQY_01	ALL	9-Probable power loss
2019-10-02 20:12:00+00:00	2019-10-02 20:12:00+00:00	AQY_01	ALL	3-Intentional shutdown for maintenance
2019-10-02 20:20:00+00:00	2019-10-02 20:20:00+00:00	AQY_01	O3	13-Data Incomplete - Sensor Malfunction - Gas Data
2019-10-02 20:22:00+00:00	2019-10-02 20:22:00+00:00	AQY_01	ALL	3-Intentional shutdown for maintenance
2019-10-02 20:30:00+00:00	2019-10-02 20:30:00+00:00	AQY_01	O3	13-Data Incomplete - Sensor Malfunction - Gas Data
2019-10-09 20:49:00+00:00	2019-10-09 20:49:00+00:00	AQY_01	ALL	3-Intentional shutdown for maintenance
2019-10-09 20:50:00+00:00	2019-10-09 21:03:00+00:00	AQY_01	ALL	4-Routine sensor maintenance - Sensor needed USB reformat

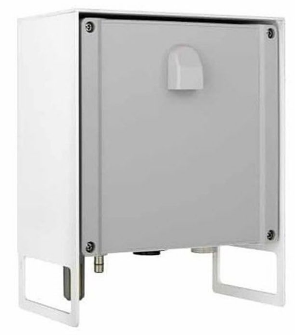

Image Attribution: Aeroqual Limited (Auckland, New Zealand)

Description of Data Flags

AQS

The U.S. EPA's Air Quality System (AQS) is the Agency's primary ambient air monitoring data archive. A comprehensive list of data flags that are recorded alongside AQS data sets, referred to by U.S. EPA as 'qualifiers', can be found at the following link: https://aqs.epa.gov/aqsweb/documents/codetables/qualifiers.html

Invalidation of reference data: AQS qualifiers are organized by qualifier type, which indicates whether data logged alongside qualifier flags should be invalidated (set null). Qualifiers with type "Null Data Qualifier" are invalidated, and includes data logged during periods that coincide with QC checks (e.g., "BF-Precision/Zero/Span", "BJ- Operator Error", "BL - QA Audit", "AZ - QC Audit") among other events such as power outages. Data logged alongside qualifiers with type "Quality Assurance Qualifiers" are not invalidated and are included in this analysis (e.g., concentrations less than the federal MDL for the reference monitor "MD – Value less than MDL", QA reviewed values "Validated Value").

Data Flags Recorded During Testing

FRM/FEM Monitor	Timestamp (UTC)	Flag
Thermo Scientific Model 49i (Acquired via AQS)	2019-09-11 07:00:00+0000 to 2019-09-11 11:00:00+0000	MD - Value less than MDL
	2019-09-12 02:00:00+0000 to 2019-09-12 12:00:00+0000	MD - Value less than MDL
	2019-09-13 02:00:00+0000 to 2019-09-13 12:00:00+0000	MD - Value less than MDL
	2019-09-15 10:00:00+0000 to 2019-09-15 12:00:00+0000	MD - Value less than MDL
	2019-09-16 03:00:00+0000 to 2019-09-16 12:00:00+0000	MD - Value less than MDL
	2019-09-16 08:00:00+0000 to 2019-09-16 09:00:00+0000	BF - Precision/Zero/Span
	2019-09-17 02:00:00+0000 to 2019-09-17 12:00:00+0000	MD - Value less than MDL
	2019-09-18 06:00:00+0000 to 2019-09-18 09:00:00+0000	MD - Value less than MDL
	2019-09-18 13:00:00+0000	BJ - Operator Error
	2019-09-19 13:00:00+0000 to 2019-09-19 14:00:00+0000	BL - QA Audit
	2019-09-21 07:00:00+0000 to 2019-09-21 12:00:00+0000	MD - Value less than MDL
	2019-09-22 03:00:00+0000 to 2019-09-22 12:00:00+0000	MD - Value less than MDL
	2019-09-23 02:00:00+0000 to 2019-09-23 12:00:00+0000	MD - Value less than MDL
	2019-09-23 08:00:00+0000 to 2019-09-23 09:00:00+0000	BF - Precision/Zero/Span
	2019-09-24 02:00:00+0000 to 2019-09-24 12:00:00+0000	MD - Value less than MDL
	2019-09-25 02:00:00+0000 to 2019-09-25 12:00:00+0000	MD - Value less than MDL
	2019-09-26 01:00:00+0000 to 2019-09-26 12:00:00+0000	MD - Value less than MDL
	2019-09-27 02:00:00+0000 to 2019-09-27 12:00:00+0000	MD - Value less than MDL
	2019-09-29 03:00:00+0000 to 2019-09-29 08:00:00+0000	MD - Value less than MDL
	2019-09-29 10:00:00+0000 to 2019-09-29 12:00:00+0000	MD - Value less than MDL
	2019-09-30 01:00:00+0000 to 2019-09-30 13:00:00+0000	MD - Value less than MDL
	2019-09-30 08:00:00+0000 to 2019-09-30 09:00:00+0000	BF - Precision/Zero/Span
	2019-09-30 20:00:00+0000	V - Validated Value
	2019-10-01 01:00:00+0000 to 2019-10-01 04:00:00+0000	MD - Value less than MDL
	2019-10-01 05:00:00+0000 to 2019-10-01 11:00:00+0000	MD - Value less than MDL
	2019-10-02 03:00:00+0000 to 2019-10-02 12:00:00+0000	MD - Value less than MDL
	2019-10-03 01:00:00+0000 to 2019-10-03 12:00:00+0000	MD - Value less than MDL
	2019-10-03 19:00:00+0000	V - Validated Value
	2019-10-04 02:00:00+0000 to 2019-10-04 12:00:00+0000	MD - Value less than MDL
	2019-10-05 02:00:00+0000	MD - Value less than MDL
	2019-10-05 04:00:00+0000 to 2019-10-05 06:00:00+0000	MD - Value less than MDL
	2019-10-07 08:00:00+0000 to 2019-10-07 09:00:00+0000	BF - Precision/Zero/Span
	2019-10-07 10:00:00+0000	MD - Value less than MDL
	2019-10-08 07:00:00+0000 to 2019-10-08 11:00:00+0000	MD - Value less than MDL
	2019-10-10 06:00:00+0000 to 2019-10-10 11:00:00+0000	MD - Value less than MDL
	2019-10-11 02:00:00+0000 to 2019-10-11 12:00:00+0000	MD - Value less than MDL

Initial Base Testing - Decatur, GA
U.S. Environmental Protection Agency
Office of Research and Development
PI: Clements.Andrea@epa.gov
919-541-1363
September 2019—October 2019

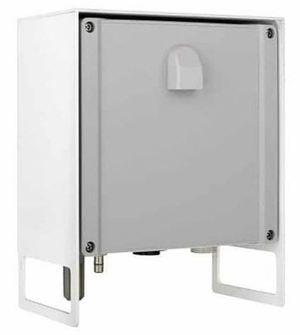
Image Attribution: Aeroqual Limited (Auckland, New Zealand)

Data Flags Recorded During Testing (Cont.)

Meteorological Instrument	Timestamp (UTC)	Flag
RM Young 41375VC Temperature Monitor (Data acquired via AQS)	2019-09-27 13:00:00+0000 to 2019-09-27 14:00:00+0000	BA - Maintenance/Routine Repairs
[Make Model] Relative Humidity Monitor (Data acquired via AQS)	2019-09-27 13:00:00+0000 to 2019-09-27 14:00:00+0000	BA - Maintenance/Routine Repairs

Testing Report - O_3 Base Testing

SENSIT RAMP

This report reflects out-of-the-box performance

Initial Base Testing - Decatur, GA

U.S. Environmental Protection Agency
Office of Research and Development
PI: Clements.Andrea@epa.gov
919-541-1363
November 2019—December 2019

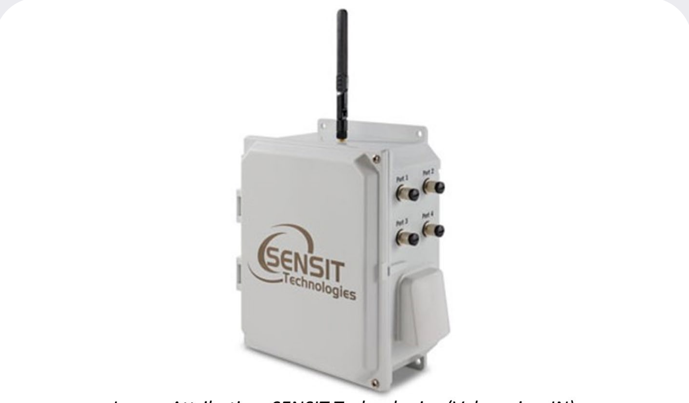
Image Attribution: SENSIT Technologies (Valparaiso, IN)

Deployment Details

Testing Organization and Site Information	
Testing organization (Name, Organization type, Contact website)	U.S. Environmental Protection Agency - Office of Research and Development<br>Federal Government<br>Air Sensor Toolbox | U.S. EPA Website
Testing location (City, State, Latitude and Longitude)	South Dekalb<br>Decatur, GA<br>33.6877, -84.2905
AQS site ID	13 - 089 - 0002
Sampling timeframe (MM-DD-YY)	11-28-19 to 12-28-19
Sensor data source	Onboard MicroSD card
Reference data source	AQS API download

Sensor Information			
Manufacturer, model	SENSIT RAMP		
Device firmware version	190313_AQ_v9.30		
Sampling time interval	15-seconds		
Sensor serial numbers	RAM_02		
Issues encountered during deployment?	☐	No Issues	

FRM/FEM Information	
Manufacturer, model, designation	Thermo Scientific Model 49i FEM
Sampling time interval	1-hour averaging
Date of calibration	As required by 40 CFR Part 58 and the Ambient Air Monitoring Plan maintained by GA DNR
Date of one-point QC check	Every two weeks as required by 40 CFR Part 58 Appendix A 3.1.1
Description, date(s) of maintenance activities	See GA-AQY-Page 6

Time Series Plot: 1-hour averaged O_3

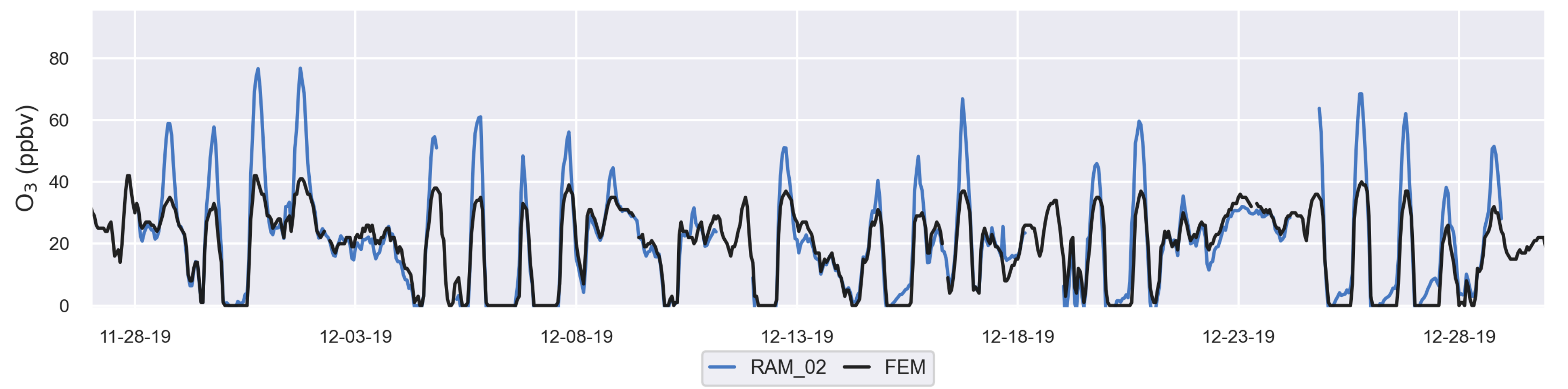

Range and average of FRM/FEM concentrations over duration of base test (ppbv)	[1-hr] 0.0-42.0, avg: 18.4, [Rolling 8-hr] 0.0-38.5, avg: 18.3
Number of 1-hr periods in FRM/FEM monitor measurements with a goal concentration ≥ 60 ppbv	0

Scatter Plot: Comparison to FRM/FEM

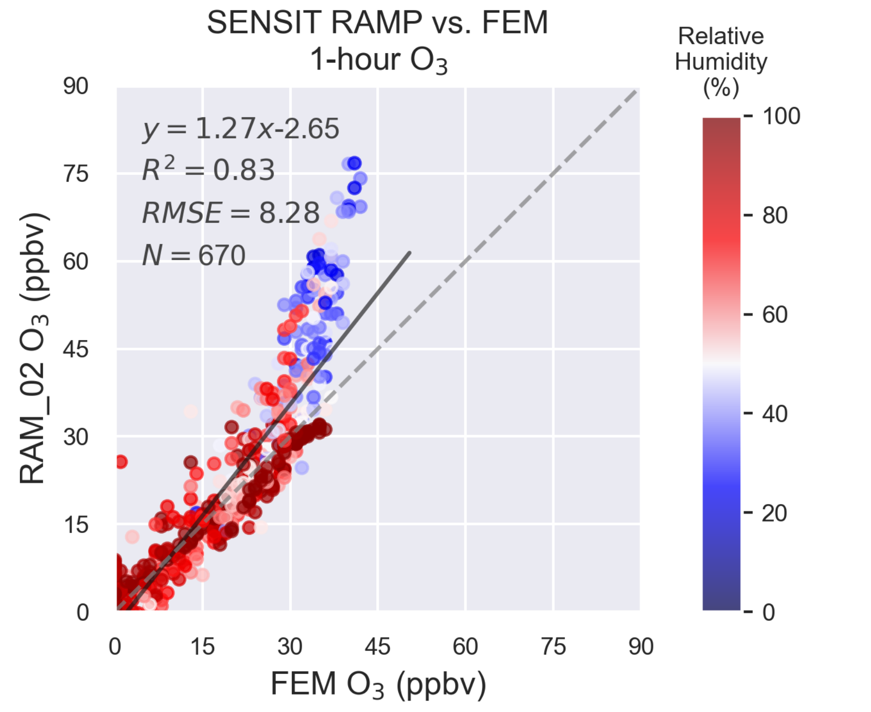

Performance Metrics

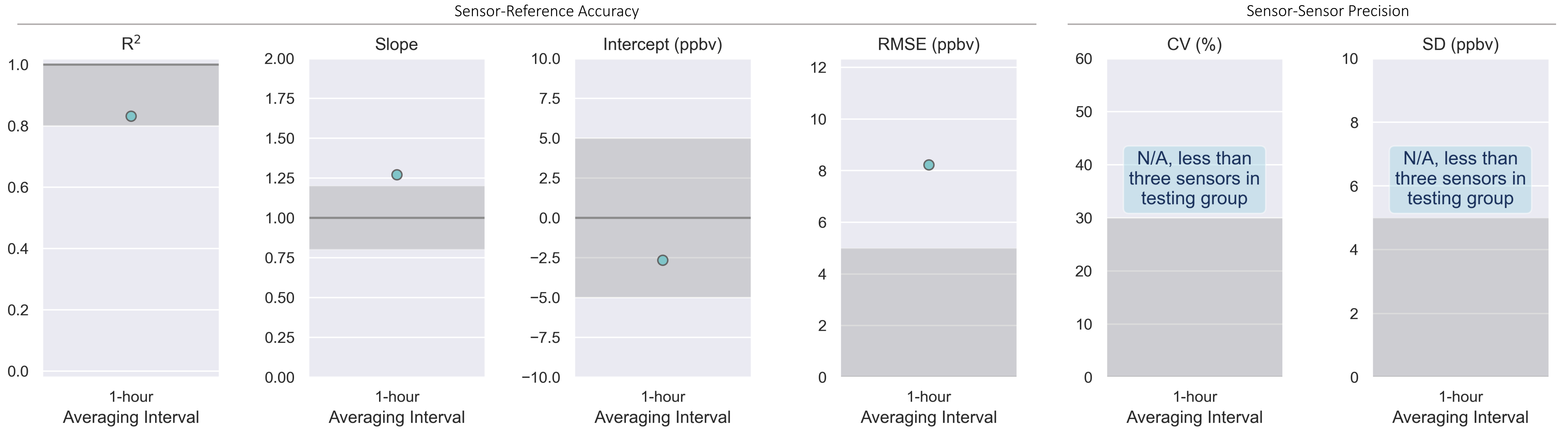

Meteorological Conditions During Deployment

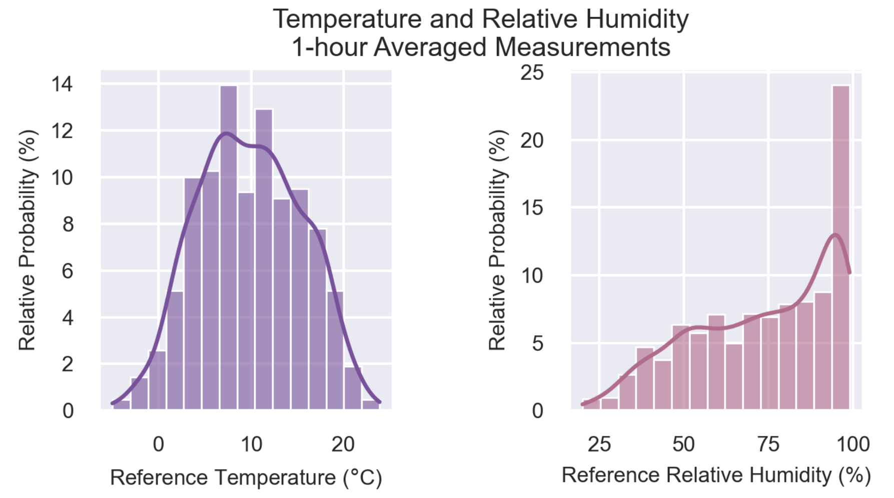

Number of 1-hr periods outside sensor manufacturer-listed temperature operational range (-20 to 50 °C)	0
Number of 1-hr periods outside sensor manufacturer-listed relative humidity operational range (no operational range specified)	-

Meteorological Influence

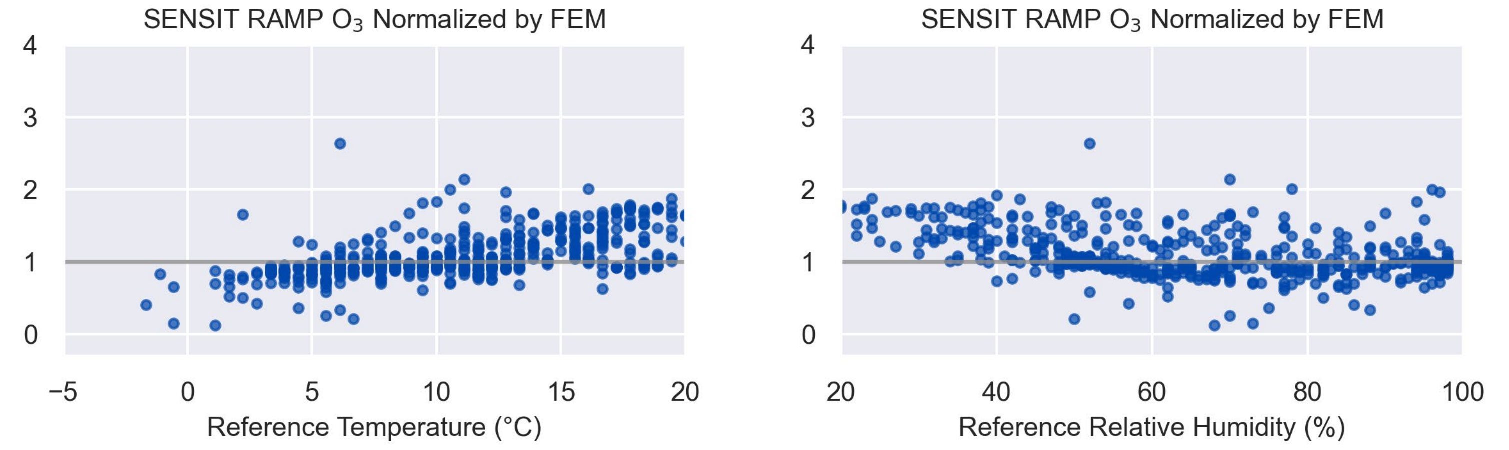

Mean number of paired, normalized concentration and temperature values (1-hr averages)	744
Mean number of paired, normalized concentration and relative humidity values (1-hr averages)	744

FEM data <5 ppbv (Federal MDL for the 49i) has been remove (Meteorological Influence section only)

Testing Report - O_3 Base Testing

SENSIT RAMP

This report reflects out-of-the-box performance

Initial Base Testing - Decatur, GA

U.S. Environmental Protection Agency
Office of Research and Development
PI: Clements.Andrea@epa.gov
919-541-1363

November 2019—December 2019

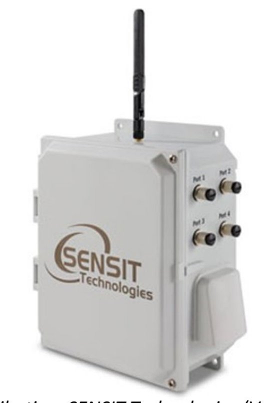

Image Attribution: SENSIT Technologies (Valparaiso, IN)

Tabular Statistics

Sensor-FRM/FEM Correlation

	Bias and Linearity			Data Quality	
	R^2	Slope	Intercept (ppbv)	Uptime (%)	Number of paired sensor and reference concentration values*
	1-Hour ●	1-Hour ○	1-Hour ●	1-Hour ●	1-Hour
Metric Target Range	≥ 0.80	1.0 ± 0.20	-5 ≤ b ≤ 5	75%*	-
Sensor RAM_02	0.83	1.27	-2.65	92	670

	Error
	RMSE (ppbv)
	1-Hour ☆
Metric Target Range	≤ 5.0
Deployment Value	8.2

Device-specific metrics (computed for each sensor in evaluation)
- ○ Metric value for none of devices tested falls within the target range
- ● Metric value for one of devices tested falls within the target range

Sensor-Sensor Precision[1]

	Precision (between collocated sensors)		Data Quality
	CV (%)	SD (ppbv)	Number of paired sensor and reference concentration values*
	1-Hour ☆	1-Hour ☆	1-Hour
Metric Target Range	≤ 30.0	≤ 5.0	-
Deployment Value	-	-	-

[1]Precision statistics are computed for evaluations with at least three collocated sensor units. Metric values are left blank for evaluations with two or fewer sensor units.

Single-valued metrics (computed via entire evaluation dataset)
- ☆ Indicates that the metric value is not within the target range
- ★ Indicates that the metric value is within the target range

*This value is only a recommendation for ensuring data quality and is not included in the list of target values discussed in Section 4 of the Performance Testing Protocols, Metrics, and Target Values for Ozone Air Sensors document.

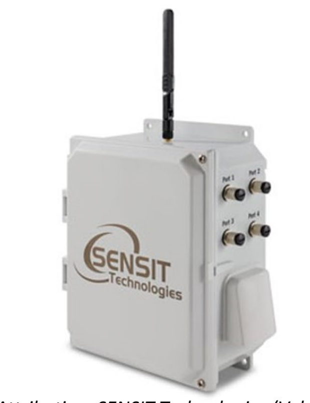

Image Attribution: SENSIT Technologies (Valparaiso, IN)

Supplemental Information

Abbreviations used in Supplemental Information

FRM	Federal Reference Method
FEM	Federal Equivalent Method
SOP	Standard Operating Procedure
QAPP	Quality Assurance Project Plan
QC	Quality Control

Supplemental Documentation	Attached	Description & URL or file path to documentation
Field observations and sensor data flags	☑	See GA-RAM-Page 5 of this testing report
Maintenance logs	☐	No logs recorded during testing
Standard operating procedure(s)	☐	U.S. EPA Office Of Research and Development SOP available upon request
Photos of equipment setup and testing	☑	See GA-RAM-Page 4 of this testing report
Product specifications sheet(s)	☑	See Appendix C, "Spec_Sheet_SENSIT_RAMP.pdf"
Product manual(s)	☑	See Appendix C, "Manual_SENSIT_RAMP.pdf"
Data storage and transmission method	☑	See GA-RAM-Page 5 of this testing report
Data correction approach	☑	See GA-RAM-Page 5 of this testing report
Issues encountered	☑	See GA-RAM-Page 5 of this testing report
Data analysis/correction scripts and version	☑	Averaging and processing of data, calculation of performance metrics, and generation of figures and other supplementary material for analysis were obtained using Python 3.9.7 with the packages sensortoolkit v0.8.3b2, pandas 1.3.5, NumPy 1.21.2, Matplotlib 3.5.0, statsmodels 0.13.0, and seaborn 0.11.2. All packages are available from the Python Package Index (PyPI) at https://pypi.org. The integrated development environment (IDE) Spyder 5.1.5 was used for scripting and data visualization. Version control for the Python base, packages, and IDE were all managed by conda 4.11.0.
Air Monitoring Station QAPP	☐	U.S. EPA Office Of Research and Development QAPP available upon request
Summary of FRM/FEM monitor QC checks	☑	See GA-RAM-Page 6 of this testing report
Manufacturer website for FRM/FEM monitor	☑	ThermoFisher Scientific: Model 49i Product website
FRM/FEM monitor manual	☑	See Appendix B, "Spec_Sheet_Thermo_49i.pdf"
FRM/FEM monitor specifications sheet(s)	☑	See Appendix B, "Manual_Thermo_49i.pdf"
Other documents	☐	

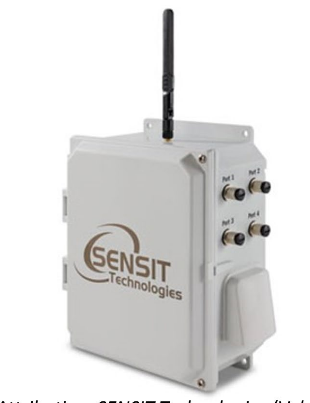

Site Description:
The South Dekalb monitoring station was established as O3 site located in Decatur, Georgia.

Figure 1: SENSIT RAMP sensor (indicated by red arrow) attached to metal railing atop the sampling shelter at the monitoring site.

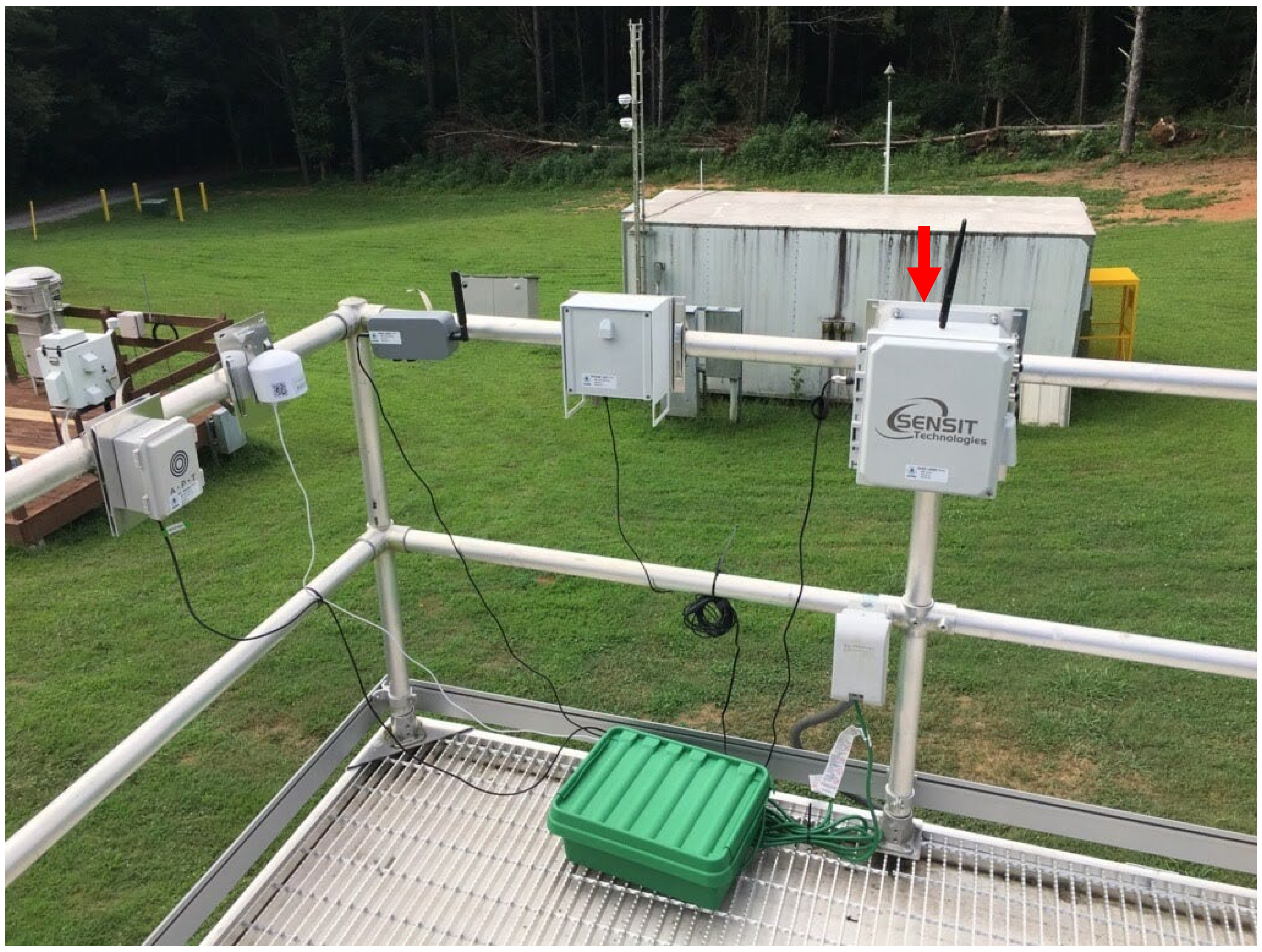

Figure 2: South Dekalb Monitoring Station

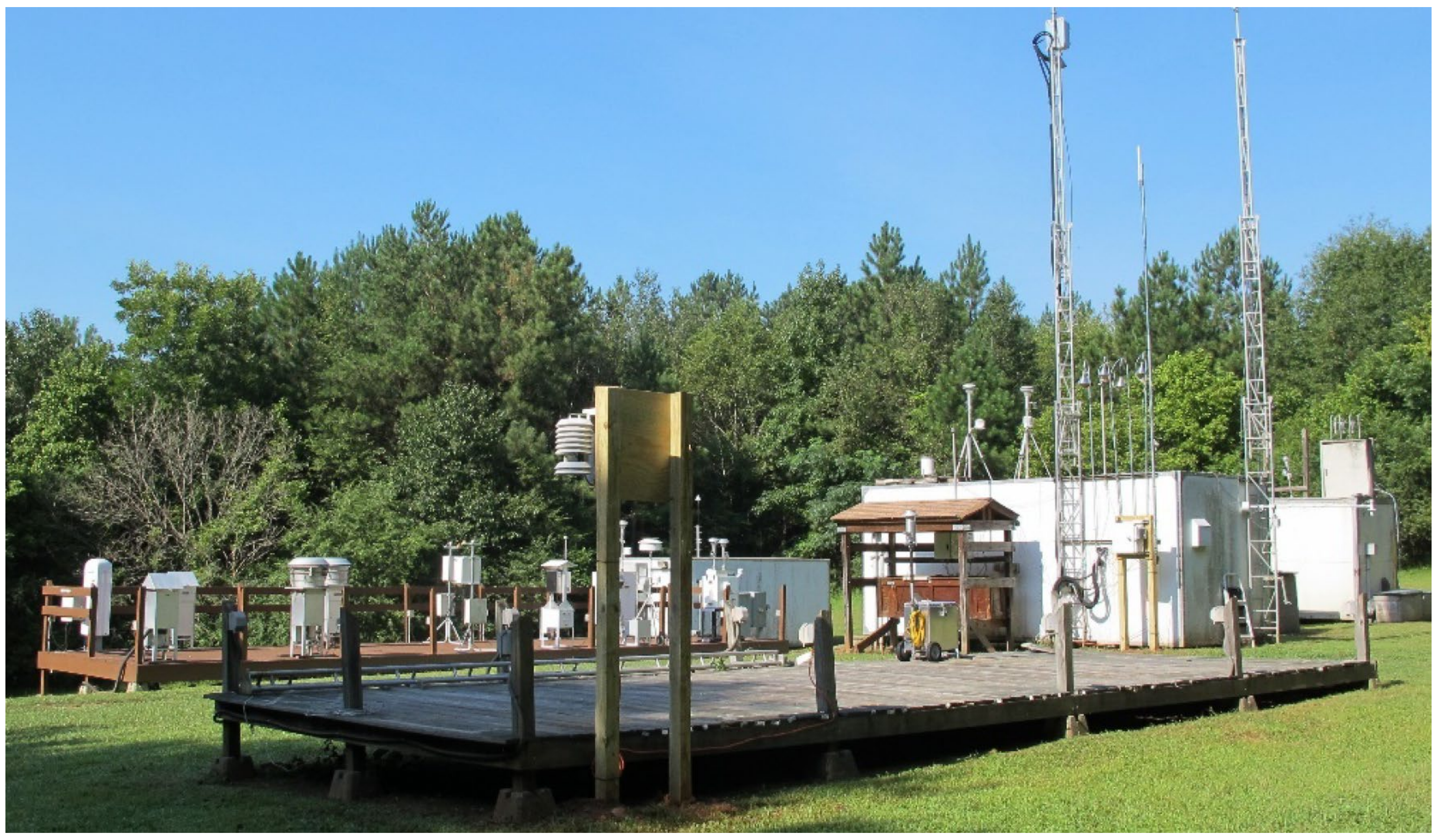

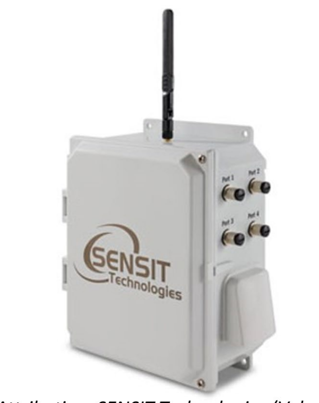

Image Attribution: SENSIT Technologies (Valparaiso, IN)

Supplemental Information: Data Storage, Correction Approach, and Issues Encountered

Data Storage and Transmission Method

The SENSIT RAMP was configured to record data at a 15-second sampling interval. Data are stored as daily text files (.txt format) on an onboard MicroSD card. Data files were obtained weekly via SD cards. Each field site operator was provided two labeled MicroSD cards for sensor units that they used to swap out each week. Data from the collected card was then read and processed off-site.

Data Correction Approach

SENSIT RAMP units were pre-configured by the manufacturer with a linear correction (i.e., concentration gain = 1.0 and offset = 0.0 ppbv). These presets reflect out-of-the box performance and were not modified by EPA prior or during testing.

After acquisition, the raw data was processed using the *sensortoolkit* python code library (v0.8.3b2). A continuous data set at the recorded sampling frequency was written to a .csv file. 1-hour averaged data sets were generated using a 75% completeness threshold and saved as separate .csv files. Outliers were **not** removed from data sets in order to assess “out-of-the-box” sensor performance.

The duration of the warm-up period required for sensor measurements to equilibrate was determined during bench-top testing (additional detail in pre-deployment observations) to be approximately 2 hours. Data recorded during warm up periods has been removed from data sets.

Issues Encountered

Pre-deployment observations

- *Changing logging interval*: SENSIT RAMP units were received without documentation or manuals. After communicating the need to change default settings (logging interval and time zone) with the manufacturer, a draft user’s manual and a USB cable were supplied. With the use of this USB cable, instrument settings could be changed, and real-time data could be logged using a serial communication software (CoolTerm, v.1.5.0). Because the sensor did not record data at the top of every minute, the RAMP was configured to record data at 15-second intervals so that the data could be averaged more closely to complete minutes.
- *Gas Sensor Warmup*: Prior to deployment, RAMP units were collocated in a bench-top evaluation to verify operational status and determine the extent of data invalidity (i.e., determine equilibration period) after an initial start-up event. The recorded response for parameters measured by the RAMP suggests that the gas sensors (CO, NO, NO_2, O_3) required approximately a 2-hour equilibration period, while the remaining sensors (temperature, relative humidity, particulate matter) did not require any equilibration period.

Field observations and sensor data flags

- Shipping damage: During shipment of the SENSIT RAMP to the South Dekalb site in August 2019, the initial unit was severely damaged due to a failure of the main bracket holding the unit’s battery pack and main circuit board within the device enclosure. A replacement unit (referred to in this report as RAM_01) replaced the initial unit and was deployed on 8/15/2019.
- Field technician miscommunication: Due to a miscommunication, the replacement unit was temporarily removed from the site on 8/28/2019 and was reinstalled on 9/11/2019.

During the testing period described here, the RAMP operated nominally and did not require replacement or repair.

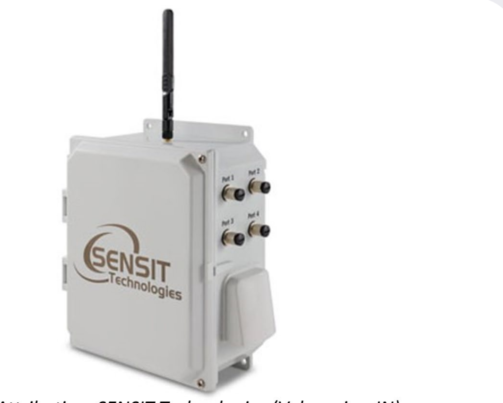

Image Attribution: SENSIT Technologies (Valparaiso, IN)

Supplemental Information: Description of FRM/FEM QC Checks and Data Flags

Description of Data Flags

AQS

The U.S. EPA's Air Quality System (AQS) is the Agency's primary ambient air monitoring data archive. A comprehensive list of data flags that are recorded alongside AQS data sets, referred to by U.S. EPA as 'qualifiers', can be found at the following link: https://aqs.epa.gov/aqsweb/documents/codetables/qualifiers.html

Invalidation of reference data: AQS qualifiers are organized by qualifier type, which indicates whether data logged alongside qualifier flags should be invalidated (set null). Qualifiers with type "Null Data Qualifier" are invalidated, and includes data logged during periods that coincide with QC checks (e.g., "BF-Precision/Zero/Span", "BJ- Operator Error", "BL - QA Audit", "AZ - QC Audit") among other events such as power outages. Data logged alongside qualifiers with type "Quality Assurance Qualifiers" are not invalidated and are included in this analysis (e.g., concentrations less than the federal MDL for the reference monitor "MD – Value less than MDL", QA reviewed values "Validated Value").

Data Flags Recorded During Testing

FRM/FEM Monitor	Timestamp (UTC)	Flag
Thermo Scientific Model 49i (Acquired via AQS)	2019-11-29 12:00:00+0000 to 2019-11-29 13:00:00+0000	MD - Value less than MDL
	2019-11-30 00:00:00+0000 to 2019-11-30 13:00:00+0000	MD - Value less than MDL
	2019-12-02 08:00:00+0000 to 2019-12-02 09:00:00+0000	BF - Precision/Zero/Span
	2019-12-04 08:00:00+0000	MD - Value less than MDL
	2019-12-04 11:00:00+0000 to 2019-12-04 12:00:00+0000	MD - Value less than MDL
	2019-12-05 03:00:00+0000 to 2019-12-05 05:00:00+0000	MD - Value less than MDL
	2019-12-05 10:00:00+0000 to 2019-12-05 13:00:00+0000	MD - Value less than MDL
	2019-12-05 23:00:00+0000 to 2019-12-06 15:00:00+0000	MD - Value less than MDL
	2019-12-07 01:00:00+0000 to 2019-12-07 14:00:00+0000	MD - Value less than MDL
	2019-12-09 08:00:00+0000 to 2019-12-09 09:00:00+0000	BF - Precision/Zero/Span
	2019-12-10 00:00:00+0000 to 2019-12-10 02:00:00+0000	MD - Value less than MDL
	2019-12-10 05:00:00+0000 to 2019-12-10 07:00:00+0000	MD - Value less than MDL
	2019-12-12 03:00:00+0000 to 2019-12-12 12:00:00+0000	MD - Value less than MDL
	2019-12-14 06:00:00+0000 to 2019-12-14 09:00:00+0000	MD - Value less than MDL
	2019-12-15 01:00:00+0000 to 2019-12-15 14:00:00+0000	MD - Value less than MDL
	2019-12-16 08:00:00+0000 to 2019-12-16 09:00:00+0000	BF - Precision/Zero/Span
	2019-12-19 12:00:00+0000	MD - Value less than MDL
	2019-12-20 01:00:00+0000 to 2019-12-20 14:00:00+0000	MD - Value less than MDL
	2019-12-21 02:00:00+0000 to 2019-12-21 03:00:00+0000	MD - Value less than MDL
	2019-12-23 08:00:00+0000 to 2019-12-23 09:00:00+0000	BF - Precision/Zero/Span
	2019-12-25 01:00:00+0000 to 2019-12-25 13:00:00+0000	MD - Value less than MDL
	2019-12-26 01:00:00+0000 to 2019-12-26 14:00:00+0000	MD - Value less than MDL
	2019-12-27 00:00:00+0000 to 2019-12-27 13:00:00+0000	MD - Value less than MDL
	2019-12-28 00:00:00+0000 to 2019-12-28 03:00:00+0000	MD - Value less than MDL
	2019-12-28 07:00:00+0000 to 2019-12-28 08:00:00+0000	MD - Value less than MDL

Testing Report - O_3 Base Testing

Aeroqual AQY

This report reflects out-of-the-box performance

Initial Base Testing - RTP, NC

U.S. Environmental Protection Agency
Office of Research and Development
PI: Clements.Andrea@epa.gov
919-541-1363
August 2019—September 2019

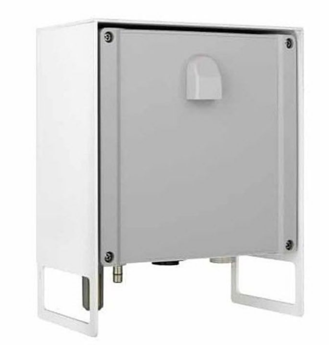

Image Attribution: Aeroqual Limited (Auckland, New Zealand)

Deployment Details

Testing Organization and Site Information	
Testing organization (Name, Organization type, Contact website)	U.S. Environmental Protection Agency - Office of Research and Development<br>Federal Government<br>Air Sensor Toolbox | U.S. EPA Website
Testing location (City, State, Latitude and Longitude)	Ambient Monitoring Innovative Research Station (AIRS)<br>RTP, NC<br>35.88951, -78.874572
AQS site ID	37 – 063 – 0099
Sampling timeframe (MM-DD-YY)	08-02-19 to 09-01-19
Sensor data source	Aeroqual Cloud
Reference data source	OAQPS file transfer

Sensor Information			
Manufacturer, model	Aeroqual AQY		
Device firmware version	1.14.2		
Sampling time interval	1-minute		
Sensor serial numbers	AQY_01	AQY_02	AQY_03
Issues encountered during deployment?	☐	No Issues	

FRM/FEM Information	
Manufacturer, model, designation	Teledyne API T265 FEM
Sampling time interval	1-hour averaging
Date of calibration	As required by 40 CFR Part 58 and the Burdens Creek QAPP maintained by OAQPS
Date of one-point QC check	At least every two weeks as required by 40 CFR Part 58 Appendix A 3.1.1
Description, date(s) of maintenance activities	N/A

Time Series Plot: 1-hour averaged O_3

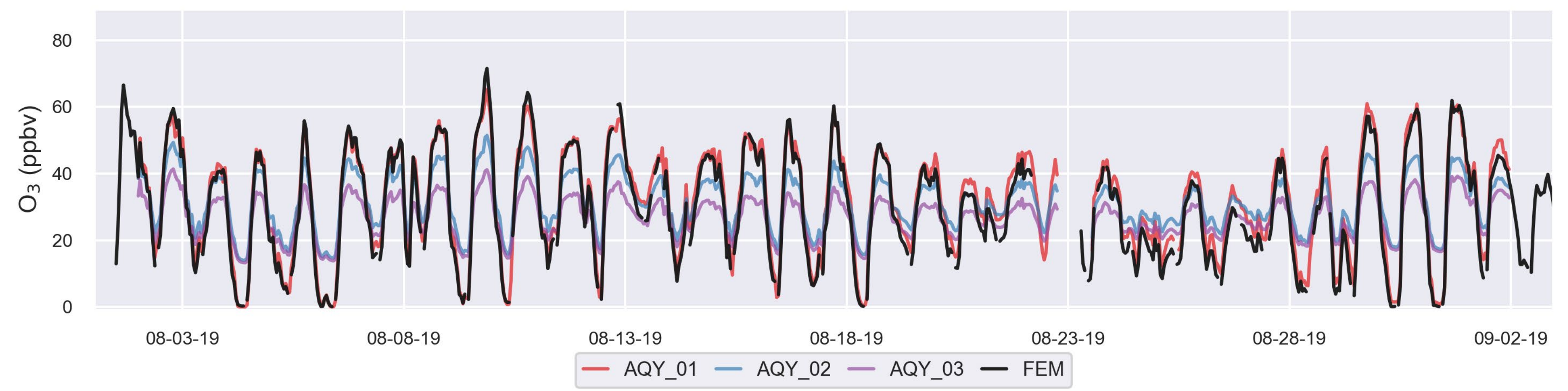

Range and average of FRM/FEM concentrations over duration of base test (ppbv)	[1-hr] 0.1-71.5, avg: 29.5,<br>[Rolling 8-hr] 1.2-61.7, avg: 33.9
Number of 1-hr periods in FRM/FEM monitor measurements with a goal concentration ≥ 60 ppbv	13

Scatter Plot: Comparison to FRM/FEM

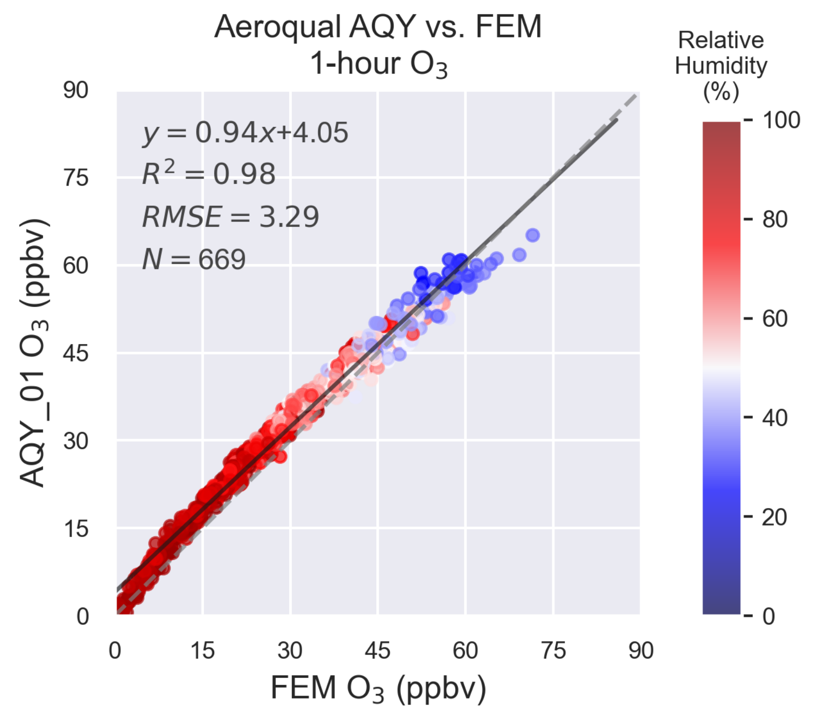

Performance Metrics★

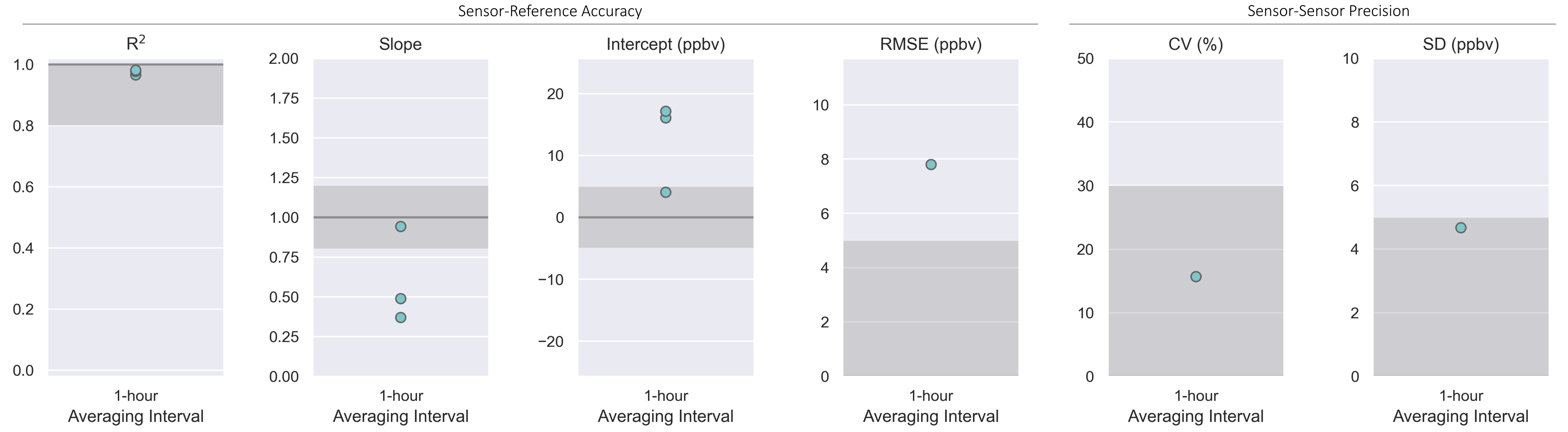

Meteorological Conditions During Deployment

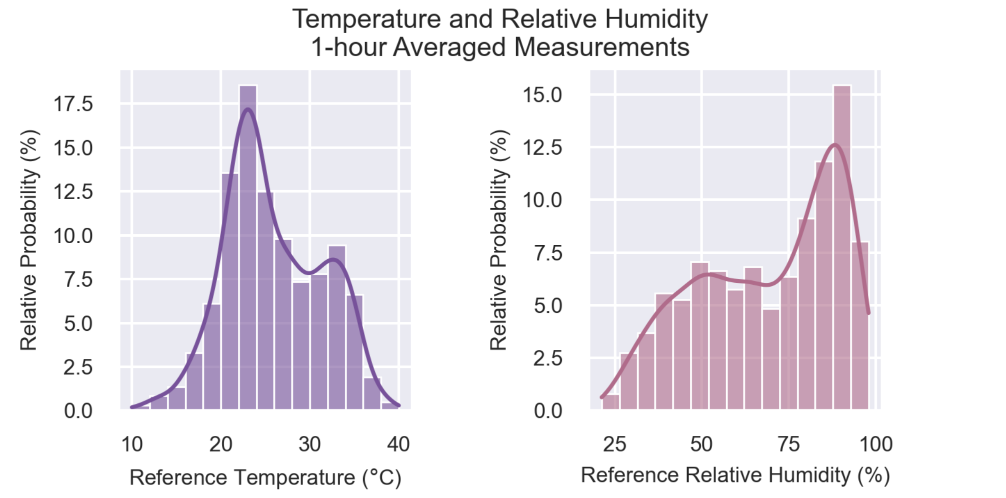

Number of 1-hr periods outside sensor manufacturer-listed temperature operational range (-10 to 40 °C)	0
Number of 1-hr periods outside sensor manufacturer-listed relative humidity operational range (no operational range specified)	-

Meteorological Influence

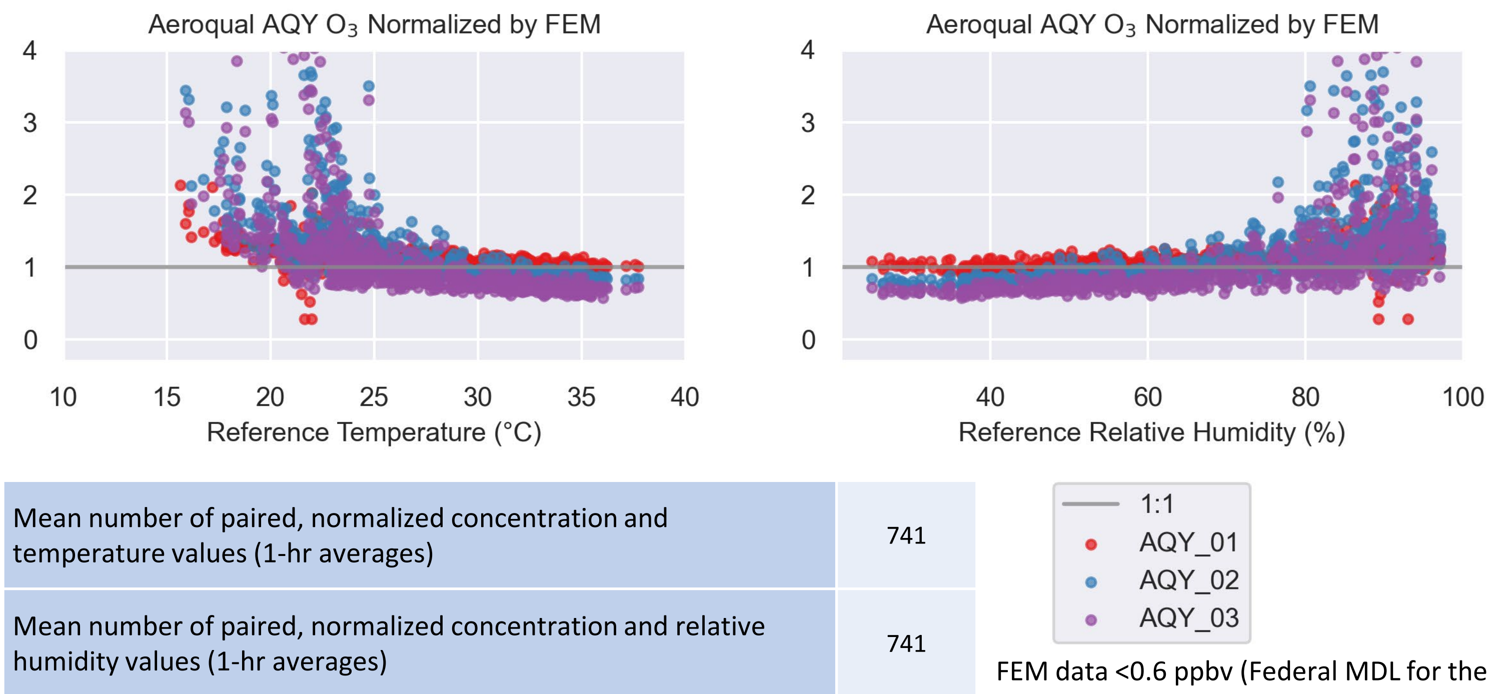

Mean number of paired, normalized concentration and temperature values (1-hr averages)	741
Mean number of paired, normalized concentration and relative humidity values (1-hr averages)	741

FEM data <0.6 ppbv (Federal MDL for the T265) has been remove (Meteorological Influence section only)

★For evaluations with greater than three sensors, grouping individual sensor metrics into boxplots is recommended for displaying results. Note that this recommendation does not apply to metrics computed as a single value for all sensors over the whole evaluation group, such as RMSE, NRMSE, CV, and standard deviation.

Testing Report - O_3 Base Testing

Aeroqual AQY

This report reflects out-of-the-box performance

Initial Base Testing - RTP, NC
U.S. Environmental Protection Agency
Office of Research and Development
PI: Clements.Andrea@epa.gov
919-541-1363
August 2019—September 2019

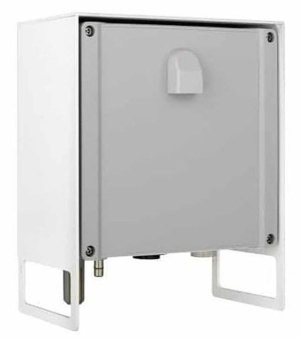
Image Attribution: Aeroqual Limited (Auckland, New Zealand)

Tabular Statistics

Sensor-FRM/FEM Correlation

	Bias and Linearity			Data Quality	
	R²	Slope	Intercept (ppbv)	Uptime (%)	Number of paired sensor and reference concentration values*
	1-Hour ●●●	1-Hour ●○○	1-Hour ●○○	1-Hour ●●●	1-Hour
Metric Target Range	≥ 0.80	1.0 ± 0.20	-5 ≤ b ≤ 5	75%*	-
Sensor AQY_01	0.98	0.94	4.05	97	669
Sensor AQY_02	0.98	0.49	17.13	98	670
Sensor AQY_03	0.97	0.37	16.06	98	670
Mean	0.97	0.60	12.41	97.49	669.67

	Error
	RMSE (ppbv)
	1-Hour ☆
Metric Target Range	≤ 5.0
Deployment Value	7.8

Device-specific metrics (computed for each sensor in evaluation)
○○○ Metric value for none of devices tested falls within the target range
●○○ Metric value for one of devices tested falls within the target range
●●○ Metric value for two of devices tested falls within the target range
●●● Metric value for three of devices tested falls within the target range

Single-valued metrics (computed via entire evaluation dataset)
☆ Indicates that the metric value is not within the target range
★ Indicates that the metric value is within the target range

Sensor-Sensor Precision

	Precision (between collocated sensors)		Data Quality
	CV (%)	SD (ppbv)	Number of paired sensor and reference concentration values*
	1-Hour ★	1-Hour ★	1-Hour
Metric Target Range	≤ 30.0	≤ 5.0	-
Deployment Value	15.7	4.7	724

*This value is only a recommendation for ensuring data quality and is not included in the list of target values discussed in Section 4 of the Performance Testing Protocols, Metrics, and Target Values for Ozone Air Sensors document.

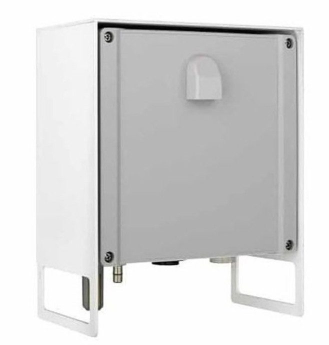

Image Attribution: Aeroqual Limited (Auckland, New Zealand)

Sensor-FRM/FEM Scatter Plots

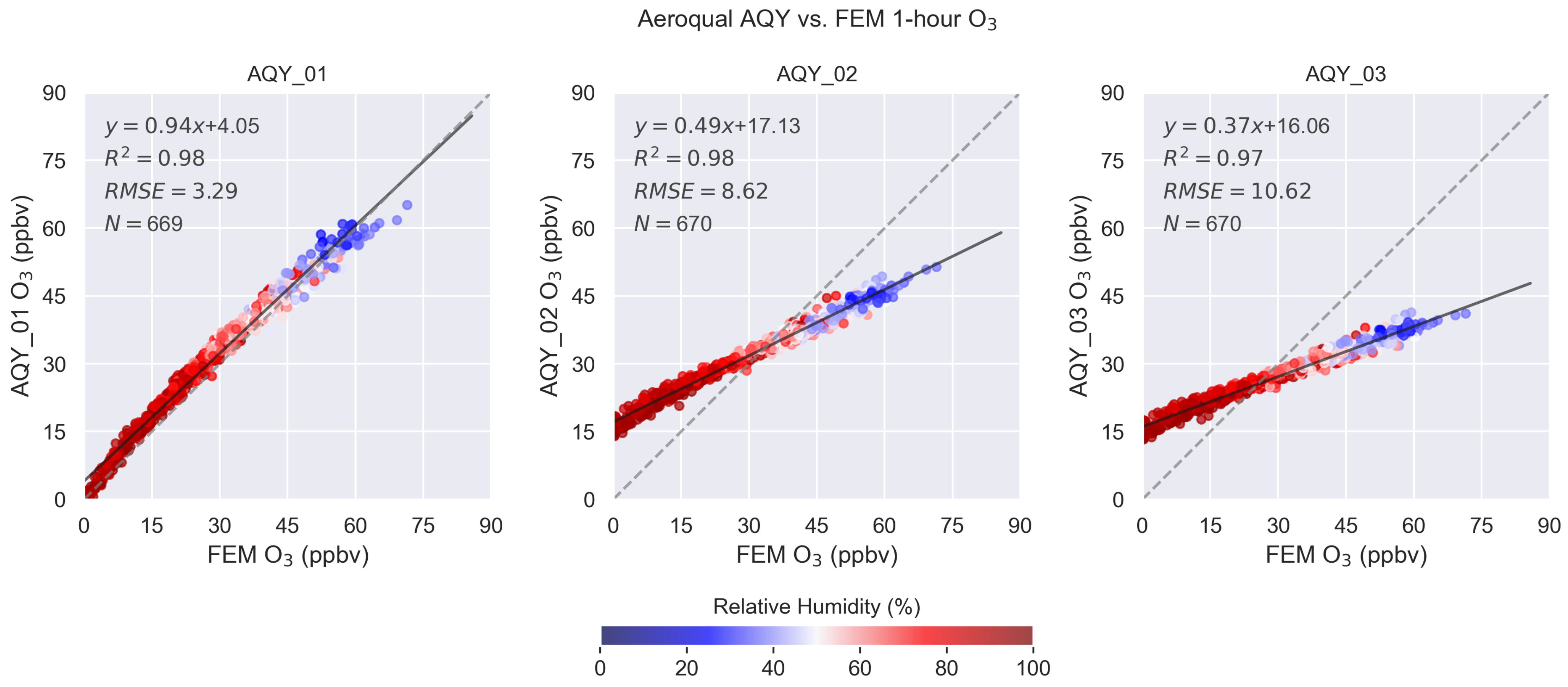

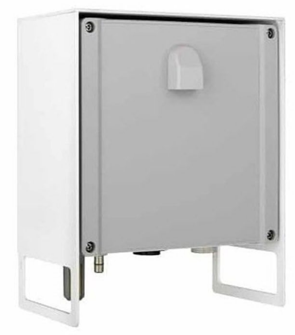

Image Attribution: Aeroqual Limited (Auckland, New Zealand)

Supplemental Information

Abbreviations used in Supplemental Information

FRM	Federal Reference Method
FEM	Federal Equivalent Method
SOP	Standard Operating Procedure
QAPP	Quality Assurance Project Plan
QC	Quality Control

Supplemental Documentation	Attached	Description & URL or file path to documentation
Field observations and sensor data flags	☑	See NC-AQY-Page 6 of this testing report
Maintenance logs	☐	No logs recorded during testing
Standard operating procedure(s)	☐	U.S. EPA Office Of Research and Development SOP available upon request
Photos of equipment setup and testing	☑	See NC-AQY-Page 5 of this testing report
Product specifications sheet(s)	☑	See Appendix C, “Spec_Sheet_Aeroqual_AQY.pdf”*
Product manual(s)	☑	See Appendix C, “Manual_Aeroqual_AQY.pdf”*
Data storage and transmission method	☑	See NC-AQY-Page 6 of this testing report
Data correction approach	☑	See NC-AQY-Page 6 of this testing report
Issues encountered	☑	See NC-AQY-Page 6 of this testing report
Data analysis/correction scripts and version	☑	Averaging and processing of data, calculation of performance metrics, and generation of figures and other supplementary material for analysis were obtained using Python 3.9.7 with the packages sensortoolkit v0.8.3b2, pandas 1.3.5, NumPy 1.21.2, Matplotlib 3.5.0, statsmodels 0.13.0, and seaborn 0.11.2. All packages are available from the Python Package Index (PyPI) at https://pypi.org. The integrated development environment (IDE) Spyder 5.1.5 was used for scripting and data visualization. Version control for the Python base, packages, and IDE were all managed by conda 4.11.0.
Air Monitoring Station QAPP	☐	U.S. EPA Office Of Research and Development QAPP available upon request
Summary of FRM/FEM monitor QC checks	☑	See NC-AQY-Pages 7-10 of this testing report
Manufacturer website for FRM/FEM monitor	☑	Teledyne API: T265 Product website
FRM/FEM monitor manual	☑	See Appendix B, “Spec_Sheet_TeledyneAPI_T265.pdf”
FRM/FEM monitor specifications sheet(s)	☑	See Appendix B, “Manual_TeledyneAPI_T265.pdf”
Other documents	☑	Manufacturer notice of AQY sales on hold

*As of 3/18/2021, the manufacturer of the AQY has placed sales of a similar unit on hold. Documentation for the AQY is currently unavailable from the manufacturer’s website.

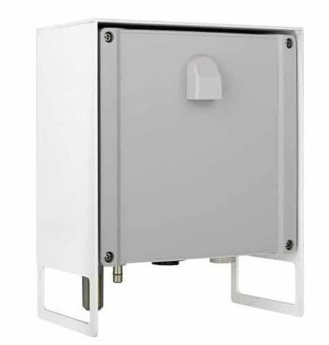

Image Attribution: Aeroqual Limited (Auckland, New Zealand)

Site Description:

The Burdens Creek Ambient Monitoring Innovation Research Station (AIRS) site is located on the U.S. EPA, RTP campus and is situated between Alexander Drive and Route 147. The site is intended to represent a neighborhood-scale site as defined in *40 CFR Part 58, Appendix D*. U.S. EPA's Office of Air Quality Planning and Standards (OAQPS) operates reference grade instruments in a free-standing shelter situated directly adjacent to the sensor testing platform.

Figure 1: Aeroqual AQY sensors (indicated by red arrow) attached to mounting assembly at evaluation site.

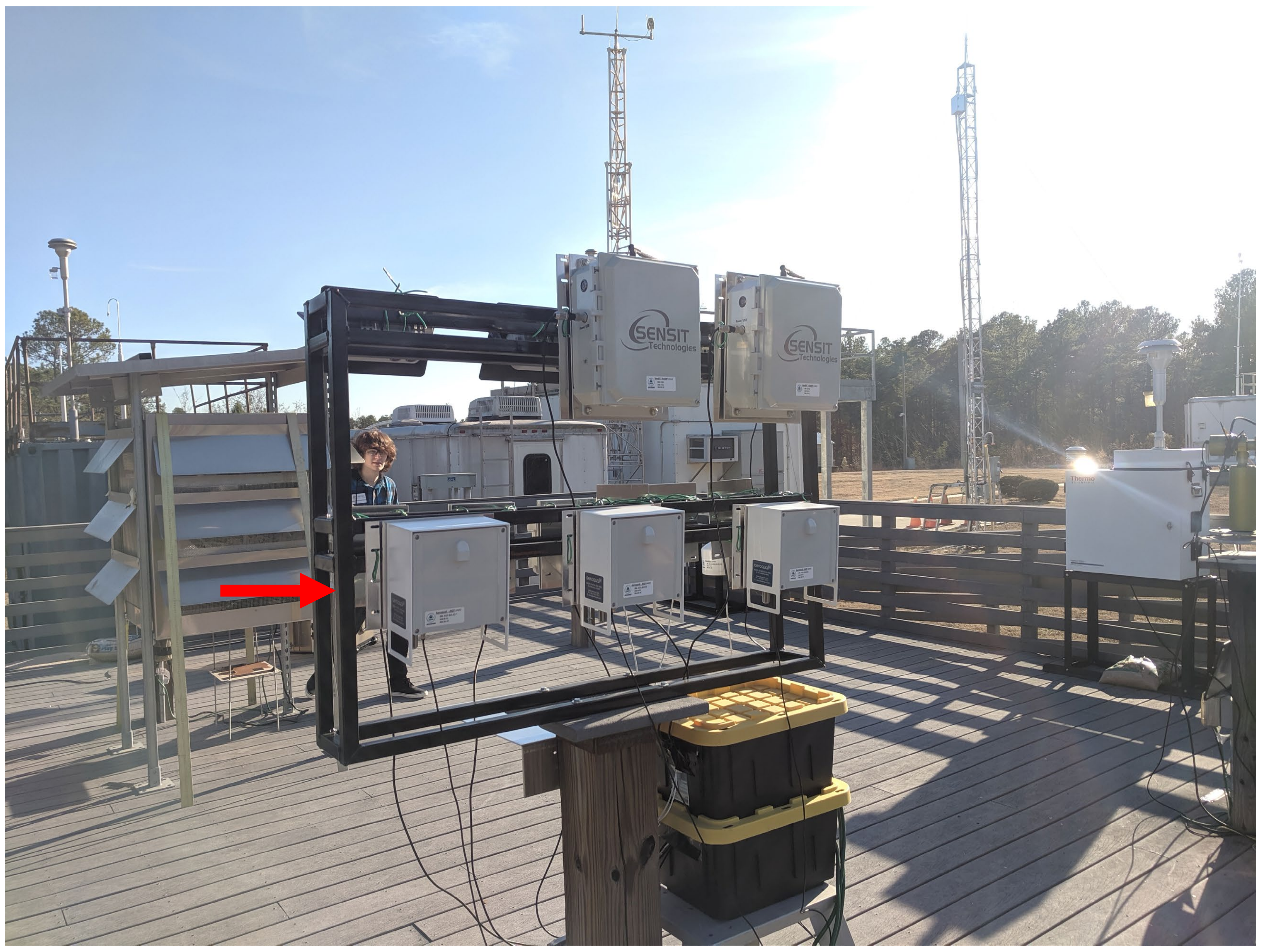

Figure 2: Observation deck at evaluation site. Approximate location of sensors indicated by the red arrow, the approximate location of T640x FEM (housed within sampling trailer) indicated by the black arrow. Sensors and FEM instrumentation are separated by approximately 15 meters.

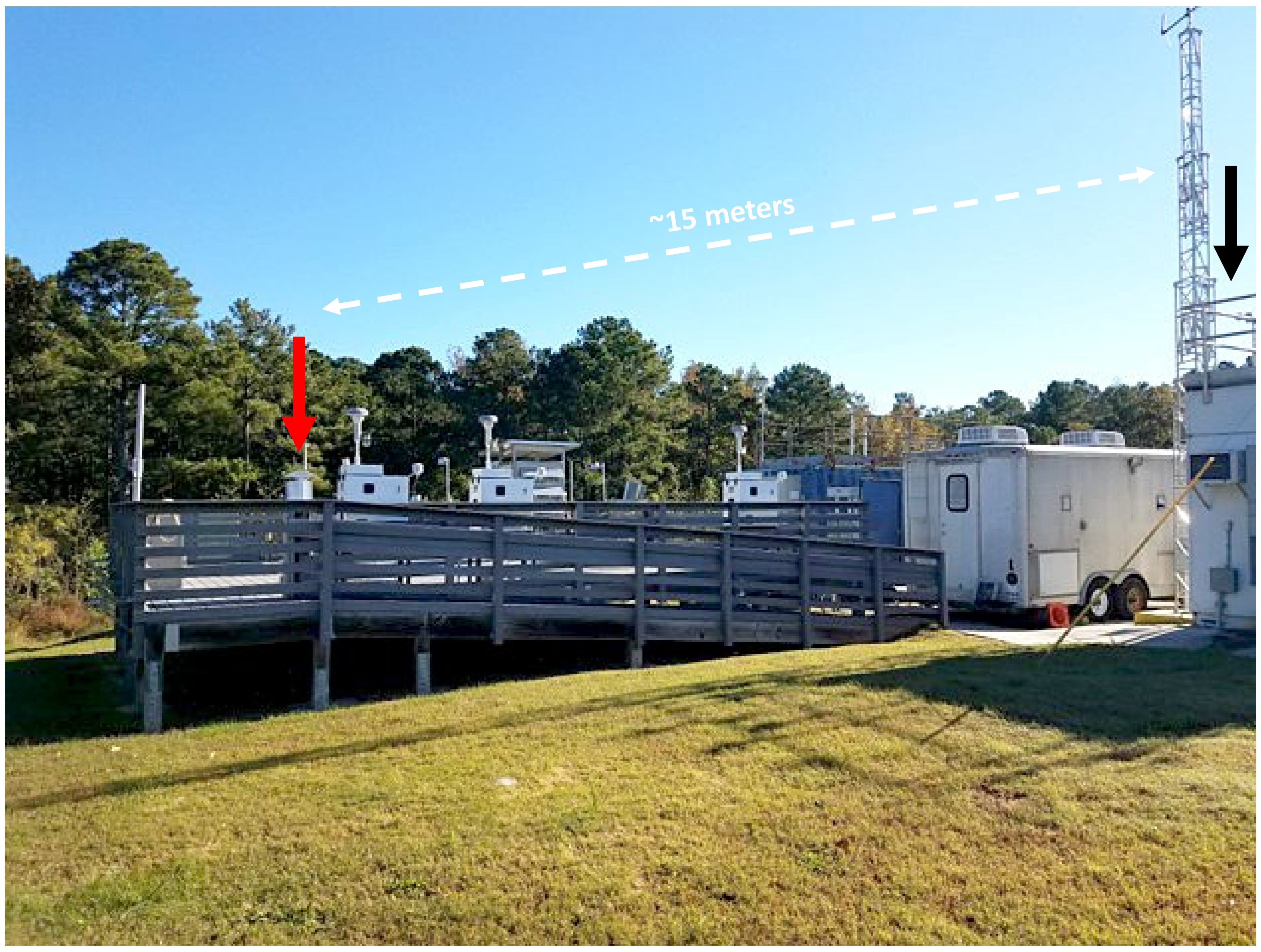

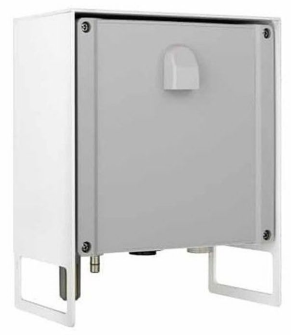
Image Attribution: Aeroqual Limited (Auckland, New Zealand)

Supplemental Information: Data Storage, Correction Approach, and Issues Encountered

Data Storage and Transmission Method

For this study, SIM cards were obtained from Aeroqual and installed in units with serials numbers beginning with BB. Units with serial numbers beginning with BA were connected to Wi-Fi via a mobile hotspot. Data from all units flowed to the Aeroqual Cloud. The 1-minute raw data was acquired weekly using the Aeroqual Cloud user interface (UI). The AQY possesses an internal data storage USB flash drive as a data backup, however access requires software proprietary to Aeroqual.

Data Correction Approach

This evaluation report reflects “out-of-the-box” performance of the AQY. The manufacturer provides a procedure by which local collocation (sensor operated along side an FRM/FEM) data can be collected, a gain (slope) and offset (intercept) determined, and parameters entered into the Aeroqual Cloud user interface to be applied to all subsequently collected data. This procedure and feature was **not** used prior to this evaluation. Prospective consumers may get different performance from this device if they utilize this feature.

After acquisition, the raw data was processed using the *sensortoolkit* python code library (v0.8.3b2). A continuous data set at the recorded sampling frequency was written to a .csv file. 1-hour averaged data sets were generated using a 75% completeness threshold and saved as separate .csv files. Outliers were **not** removed from data sets in order to assess “out-of-the-box” sensor performance.

The duration of the warm-up period required for sensor measurements to equilibrate was determined from field data to be 10 minutes. Warm up periods were considered to occur following any power outage to sensor units, either due to routine field visits or unscheduled site power outages. Data recorded during warm up periods has been removed from data sets.

Issues Encountered

<u>Pre-deployment observations</u>

- *Timestamp inaccuracies*: During the pre-deployment phase, it was observed that the AQY devices did not store the Real Time Clock’s (RTC) time. For the units to maintain accurate time, they needed to be connected to the internet after each shutdown event. Since data could be downloaded over Wi-Fi while the sensor pods remained operational, the units were connected to a mobile hotspot at the beginning of the evaluation.
- *Hotspot connectivity*: The process of connecting the AQYs to a hotspot required their Wi-Fi adapter’s operating mode to be changed from “Access Point” to “Client” and the hotspot’s service set identifier (SSID) and password needed to be provided to the AQY’s configuration. The repeatability and reproducibility of the processes to acquire internet time and subsequent data extraction were verified for instruments prior to deployment.

<u>Field observations and sensor data flags</u>

Aeroqual AQY units were deployed at the AIRS monitoring site on 8/1/2019. On 8/22/2019, a power outage was reported which extended into the following day at 11:52 AM. During this period, none of the AQY units were able to recorded data. A subsequent outage was reported for AQY unit AQY_01 on 8/25/2019 and lasted less than two hours. The field technician was not at the monitoring site when either outages were reported, indicating that the events were likely not caused by operator error.

The following table contains data flags describing events that were encountered during the testing period.

Start Time (UTC)	End Time (UTC)	Sensor Serial ID	Parameters Impacted	Flag
2019-08-22 18:26:00+00:00	2019-08-23 11:52:00+00:00	AQY_02	ALL	9.0-Data loss - Power connection error
2019-08-22 18:26:00+00:00	2019-08-23 11:52:00+00:00	AQY_03	ALL	9.0-Data loss - Power connection error
2019-08-22 18:26:00+00:00	2019-08-23 11:52:00+00:00	AQY_01	ALL	9.0-Data loss - Power connection error
2019-08-25 08:50:00+00:00	2019-08-25 10:17:00+00:00	AQY_01	ALL	9.0-Data loss - Power connection error

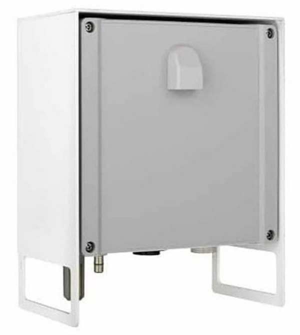

Image Attribution: Aeroqual Limited (Auckland, New Zealand)

Description of Data Flags

OAQPS manages data logged by reference monitors at the AIRS evaluation site using the Envidas data acquisition system software from DR DAS LTD[1]. Envidas contains over 100 data flags which are configurable by the monitoring agency and can be triggered based on instrument status. Appendix B Table 1 contains a description of Envidas data flags including the numeric code and status code name (a brief textual description, the table containing data flags below includes code names for events encountered by the reference monitor under the "Flag" column). Appendix B Table 1 also indicates whether reference data are invalidated for a given data flag. For this report, reference data were invalidated for periods where a data flag was logged and if the corresponding entry for the data flag in the "Data Status Invalidation" column in Appendix B Table 1 indicates data should be invalidated.

Data Flags Recorded During Testing

FRM/FEM Monitor	Timestamp (UTC)	Flag
Teledyne API T265 (Data acquired via local transfer from OAQPS)	2019-08-02 09:46:00+0000 to 2019-08-02 10:00:00+0000	Zero
	2019-08-02 10:01:00+0000 to 2019-08-02 10:15:00+0000	Span
	2019-08-02 10:16:00+0000 to 2019-08-02 10:30:00+0000	Spare
	2019-08-02 10:31:00+0000 to 2019-08-02 10:35:00+0000	Purge
	2019-08-03 09:46:00+0000 to 2019-08-03 10:00:00+0000	Zero
	2019-08-03 10:01:00+0000 to 2019-08-03 10:15:00+0000	Span
	2019-08-03 10:16:00+0000 to 2019-08-03 10:30:00+0000	Spare
	2019-08-03 10:31:00+0000 to 2019-08-03 10:35:00+0000	Purge
	2019-08-04 09:46:00+0000 to 2019-08-04 10:00:00+0000	Zero
	2019-08-04 10:01:00+0000 to 2019-08-04 10:15:00+0000	Span
	2019-08-04 10:16:00+0000 to 2019-08-04 10:30:00+0000	Spare
	2019-08-04 10:31:00+0000 to 2019-08-04 10:35:00+0000	Purge
	2019-08-05 09:46:00+0000 to 2019-08-05 10:00:00+0000	Zero
	2019-08-05 10:01:00+0000 to 2019-08-05 10:15:00+0000	Span
	2019-08-05 10:16:00+0000 to 2019-08-05 10:30:00+0000	Spare
	2019-08-05 10:31:00+0000 to 2019-08-05 10:35:00+0000	Purge
	2019-08-06 09:46:00+0000 to 2019-08-06 10:00:00+0000	Zero
	2019-08-06 10:01:00+0000 to 2019-08-06 10:15:00+0000	Span
	2019-08-06 10:16:00+0000 to 2019-08-06 10:30:00+0000	Spare
	2019-08-06 10:31:00+0000 to 2019-08-06 10:35:00+0000	Purge
	2019-08-07 09:46:00+0000 to 2019-08-07 10:00:00+0000	Zero
	2019-08-07 10:01:00+0000 to 2019-08-07 10:15:00+0000	Span
	2019-08-07 10:16:00+0000 to 2019-08-07 10:30:00+0000	Spare
	2019-08-07 10:31:00+0000 to 2019-08-07 10:35:00+0000	Purge
	2019-08-08 09:46:00+0000 to 2019-08-08 10:00:00+0000	Zero
	2019-08-08 10:01:00+0000 to 2019-08-08 10:15:00+0000	Span
	2019-08-08 10:16:00+0000 to 2019-08-08 10:30:00+0000	Spare
	2019-08-08 10:31:00+0000 to 2019-08-08 10:35:00+0000	Purge
	2019-08-08 14:02:00+0000	<Samp

[1]"Contact us" DR DAS LTD, 2022, https://dr-das.com/Home/Contact.html Accessed June 3 2022

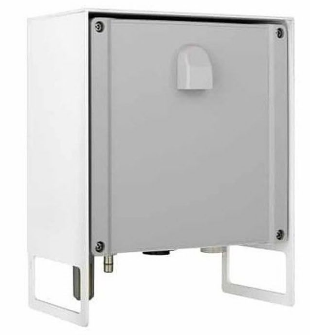

Image Attribution: Aeroqual Limited (Auckland, New Zealand)

Data Flags Recorded During Testing (Cont.)

FRM/FEM Monitor	Timestamp (UTC)	Flag
Teledyne API T265<br>(Data acquired via local transfer from OAQPS)	2019-08-09 09:46:00+0000 to 2019-08-09 10:00:00+0000	Zero
	2019-08-09 10:01:00+0000 to 2019-08-09 10:15:00+0000	Span
	2019-08-09 10:16:00+0000 to 2019-08-09 10:30:00+0000	Spare
	2019-08-09 10:31:00+0000 to 2019-08-09 10:35:00+0000	Purge
	2019-08-10 09:46:00+0000 to 2019-08-10 10:00:00+0000	Zero
	2019-08-10 10:01:00+0000 to 2019-08-10 10:15:00+0000	Span
	2019-08-10 10:16:00+0000 to 2019-08-10 10:30:00+0000	Spare
	2019-08-10 10:31:00+0000 to 2019-08-10 10:35:00+0000	Purge
	2019-08-11 09:46:00+0000 to 2019-08-11 10:00:00+0000	Zero
	2019-08-11 10:01:00+0000 to 2019-08-11 10:15:00+0000	Span
	2019-08-11 10:16:00+0000 to 2019-08-11 10:30:00+0000	Spare
	2019-08-11 10:31:00+0000 to 2019-08-11 10:35:00+0000	Purge
	2019-08-12 09:46:00+0000 to 2019-08-12 10:00:00+0000	Zero
	2019-08-12 10:01:00+0000 to 2019-08-12 10:15:00+0000	Span
	2019-08-12 10:16:00+0000 to 2019-08-12 10:30:00+0000	Spare
	2019-08-12 10:31:00+0000 to 2019-08-12 10:35:00+0000	Purge
	2019-08-12 17:48:00+0000 to 2019-08-12 18:01:00+0000	Down
	2019-08-12 18:08:00+0000	<Samp
	2019-08-12 18:09:00+0000 to 2019-08-12 19:59:00+0000	Down
	2019-08-13 09:46:00+0000 to 2019-08-13 10:00:00+0000	Zero
	2019-08-13 10:01:00+0000 to 2019-08-13 10:15:00+0000	Span
	2019-08-13 10:16:00+0000 to 2019-08-13 10:30:00+0000	Spare
	2019-08-13 10:31:00+0000 to 2019-08-13 10:35:00+0000	Purge
	2019-08-13 14:58:00+0000	<Samp
	2019-08-13 15:00:00+0000 to 2019-08-13 15:59:00+0000	Down
	2019-08-13 18:49:00+0000	<Samp
	2019-08-13 18:50:00+0000 to 2019-08-13 20:18:00+0000	Down
	2019-08-14 09:46:00+0000 to 2019-08-14 10:00:00+0000	Zero
	2019-08-14 10:01:00+0000 to 2019-08-14 10:15:00+0000	Span
	2019-08-14 10:16:00+0000 to 2019-08-14 10:30:00+0000	Spare
	2019-08-14 10:31:00+0000 to 2019-08-14 10:35:00+0000	Purge
	2019-08-15 09:46:00+0000 to 2019-08-15 10:00:00+0000	Zero
	2019-08-15 10:01:00+0000 to 2019-08-15 10:15:00+0000	Span
	2019-08-15 10:16:00+0000 to 2019-08-15 10:30:00+0000	Spare
	2019-08-15 10:31:00+0000 to 2019-08-15 10:35:00+0000	Purge
	2019-08-15 18:30:00+0000 to 2019-08-15 18:55:00+0000	<Samp
	2019-08-15 18:31:00+0000 to 2019-08-15 18:54:00+0000	Down
	2019-08-16 09:46:00+0000 to 2019-08-16 10:00:00+0000	Zero
	2019-08-16 10:01:00+0000 to 2019-08-16 10:15:00+0000	Span
	2019-08-16 10:16:00+0000 to 2019-08-16 10:30:00+0000	Spare
	2019-08-16 10:31:00+0000 to 2019-08-16 10:35:00+0000	Purge
	2019-08-17 09:46:00+0000 to 2019-08-17 10:00:00+0000	Zero
	2019-08-17 10:01:00+0000 to 2019-08-17 10:15:00+0000	Span
	2019-08-17 10:16:00+0000 to 2019-08-17 10:30:00+0000	Spare
	2019-08-17 10:31:00+0000 to 2019-08-17 10:35:00+0000	Purge
	2019-08-18 09:46:00+0000 to 2019-08-18 10:00:00+0000	Zero

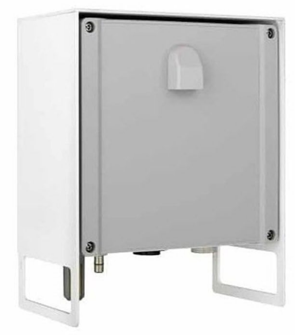

Image Attribution: Aeroqual Limited (Auckland, New Zealand)

Supplemental Information: Description of FRM/FEM QC Checks and Data Flags

Data Flags Recorded During Testing (Cont.)

FRM/FEM Monitor	Timestamp (UTC)	Flag
Teledyne API T265 (Data acquired via local transfer from OAQPS)	2019-08-18 10:01:00+0000 to 2019-08-18 10:15:00+0000	Span
	2019-08-18 10:16:00+0000 to 2019-08-18 10:30:00+0000	Spare
	2019-08-18 10:31:00+0000 to 2019-08-18 10:35:00+0000	Purge
	2019-08-19 09:46:00+0000 to 2019-08-19 10:00:00+0000	Zero
	2019-08-19 10:01:00+0000 to 2019-08-19 10:15:00+0000	Span
	2019-08-19 10:16:00+0000 to 2019-08-19 10:30:00+0000	Spare
	2019-08-19 10:31:00+0000 to 2019-08-19 10:35:00+0000	Purge
	2019-08-20 09:46:00+0000 to 2019-08-20 10:00:00+0000	Zero
	2019-08-20 10:01:00+0000 to 2019-08-20 10:15:00+0000	Span
	2019-08-20 10:16:00+0000 to 2019-08-20 10:30:00+0000	Spare
	2019-08-20 10:31:00+0000 to 2019-08-20 10:35:00+0000	Purge
	2019-08-21 09:46:00+0000 to 2019-08-21 10:00:00+0000	Zero
	2019-08-21 10:01:00+0000 to 2019-08-21 10:15:00+0000	Span
	2019-08-21 10:16:00+0000 to 2019-08-21 10:30:00+0000	Spare
	2019-08-21 10:31:00+0000 to 2019-08-21 10:35:00+0000	Purge
	2019-08-22 05:00:00+0000 to 2019-08-23 04:27:00+0000	InVld
	2019-08-23 04:28:00+0000 to 2019-08-23 07:12:00+0000	NoData
	2019-08-23 07:13:00+0000	<Samp
	2019-08-23 09:46:00+0000 to 2019-08-23 10:00:00+0000	Zero
	2019-08-23 10:01:00+0000 to 2019-08-23 10:15:00+0000	Span
	2019-08-23 10:16:00+0000 to 2019-08-23 10:30:00+0000	Spare
	2019-08-23 10:31:00+0000 to 2019-08-23 10:35:00+0000	Purge
	2019-08-23 15:11:00+0000 to 2019-08-23 15:17:00+0000	Down
	2019-08-23 15:36:00+0000 to 2019-08-23 15:40:00+0000	<Samp
	2019-08-23 15:37:00+0000 to 2019-08-23 15:38:00+0000	NoData
	2019-08-23 15:39:00+0000	Down
	2019-08-23 15:49:00+0000 to 2019-08-23 16:12:00+0000	<Samp
	2019-08-23 15:50:00+0000 to 2019-08-23 16:11:00+0000	Down
	2019-08-23 20:06:00+0000 to 2019-08-23 20:27:00+0000	Down
	2019-08-23 20:28:00+0000	<Samp
	2019-08-23 20:42:00+0000 to 2019-08-23 20:54:00+0000	<Samp
	2019-08-23 20:43:00+0000 to 2019-08-23 20:53:00+0000	Down
	2019-08-24 09:46:00+0000 to 2019-08-24 10:00:00+0000	Zero
	2019-08-24 10:01:00+0000 to 2019-08-24 10:15:00+0000	Span
	2019-08-24 10:16:00+0000 to 2019-08-24 10:30:00+0000	Spare
	2019-08-24 10:31:00+0000 to 2019-08-24 10:35:00+0000	Purge
	2019-08-25 09:46:00+0000 to 2019-08-25 10:00:00+0000	Zero
	2019-08-25 10:01:00+0000 to 2019-08-25 10:15:00+0000	Span
	2019-08-25 10:16:00+0000 to 2019-08-25 10:30:00+0000	Spare
	2019-08-25 10:31:00+0000 to 2019-08-25 10:35:00+0000	Purge
	2019-08-26 09:46:00+0000 to 2019-08-26 10:00:00+0000	Zero
	2019-08-26 10:01:00+0000 to 2019-08-26 10:15:00+0000	Span
	2019-08-26 10:16:00+0000 to 2019-08-26 10:30:00+0000	Spare
	2019-08-26 10:31:00+0000 to 2019-08-26 10:35:00+0000	Purge
	2019-08-26 20:16:00+0000 to 2019-08-26 21:17:00+0000	Down

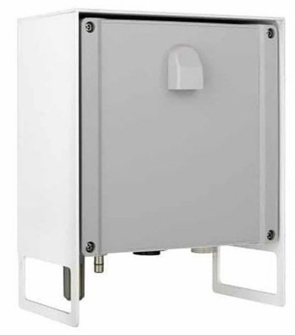

Image Attribution: Aeroqual Limited (Auckland, New Zealand)

Supplemental Information: Description of FRM/FEM QC Checks and Data Flags

Data Flags Recorded During Testing (Cont.)

FRM/FEM Monitor	Timestamp (UTC)	Flag
Teledyne API T265 (Data acquired via local transfer from OAQPS)	2019-08-26 21:18:00+0000	<Samp
	2019-08-27 09:46:00+0000 to 2019-08-27 10:00:00+0000	Zero
	2019-08-27 10:01:00+0000 to 2019-08-27 10:15:00+0000	Span
	2019-08-27 10:16:00+0000 to 2019-08-27 10:30:00+0000	Spare
	2019-08-27 10:31:00+0000 to 2019-08-27 10:35:00+0000	Purge
	2019-08-27 11:46:00+0000 to 2019-08-27 12:00:00+0000	Zero
	2019-08-27 12:01:00+0000 to 2019-08-27 12:15:00+0000	Span
	2019-08-27 12:16:00+0000 to 2019-08-27 12:30:00+0000	Spare
	2019-08-27 12:31:00+0000 to 2019-08-27 12:35:00+0000	Purge
	2019-08-28 09:46:00+0000 to 2019-08-28 10:00:00+0000	Zero
	2019-08-28 10:01:00+0000 to 2019-08-28 10:15:00+0000	Span
	2019-08-02 09:46:00+0000 to 2019-08-02 10:00:00+0000	Zero
	2019-08-02 10:01:00+0000 to 2019-08-02 10:15:00+0000	Span
	2019-08-02 10:16:00+0000 to 2019-08-02 10:30:00+0000	Spare
	2019-08-02 10:31:00+0000 to 2019-08-02 10:35:00+0000	Purge
	2019-08-03 09:46:00+0000 to 2019-08-03 10:00:00+0000	Zero
	2019-08-03 10:01:00+0000 to 2019-08-03 10:15:00+0000	Span
	2019-08-03 10:16:00+0000 to 2019-08-03 10:30:00+0000	Spare
	2019-08-03 10:31:00+0000 to 2019-08-03 10:35:00+0000	Purge
	2019-08-04 09:46:00+0000 to 2019-08-04 10:00:00+0000	Zero
	2019-08-04 10:01:00+0000 to 2019-08-04 10:15:00+0000	Span
	2019-08-04 10:16:00+0000 to 2019-08-04 10:30:00+0000	Spare
	2019-08-04 10:31:00+0000 to 2019-08-04 10:35:00+0000	Purge
	2019-08-05 09:46:00+0000 to 2019-08-05 10:00:00+0000	Zero
	2019-08-05 10:01:00+0000 to 2019-08-05 10:15:00+0000	Span
	2019-08-05 10:16:00+0000 to 2019-08-05 10:30:00+0000	Spare
	2019-08-05 10:31:00+0000 to 2019-08-05 10:35:00+0000	Purge
	2019-08-06 09:46:00+0000 to 2019-08-06 10:00:00+0000	Zero
	2019-08-06 10:01:00+0000 to 2019-08-06 10:15:00+0000	Span
	2019-08-06 10:16:00+0000 to 2019-08-06 10:30:00+0000	Spare
	2019-08-06 10:31:00+0000 to 2019-08-06 10:35:00+0000	Purge
	2019-08-07 09:46:00+0000 to 2019-08-07 10:00:00+0000	Zero
	2019-08-07 10:01:00+0000 to 2019-08-07 10:15:00+0000	Span
	2019-08-07 10:16:00+0000 to 2019-08-07 10:30:00+0000	Spare
	2019-08-07 10:31:00+0000 to 2019-08-07 10:35:00+0000	Purge
	2019-08-08 09:46:00+0000 to 2019-08-08 10:00:00+0000	Zero
	2019-08-08 10:01:00+0000 to 2019-08-08 10:15:00+0000	Span
	2019-08-08 10:16:00+0000 to 2019-08-08 10:30:00+0000	Spare
	2019-08-08 10:31:00+0000 to 2019-08-08 10:35:00+0000	Purge
	2019-08-08 14:02:00+0000	<Samp

Meteorological Instrument	Timestamp (UTC)	Flag
RM Young 41382 VC Temperature and Relative Humidity Monitor (Data acquired via local transfer from OAQPS)	2019-08-08 14:02:00+0000	<Samp
	2019-08-13 14:58:00+0000	<Samp
	2019-08-23 04:28:00+0000 to 2019-08-23 07:12:00+0000	NoData
	2019-08-23 07:13:00+0000	<Samp
	2019-08-26 21:18:00+0000	<Samp
	2019-08-28 15:07:00+0000	<Samp

Testing Report - O_3 Base Testing

SENSIT RAMP

This report reflects out-of-the-box performance

Initial Base Testing - RTP, NC

U.S. Environmental Protection Agency
Office of Research and Development
PI: Clements.Andrea@epa.gov
919-541-1363
October 2019—November 2019

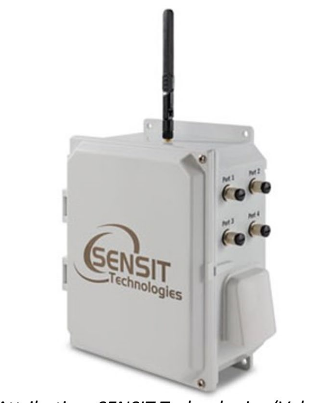

Image Attribution: SENSIT Technologies (Valparaiso, IN)

Deployment Details

Testing Organization and Site Information	
Testing organization (Name, Organization type, Contact website)	U.S. Environmental Protection Agency - Office of Research and Development<br>Federal Government<br>Air Sensor Toolbox | U.S. EPA Website
Testing location (City, State, Latitude and Longitude)	Ambient Monitoring Innovative Research Station (AIRS)<br>RTP, NC<br>35.88951, -78.874572
AQS site ID	37 – 063 – 0099
Sampling timeframe (MM-DD-YY)	10-18-19 to 11-17-19
Sensor data source	Onboard MicroSD card
Reference data source	OAQPS file transfer

Sensor Information			
Manufacturer, model	SENSIT RAMP		
Device firmware version	RAM01: 190313_AQ_v9.30,<br>RAM02: 190308_AQ_v9.30,<br>RAM04: 190730_AQ_v9.34		
Sampling time interval	15-seconds		
Sensor serial numbers	RAM_01	RAM_02	RAM_04
Issues encountered during deployment?	☐	Issues with deployment	

FRM/FEM Information	
Manufacturer, model, designation	Teledyne API T265 FEM
Sampling time interval	1-hour averaging
Date of calibration	As required by 40 CFR Part 58 and the Burdens Creek QAPP maintained by OAQPS
Date of one-point QC check	Every two weeks as required by 40 CFR Part 58 Appendix A 3.1.1
Description, date(s) of maintenance activities	N/A

Time Series Plot: 1-hour averaged O_3

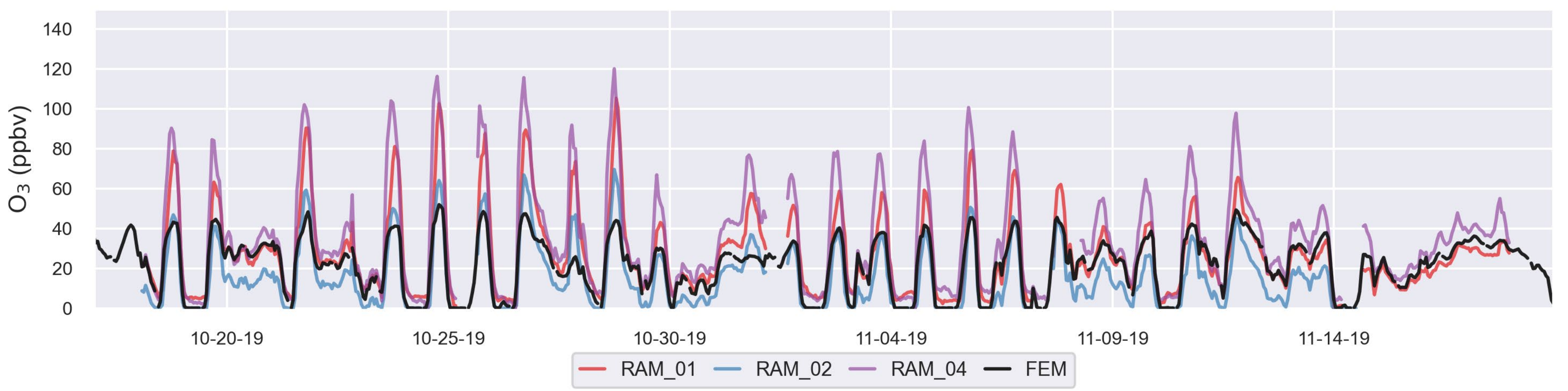

Range and average of FRM/FEM concentrations over duration of base test (ppbv)	[1-hr] 0.0-51.9, avg: 21.3,<br>[Rolling 8-hr] 0.1-44.9, avg: 23.8
Number of 1-hr periods in FRM/FEM monitor measurements with a goal concentration ≥ 60 ppbv	0

Scatter Plot: Comparison to FRM/FEM

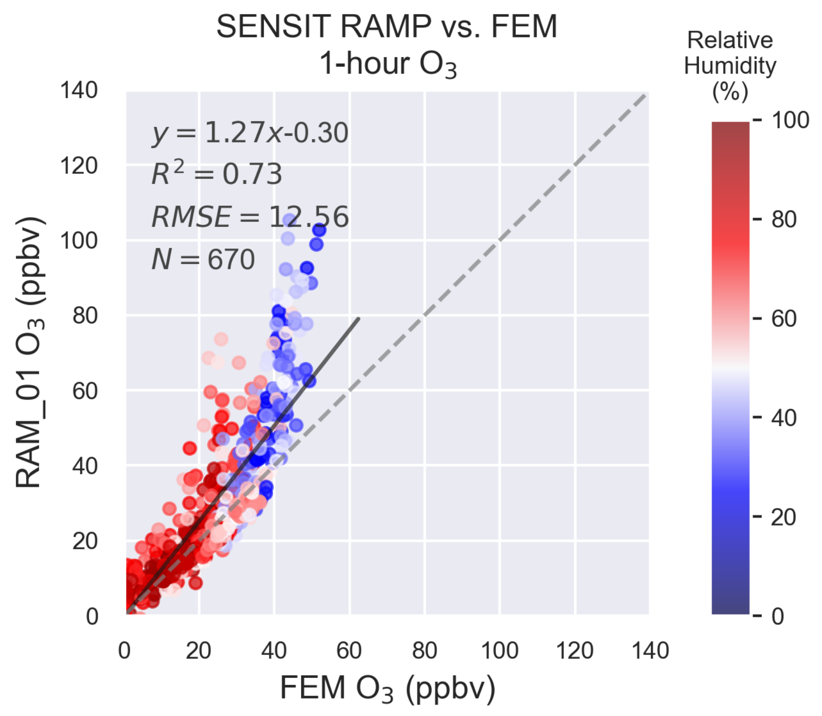

Performance Metrics★

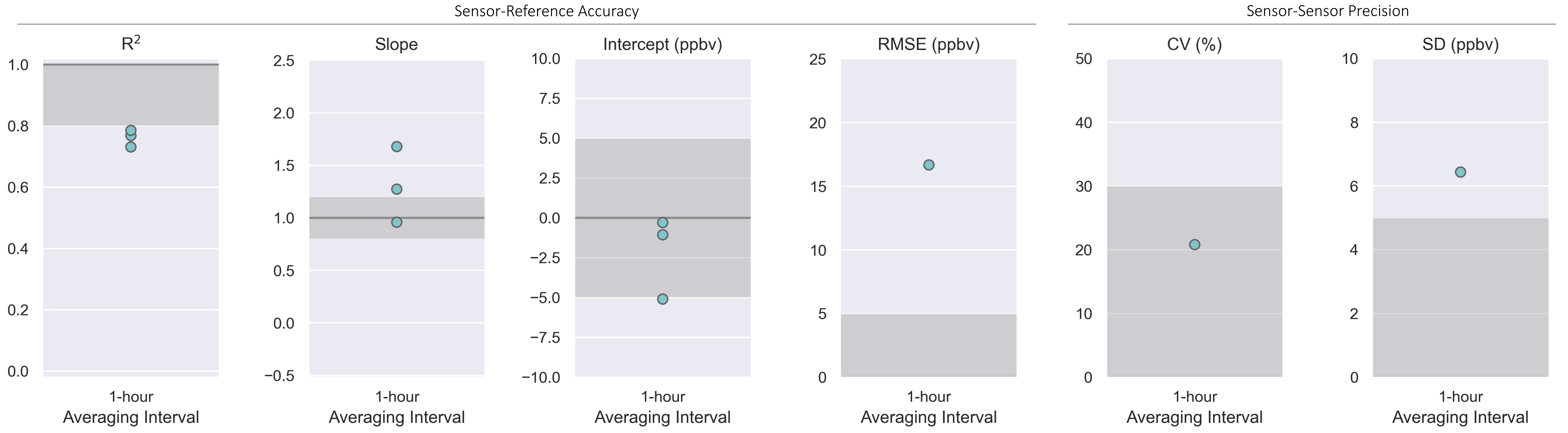

Meteorological Conditions During Deployment

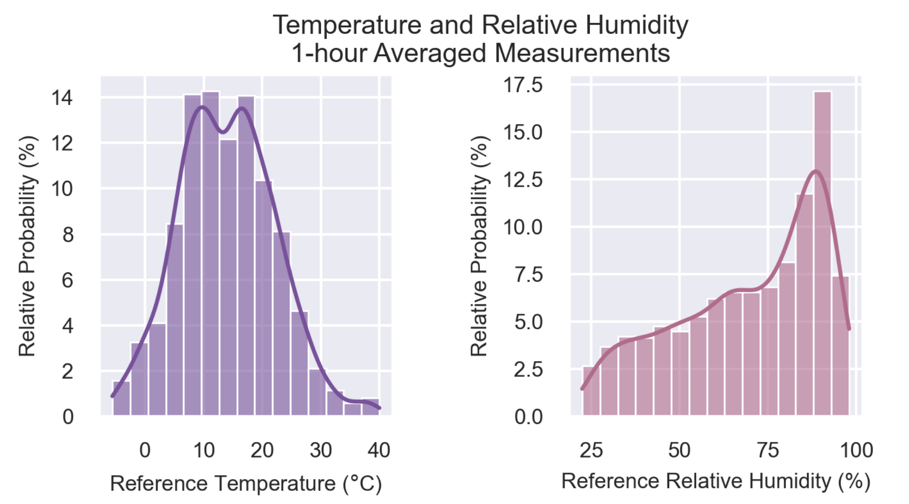

Number of 1-hr periods outside sensor manufacturer-listed temperature operational range (-20 to 50 °C)	0
Number of 1-hr periods outside sensor manufacturer-listed relative humidity operational range (no operational range specified)	-

Meteorological Influence

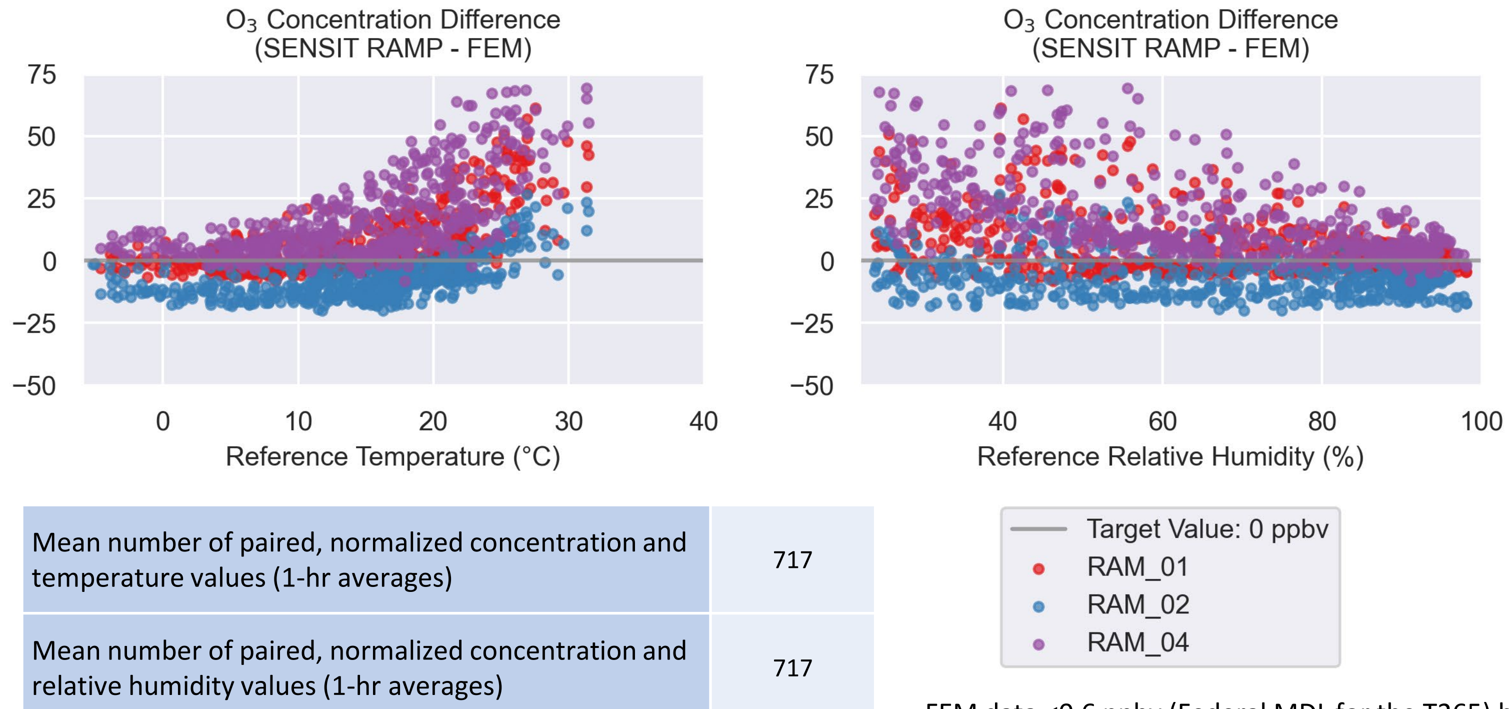

Mean number of paired, normalized concentration and temperature values (1-hr averages)	717
Mean number of paired, normalized concentration and relative humidity values (1-hr averages)	717

FEM data <0.6 ppbv (Federal MDL for the T265) has been remove (Meteorological Influence section only)

★For evaluations with greater than three sensors, grouping individual sensor metrics into boxplots is recommended for displaying results. Note that this recommendation does not apply to metrics computed as a single value for all sensors over the whole evaluation group, such as RMSE, NRMSE, CV, and standard deviation.

Testing Report - O_3 Base Testing

SENSIT RAMP

This report reflects out-of-the-box performance

Initial Base Testing - RTP, NC

U.S. Environmental Protection Agency
Office of Research and Development
PI: Clements.Andrea@epa.gov
919-541-1363

October 2019—November 2019

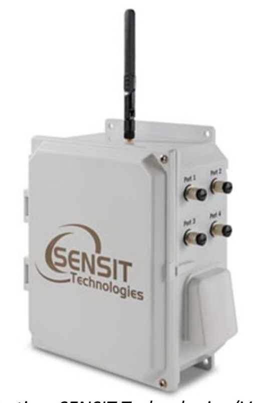

Image Attribution: SENSIT Technologies (Valparaiso, IN)

Tabular Statistics

Sensor-FRM/FEM Correlation

	Bias and Linearity			Data Quality	
	R^2	Slope	Intercept (ppbv)	Uptime (%)	Number of paired sensor and reference concentration values*
	1-Hour ○○○	1-Hour ●○○	1-Hour ●●○	1-Hour ●●●	1-Hour
Metric Target Range	≥ 0.80	1.0 ± 0.20	-5 ≤ b ≤ 5	75%*	-
Sensor RAM_01	0.73	1.27	-0.30	95	670
Sensor RAM_02	0.77	0.96	-5.10	96	600
Sensor RAM_04	0.79	1.68	-1.06	94	662
Mean	0.76	1.30	-2.15	95.05	644.00

	Error
	RMSE (ppbv)
	1-Hour ☆
Metric Target Range	≤ 5.0
Deployment Value	16.7

Device-specific metrics (computed for each sensor in evaluation)
○○○ Metric value for none of devices tested falls within the target range
●○○ Metric value for one of devices tested falls within the target range
●●○ Metric value for two of devices tested falls within the target range
●●● Metric value for three of devices tested falls within the target range

Single-valued metrics (computed via entire evaluation dataset)
☆ Indicates that the metric value is not within the target range
★ Indicates that the metric value is within the target range

Sensor-Sensor Precision

	Precision (between collocated sensors)		Data Quality
	CV (%)	SD (ppbv)	Number of paired sensor and reference concentration values*
	1-Hour ★	1-Hour ☆	1-Hour
Metric Target Range	≤ 30.0	≤ 5.0	-
Deployment Value	20.8	6.4	683

*This value is only a recommendation for ensuring data quality and is not included in the list of target values discussed in Section 4 of the Performance Testing Protocols, Metrics, and Target Values for Ozone Air Sensors document.

U.S. Environmental Protection Agency
Office of Research and Development
PI: Clements.Andrea@epa.gov
919-541-1363

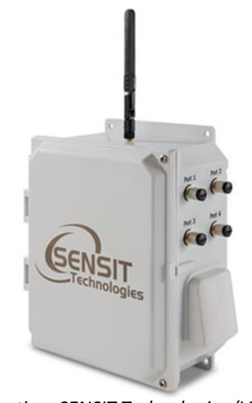

Image Attribution: SENSIT Technologies (Valparaiso, IN)

Sensor-FRM/FEM Scatter Plots

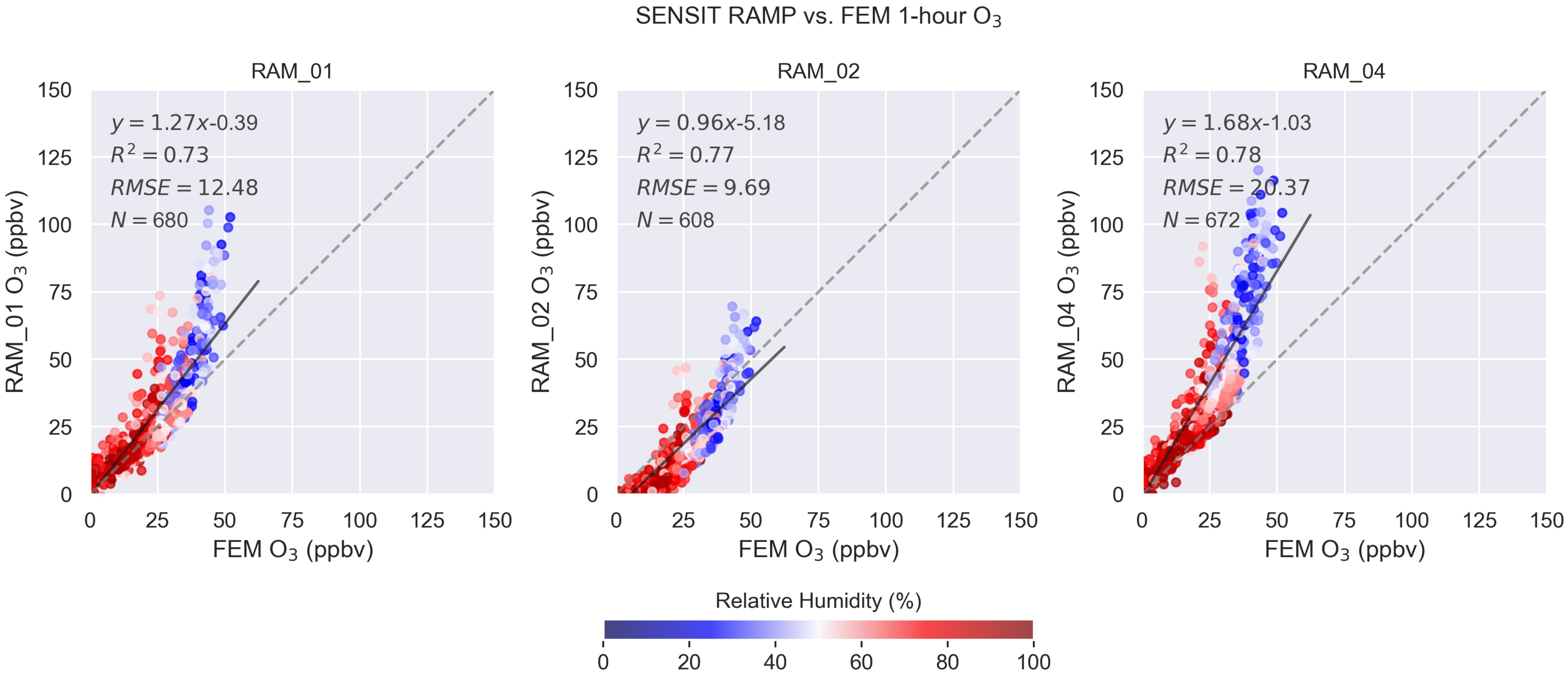

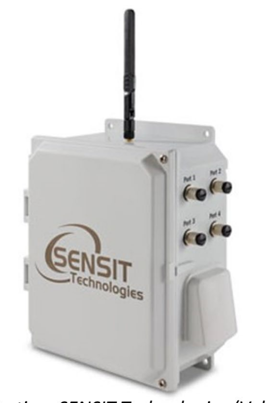

Image Attribution: SENSIT Technologies (Valparaiso, IN)

Supplemental Information

Abbreviations used in Supplemental Information

FRM	Federal Reference Method
FEM	Federal Equivalent Method
SOP	Standard Operating Procedure
QAPP	Quality Assurance Project Plan
QC	Quality Control

Supplemental Documentation	Attached	Description & URL or file path to documentation
Field observations and sensor data flags	☑	See NC-RAM-Page 6 of this testing report
Maintenance logs	☐	No logs recorded during testing
Standard operating procedure(s)	☐	U.S. EPA Office Of Research and Development SOP available upon request
Photos of equipment setup and testing	☑	See NC-RAM-Page 5 of this testing report
Product specifications sheet(s)	☑	See Appendix C, “Spec_Sheet_SENSIT_RAMP.pdf”
Product manual(s)	☑	See Appendix C, “Manual_SENSIT_RAMP.pdf”
Data storage and transmission method	☑	See NC-RAM-Page 6 of this testing report
Data correction approach	☑	See NC-RAM-Page 6 of this testing report
Issues encountered	☑	See NC-RAM-Page 6 of this testing report
Data analysis/correction scripts and version	☑	Averaging and processing of data, calculation of performance metrics, and generation of figures and other supplementary material for analysis were obtained using Python 3.9.7 with the packages sensortoolkit v0.8.3b2, pandas 1.3.5, NumPy 1.21.2, Matplotlib 3.5.0, statsmodels 0.13.0, and seaborn 0.11.2. All packages are available from the Python Package Index (PyPI) at https://pypi.org. The integrated development environment (IDE) Spyder 5.1.5 was used for scripting and data visualization. Version control for the Python base, packages, and IDE were all managed by conda 4.11.0.
Air Monitoring Station QAPP	☐	U.S. EPA Office Of Research and Development QAPP available upon request
Summary of FRM/FEM monitor QC checks	☑	See NC-RAM-Pages 7, 8, 9, 10, 11 of this testing report
Manufacturer website for FRM/FEM monitor	☑	Teledyne API: T265 Product website
FRM/FEM monitor manual	☑	See Appendix B, “Spec_Sheet_TeledyneAPI_T265.pdf”
FRM/FEM monitor specifications sheet(s)	☑	See Appendix B, “Manual_TeledyneAPI_T265.pdf”
Other documents	☐	

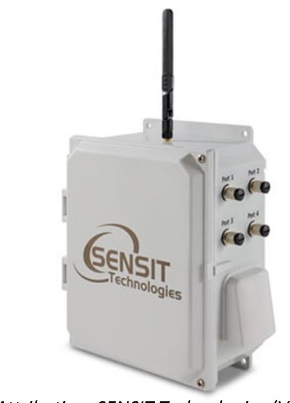

Image Attribution: SENSIT Technologies (Valparaiso, IN)

Supplemental Information: Photos of Testing Site and Equipment Setup

Site Description:

The Burdens Creek Ambient Monitoring Innovation Research Station (AIRS) site is located on the U.S. EPA, RTP campus and is situated between Alexander Drive and Route 147. The site is intended to represent a neighborhood-scale site as defined in *40 CFR Part 58, Appendix D*. U.S. EPA's Office of Air Quality Planning and Standards (OAQPS) operates reference grade instruments in a free-standing shelter situated directly adjacent to the sensor testing platform.

Figure 1: SENSIT RAMP sensors (indicated by red arrow) attached to mounting assembly at evaluation site.

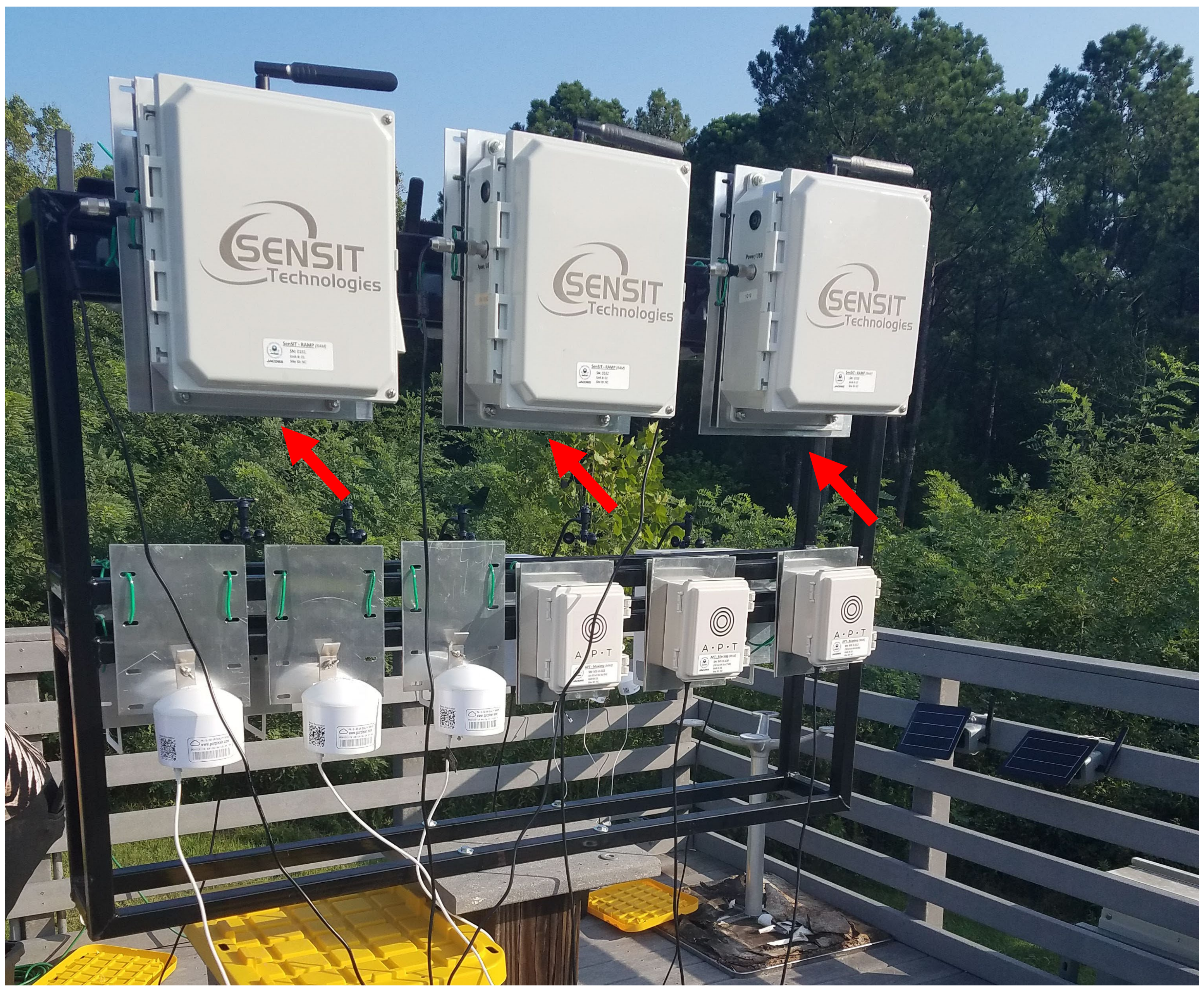

Figure 2: Observation deck at evaluation site. Approximate location of sensors indicated by the red arrow, the approximate location of T640x FEM (housed within sampling trailer) indicated by the black arrow. Sensors and FEM instrumentation are separated by approximately 15 meters.

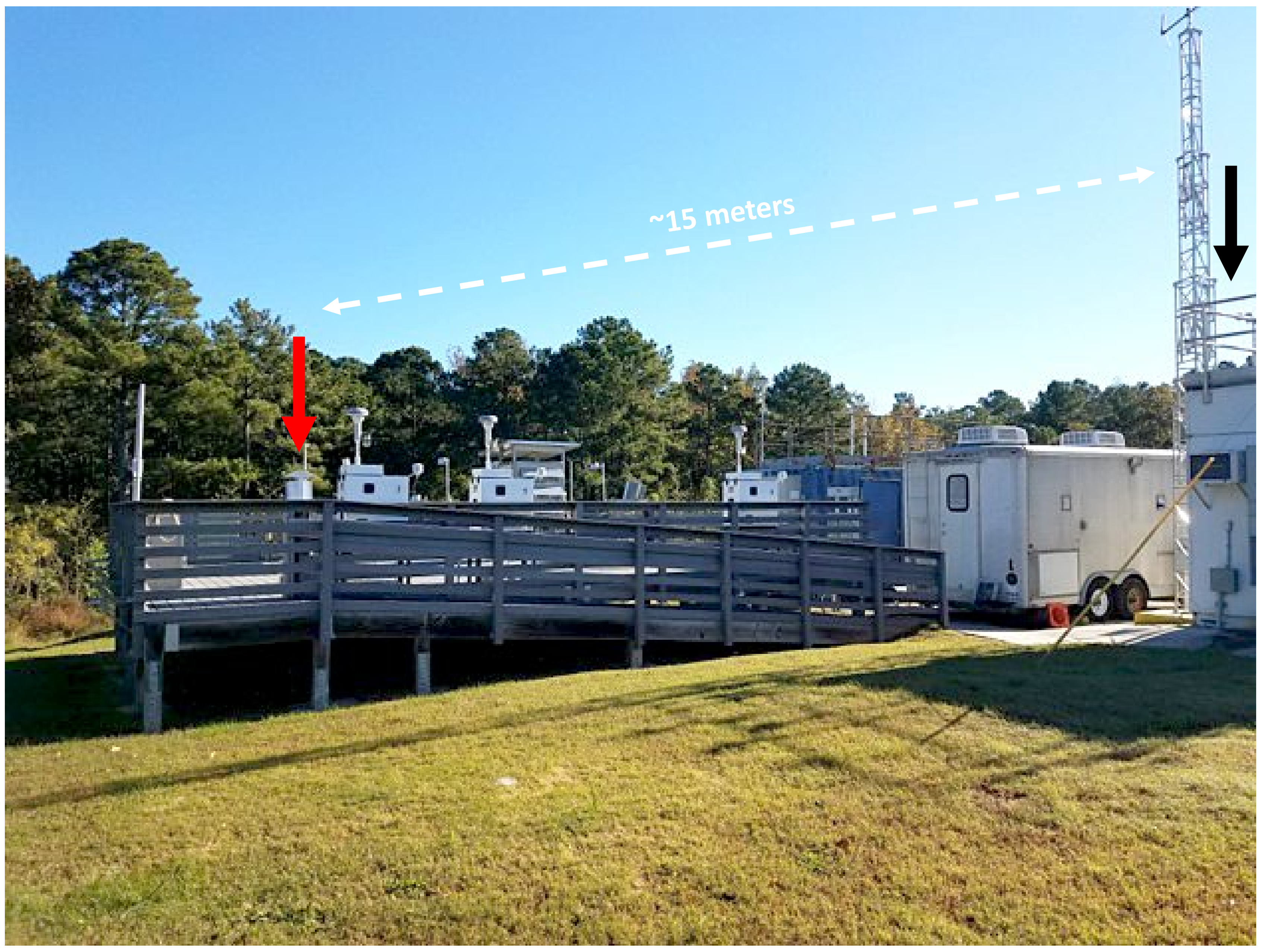

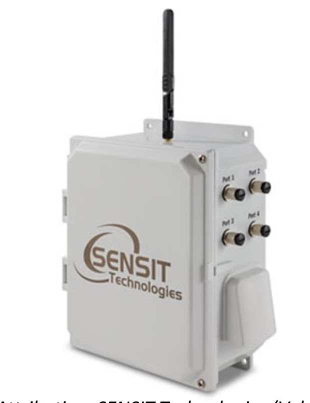

Image Attribution: SENSIT Technologies (Valparaiso, IN)

Supplemental Information: Data Storage, Correction Approach, and Issues Encountered

Data Storage and Transmission Method

The SENSIT RAMP was configured to record data at a 15-second sampling interval. Data are stored as daily text files (.txt format) on an onboard MicroSD card. Data files were obtained weekly via SD cards. Each field site operator was provided two labeled MicroSD cards for sensor units that they used to swap out each week. Data from the collected card was then read and processed off-site.

Data Correction Approach

SENSIT RAMP units were pre-configured by the manufacturer with a linear correction (i.e., concentration gain and offset). These presets (table below) reflect out-of-the box performance and were not modified by EPA prior or during testing.

Sensor ID	Preset Gain	Preset Offset
RAM_01	1.1	5.0
RAM_02	0.9	-5.0
RAM_04	1.1	2.0

After acquisition, the raw data was processed using the *sensortoolkit* python code library (v0.8.3b2). A continuous data set at the recorded sampling frequency was written to a .csv file. 1-hour averaged data sets were generated using a 75% completeness threshold and saved as separate .csv files. Outliers were **not** removed from data sets in order to assess "out-of-the-box" sensor performance.

The duration of the warm-up period required for sensor measurements to equilibrate was determined during bench-top testing (additional detail in pre-deployment observations) to be approximately 2 hours. Data recorded during warm up periods has been removed from data sets.

Issues Encountered

<u>Pre-deployment observations</u>

- *Changing logging interval*: SENSIT RAMP units were received without documentation or manuals. After communicating the need to change default settings (logging interval and time zone) with the manufacturer, a draft user's manual and a USB cable were supplied. With the use of this USB cable, instrument settings could be changed, and real-time data could be logged using a serial communication software (CoolTerm, v.1.5.0). Because the sensor did not record data at the top of every minute, the RAMP was configured to record data at 15-second intervals so that the data could be averaged more closely to complete minutes.
- *Gas Sensor Warmup*: Prior to deployment, RAMP units were collocated in a bench-top evaluation to verify operational status and determine the extent of data invalidity (i.e., determine equilibration period) after an initial start-up event. The recorded response for parameters measured by the RAMP suggests that the gas sensors (CO, NO, NO_2, O_3) required approximately a 2-hour equilibration period, while the remaining sensors (temperature, relative humidity, particulate matter) did not require any equilibration period.

<u>Field observations and sensor data flags</u>

The following table contains data flags describing events that were encountered during the testing period. Unit RAM_02 was retrieved slightly prior to the end of the testing period. RAM_02 was sent to the La Casa, CO monitoring site to replace a broken RAMP unit.

Start Time (UTC)	End Time (UTC)	Sensor Serial ID	Parameters Impacted	Flag
2019-11-14 13:01:00+00:00	2019-11-14 13:01:00+00:00	RAM_02	ALL	0.0-Sensor retrieval

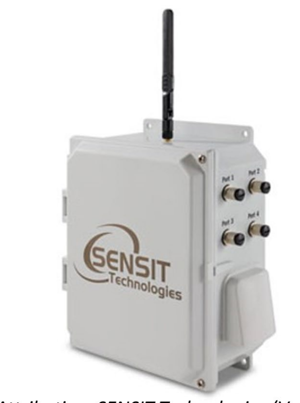
Image Attribution: SENSIT Technologies (Valparaiso, IN)

Description of Data Flags

OAQPS manages data logged by reference monitors at the AIRS evaluation site using the Envidas data acquisition system software from DR DAS LTD[1]. Envidas contains over 100 data flags which are configurable by the monitoring agency and can be triggered based on instrument status. Appendix B Table 1 contains a description of Envidas data flags including the numeric code and status code name (a brief textual description, the table containing data flags below includes code names for events encountered by the reference monitor under the "Flag" column). Appendix B Table 1 also indicates whether reference data are invalidated for a given data flag. For this report, reference data were invalidated for periods where a data flag was logged and if the corresponding entry for the data flag in the "Data Status Invalidation" column in Appendix B Table 1 indicates data should be invalidated.

Data Flags Recorded During Testing

FRM/FEM Monitor	Timestamp (UTC)	Flag
Teledyne API T265 (Data acquired via local transfer from OAQPS)	2019-10-18 09:46:00+0000 to 2019-10-18 10:00:00+0000	Zero
	2019-10-18 10:01:00+0000 to 2019-10-18 10:15:00+0000	Span
	2019-10-18 10:16:00+0000 to 2019-10-18 10:30:00+0000	Spare
	2019-10-18 10:31:00+0000 to 2019-10-18 10:35:00+0000	Purge
	2019-10-19 09:46:00+0000 to 2019-10-19 10:00:00+0000	Zero
	2019-10-19 10:01:00+0000 to 2019-10-19 10:15:00+0000	Span
	2019-10-19 10:16:00+0000 to 2019-10-19 10:30:00+0000	Spare
	2019-10-19 10:31:00+0000 to 2019-10-19 10:35:00+0000	Purge
	2019-10-20 09:46:00+0000 to 2019-10-20 10:00:00+0000	Zero
	2019-10-20 10:01:00+0000 to 2019-10-20 10:15:00+0000	Span
	2019-10-20 10:16:00+0000 to 2019-10-20 10:30:00+0000	Spare
	2019-10-20 10:31:00+0000 to 2019-10-20 10:35:00+0000	Purge
	2019-10-21 09:46:00+0000 to 2019-10-21 10:00:00+0000	Zero
	2019-10-21 10:01:00+0000 to 2019-10-21 10:15:00+0000	Span
	2019-10-21 10:16:00+0000 to 2019-10-21 10:30:00+0000	Spare
	2019-10-21 10:31:00+0000 to 2019-10-21 10:35:00+0000	Purge
	2019-10-22 09:46:00+0000 to 2019-10-22 10:00:00+0000	Zero
	2019-10-22 10:01:00+0000 to 2019-10-22 10:15:00+0000	Span
	2019-10-22 10:16:00+0000 to 2019-10-22 10:30:00+0000	Spare
	2019-10-22 10:31:00+0000 to 2019-10-22 10:35:00+0000	Purge
	2019-10-22 19:13:00+0000	<Samp
	2019-10-22 19:14:00+0000 to 2019-10-22 19:28:00+0000	Down
	2019-10-23 09:46:00+0000 to 2019-10-23 10:00:00+0000	Zero
	2019-10-23 10:01:00+0000 to 2019-10-23 10:15:00+0000	Span
	2019-10-23 10:16:00+0000 to 2019-10-23 10:30:00+0000	Spare
	2019-10-23 10:31:00+0000 to 2019-10-23 10:35:00+0000	Purge
	2019-10-24 09:46:00+0000 to 2019-10-24 10:00:00+0000	Zero
	2019-10-24 10:01:00+0000 to 2019-10-24 10:15:00+0000	Span
	2019-10-24 10:16:00+0000 to 2019-10-24 10:30:00+0000	Spare

[1] "Contact us" DR DAS LTD, 2022, https://dr-das.com/Home/Contact.html Accessed June 3 2022

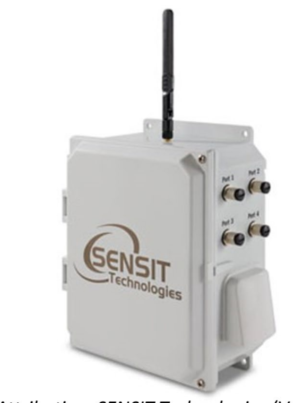

Image Attribution: SENSIT Technologies (Valparaiso, IN)

Data Flags Recorded During Testing (Cont.)

FRM/FEM Monitor	Timestamp (UTC)	Flag
Teledyne API T265 (Data acquired via local transfer from OAQPS)	2019-10-24 10:31:00+0000 to 2019-10-24 10:35:00+0000	Purge
	2019-10-25 09:46:00+0000 to 2019-10-25 10:00:00+0000	Zero
	2019-10-25 10:01:00+0000 to 2019-10-25 10:15:00+0000	Span
	2019-10-25 10:16:00+0000 to 2019-10-25 10:30:00+0000	Spare
	2019-10-25 10:31:00+0000 to 2019-10-25 10:35:00+0000	Purge
	2019-10-26 09:46:00+0000 to 2019-10-26 10:00:00+0000	Zero
	2019-10-26 10:01:00+0000 to 2019-10-26 10:15:00+0000	Span
	2019-10-26 10:16:00+0000 to 2019-10-26 10:30:00+0000	Spare
	2019-10-26 10:31:00+0000 to 2019-10-26 10:35:00+0000	Purge
	2019-10-27 09:46:00+0000 to 2019-10-27 10:00:00+0000	Zero
	2019-10-27 10:01:00+0000 to 2019-10-27 10:15:00+0000	Span
	2019-10-27 10:16:00+0000 to 2019-10-27 10:30:00+0000	Spare
	2019-10-27 10:31:00+0000 to 2019-10-27 10:35:00+0000	Purge
	2019-10-28 09:46:00+0000 to 2019-10-28 10:00:00+0000	Zero
	2019-10-28 10:01:00+0000 to 2019-10-28 10:15:00+0000	Span
	2019-10-28 10:16:00+0000 to 2019-10-28 10:30:00+0000	Spare
	2019-10-28 10:31:00+0000 to 2019-10-28 10:35:00+0000	Purge
	2019-10-29 09:46:00+0000 to 2019-10-29 10:00:00+0000	Zero
	2019-10-29 10:01:00+0000 to 2019-10-29 10:15:00+0000	Span
	2019-10-29 10:16:00+0000 to 2019-10-29 10:30:00+0000	Spare
	2019-10-29 10:31:00+0000 to 2019-10-29 10:35:00+0000	Purge
	2019-10-30 09:46:00+0000 to 2019-10-30 10:00:00+0000	Zero
	2019-10-30 10:01:00+0000 to 2019-10-30 10:15:00+0000	Span
	2019-10-30 10:16:00+0000 to 2019-10-30 10:30:00+0000	Spare
	2019-10-30 10:31:00+0000 to 2019-10-30 10:35:00+0000	Purge
	2019-10-31 09:46:00+0000 to 2019-10-31 10:00:00+0000	Zero
	2019-10-31 10:01:00+0000 to 2019-10-31 10:15:00+0000	Span
	2019-10-31 10:16:00+0000 to 2019-10-31 10:30:00+0000	Spare
	2019-10-31 10:31:00+0000 to 2019-10-31 10:35:00+0000	Purge
	2019-11-01 09:46:00+0000 to 2019-11-01 10:00:00+0000	Zero
	2019-11-01 10:01:00+0000 to 2019-11-01 10:15:00+0000	Span
	2019-11-01 10:16:00+0000 to 2019-11-01 10:30:00+0000	Spare
	2019-11-01 10:31:00+0000 to 2019-11-01 10:35:00+0000	Purge
	2019-11-02 09:46:00+0000 to 2019-11-02 10:00:00+0000	Zero
	2019-11-02 10:01:00+0000 to 2019-11-02 10:15:00+0000	Span
	2019-11-02 10:16:00+0000 to 2019-11-02 10:30:00+0000	Spare
	2019-11-02 10:31:00+0000 to 2019-11-02 10:35:00+0000	Purge
	2019-11-03 09:46:00+0000 to 2019-11-03 10:00:00+0000	Zero
	2019-11-03 10:01:00+0000 to 2019-11-03 10:15:00+0000	Span
	2019-11-03 10:16:00+0000 to 2019-11-03 10:30:00+0000	Spare
	2019-11-03 10:31:00+0000 to 2019-11-03 10:35:00+0000	Purge
	2019-11-04 09:46:00+0000 to 2019-11-04 10:00:00+0000	Zero
	2019-11-04 10:01:00+0000 to 2019-11-04 10:15:00+0000	Span
	2019-11-04 10:16:00+0000 to 2019-11-04 10:30:00+0000	Spare
	2019-11-04 10:31:00+0000 to 2019-11-04 10:35:00+0000	Purge
	2019-11-05 09:46:00+0000 to 2019-11-05 10:00:00+0000	Zero

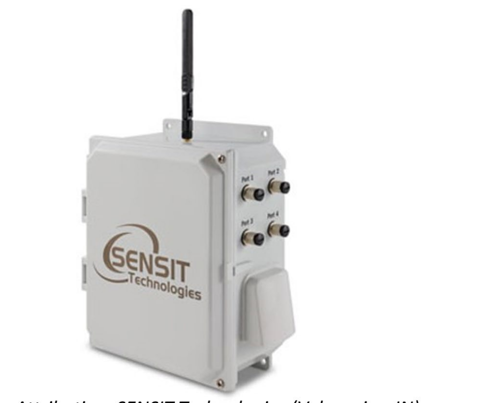

Image Attribution: SENSIT Technologies (Valparaiso, IN)

Data Flags Recorded During Testing (Cont.)

FRM/FEM Monitor	Timestamp (UTC)	Flag
Teledyne API T265 (Data acquired via local transfer from OAQPS)	2019-11-05 10:01:00+0000 to 2019-11-05 10:15:00+0000	Span
	2019-11-05 10:16:00+0000 to 2019-11-05 10:30:00+0000	Spare
	2019-11-05 10:31:00+0000 to 2019-11-05 10:35:00+0000	Purge
	2019-11-06 09:46:00+0000 to 2019-11-06 10:00:00+0000	Zero
	2019-11-06 10:01:00+0000 to 2019-11-06 10:15:00+0000	Span
	2019-11-06 10:16:00+0000 to 2019-11-06 10:30:00+0000	Spare
	2019-11-06 10:31:00+0000 to 2019-11-06 10:35:00+0000	Purge
	2019-11-07 09:46:00+0000 to 2019-11-07 10:00:00+0000	Zero
	2019-11-07 10:01:00+0000 to 2019-11-07 10:15:00+0000	Span
	2019-11-07 10:16:00+0000 to 2019-11-07 10:30:00+0000	Spare
	2019-11-07 10:31:00+0000 to 2019-11-07 10:35:00+0000	Purge
	2019-11-07 22:05:00+0000	<Samp
	2019-11-08 09:46:00+0000 to 2019-11-08 10:00:00+0000	Zero
	2019-11-08 10:01:00+0000 to 2019-11-08 10:15:00+0000	Span
	2019-11-08 10:16:00+0000 to 2019-11-08 10:30:00+0000	Spare
	2019-11-08 10:31:00+0000 to 2019-11-08 10:35:00+0000	Purge
	2019-11-08 15:52:00+0000	<Samp
	2019-11-08 15:59:00+0000	<Samp
	2019-11-08 16:09:00+0000	<Samp
	2019-11-09 09:46:00+0000 to 2019-11-09 10:00:00+0000	Zero
	2019-11-09 10:01:00+0000 to 2019-11-09 10:15:00+0000	Span
	2019-11-09 10:16:00+0000 to 2019-11-09 10:30:00+0000	Spare
	2019-11-09 10:31:00+0000 to 2019-11-09 10:35:00+0000	Purge
	2019-11-10 09:46:00+0000 to 2019-11-10 10:00:00+0000	Zero
	2019-11-10 10:01:00+0000 to 2019-11-10 10:15:00+0000	Span
	2019-11-10 10:16:00+0000 to 2019-11-10 10:30:00+0000	Spare
	2019-11-10 10:31:00+0000 to 2019-11-10 10:35:00+0000	Purge
	2019-11-11 09:46:00+0000 to 2019-11-11 10:00:00+0000	Zero
	2019-11-11 10:01:00+0000 to 2019-11-11 10:15:00+0000	Span
	2019-11-11 10:16:00+0000 to 2019-11-11 10:30:00+0000	Spare
	2019-11-11 10:31:00+0000 to 2019-11-11 10:35:00+0000	Purge
	2019-11-12 09:46:00+0000 to 2019-11-12 10:00:00+0000	Zero
	2019-11-12 10:01:00+0000 to 2019-11-12 10:15:00+0000	Span
	2019-11-12 10:16:00+0000 to 2019-11-12 10:30:00+0000	Spare
	2019-11-12 10:31:00+0000 to 2019-11-12 10:35:00+0000	Purge
	2019-11-12 18:31:00+0000 to 2019-11-12 18:32:00+0000	NoData
	2019-11-12 18:33:00+0000	<Samp
	2019-11-12 18:36:00+0000	<Samp
	2019-11-12 19:09:00+0000 to 2019-11-12 19:10:00+0000	<Samp
	2019-11-12 19:12:00+0000	<Samp
	2019-11-12 19:24:00+0000	<Samp
	2019-11-12 19:28:00+0000	<Samp
	2019-11-12 19:30:00+0000	NoData
	2019-11-12 19:31:00+0000	<Samp
	2019-11-12 19:33:00+0000	<Samp

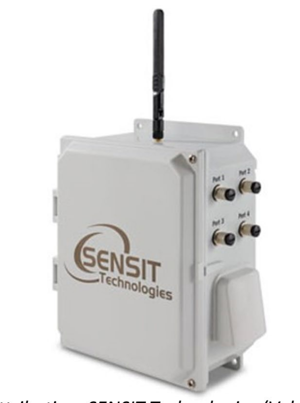

Image Attribution: SENSIT Technologies (Valparaiso, IN)

Data Flags Recorded During Testing (Cont.)

FRM/FEM Monitor	Timestamp (UTC)	Flag
Teledyne API T265 (Data acquired via local transfer from OAQPS)	2019-11-12 19:34:00+0000	NoData
	2019-11-12 19:36:00+0000	<Samp
	2019-11-12 19:38:00+0000	<Samp
	2019-11-13 09:46:00+0000 to 2019-11-13 10:00:00+0000	Zero
	2019-11-13 10:01:00+0000 to 2019-11-13 10:15:00+0000	Span
	2019-11-13 10:16:00+0000 to 2019-11-13 10:30:00+0000	Spare
	2019-11-13 10:31:00+0000 to 2019-11-13 10:35:00+0000	Purge
	2019-11-13 17:22:00+0000	NoData
	2019-11-13 17:26:00+0000	<Samp
	2019-11-13 17:29:00+0000	<Samp
	2019-11-13 17:31:00+0000	<Samp
	2019-11-13 17:36:00+0000	NoData
	2019-11-13 17:39:00+0000	NoData
	2019-11-13 17:50:00+0000	<Samp
	2019-11-13 17:55:00+0000 to 2019-11-13 17:56:00+0000	NoData
	2019-11-13 17:57:00+0000	<Samp
	2019-11-14 09:46:00+0000 to 2019-11-14 10:00:00+0000	Zero
	2019-11-14 10:01:00+0000 to 2019-11-14 10:15:00+0000	Span
	2019-11-14 10:16:00+0000 to 2019-11-14 10:30:00+0000	Spare
	2019-11-14 10:31:00+0000 to 2019-11-14 10:35:00+0000	Purge
	2019-11-15 07:02:00+0000 to 2019-11-15 07:03:00+0000	NoData
	2019-11-15 07:04:00+0000	<Samp
	2019-11-15 09:46:00+0000 to 2019-11-15 10:00:00+0000	Zero
	2019-11-15 10:01:00+0000 to 2019-11-15 10:15:00+0000	Span
	2019-11-15 10:16:00+0000 to 2019-11-15 10:30:00+0000	Spare
	2019-11-15 10:31:00+0000 to 2019-11-15 10:35:00+0000	Purge
	2019-11-16 09:46:00+0000 to 2019-11-16 10:00:00+0000	Zero
	2019-11-16 10:01:00+0000 to 2019-11-16 10:15:00+0000	Span
	2019-11-16 10:16:00+0000 to 2019-11-16 10:30:00+0000	Spare
	2019-11-16 10:31:00+0000 to 2019-11-16 10:35:00+0000	Purge
	2019-11-17 09:46:00+0000 to 2019-11-17 10:00:00+0000	Zero
	2019-11-17 10:01:00+0000 to 2019-11-17 10:15:00+0000	Span
	2019-11-17 10:16:00+0000 to 2019-11-17 10:30:00+0000	Spare
	2019-11-17 10:31:00+0000 to 2019-11-17 10:35:00+0000	Purge

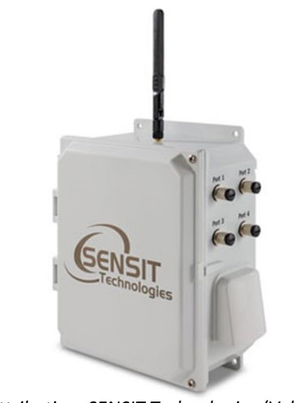

Image Attribution: SENSIT Technologies (Valparaiso, IN)

Data Flags Recorded During Testing (Cont.)

FRM/FEM Monitor	Timestamp (UTC)	Flag
RM Young 41382 VC Temperature and Relative Humidity Monitor (Data acquired via local transfer from OAQPS)	2019-11-07 22:05:00+0000	<Samp
	2019-11-08 15:52:00+0000	<Samp
	2019-11-08 15:59:00+0000	<Samp
	2019-11-08 16:09:00+0000	<Samp
	2019-11-12 18:31:00+0000 to 2019-11-12 18:32:00+0000	NoData [maintenance]
	2019-11-12 18:33:00+0000	<Samp
	2019-11-12 18:36:00+0000	<Samp
	2019-11-12 19:09:00+0000 to 2019-11-12 19:10:00+0000	<Samp
	2019-11-12 19:12:00+0000	<Samp
	2019-11-12 19:24:00+0000	<Samp
	2019-11-12 19:28:00+0000	<Samp
	2019-11-12 19:31:00+0000	<Samp
	2019-11-12 19:33:00+0000	<Samp
	2019-11-12 19:34:00+0000	NoData [maintenance]
	2019-11-12 19:36:00+0000	<Samp
	2019-11-12 19:38:00+0000	<Samp
	2019-11-13 17:22:00+0000	NoData [maintenance]
	2019-11-13 17:26:00+0000	<Samp
	2019-11-13 17:29:00+0000	<Samp
	2019-11-13 17:31:00+0000	<Samp
	2019-11-13 17:36:00+0000	NoData [maintenance]
	2019-11-13 17:39:00+0000	NoData [maintenance]
	2019-11-13 17:50:00+0000	<Samp
	2019-11-13 17:55:00+0000 to 2019-11-13 17:56:00+0000	NoData [maintenance]
	2019-11-13 17:57:00+0000	<Samp
	2019-11-14 14:37:00+0000	NoData [maintenance]
	2019-11-15 07:02:00+0000 to 2019-11-15 07:03:00+0000	NoData [maintenance]
	2019-11-15 07:04:00+0000	<Samp

Testing Report - O_3 Base Testing

Aeroqual AQY

This report reflects out-of-the-box performance

Initial Base Testing - Edmond, OK

U.S. Environmental Protection Agency
Office of Research and Development
PI: Clements.Andrea@epa.gov
919-541-1363
August 2019—September 2019

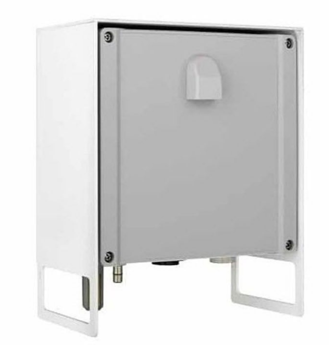

Image Attribution: Aeroqual Limited (Auckland, New Zealand)

Deployment Details

Testing Organization and Site Information	
Testing organization (Name, Organization type, Contact website)	U.S. Environmental Protection Agency - Office of Research and Development<br>Federal Government<br>Air Sensor Toolbox | U.S. EPA Website
Testing location (City, State, Latitude and Longitude)	OK Christian University<br>Edmond, OK<br>35.614131, -97.475083
AQS site ID	08 - 031 - 0026
Sampling timeframe (MM-DD-YY)	08-02-19 to 09-01-19
Sensor data source	Aeroqual Cloud
Reference data source	AQS API download

Sensor Information			
Manufacturer, model	Aeroqual AQY		
Device firmware version	1.14.2		
Sampling time interval	1-minute		
Sensor serial numbers	AQY_01		
Issues encountered during deployment?	☐	No Issues	

FRM/FEM Information	
Manufacturer, model, designation	Teledyne API T400 FEM
Sampling time interval	1-hour averaging
Date of calibration	As required by 40 CFR Part 58 and the QAPP for Ambient Air Monitoring in the State of Oklahoma maintained by ODEQ/AQD
Date of one-point QC check	Every two weeks as required by 40 CFR Part 58 Appendix A 3.1.1
Description, date(s) of maintenance activities	N/A

Time Series Plot: 1-hour averaged O_3

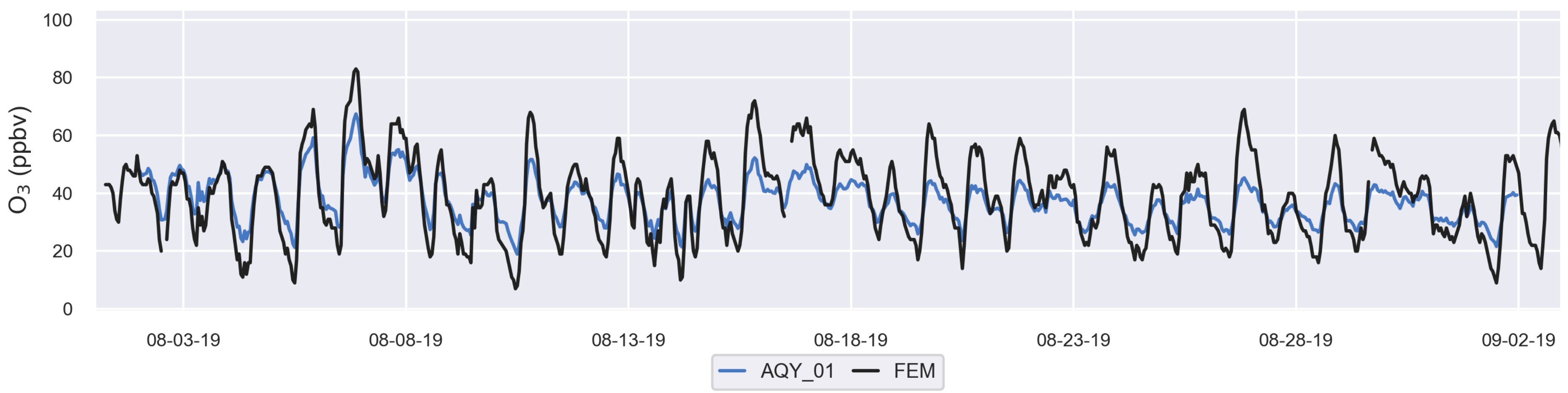

Range and average of FRM/FEM concentrations over duration of base test (ppbv)	[1-hr] 7.0-83.0, avg: 38.6,<br>[Rolling 8-hr] 12.5-76.2, avg: 38.5
Number of 1-hr periods in FRM/FEM monitor measurements with a goal concentration ≥ 60 ppbv	50

Scatter Plot: Comparison to FRM/FEM

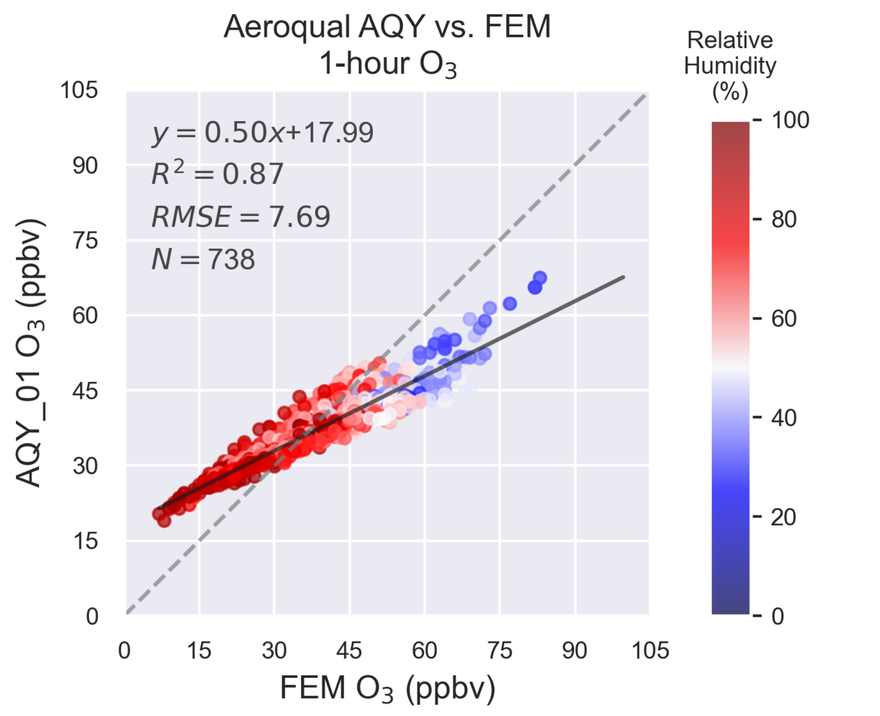

Performance Metrics

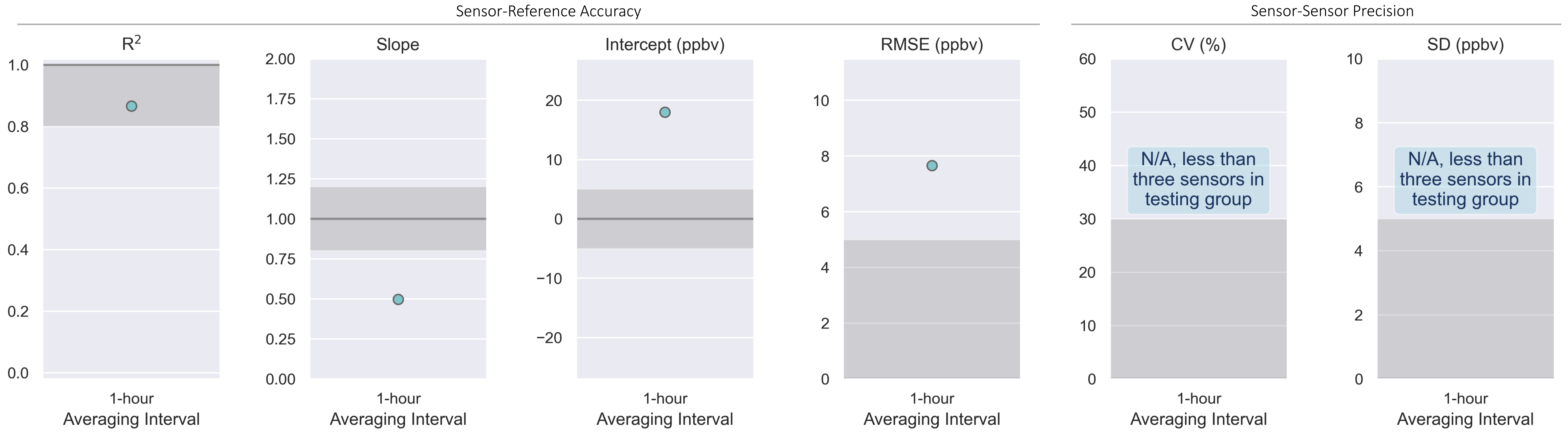

Meteorological Conditions During Deployment

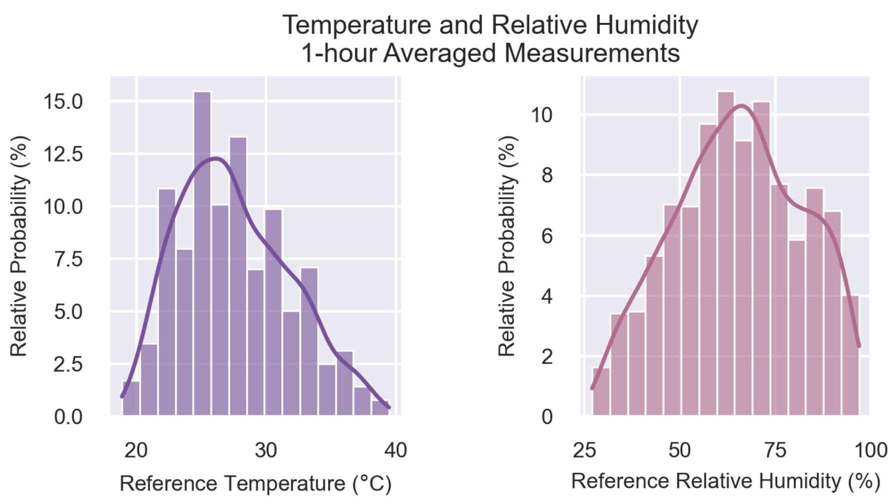

Number of 1-hr periods outside sensor manufacturer-listed temperature operational range (-10 to 40 °C)	0
Number of 1-hr periods outside sensor manufacturer-listed relative humidity operational range (no operational range specified)	-

Meteorological Influence

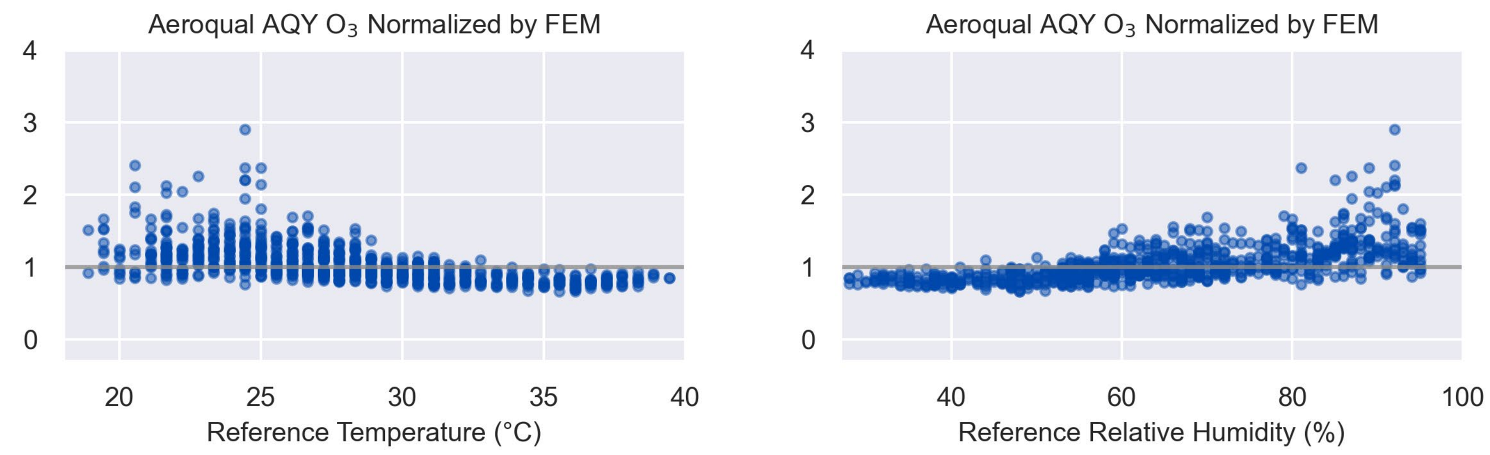

Mean number of paired, normalized concentration and temperature values (1-hr averages)	744
Mean number of paired, normalized concentration and relative humidity values (1-hr averages)	744

1:1
AQY_01

FEM data <5 ppbv (Federal MDL for the T400) has been remove (Meteorological Influence section only)

Testing Report - O_3 Base Testing

Aeroqual AQY

This report reflects out-of-the-box performance

Initial Base Testing - Edmond, OK

U.S. Environmental Protection Agency
Office of Research and Development
PI: Clements.Andrea@epa.gov
919-541-1363

August 2019—September 2019

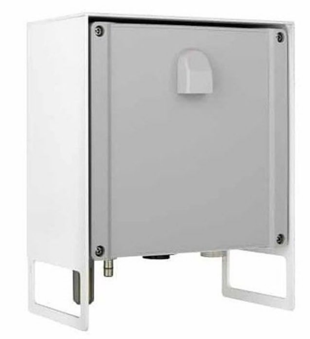

Image Attribution: Aeroqual Limited (Auckland, New Zealand)

Tabular Statistics

Sensor-FRM/FEM Correlation

	Bias and Linearity			Data Quality	
	R^2	Slope	Intercept (ppbv)	Uptime (%)	Number of paired sensor and reference concentration values*
	1-Hour ●	1-Hour ○	1-Hour ○	1-Hour ●	1-Hour
Metric Target Range	≥ 0.80	1.0 ± 0.20	-5 ≤ b ≤ 5	75%*	-
Sensor AQY_01	0.87	0.50	17.99	100	738

	Error
	RMSE (ppbv)
	1-Hour ☆
Metric Target Range	≤ 5.0
Deployment Value	7.7

Device-specific metrics (computed for each sensor in evaluation)
- ○ Metric value for none of devices tested falls within the target range
- ● Metric value for one of devices tested falls within the target range

Sensor-Sensor Precision[1]

	Precision (between collocated sensors)		Data Quality
	CV (%)	SD (ppbv)	Number of paired sensor and reference concentration values*
	1-Hour ☆	1-Hour ☆	1-Hour
Metric Target Range	≤ 30.0	≤ 5.0	-
Deployment Value	-	-	-

[1]Precision statistics are computed for evaluations with at least three collocated sensor units. Metric values are left blank for evaluations with two or fewer sensor units.

Single-valued metrics (computed via entire evaluation dataset)
- ☆ Indicates that the metric value is not within the target range
- ★ Indicates that the metric value is within the target range

*This value is only a recommendation for ensuring data quality and is not included in the list of target values discussed in Section 4 of the Performance Testing Protocols, Metrics, and Target Values for Ozone Air Sensors document.

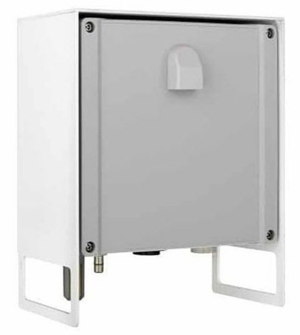

Image Attribution: Aeroqual Limited (Auckland, New Zealand)

Supplemental Information

Abbreviations used in Supplemental Information

FRM	Federal Reference Method
FEM	Federal Equivalent Method
SOP	Standard Operating Procedure
QAPP	Quality Assurance Project Plan
QC	Quality Control

Supplemental Documentation	Attached	Description & URL or file path to documentation
Field observations and sensor data flags	☑	See OK-AQY-Page 5 of this testing report
Maintenance logs	☐	No logs recorded during testing
Standard operating procedure(s)	☐	U.S. EPA Office Of Research and Development SOP available upon request
Photos of equipment setup and testing	☑	See OK-AQY-Page 4 of this testing report
Product specifications sheet(s)	☑	See Appendix C, “Spec_Sheet_Aeroqual_AQY.pdf”*
Product manual(s)	☑	See Appendix C, “Manual_Aeroqual_AQY.pdf”*
Deployment issues	☐	See OK-AQY-Page 5 of this testing report
Data storage and transmission method	☑	See OK-AQY-Page 5 of this testing report
Data correction approach	☑	See OK-AQY-Page 5 of this testing report
Data analysis/correction scripts and version	☑	Averaging and processing of data, calculation of performance metrics, and generation of figures and other supplementary material for analysis were obtained using Python 3.9.7 with the packages sensortoolkit v0.8.3b2, pandas 1.3.5, NumPy 1.21.2, Matplotlib 3.5.0, statsmodels 0.13.0, and seaborn 0.11.2. All packages are available from the Python Package Index (PyPI) at https://pypi.org. The integrated development environment (IDE) Spyder 5.1.5 was used for scripting and data visualization. Version control for the Python base, packages, and IDE were all managed by conda 4.11.0.
Air Monitoring Station QAPP	☐	U.S. EPA Office Of Research and Development QAPP available upon request
Summary of FRM/FEM monitor QC checks	☑	See OK-AQY-Page 6 of this testing report
Manufacturer website for FRM/FEM monitor	☑	Teledyne API: Model T400 Product website
FRM/FEM monitor manual	☑	See Appendix B, “Spec_Sheet_TeledyneAPI_T400.pdf”
FRM/FEM monitor specifications sheet(s)	☑	See Appendix B, “Manual_TeledyneAPI_T400.pdf”
Other documents	☑	Manufacturer notice of AQY sales on hold

*As of 3/18/2021, the manufacturer of the AQY has placed sales of a similar unit on hold. Documentation for the AQY is currently unavailable from the manufacturer’s website.

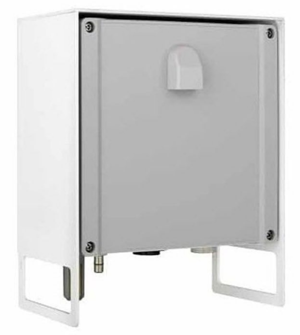

Image Attribution: Aeroqual Limited (Auckland, New Zealand)

Supplemental Information: Photos of Testing Site and Equipment Setup

Site Description:
The Oklahoma Christian University monitoring site was established in the late 1970s. There is a containerized building that houses the continuous instruments with a wooden platform directly adjacent for the filter-based instruments. Pollutants monitored include O_3, PM_{10} (filter based and continuous), $PM_{2.5}$ (filter based and continuous), $NO/NO_2/NO_x$, CO, SO_2 and meteorology. The site has solar panels to help defer the cost of power to the site.

Figure 1: Aeroqual AQY (indicated by red arrow) attached to metal railing atop the sampling shelter at the monitoring site.

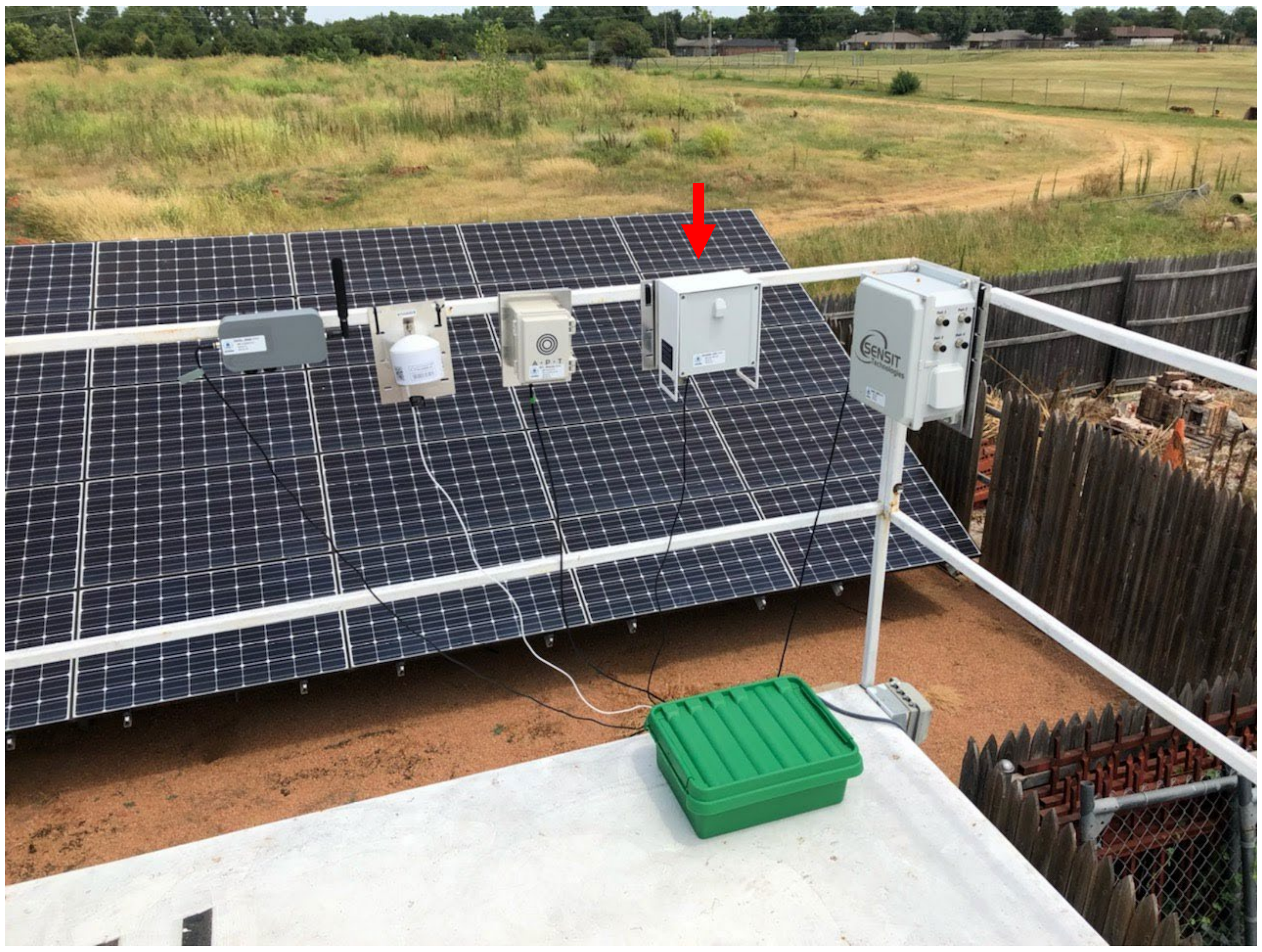

Figure 2: OK Monitoring Station sampling shelter

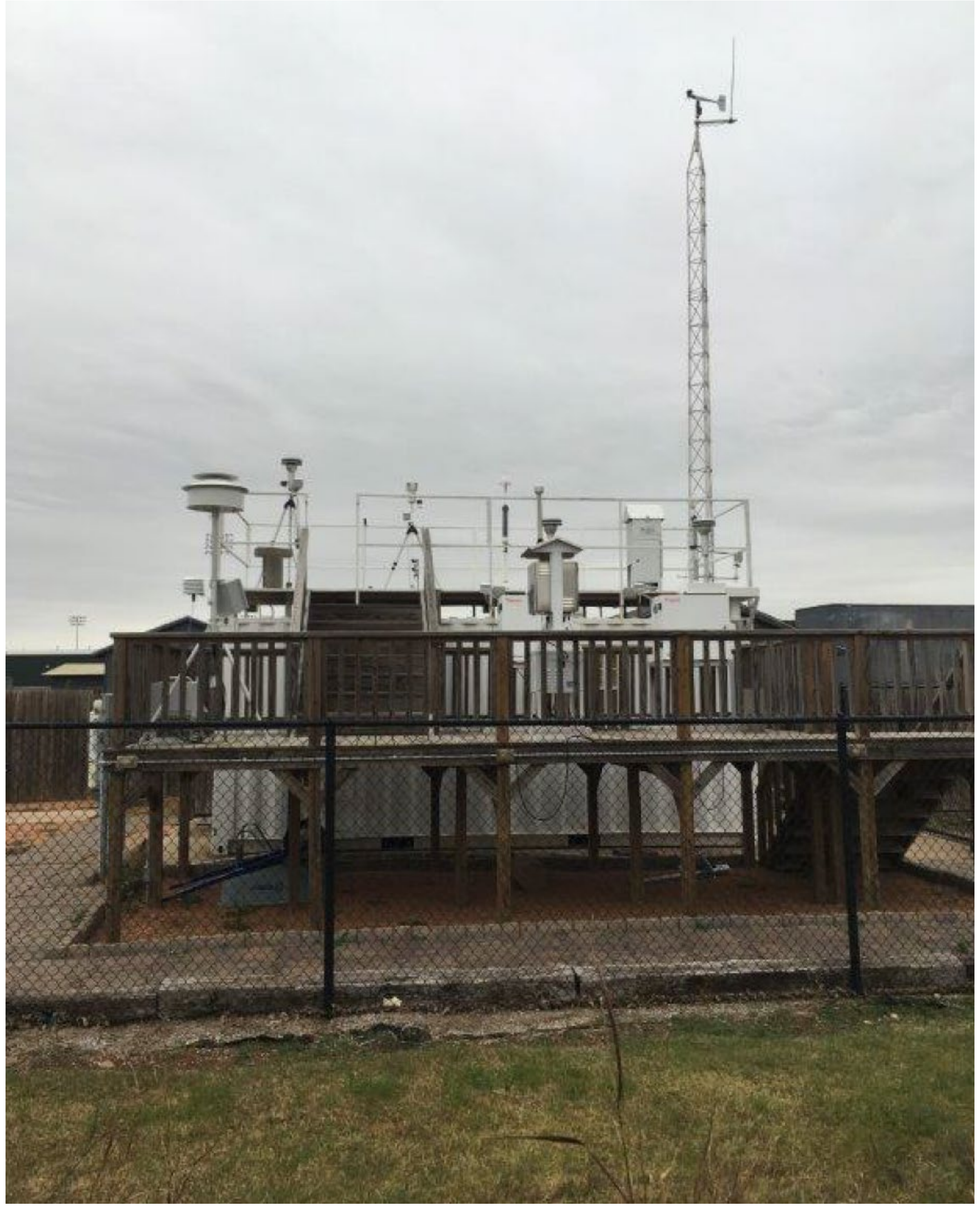

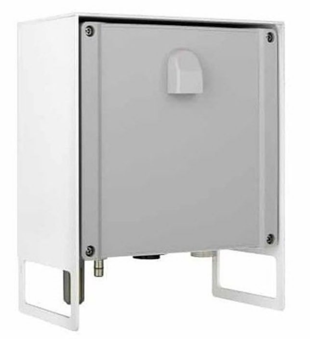

Image Attribution: Aeroqual Limited (Auckland, New Zealand)

Supplemental Information: Data Storage, Correction Approach, and Issues Encountered

Data Storage and Transmission Method

As part of CRADA #934-16 between Aeroqual and US EPA, Aeroqual supported data streaming. SIM cards were installed and data flowed to the Aeroqual Cloud. The 1-minute raw data was acquired weekly using the Aeroqual Cloud (*last accessed 5/11/22*) user interface (UI). The AQY has an internal data storage USB flash drive as a data backup, however access requires software proprietary to Aeroqual.

Data Correction Approach

This evaluation report reflects "out-of-the-box" performance of the AQY. The manufacturer provides a procedure by which local collocation (sensor operated along side an FRM/FEM) data can be collected, a gain (slope) and offset (intercept) determined, and parameters entered into the Aeroqual Cloud user interface to be applied to all subsequently collected data. This procedure and feature was **not** used prior to this evaluation. Prospective consumers may get different performance from this device if they utilize this feature.

After acquisition, the raw data was processed using the *sensortoolkit* python code library (v0.8.3b2). A continuous data set at the recorded sampling frequency was written to a .csv file. 1-hour averaged data sets were generated using a 75% completeness threshold and saved as separate .csv files. Outliers were **not** removed from data sets in order to assess "out-of-the-box" sensor performance.

The duration of the warm-up period required for sensor measurements to equilibrate was determined from field data to be 10 minutes. Warm up periods were considered to occur following any power outage to sensor units, either due to routine field visits or unscheduled site power outages. Data recorded during warm up periods has been removed from data sets.

Issues Encountered

Pre-deployment observations

- *Timestamp inaccuracies*: During pre-deployment, the AQY devices did not properly sync timestamps with the onboard Real-Time Clock. Connecting the units to the internet by cellular or Wi-Fi allowed the unit to sync with internet time and resulted in proper timestamps.

Field observations and sensor data flags

The Aeroqual AQY was deployed at the OK monitoring site on 8/1/2019. The AQY unit operated nominally during the testing period and did not require replacement or repair.

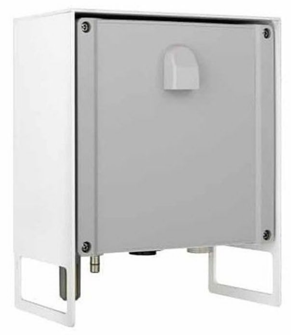

Image Attribution: Aeroqual Limited (Auckland, New Zealand)

Description of Data Flags

AQS

The U.S. EPA's Air Quality System (AQS) is the Agency's primary ambient air monitoring data archive. A comprehensive list of data flags that are recorded alongside AQS data sets, referred to by U.S. EPA as 'qualifiers', can be found at the following link: https://aqs.epa.gov/aqsweb/documents/codetables/qualifiers.html

Invalidation of reference data: AQS qualifiers are organized by qualifier type, which indicates whether data logged alongside qualifier flags should be invalidated (set null). Qualifiers with type "Null Data Qualifier" are invalidated, and includes data logged during periods that coincide with QC checks (e.g., "BF-Precision/Zero/Span", "BJ- Operator Error", "BL - QA Audit", "AZ - QC Audit") among other events such as power outages. Data logged alongside qualifiers with type "Quality Assurance Qualifiers" are not invalidated and are included in this analysis (e.g., concentrations less than the federal MDL for the reference monitor "MD – Value less than MDL", QA reviewed values "Validated Value").

Data Flags Recorded During Testing

FRM/FEM Monitor	Timestamp (UTC)	Flag
Teledyne Advanced Pollution Instrumentation T400 (Acquired via AQS)	2019-08-02 13:00:00+0000 to 2019-08-02 14:00:00+0000	BF - Precision/Zero/Span
	2019-08-16 13:00:00+0000 to 2019-08-16 15:00:00+0000	BF - Precision/Zero/Span
	2019-08-29 16:00:00+0000	BF - Precision/Zero/Span

Testing Report - O_3 Base Testing

SENSIT RAMP

This report reflects out-of-the-box performance

Initial Base Testing - Edmond, OK

U.S. Environmental Protection Agency
Office of Research and Development
PI: Clements.Andrea@epa.gov
919-541-1363

August 2019—September 2019

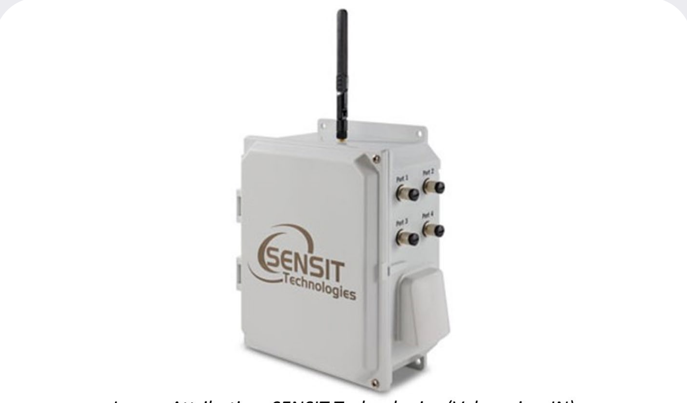

Image Attribution: SENSIT Technologies (Valparaiso, IN)

Deployment Details

Testing Organization and Site Information	
Testing organization (Name, Organization type, Contact website)	U.S. Environmental Protection Agency - Office of Research and Development<br>Federal Government<br>Air Sensor Toolbox | U.S. EPA Website
Testing location (City, State, Latitude and Longitude)	OK Christian University<br>Edmond, OK<br>35.614131, -97.475083
AQS site ID	08 - 031 - 0026
Sampling timeframe (MM-DD-YY)	08-15-19 to 09-14-19
Sensor data source	Onboard MicroSD card
Reference data source	AQS API download

Sensor Information			
Manufacturer, model	SENSIT RAMP		
Device firmware version	190313_AQ_v9.30		
Sampling time interval	15-seconds		
Sensor serial numbers	RAM_01		
Issues encountered during deployment?	☐	No Issues	

FRM/FEM Information	
Manufacturer, model, designation	Teledyne API T400 FEM
Sampling time interval	1-hour averaging
Date of calibration	As required by 40 CFR Part 58 and the QAPP for Ambient Air Monitoring in the State of Oklahoma maintained by ODEQ/AQD
Date of one-point QC check	Every two weeks as required by 40 CFR Part 58 Appendix A 3.1.1
Description, date(s) of maintenance activities	N/A

Time Series Plot: 1-hour averaged O_3

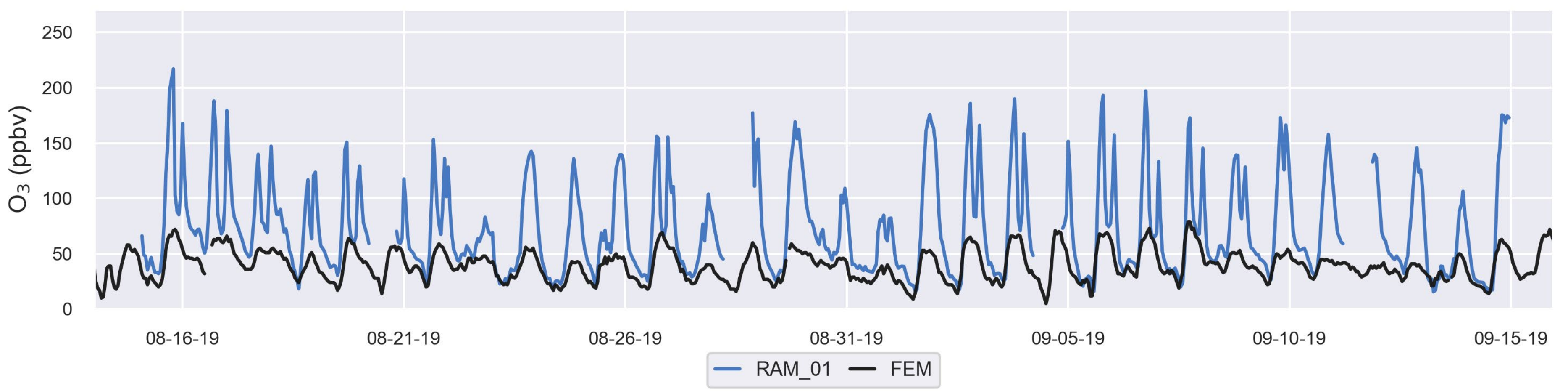

Range and average of FRM/FEM concentrations over duration of base test (ppbv)	[1-hr] 5.0-79.0, avg: 39.2,<br>[Rolling 8-hr] 15.0-70.9, avg: 39.1
Number of 1-hr periods in FRM/FEM monitor measurements with a goal concentration ≥ 60 ppbv	62

Scatter Plot: Comparison to FRM/FEM

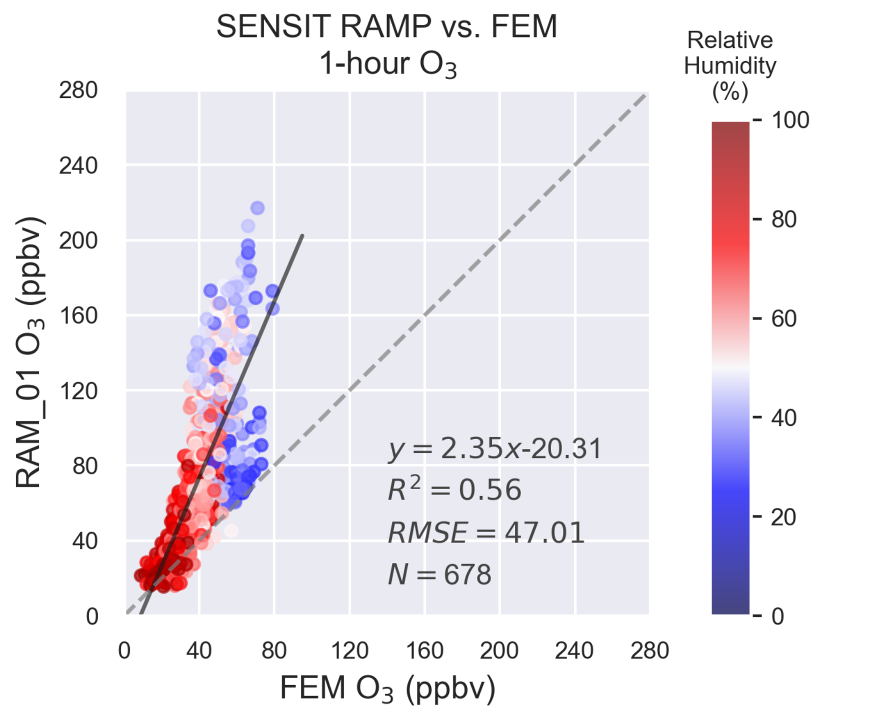

Performance Metrics

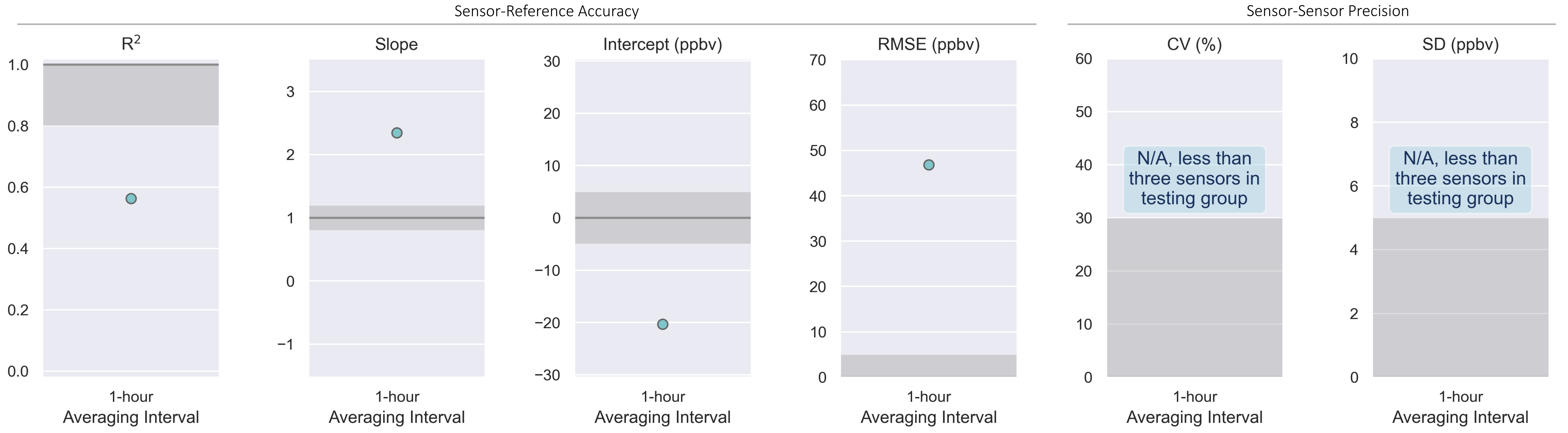

Meteorological Conditions During Deployment

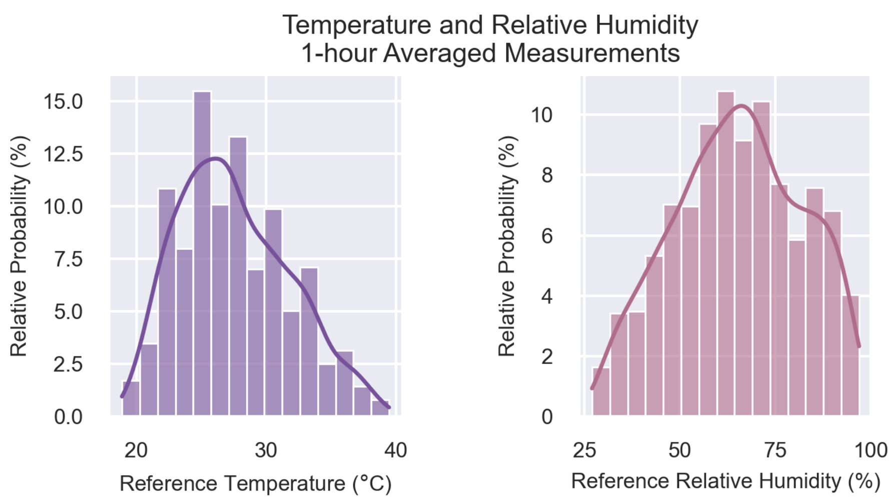

Number of 1-hr periods outside sensor manufacturer-listed temperature operational range (-20 to 50 °C)	0
Number of 1-hr periods outside sensor manufacturer-listed relative humidity operational range (no operational range specified)	-

Meteorological Influence

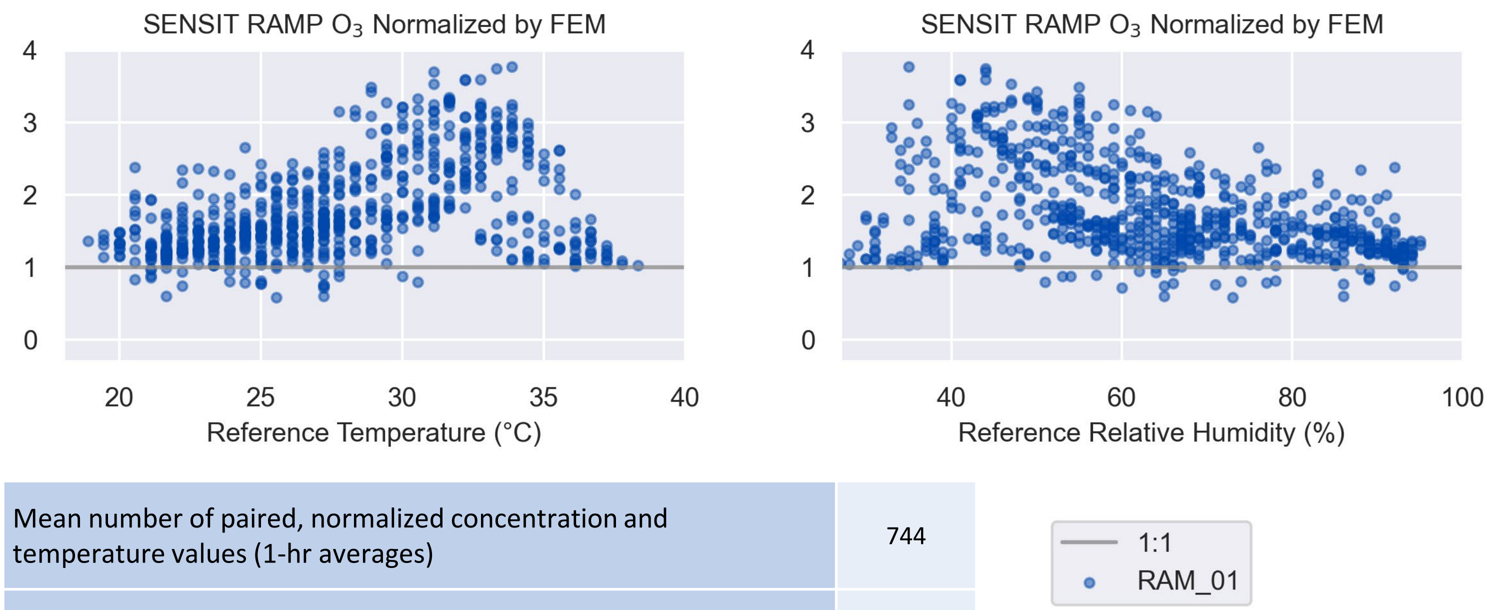

Mean number of paired, normalized concentration and temperature values (1-hr averages)	744
Mean number of paired, normalized concentration and relative humidity values (1-hr averages)	744

FEM data <5 ppbv (Federal MDL for the T400) has been remove (Meteorological Influence section only)

Testing Report - O_3 Base Testing

SENSIT RAMP

This report reflects out-of-the-box performance

Initial Base Testing - Edmond, OK

U.S. Environmental Protection Agency
Office of Research and Development
PI: Clements.Andrea@epa.gov
919-541-1363

August 2019—September 2019

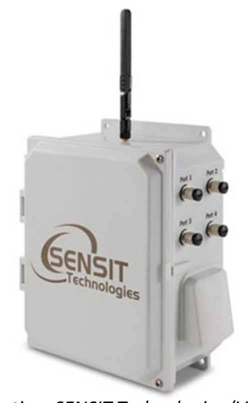

Image Attribution: SENSIT Technologies (Valparaiso, IN)

Tabular Statistics

Sensor-FRM/FEM Correlation

	Bias and Linearity			Data Quality	
	R^2	Slope	Intercept (ppbv)	Uptime (%)	Number of paired sensor and reference concentration values*
	1-Hour ○	1-Hour ○	1-Hour ○	1-Hour ●	1-Hour
Metric Target Range	≥ 0.80	1.0 ± 0.20	-5 ≤ b ≤ 5	75%*	-
Sensor RAM_01	0.56	2.35	-20.31	93	678

	Error
	RMSE (ppbv)
	1-Hour ☆
Metric Target Range	≤ 5.0
Deployment Value	46.8

Device-specific metrics (computed for each sensor in evaluation)
- ○ Metric value for none of devices tested falls within the target range
- ● Metric value for one of devices tested falls within the target range

Sensor-Sensor Precision[1]

	Precision (between collocated sensors)		Data Quality
	CV (%)	SD (ppbv)	Number of paired sensor and reference concentration values*
	1-Hour ☆	1-Hour ☆	1-Hour
Metric Target Range	≤ 30.0	≤ 5.0	-
Deployment Value	-	-	-

[1]Precision statistics are computed for evaluations with at least three collocated sensor units. Metric values are left blank for evaluations with two or fewer sensor units.

Single-valued metrics (computed via entire evaluation dataset)
- ☆ Indicates that the metric value is not within the target range
- ★ Indicates that the metric value is within the target range

*This value is only a recommendation for ensuring data quality and is not included in the list of target values discussed in Section 4 of the Performance Testing Protocols, Metrics, and Target Values for Ozone Air Sensors document.

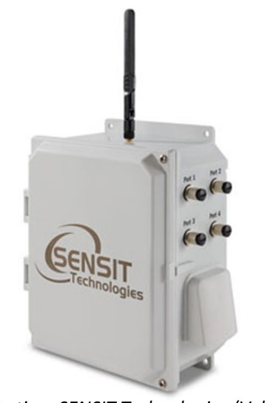

Image Attribution: SENSIT Technologies (Valparaiso, IN)

Supplemental Information

Abbreviations used in Supplemental Information

FRM	Federal Reference Method
FEM	Federal Equivalent Method
SOP	Standard Operating Procedure
QAPP	Quality Assurance Project Plan
QC	Quality Control

Supplemental Documentation	Attached	Description & URL or file path to documentation
Field observations and sensor data flags	☑	See OK-RAM-Pages 5, 6 of this testing report
Maintenance logs	☐	No logs recorded during testing
Standard operating procedure(s)	☐	U.S. EPA Office Of Research and Development SOP available upon request
Photos of equipment setup and testing	☑	See OK-RAM-Page 4 of this testing report
Product specifications sheet(s)	☑	See Appendix C, “Spec_Sheet_SENSIT_RAMP.pdf”
Product manual(s)	☑	See Appendix C, “Manual_SENSIT_RAMP.pdf”
Data storage and transmission method	☑	See OK-RAM-Page 5 of this testing report
Data correction approach	☑	See OK-RAM-Page 5 of this testing report
Issues encountered	☑	See OK-RAM-Page 5 of this testing report
Data analysis/correction scripts and version	☑	Averaging and processing of data, calculation of performance metrics, and generation of figures and other supplementary material for analysis were obtained using Python 3.9.7 with the packages sensortoolkit v0.8.3b2, pandas 1.3.5, NumPy 1.21.2, Matplotlib 3.5.0, statsmodels 0.13.0, and seaborn 0.11.2. All packages are available from the Python Package Index (PyPI) at https://pypi.org. The integrated development environment (IDE) Spyder 5.1.5 was used for scripting and data visualization. Version control for the Python base, packages, and IDE were all managed by conda 4.11.0.
Air Monitoring Station QAPP	☐	U.S. EPA Office Of Research and Development QAPP available upon request
Summary of FRM/FEM monitor QC checks	☑	See OK-RAM-Page 7 of this testing report
Manufacturer website for FRM/FEM monitor	☑	Teledyne API: Model T400 Product website
FRM/FEM monitor manual	☑	See Appendix B, “Spec_Sheet_TeledyneAPI_T400.pdf”
FRM/FEM monitor specifications sheet(s)	☑	See Appendix B, “Manual_TeledyneAPI_T400.pdf”
Other documents	☐	

Site Description:

The Oklahoma Christian University monitoring site was established in the late 1970s. There is a containerized building that houses the continuous instruments with a wooden platform directly adjacent for the filter-based instruments. Pollutants monitored include O_3, PM_{10} (filter based and continuous), $PM_{2.5}$ (filter based and continuous), $NO/NO_2/NO_x$, CO, SO_2 and meteorology. The site has solar panels to help defer the cost of power to the site.

Figure 1: SENSIT RAMP (indicated by red arrow) attached to metal railing atop the sampling shelter at the monitoring site.

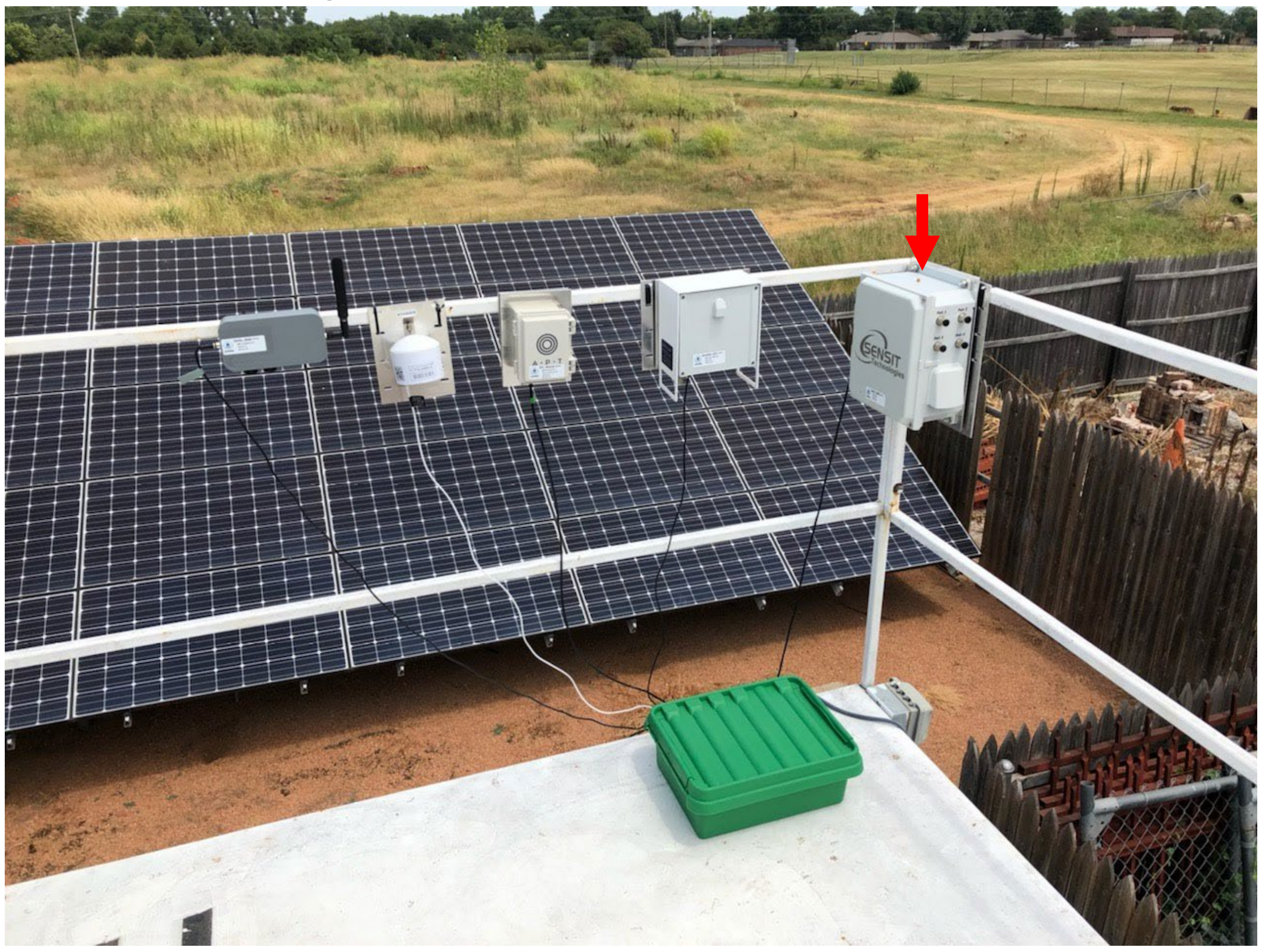

Figure 2: OK Monitoring Station sampling shelter

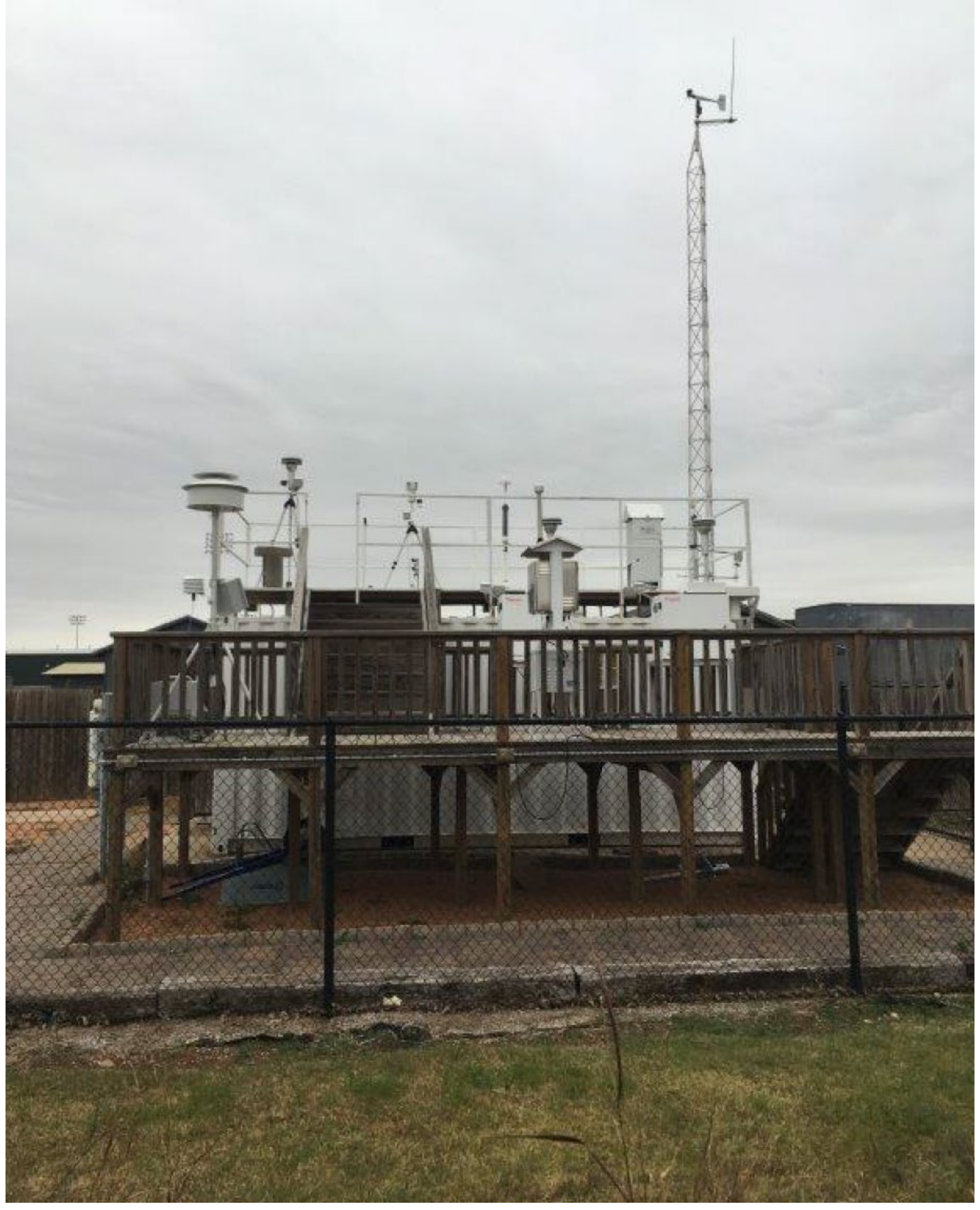

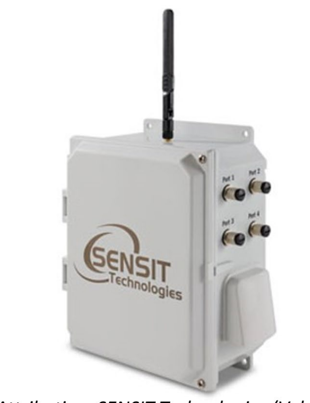

Image Attribution: SENSIT Technologies (Valparaiso, IN)

Data Storage and Transmission Method

The SENSIT RAMP was configured to record data at a 15-second sampling interval. Data are stored as daily text files (.txt format) on an onboard MicroSD card. Data files were obtained weekly via SD cards. Each field site operator was provided two labeled MicroSD cards for sensor units that they used to swap out each week. Data from the collected card was then read and processed off-site.

Data Correction Approach

SENSIT RAMP units were pre-configured by the manufacturer with a linear correction (i.e., concentration gain = 1.0 and offset = 7.0 ppbv). These presets reflect out-of-the box performance and were not modified by EPA prior or during testing.

After acquisition, the raw data was processed using the *sensortoolkit* python code library (v0.8.3b2). A continuous data set at the recorded sampling frequency was written to a .csv file. 1-hour and 24-hour averaged data sets were generated using a 75% completeness threshold and saved as separate .csv files. Outliers were **not** removed from data sets in order to assess "out-of-the-box" sensor performance.

The duration of the warm-up period required for sensor measurements to equilibrate was determined during bench-top testing (additional detail in pre-deployment observations) to be approximately 2 hours. Data recorded during warm up periods has been removed from data sets.

Issues Encountered

<u>Pre-deployment observations</u>

- *Changing logging interval*: SENSIT RAMP units were received without documentation or manuals. After communicating the need to change default settings (logging interval and time zone) with the manufacturer, a draft user's manual and a USB cable were supplied. With the use of this USB cable, instrument settings could be changed, and real-time data could be logged using a serial communication software (CoolTerm, v.1.5.0). Because the sensor did not record data at the top of every minute, the RAMP was configured to record data at 15-second intervals so that the data could be averaged more closely to complete minutes.
- *Gas Sensor Warmup*: Prior to deployment, RAMP units were collocated in a bench-top evaluation to verify operational status and determine the extent of data invalidity (i.e., determine equilibration period) after an initial start-up event. The recorded response for parameters measured by the RAMP suggests that the gas sensors (CO, NO, NO_2, O_3) required approximately a 2-hour equilibration period, while the remaining sensors (temperature, relative humidity, particulate matter) did not require any equilibration period.

<u>Field observations and sensor data flags</u>

The SENSIT RAMP unit was deployed at the OK monitoring site on 8/1/2019. The RAMP unit operated nominally during the testing period and did not require replacement or repair.

Sensor data flags included on the following page.

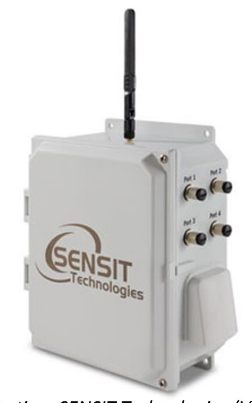

Image Attribution: SENSIT Technologies (Valparaiso, IN)

Field observations and sensor data flags

The following table contains data flags describing events that were encountered during the testing period. Throughout the testing period, it was observed that data collected from the RAMP unit were recorded in Daylight Saving time (DST) instead of Central Standard Time (CST), which is the time zone in which the site is located. During each visit to the monitoring site, a flag was reported indicating this timestamp configuration. Timestamps were subsequently converted from Central Daylight Time to Coordinated Universal Time (UTC) for analysis.

Start Time (UTC)	End Time (UTC)	Sensor Serial ID	Parameters Impacted	Flag
2019-08-20 16:15:00+00:00	2019-08-20 16:15:00+00:00	RAM_01	ALL	3.0-Intentional shutdown for data collection
2019-08-20 16:15:00+00:00	2019-08-28 16:10:00+00:00	RAM_01	ALL	17.0-Date/time set in DST instead of LST
2019-08-20 16:53:00+00:00	2019-08-20 17:33:00+00:00	RAM_01	ALL	5.0-Operator working near device (scheduled site visit)
2019-08-28 16:10:00+00:00	2019-08-28 16:10:00+00:00	RAM_01	ALL	3.0-Intentional shutdown for data collection
2019-08-28 16:10:00+00:00	2019-09-04 16:20:00+00:00	RAM_01	ALL	17.0-Date/Time in Daylight Savings instead of Standard
2019-08-28 16:46:00+00:00	2019-08-28 17:25:00+00:00	RAM_01	ALL	5.0-Operator working near device (scheduled site visit)
2019-09-04 16:20:00+00:00	2019-09-04 16:20:00+00:00	RAM_01	ALL	3.0-Intentional shutdown for data collection
2019-09-04 16:58:00+00:00	2019-09-04 17:37:00+00:00	RAM_01	ALL	5.0-Operator working near device (scheduled site visit)
2019-09-04 17:30:00+00:00	2019-09-11 17:25:00+00:00	RAM_01	ALL	17.0-Date/Time in Daylight Savings instead of Standard
2019-09-11 16:25:00+00:00	2019-09-11 16:25:00+00:00	RAM_01	ALL	3.0-Intentional shutdown for data collection
2019-09-11 16:58:00+00:00	2019-09-11 17:39:00+00:00	RAM_01	ALL	5.0-Operator working near device (scheduled site visit)
2019-09-11 17:31:00+00:00	2019-09-18 17:13:00+00:00	RAM_01	ALL	17.0-Date/Time in Daylight Savings instead of Standard

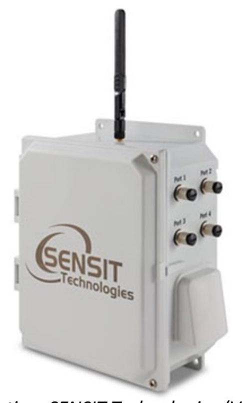

Image Attribution: SENSIT Technologies (Valparaiso, IN)

Supplemental Information: Description of FRM/FEM QC Checks and Data Flags

Description of Data Flags

AQS

The U.S. EPA's Air Quality System (AQS) is the Agency's primary ambient air monitoring data archive. A comprehensive list of data flags that are recorded alongside AQS data sets, referred to by U.S. EPA as 'qualifiers', can be found at the following link: https://aqs.epa.gov/aqsweb/documents/codetables/qualifiers.html

Invalidation of reference data: AQS qualifiers are organized by qualifier type, which indicates whether data logged alongside qualifier flags should be invalidated (set null). Qualifiers with type "Null Data Qualifier" are invalidated, and includes data logged during periods that coincide with QC checks (e.g., "BF-Precision/Zero/Span", "BJ- Operator Error", "BL - QA Audit", "AZ - QC Audit") among other events such as power outages. Data logged alongside qualifiers with type "Quality Assurance Qualifiers" are not invalidated and are included in this analysis (e.g., concentrations less than the federal MDL for the reference monitor "MD – Value less than MDL", QA reviewed values "Validated Value").

Data Flags Recorded During Testing

FRM/FEM Monitor	Timestamp (UTC)	Flag
Teledyne Advanced Pollution Instrumentation T400 (Acquired via AQS)	2019-08-16 13:00:00+0000 to 2019-08-16 15:00:00+0000	BF - Precision/Zero/Span
	2019-08-29 16:00:00+0000	BF - Precision/Zero/Span
	2019-09-13 15:00:00+0000	BF - Precision/Zero/Span

Testing Report - O_3 Base Testing

Aeroqual AQY

This report reflects out-of-the-box performance

Initial Base Testing - Milwaukee, WI

U.S. Environmental Protection Agency
Office of Research and Development
PI: Clements.Andrea@epa.gov
919-541-1363

July 2019—August 2019

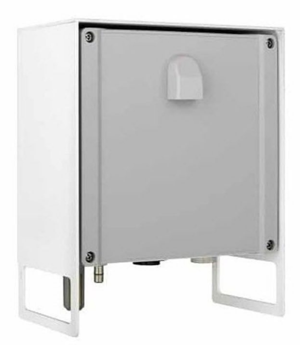

Image Attribution: Aeroqual Limited (Auckland, New Zealand)

Deployment Details

Testing Organization and Site Information	
Testing organization (Name, Organization type, Contact website)	U.S. Environmental Protection Agency - Office of Research and Development<br>Federal Government<br>Air Sensor Toolbox | U.S. EPA Website
Testing location (City, State, Latitude and Longitude)	WDNR Headquarters<br>Milwaukee, WI<br>43.061, -87.9135
AQS site ID	55 - 079 - 0026
Sampling timeframe (MM-DD-YY)	07-25-19 to 08-24-19
Sensor data source	Aeroqual Cloud
Reference data source	AQS API download

Sensor Information			
Manufacturer, model	Aeroqual AQY		
Device firmware version	1.14.2		
Sampling time interval	1-minute		
Sensor serial numbers	AQ_01		
Issues encountered during deployment?	☐	No Issues	

FRM/FEM Information	
Manufacturer, model, designation	Teledyne API T400
Sampling time interval	1-hour averaging
Date of calibration	As required by 40 CFR Part 58 and the Ozone SLAMS Network QAPP maintained by WDNR
Date of one-point QC check	Every two weeks as required by 40 CFR Part 58 Appendix A 3.1.1
Description, date(s) of maintenance activities	N/A

Time Series Plot: 1-hour averaged O_3

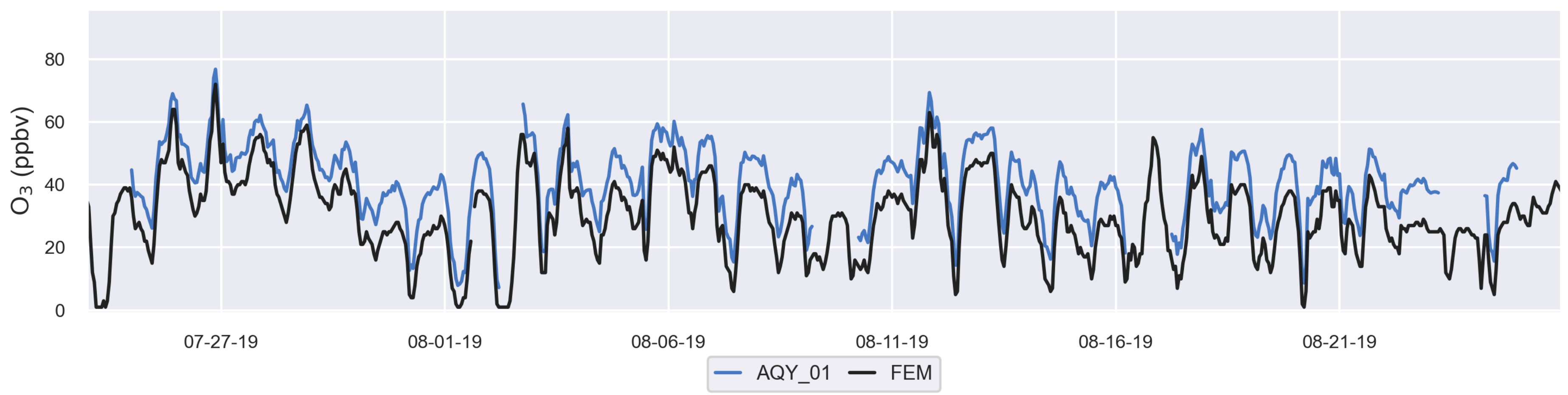

Range and average of FRM/FEM concentrations over duration of base test (ppbv)	[1-hr] 1.0-72.0, avg: 30.3, [Rolling 8-hr] 1.4-58.8, avg: 30.4
Number of 1-hr periods in FRM/FEM monitor measurements with a goal concentration ≥ 60 ppbv	7

Scatter Plot: Comparison to FRM/FEM

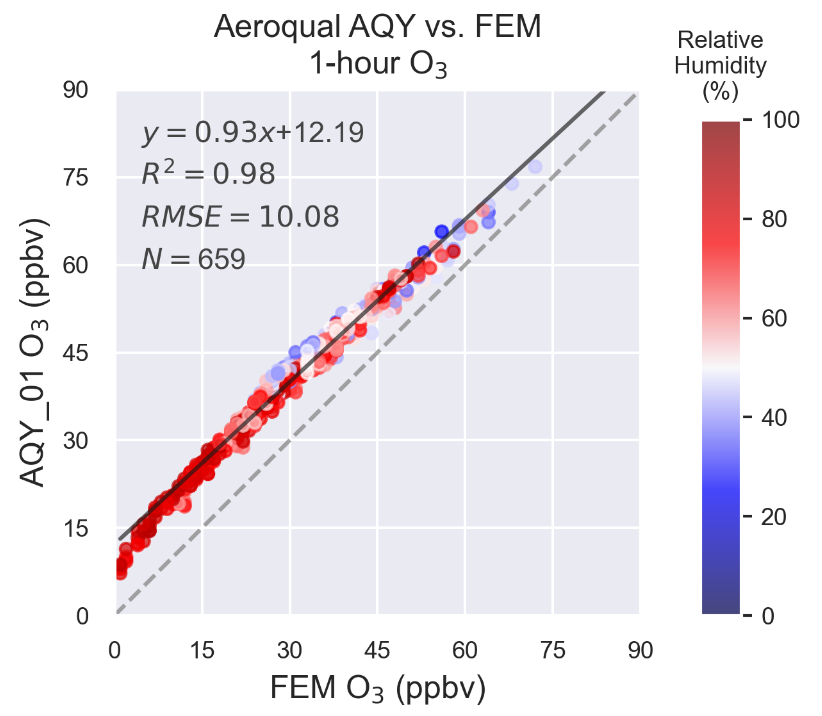

Performance Metrics

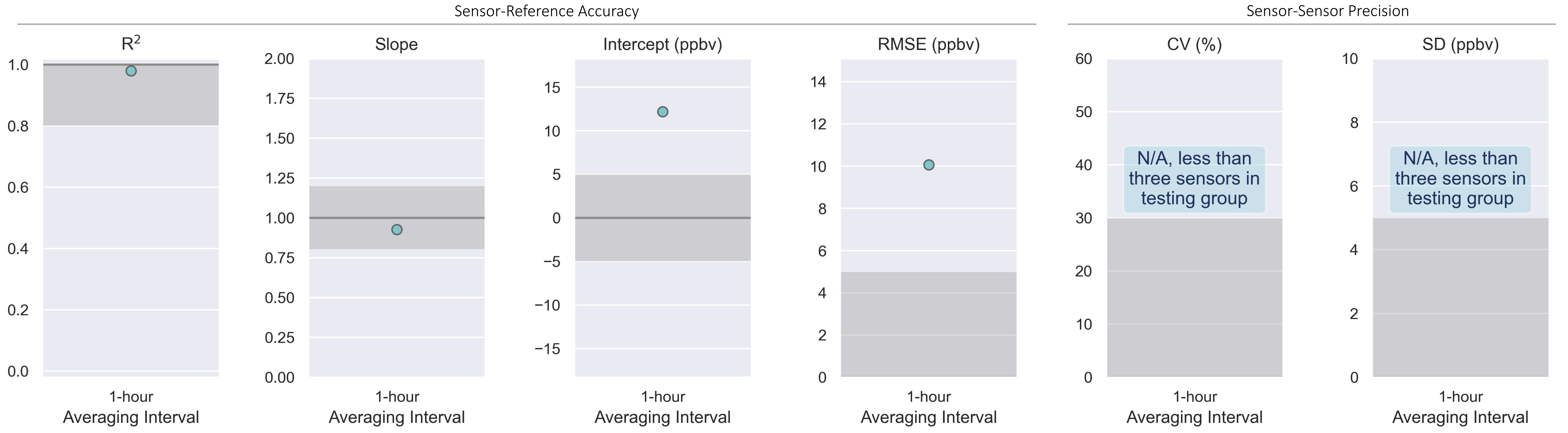

Meteorological Conditions During Deployment

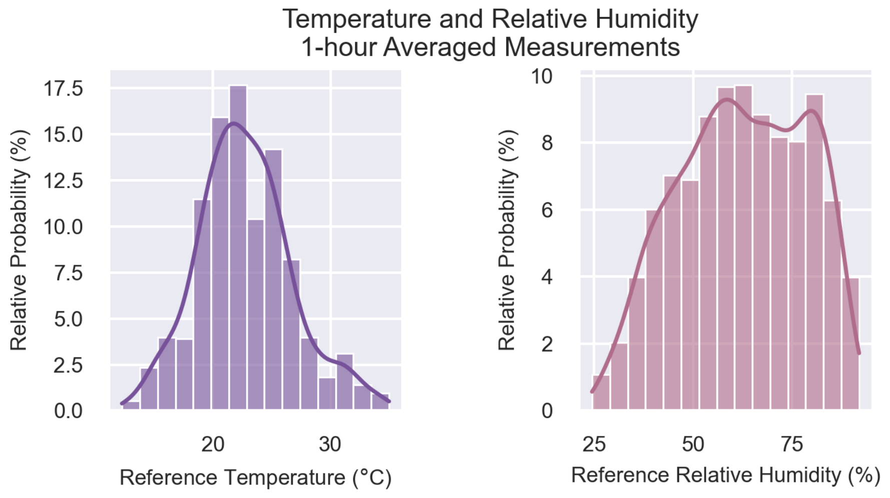

Number of 1-hr periods outside sensor manufacturer-listed temperature operational range (-10 to 40 °C)	0
Number of 1-hr periods outside sensor manufacturer-listed relative humidity operational range (no operational range specified)	-

Meteorological Influence

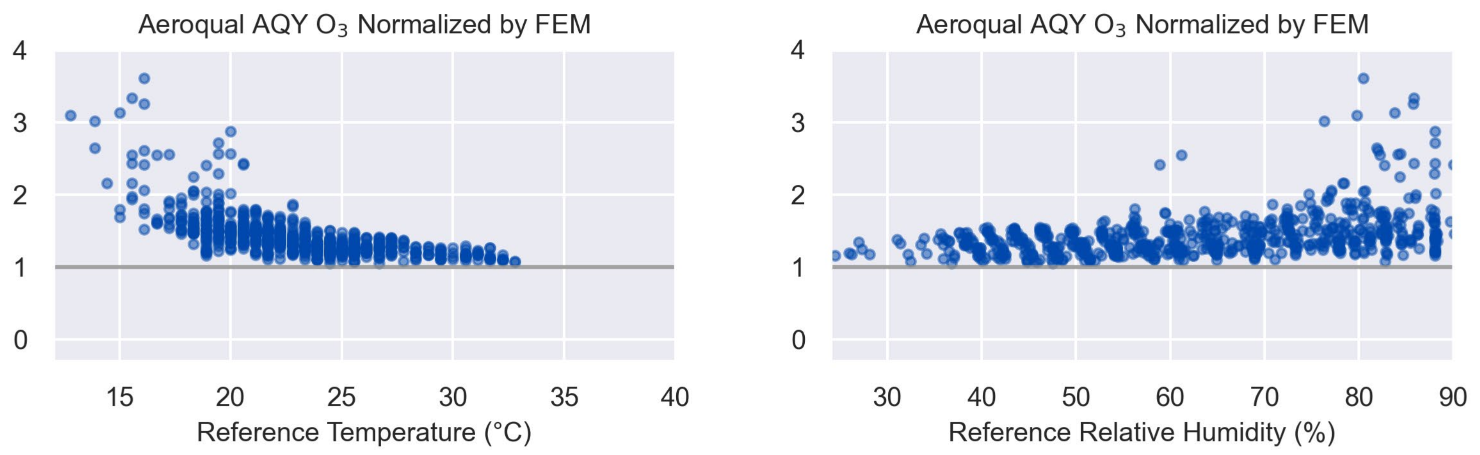

Mean number of paired, normalized concentration and temperature values (1-hr averages)	742
Mean number of paired, normalized concentration and relative humidity values (1-hr averages)	0

FEM data <5 ppbv (Federal MDL for the T400) has been remove (Meteorological Influence section only)

Testing Report - O_3 Base Testing

Aeroqual AQY

This report reflects out-of-the-box performance

Initial Base Testing - Milwaukee, WI
U.S. Environmental Protection Agency
Office of Research and Development
PI: Clements.Andrea@epa.gov
919-541-1363
July 2019—August 2019

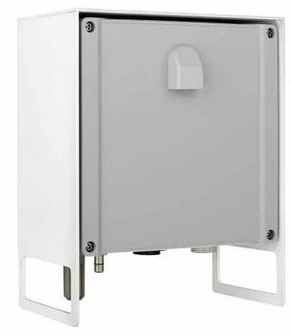
Image Attribution: Aeroqual Limited (Auckland, New Zealand)

Tabular Statistics

Sensor-FRM/FEM Correlation

	Bias and Linearity			Data Quality	
	R^2	Slope	Intercept (ppbv)	Uptime (%)	Number of paired sensor and reference concentration values*
	1-Hour ●	1-Hour ●	1-Hour ○	1-Hour ●	1-Hour
Metric Target Range	≥ 0.80	1.0 ± 0.20	-5 ≤ b ≤ 5	75%*	-
Sensor AQY_01	0.98	0.93	12.19	100	659

	Error
	RMSE (ppbv)
	1-Hour ☆
Metric Target Range	≤ 5.0
Deployment Value	10.1

Device-specific metrics (computed for each sensor in evaluation)
○ Metric value for none of devices tested falls within the target range
● Metric value for one of devices tested falls within the target range

Sensor-Sensor Precision[1]

	Precision (between collocated sensors)		Data Quality
	CV (%)	SD (ppbv)	Number of paired sensor and reference concentration values*
	1-Hour ☆	1-Hour ☆	1-Hour
Metric Target Range	≤ 30.0	≤ 5.0	-
Deployment Value	-	-	-

[1]Precision statistics are computed for evaluations with at least three collocated sensor units. Metric values are left blank for evaluations with two or fewer sensor units.

Single-valued metrics (computed via entire evaluation dataset)
☆ Indicates that the metric value is not within the target range
★ Indicates that the metric value is within the target range

*This value is only a recommendation for ensuring data quality and is not included in the list of target values discussed in Section 4 of the Performance Testing Protocols, Metrics, and Target Values for Ozone Air Sensors document.

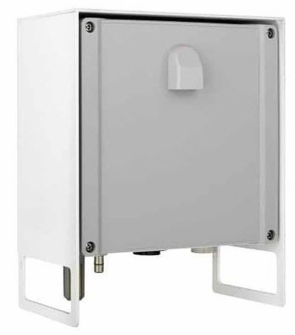

Image Attribution: Aeroqual Limited (Auckland, New Zealand)

Supplemental Information

Abbreviations used in Supplemental Information

FRM	Federal Reference Method
FEM	Federal Equivalent Method
SOP	Standard Operating Procedure
QAPP	Quality Assurance Project Plan
QC	Quality Control

Supplemental Documentation	Attached	Description & URL or file path to documentation
Field observations and sensor data flags	☑	See WI-AQY-Page 5 of this testing report
Maintenance logs	☐	No logs recorded during testing
Standard operating procedure(s)	☐	U.S. EPA Office Of Research and Development SOP available upon request
Photos of equipment setup and testing	☑	See WI-AQY-Page 4 of this testing report
Product specifications sheet(s)	☑	See Appendix C, “Spec_Sheet_Aeroqual_AQY.pdf”*
Product manual(s)	☑	See Appendix C, “Manual_Aeroqual_AQY.pdf”*
Data storage and transmission method	☑	See WI-AQY-Page 5 of this testing report
Data correction approach	☑	See WI-AQY-Page 5 of this testing report
Issues encountered	☑	See WI-AQY-Page 5 of this testing report
Data analysis/correction scripts and version	☑	Averaging and processing of data, calculation of performance metrics, and generation of figures and other supplementary material for analysis were obtained using Python 3.9.7 with the packages sensortoolkit v0.8.3b2, pandas 1.3.5, NumPy 1.21.2, Matplotlib 3.5.0, statsmodels 0.13.0, and seaborn 0.11.2. All packages are available from the Python Package Index (PyPI) at https://pypi.org. The integrated development environment (IDE) Spyder 5.1.5 was used for scripting and data visualization. Version control for the Python base, packages, and IDE were all managed by conda 4.11.0.
Air Monitoring Station QAPP	☐	U.S. EPA Office Of Research and Development QAPP available upon request
Summary of FRM/FEM monitor QC checks	☑	See WI-AQY-Page 6 of this testing report
Manufacturer website for FRM/FEM monitor	☑	Teledyne API: Model T400 Product website
FRM/FEM monitor manual	☑	See Appendix B, “Spec_Sheet_TeledyneAPI_T400.pdf”
FRM/FEM monitor specifications sheet(s)	☑	See Appendix B, “Manual_TeledyneAPI_T400.pdf”
Other documents	☑	Manufacturer notice of AQY sales on hold

*As of 3/18/2021, the manufacturer of the AQY has placed sales of a similar unit on hold. Documentation for the AQY is currently unavailable from the manufacturer’s website.

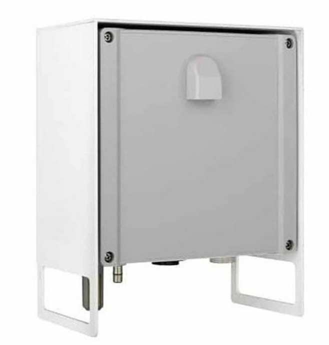

Image Attribution: Aeroqual Limited (Auckland, New Zealand)

Supplemental Information: Photos of Testing Site and Equipment Setup

Site Description:

The urban site was located in the parking lot at the former WDNR Southeast Regional Headquarters located at 2300 N. Martin Luther King Blvd. Sample inlets are roughly 5 meters above ground level and 50 meters from the nearest road. Verified through annual WDNR audits, the site meets the requirements of 40 CFT 58, Appendices C, D, E and G.

Figure 1: Aeroqual AQY sensor (indicated by red arrow) attached to metal railing atop the sampling shelter at the monitoring site.

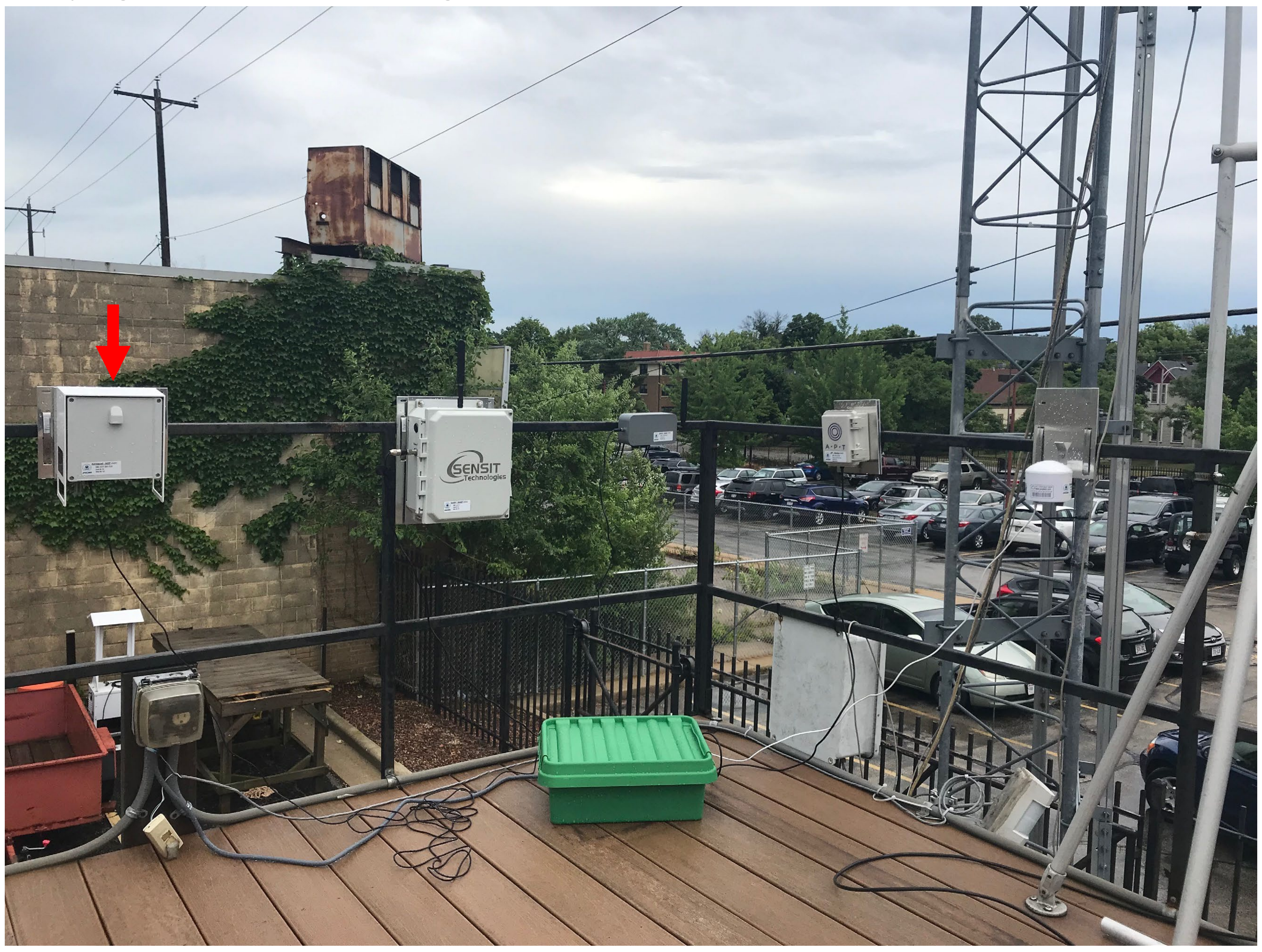

Figure 2: WDNR Monitoring Station

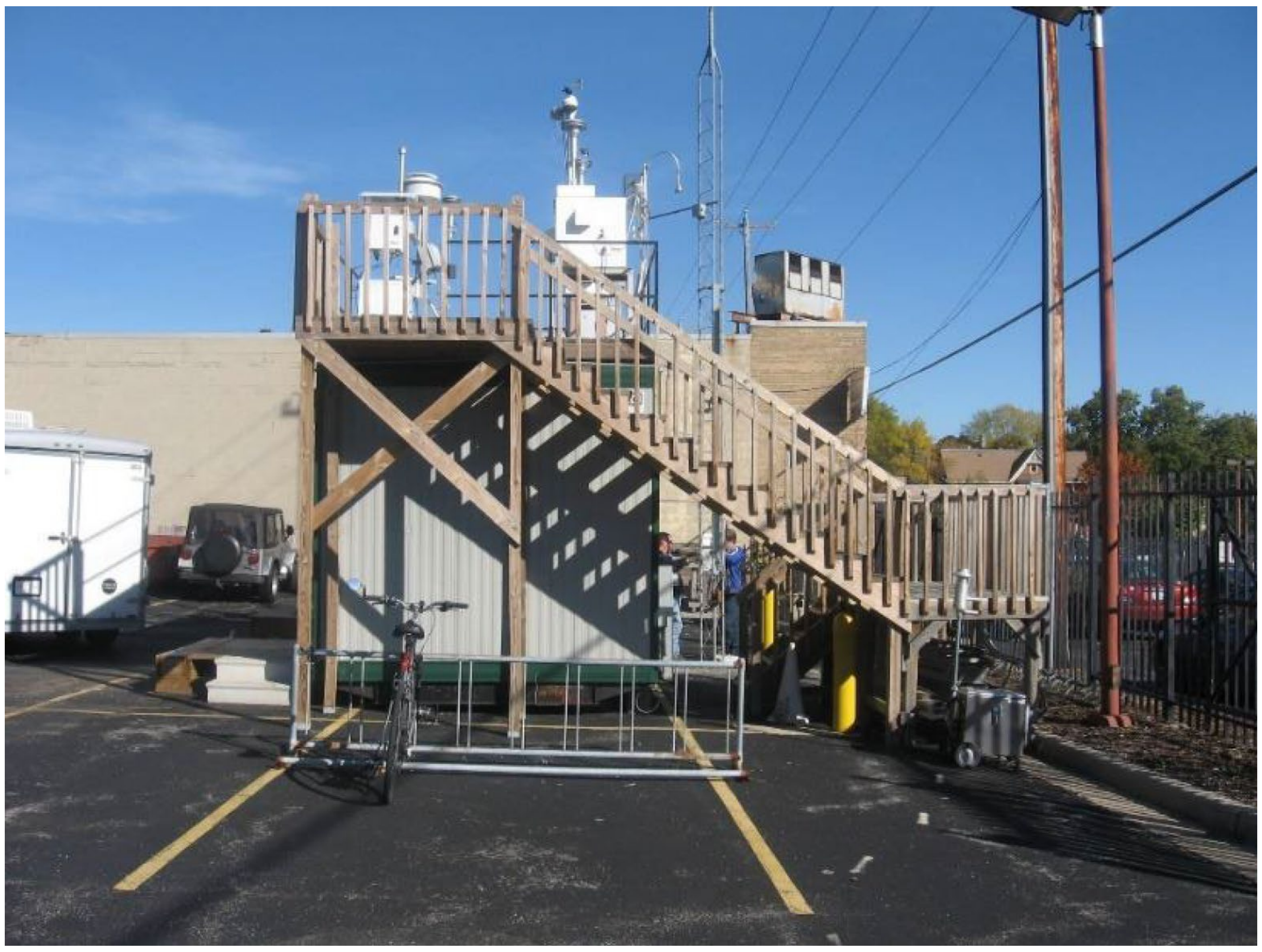

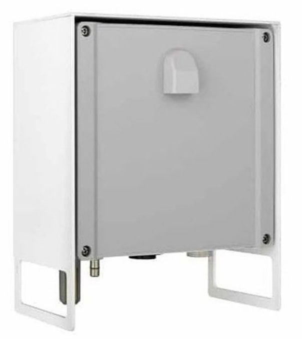

Image Attribution: Aeroqual Limited (Auckland, New Zealand)

Supplemental Information: Data Storage, Correction Approach, and Issues Encountered

Data Storage and Transmission Method

As part of CRADA #934-16 between Aeroqual and US EPA, Aeroqual supported data streaming. SIM cards were installed and data flowed to the Aeroqual Cloud. The 1-minute raw data was acquired weekly using the Aeroqual Cloud (*last accessed 5/11/22*) user interface (UI). The AQY has an internal data storage USB flash drive as a data backup, however access requires software proprietary to Aeroqual.

Data Correction Approach

This evaluation report reflects “out-of-the-box” performance of the AQY. The manufacturer provides a procedure by which local collocation (sensor operated along side an FRM/FEM) data can be collected, a gain (slope) and offset (intercept) determined, and parameters entered into the Aeroqual Cloud user interface to be applied to all subsequently collected data. This procedure and feature was **not** used prior to this evaluation. Prospective consumers may get different performance from this device if they utilize this feature.

After acquisition, the raw data was processed using the *sensortoolkit* python code library (v0.8.3b2). A continuous data set at the recorded sampling frequency was written to a .csv file. 1-hour averaged data sets were generated using a 75% completeness threshold and saved as separate .csv files. Outliers were **not** removed from data sets in order to assess “out-of-the-box” sensor performance.

The duration of the warm-up period required for sensor measurements to equilibrate was determined from field data to be 10 minutes. Warm up periods were considered to occur following any power outage to sensor units, either due to routine field visits or unscheduled site power outages. Data recorded during warm up periods has been removed from data sets.

Issues Encountered

Pre-deployment observations

- *Timestamp inaccuracies*: During pre-deployment, the AQY devices did not properly sync timestamps with the onboard Real-Time Clock. Connecting the units to the internet by cellular or Wi-Fi allowed the unit to sync with internet time and resulted in proper timestamps.

Field observations and sensor data flags

The Aeroqual AQY was deployed at the WDNR monitoring site on 7/22/2019. The AQY unit operated nominally during the testing period and did not require replacement or repair.

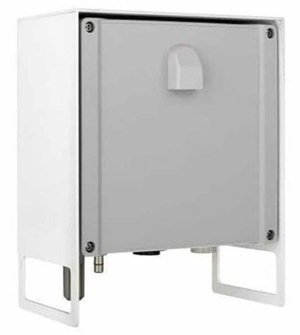

Image Attribution: Aeroqual Limited (Auckland, New Zealand)

Supplemental Information: Description of FRM/FEM QC Checks and Data Flags

Description of Data Flags

AQS

The U.S. EPA's Air Quality System (AQS) is the Agency's primary ambient air monitoring data archive. A comprehensive list of data flags that are recorded alongside AQS data sets, referred to by U.S. EPA as 'qualifiers', can be found at the following link: https://aqs.epa.gov/aqsweb/documents/codetables/qualifiers.html

Invalidation of reference data: AQS qualifiers are organized by qualifier type, which indicates whether data logged alongside qualifier flags should be invalidated (set null). Qualifiers with type "Null Data Qualifier" are invalidated, and includes data logged during periods that coincide with QC checks (e.g., "BF-Precision/Zero/Span", "BJ- Operator Error", "BL - QA Audit", "AZ - QC Audit") among other events such as power outages. Data logged alongside qualifiers with type "Quality Assurance Qualifiers" are not invalidated and are included in this analysis (e.g., concentrations less than the federal MDL for the reference monitor "MD – Value less than MDL", QA reviewed values "Validated Value").

Data Flags Recorded During Testing

FRM/FEM Monitor	Timestamp (UTC)	Flag
Teledyne Advanced Pollution Instrumentation T400<br>(Data acquired from AQS)	2019-08-01 15:00:00+0000	BF - Precision/Zero/Span

Meteorological Instrument	Timestamp (UTC)	Flag
MetOne Temperature Monitor<br>(Data acquired from AQS)	2019-08-07 16:00:00+0000 to 2019-08-07 17:00:00+0000	AZ - QC Audit

Testing Report - O_3 Base Testing

SENSIT RAMP

This report reflects out-of-the-box performance

Initial Base Testing - Milwaukee, WI

U.S. Environmental Protection Agency

Office of Research and Development

PI: Clements.Andrea@epa.gov

919-541-1363

August 2019

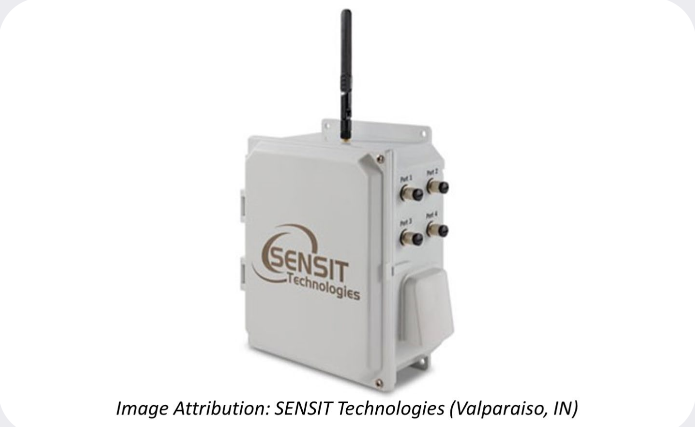

Image Attribution: SENSIT Technologies (Valparaiso, IN)

Deployment Details

Testing Organization and Site Information	
Testing organization (Name, Organization type, Contact website)	U.S. Environmental Protection Agency - Office of Research and Development<br>Federal Government<br>Air Sensor Toolbox | U.S. EPA Website
Testing location (City, State, Latitude and Longitude)	WDNR Headquarters<br>Milwaukee, WI<br>43.061, -87.9135
AQS site ID	55 - 079 - 0026
Sampling timeframe (MM-DD-YY)	08-01-19 to 08-31-19
Sensor data source	Onboard MicroSD card
Reference data source	AQS API download

Sensor Information			
Manufacturer, model	SENSIT RAMP		
Device firmware version	190308_AQ_v9.30		
Sampling time interval	15-seconds		
Sensor serial numbers	RAM_01		
Issues encountered during deployment?	☐	No Issues	

FRM/FEM Information	
Manufacturer, model, designation	Teledyne API T400
Sampling time interval	1-hour averaging
Date of calibration	As required by 40 CFR Part 58 and the Ozone SLAMS Network QAPP maintained by WDNR
Date of one-point QC check	Every two weeks as required by 40 CFR Part 58 Appendix A 3.1.1
Description, date(s) of maintenance activities	N/A

Time Series Plot: 1-hour averaged O_3

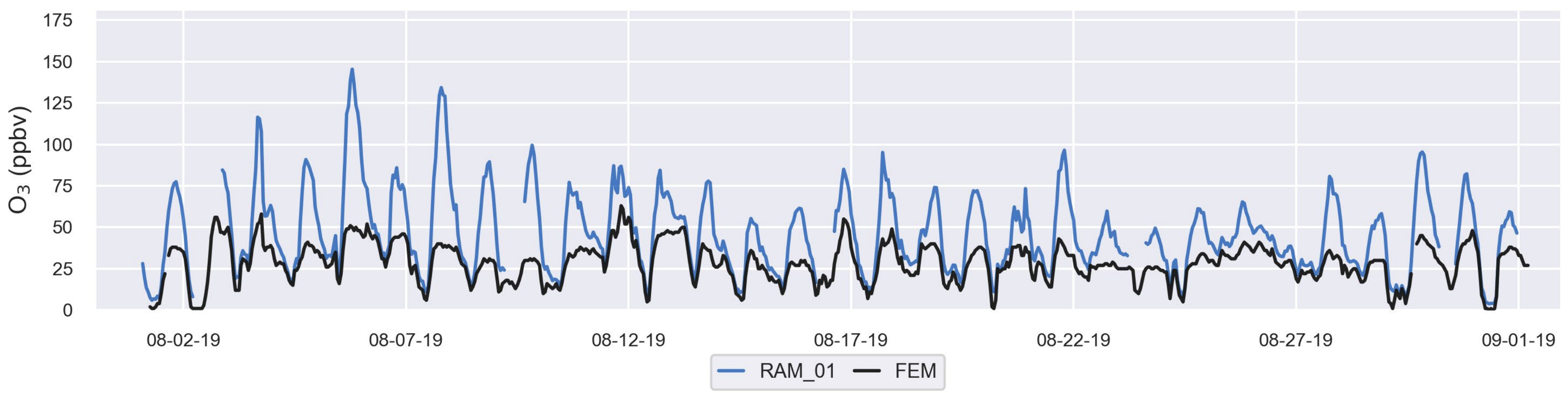

Range and average of FRM/FEM concentrations over duration of base test (ppbv)	[1-hr] 0.0-63.0, avg: 28.3,<br>[Rolling 8-hr] 1.4-55.0, avg: 28.5
Number of 1-hr periods in FRM/FEM monitor measurements with a goal concentration ≥ 60 ppbv	2

Scatter Plot: Comparison to FRM/FEM

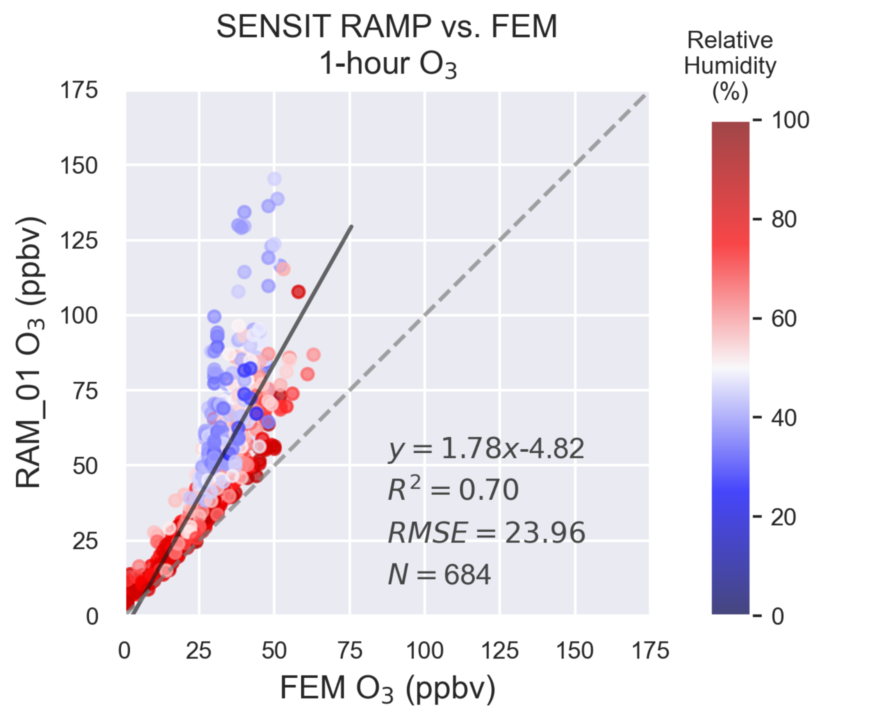

Performance Metrics

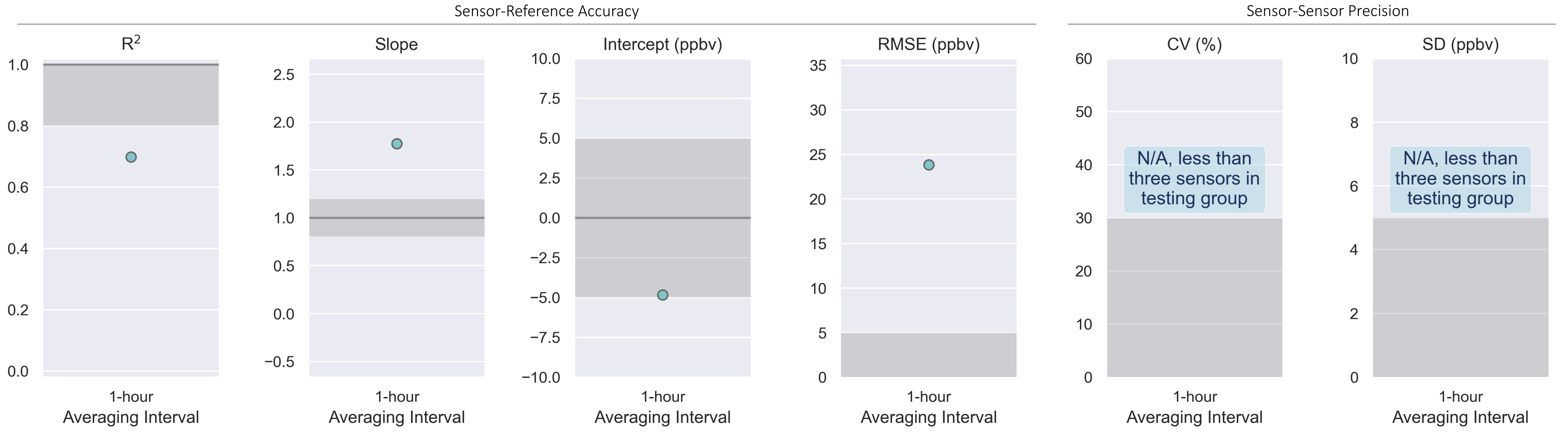

Meteorological Conditions During Deployment

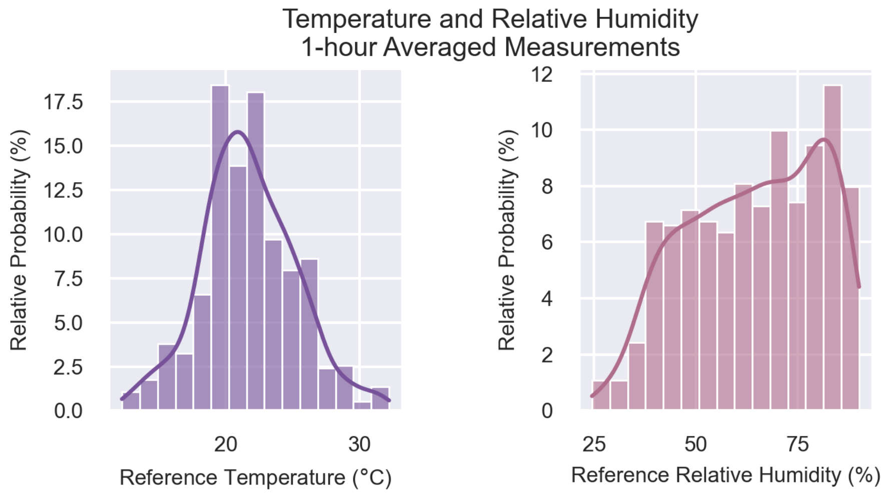

Number of 1-hr periods outside sensor manufacturer-listed temperature operational range (-20 to 50 °C)	0
Number of 1-hr periods outside sensor manufacturer-listed relative humidity operational range (no operational range specified)	-

Meteorological Influence

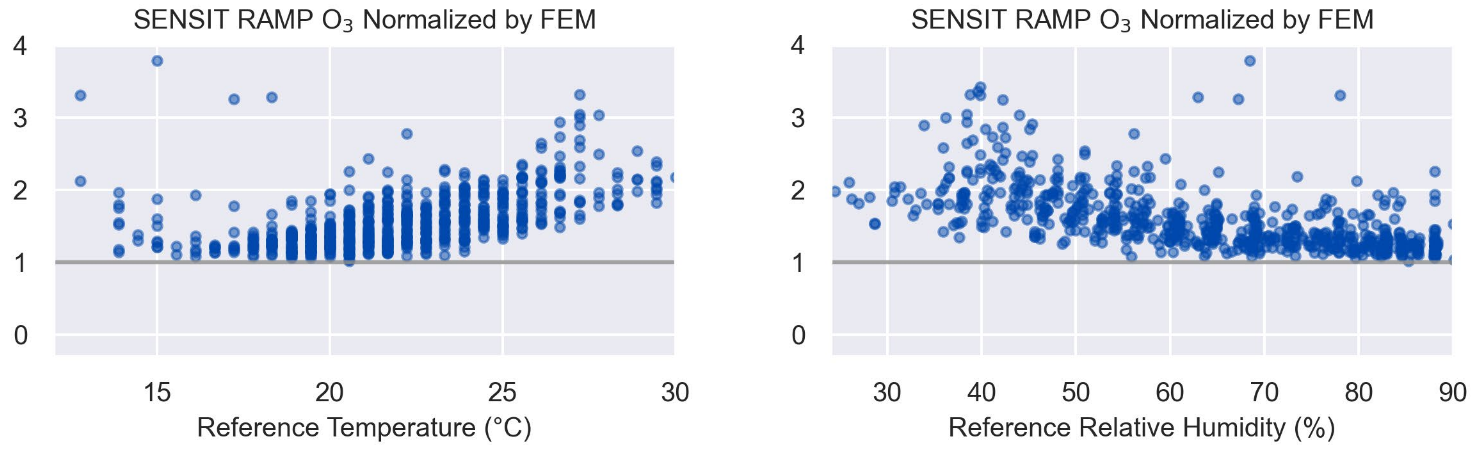

Mean number of paired, normalized concentration and temperature values (1-hr averages)	736
Mean number of paired, normalized concentration and relative humidity values (1-hr averages)	0

FEM data <5 ppbv (Federal MDL for the T400) has been remove (Meteorological Influence section only)

Testing Report - O_3 Base Testing

SENSIT RAMP

This report reflects out-of-the-box performance

Initial Base Testing - Milwaukee, WI

U.S. Environmental Protection Agency
Office of Research and Development
PI: Clements.Andrea@epa.gov
919-541-1363
August 2019

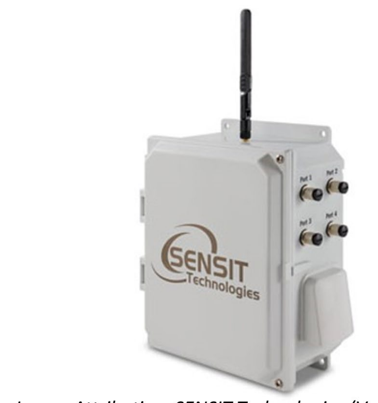

Image Attribution: SENSIT Technologies (Valparaiso, IN)

Tabular Statistics

Sensor-FRM/FEM Correlation

	Bias and Linearity			Data Quality	
	R^2	Slope	Intercept (ppbv)	Uptime (%)	Number of paired sensor and reference concentration values*
	1-Hour ○	1-Hour ○	1-Hour ○	1-Hour ●	1-Hour
Metric Target Range	≥ 0.80	1.0 ± 0.20	-5 ≤ b ≤ 5	75%*	-
Sensor RAM_01	0.71	1.79	-5.25	95	697

	Error
	RMSE (ppbv)
	1-Hour ☆
Metric Target Range	≤ 5.0
Deployment Value	24.0

Device-specific metrics (computed for each sensor in evaluation)
- ○ Metric value for none of devices tested falls within the target range
- ● Metric value for one of devices tested falls within the target range

Sensor-Sensor Precision[1]

	Precision (between collocated sensors)		Data Quality
	CV (%)	SD (ppbv)	Number of paired sensor and reference concentration values*
	1-Hour ☆	1-Hour ☆	1-Hour
Metric Target Range	≤ 30.0	≤ 5.0	-
Deployment Value	-	-	-

[1]Precision statistics are computed for evaluations with at least three collocated sensor units. Metric values are left blank for evaluations with two or fewer sensor units.

Single-valued metrics (computed via entire evaluation dataset)
- ☆ Indicates that the metric value is not within the target range
- ★ Indicates that the metric value is within the target range

*This value is only a recommendation for ensuring data quality and is not included in the list of target values discussed in Section 4 of the Performance Testing Protocols, Metrics, and Target Values for Ozone Air Sensors document.

U.S. Environmental Protection Agency
Office of Research and Development
PI: Clements.Andrea@epa.gov
919-541-1363

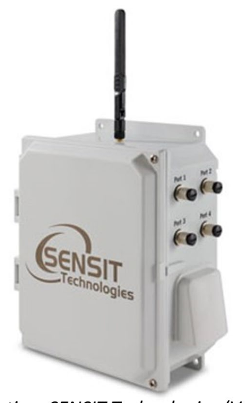

Image Attribution: SENSIT Technologies (Valparaiso, IN)

Supplemental Information

Abbreviations used in Supplemental Information

FRM	Federal Reference Method
FEM	Federal Equivalent Method
SOP	Standard Operating Procedure
QAPP	Quality Assurance Project Plan
QC	Quality Control

Supplemental Documentation	Attached	Description & URL or file path to documentation
Field observations and sensor data flags	☑	See WI-RAM-Page 5 of this testing report
Maintenance logs	☐	No logs recorded during testing
Standard operating procedure(s)	☐	U.S. EPA Office Of Research and Development SOP available upon request
Photos of equipment setup and testing	☑	See WI-RAM-Page 4 of this testing report
Product specifications sheet(s)	☑	See Appendix C, “Spec_Sheet_SENSIT_RAMP.pdf”
Product manual(s)	☑	See Appendix C, “Manual_SENSIT_RAMP.pdf”
Data storage and transmission method	☑	See WI-RAM-Page 5 of this testing report
Data correction approach	☑	See WI-RAM-Page 5 of this testing report
Issues encountered	☑	See WI-RAM-Page 5 of this testing report
Data analysis/correction scripts and version	☑	Averaging and processing of data, calculation of performance metrics, and generation of figures and other supplementary material for analysis were obtained using Python 3.9.7 with the packages sensortoolkit v0.8.3b2, pandas 1.3.5, NumPy 1.21.2, Matplotlib 3.5.0, statsmodels 0.13.0, and seaborn 0.11.2. All packages are available from the Python Package Index (PyPI) at https://pypi.org. The integrated development environment (IDE) Spyder 5.1.5 was used for scripting and data visualization. Version control for the Python base, packages, and IDE were all managed by conda 4.11.0.
Air Monitoring Station QAPP	☐	U.S. EPA Office Of Research and Development QAPP available upon request
Summary of FRM/FEM monitor QC checks	☑	See WI-RAM-Page 6 of this testing report
Manufacturer website for FRM/FEM monitor	☑	Teledyne API: Model T400 Product website
FRM/FEM monitor manual	☑	See Appendix B, “Spec_Sheet_TeledyneAPI_T400.pdf”
FRM/FEM monitor specifications sheet(s)	☑	See Appendix B, “Manual_TeledyneAPI_T400.pdf”
Other documents	☐	

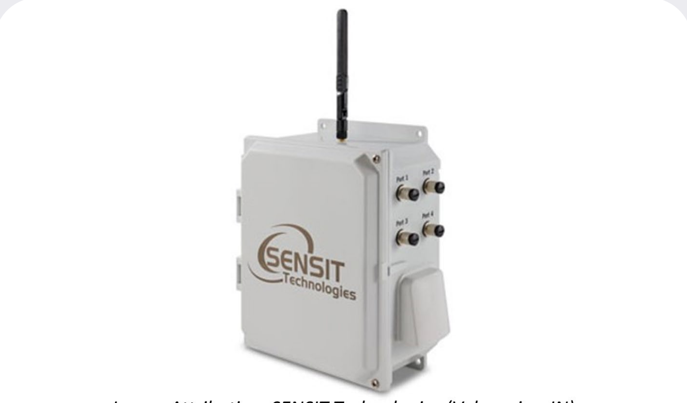

Image Attribution: SENSIT Technologies (Valparaiso, IN)

Supplemental Information: Photos of Testing Site and Equipment Setup

Site Description:

The urban site was located in the parking lot at the former WDNR Southeast Regional Headquarters located at 2300 N. Martin Luther King Blvd. Sample inlets are roughly 5 meters above ground level and 50 meters from the nearest road. Verified through annual WDNR audits, the site meets the requirements of 40 CFT 58, Appendices C, D, E and G.

Figure 1: SENSIT RAMP sensor (indicated by red arrow) attached to metal railing atop the sampling shelter at the monitoring site.

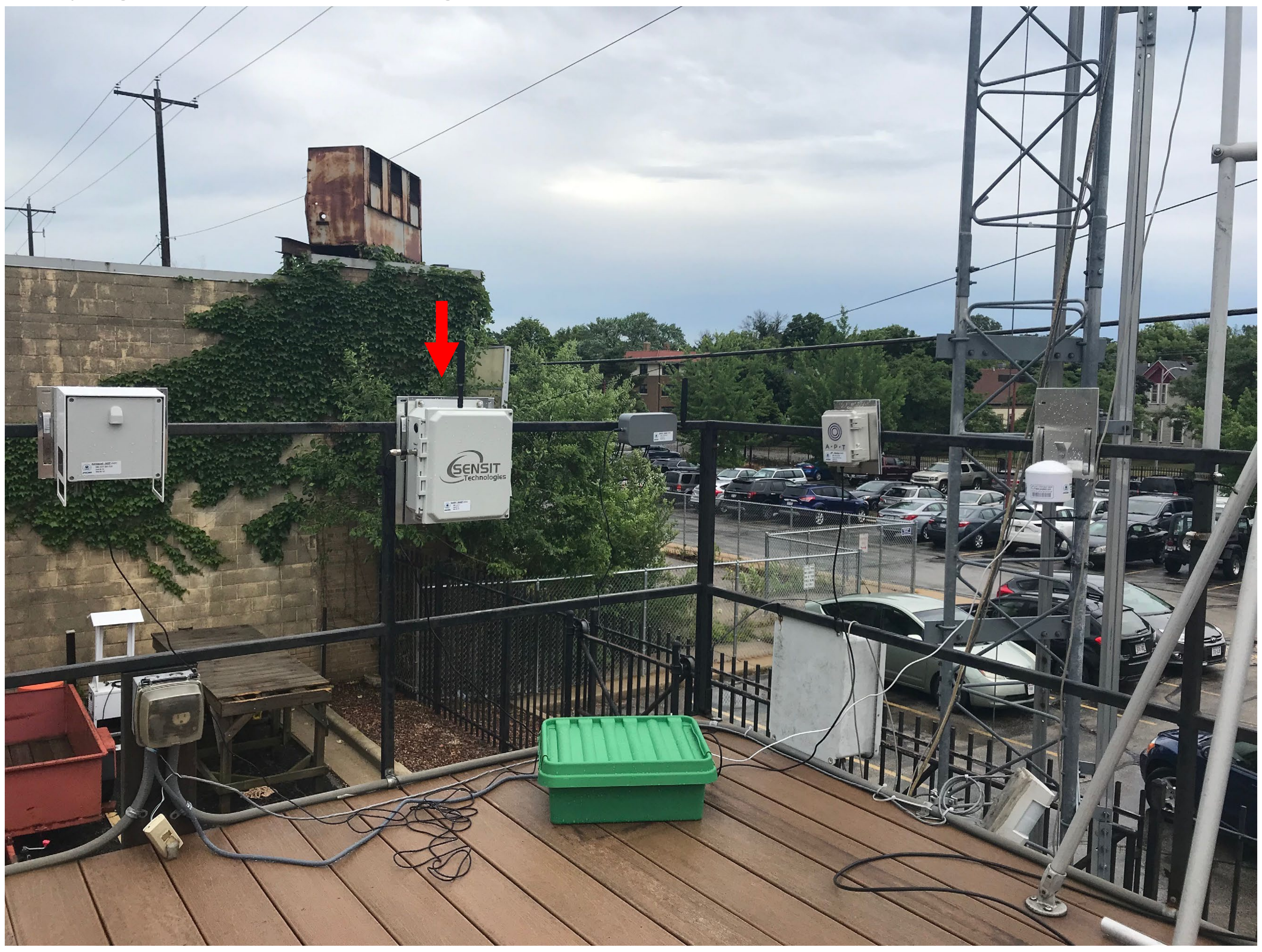

Figure 2: WDNR Monitoring Station

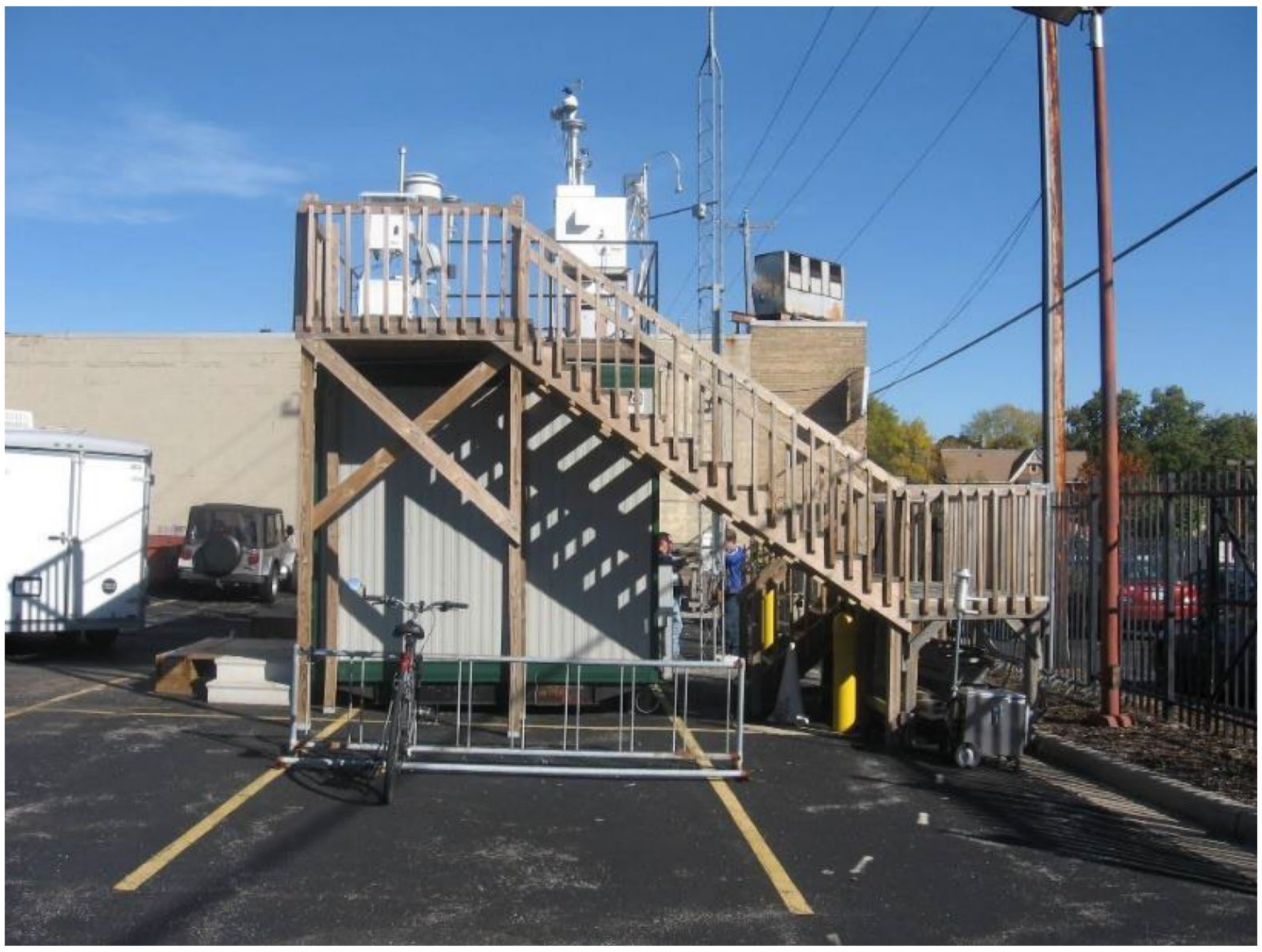

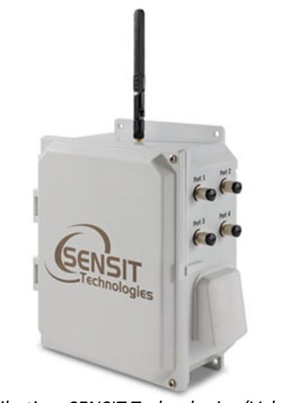
Image Attribution: SENSIT Technologies (Valparaiso, IN)

Supplemental Information: Data Storage, Correction Approach, and Issues Encountered

Data Storage and Transmission Method

The SENSIT RAMP was configured to record data at a 15-second sampling interval. Data are stored as daily text files (.txt format) on an onboard MicroSD card. Data files were obtained weekly via SD cards. Each field site operator was provided two labeled MicroSD cards for sensor units that they used to swap out each week. Data from the collected card was then read and processed off-site.

Data Correction Approach

SENSIT RAMP units were pre-configured by the manufacturer with a linear correction (i.e., concentration gain = 1.0 and offset = 1.0 ppbv). The linear correction was specific to each RAMP and the manufacturer did not set each gain and offset identically. These presets reflect out-of-the box performance and were not modified by EPA prior or during testing.

After acquisition, the raw data was processed using the *sensortoolkit* python code library (v0.8.3b2). A continuous data set at the recorded sampling frequency was written to a .csv file. 1-hour and 24-hour averaged data sets were generated using a 75% completeness threshold and saved as separate .csv files. Outliers were **not** removed from data sets in order to assess "out-of-the-box" sensor performance.

The duration of the warm-up period required for sensor measurements to equilibrate was determined during bench-top testing (additional detail in pre-deployment observations) to be approximately 2 hours. Data recorded during warm up periods has been removed from data sets.

Issues Encountered

Pre-deployment observations

- *Changing logging interval*: SENSIT RAMP units were received without documentation or manuals. After communicating the need to change default settings (logging interval and time zone) with the manufacturer, a draft user's manual and a USB cable were supplied. With the use of this USB cable, instrument settings could be changed, and real-time data could be logged using a serial communication software (CoolTerm, v.1.5.0). Because the sensor did not record data at the top of every minute, the RAMP was configured to record data at 15-second intervals so that the data could be averaged more closely to complete minutes.
- *Gas Sensor Warmup*: Prior to deployment, RAMP units were collocated in a bench-top evaluation to verify operational status and determine the extent of data invalidity (i.e., determine equilibration period) after an initial start-up event. The recorded response for parameters measured by the RAMP suggests that the gas sensors (CO, NO, NO_2, O_3) required approximately a 2-hour equilibration period, while the remaining sensors (temperature, relative humidity, particulate matter) did not require any equilibration period.

Field observations and sensor data flags

The following table contains data flags describing events that were encountered during the testing period. On 8/2/2019, a sampling interval abnormality was flagged and was associated with a brief period in which the sampling frequency changed from a constant, consecutive 15-second interval to a longer interval. For three samples, the interval between recorded values ranged from roughly 2 minutes to over 7 minutes. This event lasted less than 15 minutes, and following these abnormal intervals, the sampling frequency resumed the configured 15-second interval.

Start Time (UTC)	End Time (UTC)	Sensor Serial ID	Parameters Impacted	Flag
2019-08-02 17:27:00+00:00	2019-08-02 17:34:00+00:00	RAM_01	ALL	6.0-Sampling interval abnormality

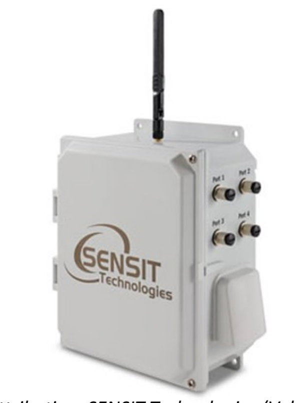

Image Attribution: SENSIT Technologies (Valparaiso, IN)

Supplemental Information: Description of FRM/FEM QC Checks and Data Flags

Description of Data Flags

AQS

The U.S. EPA's Air Quality System (AQS) is the Agency's primary ambient air monitoring data archive. A comprehensive list of data flags that are recorded alongside AQS data sets, referred to by U.S. EPA as 'qualifiers', can be found at the following link: https://aqs.epa.gov/aqsweb/documents/codetables/qualifiers.html

Invalidation of reference data: AQS qualifiers are organized by qualifier type, which indicates whether data logged alongside qualifier flags should be invalidated (set null). Qualifiers with type "Null Data Qualifier" are invalidated, and includes data logged during periods that coincide with QC checks (e.g., "BF-Precision/Zero/Span", "BJ- Operator Error", "BL - QA Audit", "AZ - QC Audit") among other events such as power outages. Data logged alongside qualifiers with type "Quality Assurance Qualifiers" are not invalidated and are included in this analysis (e.g., concentrations less than the federal MDL for the reference monitor "MD – Value less than MDL", QA reviewed values "Validated Value").

Data Flags Recorded During Testing

FRM/FEM Monitor	Timestamp (UTC)	Flag
Teledyne Advanced Pollution Instrumentation T400 (Data acquired from AQS)	2019-08-01 15:00:00+0000	BF - Precision/Zero/Span
	2019-08-29 15:00:00+0000 to 2019-08-29 16:00:00+0000	BF - Precision/Zero/Span

Meteorological Instrument	Timestamp (UTC)	Flag
MetOne Temperature Monitor (Data acquired from AQS)	2019-08-07 16:00:00+0000 to 2019-08-07 17:00:00+0000	AZ - QC Audit

Testing Report - O_3 Base Testing

Apis APM01

This report reflects out-of-the-box performance

Initial Base Testing - RTP, NC

U.S. Environmental Protection Agency
Office of Research and Development
PI: Clements.Andrea@epa.gov
919-541-1363

July 2020—August 2020

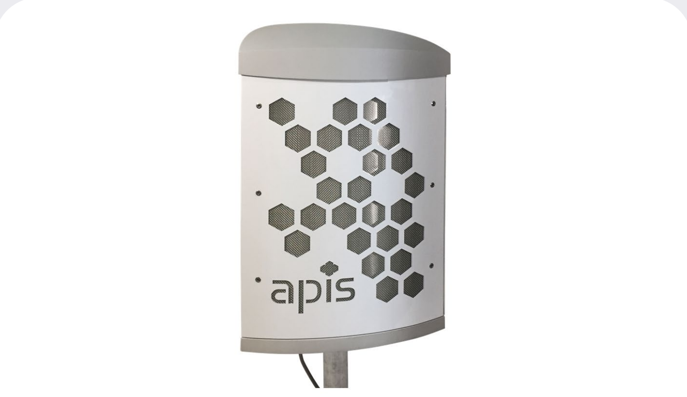

Image Attribution: Apis, Inc (Gants Pass, OR)

Deployment Details

Testing Organization and Site Information	
Testing organization (Name, Organization type, Contact website)	U.S. Environmental Protection Agency - Office of Research and Development<br>Federal Government<br>Air Sensor Toolbox | U.S. EPA Website
Testing location (City, State, Latitude and Longitude)	Ambient Monitoring Innovative Research Station (AIRS)<br>RTP, NC<br>35.88951, -78.874572
AQS site ID	37 – 063 – 0099
Sampling timeframe (MM-DD-YY)	07-11-20 to 08-10-20
Sensor data source	
Reference data source	OAQPS file transfer

Sensor Information			
Manufacturer, model	Apis APM01		
Device firmware version			
Sampling time interval	5-minutes		
Sensor serial numbers	1022	1026	1031
Issues encountered during deployment?	☐	No Issues	

FRM/FEM Information	
Manufacturer, model, designation	Teledyne API T265 FEM
Sampling time interval	1-hour averaging
Date of calibration	As required by 40 CFR Part 58 and the Burdens Creek QAPP maintained by OAQPS
Date of one-point QC check	Every two weeks as required by 40 CFR Part 58 Appendix A 3.1.1
Description, date(s) of maintenance activities	N/A

Time Series Plot: 1-hour averaged O_3

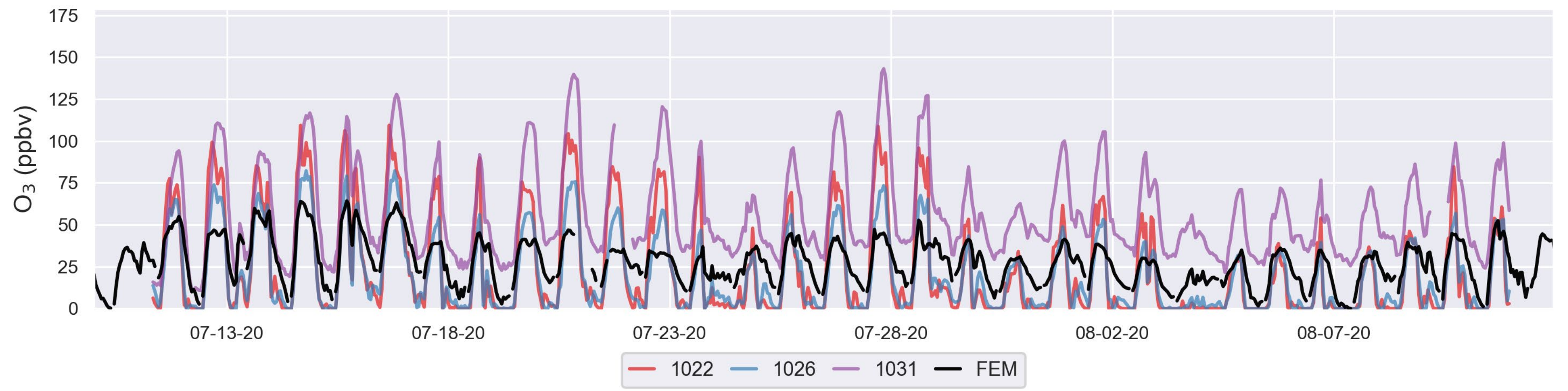

Range and average of FRM/FEM concentrations over duration of base test (ppbv)	[1-hr] 0.1-64.2, avg: 27.7,<br>[Rolling 8-hr] 1.6-59.3, avg: 30.9
Number of 1-hr periods in FRM/FEM monitor measurements with a goal concentration ≥ 60 ppbv	7

Scatter Plot: Comparison to FRM/FEM

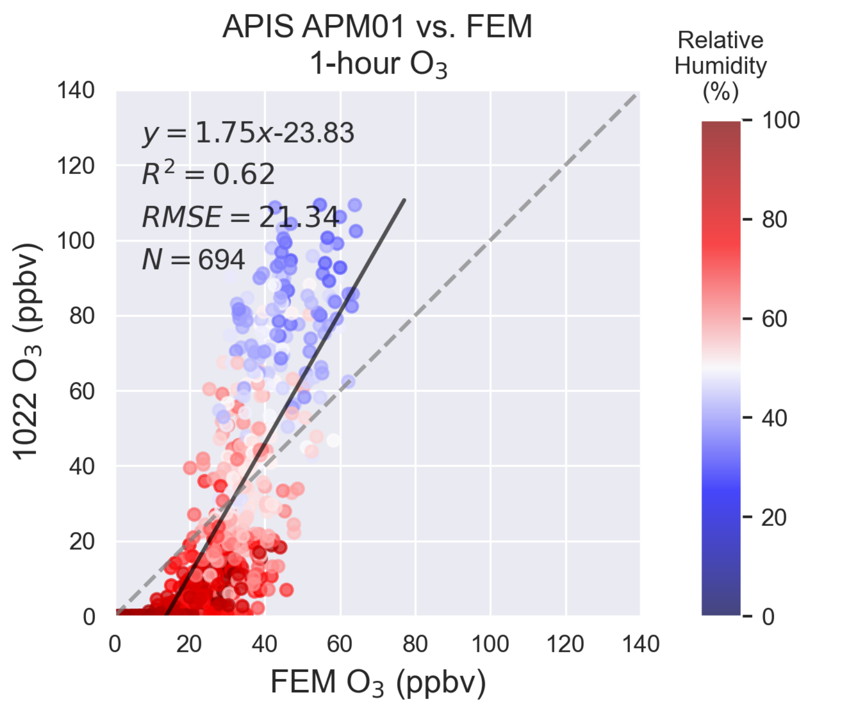

Performance Metrics★

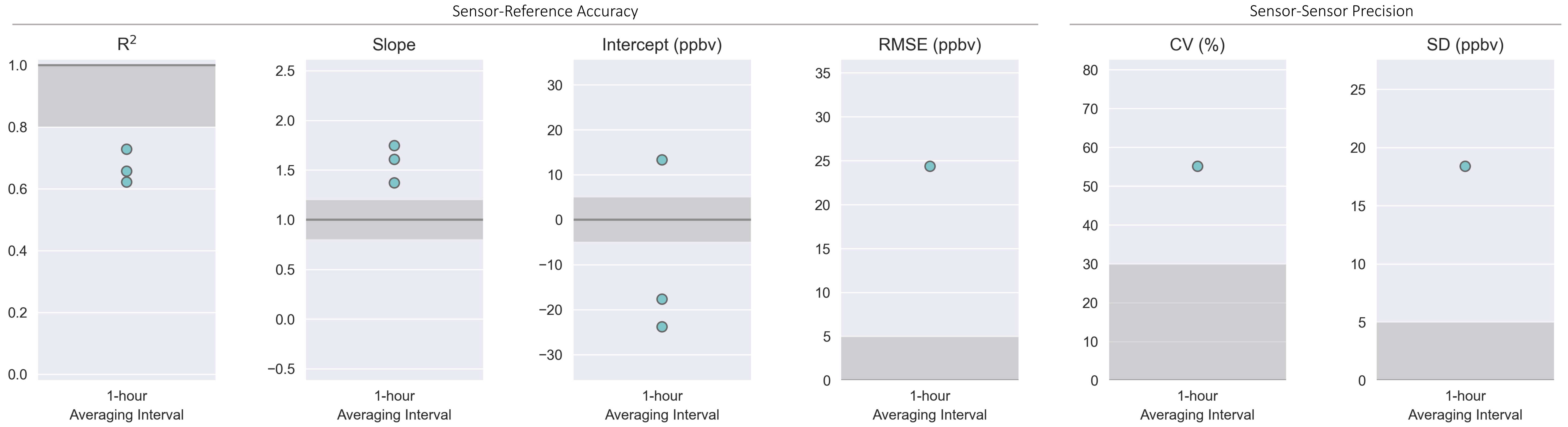

Meteorological Conditions During Deployment

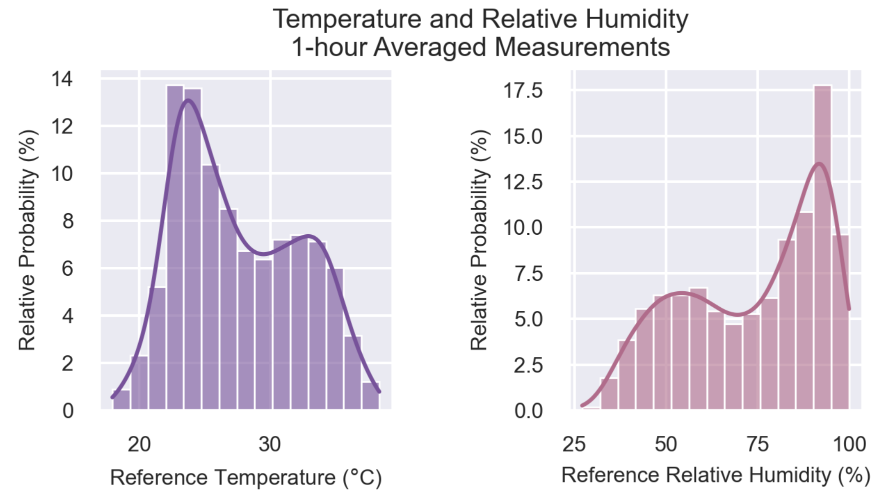

Number of 1-hr periods outside sensor manufacturer-listed temperature operational range (-30 to 40 °C)	0
Number of 1-hr periods outside sensor manufacturer-listed relative humidity operational range (no operational range specified)	-

Meteorological Influence

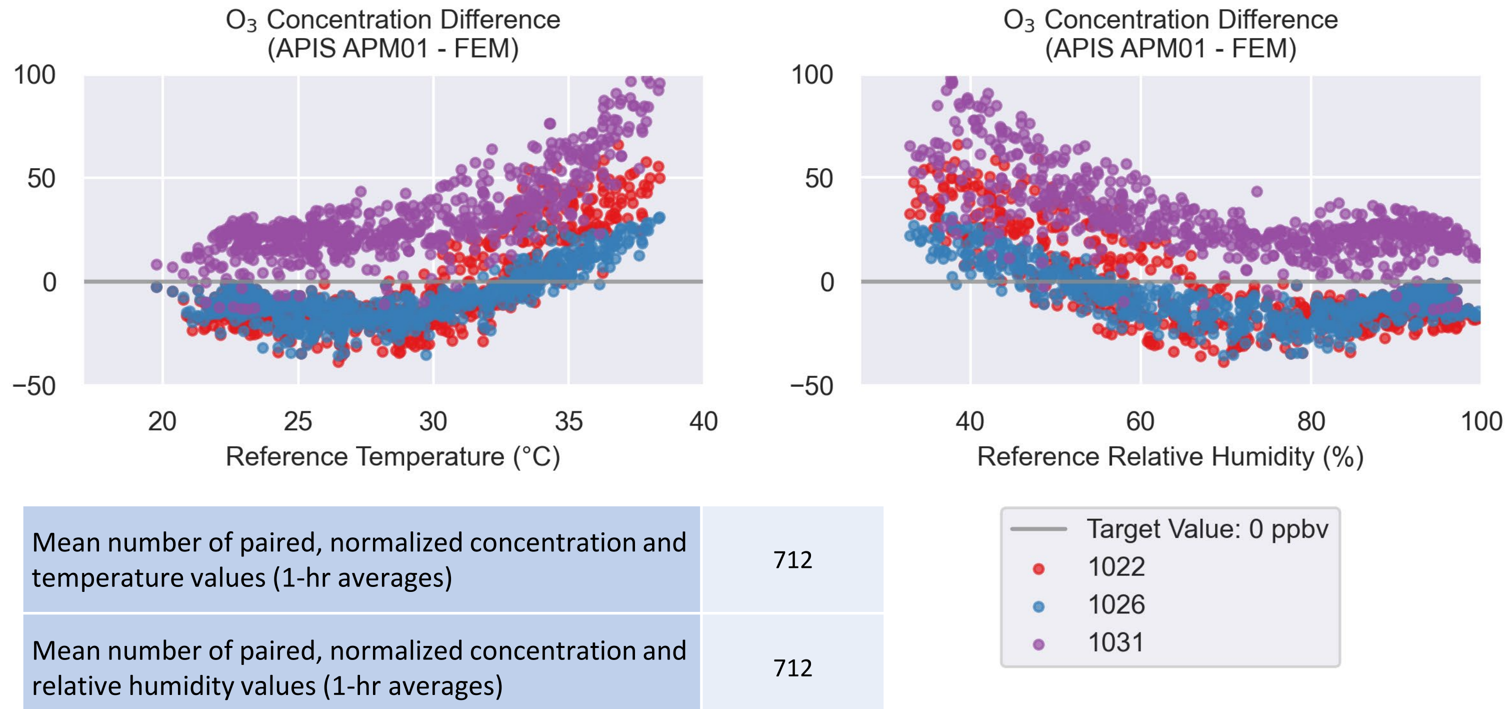

Mean number of paired, normalized concentration and temperature values (1-hr averages)	712
Mean number of paired, normalized concentration and relative humidity values (1-hr averages)	712

★For evaluations with greater than three sensors, grouping individual sensor metrics into boxplots is recommended for displaying results. Note that this recommendation does not apply to metrics computed as a single value for all sensors over the whole evaluation group, such as RMSE, NRMSE, CV, and standard deviation.

Testing Report - O_3 Base Testing

Apis APM01

This report reflects out-of-the-box performance

Initial Base Testing - RTP, NC
U.S. Environmental Protection Agency
Office of Research and Development
PI: Clements.Andrea@epa.gov
919-541-1363
July 2020—August 2020

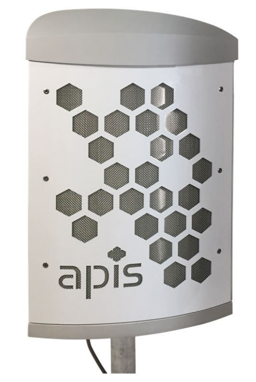

Image Attribution: Apis, Inc (Gants Pass, OR)

Tabular Statistics

Sensor-FRM/FEM Correlation

	Bias and Linearity			Data Quality	
	R^2	Slope	Intercept (ppbv)	Uptime (%)	Number of paired sensor and reference concentration values*
	1-Hour ○○○	1-Hour ○○○	1-Hour ○○○	1-Hour ●●●	1-Hour
Metric Target Range	≥ 0.80	1.0 ± 0.20	-5 ≤ b ≤ 5	75%*	-
Sensor 1022	0.62	1.75	-23.83	100	694
Sensor 1026	0.73	1.37	-17.67	100	694
Sensor 1031	0.66	1.61	13.30	100	677
Mean	0.67	1.57	-9.40	100.00	688.33

	Error
	RMSE (ppbv)
	1-Hour ☆
Metric Target Range	≤ 5.0
Deployment Value	24.4

Device-specific metrics (computed for each sensor in evaluation)
○○○ Metric value for none of devices tested falls within the target range
●○○ Metric value for one of devices tested falls within the target range
●●○ Metric value for two of devices tested falls within the target range
●●● Metric value for three of devices tested falls within the target range

Single-valued metrics (computed via entire evaluation dataset)
☆ Indicates that the metric value is not within the target range
★ Indicates that the metric value is within the target range

Sensor-Sensor Precision

	Precision (between collocated sensors)		Data Quality
	CV (%)	SD (ppbv)	Number of paired sensor and reference concentration values*
	1-Hour ☆	1-Hour ☆	1-Hour
Metric Target Range	≤ 30.0	≤ 5.0	-
Deployment Value	55.2	18.4	718

*This value is only a recommendation for ensuring data quality and is not included in the list of target values discussed in Section 4 of the Performance Testing Protocols, Metrics, and Target Values for Ozone Air Sensors document.

Testing Report - O_3 Base Testing

Apis APM01

This report reflects out-of-the-box performance

Initial Base Testing - RTP, NC
U.S. Environmental Protection Agency
Office of Research and Development
PI: Clements.Andrea@epa.gov
919-541-1363
July 2020—August 2020

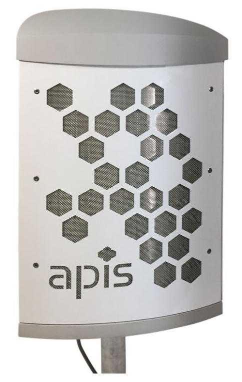

Image Attribution: Apis, Inc (Gants Pass, OR)

Sensor-FRM/FEM Scatter Plots

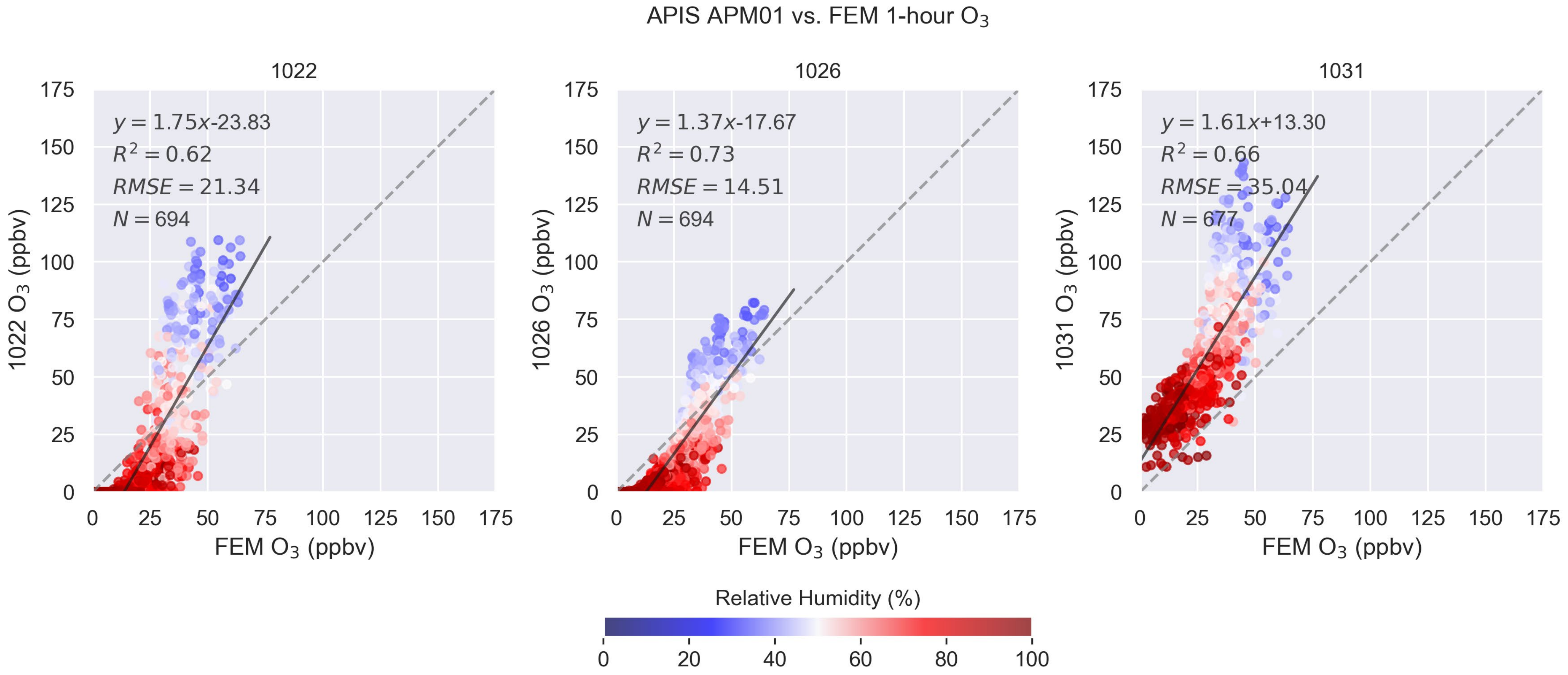

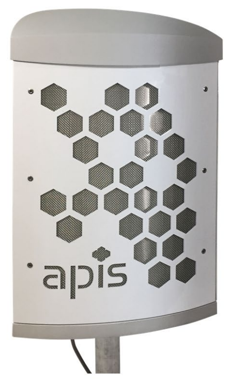

Image Attribution: Apis, Inc (Gants Pass, OR)

Supplemental Information

Abbreviations used in Supplemental Information

FRM	Federal Reference Method
FEM	Federal Equivalent Method
SOP	Standard Operating Procedure
QAPP	Quality Assurance Project Plan
QC	Quality Control

Supplemental Documentation	Attached	Description & URL or file path to documentation
Field observations and sensor data flags	☑	See NC-APM-Page 6 of this testing report
Maintenance logs	☑	No logs recorded during testing
Standard operating procedure(s)	☐	U.S. EPA Office Of Research and Development SOP available upon request
Photos of equipment setup and testing	☑	See NC-APM-Page 5 of this testing report
Product specifications sheet(s)	☐	See Appendix C, “Spec_Sheet_Apis_APM01.pdf”
Product manual(s)	☐	See Appendix C, “Manual_Apis_APM01.pdf”
Data storage and transmission method	☑	See NC-APM-Page 6 of this testing report
Data correction approach	☑	See NC-APM-Page 6 of this testing report
Issues encountered	☑	See NC-APM-Page 6 of this testing report
Data analysis/correction scripts and version	☑	Averaging and processing of data, calculation of performance metrics, and generation of figures and other supplementary material for analysis were obtained using Python 3.9.7 with the packages sensortoolkit v0.8.3b2, pandas 1.3.5, NumPy 1.21.2, Matplotlib 3.5.0, statsmodels 0.13.0, and seaborn 0.11.2. All packages are available from the Python Package Index (PyPI) at https://pypi.org. The integrated development environment (IDE) Spyder 5.1.5 was used for scripting and data visualization. Version control for the Python base, packages, and IDE were all managed by conda 4.11.0.
Air Monitoring Station QAPP	☐	U.S. EPA Office Of Research and Development QAPP available upon request
Summary of FRM/FEM monitor QC checks	☑	See NC-APM-Pages 7-18 of this testing report
Manufacturer website for FRM/FEM monitor	☑	Teledyne API: T265 Product website
FRM/FEM monitor manual	☑	See Appendix B, “Spec_Sheet_TeledyneAPI_T265.pdf”
FRM/FEM monitor specifications sheet(s)	☑	See Appendix B, “Manual_TeledyneAPI_T265.pdf”
Other documents	☐	

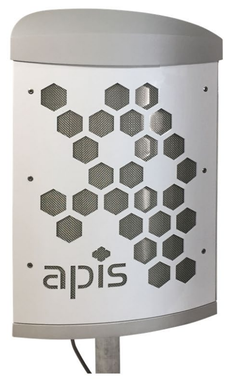

Image Attribution: Apis, Inc (Gants Pass, OR)

Supplemental Information: Photos of Testing Site and Equipment Setup

Site Description:

The Burdens Creek Ambient Monitoring Innovation Research Station (AIRS) site is located on the U.S. EPA, RTP campus and is situated between Alexander Drive and Route 147. The site is intended to represent a neighborhood-scale site as defined in *40 CFR Part 58, Appendix D*. U.S. EPA's Office of Air Quality Planning and Standards (OAQPS) operates reference grade instruments in a free-standing shelter situated directly adjacent to the sensor testing platform.

Figure 1: Apis APM01 sensors (indicated by red arrow) mounted at evaluation site. Units are attached to the exterior side of the observation deck railing.

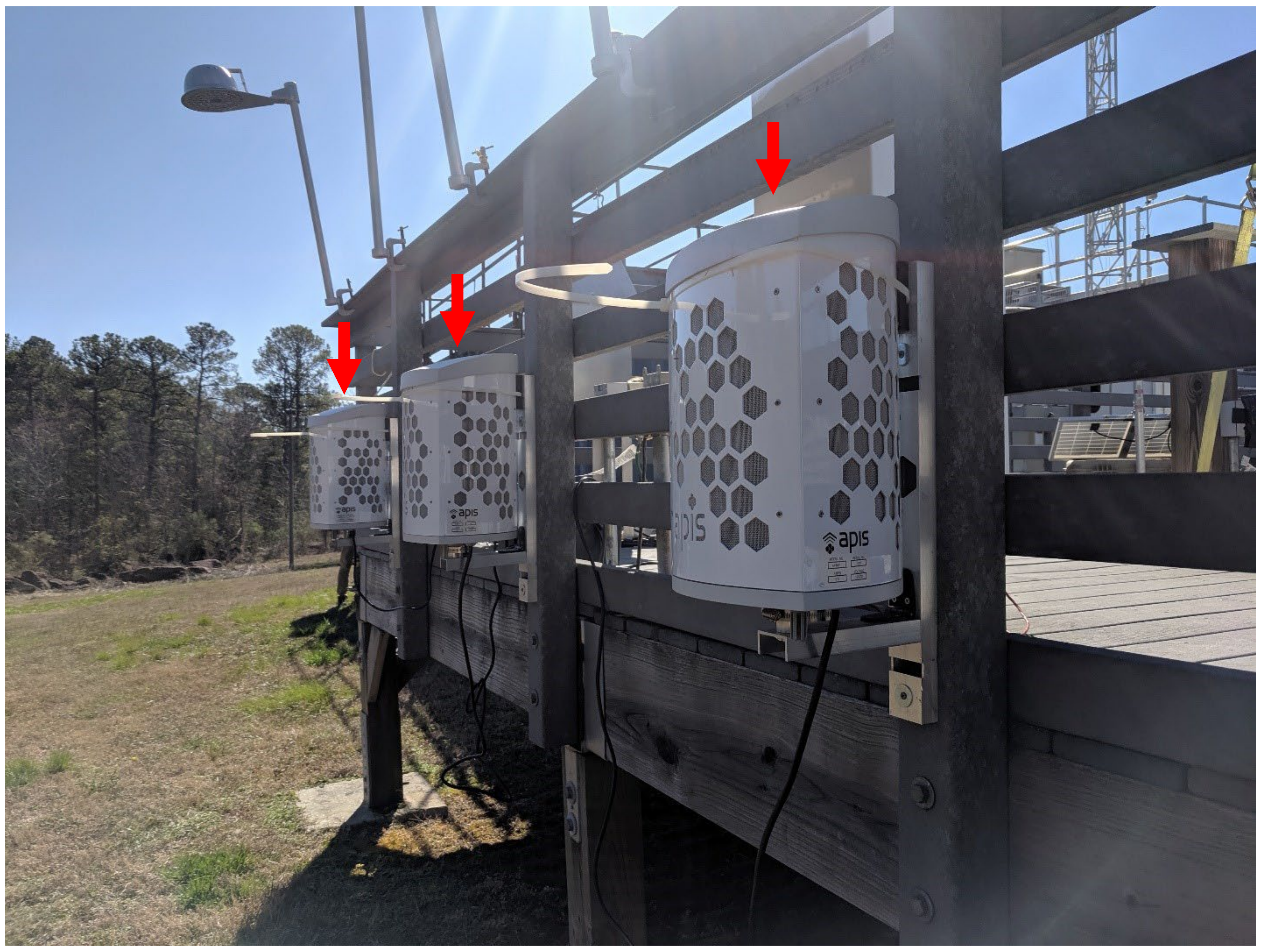

Figure 2: Observation deck at evaluation site. Approximate location of sensors indicated by the red arrow. The approximate location of FEM (housed within sampling trailer) indicated by the black arrow. Sensors and FEM instrumentation are separated by approximately 20 meters.

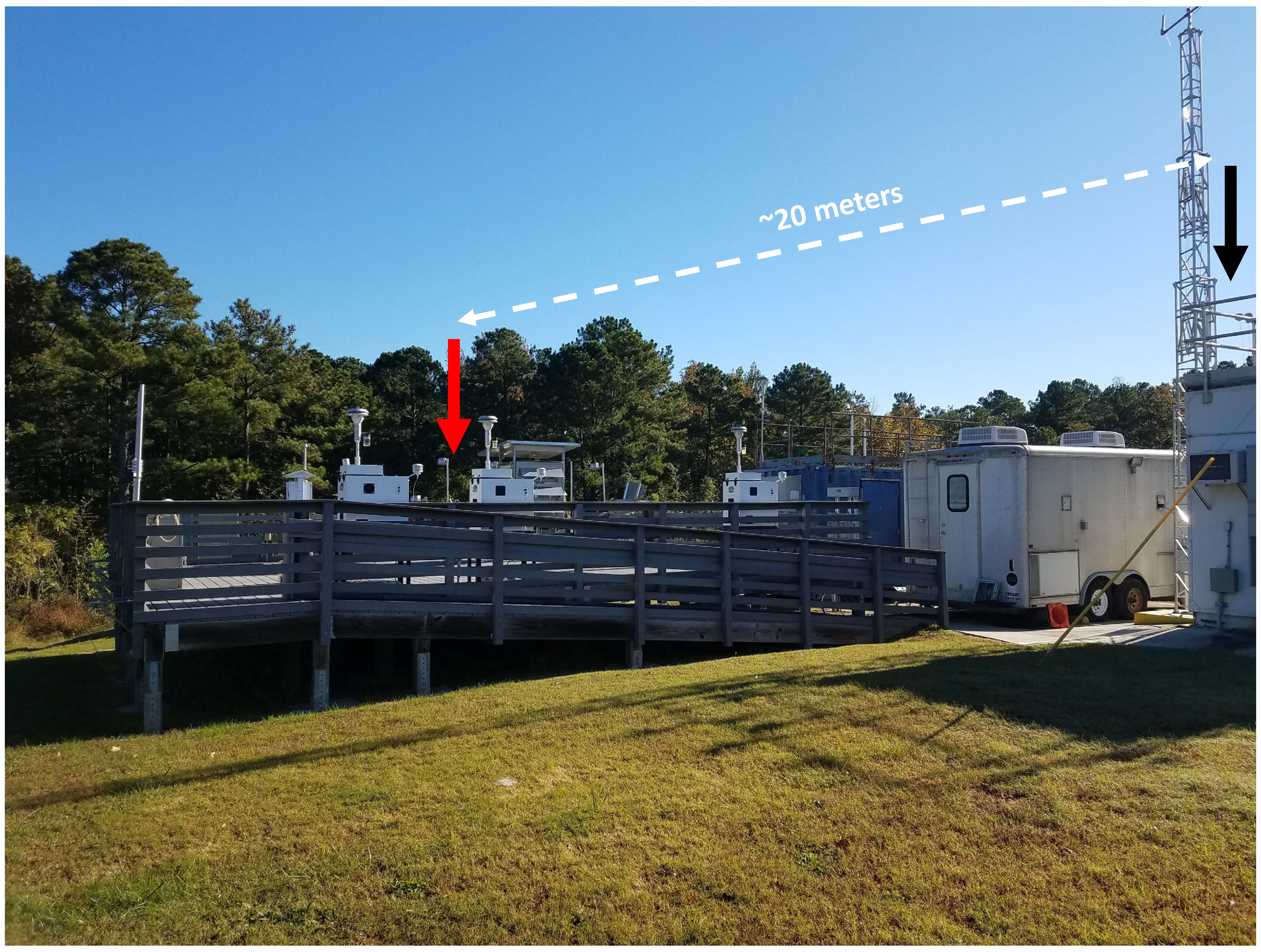

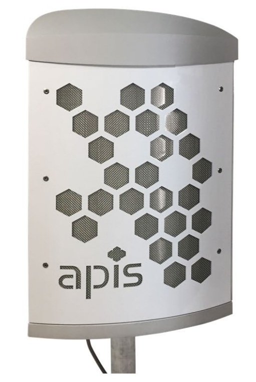

Image Attribution: Apis, Inc (Gants Pass, OR)

Data Storage and Transmission Method

The APM01 transfers recorded data to cloud servers using a cellular connection. In addition, the APM01 includes an onboard microSD card that allows storage of approximately one week of data, however, the SD card was not user accessable. For this study, the cellular connection was used to log datasets on the vendor's cloud server. Data files were downloaded from the Apis website via the "Customer Login" portal. Some extended datasets required transfer via an Apis representative (see field observations for further discussion).

Data Correction Approach

This evaluation report reflects "out-of-the-box" performance of the APM01. Using the cellular connection, APM01 data are stored on Apis' cloud servers and are processed using proprietary correction algorithms using only data available on-board the sensor. Data stored to the onboard microSD card are *not processed* using correction algorithms, although as previously noted, the SD card was not accessible during the testing period. This report corresponds to processed data (proprietary correction algorithms applied).

In addition to software-based correction methods, the manufacturer requires that all APM01 models to be sent to the manufacturer at least annually for calibration and service.

After acquisition, the raw data was processed using the *sensortoolkit* python code library (v0.8.3b2). A continuous data set at the recorded sampling frequency was written to a .csv file. 1-hour averaged data sets were generated using a 75% completeness threshold and saved as separate .csv files. Outliers were **not** removed from data sets in order to assess "out-of-the-box" sensor performance.

The duration of the warm-up period required for sensor measurements to equilibrate was determined from field data to be 2 hours. Warm up periods were considered to occur following any power outage to sensor units, either due to routine field visits or unscheduled site power outages. Data recorded during warm up periods have been removed from data sets.

Issues Encountered

<u>Pre-deployment observations</u>

- *Difficulties obtaining cellular connection in building:* During benchtop testing, sensor units were not able to obtain a cellular signal indoors. We worked with the company to try to boost the signal which did not work. Ongoing connectivity issues prevented indoor filter room zero air testing prior to field deployment.
- *Damaged sensor housing*: During benchtop testing, unit 1031's fan ran considerably louder than the other 2 units. A large crack on the bottom plastic of the unit was found while unpacking, suggesting some damage may have occurred during transit. The crack in the plastic did not structurally compromise the unit. The outside casing was removed to inspect the interior for damage, and no obvious damage was found.

<u>Field observations and sensor data flags</u>

All three units were initially deployed at AIRS on 2/27/2020. Due to initial connection issues with one unit (discussed below), repairs to the unit were required and all three devices were re-deployed on 7/10/2020. This report reflects the time period following re-deployment in July 2020.

- *Issues acquiring data via online platform:* Initial attempts at downloading data via the Apis web platform indicated an error message, "*currently offline for maintenance*". During following attempts, the screen would flash as if downloading commenced, but no data would be downloaded. An Apis representative suggested the issue could have resulted from a server overload, as a large volume of data were selected for download queries. The representative indicated the platform can only package seven days of recorded data per query, and that an error message failed to display informing the user of the download limit. The Apis representative provided recorded datasets via email.
- *Invalid Sensor Connection*: During initial deployment, the technician mis-interpreted cloud connection confirmation for unit 1031. A series of power cycles were conducted, with pauses of up to 10 minutes between cycles, to try to establish the connection. The unit still failed to connect and was retrieved on 3/19/2020 and sent back to the manufacturer.

The following table contains data flags describing events that were encountered during the testing period.

Start Time (UTC)	End Time (UTC)	Sensor Serial ID	Parameters Impacted	Flag
2020-07-21 19:00:00+0000	2020-07-21 19:05:00+0000	1031	ALL	Operator working near device (scheduled site visit).
2020-08-09 05:00:00+0000	2020-08-09 05:05:00+0000	1031	ALL	Operator working near device (scheduled site visit).

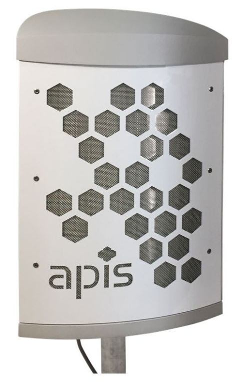

Image Attribution: Apis, Inc (Gants Pass, OR)

Description of Data Flags

OAQPS manages data logged by reference monitors at the AIRS evaluation site using the Envidas data acquisition system software from DR DAS LTD[1]. Envidas contains over 100 data flags which are configurable by the monitoring agency and can be triggered based on instrument status. Appendix B Table 1 contains a description of Envidas data flags including the numeric code and status code name (a brief textual description, the table containing data flags below includes code names for events encountered by the reference monitor under the "Flag" column). Appendix B Table 1 also indicates whether reference data are invalidated for a given data flag. For this report, reference data were invalidated for periods where a data flag was logged and if the corresponding entry for the data flag in the "Data Status Invalidation" column in Appendix B Table 1 indicates data should be invalidated.

Data Flags Recorded During Testing

FRM/FEM Monitor	Timestamp (UTC)	Flag
Teledyne API T265 (Data acquired via local transfer from OAQPS)	2020-07-11 09:46:00+0000 to 2020-07-11 10:00:00+0000	Zero
	2020-07-11 10:01:00+0000 to 2020-07-11 10:15:00+0000	Span
	2020-07-11 10:16:00+0000 to 2020-07-11 10:30:00+0000	Spare
	2020-07-11 10:31:00+0000 to 2020-07-11 10:35:00+0000	Purge
	2020-07-12 09:46:00+0000 to 2020-07-12 10:00:00+0000	Zero
	2020-07-12 10:01:00+0000 to 2020-07-12 10:15:00+0000	Span
	2020-07-12 10:16:00+0000 to 2020-07-12 10:30:00+0000	Spare
	2020-07-12 10:31:00+0000 to 2020-07-12 10:35:00+0000	Purge
	2020-07-13 09:46:00+0000 to 2020-07-13 10:00:00+0000	Zero
	2020-07-13 10:01:00+0000 to 2020-07-13 10:15:00+0000	Span
	2020-07-13 10:16:00+0000 to 2020-07-13 10:30:00+0000	Spare
	2020-07-13 10:31:00+0000	Calib
	2020-07-13 10:32:00+0000 to 2020-07-13 10:35:00+0000	Purge
	2020-07-14 09:46:00+0000 to 2020-07-14 10:00:00+0000	Zero
	2020-07-14 10:01:00+0000 to 2020-07-14 10:15:00+0000	Span
	2020-07-14 10:16:00+0000 to 2020-07-14 10:30:00+0000	Spare
	2020-07-14 10:31:00+0000 to 2020-07-14 10:35:00+0000	Purge
	2020-07-15 09:46:00+0000 to 2020-07-15 10:00:00+0000	Zero
	2020-07-15 10:01:00+0000 to 2020-07-15 10:15:00+0000	Span
	2020-07-15 10:16:00+0000 to 2020-07-15 10:30:00+0000	Spare
	2020-07-15 10:31:00+0000 to 2020-07-15 10:35:00+0000	Purge
	2020-07-16 09:46:00+0000 to 2020-07-16 10:00:00+0000	Zero
	2020-07-16 10:01:00+0000 to 2020-07-16 10:15:00+0000	Span
	2020-07-16 10:16:00+0000 to 2020-07-16 10:30:00+0000	Spare
	2020-07-16 10:31:00+0000 to 2020-07-16 10:35:00+0000	Purge
	2020-07-17 09:46:00+0000 to 2020-07-17 10:00:00+0000	Zero
	2020-07-17 10:01:00+0000 to 2020-07-17 10:15:00+0000	Span
	2020-07-17 10:16:00+0000	Calib
	2020-07-17 10:17:00+0000 to 2020-07-17 10:30:00+0000	Spare
	2020-07-17 10:31:00+0000 to 2020-07-17 10:35:00+0000	Purge

[1] "Contact us" DR DAS LTD, 2022, https://dr-das.com/Home/Contact.html Accessed June 3 2022

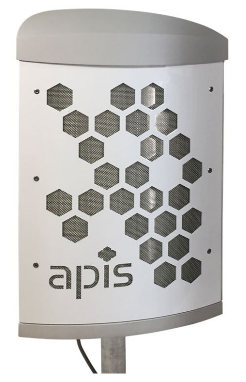

Image Attribution: Apis, Inc (Gants Pass, OR)

Data Flags Recorded During Testing (Cont.)

FRM/FEM Monitor	Timestamp (UTC)	Flag
Teledyne API T265 (Data acquired via local transfer from OAQPS)	2020-07-17 20:30:00+0000	<Samp
	2020-07-18 09:46:00+0000 to 2020-07-18 10:00:00+0000	Zero
	2020-07-18 10:01:00+0000 to 2020-07-18 10:15:00+0000	Span
	2020-07-18 10:16:00+0000 to 2020-07-18 10:30:00+0000	Spare
	2020-07-18 10:31:00+0000 to 2020-07-18 10:35:00+0000	Purge
	2020-07-19 09:46:00+0000 to 2020-07-19 10:00:00+0000	Zero
	2020-07-19 10:01:00+0000 to 2020-07-19 10:15:00+0000	Span
	2020-07-19 10:16:00+0000 to 2020-07-19 10:30:00+0000	Spare
	2020-07-19 10:31:00+0000 to 2020-07-19 10:35:00+0000	Purge
	2020-07-20 09:46:00+0000 to 2020-07-20 10:00:00+0000	Zero
	2020-07-20 10:01:00+0000 to 2020-07-20 10:15:00+0000	Span
	2020-07-20 10:16:00+0000 to 2020-07-20 10:30:00+0000	Spare
	2020-07-20 10:31:00+0000 to 2020-07-20 10:35:00+0000	Purge
	2020-07-20 20:56:00+0000	<Samp
	2020-07-20 21:14:00+0000 to 2020-07-20 21:29:00+0000	NoData
	2020-07-20 21:31:00+0000 to 2020-07-20 21:41:00+0000	NoData
	2020-07-20 21:43:00+0000 to 2020-07-20 22:01:00+0000	NoData
	2020-07-20 22:03:00+0000 to 2020-07-20 22:12:00+0000	NoData
	2020-07-20 22:14:00+0000 to 2020-07-20 22:37:00+0000	NoData
	2020-07-20 22:39:00+0000 to 2020-07-20 22:44:00+0000	NoData
	2020-07-20 22:46:00+0000 to 2020-07-20 22:58:00+0000	NoData
	2020-07-20 23:00:00+0000 to 2020-07-20 23:04:00+0000	NoData
	2020-07-20 23:06:00+0000 to 2020-07-20 23:08:00+0000	NoData
	2020-07-20 23:10:00+0000 to 2020-07-20 23:38:00+0000	NoData
	2020-07-20 23:40:00+0000 to 2020-07-20 23:44:00+0000	NoData
	2020-07-20 23:46:00+0000 to 2020-07-20 23:50:00+0000	NoData
	2020-07-20 23:52:00+0000 to 2020-07-21 00:20:00+0000	NoData
	2020-07-21 00:22:00+0000 to 2020-07-21 00:30:00+0000	NoData
	2020-07-21 00:33:00+0000 to 2020-07-21 00:58:00+0000	NoData
	2020-07-21 01:00:00+0000 to 2020-07-21 01:10:00+0000	NoData
	2020-07-21 01:12:00+0000 to 2020-07-21 01:13:00+0000	NoData
	2020-07-21 01:17:00+0000	NoData
	2020-07-21 01:19:00+0000 to 2020-07-21 01:22:00+0000	NoData
	2020-07-21 01:24:00+0000 to 2020-07-21 01:27:00+0000	NoData
	2020-07-21 01:30:00+0000 to 2020-07-21 01:31:00+0000	NoData
	2020-07-21 01:33:00+0000 to 2020-07-21 01:36:00+0000	NoData
	2020-07-21 01:37:00+0000	<Samp
	2020-07-21 01:39:00+0000 to 2020-07-21 02:02:00+0000	NoData
	2020-07-21 01:42:00+0000	<Samp
	2020-07-21 02:04:00+0000 to 2020-07-21 02:06:00+0000	NoData
	2020-07-21 02:08:00+0000 to 2020-07-21 02:42:00+0000	NoData
	2020-07-21 02:37:00+0000	<Samp
	2020-07-21 02:44:00+0000 to 2020-07-21 02:51:00+0000	NoData
	2020-07-21 02:53:00+0000 to 2020-07-21 03:20:00+0000	NoData
	2020-07-21 03:22:00+0000	NoData
	2020-07-21 03:24:00+0000 to 2020-07-21 03:44:00+0000	NoData

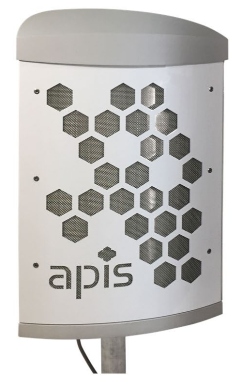

Image Attribution: Apis, Inc (Gants Pass, OR)

Data Flags Recorded During Testing (Cont.)

FRM/FEM Monitor	Timestamp (UTC)	Flag
Teledyne API T265 (Data acquired via local transfer from OAQPS)	2020-07-21 03:40:00+0000	<Samp
	2020-07-21 03:47:00+0000 to 2020-07-21 03:54:00+0000	NoData
	2020-07-21 03:56:00+0000 to 2020-07-21 04:04:00+0000	NoData
	2020-07-21 04:06:00+0000 to 2020-07-21 04:18:00+0000	NoData
	2020-07-21 04:24:00+0000	NoData
	2020-07-21 04:30:00+0000	NoData
	2020-07-21 04:38:00+0000	NoData
	2020-07-21 04:45:00+0000	NoData
	2020-07-21 04:49:00+0000	NoData
	2020-07-21 04:54:00+0000	NoData
	2020-07-21 04:56:00+0000 to 2020-07-21 04:57:00+0000	NoData
	2020-07-21 05:01:00+0000 to 2020-07-21 05:02:00+0000	NoData
	2020-07-21 05:04:00+0000 to 2020-07-21 05:06:00+0000	NoData
	2020-07-21 05:10:00+0000	NoData
	2020-07-21 05:12:00+0000	NoData
	2020-07-21 05:18:00+0000 to 2020-07-21 05:19:00+0000	NoData
	2020-07-21 05:24:00+0000	NoData
	2020-07-21 05:28:00+0000	NoData
	2020-07-21 05:42:00+0000 to 2020-07-21 05:45:00+0000	NoData
	2020-07-21 05:50:00+0000	NoData
	2020-07-21 05:52:00+0000	NoData
	2020-07-21 05:57:00+0000 to 2020-07-21 05:58:00+0000	NoData
	2020-07-21 06:02:00+0000	NoData
	2020-07-21 06:04:00+0000 to 2020-07-21 06:05:00+0000	NoData
	2020-07-21 06:11:00+0000 to 2020-07-21 06:12:00+0000	NoData
	2020-07-21 06:25:00+0000	NoData
	2020-07-21 06:30:00+0000	NoData
	2020-07-21 06:32:00+0000	NoData
	2020-07-21 06:42:00+0000	NoData
	2020-07-21 06:44:00+0000 to 2020-07-21 06:45:00+0000	NoData
	2020-07-21 06:51:00+0000 to 2020-07-21 06:52:00+0000	NoData
	2020-07-21 07:04:00+0000	NoData
	2020-07-21 07:11:00+0000 to 2020-07-21 07:12:00+0000	NoData
	2020-07-21 07:25:00+0000	NoData
	2020-07-21 07:31:00+0000 to 2020-07-21 07:32:00+0000	NoData
	2020-07-21 07:35:00+0000	NoData
	2020-07-21 07:39:00+0000	NoData
	2020-07-21 07:44:00+0000	NoData
	2020-07-21 07:50:00+0000	NoData
	2020-07-21 07:56:00+0000	NoData
	2020-07-21 07:58:00+0000 to 2020-07-21 07:59:00+0000	NoData
	2020-07-21 08:04:00+0000	NoData
	2020-07-21 08:12:00+0000	NoData
	2020-07-21 08:16:00+0000	NoData
	2020-07-21 08:24:00+0000	NoData
	2020-07-21 08:26:00+0000	NoData

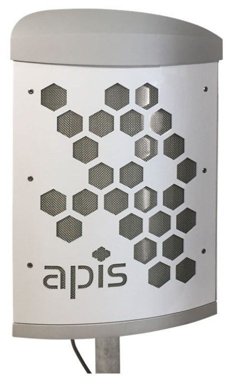

Image Attribution: Apis, Inc (Gants Pass, OR)

Data Flags Recorded During Testing (Cont.)

FRM/FEM Monitor	Timestamp (UTC)	Flag
Teledyne API T265 (Data acquired via local transfer from OAQPS)	2020-07-21 08:28:00+0000	NoData
	2020-07-21 08:30:00+0000	NoData
	2020-07-21 08:35:00+0000	NoData
	2020-07-21 08:42:00+0000	NoData
	2020-07-21 08:46:00+0000	NoData
	2020-07-21 08:52:00+0000	NoData
	2020-07-21 08:58:00+0000	NoData
	2020-07-21 09:04:00+0000 to 2020-07-21 09:05:00+0000	NoData
	2020-07-21 09:16:00+0000	NoData
	2020-07-21 09:18:00+0000	NoData
	2020-07-21 09:22:00+0000 to 2020-07-21 09:24:00+0000	NoData
	2020-07-21 09:28:00+0000	NoData
	2020-07-21 09:32:00+0000	NoData
	2020-07-21 09:42:00+0000	NoData
	2020-07-21 09:46:00+0000 to 2020-07-21 09:51:00+0000	Zero
	2020-07-21 09:48:00+0000 to 2020-07-21 09:50:00+0000	NoData
	2020-07-21 10:00:00+0000 to 2020-07-21 10:02:00+0000	NoData
	2020-07-21 10:06:00+0000	NoData
	2020-07-21 10:14:00+0000	NoData
	2020-07-21 10:16:00+0000	NoData
	2020-07-21 10:18:00+0000 to 2020-07-21 10:20:00+0000	NoData
	2020-07-21 10:27:00+0000	NoData
	2020-07-21 10:30:00+0000	NoData
	2020-07-21 10:34:00+0000	NoData
	2020-07-21 10:45:00+0000 to 2020-07-21 10:47:00+0000	NoData
	2020-07-21 10:53:00+0000	NoData
	2020-07-21 11:10:00+0000 to 2020-07-21 11:11:00+0000	NoData
	2020-07-21 11:16:00+0000	NoData
	2020-07-21 11:18:00+0000	NoData
	2020-07-21 11:23:00+0000	NoData
	2020-07-21 11:30:00+0000	NoData
	2020-07-21 11:36:00+0000	NoData
	2020-07-21 11:42:00+0000	NoData
	2020-07-21 11:48:00+0000	NoData
	2020-07-21 11:50:00+0000	NoData
	2020-07-21 11:54:00+0000	NoData
	2020-07-21 12:00:00+0000	NoData
	2020-07-21 12:03:00+0000	NoData
	2020-07-21 12:10:00+0000	NoData
	2020-07-21 12:13:00+0000	NoData
	2020-07-21 12:23:00+0000	NoData
	2020-07-21 12:32:00+0000	NoData
	2020-07-21 12:36:00+0000	NoData
	2020-07-21 12:45:00+0000	NoData
	2020-07-21 12:47:00+0000	NoData
	2020-07-21 12:54:00+0000	NoData

Data Flags Recorded During Testing (Cont.)

FRM/FEM Monitor	Timestamp (UTC)	Flag
Teledyne API T265<br>(Data acquired via local transfer from OAQPS)	2020-07-21 12:56:00+0000	NoData
	2020-07-21 13:08:00+0000	NoData
	2020-07-21 13:32:00+0000 to 2020-07-21 13:33:00+0000	NoData
	2020-07-21 13:40:00+0000	NoData
	2020-07-21 13:46:00+0000	NoData
	2020-07-21 13:50:00+0000	NoData
	2020-07-21 13:52:00+0000	NoData
	2020-07-21 13:58:00+0000	NoData
	2020-07-21 14:12:00+0000	NoData
	2020-07-21 14:18:00+0000	NoData
	2020-07-21 14:25:00+0000	NoData
	2020-07-21 14:31:00+0000	NoData
	2020-07-21 14:37:00+0000	NoData
	2020-07-21 14:43:00+0000	NoData
	2020-07-21 14:48:00+0000	NoData
	2020-07-21 14:50:00+0000	NoData
	2020-07-21 14:56:00+0000	NoData
	2020-07-21 15:02:00+0000	NoData
	2020-07-21 15:06:00+0000	NoData
	2020-07-21 15:11:00+0000 to 2020-07-21 15:15:00+0000	NoData
	2020-07-21 15:20:00+0000 to 2020-07-21 15:21:00+0000	NoData
	2020-07-21 15:27:00+0000	NoData
	2020-07-21 15:32:00+0000 to 2020-07-21 15:34:00+0000	NoData
	2020-07-21 15:39:00+0000	NoData
	2020-07-21 15:41:00+0000	NoData
	2020-07-21 15:46:00+0000 to 2020-07-21 15:47:00+0000	NoData
	2020-07-21 15:57:00+0000	NoData
	2020-07-21 15:59:00+0000 to 2020-07-21 16:00:00+0000	NoData
	2020-07-21 16:13:00+0000	NoData
	2020-07-21 16:28:00+0000	NoData
	2020-07-21 16:44:00+0000	NoData
	2020-07-21 16:54:00+0000 to 2020-07-21 16:59:00+0000	<Samp
	2020-07-21 16:55:00+0000 to 2020-07-21 16:58:00+0000	NoData
	2020-07-22 09:46:00+0000 to 2020-07-22 10:00:00+0000	Zero
	2020-07-22 10:01:00+0000 to 2020-07-22 10:15:00+0000	Span
	2020-07-22 10:16:00+0000 to 2020-07-22 10:30:00+0000	Spare
	2020-07-22 10:31:00+0000 to 2020-07-22 10:35:00+0000	Purge
	2020-07-23 09:46:00+0000 to 2020-07-23 10:00:00+0000	Zero
	2020-07-23 10:01:00+0000 to 2020-07-23 10:15:00+0000	Span
	2020-07-23 10:16:00+0000 to 2020-07-23 10:30:00+0000	Spare
	2020-07-23 10:31:00+0000 to 2020-07-23 10:35:00+0000	Purge
	2020-07-24 09:46:00+0000 to 2020-07-24 10:00:00+0000	Zero
	2020-07-24 10:01:00+0000 to 2020-07-24 10:15:00+0000	Span
	2020-07-24 10:16:00+0000 to 2020-07-24 10:30:00+0000	Spare
	2020-07-24 10:31:00+0000 to 2020-07-24 10:35:00+0000	Purge
	2020-07-25 09:46:00+0000 to 2020-07-25 10:00:00+0000	Zero

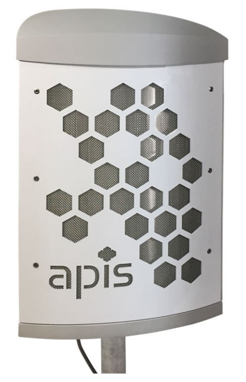

Image Attribution: Apis, Inc (Gants Pass, OR)

Data Flags Recorded During Testing (Cont.)

FRM/FEM Monitor	Timestamp (UTC)	Flag
Teledyne API T265 (Data acquired via local transfer from OAQPS)	2020-07-25 10:01:00+0000 to 2020-07-25 10:15:00+0000	Span
	2020-07-25 10:16:00+0000 to 2020-07-25 10:30:00+0000	Spare
	2020-07-25 10:31:00+0000 to 2020-07-25 10:35:00+0000	Purge
	2020-07-26 09:46:00+0000 to 2020-07-26 10:00:00+0000	Zero
	2020-07-26 10:01:00+0000 to 2020-07-26 10:15:00+0000	Span
	2020-07-26 10:16:00+0000 to 2020-07-26 10:30:00+0000	Spare
	2020-07-26 10:31:00+0000 to 2020-07-26 10:35:00+0000	Purge
	2020-07-27 09:46:00+0000 to 2020-07-27 10:00:00+0000	Zero
	2020-07-27 10:01:00+0000 to 2020-07-27 10:15:00+0000	Span
	2020-07-27 10:16:00+0000 to 2020-07-27 10:30:00+0000	Spare
	2020-07-27 10:31:00+0000 to 2020-07-27 10:35:00+0000	Purge
	2020-07-28 09:46:00+0000 to 2020-07-28 10:00:00+0000	Zero
	2020-07-28 10:01:00+0000 to 2020-07-28 10:15:00+0000	Span
	2020-07-28 10:16:00+0000 to 2020-07-28 10:30:00+0000	Spare
	2020-07-28 10:31:00+0000 to 2020-07-28 10:35:00+0000	Purge
	2020-07-29 09:46:00+0000 to 2020-07-29 10:00:00+0000	Zero
	2020-07-29 10:01:00+0000 to 2020-07-29 10:15:00+0000	Span
	2020-07-29 10:16:00+0000 to 2020-07-29 10:30:00+0000	Spare
	2020-07-29 10:31:00+0000 to 2020-07-29 10:35:00+0000	Purge
	2020-07-30 09:46:00+0000 to 2020-07-30 10:00:00+0000	Zero
	2020-07-30 10:01:00+0000 to 2020-07-30 10:15:00+0000	Span
	2020-07-30 10:16:00+0000 to 2020-07-30 10:30:00+0000	Spare
	2020-07-30 10:31:00+0000 to 2020-07-30 10:35:00+0000	Purge
	2020-07-30 14:36:00+0000	<Samp
	2020-07-30 18:08:00+0000 to 2020-07-30 18:12:00+0000	NoData
	2020-07-30 18:13:00+0000	<Samp
	2020-07-30 18:31:00+0000 to 2020-07-30 18:34:00+0000	NoData
	2020-07-30 18:35:00+0000	<Samp
	2020-07-30 20:04:00+0000	<Samp
	2020-07-31 09:46:00+0000 to 2020-07-31 10:00:00+0000	Zero
	2020-07-31 10:01:00+0000 to 2020-07-31 10:15:00+0000	Span
	2020-07-31 10:16:00+0000 to 2020-07-31 10:30:00+0000	Spare
	2020-07-31 10:31:00+0000 to 2020-07-31 10:35:00+0000	Purge
	2020-08-01 09:46:00+0000 to 2020-08-01 10:00:00+0000	Zero
	2020-08-01 10:01:00+0000 to 2020-08-01 10:15:00+0000	Span
	2020-08-01 10:16:00+0000 to 2020-08-01 10:30:00+0000	Spare
	2020-08-01 10:31:00+0000 to 2020-08-01 10:35:00+0000	Purge
	2020-08-02 09:46:00+0000 to 2020-08-02 10:00:00+0000	Zero
	2020-08-02 10:01:00+0000 to 2020-08-02 10:15:00+0000	Span
	2020-08-02 10:16:00+0000 to 2020-08-02 10:30:00+0000	Spare
	2020-08-02 10:31:00+0000 to 2020-08-02 10:35:00+0000	Purge
	2020-08-03 09:46:00+0000 to 2020-08-03 10:00:00+0000	Zero
	2020-08-03 10:01:00+0000 to 2020-08-03 10:15:00+0000	Span
	2020-08-03 10:16:00+0000 to 2020-08-03 10:30:00+0000	Spare
	2020-08-03 10:31:00+0000 to 2020-08-03 10:35:00+0000	Purge
	2020-08-04 09:46:00+0000 to 2020-08-04 10:00:00+0000	Zero

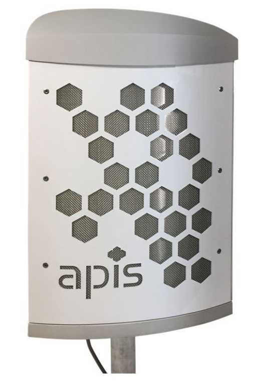

Image Attribution: Apis, Inc (Gants Pass, OR)

Data Flags Recorded During Testing (Cont.)

FRM/FEM Monitor	Timestamp (UTC)	Flag
Teledyne API T265 (Data acquired via local transfer from OAQPS)	2020-08-04 10:01:00+0000 to 2020-08-04 10:15:00+0000	Span
	2020-08-04 10:16:00+0000 to 2020-08-04 10:30:00+0000	Spare
	2020-08-04 10:31:00+0000 to 2020-08-04 10:35:00+0000	Purge
	2020-08-05 09:46:00+0000 to 2020-08-05 10:00:00+0000	Zero
	2020-08-05 10:01:00+0000 to 2020-08-05 10:15:00+0000	Span
	2020-08-05 10:16:00+0000 to 2020-08-05 10:30:00+0000	Spare
	2020-08-05 10:31:00+0000	Calib
	2020-08-05 10:32:00+0000 to 2020-08-05 10:35:00+0000	Purge
	2020-08-06 09:46:00+0000 to 2020-08-06 10:00:00+0000	Zero
	2020-08-06 10:01:00+0000 to 2020-08-06 10:15:00+0000	Span
	2020-08-06 10:16:00+0000 to 2020-08-06 10:30:00+0000	Spare
	2020-08-06 10:31:00+0000 to 2020-08-06 10:35:00+0000	Purge
	2020-08-07 09:46:00+0000 to 2020-08-07 10:00:00+0000	Zero
	2020-08-07 10:01:00+0000 to 2020-08-07 10:15:00+0000	Span
	2020-08-07 10:16:00+0000 to 2020-08-07 10:30:00+0000	Spare
	2020-08-07 10:31:00+0000 to 2020-08-07 10:35:00+0000	Purge
	2020-08-08 09:46:00+0000 to 2020-08-08 10:00:00+0000	Zero
	2020-08-08 10:01:00+0000 to 2020-08-08 10:15:00+0000	Span
	2020-08-08 10:16:00+0000 to 2020-08-08 10:30:00+0000	Spare
	2020-08-08 10:31:00+0000 to 2020-08-08 10:35:00+0000	Purge
	2020-08-09 09:46:00+0000 to 2020-08-09 10:00:00+0000	Zero
	2020-08-09 10:01:00+0000 to 2020-08-09 10:15:00+0000	Span
	2020-08-09 10:16:00+0000 to 2020-08-09 10:30:00+0000	Spare
	2020-08-09 10:31:00+0000 to 2020-08-09 10:35:00+0000	Purge
	2020-08-10 09:46:00+0000 to 2020-08-10 10:00:00+0000	Zero
	2020-08-10 10:01:00+0000 to 2020-08-10 10:15:00+0000	Span
	2020-08-10 10:16:00+0000 to 2020-08-10 10:30:00+0000	Spare
	2020-08-10 10:31:00+0000 to 2020-08-10 10:35:00+0000	Purge
	2020-08-10 20:46:00+0000	<Samp
	2020-08-04 10:01:00+0000 to 2020-08-04 10:15:00+0000	Span
	2020-08-04 10:16:00+0000 to 2020-08-04 10:30:00+0000	Spare
	2020-08-04 10:31:00+0000 to 2020-08-04 10:35:00+0000	Purge
	2020-08-05 09:46:00+0000 to 2020-08-05 10:00:00+0000	Zero
	2020-08-05 10:01:00+0000 to 2020-08-05 10:15:00+0000	Span
	2020-08-05 10:16:00+0000 to 2020-08-05 10:30:00+0000	Spare
	2020-08-05 10:31:00+0000	Calib
	2020-08-05 10:32:00+0000 to 2020-08-05 10:35:00+0000	Purge
	2020-08-06 09:46:00+0000 to 2020-08-06 10:00:00+0000	Zero
	2020-08-06 10:01:00+0000 to 2020-08-06 10:15:00+0000	Span
	2020-08-06 10:16:00+0000 to 2020-08-06 10:30:00+0000	Spare
	2020-08-06 10:31:00+0000 to 2020-08-06 10:35:00+0000	Purge
	2020-08-07 09:46:00+0000 to 2020-08-07 10:00:00+0000	Zero
	2020-08-07 10:01:00+0000 to 2020-08-07 10:15:00+0000	Span
	2020-08-07 10:16:00+0000 to 2020-08-07 10:30:00+0000	Spare
	2020-08-07 10:31:00+0000 to 2020-08-07 10:35:00+0000	Purge
	2020-08-08 09:46:00+0000 to 2020-08-08 10:00:00+0000	Zero

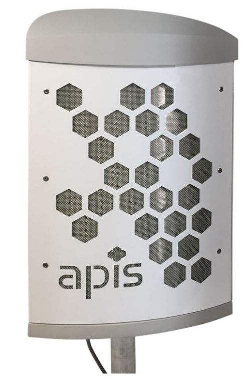

Image Attribution: Apis, Inc (Gants Pass, OR)

Data Flags Recorded During Testing (Cont.)

FRM/FEM Monitor	Timestamp (UTC)	Flag
Teledyne API T265 (Data acquired via local transfer from OAQPS)	2020-08-08 10:01:00+0000 to 2020-08-08 10:15:00+0000	Span
	2020-08-08 10:16:00+0000 to 2020-08-08 10:30:00+0000	Spare
	2020-08-08 10:31:00+0000 to 2020-08-08 10:35:00+0000	Purge
	2020-08-09 09:46:00+0000 to 2020-08-09 10:00:00+0000	Zero
	2020-08-09 10:01:00+0000 to 2020-08-09 10:15:00+0000	Span
	2020-08-09 10:16:00+0000 to 2020-08-09 10:30:00+0000	Spare
	2020-08-09 10:31:00+0000 to 2020-08-09 10:35:00+0000	Purge
	2020-08-10 09:46:00+0000 to 2020-08-10 10:00:00+0000	Zero
	2020-08-10 10:01:00+0000 to 2020-08-10 10:15:00+0000	Span
	2020-08-10 10:16:00+0000 to 2020-08-10 10:30:00+0000	Spare
	2020-08-10 10:31:00+0000 to 2020-08-10 10:35:00+0000	Purge
	2020-08-10 20:46:00+0000	<Samp
	2020-08-08 10:01:00+0000 to 2020-08-08 10:15:00+0000	Span
	2020-08-08 10:16:00+0000 to 2020-08-08 10:30:00+0000	Spare
	2020-08-08 10:31:00+0000 to 2020-08-08 10:35:00+0000	Purge
	2020-08-09 09:46:00+0000 to 2020-08-09 10:00:00+0000	Zero
	2020-08-09 10:01:00+0000 to 2020-08-09 10:15:00+0000	Span
	2020-08-09 10:16:00+0000 to 2020-08-09 10:30:00+0000	Spare
	2020-08-09 10:31:00+0000 to 2020-08-09 10:35:00+0000	Purge
	2020-08-10 09:46:00+0000 to 2020-08-10 10:00:00+0000	Zero
	2020-08-10 10:01:00+0000 to 2020-08-10 10:15:00+0000	Span
	2020-08-10 10:16:00+0000 to 2020-08-10 10:30:00+0000	Spare
	2020-08-10 10:31:00+0000 to 2020-08-10 10:35:00+0000	Purge
	2020-08-10 20:46:00+0000	<Samp

Meteorological Instrument	Timestamp (UTC)	Flag
RM Young 41382 VC Temperature and Relative Humidity Monitor (Data acquired via local transfer from OAQPS)	2020-07-17 20:30:00+0000	<Samp
	2020-07-20 20:56:00+0000	<Samp
	2020-07-20 20:58:00+0000 to 2020-07-20 20:59:00+0000	<Samp
	2020-07-20 21:07:00+0000 to 2020-07-20 21:08:00+0000	<Samp
	2020-07-20 21:14:00+0000 to 2020-07-21 04:18:00+0000	NoData
	2020-07-21 00:31:00+0000 to 2020-07-21 03:45:00+0000	<Samp
	2020-07-21 04:22:00+0000 to 2020-07-21 04:24:00+0000	NoData
	2020-07-21 04:30:00+0000 to 2020-07-21 04:31:00+0000	NoData
	2020-07-21 04:36:00+0000	NoData
	2020-07-21 04:38:00+0000	NoData
	2020-07-21 04:42:00+0000	NoData
	2020-07-21 04:44:00+0000 to 2020-07-21 04:45:00+0000	NoData
	2020-07-21 04:49:00+0000 to 2020-07-21 04:50:00+0000	NoData
	2020-07-21 04:54:00+0000	NoData
	2020-07-21 04:56:00+0000 to 2020-07-21 04:57:00+0000	NoData
	2020-07-21 05:04:00+0000 to 2020-07-21 05:06:00+0000	NoData
	2020-07-21 05:10:00+0000	NoData
	2020-07-21 05:12:00+0000	NoData
	2020-07-21 05:16:00+0000 to 2020-07-21 05:19:00+0000	NoData
	2020-07-21 05:24:00+0000	NoData

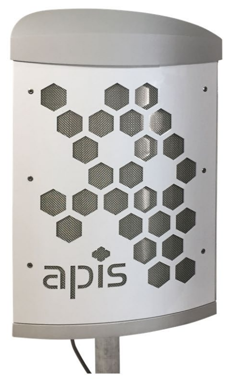

Image Attribution: Apis, Inc (Gants Pass, OR)

Supplemental Information: Description of FRM/FEM QC Checks and Data Flags

Data Flags Recorded During Testing (Cont.)

FRM/FEM Monitor	Timestamp (UTC)	Flag
RM Young 41382 VC Temperature and Relative Humidity Monitor (Data acquired via local transfer from OAQPS)	2020-07-21 05:32:00+0000	NoData
	2020-07-21 05:38:00+0000	NoData
	2020-07-21 05:41:00+0000 to 2020-07-21 05:45:00+0000	NoData
	2020-07-21 05:50:00+0000 to 2020-07-21 05:52:00+0000	NoData
	2020-07-21 05:56:00+0000 to 2020-07-21 05:58:00+0000	NoData
	2020-07-21 06:02:00+0000 to 2020-07-21 06:05:00+0000	NoData
	2020-07-21 06:10:00+0000 to 2020-07-21 06:12:00+0000	NoData
	2020-07-21 06:18:00+0000	NoData
	2020-07-21 06:22:00+0000	NoData
	2020-07-21 06:24:00+0000 to 2020-07-21 06:25:00+0000	NoData
	2020-07-21 06:30:00+0000 to 2020-07-21 06:32:00+0000	NoData
	2020-07-21 06:36:00+0000 to 2020-07-21 06:38:00+0000	NoData
	2020-07-21 06:40:00+0000	NoData
	2020-07-21 06:42:00+0000	NoData
	2020-07-21 06:44:00+0000 to 2020-07-21 06:45:00+0000	NoData
	2020-07-21 06:49:00+0000 to 2020-07-21 06:52:00+0000	NoData
	2020-07-21 06:58:00+0000	NoData
	2020-07-21 07:00:00+0000 to 2020-07-21 07:02:00+0000	NoData
	2020-07-21 07:04:00+0000 to 2020-07-21 07:05:00+0000	NoData
	2020-07-21 07:11:00+0000 to 2020-07-21 07:12:00+0000	NoData
	2020-07-21 07:16:00+0000	NoData
	2020-07-21 07:18:00+0000	NoData
	2020-07-21 07:23:00+0000 to 2020-07-21 07:25:00+0000	NoData
	2020-07-21 07:30:00+0000 to 2020-07-21 07:32:00+0000	NoData
	2020-07-21 07:35:00+0000	NoData
	2020-07-21 07:39:00+0000	NoData
	2020-07-21 07:43:00+0000 to 2020-07-21 07:44:00+0000	NoData
	2020-07-21 07:49:00+0000 to 2020-07-21 07:50:00+0000	NoData
	2020-07-21 07:55:00+0000 to 2020-07-21 07:56:00+0000	NoData
	2020-07-21 07:58:00+0000 to 2020-07-21 07:59:00+0000	NoData
	2020-07-21 08:03:00+0000 to 2020-07-21 08:04:00+0000	NoData
	2020-07-21 08:10:00+0000 to 2020-07-21 08:12:00+0000	NoData
	2020-07-21 08:14:00+0000	NoData
	2020-07-21 08:16:00+0000	NoData
	2020-07-21 08:18:00+0000	NoData
	2020-07-21 08:21:00+0000	NoData
	2020-07-21 08:24:00+0000 to 2020-07-21 08:26:00+0000	NoData
	2020-07-21 08:28:00+0000	NoData
	2020-07-21 08:30:00+0000	NoData
	2020-07-21 08:32:00+0000 to 2020-07-21 08:33:00+0000	NoData
	2020-07-21 08:35:00+0000	NoData
	2020-07-21 08:39:00+0000	NoData
	2020-07-21 08:42:00+0000 to 2020-07-21 08:43:00+0000	NoData
	2020-07-21 08:45:00+0000 to 2020-07-21 08:46:00+0000	NoData
	2020-07-21 08:49:00+0000	NoData
	2020-07-21 08:52:00+0000 to 2020-07-21 08:53:00+0000	NoData
	2020-07-21 08:57:00+0000 to 2020-07-21 08:58:00+0000	NoData

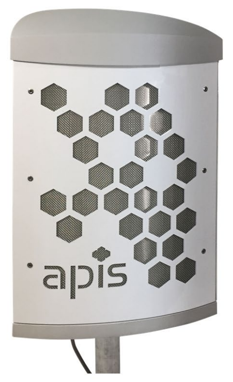

Image Attribution: Apis, Inc (Gants Pass, OR)

Data Flags Recorded During Testing (Cont.)

FRM/FEM Monitor	Timestamp (UTC)	Flag
RM Young 41382 VC Temperature and Relative Humidity Monitor (Data acquired via local transfer from OAQPS)	2020-07-21 09:03:00+0000 to 2020-07-21 09:05:00+0000	NoData
	2020-07-21 09:08:00+0000	NoData
	2020-07-21 09:10:00+0000 to 2020-07-21 09:11:00+0000	NoData
	2020-07-21 09:16:00+0000	NoData
	2020-07-21 09:18:00+0000	NoData
	2020-07-21 09:23:00+0000 to 2020-07-21 09:24:00+0000	NoData
	2020-07-21 09:28:00+0000 to 2020-07-21 09:30:00+0000	NoData
	2020-07-21 09:32:00+0000	NoData
	2020-07-21 09:34:00+0000	NoData
	2020-07-21 09:36:00+0000 to 2020-07-21 09:37:00+0000	NoData
	2020-07-21 09:39:00+0000	NoData
	2020-07-21 09:42:00+0000 to 2020-07-21 09:43:00+0000	NoData
	2020-07-21 09:45:00+0000 to 2020-07-21 09:50:00+0000	NoData
	2020-07-21 09:54:00+0000	NoData
	2020-07-21 09:56:00+0000	NoData
	2020-07-21 10:00:00+0000 to 2020-07-21 10:02:00+0000	NoData
	2020-07-21 10:06:00+0000 to 2020-07-21 10:08:00+0000	NoData
	2020-07-21 10:11:00+0000	NoData
	2020-07-21 10:13:00+0000 to 2020-07-21 10:14:00+0000	NoData
	2020-07-21 10:16:00+0000	NoData
	2020-07-21 10:18:00+0000 to 2020-07-21 10:21:00+0000	NoData
	2020-07-21 10:26:00+0000 to 2020-07-21 10:27:00+0000	NoData
	2020-07-21 10:30:00+0000 to 2020-07-21 10:34:00+0000	NoData
	2020-07-21 10:38:00+0000 to 2020-07-21 10:40:00+0000	NoData
	2020-07-21 10:45:00+0000 to 2020-07-21 10:47:00+0000	NoData
	2020-07-21 10:50:00+0000 to 2020-07-21 10:52:00+0000	NoData
	2020-07-21 10:56:00+0000	NoData
	2020-07-21 11:01:00+0000 to 2020-07-21 11:02:00+0000	NoData
	2020-07-21 11:04:00+0000	NoData
	2020-07-21 11:08:00+0000	NoData
	2020-07-21 11:10:00+0000 to 2020-07-21 11:12:00+0000	NoData
	2020-07-21 11:16:00+0000	NoData
	2020-07-21 11:18:00+0000	NoData
	2020-07-21 11:23:00+0000 to 2020-07-21 11:24:00+0000	NoData
	2020-07-21 11:26:00+0000	NoData
	2020-07-21 11:28:00+0000 to 2020-07-21 11:30:00+0000	NoData
	2020-07-21 11:35:00+0000 to 2020-07-21 11:37:00+0000	NoData
	2020-07-21 11:43:00+0000	NoData
	2020-07-21 11:48:00+0000 to 2020-07-21 11:50:00+0000	NoData
	2020-07-21 11:53:00+0000 to 2020-07-21 11:54:00+0000	NoData
	2020-07-21 11:59:00+0000 to 2020-07-21 12:03:00+0000	NoData
	2020-07-21 12:07:00+0000	NoData
	2020-07-21 12:09:00+0000 to 2020-07-21 12:10:00+0000	NoData
	2020-07-21 12:13:00+0000 to 2020-07-21 12:16:00+0000	NoData
	2020-07-21 12:20:00+0000	NoData
	2020-07-21 12:22:00+0000 to 2020-07-21 12:23:00+0000	NoData
	2020-07-21 12:26:00+0000	NoData

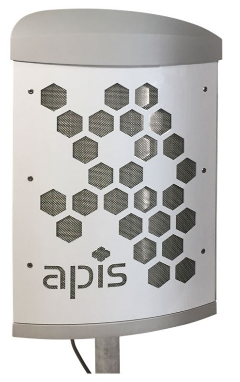

Image Attribution: Apis, Inc (Gants Pass, OR)

Data Flags Recorded During Testing (Cont.)

FRM/FEM Monitor	Timestamp (UTC)	Flag
RM Young 41382 VC Temperature and Relative Humidity Monitor (Data acquired via local transfer from OAQPS)	2020-07-21 12:28:00+0000 to 2020-07-21 12:29:00+0000	NoData
	2020-07-21 12:32:00+0000	NoData
	2020-07-21 12:34:00+0000 to 2020-07-21 12:36:00+0000	NoData
	2020-07-21 12:40:00+0000 to 2020-07-21 12:43:00+0000	NoData
	2020-07-21 12:45:00+0000	NoData
	2020-07-21 12:47:00+0000 to 2020-07-21 12:49:00+0000	NoData
	2020-07-21 12:54:00+0000 to 2020-07-21 12:56:00+0000	NoData
	2020-07-21 12:59:00+0000 to 2020-07-21 13:00:00+0000	NoData
	2020-07-21 13:06:00+0000	NoData
	2020-07-21 13:08:00+0000	NoData
	2020-07-21 13:14:00+0000	NoData
	2020-07-21 13:20:00+0000 to 2020-07-21 13:21:00+0000	NoData
	2020-07-21 13:23:00+0000	NoData
	2020-07-21 13:25:00+0000 to 2020-07-21 13:26:00+0000	NoData
	2020-07-21 13:31:00+0000 to 2020-07-21 13:33:00+0000	NoData
	2020-07-21 13:37:00+0000 to 2020-07-21 13:40:00+0000	NoData
	2020-07-21 13:45:00+0000 to 2020-07-21 13:46:00+0000	NoData
	2020-07-21 13:50:00+0000	NoData
	2020-07-21 13:52:00+0000 to 2020-07-21 13:53:00+0000	NoData
	2020-07-21 13:56:00+0000	NoData
	2020-07-21 13:58:00+0000 to 2020-07-21 13:59:00+0000	NoData
	2020-07-21 14:03:00+0000	NoData
	2020-07-21 14:05:00+0000	NoData
	2020-07-21 14:10:00+0000	NoData
	2020-07-21 14:12:00+0000	NoData
	2020-07-21 14:17:00+0000 to 2020-07-21 14:19:00+0000	NoData
	2020-07-21 14:22:00+0000 to 2020-07-21 14:25:00+0000	NoData
	2020-07-21 14:28:00+0000 to 2020-07-21 14:31:00+0000	NoData
	2020-07-21 14:35:00+0000	NoData
	2020-07-21 14:37:00+0000	NoData
	2020-07-21 14:42:00+0000 to 2020-07-21 14:44:00+0000	NoData
	2020-07-21 14:48:00+0000	NoData
	2020-07-21 14:50:00+0000	NoData
	2020-07-21 14:55:00+0000 to 2020-07-21 14:57:00+0000	NoData
	2020-07-21 15:01:00+0000 to 2020-07-21 15:02:00+0000	NoData
	2020-07-21 15:05:00+0000 to 2020-07-21 15:08:00+0000	NoData
	2020-07-21 15:11:00+0000 to 2020-07-21 15:15:00+0000	NoData
	2020-07-21 15:18:00+0000	NoData
	2020-07-21 15:20:00+0000 to 2020-07-21 15:21:00+0000	NoData
	2020-07-21 15:26:00+0000 to 2020-07-21 15:28:00+0000	NoData
	2020-07-21 15:32:00+0000 to 2020-07-21 15:34:00+0000	NoData
	2020-07-21 15:37:00+0000	NoData
	2020-07-21 15:39:00+0000 to 2020-07-21 15:41:00+0000	NoData
	2020-07-21 15:44:00+0000 to 2020-07-21 15:47:00+0000	NoData
	2020-07-21 15:52:00+0000	NoData
	2020-07-21 15:55:00+0000	NoData
	2020-07-21 15:57:00+0000 to 2020-07-21 16:00:00+0000	NoData

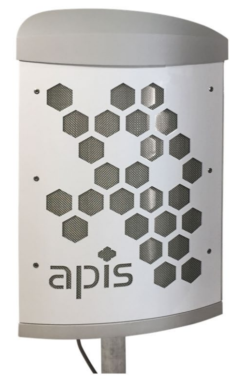

Image Attribution: Apis, Inc (Gants Pass, OR)

Data Flags Recorded During Testing (Cont.)

FRM/FEM Monitor	Timestamp (UTC)	Flag
RM Young 41382 VC Temperature and Relative Humidity Monitor (Data acquired via local transfer from OAQPS)	2020-07-21 16:04:00+0000 to 2020-07-21 16:07:00+0000	NoData
	2020-07-21 16:11:00+0000	NoData
	2020-07-21 16:13:00+0000 to 2020-07-21 16:14:00+0000	NoData
	2020-07-21 16:18:00+0000	NoData
	2020-07-21 16:20:00+0000 to 2020-07-21 16:21:00+0000	NoData
	2020-07-21 16:25:00+0000 to 2020-07-21 16:26:00+0000	NoData
	2020-07-21 16:28:00+0000	NoData
	2020-07-21 16:34:00+0000 to 2020-07-21 16:36:00+0000	NoData
	2020-07-21 16:42:00+0000 to 2020-07-21 16:44:00+0000	NoData
	2020-07-21 16:48:00+0000 to 2020-07-21 16:50:00+0000	NoData
	2020-07-21 16:54:00+0000 to 2020-07-21 16:59:00+0000	<Samp
	2020-07-21 16:55:00+0000 to 2020-07-21 16:58:00+0000	NoData
	2020-07-30 14:36:00+0000	<Samp
	2020-07-30 18:08:00+0000 to 2020-07-30 18:12:00+0000	NoData
	2020-07-30 18:13:00+0000	<Samp
	2020-07-30 18:31:00+0000 to 2020-07-30 18:34:00+0000	NoData
	2020-07-30 18:35:00+0000	<Samp
	2020-07-30 20:04:00+0000	<Samp
	2020-08-10 20:46:00+0000	<Samp

Testing Report - O_3 Base Testing

Myriad Sensors PocketLab Air

This report reflects out-of-the-box performance

Initial Base Testing - RTP, NC
U.S. Environmental Protection Agency
Office of Research and Development
PI: Clements.Andrea@epa.gov
919-541-1363

March 2021—April 2021

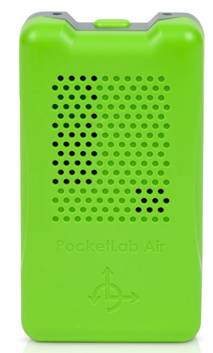

Image Attribution: Myriad Sensors, Inc. (San Jose, CA)

Deployment Details

Testing Organization and Site Information	
Testing organization (Name, Organization type, Contact website)	U.S. Environmental Protection Agency - Office of Research and Development<br>Federal Government<br>Air Sensor Toolbox | U.S. EPA Website
Testing location (City, State, Latitude and Longitude)	Ambient Monitoring Innovative Research Station (AIRS)<br>RTP, NC<br>35.88951, -78.874572
AQS site ID	37 – 063 – 0099
Sampling timeframe (MM-DD-YY)	03-17-21 to 04-19-21
Sensor data source	Onboard flash memory card
Reference data source	OAQPS file transfer

Sensor Information			
Manufacturer, model	Myriad Sensors PocketLab Air		
Device firmware version			
Sampling time interval	1-minute		
Sensor serial numbers	E0	EC	F1
Issues encountered during deployment?	☐	No Issues	

FRM/FEM Information	
Manufacturer, model, designation	Teledyne API T265 FEM
Sampling time interval	1-hour averaging
Date of calibration	As required by 40 CFR Part 58 and the Burdens Creek QAPP maintained by OAQPS
Date of one-point QC check	Every two weeks as required by 40 CFR Part 58 Appendix A 3.1.1
Description, date(s) of maintenance activities	N/A

Time Series Plot: 1-hour averaged O_3

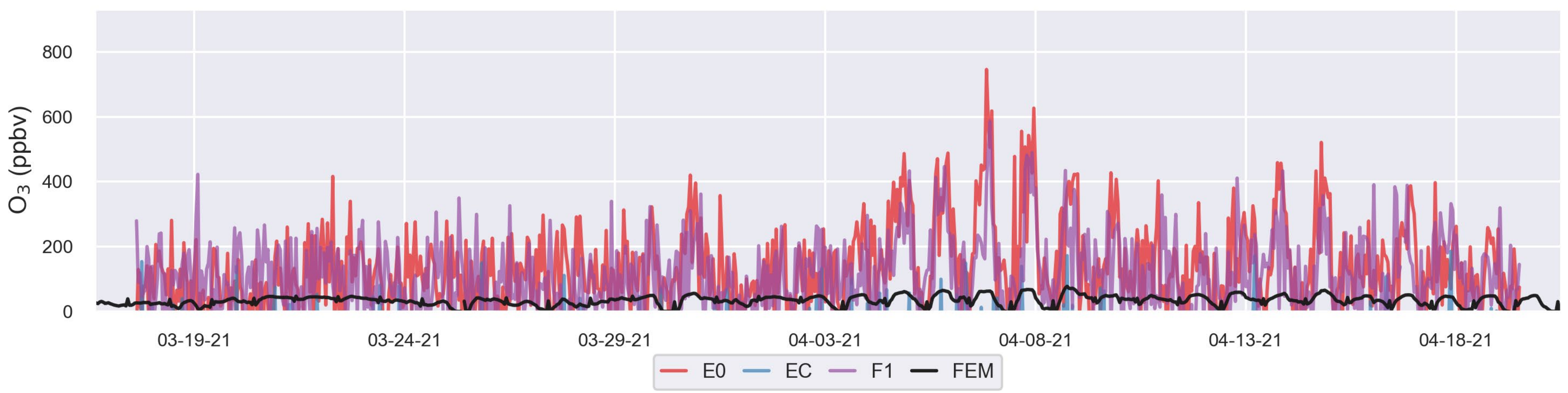

Range and average of FRM/FEM concentrations over duration of base test (ppbv)	[1-hr] -0.0-77.5, avg: 33.9,<br>[Rolling 8-hr] 1.9-70.1, avg: 37.5
Number of 1-hr periods in FRM/FEM monitor measurements with a goal concentration ≥ 60 ppbv	34

Scatter Plot: Comparison to FRM/FEM

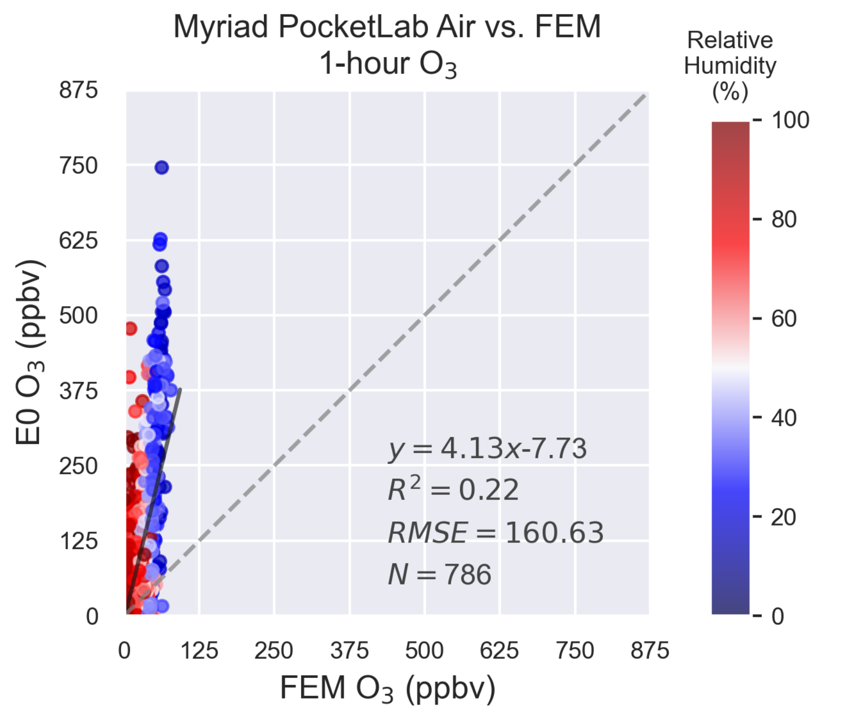

Performance Metrics★

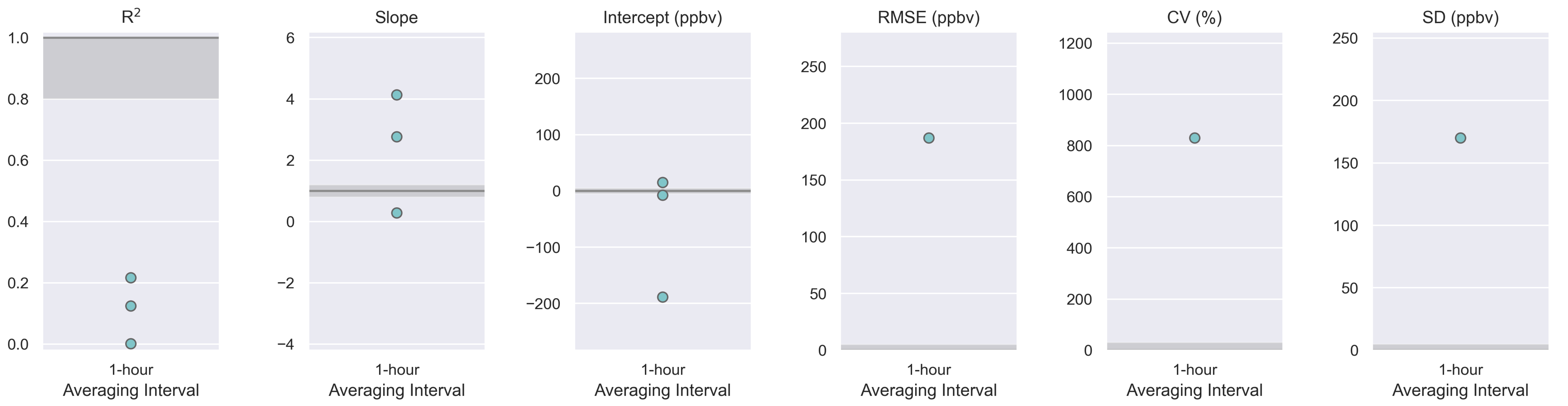

Meteorological Conditions During Deployment

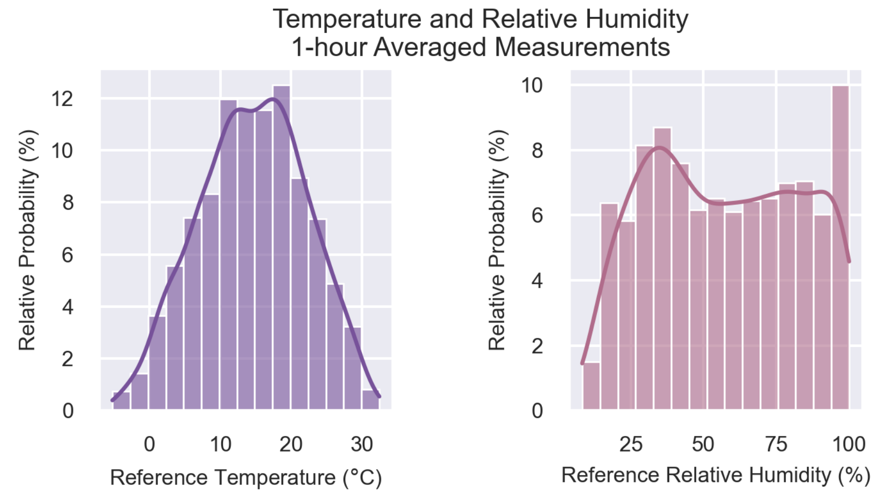

Number of 1-hr periods outside sensor manufacturer-listed temperature operational range (no operational range specified)	-
Number of 1-hr periods outside sensor manufacturer-listed relative humidity operational range (no operational range specified)	-

Meteorological Influence

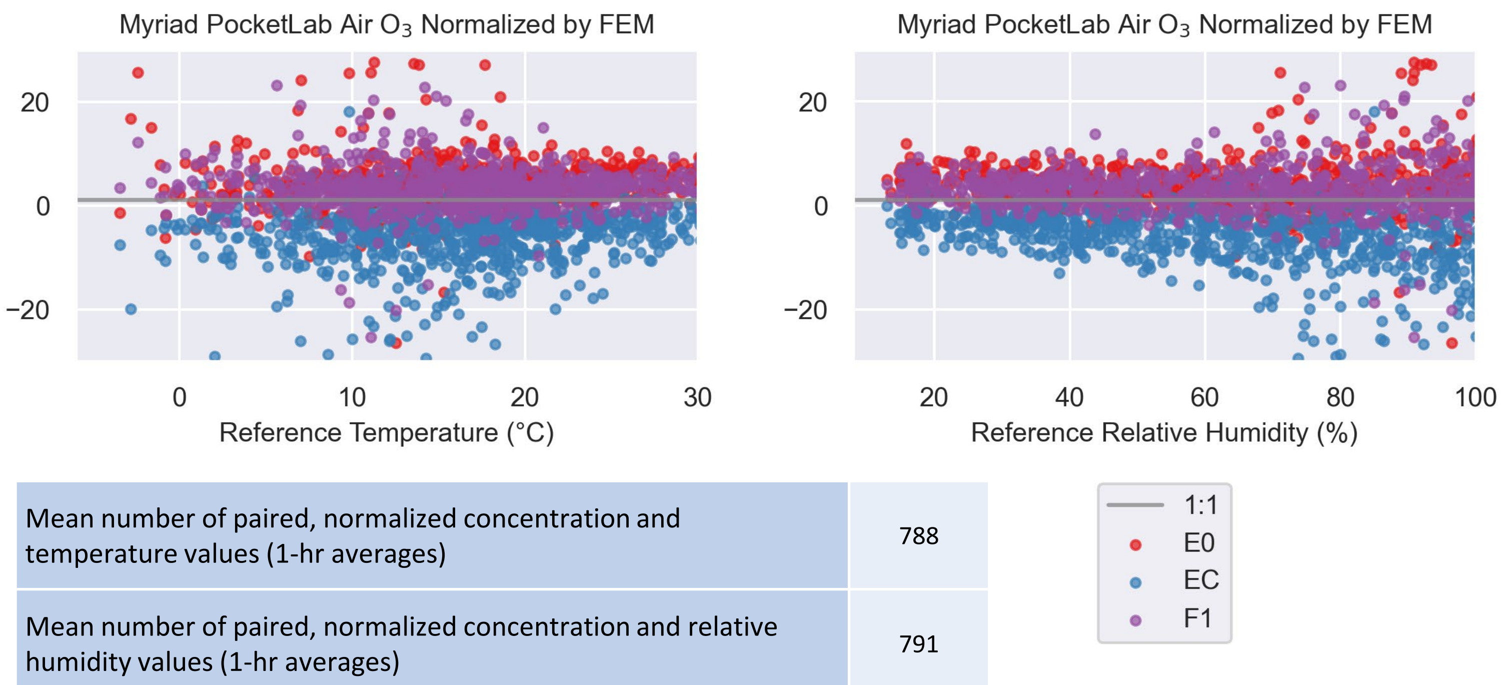

Mean number of paired, normalized concentration and temperature values (1-hr averages)	788
Mean number of paired, normalized concentration and relative humidity values (1-hr averages)	791

★For evaluations with greater than three sensors, grouping individual sensor metrics into boxplots is recommended for displaying results. Note that this recommendation does not apply to metrics computed as a single value for all sensors over the whole evaluation group, such as RMSE, NRMSE, CV, and standard deviation.

Testing Report - O_3 Base Testing

Myriad Sensors PocketLab Air

This report reflects out-of-the-box performance

Initial Base Testing - RTP, NC

U.S. Environmental Protection Agency
Office of Research and Development
PI: Clements.Andrea@epa.gov
919-541-1363
March 2021—April 2021

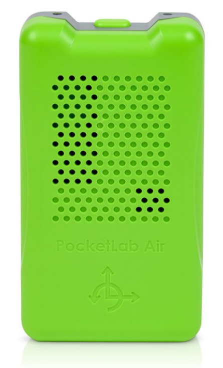

Image Attribution: Myriad Sensors, Inc. (San Jose, CA)

Tabular Statistics

Sensor-FRM/FEM Correlation

	Bias and Linearity			Data Quality	
	R^2	Slope	Intercept (ppbv)	Uptime (%)	Number of paired sensor and reference concentration values*
	1-Hour ○○○	1-Hour ○○○	1-Hour ○○○	1-Hour ●●●	1-Hour
Metric Target Range	≥ 0.80	1.0 ± 0.20	-5 ≤ b ≤ 5	75%*	-
Sensor E0	0.22	4.13	-7.73	100	786
Sensor EC	0.00	0.28	-188.83	100	786
Sensor F1	0.12	2.76	14.94	100	784
Mean	0.11	2.39	-60.54	99.79	785.33

	Error
	RMSE (ppbv)
	1-Hour ☆
Metric Target Range	≤ 5.0
Deployment Value	187.1

Device-specific metrics (computed for each sensor in evaluation)
○○○ Metric value for none of devices tested falls within the target range
●○○ Metric value for one of devices tested falls within the target range
●●○ Metric value for two of devices tested falls within the target range
●●● Metric value for three of devices tested falls within the target range

Single-valued metrics (computed via entire evaluation dataset)
☆ Indicates that the metric value is not within the target range
★ Indicates that the metric value is within the target range

Sensor-Sensor Precision

	Precision (between collocated sensors)		Data Quality
	CV (%)	SD (ppbv)	Number of paired sensor and reference concentration values*
	1-Hour ☆	1-Hour ☆	1-Hour
Metric Target Range	≤ 30.0	≤ 5.0	-
Deployment Value	830.1	170.1	788

*This value is only a recommendation for ensuring data quality and is not included in the list of target values discussed in Section 4 of the Performance Testing Protocols, Metrics, and Target Values for Ozone Air Sensors document.

Testing Report - O_3 Base Testing

Myriad Sensors PocketLab Air

This report reflects out-of-the-box performance

Initial Base Testing - RTP, NC
U.S. Environmental Protection Agency
Office of Research and Development
PI: Clements.Andrea@epa.gov
919-541-1363
March 2021—April 2021

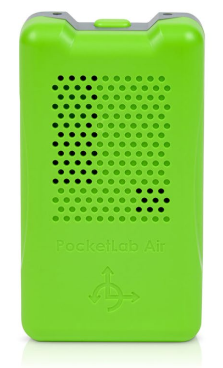
Image Attribution: Myriad Sensors, Inc. (San Jose, CA)

Sensor-FRM/FEM Scatter Plots

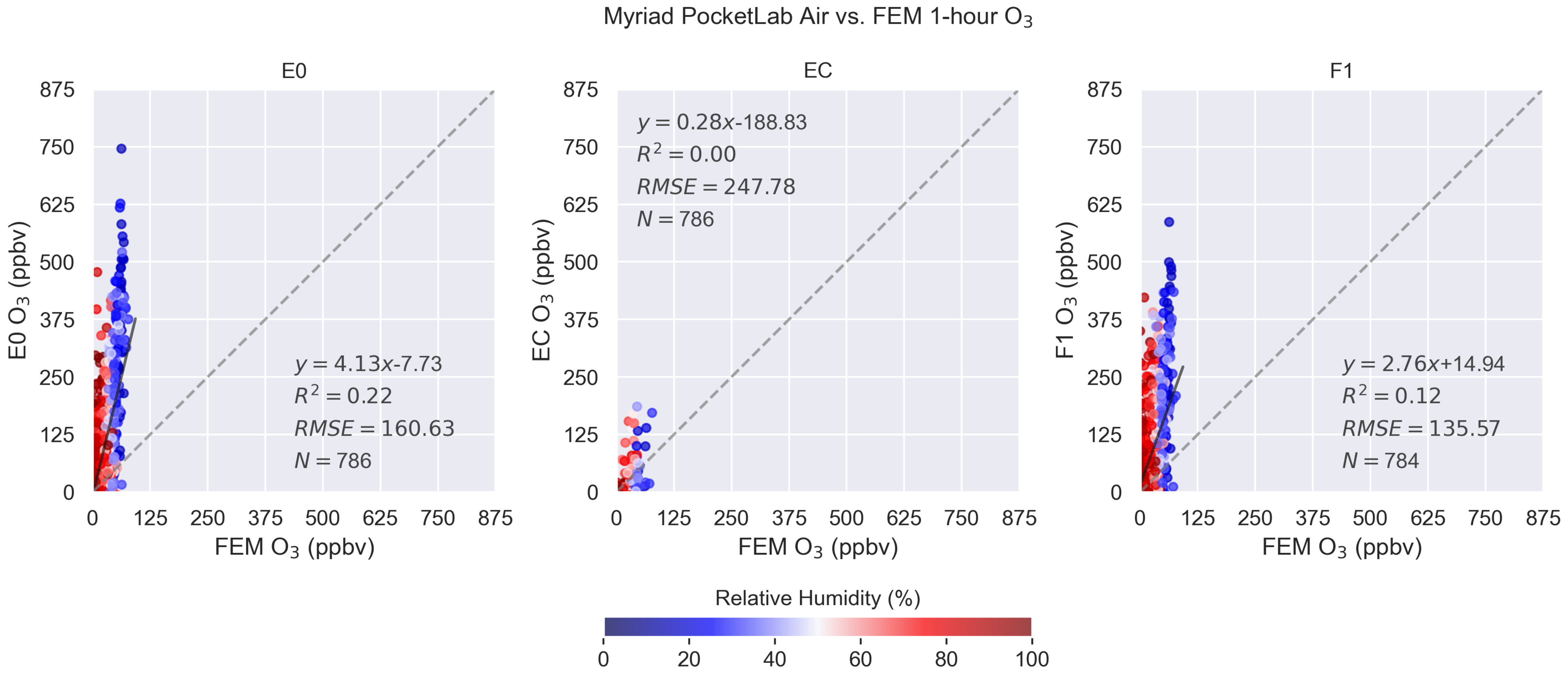

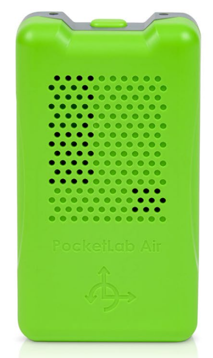

Image Attribution: Myriad Sensors, Inc. (San Jose, CA)

Supplemental Information

Abbreviations used in Supplemental Information

FRM	Federal Reference Method
FEM	Federal Equivalent Method
SOP	Standard Operating Procedure
QAPP	Quality Assurance Project Plan
QC	Quality Control

Supplemental Documentation	Attached	Description & URL or file path to documentation
Field observations and sensor data flags	☑	See NC-PKT-Page 6 of this testing report
Maintenance logs	☑	No logs recorded during testing
Standard operating procedure(s)	☐	U.S. EPA Office Of Research and Development SOP available upon request
Photos of equipment setup and testing	☑	See NC-PKT-Page 5 of this testing report
Product specifications sheet(s)	☐	See Appendix C, "Spec_Sheet_Myriad_PocketLab_Air.pdf"
Product manual(s)	☐	N/A
Data storage and transmission method	☑	See NC-PKT-Page 6 of this testing report
Data correction approach	☑	See NC-PKT-Page 6 of this testing report
Issues encountered	☑	See NC-PKT-Page 6 of this testing report
Data analysis/correction scripts and version	☑	Averaging and processing of data, calculation of performance metrics, and generation of figures and other supplementary material for analysis were obtained using Python 3.9.7 with the packages sensortoolkit v0.8.3b2, pandas 1.3.5, NumPy 1.21.2, Matplotlib 3.5.0, statsmodels 0.13.0, and seaborn 0.11.2. All packages are available from the Python Package Index (PyPI) at https://pypi.org. The integrated development environment (IDE) Spyder 5.1.5 was used for scripting and data visualization. Version control for the Python base, packages, and IDE were all managed by conda 4.11.0.
Air Monitoring Station QAPP	☐	U.S. EPA Office Of Research and Development QAPP available upon request
Summary of FRM/FEM monitor QC checks	☑	See NC-PKT-Pages 7-9 of this testing report
Manufacturer website for FRM/FEM monitor	☑	Teledyne API: T265 Product website
FRM/FEM monitor manual	☑	See Appendix B, "Spec_Sheet_TeledyneAPI_T265.pdf"
FRM/FEM monitor specifications sheet(s)	☑	See Appendix B, "Manual_TeledyneAPI_T265.pdf"
Other documents	☐	

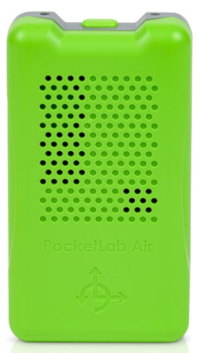

Image Attribution: Myriad Sensors, Inc. (San Jose, CA)

Supplemental Information: Photos of Testing Site and Equipment Setup

Site Description:

The Burdens Creek Ambient Monitoring Innovation Research Station (AIRS) site is located on the U.S. EPA, RTP campus and is situated between Alexander Drive and Route 147. The site is intended to represent a neighborhood-scale site as defined in *40 CFR Part 58, Appendix D*. U.S. EPA's Office of Air Quality Planning and Standards (OAQPS) operates reference grade instruments in a free-standing shelter situated directly adjacent to the sensor testing platform.

Figure 1 (a): Metal enclosure used to store sensors during testing. The enclosure contains louvered siding and vented flooring to allow free air flow.

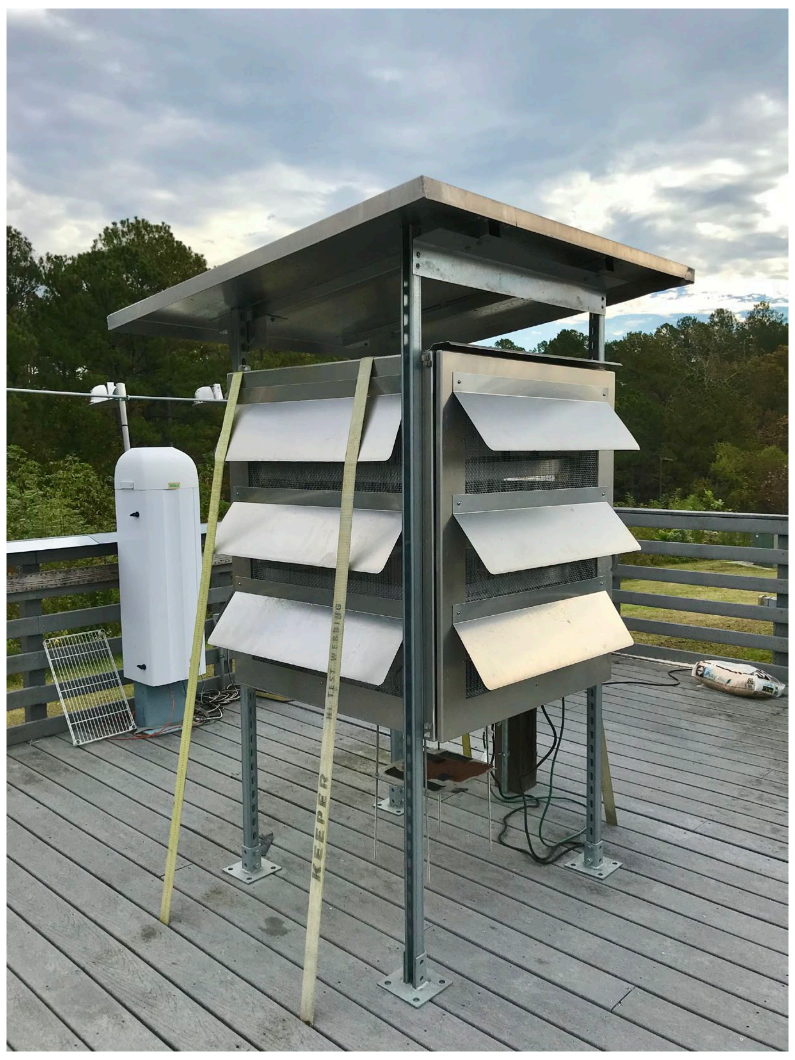

Figure 1 (b): PocketLab Air sensors situated inside the metal enclosure. The enclosure ensures that non-weatherized devices are protected from the elements during ambient testing.

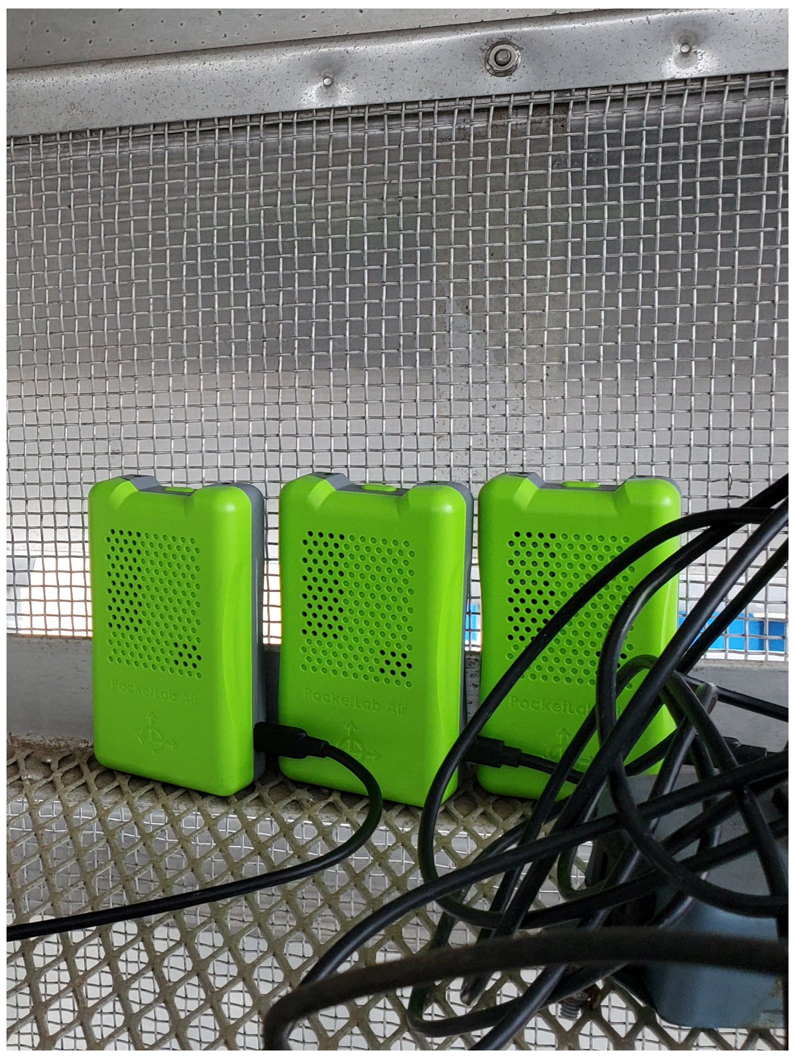

Figure 2: Observation deck at evaluation site. Approximate location of sensors indicated by the red arrow. The approximate location of FEM (housed within sampling trailer) indicated by the black arrow. Sensors and FEM instrumentation are separated by approximately 15 meters.

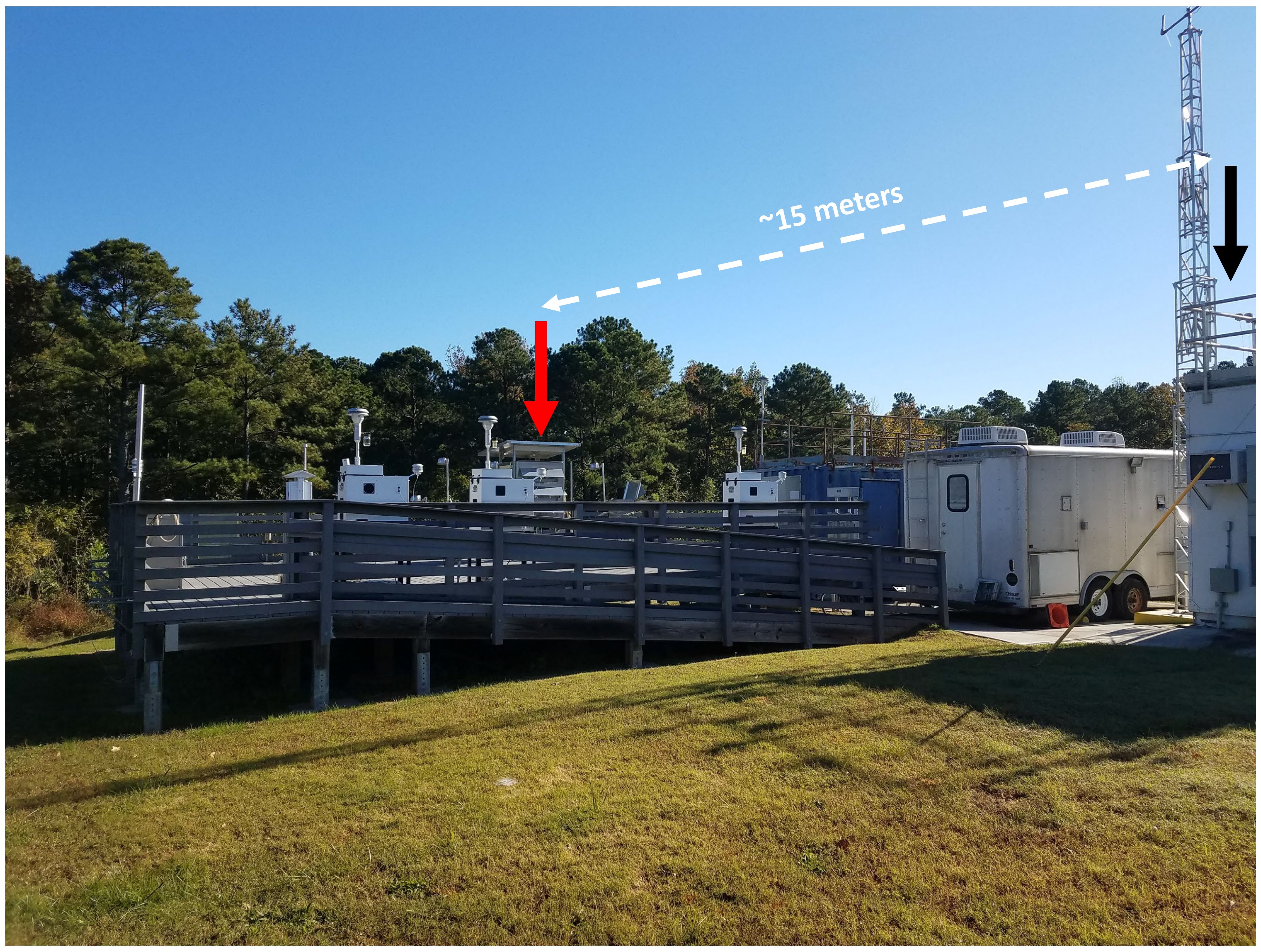

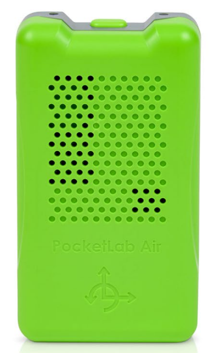

Image Attribution: Myriad Sensors, Inc. (San Jose, CA)

Supplemental Information: Data Storage, Correction Approach, and Issues Encountered

Data Storage and Transmission Method

The PocketLab Air records data at 1-second intervals by default. For this study, units were configured to log data at 1-minute intervals. Devices were operated in 'Memory mode' and data were stored on an internal flash memory card. Due to limited onboard storage (vendor quotes onboard storage size as 30,000 data points), frequent visits (every 2-3 days) to the evaluation site were required by field technicians to retrieve data. Data were downloaded via the PocketLab Air web app which requires a Bluetooth connection between the sensor and computer.

Data Correction Approach

This evaluation report reflects "out-of-the-box" performance of the PocketLab Air. After acquisition, the raw data was processed using the *sensortoolkit* python code library (v0.8.3b2). A continuous data set at the recorded sampling frequency was written to a .csv file. 1-hour averaged data sets were generated using a 75% completeness threshold and saved as separate .csv files. Outliers were **not** removed from data sets in order to assess "out-of-the-box" sensor performance.

The PocketLab Air internal O_3 sensor did not exhibit a distinct warm-up period. Upon inspection of concentration timeseries for each sensor unit, values logged near initial power up are consistent with the concentration range and variability found in each unit's subsequent measurements

Issues Encountered

<u>Pre-deployment observations</u>

- *Limited Documentation and challenges with web app:* Documentation provided by the vendor was very limited, and often had ambiguities that required vendor clarification. The web app, which is required to operate the device and download data, was also an issue on occasion, as it was updated frequently *(by the vendor)* and without notice. Continued application issues resulted in numerous evaluation delays. Most app issues were resolved by the vendor within 1-2 weeks.
- *Inaccurate and limited time information logged by sensor:* The PocketLab Air records elapsed seconds in logged datafiles alongside with time that always starts at 7:00 PM regardless of when data collection is initiated. To evaluate any data properly, the time must be recorded using a reliable source when the data collection is started. The elapsed seconds are then used with the recorded time to calculate a timestamp.
- *Limited onboard memory:* If the PocketLab Air is configured to sample all parameters (temperature, barometric pressure, relative humidity, light, dew point, heat index, O_3, CO_2, PM_1, $PM_{2.5}$, PM_{10}, and the air quality index), the device memory can only hold around 2.5 days of data when recording at 1-minute intervals.

<u>Field observations and sensor data flags</u>

PocketLab Air units were deployed inside the weatherized shelter at AIRS on 3/17/2021. Each unit was deployed in a vertical position to avoid a buildup of water and debris in the inlet/outlet holes. Software bugs with the PocketLab web app made data collections difficult, especially when collections were needed so frequently. The units were retrieved from the field on 4/19/2021. The following table contains data flags describing events that were encountered during the testing period.

Start Time (UTC)	End Time (UTC)	Sensor Serial ID(s)	Parameters Impacted	Flag
2021-03-18 17:02:00+0000	2021-03-18 17:10:00+0000	E0, EC, F1	ALL	Operator working near device (scheduled site visit).
2021-03-23 12:35:00+0000	2021-03-23 12:40:00+0000	E0, EC, F1	ALL	Operator working near device (scheduled site visit).
2021-03-24 19:16:00+0000	2021-03-24 19:17:00+0000	E0, EC, F1	ALL	Operator working near device (scheduled site visit).
2021-03-29 12:11:00+0000	2021-03-29 12:15:00+0000	E0, EC, F1	ALL	Operator working near device (scheduled site visit).
2021-03-31 18:55:00+0000	2021-03-31 18:57:00+0000	E0, EC, F1	ALL	Operator working near device (scheduled site visit).
2021-04-05 18:53:00+0000	2021-04-05 18:56:00+0000	E0, EC, F1	ALL	Operator working near device (scheduled site visit).
2021-04-06 19:38:00+0000	2021-04-06 19:39:00+0000	E0, EC, F1	ALL	Operator working near device (scheduled site visit).
2021-04-07 19:32:00+0000	2021-04-07 19:33:00+0000	E0, EC, F1	ALL	Operator working near device (scheduled site visit).
2021-04-09 18:05:00+0000	2021-04-09 18:07:00+0000	E0, EC, F1	ALL	Operator working near device (scheduled site visit).
2021-04-12 19:01:00+0000	2021-04-12 19:04:00+0000	E0, EC, F1	ALL	Operator working near device (scheduled site visit).
2021-04-13 19:04:00+0000	2021-04-13 19:05:00+0000	E0, EC, F1	ALL	Operator working near device (scheduled site visit).
2021-04-16 18:24:00+0000	2021-04-16 18:30:00+0000	E0, EC, F1	ALL	Operator working near device (scheduled site visit).

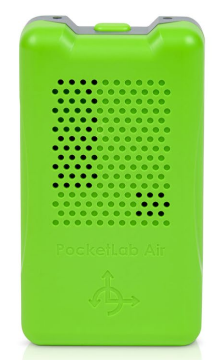

Image Attribution: Myriad Sensors, Inc. (San Jose, CA)

Description of Data Flags

OAQPS manages data logged by reference monitors at the AIRS evaluation site using the Envidas data acquisition system software from DR DAS LTD[1]. Envidas contains over 100 data flags which are configurable by the monitoring agency and can be triggered based on instrument status. Appendix B Table 1 contains a description of Envidas data flags including the numeric code and status code name (a brief textual description, the table containing data flags below includes code names for events encountered by the reference monitor under the "Flag" column). Appendix B Table 1 also indicates whether reference data are invalidated for a given data flag. For this report, reference data were invalidated for periods where a data flag was logged and if the corresponding entry for the data flag in the "Data Status Invalidation" column in Appendix B Table 1 indicates data should be invalidated.

Data Flags Recorded During Testing

FRM/FEM Monitor	Timestamp (UTC)	Flag
Teledyne API T265 (Data acquired via local transfer from OAQPS)	2021-03-17 18:54:00+0000	<Samp
	2021-03-18 09:47:00+0000 to 2021-03-18 10:01:00+0000	Zero
	2021-03-18 10:02:00+0000 to 2021-03-18 10:16:00+0000	Span
	2021-03-18 10:17:00+0000 to 2021-03-18 10:31:00+0000	Span1
	2021-03-18 10:32:00+0000 to 2021-03-18 10:36:00+0000	Purge
	2021-03-18 12:36:00+0000	OK_RS232
	2021-03-18 12:44:00+0000	OK_RS232
	2021-03-18 13:12:00+0000	OK_RS232
	2021-03-18 13:39:00+0000	OK_RS232
	2021-03-18 19:05:00+0000	OK_RS232
	2021-03-18 20:08:00+0000	OK_RS232
	2021-03-18 20:53:00+0000	OK_RS232
	2021-03-18 21:53:00+0000	OK_RS232
	2021-03-19 03:54:00+0000	OK_RS232
	2021-03-19 03:57:00+0000	OK_RS232
	2021-03-19 04:03:00+0000	OK_RS232
	2021-03-19 04:42:00+0000	OK_RS232
	2021-03-19 05:28:00+0000	OK_RS232
	2021-03-19 05:35:00+0000	OK_RS232
	2021-03-19 06:09:00+0000	OK_RS232
	2021-03-19 09:47:00+0000 to 2021-03-19 10:01:00+0000	Zero
	2021-03-19 10:02:00+0000 to 2021-03-19 10:16:00+0000	Span
	2021-03-19 10:17:00+0000 to 2021-03-19 10:31:00+0000	Span1
	2021-03-19 10:32:00+0000 to 2021-03-19 10:36:00+0000	Purge
	2021-03-19 11:16:00+0000	OK_RS232
	2021-03-19 11:31:00+0000	OK_RS232
	2021-03-19 12:03:00+0000	OK_RS232
	2021-03-19 12:09:00+0000	OK_RS232
	2021-03-19 12:16:00+0000	OK_RS232
	2021-03-19 12:48:00+0000	<Samp

[1] "Contact us" DR DAS LTD, 2022, https://dr-das.com/Home/Contact.html Accessed June 3 2022

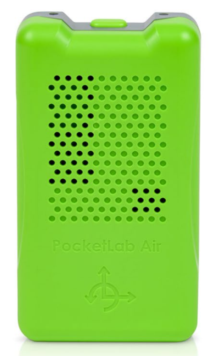

Image Attribution: Myriad Sensors, Inc. (San Jose, CA)

Data Flags Recorded During Testing (Cont.)

FRM/FEM Monitor	Timestamp (UTC)	Flag
Teledyne API T265 (Data acquired via local transfer from OAQPS)	2021-03-19 14:09:00+0000	OK_RS232
	2021-03-19 15:04:00+0000	OK_RS232
	2021-03-19 15:26:00+0000	OK_RS232
	2021-03-19 15:42:00+0000	OK_RS232
	2021-03-19 15:54:00+0000	OK_RS232
	2021-03-19 16:16:00+0000	OK_RS232
	2021-03-19 17:12:00+0000	OK_RS232
	2021-03-19 22:47:00+0000	OK_RS232
	2021-03-19 22:54:00+0000	OK_RS232
	2021-03-19 23:12:00+0000	OK_RS232
	2021-03-19 23:35:00+0000	OK_RS232
	2021-03-19 23:38:00+0000	OK_RS232
	2021-03-19 23:54:00+0000	OK_RS232
	2021-03-20 00:04:00+0000	OK_RS232
	2021-03-20 00:07:00+0000	OK_RS232
	2021-03-20 00:26:00+0000	OK_RS232
	2021-03-20 00:37:00+0000	OK_RS232
	2021-03-20 00:42:00+0000	OK_RS232
	2021-03-20 00:45:00+0000	OK_RS232
	2021-03-20 01:30:00+0000	OK_RS232
	2021-03-20 01:36:00+0000	OK_RS232
	2021-03-20 09:47:00+0000 to 2021-03-20 10:01:00+0000	Zero
	2021-03-20 10:02:00+0000 to 2021-03-20 10:16:00+0000	Span
	2021-03-20 10:17:00+0000 to 2021-03-20 10:31:00+0000	Span1
	2021-03-20 10:32:00+0000 to 2021-03-20 10:36:00+0000	Purge
	2021-03-21 09:47:00+0000 to 2021-03-21 10:01:00+0000	Zero
	2021-03-21 10:02:00+0000 to 2021-03-21 10:16:00+0000	Span
	2021-03-21 10:17:00+0000 to 2021-03-21 10:31:00+0000	Span1
	2021-03-21 10:32:00+0000 to 2021-03-21 10:36:00+0000	Purge
	2021-03-22 09:47:00+0000 to 2021-03-22 10:01:00+0000	Zero
	2021-03-22 10:02:00+0000 to 2021-03-22 10:16:00+0000	Span
	2021-03-22 10:17:00+0000 to 2021-03-22 10:31:00+0000	Span1
	2021-03-22 10:32:00+0000 to 2021-03-22 10:36:00+0000	Purge
	2021-03-23 09:47:00+0000 to 2021-03-23 10:01:00+0000	Zero
	2021-03-23 10:02:00+0000 to 2021-03-23 10:16:00+0000	Span
	2021-03-23 10:17:00+0000 to 2021-03-23 10:31:00+0000	Span1
	2021-03-23 10:32:00+0000 to 2021-03-23 10:36:00+0000	Purge
	2021-03-23 16:40:00+0000 to 2021-03-23 16:46:00+0000	<Samp
	2021-03-23 16:41:00+0000 to 2021-03-23 16:45:00+0000	OffScan
	2021-03-24 09:47:00+0000 to 2021-03-24 10:01:00+0000	Zero
	2021-03-24 10:02:00+0000 to 2021-03-24 10:16:00+0000	Span
	2021-03-24 10:17:00+0000 to 2021-03-24 10:31:00+0000	Span1
	2021-03-24 10:32:00+0000 to 2021-03-24 10:36:00+0000	Purge
	2021-03-24 22:14:00+0000	OK_RS232
	2021-03-25 09:29:00+0000	OK_RS232
	2021-03-25 09:33:00+0000	OK_RS232
	2021-03-25 09:47:00+0000 to 2021-03-25 10:01:00+0000	Zero

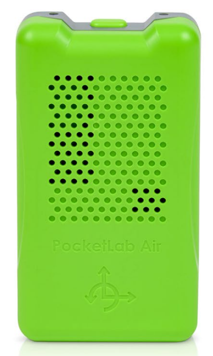

Image Attribution: Myriad Sensors, Inc. (San Jose, CA)

Data Flags Recorded During Testing (Cont.)

FRM/FEM Monitor	Timestamp (UTC)	Flag
Teledyne API T265 (Data acquired via local transfer from OAQPS)	2021-03-25 10:02:00+0000 to 2021-03-25 10:16:00+0000	Span
	2021-03-25 10:17:00+0000 to 2021-03-25 10:31:00+0000	Span1
	2021-03-25 10:32:00+0000 to 2021-03-25 10:36:00+0000	Purge
	2021-03-25 17:12:00+0000	OK_RS232
	2021-03-25 17:19:00+0000	OK_RS232
	2021-03-25 17:58:00+0000	OK_RS232
	2021-03-25 18:03:00+0000	OK_RS232
	2021-03-25 18:31:00+0000	OK_RS232
	2021-03-25 19:02:00+0000	OK_RS232
	2021-03-26 01:52:00+0000	OK_RS232
	2021-03-26 03:08:00+0000	OK_RS232
	2021-03-26 03:22:00+0000	OK_RS232
	2021-03-26 03:25:00+0000	OK_RS232
	2021-03-26 03:33:00+0000	OK_RS232
	2021-03-26 04:25:00+0000	OK_RS232
	2021-03-26 07:35:00+0000	OK_RS232
	2021-03-26 07:39:00+0000	OK_RS232
	2021-03-26 07:53:00+0000	OK_RS232
	2021-03-26 08:40:00+0000	OK_RS232
	2021-03-26 09:47:00+0000 to 2021-03-26 10:01:00+0000	Zero
	2021-03-26 10:02:00+0000 to 2021-03-26 10:16:00+0000	Span
	2021-03-26 10:17:00+0000 to 2021-03-26 10:31:00+0000	Span1
	2021-03-26 10:32:00+0000 to 2021-03-26 10:36:00+0000	Purge
	2021-03-26 16:40:00+0000	OK_RS232
	2021-03-26 16:43:00+0000	OK_RS232
	2021-03-26 16:45:00+0000	OK_RS232
	2021-03-26 16:49:00+0000	OK_RS232
	2021-03-26 16:52:00+0000	OK_RS232
	2021-03-26 17:05:00+0000	OK_RS232
	2021-03-26 17:17:00+0000	OK_RS232
	2021-03-26 17:23:00+0000	OK_RS232
	2021-03-26 17:28:00+0000	OK_RS232
	2021-03-26 17:30:00+0000	OK_RS232
	2021-03-26 17:34:00+0000	OK_RS232
	2021-03-26 17:45:00+0000 to 2021-03-26 17:46:00+0000	OK_RS232
	2021-03-26 17:50:00+0000	<Samp
	2021-03-26 19:07:00+0000	OK_RS232
	2021-03-26 19:38:00+0000	OK_RS232
	2021-03-26 19:58:00+0000 to 2021-03-26 19:59:00+0000	OK_RS232
	2021-03-26 20:07:00+0000 to 2021-03-26 20:08:00+0000	OK_RS232
	2021-03-26 20:12:00+0000	OK_RS232
	2021-03-26 20:17:00+0000	OK_RS232
	2021-03-26 20:40:00+0000	OK_RS232
	2021-03-26 20:45:00+0000	OK_RS232
	2021-03-26 20:50:00+0000	OK_RS232
	2021-03-26 20:55:00+0000	OK_RS232
	2021-03-26 20:57:00+0000	OK_RS232

Data Flags Recorded During Testing (Cont.)

FRM/FEM Monitor	Timestamp (UTC)	Flag
Teledyne API T265 (Data acquired via local transfer from OAQPS)	2021-03-26 21:00:00+0000	OK_RS232
	2021-03-26 21:08:00+0000 to 2021-03-26 21:09:00+0000	OK_RS232
	2021-03-26 21:13:00+0000	OK_RS232
	2021-03-26 21:18:00+0000	OK_RS232
	2021-03-26 21:21:00+0000	OK_RS232
	2021-03-26 21:32:00+0000	OK_RS232
	2021-03-26 22:35:00+0000	OK_RS232
	2021-03-26 22:44:00+0000	OK_RS232
	2021-03-26 23:04:00+0000	OK_RS232
	2021-03-26 23:08:00+0000	OK_RS232
	2021-03-27 03:55:00+0000	OK_RS232
	2021-03-27 04:11:00+0000	OK_RS232
	2021-03-27 09:47:00+0000 to 2021-03-27 10:01:00+0000	Zero
	2021-03-27 10:02:00+0000 to 2021-03-27 10:16:00+0000	Span
	2021-03-27 10:17:00+0000 to 2021-03-27 10:31:00+0000	Span1
	2021-03-27 10:32:00+0000 to 2021-03-27 10:36:00+0000	Purge
	2021-03-28 09:47:00+0000 to 2021-03-28 10:01:00+0000	Zero
	2021-03-28 10:02:00+0000 to 2021-03-28 10:16:00+0000	Span
	2021-03-28 10:17:00+0000 to 2021-03-28 10:31:00+0000	Span1
	2021-03-28 10:32:00+0000 to 2021-03-28 10:36:00+0000	Purge
	2021-03-29 09:47:00+0000 to 2021-03-29 10:01:00+0000	Zero
	2021-03-29 10:02:00+0000 to 2021-03-29 10:16:00+0000	Span
	2021-03-29 10:17:00+0000 to 2021-03-29 10:31:00+0000	Span1
	2021-03-29 10:32:00+0000 to 2021-03-29 10:36:00+0000	Purge
	2021-03-30 09:47:00+0000 to 2021-03-30 10:01:00+0000	Zero
	2021-03-30 10:02:00+0000 to 2021-03-30 10:16:00+0000	Span
	2021-03-30 10:17:00+0000 to 2021-03-30 10:31:00+0000	Span1
	2021-03-30 10:32:00+0000 to 2021-03-30 10:36:00+0000	Purge
	2021-03-30 13:37:00+0000 to 2021-03-30 13:56:00+0000	<Samp
	2021-03-30 13:38:00+0000 to 2021-03-30 13:55:00+0000	OffScan
	2021-03-31 09:47:00+0000 to 2021-03-31 10:01:00+0000	Zero
	2021-03-31 10:02:00+0000 to 2021-03-31 10:16:00+0000	Span
	2021-03-31 10:17:00+0000 to 2021-03-31 10:31:00+0000	Span1
	2021-03-31 10:32:00+0000 to 2021-03-31 10:36:00+0000	Purge
	2021-03-31 23:01:00+0000	OK_RS232
	2021-04-01 00:46:00+0000	OK_RS232
	2021-04-01 08:05:00+0000	OK_RS232
	2021-04-01 08:18:00+0000	OK_RS232
	2021-04-01 09:33:00+0000	OK_RS232
	2021-04-01 09:35:00+0000	OK_RS232
	2021-04-01 09:47:00+0000 to 2021-04-01 10:01:00+0000	Zero
	2021-04-01 10:02:00+0000 to 2021-04-01 10:16:00+0000	Span
	2021-04-01 10:17:00+0000 to 2021-04-01 10:31:00+0000	Span1
	2021-04-01 10:32:00+0000 to 2021-04-01 10:36:00+0000	Purge
	2021-04-01 16:09:00+0000	OK_RS232
	2021-04-01 17:35:00+0000	OK_RS232
	2021-04-01 18:01:00+0000	OK_RS232

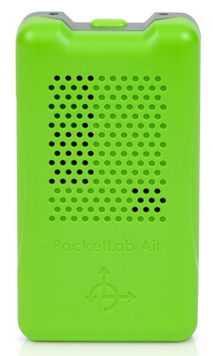

Image Attribution: Myriad Sensors, Inc. (San Jose, CA)

Data Flags Recorded During Testing (Cont.)

FRM/FEM Monitor	Timestamp (UTC)	Flag
Teledyne API T265<br>(Data acquired via local transfer from OAQPS)	2021-04-01 18:08:00+0000	OK_RS232
	2021-04-01 18:18:00+0000	OK_RS232
	2021-04-01 18:25:00+0000	OK_RS232
	2021-04-01 18:33:00+0000	OK_RS232
	2021-04-01 19:34:00+0000	OK_RS232
	2021-04-01 19:54:00+0000	OK_RS232
	2021-04-02 01:33:00+0000	OK_RS232
	2021-04-02 02:11:00+0000	OK_RS232
	2021-04-02 02:49:00+0000	OK_RS232
	2021-04-02 07:36:00+0000	OK_RS232
	2021-04-02 07:57:00+0000	OK_RS232
	2021-04-02 08:34:00+0000	OK_RS232
	2021-04-02 09:47:00+0000 to 2021-04-02 10:01:00+0000	Zero
	2021-04-02 10:02:00+0000 to 2021-04-02 10:16:00+0000	Span
	2021-04-02 10:17:00+0000 to 2021-04-02 10:31:00+0000	Span1
	2021-04-02 10:32:00+0000 to 2021-04-02 10:36:00+0000	Purge
	2021-04-02 13:33:00+0000	OK_RS232
	2021-04-02 14:08:00+0000	OK_RS232
	2021-04-02 14:14:00+0000	OK_RS232
	2021-04-02 14:28:00+0000	OK_RS232
	2021-04-02 14:58:00+0000	OK_RS232
	2021-04-02 15:59:00+0000	OK_RS232
	2021-04-02 16:18:00+0000	OK_RS232
	2021-04-02 16:31:00+0000	<Samp
	2021-04-02 17:29:00+0000	OK_RS232
	2021-04-02 18:31:00+0000	OK_RS232
	2021-04-02 18:52:00+0000	OK_RS232
	2021-04-02 18:59:00+0000	OK_RS232
	2021-04-02 19:24:00+0000	OK_RS232
	2021-04-02 19:26:00+0000	OK_RS232
	2021-04-02 19:28:00+0000 to 2021-04-02 19:29:00+0000	OK_RS232
	2021-04-02 19:31:00+0000	OK_RS232
	2021-04-02 19:36:00+0000	OK_RS232
	2021-04-02 20:15:00+0000	OK_RS232
	2021-04-02 20:19:00+0000	OK_RS232
	2021-04-02 20:28:00+0000	OK_RS232
	2021-04-02 20:33:00+0000	OK_RS232
	2021-04-02 20:36:00+0000	OK_RS232
	2021-04-02 20:39:00+0000	OK_RS232
	2021-04-02 20:45:00+0000	OK_RS232
	2021-04-02 20:52:00+0000	OK_RS232
	2021-04-02 21:13:00+0000	OK_RS232
	2021-04-02 21:17:00+0000	OK_RS232
	2021-04-03 09:47:00+0000 to 2021-04-03 10:01:00+0000	Zero
	2021-04-03 10:02:00+0000 to 2021-04-03 10:16:00+0000	Span
	2021-04-03 10:17:00+0000 to 2021-04-03 10:31:00+0000	Span1
	2021-04-03 10:32:00+0000 to 2021-04-03 10:36:00+0000	Purge

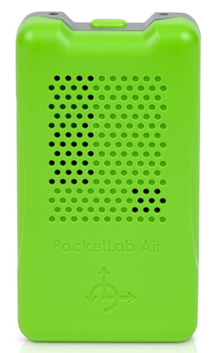

Image Attribution: Myriad Sensors, Inc. (San Jose, CA)

Data Flags Recorded During Testing (Cont.)

FRM/FEM Monitor	Timestamp (UTC)	Flag
Teledyne API T265 (Data acquired via local transfer from OAQPS)	2021-04-04 09:47:00+0000 to 2021-04-04 10:01:00+0000	Zero
	2021-04-04 10:02:00+0000 to 2021-04-04 10:16:00+0000	Span
	2021-04-04 10:17:00+0000 to 2021-04-04 10:31:00+0000	Span1
	2021-04-04 10:32:00+0000 to 2021-04-04 10:36:00+0000	Purge
	2021-04-05 09:47:00+0000 to 2021-04-05 10:01:00+0000	Zero
	2021-04-05 10:02:00+0000 to 2021-04-05 10:16:00+0000	Span
	2021-04-05 10:17:00+0000 to 2021-04-05 10:31:00+0000	Span1
	2021-04-05 10:32:00+0000 to 2021-04-05 10:36:00+0000	Purge
	2021-04-06 09:47:00+0000 to 2021-04-06 10:01:00+0000	Zero
	2021-04-06 10:02:00+0000 to 2021-04-06 10:16:00+0000	Span
	2021-04-06 10:17:00+0000 to 2021-04-06 10:31:00+0000	Span1
	2021-04-06 10:32:00+0000 to 2021-04-06 10:36:00+0000	Purge
	2021-04-07 09:47:00+0000 to 2021-04-07 10:01:00+0000	Zero
	2021-04-07 10:02:00+0000 to 2021-04-07 10:16:00+0000	Span
	2021-04-07 10:17:00+0000 to 2021-04-07 10:31:00+0000	Span1
	2021-04-07 10:32:00+0000 to 2021-04-07 10:36:00+0000	Purge
	2021-04-07 17:44:00+0000	OK_RS232
	2021-04-08 01:56:00+0000	OK_RS232
	2021-04-08 09:14:00+0000	OK_RS232
	2021-04-08 09:32:00+0000	OK_RS232
	2021-04-08 09:47:00+0000 to 2021-04-08 10:01:00+0000	Zero
	2021-04-08 10:02:00+0000 to 2021-04-08 10:16:00+0000	Span
	2021-04-08 10:17:00+0000 to 2021-04-08 10:31:00+0000	Span1
	2021-04-08 10:32:00+0000 to 2021-04-08 10:36:00+0000	Purge
	2021-04-08 11:15:00+0000	OK_RS232
	2021-04-08 13:25:00+0000	<Samp
	2021-04-08 16:13:00+0000	OK_RS232
	2021-04-08 16:26:00+0000	OK_RS232
	2021-04-08 17:33:00+0000	OK_RS232
	2021-04-08 17:50:00+0000	OK_RS232
	2021-04-08 17:52:00+0000	OK_RS232
	2021-04-08 18:06:00+0000	OK_RS232
	2021-04-08 18:10:00+0000	OK_RS232
	2021-04-09 00:32:00+0000	OK_RS232
	2021-04-09 00:38:00+0000	OK_RS232
	2021-04-09 00:43:00+0000	OK_RS232
	2021-04-09 01:02:00+0000	OK_RS232
	2021-04-09 01:08:00+0000	OK_RS232
	2021-04-09 01:19:00+0000	OK_RS232
	2021-04-09 01:38:00+0000	OK_RS232
	2021-04-09 01:41:00+0000	OK_RS232
	2021-04-09 01:43:00+0000	OK_RS232
	2021-04-09 02:18:00+0000	OK_RS232
	2021-04-09 08:00:00+0000	OK_RS232
	2021-04-09 08:43:00+0000	OK_RS232
	2021-04-09 08:56:00+0000	OK_RS232
	2021-04-09 09:07:00+0000	OK_RS232

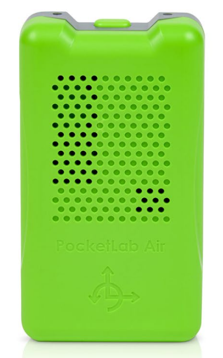

Image Attribution: Myriad Sensors, Inc. (San Jose, CA)

Data Flags Recorded During Testing (Cont.)

FRM/FEM Monitor	Timestamp (UTC)	Flag
Teledyne API T265 (Data acquired via local transfer from OAQPS)	2021-04-09 09:11:00+0000 to 2021-04-09 09:12:00+0000	OK_RS232
	2021-04-09 09:24:00+0000	OK_RS232
	2021-04-09 09:28:00+0000	OK_RS232
	2021-04-09 09:32:00+0000	OK_RS232
	2021-04-09 09:36:00+0000	OK_RS232
	2021-04-09 09:47:00+0000 to 2021-04-09 10:01:00+0000	Zero
	2021-04-09 10:02:00+0000 to 2021-04-09 10:16:00+0000	Span
	2021-04-09 10:17:00+0000 to 2021-04-09 10:31:00+0000	Span1
	2021-04-09 10:32:00+0000 to 2021-04-09 10:36:00+0000	Purge
	2021-04-09 10:38:00+0000	OK_RS232
	2021-04-09 11:08:00+0000	OK_RS232
	2021-04-09 16:49:00+0000	OK_RS232
	2021-04-09 16:58:00+0000	OK_RS232
	2021-04-09 17:34:00+0000	OK_RS232
	2021-04-09 17:41:00+0000	OK_RS232
	2021-04-09 17:49:00+0000	OK_RS232
	2021-04-09 17:51:00+0000 to 2021-04-09 17:52:00+0000	OK_RS232
	2021-04-09 17:57:00+0000	OK_RS232
	2021-04-09 18:03:00+0000	OK_RS232
	2021-04-09 18:11:00+0000	OK_RS232
	2021-04-09 18:30:00+0000	OK_RS232
	2021-04-09 18:44:00+0000	OK_RS232
	2021-04-09 18:48:00+0000	OK_RS232
	2021-04-09 18:54:00+0000	OK_RS232
	2021-04-09 19:12:00+0000	OK_RS232
	2021-04-09 19:33:00+0000	OK_RS232
	2021-04-09 19:40:00+0000	OK_RS232
	2021-04-09 19:44:00+0000	OK_RS232
	2021-04-09 20:45:00+0000	OK_RS232
	2021-04-10 00:44:00+0000	OK_RS232
	2021-04-10 01:19:00+0000	OK_RS232
	2021-04-10 01:27:00+0000	OK_RS232
	2021-04-10 01:33:00+0000	OK_RS232
	2021-04-10 01:39:00+0000	OK_RS232
	2021-04-10 01:44:00+0000	OK_RS232
	2021-04-10 09:47:00+0000 to 2021-04-10 10:01:00+0000	Zero
	2021-04-10 10:02:00+0000 to 2021-04-10 10:16:00+0000	Span
	2021-04-10 10:17:00+0000 to 2021-04-10 10:31:00+0000	Span1
	2021-04-10 10:32:00+0000 to 2021-04-10 10:36:00+0000	Purge
	2021-04-11 09:47:00+0000 to 2021-04-11 10:01:00+0000	Zero
	2021-04-11 10:02:00+0000 to 2021-04-11 10:16:00+0000	Span
	2021-04-11 10:17:00+0000 to 2021-04-11 10:31:00+0000	Span1
	2021-04-11 10:32:00+0000 to 2021-04-11 10:36:00+0000	Purge
	2021-04-12 09:47:00+0000 to 2021-04-12 10:01:00+0000	Zero
	2021-04-12 10:02:00+0000 to 2021-04-12 10:16:00+0000	Span
	2021-04-12 10:17:00+0000 to 2021-04-12 10:31:00+0000	Span1
	2021-04-12 10:32:00+0000 to 2021-04-12 10:36:00+0000	Purge

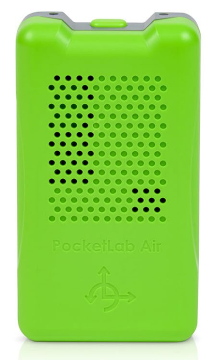

Image Attribution: Myriad Sensors, Inc. (San Jose, CA)

Data Flags Recorded During Testing (Cont.)

FRM/FEM Monitor	Timestamp (UTC)	Flag
Teledyne API T265 (Data acquired via local transfer from OAQPS)	2021-04-13 09:47:00+0000 to 2021-04-13 10:01:00+0000	Zero
	2021-04-13 10:02:00+0000 to 2021-04-13 10:16:00+0000	Span
	2021-04-13 10:17:00+0000 to 2021-04-13 10:31:00+0000	Span1
	2021-04-13 10:32:00+0000 to 2021-04-13 10:36:00+0000	Purge
	2021-04-13 12:58:00+0000	<Samp
	2021-04-13 13:22:00+0000	<Samp
	2021-04-13 13:25:00+0000	<Samp
	2021-04-13 13:31:00+0000	<Samp
	2021-04-13 14:20:00+0000	<Samp
	2021-04-13 14:22:00+0000	<Samp
	2021-04-13 15:08:00+0000	<Samp
	2021-04-13 15:10:00+0000	<Samp
	2021-04-13 16:37:00+0000	<Samp
	2021-04-13 16:39:00+0000	<Samp
	2021-04-13 16:54:00+0000	<Samp
	2021-04-14 09:47:00+0000 to 2021-04-14 10:01:00+0000	Zero
	2021-04-14 10:02:00+0000 to 2021-04-14 10:16:00+0000	Span
	2021-04-14 10:17:00+0000 to 2021-04-14 10:31:00+0000	Span1
	2021-04-14 10:32:00+0000 to 2021-04-14 10:36:00+0000	Purge
	2021-04-15 01:14:00+0000	OK_RS232
	2021-04-15 02:58:00+0000	OK_RS232
	2021-04-15 08:45:00+0000	OK_RS232
	2021-04-15 08:54:00+0000	OK_RS232
	2021-04-15 09:45:00+0000	OK_RS232
	2021-04-15 09:47:00+0000 to 2021-04-15 10:01:00+0000	Zero
	2021-04-15 10:02:00+0000 to 2021-04-15 10:16:00+0000	Span
	2021-04-15 10:17:00+0000 to 2021-04-15 10:31:00+0000	Span1
	2021-04-15 10:32:00+0000 to 2021-04-15 10:36:00+0000	Purge
	2021-04-15 17:05:00+0000	OK_RS232
	2021-04-15 17:12:00+0000	OK_RS232
	2021-04-15 17:25:00+0000	OK_RS232
	2021-04-15 18:32:00+0000	OK_RS232
	2021-04-15 18:37:00+0000	OK_RS232
	2021-04-15 18:45:00+0000	OK_RS232
	2021-04-15 18:51:00+0000	OK_RS232
	2021-04-15 19:20:00+0000	OK_RS232
	2021-04-16 01:55:00+0000	OK_RS232
	2021-04-16 02:39:00+0000	OK_RS232
	2021-04-16 03:10:00+0000	OK_RS232
	2021-04-16 03:19:00+0000	OK_RS232
	2021-04-16 04:59:00+0000	OK_RS232
	2021-04-16 07:25:00+0000	OK_RS232
	2021-04-16 07:31:00+0000	OK_RS232
	2021-04-16 07:35:00+0000	OK_RS232
	2021-04-16 09:47:00+0000 to 2021-04-16 10:01:00+0000	Zero
	2021-04-16 10:02:00+0000 to 2021-04-16 10:16:00+0000	Span
	2021-04-16 10:17:00+0000 to 2021-04-16 10:31:00+0000	Span1

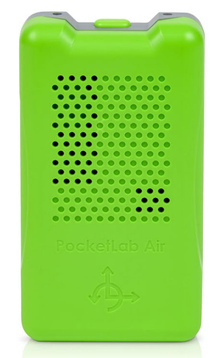

Image Attribution: Myriad Sensors, Inc. (San Jose, CA)

Data Flags Recorded During Testing (Cont.)

FRM/FEM Monitor	Timestamp (UTC)	Flag
Teledyne API T265<br>(Data acquired via local transfer from OAQPS)	2021-04-16 10:32:00+0000 to 2021-04-16 10:36:00+0000	Purge
	2021-04-16 13:59:00+0000	OK_RS232
	2021-04-16 14:15:00+0000	OK_RS232
	2021-04-16 14:25:00+0000	OK_RS232
	2021-04-16 14:28:00+0000	OK_RS232
	2021-04-16 14:33:00+0000	OK_RS232
	2021-04-16 14:37:00+0000	OK_RS232
	2021-04-16 14:53:00+0000	<Samp
	2021-04-16 14:55:00+0000	<Samp
	2021-04-16 15:45:00+0000	OK_RS232
	2021-04-16 15:49:00+0000	<Samp
	2021-04-16 16:16:00+0000	<Samp
	2021-04-16 19:19:00+0000	OK_RS232
	2021-04-16 19:59:00+0000	OK_RS232
	2021-04-16 20:05:00+0000	OK_RS232
	2021-04-16 20:07:00+0000	OK_RS232
	2021-04-16 20:13:00+0000	OK_RS232
	2021-04-16 20:19:00+0000	OK_RS232
	2021-04-16 20:22:00+0000	OK_RS232
	2021-04-16 20:47:00+0000	OK_RS232
	2021-04-16 21:08:00+0000	OK_RS232
	2021-04-16 21:11:00+0000	OK_RS232
	2021-04-16 21:15:00+0000	OK_RS232
	2021-04-16 21:18:00+0000	OK_RS232
	2021-04-16 21:20:00+0000	OK_RS232
	2021-04-16 21:28:00+0000	OK_RS232
	2021-04-16 21:34:00+0000 to 2021-04-16 21:35:00+0000	OK_RS232
	2021-04-16 21:37:00+0000	OK_RS232
	2021-04-16 21:41:00+0000	OK_RS232
	2021-04-16 21:53:00+0000	OK_RS232
	2021-04-16 22:32:00+0000	OK_RS232
	2021-04-16 22:48:00+0000	OK_RS232
	2021-04-16 23:03:00+0000	OK_RS232
	2021-04-17 09:47:00+0000 to 2021-04-17 10:01:00+0000	Zero
	2021-04-17 10:02:00+0000 to 2021-04-17 10:16:00+0000	Span
	2021-04-17 10:17:00+0000 to 2021-04-17 10:31:00+0000	Span1
	2021-04-17 10:32:00+0000 to 2021-04-17 10:36:00+0000	Purge
	2021-04-18 09:47:00+0000 to 2021-04-18 10:01:00+0000	Zero
	2021-04-18 10:02:00+0000 to 2021-04-18 10:16:00+0000	Span
	2021-04-18 10:17:00+0000 to 2021-04-18 10:31:00+0000	Span1
	2021-04-18 10:32:00+0000 to 2021-04-18 10:36:00+0000	Purge
	2021-04-19 09:47:00+0000 to 2021-04-19 10:01:00+0000	Zero
	2021-04-19 10:02:00+0000 to 2021-04-19 10:16:00+0000	Span
	2021-04-19 10:17:00+0000 to 2021-04-19 10:31:00+0000	Span1
	2021-04-19 10:32:00+0000 to 2021-04-19 10:36:00+0000	Purge

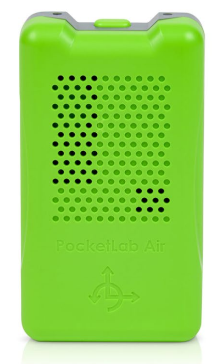

Data Flags Recorded During Testing (Cont.)

Meteorological Instrument	Timestamp (UTC)	Flag
RM Young 41382 VC Temperature and Relative Humidity Monitor (Data acquired via local transfer from OAQPS)	2021-03-17 18:54:00+0000	<Samp
	2021-03-19 12:48:00+0000	<Samp
	2021-03-26 17:50:00+0000	<Samp
	2021-04-02 16:31:00+0000	<Samp
	2021-04-08 13:25:00+0000	<Samp
	2021-04-13 12:58:00+0000	<Samp
	2021-04-13 13:22:00+0000	<Samp
	2021-04-13 13:25:00+0000	<Samp
	2021-04-13 13:31:00+0000	<Samp
	2021-04-13 14:20:00+0000	<Samp
	2021-04-13 14:22:00+0000	<Samp
	2021-04-13 15:08:00+0000	<Samp
	2021-04-13 15:10:00+0000	<Samp
	2021-04-13 16:37:00+0000	<Samp
	2021-04-13 16:39:00+0000	<Samp
	2021-04-13 16:54:00+0000	<Samp
	2021-04-16 14:53:00+0000	<Samp
	2021-04-16 14:55:00+0000	<Samp
	2021-04-16 15:49:00+0000	<Samp
	2021-04-16 16:16:00+0000	<Samp

Testing Report - O_3 Base Testing

Vaisala AQT420

This report reflects out-of-the-box performance

Initial Base Testing - RTP, NC

U.S. Environmental Protection Agency
Office of Research and Development
PI: Clements.Andrea@epa.gov
919-541-1363

June 2018—July 2018

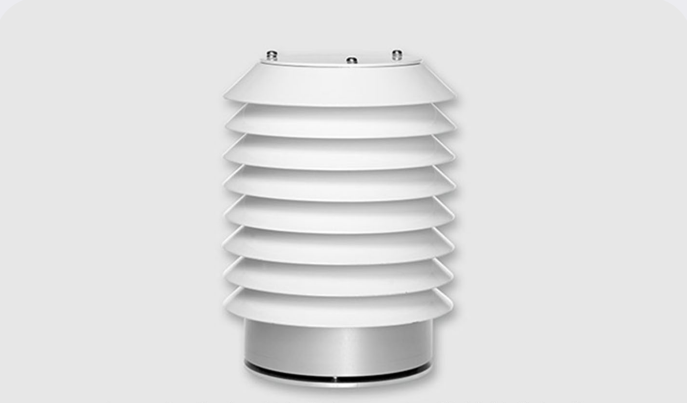

Image Attribution: Vaisala Corporation (Helsinki, Finland)

Deployment Details

Testing Organization and Site Information	
Testing organization (Name, Organization type, Contact website)	U.S. Environmental Protection Agency - Office of Research and Development<br>Federal Government<br>Air Sensor Toolbox | U.S. EPA Website
Testing location (City, State, Latitude and Longitude)	Ambient Monitoring Innovative Research Station (AIRS)<br>RTP, NC<br>35.88951, -78.874572
AQS site ID	37 – 063 – 0099
Sampling timeframe (MM-DD-YY)	06-23-18 to 07-23-18
Sensor data source	Connected to local datalogger
Reference data source	OAQPS file transfer

Sensor Information			
Manufacturer, model	Vaisala AQT420		
Device firmware version	1.16-PRE3		
Sampling time interval	1-minute, 1-second		
Sensor serial numbers	Vaisala_1	Vaisala_2	Vaisala_3
Issues encountered during deployment?	☐	No Issues	

FRM/FEM Information	
Manufacturer, model, designation	Teledyne API T265 FEM
Sampling time interval	1-hour averaging
Date of calibration	As required by 40 CFR Part 58 and the Burdens Creek QAPP maintained by OAQPS
Date of one-point QC check	Every two weeks as required by 40 CFR Part 58 Appendix A 3.1.1
Description, date(s) of maintenance activities	N/A

Time Series Plot: 1-hour averaged O_3

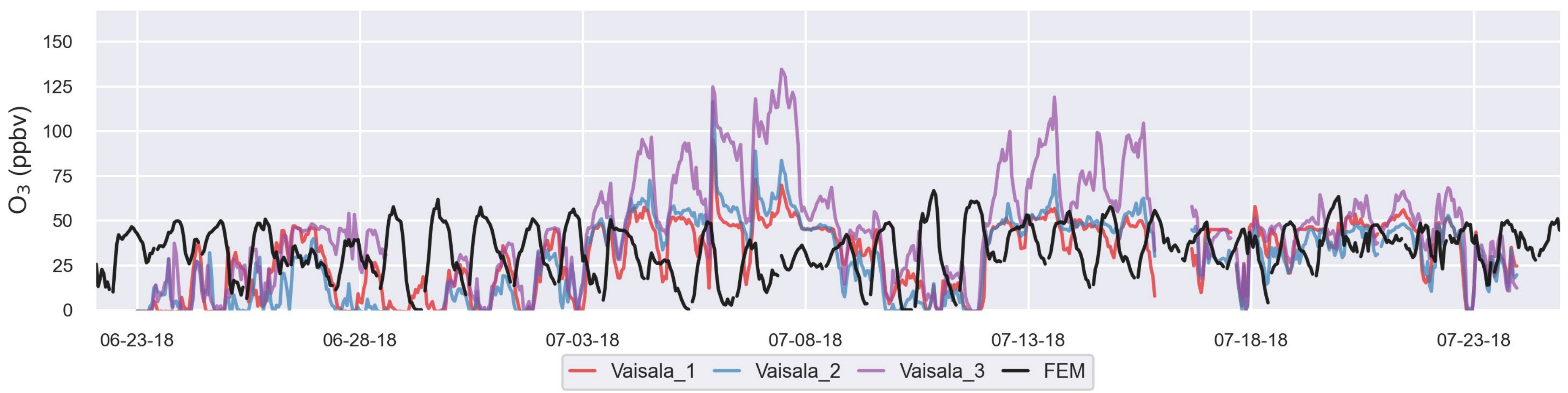

Range and average of FRM/FEM concentrations over duration of base test (ppbv)	[1-hr] -0.0-66.7, avg: 33.4,<br>[Rolling 8-hr] 2.5-59.1, avg: 37.0
Number of 1-hr periods in FRM/FEM monitor measurements with a goal concentration ≥ 60 ppbv	11

Scatter Plot: Comparison to FRM/FEM

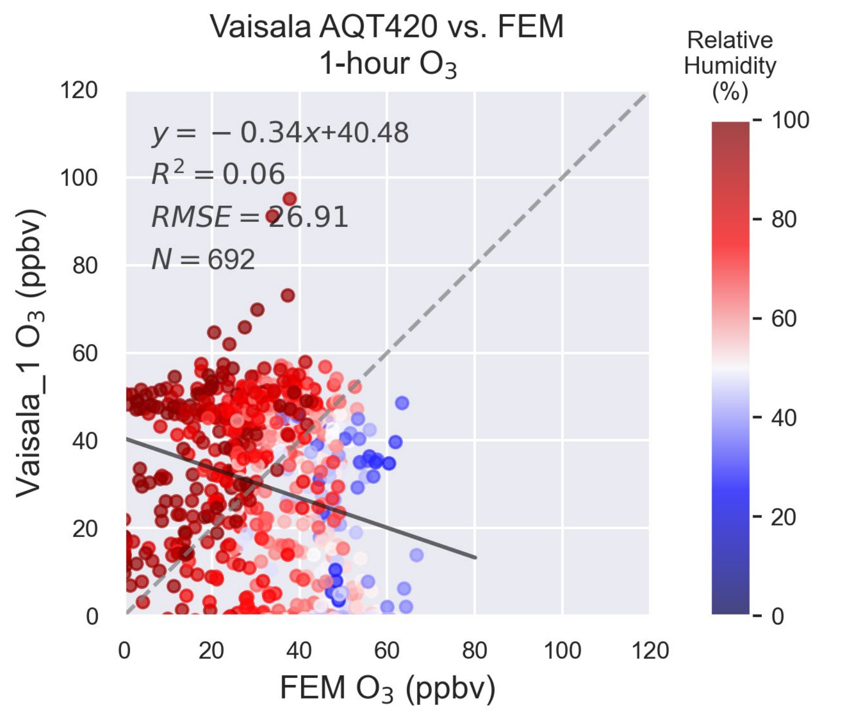

Performance Metrics*

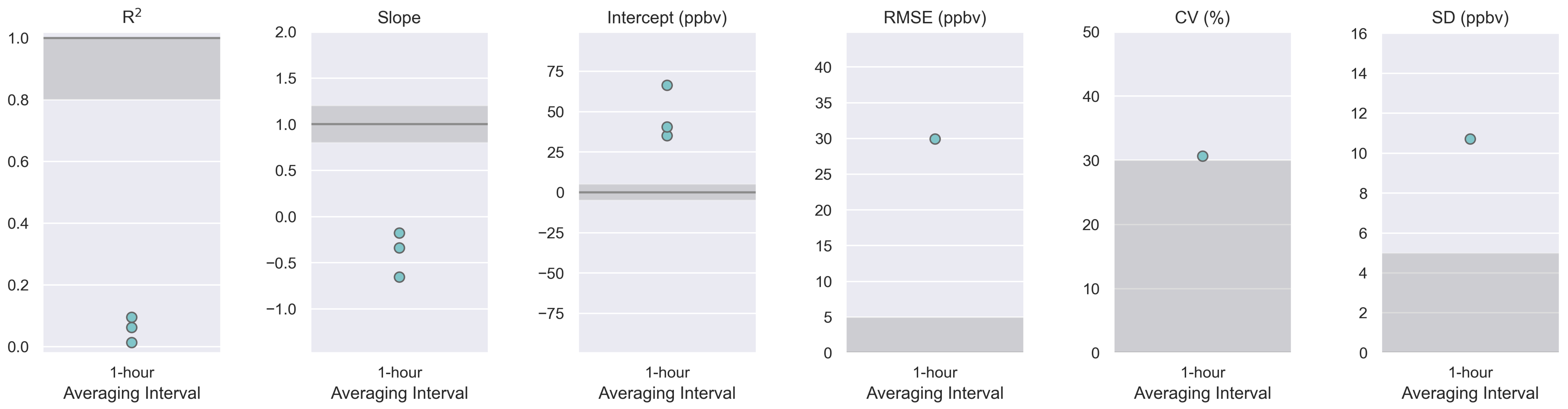

Meteorological Conditions During Deployment

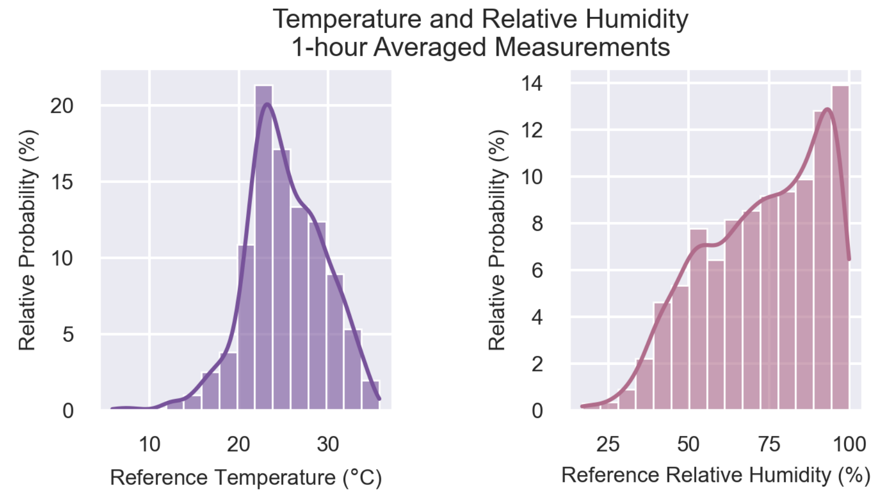

Number of 1-hr periods outside sensor manufacturer-listed temperature operational range (no operational range specified)	-
Number of 1-hr periods outside sensor manufacturer-listed relative humidity operational range (no operational range specified)	-

Meteorological Influence

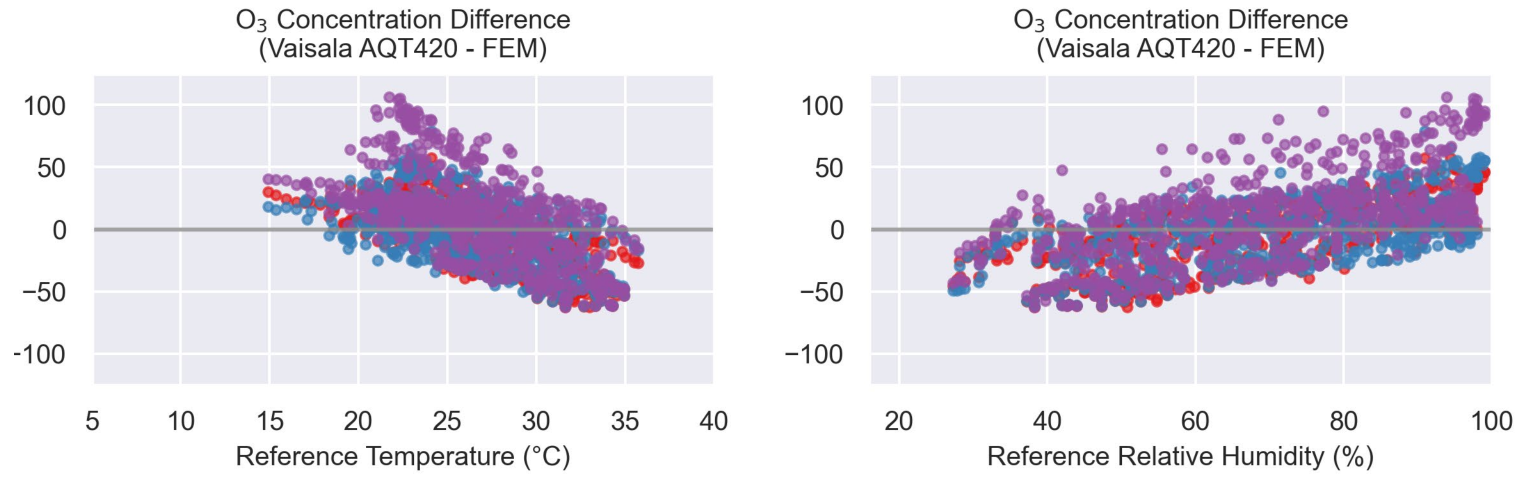

Mean number of paired, normalized concentration and temperature values (1-hr averages)	744
Mean number of paired, normalized concentration and relative humidity values (1-hr averages)	744

*For evaluations with greater than three sensors, grouping individual sensor metrics into boxplots is recommended for displaying results. Note that this recommendation does not apply to metrics computed as a single value for all sensors over the whole evaluation group, such as RMSE, NRMSE, CV, and standard deviation.

Testing Report - O_3 Base Testing

Vaisala AQT420

This report reflects out-of-the-box performance

Initial Base Testing - RTP, NC
U.S. Environmental Protection Agency
Office of Research and Development
PI: Clements.Andrea@epa.gov
919-541-1363
June 2018—July 2018

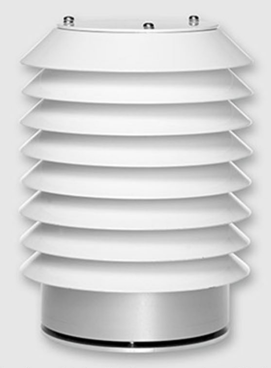
Image Attribution: Vaisala Corporation (Helsinki, Finland)

Tabular Statistics

Sensor-FRM/FEM Correlation

	Bias and Linearity			Data Quality	
	R^2	Slope	Intercept (ppbv)	Uptime (%)	Number of paired sensor and reference concentration values*
	1-Hour ○○○	1-Hour ○○○	1-Hour ○○○	1-Hour ●●●	1-Hour
Metric Target Range	≥ 0.80	1.0 ± 0.20	-5 ≤ b ≤ 5	75%*	-
Sensor Vaisala_1	0.06	-0.34	40.48	97	692
Sensor Vaisala_2	0.01	-0.18	35.06	93	663
Sensor Vaisala_3	0.09	-0.66	66.31	93	663
Mean	0.06	-0.39	47.28	94.36	672.67

	Error
	RMSE (ppbv)
	1-Hour ☆
Metric Target Range	≤ 5.0
Deployment Value	29.9

Device-specific metrics (computed for each sensor in evaluation)
○○○ Metric value for none of devices tested falls within the target range
●○○ Metric value for one of devices tested falls within the target range
●●○ Metric value for two of devices tested falls within the target range
●●● Metric value for three of devices tested falls within the target range

Single-valued metrics (computed via entire evaluation dataset)
☆ Indicates that the metric value is not within the target range
★ Indicates that the metric value is within the target range

Sensor-Sensor Precision

	Precision (between collocated sensors)		Data Quality
	CV (%)	SD (ppbv)	Number of paired sensor and reference concentration values*
	1-Hour ☆	1-Hour ☆	1-Hour
Metric Target Range	≤ 30.0	≤ 5.0	-
Deployment Value	30.7	10.7	692

*This value is only a recommendation for ensuring data quality and is not included in the list of target values discussed in Section 4 of the Performance Testing Protocols, Metrics, and Target Values for Ozone Air Sensors document.

Testing Report - O_3 Base Testing

Vaisala AQT420

This report reflects out-of-the-box performance

Initial Base Testing - RTP, NC
U.S. Environmental Protection Agency
Office of Research and Development
PI: Clements.Andrea@epa.gov
919-541-1363
June 2018—July 2018

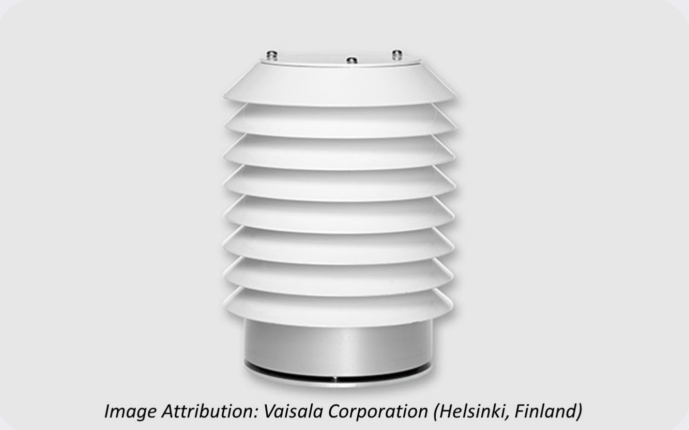

Sensor-FRM/FEM Scatter Plots

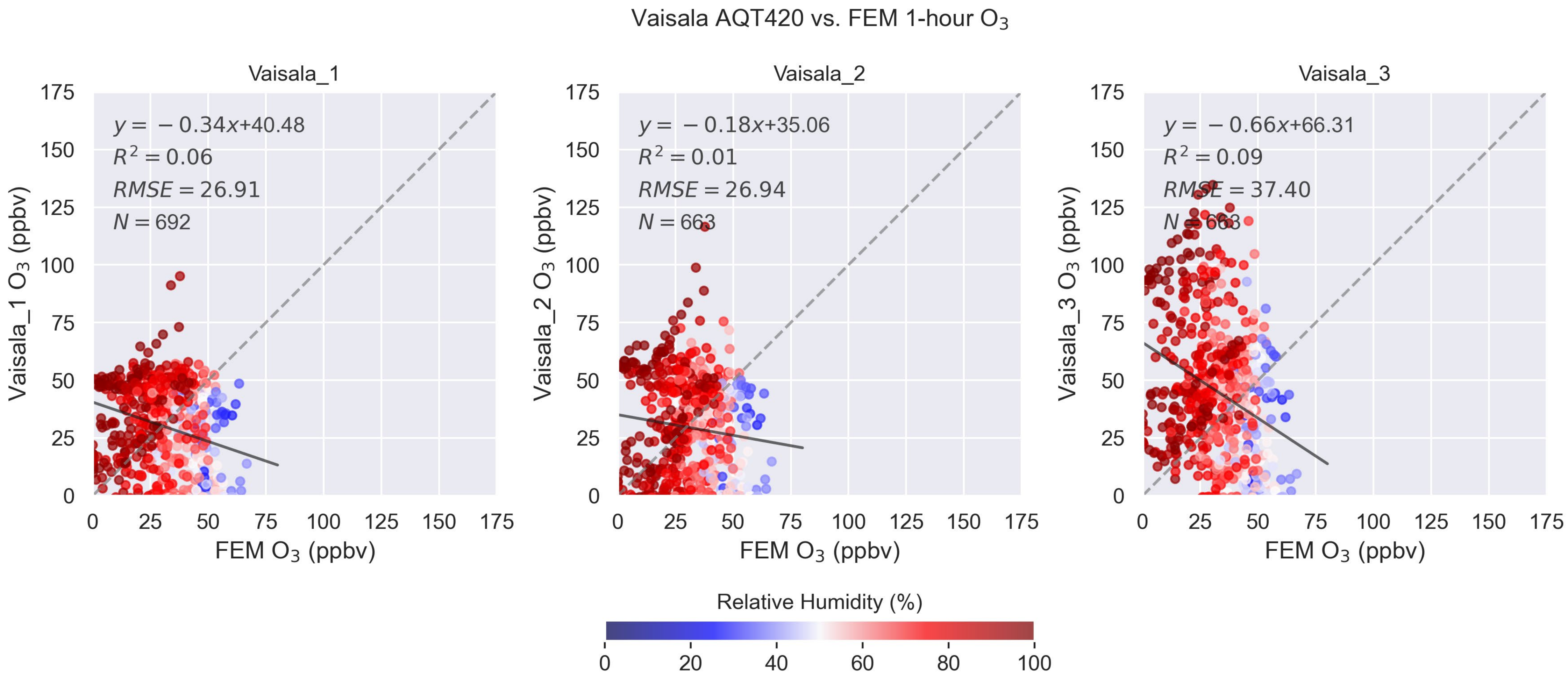

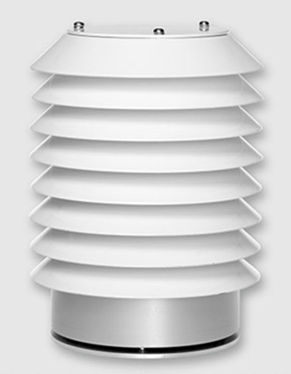

Image Attribution: Vaisala Corporation (Helsinki, Finland)

Supplemental Information

Abbreviations used in Supplemental Information

FRM	Federal Reference Method
FEM	Federal Equivalent Method
SOP	Standard Operating Procedure
QAPP	Quality Assurance Project Plan
QC	Quality Control

Supplemental Documentation	Attached	Description & URL or file path to documentation
Field observations and sensor data flags	☑	No observations or data flags recorded
Maintenance logs	☑	No logs recorded during testing
Standard operating procedure(s)	☐	U.S. EPA Office Of Research and Development SOP available upon request
Photos of equipment setup and testing	☑	See NC-VSA-Page 5 of this testing report
Product specifications sheet(s)	☐	See Appendix C, “Spec_Sheet_Vaisala_AQT420.pdf”
Product manual(s)	☐	See Appendix C, “Manual_Vaisala_AQT420.pdf”
Data storage and transmission method	☑	See NC-VSA-Page 6 of this testing report
Data correction approach	☑	See NC-VSA-Page 6 of this testing report
Issues encountered	☑	No issues logged during testing
Data analysis/correction scripts and version	☑	Averaging and processing of data, calculation of performance metrics, and generation of figures and other supplementary material for analysis were obtained using Python 3.9.7 with the packages sensortoolkit v0.8.3b2, pandas 1.3.5, NumPy 1.21.2, Matplotlib 3.5.0, statsmodels 0.13.0, and seaborn 0.11.2. All packages are available from the Python Package Index (PyPI) at https://pypi.org. The integrated development environment (IDE) Spyder 5.1.5 was used for scripting and data visualization. Version control for the Python base, packages, and IDE were all managed by conda 4.11.0.
Air Monitoring Station QAPP	☐	U.S. EPA Office Of Research and Development QAPP available upon request
Summary of FRM/FEM monitor QC checks	☑	See NC-VSA-Pages 7-10 of this testing report
Manufacturer website for FRM/FEM monitor	☑	Teledyne API: T265 Product website
FRM/FEM monitor manual	☑	See Appendix B, “Spec_Sheet_TeledyneAPI_T265.pdf”
FRM/FEM monitor specifications sheet(s)	☑	See Appendix B, “Manual_TeledyneAPI_T265.pdf”
Other documents	☐	

Initial Base Testing - RTP, NC
U.S. Environmental Protection Agency
Office of Research and Development
PI: Clements.Andrea@epa.gov
919-541-1363
June 2018—July 2018

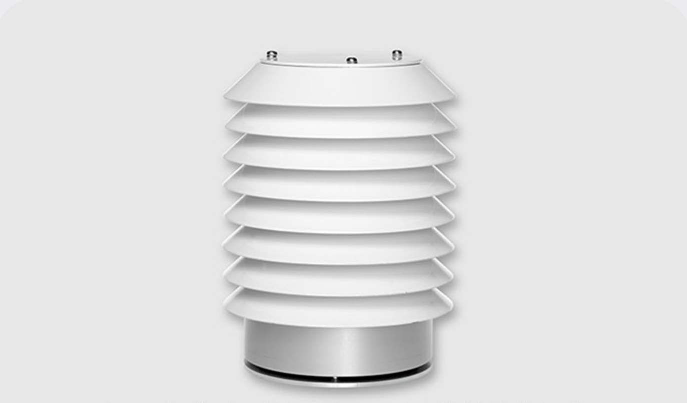
Image Attribution: Vaisala Corporation (Helsinki, Finland)

Supplemental Information: Photos of Testing Site and Equipment Setup

Site Description:
The Burdens Creek Ambient Monitoring Innovation Research Station (AIRS) site is located on the U.S. EPA, RTP campus and is situated between Alexander Drive and Route 147. The site is intended to represent a neighborhood-scale site as defined in *40 CFR Part 58, Appendix D*. U.S. EPA's Office of Air Quality Planning and Standards (OAQPS) operates reference grade instruments in a free-standing shelter situated directly adjacent to the sensor testing platform.

Figure 1: Vaisala AQT420 unit mounted on observation deck railing during testing period.

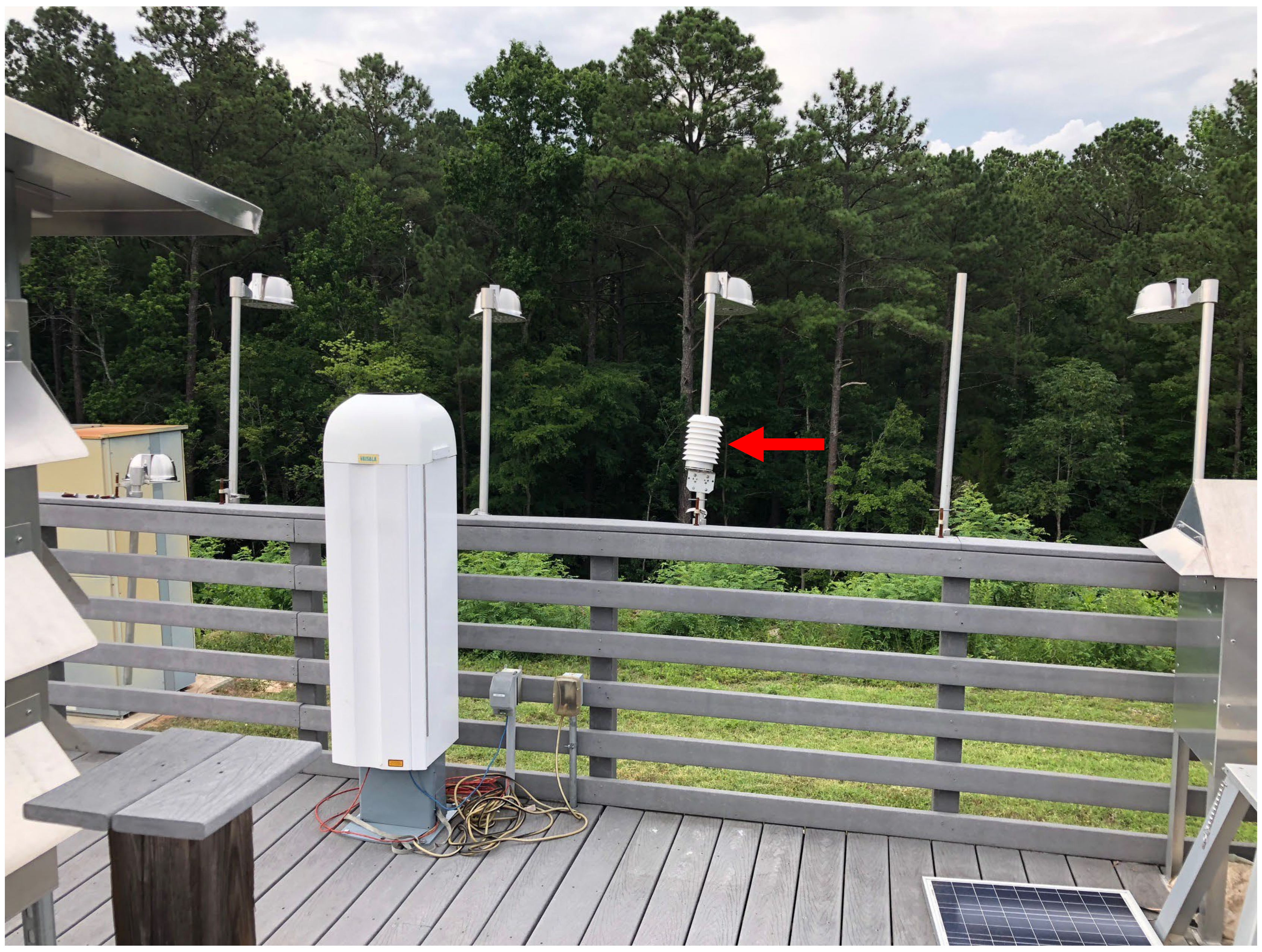

Figure 2: Observation deck at evaluation site. Approximate location of sensors indicated by the red arrow. The approximate location of FEM (housed within sampling trailer) indicated by the black arrow. Sensors and FEM instrumentation are separated by approximately 15 meters.

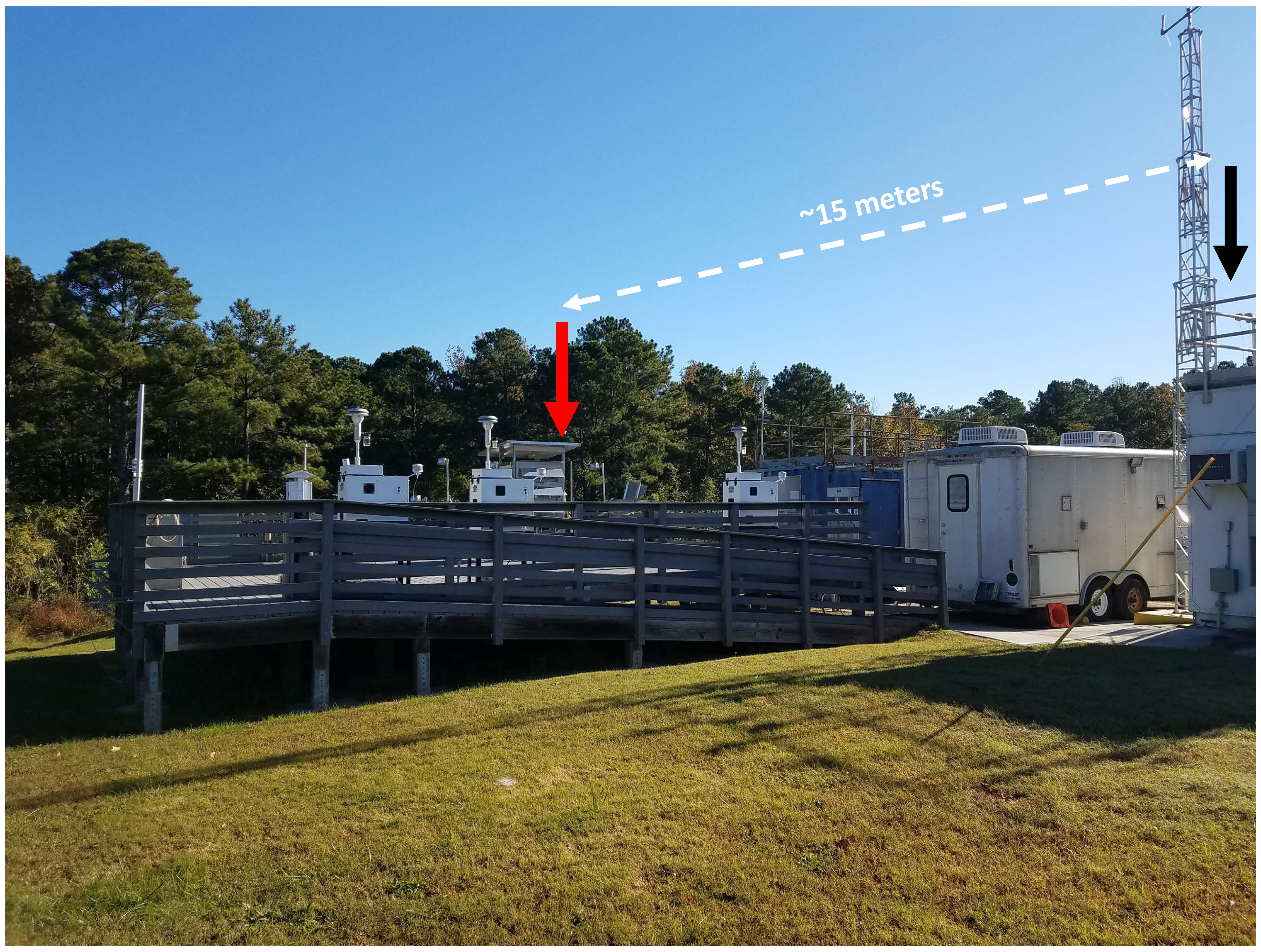

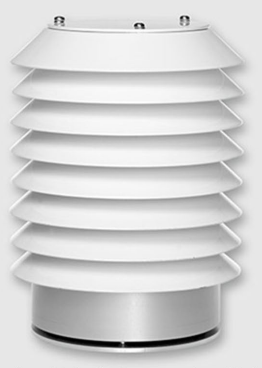

Image Attribution: Vaisala Corporation (Helsinki, Finland)

Supplemental Information: Data Storage, Correction Approach, and Issues Encountered

Data Storage and Transmission Method

Data logged by sensors was transferred via serial cable (M12 to RS485 to USB) to a laptop running PuTTY in 'Logging' mode. PuTTY recorded data in comma-separated value files to a site computer. Field technicians visited the evaluation site on at least a weekly basis to transfer data files to a USB drive. Files were subsequently copied to a local computer for backup and data analysis. Data files were labeled noting the time data collection was stopped/started and the local computer time was checked against a NIST traceable timepiece at each visit.

Data Correction Approach

Communication with the sensor manufacturer indicated that all data processing (conversion of sensor voltages to concentration units) using a proprietary algorithm was done on the sensor itself so that data downloaded locally using this method was the same as data that would be transmitted should the sensor have been setup to steam data to a remote server.

This evaluation report reflects "out-of-the-box" performance of the AQT420. After acquisition, the data was processed using the *sensortoolkit* python code library (v0.8.3b2). A continuous data set at the recorded sampling frequency was written to a .csv file. 1-hour averaged data sets were generated using a 75% completeness threshold and saved as separate .csv files. Outliers were **not** removed from data sets in order to assess "out-of-the-box" sensor performance.

The Vaisala AQT420 internal O_3 sensor did not exhibit a distinct warm-up period. Upon inspection of concentration timeseries for each sensor unit, values logged near initial power up are consistent with the concentration range and variability found in each unit's subsequent measurements

Issues Encountered

<u>Pre-deployment observations</u>

No observations were reported during the pre-deployment phase.

<u>Field observations and sensor data flags</u>

No observations were reported during testing.

Operator error restarting PuTTY resulted in two periods of sensor data loss. More frequent site visits were initiated to try and minimize the potential for data loss.

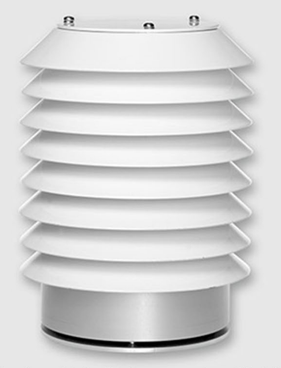

Image Attribution: Vaisala Corporation (Helsinki, Finland)

Description of Data Flags

OAQPS manages data logged by reference monitors at the AIRS evaluation site using the Envidas data acquisition system software from DR DAS LTD[1]. Envidas contains over 100 data flags which are configurable by the monitoring agency and can be triggered based on instrument status. Appendix B Table 1 contains a description of Envidas data flags including the numeric code and status code name (a brief textual description, the table containing data flags below includes code names for events encountered by the reference monitor under the "Flag" column). Appendix B Table 1 also indicates whether reference data are invalidated for a given data flag. For this report, reference data were invalidated for periods where a data flag was logged and if the corresponding entry for the data flag in the "Data Status Invalidation" column in Appendix B Table 1 indicates data should be invalidated.

Data Flags Recorded During Testing

FRM/FEM Monitor	Timestamp (UTC)	Flag
Teledyne API T265 (Data acquired via local transfer from OAQPS)	2018-06-23 09:46:00+0000 to 2018-06-23 10:00:00+0000	Zero
	2018-06-23 10:01:00+0000 to 2018-06-23 10:15:00+0000	Span
	2018-06-23 10:16:00+0000 to 2018-06-23 10:30:00+0000	Spare
	2018-06-23 10:31:00+0000 to 2018-06-23 10:35:00+0000	Purge
	2018-06-24 09:46:00+0000 to 2018-06-24 10:00:00+0000	Zero
	2018-06-24 10:01:00+0000 to 2018-06-24 10:15:00+0000	Span
	2018-06-24 10:16:00+0000 to 2018-06-24 10:30:00+0000	Spare
	2018-06-24 10:31:00+0000 to 2018-06-24 10:35:00+0000	Purge
	2018-06-25 09:46:00+0000 to 2018-06-25 10:00:00+0000	Zero
	2018-06-25 10:01:00+0000 to 2018-06-25 10:15:00+0000	Span
	2018-06-25 10:16:00+0000 to 2018-06-25 10:30:00+0000	Spare
	2018-06-25 10:31:00+0000 to 2018-06-25 10:35:00+0000	Purge
	2018-06-26 09:46:00+0000 to 2018-06-26 10:00:00+0000	Zero
	2018-06-26 10:01:00+0000 to 2018-06-26 10:15:00+0000	Span
	2018-06-26 10:16:00+0000 to 2018-06-26 10:30:00+0000	Spare
	2018-06-26 10:31:00+0000 to 2018-06-26 10:35:00+0000	Purge
	2018-06-27 09:46:00+0000 to 2018-06-27 10:00:00+0000	Zero
	2018-06-27 10:01:00+0000 to 2018-06-27 10:15:00+0000	Span
	2018-06-27 10:16:00+0000 to 2018-06-27 10:30:00+0000	Spare
	2018-06-27 10:31:00+0000 to 2018-06-27 10:35:00+0000	Purge
	2018-06-28 09:46:00+0000 to 2018-06-28 10:00:00+0000	Zero
	2018-06-28 10:01:00+0000 to 2018-06-28 10:15:00+0000	Span
	2018-06-28 10:16:00+0000 to 2018-06-28 10:30:00+0000	Spare
	2018-06-28 10:31:00+0000 to 2018-06-28 10:35:00+0000	Purge
	2018-06-28 16:24:00+0000	NoData
	2018-06-28 16:25:00+0000	<Samp
	2018-06-29 09:46:00+0000 to 2018-06-29 10:00:00+0000	Zero
	2018-06-29 10:01:00+0000 to 2018-06-29 10:15:00+0000	Span
	2018-06-29 10:16:00+0000 to 2018-06-29 10:30:00+0000	Spare
	2018-06-29 10:31:00+0000 to 2018-06-29 10:35:00+0000	Purge

[1] "Contact us" DR DAS LTD, 2022, https://dr-das.com/Home/Contact.html Accessed June 3 2022

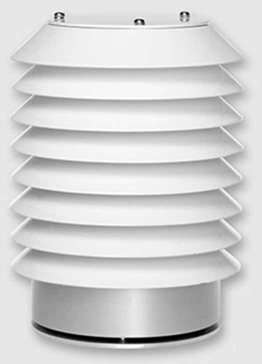

Image Attribution: Vaisala Corporation (Helsinki, Finland)

Supplemental Information: Description of FRM/FEM QC Checks and Data Flags

Data Flags Recorded During Testing (Cont.)

FRM/FEM Monitor	Timestamp (UTC)	Flag
Teledyne API T265 (Data acquired via local transfer from OAQPS)	2018-06-30 09:46:00+0000 to 2018-06-30 10:00:00+0000	Zero
	2018-06-30 10:01:00+0000 to 2018-06-30 10:15:00+0000	Span
	2018-06-30 10:16:00+0000 to 2018-06-30 10:30:00+0000	Spare
	2018-06-30 10:31:00+0000 to 2018-06-30 10:35:00+0000	Purge
	2018-07-01 09:46:00+0000 to 2018-07-01 10:00:00+0000	Zero
	2018-07-01 10:01:00+0000 to 2018-07-01 10:15:00+0000	Span
	2018-07-01 10:16:00+0000 to 2018-07-01 10:30:00+0000	Spare
	2018-07-01 10:31:00+0000 to 2018-07-01 10:35:00+0000	Purge
	2018-07-02 09:46:00+0000 to 2018-07-02 10:00:00+0000	Zero
	2018-07-02 10:01:00+0000 to 2018-07-02 10:15:00+0000	Span
	2018-07-02 10:16:00+0000 to 2018-07-02 10:30:00+0000	Spare
	2018-07-02 10:31:00+0000 to 2018-07-02 10:35:00+0000	Purge
	2018-07-03 09:46:00+0000 to 2018-07-03 10:00:00+0000	Zero
	2018-07-03 10:01:00+0000 to 2018-07-03 10:15:00+0000	Span
	2018-07-03 10:16:00+0000 to 2018-07-03 10:30:00+0000	Spare
	2018-07-03 10:31:00+0000 to 2018-07-03 10:35:00+0000	Purge
	2018-07-04 09:46:00+0000 to 2018-07-04 10:00:00+0000	Zero
	2018-07-04 10:01:00+0000 to 2018-07-04 10:15:00+0000	Span
	2018-07-04 10:16:00+0000 to 2018-07-04 10:30:00+0000	Spare
	2018-07-04 10:31:00+0000 to 2018-07-04 10:35:00+0000	Purge
	2018-07-05 09:46:00+0000 to 2018-07-05 10:00:00+0000	Zero
	2018-07-05 10:01:00+0000 to 2018-07-05 10:15:00+0000	Span
	2018-07-05 10:16:00+0000 to 2018-07-05 10:30:00+0000	Spare
	2018-07-05 10:31:00+0000 to 2018-07-05 10:35:00+0000	Purge
	2018-07-06 09:46:00+0000 to 2018-07-06 10:00:00+0000	Zero
	2018-07-06 10:01:00+0000 to 2018-07-06 10:15:00+0000	Span
	2018-07-06 10:16:00+0000 to 2018-07-06 10:30:00+0000	Spare
	2018-07-06 10:31:00+0000 to 2018-07-06 10:35:00+0000	Purge
	2018-07-07 09:46:00+0000 to 2018-07-07 10:00:00+0000	Zero
	2018-07-07 10:01:00+0000 to 2018-07-07 10:15:00+0000	Span
	2018-07-07 10:16:00+0000 to 2018-07-07 10:30:00+0000	Spare
	2018-07-07 10:31:00+0000 to 2018-07-07 10:35:00+0000	Purge
	2018-07-08 09:46:00+0000 to 2018-07-08 10:00:00+0000	Zero
	2018-07-08 10:01:00+0000 to 2018-07-08 10:15:00+0000	Span
	2018-07-08 10:16:00+0000 to 2018-07-08 10:30:00+0000	Spare
	2018-07-08 10:31:00+0000 to 2018-07-08 10:35:00+0000	Purge
	2018-07-09 09:46:00+0000 to 2018-07-09 10:00:00+0000	Zero
	2018-07-09 10:01:00+0000 to 2018-07-09 10:15:00+0000	Span
	2018-07-09 10:16:00+0000 to 2018-07-09 10:30:00+0000	Spare
	2018-07-09 10:31:00+0000 to 2018-07-09 10:35:00+0000	Purge
	2018-07-10 09:46:00+0000 to 2018-07-10 10:00:00+0000	Zero
	2018-07-10 10:01:00+0000 to 2018-07-10 10:15:00+0000	Span
	2018-07-10 10:16:00+0000 to 2018-07-10 10:30:00+0000	Spare
	2018-07-10 10:31:00+0000 to 2018-07-10 10:35:00+0000	Purge
	2018-07-10 15:43:00+0000	NoData
	2018-07-11 09:46:00+0000 to 2018-07-11 10:00:00+0000	Zero
	2018-07-11 10:01:00+0000 to 2018-07-11 10:15:00+0000	Span

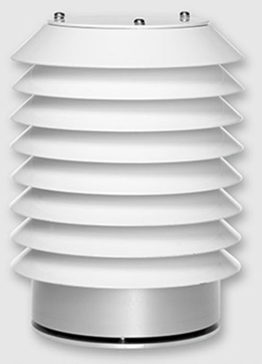

Image Attribution: Vaisala Corporation (Helsinki, Finland)

Data Flags Recorded During Testing (Cont.)

FRM/FEM Monitor	Timestamp (UTC)	Flag
Teledyne API T265 (Data acquired via local transfer from OAQPS)	2018-07-11 10:16:00+0000 to 2018-07-11 10:30:00+0000	Spare
	2018-07-11 10:31:00+0000 to 2018-07-11 10:35:00+0000	Purge
	2018-07-12 09:46:00+0000 to 2018-07-12 10:00:00+0000	Zero
	2018-07-12 10:01:00+0000 to 2018-07-12 10:15:00+0000	Span
	2018-07-12 10:16:00+0000 to 2018-07-12 10:30:00+0000	Spare
	2018-07-12 10:31:00+0000 to 2018-07-12 10:35:00+0000	Purge
	2018-07-12 21:24:00+0000	NoData
	2018-07-12 21:27:00+0000	NoData
	2018-07-13 09:46:00+0000 to 2018-07-13 10:00:00+0000	Zero
	2018-07-13 10:01:00+0000 to 2018-07-13 10:15:00+0000	Span
	2018-07-13 10:16:00+0000 to 2018-07-13 10:30:00+0000	Spare
	2018-07-13 10:31:00+0000 to 2018-07-13 10:35:00+0000	Purge
	2018-07-13 16:26:00+0000	<Samp
	2018-07-14 09:46:00+0000 to 2018-07-14 10:00:00+0000	Zero
	2018-07-14 10:01:00+0000 to 2018-07-14 10:15:00+0000	Span
	2018-07-14 10:16:00+0000 to 2018-07-14 10:30:00+0000	Spare
	2018-07-14 10:31:00+0000 to 2018-07-14 10:35:00+0000	Purge
	2018-07-15 09:46:00+0000 to 2018-07-15 10:00:00+0000	Zero
	2018-07-15 10:01:00+0000 to 2018-07-15 10:15:00+0000	Span
	2018-07-15 10:16:00+0000 to 2018-07-15 10:30:00+0000	Spare
	2018-07-15 10:31:00+0000 to 2018-07-15 10:35:00+0000	Purge
	2018-07-16 09:46:00+0000 to 2018-07-16 10:00:00+0000	Zero
	2018-07-16 10:01:00+0000 to 2018-07-16 10:15:00+0000	Span
	2018-07-16 10:16:00+0000 to 2018-07-16 10:30:00+0000	Spare
	2018-07-16 10:31:00+0000 to 2018-07-16 10:35:00+0000	Purge
	2018-07-17 09:46:00+0000 to 2018-07-17 10:00:00+0000	Zero
	2018-07-17 10:01:00+0000 to 2018-07-17 10:15:00+0000	Span
	2018-07-17 10:16:00+0000 to 2018-07-17 10:30:00+0000	Spare
	2018-07-17 10:31:00+0000 to 2018-07-17 10:35:00+0000	Purge
	2018-07-18 09:46:00+0000 to 2018-07-18 10:00:00+0000	Zero
	2018-07-18 10:01:00+0000 to 2018-07-18 10:15:00+0000	Span
	2018-07-18 10:16:00+0000 to 2018-07-18 10:30:00+0000	Spare
	2018-07-18 10:31:00+0000 to 2018-07-18 10:35:00+0000	Purge
	2018-07-19 09:46:00+0000 to 2018-07-19 10:00:00+0000	Zero
	2018-07-19 10:01:00+0000 to 2018-07-19 10:15:00+0000	Span
	2018-07-19 10:16:00+0000 to 2018-07-19 10:30:00+0000	Spare
	2018-07-19 10:31:00+0000 to 2018-07-19 10:35:00+0000	Purge
	2018-07-19 14:18:00+0000 to 2018-07-19 14:29:00+0000	<Samp
	2018-07-19 14:19:00+0000 to 2018-07-19 14:28:00+0000	Down
	2018-07-19 14:32:00+0000	<Samp
	2018-07-20 09:46:00+0000 to 2018-07-20 10:00:00+0000	Zero
	2018-07-20 10:01:00+0000 to 2018-07-20 10:15:00+0000	Span
	2018-07-20 10:16:00+0000 to 2018-07-20 10:30:00+0000	Spare
	2018-07-20 10:31:00+0000 to 2018-07-20 10:35:00+0000	Purge
	2018-07-20 14:55:00+0000	<Samp
	2018-07-20 14:56:00+0000 to 2018-07-20 15:10:00+0000	Down
	2018-07-20 14:58:00+0000 to 2018-07-20 15:08:00+0000	NoData

Data Flags Recorded During Testing (Cont.)

FRM/FEM Monitor	Timestamp (UTC)	Flag
Teledyne API T265 (Data acquired via local transfer from OAQPS)	2018-07-20 18:53:00+0000 to 2018-07-20 18:54:00+0000	<Samp
	2018-07-20 18:55:00+0000 to 2018-07-20 18:56:00+0000	Down
	2018-07-20 18:58:00+0000	<Samp
	2018-07-20 18:59:00+0000	Down
	2018-07-21 09:46:00+0000 to 2018-07-21 10:00:00+0000	Zero
	2018-07-21 10:01:00+0000 to 2018-07-21 10:15:00+0000	Span
	2018-07-21 10:16:00+0000 to 2018-07-21 10:30:00+0000	Spare
	2018-07-21 10:31:00+0000 to 2018-07-21 10:35:00+0000	Purge
	2018-07-22 09:46:00+0000 to 2018-07-22 10:00:00+0000	Zero
	2018-07-22 10:01:00+0000 to 2018-07-22 10:15:00+0000	Span
	2018-07-22 10:16:00+0000 to 2018-07-22 10:30:00+0000	Spare
	2018-07-22 10:31:00+0000 to 2018-07-22 10:35:00+0000	Purge
	2018-07-23 09:46:00+0000 to 2018-07-23 10:00:00+0000	Zero
	2018-07-23 10:01:00+0000 to 2018-07-23 10:15:00+0000	Span
	2018-07-23 10:16:00+0000 to 2018-07-23 10:30:00+0000	Spare
	2018-07-23 10:31:00+0000 to 2018-07-23 10:35:00+0000	Purge
	2018-07-23 20:12:00+0000	<Samp

Meteorological Instrument	Timestamp (UTC)	Flag
RM Young 41382 VC Temperature and Relative Humidity Monitor (Data acquired via local transfer from OAQPS)	2018-06-28 16:24:00+0000	NoData
	2018-06-28 16:25:00+0000	<Samp
	2018-07-10 15:43:00+0000	NoData
	2018-07-12 21:24:00+0000	NoData
	2018-07-12 21:27:00+0000	NoData
	2018-07-13 16:26:00+0000	<Samp
	2018-07-23 20:12:00+0000	<Samp
