# Supplementary material for: Sensortoolkit—A Python Library for Standardizing the Ingestion, Analysis, and Reporting of Air Sensor Data for Performance Evaluation
Source: Sensors (Basel). 2025 Sep 10;25(18):5645. doi: 10.3390/s25185645 (PMC12473223; doi:10.3390/s25185645)
Supplement: Supplementary file 1 [file sensors-25-05645-s001.zip › Supplemental4_EPA_LTPP_PM25_Test_Reports2022.pdf]

Long Term Performance Project
Appendix A
$PM_{2.5}$ Base Testing Reports

Initial Base Testing

U.S. Environmental Protection Agency
Office of Research and Development
PI: Clements.Andrea@epa.gov
919-541-1363

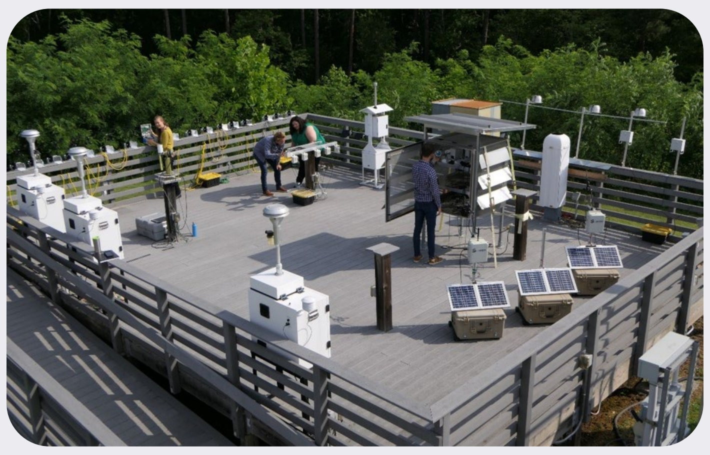

Disclaimer

This document has been reviewed by the U.S. Environmental Protection Agency (EPA), Office of Research and Development, and approved for publication. Any mention of trade names, products, or services does not imply an endorsement by the U.S. Government or the U.S. Environmental Protection Agency. The EPA does not endorse any commercial products, services, or enterprises. Links to websites outside the EPA website are provided for the convenience of the user. Inclusion of information about a website, an organization, a product or a service does not represent endorsement or approval by EPA, nor does it represent EPA opinion, policy or guidance unless specifically indicated. EPA does not exercise any editorial control over the information that may be found at any non-EPA website.

Aeroqual Ltd. provided sensor technologies as part of a Cooperative Research and Development Agreement (CRADA, #934-16) with the EPA. The views expressed in this document are solely those of the authors and EPA does not endorse any products or commercial services mentioned in this publication.

Table of Contents

Base Testing Reports (organized by testing site)

Phoenix, Arizona
- Aeroqual AQY
- APT Maxima
- Clarity Node
- PurpleAir PA-II-SD
- SENSIT RAMP
- QuantAQ ARISense

Denver, Colorado
- Aeroqual AQY
- APT Maxima
- Clarity Node
- PurpleAir PA-II-SD
- SENSIT RAMP
- QuantAQ ARISense

Wilmington, Delaware
- Aeroqual AQY
- APT Maxima
- Clarity Node
- PurpleAir PA-II-SD
- SENSIT RAMP
- QuantAQ ARISense

Decatur, Georgia
- Aeroqual AQY
- APT Maxima
- Clarity Node
- PurpleAir PA-II-SD
- SENSIT RAMP
- QuantAQ ARISense

Research Triangle Park, North Carolina
- Aeroqual AQY
- APT Maxima
- Clarity Node
- PurpleAir PA-II-SD
- SENSIT RAMP
- QuantAQ ARISense

Edmond, Oklahoma
- Aeroqual AQY
- APT Maxima
- Clarity Node
- PurpleAir PA-II-SD
- SENSIT RAMP
- QuantAQ ARISense

Milwaukee, Wisconsin
- Aeroqual AQY
- APT Maxima
- Clarity Node
- PurpleAir PA-II-SD
- SENSIT RAMP
- QuantAQ ARISense

Initial Base Testing
U.S. Environmental Protection Agency
Office of Research and Development
PI: Clements.Andrea@epa.gov
919-541-1363

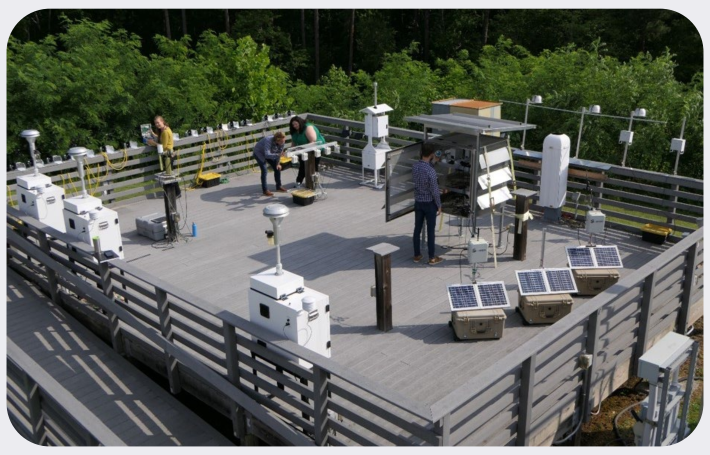

List of Acronyms and Abbreviations

µg/m³	Micrograms per cubic meter
AIRS	Ambient Air Innovation and Research Site
AQS	Air Quality System
AZ	Arizona
CF	Correction factor
CFR	Code of Federal Regulations
CO (state)	Colorado
CO (pollutant)	Carbon monoxide
CRADA	Cooperative Research and Development Agreement
CV	Coefficient of variation
DE	Delaware
Dichot	Dichotomous
DMAS	Denver Municipal Animal Shelter
EDT	Eastern Daylight Time
EPA	United States Environmental Protection Agency
FDMS	Filer Dynamics Measurement System
FEM	Federal Equivalent Method
FRM	Federal Reference Method
GA	Georgia
IDE	Integrated Development Environment
MCAQD	Maricopa County Air Quality Department
NC	North Carolina
NCore	National Core (Multipollutant Network)
NO	Nitrogen monoxide
NO2	Nitrogen dioxide
NOx	Oxides of nitrogen
NOy	Reactive nitrogen compounds
NRMSE	Normalized root mean squared error
O3	Ozone
OAQPS	Office of Air Quality Planning and Standards
OEM	Original Equipment Manufacturer
OK	Oklahoma
ORD	Office of Research and Development
PI	Principal Investigator
PM10	Particulate matter with aerodynamic diameter less than 10 microns
PM2.5	Particulate matter with aerodynamic diameter less than 2.5 microns
QA	Quality assurance
QAPP	Quality Assurance Project Plan
QC	Quality control
RH	Relative humidity
RMSE	Root mean square error
RTP	Research Triangle Park
SD	Standard deviation
SLAMS	State or Local Air Monitoring Stations Network
SO2	Sulfur dioxide
SOP	Standard Operating Procedure
SSID	Service set identifier
T	Temperature
TEOM	Tapered Element Oscillating Microbalance
UI	User Interface
USB	Universal Serial Bus
WDNR	Wisconsin Department of Natural Resources
WI	Wisconsin

Testing Report - $PM_{2.5}$ Base Testing

Aeroqual AQY

This report reflects out-of-the-box performance

Initial Base Testing - Phoenix, AZ
U.S. Environmental Protection Agency
Office of Research and Development
PI: Clements.Andrea@epa.gov
919-541-1363
September 2019—October 2019

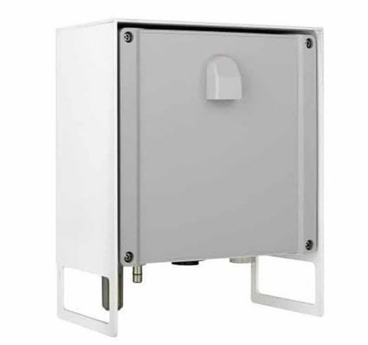

Image Attribution: Aeroqual Limited (Auckland, New Zealand)

Deployment Details

Testing Organization and Site Information	
Testing organization (Name, Organization type, Contact website)	U.S. Environmental Protection Agency - Office of Research and Development<br>Federal Government<br>Air Sensor Toolbox | U.S. EPA Website
Testing location (City, State, Latitude and Longitude)	West Phoenix<br>Phoenix, AZ<br>33.48385, -112.14257
AQS site ID	04 - 013 - 0019
Sampling timeframe (MM-DD-YY)	09-16-19 to 10-16-19
Sensor data source	Aeroqual Cloud download
Reference data source	AQS API download

Sensor Information			
Manufacturer, model	Aeroqual AQY		
Device firmware version	1.14.2		
Sampling time interval	1-minute		
Sensor serial numbers	AQ_01		
Issues encountered during deployment?	☑	See AZ-AQY-Page 6 of this testing report	

FRM/FEM Information	
Manufacturer, model, designation	Thermo Scientific TEOM 1405-DF Dichot. with FDMS FEM
Sampling time interval	1-hour averaging
Date of calibration	As required by 40 CFR Part 58 and the Air Monitoring Network Plan maintained by MCAQD
Date of flowrate verification check	Monthly as required by 40 CFR Part 58 Appendix A
Description, date(s) of maintenance activities	N/A

Time Series Plots

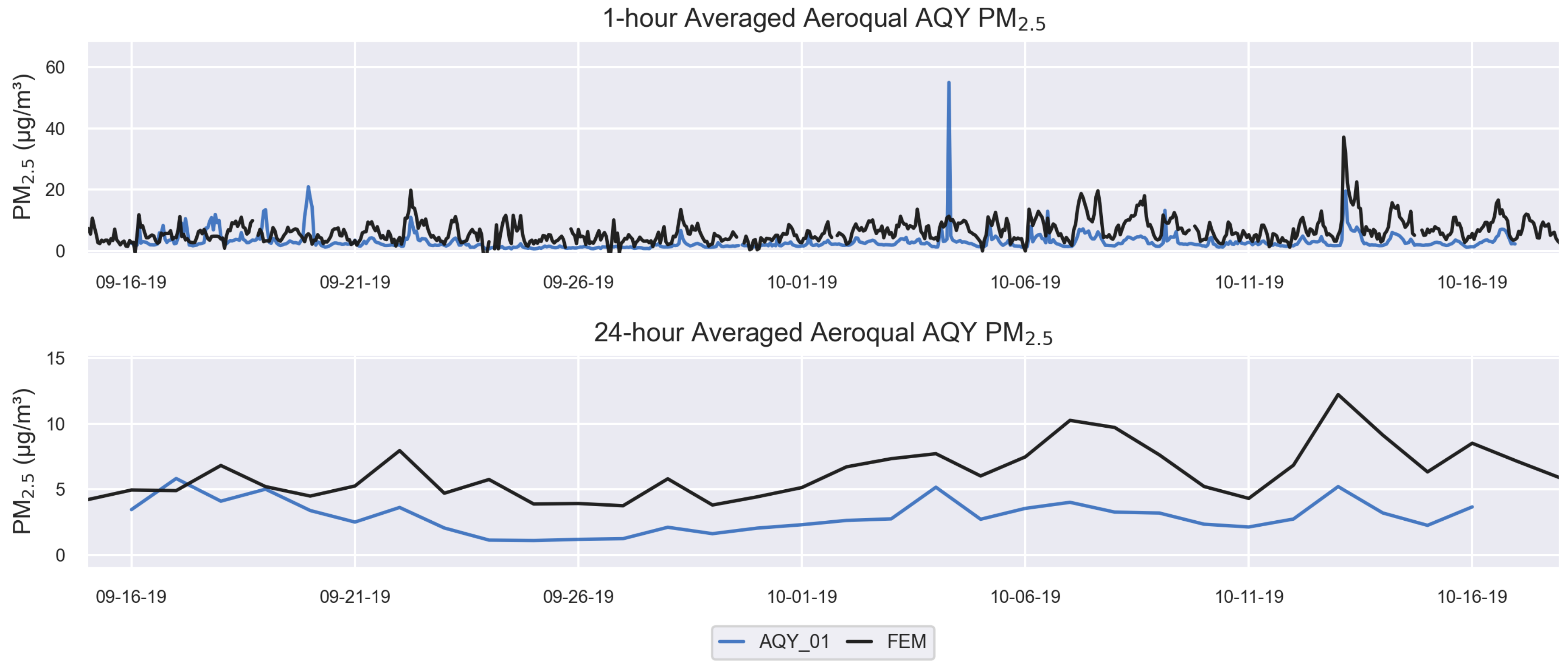

Scatter Plots: Comparison to FRM/FEM

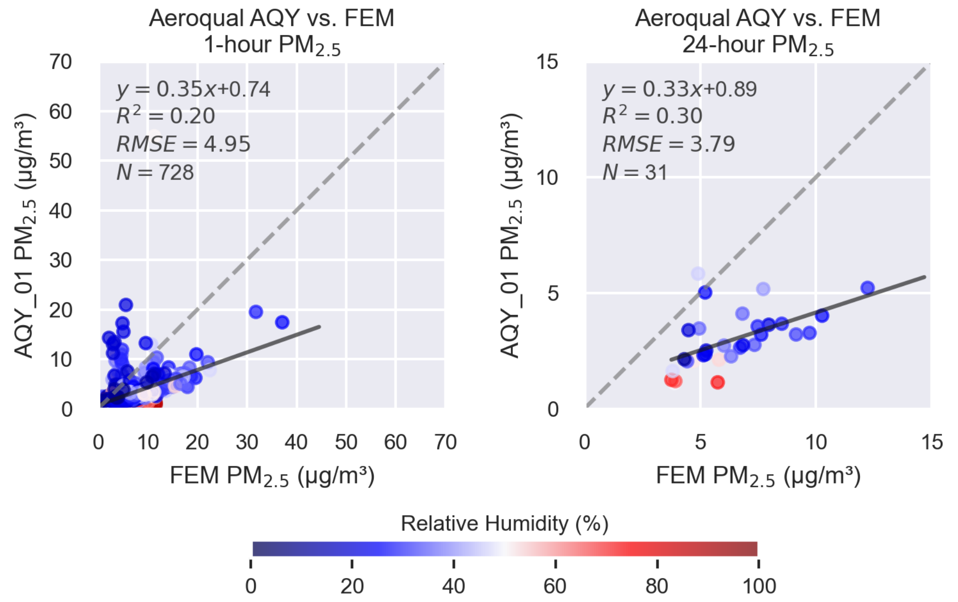

Range of FRM/FEM concentrations over duration of base test (μg/m³)	-4.4-37.1 (1-hr),<br>3.8-12.2 (24-hr)
Number of 24-hr periods in FRM/FEM monitor measurements with a goal concentration ≥ 25 μg/m³	0

Performance Metrics

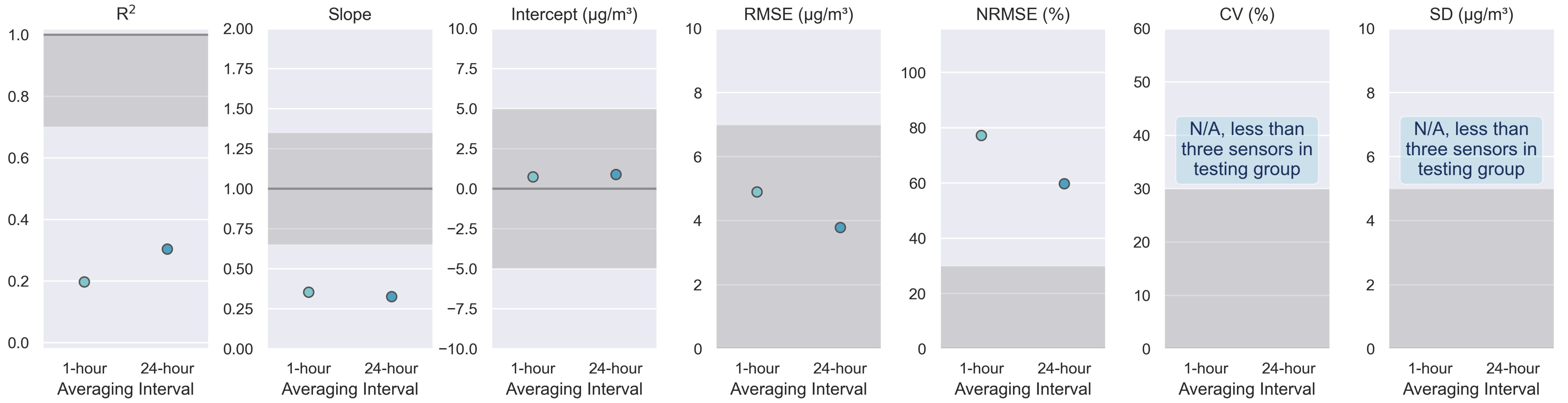

Meteorological Conditions During Deployment

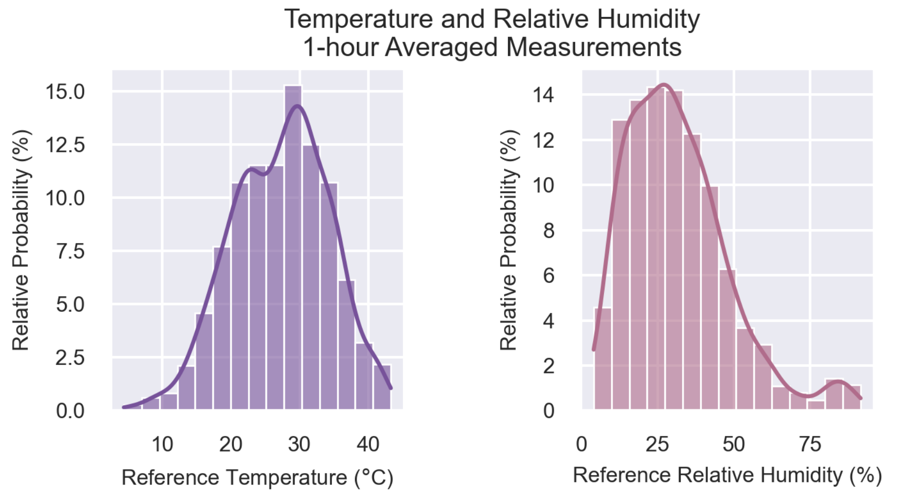

Number of 24-hr periods outside sensor manufacture-listed temperature operational range (-10 to 40 °C)	0
Number of 24-hr periods outside sensor manufacture-listed relative humidity operational range (no operational range specified)	-

Meteorological Influence

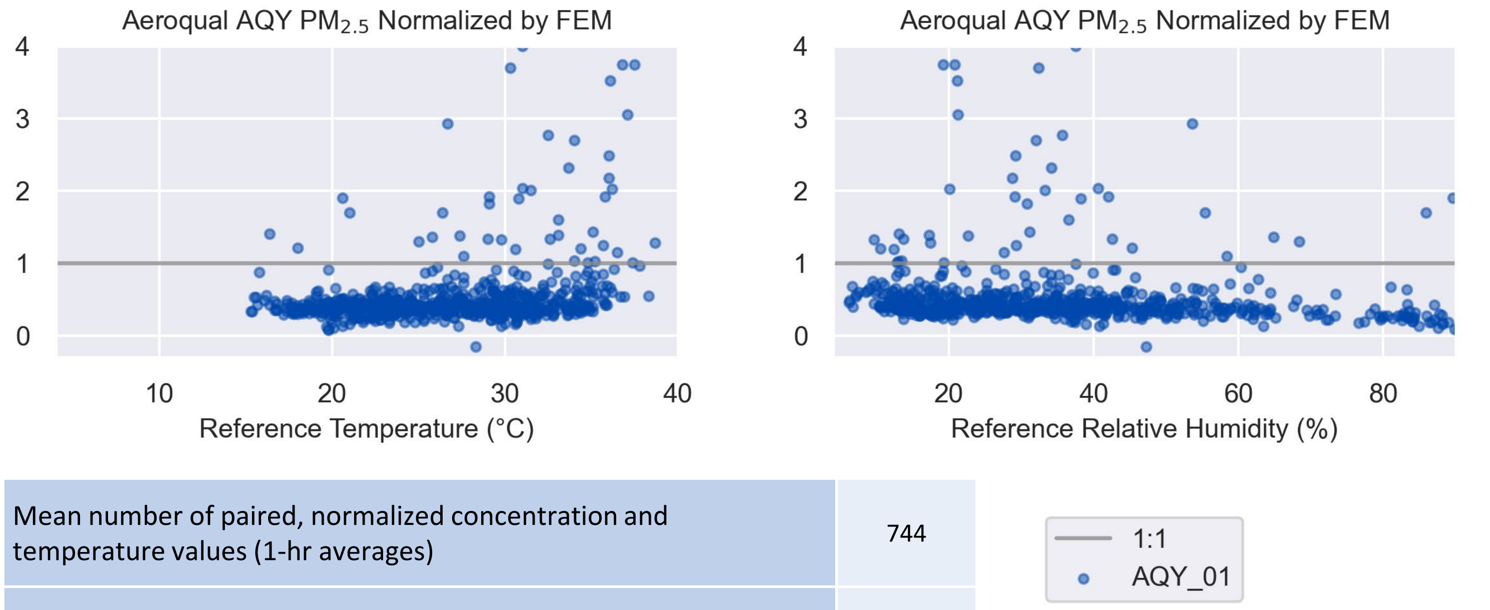

Mean number of paired, normalized concentration and temperature values (1-hr averages)	744
Mean number of paired, normalized concentration and relative humidity values (1-hr averages)	744

Testing Report - $PM_{2.5}$ Base Testing

Aeroqual AQY

This report reflects out-of-the-box performance

Initial Base Testing - Phoenix, AZ

U.S. Environmental Protection Agency
Office of Research and Development
PI: Clements.Andrea@epa.gov
919-541-1363

September 2019—October 2019

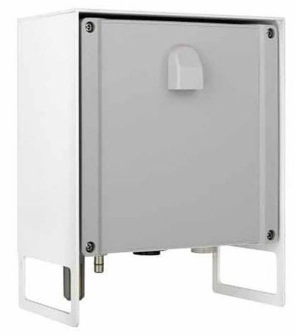

Image Attribution: Aeroqual Limited (Auckland, New Zealand)

Tabular Statistics

Sensor-FRM/FEM Correlation

	Bias and Linearity						Data Quality			
	R^2		Slope		Intercept (µg/m³)		Uptime (%)		Number of paired sensor and FRM/FEM concentration values	
	1-Hour ○	24-Hour ○	1-Hour ○	24-Hour ○	1-Hour ●	24-Hour ●	1-Hour ●	24-Hour ●	1-Hour	24-Hour
Metric Target Range	≥ 0.70	≥ 0.70	1.0 ± 0.35	1.0 ± 0.35	-5 ≤ b ≤ 5	-5 ≤ b ≤ 5	75%*	75%*	-	-
Sensor AQY_01	0.20	0.30	0.35	0.33	0.74	0.89	100	100	728	31

	Error			
	RMSE (µg/m³)		NRMSE (%)	
	1-Hour ★	24-Hour ★	1-Hour ☆	24-Hour ☆
Metric Target Range	≤ 7.0	≤ 7.0	≤ 30.0	≤ 30.0
Deployment Value	4.9	3.8	77.2	59.8

Device-specific metrics (computed for each sensor in evaluation)
○○○ Metric value for none of devices tested falls within the target range
●○○ Metric value for one of devices tested falls within the target range
●●○ Metric value for two of devices tested falls within the target range
●●● Metric value for three of devices tested falls within the target range

Single-valued metrics (computed via entire evaluation dataset)
☆ Indicates that the metric value is not within the target range
★ Indicates that the metric value is within the target range

Sensor-Sensor Precision[1]

	Precision (between collocated sensors)				Data Quality	
	CV (%)		SD (µg/m³)		Number of concurrent sensor concentration pairs	
	1-Hour ☆	24-Hour ☆	1-Hour ☆	24-Hour ☆	1-Hour	24-Hour
Metric Target Range	≤ 30.0	≤ 30.0	≤ 5.0	≤ 5.0	-	-
Deployment Value	-	-	-	-	-	-

[1]Precision statistics are computed for evaluations with at least three collocated sensor units. Metric values are left blank for evaluations with two or fewer sensor units.

*This value is only a recommendation for ensuring data quality and is not included in the list of target values discussed in Section 4 of the Performance Testing Protocols, Metrics, and Target Values for Fine Particulate Matter Air Sensors document.

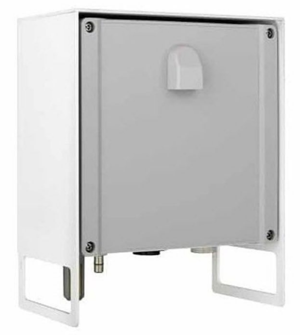

Image Attribution: Aeroqual Limited (Auckland, New Zealand)

Sensor-FRM/FEM Scatter Plots

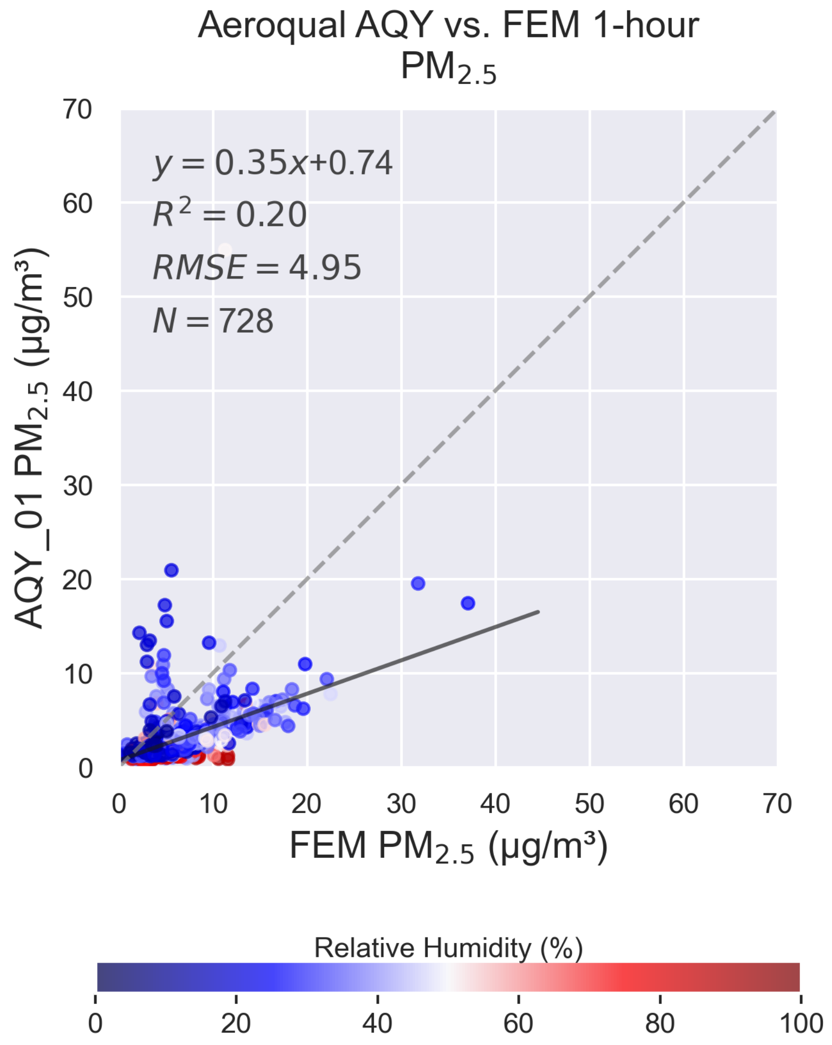

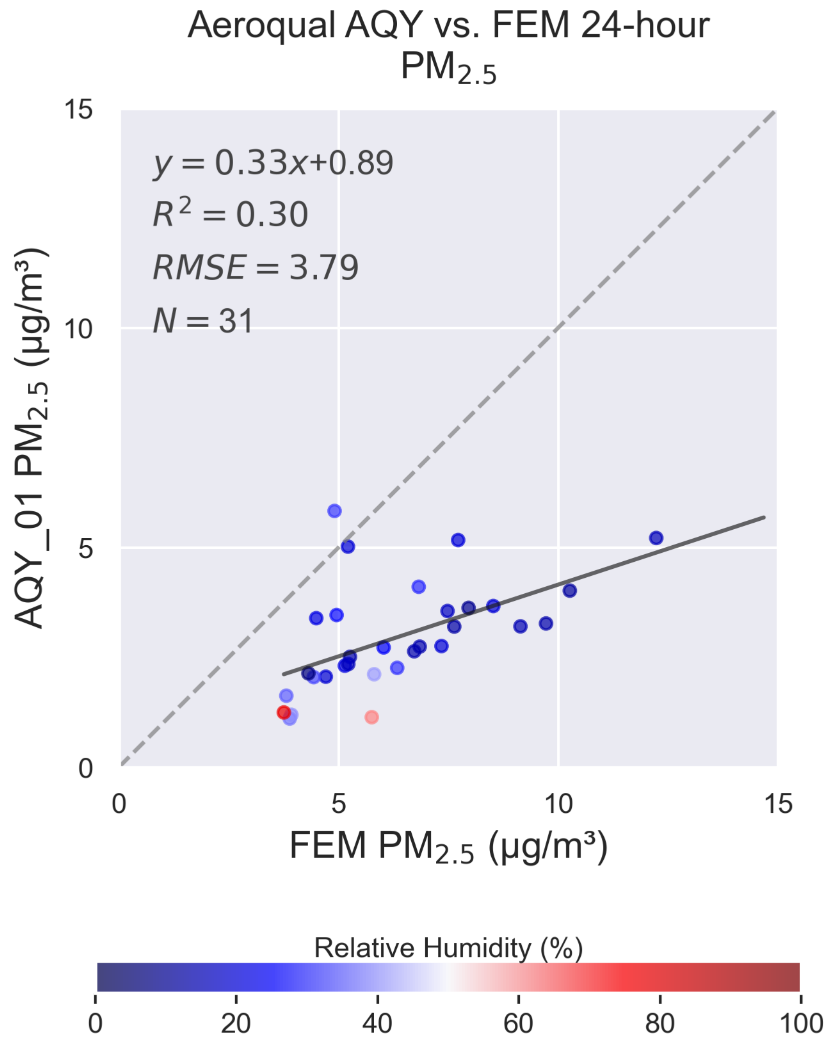

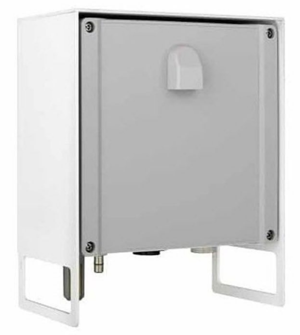

Image Attribution: Aeroqual Limited (Auckland, New Zealand)

Supplemental Information

Abbreviations used in Supplemental Information

FRM	Federal Reference Method
FEM	Federal Equivalent Method
SOP	Standard Operating Procedure
QAPP	Quality Assurance Project Plan
QC	Quality Control

Supplemental Documentation	Attached	Description & URL or file path to documentation
Field observations and sensor data flags	☑	See AZ-AQY-Page 6 of this testing report
Maintenance logs	☐	No logs recorded during testing
Standard operating procedure(s)	☐	U.S. EPA Office Of Research and Development SOP available upon request
Photos of equipment setup and testing	☑	See AZ-AQY-Page 5 of this testing report
Product specifications sheet(s)	☐	N/A*
Product manual(s)	☐	N/A*
Data storage and transmission method	☑	See AZ-AQY-Page 6 of this testing report
Data correction approach	☑	See AZ-AQY-Page 6 of this testing report
Issues encountered	☑	See AZ-AQY-Page 6 of this testing report
Data analysis/correction scripts and version	☑	Averaging and processing of data, calculation of performance metrics, and generation of figures and other supplementary material for analysis were obtained using Python 3.9.7 with the packages sensortoolkit v0.8.3b2, pandas 1.3.5, NumPy 1.21.2, Matplotlib 3.5.0, statsmodels 0.13.0, and seaborn 0.11.2. All packages are available from the Python Package Index (PyPI) at https://pypi.org/. The integrated development environment (IDE) Spyder 5.1.5 was used for scripting and data visualization. Version control for the Python base, packages, and IDE were all managed by conda 4.11.0.
Air Monitoring Station QAPP	☐	U.S. EPA Office Of Research and Development QAPP available upon request
Summary of FRM/FEM monitor QC checks	☑	See AZ-AQY-Page 7 of this testing report
Manufacturer website for FRM/FEM monitor	☑	Thermo Fisher Scientific: TEOM 1405 Product website
FRM/FEM monitor manual	☑	Thermo Fisher Scientific: TEOM 1405 Product Manual
FRM/FEM monitor specifications sheet(s)	☑	Thermo Fisher Scientific: TEOM 1405 Specification Sheet
Other documents	☑	Manufacturer notice of AQY sales on hold

*As of 3/18/2021, the manufacturer of the AQY has placed sales of a similar unit on hold. Documentation for the AQY is currently unavailable from the manufacturer's website.

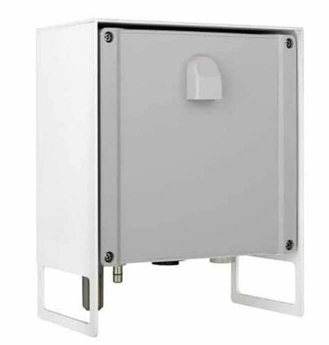

Image Attribution: Aeroqual Limited (Auckland, New Zealand)

Supplemental Information: Photos of Testing Site and Equipment Setup

Site Description:
The West Phoenix Monitoring Station has been operational since 1984. The spatial scale for the West Phoenix site is Neighborhood. It is located in an area of stable, high-density residential properties. This State or Local Air Monitoring Stations (SLAMS) location monitors for CO, NO_2, O_3, PM_{10}, and $PM_{2.5}$. In addition, this is a quality assurance (QA) collocation site for $PM_{2.5}$ where the Maricopa County Air Quality Department (MCAQD) operates one filter-based $PM_{2.5}$ FRM sampler along with one continuous $PM_{2.5}$ FEM analyzer as per 40 CFR Part 58 Appendix A. Resources detailing air quality monitoring QA programs and procedures are detailed on EPA's Ambient Monitoring Technology Information Center website (https://www.epa.gov/amtic/ambient-air-monitoring-quality-assurance, *last accessed 5/11/2022*). Meteorological monitors operating at this site measure ambient temperature (T), barometric pressure, delta T (temperature inversion), and wind speed/direction.

Figure 1: Aeroqual AQY sensor (indicated by red arrow) attached to metal railing atop the sampling shelter at the monitoring site.

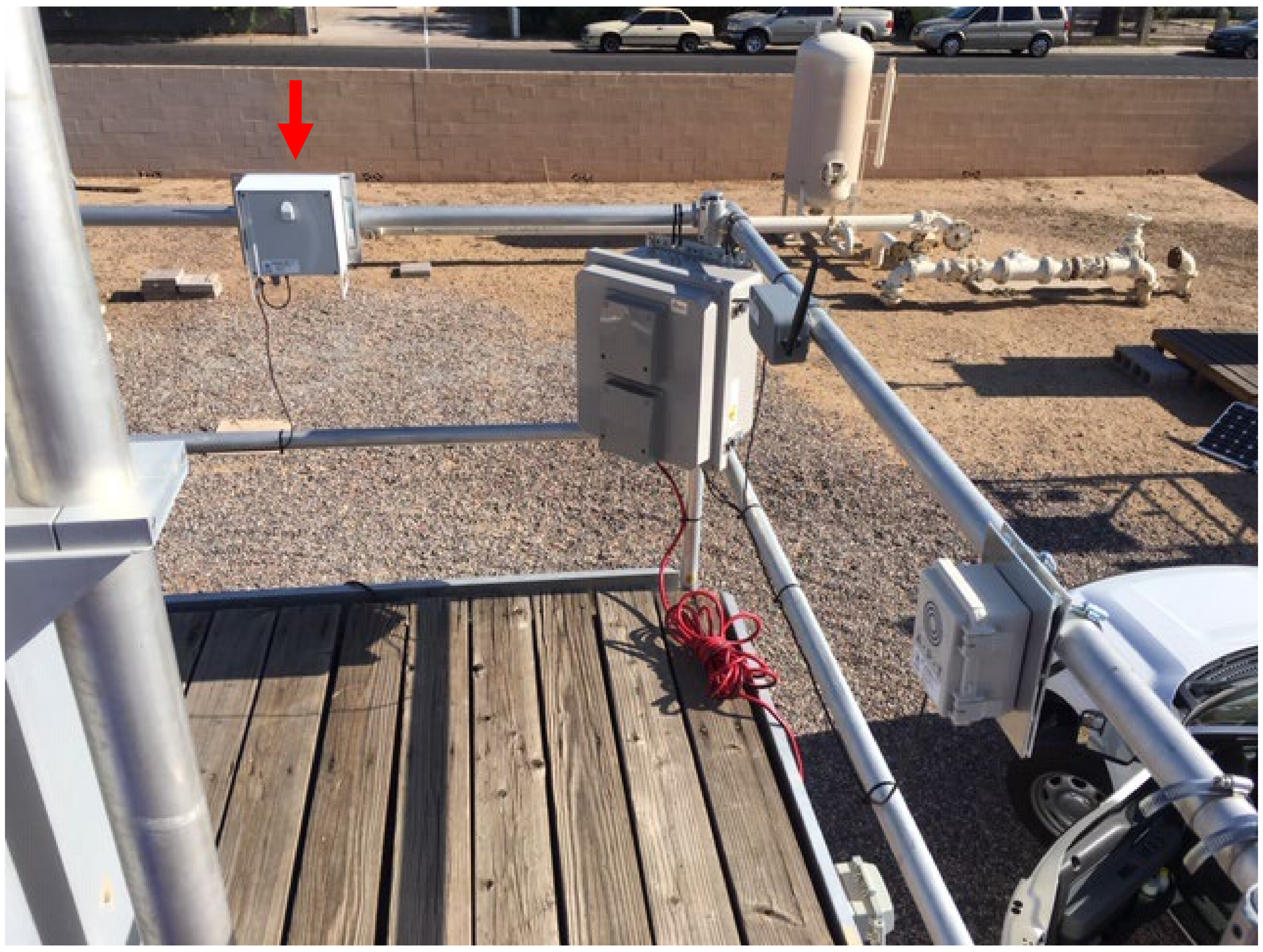

Figure 2: West Phoenix Monitoring Station

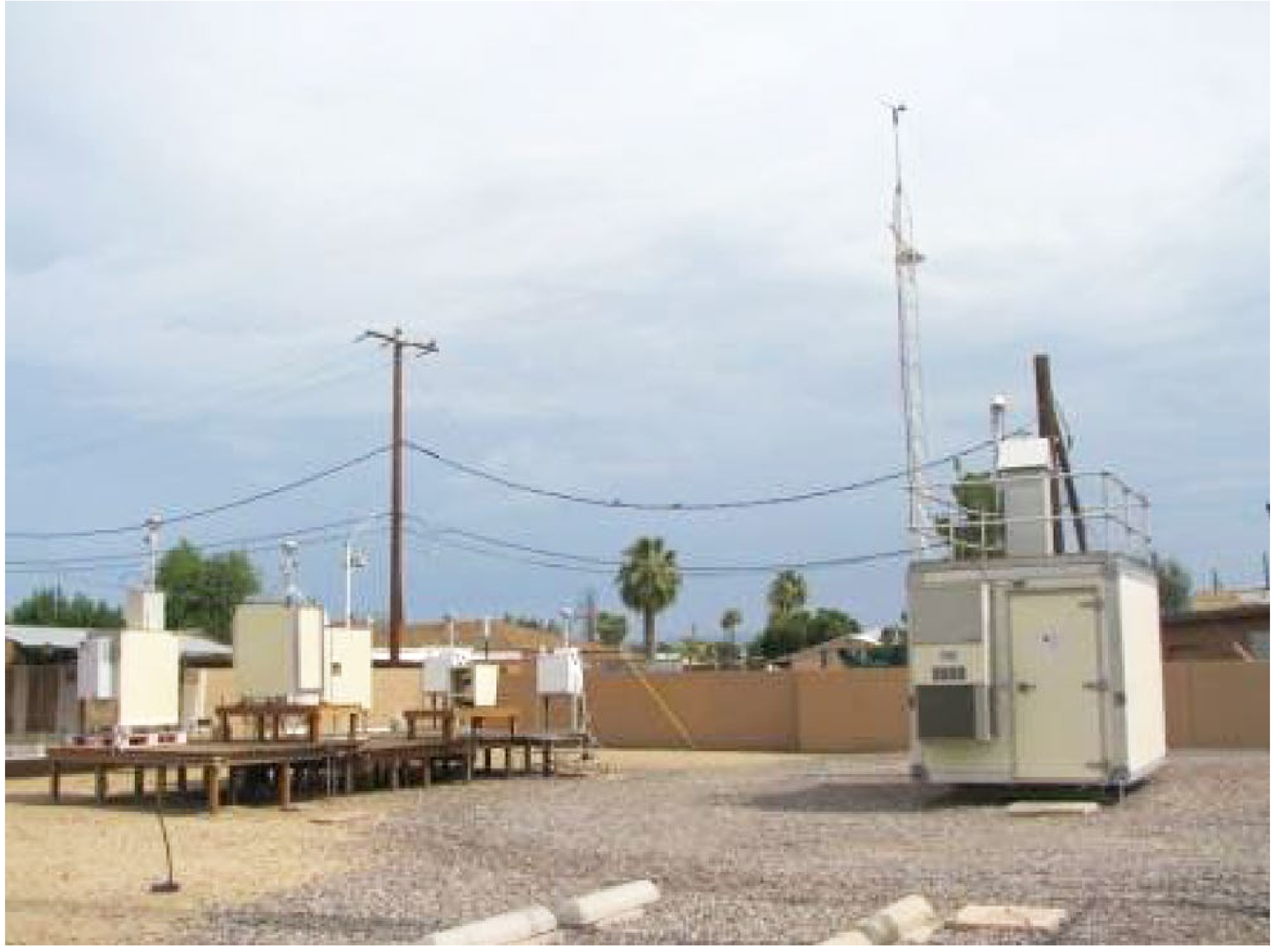

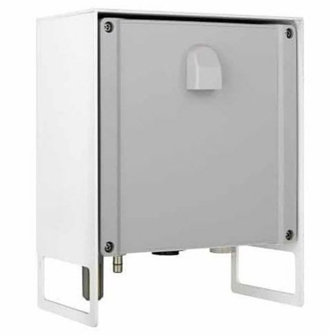
Image Attribution: Aeroqual Limited (Auckland, New Zealand)

Data Storage and Transmission Method

As part of CRADA #934-16 between Aeroqual and US EPA, Aeroqual supported data streaming. SIM cards were installed and data flowed to the Aeroqual Cloud. The 1-minute raw data was acquired weekly using the Aeroqual Cloud (*last accessed 5/11/22*) user interface (UI). The AQY has an internal data storage USB flash drive as a data backup, however the software on the drive is proprietary to Aeroqual and unreadable by outside systems.

Data Correction Approach

This evaluation report reflects "out-of-the-box" performance of the AQY. The manufacturer provides a procedure by which local collocation (sensor operated along side an FRM/FEM) data can be collected, a gain (slope) and offset (intercept) determined, and parameters entered into the Aeroqual Cloud user interface to be applied to all subsequently collected data. This procedure and feature was NOT used prior to this evaluation. Prospective consumers may get different performance from this device if they utilize this feature.

After acquisition, the raw data was processed using the *sensortoolkit* python code library (v0.8.3b2). A continuous data set at the recorded sampling frequency was written to a .csv file. 1-hour and 24-hour averaged data sets were generated using a 75% completeness threshold and saved as separate .csv files. Outliers were NOT removed from data sets in order to assess "out-of-the-box" sensor performance.

Issues Encountered

Pre-deployment observations

- *Timestamp inaccuracies*: During pre-deployment, the AQY devices did not properly sync timestamps with the onboard Real-Time Clock. Connecting the units to the internet by cellular or Wi-Fi allowed the unit to sync with internet time and resulted in proper timestamps.
- *Concentration baseline offset*: The AQY unit experienced a large baseline offset from approximately 2019-09-02 018:00 MST to 2019-09-09 16:00 MST. As a result, $PM_{2.5}$ concentrations measured by the AQY exceeded 1000 μg/m^3 and significantly deviated from ambient concentration as measured by the FEM during the corresponding time frame. This failure mode has been observed during multiple U.S. EPA evaluations of the AQY model and is associated with dust buildup within the sensor's PM inlet. The inlet tubing was cleaned, and the unit was redeployed.
- *Unit connectivity error:* The AQY unit experienced initial startup connectivity issues from August 12, 2019, when the sensor arrived at the monitoring site, through August 15, 2019 when the issue was resolved.

Field observations and sensor data flags

The following table contains data flags describing events that were encountered during the testing period. A brief power loss to the sensor occurred on 9/29/2019 and lasted approximately 10 minutes. This event occurred when the field technician was not at the monitoring site, indicating that the outage was likely not attributed to operator error.

Start Time (UTC)	End Time (UTC)	Sensor Serial ID	Parameters Impacted	Flag
2019-09-29 14:30:00+00:00	2019-09-29 14:41:00+00:00	AQY_01	ALL	9-Data Loss - Power Connection Error

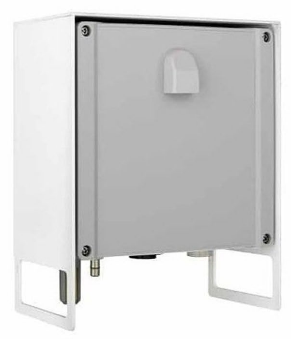

Image Attribution: Aeroqual Limited (Auckland, New Zealand)

Description of Data Flags

AQS

The U.S. EPA's Air Quality System (AQS) is the Agency's primary ambient air monitoring data archive. A comprehensive list of data flags that are recorded alongside AQS data sets, referred to by U.S. EPA as 'qualifiers', can be found at the following link: https://aqs.epa.gov/aqsweb/documents/codetables/qualifiers.html

AirNow-Tech

AirNow-Tech is an additional ambient air monitoring data service maintained by U.S. EPA and is commonly used by monitoring agencies to upload and validate monitoring data. Data which have yet to be QC'd for inclusion in AQS as well as monitoring data sets which are not planned for inclusion in AQS are typically available for near real-time download from AirNow-Tech.

Invalidation of reference data: AQS qualifiers are organized by qualifier type, which indicates whether data logged alongside qualifier flags should be invalidated (set null). Qualifiers with type "Null Data Qualifier" are invalidated, and includes data logged during periods that coincide with QC checks (e.g., "BF-Precision/Zero/Span", "BJ- Operator Error", "BL - QA Audit", "AZ - QC Audit") among other events such as power outages. Data logged alongside qualifiers with type "Quality Assurance Qualifiers" are not invalidated and are included in this analysis (e.g., concentrations less than the federal MDL for the reference monitor "MD – Value less than MDL", QA reviewed values "Validated Value").

Data Flags Recorded During Testing

FRM/FEM Monitor	Timestamp (UTC)	Flag
Thermo Fisher 1405-DF TEOM FDMS Dichotomous FEM (Data acquired via AQS)	2019-09-18 18:00:00+0000	BL - QA Audit
	2019-09-18 19:00:00+0000	BM - Accuracy check
	2019-09-24 01:00:00+0000 to 2019-09-24 02:00:00+0000	AV - Power Failure
	2019-09-25 18:00:00+0000 to 2019-09-25 19:00:00+0000	AY - QC Control Points (zero/span)
	2019-09-29 14:00:00+0000 to 2019-09-29 16:00:00+0000	AV - Power Failure
	2019-10-01 17:00:00+0000 to 2019-10-01 18:00:00+0000	BM - Accuracy check
	2019-10-09 17:00:00+0000 to 2019-10-09 18:00:00+0000	AY - QC Control Points (zero/span)
	2019-10-14 18:00:00+0000 to 2019-10-14 20:00:00+0000	BM - Accuracy check

Meteorological Instrument	Timestamp (UTC)	Flag
MetOne Temperature Monitor (Data acquired via AirNow-Tech)	2019-09-29 14:00:00+0000	9 - Invalid
	2019-10-01 19:00:00+0000 to 2019-10-01 20:00:00+0000	9 - Invalid
RM Young Relative Humidity Monitor (Data acquired via AirNow-Tech)	2019-09-29 14:00:00+0000	9 - Invalid

Testing Report - PM$_{2.5}$ Base Testing

APT Maxima

This report reflects out-of-the-box performance

Initial Base Testing - Phoenix, AZ
U.S. Environmental Protection Agency
Office of Research and Development
PI: Clements.Andrea@epa.gov
919-541-1363
July 2019—August 2019

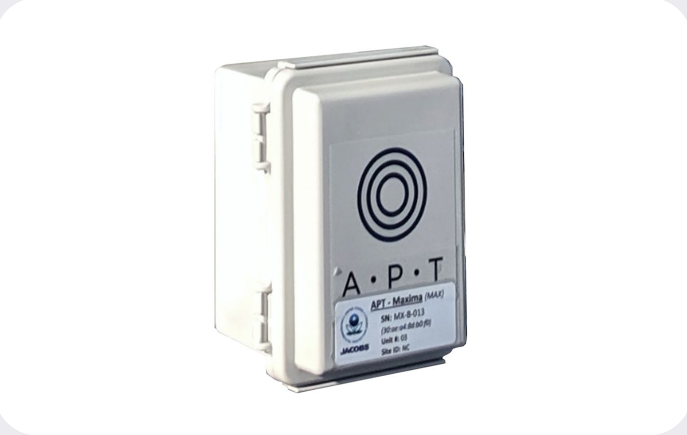

Deployment Details

Testing Organization and Site Information	
Testing organization (Name, Organization type, Contact website)	U.S. Environmental Protection Agency - Office of Research and Development<br>Federal Government<br>Air Sensor Toolbox | U.S. EPA Website
Testing location (City, State, Latitude and Longitude)	West Phoenix<br>Phoenix, AZ<br>33.48385, -112.14257
AQS site ID	04 - 013 - 0019
Sampling timeframe (MM-DD-YY)	07-28-19 to 08-27-19
Sensor data source	Onboard MicroSD card
Reference data source	AQS API download

Sensor Information			
Manufacturer, model	APT Maxima		
Device firmware version	v5.0 (installed 5/2/2019)		
Sampling time interval	30-seconds		
Sensor serial numbers	MAX_01		
Issues encountered during deployment?	☐	No Issues	

FRM/FEM Information	
Manufacturer, model, designation	Thermo Scientific TEOM 1405-DF Dichot. with FDMS FEM
Sampling time interval	1-hour averaging
Date of calibration	As required by 40 CFR Part 58 and the Air Monitoring Network Plan maintained by MCAQD
Date of flowrate verification check	Monthly as required by 40 CFR Part 58 Appendix A
Description, date(s) of maintenance activities	N/A

Time Series Plots

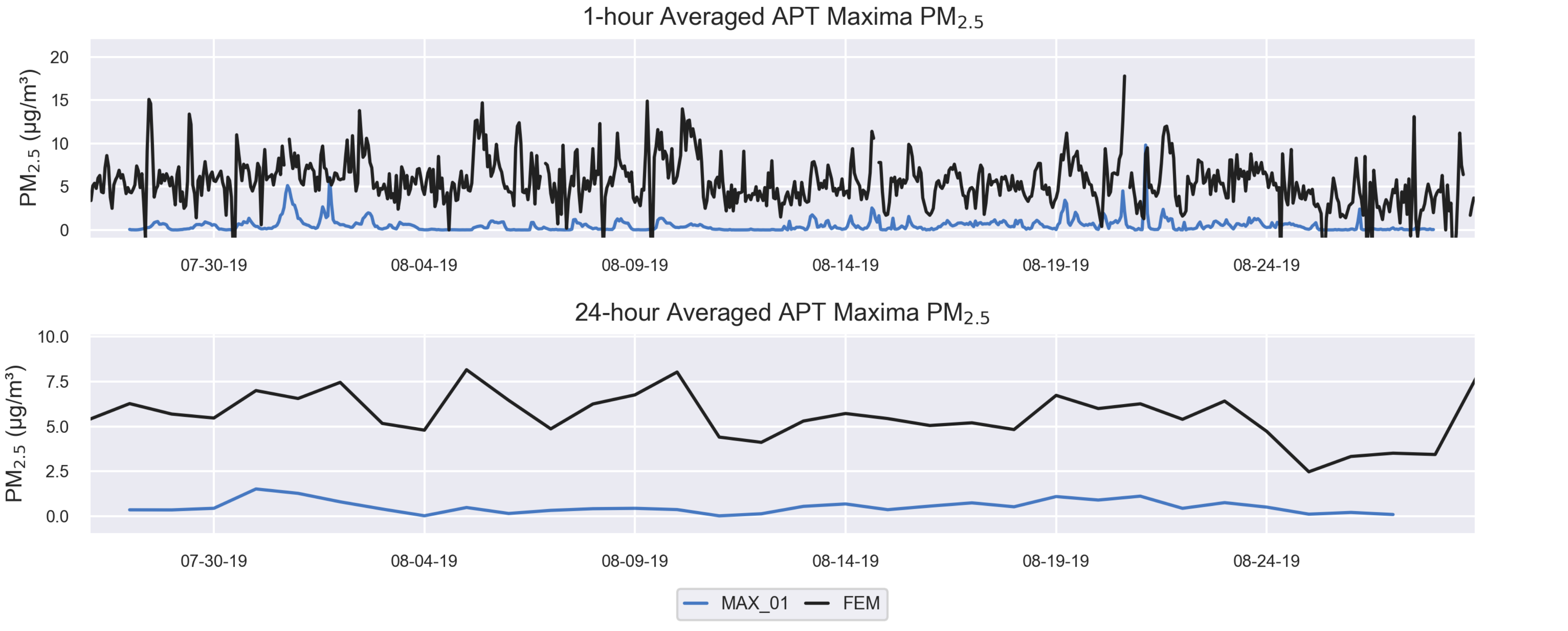

Scatter Plots: Comparison to FRM/FEM

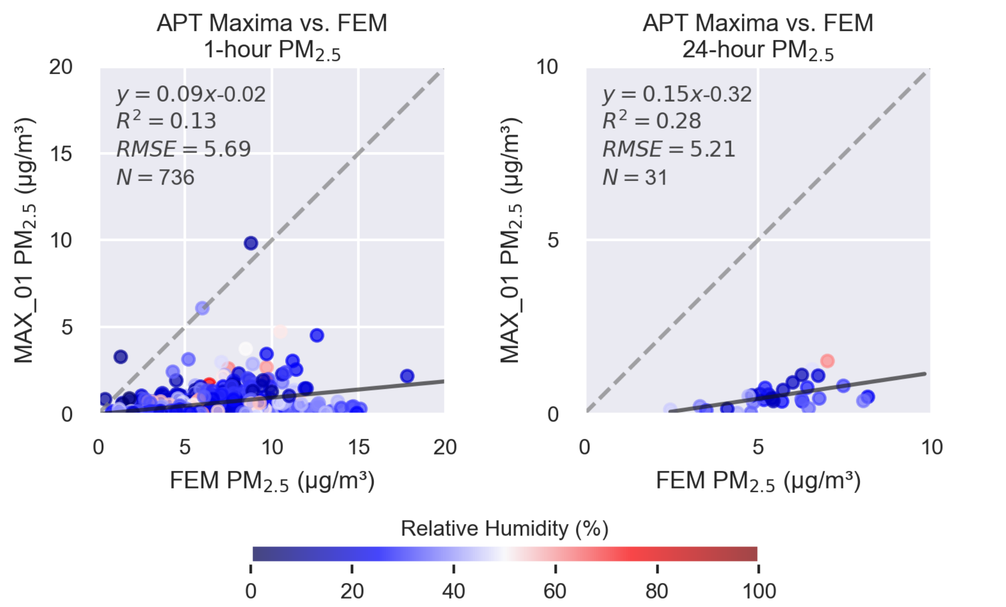

Range of FRM/FEM concentrations over duration of base test (μg/m³)	-3.8-17.8 (1-hr), 2.5-8.2 (24-hr)
Number of 24-hr periods in FRM/FEM monitor measurements with a goal concentration ≥ 25 μg/m³	0

Performance Metrics

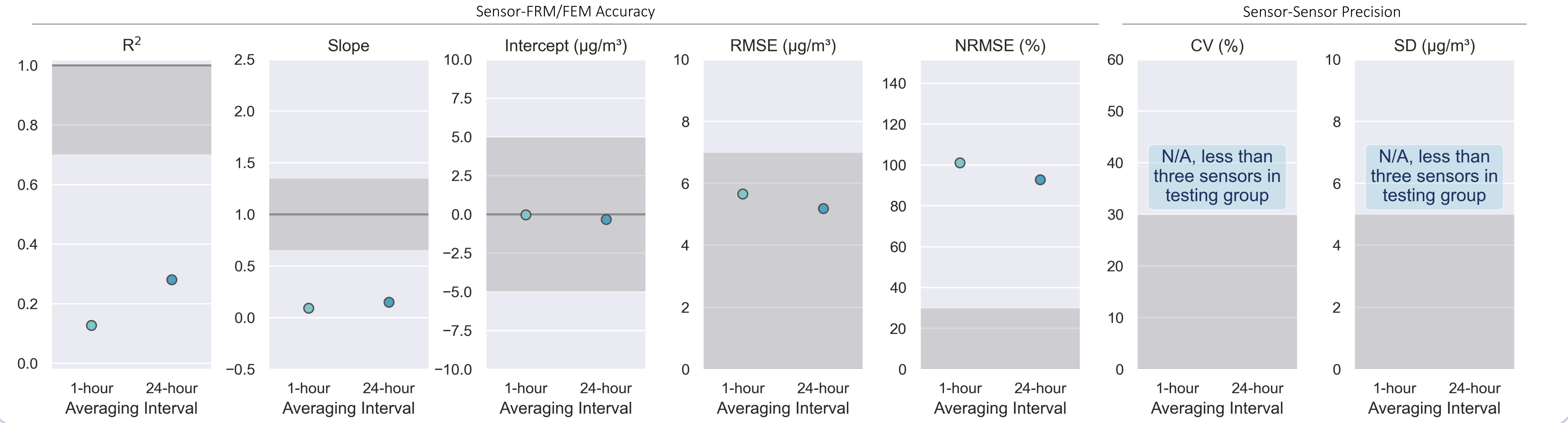

Meteorological Conditions During Deployment

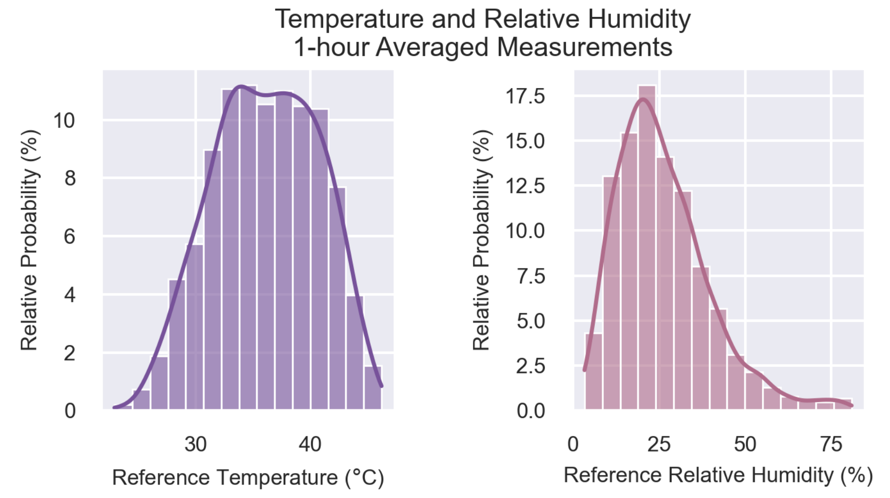

Number of 24-hr periods outside sensor manufacture-listed temperature operational range (no operational range specified)	-
Number of 24-hr periods outside sensor manufacture-listed relative humidity operational range (no operational range specified)	-

Meteorological Influence

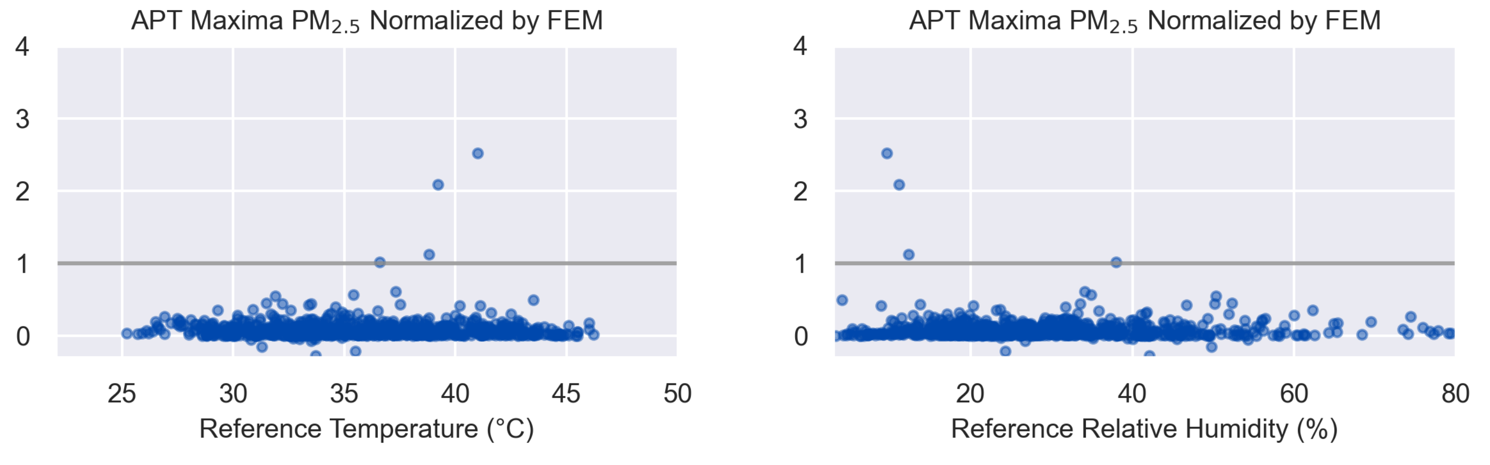

Mean number of paired, normalized concentration and temperature values (1-hr averages)	744
Mean number of paired, normalized concentration and relative humidity values (1-hr averages)	744

Testing Report - $PM_{2.5}$ Base Testing

APT Maxima

This report reflects out-of-the-box performance

Initial Base Testing - Phoenix, AZ
U.S. Environmental Protection Agency
Office of Research and Development
PI: Clements.Andrea@epa.gov
919-541-1363
July 2019—August 2019

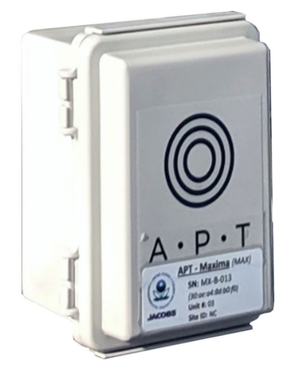

Tabular Statistics

Sensor-FRM/FEM Correlation

	Bias and Linearity						Data Quality			
	R^2		Slope		Intercept (µg/m³)		Uptime (%)		Number of paired sensor and FRM/FEM concentration values	
	1-Hour ○	24-Hour ○	1-Hour ○	24-Hour ○	1-Hour ●	24-Hour ●	1-Hour ●	24-Hour ●	1-Hour	24-Hour
Metric Target Range	≥ 0.70	≥ 0.70	1.0 ± 0.35	1.0 ± 0.35	-5 ≤ b ≤ 5	-5 ≤ b ≤ 5	75%*	75%*	-	-
Sensor MAX_01	0.13	0.28	0.09	0.15	-0.02	-0.32	100	100	736	31

	Error			
	RMSE (µg/m³)		NRMSE (%)	
	1-Hour ★	24-Hour ★	1-Hour ☆	24-Hour ☆
Metric Target Range	≤ 7.0	≤ 7.0	≤ 30.0	≤ 30.0
Deployment Value	5.7	5.2	101.1	92.8

Device-specific metrics (computed for each sensor in evaluation)
○○○ Metric value for none of devices tested falls within the target range
●○○ Metric value for one of devices tested falls within the target range
●●○ Metric value for two of devices tested falls within the target range
●●● Metric value for three of devices tested falls within the target range

Single-valued metrics (computed via entire evaluation dataset)
☆ Indicates that the metric value is not within the target range
★ Indicates that the metric value is within the target range

Sensor-Sensor Precision[1]

	Precision (between collocated sensors)				Data Quality	
	CV (%)		SD (µg/m³)		Number of concurrent sensor concentration pairs	
	1-Hour ☆	24-Hour ☆	1-Hour ☆	24-Hour ☆	1-Hour	24-Hour
Metric Target Range	≤ 30.0	≤ 30.0	≤ 5.0	≤ 5.0	-	-
Deployment Value	-	-	-	-	-	-

[1]Precision statistics are computed for evaluations with at least three collocated sensor units. Metric values are left blank for evaluations with two or fewer sensor units.

*This value is only a recommendation for ensuring data quality and is not included in the list of target values discussed in Section 4 of the Performance Testing Protocols, Metrics, and Target Values for Fine Particulate Matter Air Sensors document.

Testing Report - $PM_{2.5}$ Base Testing

APT Maxima

This report reflects out-of-the-box performance

Initial Base Testing - Phoenix, AZ

U.S. Environmental Protection Agency
Office of Research and Development
PI: Clements.Andrea@epa.gov
919-541-1363

July 2019—August 2019

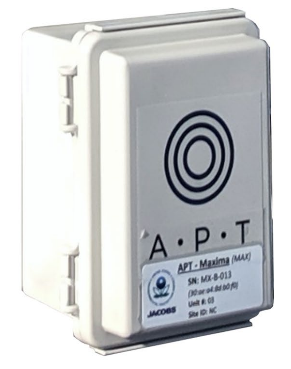

Sensor-FRM/FEM Scatter Plots

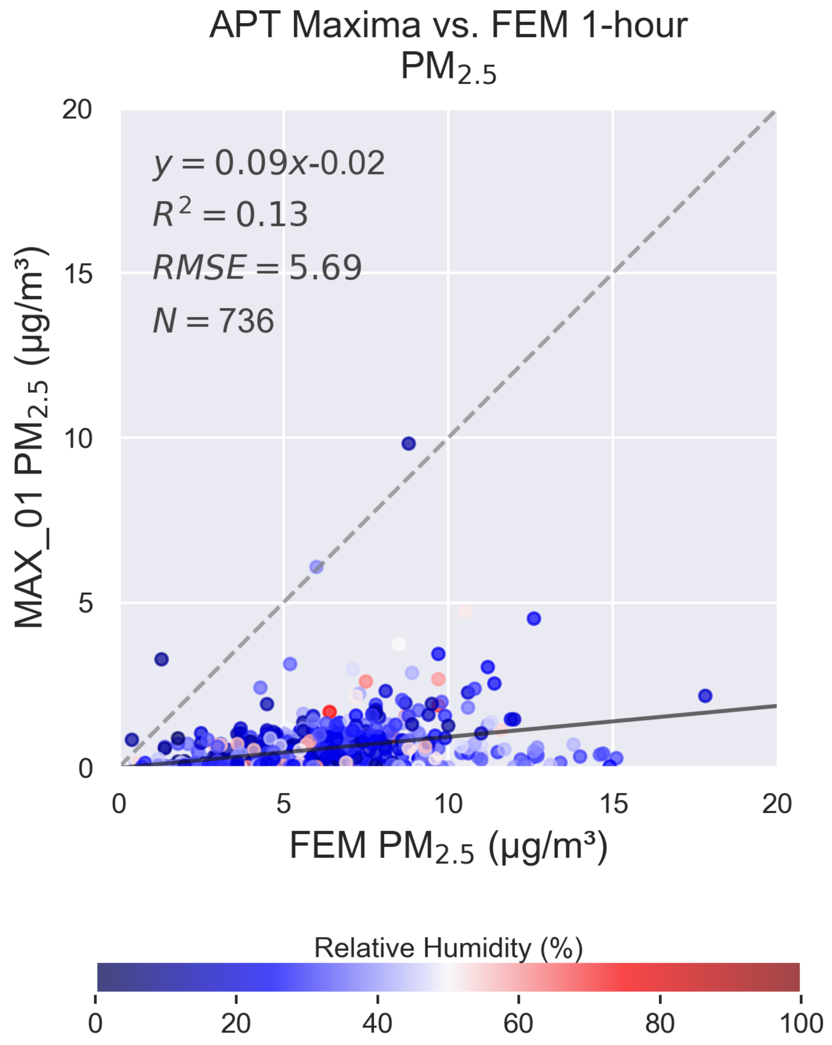

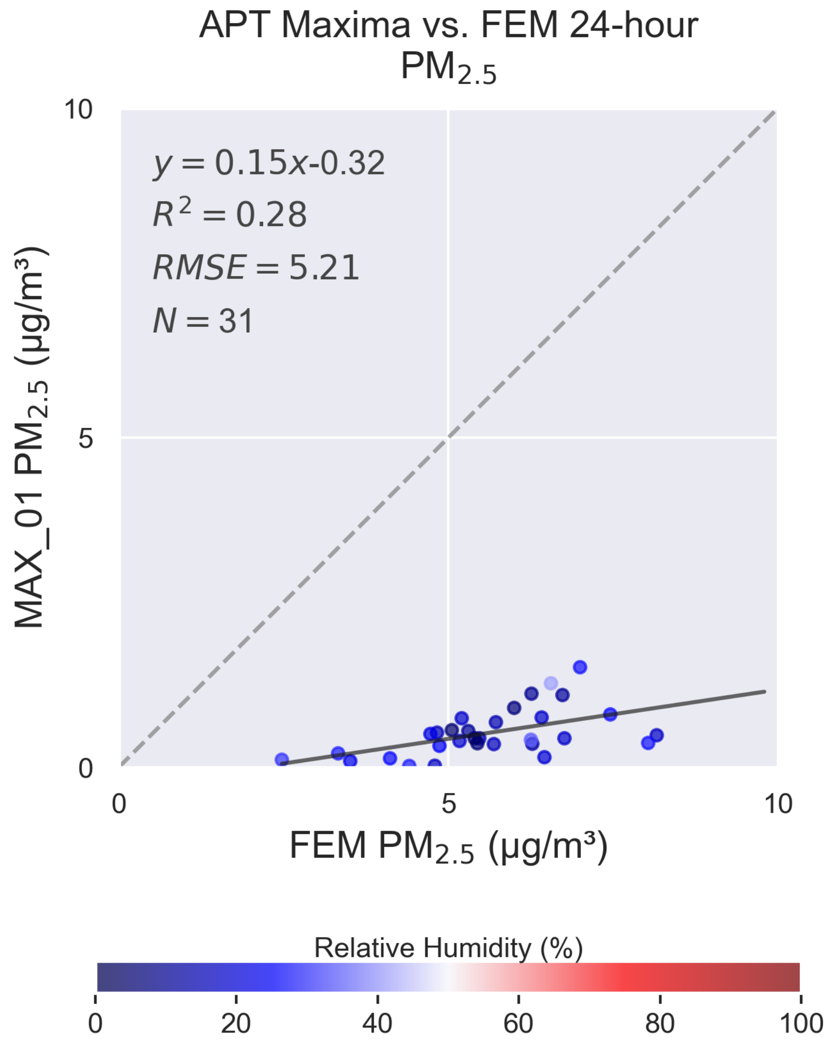

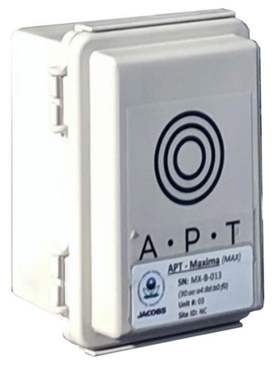

Supplemental Information

Abbreviations used in Supplemental Information

FRM	Federal Reference Method
FEM	Federal Equivalent Method
SOP	Standard Operating Procedure
QAPP	Quality Assurance Project Plan
QC	Quality Control

Supplemental Documentation	Attached	Description & URL or file path to documentation
Field observations and sensor data flags	☑	See AZ-MAX-Page 6 of this testing report
Maintenance logs	☐	No logs recorded during testing
Standard operating procedure(s)	☐	U.S. EPA Office Of Research and Development SOP available upon request
Photos of equipment setup and testing	☑	See AZ-MAX-Page 5 of this testing report
Product specifications sheet(s)	☐	N/A
Product manual(s)	☐	N/A
Data storage and transmission method	☑	See AZ-MAX-Page 6 of this testing report
Data correction approach	☑	See AZ-MAX-Page 6 of this testing report
Issues encountered	☑	See AZ-MAX-Page 6 of this testing report. No issues were encountered during testing, however, an abnormality in the duration of sampling intervals was noted during the pre-deployment phase.
Data analysis/correction scripts and version	☑	Averaging and processing of data, calculation of performance metrics, and generation of figures and other supplementary material for analysis were obtained using Python 3.9.7 with the packages sensortoolkit v0.8.3b2, pandas 1.3.5, NumPy 1.21.2, Matplotlib 3.5.0, statsmodels 0.13.0, and seaborn 0.11.2. All packages are available from the Python Package Index (PyPI) at https://pypi.org/. The integrated development environment (IDE) Spyder 5.1.5 was used for scripting and data visualization. Version control for the Python base, packages, and IDE were all managed by conda 4.11.0.
Air Monitoring Station QAPP	☐	U.S. EPA Office Of Research and Development QAPP available upon request
Summary of FRM/FEM monitor QC checks	☑	See AZ-MAX-Page 7 of this testing report
Manufacturer website for FRM/FEM monitor	☑	Thermo Fisher Scientific: TEOM 1405 Product website
FRM/FEM monitor manual	☑	Thermo Fisher Scientific: TEOM 1405 Product Manual
FRM/FEM monitor specifications sheet(s)	☑	Thermo Fisher Scientific: TEOM 1405 Specification Sheet
Other documents	☐	

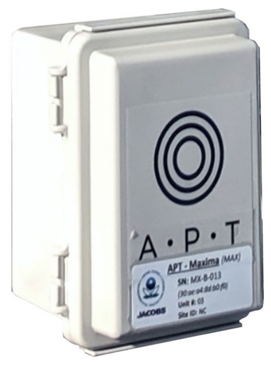

Supplemental Information: Photos of Testing Site and Equipment Setup

Site Description:

The West Phoenix Monitoring Station has been operational since 1984. The spatial scale for the West Phoenix site is Neighborhood. It is located in an area of stable, high-density residential properties. This State or Local Air Monitoring Stations (SLAMS) location monitors for CO, NO_2, O_3, PM_{10}, and $PM_{2.5}$. In addition, this is a quality assurance (QA) collocation site for $PM_{2.5}$ where the Maricopa County Air Quality Department (MCAQD) operates one filter-based $PM_{2.5}$ FRM sampler along with one continuous $PM_{2.5}$ FEM analyzer as per 40 CFR Part 58 Appendix A. Resources detailing air quality monitoring QA programs and procedures are detailed on EPA's Ambient Monitoring Technology Information Center website (https://www.epa.gov/amtic/ambient-air-monitoring-quality-assurance, *last accessed 5/11/2022*). Meteorological monitors operating at this site measure ambient temperature (T), barometric pressure, delta T (temperature inversion), and wind speed/direction.

Figure 1: APT Maxima sensor (indicated by red arrow) attached to metal railing atop the sampling shelter at the monitoring site.

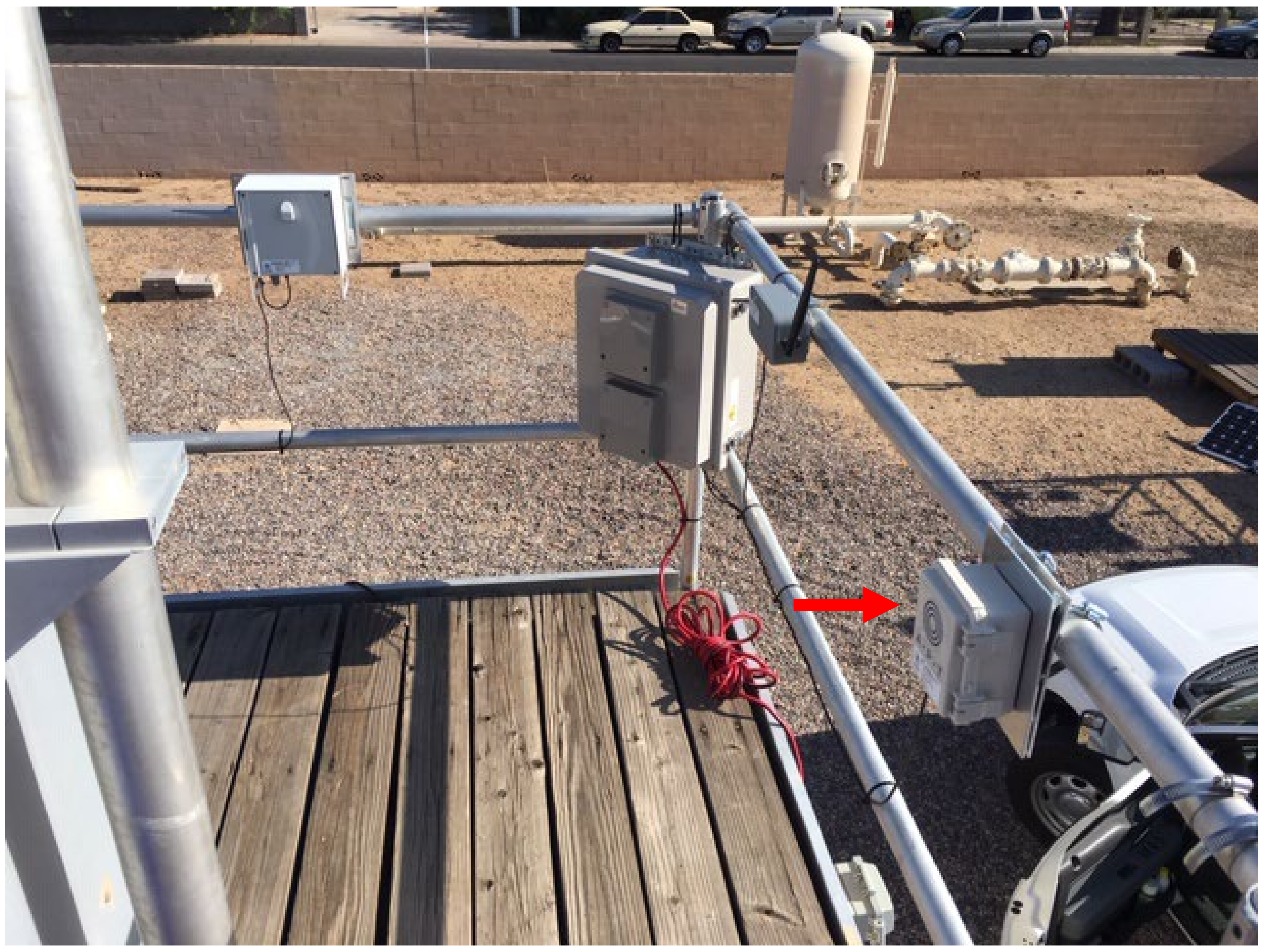

Figure 2: West Phoenix Monitoring Station

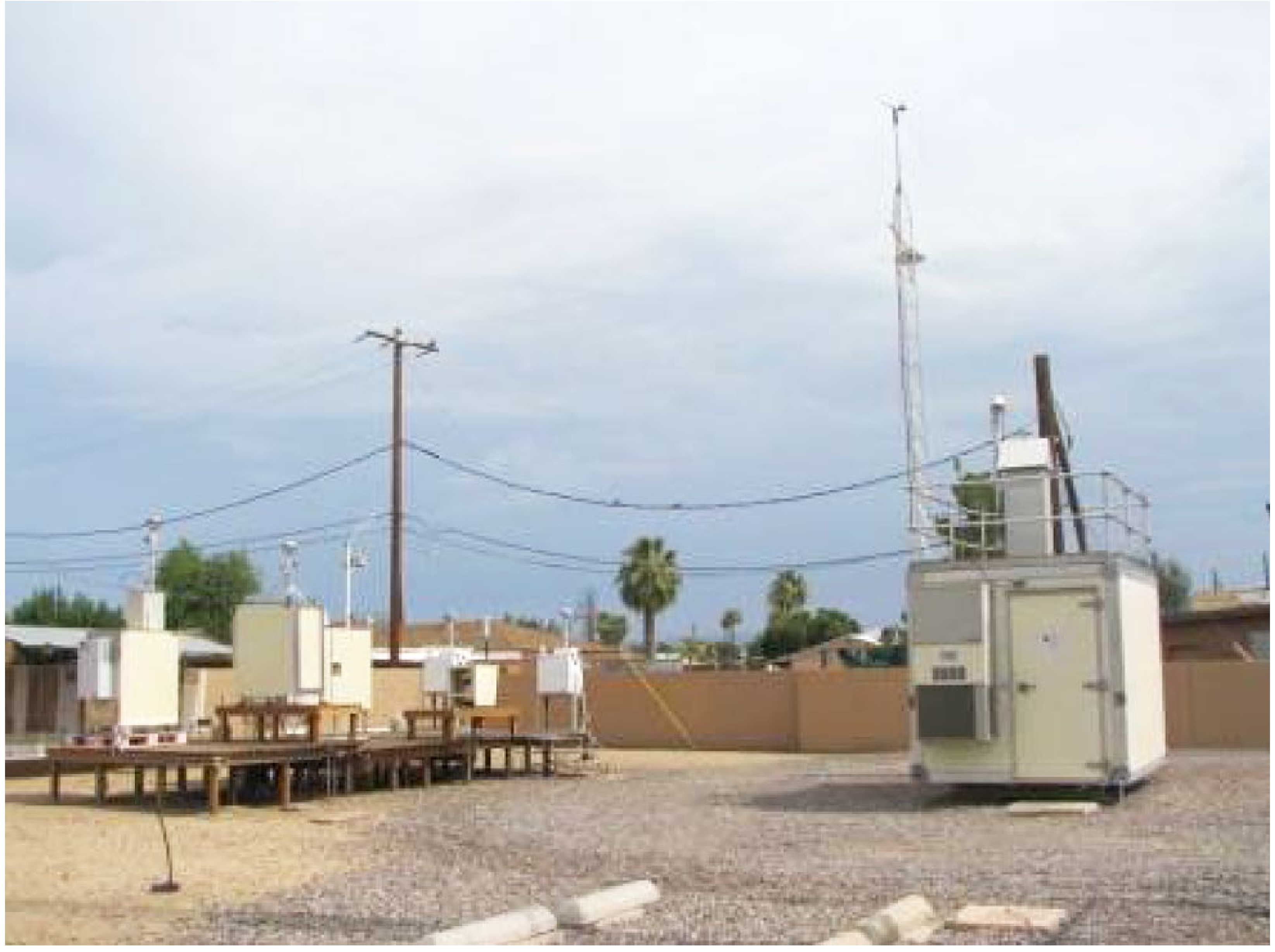

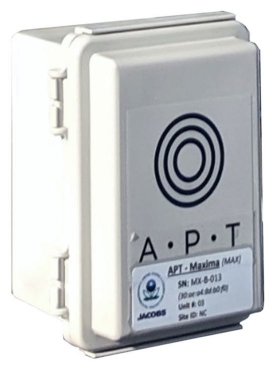

Supplemental Information: Data Storage, Correction Approach, and Issues Encountered

Data Storage and Transmission Method

The APT Maxima records data at 30 second intervals and stores the data on an internal microSD card and transmits data to the APT cloud server (last accessed 5/11/22) via Wi-Fi connection. The user interface for the Maxima sensors was under development during sensor testing, thus weekly raw data files were obtained from the internal MicroSD card. Each field site operator was provided two labeled MicroSD cards for the Maxima that they used to swap out each week. Data from the collected card was then read and processed off-site.

Data Correction Approach

This evaluation report reflects “out-of-the-box” performance of the APT Maxima. The Maxima data sets include two data columns contain $PM_{2.5}$ concentration measurements, “PM2.5(ug/m3)” and “PM2.5_Atm(ug/m3)”. These columns correspond to separate correction factors provided by the onboard Original Equipment Manufacturer (OEM) PM sensor (Plantower PMSA003). For this report, $PM_{2.5}$ measurements for the column labeled “PM2.5(ug/m3)” were selected for analysis.

After acquisition, the raw data was processed using the *sensortoolkit* python code library (v0.8.3b2). A continuous data set at the recorded sampling frequency was written to a .csv file. 1-hour and 24-hour averaged data sets were generated using a 75% completeness threshold and saved as separate .csv files. Outliers were NOT removed from data sets in order to assess “out-of-the-box” sensor performance.

Issues Encountered

Pre-deployment observations

- *Sampling internal abnormality:* Maxima devices did not continuously sample at 30-second intervals as stated in the sensor user guide. Instead, Maxima units occasionally sampled at 31 seconds. This increased sample interval is due to the accumulation of processing time over a certain number of samples. Each 30-second sample is a collection of exactly 30 seconds of sampling followed by a brief fraction of a second used for processing the collected data. Once processed, the next 30-second data collection period begins. When the sum of the processing time reaches one full second, the sample interval then jumps to 31 seconds. Data from this pre-deployment testing indicated that the processing speed for each sensor varies such that 31-second sampling duration occur at random intervals across Maxima units.

Field observations and sensor data flags

The APT Maxima was deployed at the West Phoenix monitoring site on 8/12/2019. The Maxima unit operated normally during the testing period and did not require replacement or repair.

During the testing period, no data flags logged by field technicians were recorded.

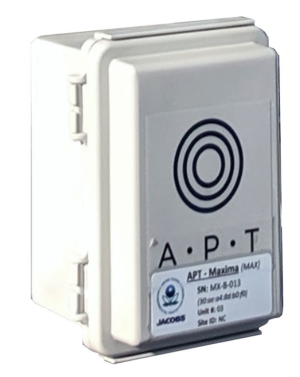

Description of Data Flags

AQS

The U.S. EPA's Air Quality System (AQS) is the Agency's primary ambient air monitoring data archive. A comprehensive list of data flags that are recorded alongside AQS data sets, referred to by U.S. EPA as 'qualifiers', can be found at the following link: https://aqs.epa.gov/aqsweb/documents/codetables/qualifiers.html

Invalidation of reference data: AQS qualifiers are organized by qualifier type, which indicates whether data logged alongside qualifier flags should be invalidated (set null). Qualifiers with type "Null Data Qualifier" are invalidated, and includes data logged during periods that coincide with QC checks (e.g., "BF-Precision/Zero/Span", "BJ- Operator Error", "BL - QA Audit", "AZ - QC Audit") among other events such as power outages. Data logged alongside qualifiers with type "Quality Assurance Qualifiers" are not invalidated and are included in this analysis (e.g., concentrations less than the federal MDL for the reference monitor "MD – Value less than MDL", QA reviewed values "Validated Value").

Data Flags Recorded During Testing

FRM/FEM Monitor	Timestamp (UTC)	Flag
Thermo Fisher 1405-DF TEOM FDMS Dichotomous FEM (Data acquired via AQS)	2019-07-31 17:00:00+0000 to 2019-07-31 18:00:00+0000	AY - QC Control Points (zero/span)
	2019-08-06 19:00:00+0000 to 2019-08-06 20:00:00+0000	BM - Accuracy check
	2019-08-14 17:00:00+0000 to 2019-08-14 18:00:00+0000	AY - QC Control Points (zero/span)
	2019-08-20 16:00:00+0000 to 2019-08-20 17:00:00+0000	BM - Accuracy check

Testing Report - $PM_{2.5}$ Base Testing

Clarity Node

This report reflects out-of-the-box performance

Initial Base Testing - Phoenix, AZ
U.S. Environmental Protection Agency
Office of Research and Development
PI: Clements.Andrea@epa.gov
919-541-1363
August 2019—September 2019

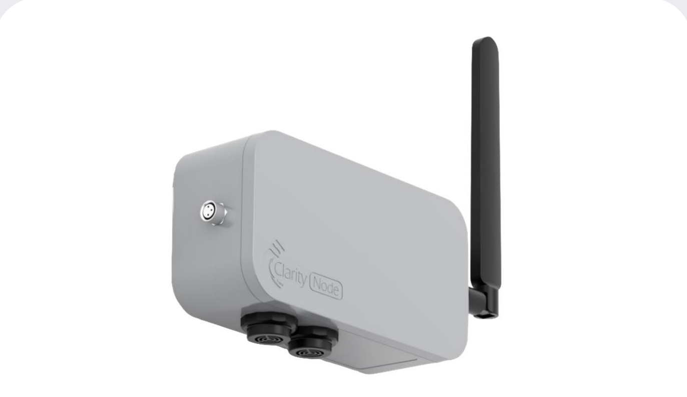

Image Attribution: Clarity Movement Co. (Berkeley, CA)

Deployment Details

Testing Organization and Site Information	
Testing organization (Name, Organization type, Contact website)	U.S. Environmental Protection Agency - Office of Research and Development<br>Federal Government<br>Air Sensor Toolbox | U.S. EPA Website
Testing location (City, State, Latitude and Longitude)	West Phoenix<br>Phoenix, AZ<br>33.48385, -112.14257
AQS site ID	04 - 013 - 0019
Sampling timeframe (MM-DD-YY)	08-16-19 to 09-15-19
Sensor data source	Clarity Dashboard download
Reference data source	AQS API download

Sensor Information		
Manufacturer, model	Clarity Node	
Device firmware version	Received by EPA May 2019	
Sampling time interval	3-minutes	
Sensor serial numbers	CNO_01	
Issues encountered during deployment?	☑	See AZ-CNO-Page 6 of this testing report

FRM/FEM Information	
Manufacturer, model, designation	Thermo Scientific TEOM 1405-DF Dichot. with FDMS FEM
Sampling time interval	1-hour averaging
Date of calibration	As required by 40 CFR Part 58 and the Air Monitoring Network Plan maintained by MCAQD
Date of flowrate verification check	Monthly as required by 40 CFR Part 58 Appendix A
Description, date(s) of maintenance activities	N/A

Time Series Plots

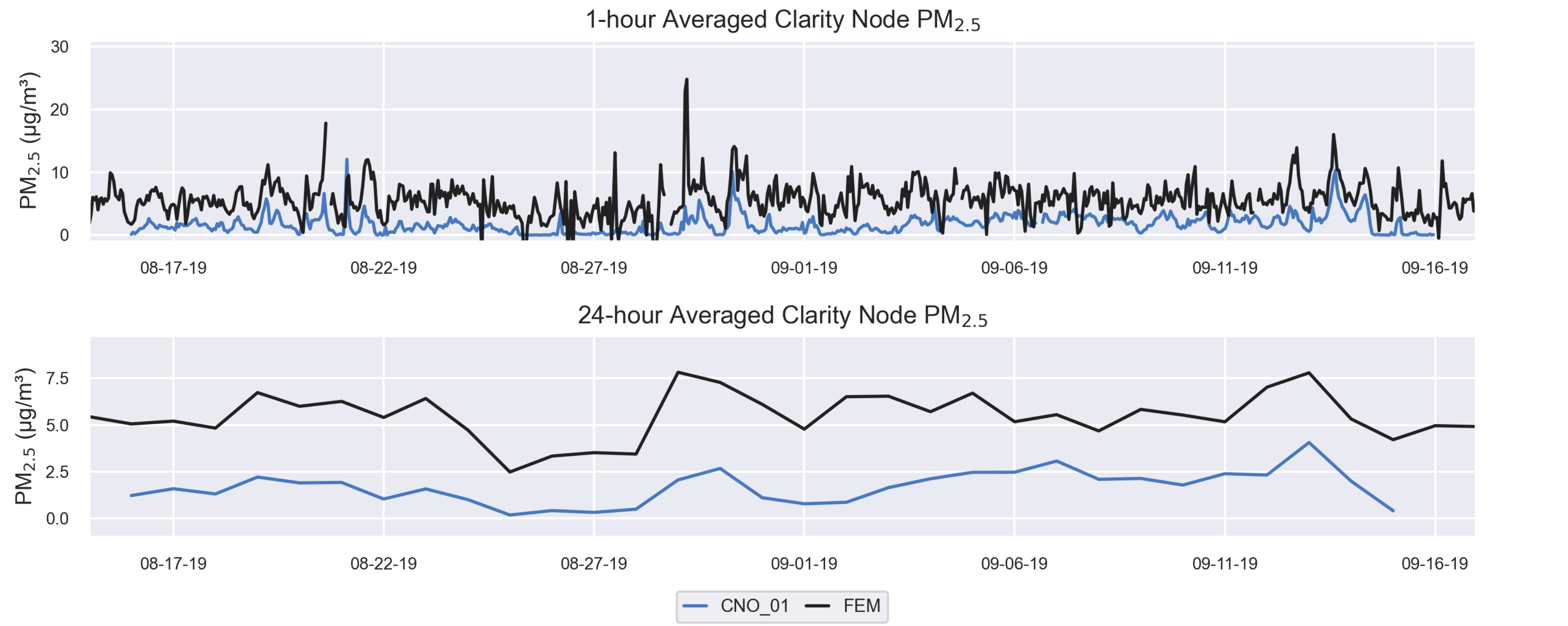

Scatter Plots: Comparison to FRM/FEM

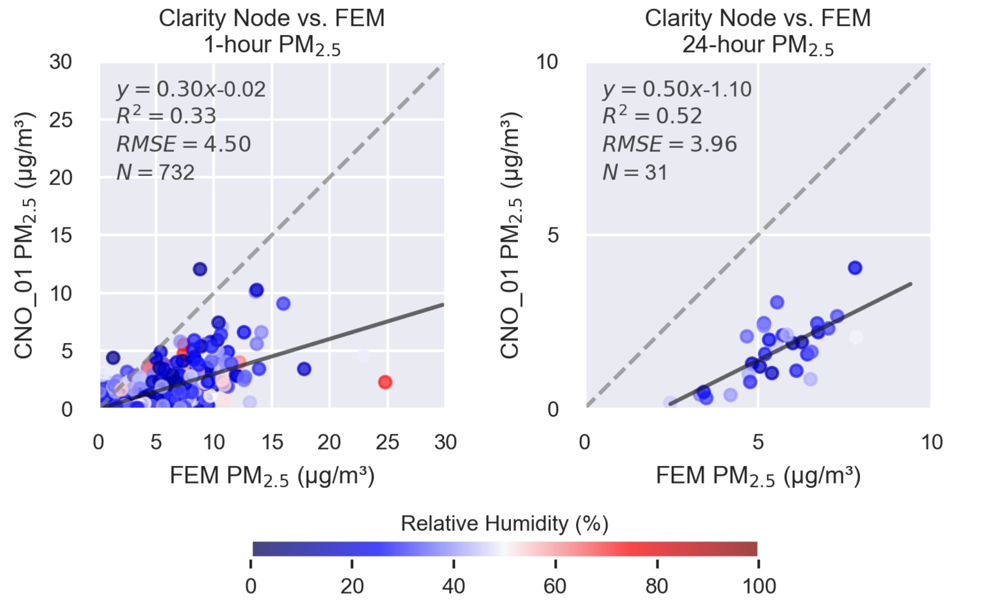

Range of FRM/FEM concentrations over duration of base test (µg/m³)	-3.8-24.8 (1-hr), 2.5-7.8 (24-hr)
Number of 24-hr periods in FRM/FEM monitor measurements with a goal concentration ≥ 25 µg/m³	0

Performance Metrics

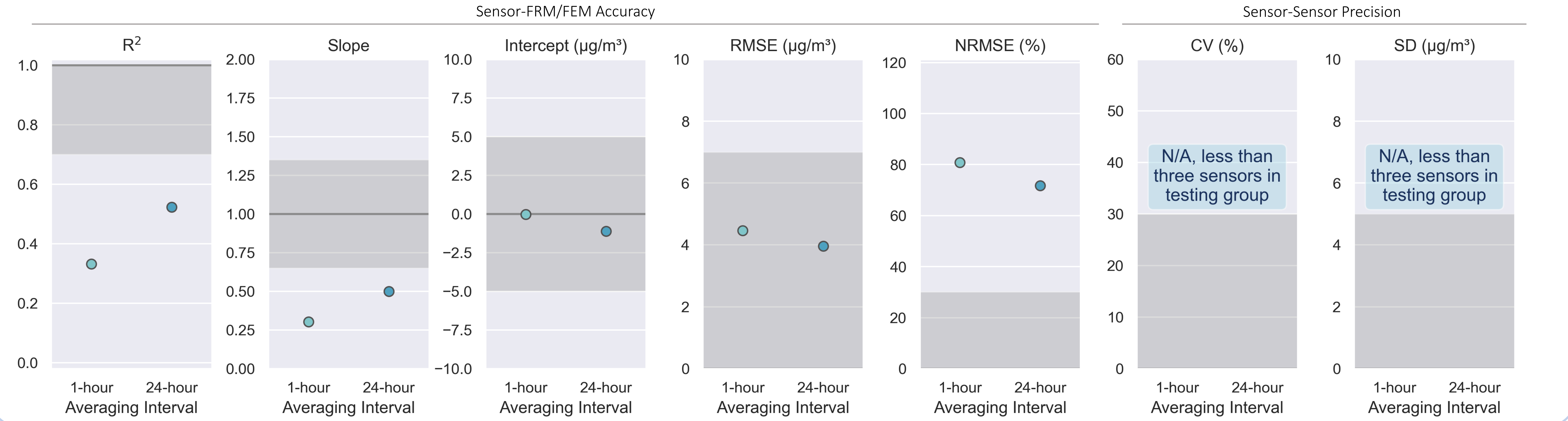

Meteorological Conditions During Deployment

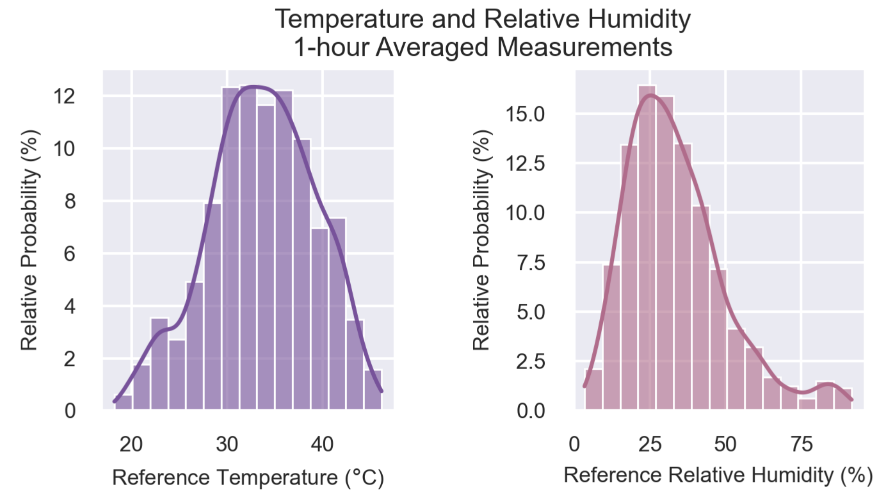

Number of 24-hr periods outside sensor manufacture-listed temperature operational range (-10 to 55 °C)	0
Number of 24-hr periods outside sensor manufacture-listed relative humidity operational range (10 to 98%)	0

Meteorological Influence

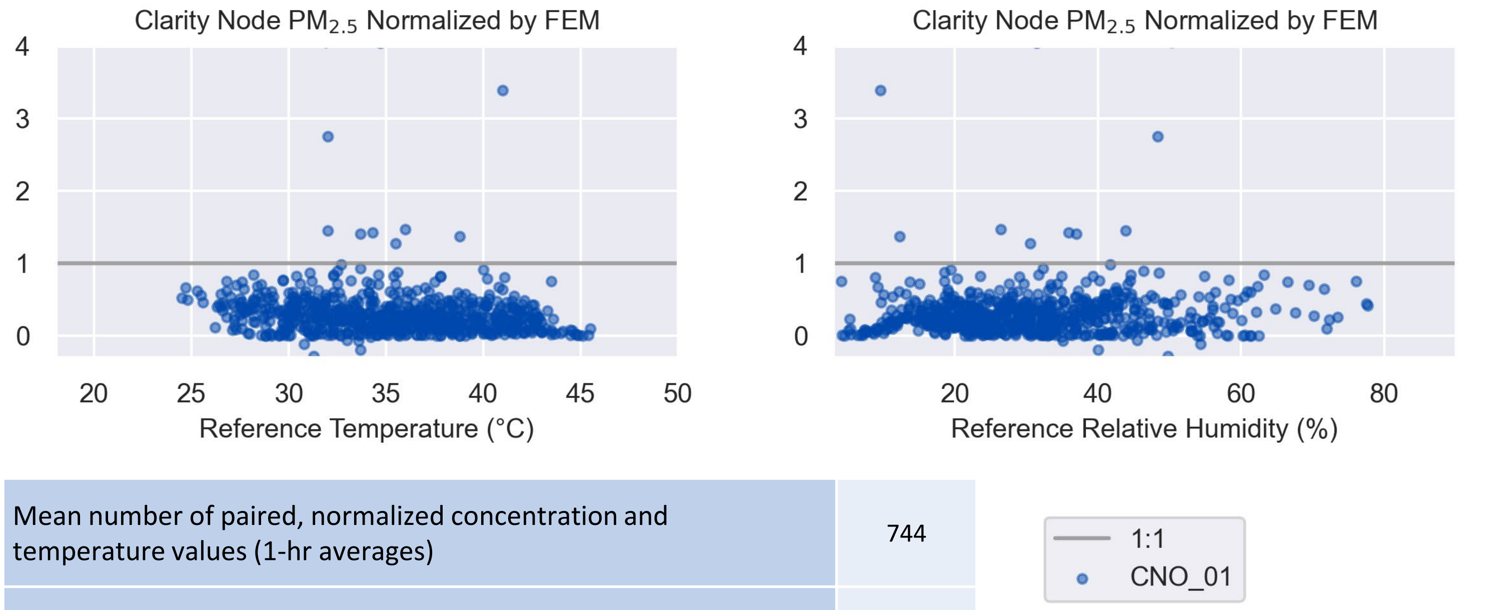

Mean number of paired, normalized concentration and temperature values (1-hr averages)	744
Mean number of paired, normalized concentration and relative humidity values (1-hr averages)	744

Testing Report - $PM_{2.5}$ Base Testing

Clarity Node

This report reflects out-of-the-box performance

Initial Base Testing - Phoenix, AZ
U.S. Environmental Protection Agency
Office of Research and Development
PI: Clements.Andrea@epa.gov
919-541-1363
August 2019—September 2019

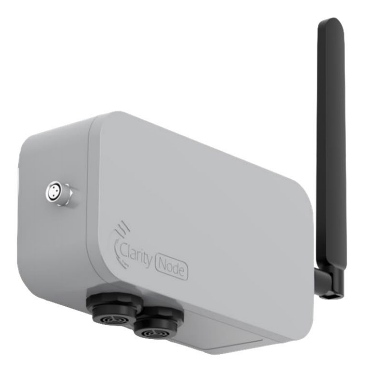

Image Attribution: Clarity Movement Co. (Berkeley, CA)

Tabular Statistics

Sensor-FRM/FEM Correlation

	Bias and Linearity						Data Quality			
	R^2		Slope		Intercept (µg/m³)		Uptime (%)		Number of paired sensor and FRM/FEM concentration values	
	1-Hour ○	24-Hour ○	1-Hour ○	24-Hour ○	1-Hour ●	24-Hour ●	1-Hour ●	24-Hour ●	1-Hour	24-Hour
Metric Target Range	≥ 0.70	≥ 0.70	1.0 ± 0.35	1.0 ± 0.35	-5 ≤ b ≤ 5	-5 ≤ b ≤ 5	75%*	75%*	-	-
Sensor CNO_01	0.33	0.52	0.30	0.50	-0.02	-1.10	100	100	732	31

	Error			
	RMSE (µg/m³)		NRMSE (%)	
	1-Hour ★	24-Hour ★	1-Hour ☆	24-Hour ☆
Metric Target Range	≤ 7.0	≤ 7.0	≤ 30.0	≤ 30.0
Deployment Value	4.5	4.0	80.8	71.8

Device-specific metrics (computed for each sensor in evaluation)
○○○ Metric value for none of devices tested falls within the target range
●○○ Metric value for one of devices tested falls within the target range
●●○ Metric value for two of devices tested falls within the target range
●●● Metric value for three of devices tested falls within the target range

Single-valued metrics (computed via entire evaluation dataset)
☆ Indicates that the metric value is not within the target range
★ Indicates that the metric value is within the target range

Sensor-Sensor Precision[1]

	Precision (between collocated sensors)				Data Quality	
	CV (%)		SD (µg/m³)		Number of concurrent sensor concentration pairs	
	1-Hour ☆	24-Hour ☆	1-Hour ☆	24-Hour ☆	1-Hour	24-Hour
Metric Target Range	≤ 30.0	≤ 30.0	≤ 5.0	≤ 5.0	-	-
Deployment Value	-	-	-	-	-	-

[1]Precision statistics are computed for evaluations with at least three collocated sensor units. Metric values are left blank for evaluations with two or fewer sensor units.

*This value is only a recommendation for ensuring data quality and is not included in the list of target values discussed in Section 4 of the Performance Testing Protocols, Metrics, and Target Values for Fine Particulate Matter Air Sensors document.

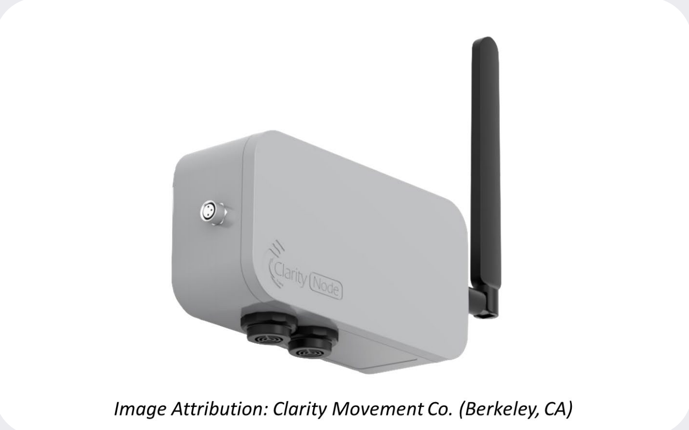

Sensor-FRM/FEM Scatter Plots

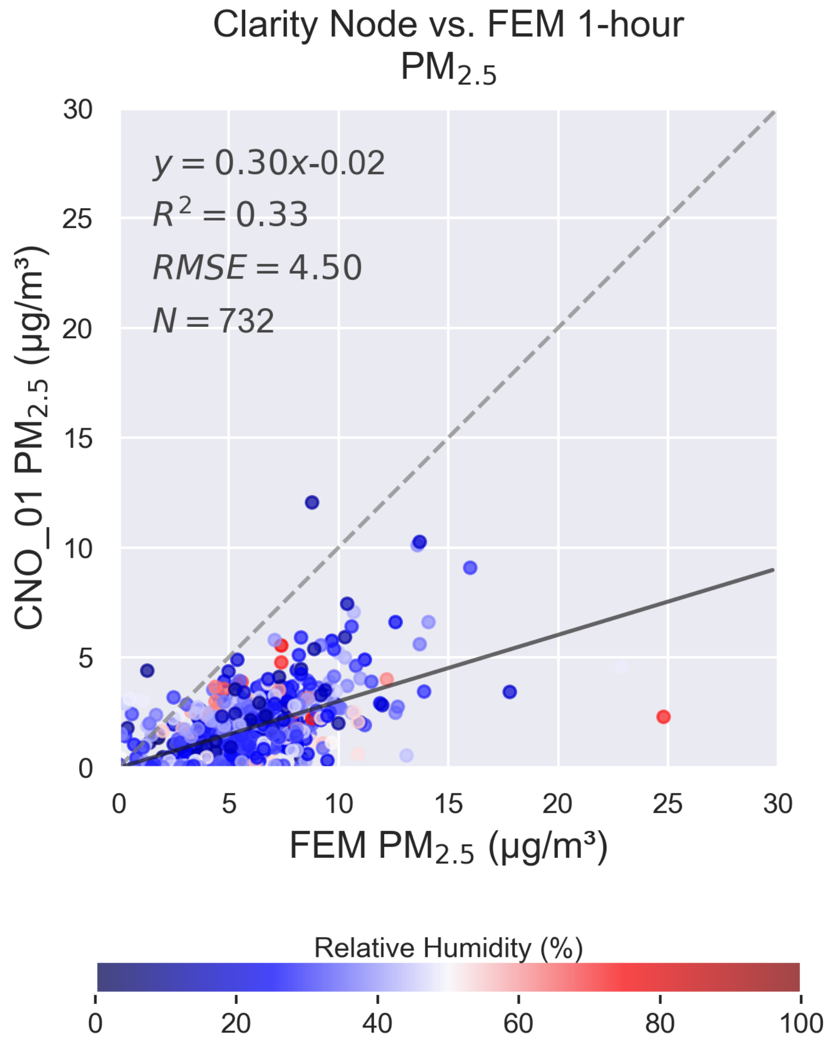

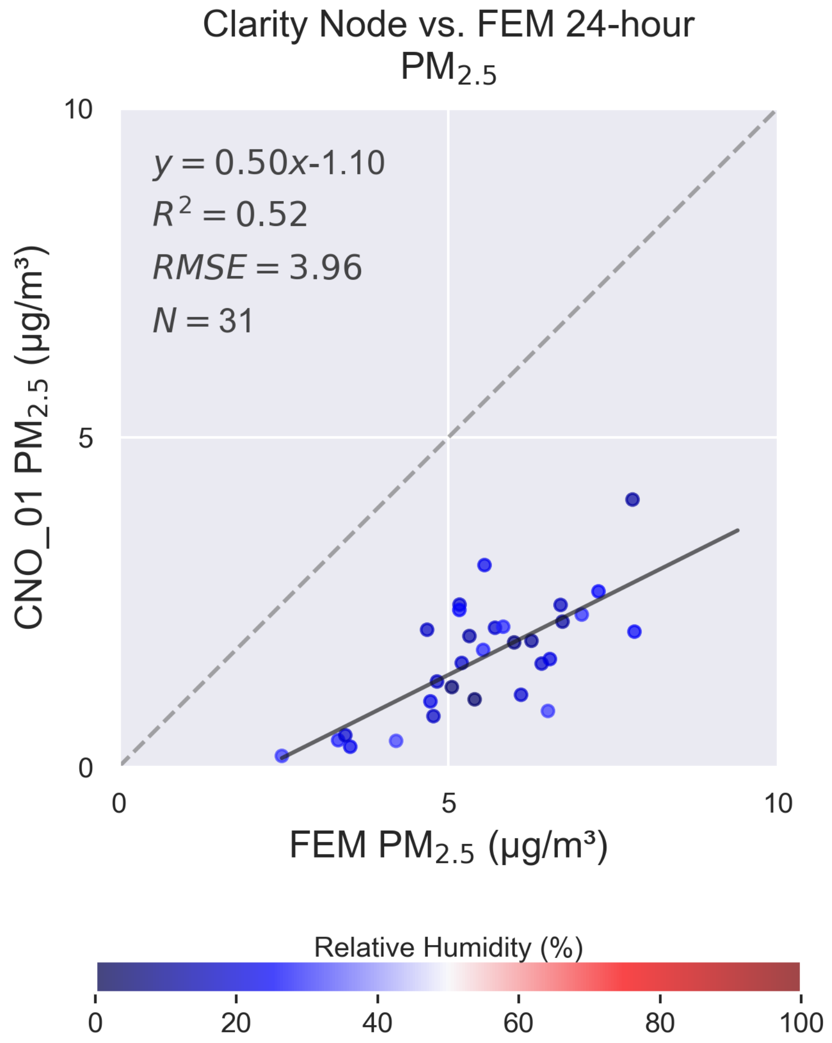

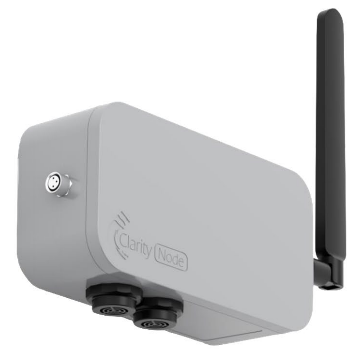

Image Attribution: Clarity Movement Co. (Berkeley, CA)

Supplemental Information

Abbreviations used in Supplemental Information

FRM	Federal Reference Method
FEM	Federal Equivalent Method
SOP	Standard Operating Procedure
QAPP	Quality Assurance Project Plan
QC	Quality Control

Supplemental Documentation	Attached	Description & URL or file path to documentation
Field observations and sensor data flags	☑	See AZ-CNO-Page 6 of this testing report
Maintenance logs	☐	No logs recorded during testing
Standard operating procedure(s)	☐	U.S. EPA Office Of Research and Development SOP available upon request
Photos of equipment setup and testing	☑	See AZ-CNO-Page 5 of this testing report
Product specifications sheet(s)	☐	N/A
Product manual(s)	☐	N/A
Data storage and transmission method	☑	See AZ-CNO-Page 6 of this testing report
Data correction approach	☑	See AZ-CNO-Page 6 of this testing report
Issues encountered	☑	No issues reported during testing. See AZ-CNO-Page 6 of this testing report for additional notes regarding issues encountered prior to the testing period.
Data analysis/correction scripts and version	☑	Averaging and processing of data, calculation of performance metrics, and generation of figures and other supplementary material for analysis were obtained using Python 3.9.7 with the packages sensortoolkit v0.8.3b2, pandas 1.3.5, NumPy 1.21.2, Matplotlib 3.5.0, statsmodels 0.13.0, and seaborn 0.11.2. All packages are available from the Python Package Index (PyPI) at https://pypi.org/. The integrated development environment (IDE) Spyder 5.1.5 was used for scripting and data visualization. Version control for the Python base, packages, and IDE were all managed by conda 4.11.0.
Air Monitoring Station QAPP	☐	U.S. EPA Office Of Research and Development QAPP available upon request
Summary of FRM/FEM monitor QC checks	☑	See AZ-CNO-Page 7 of this testing report
Manufacturer website for FRM/FEM monitor	☑	Thermo Fisher Scientific: TEOM 1405 Product website
FRM/FEM monitor manual	☑	Thermo Fisher Scientific: TEOM 1405 Product Manual
FRM/FEM monitor specifications sheet(s)	☑	Thermo Fisher Scientific: TEOM 1405 Specification Sheet
Other documents	☐	

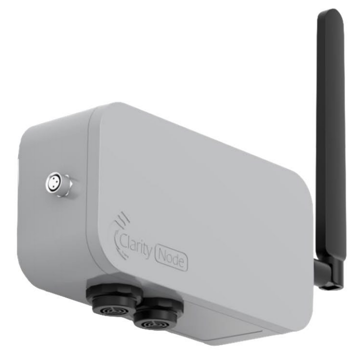

Image Attribution: Clarity Movement Co. (Berkeley, CA)

Supplemental Information: Photos of Testing Site and Equipment Setup

Site Description:

The West Phoenix Monitoring Station has been operational since 1984. The spatial scale for the West Phoenix site is Neighborhood. It is located in an area of stable, high-density residential properties. This State or Local Air Monitoring Stations (SLAMS) location monitors for CO, NO_2, O_3, PM_{10}, and $PM_{2.5}$. In addition, this is a quality assurance (QA) collocation site for $PM_{2.5}$ where the Maricopa County Air Quality Department (MCAQD) operates one filter-based $PM_{2.5}$ FRM sampler along with one continuous $PM_{2.5}$ FEM analyzer as per 40 CFR Part 58 Appendix A. Resources detailing air quality monitoring QA programs and procedures are detailed on EPA's Ambient Monitoring Technology Information Center website (https://www.epa.gov/amtic/ambient-air-monitoring-quality-assurance, *last accessed 5/11/2022*). Meteorological monitors operating at this site measure ambient temperature (T), barometric pressure, delta T (temperature inversion), and wind speed/direction.

Figure 1: Clarity Node sensor (indicated by red arrow) attached to metal railing atop the sampling shelter at the monitoring site.

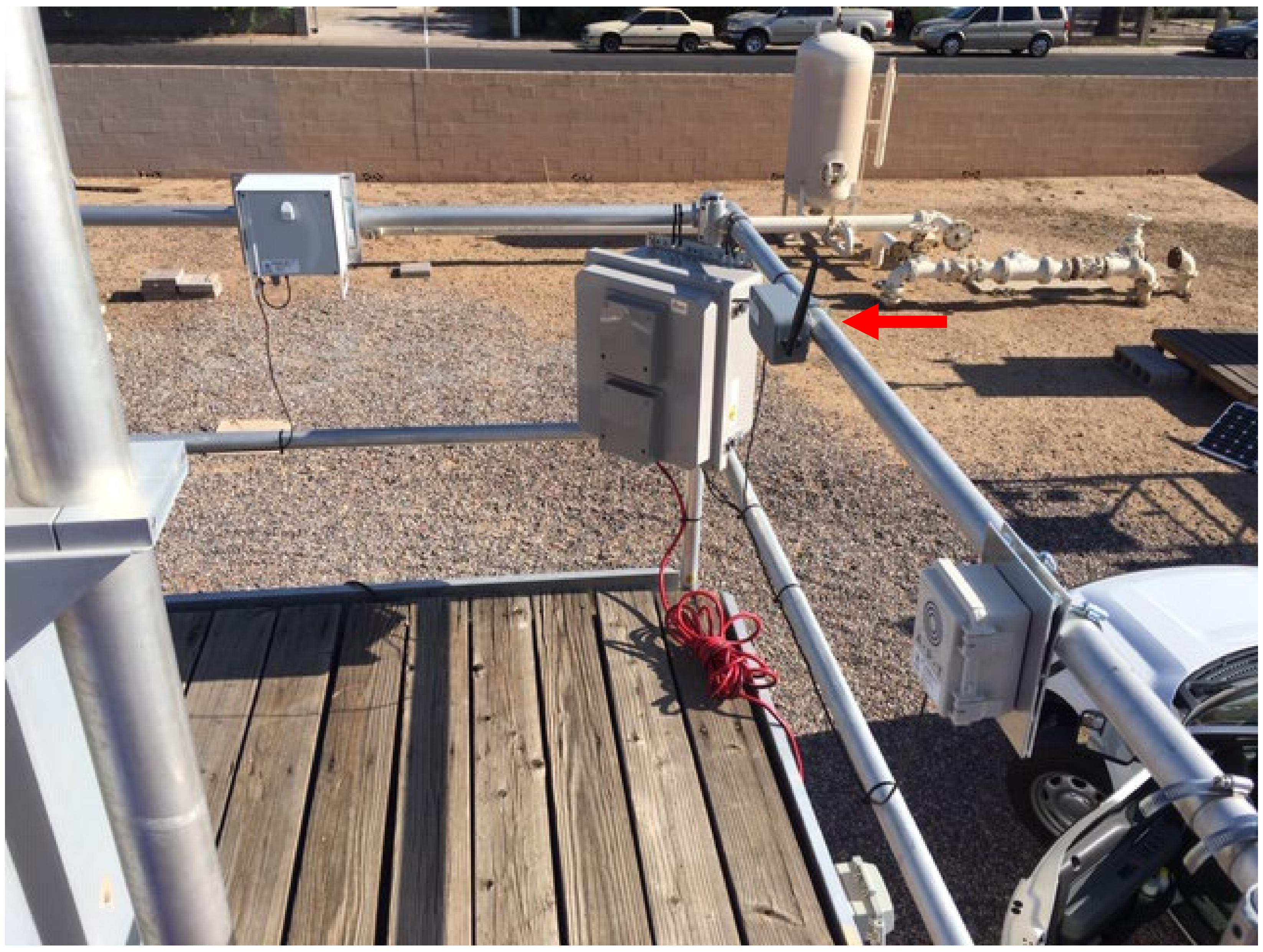

Figure 2: West Phoenix Monitoring Station

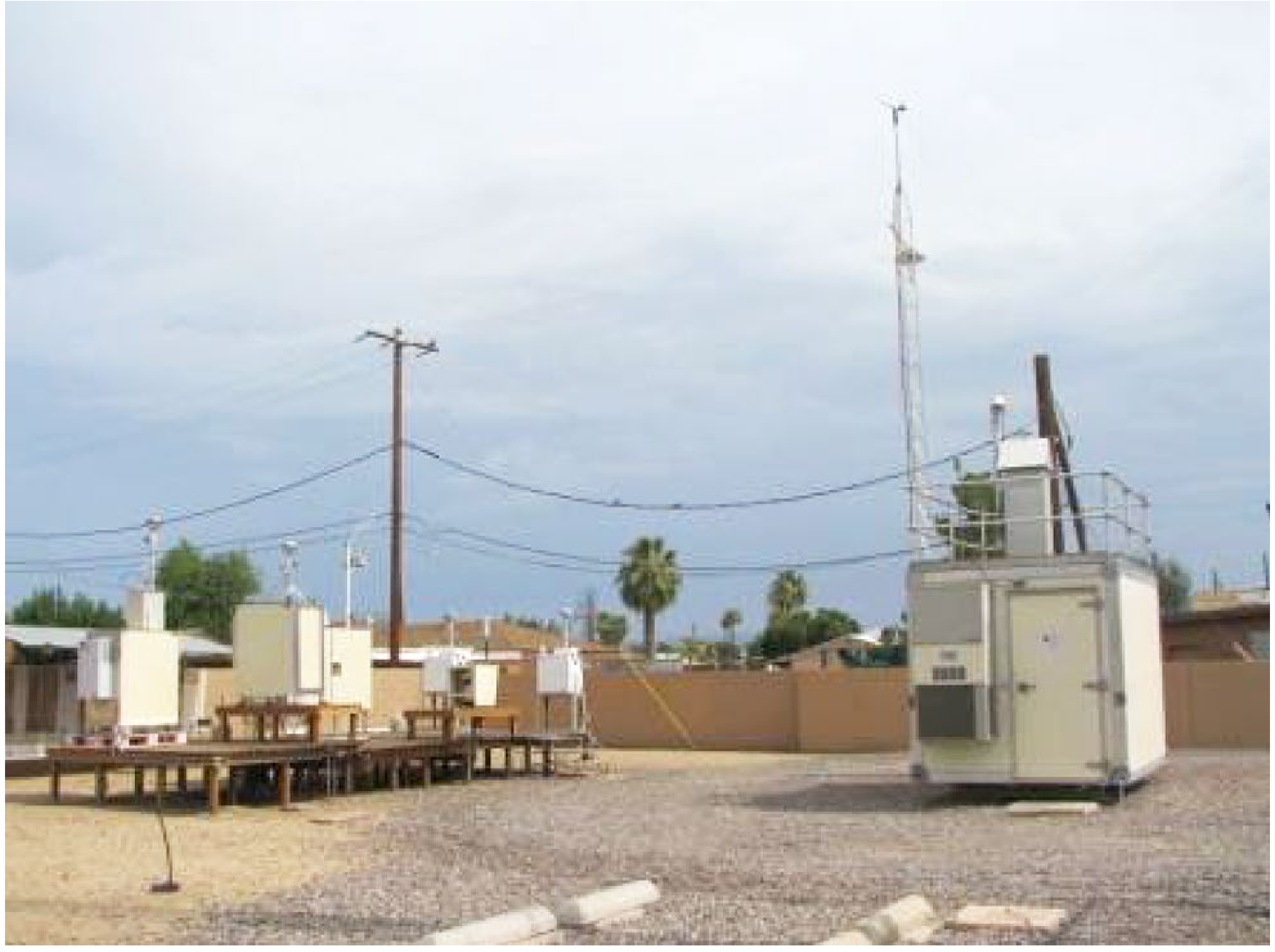

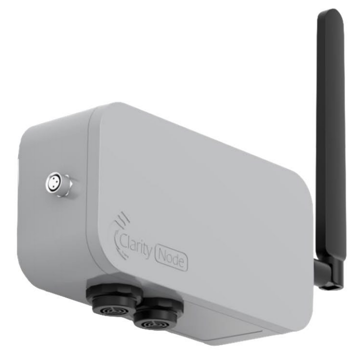

Image Attribution: Clarity Movement Co. (Berkeley, CA)

Data Storage and Transmission Method

The Clarity Node transmits data via cellular SIM card. Weekly raw data collections were performed over Wi-Fi using the Clarity dashboard (*last accessed 5/11/22*). The observed sampling interval fluctuated and is further discussed under pre-deployment observations below. On average, the Node models sampled every 2-3 minutes, and the Node-S sampled every 15-17 minutes.

Data Correction Approach

This evaluation report reflects "out-of-the-box" performance of the Clarity Node. The manufacturer provides a dashboard feature for calibrating sensor data against collocated reference monitors. Calibrated data were NOT used for this evaluation. Prospective consumers may get better performance from this device if they utilize this feature.

After acquisition, the raw data was processed using the *sensortoolkit* python code library (v0.8.3b2). A continuous data set at the recorded sampling frequency was written to a .csv file. 1-hour and 24-hour averaged data sets were generated using a 75% completeness threshold and saved as separate .csv files. Due to the variable sampling interval observed for Clarity Node units, 75% completeness was determined against the number of data points that would be recorded within 1-hour/24-hour periods if the sensor were to log measurements at the most commonly recorded sampling interval (i.e., the mode of recorded intervals). Outliers were NOT removed from data sets in order to assess "out-of-the-box" sensor performance.

Issues Encountered

Pre-deployment observations

- *Unsteady sampling interval:* Clarity Node sensors were received by EPA on May 28, 2019. A lab bench-top evaluation was performed from May 29, 2019 – May 31, 2019. During bench-top testing, it was observed that the sampling intervals noted by Clarity (5 minutes for non-solar Node, 15 minutes for the solar Node-S) were not reflective of recorded intervals. Communication with the vendor concluded that the sampling interval will fluctuate depending on the settings, battery charge, and strength of the cellular signal and that this sampling interval cannot be adjusted by the user at present. On average, the Node models sampled every 2-3 minutes, and the Node-S sampled every 15-17 minutes.
- *Connectivity Issues:* Prior to the testing period indicated in this report, the Clarity Node unit deployed at the West Phoenix site experienced initial startup connectivity issues when the sensor arrived at the monitoring site on August 12, 2019 through August 15, 2019 when the issue was resolved.

Field observations and sensor data flags

The following table contains data flags describing events that were encountered during the testing period. On 9/6/2019, a sampling interval abnormality flag was logged, associated with an occasional change in the sampling frequency of the Clarity Node. As noted during pre-deployment testing, the Node typically samples at 2 to 3-minute intervals. On 9/6/2019, this behavior changed slightly whereby the Node would occasionally record samples at 4-minute intervals, followed by a period of alternating 2 to 3-minute sampling intervals. This longer-duration sampling interval repeatedly occurred at roughly 30-minute to 1-hour intervals. This change in sampling frequency was not a result of unit configuration changes applied by a field technician and reflect the out-of-the-box dynamic sampling interval as programmed by the manufacturer.

Start Time (UTC)	End Time (UTC)	Sensor Serial ID	Parameters Impacted	Flag
2019-09-06 16:14:00+00:00	2019-09-20 14:47:00+00:00	CNO_01	ALL	6-Sampling interval abnormality

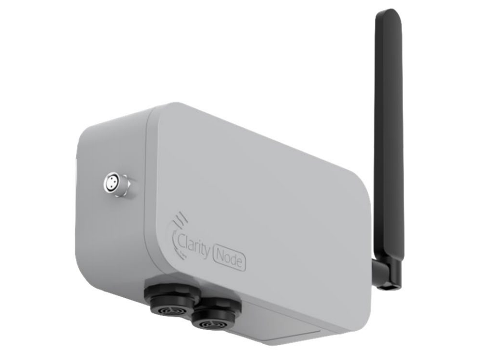

Image Attribution: Clarity Movement Co. (Berkeley, CA)

Description of Data Flags

AQS

The U.S. EPA's Air Quality System (AQS) is the Agency's primary ambient air monitoring data archive. A comprehensive list of data flags that are recorded alongside AQS data sets, referred to by U.S. EPA as 'qualifiers', can be found at the following link: https://aqs.epa.gov/aqsweb/documents/codetables/qualifiers.html

Invalidation of reference data: AQS qualifiers are organized by qualifier type, which indicates whether data logged alongside qualifier flags should be invalidated (set null). Qualifiers with type "Null Data Qualifier" are invalidated, and includes data logged during periods that coincide with QC checks (e.g., "BF-Precision/Zero/Span", "BJ- Operator Error", "BL - QA Audit", "AZ - QC Audit") among other events such as power outages. Data logged alongside qualifiers with type "Quality Assurance Qualifiers" are not invalidated and are included in this analysis (e.g., concentrations less than the federal MDL for the reference monitor "MD – Value less than MDL", QA reviewed values "Validated Value").

Data Flags Recorded During Testing

FRM/FEM Monitor	Timestamp (UTC)	Flag
Thermo Fisher 1405-DF TEOM FDMS Dichotomous FEM (Data acquired via AQS)	2019-08-20 16:00:00+0000 to 2019-08-20 17:00:00+0000	BM - Accuracy check
	2019-08-28 17:00:00+0000 to 2019-08-28 19:00:00+0000	AY - QC Control Points (zero/span)
	2019-09-04 16:00:00+0000 to 2019-09-04 17:00:00+0000	BM - Accuracy check
	2019-09-11 17:00:00+0000 to 2019-09-11 18:00:00+0000	AY - QC Control Points (zero/span)

Testing Report - $PM_{2.5}$ Base Testing

PurpleAir PA-II-SD

This report reflects out-of-the-box performance

Initial Base Testing - Phoenix, AZ

U.S. Environmental Protection Agency
Office of Research and Development
PI: Clements.Andrea@epa.gov
919-541-1363
August 2019

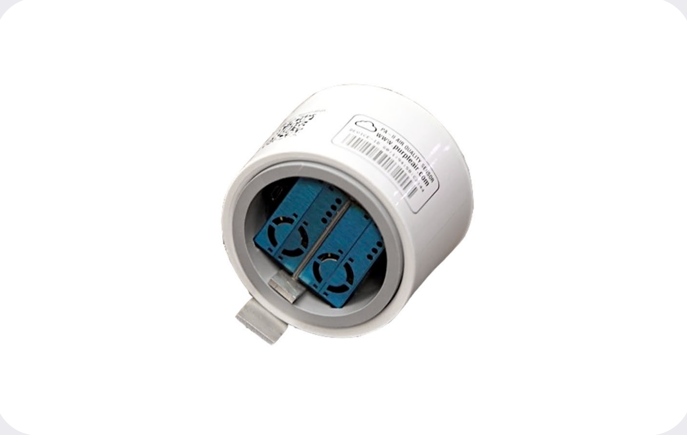

Deployment Details

Testing Organization and Site Information	
Testing organization (Name, Organization type, Contact website)	U.S. Environmental Protection Agency - Office of Research and Development<br>Federal Government<br>Air Sensor Toolbox | U.S. EPA Website
Testing location (City, State, Latitude and Longitude)	West Phoenix<br>Phoenix, AZ<br>33.48385, -112.14257
AQS site ID	04 - 013 - 0019
Sampling timeframe (MM-DD-YY)	08-01-19 to 08-31-19
Sensor data source	Onboard MicroSD card
Reference data source	AQS API download

Sensor Information			
Manufacturer, model	PurpleAir PA-II-SD		
Device firmware version	V4.02		
Sampling time interval	2-minutes		
Sensor serial numbers	PA01		
Issues encountered during deployment?	☐	No Issues	

FRM/FEM Information	
Manufacturer, model, designation	Thermo Scientific TEOM 1405-DF Dichot. with FDMS FEM
Sampling time interval	1-hour averaging
Date of calibration	As required by 40 CFR Part 58 and the Air Monitoring Network Plan maintained by MCAQD
Date of flowrate verification check	Monthly as required by 40 CFR Part 58 Appendix A
Description, date(s) of maintenance activities	N/A

Time Series Plots

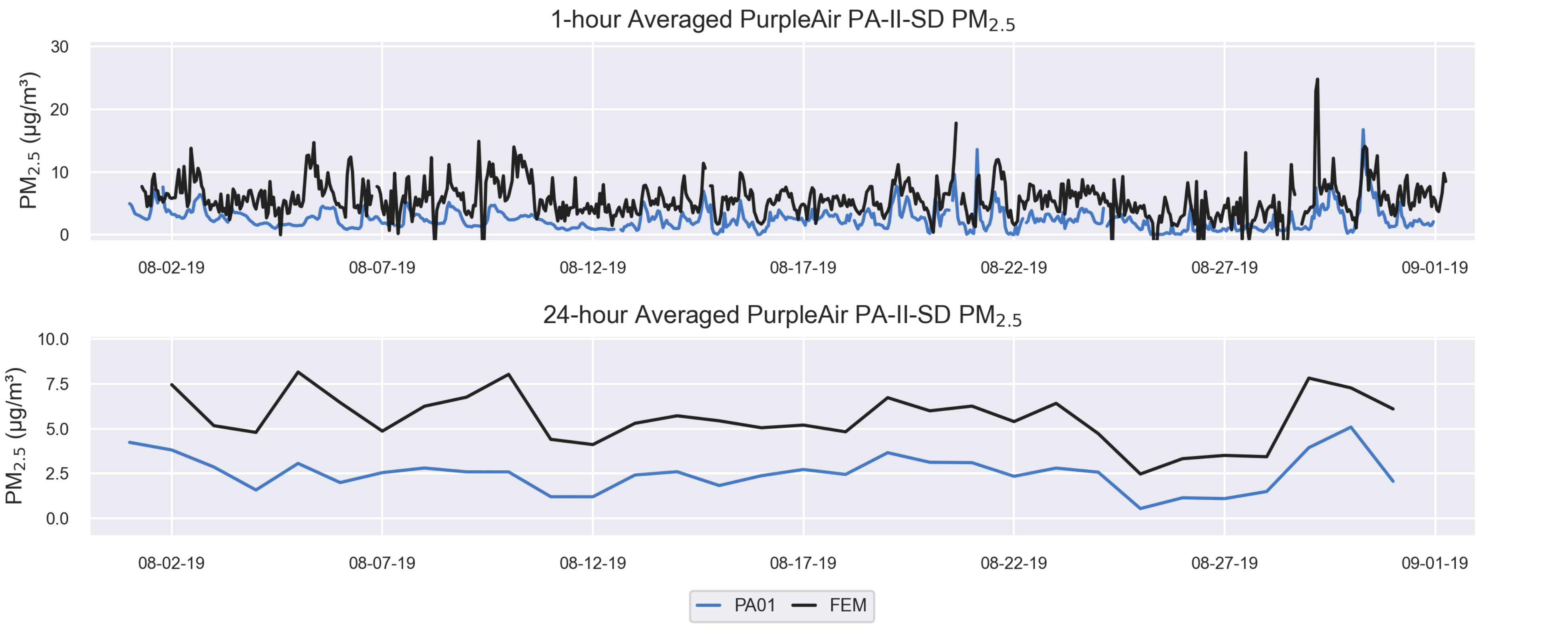

Scatter Plots: Comparison to FRM/FEM

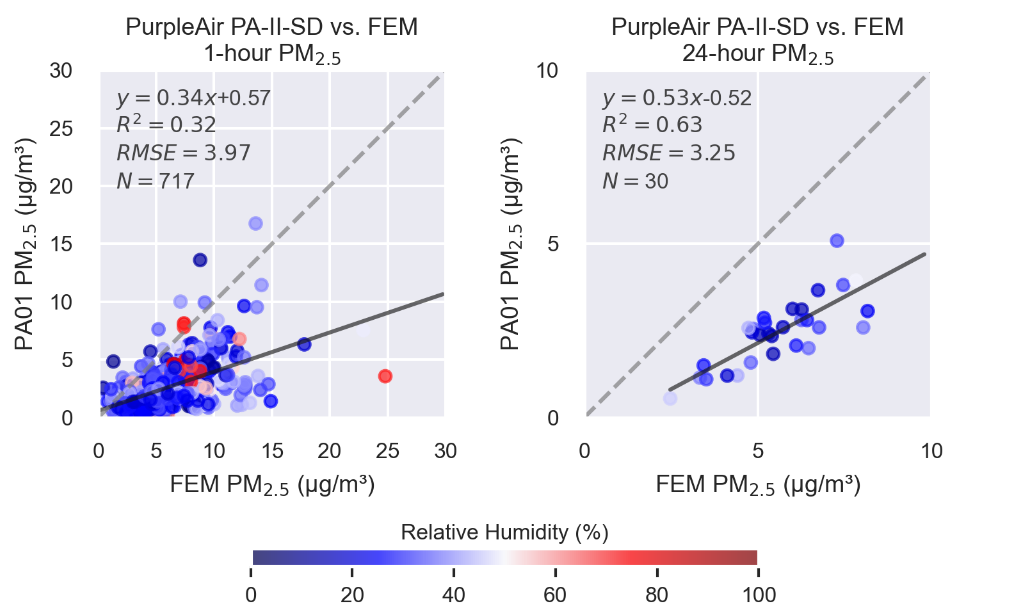

Range of FRM/FEM concentrations over duration of base test (μg/m³)	-3.8-24.8 (1-hr), 2.5-8.2 (24-hr)
Number of 24-hr periods in FRM/FEM monitor measurements with a goal concentration ≥ 25 μg/m³	0

Performance Metrics

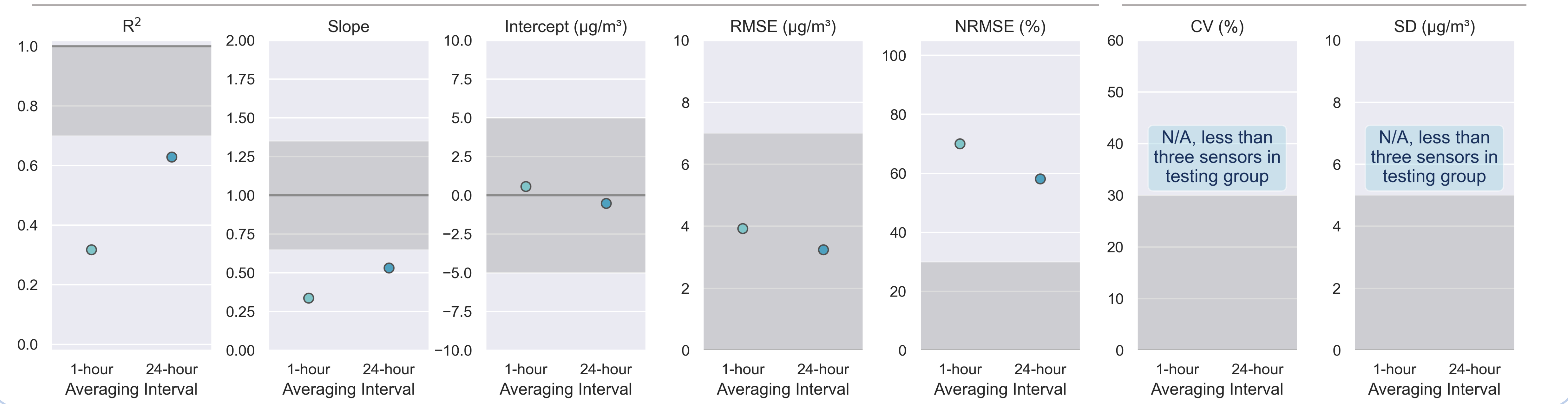

Meteorological Conditions During Deployment

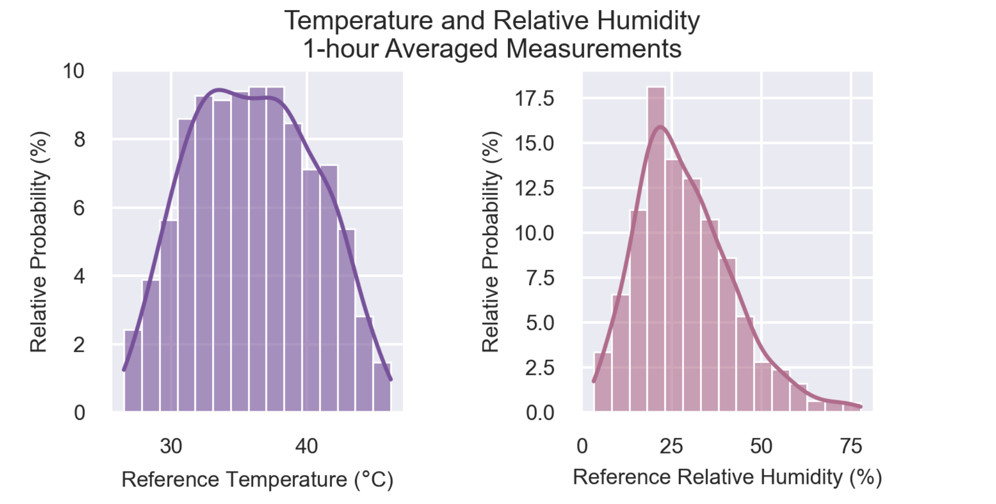

Number of 24-hr periods outside sensor manufacturer-listed temperature operational range (no operational range specified)	-
Number of 24-hr periods outside sensor manufacturer-listed relative humidity operational range (no operational range specified)	-

Meteorological Influence

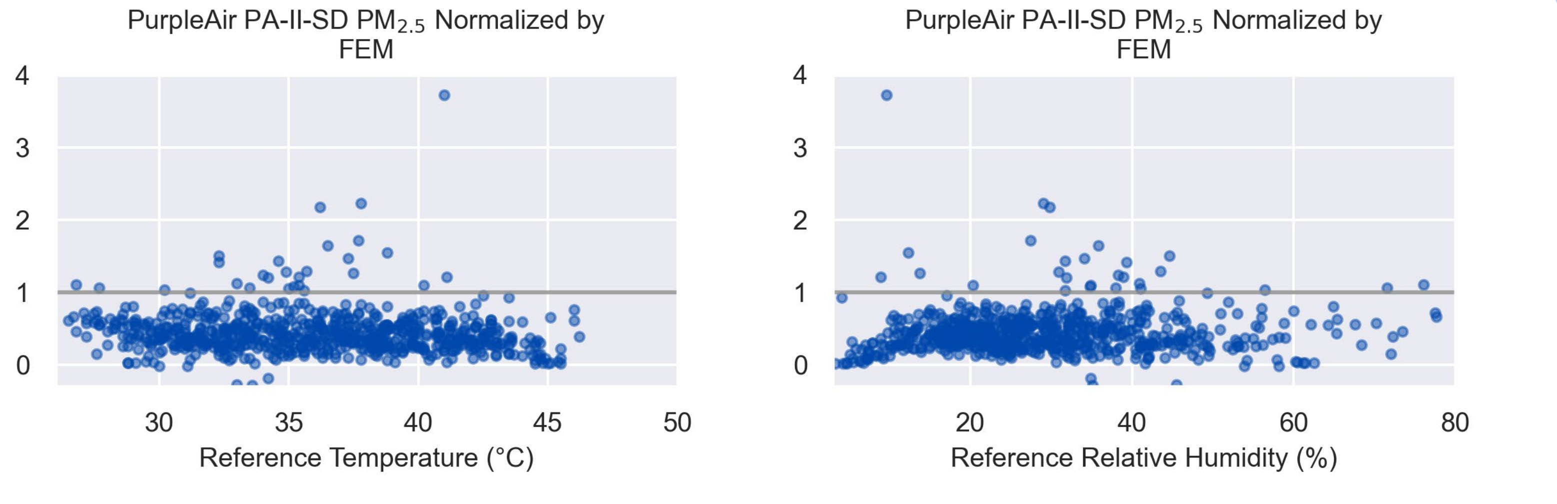

Mean number of paired, normalized concentration and temperature values (1-hr averages)	744
Mean number of paired, normalized concentration and relative humidity values (1-hr averages)	744

Testing Report - $PM_{2.5}$ Base Testing

PurpleAir PA-II-SD

This report reflects out-of-the-box performance

Initial Base Testing - Phoenix, AZ

U.S. Environmental Protection Agency
Office of Research and Development
PI: Clements.Andrea@epa.gov
919-541-1363
August 2019

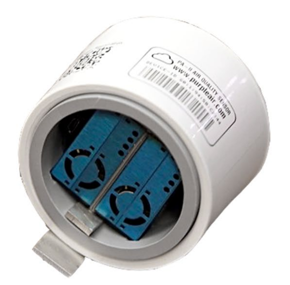

Tabular Statistics

Sensor-FRM/FEM Correlation

	Bias and Linearity						Data Quality			
	R^2		Slope		Intercept (µg/m³)		Uptime (%)		Number of paired sensor and FRM/FEM concentration values	
	1-Hour ○	24-Hour ○	1-Hour ○	24-Hour ○	1-Hour ●	24-Hour ●	1-Hour ●	24-Hour ●	1-Hour	24-Hour
Metric Target Range	≥ 0.70	≥ 0.70	1.0 ± 0.35	1.0 ± 0.35	-5 ≤ b ≤ 5	-5 ≤ b ≤ 5	75%*	75%*	-	-
Sensor PA01	0.32	0.63	0.34	0.53	0.57	-0.52	99	100	717	30

	Error			
	RMSE (µg/m³)		NRMSE (%)	
	1-Hour ★	24-Hour ★	1-Hour ☆	24-Hour ☆
Metric Target Range	≤ 7.0	≤ 7.0	≤ 30.0	≤ 30.0
Deployment Value	3.9	3.2	70.1	58.2

Device-specific metrics (computed for each sensor in evaluation)
○○○ Metric value for none of devices tested falls within the target range
●○○ Metric value for one of devices tested falls within the target range
●●○ Metric value for two of devices tested falls within the target range
●●● Metric value for three of devices tested falls within the target range

Single-valued metrics (computed via entire evaluation dataset)
☆ Indicates that the metric value is not within the target range
★ Indicates that the metric value is within the target range

Sensor-Sensor Precision[1]

	Precision (between collocated sensors)				Data Quality	
	CV (%)		SD (µg/m³)		Number of concurrent sensor concentration pairs	
	1-Hour ☆	24-Hour ☆	1-Hour ☆	24-Hour ☆	1-Hour	24-Hour
Metric Target Range	≤ 30.0	≤ 30.0	≤ 5.0	≤ 5.0	-	-
Deployment Value	-	-	-	-	-	-

[1]Precision statistics are computed for evaluations with at least three collocated sensor units. Metric values are left blank for evaluations with two or fewer sensor units.

*This value is only a recommendation for ensuring data quality and is not included in the list of target values discussed in Section 4 of the Performance Testing Protocols, Metrics, and Target Values for Fine Particulate Matter Air Sensors document.

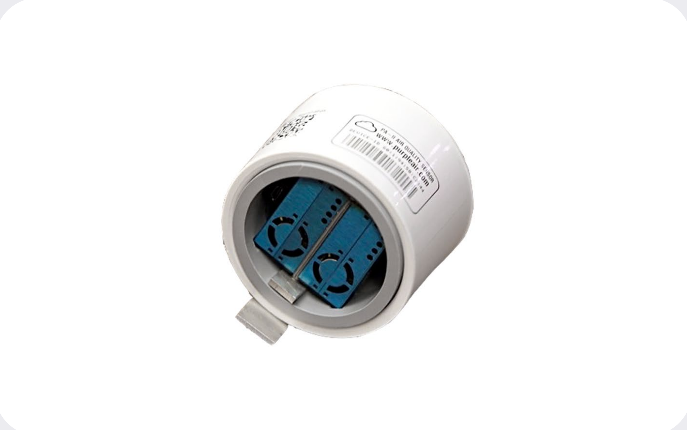

Sensor-FRM/FEM Scatter Plots

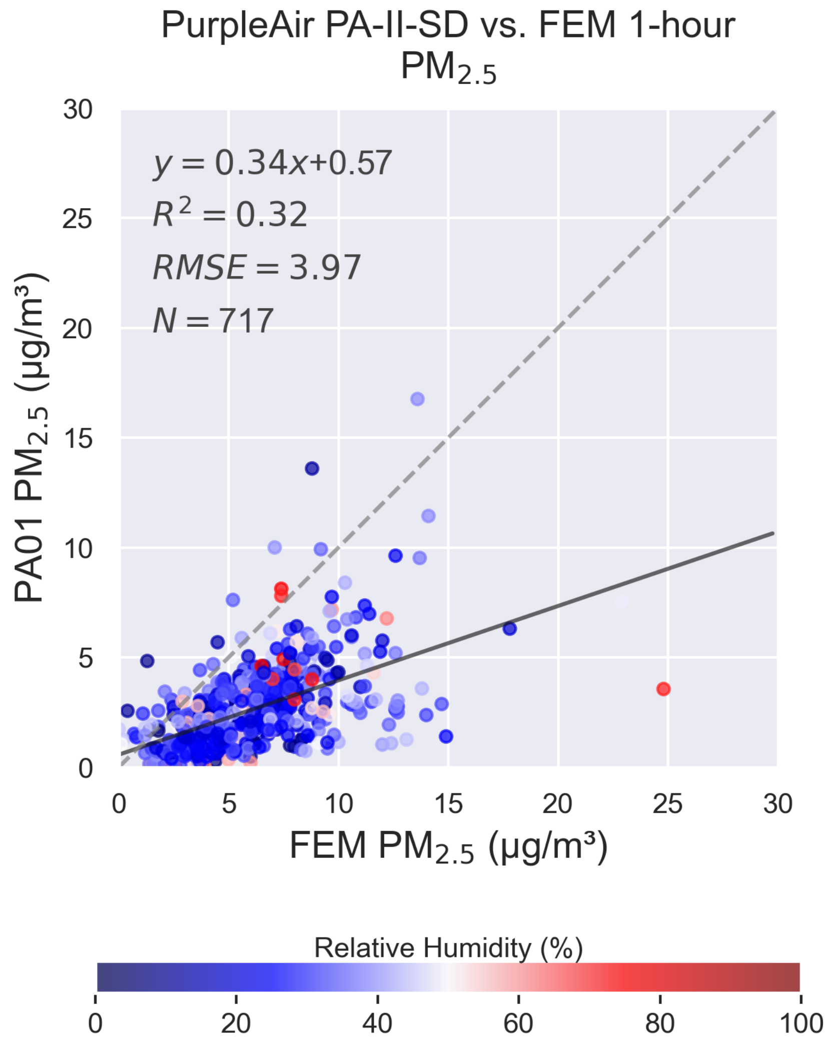

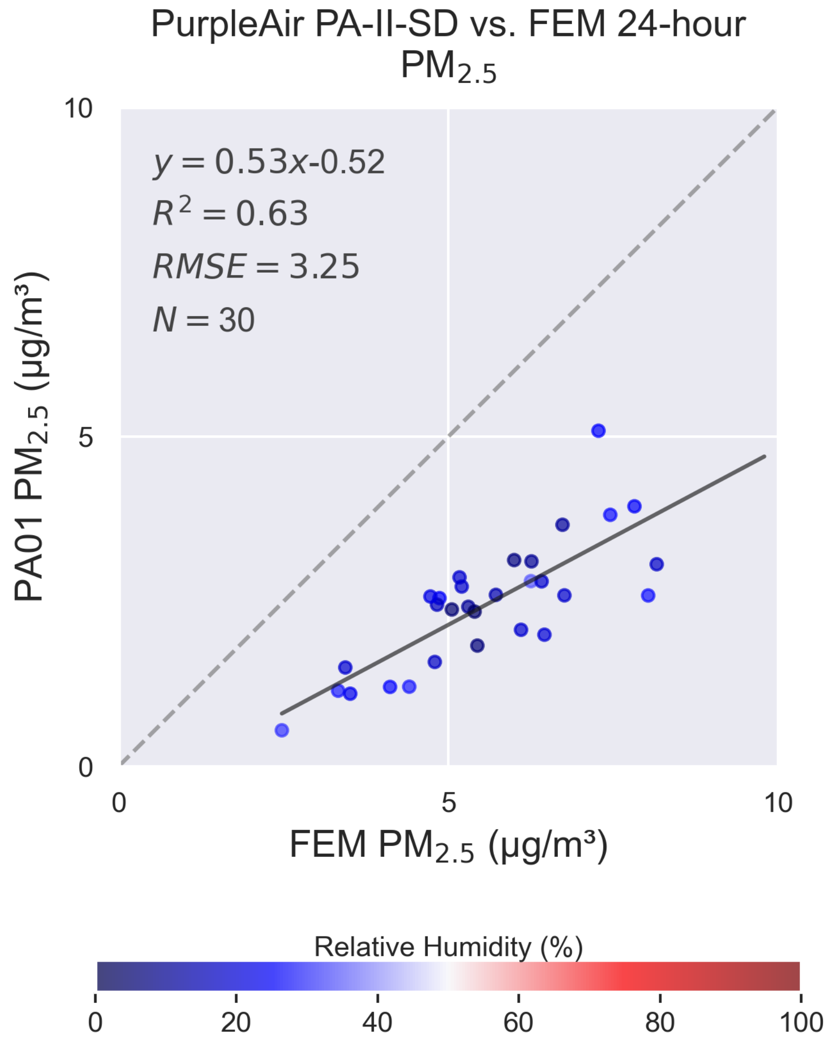

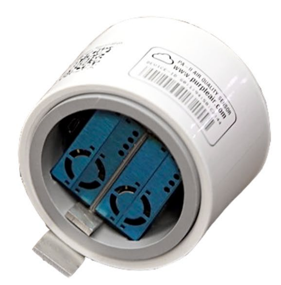

Supplemental Information

Abbreviations used in Supplemental Information

FRM	Federal Reference Method
FEM	Federal Equivalent Method
SOP	Standard Operating Procedure
QAPP	Quality Assurance Project Plan
QC	Quality Control

Supplemental Documentation	Attached	Description & URL or file path to documentation
Field observations and sensor data flags	☑	See AZ-PAR-Page 6 of this testing report
Maintenance logs	☐	No logs recorded during testing
Standard operating procedure(s)	☐	U.S. EPA Office Of Research and Development SOP available upon request
Photos of equipment setup and testing	☑	See AZ-PAR-Page 5 of this testing report
Product specifications sheet(s)	☑	PurpleAir: PA-II-SD Product website
Product manual(s)	☐	N/A
Data storage and transmission method	☑	See AZ-PAR-Page 6 of this testing report
Data correction approach	☑	See AZ-PAR-Page 6 of this testing report
Issues encountered	☑	See AZ-PAR-Page 6 of this testing report. No issues were encountered during testing; however, various issues were faced during the pre-deployment phase.
Data analysis/correction scripts and version	☑	Averaging and processing of data, calculation of performance metrics, and generation of figures and other supplementary material for analysis were obtained using Python 3.9.7 with the packages sensortoolkit v0.8.3b2, pandas 1.3.5, NumPy 1.21.2, Matplotlib 3.5.0, statsmodels 0.13.0, and seaborn 0.11.2. All packages are available from the Python Package Index (PyPI) at https://pypi.org/. The integrated development environment (IDE) Spyder 5.1.5 was used for scripting and data visualization. Version control for the Python base, packages, and IDE were all managed by conda 4.11.0.
Air Monitoring Station QAPP	☐	U.S. EPA Office Of Research and Development QAPP available upon request
Summary of FRM/FEM monitor QC checks	☑	See AZ-PAR-Page 7 of this testing report
Manufacturer website for FRM/FEM monitor	☑	Thermo Fisher Scientific: TEOM 1405 Product website
FRM/FEM monitor manual	☑	Thermo Fisher Scientific: TEOM 1405 Product Manual
FRM/FEM monitor specifications sheet(s)	☑	Thermo Fisher Scientific: TEOM 1405 Specification Sheet
Other documents	☐	

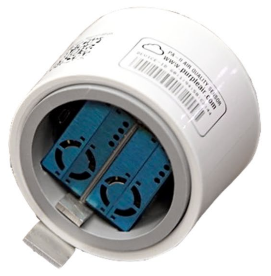

Supplemental Information: Photos of Testing Site and Equipment Setup

Site Description:
The West Phoenix Monitoring Station has been operational since 1984. The spatial scale for the West Phoenix site is Neighborhood. It is located in an area of stable, high-density residential properties. This State or Local Air Monitoring Stations (SLAMS) location monitors for CO, NO_2, O_3, PM_{10}, and $PM_{2.5}$. In addition, this is a quality assurance (QA) collocation site for $PM_{2.5}$ where the Maricopa County Air Quality Department (MCAQD) operates one filter-based $PM_{2.5}$ FRM sampler along with one continuous $PM_{2.5}$ FEM analyzer as per 40 CFR Part 58 Appendix A. Resources detailing air quality monitoring QA programs and procedures are detailed on EPA's Ambient Monitoring Technology Information Center website (https://www.epa.gov/amtic/ambient-air-monitoring-quality-assurance, *last accessed 5/11/2022*). Meteorological monitors operating at this site measure ambient temperature (T), barometric pressure, delta T (temperature inversion), and wind speed/direction.

Figure 1: PurpleAir PA-II-SD sensor (indicated by red arrow) attached to metal railing atop the sampling shelter at the monitoring site.

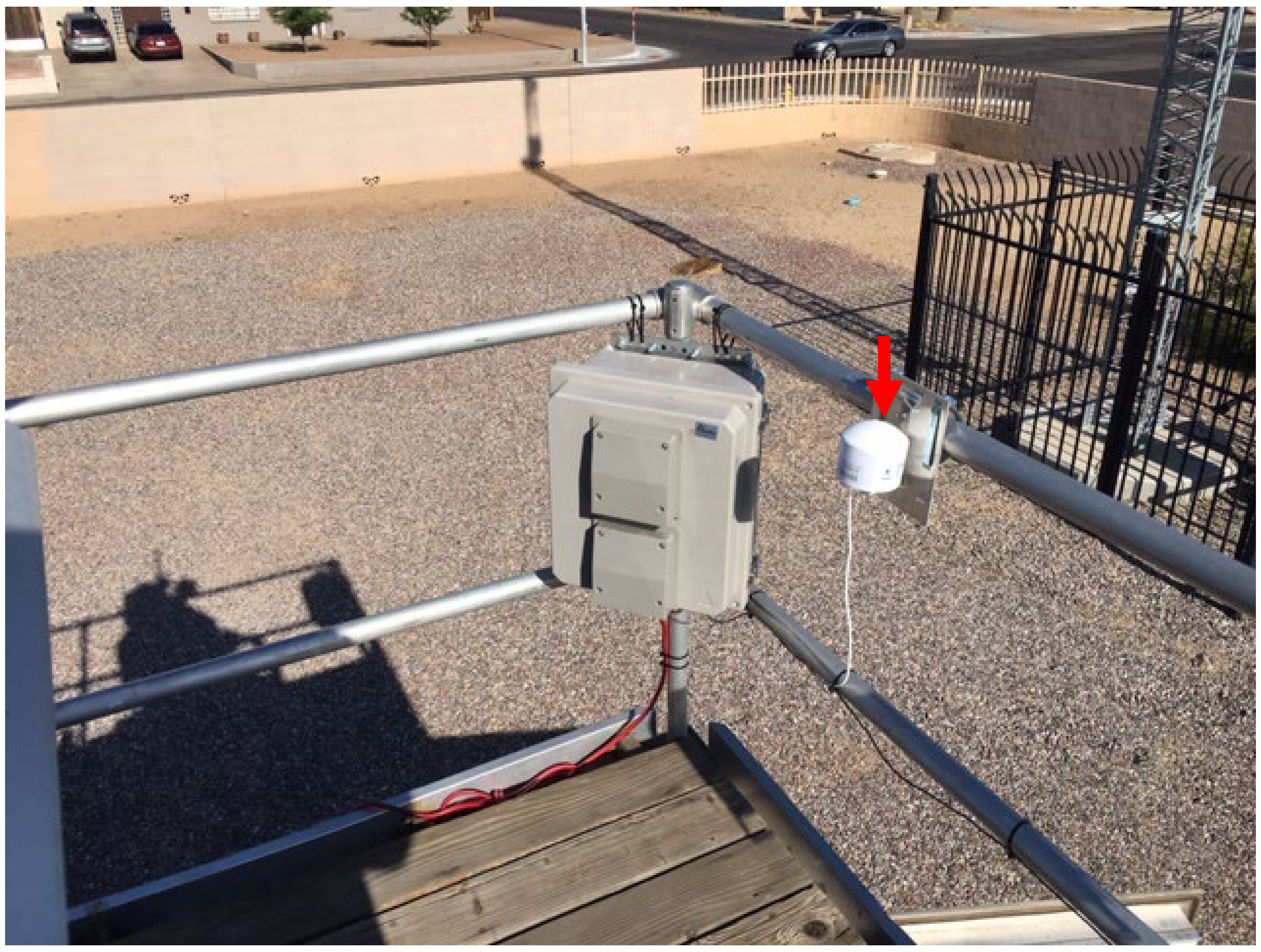

Figure 2: West Phoenix Monitoring Station

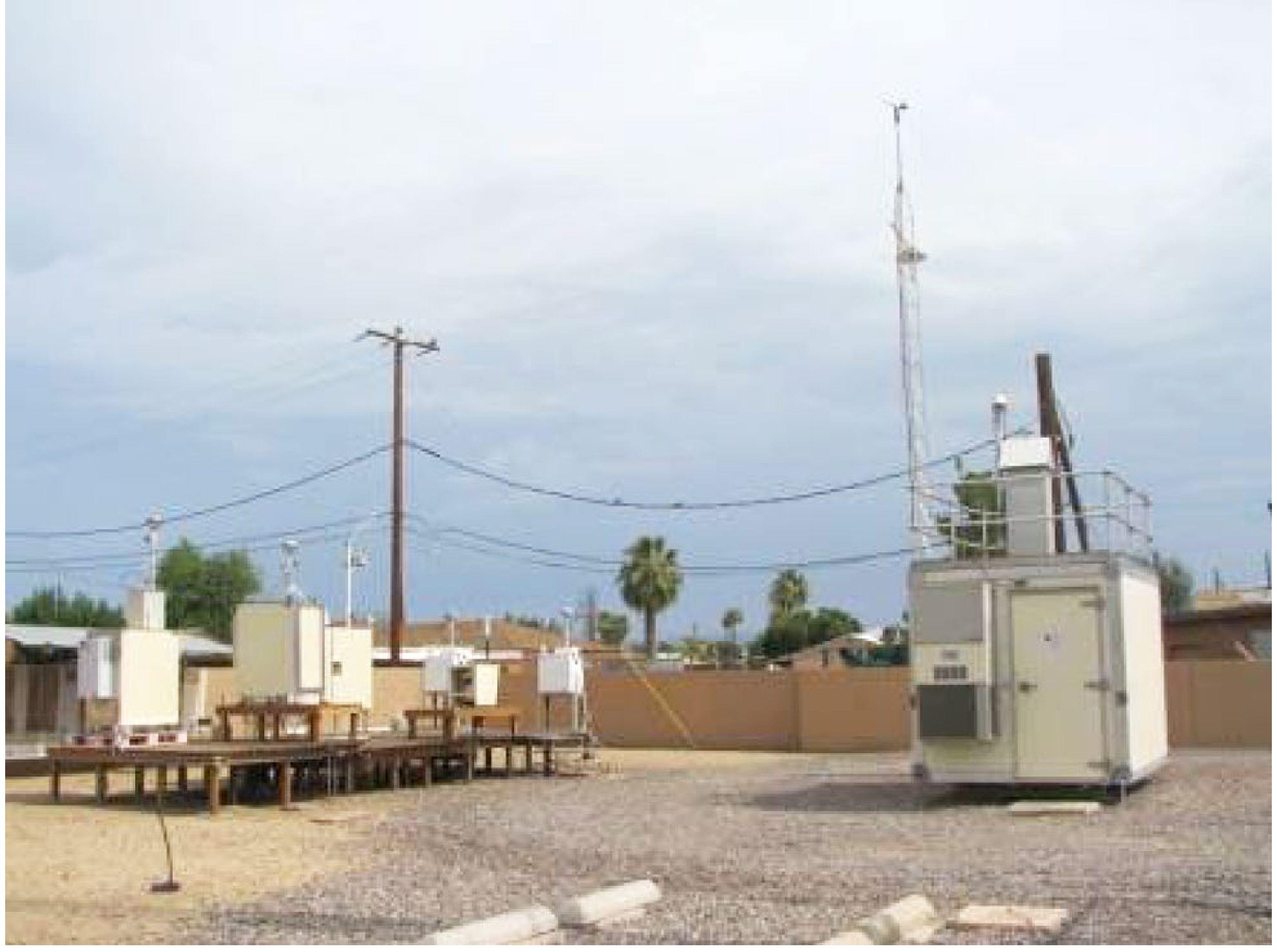

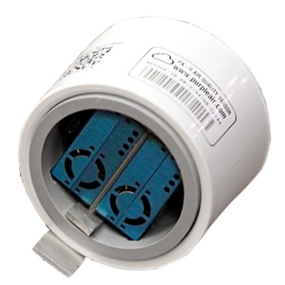

Data Storage and Transmission Method

The PurpleAir PA-II-SD records data at a 2-minute[1] sampling interval. Data is transmitted to the PurpleAir Cloud via Wi-Fi and stored on an internal MicroSD card. Due to the differing file structure between remote and direct download methods and the lag associated with cloud data download, weekly raw data were obtained primarily via onboard SD card. Each field site operator was provided two labeled MicroSD cards for sensor units that they used to swap out each week. Data from the collected card was then read and processed off-site.

Data Correction Approach

Data sets recorded by PurpleAir PA-II-SD contain two correction factors for PM concentrations, referred to internally as "CF=ATM" and "CF=1". $PM_{2.5}$ concentrations are recorded concurrently for each correction factor, and corresponding concentrations agree closely up to approximately 30 $\mu g/m^3$, above which one correction factor reflects concentrations roughly 1.5 times concentrations for the other correction factor. During a separate analysis by ORD, concentrations for the higher correction factor were determined to have slightly higher correlation with collocated reference measurements. As a result, data for the higher correction factor were used for this study. Prior to firmware version 5.00 (released on 11/30/2019), headers for concentrations corresponding to each correction factor were incorrectly labeled (i.e., CF=1 was labeled CF=ATM and vice versa). Testing indicated in this report was completed prior to this firmware update, and thus data sets acquired for PA-II-SD units had swapped $PM_{2.5}$ CF labels. Recorded PA-II-SD data sets indicate that the CF=ATM correction factor was the higher CF as labeled (following firmware update 5.00, this data channel corresponds to CF=1) and sensor data shown in this report correspond to this correction factor.

After acquisition, the raw data was processed using the *sensortoolkit* python code library (v0.8.3b2). A continuous data set at the recorded sampling frequency was written to a .csv file. 1-hour and 24-hour averaged data sets were generated using a 75% completeness threshold and saved as separate .csv files. Simultaneous measurement from the PurpleAir PA-II's dual PMS5003 sensors (labeled channels A and B) allows for identification of outliers resulting in large-scale disagreement between recorded values. The outlier removal method of Barkjohn et al. 2021[2] was applied to both 1-hour and 24-hour averaged A and B measurement pairs. This method includes thresholds for the absolute difference and the percent difference between hourly channel values. A-B channel averages were not included for hours where the absolute difference between channel values was greater than 5 $\mu g/m^3$ and the percent difference exceeded 70%. This outlier removal method is in use on the AirNow Fire and Smoke map and has been used for this evaluation since it requires no external information (e.g., data from the monitor) and no assumptions about typical PM2.5 concentrations (e.g., removing data over a certain threshold concentration).

Issues Encountered

Pre-deployment observations

- Prior to the testing period indicated in this report, the PurpleAir PA-II-SD unit was briefly collocated for a period of approximately one week at EPA's Ambient Monitoring Innovative Research Station (AIRS) in Research Triangle Park, NC. The unit was tested for proper operation, including powering on and data logging (either to the onboard SD card or via hotspot Wi-Fi connection to the PurpleAir Cloud), sampling, and acquisition of recorded data from either onboard memory or cloud storage. Units that did not pass this pre-deployment testing were returned to the manufacturer and constituted approximately 1 in 10 units tested for subsequent performance evaluation.

Field observations and sensor data flags

The PurpleAir PA-II-SD was deployed at the West Phoenix monitoring site on 8/12/2019. The PA-II-SD unit operated nominally during the testing period and did not require replacement or repair.

During the testing period, no data flags logged by field technicians were recorded.

[1]As of version 4.02, updated across devices on 5/31/2019. Prior to this firmware version, devices recorded measurements at 80-second intervals
[2]Barkjohn, K. K., Gantt, B., and Clements, A. L.: Development and application of a United States-wide correction for PM2.5 data collected with the PurpleAir sensor, Atmos. Meas. Tech., 14, 4617–4637, https://doi.org/10.5194/amt-14-4617-2021, 2021.

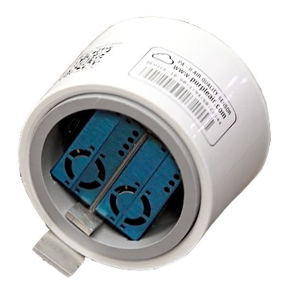

Description of Data Flags

AQS

The U.S. EPA's Air Quality System (AQS) is the Agency's primary ambient air monitoring data archive. A comprehensive list of data flags that are recorded alongside AQS data sets, referred to by U.S. EPA as 'qualifiers', can be found at the following link: https://aqs.epa.gov/aqsweb/documents/codetables/qualifiers.html

Invalidation of reference data: AQS qualifiers are organized by qualifier type, which indicates whether data logged alongside qualifier flags should be invalidated (set null). Qualifiers with type "Null Data Qualifier" are invalidated, and includes data logged during periods that coincide with QC checks (e.g., "BF-Precision/Zero/Span", "BJ- Operator Error", "BL - QA Audit", "AZ - QC Audit") among other events such as power outages. Data logged alongside qualifiers with type "Quality Assurance Qualifiers" are not invalidated and are included in this analysis (e.g., concentrations less than the federal MDL for the reference monitor "MD – Value less than MDL", QA reviewed values "Validated Value").

Data Flags Recorded During Testing

FRM/FEM Monitor	Timestamp (UTC)	Flag
Thermo Fisher 1405-DF TEOM FDMS Dichotomous FEM (Data acquired via AQS)	2019-08-06 19:00:00+0000 to 2019-08-06 20:00:00+0000	BM - Accuracy check
	2019-08-14 17:00:00+0000 to 2019-08-14 18:00:00+0000	AY - QC Control Points (zero/span)
	2019-08-20 16:00:00+0000 to 2019-08-20 17:00:00+0000	BM - Accuracy check
	2019-08-28 17:00:00+0000 to 2019-08-28 19:00:00+0000	AY - QC Control Points (zero/span)

Testing Report - $PM_{2.5}$ Base Testing

SENSIT RAMP

This report reflects out-of-the-box performance

Initial Base Testing - Phoenix, AZ
U.S. Environmental Protection Agency
Office of Research and Development
PI: Clements.Andrea@epa.gov
919-541-1363
September 2019—October 2019

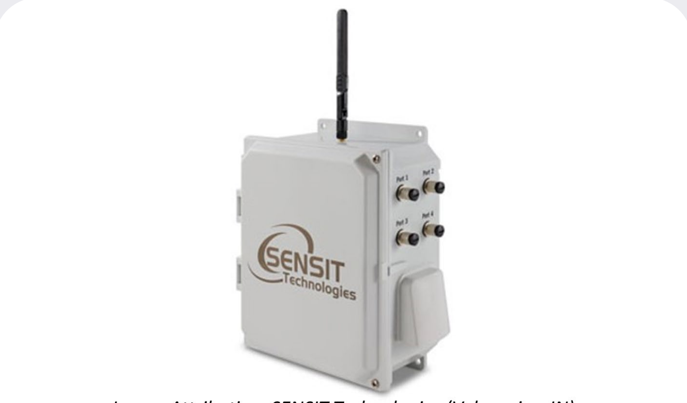

Image Attribution: SENSIT Technologies (Valparaiso, IN)

Deployment Details

Testing Organization and Site Information	
Testing organization (Name, Organization type, Contact website)	U.S. Environmental Protection Agency - Office of Research and Development<br>Federal Government<br>Air Sensor Toolbox | U.S. EPA Website
Testing location (City, State, Latitude and Longitude)	West Phoenix<br>Phoenix, AZ<br>33.48385, -112.14257
AQS site ID	04 - 013 - 0019
Sampling timeframe (MM-DD-YY)	09-01-19 to 10-01-19
Sensor data source	Onboard MicroSD card
Reference data source	AQS API download

Sensor Information			
Manufacturer, model	SENSIT RAMP		
Device firmware version	190308_AQ_v9.30		
Sampling time interval	15-seconds		
Sensor serial numbers	RAM_01		
Issues encountered during deployment?	☑	See AZ-RAM-Page 6 of this testing report	

FRM/FEM Information	
Manufacturer, model, designation	Thermo Scientific TEOM 1405-DF Dichot. with FDMS FEM
Sampling time interval	1-hour averaging
Date of calibration	As required by 40 CFR Part 58 and the Air Monitoring Network Plan maintained by MCAQD
Date of flowrate verification check	Monthly as required by 40 CFR Part 58 Appendix A
Description, date(s) of maintenance activities	N/A

Time Series Plots

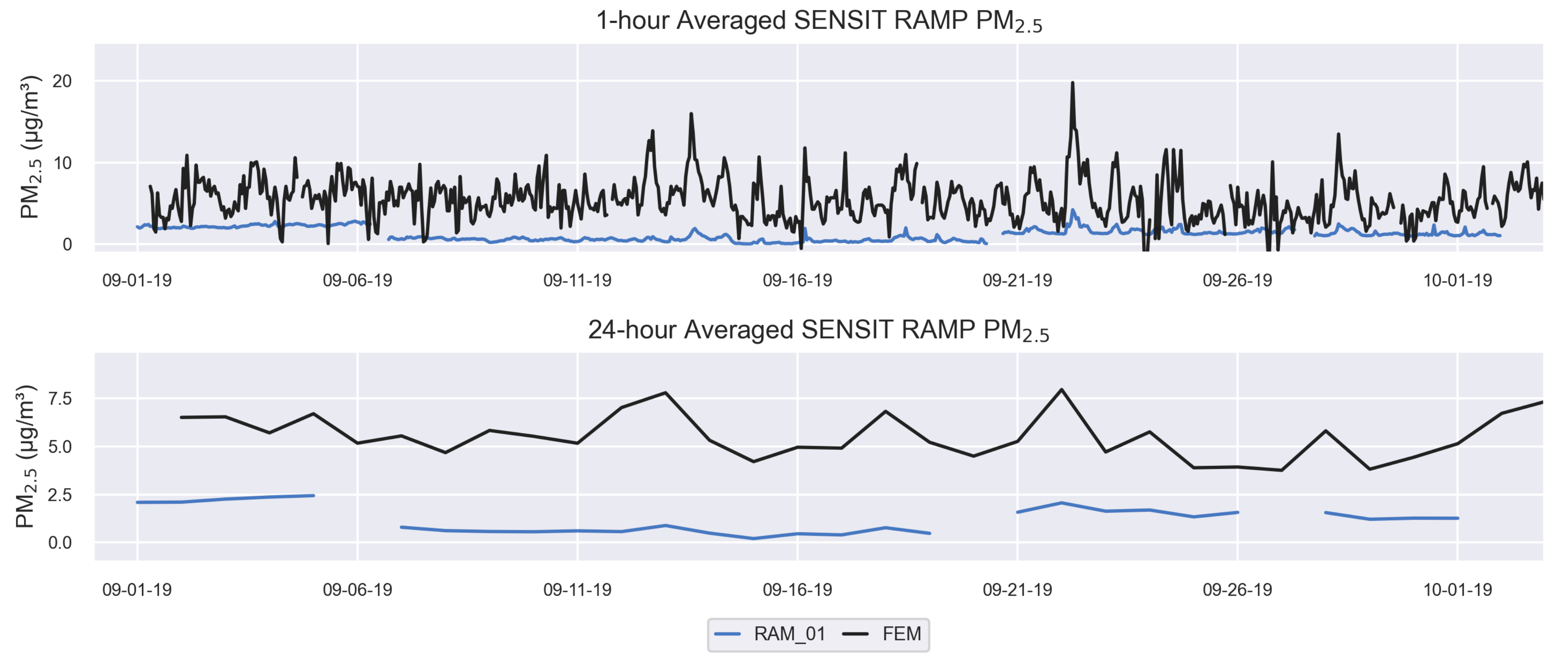

Scatter Plots: Comparison to FRM/FEM

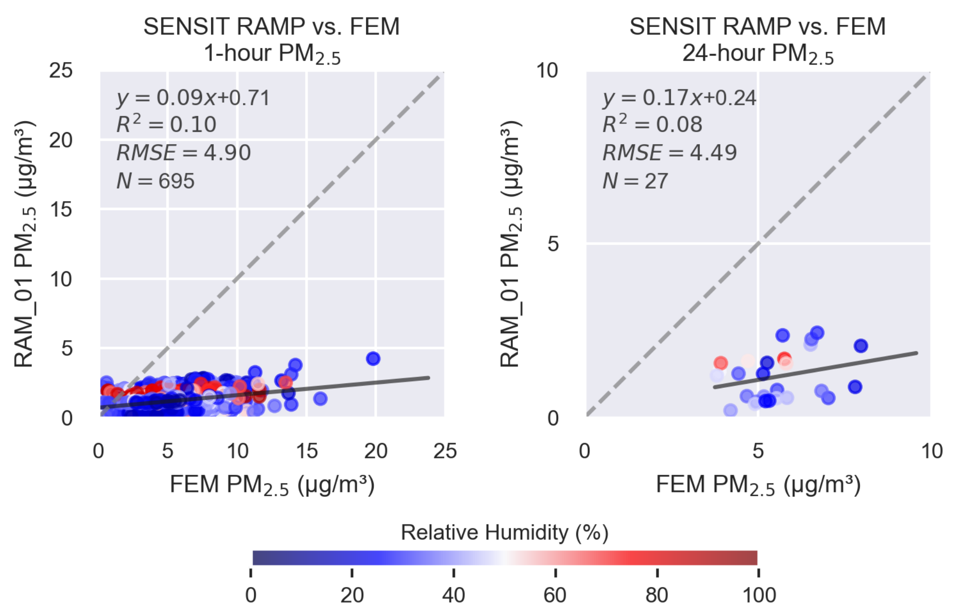

Range of FRM/FEM concentrations over duration of base test (µg/m³)	-4.4-19.8 (1-hr),<br>3.8-8.0 (24-hr)
Number of 24-hr periods in FRM/FEM monitor measurements with a goal concentration ≥ 25 µg/m³	0

Performance Metrics

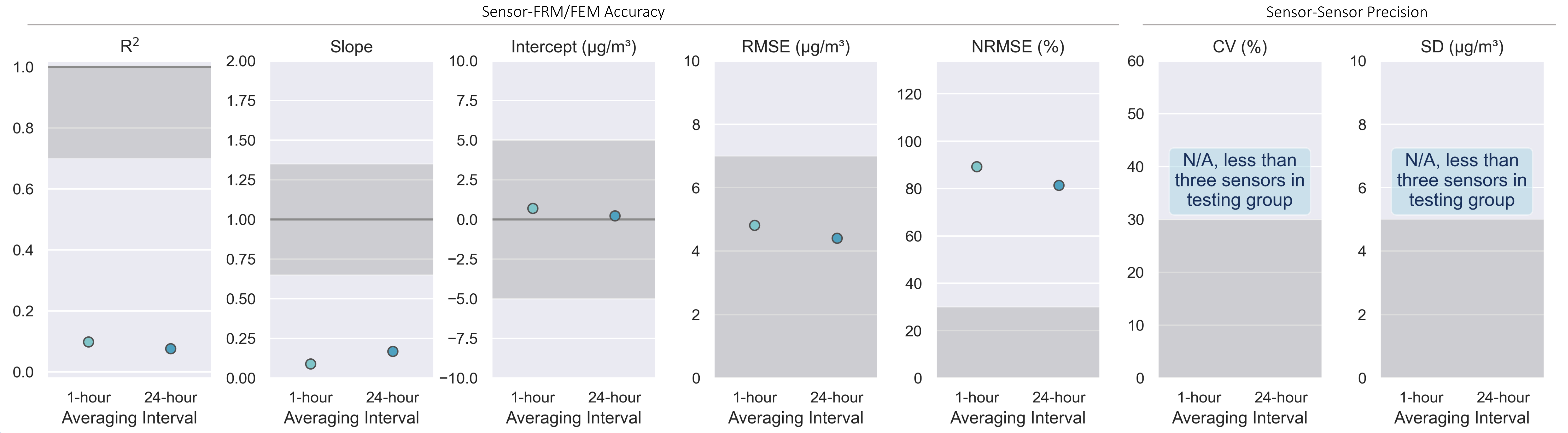

Meteorological Conditions During Deployment

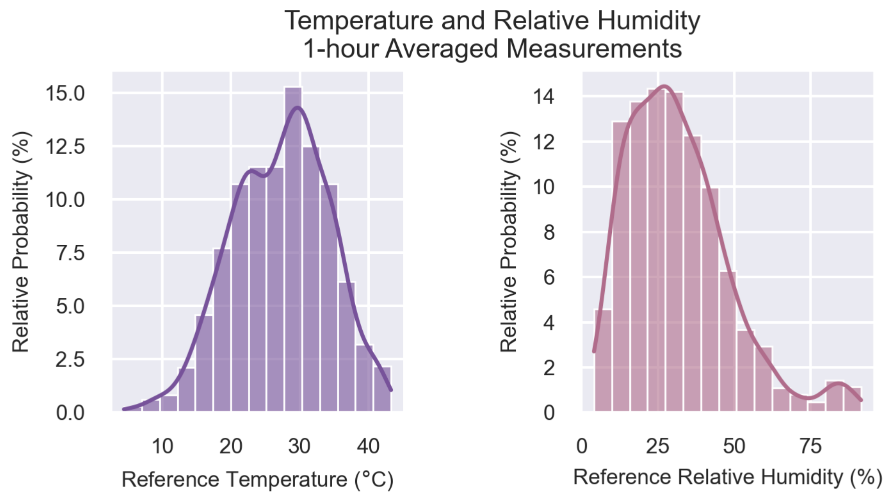

Number of 24-hr periods outside sensor manufacture-listed temperature operational range (-20 to 50 °C)	0
Number of 24-hr periods outside sensor manufacture-listed relative humidity operational range (no operational range specified)	-

Meteorological Influence

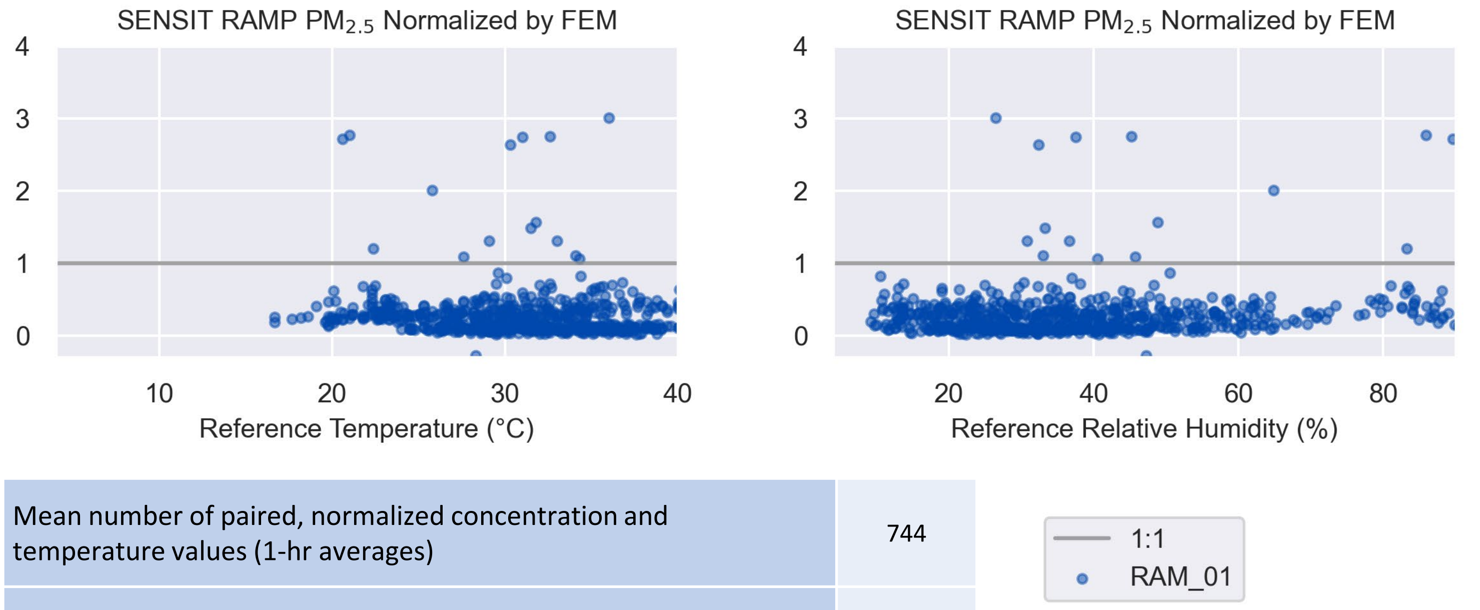

Mean number of paired, normalized concentration and temperature values (1-hr averages)	744
Mean number of paired, normalized concentration and relative humidity values (1-hr averages)	744

Testing Report - $PM_{2.5}$ Base Testing

SENSIT RAMP

This report reflects out-of-the-box performance

Initial Base Testing - Phoenix, AZ
U.S. Environmental Protection Agency
Office of Research and Development
PI: Clements.Andrea@epa.gov
919-541-1363
September 2019—October 2019

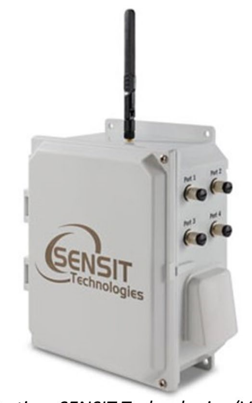

Image Attribution: SENSIT Technologies (Valparaiso, IN)

Tabular Statistics

Sensor-FRM/FEM Correlation

	Bias and Linearity						Data Quality			
	R^2		Slope		Intercept (µg/m³)		Uptime (%)		Number of paired sensor and FRM/FEM concentration values	
	1-Hour ○	24-Hour ○	1-Hour ○	24-Hour ○	1-Hour ●	24-Hour ●	1-Hour ●	24-Hour ●	1-Hour	24-Hour
Metric Target Range	≥ 0.70	≥ 0.70	1.0 ± 0.35	1.0 ± 0.35	-5 ≤ b ≤ 5	-5 ≤ b ≤ 5	75%*	75%*	-	-
Sensor RAM_01	0.10	0.08	0.09	0.17	0.71	0.24	96	90	695	27

	Error			
	RMSE (µg/m³)		NRMSE (%)	
	1-Hour ★	24-Hour ★	1-Hour ☆	24-Hour ☆
Metric Target Range	≤ 7.0	≤ 7.0	≤ 30.0	≤ 30.0
Deployment Value	4.8	4.4	89.3	81.4

Device-specific metrics (computed for each sensor in evaluation)
○○○ Metric value for none of devices tested falls within the target range
●○○ Metric value for one of devices tested falls within the target range
●●○ Metric value for two of devices tested falls within the target range
●●● Metric value for three of devices tested falls within the target range

Single-valued metrics (computed via entire evaluation dataset)
☆ Indicates that the metric value is not within the target range
★ Indicates that the metric value is within the target range

Sensor-Sensor Precision[1]

	Precision (between collocated sensors)				Data Quality	
	CV (%)		SD (µg/m³)		Number of concurrent sensor concentration pairs	
	1-Hour ☆	24-Hour ☆	1-Hour ☆	24-Hour ☆	1-Hour	24-Hour
Metric Target Range	≤ 30.0	≤ 30.0	≤ 5.0	≤ 5.0	-	-
Deployment Value	-	-	-	-	-	-

[1]Precision statistics are computed for evaluations with at least three collocated sensor units. Metric values are left blank for evaluations with two or fewer sensor units.

*This value is only a recommendation for ensuring data quality and is not included in the list of target values discussed in Section 4 of the Performance Testing Protocols, Metrics, and Target Values for Fine Particulate Matter Air Sensors document.

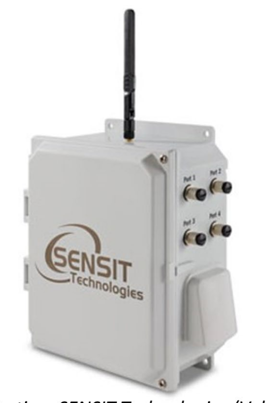

Image Attribution: SENSIT Technologies (Valparaiso, IN)

Sensor-FRM/FEM Scatter Plots

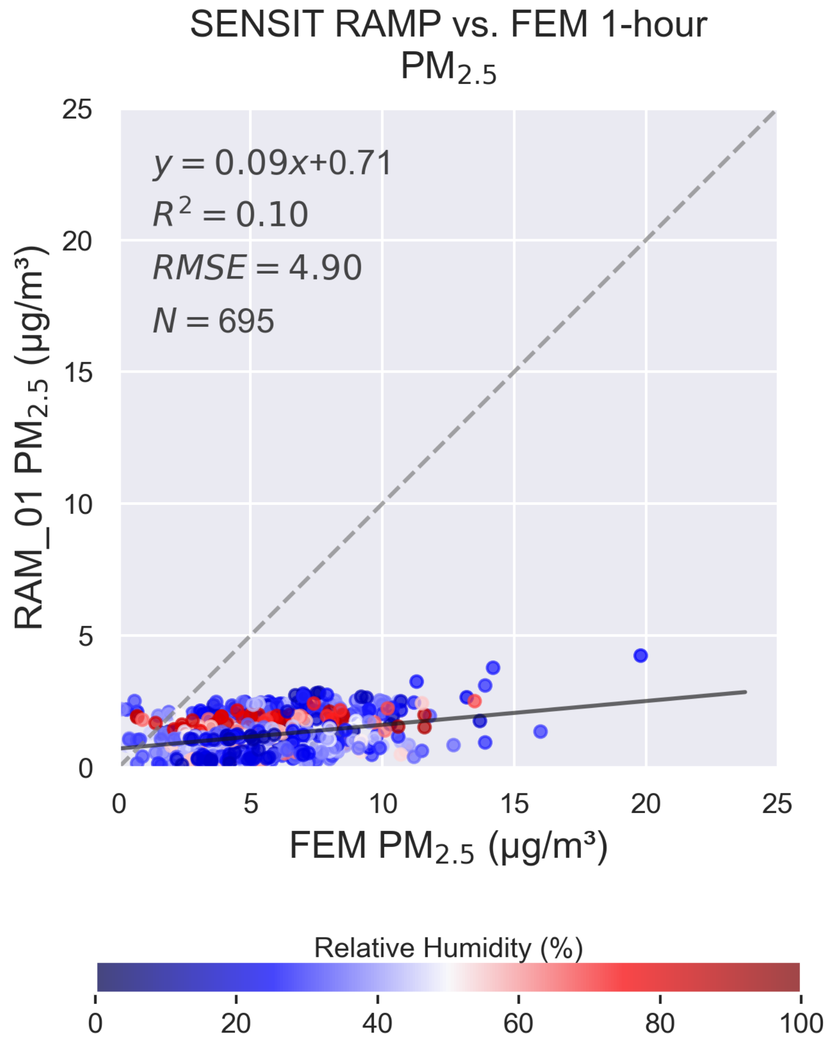

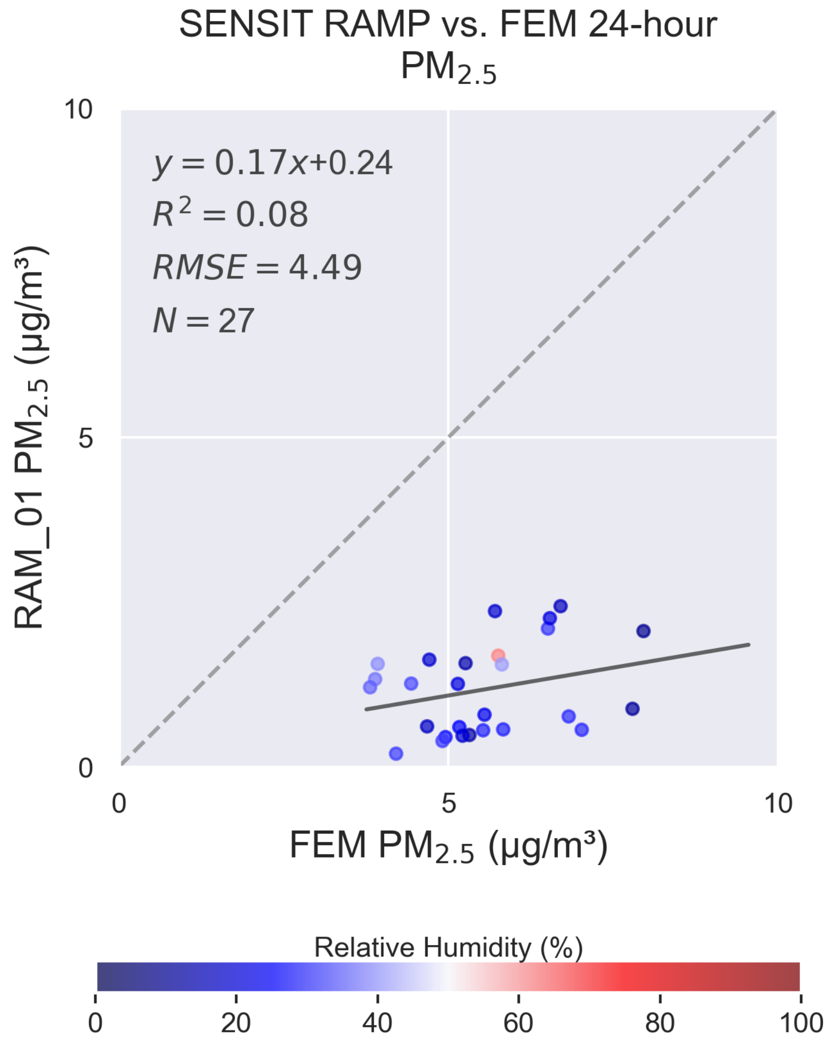

Initial Base Testing - Phoenix, AZ
U.S. Environmental Protection Agency
Office of Research and Development
PI: Clements.Andrea@epa.gov
919-541-1363
September 2019—October 2019

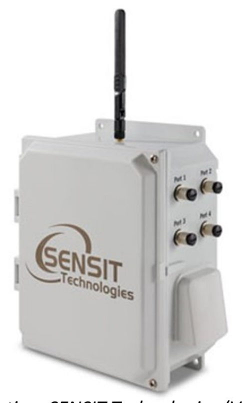

Image Attribution: SENSIT Technologies (Valparaiso, IN)

Supplemental Information

Abbreviations used in Supplemental Information

FRM	Federal Reference Method
FEM	Federal Equivalent Method
SOP	Standard Operating Procedure
QAPP	Quality Assurance Project Plan
QC	Quality Control

Supplemental Documentation	Attached	Description & URL or file path to documentation
Field observations and sensor data flags	☑	See AZ-RAM-Page 6 of this testing report
Maintenance logs	☐	No logs recorded during testing
Standard operating procedure(s)	☐	U.S. EPA Office Of Research and Development SOP available upon request
Photos of equipment setup and testing	☑	See AZ-RAM-Page 5 of this testing report
Product specifications sheet(s)	☑	SENSIT Technologies: RAMP Product Brochure
Product manual(s)	☑	SENSIT Technologies: RAMP Product Manual
Data storage and transmission method	☑	See AZ-RAM-Page 6 of this testing report
Data correction approach	☑	See AZ-RAM-Page 6 of this testing report
Issues encountered	☑	See AZ-RAM-Page 6 of this testing report
Data analysis/correction scripts and version	☑	Averaging and processing of data, calculation of performance metrics, and generation of figures and other supplementary material for analysis were obtained using Python 3.9.7 with the packages sensortoolkit v0.8.3b2, pandas 1.3.5, NumPy 1.21.2, Matplotlib 3.5.0, statsmodels 0.13.0, and seaborn 0.11.2. All packages are available from the Python Package Index (PyPI) at https://pypi.org/. The integrated development environment (IDE) Spyder 5.1.5 was used for scripting and data visualization. Version control for the Python base, packages, and IDE were all managed by conda 4.11.0.
Air Monitoring Station QAPP	☐	U.S. EPA Office Of Research and Development QAPP available upon request
Summary of FRM/FEM monitor QC checks	☑	See AZ-RAM-Page 7 of this testing report
Manufacturer website for FRM/FEM monitor	☑	Thermo Fisher Scientific: TEOM 1405 Product website
FRM/FEM monitor manual	☑	Thermo Fisher Scientific: TEOM 1405 Product Manual
FRM/FEM monitor specifications sheet(s)	☑	Thermo Fisher Scientific: TEOM 1405 Specification Sheet
Other documents	☐	

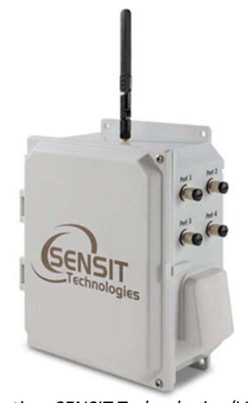

Image Attribution: SENSIT Technologies (Valparaiso, IN)

Site Description:
The West Phoenix Monitoring Station has been operational since 1984. The spatial scale for the West Phoenix site is Neighborhood. It is located in an area of stable, high-density residential properties. This State or Local Air Monitoring Stations (SLAMS) location monitors for CO, NO_2, O_3, PM_{10}, and $PM_{2.5}$. In addition, this is a quality assurance (QA) collocation site for $PM_{2.5}$ where the Maricopa County Air Quality Department (MCAQD) operates one filter-based $PM_{2.5}$ FRM sampler along with one continuous $PM_{2.5}$ FEM analyzer as per 40 CFR Part 58 Appendix A. Resources detailing air quality monitoring QA programs and procedures are detailed on EPA's Ambient Monitoring Technology Information Center website (https://www.epa.gov/amtic/ambient-air-monitoring-quality-assurance, *last accessed 5/11/2022*). Meteorological monitors operating at this site measure ambient temperature (T), barometric pressure, delta T (temperature inversion), and wind speed/direction.

Figure 1: SENSIT RAMP sensor (indicated by red arrow) attached to metal railing atop the sampling shelter at the monitoring site.

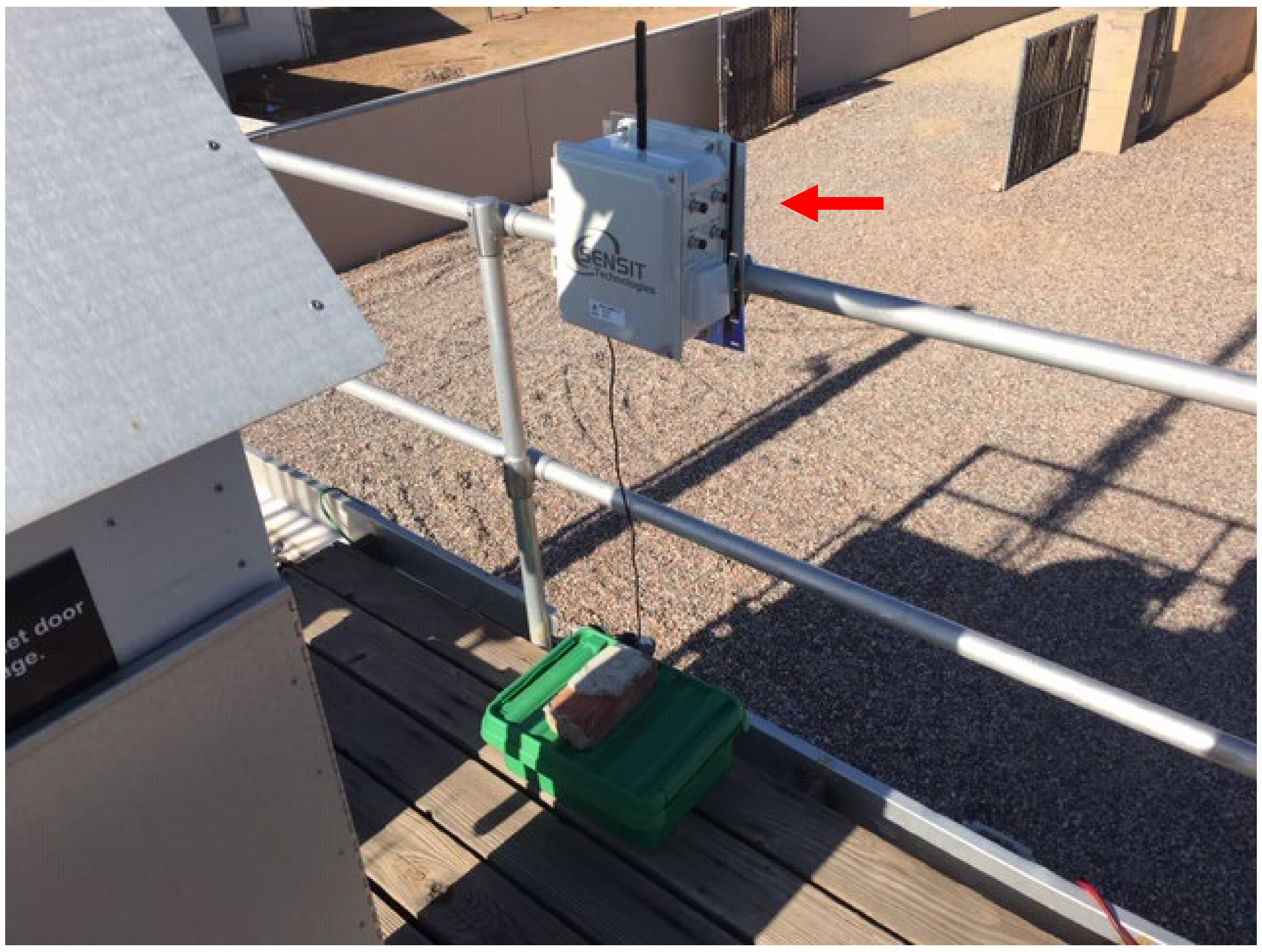

Figure 2: West Phoenix Monitoring Station

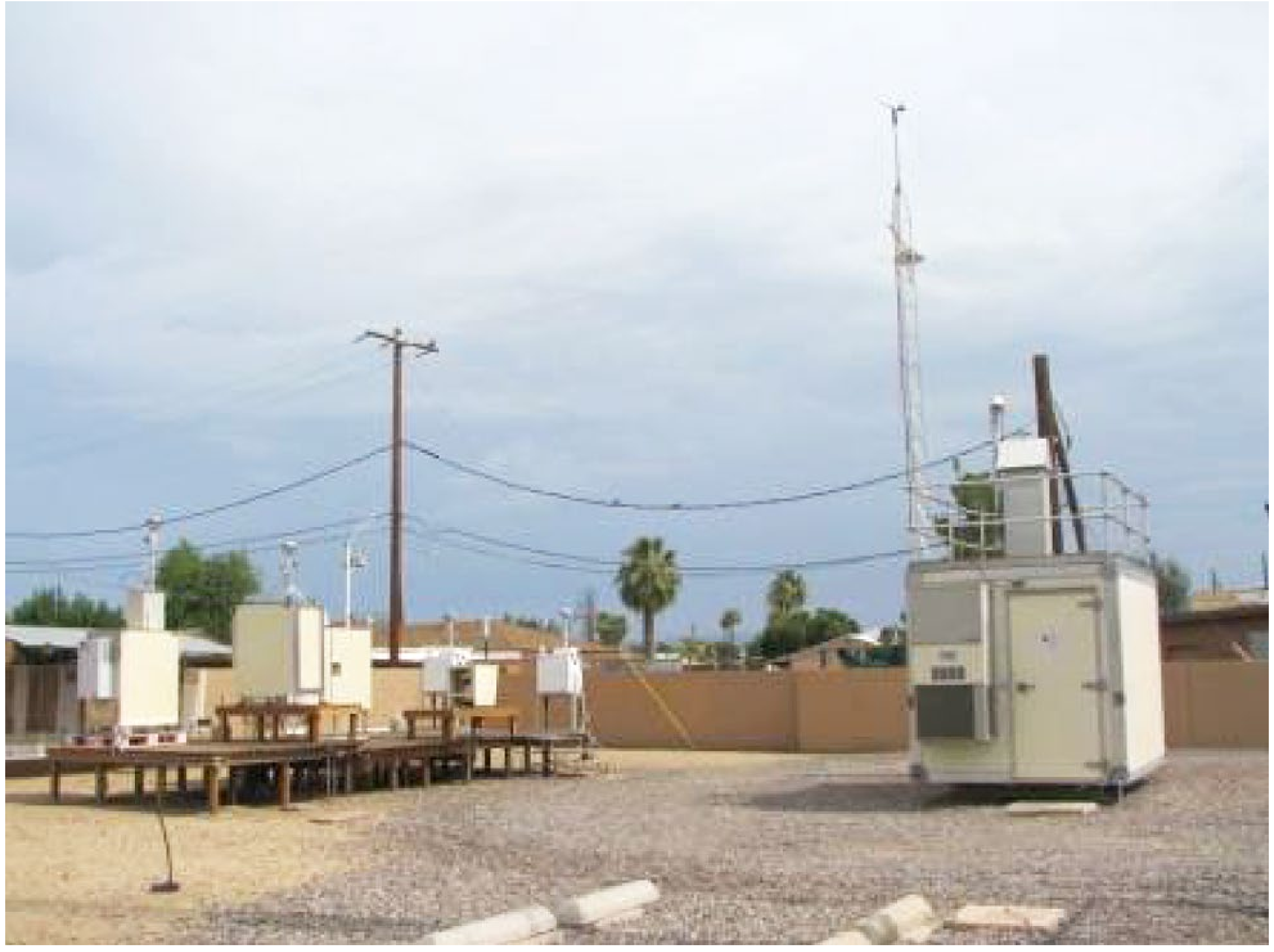

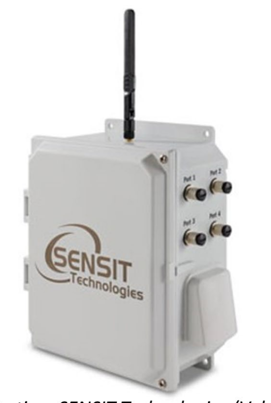
Image Attribution: SENSIT Technologies (Valparaiso, IN)

Supplemental Information: Data Storage, Correction Approach, and Issues Encountered

Data Storage and Transmission Method

The SENSIT RAMP was configured to record data at a 15-second sampling interval. Data are stored as daily text files (.txt format) on an onboard MicroSD card. Data files were obtained weekly via SD cards. Each field site operator was provided two labeled MicroSD cards for sensor units that they used to swap out each week. Data from the collected card was then read and processed off-site.

Data Correction Approach

SENSIT RAMP units were pre-configured by the manufacturer with a linear correction (i.e., concentration gain = 1.0 and offset = 0.0 µg m^{-3}). These presets reflect out-of-the box performance and were not modified by EPA prior or during testing.

After acquisition, the raw data was processed using the *sensortoolkit* python code library (v0.8.3b2). A continuous data set at the recorded sampling frequency was written to a .csv file. 1-hour and 24-hour averaged data sets were generated using a 75% completeness threshold and saved as separate .csv files. Outliers were NOT removed from data sets in order to assess "out-of-the-box" sensor performance.

Issues Encountered

Pre-deployment observations

- SENSIT RAMP units were received without documentation or manuals. After communicating the need to change default settings (logging interval and time zone) with the manufacturer, a draft user's manual and a USB cable were supplied. With the use of this USB cable, instrument settings could be changed, and real-time data could be logged using a serial communication software (CoolTerm, v.1.5.0). Because the sensor did not record data at the top of every minute, the RAMP was configured to record data at 15-second intervals so that the data could be averaged more closely to complete minutes. Prior to deployment, RAMP units were collocated in a bench-top evaluation to verify operational status and determine the extent of data invalidity (i.e., determine equilibration period) after an initial start-up event. The recorded response for parameters measured by the RAMP suggests that the gas sensors (CO, NO, NO_2, O_3) required approximately a 2-hour equilibration period, while the remaining sensors (temperature, relative humidity, particulate matter) did not require any equilibration period.

Field observations and sensor data flags

During operator visits for sensor data collection on 9/6/2019, 9/20/2019, and 9/27/2019, slight baseline offsets were observed in sensor concentrations following data acquisition and redeployment. The magnitude of these sudden changes in baseline offsets was approximately 2 µg/m^3 or less.

During the testing period, no data flags logged by field technicians were recorded.

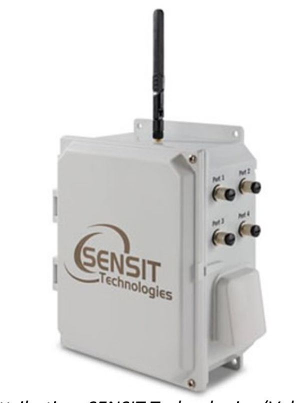

Image Attribution: SENSIT Technologies (Valparaiso, IN)

Description of Data Flags

AQS

The U.S. EPA's Air Quality System (AQS) is the Agency's primary ambient air monitoring data archive. A comprehensive list of data flags that are recorded alongside AQS data sets, referred to by U.S. EPA as 'qualifiers', can be found at the following link: https://aqs.epa.gov/aqsweb/documents/codetables/qualifiers.html

AirNow-Tech

AirNow-Tech is an additional ambient air monitoring data service maintained by U.S. EPA and is commonly used by monitoring agencies to upload and validate monitoring data. Data which have yet to be QC'd for inclusion in AQS as well as monitoring data sets which are not planned for inclusion in AQS are typically available for near real-time download from AirNow-Tech.

Invalidation of reference data: AQS qualifiers are organized by qualifier type, which indicates whether data logged alongside qualifier flags should be invalidated (set null). Qualifiers with type "Null Data Qualifier" are invalidated, and includes data logged during periods that coincide with QC checks (e.g., "BF-Precision/Zero/Span", "BJ- Operator Error", "BL - QA Audit", "AZ - QC Audit") among other events such as power outages. Data logged alongside qualifiers with type "Quality Assurance Qualifiers" are not invalidated and are included in this analysis (e.g., concentrations less than the federal MDL for the reference monitor "MD – Value less than MDL", QA reviewed values "Validated Value").

Data Flags Recorded During Testing

FRM/FEM Monitor	Timestamp (UTC)	Flag
Thermo Fisher 1405-DF TEOM FDMS Dichotomous FEM (Data acquired via AQS)	2019-09-04 16:00:00+0000 to 2019-09-04 17:00:00+0000	BM - Accuracy check
	2019-09-11 17:00:00+0000 to 2019-09-11 18:00:00+0000	AY - QC Control Points (zero/span)
	2019-09-18 18:00:00+0000	BL - QA Audit
	2019-09-18 19:00:00+0000	BM - Accuracy check
	2019-09-24 01:00:00+0000 to 2019-09-24 02:00:00+0000	AV - Power Failure
	2019-09-25 18:00:00+0000 to 2019-09-25 19:00:00+0000	AY - QC Control Points (zero/span)
	2019-09-29 14:00:00+0000 to 2019-09-29 16:00:00+0000	AV - Power Failure
	2019-10-01 17:00:00+0000 to 2019-10-01 18:00:00+0000	BM - Accuracy check

Meteorological Instrument	Timestamp (UTC)	Flag
MetOne Temperature Monitor (Data acquired via AirNow-Tech)	2019-09-29 14:00:00+0000	9 - Invalid
	2019-10-01 19:00:00+0000 to 2019-10-01 20:00:00+0000	9 - Invalid
RM Young Relative Humidity Monitor (Data acquired via AirNow-Tech)	2019-09-29 14:00:00+0000	9 - Invalid

Testing Report - $PM_{2.5}$ Base Testing

QuantAQ ARISense

This report reflects out-of-the-box performance

Initial Base Testing - Phoenix, AZ

U.S. Environmental Protection Agency
Office of Research and Development
PI: Clements.Andrea@epa.gov
919-541-1363

February 2020—March 2020

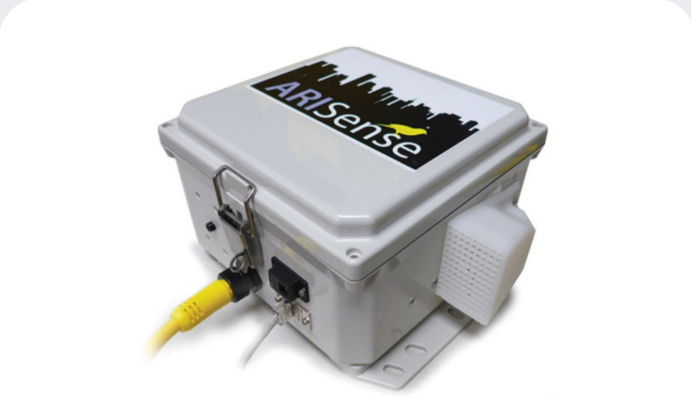

Image Attribution: QuantAQ, Inc (Somerville, MA)

Deployment Details

Testing Organization and Site Information	
Testing organization (Name, Organization type, Contact website)	U.S. Environmental Protection Agency - Office of Research and Development<br>Federal Government<br>Air Sensor Toolbox | U.S. EPA Website
Testing location (City, State, Latitude and Longitude)	West Phoenix<br>Phoenix, AZ<br>33.48385, -112.14257
AQS site ID	04 - 013 - 0019
Sampling timeframe (MM-DD-YY)	02-07-20 to 03-08-20
Sensor data source	QuantAQ Cloud download
Reference data source	AQS API download

Sensor Information			
Manufacturer, model	QuantAQ ARISense		
Device firmware version	Received by EPA on 4/30/2019		
Sampling time interval	1-minute		
Sensor serial numbers	ARS_01		
Issues encountered during deployment?	☑	See AZ-ARS-Page 6 of this testing report	

FRM/FEM Information	
Manufacturer, model, designation	Thermo Scientific TEOM 1405-DF Dichot. with FDMS FEM
Sampling time interval	1-hour averaging
Date of calibration	As required by 40 CFR Part 58 and the Air Monitoring Network Plan maintained by MCAQD
Date of flowrate verification check	Monthly as required by 40 CFR Part 58 Appendix A
Description, date(s) of maintenance activities	N/A

Time Series Plots

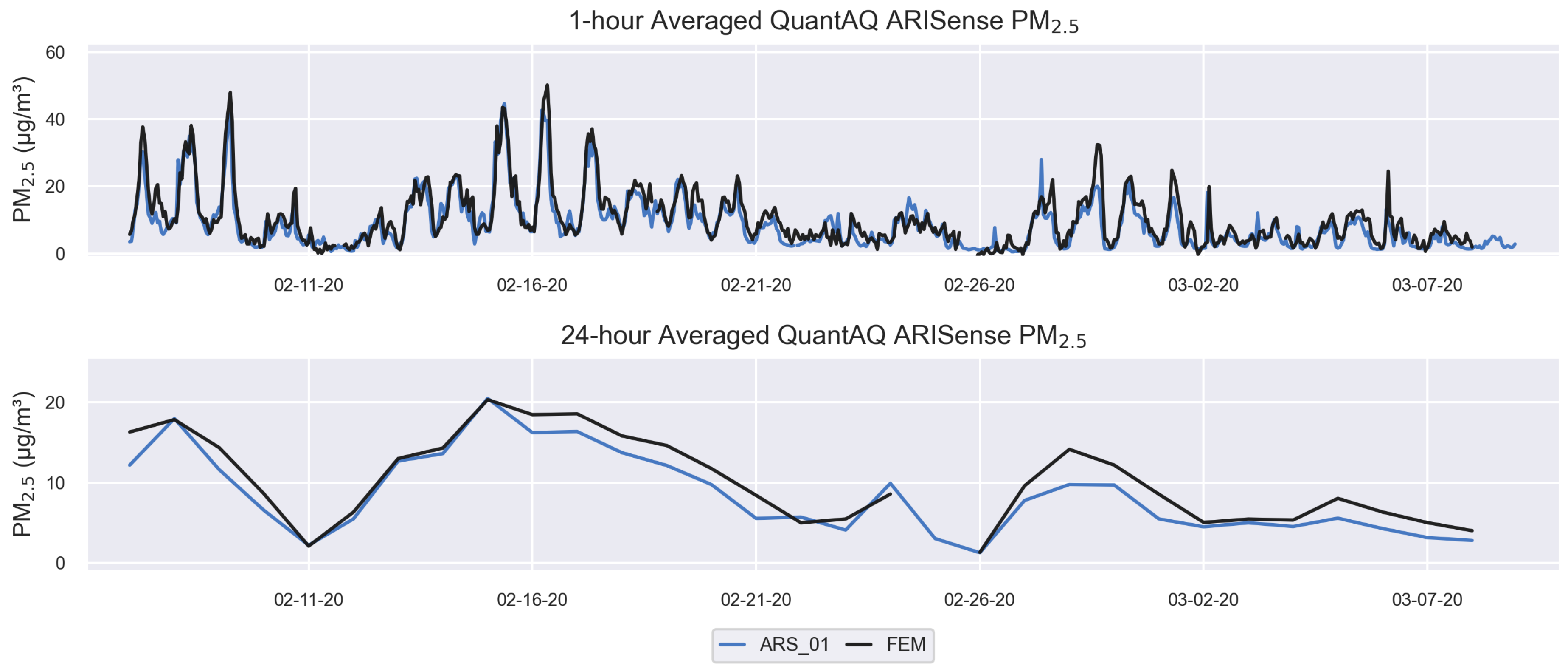

Scatter Plots: Comparison to FRM/FEM

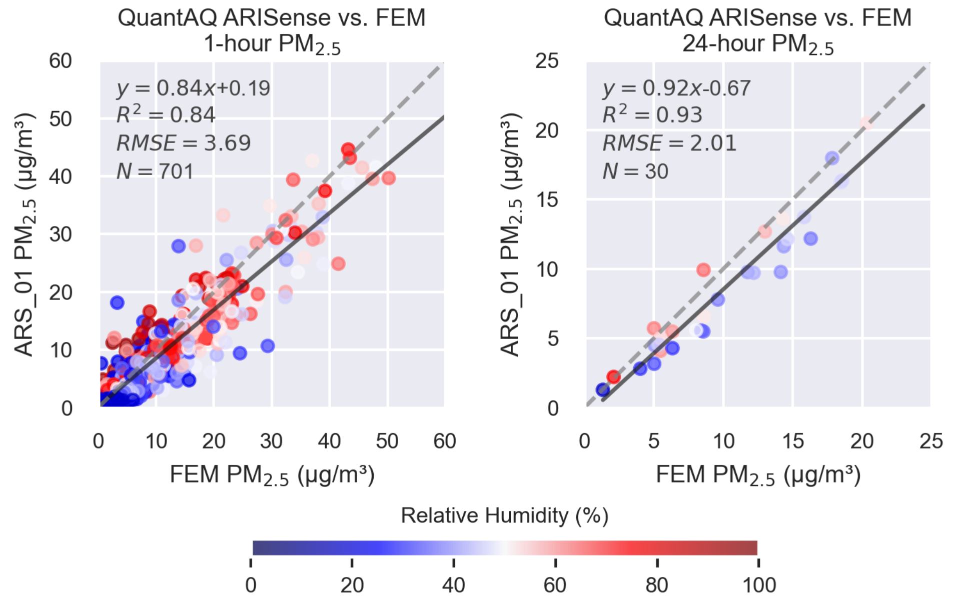

Range of FRM/FEM concentrations over duration of base test (µg/m³)	-2.1-50.2 (1-hr), 1.3-20.3 (24-hr)
Number of 24-hr periods in FRM/FEM monitor measurements with a goal concentration ≥ 25 µg/m³	0

Performance Metrics

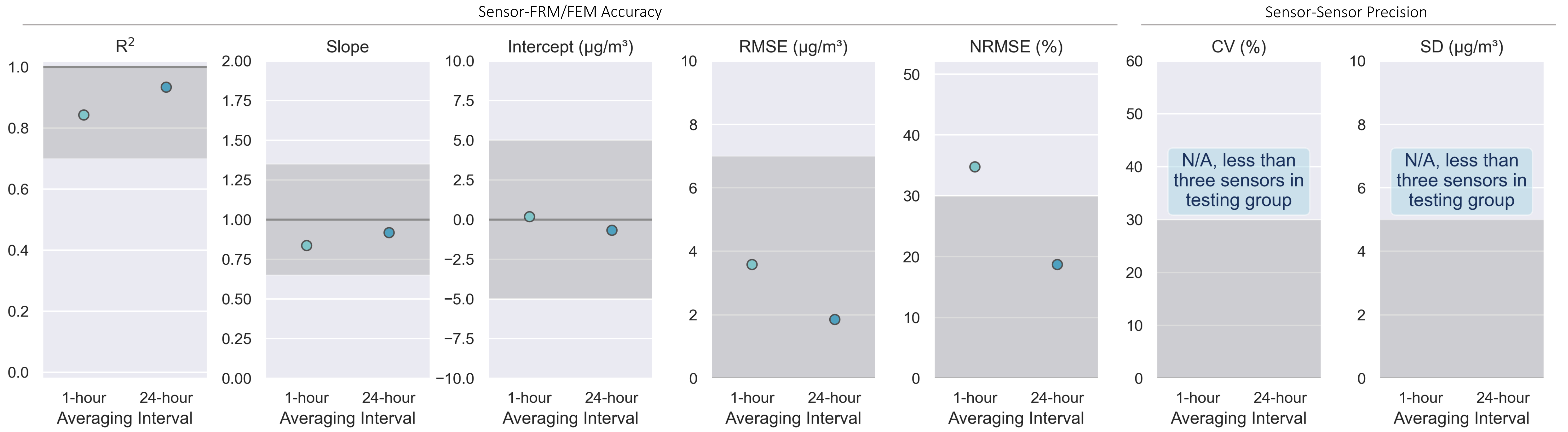

Meteorological Conditions During Deployment

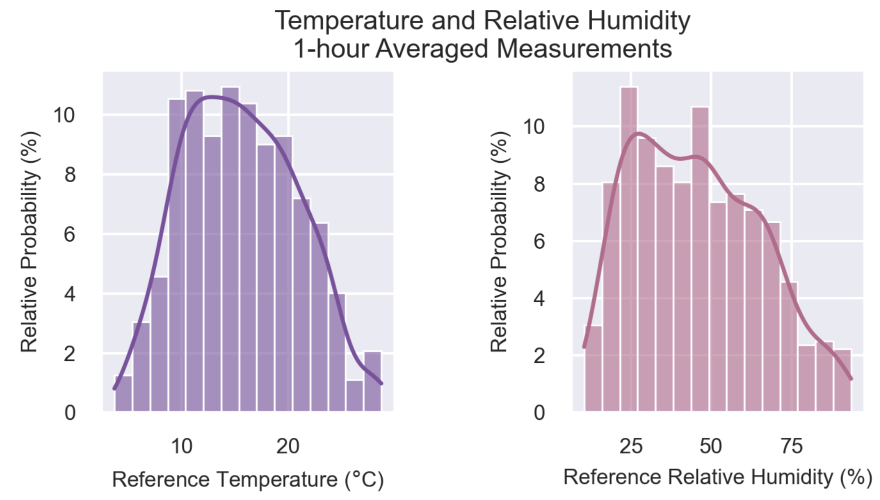

Number of 24-hr periods outside sensor manufacture-listed temperature operational range (no operational range specified)	-
Number of 24-hr periods outside sensor manufacture-listed relative humidity operational range (no operational range specified)	-

Meteorological Influence

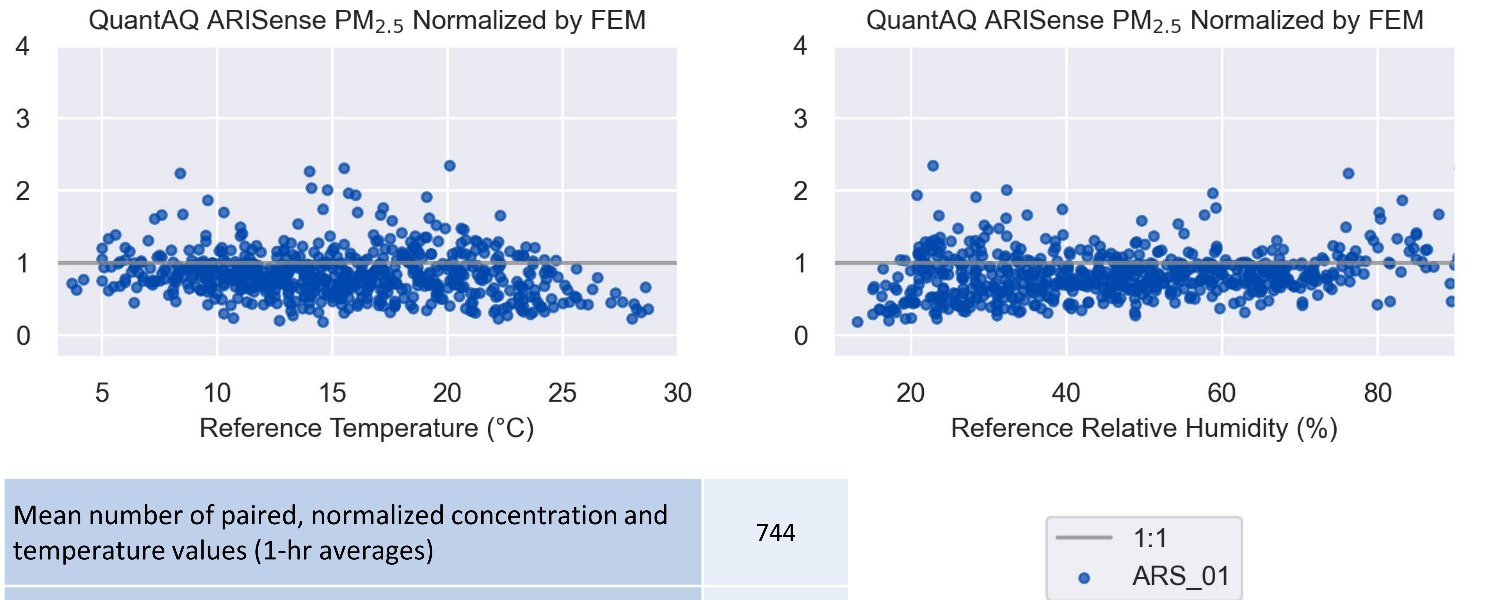

Mean number of paired, normalized concentration and temperature values (1-hr averages)	744
Mean number of paired, normalized concentration and relative humidity values (1-hr averages)	744

U.S. Environmental Protection Agency
Office of Research and Development
PI: Clements.Andrea@epa.gov
919-541-1363

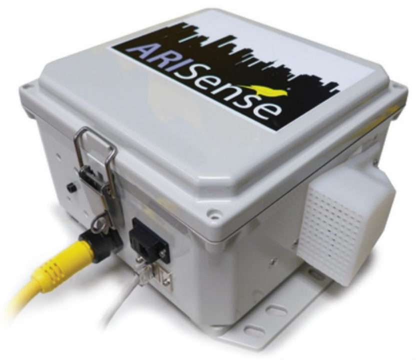

Image Attribution: QuantAQ, Inc (Somerville, MA)

Tabular Statistics

Sensor-FRM/FEM Correlation

	Bias and Linearity						Data Quality			
	R^2		Slope		Intercept (µg/m³)		Uptime (%)		Number of paired sensor and FRM/FEM concentration values	
	1-Hour ●	24-Hour ●	1-Hour ●	24-Hour ●	1-Hour ●	24-Hour ●	1-Hour ●	24-Hour ●	1-Hour	24-Hour
Metric Target Range	≥ 0.70	≥ 0.70	1.0 ± 0.35	1.0 ± 0.35	-5 ≤ b ≤ 5	-5 ≤ b ≤ 5	75%*	75%*	-	-
Sensor ARS_01	0.84	0.93	0.84	0.92	0.19	-0.67	100	100	701	30

	Error			
	RMSE (µg/m³)		NRMSE (%)	
	1-Hour ★	24-Hour ★	1-Hour ☆	24-Hour ★
Metric Target Range	≤ 7.0	≤ 7.0	≤ 30.0	≤ 30.0
Deployment Value	3.6	1.9	34.8	18.7

Device-specific metrics (computed for each sensor in evaluation)
○○○ Metric value for none of devices tested falls within the target range
●○○ Metric value for one of devices tested falls within the target range
●●○ Metric value for two of devices tested falls within the target range
●●● Metric value for three of devices tested falls within the target range

Single-valued metrics (computed via entire evaluation dataset)
☆ Indicates that the metric value is not within the target range
★ Indicates that the metric value is within the target range

Sensor-Sensor Precision[1]

	Precision (between collocated sensors)				Data Quality	
	CV (%)		SD (µg/m³)		Number of concurrent sensor concentration pairs	
	1-Hour ☆	24-Hour ☆	1-Hour ☆	24-Hour ☆	1-Hour	24-Hour
Metric Target Range	≤ 30.0	≤ 30.0	≤ 5.0	≤ 5.0	-	-
Deployment Value	-	-	-	-	-	-

[1]Precision statistics are computed for evaluations with at least three collocated sensor units. Metric values are left blank for evaluations with two or fewer sensor units.

*This value is only a recommendation for ensuring data quality and is not included in the list of target values discussed in Section 4 of the Performance Testing Protocols, Metrics, and Target Values for Fine Particulate Matter Air Sensors document.

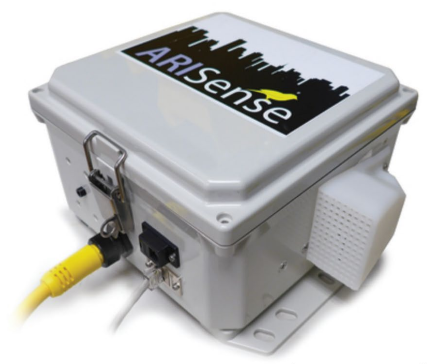

Image Attribution: QuantAQ, Inc (Somerville, MA)

Sensor-FRM/FEM Scatter Plots

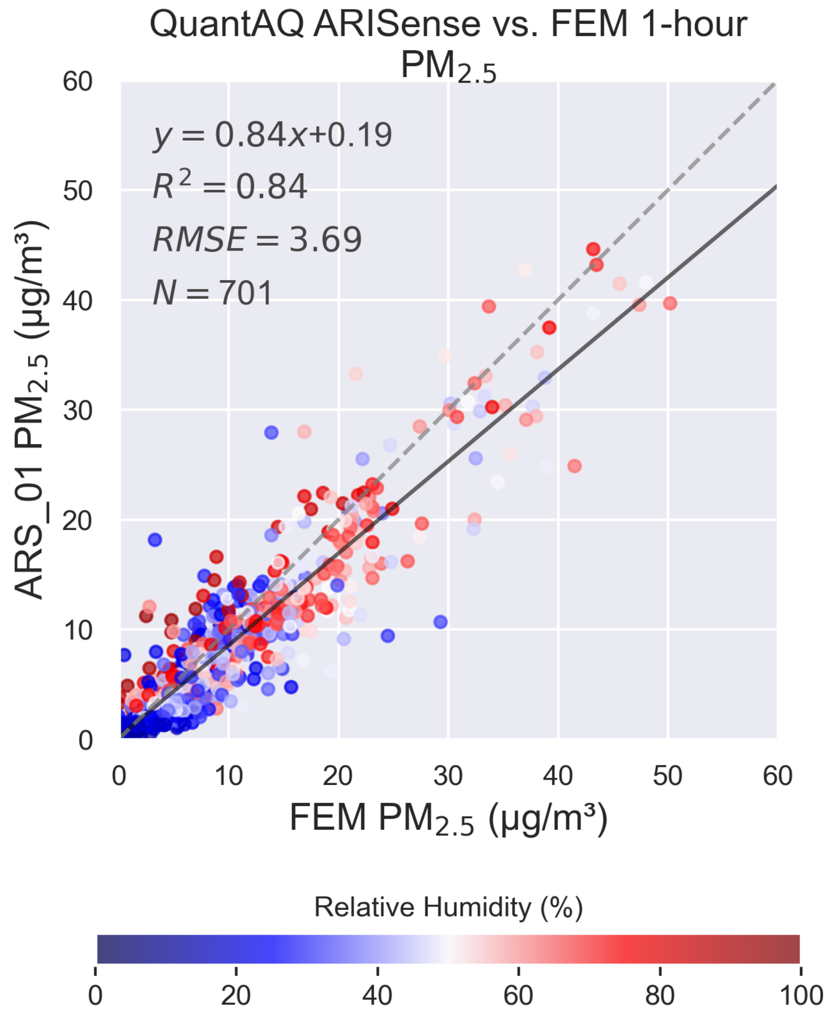

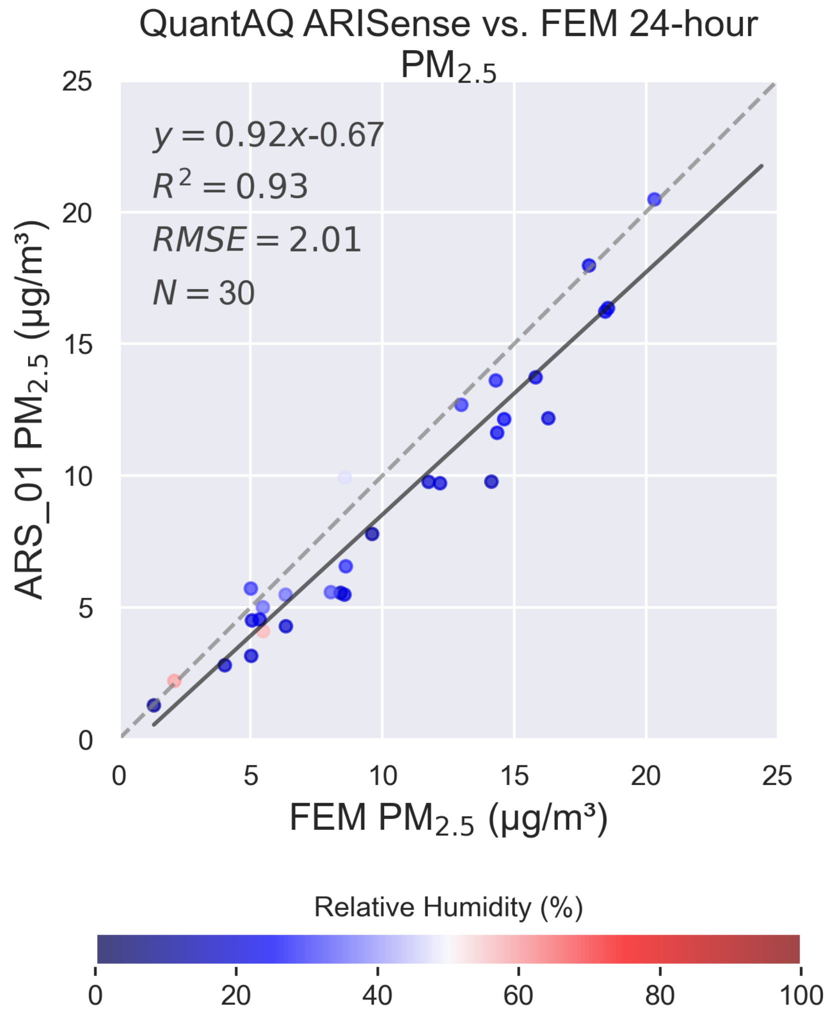

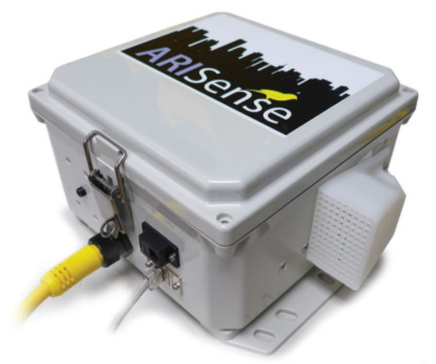

Image Attribution: QuantAQ, Inc (Somerville, MA)

Supplemental Information

Abbreviations used in Supplemental Information

FRM	Federal Reference Method
FEM	Federal Equivalent Method
SOP	Standard Operating Procedure
QAPP	Quality Assurance Project Plan
QC	Quality Control

Supplemental Documentation	Attached	Description & URL or file path to documentation
Field observations and sensor data flags	☑	See AZ-ARS-Page 7 of this testing report
Maintenance logs	☐	No logs recorded during testing
Standard operating procedure(s)	☐	U.S. EPA Office Of Research and Development SOP available upon request
Photos of equipment setup and testing	☑	See AZ-ARS-Page 5 of this testing report
Product specifications sheet(s)	☑	QuantAQ and Particles Plus: ARISense Specification Sheet
Product manual(s)	☐	N/A
Data storage and transmission method	☑	See AZ-ARS-Page 6 of this testing report
Data correction approach	☑	See AZ-ARS-Page 6 of this testing report
Issues encountered	☑	See AZ-ARS-Page 6 of this testing report
Data analysis/correction scripts and version	☑	Averaging and processing of data, calculation of performance metrics, and generation of figures and other supplementary material for analysis were obtained using Python 3.9.7 with the packages sensortoolkit v0.8.3b2, pandas 1.3.5, NumPy 1.21.2, Matplotlib 3.5.0, statsmodels 0.13.0, and seaborn 0.11.2. All packages are available from the Python Package Index (PyPI) at https://pypi.org/. The integrated development environment (IDE) Spyder 5.1.5 was used for scripting and data visualization. Version control for the Python base, packages, and IDE were all managed by conda 4.11.0.
Air Monitoring Station QAPP	☐	U.S. EPA Office Of Research and Development QAPP available upon request
Summary of FRM/FEM monitor QC checks	☑	See AZ-ARS-Page 8 of this testing report
Manufacturer website for FRM/FEM monitor	☑	Thermo Fisher Scientific: TEOM 1405 Product website
FRM/FEM monitor manual	☑	Thermo Fisher Scientific: TEOM 1405 Product Manual
FRM/FEM monitor specifications sheet(s)	☑	Thermo Fisher Scientific: TEOM 1405 Specification Sheet
Other documents	☐	

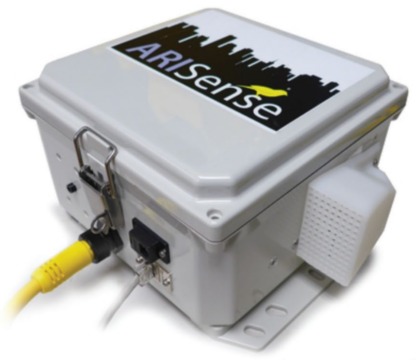

Image Attribution: QuantAQ, Inc (Somerville, MA)

Supplemental Information: Photos of Testing Site and Equipment Setup

Site Description:

The West Phoenix Monitoring Station has been operational since 1984. The spatial scale for the West Phoenix site is Neighborhood. It is located in an area of stable, high-density residential properties. This State or Local Air Monitoring Stations (SLAMS) location monitors for CO, NO_2, O_3, PM_{10}, and $PM_{2.5}$. In addition, this is a quality assurance (QA) collocation site for $PM_{2.5}$ where the Maricopa County Air Quality Department (MCAQD) operates one filter-based $PM_{2.5}$ FRM sampler along with one continuous $PM_{2.5}$ FEM analyzer as per 40 CFR Part 58 Appendix A. Resources detailing air quality monitoring QA programs and procedures are detailed on EPA's Ambient Monitoring Technology Information Center website (https://www.epa.gov/amtic/ambient-air-monitoring-quality-assurance, *last accessed 5/11/2022*). Meteorological monitors operating at this site measure ambient temperature (T), barometric pressure, delta T (temperature inversion), and wind speed/direction.

Figure 1: West Phoenix Monitoring Station

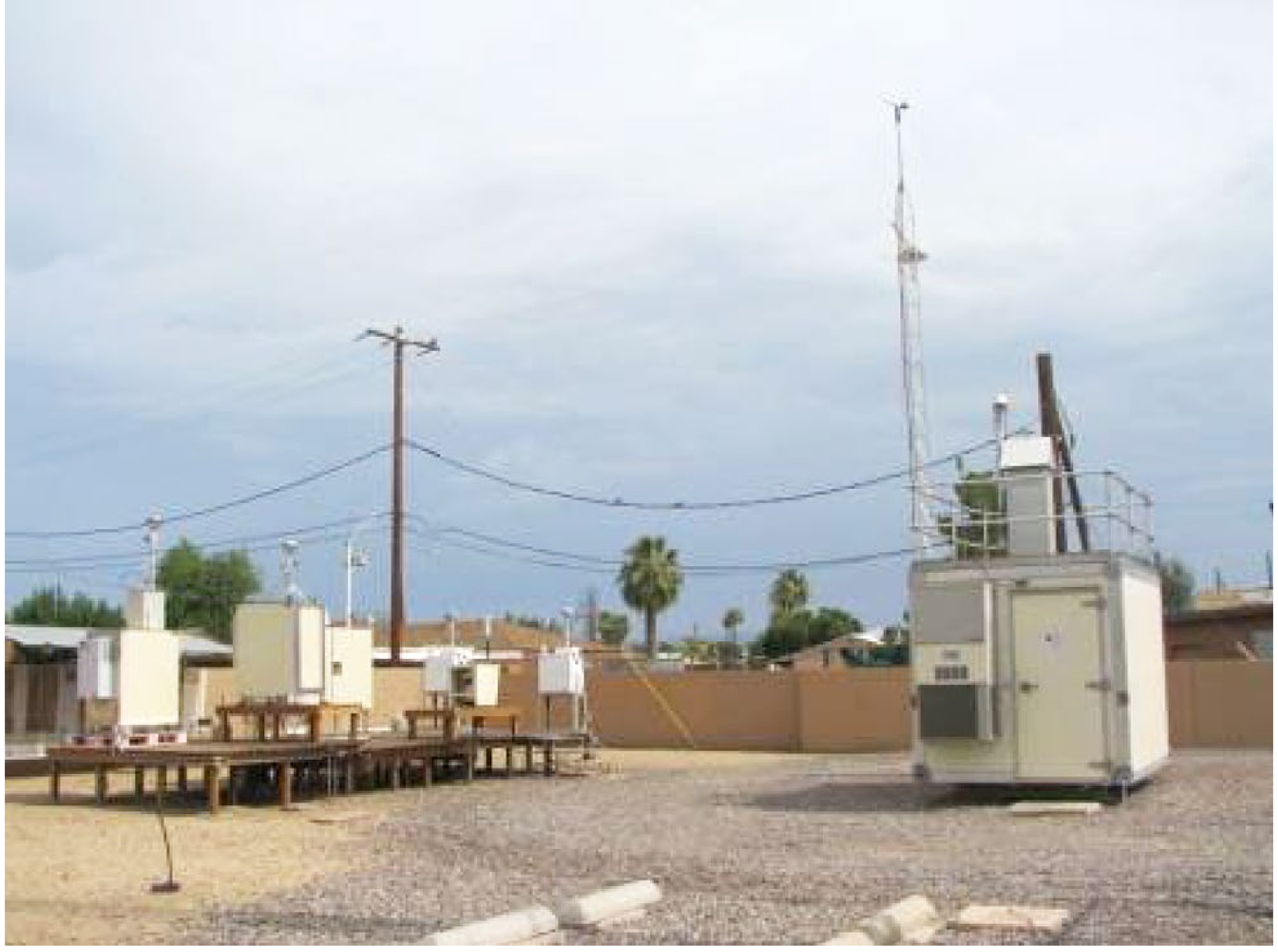

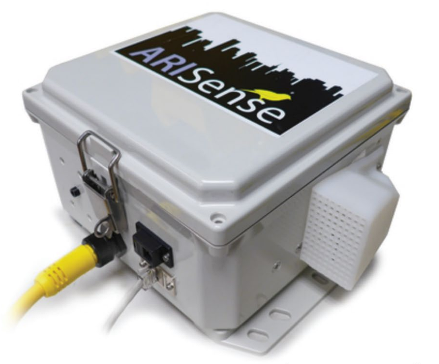

Image Attribution: QuantAQ, Inc (Somerville, MA)

Data Storage and Transmission Method

ARISense units upload data to an external cloud server at 2-minute averages via cellular signal. ARISense weekly data collections were performed by downloading data from the QuantAQ cloud server (last accessed 5/11/22).

Data Correction Approach

QuantAQ ARISense units measure PM2.5 concentrations via an optical particle counter (OPC). The OPC measures particle counts within particle size bins ranging from 0.3 microns to 10 microns, and mass concentrations for each bins are estimated from particle counts assuming constant values for density, spherical morphology, and average particle diameter. The manufacturer applies a general correction to account for the detection limitations of the OPC leading to concentrations that are closer to reality. These presets were configured by the sensor manufacturer and were not modified by EPA prior or during testing.

After acquisition, the raw data was processed using the *sensortoolkit* python code library (v0.8.3b2). A continuous data set at the recorded sampling frequency was written to a .csv file. 1-hour and 24-hour averaged data sets were generated using a 75% completeness threshold and saved as separate .csv files. Outliers were NOT removed from data sets in order to assess "out-of-the-box" sensor performance.

Issues Encountered

Pre-deployment observations

Numerous issues were encountered with the ARISense devices. Although previous models of the ARISense had used AlphaSense OPC-N2 these OPCs were unavailable at the time of manufacture. So instead, components from a particle plus OPC handheld were used.

- *Shipping damage:* On arrival to EPA many of the units were damaged due to the heavy pump breaking free during shipping and shaking around inside of the box breaking much of the plastic scaffolding and, in some cases, components. The manufacturer worked with us to fix these units.
- *Hardware and Firmware issues:* Multiple hardware and firmware issues were experienced that had to be fixed before starting the deployment and multiple manual firmware updates on both the gas sensor microcontrollers and the OPC microcontrollers were completed by researchers. The USB drives were frequently corrupted which required replacement and eventually a hardware update. In addition, the OPC microcontroller and the gas sensor microcontroller often miscommunicated. One firmware version reduced the number of bins reported by the OPC since the manufacturer determined the additional bins were not providing additional accuracy. New PCB boards were sent and installed before the tests could be run as well.
- *Initiation:* There was a high power draw when the cellular connectivity initiated and the pump was turned on. This meant that units occasionally failed to turn on at the same time and it meant these could not be powered by solar.
- *Water intake:* In addition, the OPC pump often drew in water during rainstorms leading the inline filter to saturate and the pump to fail due to overheating. Many pumps had to be replaced throughout the testing.
- *Cellular connectivity:* Some units could not consistently stay connected to the cellular, though other sensors at the same site did not experience issues. The manufacturer could not recreate this issue when they were sent back for service and the problems persisted.
- *Reset:* These units ended up being operated on timers so that they were power cycled every night which would typically get them back online if a software error had been encountered, leading to lower data loss than would have occurred with only weekly checks.

Field observations and sensor data flags are included on the following page

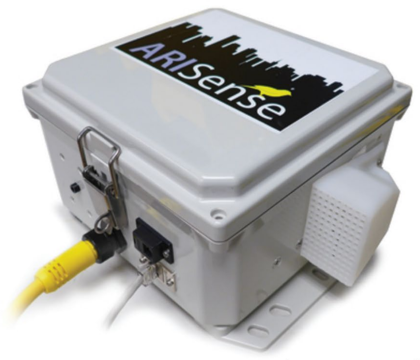

Image Attribution: QuantAQ, Inc (Somerville, MA)

Field observations and sensor data flags

The following table contains data flags describing events that were encountered during the testing period. The QuantAQ ARISense was configured to record data at regular 60-second sampling intervals, however, occasionally the unit recorded irregular sampling intervals ranging from 2-minute intervals to nearly 15 minutes at longest interval duration. The flags below note when these irregular sampling intervals occurred during the testing period. Sampling interval irregularities were not a result of modification to the device firmware configuration and are potentially associated power loading during periods where the ARISense unit initiated a cellular data upload.

Start Time (UTC)	End Time (UTC)	Sensor Serial ID	Parameters Impacted	Flag
2020-02-07 21:04:00+00:00	2020-02-07 21:11:00+00:00	ARS_01	ALL	6-Sampling interval abnormality
2020-02-09 00:59:00+00:00	2020-02-09 01:06:00+00:00	ARS_01	ALL	6-Sampling interval abnormality
2020-02-09 01:30:00+00:00	2020-02-09 01:38:00+00:00	ARS_01	ALL	6-Sampling interval abnormality
2020-02-09 04:02:00+00:00	2020-02-09 04:09:00+00:00	ARS_01	ALL	6-Sampling interval abnormality
2020-02-09 07:24:00+00:00	2020-02-09 07:32:00+00:00	ARS_01	ALL	6-Sampling interval abnormality
2020-02-09 09:56:00+00:00	2020-02-09 10:04:00+00:00	ARS_01	ALL	6-Sampling interval abnormality
2020-02-09 12:08:00+00:00	2020-02-09 12:16:00+00:00	ARS_01	ALL	6-Sampling interval abnormality
2020-02-09 15:11:00+00:00	2020-02-09 15:19:00+00:00	ARS_01	ALL	6-Sampling interval abnormality
2020-02-09 16:33:00+00:00	2020-02-09 16:41:00+00:00	ARS_01	ALL	6-Sampling interval abnormality
2020-02-09 18:15:00+00:00	2020-02-09 18:27:00+00:00	ARS_01	ALL	6-Sampling interval abnormality
2020-02-12 11:51:00+00:00	2020-02-12 11:57:00+00:00	ARS_01	ALL	6-Sampling interval abnormality
2020-02-12 20:36:00+00:00	2020-02-12 20:42:00+00:00	ARS_01	ALL	6-Sampling interval abnormality
2020-02-14 01:26:00+00:00	2020-02-14 01:32:00+00:00	ARS_01	ALL	6-Sampling interval abnormality
2020-02-14 01:36:00+00:00	2020-02-14 01:42:00+00:00	ARS_01	ALL	6-Sampling interval abnormality
2020-02-26 20:20:00+00:00	2020-02-26 20:31:00+00:00	ARS_01	ALL	6-Sampling interval abnormality

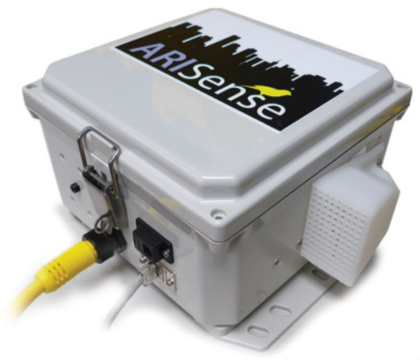

Image Attribution: QuantAQ, Inc (Somerville, MA)

Description of Data Flags

AQS

The U.S. EPA's Air Quality System (AQS) is the Agency's primary ambient air monitoring data archive. A comprehensive list of data flags that are recorded alongside AQS data sets, referred to by U.S. EPA as 'qualifiers', can be found at the following link: https://aqs.epa.gov/aqsweb/documents/codetables/qualifiers.html

AirNow-Tech

AirNow-Tech is an additional ambient air monitoring data service maintained by U.S. EPA and is commonly used by monitoring agencies to upload and validate monitoring data. Data which have yet to be QC'd for inclusion in AQS as well as monitoring data sets which are not planned for inclusion in AQS are typically available for near real-time download from AirNow-Tech.

Invalidation of reference data: AQS qualifiers are organized by qualifier type, which indicates whether data logged alongside qualifier flags should be invalidated (set null). Qualifiers with type "Null Data Qualifier" are invalidated, and includes data logged during periods that coincide with QC checks (e.g., "BF-Precision/Zero/Span", "BJ- Operator Error", "BL - QA Audit", "AZ - QC Audit") among other events such as power outages. Data logged alongside qualifiers with type "Quality Assurance Qualifiers" are not invalidated and are included in this analysis (e.g., concentrations less than the federal MDL for the reference monitor "MD – Value less than MDL", QA reviewed values "Validated Value").

Data Flags Recorded During Testing

FRM/FEM Monitor	Timestamp (UTC)	Flag
Thermo Fisher 1405-DF TEOM FDMS Dichotomous FEM (Data acquired via AQS)	2020-02-11 15:00:00+0000 to 2020-02-11 18:00:00+0000	AY - QC Control Points (zero/span)
	2020-02-18 17:00:00+0000 to 2020-02-18 18:00:00+0000	BM - Accuracy check
	2020-02-25 14:00:00+0000 to 2020-02-25 21:00:00+0000	AY - QC Control Points (zero/span)
	2020-02-25 15:00:00+0000 to 2020-02-25 18:00:00+0000	BA - Maintenance/Routine Repairs
	2020-03-03 17:00:00+0000	BL - QA Audit
	2020-03-03 18:00:00+0000 to 2020-03-03 19:00:00+0000	BM - Accuracy check
	2020-03-06 14:00:00+0000 to 2020-03-06 15:00:00+0000	AY - QC Control Points (zero/span)

Meteorological Instrument	Timestamp (UTC)	Flag
RM Young Relative Humidity Monitor (Data acquired via AirNow-Tech)	2020-02-25 16:00:00+0000 to 2020-02-25 17:00:00+0000	9 – Invalid

Testing Report - $PM_{2.5}$ Base Testing

Aeroqual AQY

This report reflects out-of-the-box performance

Initial Base Testing - Denver, CO

U.S. Environmental Protection Agency
Office of Research and Development
PI: Clements.Andrea@epa.gov
919-541-1363

August 2019—September 2019

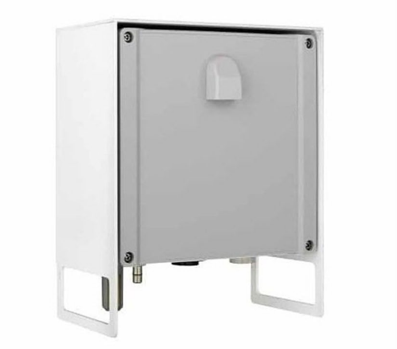

Image Attribution: Aeroqual Limited (Auckland, New Zealand)

Deployment Details

Testing Organization and Site Information	
Testing organization (Name, Organization type, Contact website)	U.S. Environmental Protection Agency - Office of Research and Development<br>Federal Government<br>Air Sensor Toolbox | U.S. EPA Website
Testing location (City, State, Latitude and Longitude)	La Casa<br>Denver, CO<br>39.779429, -105.005174
AQS site ID	08 - 031 - 0026
Sampling timeframe (MM-DD-YY)	08-17-19 to 09-16-19
Sensor data source	Aeroqual Cloud download
Reference data source	AQS API download

Sensor Information			
Manufacturer, model	Aeroqual AQY		
Device firmware version	1.14.2		
Sampling time interval	1-minute		
Sensor serial numbers	AQ_01		
Issues encountered during deployment?	☐	No Issues	

FRM/FEM Information	
Manufacturer, model, designation	Teledyne Advanced Pollution Instrumentation T640 FEM
Sampling time interval	1-hour averaging
Date of calibration	As required by 40 CFR Part 58 and the CO Regional Monitoring Site QAPP maintained by CDPHE
Date of flowrate verification check	Monthly as required by 40 CFR Part 58 Appendix A
Description, date(s) of maintenance activities	See CO-AQY-Page 7 of this testing report

Time Series Plots

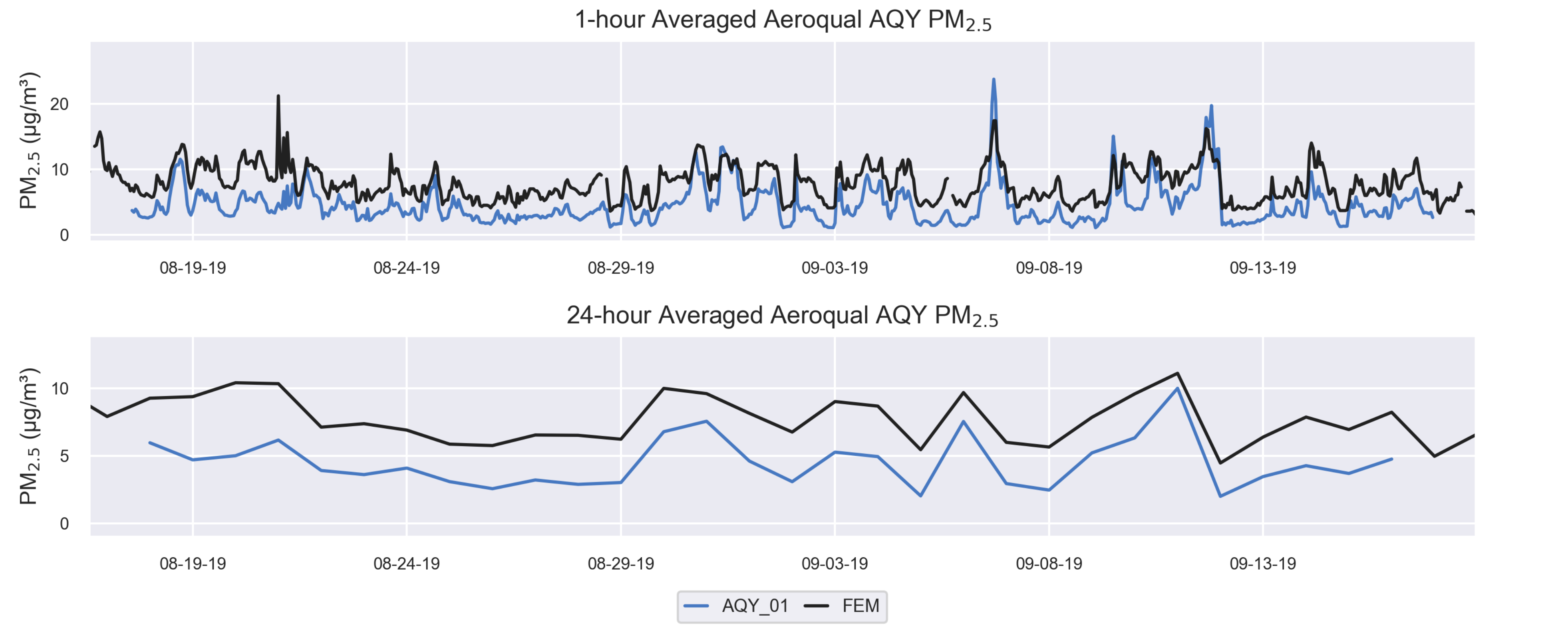

Scatter Plots: Comparison to FRM/FEM

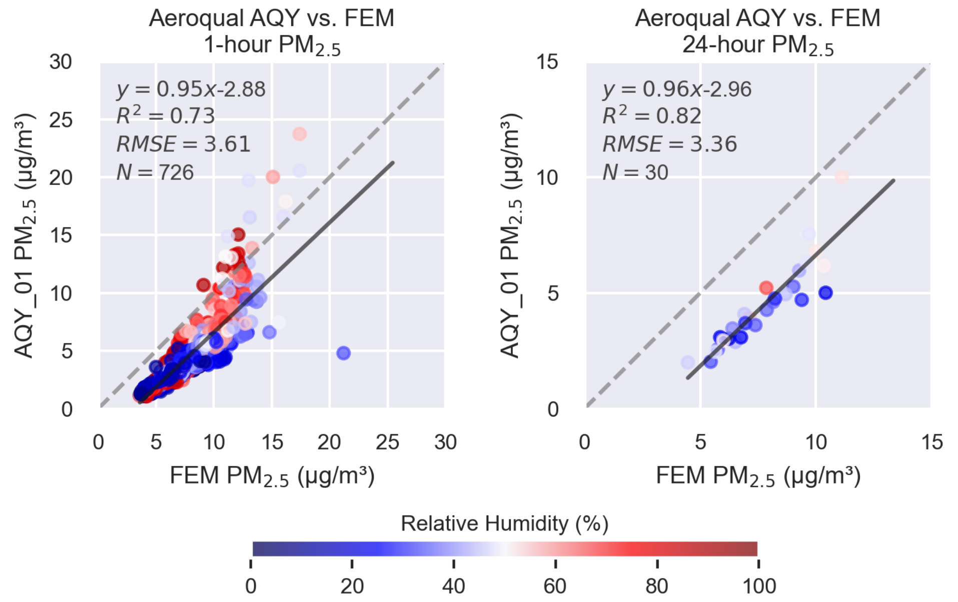

Range of FRM/FEM concentrations over duration of base test (µg/m³)	3.6-21.2 (1-hr), 4.5-11.1 (24-hr)
Number of 24-hr periods in FRM/FEM monitor measurements with a goal concentration ≥ 25 µg/m³	0

Performance Metrics

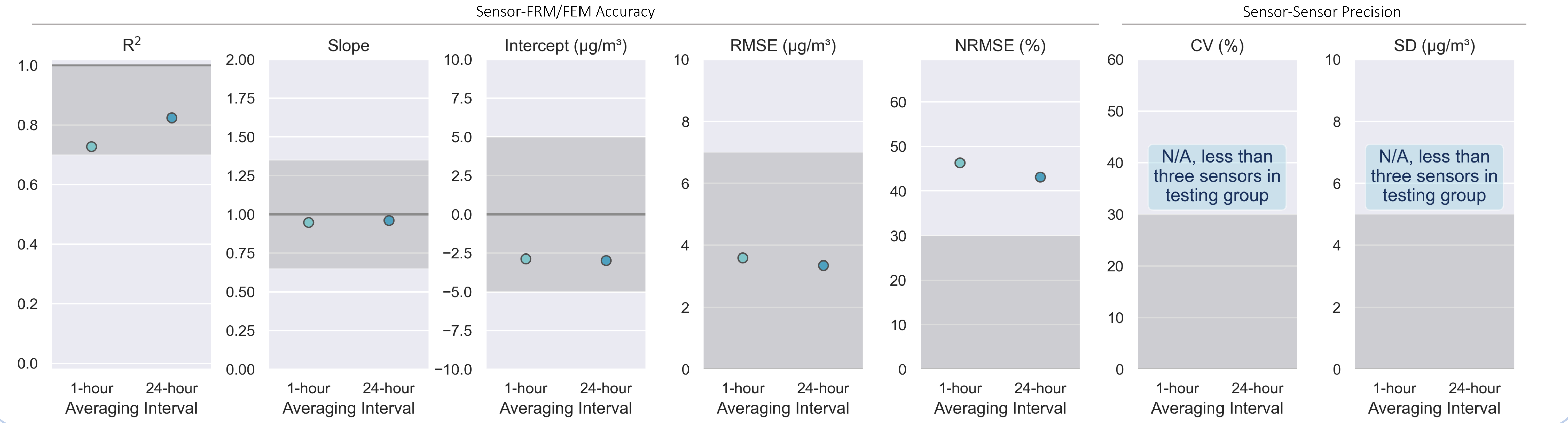

Meteorological Conditions During Deployment

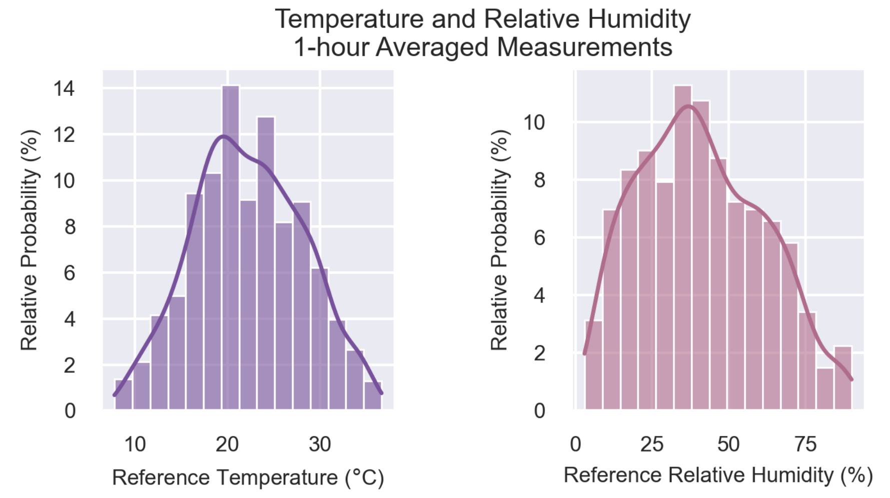

Number of 24-hr periods outside sensor manufacturer-listed temperature operational range (-10 to 40 °C)	0
Number of 24-hr periods outside sensor manufacturer-listed relative humidity operational range (no operational range specified)	-

Meteorological Influence

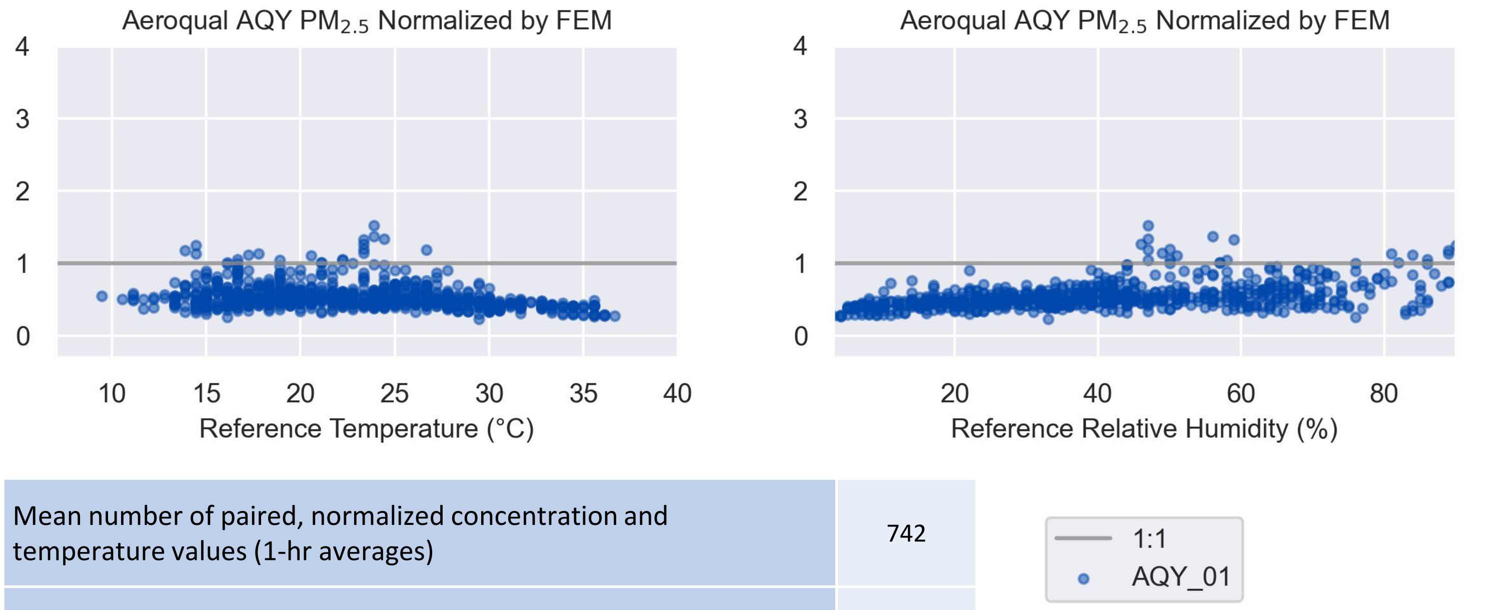

Mean number of paired, normalized concentration and temperature values (1-hr averages)	742
Mean number of paired, normalized concentration and relative humidity values (1-hr averages)	742

Testing Report - $PM_{2.5}$ Base Testing

Aeroqual AQY

This report reflects out-of-the-box performance

Initial Base Testing - Denver, CO

U.S. Environmental Protection Agency
Office of Research and Development
PI: Clements.Andrea@epa.gov
919-541-1363

August 2019—September 2019

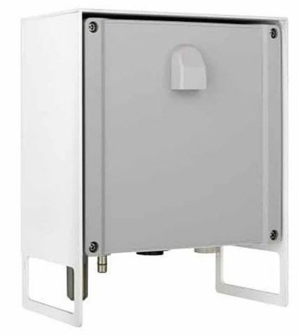

Image Attribution: Aeroqual Limited (Auckland, New Zealand)

Tabular Statistics

Sensor-FRM/FEM Correlation

	Bias and Linearity						Data Quality			
	R^2		Slope		Intercept (µg/m³)		Uptime (%)		Number of paired sensor and FRM/FEM concentration values	
	1-Hour ●	24-Hour ●	1-Hour ●	24-Hour ●	1-Hour ●	24-Hour ●	1-Hour ●	24-Hour ●	1-Hour	24-Hour
Metric Target Range	≥ 0.70	≥ 0.70	1.0 ± 0.35	1.0 ± 0.35	-5 ≤ b ≤ 5	-5 ≤ b ≤ 5	75%*	75%*	-	-
Sensor AQY_01	0.73	0.82	0.95	0.96	-2.88	-2.96	100	100	726	30

	Error			
	RMSE (µg/m³)		NRMSE (%)	
	1-Hour ★	24-Hour ★	1-Hour ☆	24-Hour ☆
Metric Target Range	≤ 7.0	≤ 7.0	≤ 30.0	≤ 30.0
Deployment Value	3.6	3.4	46.4	43.2

Device-specific metrics (computed for each sensor in evaluation)
○○○ Metric value for none of devices tested falls within the target range
●○○ Metric value for one of devices tested falls within the target range
●●○ Metric value for two of devices tested falls within the target range
●●● Metric value for three of devices tested falls within the target range

Single-valued metrics (computed via entire evaluation dataset)
☆ Indicates that the metric value is not within the target range
★ Indicates that the metric value is within the target range

Sensor-Sensor Precision[1]

	Precision (between collocated sensors)				Data Quality	
	CV (%)		SD (µg/m³)		Number of concurrent sensor concentration pairs	
	1-Hour ☆	24-Hour ☆	1-Hour ☆	24-Hour ☆	1-Hour	24-Hour
Metric Target Range	≤ 30.0	≤ 30.0	≤ 5.0	≤ 5.0	-	-
Deployment Value	-	-	-	-	-	-

[1]Precision statistics are computed for evaluations with at least three collocated sensor units. Metric values are left blank for evaluations with two or fewer sensor units.

*This value is only a recommendation for ensuring data quality and is not included in the list of target values discussed in Section 4 of the Performance Testing Protocols, Metrics, and Target Values for Fine Particulate Matter Air Sensors document.

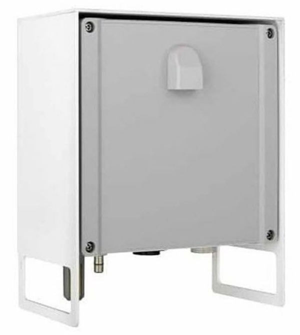

Image Attribution: Aeroqual Limited (Auckland, New Zealand)

Sensor-FRM/FEM Scatter Plots

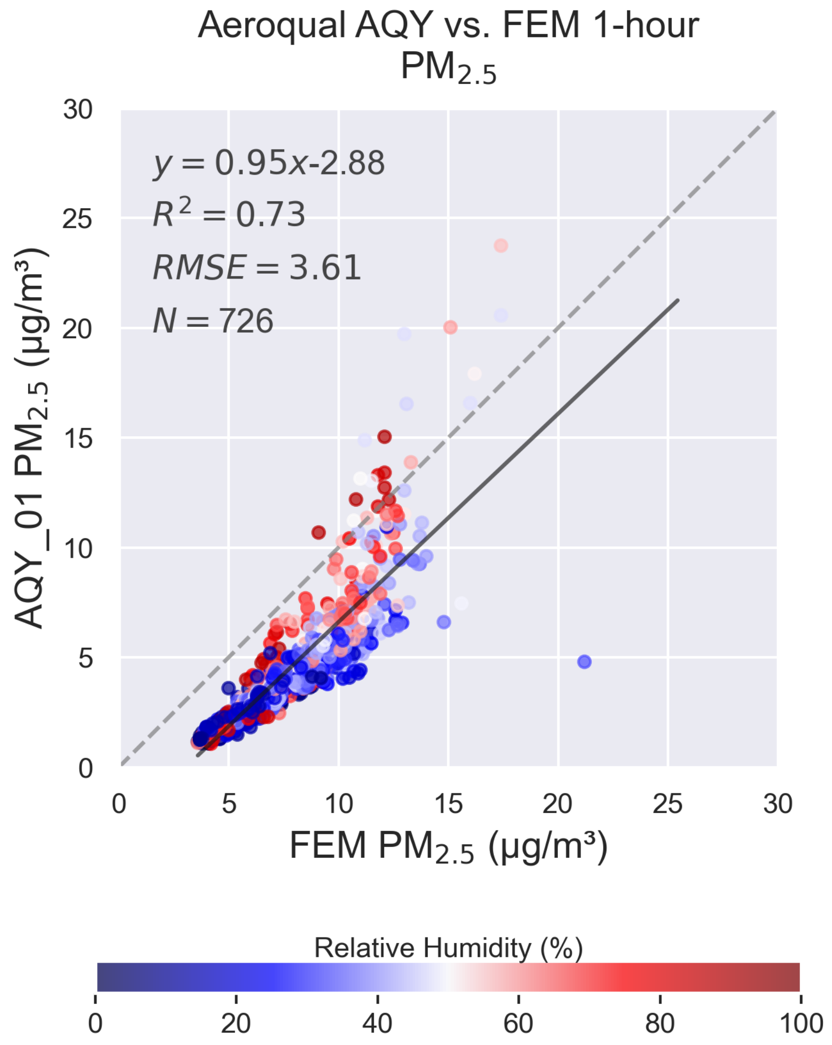

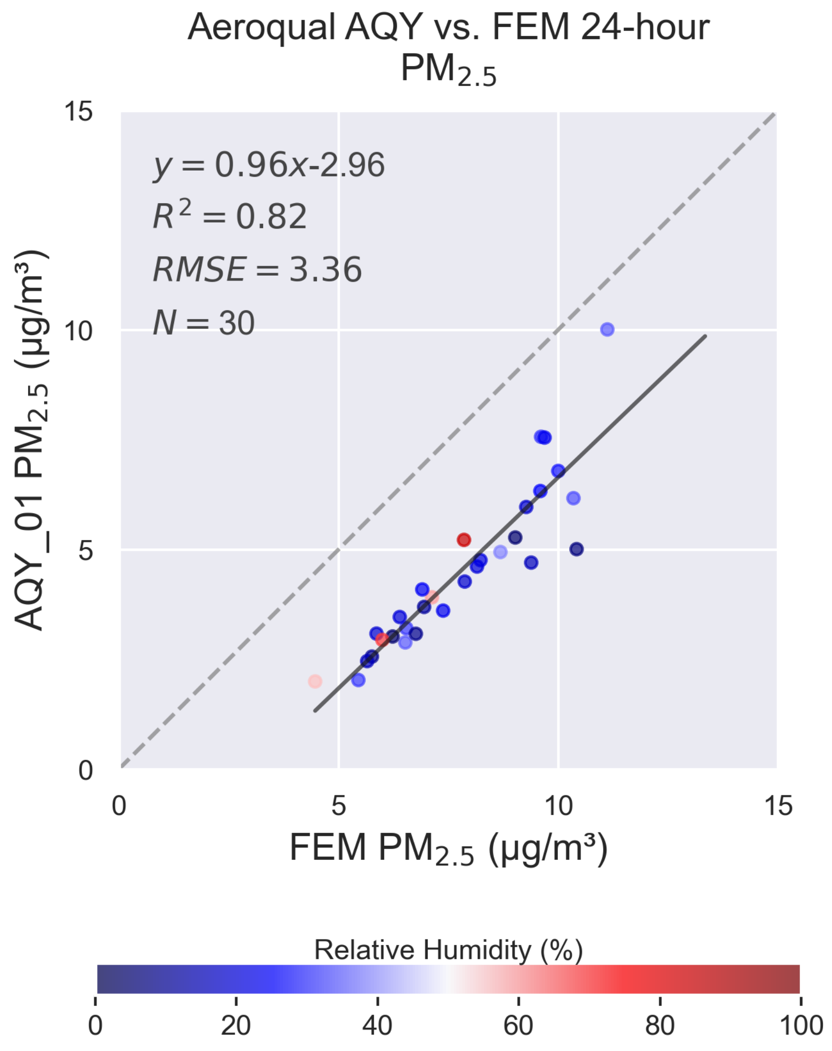

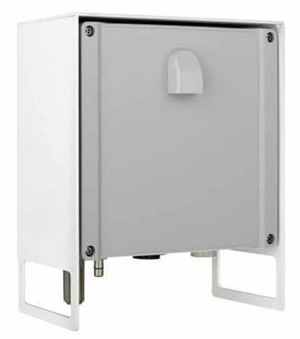

Image Attribution: Aeroqual Limited (Auckland, New Zealand)

Supplemental Information

Abbreviations used in Supplemental Information

FRM	Federal Reference Method
FEM	Federal Equivalent Method
SOP	Standard Operating Procedure
QAPP	Quality Assurance Project Plan
QC	Quality Control

Supplemental Documentation	Attached	Description & URL or file path to documentation
Field observations and sensor data flags	☑	See CO-AQY-Page 6 of this testing report
Maintenance logs	☐	No logs recorded during testing
Standard operating procedure(s)	☐	U.S. EPA Office Of Research and Development SOP available upon request
Photos of equipment setup and testing	☑	See CO-AQY-Page 5 of this testing report
Product specifications sheet(s)	☐	N/A*
Product manual(s)	☐	N/A*
Data storage and transmission method	☑	See CO-AQY-Page 6 of this testing report
Data correction approach	☑	See CO-AQY-Page 6 of this testing report
Issues encountered	☑	See CO-AQY-Page 6 of this testing report. No issues were encountered during testing; however, various issues were faced during the pre-deployment phase.
Data analysis/correction scripts and version	☑	Averaging and processing of data, calculation of performance metrics, and generation of figures and other supplementary material for analysis were obtained using Python 3.9.7 with the packages sensortoolkit v0.8.3b2, pandas 1.3.5, NumPy 1.21.2, Matplotlib 3.5.0, statsmodels 0.13.0, and seaborn 0.11.2. All packages are available from the Python Package Index (PyPI) at https://pypi.org. The integrated development environment (IDE) Spyder 5.1.5 was used for scripting and data visualization. Version control for the Python base, packages, and IDE were all managed by conda 4.11.0.
Air Monitoring Station QAPP	☐	U.S. EPA Office Of Research and Development QAPP available upon request
Summary of FRM/FEM monitor QC checks	☑	See CO-AQY-Page 7 of this testing report
Manufacturer website for FRM/FEM monitor	☑	Teledyne API: T640/T640X Product website
FRM/FEM monitor manual	☑	Teledyne API: T640/T640X Product Manual
FRM/FEM monitor specifications sheet(s)	☑	Teledyne API: T640/T640X Specification Sheet
Other documents	☑	Manufacturer notice of AQY sales on hold

*As of 3/18/2021, the manufacturer of the AQY has placed sales of a similar unit on hold. Documentation for the AQY is currently unavailable from the manufacturer's website.

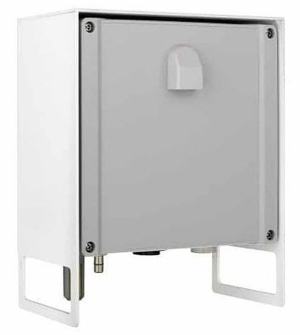

Image Attribution: Aeroqual Limited (Auckland, New Zealand)

Site Description:

The La Casa site was established in January of 2013 as a replacement for the Denver Municipal Animal Shelter (DMAS) site when a land use change forced the relocation of the site. The La Casa location has been established as the NCore site for the Denver Metropolitan area. Measurements include trace gas/precursor-level CO analyzer, and a NO_y analyzer, in addition to the trace level SO_2, O_3, meteorology, and particulate monitors. The site represents a population-oriented neighborhood scale monitoring area.

Figure 1: Aeroqual AQY sensor (indicated by red arrow) attached to metal railing atop the sampling shelter at the monitoring site.

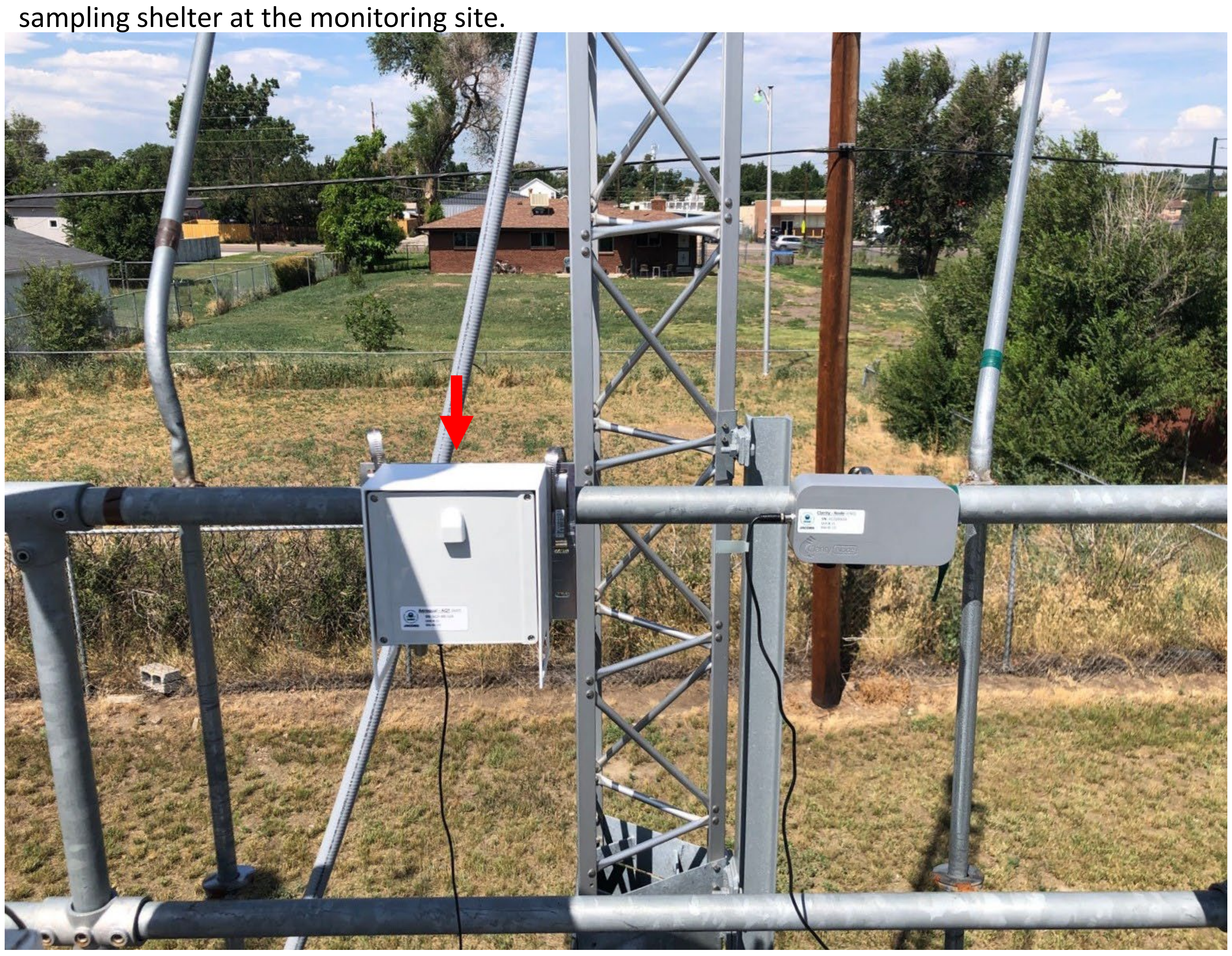

Figure 2: La Casa Monitoring Station sampling shelter, side view

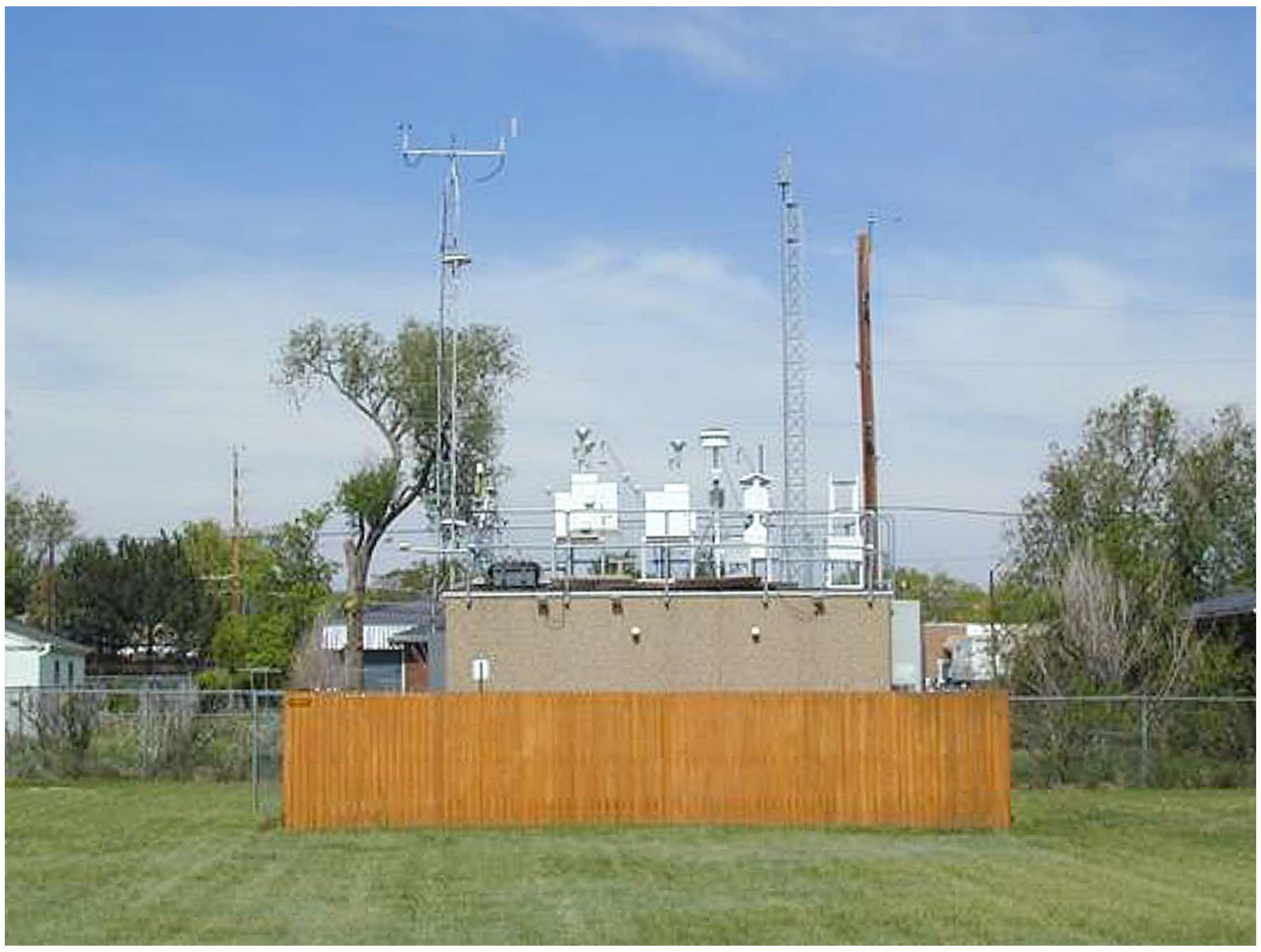

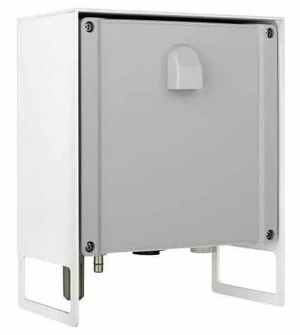

Image Attribution: Aeroqual Limited (Auckland, New Zealand)

Supplemental Information: Data Storage, Correction Approach, and Issues Encountered

Data Storage and Transmission Method

As part of CRADA #934-16 between Aeroqual and US EPA, Aeroqual supported data streaming. SIM cards were installed and data flowed to the Aeroqual Cloud. The 1-minute raw data was acquired weekly using the Aeroqual Cloud (*last accessed 5/11/22*) user interface (UI). The AQY has an internal data storage USB flash drive as a data backup, however the software on the drive is proprietary to Aeroqual and unreadable by outside systems.

Data Correction Approach

This evaluation report reflects "out-of-the-box" performance of the AQY. The manufacturer provides a procedure by which local collocation (sensor operated along side an FRM/FEM) data can be collected, a gain (slope) and offset (intercept) determined, and parameters entered into the Aeroqual Cloud user interface to be applied to all subsequently collected data. This procedure and feature was NOT used prior to this evaluation. Prospective consumers may get different performance from this device if they utilize this feature.

After acquisition, the raw data was processed using the *sensortoolkit* python code library (v0.8.3b2). A continuous data set at the recorded sampling frequency was written to a .csv file. 1-hour and 24-hour averaged data sets were generated using a 75% completeness threshold and saved as separate .csv files. Outliers were NOT removed from data sets in order to assess "out-of-the-box" sensor performance.

Issues Encountered

Pre-deployment observations

- *Timestamp inaccuracies*: During pre-deployment, the AQY devices did not properly sync timestamps with the onboard Real-Time Clock. Connecting the units to the internet by cellular or Wi-Fi allowed the unit to sync with internet time and resulted in proper timestamps.

Field observations and sensor data flags

The Aeroqual AQY was deployed at the La Casa monitoring site on 7/31/2019. The Aeroqual unit operated nominally during the testing period and did not require replacement or repair.

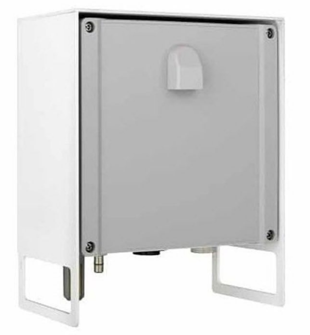
Image Attribution: Aeroqual Limited (Auckland, New Zealand)

Description of Data Flags

AQS

The U.S. EPA's Air Quality System (AQS) is the Agency's primary ambient air monitoring data archive. A comprehensive list of data flags that are recorded alongside AQS data sets, referred to by U.S. EPA as 'qualifiers', can be found at the following link: https://aqs.epa.gov/aqsweb/documents/codetables/qualifiers.html

Invalidation of reference data: AQS qualifiers are organized by qualifier type, which indicates whether data logged alongside qualifier flags should be invalidated (set null). Qualifiers with type "Null Data Qualifier" are invalidated, and includes data logged during periods that coincide with QC checks (e.g., "BF-Precision/Zero/Span", "BJ- Operator Error", "BL - QA Audit", "AZ - QC Audit") among other events such as power outages. Data logged alongside qualifiers with type "Quality Assurance Qualifiers" are not invalidated and are included in this analysis (e.g., concentrations less than the federal MDL for the reference monitor "MD – Value less than MDL", QA reviewed values "Validated Value").

Data Flags Recorded During Testing

FRM/FEM Monitor	Timestamp (UTC)	Flag
Teledyne API T640 (Acquired via AQS)	2019-08-28 14:00:00+0000 to 2019-08-28 15:00:00+0000	BA - Maintenance/Routine Repairs
	2019-09-05 16:00:00+0000 to 2019-09-05 17:00:00+0000	BL - QA Audit

Meteorological Instrument	Timestamp (UTC)	Flag
MetOne Temperature Monitor (Acquired via AQS)	2019-09-13 17:00:00+0000 to 2019-09-13 18:00:00+0000	AZ - QC Audit
Climatronics Relative Humidity Monitor (Acquired via AQS)	2019-09-13 17:00:00+0000 to 2019-09-13 18:00:00+0000	AZ - QC Audit

Testing Report - $PM_{2.5}$ Base Testing

APT Maxima

This report reflects out-of-the-box performance

Initial Base Testing - Denver, CO
U.S. Environmental Protection Agency
Office of Research and Development
PI: Clements.Andrea@epa.gov
919-541-1363
September 2019—October 2019

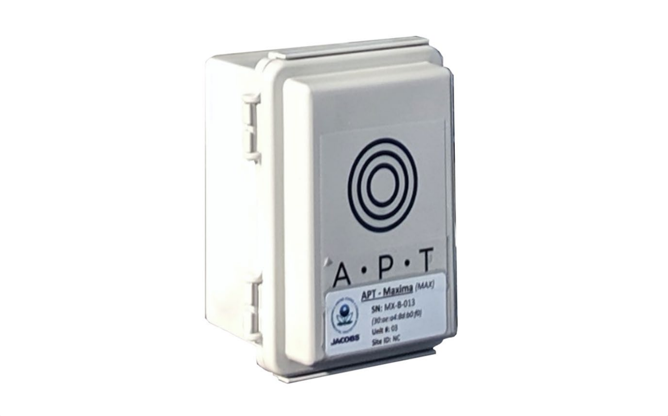

Deployment Details

Testing Organization and Site Information	
Testing organization (Name, Organization type, Contact website)	U.S. Environmental Protection Agency - Office of Research and Development<br>Federal Government<br>Air Sensor Toolbox | U.S. EPA Website
Testing location (City, State, Latitude and Longitude)	La Casa<br>Denver, CO<br>39.779429, -105.005174
AQS site ID	08 - 031 - 0026
Sampling timeframe (MM-DD-YY)	09-12-19 to 10-12-19
Sensor data source	Onboard MicroSD card
Reference data source	AQS API download

Sensor Information			
Manufacturer, model	APT Maxima		
Device firmware version	v5.0 (installed 5/2/2019)		
Sampling time interval	30-seconds		
Sensor serial numbers	MAX_02		
Issues encountered during deployment?	☐	No Issues	

FRM/FEM Information	
Manufacturer, model, designation	Teledyne Advanced Pollution Instrumentation T640 FEM
Sampling time interval	1-hour averaging
Date of calibration	As required by 40 CFR Part 58 and the CO Regional Monitoring Site QAPP maintained by CDPHE
Date of flowrate verification check	Monthly as required by 40 CFR Part 58 Appendix A
Description, date(s) of maintenance activities	See CO-MAX-Page 7 of this testing report

Time Series Plots

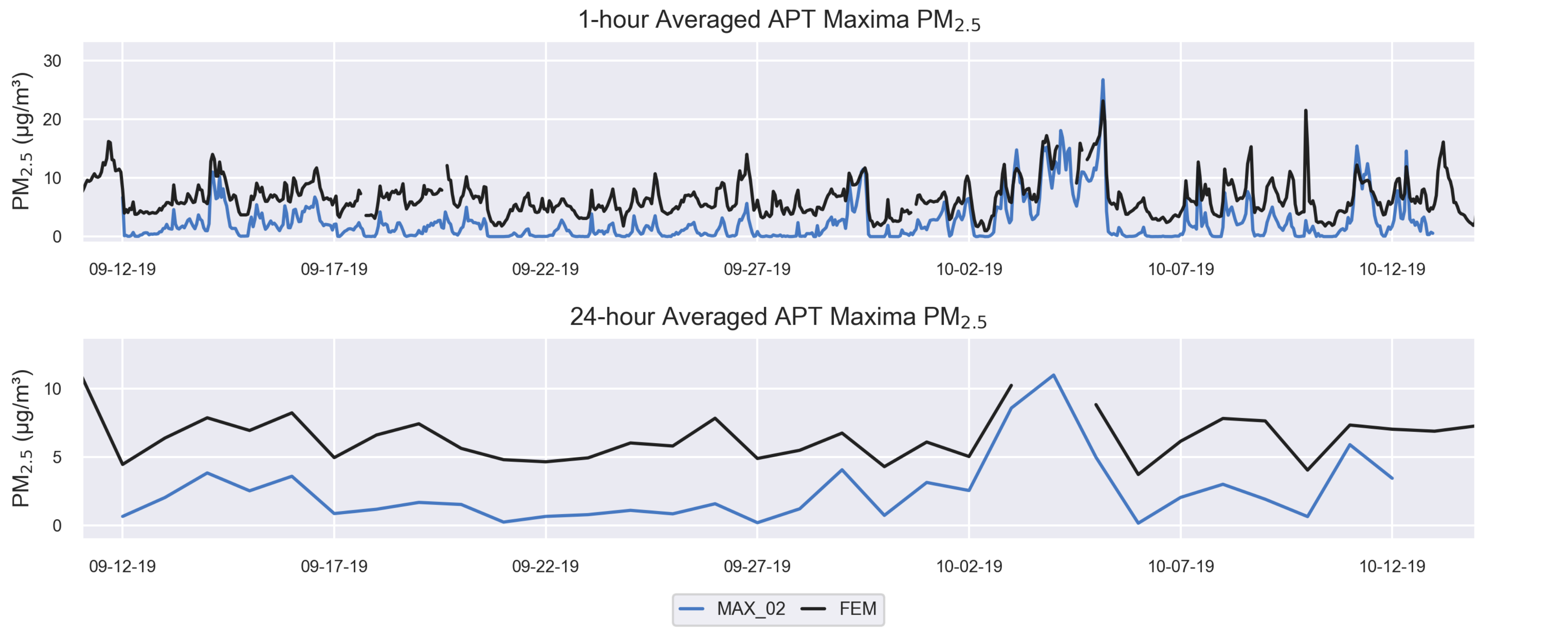

Scatter Plots: Comparison to FRM/FEM

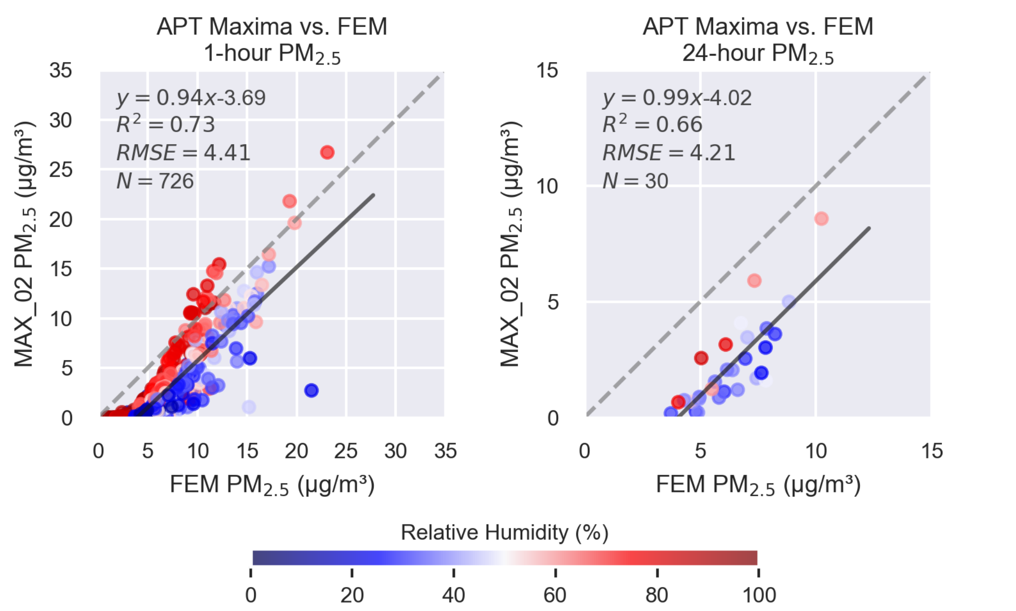

Range of FRM/FEM concentrations over duration of base test (µg/m³)	1.0-23.1 (1-hr), 3.7-10.2 (24-hr)
Number of 24-hr periods in FRM/FEM monitor measurements with a goal concentration ≥ 25 µg/m³	0

Performance Metrics

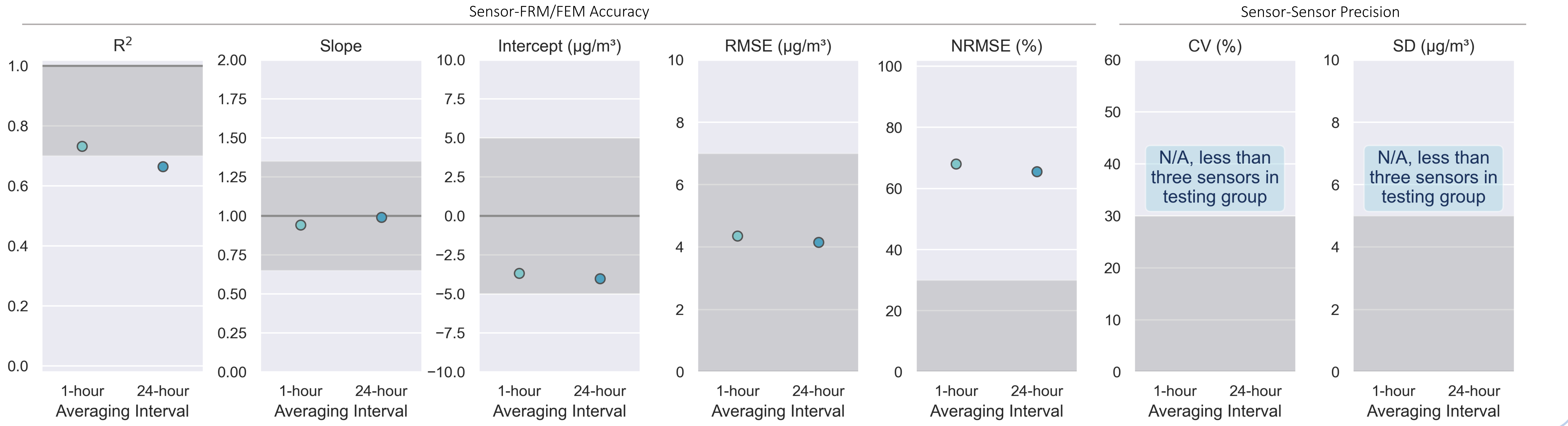

Meteorological Conditions During Deployment

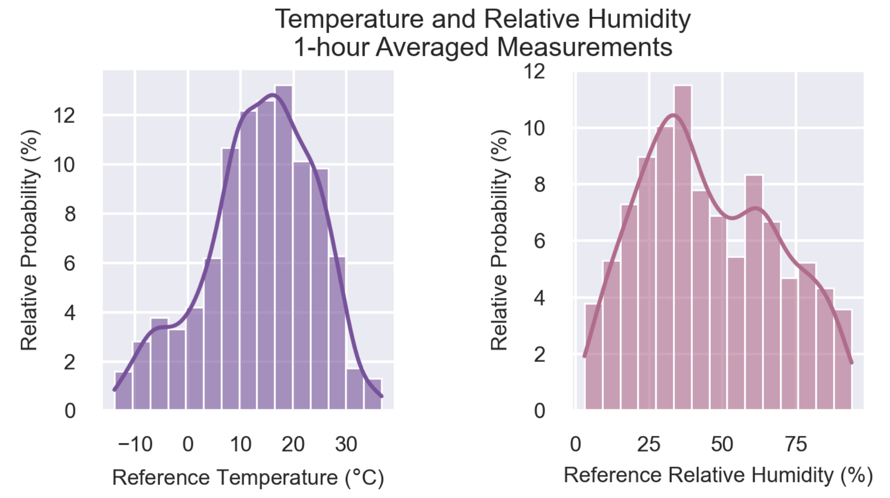

Number of 24-hr periods outside sensor manufacture-listed temperature operational range (no operational range specified)	-
Number of 24-hr periods outside sensor manufacture-listed relative humidity operational range (no operational range specified)	-

Meteorological Influence

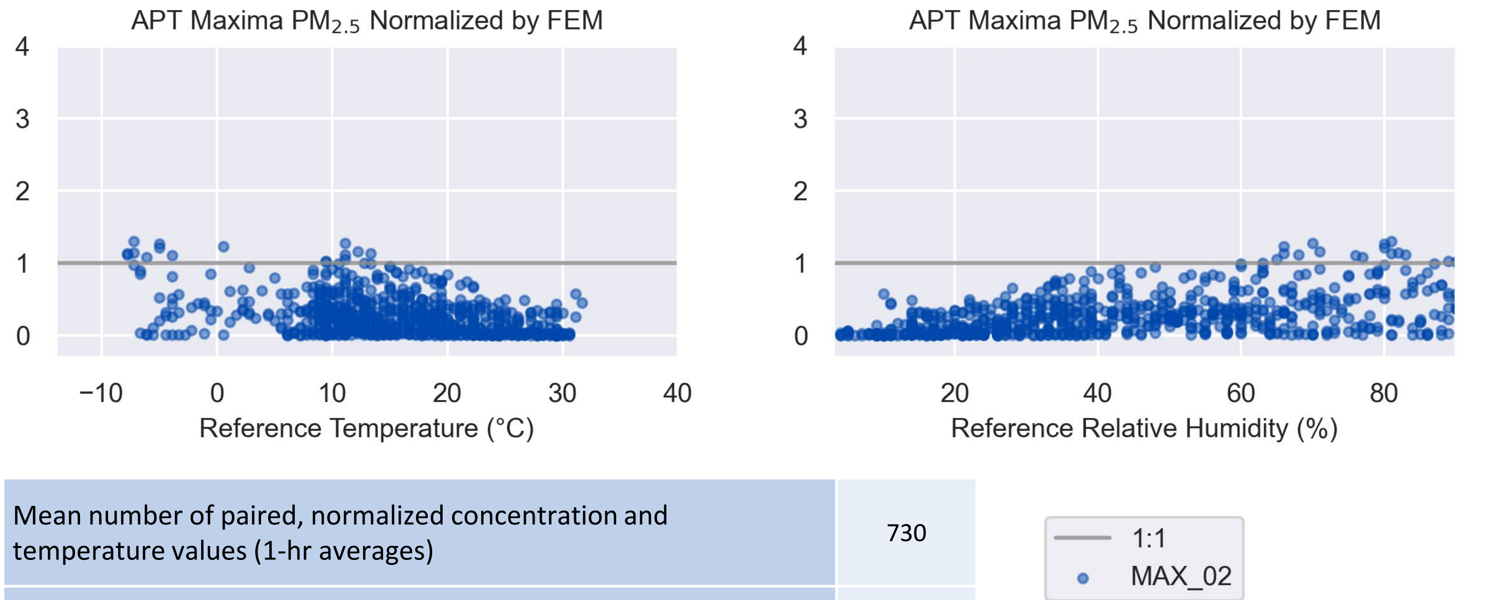

Mean number of paired, normalized concentration and temperature values (1-hr averages)	730
Mean number of paired, normalized concentration and relative humidity values (1-hr averages)	730

Testing Report - $PM_{2.5}$ Base Testing

APT Maxima

This report reflects out-of-the-box performance

Initial Base Testing - Denver, CO
U.S. Environmental Protection Agency
Office of Research and Development
PI: Clements.Andrea@epa.gov
919-541-1363
September 2019—October 2019

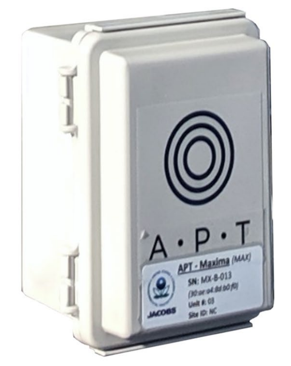

Tabular Statistics

Sensor-FRM/FEM Correlation

	Bias and Linearity						Data Quality			
	R^2		Slope		Intercept (µg/m³)		Uptime (%)		Number of paired sensor and FRM/FEM concentration values	
	1-Hour ●	24-Hour ○	1-Hour ●	24-Hour ●	1-Hour ●	24-Hour ●	1-Hour ●	24-Hour ●	1-Hour	24-Hour
Metric Target Range	≥ 0.70	≥ 0.70	1.0 ± 0.35	1.0 ± 0.35	-5 ≤ b ≤ 5	-5 ≤ b ≤ 5	75%*	75%*	-	-
Sensor MAX_02	0.73	0.66	0.94	0.99	-3.69	-4.02	100	100	726	30

	Error			
	RMSE (µg/m³)		NRMSE (%)	
	1-Hour ★	24-Hour ★	1-Hour ☆	24-Hour ☆
Metric Target Range	≤ 7.0	≤ 7.0	≤ 30.0	≤ 30.0
Deployment Value	4.4	4.2	68.0	65.5

Device-specific metrics (computed for each sensor in evaluation)
○○○ Metric value for none of devices tested falls within the target range
●○○ Metric value for one of devices tested falls within the target range
●●○ Metric value for two of devices tested falls within the target range
●●● Metric value for three of devices tested falls within the target range

Single-valued metrics (computed via entire evaluation dataset)
☆ Indicates that the metric value is not within the target range
★ Indicates that the metric value is within the target range

Sensor-Sensor Precision[1]

	Precision (between collocated sensors)				Data Quality	
	CV (%)		SD (µg/m³)		Number of concurrent sensor concentration pairs	
	1-Hour ☆	24-Hour ☆	1-Hour ☆	24-Hour ☆	1-Hour	24-Hour
Metric Target Range	≤ 30.0	≤ 30.0	≤ 5.0	≤ 5.0	-	-
Deployment Value	-	-	-	-	-	-

[1]Precision statistics are computed for evaluations with at least three collocated sensor units. Metric values are left blank for evaluations with two or fewer sensor units.

*This value is only a recommendation for ensuring data quality and is not included in the list of target values discussed in Section 4 of the Performance Testing Protocols, Metrics, and Target Values for Fine Particulate Matter Air Sensors document.

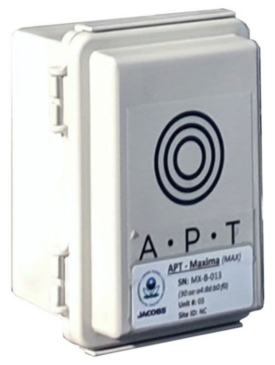

Sensor-FRM/FEM Scatter Plots

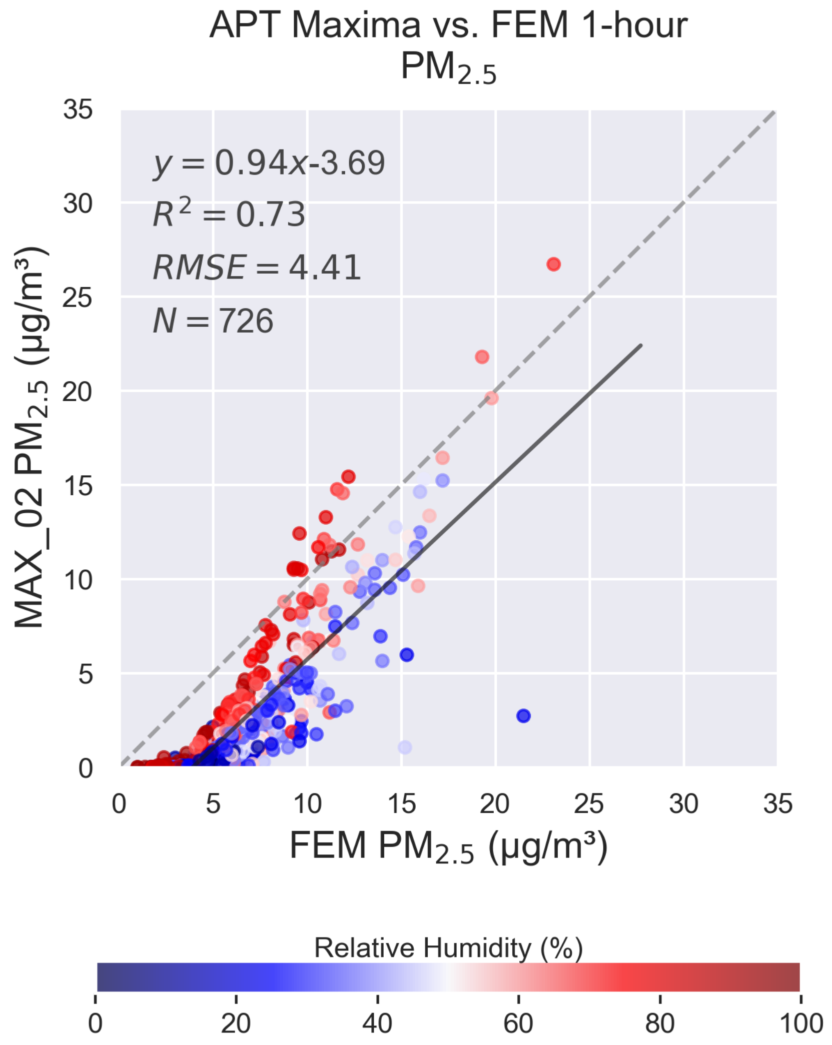

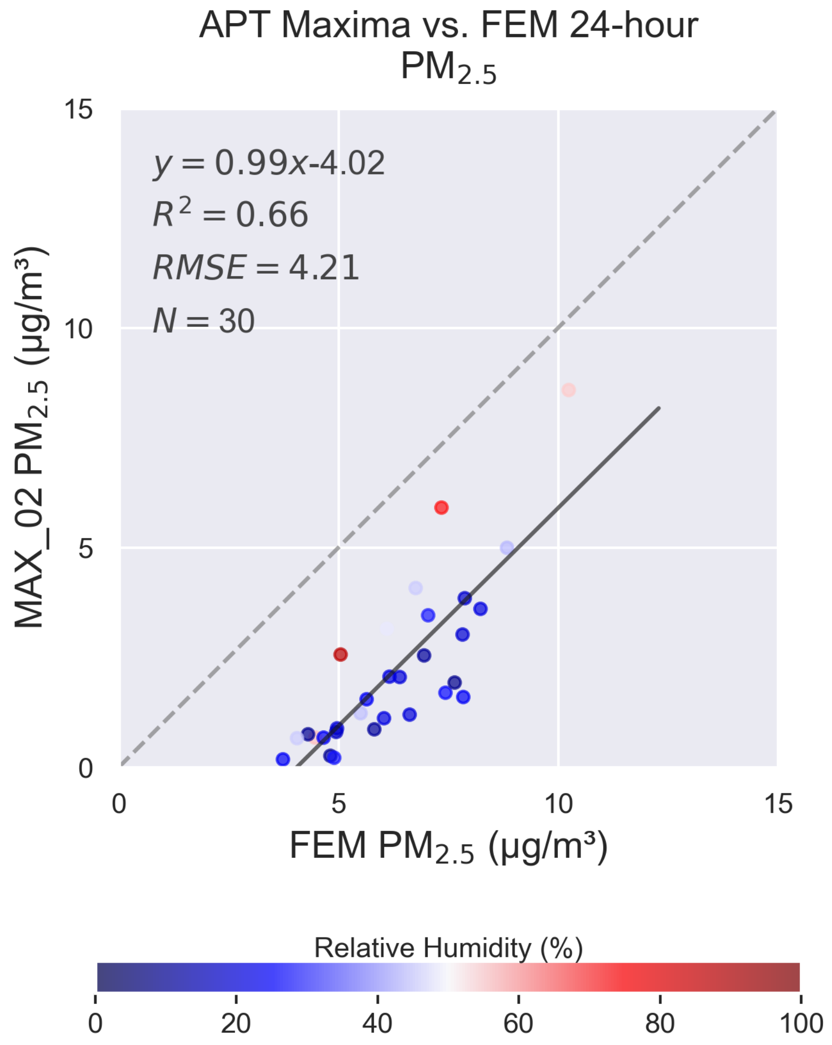

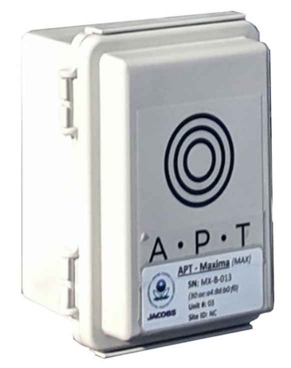

Supplemental Information

Abbreviations used in Supplemental Information

FRM	Federal Reference Method
FEM	Federal Equivalent Method
SOP	Standard Operating Procedure
QAPP	Quality Assurance Project Plan
QC	Quality Control

Supplemental Documentation	Attached	Description & URL or file path to documentation
Field observations and sensor data flags	☑	See CO-MAX-Page 6 of this testing report
Maintenance logs	☐	No logs recorded during testing
Standard operating procedure(s)	☐	U.S. EPA Office Of Research and Development SOP available upon request
Photos of equipment setup and testing	☑	See CO-MAX-Page 5 of this testing report
Product specifications sheet(s)	☐	N/A
Product manual(s)	☐	N/A
Data storage and transmission method	☑	See CO-MAX-Page 6 of this testing report
Data correction approach	☑	See CO-MAX-Page 6 of this testing report
Issues encountered	☑	See CO-MAX-Page 6 of this testing report. No issues were encountered during testing; however, multiple observations were logged during the pre-deployment phase.
Data analysis/correction scripts and version	☑	Averaging and processing of data, calculation of performance metrics, and generation of figures and other supplementary material for analysis were obtained using Python 3.9.7 with the packages sensortoolkit v0.8.3b2, pandas 1.3.5, NumPy 1.21.2, Matplotlib 3.5.0, statsmodels 0.13.0, and seaborn 0.11.2. All packages are available from the Python Package Index (PyPI) at https://pypi.org. The integrated development environment (IDE) Spyder 5.1.5 was used for scripting and data visualization. Version control for the Python base, packages, and IDE were all managed by conda 4.11.0.
Air Monitoring Station QAPP	☐	U.S. EPA Office Of Research and Development QAPP available upon request
Summary of FRM/FEM monitor QC checks	☑	See CO-MAX-Page 7 of this testing report
Manufacturer website for FRM/FEM monitor	☑	Teledyne API: T640 Product website
FRM/FEM monitor manual	☑	Teledyne API: T640 Product Manual
FRM/FEM monitor specifications sheet(s)	☑	Teledyne API: T640 Specification Sheet
Other documents	☐	

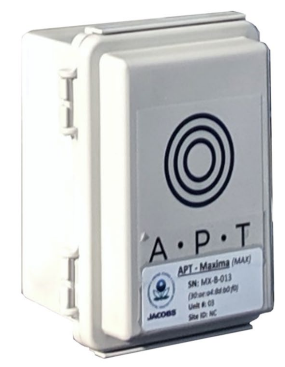

Supplemental Information: Photos of Testing Site and Equipment Setup

Site Description:

The La Casa site was established in January of 2013 as a replacement for the Denver Municipal Animal Shelter (DMAS) site when a land use change forced the relocation of the site. The La Casa location has been established as the NCore site for the Denver Metropolitan area. Measurements include trace gas/precursor-level CO analyzer, and a NO_y analyzer, in addition to the trace level SO_2, O_3, meteorology, and particulate monitors. The site represents a population-oriented neighborhood scale monitoring area.

Figure 1: APT Maxima sensor (indicated by red arrow) attached to metal railing atop the sampling shelter at the monitoring site.

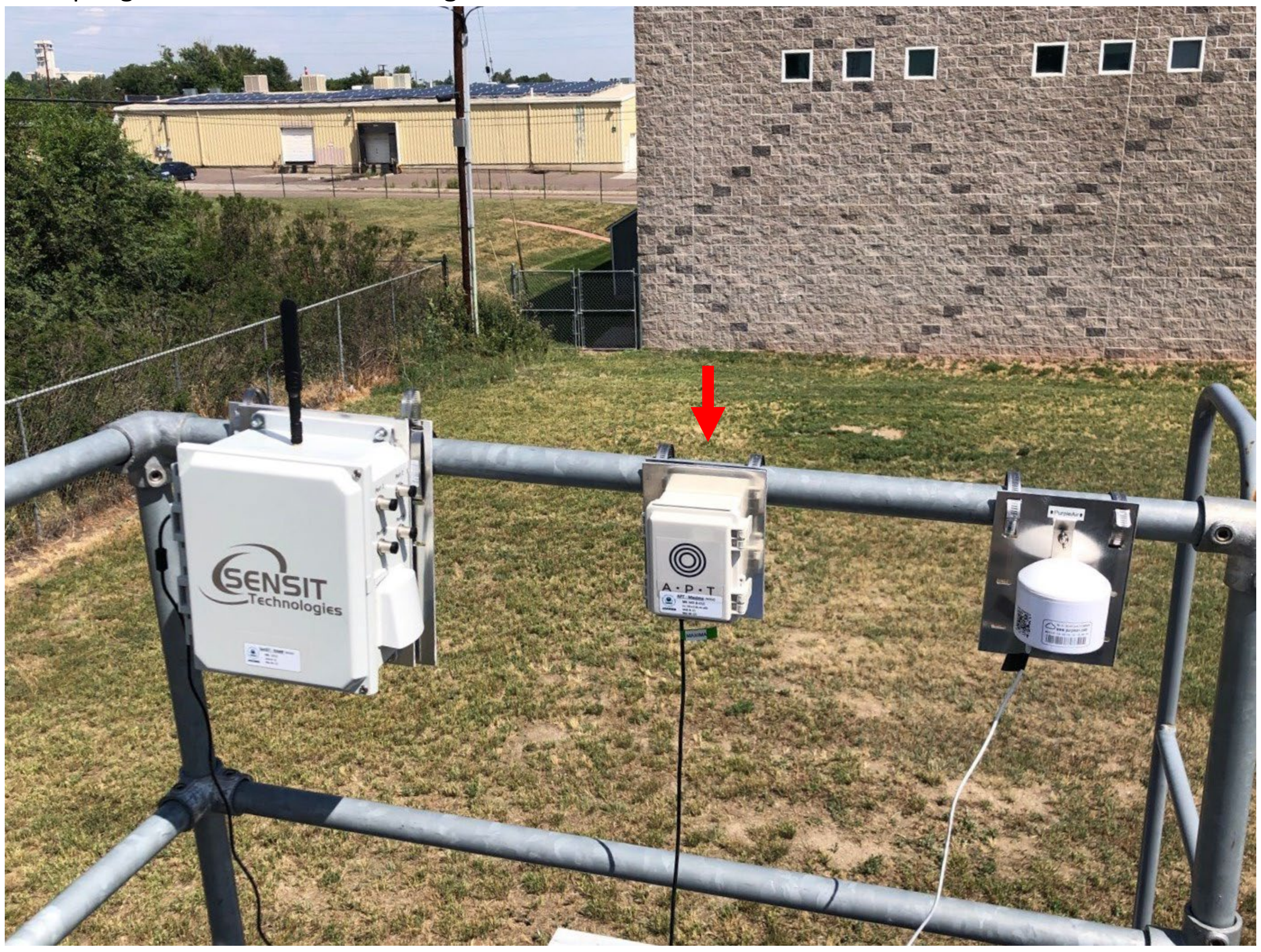

Figure 2: La Casa Monitoring Station sampling shelter, side view

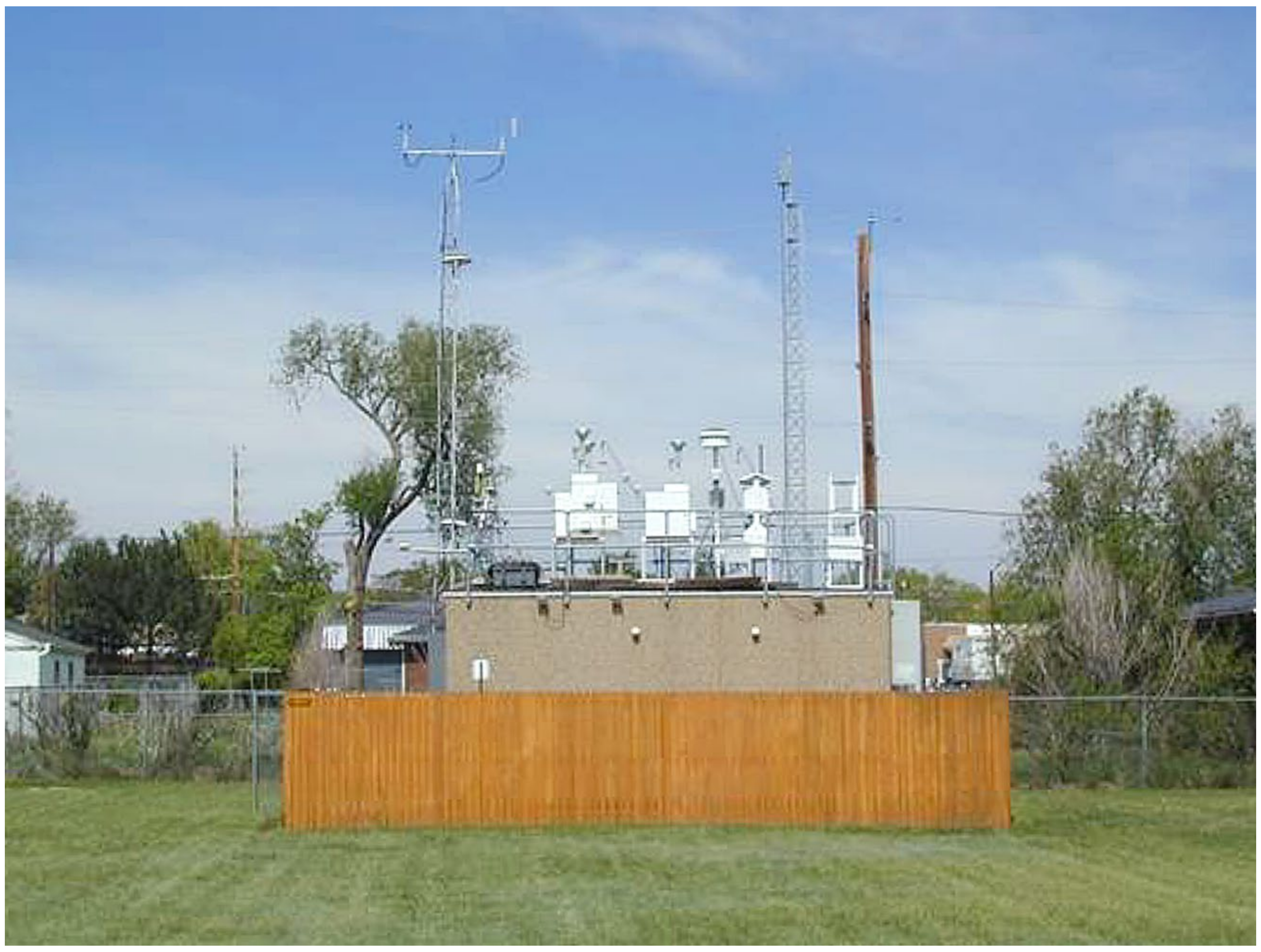

U.S. Environmental Protection Agency
Office of Research and Development
PI: Clements.Andrea@epa.gov
919-541-1363

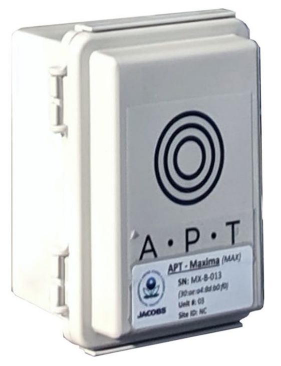

Supplemental Information: Data Storage, Correction Approach, and Issues Encountered

Data Storage and Transmission Method

The APT Maxima records data at 30 second intervals and stores the data on an internal microSD card and transmits data to the APT cloud server (last accessed 5/11/22) via Wi-Fi connection. The user interface for the Maxima sensors was under development during sensor testing, thus weekly raw data files were obtained from the internal MicroSD card. Each field site operator was provided two labeled MicroSD cards for the Maxima that they used to swap out each week. Data from the collected card was then read and processed off-site.

Data Correction Approach

This evaluation report reflects “out-of-the-box” performance of the APT Maxima. The Maxima data sets include two data columns contain $PM_{2.5}$ concentration measurements, “PM2.5(ug/m3)” and “PM2.5_Atm(ug/m3)”. These columns correspond to separate correction factors provided by the onboard OEM PM sensor (Plantower PMSA003). For this report, $PM_{2.5}$ measurements for the column labeled “PM2.5(ug/m3)” were selected for analysis.

After acquisition, the raw data was processed using the *sensortoolkit* python code library (v0.8.3b2). A continuous data set at the recorded sampling frequency was written to a .csv file. 1-hour and 24-hour averaged data sets were generated using a 75% completeness threshold and saved as separate .csv files. Outliers were NOT removed from data sets in order to assess “out-of-the-box” sensor performance.

Issues Encountered

Pre-deployment observations

- *Sampling internal abnormality:* During the pre-deployment phase, it was observed that Maxima devices did not continuously sample at 30-second intervals as stated in the sensor user guide. Instead, Maxima units occasionally sampled at 31 seconds. This increased sample interval is due to the accumulation of processing time over a certain number of samples. Each 30-second sample is a collection of exactly 30 seconds of sampling followed by a brief fraction of a second used for processing the collected data. Once processed, the next 30-second data collection period begins. When the sum of the processing time reaches one full second, the sample interval then jumps to 31 seconds. Data from this pre-deployment testing indicated that the processing speed for each sensor varies such that 31-second sampling duration occur at random intervals across Maxima units.
- *Startup connectivity issues:* Prior to the testing period indicated in this report, the APT Maxima unit deployed at the La Casa site experienced initial startup issues due to faulty battery connections upon arrival at the monitoring site on July 31, 2019. A replacement APT Maxima unit (referred to in this report as MAX_01) was deployed at the monitoring site on August 7, 2019.

Field observations and sensor data flags

During the testing period, the APT Maxima operated nominally and did not require replacement or repair.

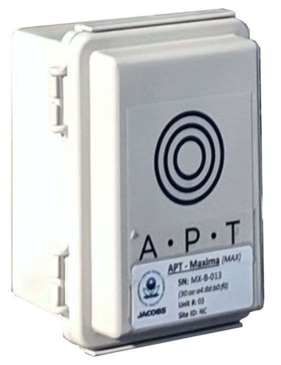

Description of Data Flags

AQS

The U.S. EPA’s Air Quality System (AQS) is the Agency’s primary ambient air monitoring data archive. A comprehensive list of data flags that are recorded alongside AQS data sets, referred to by U.S. EPA as ‘qualifiers’, can be found at the following link: https://aqs.epa.gov/aqsweb/documents/codetables/qualifiers.html

Invalidation of reference data: AQS qualifiers are organized by qualifier type, which indicates whether data logged alongside qualifier flags should be invalidated (set null). Qualifiers with type “Null Data Qualifier” are invalidated, and includes data logged during periods that coincide with QC checks (e.g., "BF-Precision/Zero/Span", "BJ- Operator Error", "BL - QA Audit“, “AZ - QC Audit”) among other events such as power outages. Data logged alongside qualifiers with type “Quality Assurance Qualifiers” are not invalidated and are included in this analysis (e.g., concentrations less than the federal MDL for the reference monitor “MD – Value less than MDL”, QA reviewed values "Validated Value“).

Data Flags Recorded During Testing

FRM/FEM Monitor	Timestamp (UTC)	Flag
Teledyne API T640 (Acquired via AQS)	2019-09-17 16:00:00+0000 to 2019-09-17 17:00:00+0000	BA - Maintenance/Routine Repairs
	2019-09-19 14:00:00+0000 to 2019-09-19 15:00:00+0000	BA - Maintenance/Routine Repairs
	2019-09-30 16:00:00+0000 to 2019-09-30 17:00:00+0000	BA - Maintenance/Routine Repairs
	2019-10-04 03:00:00+0000 to 2019-10-04 11:00:00+0000	AN - Machine Malfunction
	2019-10-04 12:00:00+0000	BA - Maintenance/Routine Repairs
	2019-10-04 17:00:00+0000 to 2019-10-04 18:00:00+0000	BA - Maintenance/Routine Repairs

Meteorological Instrument	Timestamp (UTC)	Flag
MetOne Temperature Monitor (Acquired via AQS)	2019-09-13 17:00:00+0000 to 2019-09-13 18:00:00+0000	AZ - QC Audit
	2019-10-04 03:00:00+0000 to 2019-10-04 12:00:00+0000	BK - Site computer/data logger down
	2019-10-04 17:00:00+0000 to 2019-10-04 18:00:00+0000	AN - Machine Malfunction
Climatronics Relative Humidity Monitor (Acquired via AQS)	2019-09-13 17:00:00+0000 to 2019-09-13 18:00:00+0000	AZ - QC Audit
	2019-10-04 03:00:00+0000 to 2019-10-04 12:00:00+0000	BK - Site computer/data logger down
	2019-10-04 17:00:00+0000 to 2019-10-04 18:00:00+0000	AN - Machine Malfunction

Testing Report - $PM_{2.5}$ Base Testing

Clarity Node

This report reflects out-of-the-box performance

Initial Base Testing - Denver, CO

U.S. Environmental Protection Agency
Office of Research and Development
PI: Clements.Andrea@epa.gov
919-541-1363
August 2019—September 2019

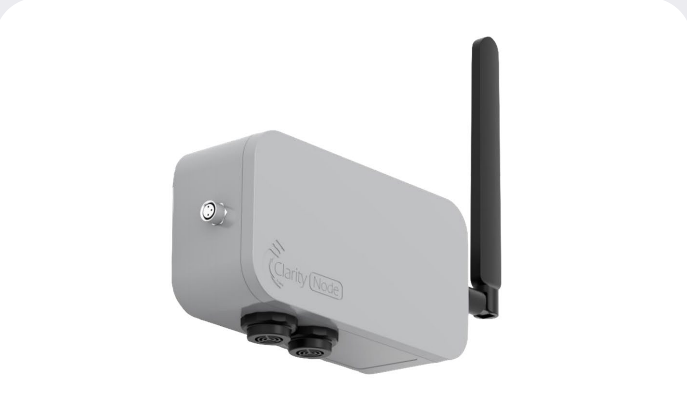

Image Attribution: Clarity Movement Co. (Berkeley, CA)

Deployment Details

Testing Organization and Site Information	
Testing organization (Name, Organization type, Contact website)	U.S. Environmental Protection Agency - Office of Research and Development<br>Federal Government<br>Air Sensor Toolbox | U.S. EPA Website
Testing location (City, State, Latitude and Longitude)	La Casa<br>Denver, CO<br>39.779429, -105.005174
AQS site ID	08 - 031 - 0026
Sampling timeframe (MM-DD-YY)	08-21-19 to 09-20-19
Sensor data source	Clarity Dashboard download
Reference data source	AQS API download

Sensor Information			
Manufacturer, model	Clarity Node		
Device firmware version	Received by EPA May 2019		
Sampling time interval	3-minutes		
Sensor serial numbers	CNO_01		
Issues encountered during deployment?	☑	See CO-CNO-Page 6 of this testing report	

FRM/FEM Information	
Manufacturer, model, designation	Teledyne Advanced Pollution Instrumentation T640 FEM
Sampling time interval	1-hour averaging
Date of calibration	As required by 40 CFR Part 58 and the CO Regional Monitoring Site QAPP maintained by CDPHE
Date of flowrate verification check	Monthly as required by 40 CFR Part 58 Appendix A
Description, date(s) of maintenance activities	See CO-CNO-Page 7 of this testing report

Time Series Plots

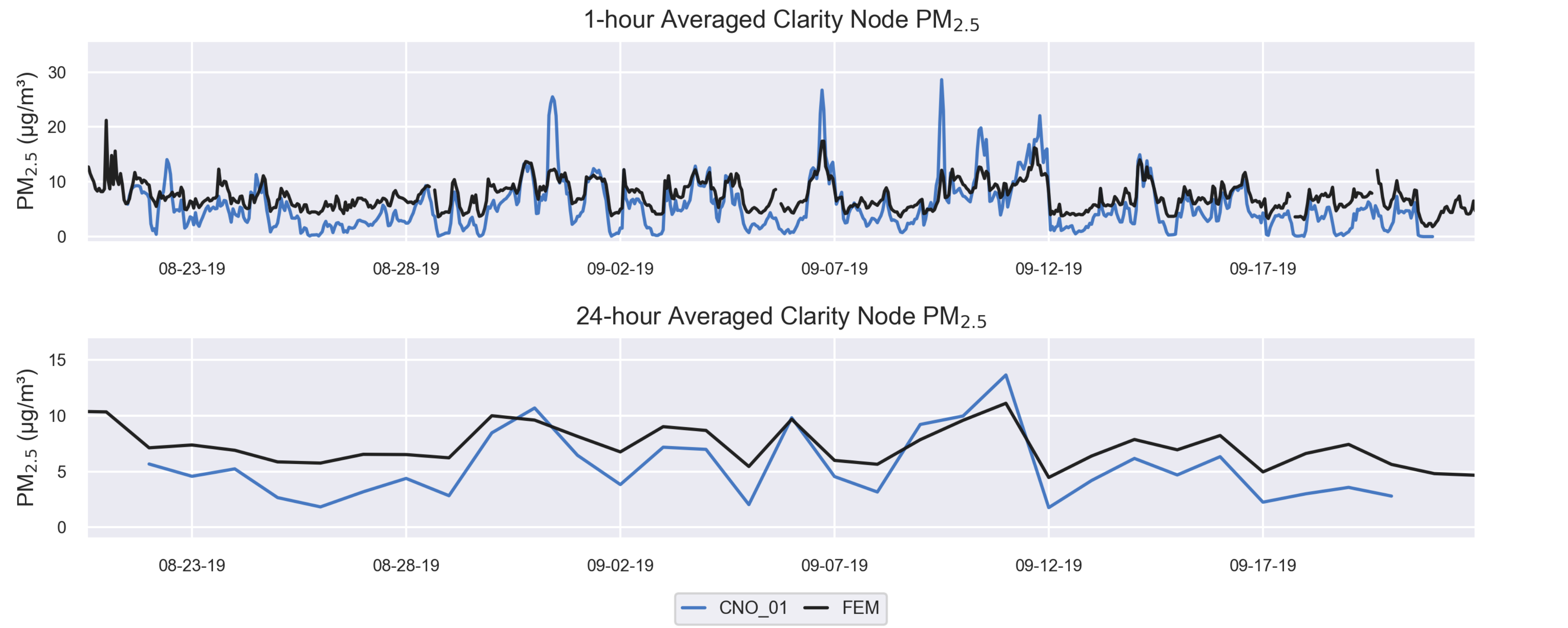

Scatter Plots: Comparison to FRM/FEM

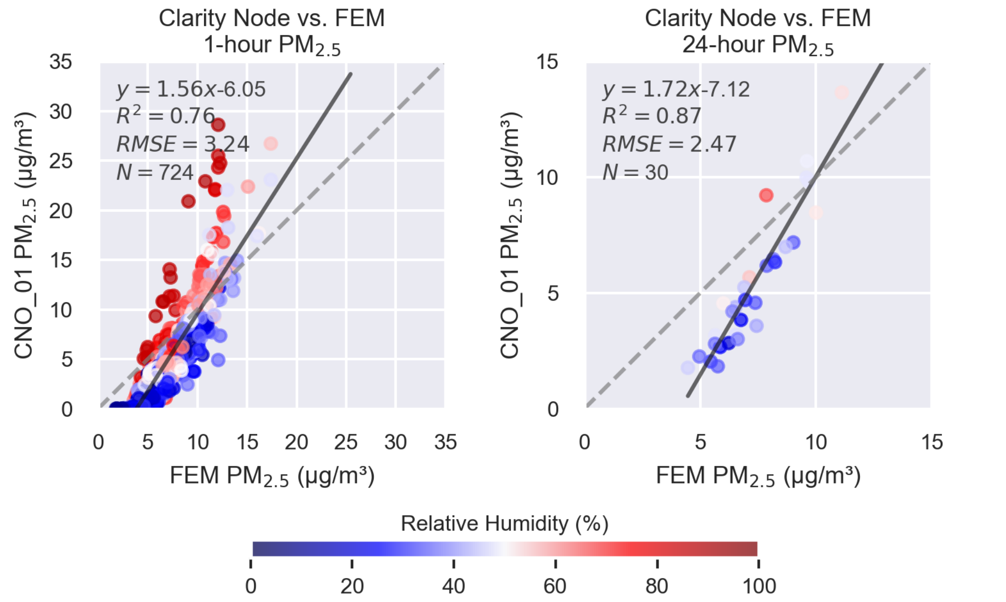

Range of FRM/FEM concentrations over duration of base test (μg/m³)	1.8-17.4 (1-hr), 4.5-11.1 (24-hr)
Number of 24-hr periods in FRM/FEM monitor measurements with a goal concentration ≥ 25 μg/m³	0

Performance Metrics

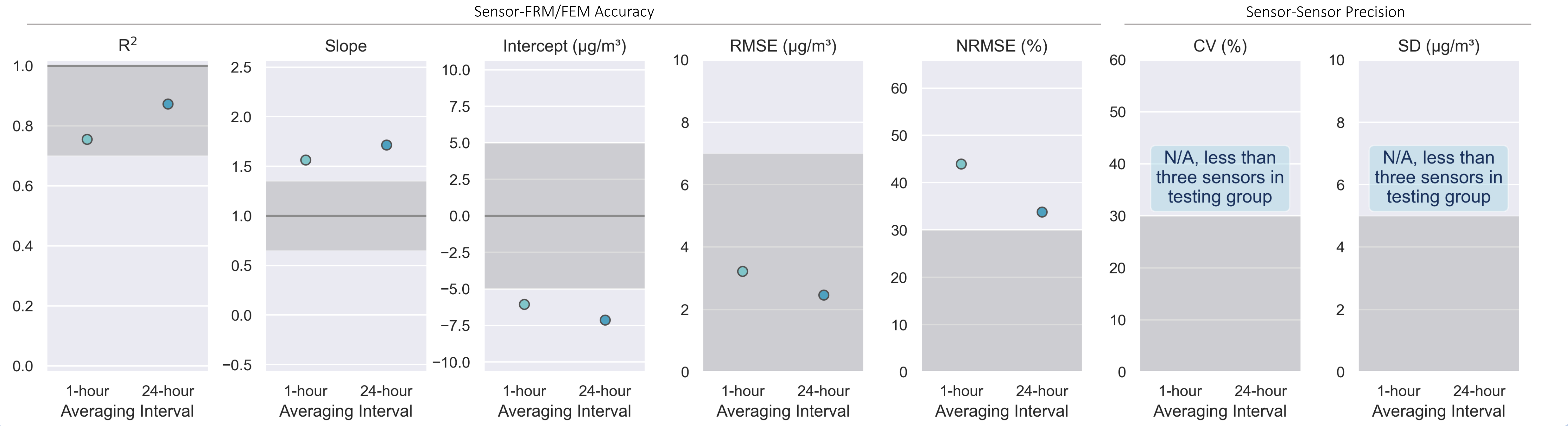

Meteorological Conditions During Deployment

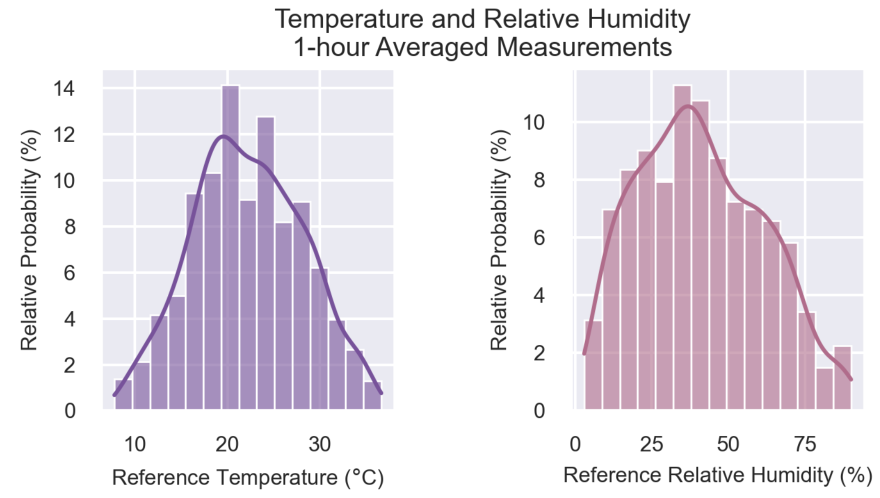

Number of 24-hr periods outside sensor manufacture-listed temperature operational range (-10 to 55 °C)	0
Number of 24-hr periods outside sensor manufacture-listed relative humidity operational range (10 to 98%)	0

Meteorological Influence

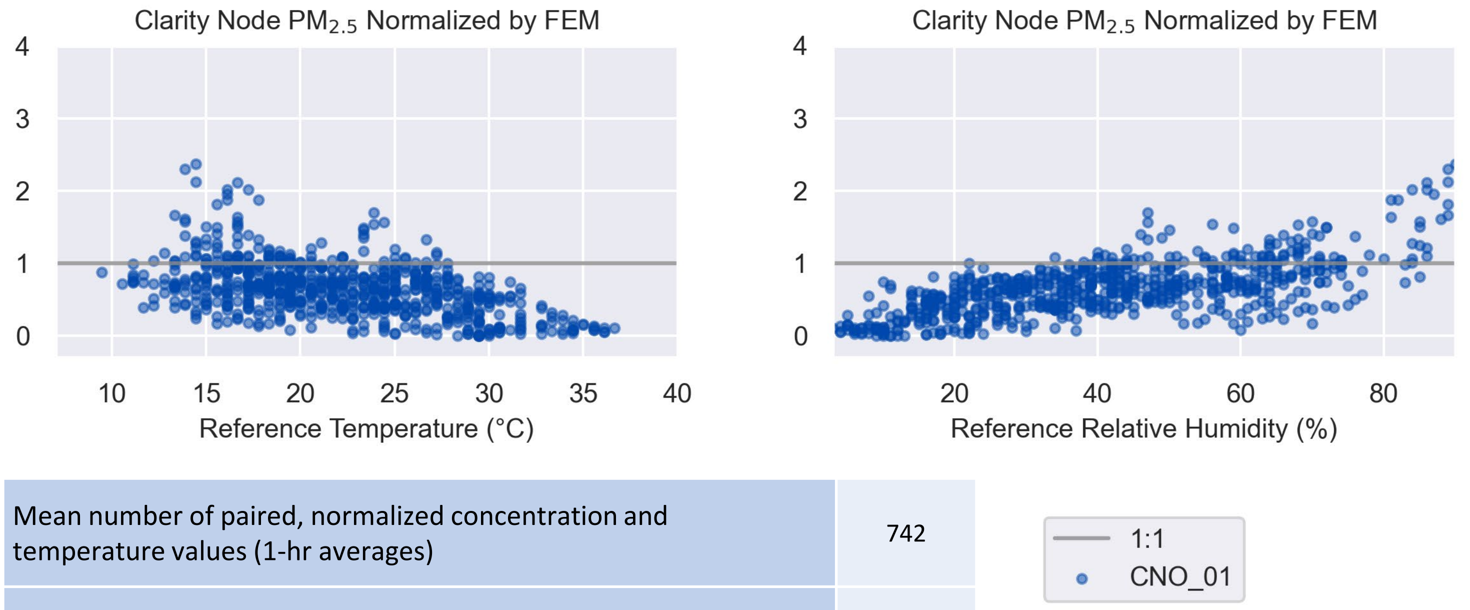

Mean number of paired, normalized concentration and temperature values (1-hr averages)	742
Mean number of paired, normalized concentration and relative humidity values (1-hr averages)	742

Testing Report - $PM_{2.5}$ Base Testing

Clarity Node

This report reflects out-of-the-box performance

Initial Base Testing - Denver, CO
U.S. Environmental Protection Agency
Office of Research and Development
PI: Clements.Andrea@epa.gov
919-541-1363
August 2019—September 2019

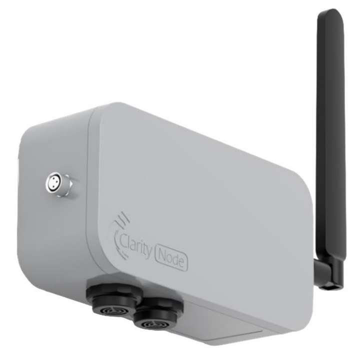

Image Attribution: Clarity Movement Co. (Berkeley, CA)

Tabular Statistics

Sensor-FRM/FEM Correlation

	Bias and Linearity						Data Quality			
	R^2		Slope		Intercept (µg/m³)		Uptime (%)		Number of paired sensor and FRM/FEM concentration values	
	1-Hour ●	24-Hour ●	1-Hour ○	24-Hour ○	1-Hour ○	24-Hour ○	1-Hour ●	24-Hour ●	1-Hour	24-Hour
Metric Target Range	≥ 0.70	≥ 0.70	1.0 ± 0.35	1.0 ± 0.35	-5 ≤ b ≤ 5	-5 ≤ b ≤ 5	75%*	75%*	-	-
Sensor CNO_01	0.76	0.87	1.56	1.72	-6.05	-7.12	100	100	724	30

	Error			
	RMSE (µg/m³)		NRMSE (%)	
	1-Hour ★	24-Hour ★	1-Hour ☆	24-Hour ☆
Metric Target Range	≤ 7.0	≤ 7.0	≤ 30.0	≤ 30.0
Deployment Value	3.2	2.5	44.0	33.9

Device-specific metrics (computed for each sensor in evaluation)
○○○ Metric value for none of devices tested falls within the target range
●○○ Metric value for one of devices tested falls within the target range
●●○ Metric value for two of devices tested falls within the target range
●●● Metric value for three of devices tested falls within the target range

Single-valued metrics (computed via entire evaluation dataset)
☆ Indicates that the metric value is not within the target range
★ Indicates that the metric value is within the target range

Sensor-Sensor Precision[1]

	Precision (between collocated sensors)				Data Quality	
	CV (%)		SD (µg/m³)		Number of concurrent sensor concentration pairs	
	1-Hour ☆	24-Hour ☆	1-Hour ☆	24-Hour ☆	1-Hour	24-Hour
Metric Target Range	≤ 30.0	≤ 30.0	≤ 5.0	≤ 5.0	-	-
Deployment Value	-	-	-	-	-	-

[1]Precision statistics are computed for evaluations with at least three collocated sensor units. Metric values are left blank for evaluations with two or fewer sensor units.

*This value is only a recommendation for ensuring data quality and is not included in the list of target values discussed in Section 4 of the Performance Testing Protocols, Metrics, and Target Values for Fine Particulate Matter Air Sensors document.

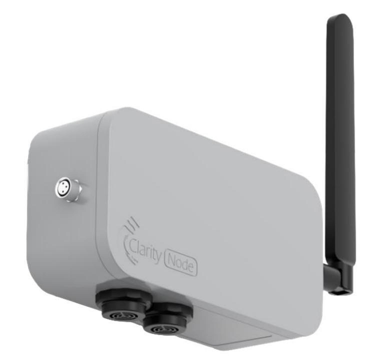

Image Attribution: Clarity Movement Co. (Berkeley, CA)

Sensor-FRM/FEM Scatter Plots

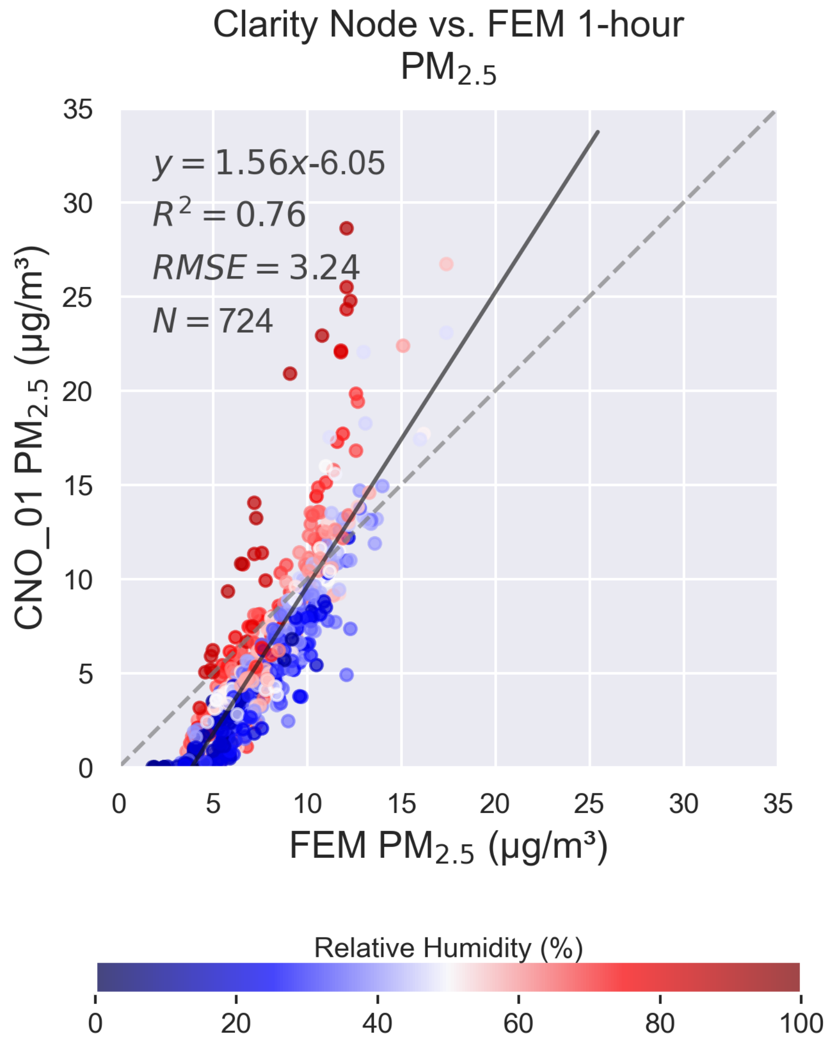

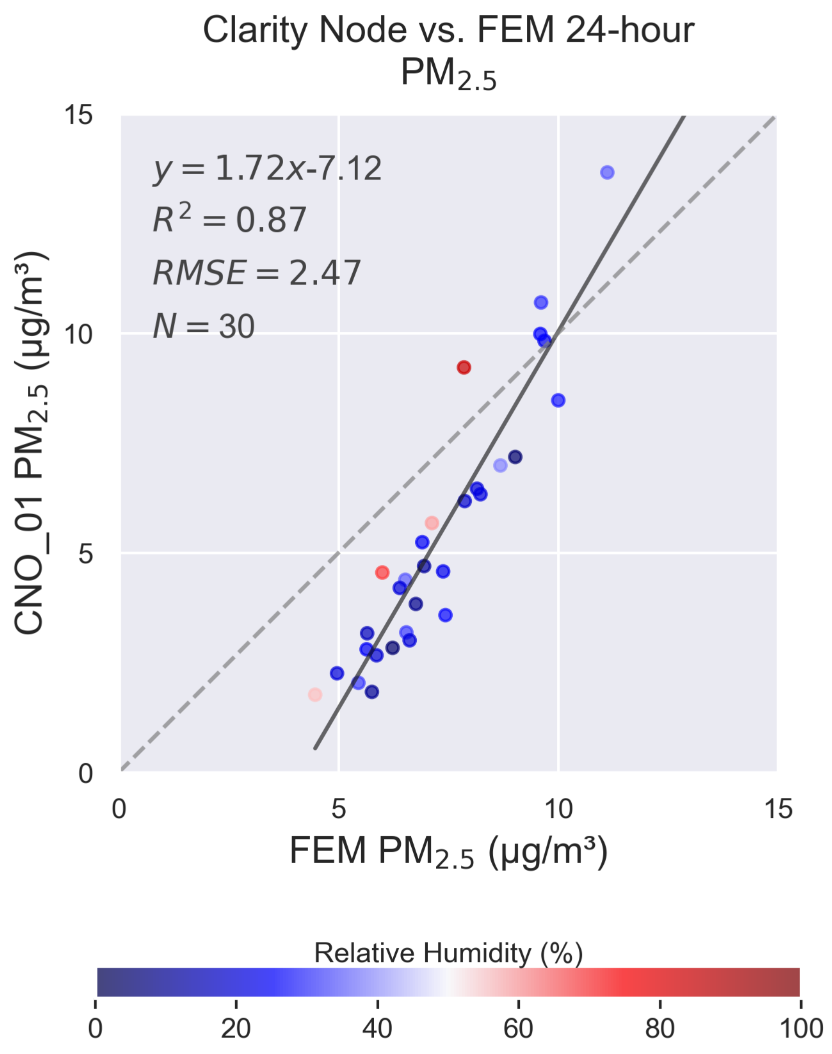

U.S. Environmental Protection Agency
Office of Research and Development
PI: Clements.Andrea@epa.gov
919-541-1363

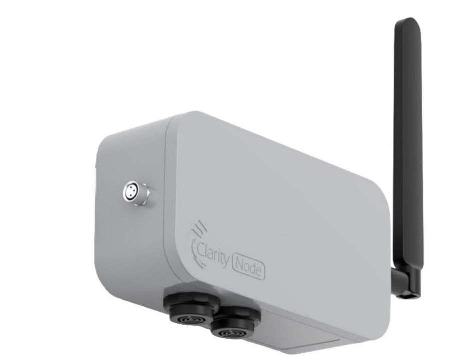

Image Attribution: Clarity Movement Co. (Berkeley, CA)

Supplemental Information

Abbreviations used in Supplemental Information

FRM	Federal Reference Method
FEM	Federal Equivalent Method
SOP	Standard Operating Procedure
QAPP	Quality Assurance Project Plan
QC	Quality Control

Supplemental Documentation	Attached	Description & URL or file path to documentation
Field observations and sensor data flags	☑	See CO-CNO-Page 6 of this testing report
Maintenance logs	☐	No logs recorded during testing
Standard operating procedure(s)	☐	U.S. EPA Office Of Research and Development SOP available upon request
Photos of equipment setup and testing	☑	See CO-CNO-Page 5 of this testing report
Product specifications sheet(s)	☐	N/A
Product manual(s)	☐	N/A
Data storage and transmission method	☑	See CO-CNO-Page 6 of this testing report
Data correction approach	☑	See CO-CNO-Page 6 of this testing report
Issues encountered	☑	See CO-CNO-Page 6 of this testing report
Data analysis/correction scripts and version	☑	Averaging and processing of data, calculation of performance metrics, and generation of figures and other supplementary material for analysis were obtained using Python 3.9.7 with the packages sensortoolkit v0.8.3b2, pandas 1.3.5, NumPy 1.21.2, Matplotlib 3.5.0, statsmodels 0.13.0, and seaborn 0.11.2. All packages are available from the Python Package Index (PyPI) at https://pypi.org. The integrated development environment (IDE) Spyder 5.1.5 was used for scripting and data visualization. Version control for the Python base, packages, and IDE were all managed by conda 4.11.0.
Air Monitoring Station QAPP	☐	U.S. EPA Office Of Research and Development QAPP available upon request
Summary of FRM/FEM monitor QC checks	☑	See CO-CNO-Page 7 of this testing report
Manufacturer website for FRM/FEM monitor	☑	Teledyne API: T640 Product website
FRM/FEM monitor manual	☑	Teledyne API: T640 Product Manual
FRM/FEM monitor specifications sheet(s)	☑	Teledyne API: T640 Specification Sheet
Other documents	☐	

Supplemental Information: Photos of Testing Site and Equipment Setup

Site Description:

The La Casa site was established in January of 2013 as a replacement for the Denver Municipal Animal Shelter (DMAS) site when a land use change forced the relocation of the site. The La Casa location has been established as the NCore site for the Denver Metropolitan area. Measurements include trace gas/precursor-level CO analyzer, and a NO_y analyzer, in addition to the trace level SO_2, O_3, meteorology, and particulate monitors. The site represents a population-oriented neighborhood scale monitoring area.

Figure 1: Clarity Node sensor (indicated by red arrow) attached to metal railing atop the sampling shelter at the monitoring site.

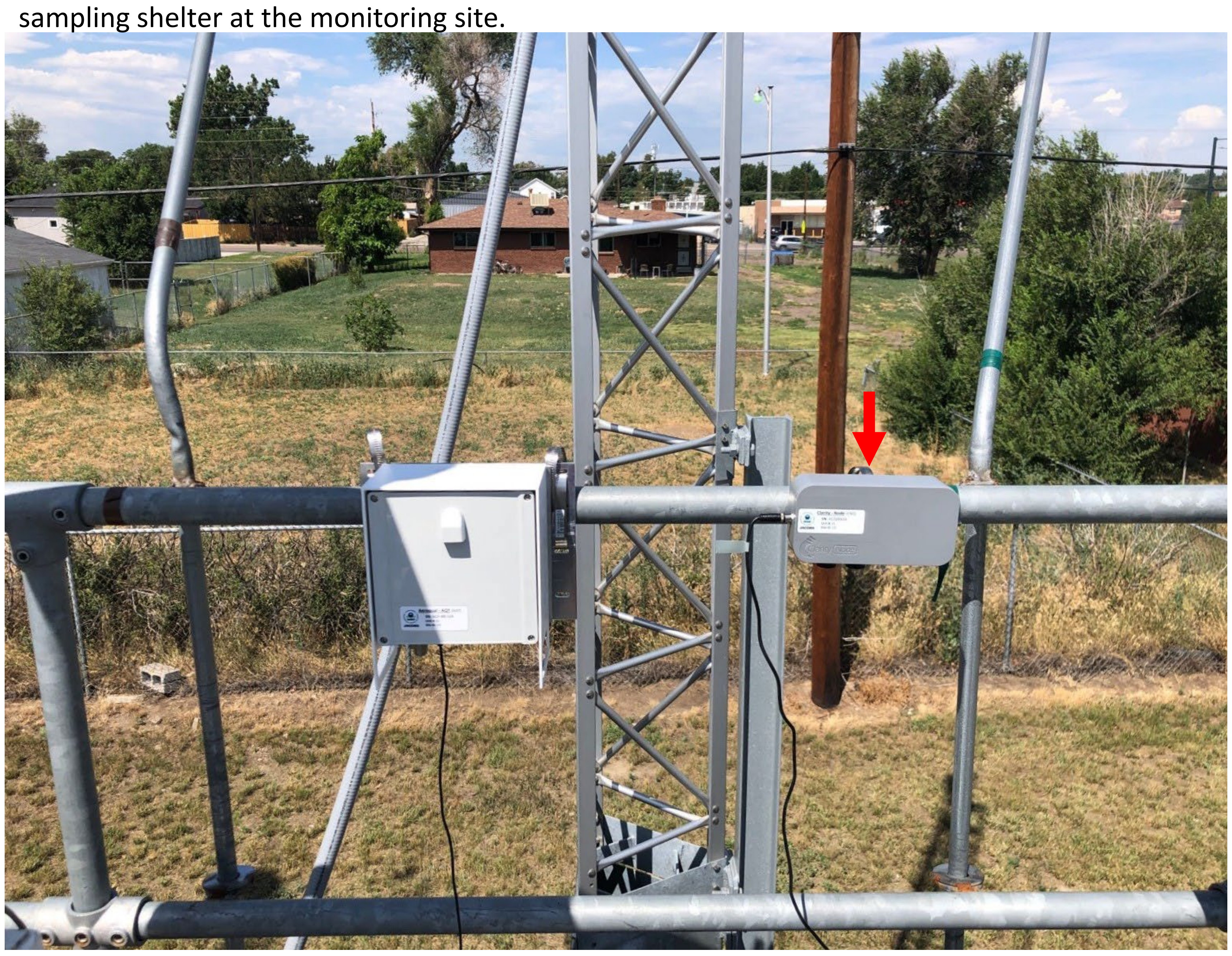

Figure 2: La Casa Monitoring Station sampling shelter, side view

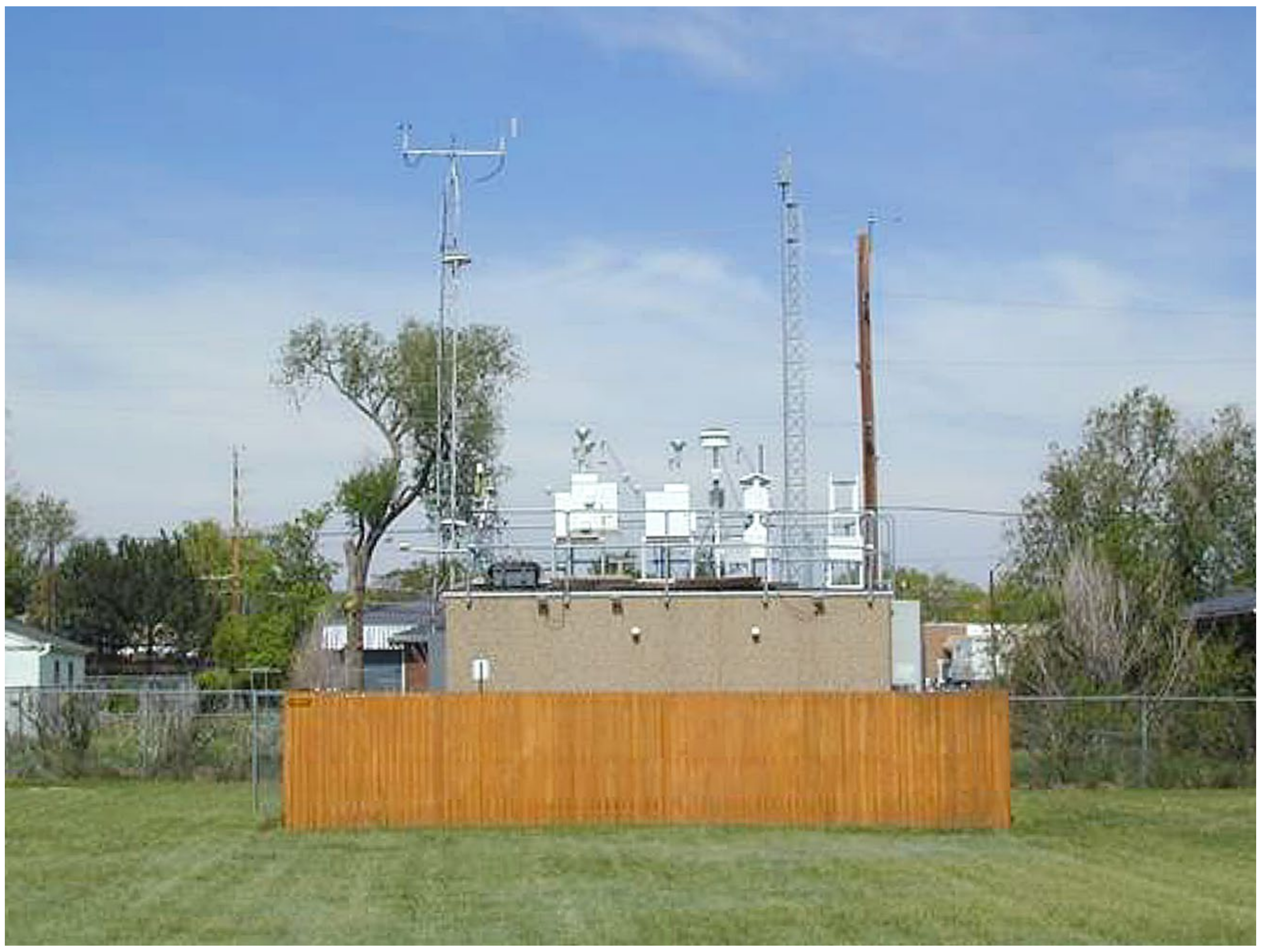

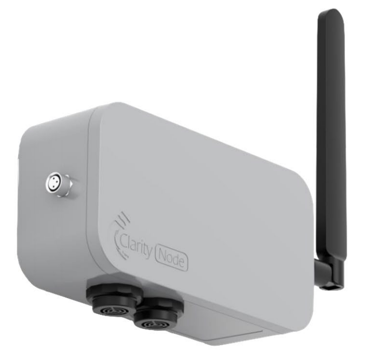
Image Attribution: Clarity Movement Co. (Berkeley, CA)

Data Storage and Transmission Method

The Clarity Node transmits data via cellular SIM card. Weekly raw data collections were performed over Wi-Fi using the Clarity dashboard (*last accessed 5/11/22*). The observed sampling interval fluctuated and is further discussed under pre-deployment observations below. On average, the Node models sampled every 2-3 minutes, and the Node-S sampled every 15-17 minutes.

Data Correction Approach

This evaluation report reflects "out-of-the-box" performance of the Clarity Node. The manufacturer provides a dashboard feature for calibrating sensor data against collocated reference monitors. Calibrated data were NOT used for this evaluation. Prospective consumers may get better performance from this device if they utilize this feature.

After acquisition, the raw data was processed using the *sensortoolkit* python code library (v0.8.3b2). A continuous data set at the recorded sampling frequency was written to a .csv file. 1-hour and 24-hour averaged data sets were generated using a 75% completeness threshold and saved as separate .csv files. Due to the variable sampling interval observed for Clarity Node units, 75% completeness was determined against the number of data points that would be recorded within 1-hour/24-hour periods if the sensor were to log measurements at the most commonly recorded sampling interval (i.e., the mode of recorded intervals). Outliers were NOT removed from data sets in order to assess "out-of-the-box" sensor performance.

Issues Encountered

Pre-deployment observations

- *Unsteady sampling interval:* Clarity Node sensors were received by EPA on May 28, 2019. A lab bench-top evaluation was performed from May 29, 2019 – May 31, 2019. During bench-top testing, it was observed that the sampling intervals noted by Clarity (5 minutes for non-solar Node, 15 minutes for the solar Node-S) were not reflective of recorded intervals. Communication with the vendor concluded that the sampling interval will fluctuate depending on the settings, battery charge, and strength of the cellular signal and that this sampling interval cannot be adjusted by the user at present. On average, the Node models sampled every 2-3 minutes, and the Node-S sampled every 15-17 minutes.

Field observations and sensor data flags

The Clarity Node was deployed at the La Casa monitoring site on 7/31/2019. Throughout the testing period, numerous sampling interval abnormality flags were logged, associated with a change in the sampling frequency of the Clarity Node. As noted during pre-deployment testing, the Node typically samples at 2 to 3-minute intervals. Occasionally, the device recorded samples at longer intervals ranging from 4 to 6 minutes, followed by a period of alternating 2 to 3-minute sampling intervals. This longer-duration sampling interval repeatedly occurred at seemingly random intervals. This change in sampling frequency was not a result of unit configuration changes applied by a field technician and reflect the out-of-the-box fluctuating sampling interval.

Start Time (UTC)	End Time (UTC)	Sensor Serial ID	Parameters Impacted	Flag
2019-09-16 01:56:00+00:00	2019-09-16 02:02:00+00:00	CNO_01	ALL	6-Sampling interval abnormality
2019-09-17 06:54:00+00:00	2019-09-17 07:00:00+00:00	CNO_01	ALL	6-Sampling interval abnormality
2019-09-18 00:59:00+00:00	2019-09-18 01:05:00+00:00	CNO_01	ALL	6-Sampling interval abnormality

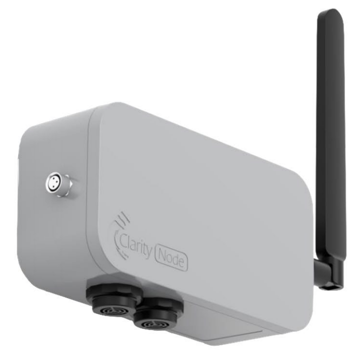

Image Attribution: Clarity Movement Co. (Berkeley, CA)

Description of Data Flags

AQS

The U.S. EPA's Air Quality System (AQS) is the Agency's primary ambient air monitoring data archive. A comprehensive list of data flags that are recorded alongside AQS data sets, referred to by U.S. EPA as 'qualifiers', can be found at the following link: https://aqs.epa.gov/aqsweb/documents/codetables/qualifiers.html

Invalidation of reference data: AQS qualifiers are organized by qualifier type, which indicates whether data logged alongside qualifier flags should be invalidated (set null). Qualifiers with type "Null Data Qualifier" are invalidated, and includes data logged during periods that coincide with QC checks (e.g., "BF-Precision/Zero/Span", "BJ- Operator Error", "BL - QA Audit", "AZ - QC Audit") among other events such as power outages. Data logged alongside qualifiers with type "Quality Assurance Qualifiers" are not invalidated and are included in this analysis (e.g., concentrations less than the federal MDL for the reference monitor "MD – Value less than MDL", QA reviewed values "Validated Value").

Data Flags Recorded During Testing

FRM/FEM Monitor	Timestamp (UTC)	Flag
Teledyne API T640 (Acquired via AQS)	2019-08-28 14:00:00+0000 to 2019-08-28 15:00:00+0000	BA - Maintenance/Routine Repairs
	2019-09-05 16:00:00+0000 to 2019-09-05 17:00:00+0000	BL - QA Audit
	2019-09-17 16:00:00+0000 to 2019-09-17 17:00:00+0000	BA - Maintenance/Routine Repairs
	2019-09-19 14:00:00+0000 to 2019-09-19 15:00:00+0000	BA - Maintenance/Routine Repairs

Meteorological Instrument	Timestamp (UTC)	Flag
MetOne Temperature Monitor (Acquired via AQS)	2019-09-13 17:00:00+0000 to 2019-09-13 18:00:00+0000	AZ - QC Audit
Climatronics Relative Humidity Monitor (Acquired via AQS)	2019-09-13 17:00:00+0000 to 2019-09-13 18:00:00+0000	AZ - QC Audit

Testing Report - $PM_{2.5}$ Base Testing

PurpleAir PA-II-SD

This report reflects out-of-the-box performance

Initial Base Testing - Denver, CO

U.S. Environmental Protection Agency
Office of Research and Development
PI: Clements.Andrea@epa.gov
919-541-1363

August 2019—September 2019

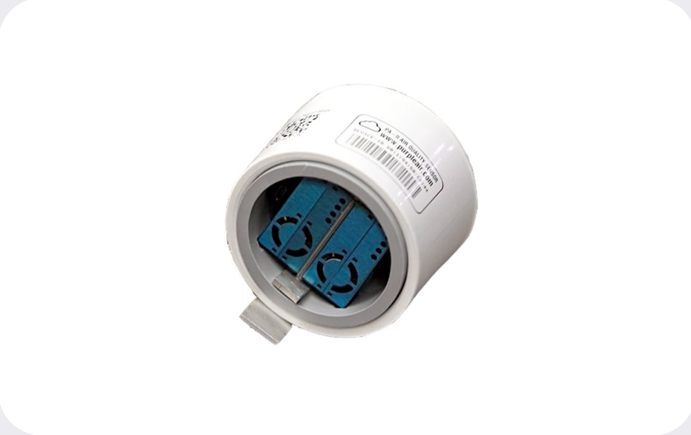

Deployment Details

Testing Organization and Site Information	
Testing organization (Name, Organization type, Contact website)	U.S. Environmental Protection Agency - Office of Research and Development<br>Federal Government<br>Air Sensor Toolbox | U.S. EPA Website
Testing location (City, State, Latitude and Longitude)	La Casa<br>Denver, CO<br>39.779429, -105.005174
AQS site ID	08 - 031 - 0026
Sampling timeframe (MM-DD-YY)	08-02-19 to 09-01-19
Sensor data source	Onboard MicroSD card
Reference data source	AQS API download

Sensor Information			
Manufacturer, model	PurpleAir PA-II-SD		
Device firmware version	v3.00 to v4.02		
Sampling time interval	80 seconds, 2-minutes		
Sensor serial numbers	PAR_01		
Issues encountered during deployment?	☑	See CO-PAR-Page 6 of this testing report	

FRM/FEM Information	
Manufacturer, model, designation	Teledyne Advanced Pollution Instrumentation T640 FEM
Sampling time interval	1-hour averaging
Date of calibration	As required by 40 CFR Part 58 and the CO Regional Monitoring Site QAPP maintained by CDPHE
Date of flowrate verification check	Monthly as required by 40 CFR Part 58 Appendix A
Description, date(s) of maintenance activities	See CO-PAR-Page 8 of this testing report

Time Series Plots

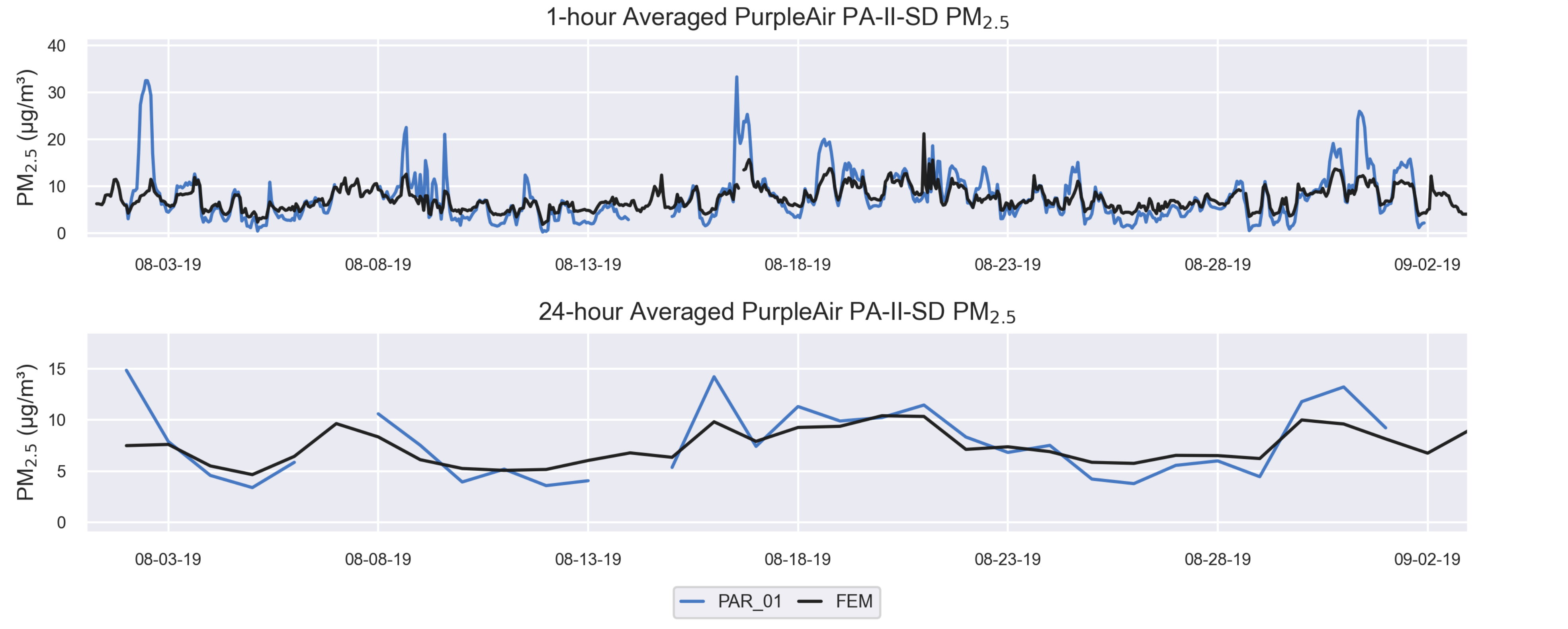

Scatter Plots: Comparison to FRM/FEM

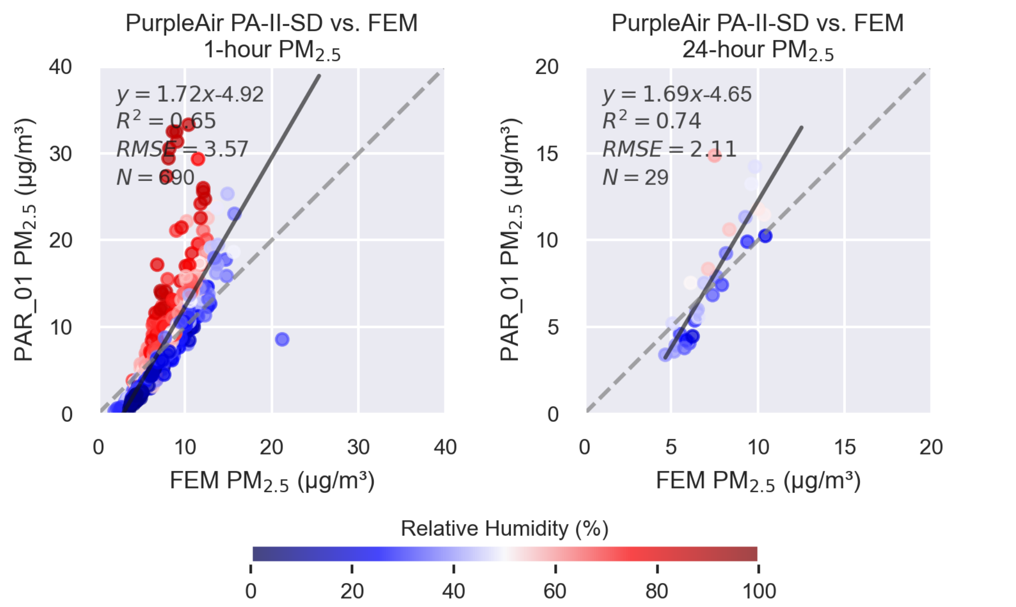

Range of FRM/FEM concentrations over duration of base test (µg/m³)	1.9-21.2 (1-hr), 4.7-10.4 (24-hr)
Number of 24-hr periods in FRM/FEM monitor measurements with a goal concentration ≥ 25 µg/m³	0

Performance Metrics

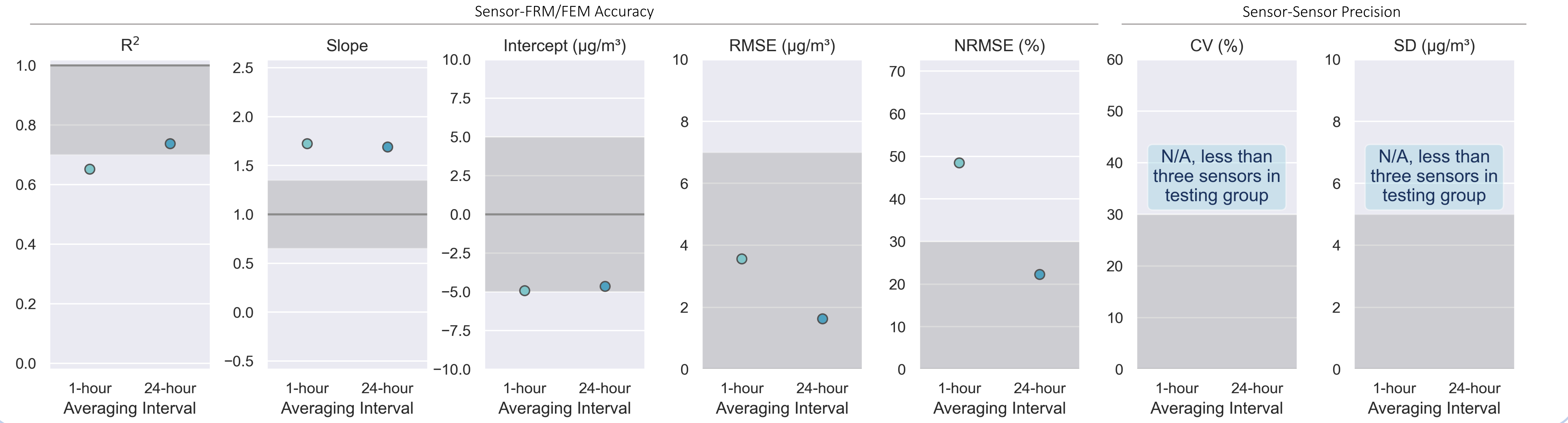

Meteorological Conditions During Deployment

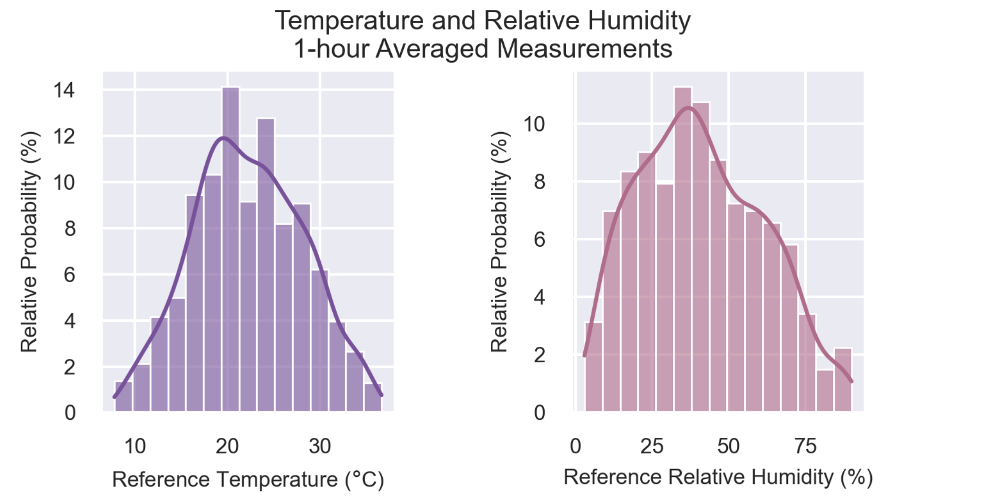

Number of 24-hr periods outside sensor manufacturer-listed temperature operational range (no operational range specified)	-
Number of 24-hr periods outside sensor manufacturer-listed relative humidity operational range (no operational range specified)	-

Meteorological Influence

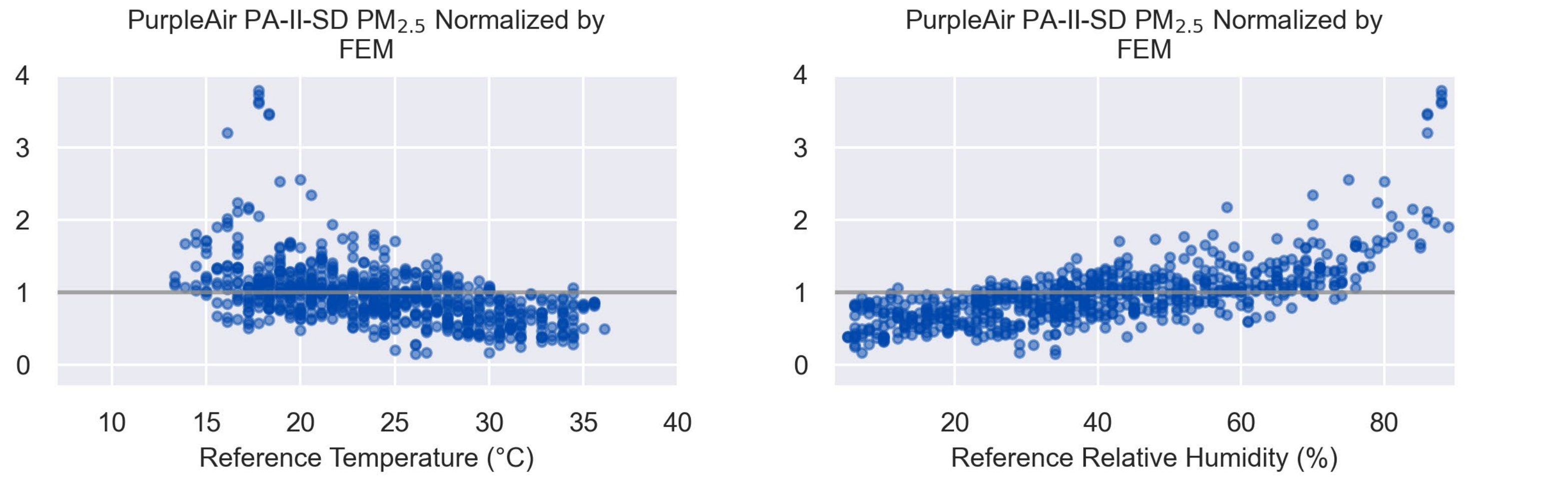

Mean number of paired, normalized concentration and temperature values (1-hr averages)	744
Mean number of paired, normalized concentration and relative humidity values (1-hr averages)	744

Testing Report - $PM_{2.5}$ Base Testing

PurpleAir PA-II-SD

This report reflects out-of-the-box performance

Initial Base Testing - Denver, CO

U.S. Environmental Protection Agency
Office of Research and Development
PI: Clements.Andrea@epa.gov
919-541-1363

August 2019—September 2019

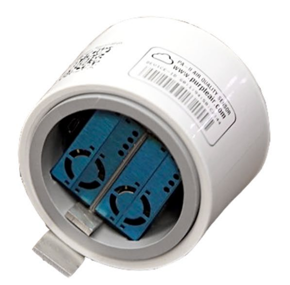

Tabular Statistics

Sensor-FRM/FEM Correlation

	Bias and Linearity						Data Quality			
	R^2		Slope		Intercept (µg/m³)		Uptime (%)		Number of paired sensor and FRM/FEM concentration values	
	1-Hour ○	24-Hour ●	1-Hour ○	24-Hour ○	1-Hour ●	24-Hour ●	1-Hour ●	24-Hour ●	1-Hour	24-Hour
Metric Target Range	≥ 0.70	≥ 0.70	1.0 ± 0.35	1.0 ± 0.35	-5 ≤ b ≤ 5	-5 ≤ b ≤ 5	75%*	75%*	-	-
Sensor PAR_01	0.65	0.74	1.72	1.69	-4.92	-4.65	93	94	690	29

	Error			
	RMSE (µg/m³)		NRMSE (%)	
	1-Hour ★	24-Hour ★	1-Hour ☆	24-Hour ★
Metric Target Range	≤ 7.0	≤ 7.0	≤ 30.0	≤ 30.0
Deployment Value	3.6	1.6	48.5	22.3

Device-specific metrics (computed for each sensor in evaluation)
○○○ Metric value for none of devices tested falls within the target range
●○○ Metric value for one of devices tested falls within the target range
●●○ Metric value for two of devices tested falls within the target range
●●● Metric value for three of devices tested falls within the target range

Single-valued metrics (computed via entire evaluation dataset)
☆ Indicates that the metric value is not within the target range
★ Indicates that the metric value is within the target range

Sensor-Sensor Precision[1]

	Precision (between collocated sensors)				Data Quality	
	CV (%)		SD (µg/m³)		Number of concurrent sensor concentration pairs	
	1-Hour ☆	24-Hour ☆	1-Hour ☆	24-Hour ☆	1-Hour	24-Hour
Metric Target Range	≤ 30.0	≤ 30.0	≤ 5.0	≤ 5.0	-	-
Deployment Value	-	-	-	-	-	-

[1]Precision statistics are computed for evaluations with at least three collocated sensor units. Metric values are left blank for evaluations with two or fewer sensor units.

*This value is only a recommendation for ensuring data quality and is not included in the list of target values discussed in Section 4 of the Performance Testing Protocols, Metrics, and Target Values for Fine Particulate Matter Air Sensors document.

Testing Report - PM$_{2.5}$ Base Testing

PurpleAir PA-II-SD

This report reflects out-of-the-box performance

Initial Base Testing - Denver, CO

U.S. Environmental Protection Agency
Office of Research and Development
PI: Clements.Andrea@epa.gov
919-541-1363

August 2019—September 2019

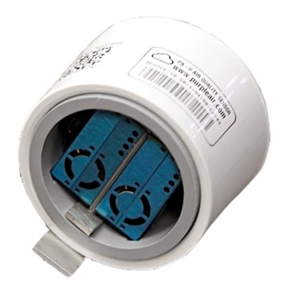

Sensor-FRM/FEM Scatter Plots

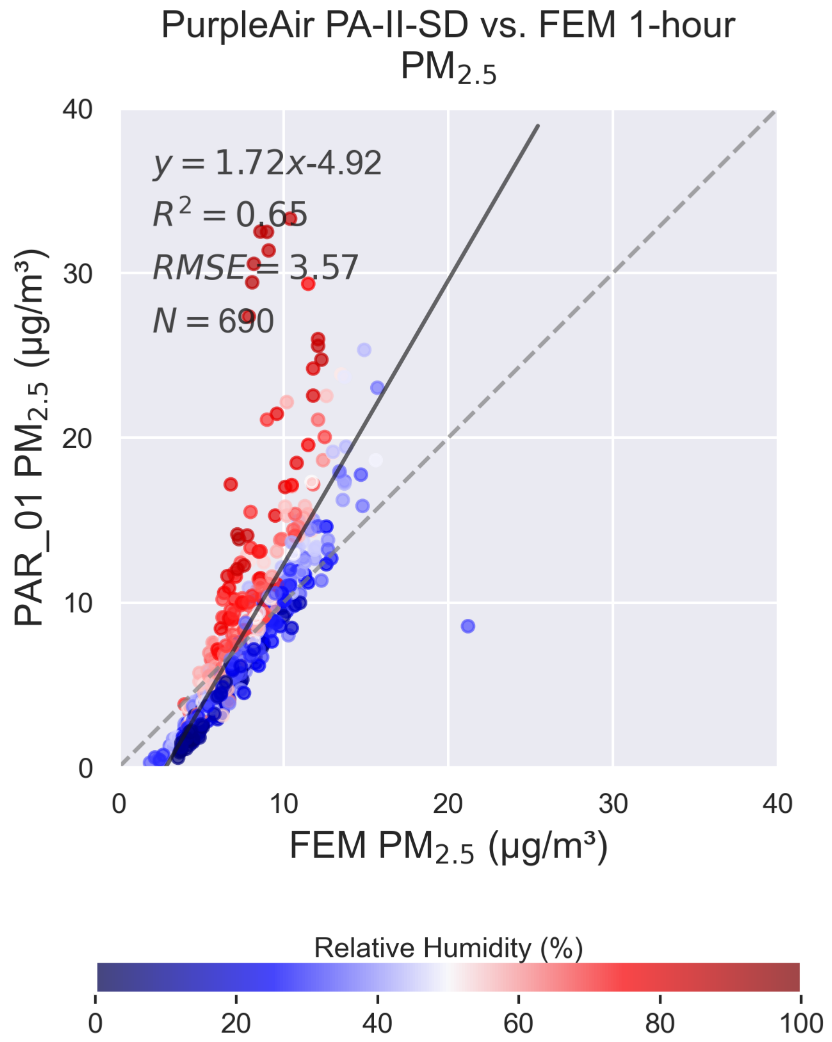

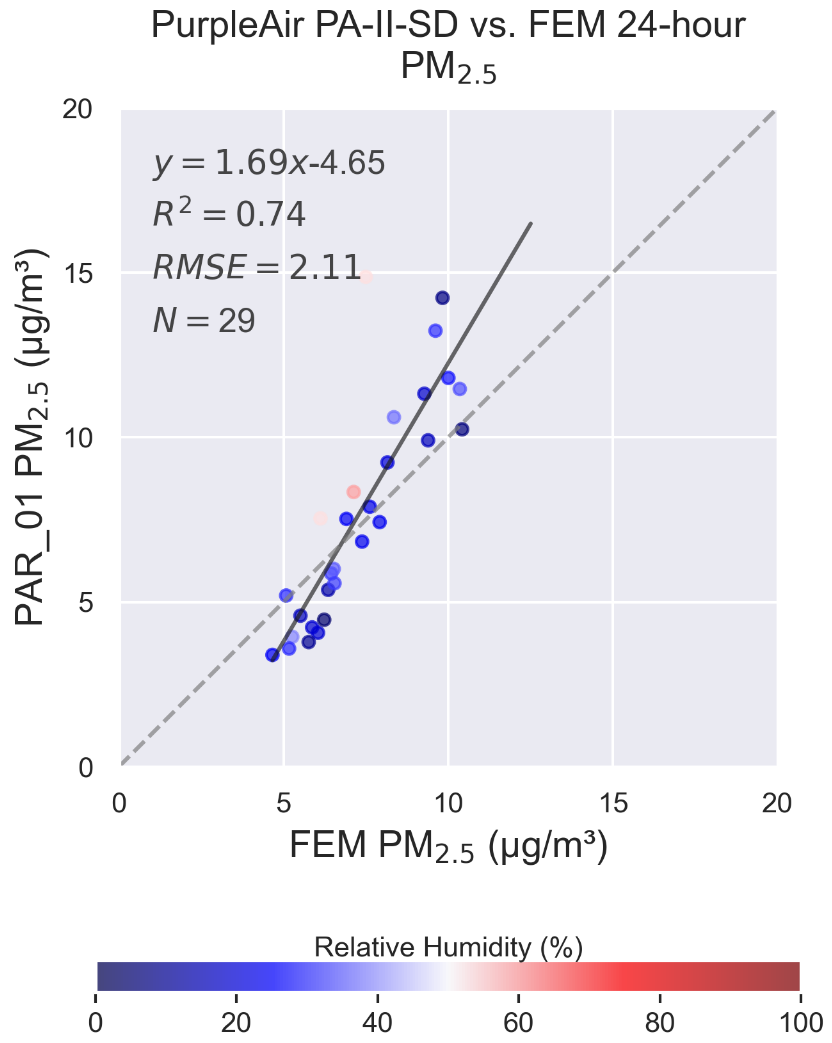

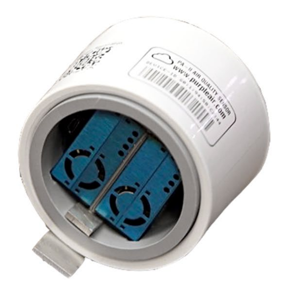

Supplemental Information

Abbreviations used in Supplemental Information

FRM	Federal Reference Method
FEM	Federal Equivalent Method
SOP	Standard Operating Procedure
QAPP	Quality Assurance Project Plan
QC	Quality Control

Supplemental Documentation	Attached	Description & URL or file path to documentation
Field observations and sensor data flags	☑	See CO-PAR-Page 7 of this testing report
Maintenance logs	☐	No logs recorded during testing
Standard operating procedure(s)	☐	U.S. EPA Office Of Research and Development SOP available upon request
Photos of equipment setup and testing	☑	See CO-PAR-Page 5 of this testing report
Product specifications sheet(s)	☑	PurpleAir: PA-II-SD Product website
Product manual(s)	☐	N/A
Data storage and transmission method	☑	See CO-PAR-Page 6 of this testing report
Data correction approach	☑	See CO-PAR-Page 6 of this testing report
Issues encountered	☑	See CO-PAR-Page 6 of this testing report
Data analysis/correction scripts and version	☑	Averaging and processing of data, calculation of performance metrics, and generation of figures and other supplementary material for analysis were obtained using Python 3.9.7 with the packages sensortoolkit v0.8.3b2, pandas 1.3.5, NumPy 1.21.2, Matplotlib 3.5.0, statsmodels 0.13.0, and seaborn 0.11.2. All packages are available from the Python Package Index (PyPI) at https://pypi.org. The integrated development environment (IDE) Spyder 5.1.5 was used for scripting and data visualization. Version control for the Python base, packages, and IDE were all managed by conda 4.11.0.
Air Monitoring Station QAPP	☐	U.S. EPA Office Of Research and Development QAPP available upon request
Summary of FRM/FEM monitor QC checks	☑	See CO-PAR-Page 8 of this testing report
Manufacturer website for FRM/FEM monitor	☑	Teledyne API: T640 Product website
FRM/FEM monitor manual	☑	Teledyne API: T640 Product Manual
FRM/FEM monitor specifications sheet(s)	☑	Teledyne API: T640 Specification Sheet
Other documents	☐	

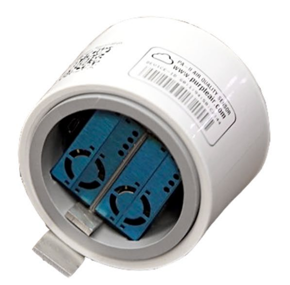

Supplemental Information: Photos of Testing Site and Equipment Setup

Site Description:

The La Casa site was established in January of 2013 as a replacement for the Denver Municipal Animal Shelter (DMAS) site when a land use change forced the relocation of the site. The La Casa location has been established as the NCore site for the Denver Metropolitan area. Measurements include trace gas/precursor-level CO analyzer, and a NO_y analyzer, in addition to the trace level SO_2, O_3, meteorology, and particulate monitors. The site represents a population-oriented neighborhood scale monitoring area.

Figure 1: PurpleAir PA-II-SD sensor (indicated by red arrow) attached to metal railing atop the sampling shelter at the monitoring site.

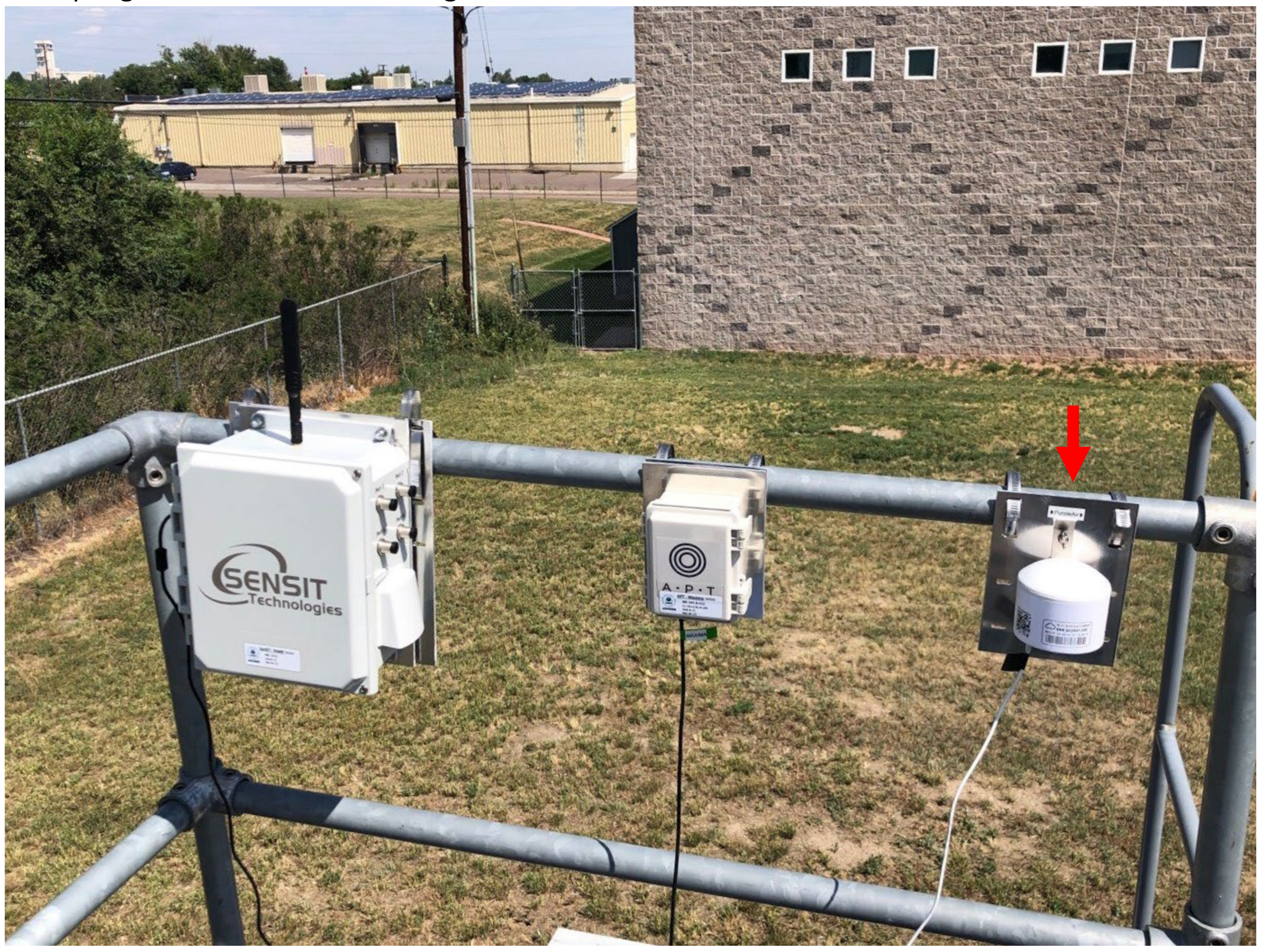

Figure 2: La Casa Monitoring Station sampling shelter, side view

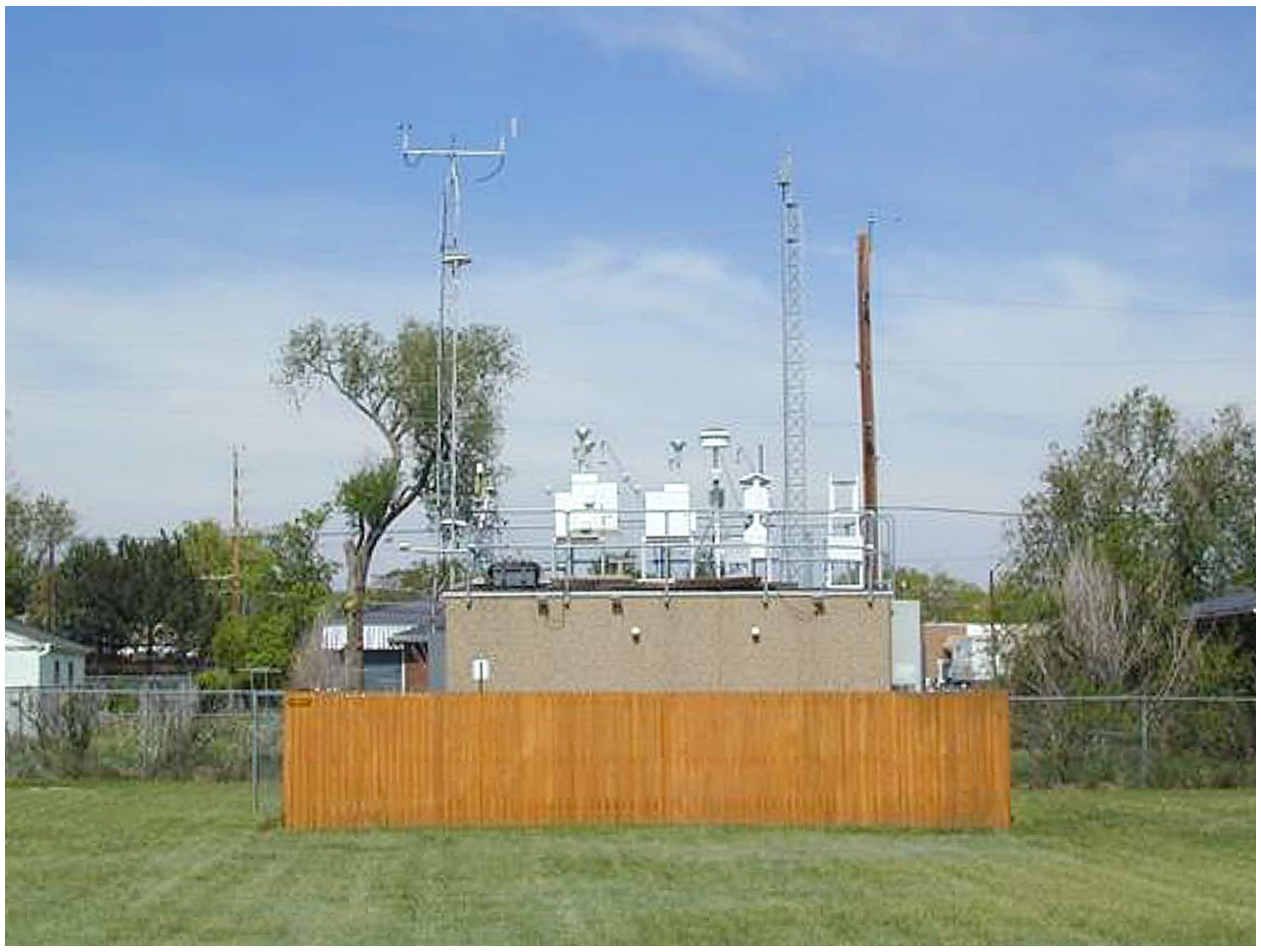

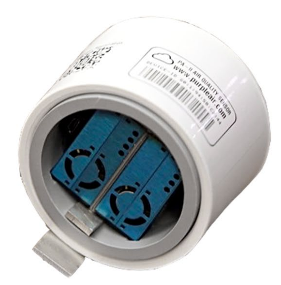

Supplemental Information: Data Storage, Correction Approach, and Issues Encountered

Data Storage and Transmission Method

The PurpleAir PA-II-SD records data at a 2-minute[1] sampling interval. Data is transmitted to the PurpleAir Cloud via Wi-Fi and stored on an internal MicroSD card. Due to the differing file structure between remote and direct download methods and the lag associated with cloud data download, weekly raw data were obtained primarily via onboard SD card. Each field site operator was provided two labeled MicroSD cards for sensor units that they used to swap out each week. Data from the collected card was then read and processed off-site.

Data Correction Approach

Data sets recorded by PurpleAir PA-II-SD contain two correction factors for PM concentrations, referred to internally as "CF=ATM" and "CF=1". PM$_{2.5}$ concentrations are recorded concurrently for each correction factor, and corresponding concentrations agree closely up to approximately 30 µg/m^3, above which one correction factor reflects concentrations roughly 1.5 times concentrations for the other correction factor. During a separate analysis by ORD, concentrations for the higher correction factor were determined to have slightly higher correlation with collocated reference measurements. As a result, data for the higher correction factor were used for this study. Prior to firmware version 5.00 (released on 11/30/2019), headers for concentrations corresponding to each correction factor were incorrectly labeled (i.e., CF=1 was labeled CF=ATM and vice versa). Testing indicated in this report was completed prior to this firmware update, and thus data sets acquired for PA-II-SD units had swapped PM$_{2.5}$ CF labels. Recorded PA-II-SD data sets indicate that the CF=ATM correction factor was the higher CF as labeled (following firmware update 5.00, this data channel corresponds to CF=1) and sensor data shown in this report correspond to this correction factor.

After acquisition, the raw data was processed using the *sensortoolkit* python code library (v0.8.3b2). A continuous data set at the recorded sampling frequency was written to a .csv file. 1-hour and 24-hour averaged data sets were generated using a 75% completeness threshold and saved as separate .csv files. Simultaneous measurement from the PurpleAir PA-II's dual PMS5003 sensors (labeled channels A and B) allows for identification of outliers resulting in large-scale disagreement between recorded values. The outlier removal method of Barkjohn et al. 2021[2] was applied to both 1-hour and 24-hour averaged A and B measurement pairs. This method includes thresholds for the absolute difference and the percent difference between hourly channel values. A-B channel averages were not included for hours where the absolute difference between channel values was greater than 5 µg/m^3 and the percent difference exceeded 70%. This outlier removal method is in use on the AirNow Fire and Smoke map and has been used for this evaluation since it requires no external information (e.g., data from the monitor) and no assumptions about typical PM2.5 concentrations (e.g., removing data over a certain threshold concentration).

Issues Encountered

<u>Pre-deployment observations</u>

- Prior to the testing period indicated in this report, the PurpleAir PA-II-SD unit was briefly collocated for a period of approximately one week at EPA's Ambient Monitoring Innovative Research Station (AIRS) in Research Triangle Park, NC. The unit was tested for proper operation, including powering on and data logging (either to the onboard SD card or via hotspot Wi-Fi connection to the PurpleAir Cloud), sampling, and acquisition of recorded data from either onboard memory or cloud storage. Units that did not pass this pre-deployment testing were returned to the manufacturer and constituted approximately 1 in 10 units tested for subsequent performance evaluation.

Field observations and sensor data flags are included on the next page of this report

[1]As of version 4.02, updated across devices on 5/31/2019. Prior to this firmware version, devices recorded measurements at 80-second intervals
[2]Barkjohn, K. K., Gantt, B., and Clements, A. L.: Development and application of a United States-wide correction for PM2.5 data collected with the PurpleAir sensor, Atmos. Meas. Tech., 14, 4617–4637, https://doi.org/10.5194/amt-14-4617-2021, 2021.

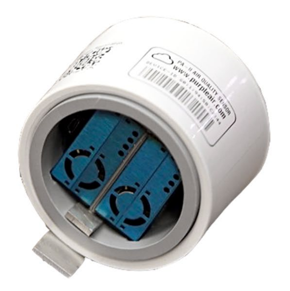

Supplemental Information: Data Storage, Correction Approach, and Issues Encountered

Field observations and sensor data flags

The following table contains data flags describing events that were encountered during the testing period.

The PurpleAir PA-II-SD was deployed at the La Casa monitoring site on 7/31/2019. At the start of the testing period, the PurpleAir recorded measurements at 80-second intervals. The sampling interval would follow a similar, yet not always consistent pattern approximately every 10 minutes, whereby the sampling interval would occasionally dip to approximately 30 seconds, followed immediately by an interval of 160 seconds. These shorter and longer intervals were subsequently followed by sampling at the configured 80-second interval. Occasionally, even longer sampling interval durations ranging from approximately 10 to 30 minutes would be logged and are indicated in the table below. Additionally, during the testing period, the PA-II-SD unit firmware was updated from v3.00 to v4.02. This update occurred on 8/15/2019 and changed the default sampling interval from 80 seconds prior to the firmware update to 120 seconds. This update also modified the characteristic variation in sampling interval as measured between consecutive recorded timestamps, with the sampling interval occasionally extending to 240 seconds. As with sampling interval variation prior to the firmware update, no discernible pattern in the frequency or occurrence of these longer sampling intervals is apparent from PA-II-SD data sets.

Start Time (UTC)	End Time (UTC)	Sensor Serial ID	Parameters Impacted	Flag
2019-08-10 22:40:00+00:00	2019-08-10 22:53:00+00:00	PAR_01	ALL	6-Sampling Interval Abnormality
2019-08-16 10:59:00+00:00	2019-08-16 11:13:00+00:00	PAR_01	ALL	6-Sampling Interval Abnormality
2019-08-20 01:50:00+00:00	2019-08-20 02:20:00+00:00	PAR_01	ALL	6-Sampling Interval Abnormality
2019-08-22 01:56:00+00:00	2019-08-22 02:02:00+00:00	PAR_01	ALL	6-Sampling Interval Abnormality
2019-08-22 23:08:00+00:00	2019-08-22 23:16:00+00:00	PAR_01	ALL	6-Sampling Interval Abnormality
2019-08-23 20:46:00+00:00	2019-08-23 20:54:00+00:00	PAR_01	ALL	6-Sampling Interval Abnormality
2019-08-24 13:06:00+00:00	2019-08-24 13:14:00+00:00	PAR_01	ALL	6-Sampling Interval Abnormality
2019-08-24 14:56:00+00:00	2019-08-24 15:04:00+00:00	PAR_01	ALL	6-Sampling Interval Abnormality
2019-08-25 11:42:00+00:00	2019-08-25 11:50:00+00:00	PAR_01	ALL	6-Sampling Interval Abnormality
2019-08-25 19:54:00+00:00	2019-08-25 20:02:00+00:00	PAR_01	ALL	6-Sampling Interval Abnormality
2019-08-26 11:42:00+00:00	2019-08-26 11:50:00+00:00	PAR_01	ALL	6-Sampling Interval Abnormality
2019-08-26 19:56:00+00:00	2019-08-26 20:04:00+00:00	PAR_01	ALL	6-Sampling Interval Abnormality
2019-08-27 11:42:00+00:00	2019-08-27 11:50:00+00:00	PAR_01	ALL	6-Sampling Interval Abnormality
2019-08-27 19:52:00+00:00	2019-08-27 20:00:00+00:00	PAR_01	ALL	6-Sampling Interval Abnormality
2019-08-30 09:45:00+00:00	2019-08-30 09:59:00+00:00	PAR_01	ALL	6-Sampling interval abnormality
2019-08-31 13:13:00+00:00	2019-08-31 13:19:00+00:00	PAR_01	ALL	6-Sampling interval abnormality
2019-09-01 04:09:00+00:00	2019-09-01 04:17:00+00:00	PAR_01	ALL	6-Sampling interval abnormality
2019-09-01 22:11:00+00:00	2019-09-01 22:31:00+00:00	PAR_01	ALL	6-Sampling interval abnormality

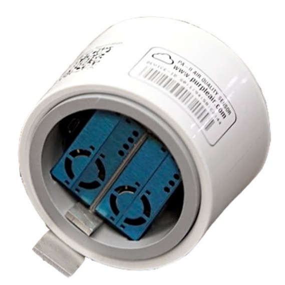

Description of Data Flags

AQS

The U.S. EPA's Air Quality System (AQS) is the Agency's primary ambient air monitoring data archive. A comprehensive list of data flags that are recorded alongside AQS data sets, referred to by U.S. EPA as 'qualifiers', can be found at the following link: https://aqs.epa.gov/aqsweb/documents/codetables/qualifiers.html

Invalidation of reference data: AQS qualifiers are organized by qualifier type, which indicates whether data logged alongside qualifier flags should be invalidated (set null). Qualifiers with type "Null Data Qualifier" are invalidated, and includes data logged during periods that coincide with QC checks (e.g., "BF-Precision/Zero/Span", "BJ- Operator Error", "BL - QA Audit", "AZ - QC Audit") among other events such as power outages. Data logged alongside qualifiers with type "Quality Assurance Qualifiers" are not invalidated and are included in this analysis (e.g., concentrations less than the federal MDL for the reference monitor "MD – Value less than MDL", QA reviewed values "Validated Value").

Data Flags Recorded During Testing

FRM/FEM Monitor	Timestamp (UTC)	Flag
Teledyne API T640 (Acquired via AQS)	2019-08-16 15:00:00+0000 to 2019-08-16 16:00:00+0000	BA - Maintenance/Routine Repairs
	2019-08-28 14:00:00+0000 to 2019-08-28 15:00:00+0000	BA - Maintenance/Routine Repairs

Testing Report - PM$_{2.5}$ Base Testing

SENSIT RAMP

This report reflects out-of-the-box performance

Initial Base Testing - Denver, CO
U.S. Environmental Protection Agency
Office of Research and Development
PI: Clements.Andrea@epa.gov
919-541-1363
August 2019—September 2019

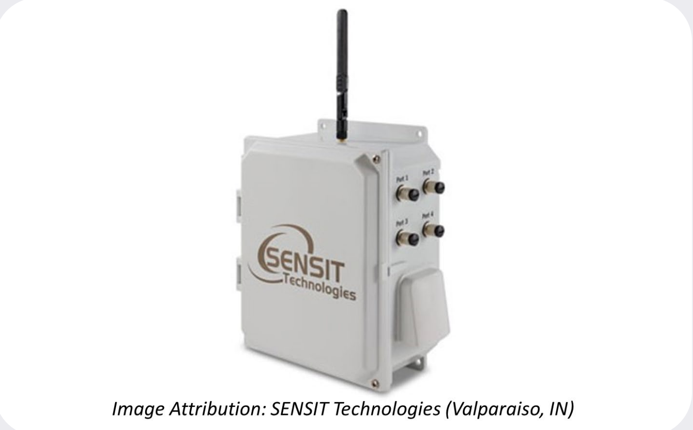

Image Attribution: SENSIT Technologies (Valparaiso, IN)

Deployment Details

Testing Organization and Site Information	
Testing organization (Name, Organization type, Contact website)	U.S. Environmental Protection Agency - Office of Research and Development<br>Federal Government<br>Air Sensor Toolbox | U.S. EPA Website
Testing location (City, State, Latitude and Longitude)	La Casa<br>Denver, CO<br>39.779429, -105.005174
AQS site ID	08 - 031 - 0026
Sampling timeframe (MM-DD-YY)	08-14-19 to 09-11-19
Sensor data source	Onboard MicroSD card
Reference data source	AQS API download

Sensor Information			
Manufacturer, model	SENSIT RAMP		
Device firmware version	190313_AQ_v9.30		
Sampling time interval	15-seconds		
Sensor serial numbers	RAM_01		
Issues encountered during deployment?	☐	No Issues	

FRM/FEM Information	
Manufacturer, model, designation	Teledyne Advanced Pollution Instrumentation T640 FEM
Sampling time interval	1-hour averaging
Date of calibration	As required by 40 CFR Part 58 and the CO Regional Monitoring Site QAPP maintained by CDPHE
Date of flowrate verification check	Monthly as required by 40 CFR Part 58 Appendix A
Description, date(s) of maintenance activities	See CO-RAM-Page 7 of this testing report

Time Series Plots

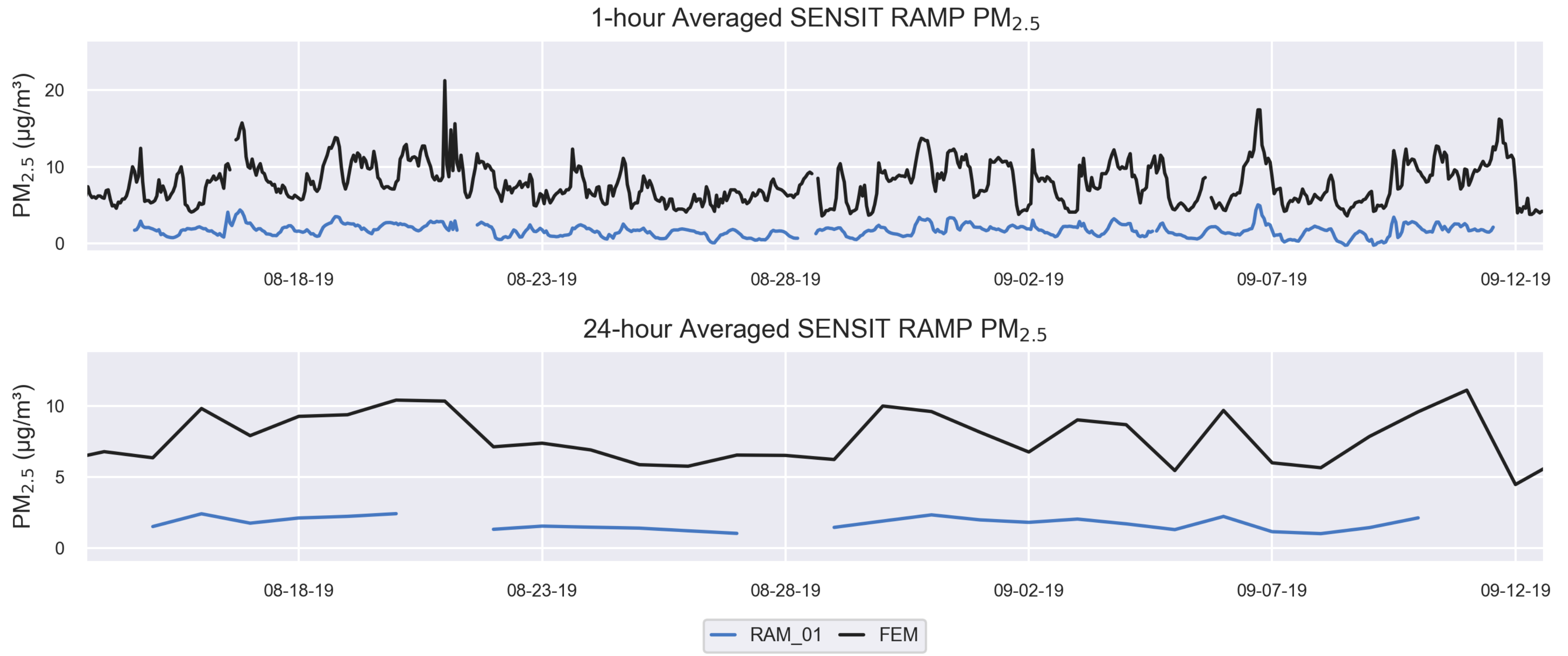

Scatter Plots: Comparison to FRM/FEM

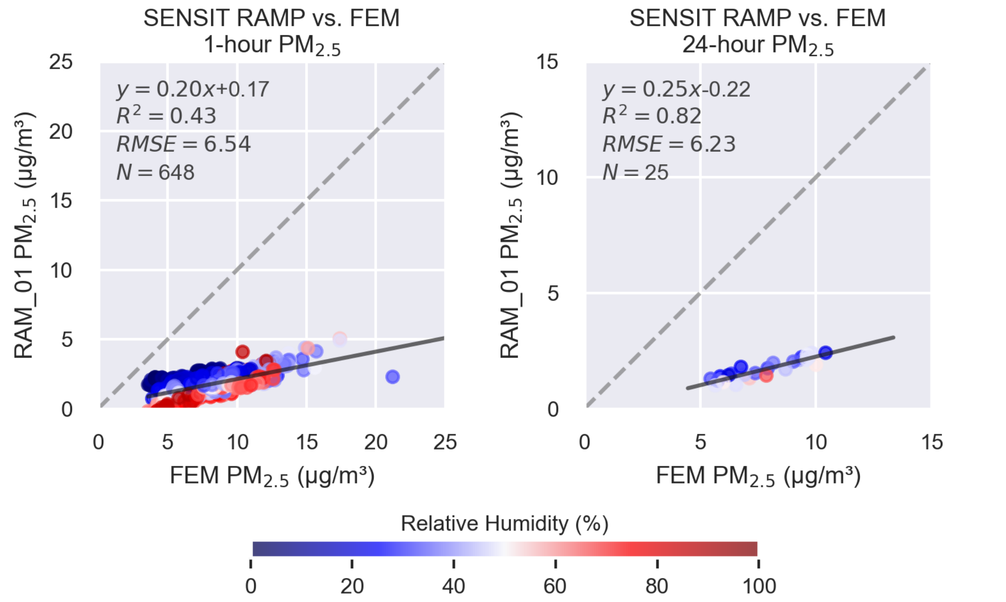

Range of FRM/FEM concentrations over duration of base test (µg/m³)	3.6-21.2 (1-hr),<br>5.5-11.1 (24-hr)
Number of 24-hr periods in FRM/FEM monitor measurements with a goal concentration ≥ 25 µg/m³	0

Performance Metrics

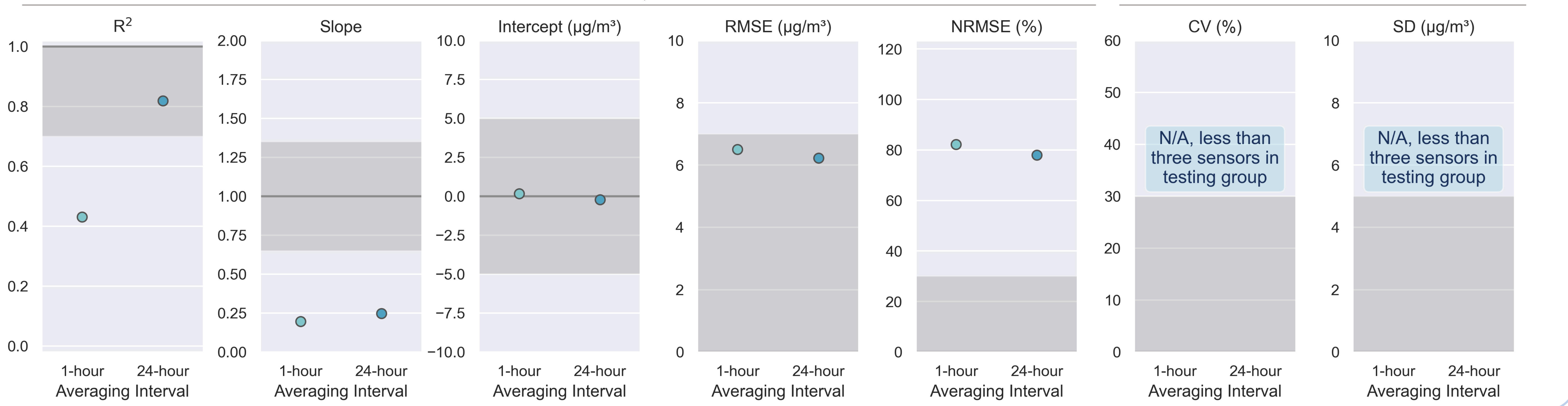

Meteorological Conditions During Deployment

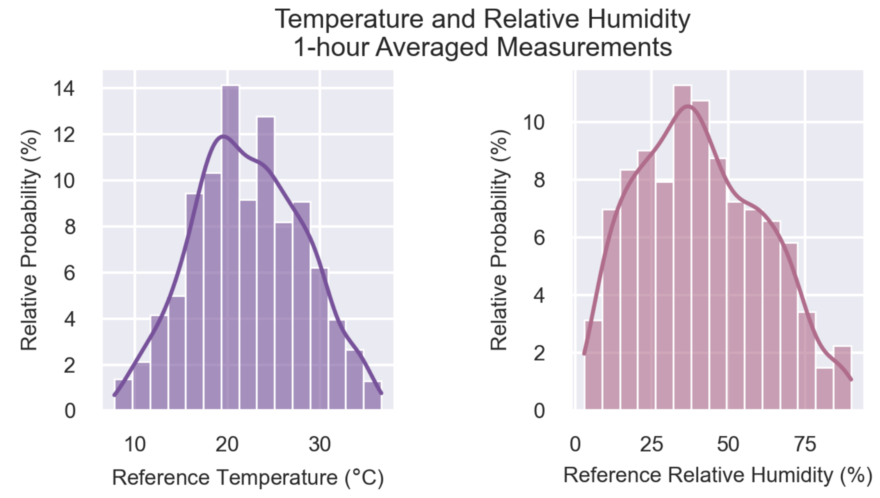

Number of 24-hr periods outside sensor manufacture-listed temperature operational range (-20 to 50 °C)	0
Number of 24-hr periods outside sensor manufacture-listed relative humidity operational range (no operational range specified)	-

Meteorological Influence

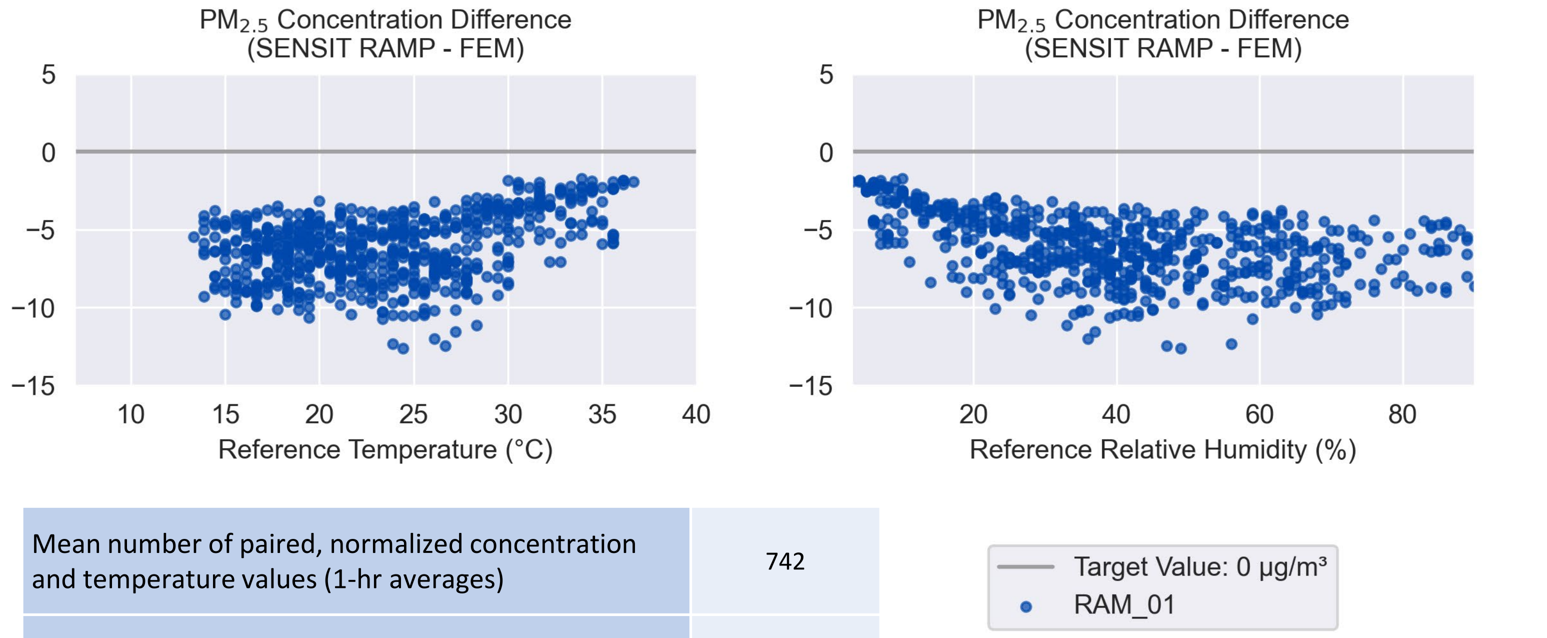

Mean number of paired, normalized concentration and temperature values (1-hr averages)	742
Mean number of paired, normalized concentration and relative humidity values (1-hr averages)	742

Testing Report - $PM_{2.5}$ Base Testing

SENSIT RAMP

This report reflects out-of-the-box performance

Initial Base Testing - Denver, CO

U.S. Environmental Protection Agency
Office of Research and Development
PI: Clements.Andrea@epa.gov
919-541-1363

August 2019—September 2019

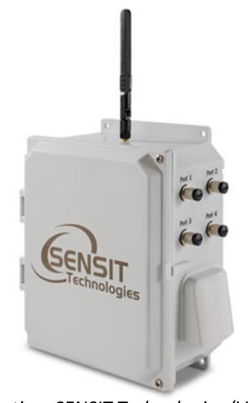

Image Attribution: SENSIT Technologies (Valparaiso, IN)

Tabular Statistics

Sensor-FRM/FEM Correlation

	Bias and Linearity						Data Quality			
	R^2		Slope		Intercept (µg/m³)		Uptime (%)		Number of paired sensor and FRM/FEM concentration values	
	1-Hour ○	24-Hour ●	1-Hour ○	24-Hour ○	1-Hour ●	24-Hour ●	1-Hour ●	24-Hour ●	1-Hour	24-Hour
Metric Target Range	≥ 0.70	≥ 0.70	1.0 ± 0.35	1.0 ± 0.35	-5 ≤ b ≤ 5	-5 ≤ b ≤ 5	75%*	75%*	-	-
Sensor RAM_01	0.43	0.82	0.20	0.25	0.17	-0.22	97	89	648	25

	Error			
	RMSE (µg/m³)		NRMSE (%)	
	1-Hour ★	24-Hour ★	1-Hour ☆	24-Hour ☆
Metric Target Range	≤ 7.0	≤ 7.0	≤ 30.0	≤ 30.0
Deployment Value	6.5	6.2	82.2	78.0

Device-specific metrics (computed for each sensor in evaluation)
○○○ Metric value for none of devices tested falls within the target range
●○○ Metric value for one of devices tested falls within the target range
●●○ Metric value for two of devices tested falls within the target range
●●● Metric value for three of devices tested falls within the target range

Single-valued metrics (computed via entire evaluation dataset)
☆ Indicates that the metric value is not within the target range
★ Indicates that the metric value is within the target range

Sensor-Sensor Precision[1]

	Precision (between collocated sensors)				Data Quality	
	CV (%)		SD (µg/m³)		Number of concurrent sensor concentration pairs	
	1-Hour ☆	24-Hour ☆	1-Hour ☆	24-Hour ☆	1-Hour	24-Hour
Metric Target Range	≤ 30.0	≤ 30.0	≤ 5.0	≤ 5.0	-	-
Deployment Value	-	-	-	-	-	-

[1]Precision statistics are computed for evaluations with at least three collocated sensor units. Metric values are left blank for evaluations with two or fewer sensor units.

*This value is only a recommendation for ensuring data quality and is not included in the list of target values discussed in Section 4 of the Performance Testing Protocols, Metrics, and Target Values for Fine Particulate Matter Air Sensors document.

Testing Report - $PM_{2.5}$ Base Testing

SENSIT RAMP

This report reflects out-of-the-box performance

Initial Base Testing - Denver, CO

U.S. Environmental Protection Agency
Office of Research and Development
PI: Clements.Andrea@epa.gov
919-541-1363

August 2019—September 2019

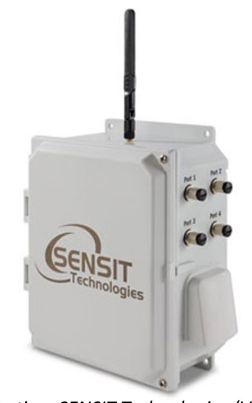

Image Attribution: SENSIT Technologies (Valparaiso, IN)

Sensor-FRM/FEM Scatter Plots

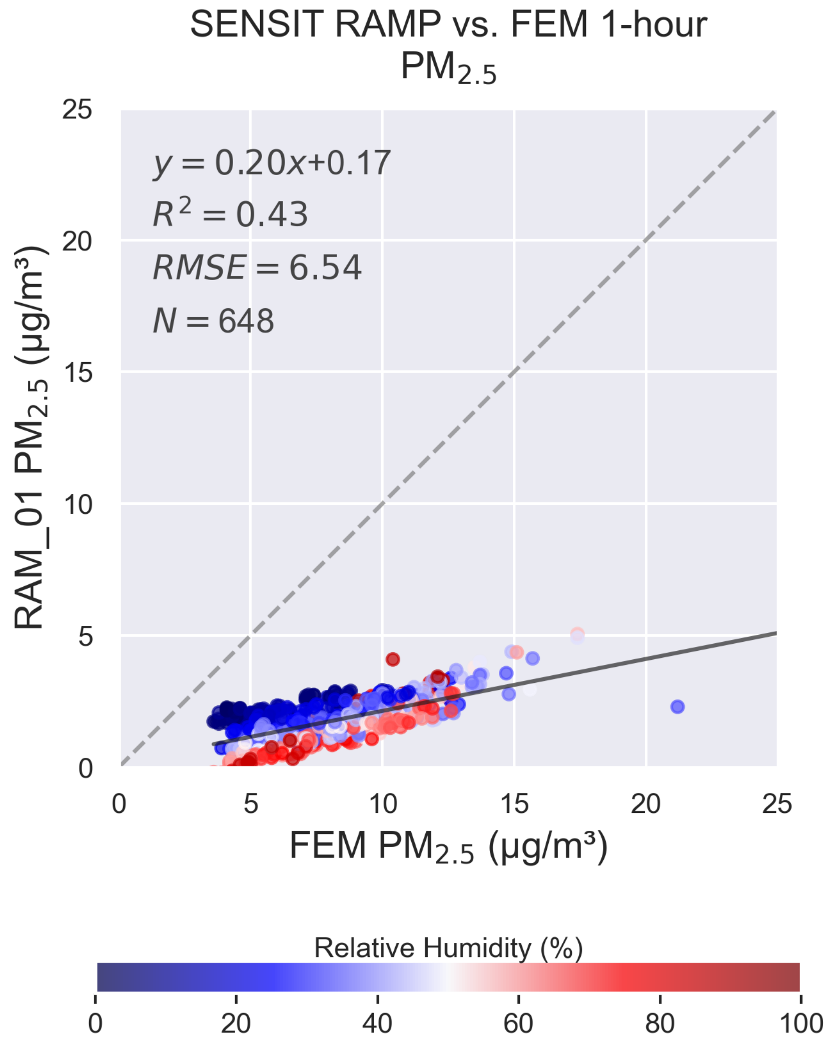

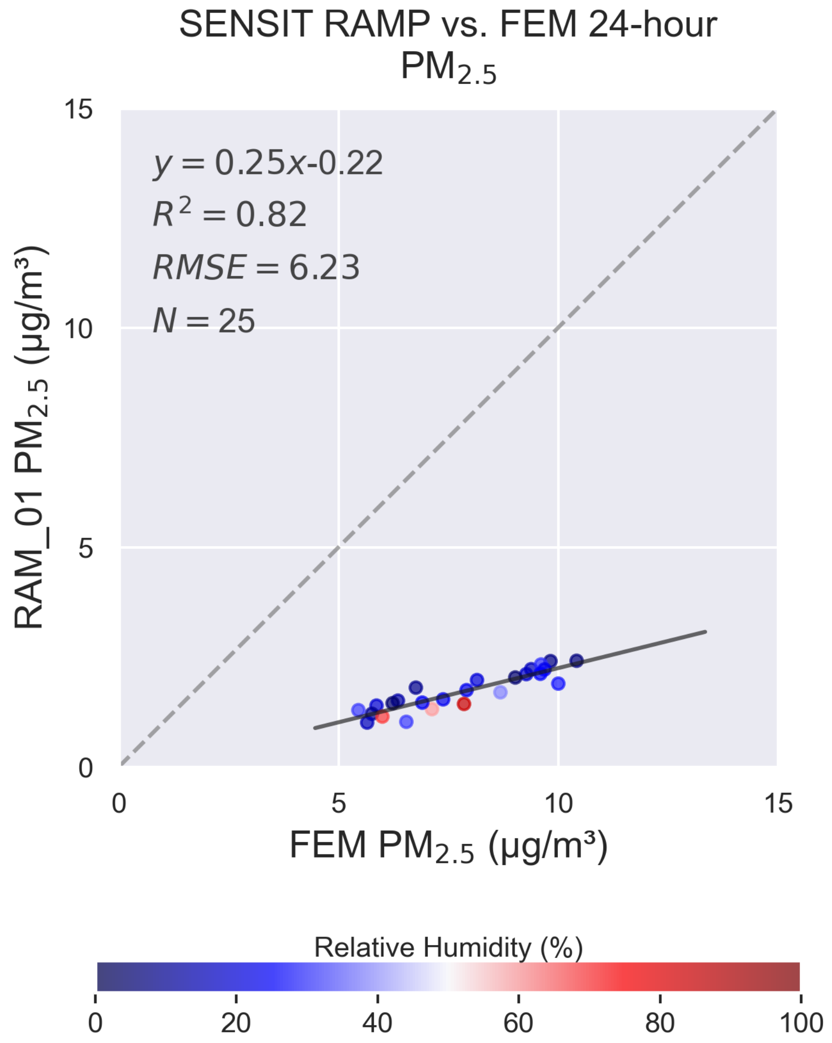

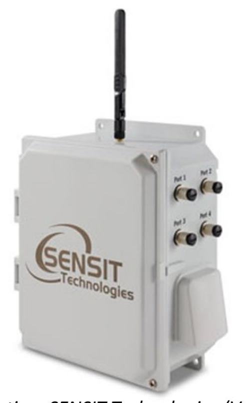

Image Attribution: SENSIT Technologies (Valparaiso, IN)

Supplemental Information

Abbreviations used in Supplemental Information

FRM	Federal Reference Method
FEM	Federal Equivalent Method
SOP	Standard Operating Procedure
QAPP	Quality Assurance Project Plan
QC	Quality Control

Supplemental Documentation	Attached	Description & URL or file path to documentation
Field observations and sensor data flags	☑	See CO-RAM-Page 6 of this testing report
Maintenance logs	☐	No logs recorded during testing
Standard operating procedure(s)	☐	U.S. EPA Office Of Research and Development SOP available upon request
Photos of equipment setup and testing	☑	See CO-RAM-Page 5 of this testing report
Product specifications sheet(s)	☑	SENSIT Technologies: RAMP Product Brochure
Product manual(s)	☑	SENSIT Technologies: RAMP Product Manual
Data storage and transmission method	☑	See CO-RAM-Page 6 of this testing report
Data correction approach	☑	See CO-RAM-Page 6 of this testing report
Issues encountered	☑	See CO-RAM-Page 6 of this testing report. No issues were encountered during testing; however, various issues were faced during the pre-deployment phase.
Data analysis/correction scripts and version	☑	Averaging and processing of data, calculation of performance metrics, and generation of figures and other supplementary material for analysis were obtained using Python 3.9.7 with the packages sensortoolkit v0.8.3b2, pandas 1.3.5, NumPy 1.21.2, Matplotlib 3.5.0, statsmodels 0.13.0, and seaborn 0.11.2. All packages are available from the Python Package Index (PyPI) at https://pypi.org. The integrated development environment (IDE) Spyder 5.1.5 was used for scripting and data visualization. Version control for the Python base, packages, and IDE were all managed by conda 4.11.0.
Air Monitoring Station QAPP	☐	U.S. EPA Office Of Research and Development QAPP available upon request
Summary of FRM/FEM monitor QC checks	☑	See CO-RAM-Page 7 of this testing report
Manufacturer website for FRM/FEM monitor	☑	Teledyne API: T640 Product website
FRM/FEM monitor manual	☑	Teledyne API: T640 Product Manual
FRM/FEM monitor specifications sheet(s)	☑	Teledyne API: T640 Specification Sheet
Other documents	☐	

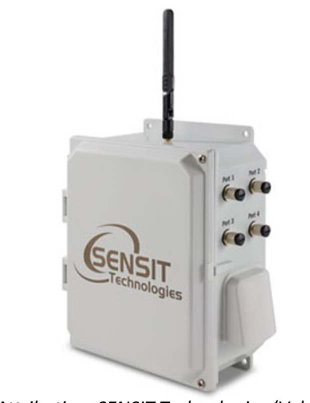

Image Attribution: SENSIT Technologies (Valparaiso, IN)

Supplemental Information: Photos of Testing Site and Equipment Setup

Site Description:

The La Casa site was established in January of 2013 as a replacement for the Denver Municipal Animal Shelter (DMAS) site when a land use change forced the relocation of the site. The La Casa location has been established as the NCore site for the Denver Metropolitan area. Measurements include trace gas/precursor-level CO analyzer, and a NO_y analyzer, in addition to the trace level SO_2, O_3, meteorology, and particulate monitors. The site represents a population-oriented neighborhood scale monitoring area.

Figure 1: SENSIT RAMP sensor (indicated by red arrow) attached to metal railing atop the sampling shelter at the monitoring site.

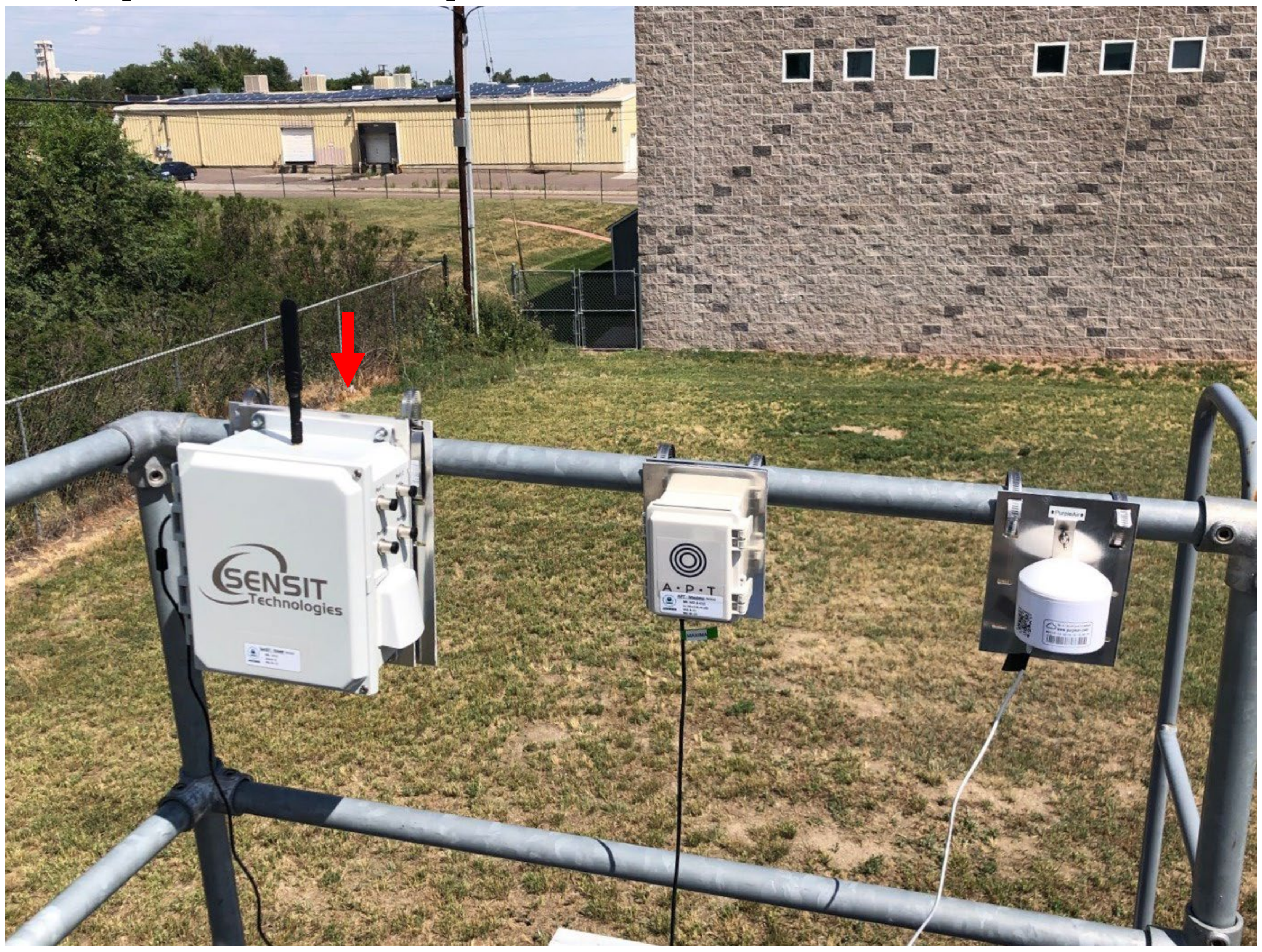

Figure 2: La Casa Monitoring Station sampling shelter, side view

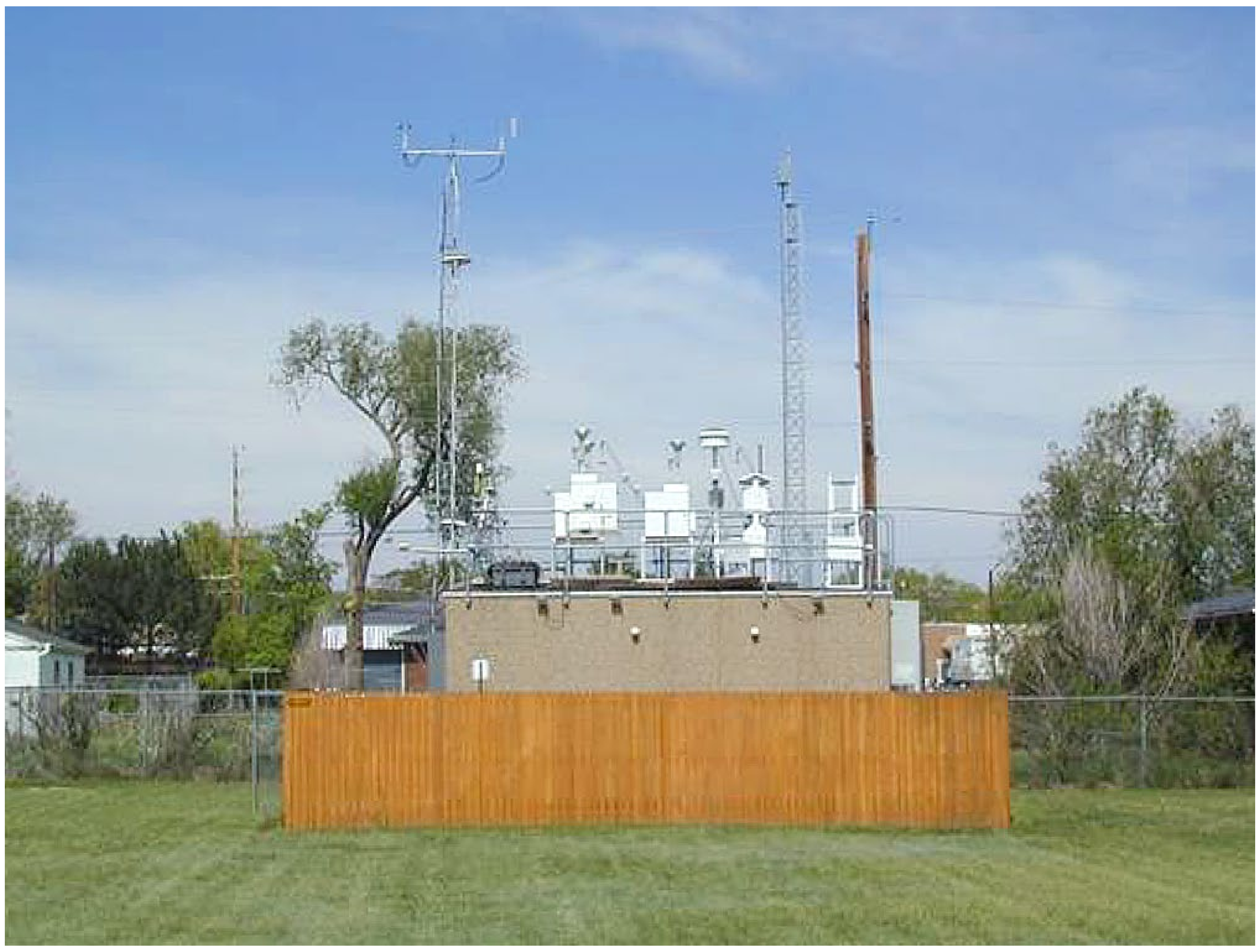

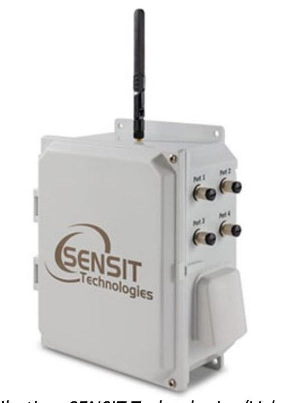

Image Attribution: SENSIT Technologies (Valparaiso, IN)

Data Storage and Transmission Method

The SENSIT RAMP was configured to record data at a 15-second sampling interval. Data are stored as daily text files (.txt format) on an onboard MicroSD card. Data files were obtained weekly via SD cards. Each field site operator was provided two labeled MicroSD cards for sensor units that they used to swap out each week. Data from the collected card was then read and processed off-site.

Data Correction Approach

SENSIT RAMP units were pre-configured by the manufacturer with a linear correction (i.e., concentration gain = 1.0 and offset = 0.0 µg m^{-3}). These presets reflect out-of-the box performance and were not modified by EPA prior or during testing.

After acquisition, the raw data was processed using the *sensortoolkit* python code library (v0.8.3b2). A continuous data set at the recorded sampling frequency was written to a .csv file. 1-hour and 24-hour averaged data sets were generated using a 75% completeness threshold and saved as separate .csv files. Outliers were NOT removed from data sets in order to assess “out-of-the-box” sensor performance.

Issues Encountered

Pre-deployment observations

- SENSIT RAMP units were received without documentation or manuals. After communicating the need to change default settings (logging interval and time zone) with the manufacturer, a draft user’s manual and a USB cable were supplied. With the use of this USB cable, instrument settings could be changed, and real-time data could be logged using a serial communication software (CoolTerm, v.1.5.0). Because the sensor did not record data at the top of every minute, the RAMP was configured to record data at 15-second intervals so that the data could be averaged more closely to complete minutes. Prior to deployment, RAMP units were collocated in a bench-top evaluation to verify operational status and determine the extent of data invalidity (i.e., determine equilibration period) after an initial start-up event. The recorded response for parameters measured by the RAMP suggests that the gas sensors (CO, NO, NO_2, O_3) required approximately a 2-hour equilibration period, while the remaining sensors (temperature, relative humidity, particulate matter) did not require any equilibration period.

Field observations and sensor data flags

The SENSIT RAMP was deployed at the La Casa monitoring site on 7/31/2019. The RAMP unit operated nominally during the testing period and did not require replacement or repair.

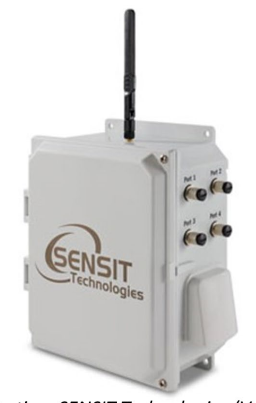

Image Attribution: SENSIT Technologies (Valparaiso, IN)

Supplemental Information: Description of FRM/FEM QC Checks and Data Flags

Description of Data Flags

AQS

The U.S. EPA's Air Quality System (AQS) is the Agency's primary ambient air monitoring data archive. A comprehensive list of data flags that are recorded alongside AQS data sets, referred to by U.S. EPA as 'qualifiers', can be found at the following link: https://aqs.epa.gov/aqsweb/documents/codetables/qualifiers.html

Invalidation of reference data: AQS qualifiers are organized by qualifier type, which indicates whether data logged alongside qualifier flags should be invalidated (set null). Qualifiers with type "Null Data Qualifier" are invalidated, and includes data logged during periods that coincide with QC checks (e.g., "BF-Precision/Zero/Span", "BJ- Operator Error", "BL - QA Audit", "AZ - QC Audit") among other events such as power outages. Data logged alongside qualifiers with type "Quality Assurance Qualifiers" are not invalidated and are included in this analysis (e.g., concentrations less than the federal MDL for the reference monitor "MD – Value less than MDL", QA reviewed values "Validated Value").

Data Flags Recorded During Testing

FRM/FEM Monitor	Timestamp (UTC)	Flag
Teledyne API T640 (Acquired via AQS)	2019-08-16 15:00:00+0000 to 2019-08-16 16:00:00+0000	BA - Maintenance/Routine Repairs
	2019-08-28 14:00:00+0000 to 2019-08-28 15:00:00+0000	BA - Maintenance/Routine Repairs
	2019-09-05 16:00:00+0000 to 2019-09-05 17:00:00+0000	BL - QA Audit

Meteorological Instrument	Timestamp (UTC)	Flag
MetOne Temperature Monitor (Acquired via AQS)	2019-09-13 17:00:00+0000 to 2019-09-13 18:00:00+0000	AZ - QC Audit
Climatronics Relative Humidity Monitor (Acquired via AQS)	2019-09-13 17:00:00+0000 to 2019-09-13 18:00:00+0000	AZ - QC Audit

Testing Report - $PM_{2.5}$ Base Testing

QuantAQ ARISense

This report reflects out-of-the-box performance

Initial Base Testing - Denver, CO
U.S. Environmental Protection Agency
Office of Research and Development
PI: Clements.Andrea@epa.gov
919-541-1363
September 2020—October 2020

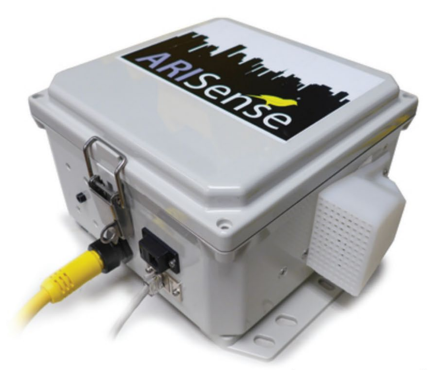

Image Attribution: QuantAQ, Inc (Somerville, MA)

Deployment Details

Testing Organization and Site Information	
Testing organization (Name, Organization type, Contact website)	U.S. Environmental Protection Agency - Office of Research and Development<br>Federal Government<br>Air Sensor Toolbox | U.S. EPA Website
Testing location (City, State, Latitude and Longitude)	La Casa<br>Denver, CO<br>39.779429, -105.005174
AQS site ID	08 - 031 - 0026
Sampling timeframe (MM-DD-YY)	09-17-20 to 10-17-20
Sensor data source	QuantAQ Cloud download
Reference data source	AQS API download

Sensor Information			
Manufacturer, model	QuantAQ ARISense		
Device firmware version	Received by EPA on 4/30/2019		
Sampling time interval	1-minute		
Sensor serial numbers	ARS_01		
Issues encountered during deployment?	☑	See CO-ARS-Page 6 of this testing report	

FRM/FEM Information	
Manufacturer, model, designation	Teledyne Advanced Pollution Instrumentation T640 FEM
Sampling time interval	1-hour averaging
Date of calibration	As required by 40 CFR Part 58 and the CO Regional Monitoring Site QAPP maintained by CDPHE
Date of flowrate verification check	Monthly as required by 40 CFR Part 58 Appendix A
Description, date(s) of maintenance activities	See CO-ARS-Page 8 of this testing report

Time Series Plots

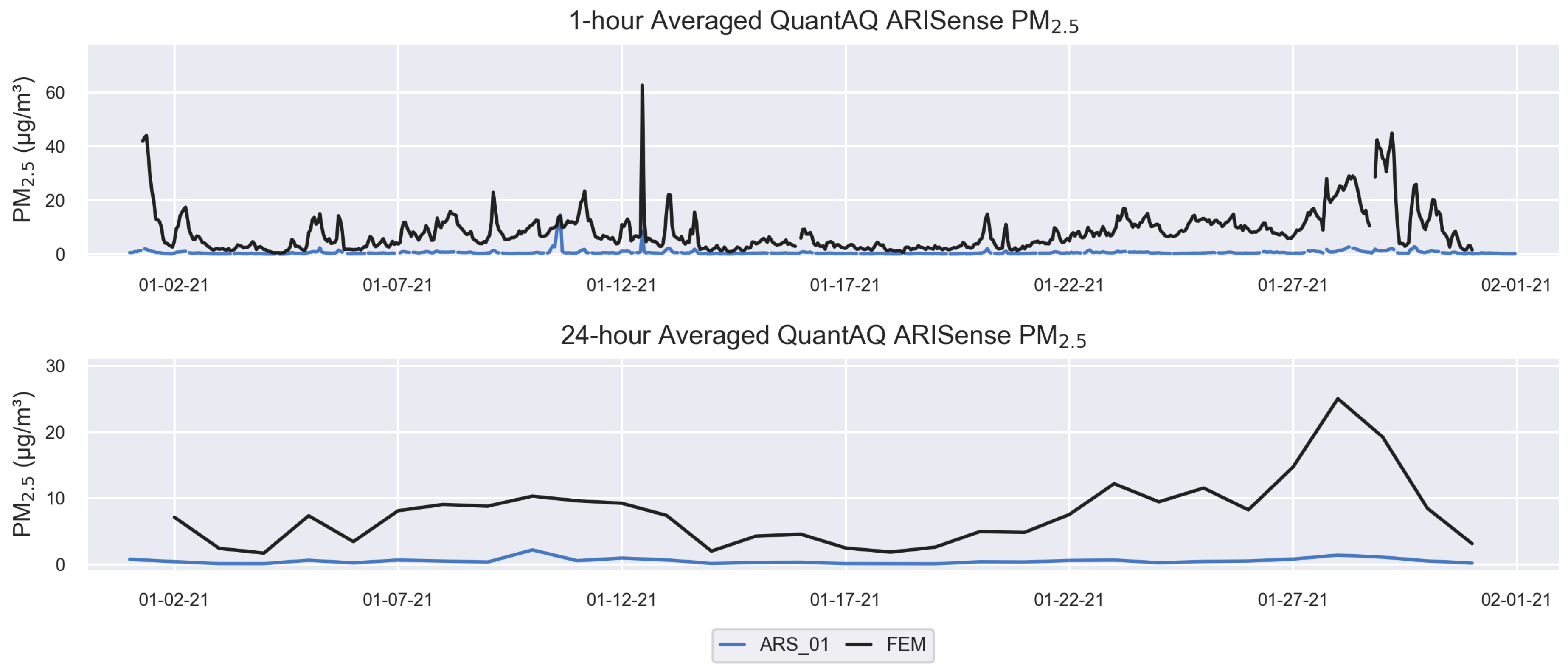

Scatter Plots: Comparison to FRM/FEM

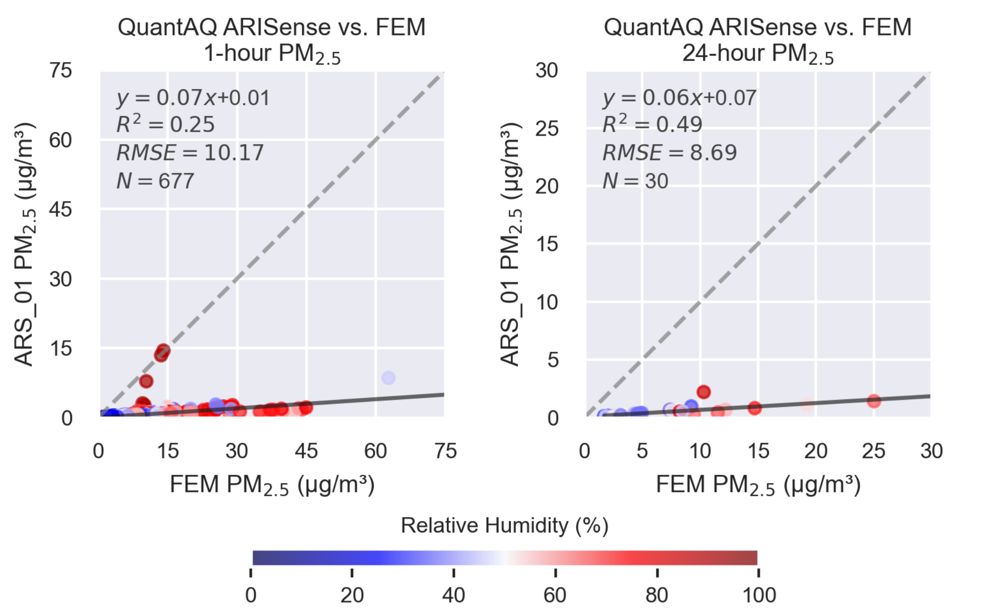

Range of FRM/FEM concentrations over duration of base test (µg/m³)	1.8-206.5 (1-hr), 4.2-44.3 (24-hr)
Number of 24-hr periods in FRM/FEM monitor measurements with a goal concentration ≥ 25 µg/m³	7

Performance Metrics

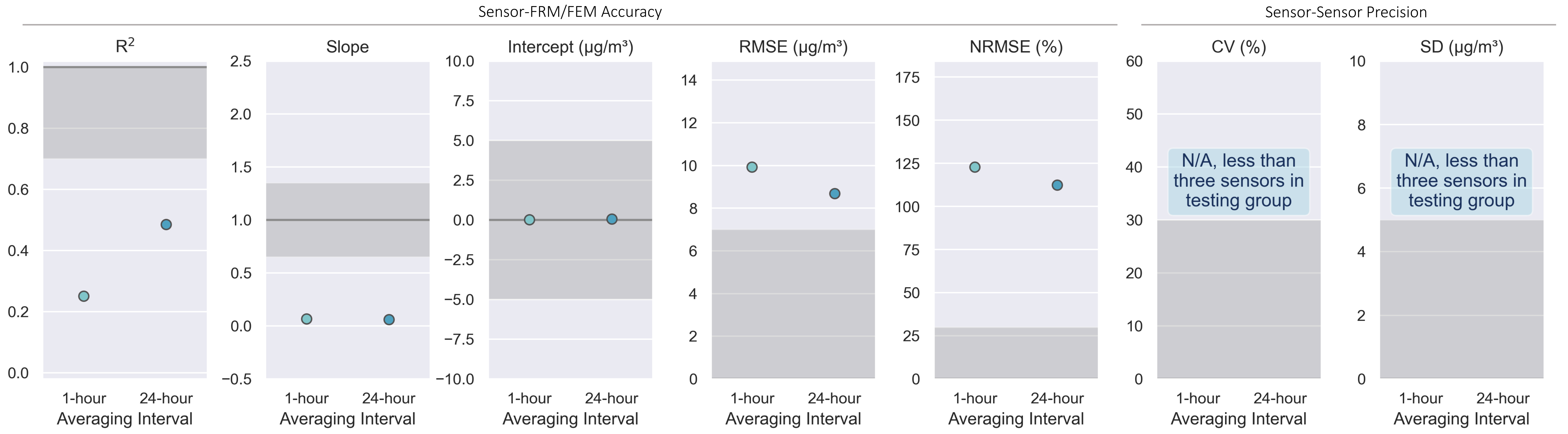

Meteorological Conditions During Deployment

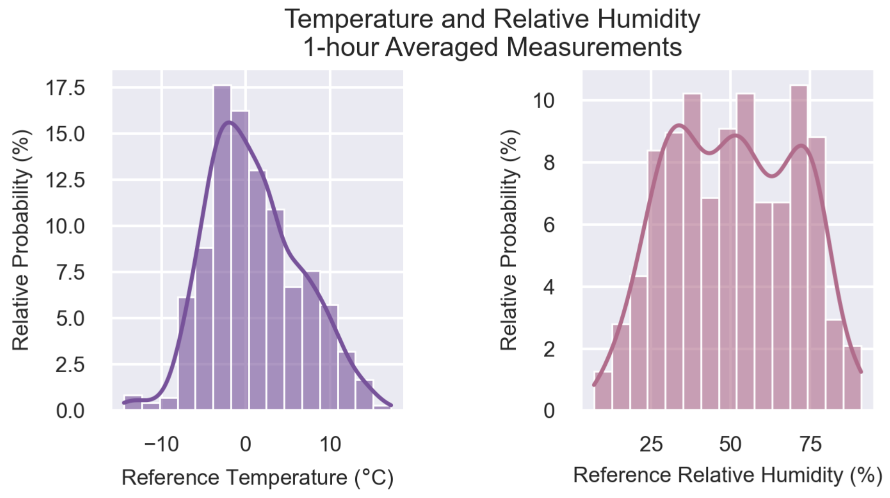

Number of 24-hr periods outside sensor manufacture-listed temperature operational range (no operational range specified)	-
Number of 24-hr periods outside sensor manufacture-listed relative humidity operational range (no operational range specified)	-

Meteorological Influence

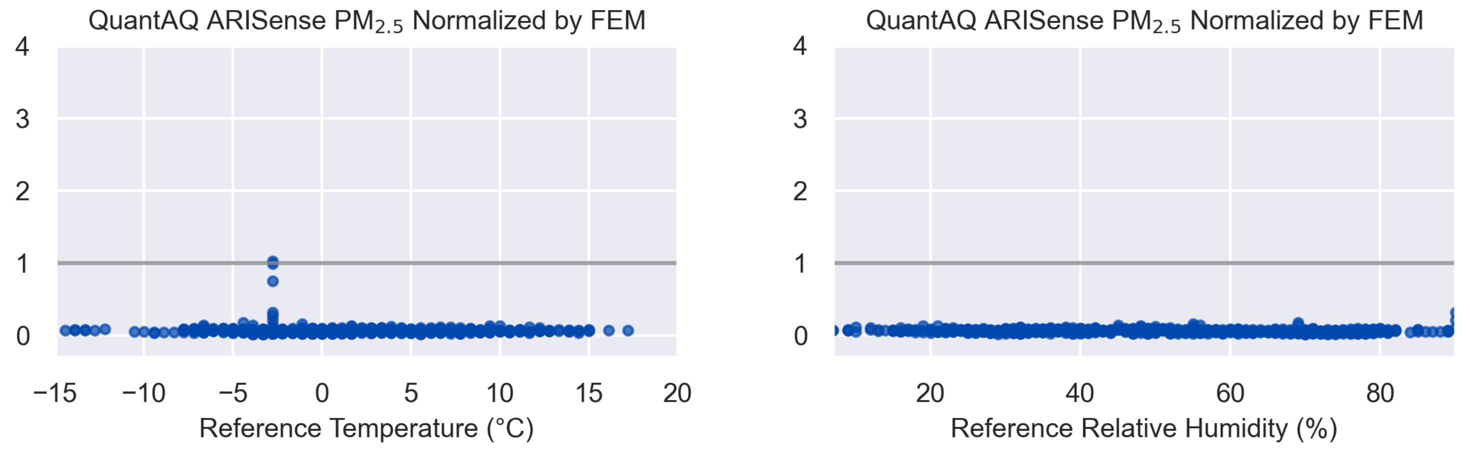

Mean number of paired, normalized concentration and temperature values (1-hr averages)	744
Mean number of paired, normalized concentration and relative humidity values (1-hr averages)	744

Testing Report - $PM_{2.5}$ Base Testing

QuantAQ ARISense

This report reflects out-of-the-box performance

Initial Base Testing - Denver, CO
U.S. Environmental Protection Agency
Office of Research and Development
PI: Clements.Andrea@epa.gov
919-541-1363
September 2020—October 2020

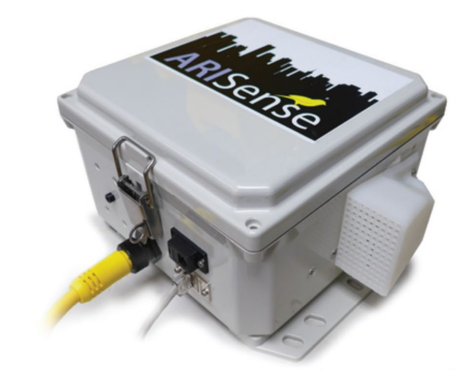

Image Attribution: QuantAQ, Inc (Somerville, MA)

Tabular Statistics

Sensor-FRM/FEM Correlation

	Bias and Linearity						Data Quality			
	R^2		Slope		Intercept (µg/m³)		Uptime (%)		Number of paired sensor and FRM/FEM concentration values	
	1-Hour ○	24-Hour ○	1-Hour ○	24-Hour ○	1-Hour ●	24-Hour ●	1-Hour ●	24-Hour ●	1-Hour	24-Hour
Metric Target Range	≥ 0.70	≥ 0.70	1.0 ± 0.35	1.0 ± 0.35	-5 ≤ b ≤ 5	-5 ≤ b ≤ 5	75%*	75%*	-	-
Sensor ARS_01	0.25	0.49	0.07	0.06	0.01	0.07	96	100	677	30

	Error			
	RMSE (µg/m³)		NRMSE (%)	
	1-Hour ☆	24-Hour ☆	1-Hour ☆	24-Hour ☆
Metric Target Range	≤ 7.0	≤ 7.0	≤ 30.0	≤ 30.0
Deployment Value	9.9	8.7	122.9	112.4

Device-specific metrics (computed for each sensor in evaluation)
○○○ Metric value for none of devices tested falls within the target range
●○○ Metric value for one of devices tested falls within the target range
●●○ Metric value for two of devices tested falls within the target range
●●● Metric value for three of devices tested falls within the target range

Single-valued metrics (computed via entire evaluation dataset)
☆ Indicates that the metric value is not within the target range
★ Indicates that the metric value is within the target range

Sensor-Sensor Precision[1]

	Precision (between collocated sensors)				Data Quality	
	CV (%)		SD (µg/m³)		Number of concurrent sensor concentration pairs	
	1-Hour ☆	24-Hour ☆	1-Hour ☆	24-Hour ☆	1-Hour	24-Hour
Metric Target Range	≤ 30.0	≤ 30.0	≤ 5.0	≤ 5.0	-	-
Deployment Value	-	-	-	-	-	-

[1]Precision statistics are computed for evaluations with at least three collocated sensor units. Metric values are left blank for evaluations with two or fewer sensor units.

*This value is only a recommendation for ensuring data quality and is not included in the list of target values discussed in Section 4 of the Performance Testing Protocols, Metrics, and Target Values for Fine Particulate Matter Air Sensors document.

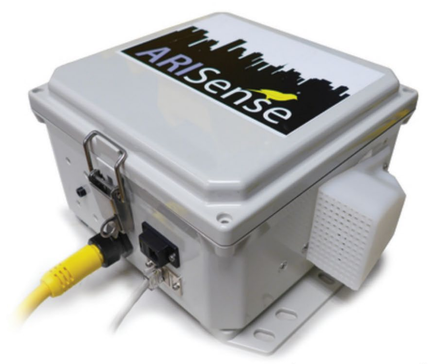

Image Attribution: QuantAQ, Inc (Somerville, MA)

Sensor-FRM/FEM Scatter Plots

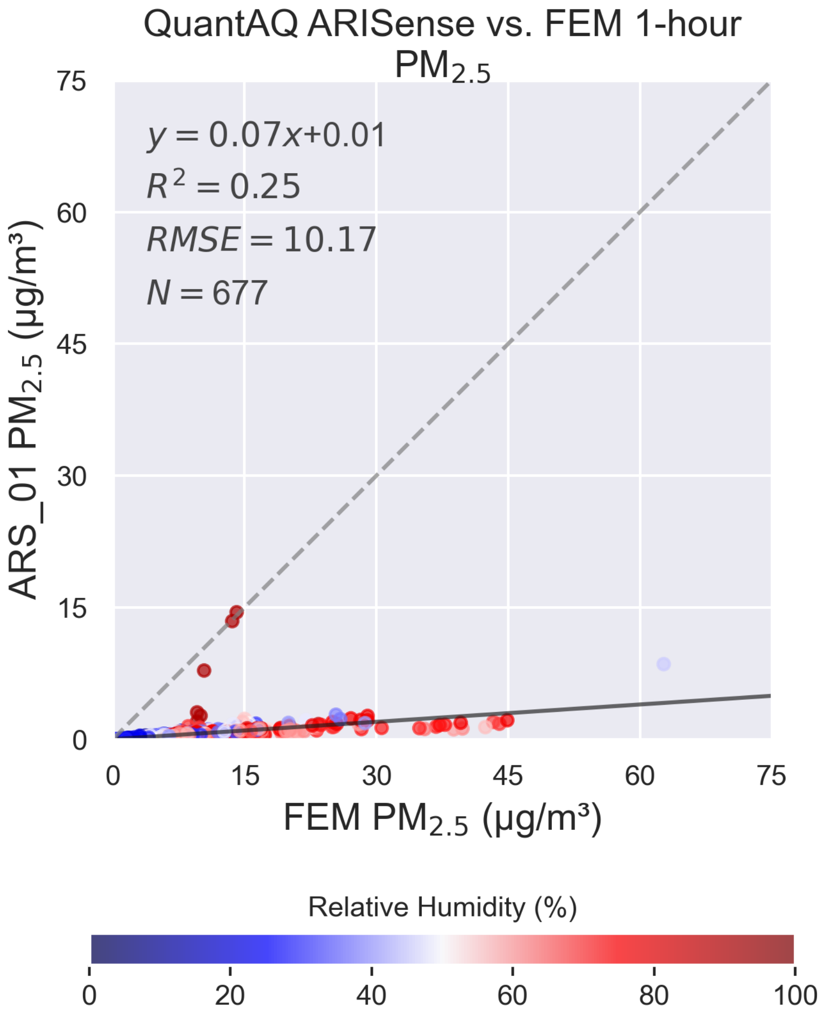

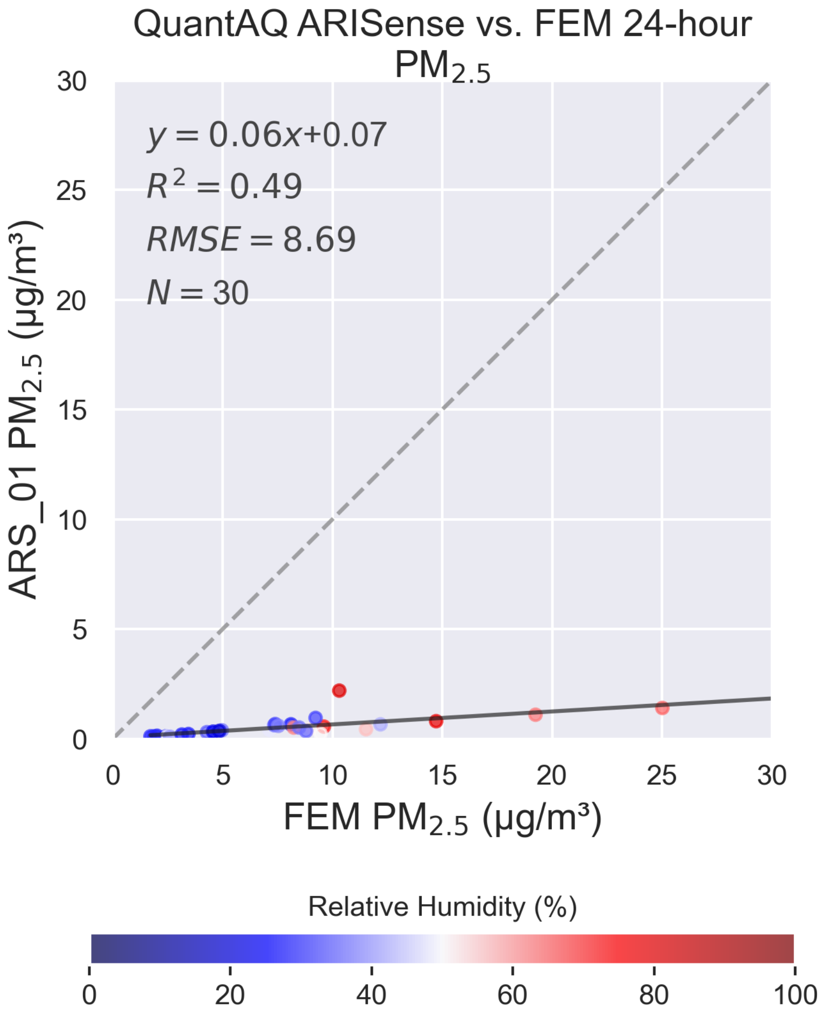

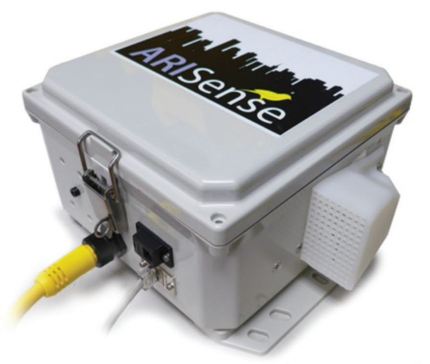

Image Attribution: QuantAQ, Inc (Somerville, MA)

Supplemental Information

Abbreviations used in Supplemental Information

FRM	Federal Reference Method
FEM	Federal Equivalent Method
SOP	Standard Operating Procedure
QAPP	Quality Assurance Project Plan
QC	Quality Control

Supplemental Documentation	Attached	Description & URL or file path to documentation
Field observations and sensor data flags	☑	See CO-ARS-Page 7 of this testing report
Maintenance logs	☐	No logs recorded during testing
Standard operating procedure(s)	☐	U.S. EPA Office Of Research and Development SOP available upon request
Photos of equipment setup and testing	☑	See CO-ARS-Page 5 of this testing report
Product specifications sheet(s)	☑	QuantAQ and Particles Plus: ARISense Specification Sheet
Product manual(s)	☐	N/A
Data storage and transmission method	☑	See CO-ARS-Page 6 of this testing report
Data correction approach	☑	See CO-ARS-Page 6 of this testing report
Issues encountered	☑	See CO-ARS-Page 6 of this testing report
Data analysis/correction scripts and version	☑	Averaging and processing of data, calculation of performance metrics, and generation of figures and other supplementary material for analysis were obtained using Python 3.9.7 with the packages sensortoolkit v0.8.3b2, pandas 1.3.5, NumPy 1.21.2, Matplotlib 3.5.0, statsmodels 0.13.0, and seaborn 0.11.2. All packages are available from the Python Package Index (PyPI) at https://pypi.org. The integrated development environment (IDE) Spyder 5.1.5 was used for scripting and data visualization. Version control for the Python base, packages, and IDE were all managed by conda 4.11.0.
Air Monitoring Station QAPP	☐	U.S. EPA Office Of Research and Development QAPP available upon request
Summary of FRM/FEM monitor QC checks	☑	See CO-ARS-Page 8 of this testing report
Manufacturer website for FRM/FEM monitor	☑	Teledyne API: T640 Product website
FRM/FEM monitor manual	☑	Teledyne API: T640 Product Manual
FRM/FEM monitor specifications sheet(s)	☑	Teledyne API: T640 Specification Sheet
Other documents	☐	

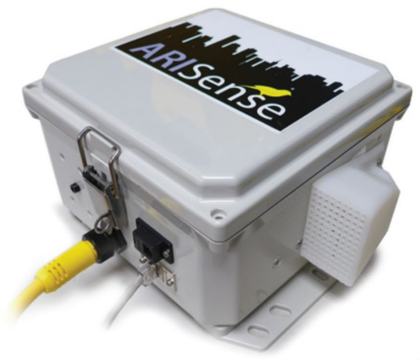

Image Attribution: QuantAQ, Inc (Somerville, MA)

Supplemental Information: Photos of Testing Site and Equipment Setup

Site Description:
The La Casa site was established in January of 2013 as a replacement for the Denver Municipal Animal Shelter (DMAS) site when a land use change forced the relocation of the site. The La Casa location has been established as the NCore site for the Denver Metropolitan area. Measurements include trace gas/precursor-level CO analyzer, and a NO_y analyzer, in addition to the trace level SO_2, O_3, meteorology, and particulate monitors. The site represents a population-oriented neighborhood scale monitoring area.

Figure 1: La Casa Monitoring Station sampling shelter, side view

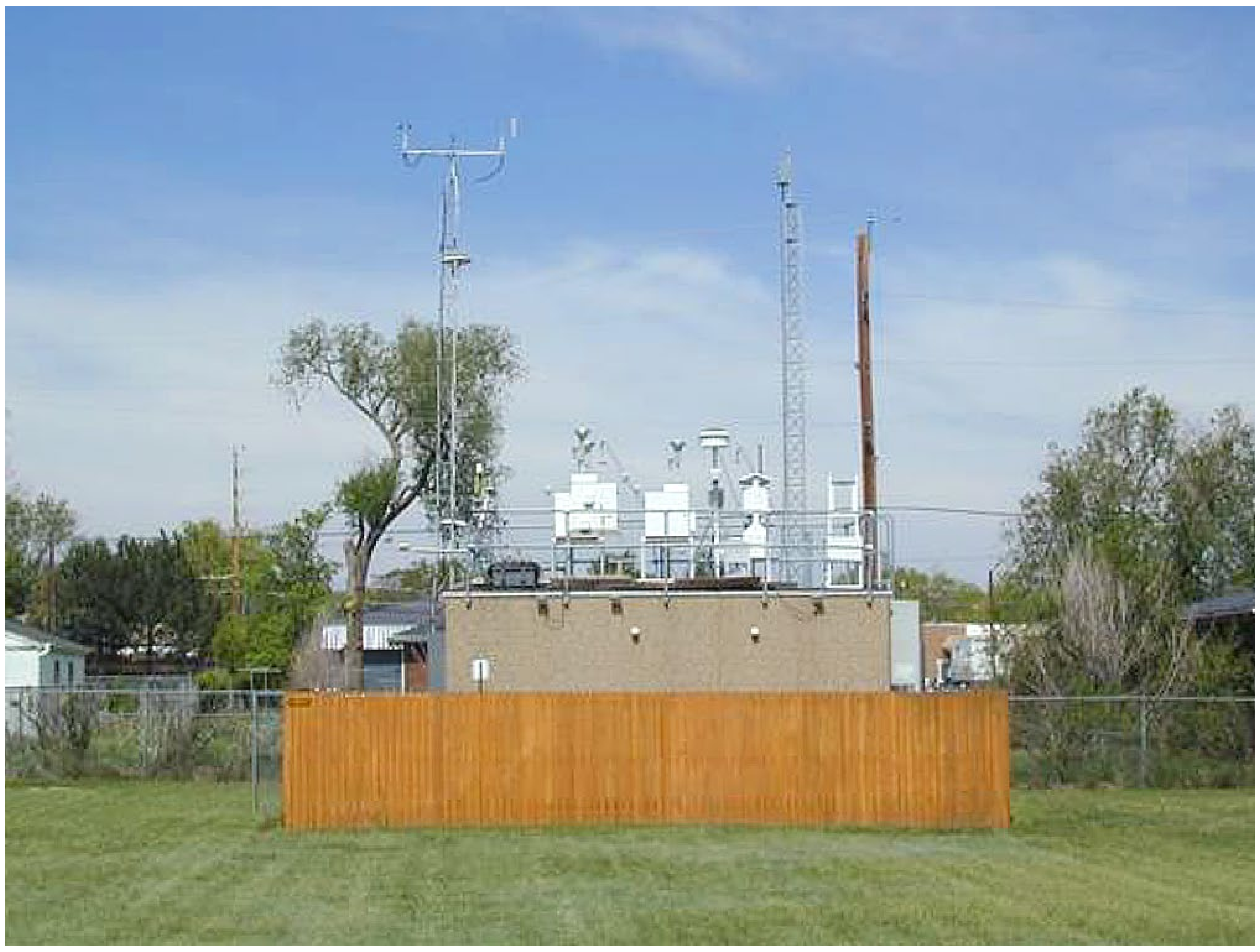

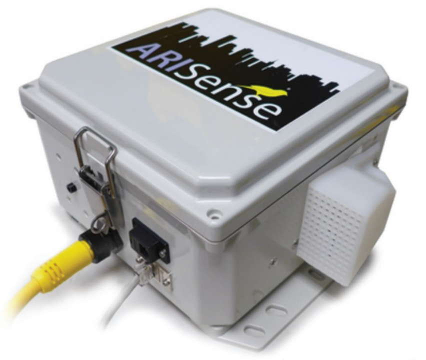

Image Attribution: QuantAQ, Inc (Somerville, MA)

Data Storage and Transmission Method

ARISense units upload data to an external cloud server at 2-minute averages via cellular signal. ARISense weekly data collections were performed by downloading data from the QuantAQ cloud server (last accessed 5/11/22).

Data Correction Approach

QuantAQ ARISense units measure PM2.5 concentrations via an optical particle counter (OPC). The OPC measures particle counts within particle size bins ranging from 0.3 microns to 10 microns, and mass concentrations for each bins are estimated from particle counts assuming constant values for density, spherical morphology, and average particle diameter. The manufacturer applies a general correction to account for the detection limitations of the OPC leading to concentrations that are closer to reality. These presets were configured by the sensor manufacturer and were not modified by EPA prior or during testing.

After acquisition, the raw data was processed using the *sensortoolkit* python code library (v0.8.3b2). A continuous data set at the recorded sampling frequency was written to a .csv file. 1-hour and 24-hour averaged data sets were generated using a 75% completeness threshold and saved as separate .csv files. Outliers were NOT removed from data sets in order to assess “out-of-the-box” sensor performance.

Issues Encountered

Pre-deployment observations

Numerous issues were encountered with the ARISense devices. Although previous models of the ARISense had used AlphaSense OPC-N2 these OPCs were unavailable at the time of manufacture. So instead, components from a particle plus OPC handheld were used.

- *Shipping damage:* On arrival to EPA many of the units were damaged due to the heavy pump breaking free during shipping and shaking around inside of the box breaking much of the plastic scaffolding and, in some cases, components. The manufacturer worked with us to fix these units.
- *Hardware and Firmware issues:* Multiple hardware and firmware issues were experienced that had to be fixed before starting the deployment and multiple manual firmware updates on both the gas sensor microcontrollers and the OPC microcontrollers were completed by researchers. The USB drives were frequently corrupted which required replacement and eventually a hardware update. In addition, the OPC microcontroller and the gas sensor microcontroller often miscommunicated. One firmware version reduced the number of bins reported by the OPC since the manufacturer determined the additional bins were not providing additional accuracy. New PCB boards were sent and installed before the tests could be run as well.
- *Initiation:* There was a high power draw when the cellular connectivity initiated and the pump was turned on. This meant that units occasionally failed to turn on at the same time and it meant these could not be powered by solar.
- *Water intake:* In addition, the OPC pump often drew in water during rainstorms leading the inline filter to saturate and the pump to fail due to overheating. Many pumps had to be replaced throughout the testing.
- *Cellular connectivity:* Some units could not consistently stay connected to the cellular, though other sensors at the same site did not experience issues. The manufacturer could not recreate this issue when they were sent back for service and the problems persisted.
- *Reset:* These units ended up being operated on timers so that they were power cycled every night which would typically get them back online if a software error had been encountered, leading to lower data loss than would have occurred with only weekly checks.

Field observations and sensor data flags are included on the following page

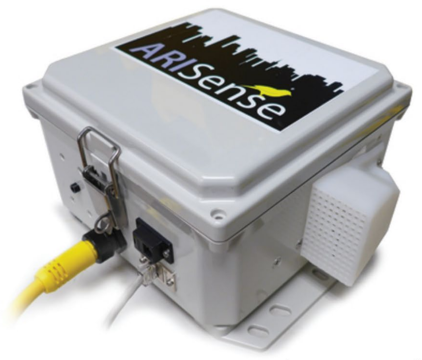

Image Attribution: QuantAQ, Inc (Somerville, MA)

Field observations and sensor data flags

The following table contains data flags describing events that were encountered during the testing period. The QuantAQ ARISense was configured to record data at regular 60-second sampling intervals, however, occasionally the unit recorded irregular sampling intervals ranging from 2-minute intervals to nearly 15 minutes at longest interval duration. The flags below note when these irregular sampling intervals occurred during the testing period. Sampling interval irregularities were not a result of modification to the device firmware configuration and are potentially associated power loading during periods where the ARISense unit initiated a cellular data upload.

Start Time (UTC)	End Time (UTC)	Sensor Serial ID	Parameters Impacted	Flag
2020-09-22 23:02:00+00:00	2021-01-31 23:59:00+00:00	ARS_01	ALL	6-Sampling interval abnormality

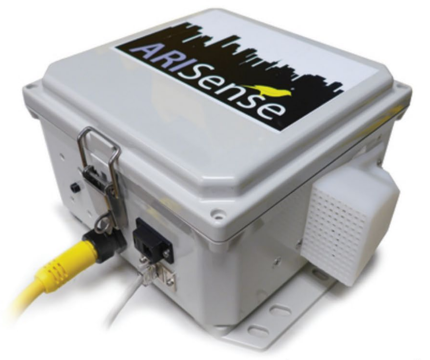

Image Attribution: QuantAQ, Inc (Somerville, MA)

Description of Data Flags

AQS

The U.S. EPA's Air Quality System (AQS) is the Agency's primary ambient air monitoring data archive. A comprehensive list of data flags that are recorded alongside AQS data sets, referred to by U.S. EPA as 'qualifiers', can be found at the following link: https://aqs.epa.gov/aqsweb/documents/codetables/qualifiers.html

Invalidation of reference data: AQS qualifiers are organized by qualifier type, which indicates whether data logged alongside qualifier flags should be invalidated (set null). Qualifiers with type "Null Data Qualifier" are invalidated, and includes data logged during periods that coincide with QC checks (e.g., "BF-Precision/Zero/Span", "BJ- Operator Error", "BL - QA Audit", "AZ - QC Audit") among other events such as power outages. Data logged alongside qualifiers with type "Quality Assurance Qualifiers" are not invalidated and are included in this analysis (e.g., concentrations less than the federal MDL for the reference monitor "MD – Value less than MDL", QA reviewed values "Validated Value").

Data Flags Recorded During Testing

FRM/FEM Monitor	Timestamp (UTC)	Flag
Teledyne API T640 (Acquired via AQS)	2020-09-17 15:00:00+0000 to 2020-09-17 16:00:00+0000	BA - Maintenance/Routine Repairs
	2020-10-09 17:00:00+0000 to 2020-10-09 18:00:00+0000	BA - Maintenance/Routine Repairs

Testing Report - $PM_{2.5}$ Base Testing

Aeroqual AQY

This report reflects out-of-the-box performance

Initial Base Testing - Wilmington, DE
U.S. Environmental Protection Agency
Office of Research and Development
PI: Clements.Andrea@epa.gov
919-541-1363
July 2019—August 2019

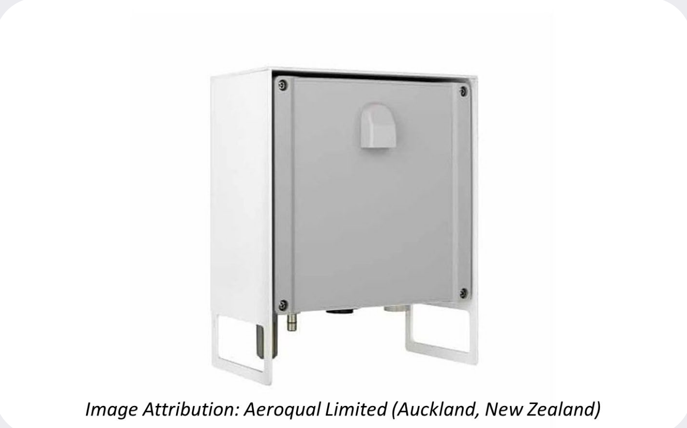

Image Attribution: Aeroqual Limited (Auckland, New Zealand)

Deployment Details

Testing Organization and Site Information	
Testing organization (Name, Organization type, Contact website)	U.S. Environmental Protection Agency - Office of Research and Development<br>Federal Government<br>Air Sensor Toolbox | U.S. EPA Website
Testing location (City, State, Latitude and Longitude)	MLK - Corner Of MLK Blvd And Justison St<br>Wilmington, DE<br>39.7395, -75.5575
AQS site ID	10 - 003 - 2004
Sampling timeframe (MM-DD-YY)	07-26-19 to 08-25-19
Sensor data source	Aeroqual Cloud download
Reference data source	AQS API download

Sensor Information			
Manufacturer, model	Aeroqual AQY		
Device firmware version	1.14.2		
Sampling time interval	1-minute		
Sensor serial numbers	AQY_01		
Issues encountered during deployment?	☑	See DE-AQY-Page 6 of this testing report	

FRM/FEM Information	
Manufacturer, model, designation	Teledyne Advanced Pollution Instrumentation T640 FEM
Sampling time interval	1-hour averaging
Date of calibration	As required by 40 CFR Part 58 and the DE Ambient Air Quality Monitoring Program QAPP maintained by DNREC
Date of flowrate verification check	Monthly as required by 40 CFR Part 58 Appendix A
Description, date(s) of maintenance activities	N/A

Time Series Plots

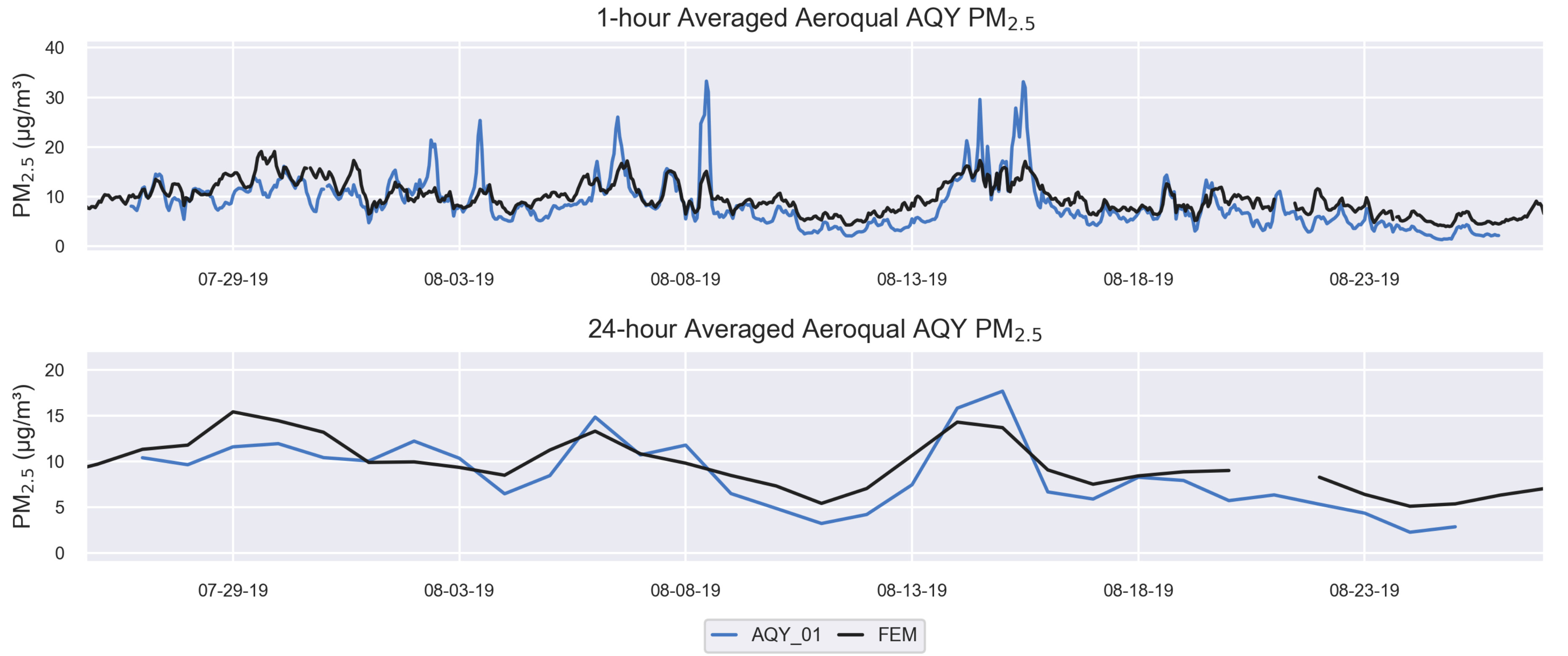

Scatter Plots: Comparison to FRM/FEM

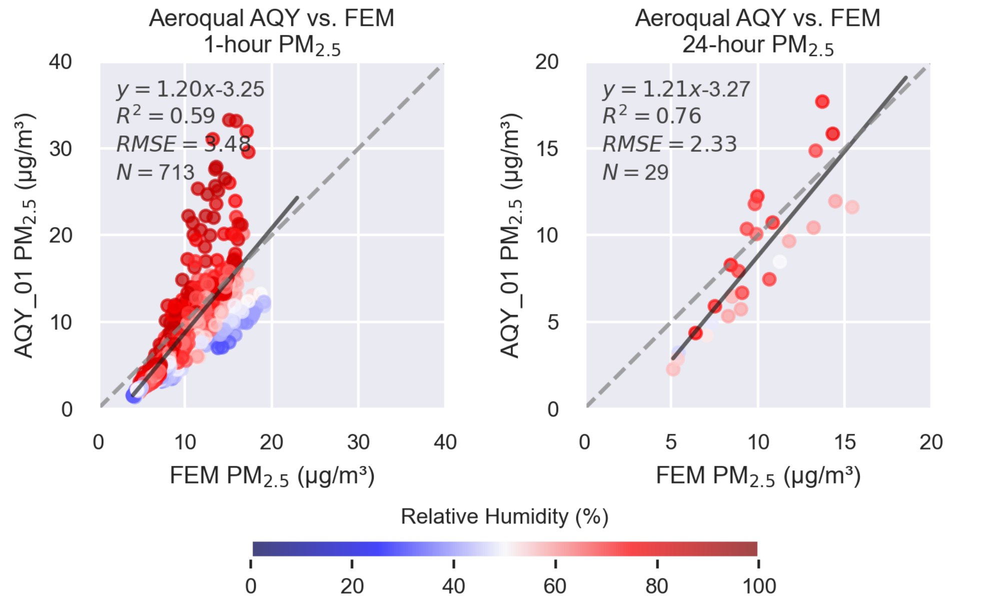

Range of FRM/FEM concentrations over duration of base test (µg/m³)	4.0-19.1 (1-hr), 5.1-15.4 (24-hr)
Number of 24-hr periods in FRM/FEM monitor measurements with a goal concentration ≥ 25 µg/m³	0

Performance Metrics

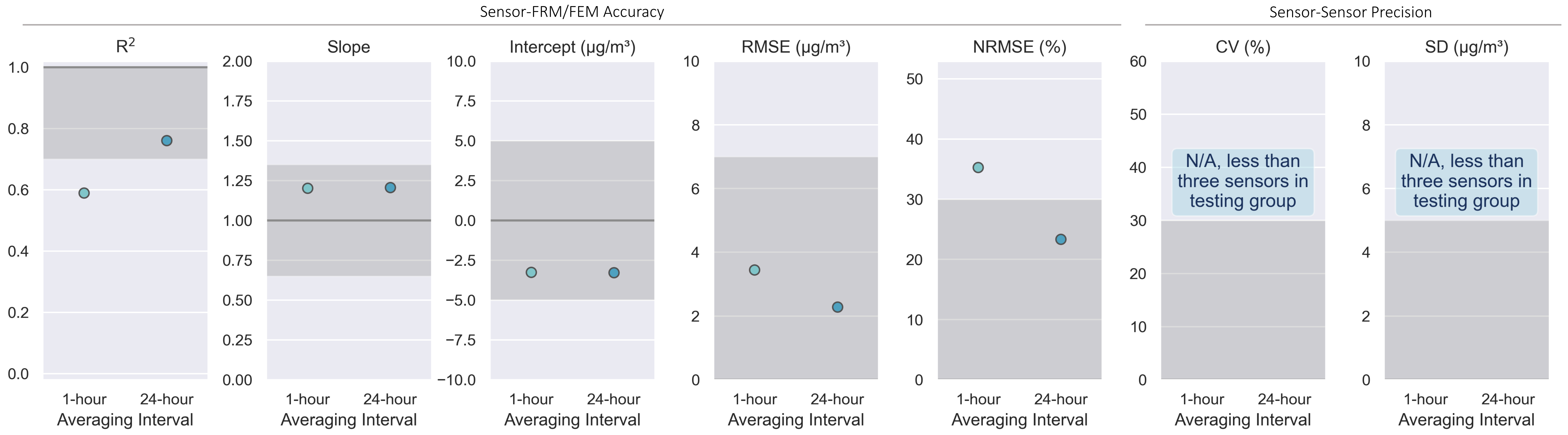

Meteorological Conditions During Deployment

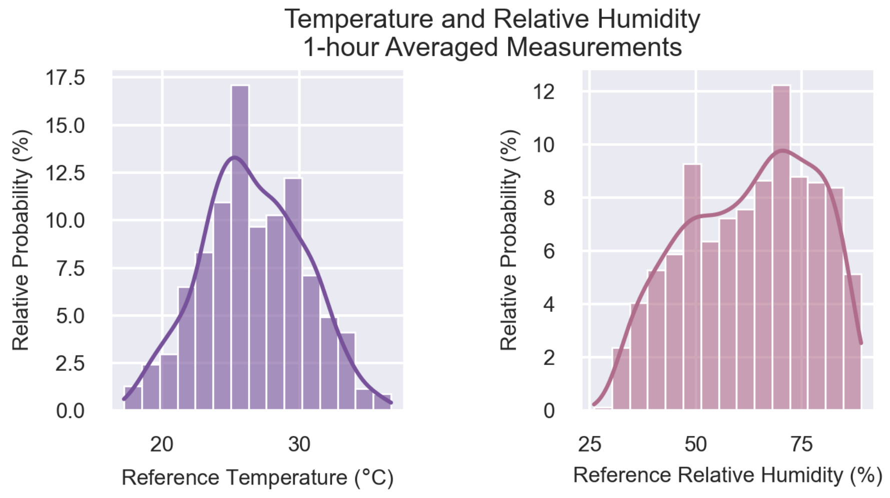

Number of 24-hr periods outside sensor manufacturer-listed temperature operational range (-10 to 40 °C)	0
Number of 24-hr periods outside sensor manufacturer-listed relative humidity operational range (no operational range specified)	-

Meteorological Influence

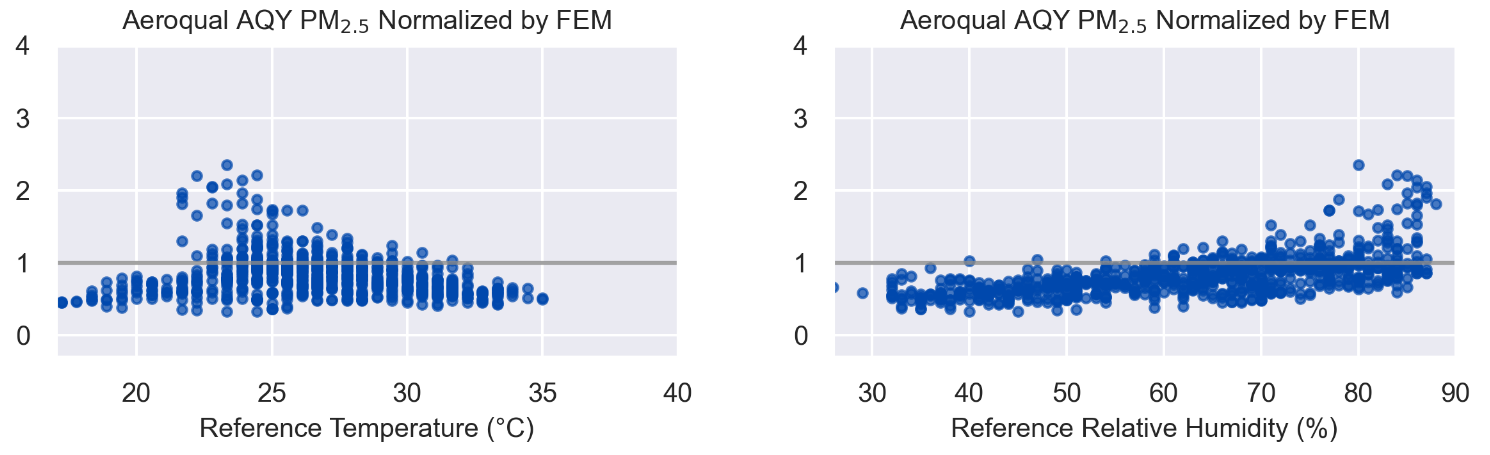

Mean number of paired, normalized concentration and temperature values (1-hr averages)	717
Mean number of paired, normalized concentration and relative humidity values (1-hr averages)	717

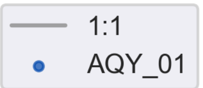

Testing Report - $PM_{2.5}$ Base Testing

Aeroqual AQY

This report reflects out-of-the-box performance

Initial Base Testing - Wilmington, DE
U.S. Environmental Protection Agency
Office of Research and Development
PI: Clements.Andrea@epa.gov
919-541-1363
July 2019—August 2019

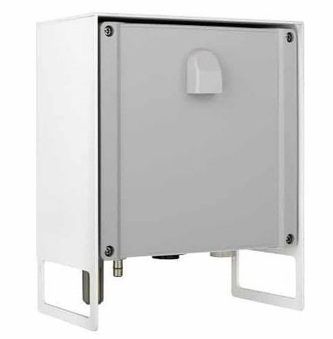

Image Attribution: Aeroqual Limited (Auckland, New Zealand)

Tabular Statistics

Sensor-FRM/FEM Correlation

	Bias and Linearity						Data Quality			
	R^2		Slope		Intercept (µg/m³)		Uptime (%)		Number of paired sensor and FRM/FEM concentration values	
	1-Hour ○	24-Hour ●	1-Hour ●	24-Hour ●	1-Hour ●	24-Hour ●	1-Hour ●	24-Hour ●	1-Hour	24-Hour
Metric Target Range	≥ 0.70	≥ 0.70	1.0 ± 0.35	1.0 ± 0.35	-5 ≤ b ≤ 5	-5 ≤ b ≤ 5	75%*	75%*	-	-
Sensor AQY_01	0.59	0.76	1.20	1.21	-3.25	-3.27	100	100	713	29

	Error			
	RMSE (µg/m³)		NRMSE (%)	
	1-Hour ★	24-Hour ★	1-Hour ☆	24-Hour ★
Metric Target Range	≤ 7.0	≤ 7.0	≤ 30.0	≤ 30.0
Deployment Value	3.4	2.3	35.3	23.4

Device-specific metrics (computed for each sensor in evaluation)
○○○ Metric value for none of devices tested falls within the target range
●○○ Metric value for one of devices tested falls within the target range
●●○ Metric value for two of devices tested falls within the target range
●●● Metric value for three of devices tested falls within the target range

Single-valued metrics (computed via entire evaluation dataset)
☆ Indicates that the metric value is not within the target range
★ Indicates that the metric value is within the target range

Sensor-Sensor Precision[1]

	Precision (between collocated sensors)				Data Quality	
	CV (%)		SD (µg/m³)		Number of concurrent sensor concentration pairs	
	1-Hour ☆	24-Hour ☆	1-Hour ☆	24-Hour ☆	1-Hour	24-Hour
Metric Target Range	≤ 30.0	≤ 30.0	≤ 5.0	≤ 5.0	-	-
Deployment Value	-	-	-	-	-	-

[1]Precision statistics are computed for evaluations with at least three collocated sensor units. Metric values are left blank for evaluations with two or fewer sensor units.

*This value is only a recommendation for ensuring data quality and is not included in the list of target values discussed in Section 4 of the Performance Testing Protocols, Metrics, and Target Values for Fine Particulate Matter Air Sensors document.

Sensor-FRM/FEM Scatter Plots

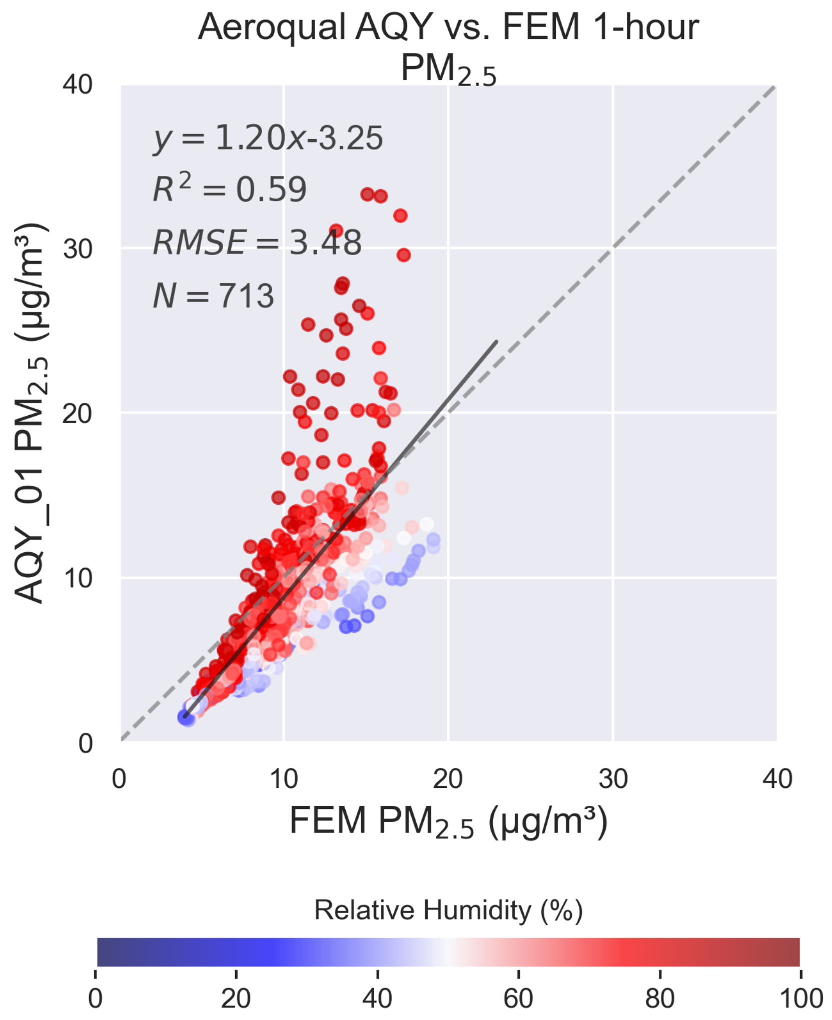

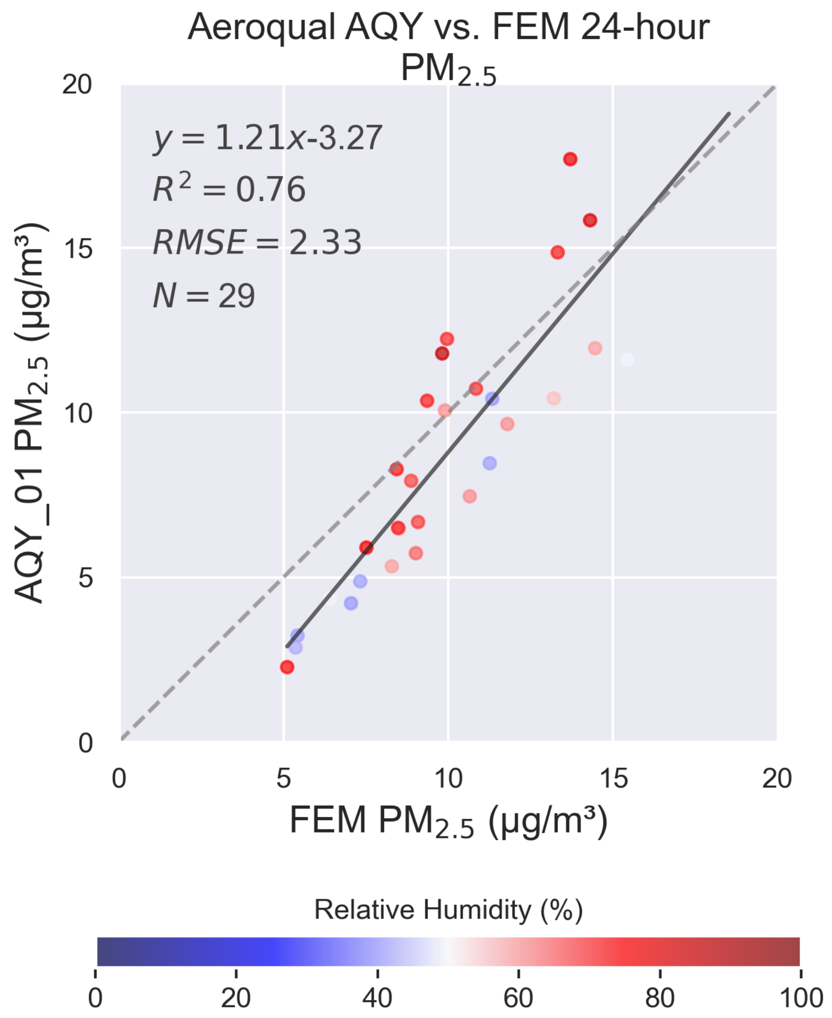

Testing Report - $PM_{2.5}$ Base Testing

Aeroqual AQY

This report reflects out-of-the-box performance

Initial Base Testing - Wilmington, DE

U.S. Environmental Protection Agency
Office of Research and Development
PI: Clements.Andrea@epa.gov
919-541-1363

July 2019—August 2019

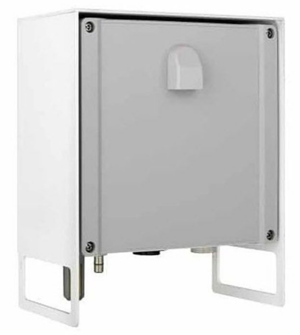

Image Attribution: Aeroqual Limited (Auckland, New Zealand)

Supplemental Information

Abbreviations used in Supplemental Information

FRM	Federal Reference Method
FEM	Federal Equivalent Method
SOP	Standard Operating Procedure
QAPP	Quality Assurance Project Plan
QC	Quality Control

Supplemental Documentation	Attached	Description & URL or file path to documentation
Field observations and sensor data flags	☑	See DE-AQY-Page 6 of this testing report
Maintenance logs	☐	No logs recorded during testing
Standard operating procedure(s)	☐	U.S. EPA Office Of Research and Development SOP available upon request
Photos of equipment setup and testing	☑	See DE-AQY-Page 5 of this testing report
Product specifications sheet(s)	☐	N/A*
Product manual(s)	☐	N/A*
Data storage and transmission method	☑	See DE-AQY-Page 6 of this testing report
Data correction approach	☑	See DE-AQY-Page 6 of this testing report
Issues encountered	☑	See DE-AQY-Page 6 of this testing report
Data analysis/correction scripts and version	☑	Averaging and processing of data, calculation of performance metrics, and generation of figures and other supplementary material for analysis were obtained using Python 3.9.7 with the packages sensortoolkit v0.8.3b2, pandas 1.3.5, NumPy 1.21.2, Matplotlib 3.5.0, statsmodels 0.13.0, and seaborn 0.11.2. All packages are available from the Python Package Index (PyPI) at https://pypi.org. The integrated development environment (IDE) Spyder 5.1.5 was used for scripting and data visualization. Version control for the Python base, packages, and IDE were all managed by conda 4.11.0.
Air Monitoring Station QAPP	☐	U.S. EPA Office Of Research and Development QAPP available upon request
Summary of FRM/FEM monitor QC checks	☑	See DE-AQY-Page 7 of this testing report
Manufacturer website for FRM/FEM monitor	☑	Teledyne API: T640/T640X Product website
FRM/FEM monitor manual	☑	Teledyne API: T640/T640X Product Manual
FRM/FEM monitor specifications sheet(s)	☑	Teledyne API: T640/T640X Specification Sheet
Other documents	☑	Manufacturer notice of AQY sales on hold

*As of 3/18/2021, the manufacturer of the AQY has placed sales of a similar unit on hold. Documentation for the AQY is currently unavailable from the manufacturer's website.

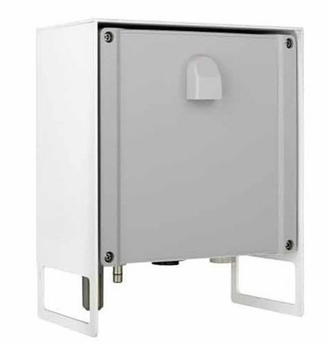

Site Description:

The DE site is located in Wilmington, Delaware at the intersection of Justison St. and MLK Blvd and has been operated at this site since 1999. This site is the state NCore site and represents urban population exposure to multiple pollution sources. This site meets all EPA *40 CFR Part 58 App D and E* siting criteria.

Figure 1: Aeroqual AQY sensor (indicated by red arrow) attached to metal railing atop the sampling shelter at the monitoring site.

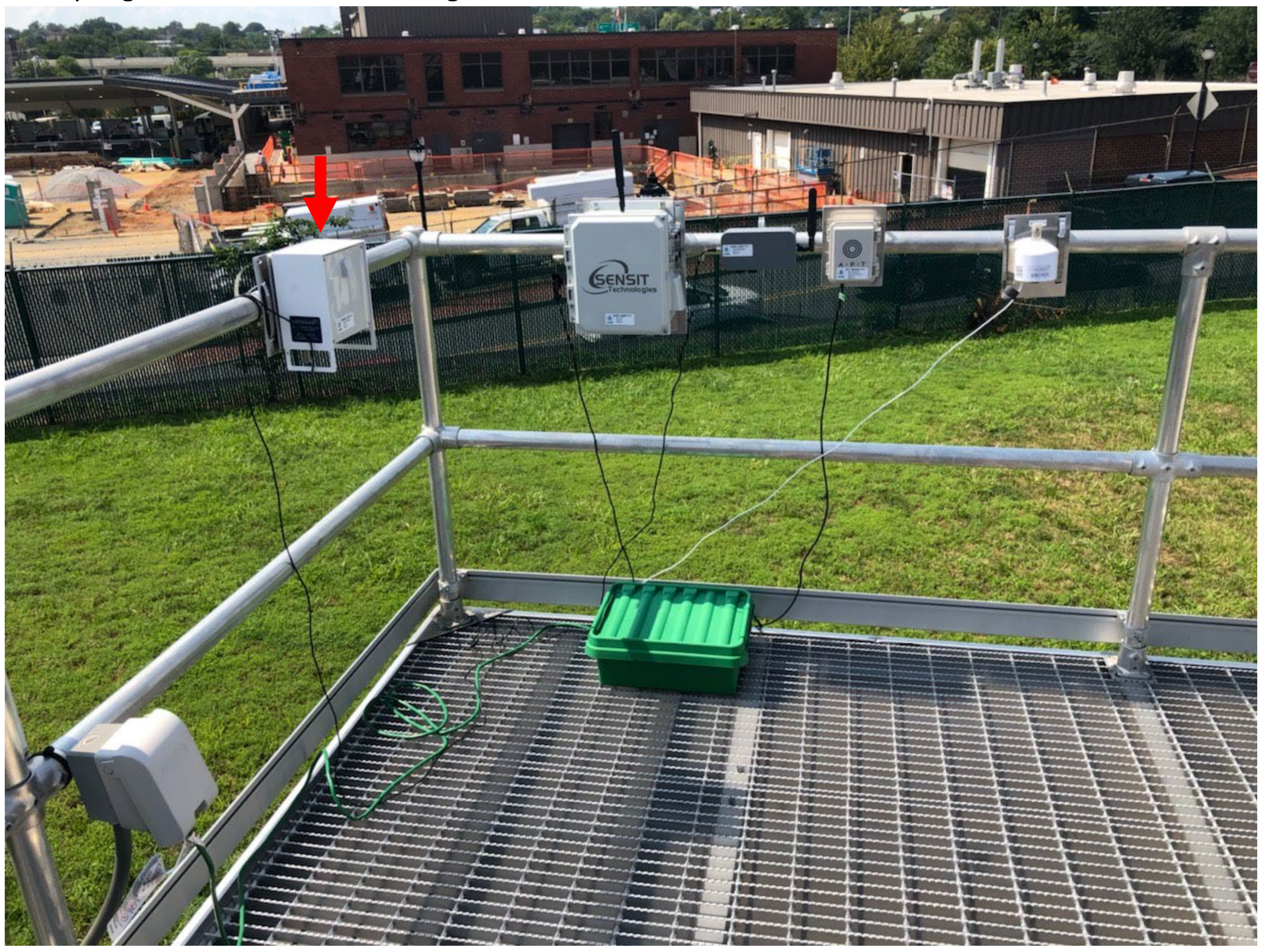

Figure 2: MLK Monitoring Station

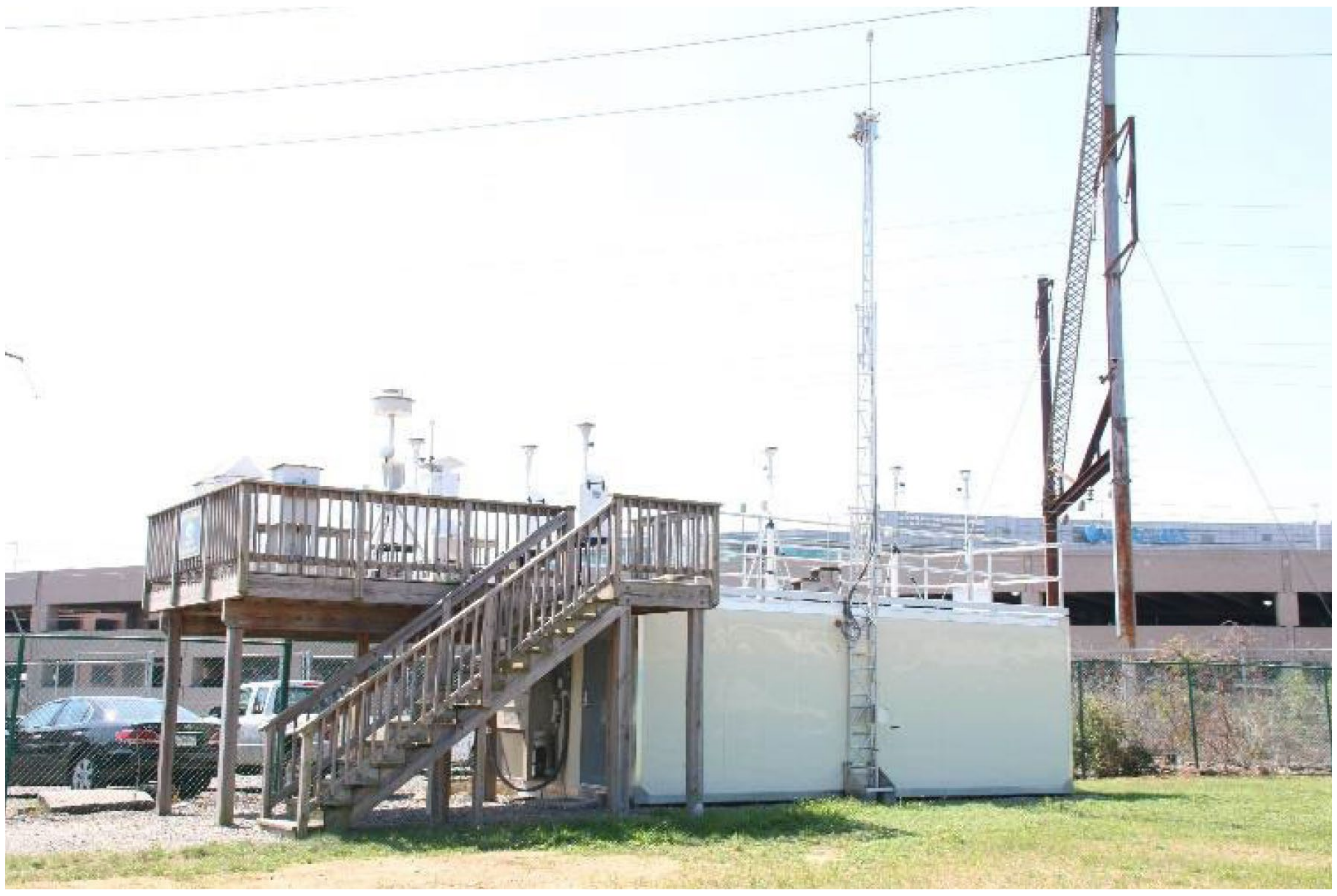

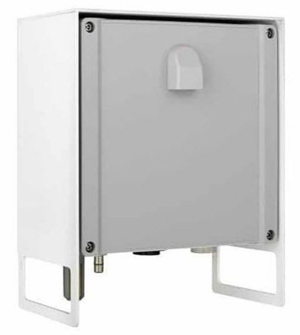

Image Attribution: Aeroqual Limited (Auckland, New Zealand)

Supplemental Information: Data Storage, Correction Approach, and Issues Encountered

Data Storage and Transmission Method

As part of CRADA #934-16 between Aeroqual and US EPA, Aeroqual supported data streaming. SIM cards were installed and data flowed to the Aeroqual Cloud. The 1-minute raw data was acquired weekly using the Aeroqual Cloud (*last accessed 5/11/22*) user interface (UI). The AQY has an internal data storage USB flash drive as a data backup, however the software on the drive is proprietary to Aeroqual and unreadable by outside systems.

Data Correction Approach

This evaluation report reflects "out-of-the-box" performance of the AQY. The manufacturer provides a procedure by which local collocation (sensor operated along side an FRM/FEM) data can be collected, a gain (slope) and offset (intercept) determined, and parameters entered into the Aeroqual Cloud user interface to be applied to all subsequently collected data. This procedure and feature was NOT used prior to this evaluation. Prospective consumers may get different performance from this device if they utilize this feature.

After acquisition, the raw data was processed using the *sensortoolkit* python code library (v0.8.3b2). A continuous data set at the recorded sampling frequency was written to a .csv file. 1-hour and 24-hour averaged data sets were generated using a 75% completeness threshold and saved as separate .csv files. Outliers were NOT removed from data sets in order to assess "out-of-the-box" sensor performance.

Issues Encountered

Pre-deployment observations

- *Timestamp inaccuracies*: During pre-deployment, the AQY devices did not properly sync timestamps with the onboard Real-Time Clock. Connecting the units to the internet by cellular or Wi-Fi allowed the unit to sync with internet time and resulted in proper timestamps.

Field observations and sensor data flags

The following table contains data flags describing events that were encountered during the testing period. During the testing period, two power outages were reported on 7/29/2019 and 7/31/2019. Both outages were relatively brief, with each lasting ~15 minutes and 3 minutes for the 7/29/2019 and 7/31/2019 events, respectively. The field technician was not reported at the site during either outage, indicating that each outage was likely not attributed to operator error. A sampling interval abnormality was reported on 7/31/2019, during which the unit deviated from its configured 1-minute sampling interval, and instead logged an interval lasting 53 minutes. Following this event, the sensor logged samples at regular 1-minute intervals.

Start Time (UTC)	End Time (UTC)	Sensor Serial ID	Parameters Impacted	Flag
2019-07-29 15:41:00+00:00	2019-07-29 15:55:00+00:00	AQY_01	ALL	9.0-Data Loss - Power Connection Error
2019-07-31 00:08:00+00:00	2019-07-31 01:01:00+00:00	AQY_01	ALL	6.0-Sampling interval abnormality
2019-07-31 17:36:00+00:00	2019-07-31 17:39:00+00:00	AQY_01	ALL	9.0-Data Loss - Power Connection Error

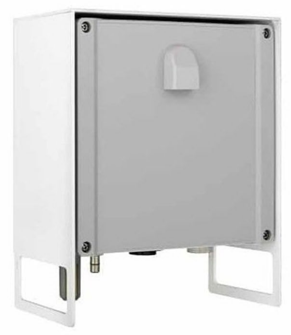

Image Attribution: Aeroqual Limited (Auckland, New Zealand)

Description of Data Flags

AQS

The U.S. EPA's Air Quality System (AQS) is the Agency's primary ambient air monitoring data archive. A comprehensive list of data flags that are recorded alongside AQS data sets, referred to by U.S. EPA as 'qualifiers', can be found at the following link: https://aqs.epa.gov/aqsweb/documents/codetables/qualifiers.html

Invalidation of reference data: AQS qualifiers are organized by qualifier type, which indicates whether data logged alongside qualifier flags should be invalidated (set null). Qualifiers with type "Null Data Qualifier" are invalidated, and includes data logged during periods that coincide with QC checks (e.g., "BF-Precision/Zero/Span", "BJ- Operator Error", "BL - QA Audit", "AZ - QC Audit") among other events such as power outages. Data logged alongside qualifiers with type "Quality Assurance Qualifiers" are not invalidated and are included in this analysis (e.g., concentrations less than the federal MDL for the reference monitor "MD – Value less than MDL", QA reviewed values "Validated Value").

Data Flags Recorded During Testing

FRM/FEM Monitor	Timestamp (UTC)	Flag
Teledyne API T640 (Acquired via AQS)	2019-07-30 16:00:00+0000	AI - Insufficient Data (cannot calculate)
	2019-08-21 01:00:00+0000 to 2019-08-21 09:00:00+0000	AM - Miscellaneous Void
	2019-08-21 10:00:00+0000	AI - Insufficient Data (cannot calculate)
	2019-08-23 16:00:00+0000	AI - Insufficient Data (cannot calculate)

Meteorological Instrument	Timestamp (UTC)	Flag
Vaisala WXT-520 Temperature Monitor (Acquired via AQS)	2019-08-21 01:00:00+0000 to 2019-08-21 09:00:00+0000	AM - Miscellaneous Void
	2019-08-21 10:00:00+0000	AI - Insufficient Data (cannot calculate)
Vaisala WXT-520 Relative Humidity Monitor (Acquired via AQS)	2019-08-21 01:00:00+0000 to 2019-08-21 09:00:00+0000	AM - Miscellaneous Void
	2019-08-21 10:00:00+0000	AI - Insufficient Data (cannot calculate)

Testing Report - $PM_{2.5}$ Base Testing

APT Maxima

This report reflects out-of-the-box performance

Initial Base Testing - Wilmington, DE

U.S. Environmental Protection Agency
Office of Research and Development
PI: Clements.Andrea@epa.gov
919-541-1363

July 2019—August 2019

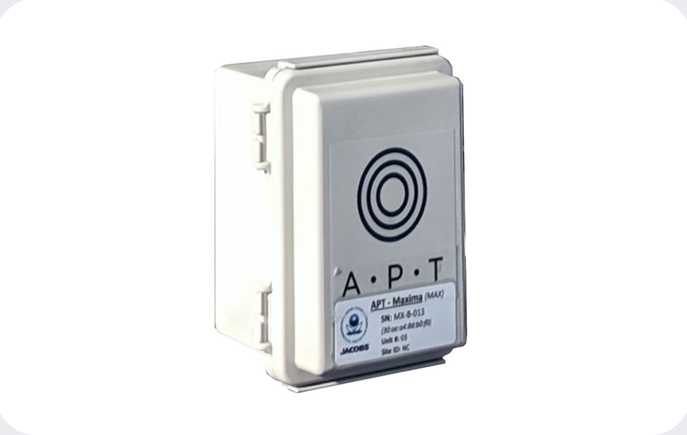

Deployment Details

Testing Organization and Site Information	
Testing organization (Name, Organization type, Contact website)	U.S. Environmental Protection Agency - Office of Research and Development<br>Federal Government<br>Air Sensor Toolbox | U.S. EPA Website
Testing location (City, State, Latitude and Longitude)	MLK - Corner Of MLK Blvd And Justison St<br>Wilmington, DE<br>39.7395, -75.5575
AQS site ID	10 - 003 - 2004
Sampling timeframe (MM-DD-YY)	07-26-19 to 08-25-19
Sensor data source	Onboard MicroSD card
Reference data source	AQS API download

Sensor Information			
Manufacturer, model	APT Maxima		
Device firmware version	v5.0 (installed 5/2/2019)		
Sampling time interval	30-seconds		
Sensor serial numbers	MAX_01		
Issues encountered during deployment?	☑	See DE-MAX-Page 6 of this testing report	

FRM/FEM Information	
Manufacturer, model, designation	Teledyne Advanced Pollution Instrumentation T640 FEM
Sampling time interval	1-hour averaging
Date of calibration	As required by 40 CFR Part 58 and the DE Ambient Air Quality Monitoring Program QAPP maintained by DNREC
Date of flowrate verification check	Monthly as required by 40 CFR Part 58 Appendix A
Description, date(s) of maintenance activities	N/A

Time Series Plots

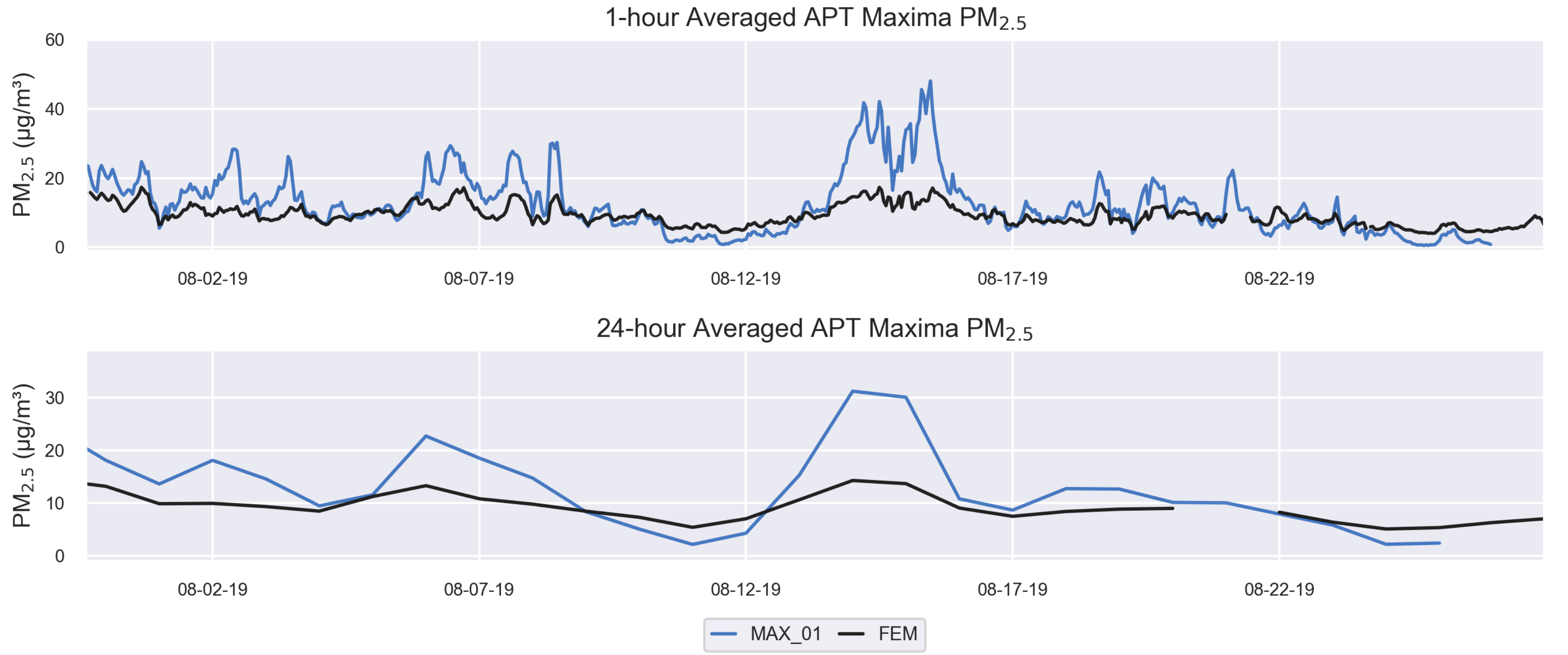

Scatter Plots: Comparison to FRM/FEM

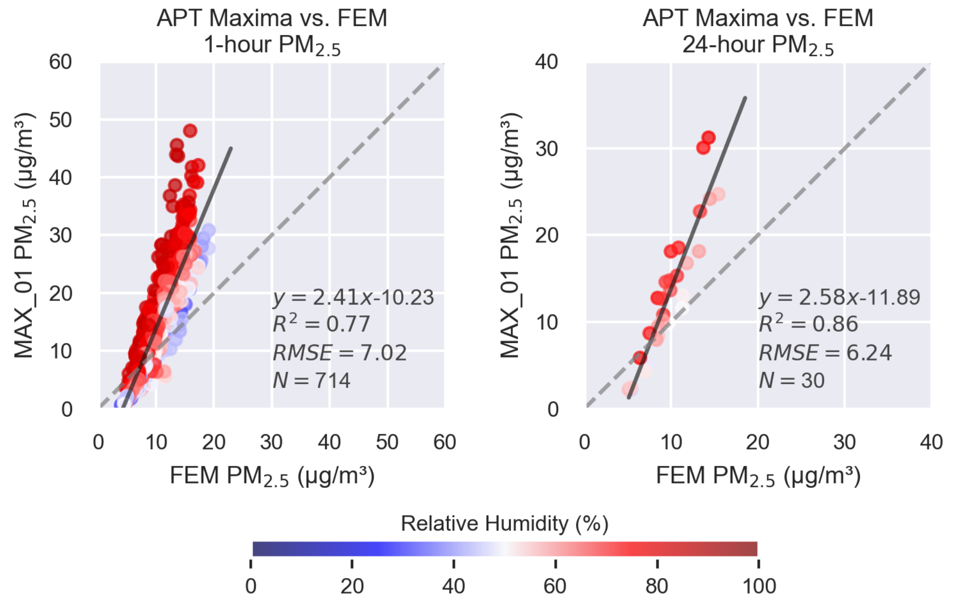

Range of FRM/FEM concentrations over duration of base test (µg/m³)	4.0-17.3 (1-hr), 5.1-14.3 (24-hr)
Number of 24-hr periods in FRM/FEM monitor measurements with a goal concentration ≥ 25 µg/m³	0

Performance Metrics

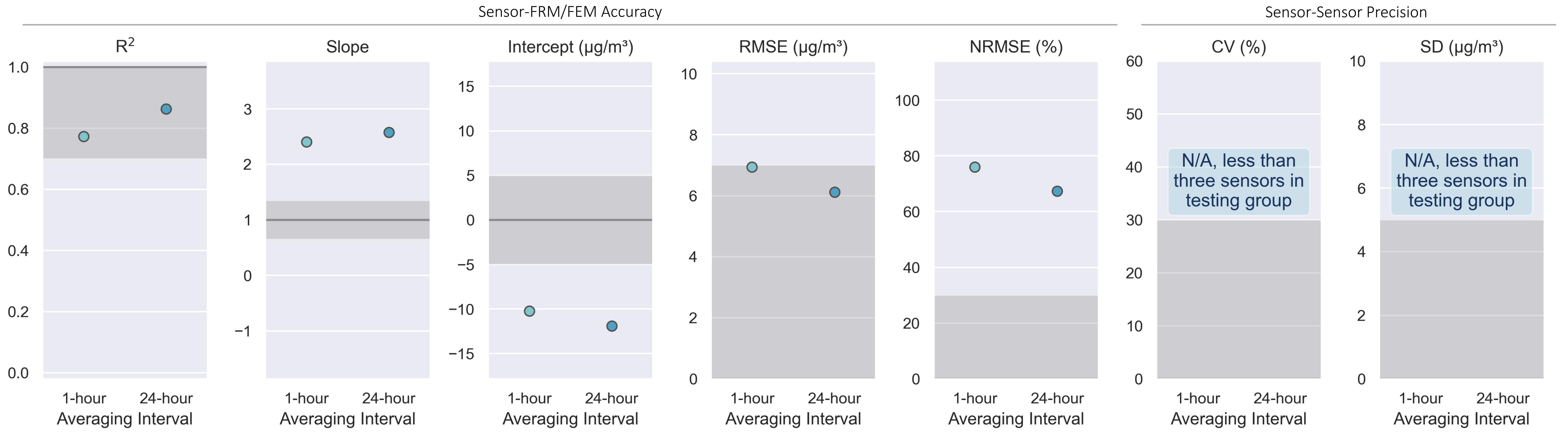

Meteorological Conditions During Deployment

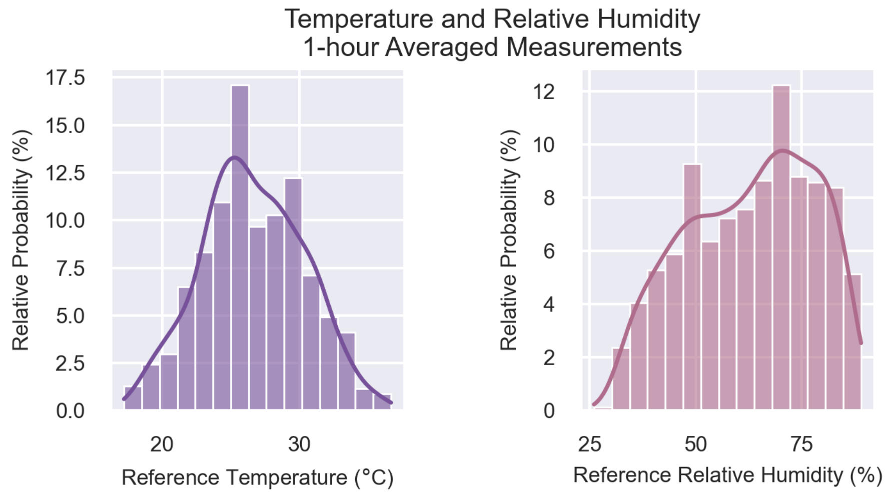

Number of 24-hr periods outside sensor manufacturer-listed temperature operational range (no operational range specified)	-
Number of 24-hr periods outside sensor manufacturer-listed relative humidity operational range (no operational range specified)	-

Meteorological Influence

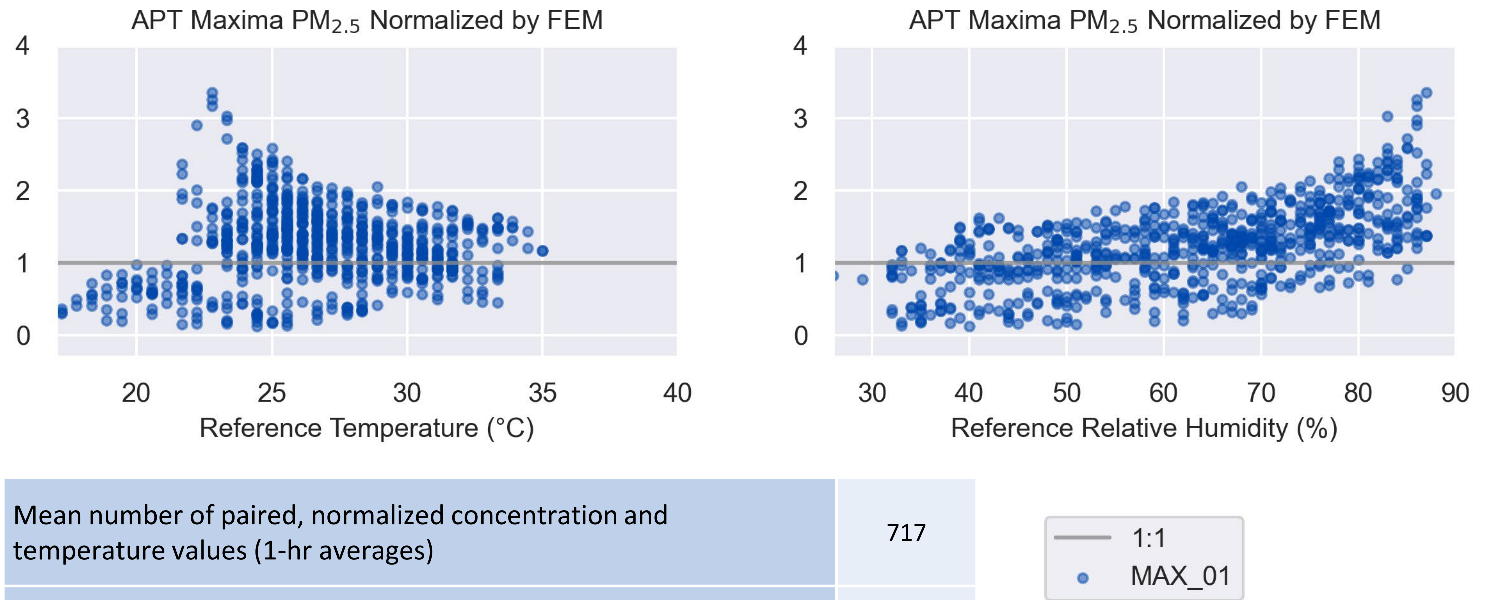

Mean number of paired, normalized concentration and temperature values (1-hr averages)	717
Mean number of paired, normalized concentration and relative humidity values (1-hr averages)	717

Testing Report - $PM_{2.5}$ Base Testing

APT Maxima

This report reflects out-of-the-box performance

Initial Base Testing - Wilmington, DE
U.S. Environmental Protection Agency
Office of Research and Development
PI: Clements.Andrea@epa.gov
919-541-1363
July 2019—August 2019

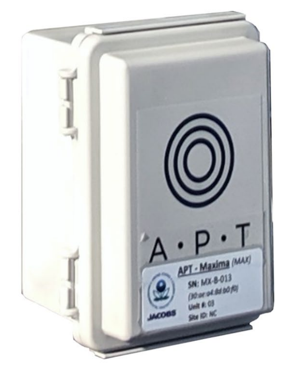

Tabular Statistics

Sensor-FRM/FEM Correlation

	Bias and Linearity						Data Quality			
	R^2		Slope		Intercept (μg/m³)		Uptime (%)		Number of paired sensor and FRM/FEM concentration values	
	1-Hour ●	24-Hour ●	1-Hour ○	24-Hour ○	1-Hour ○	24-Hour ○	1-Hour ●	24-Hour ●	1-Hour	24-Hour
Metric Target Range	≥ 0.70	≥ 0.70	1.0 ± 0.35	1.0 ± 0.35	-5 ≤ b ≤ 5	-5 ≤ b ≤ 5	75%*	75%*	-	-
Sensor MAX_01	0.77	0.86	2.41	2.58	-10.23	-11.89	100	100	714	30

	Error			
	RMSE (μg/m³)		NRMSE (%)	
	1-Hour ★	24-Hour ★	1-Hour ☆	24-Hour ☆
Metric Target Range	≤ 7.0	≤ 7.0	≤ 30.0	≤ 30.0
Deployment Value	6.9	6.1	76.0	67.4

Device-specific metrics (computed for each sensor in evaluation)
○○○ Metric value for none of devices tested falls within the target range
●○○ Metric value for one of devices tested falls within the target range
●●○ Metric value for two of devices tested falls within the target range
●●● Metric value for three of devices tested falls within the target range

Single-valued metrics (computed via entire evaluation dataset)
☆ Indicates that the metric value is not within the target range
★ Indicates that the metric value is within the target range

Sensor-Sensor Precision[1]

	Precision (between collocated sensors)				Data Quality	
	CV (%)		SD (μg/m³)		Number of concurrent sensor concentration pairs	
	1-Hour ☆	24-Hour ☆	1-Hour ☆	24-Hour ☆	1-Hour	24-Hour
Metric Target Range	≤ 30.0	≤ 30.0	≤ 5.0	≤ 5.0	-	-
Deployment Value	-	-	-	-	-	-

[1]Precision statistics are computed for evaluations with at least three collocated sensor units. Metric values are left blank for evaluations with two or fewer sensor units.

*This value is only a recommendation for ensuring data quality and is not included in the list of target values discussed in Section 4 of the Performance Testing Protocols, Metrics, and Target Values for Fine Particulate Matter Air Sensors document.

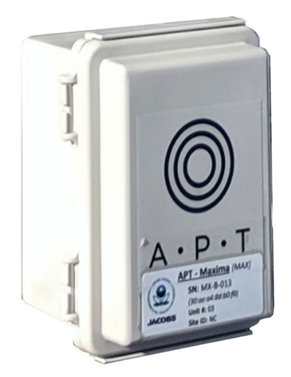

Sensor-FRM/FEM Scatter Plots

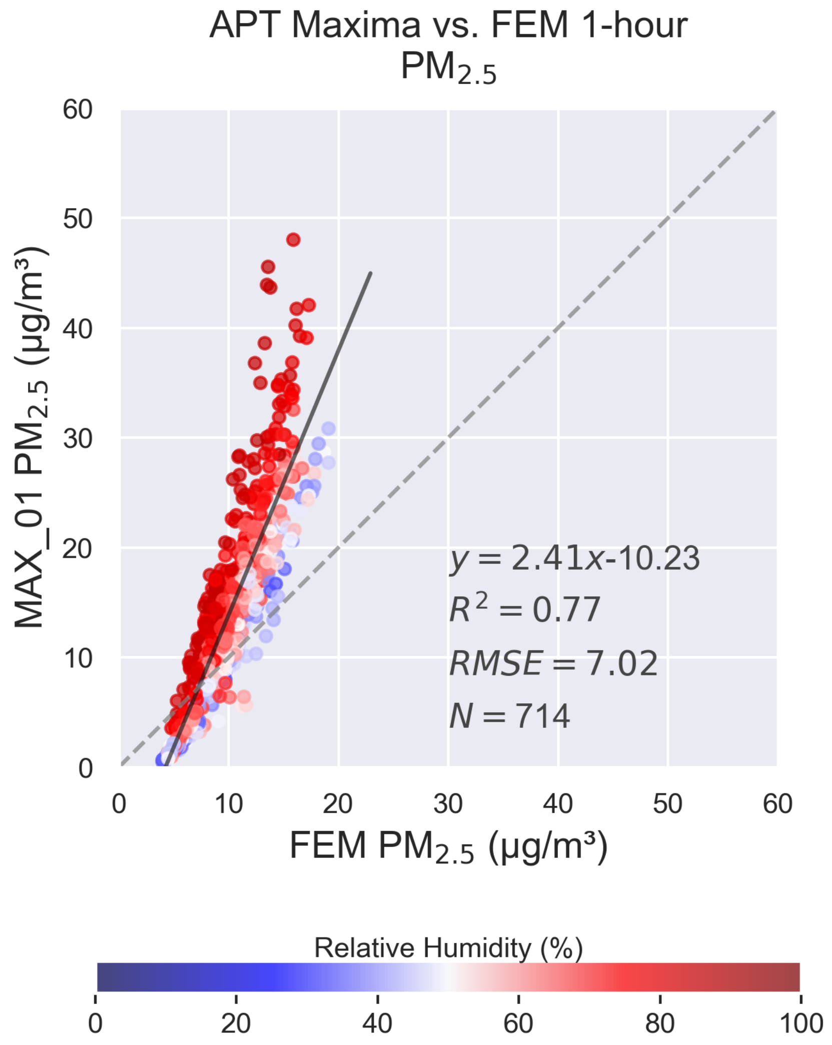

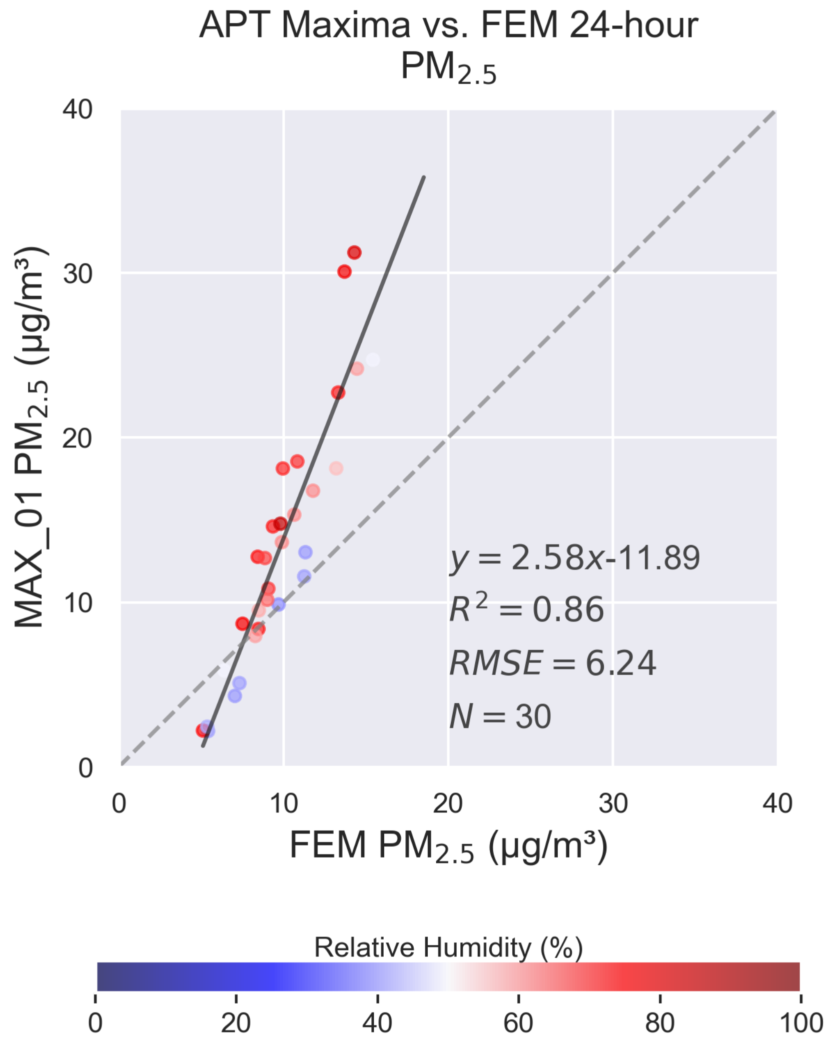

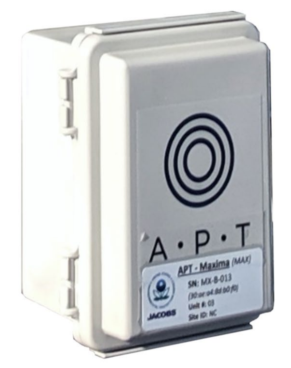

Supplemental Information

Abbreviations used in Supplemental Information

FRM	Federal Reference Method
FEM	Federal Equivalent Method
SOP	Standard Operating Procedure
QAPP	Quality Assurance Project Plan
QC	Quality Control

Supplemental Documentation	Attached	Description & URL or file path to documentation
Field observations and sensor data flags	☑	See DE-MAX-Page 6 of this testing report
Maintenance logs	☐	No logs recorded during testing
Standard operating procedure(s)	☐	U.S. EPA Office Of Research and Development SOP available upon request
Photos of equipment setup and testing	☑	See DE-MAX-Page 5 of this testing report
Product specifications sheet(s)	☐	N/A
Product manual(s)	☐	N/A
Data storage and transmission method	☑	See DE-MAX-Page 6 of this testing report
Data correction approach	☑	See DE-MAX-Page 6 of this testing report
Issues encountered	☑	See DE-MAX-Page 6 of this testing report
Data analysis/correction scripts and version	☑	Averaging and processing of data, calculation of performance metrics, and generation of figures and other supplementary material for analysis were obtained using Python 3.9.7 with the packages sensortoolkit v0.8.3b2, pandas 1.3.5, NumPy 1.21.2, Matplotlib 3.5.0, statsmodels 0.13.0, and seaborn 0.11.2. All packages are available from the Python Package Index (PyPI) at https://pypi.org. The integrated development environment (IDE) Spyder 5.1.5 was used for scripting and data visualization. Version control for the Python base, packages, and IDE were all managed by conda 4.11.0.
Air Monitoring Station QAPP	☐	U.S. EPA Office Of Research and Development QAPP available upon request
Summary of FRM/FEM monitor QC checks	☑	See DE-MAX-Page 7 of this testing report
Manufacturer website for FRM/FEM monitor	☑	Teledyne API: T640 Product website
FRM/FEM monitor manual	☑	Teledyne API: T640 Product Manual
FRM/FEM monitor specifications sheet(s)	☑	Teledyne API: T640 Specification Sheet
Other documents	☐	

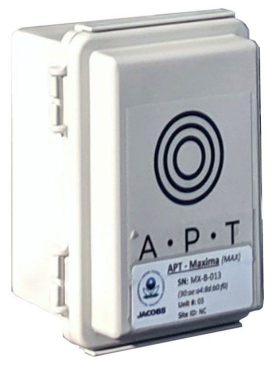

Supplemental Information: Photos of Testing Site and Equipment Setup

Site Description:
The DE site is located in Wilmington, Delaware at the intersection of Justison St. and MLK Blvd and has been operated at this site since 1999. This site is the state NCore site and represents urban population exposure to multiple pollution sources. This site meets all EPA *40 CFR Part 58 App D and E* siting criteria.

Figure 1: APT Maxima sensor (indicated by red arrow) attached to metal railing atop the sampling shelter at the monitoring site.

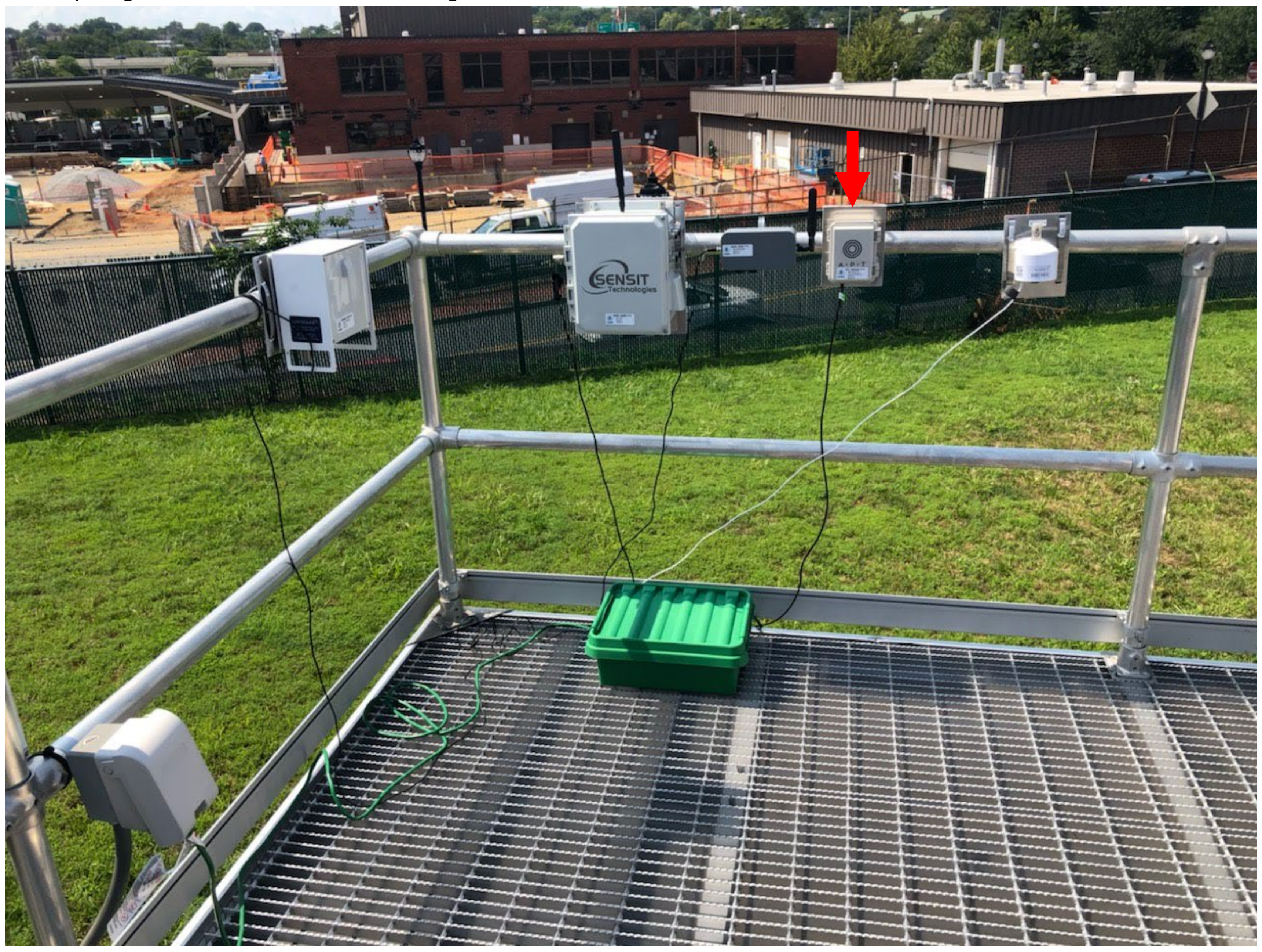

Figure 2: MLK Monitoring Station

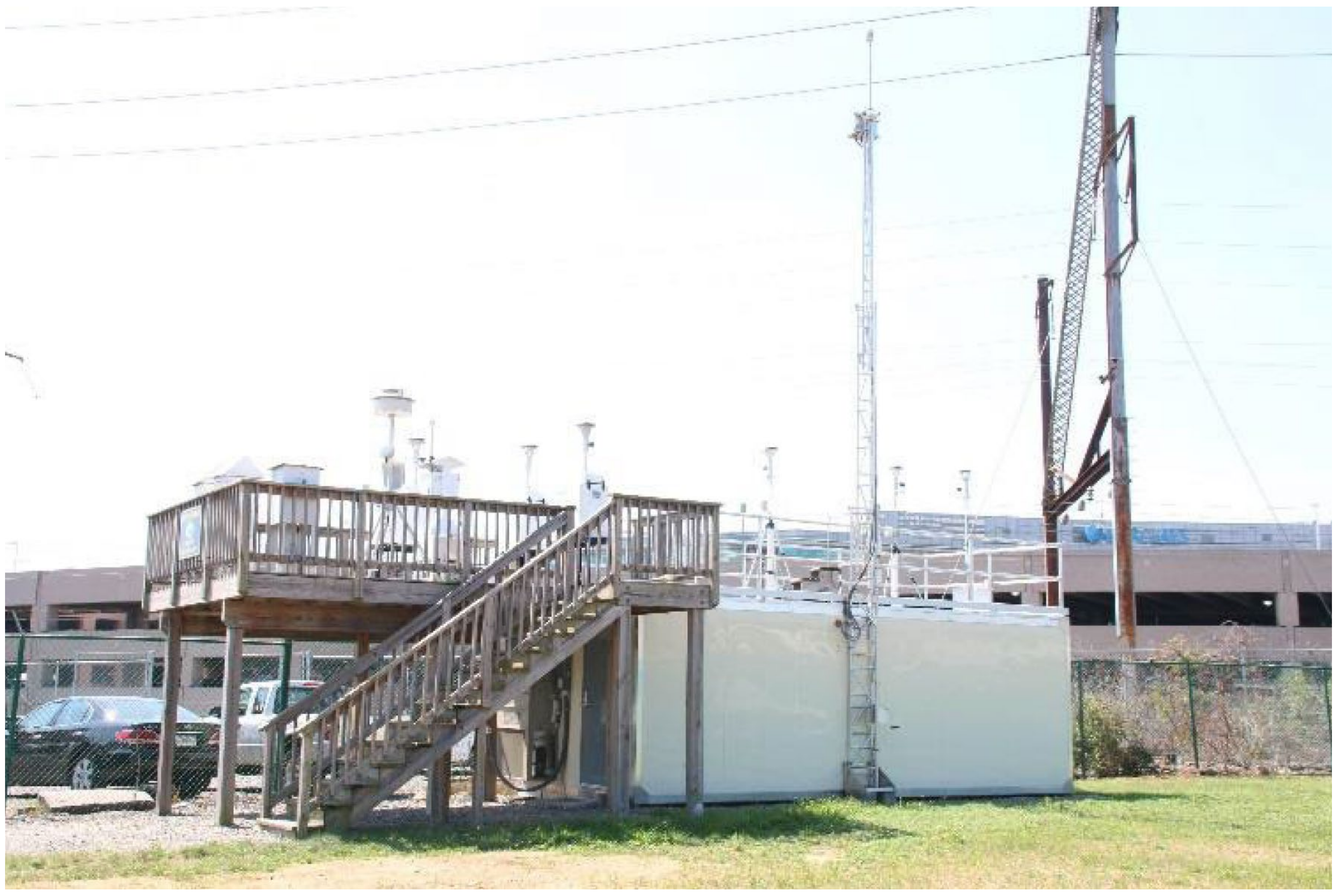

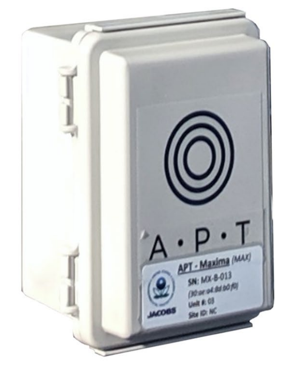

Supplemental Information: Data Storage, Correction Approach, and Issues Encountered

Data Storage and Transmission Method

The APT Maxima records data at 30 second intervals and stores the data on an internal microSD card and transmits data to the APT cloud server (last accessed 5/11/22) via Wi-Fi connection. The user interface for the Maxima sensors was under development during sensor testing, thus weekly raw data files were obtained from the internal MicroSD card. Each field site operator was provided two labeled MicroSD cards for the Maxima that they used to swap out each week. Data from the collected card was then read and processed off-site.

Data Correction Approach

This evaluation report reflects “out-of-the-box” performance of the APT Maxima. The Maxima data sets include two data columns contain $PM_{2.5}$ concentration measurements, “PM2.5(ug/m3)” and “PM2.5_Atm(ug/m3)”. These columns correspond to separate correction factors provided by the onboard OEM PM sensor (Plantower PMSA003). For this report, $PM_{2.5}$ measurements for the column labeled “PM2.5(ug/m3)” were selected for analysis.

After acquisition, the raw data was processed using the *sensortoolkit* python code library (v0.8.3b2). A continuous data set at the recorded sampling frequency was written to a .csv file. 1-hour and 24-hour averaged data sets were generated using a 75% completeness threshold and saved as separate .csv files. Outliers were NOT removed from data sets in order to assess “out-of-the-box” sensor performance.

Issues Encountered

Pre-deployment observations

- *Sampling internal abnormality:* Maxima devices did not continuously sample at 30-second intervals as stated in the sensor user guide. Instead, Maxima units occasionally sampled at 31 seconds. This increased sample interval is due to the accumulation of processing time over a certain number of samples. Each 30-second sample is a collection of exactly 30 seconds of sampling followed by a brief fraction of a second used for processing the collected data. Once processed, the next 30-second data collection period begins. When the sum of the processing time reaches one full second, the sample interval then jumps to 31 seconds. Data from this pre-deployment testing indicated that the processing speed for each sensor varies such that 31-second sampling duration occur at random intervals across Maxima units.

Field observations and sensor data flags

The following table contains data flags describing events that were encountered during the testing period. In addition to the sampling interval abnormality discussed in the pre-deployment observations, during the testing period it was observed that the Maxima occasionally recorded longer-duration sampling intervals, ranging from approximately 40 seconds to over 120 seconds. These longer intervals occurred approximately daily at seemingly random intervals, without any discernible pattern regarding the timing of these events. On 7/31/19 at approximately 14:30 UTC, the Maxima’s sampling interval began to alternate between the configured 30-second interval and various shorter intervals, ranging from 1 second to 16 seconds per sample. This behavior continued until 8/22/19 at 12:00 UTC when the sampling interval resumed the anticipated, consistent 30-second interval.

Start Time (UTC)	End Time (UTC)	Sensor Serial ID	Parameters Impacted	Flag
2019-08-10 20:07:00+00:00	2019-08-10 21:08:00+00:00	MAX_01	ALL	6.0-Sampling interval abnormality

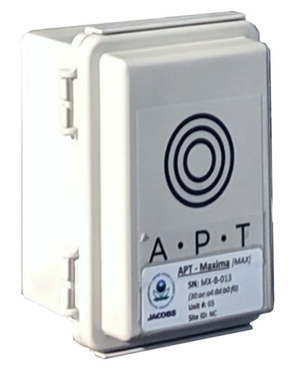

Description of Data Flags

AQS

The U.S. EPA's Air Quality System (AQS) is the Agency's primary ambient air monitoring data archive. A comprehensive list of data flags that are recorded alongside AQS data sets, referred to by U.S. EPA as 'qualifiers', can be found at the following link: https://aqs.epa.gov/aqsweb/documents/codetables/qualifiers.html

Invalidation of reference data: AQS qualifiers are organized by qualifier type, which indicates whether data logged alongside qualifier flags should be invalidated (set null). Qualifiers with type "Null Data Qualifier" are invalidated, and includes data logged during periods that coincide with QC checks (e.g., "BF-Precision/Zero/Span", "BJ- Operator Error", "BL - QA Audit", "AZ - QC Audit") among other events such as power outages. Data logged alongside qualifiers with type "Quality Assurance Qualifiers" are not invalidated and are included in this analysis (e.g., concentrations less than the federal MDL for the reference monitor "MD – Value less than MDL", QA reviewed values "Validated Value").

Data Flags Recorded During Testing

FRM/FEM Monitor	Timestamp (UTC)	Flag
Teledyne API T640 (Acquired via AQS)	2019-07-30 16:00:00+0000	AI - Insufficient Data (cannot calculate)
	2019-08-21 01:00:00+0000 to 2019-08-21 09:00:00+0000	AM - Miscellaneous Void
	2019-08-21 10:00:00+0000	AI - Insufficient Data (cannot calculate)
	2019-08-23 16:00:00+0000	AI - Insufficient Data (cannot calculate)

Meteorological Instrument	Timestamp (UTC)	Flag
Vaisala WXT-520 Temperature Monitor (Acquired via AQS)	2019-08-21 01:00:00+0000 to 2019-08-21 09:00:00+0000	AM - Miscellaneous Void
	2019-08-21 10:00:00+0000	AI - Insufficient Data (cannot calculate)
Vaisala WXT-520 Relative Humidity Monitor (Acquired via AQS)	2019-08-21 01:00:00+0000 to 2019-08-21 09:00:00+0000	AM - Miscellaneous Void
	2019-08-21 10:00:00+0000	AI - Insufficient Data (cannot calculate)

Testing Report - $PM_{2.5}$ Base Testing

Clarity Node

This report reflects out-of-the-box performance

Initial Base Testing - Wilmington, DE

U.S. Environmental Protection Agency
Office of Research and Development
PI: Clements.Andrea@epa.gov
919-541-1363

July 2019—August 2019

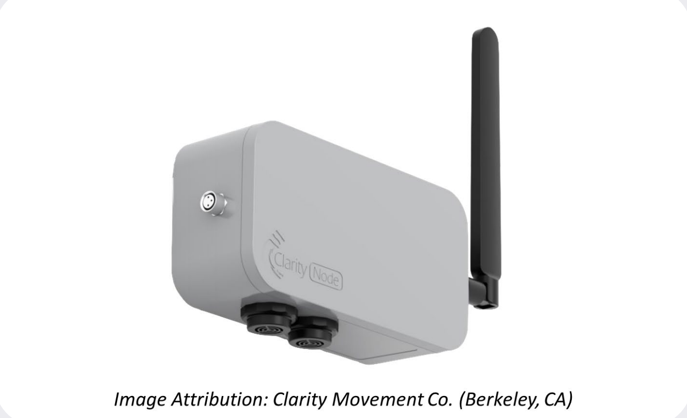

Image Attribution: Clarity Movement Co. (Berkeley, CA)

Deployment Details

Testing Organization and Site Information	
Testing organization (Name, Organization type, Contact website)	U.S. Environmental Protection Agency - Office of Research and Development<br>Federal Government<br>Air Sensor Toolbox | U.S. EPA Website
Testing location (City, State, Latitude and Longitude)	MLK - Corner Of MLK Blvd And Justison St<br>Wilmington, DE<br>39.7395, -75.5575
AQS site ID	10 - 003 - 2004
Sampling timeframe (MM-DD-YY)	07-26-19 to 08-25-19
Sensor data source	Clarity Dashboard download
Reference data source	AQS API download

Sensor Information			
Manufacturer, model	Clarity Node		
Device firmware version	Received by EPA May 2019		
Sampling time interval	3-minutes		
Sensor serial numbers	CNO_01		
Issues encountered during deployment?	☑	See DE-CNO-Page 6 of this testing report	

FRM/FEM Information	
Manufacturer, model, designation	Teledyne Advanced Pollution Instrumentation T640 FEM
Sampling time interval	1-hour averaging
Date of calibration	As required by 40 CFR Part 58 and the DE Ambient Air Quality Monitoring Program QAPP maintained by DNREC
Date of flowrate verification check	Monthly as required by 40 CFR Part 58 Appendix A
Description, date(s) of maintenance activities	N/A

Time Series Plots

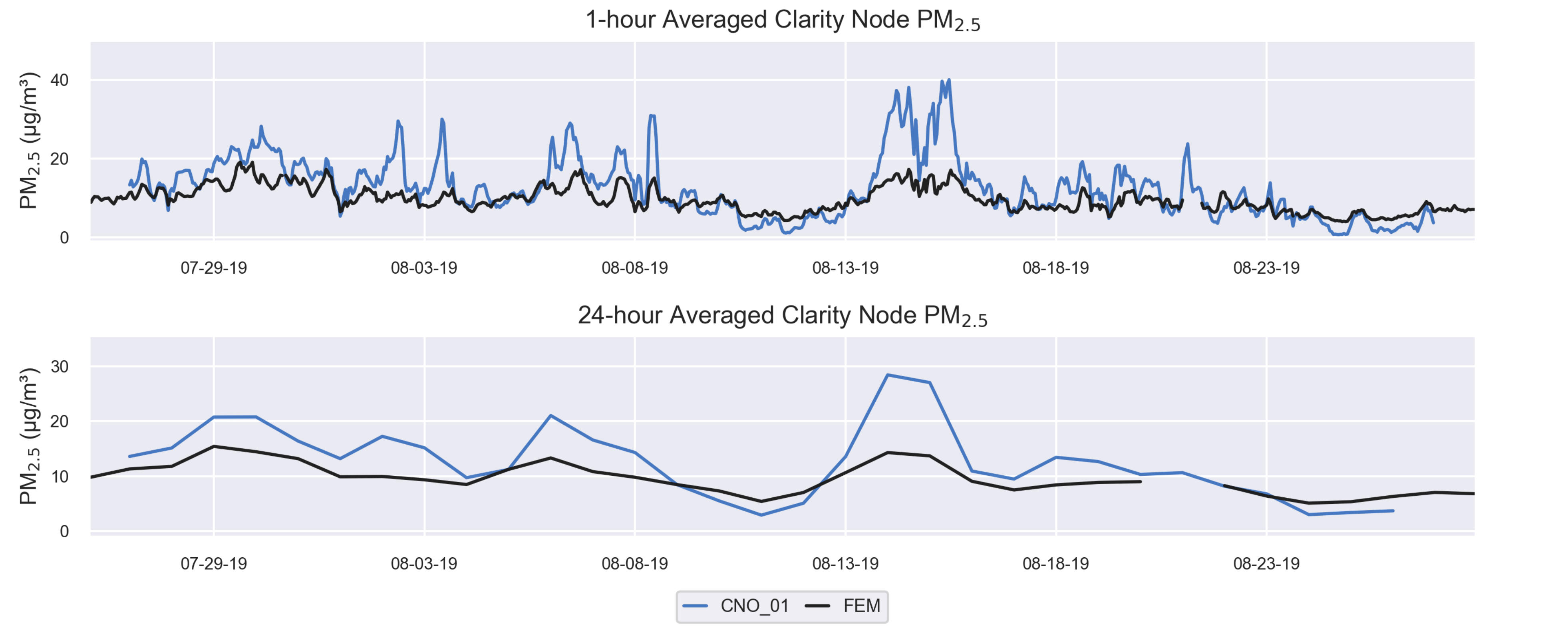

Scatter Plots: Comparison to FRM/FEM

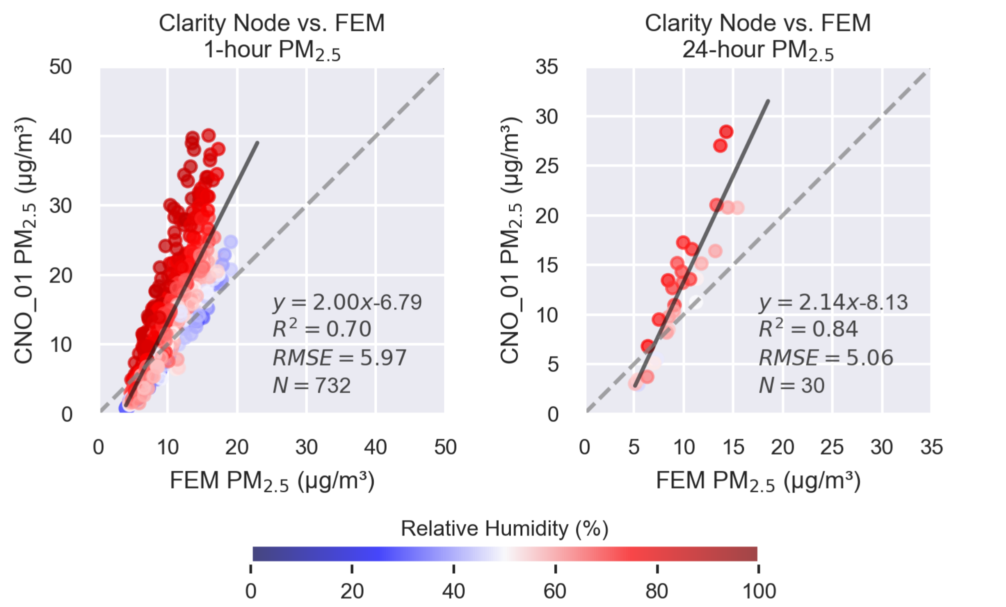

Range of FRM/FEM concentrations over duration of base test (µg/m³)	4.0-19.1 (1-hr), 5.1-15.4 (24-hr)
Number of 24-hr periods in FRM/FEM monitor measurements with a goal concentration ≥ 25 µg/m³	0

Performance Metrics

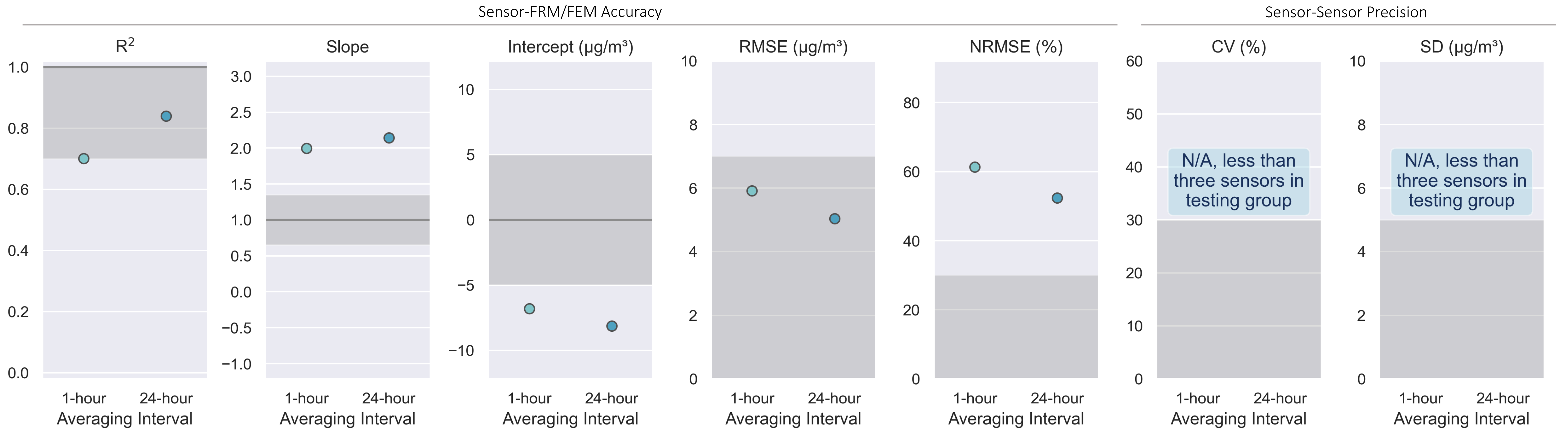

Meteorological Conditions During Deployment

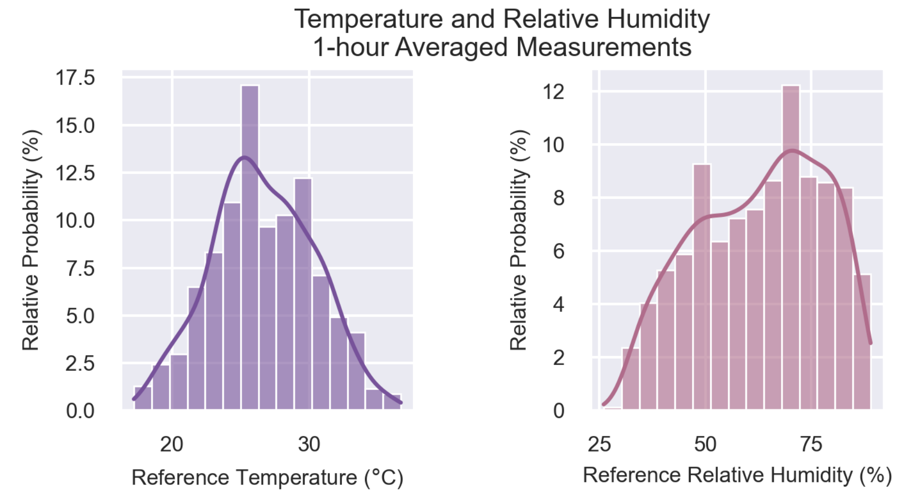

Number of 24-hr periods outside sensor manufacture-listed temperature operational range (-10 to 55 °C)	0
Number of 24-hr periods outside sensor manufacture-listed relative humidity operational range (10 to 98%)	0

Meteorological Influence

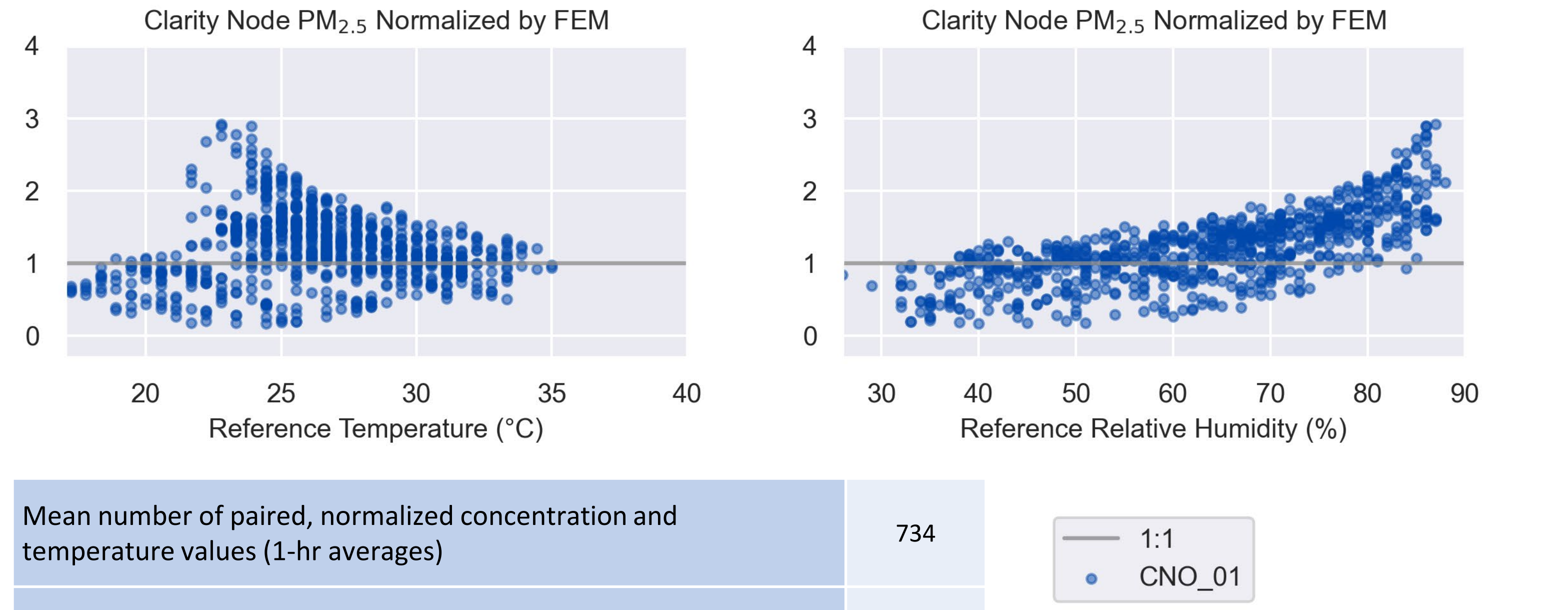

Mean number of paired, normalized concentration and temperature values (1-hr averages)	734
Mean number of paired, normalized concentration and relative humidity values (1-hr averages)	734

Testing Report - $PM_{2.5}$ Base Testing

Clarity Node

This report reflects out-of-the-box performance

Initial Base Testing - Wilmington, DE

U.S. Environmental Protection Agency
Office of Research and Development
PI: Clements.Andrea@epa.gov
919-541-1363

July 2019—August 2019

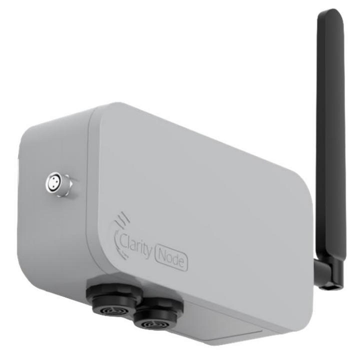

Image Attribution: Clarity Movement Co. (Berkeley, CA)

Tabular Statistics

Sensor-FRM/FEM Correlation

	Bias and Linearity						Data Quality			
	R^2		Slope		Intercept (µg/m³)		Uptime (%)		Number of paired sensor and FRM/FEM concentration values	
	1-Hour ●	24-Hour ●	1-Hour ○	24-Hour ○	1-Hour ○	24-Hour ○	1-Hour ●	24-Hour ●	1-Hour	24-Hour
Metric Target Range	≥ 0.70	≥ 0.70	1.0 ± 0.35	1.0 ± 0.35	-5 ≤ b ≤ 5	-5 ≤ b ≤ 5	75%*	75%*	-	-
Sensor CNO_01	0.70	0.84	2.00	2.14	-6.79	-8.13	100	100	732	30

	Error			
	RMSE (µg/m³)		NRMSE (%)	
	1-Hour ★	24-Hour ★	1-Hour ☆	24-Hour ☆
Metric Target Range	≤ 7.0	≤ 7.0	≤ 30.0	≤ 30.0
Deployment Value	5.9	5.0	61.4	52.4

Device-specific metrics (computed for each sensor in evaluation)
○○○ Metric value for none of devices tested falls within the target range
●○○ Metric value for one of devices tested falls within the target range
●●○ Metric value for two of devices tested falls within the target range
●●● Metric value for three of devices tested falls within the target range

Single-valued metrics (computed via entire evaluation dataset)
☆ Indicates that the metric value is not within the target range
★ Indicates that the metric value is within the target range

Sensor-Sensor Precision[1]

	Precision (between collocated sensors)				Data Quality	
	CV (%)		SD (µg/m³)		Number of concurrent sensor concentration pairs	
	1-Hour ☆	24-Hour ☆	1-Hour ☆	24-Hour ☆	1-Hour	24-Hour
Metric Target Range	≤ 30.0	≤ 30.0	≤ 5.0	≤ 5.0	-	-
Deployment Value	-	-	-	-	-	-

[1]Precision statistics are computed for evaluations with at least three collocated sensor units. Metric values are left blank for evaluations with two or fewer sensor units.

*This value is only a recommendation for ensuring data quality and is not included in the list of target values discussed in Section 4 of the Performance Testing Protocols, Metrics, and Target Values for Fine Particulate Matter Air Sensors document.

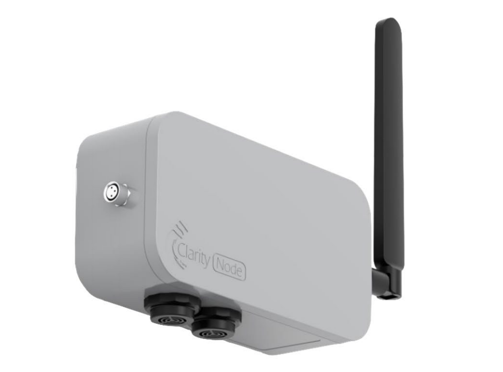

Image Attribution: Clarity Movement Co. (Berkeley, CA)

Sensor-FRM/FEM Scatter Plots

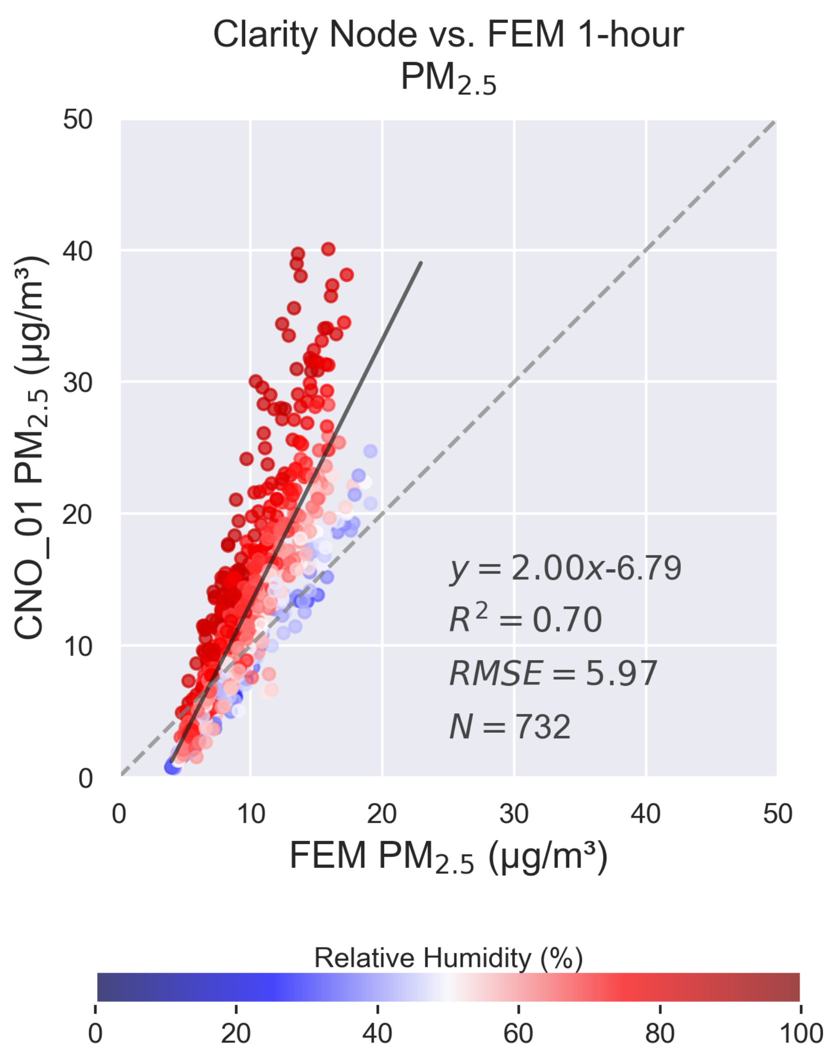

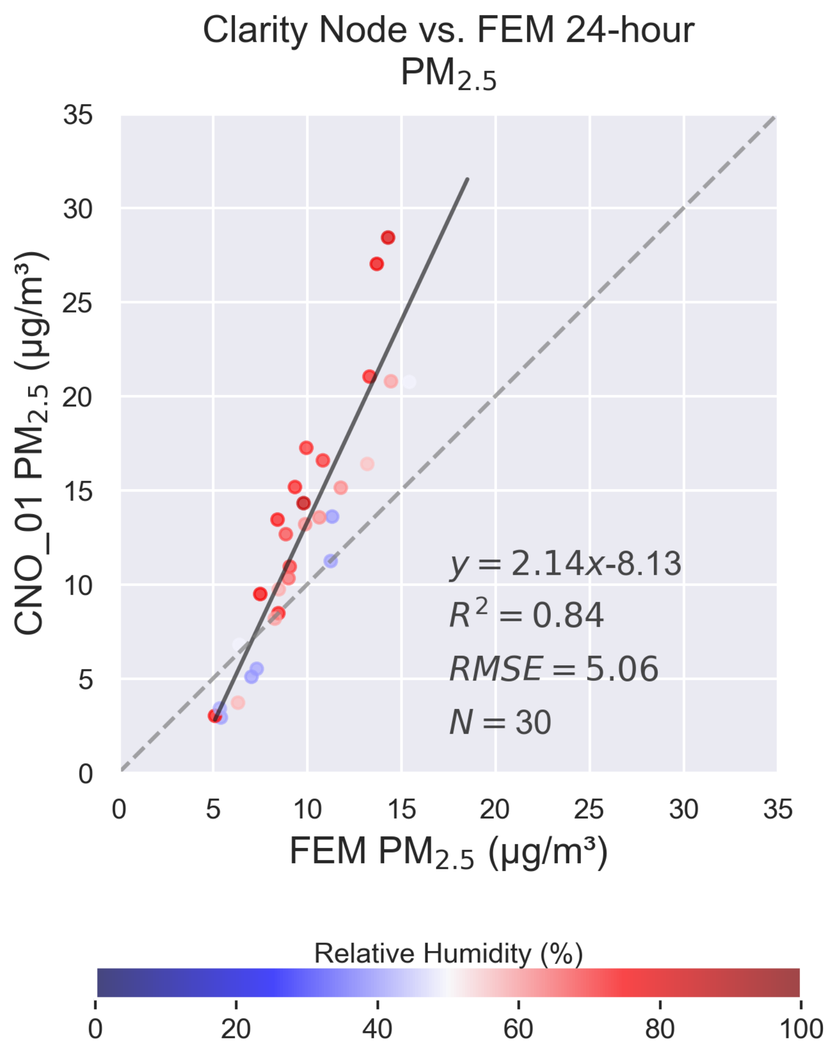

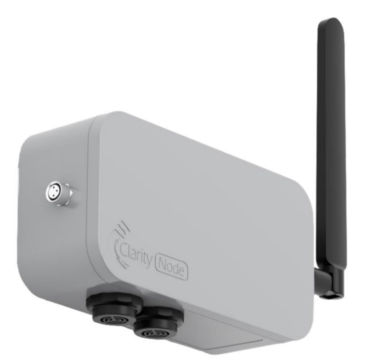

Image Attribution: Clarity Movement Co. (Berkeley, CA)

Supplemental Information

Abbreviations used in Supplemental Information

FRM	Federal Reference Method
FEM	Federal Equivalent Method
SOP	Standard Operating Procedure
QAPP	Quality Assurance Project Plan
QC	Quality Control

Supplemental Documentation	Attached	Description & URL or file path to documentation
Field observations and sensor data flags	☑	See DE-CNO-Page 6 of this testing report
Maintenance logs	☐	No logs recorded during testing
Standard operating procedure(s)	☐	U.S. EPA Office Of Research and Development SOP available upon request
Photos of equipment setup and testing	☑	See DE-CNO-Page 5 of this testing report
Product specifications sheet(s)	☐	N/A
Product manual(s)	☐	N/A
Data storage and transmission method	☑	See DE-CNO-Page 6 of this testing report
Data correction approach	☑	See DE-CNO-Page 6 of this testing report
Issues encountered	☑	See DE-CNO-Page 6 of this testing report
Data analysis/correction scripts and version	☑	Averaging and processing of data, calculation of performance metrics, and generation of figures and other supplementary material for analysis were obtained using Python 3.9.7 with the packages sensortoolkit v0.8.3b2, pandas 1.3.5, NumPy 1.21.2, Matplotlib 3.5.0, statsmodels 0.13.0, and seaborn 0.11.2. All packages are available from the Python Package Index (PyPI) at https://pypi.org. The integrated development environment (IDE) Spyder 5.1.5 was used for scripting and data visualization. Version control for the Python base, packages, and IDE were all managed by conda 4.11.0.
Air Monitoring Station QAPP	☐	U.S. EPA Office Of Research and Development QAPP available upon request
Summary of FRM/FEM monitor QC checks	☑	See DE-CNO-Page 7 of this testing report
Manufacturer website for FRM/FEM monitor	☑	Teledyne API: T640 Product website
FRM/FEM monitor manual	☑	Teledyne API: T640 Product Manual
FRM/FEM monitor specifications sheet(s)	☑	Teledyne API: T640 Specification Sheet
Other documents	☐	

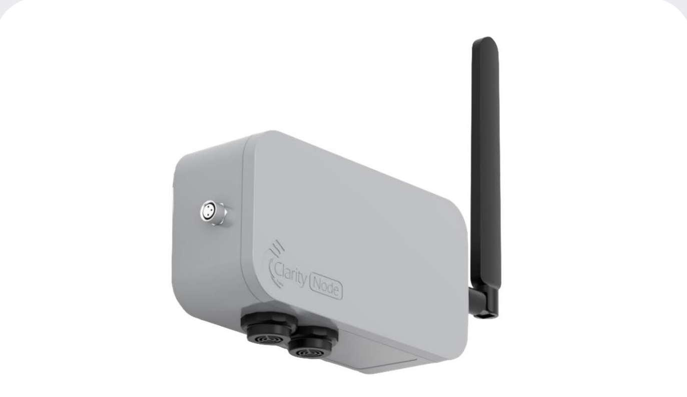

Image Attribution: Clarity Movement Co. (Berkeley, CA)

Supplemental Information: Photos of Testing Site and Equipment Setup

Site Description:

The DE site is located in Wilmington, Delaware at the intersection of Justison St. and MLK Blvd and has been operated at this site since 1999. This site is the state NCore site and represents urban population exposure to multiple pollution sources. This site meets all EPA *40 CFR Part 58 App D and E* siting criteria.

Figure 1: Clarity Node sensor (indicated by red arrow) attached to metal railing atop the sampling shelter at the monitoring site.

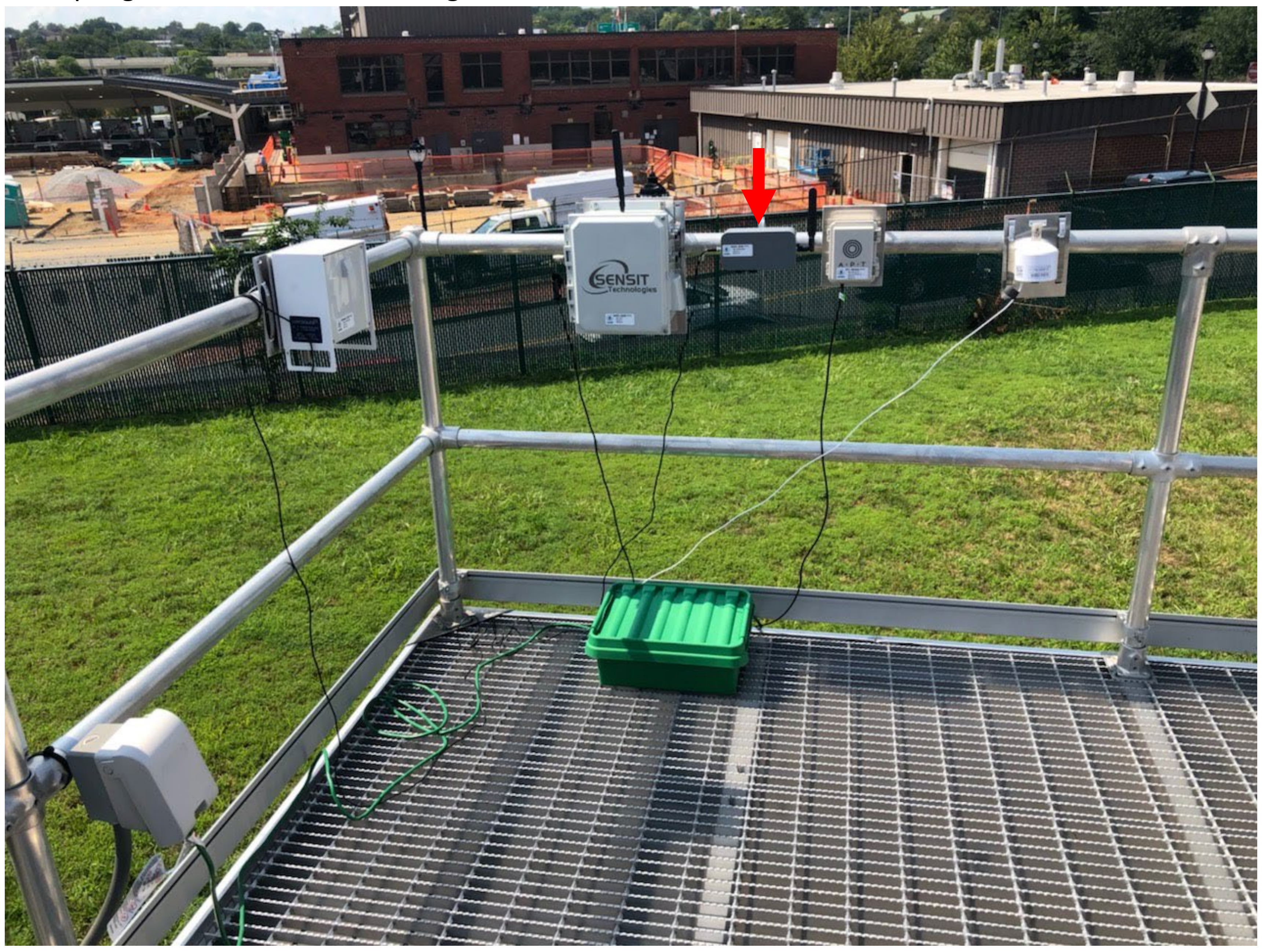

Figure 2: MLK Monitoring Station

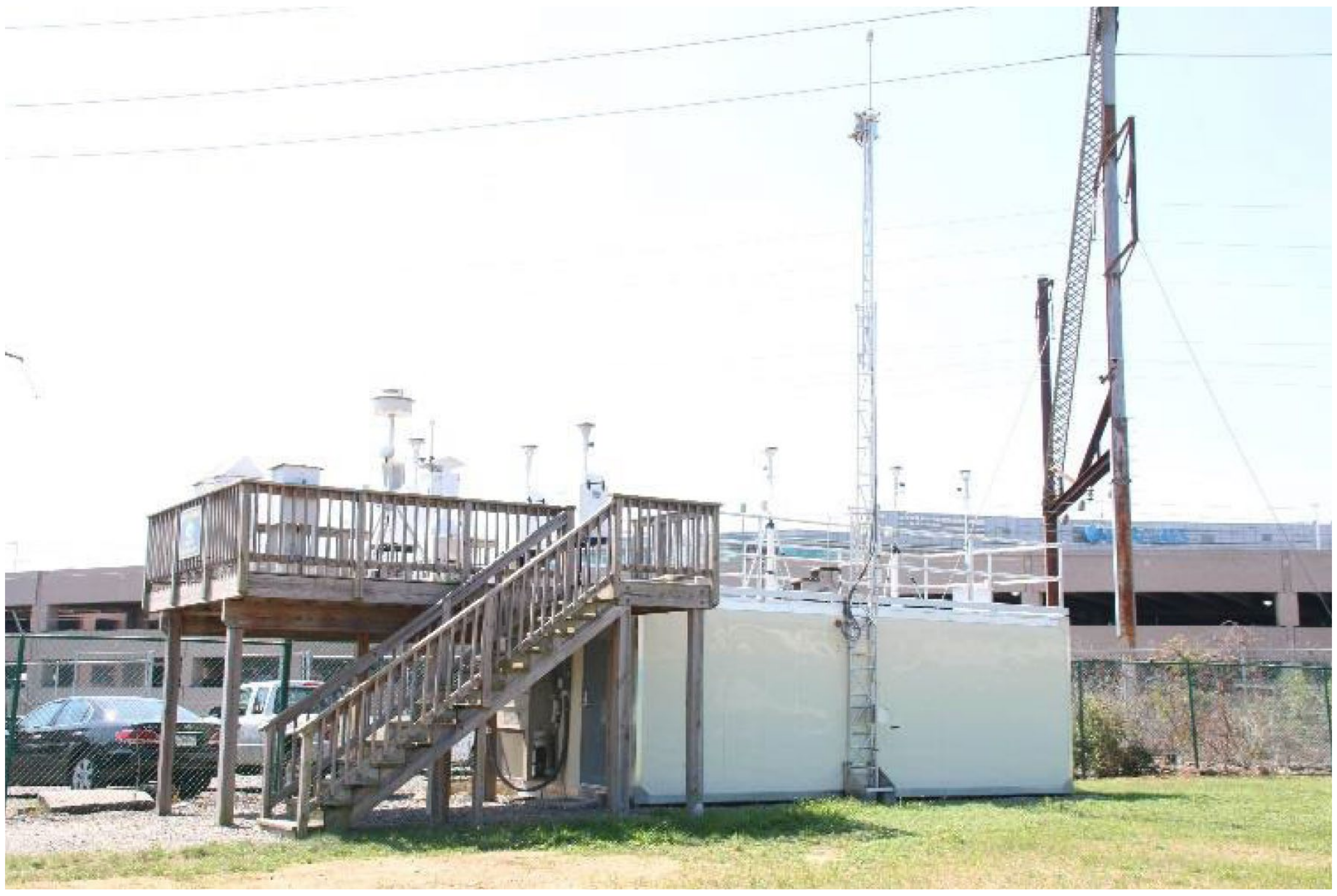

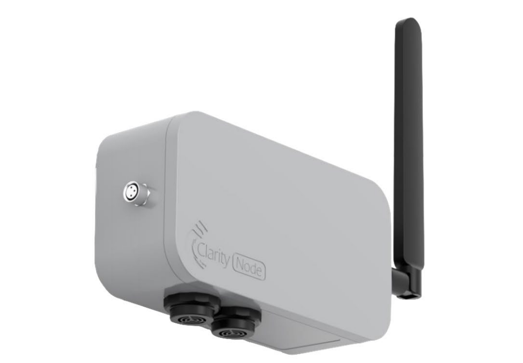

Image Attribution: Clarity Movement Co. (Berkeley, CA)

Data Storage and Transmission Method

The Clarity Node transmits data via cellular SIM card. Weekly raw data collections were performed over Wi-Fi using the Clarity dashboard (*last accessed 5/11/22*). The observed sampling interval fluctuated and is further discussed under pre-deployment observations below. On average, the Node models sampled every 2-3 minutes, and the Node-S sampled every 15-17 minutes.

Data Correction Approach

This evaluation report reflects "out-of-the-box" performance of the Clarity Node. The manufacturer provides a dashboard feature for calibrating sensor data against collocated reference monitors. Calibrated data were NOT used for this evaluation. Prospective consumers may get better performance from this device if they utilize this feature.

After acquisition, the raw data was processed using the *sensortoolkit* python code library (v0.8.3b2). A continuous data set at the recorded sampling frequency was written to a .csv file. 1-hour and 24-hour averaged data sets were generated using a 75% completeness threshold and saved as separate .csv files. Due to the variable sampling interval observed for Clarity Node units, 75% completeness was determined against the number of data points that would be recorded within 1-hour/24-hour periods if the sensor were to log measurements at the most commonly recorded sampling interval (i.e., the mode of recorded intervals). Outliers were NOT removed from data sets in order to assess "out-of-the-box" sensor performance.

Issues Encountered

Pre-deployment observations

- *Unsteady sampling interval:* Clarity Node sensors were received by EPA on May 28, 2019. A lab bench-top evaluation was performed from May 29, 2019 – May 31, 2019. During bench-top testing, it was observed that the sampling intervals noted by Clarity (5 minutes for non-solar Node, 15 minutes for the solar Node-S) were not reflective of recorded intervals. Communication with the vendor concluded that the sampling interval will fluctuate depending on the settings, battery charge, and strength of the cellular signal and that this sampling interval cannot be adjusted by the user at present. On average, the Node models sampled every 2-3 minutes, and the Node-S sampled every 15-17 minutes.

Field observations and sensor data flags

The following table contains data flags describing events that were encountered during the testing period. A brief power loss to the sensor occurred on 7/29/2019 and lasted approximately 20 minutes. This event occurred when the field technician was at the monitoring site, indicating that the outage was likely attributed to operator error.

Start Time (UTC)	End Time (UTC)	Sensor Serial ID	Parameters Impacted	Flag
2019-07-29 15:38:00+00:00	2019-07-29 15:58:00+00:00	CNO_01	ALL	9.0-Data Loss - Power Connection Error

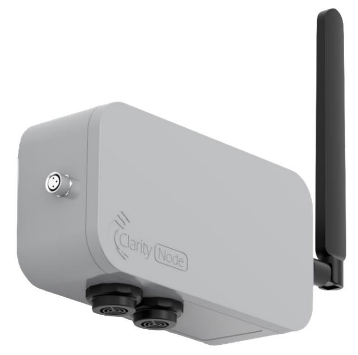

Image Attribution: Clarity Movement Co. (Berkeley, CA)

Description of Data Flags

AQS

The U.S. EPA's Air Quality System (AQS) is the Agency's primary ambient air monitoring data archive. A comprehensive list of data flags that are recorded alongside AQS data sets, referred to by U.S. EPA as 'qualifiers', can be found at the following link: https://aqs.epa.gov/aqsweb/documents/codetables/qualifiers.html

Invalidation of reference data: AQS qualifiers are organized by qualifier type, which indicates whether data logged alongside qualifier flags should be invalidated (set null). Qualifiers with type "Null Data Qualifier" are invalidated, and includes data logged during periods that coincide with QC checks (e.g., "BF-Precision/Zero/Span", "BJ- Operator Error", "BL - QA Audit", "AZ - QC Audit") among other events such as power outages. Data logged alongside qualifiers with type "Quality Assurance Qualifiers" are not invalidated and are included in this analysis (e.g., concentrations less than the federal MDL for the reference monitor "MD – Value less than MDL", QA reviewed values "Validated Value").

Data Flags Recorded During Testing

FRM/FEM Monitor	Timestamp (UTC)	Flag
Teledyne API T640 (Acquired via AQS)	2019-07-30 16:00:00+0000	AI - Insufficient Data (cannot calculate)
	2019-08-21 01:00:00+0000 to 2019-08-21 09:00:00+0000	AM - Miscellaneous Void
	2019-08-21 10:00:00+0000	AI - Insufficient Data (cannot calculate)
	2019-08-23 16:00:00+0000	AI - Insufficient Data (cannot calculate)

Meteorological Instrument	Timestamp (UTC)	Flag
Vaisala WXT-520 Temperature Monitor (Acquired via AQS)	2019-08-21 01:00:00+0000 to 2019-08-21 09:00:00+0000	AM - Miscellaneous Void
	2019-08-21 10:00:00+0000	AI - Insufficient Data (cannot calculate)
Vaisala WXT-520 Relative Humidity Monitor (Acquired via AQS)	2019-08-21 01:00:00+0000 to 2019-08-21 09:00:00+0000	AM - Miscellaneous Void
	2019-08-21 10:00:00+0000	AI - Insufficient Data (cannot calculate)

Testing Report - $PM_{2.5}$ Base Testing

PurpleAir PA-II-SD

This report reflects out-of-the-box performance

Initial Base Testing - Wilmington, DE

U.S. Environmental Protection Agency
Office of Research and Development
PI: Clements.Andrea@epa.gov
919-541-1363

July 2019—August 2019

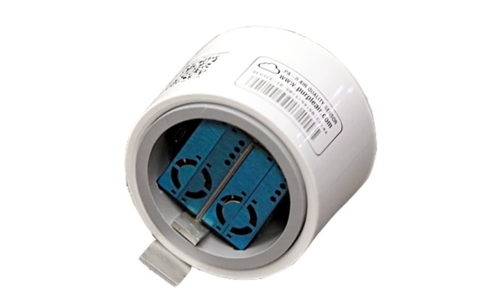

Deployment Details

Testing Organization and Site Information	
Testing organization (Name, Organization type, Contact website)	U.S. Environmental Protection Agency - Office of Research and Development<br>Federal Government<br>Air Sensor Toolbox | U.S. EPA Website
Testing location (City, State, Latitude and Longitude)	MLK - Corner Of MLK Blvd And Justison St<br>Wilmington, DE<br>39.7395, -75.5575
AQS site ID	10 - 003 - 2004
Sampling timeframe (MM-DD-YY)	07-30-19 to 08-29-19
Sensor data source	Onboard MicroSD card
Reference data source	AQS API download

Sensor Information			
Manufacturer, model	PurpleAir PA-II-SD		
Device firmware version	v4.02		
Sampling time interval	2-minutes		
Sensor serial numbers	PAR_01		
Issues encountered during deployment?	☑	See DE-PAR-Page 6 of this testing report	

FRM/FEM Information	
Manufacturer, model, designation	Teledyne Advanced Pollution Instrumentation T640 FEM
Sampling time interval	1-hour averaging
Date of calibration	As required by 40 CFR Part 58 and the DE Ambient Air Quality Monitoring Program QAPP maintained by DNREC
Date of flowrate verification check	Monthly as required by 40 CFR Part 58 Appendix A
Description, date(s) of maintenance activities	N/A

Time Series Plots

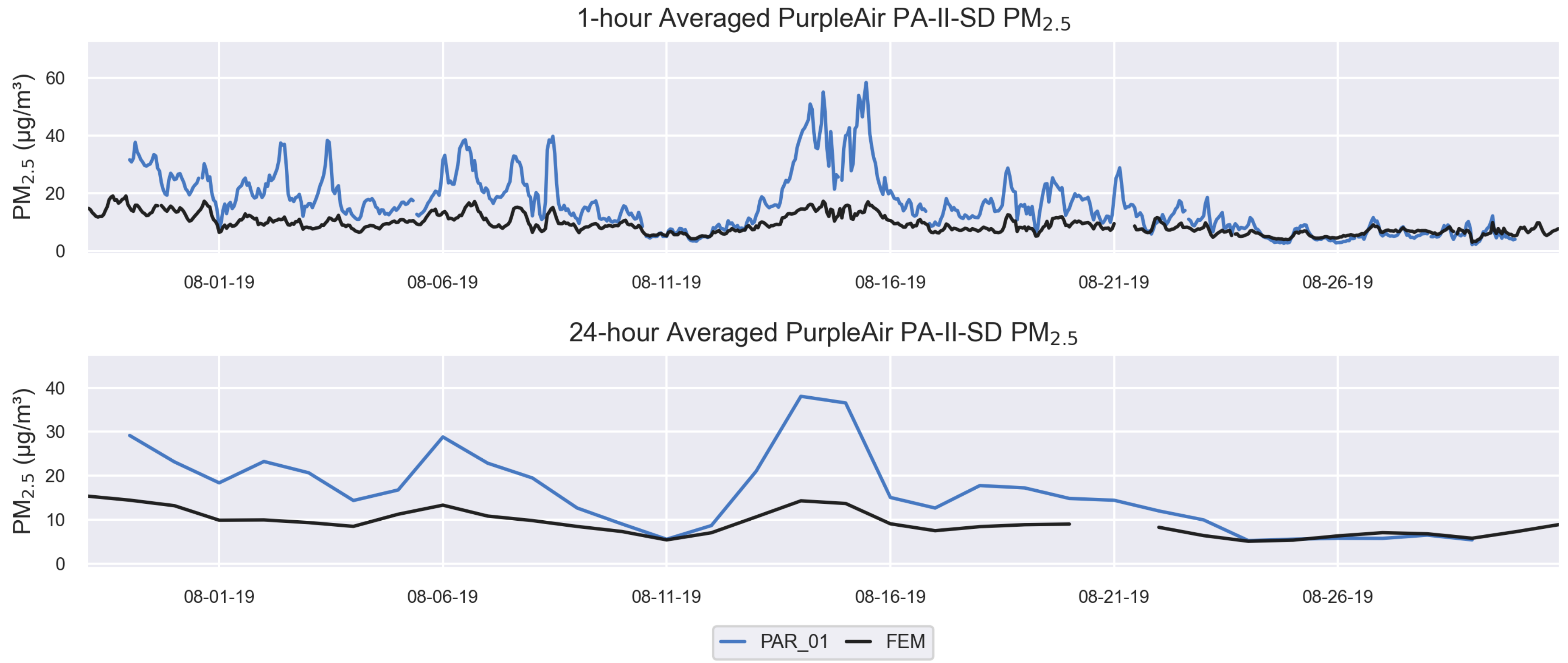

Scatter Plots: Comparison to FRM/FEM

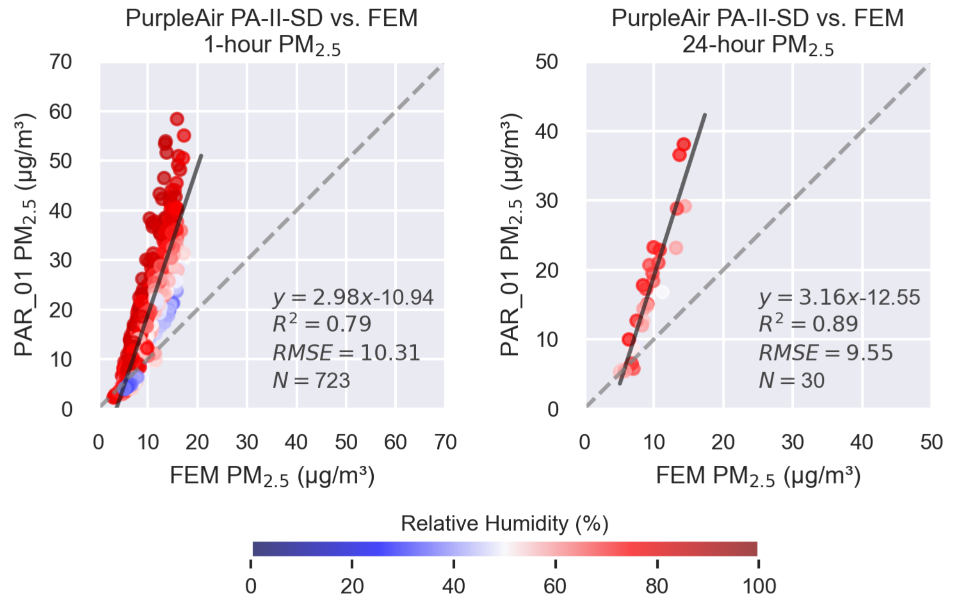

Range of FRM/FEM concentrations over duration of base test (µg/m³)	3.1-17.3 (1-hr), 5.1-14.3 (24-hr)
Number of 24-hr periods in FRM/FEM monitor measurements with a goal concentration ≥ 25 µg/m³	0

Performance Metrics

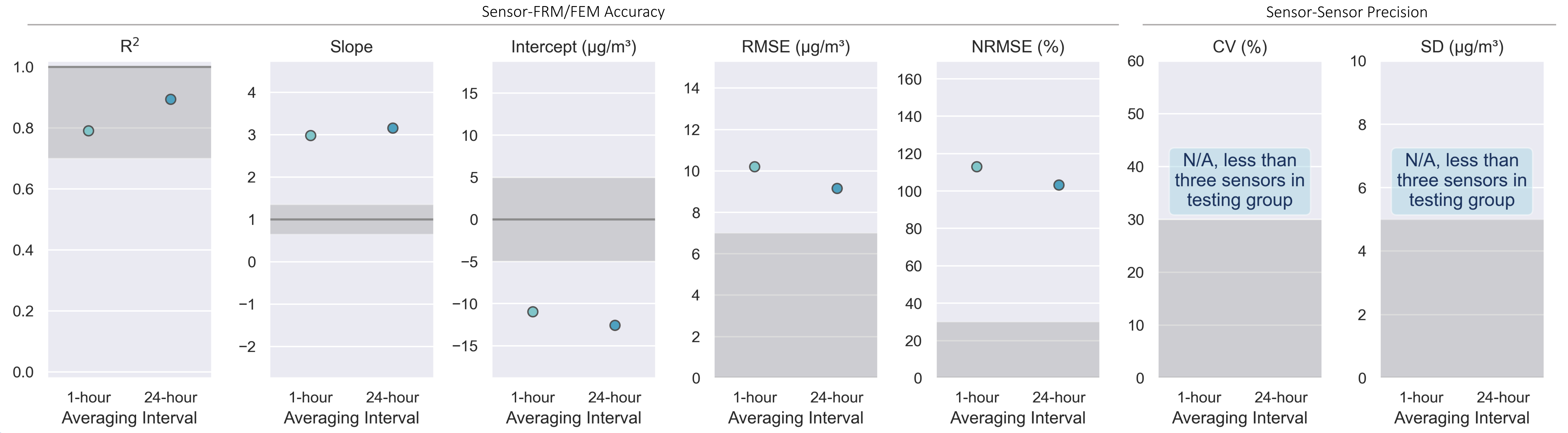

Meteorological Conditions During Deployment

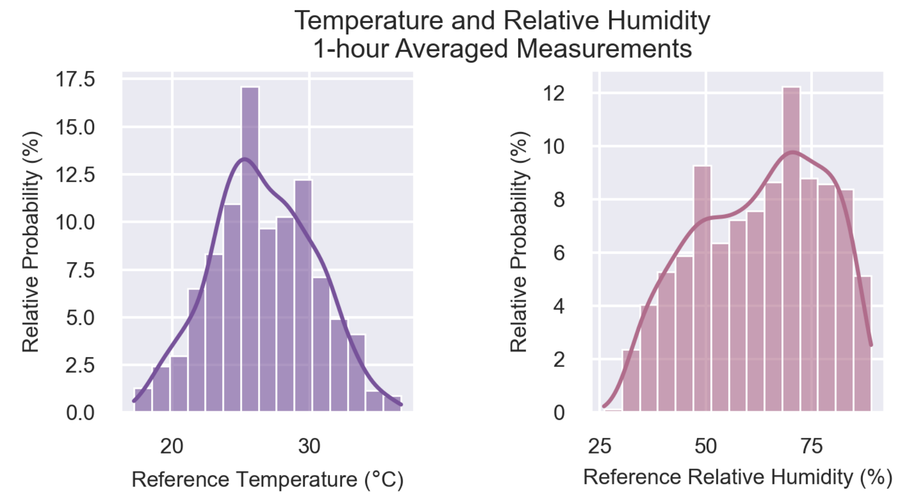

Number of 24-hr periods outside sensor manufacture-listed temperature operational range (no operational range specified)	-
Number of 24-hr periods outside sensor manufacture-listed relative humidity operational range (no operational range specified)	-

Meteorological Influence

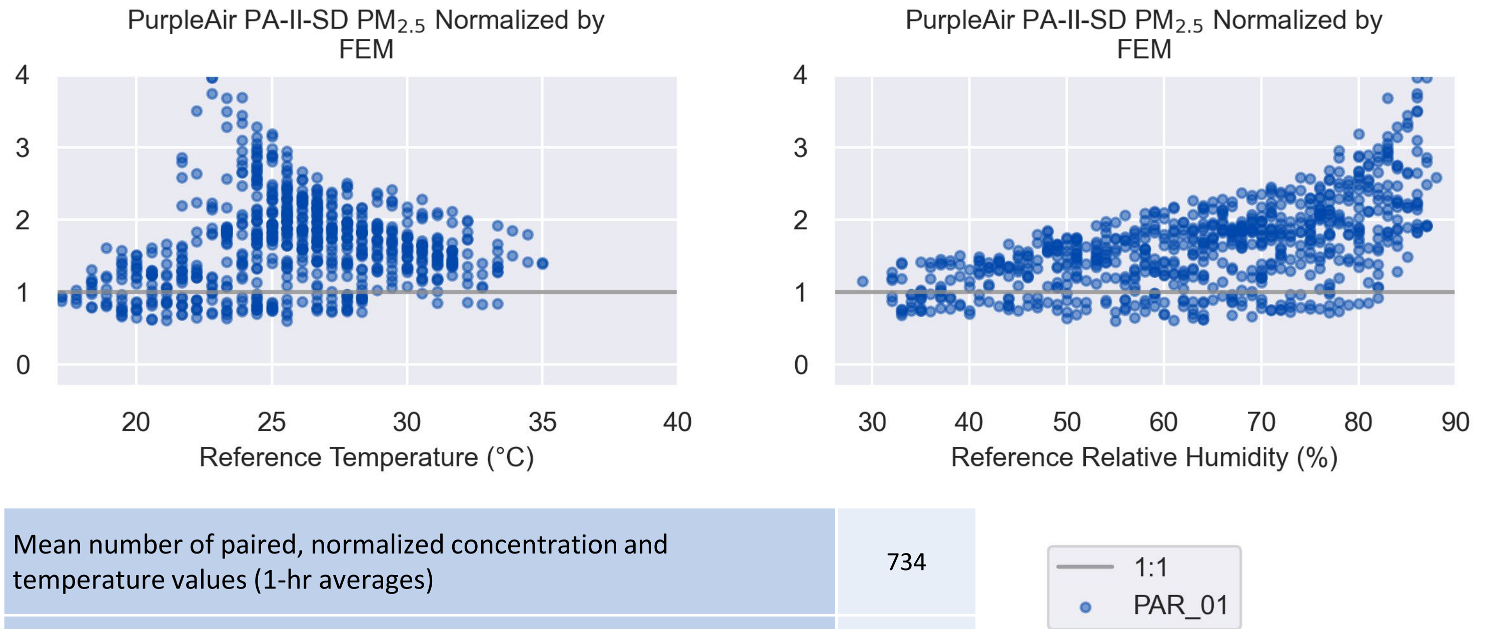

Mean number of paired, normalized concentration and temperature values (1-hr averages)	734
Mean number of paired, normalized concentration and relative humidity values (1-hr averages)	734

Testing Report - $PM_{2.5}$ Base Testing

PurpleAir PA-II-SD

This report reflects out-of-the-box performance

Initial Base Testing - Wilmington, DE

U.S. Environmental Protection Agency
Office of Research and Development
PI: Clements.Andrea@epa.gov
919-541-1363

July 2019—August 2019

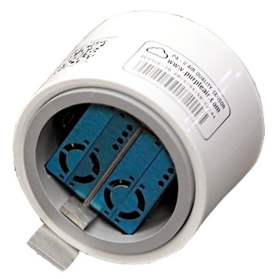

Tabular Statistics

Sensor-FRM/FEM Correlation

	Bias and Linearity						Data Quality			
	R^2		Slope		Intercept (µg/m³)		Uptime (%)		Number of paired sensor and FRM/FEM concentration values	
	1-Hour ●	24-Hour ●	1-Hour ○	24-Hour ○	1-Hour ○	24-Hour ○	1-Hour ●	24-Hour ●	1-Hour	24-Hour
Metric Target Range	≥ 0.70	≥ 0.70	1.0 ± 0.35	1.0 ± 0.35	-5 ≤ b ≤ 5	-5 ≤ b ≤ 5	75%*	75%*	-	-
Sensor PAR_01	0.79	0.89	2.98	3.16	-10.94	-12.55	99	100	723	30

	Error			
	RMSE (µg/m³)		NRMSE (%)	
	1-Hour ☆	24-Hour ☆	1-Hour ☆	24-Hour ☆
Metric Target Range	≤ 7.0	≤ 7.0	≤ 30.0	≤ 30.0
Deployment Value	10.2	9.2	113.1	103.3

Device-specific metrics (computed for each sensor in evaluation)
○○○ Metric value for none of devices tested falls within the target range
●○○ Metric value for one of devices tested falls within the target range
●●○ Metric value for two of devices tested falls within the target range
●●● Metric value for three of devices tested falls within the target range

Single-valued metrics (computed via entire evaluation dataset)
☆ Indicates that the metric value is not within the target range
★ Indicates that the metric value is within the target range

Sensor-Sensor Precision[1]

	Precision (between collocated sensors)				Data Quality	
	CV (%)		SD (µg/m³)		Number of concurrent sensor concentration pairs	
	1-Hour ☆	24-Hour ☆	1-Hour ☆	24-Hour ☆	1-Hour	24-Hour
Metric Target Range	≤ 30.0	≤ 30.0	≤ 5.0	≤ 5.0	-	-
Deployment Value	-	-	-	-	-	-

[1]Precision statistics are computed for evaluations with at least three collocated sensor units. Metric values are left blank for evaluations with two or fewer sensor units.

*This value is only a recommendation for ensuring data quality and is not included in the list of target values discussed in Section 4 of the Performance Testing Protocols, Metrics, and Target Values for Fine Particulate Matter Air Sensors document.

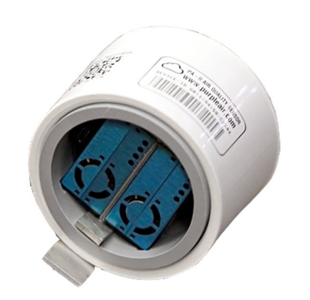

Sensor-FRM/FEM Scatter Plots

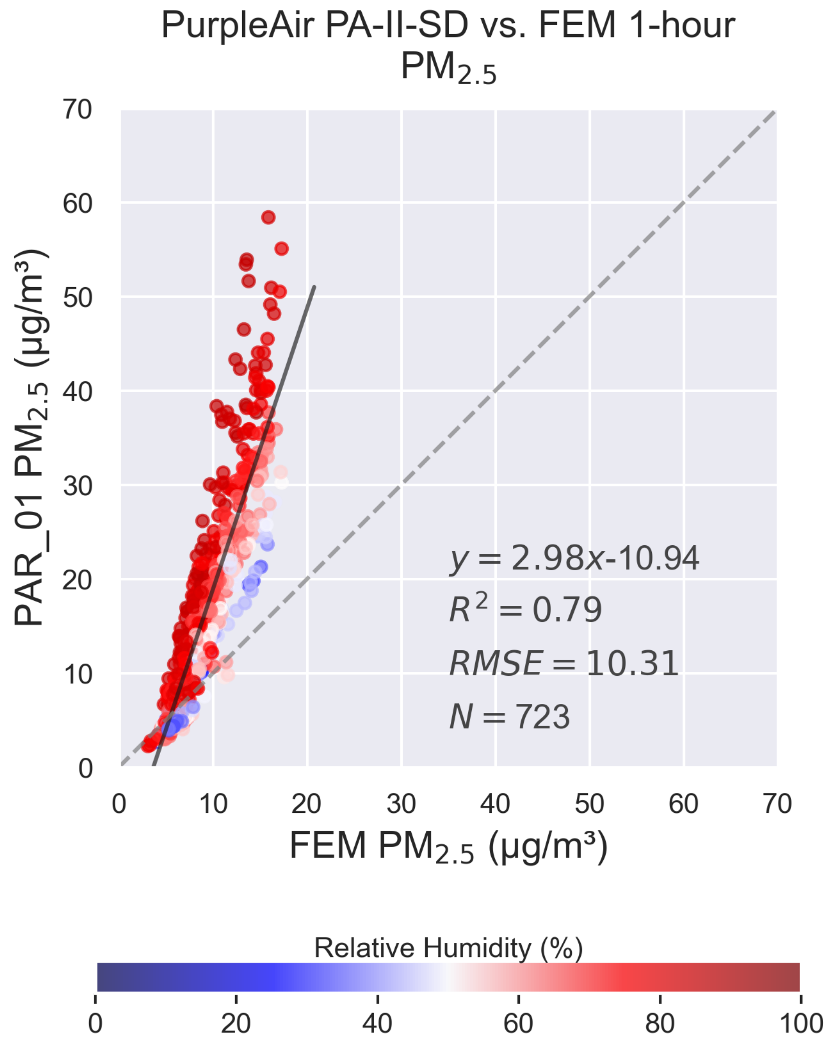

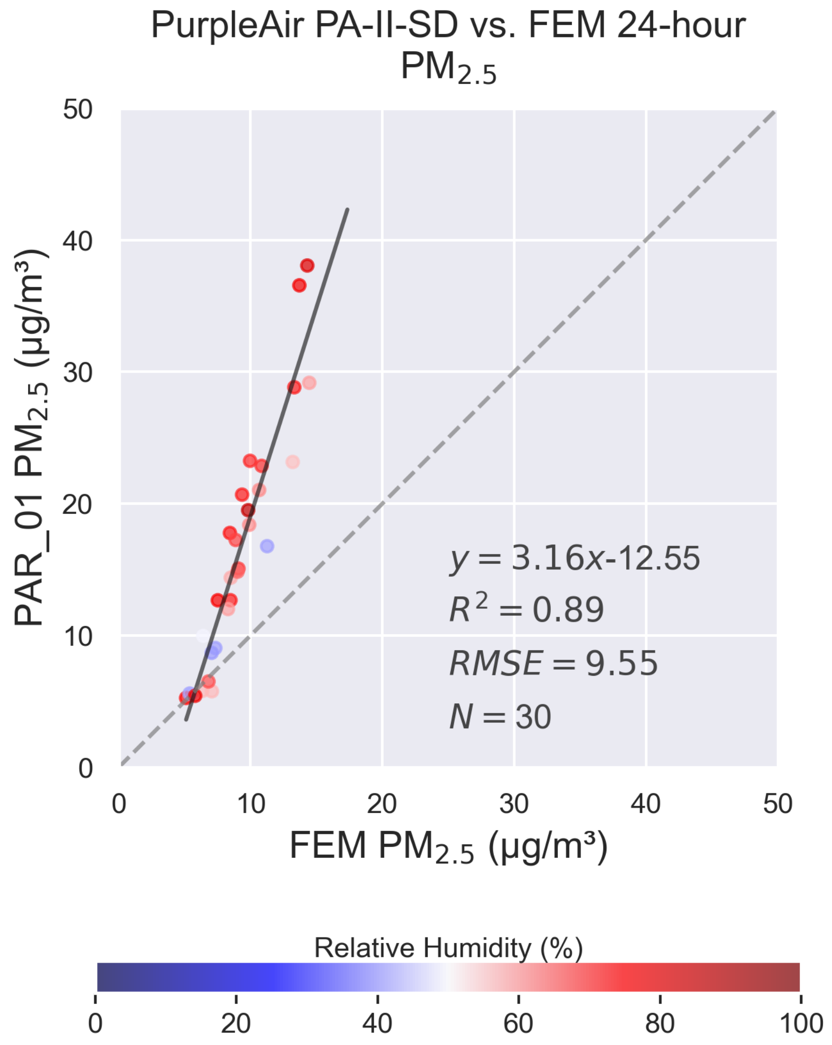

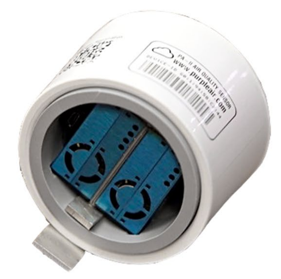

Supplemental Information

Abbreviations used in Supplemental Information

FRM	Federal Reference Method
FEM	Federal Equivalent Method
SOP	Standard Operating Procedure
QAPP	Quality Assurance Project Plan
QC	Quality Control

Supplemental Documentation	Attached	Description & URL or file path to documentation
Field observations and sensor data flags	☑	See DE-PAR-Page 7 of this testing report
Maintenance logs	☐	No logs recorded during testing
Standard operating procedure(s)	☐	U.S. EPA Office Of Research and Development SOP available upon request
Photos of equipment setup and testing	☑	See DE-PAR-Page 5 of this testing report
Product specifications sheet(s)	☑	PurpleAir: PA-II-SD Product website
Product manual(s)	☐	N/A
Data storage and transmission method	☑	See DE-PAR-Page 6 of this testing report
Data correction approach	☑	See DE-PAR-Page 6 of this testing report
Issues encountered	☑	See DE-PAR-Page 6 of this testing report
Data analysis/correction scripts and version	☑	Averaging and processing of data, calculation of performance metrics, and generation of figures and other supplementary material for analysis were obtained using Python 3.9.7 with the packages sensortoolkit v0.8.3b2, pandas 1.3.5, NumPy 1.21.2, Matplotlib 3.5.0, statsmodels 0.13.0, and seaborn 0.11.2. All packages are available from the Python Package Index (PyPI) at https://pypi.org. The integrated development environment (IDE) Spyder 5.1.5 was used for scripting and data visualization. Version control for the Python base, packages, and IDE were all managed by conda 4.11.0.
Air Monitoring Station QAPP	☐	U.S. EPA Office Of Research and Development QAPP available upon request
Summary of FRM/FEM monitor QC checks	☑	See DE-PAR-Page 8 of this testing report
Manufacturer website for FRM/FEM monitor	☑	Teledyne API: T640 Product website
FRM/FEM monitor manual	☑	Teledyne API: T640 Product Manual
FRM/FEM monitor specifications sheet(s)	☑	Teledyne API: T640 Specification Sheet
Other documents	☐	

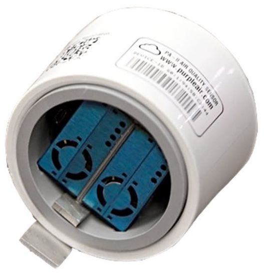

Supplemental Information: Photos of Testing Site and Equipment Setup

Site Description:

The DE site is located in Wilmington, Delaware at the intersection of Justison St. and MLK Blvd and has been operated at this site since 1999. This site is the state NCore site and represents urban population exposure to multiple pollution sources. This site meets all EPA *40 CFR Part 58 App D and E* siting criteria.

Figure 1: PurpleAir PA-II-SD sensor (indicated by red arrow) attached to metal railing atop the sampling shelter at the monitoring site.

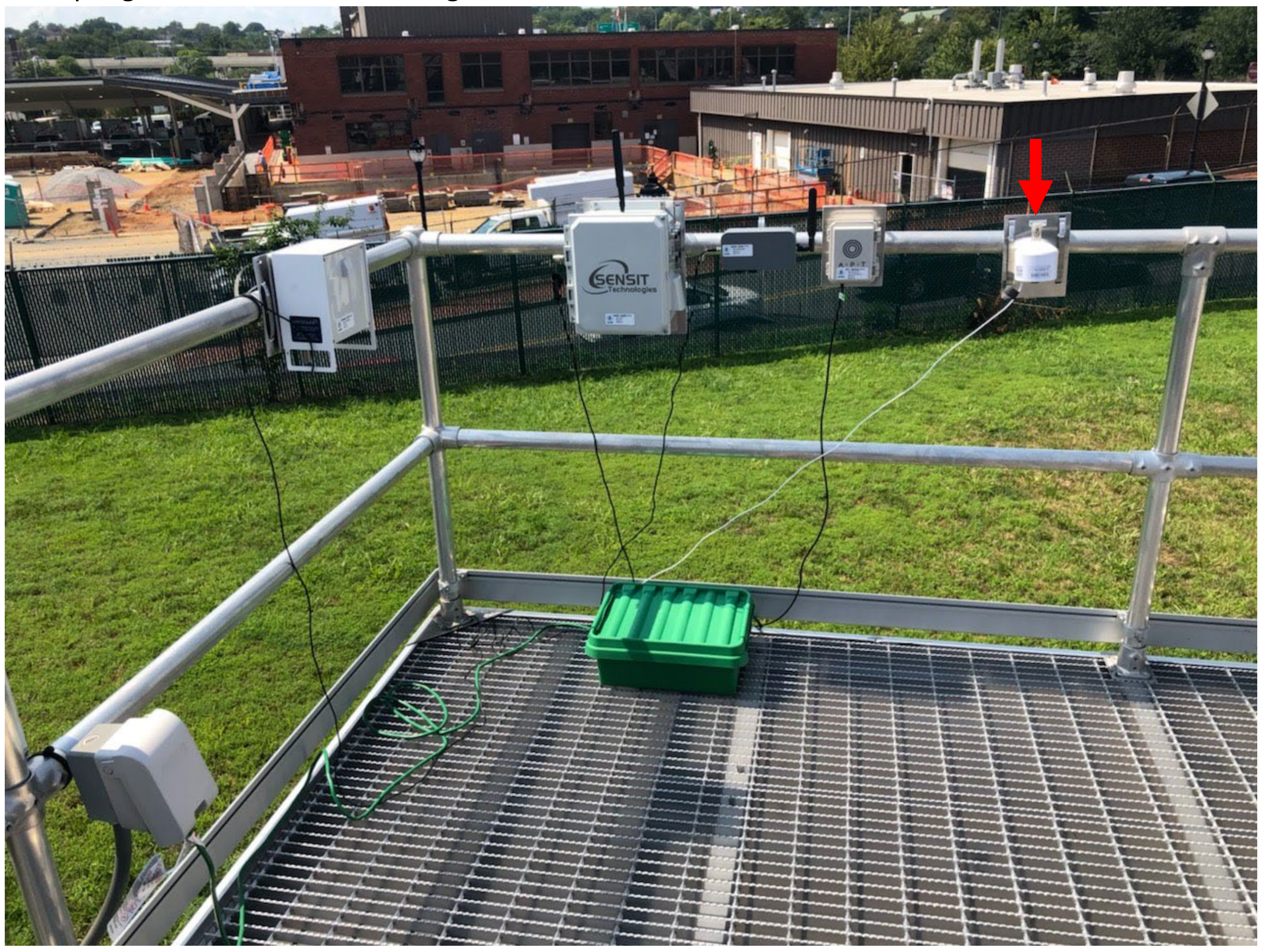

Figure 2: MLK Monitoring Station

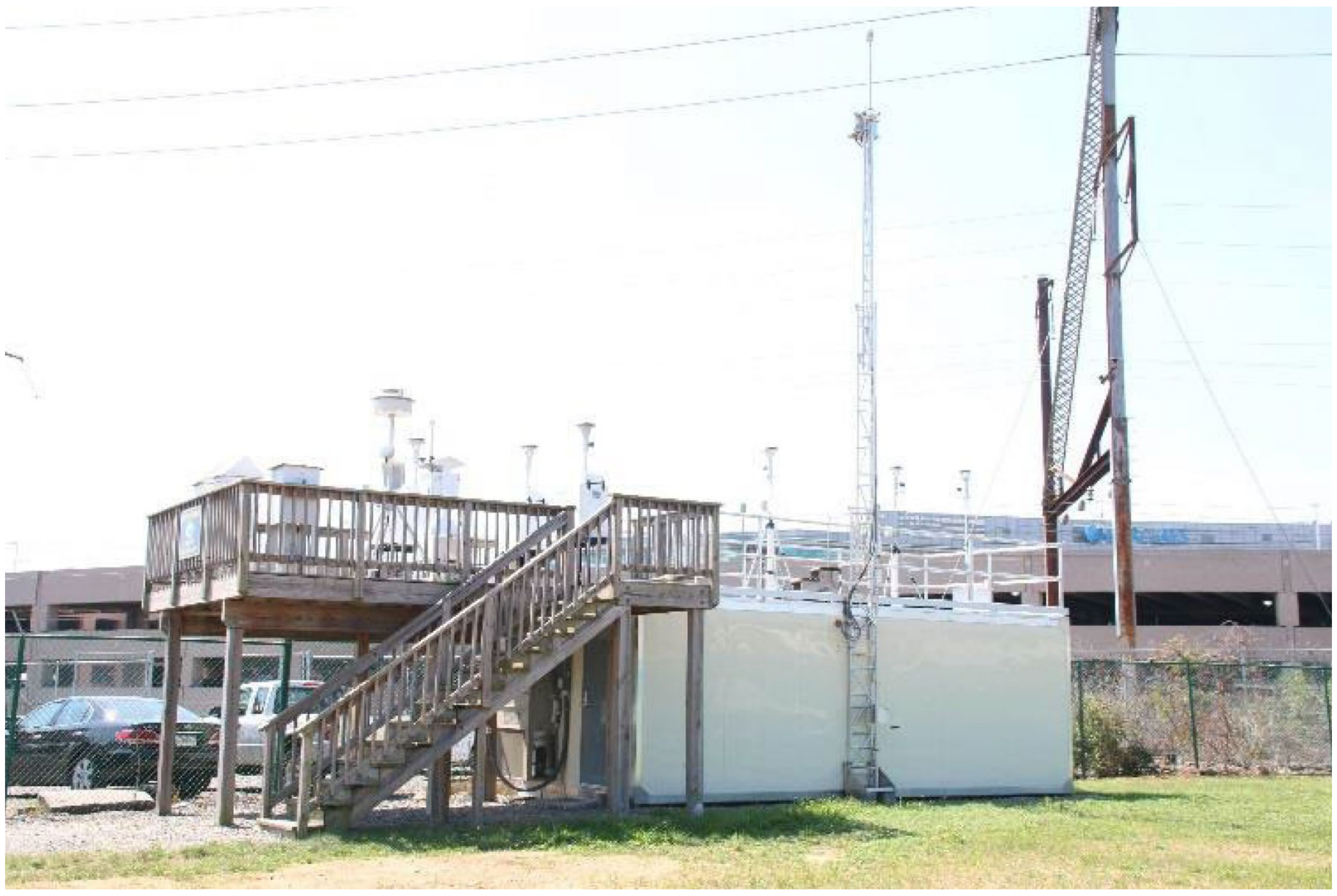

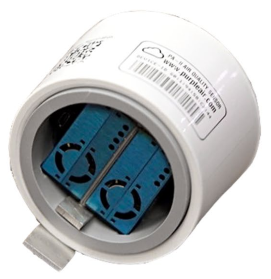

Data Storage and Transmission Method

The PurpleAir PA-II-SD records data at a 2-minute[1] sampling interval. Data is transmitted to the PurpleAir Cloud via Wi-Fi and stored on an internal MicroSD card. Due to the differing file structure between remote and direct download methods and the lag associated with cloud data download, weekly raw data were obtained primarily via onboard SD card. Each field site operator was provided two labeled MicroSD cards for sensor units that they used to swap out each week. Data from the collected card was then read and processed off-site.

Data Correction Approach

Data sets recorded by PurpleAir PA-II-SD contain two correction factors for PM concentrations, referred to internally as “CF=ATM” and “CF=1”. $PM_{2.5}$ concentrations are recorded concurrently for each correction factor, and corresponding concentrations agree closely up to approximately 30 $\mu g/m^3$, above which one correction factor (CF=1) reflects concentrations roughly 1.5 times concentrations for the other correction factor (CF=ATM). During a separate analysis by ORD, concentrations for the higher CF=1 correction factor were determined to have slightly higher correlation with collocated reference measurements. As a result, data for the higher correction factor were used for this study. Prior to firmware version 5.00 (released on 11/30/2019), headers for concentrations corresponding to each correction factor were incorrectly labeled (i.e., CF=1 was labeled CF=ATM and vice versa). Testing indicated in this report was completed prior to this firmware update, and thus data sets acquired for PA-II-SD units had swapped $PM_{2.5}$ CF labels. Recorded PA-II-SD data sets indicate that the CF=ATM correction factor was the higher CF as labeled (following firmware update 5.00, this data channel corresponds to CF=1) and sensor data shown in this report correspond to this correction factor.

After acquisition, the raw data was processed using the *sensortoolkit* python code library (v0.8.3b2). A continuous data set at the recorded sampling frequency was written to a .csv file. 1-hour and 24-hour averaged data sets were generated using a 75% completeness threshold and saved as separate .csv files. Simultaneous measurement from the PurpleAir PA-II’s dual PMS5003 sensors (labeled channels A and B) allows for identification of outliers resulting in large-scale disagreement between recorded values. The outlier removal method of Barkjohn et al. 2021[2] was applied to both 1-hour and 24-hour averaged A and B measurement pairs. This method includes thresholds for the absolute difference and the percent difference between hourly channel values. A-B channel averages were not included for hours where the absolute difference between channel values was greater than 5 $\mu g/m^3$ and the percent difference exceeded 70%. This outlier removal method is in use on the AirNow Fire and Smoke map and has been used for this evaluation since it requires no external information (e.g., data from the monitor) and no assumptions about typical PM2.5 concentrations (e.g., removing data over a certain threshold concentration).

Issues Encountered

Pre-deployment observations

- Prior to the testing period indicated in this report, the PurpleAir PA-II-SD unit was briefly collocated for a period of approximately one week at EPA’s Ambient Monitoring Innovative Research Station (AIRS) in Research Triangle Park, NC. The unit was tested for proper operation. Units that did not pass this pre-deployment testing were returned to the manufacturer and constituted approximately 1 in 10 units tested.

Field observations and sensor data flags are included on the following page

[1] As of version 4.02, updated across devices on 5/31/2019. Prior to this firmware version, devices recorded measurements at 80-second intervals
[2] Barkjohn, K. K., Gantt, B., and Clements, A. L.: Development and application of a United States-wide correction for PM2.5 data collected with the PurpleAir sensor, Atmos. Meas. Tech., 14, 4617–4637, https://doi.org/10.5194/amt-14-4617-2021, 2021.

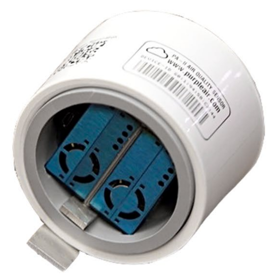

Field observations and sensor data flags

The following table contains data flags describing events that were encountered during the testing period. The PurpleAir was configured to record measurements at intervals. Occasionally, the sampling interval would be doubled to 240 seconds. These longer sampling intervals would occur on a roughly hourly basis; however, the occurrence of these longer intervals was highly unpredictable. Additionally, even longer sampling interval durations ranging from approximately 10 to 30 minutes would be logged with no discernible pattern in the frequency or occurrence. These longer 10-to-30-minute abnormalities are indicated in the table below.

Start Time (UTC)	End Time (UTC)	Sensor Serial ID	Parameters Impacted	Flag
2019-08-05 08:26:00+00:00	2019-08-05 08:48:00+00:00	PAR_01	ALL	6.0-Sampling interval abnormality
2019-08-10 11:17:00+00:00	2019-08-10 11:41:00+00:00	PAR_01	ALL	6.0-Sampling interval abnormality
2019-08-12 12:45:00+00:00	2019-08-12 13:05:00+00:00	PAR_01	ALL	6.0-Sampling interval abnormality
2019-08-14 20:13:00+00:00	2019-08-14 20:29:00+00:00	PAR_01	ALL	6.0-Sampling interval abnormality
2019-08-16 19:17:00+00:00	2019-08-16 19:45:00+00:00	PAR_01	ALL	6.0-Sampling interval abnormality
2019-08-19 10:15:00+00:00	2019-08-19 10:27:00+00:00	PAR_01	ALL	6.0-Sampling interval abnormality
2019-08-21 07:47:00+00:00	2019-08-21 08:03:00+00:00	PAR_01	ALL	6.0-Sampling interval abnormality
2019-08-24 09:59:00+00:00	2019-08-24 10:13:00+00:00	PAR_01	ALL	6.0-Sampling interval abnormality
2019-08-26 09:39:00+00:00	2019-08-26 09:57:00+00:00	PAR_01	ALL	6.0-Sampling interval abnormality
2019-08-28 00:27:00+00:00	2019-08-28 00:47:00+00:00	PAR_01	ALL	6.0-Sampling interval abnormality

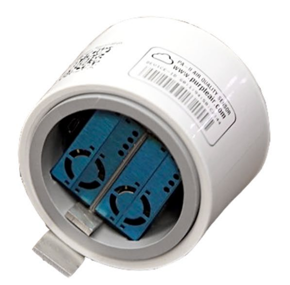

Description of Data Flags

AQS

The U.S. EPA's Air Quality System (AQS) is the Agency's primary ambient air monitoring data archive. A comprehensive list of data flags that are recorded alongside AQS data sets, referred to by U.S. EPA as 'qualifiers', can be found at the following link: https://aqs.epa.gov/aqsweb/documents/codetables/qualifiers.html

Invalidation of reference data: AQS qualifiers are organized by qualifier type, which indicates whether data logged alongside qualifier flags should be invalidated (set null). Qualifiers with type "Null Data Qualifier" are invalidated, and includes data logged during periods that coincide with QC checks (e.g., "BF-Precision/Zero/Span", "BJ- Operator Error", "BL - QA Audit", "AZ - QC Audit") among other events such as power outages. Data logged alongside qualifiers with type "Quality Assurance Qualifiers" are not invalidated and are included in this analysis (e.g., concentrations less than the federal MDL for the reference monitor "MD – Value less than MDL", QA reviewed values "Validated Value").

Data Flags Recorded During Testing

FRM/FEM Monitor	Timestamp (UTC)	Flag
Teledyne API T640 (Acquired via AQS)	2019-07-30 16:00:00+0000	AI - Insufficient Data (cannot calculate)
	2019-08-21 01:00:00+0000 to 2019-08-21 09:00:00+0000	AM - Miscellaneous Void
	2019-08-21 10:00:00+0000	AI - Insufficient Data (cannot calculate)
	2019-08-23 16:00:00+0000	AI - Insufficient Data (cannot calculate)
	2019-08-28 15:00:00+0000	AI - Insufficient Data (cannot calculate)

Meteorological Instrument	Timestamp (UTC)	Flag
Vaisala WXT-520 Temperature Monitor (Acquired via AQS)	2019-08-21 01:00:00+0000 to 2019-08-21 09:00:00+0000	AM - Miscellaneous Void
	2019-08-21 10:00:00+0000	AI - Insufficient Data (cannot calculate)
Vaisala WXT-520 Relative Humidity Monitor (Acquired via AQS)	2019-08-21 01:00:00+0000 to 2019-08-21 09:00:00+0000	AM - Miscellaneous Void
	2019-08-21 10:00:00+0000	AI - Insufficient Data (cannot calculate)

Testing Report - $PM_{2.5}$ Base Testing

SENSIT RAMP

This report reflects out-of-the-box performance

Initial Base Testing - Wilmington, DE
U.S. Environmental Protection Agency
Office of Research and Development
PI: Clements.Andrea@epa.gov
919-541-1363
July 2019—August 2019

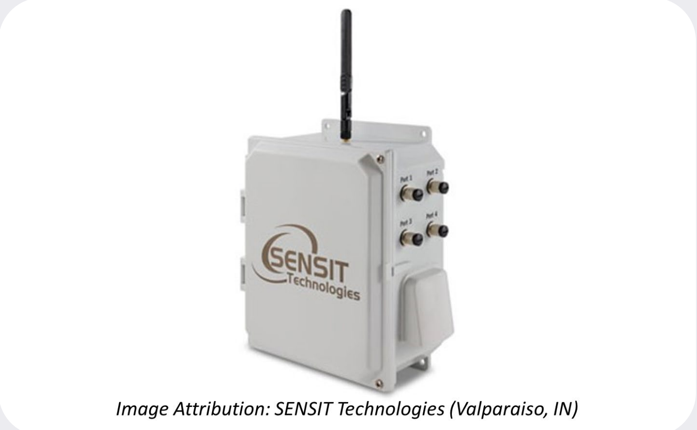

Image Attribution: SENSIT Technologies (Valparaiso, IN)

Deployment Details

Testing Organization and Site Information	
Testing organization (Name, Organization type, Contact website)	U.S. Environmental Protection Agency - Office of Research and Development<br>Federal Government<br>Air Sensor Toolbox | U.S. EPA Website
Testing location (City, State, Latitude and Longitude)	MLK - Corner Of MLK Blvd And Justison St<br>Wilmington, DE<br>39.7395, -75.5575
AQS site ID	10 - 003 - 2004
Sampling timeframe (MM-DD-YY)	07-30-19 to 08-29-19
Sensor data source	Onboard MicroSD card
Reference data source	AQS API download

Sensor Information			
Manufacturer, model	SENSIT RAMP		
Device firmware version	200203_AQ_v9.36_ALT		
Sampling time interval	15-seconds		
Sensor serial numbers	RAM_01		
Issues encountered during deployment?	☑	See DE-RAM-Page 6 of this testing report	

FRM/FEM Information	
Manufacturer, model, designation	Teledyne Advanced Pollution Instrumentation T640 FEM
Sampling time interval	1-hour averaging
Date of calibration	As required by 40 CFR Part 58 and the DE Ambient Air Quality Monitoring Program QAPP maintained by DNREC
Date of flowrate verification check	Monthly as required by 40 CFR Part 58 Appendix A
Description, date(s) of maintenance activities	N/A

Time Series Plots

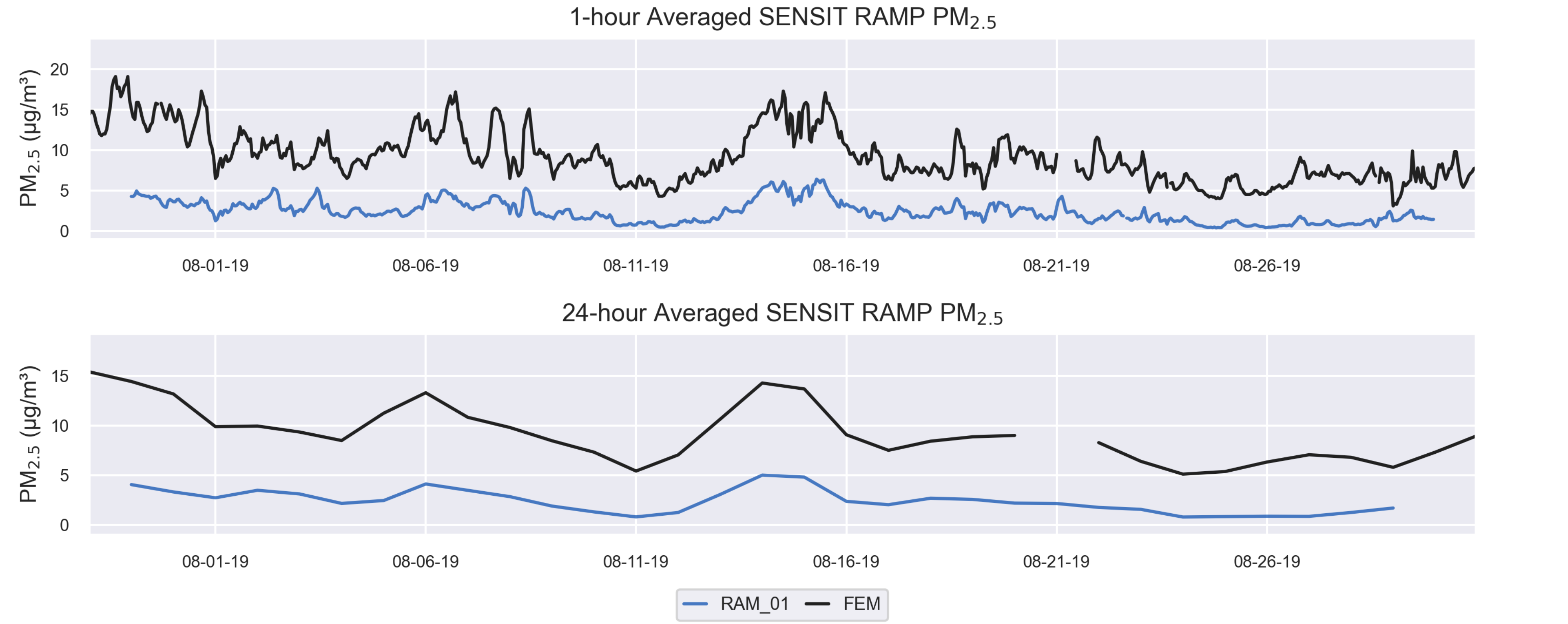

Scatter Plots: Comparison to FRM/FEM

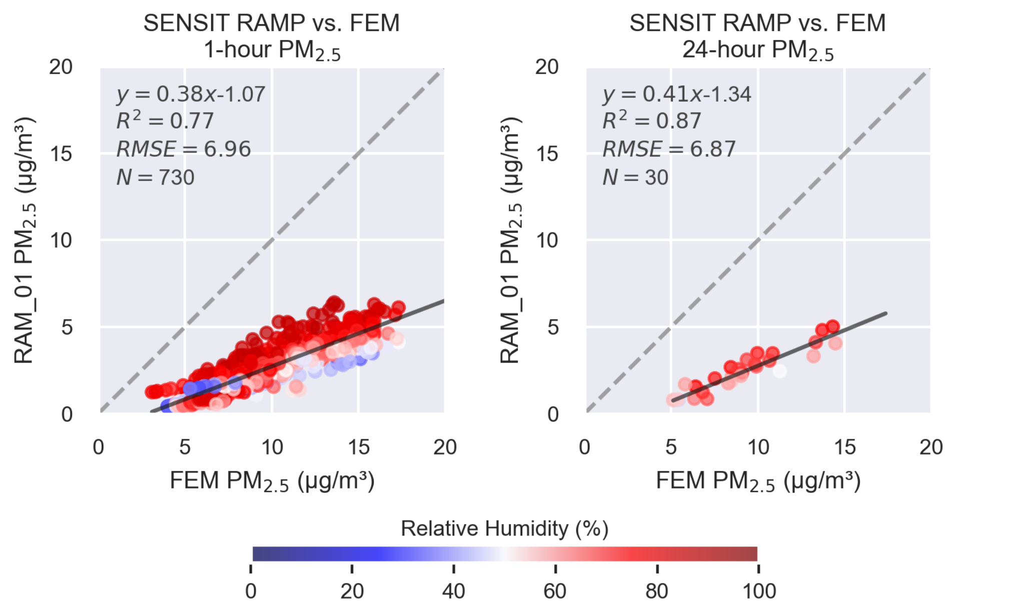

Range of FRM/FEM concentrations over duration of base test (µg/m³)	3.1-17.3 (1-hr), 5.1-14.5 (24-hr)
Number of 24-hr periods in FRM/FEM monitor measurements with a goal concentration ≥ 25 µg/m³	0

Performance Metrics

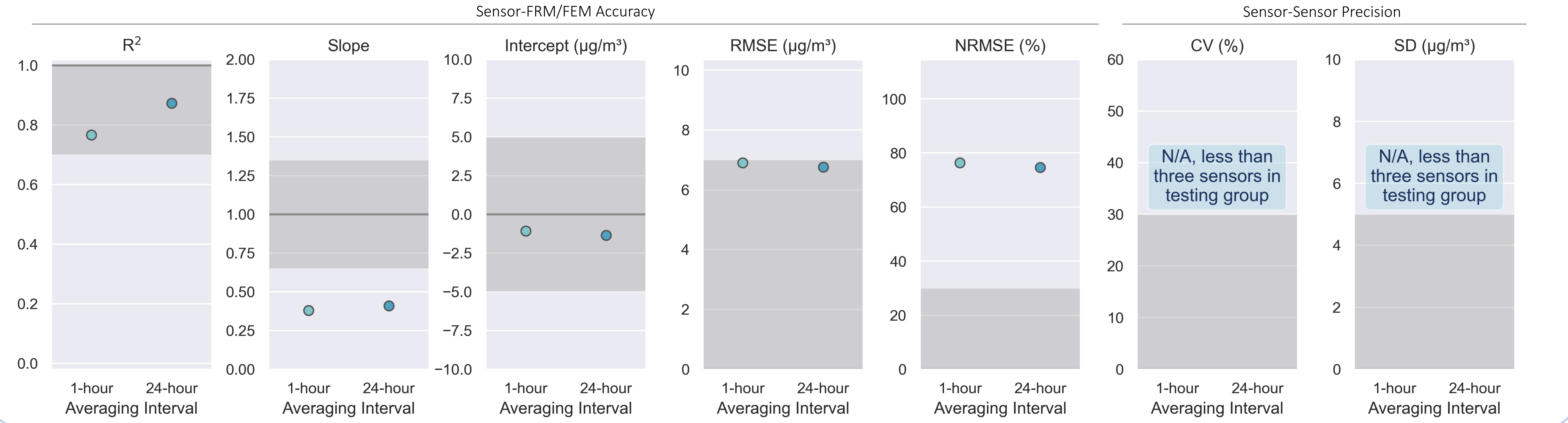

Meteorological Conditions During Deployment

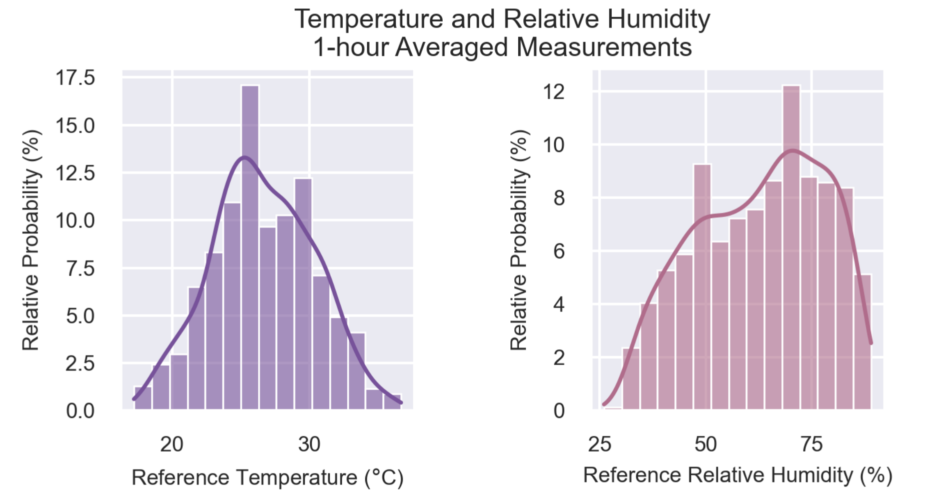

Number of 24-hr periods outside sensor manufacturer-listed temperature operational range (-20 to 50 °C)	0
Number of 24-hr periods outside sensor manufacturer-listed relative humidity operational range (no operational range specified)	-

Meteorological Influence

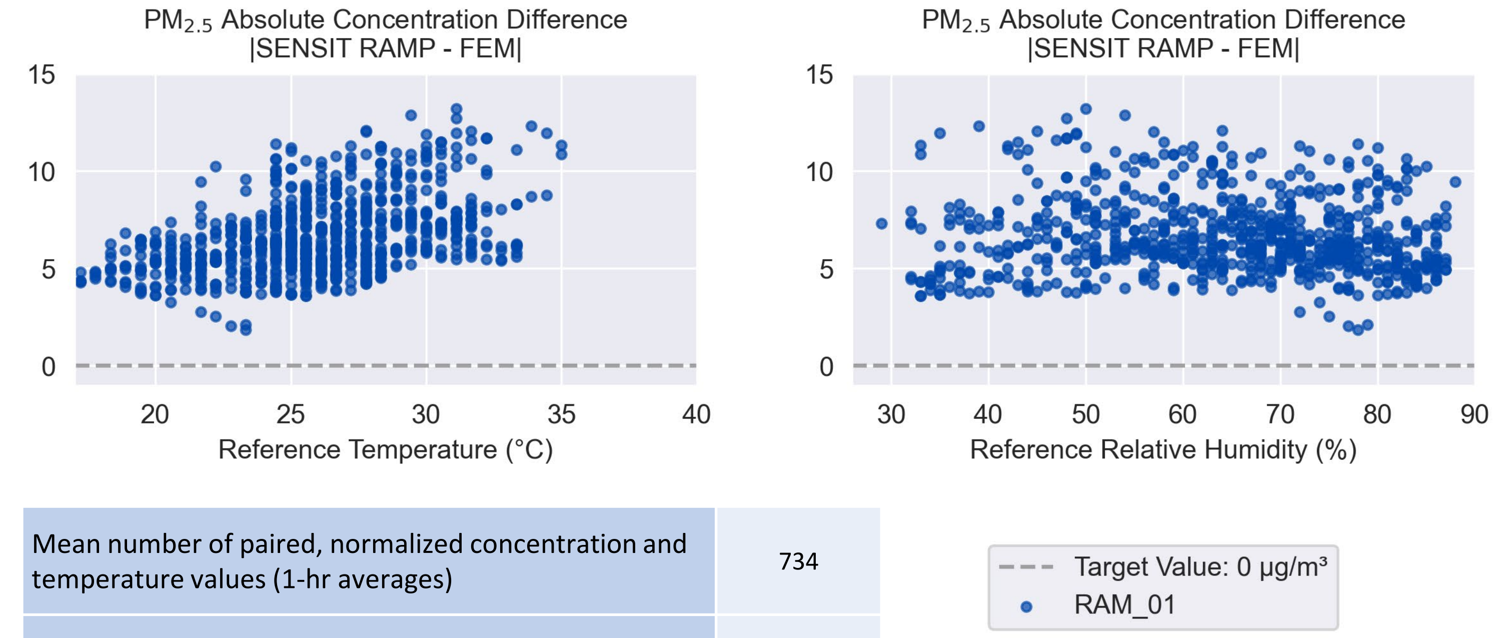

Mean number of paired, normalized concentration and temperature values (1-hr averages)	734
Mean number of paired, normalized concentration and relative humidity values (1-hr averages)	734

Testing Report - $PM_{2.5}$ Base Testing

SENSIT RAMP

This report reflects out-of-the-box performance

Initial Base Testing - Wilmington, DE
U.S. Environmental Protection Agency
Office of Research and Development
PI: Clements.Andrea@epa.gov
919-541-1363
July 2019—August 2019

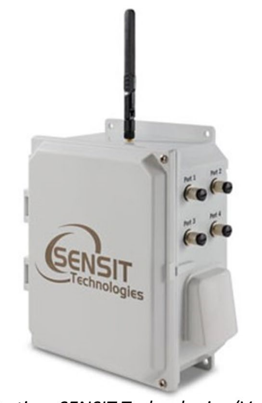

Image Attribution: SENSIT Technologies (Valparaiso, IN)

Tabular Statistics

Sensor-FRM/FEM Correlation

	Bias and Linearity						Data Quality			
	R^2		Slope		Intercept (µg/m³)		Uptime (%)		Number of paired sensor and FRM/FEM concentration values	
	1-Hour ●	24-Hour ●	1-Hour ○	24-Hour ○	1-Hour ●	24-Hour ●	1-Hour ●	24-Hour ●	1-Hour	24-Hour
Metric Target Range	≥ 0.70	≥ 0.70	1.0 ± 0.35	1.0 ± 0.35	-5 ≤ b ≤ 5	-5 ≤ b ≤ 5	75%*	75%*	-	-
Sensor RAM_01	0.77	0.87	0.38	0.41	-1.07	-1.34	100	100	730	30

	Error			
	RMSE (µg/m³)		NRMSE (%)	
	1-Hour ★	24-Hour ★	1-Hour ☆	24-Hour ☆
Metric Target Range	≤ 7.0	≤ 7.0	≤ 30.0	≤ 30.0
Deployment Value	6.9	6.8	76.4	74.7

Device-specific metrics (computed for each sensor in evaluation)
○○○ Metric value for none of devices tested falls within the target range
●○○ Metric value for one of devices tested falls within the target range
●●○ Metric value for two of devices tested falls within the target range
●●● Metric value for three of devices tested falls within the target range

Single-valued metrics (computed via entire evaluation dataset)
☆ Indicates that the metric value is not within the target range
★ Indicates that the metric value is within the target range

Sensor-Sensor Precision[1]

	Precision (between collocated sensors)				Data Quality	
	CV (%)		SD (µg/m³)		Number of concurrent sensor concentration pairs	
	1-Hour ☆	24-Hour ☆	1-Hour ☆	24-Hour ☆	1-Hour	24-Hour
Metric Target Range	≤ 30.0	≤ 30.0	≤ 5.0	≤ 5.0	-	-
Deployment Value	-	-	-	-	-	-

[1]Precision statistics are computed for evaluations with at least three collocated sensor units. Metric values are left blank for evaluations with two or fewer sensor units.

*This value is only a recommendation for ensuring data quality and is not included in the list of target values discussed in Section 4 of the Performance Testing Protocols, Metrics, and Target Values for Fine Particulate Matter Air Sensors document.

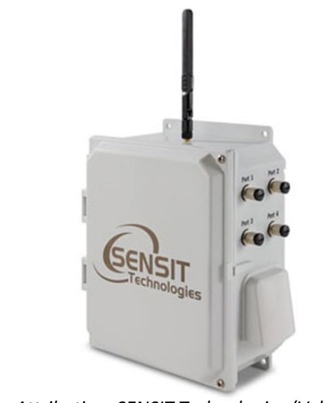

Image Attribution: SENSIT Technologies (Valparaiso, IN)

Sensor-FRM/FEM Scatter Plots

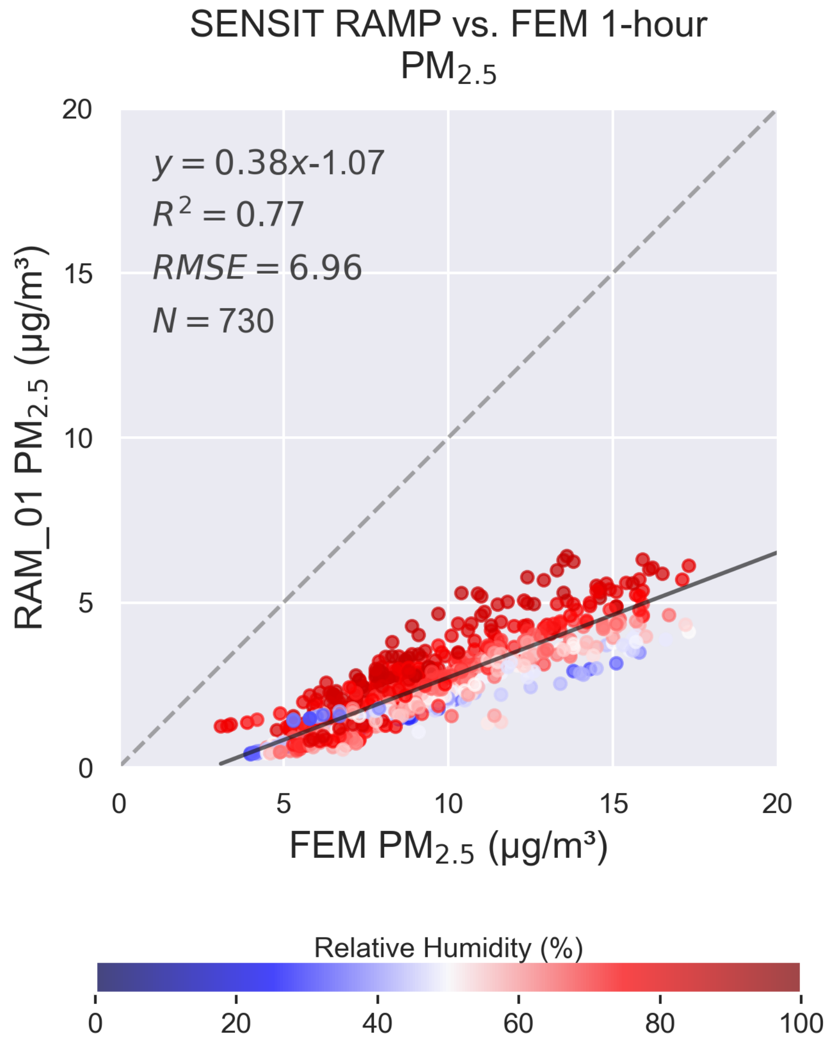

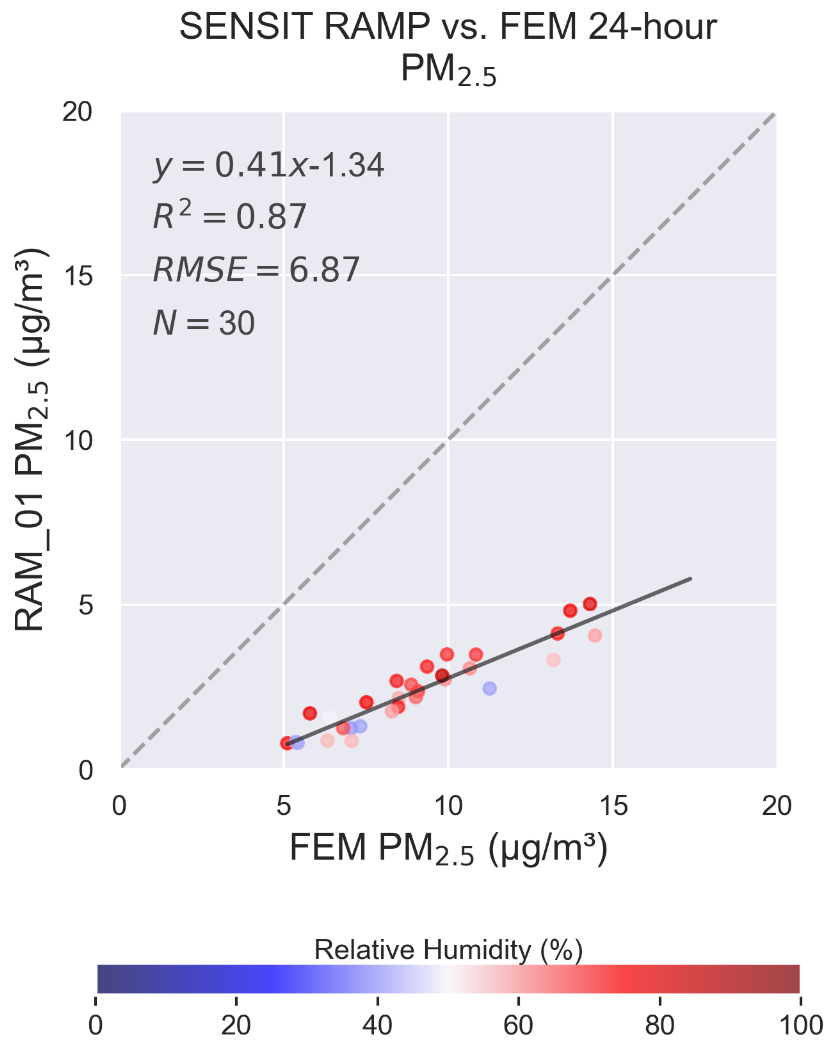

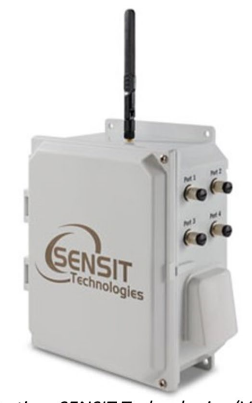
Image Attribution: SENSIT Technologies (Valparaiso, IN)

Supplemental Information

Abbreviations used in Supplemental Information

FRM	Federal Reference Method
FEM	Federal Equivalent Method
SOP	Standard Operating Procedure
QAPP	Quality Assurance Project Plan
QC	Quality Control

Supplemental Documentation	Attached	Description & URL or file path to documentation
Field observations and sensor data flags	☑	See DE-RAM-Page 6 of this testing report
Maintenance logs	☐	No logs recorded during testing
Standard operating procedure(s)	☐	U.S. EPA Office Of Research and Development SOP available upon request
Photos of equipment setup and testing	☑	See DE-RAM-Page 5 of this testing report
Product specifications sheet(s)	☑	SENSIT Technologies: RAMP Product Brochure
Product manual(s)	☑	SENSIT Technologies: RAMP Product Manual
Data storage and transmission method	☑	See DE-RAM-Page 6 of this testing report
Data correction approach	☑	See DE-RAM-Page 6 of this testing report
Issues encountered	☑	See DE-RAM-Page 6 of this testing report
Data analysis/correction scripts and version	☑	Averaging and processing of data, calculation of performance metrics, and generation of figures and other supplementary material for analysis were obtained using Python 3.9.7 with the packages sensortoolkit v0.8.3b2, pandas 1.3.5, NumPy 1.21.2, Matplotlib 3.5.0, statsmodels 0.13.0, and seaborn 0.11.2. All packages are available from the Python Package Index (PyPI) at https://pypi.org. The integrated development environment (IDE) Spyder 5.1.5 was used for scripting and data visualization. Version control for the Python base, packages, and IDE were all managed by conda 4.11.0.
Air Monitoring Station QAPP	☐	U.S. EPA Office Of Research and Development QAPP available upon request
Summary of FRM/FEM monitor QC checks	☑	See DE-RAM-Page 7 of this testing report
Manufacturer website for FRM/FEM monitor	☑	Teledyne API: T640 Product website
FRM/FEM monitor manual	☑	Teledyne API: T640 Product Manual
FRM/FEM monitor specifications sheet(s)	☑	Teledyne API: T640 Specification Sheet
Other documents	☐	

Site Description:

The DE site is located in Wilmington, Delaware at the intersection of Justison St. and MLK Blvd and has been operated at this site since 1999. This site is the state NCore site and represents urban population exposure to multiple pollution sources. This site meets all EPA *40 CFR Part 58 App D and E* siting criteria.

Figure 1: SENSIT RAMP sensor (indicated by red arrow) attached to metal railing atop the sampling shelter at the monitoring site.

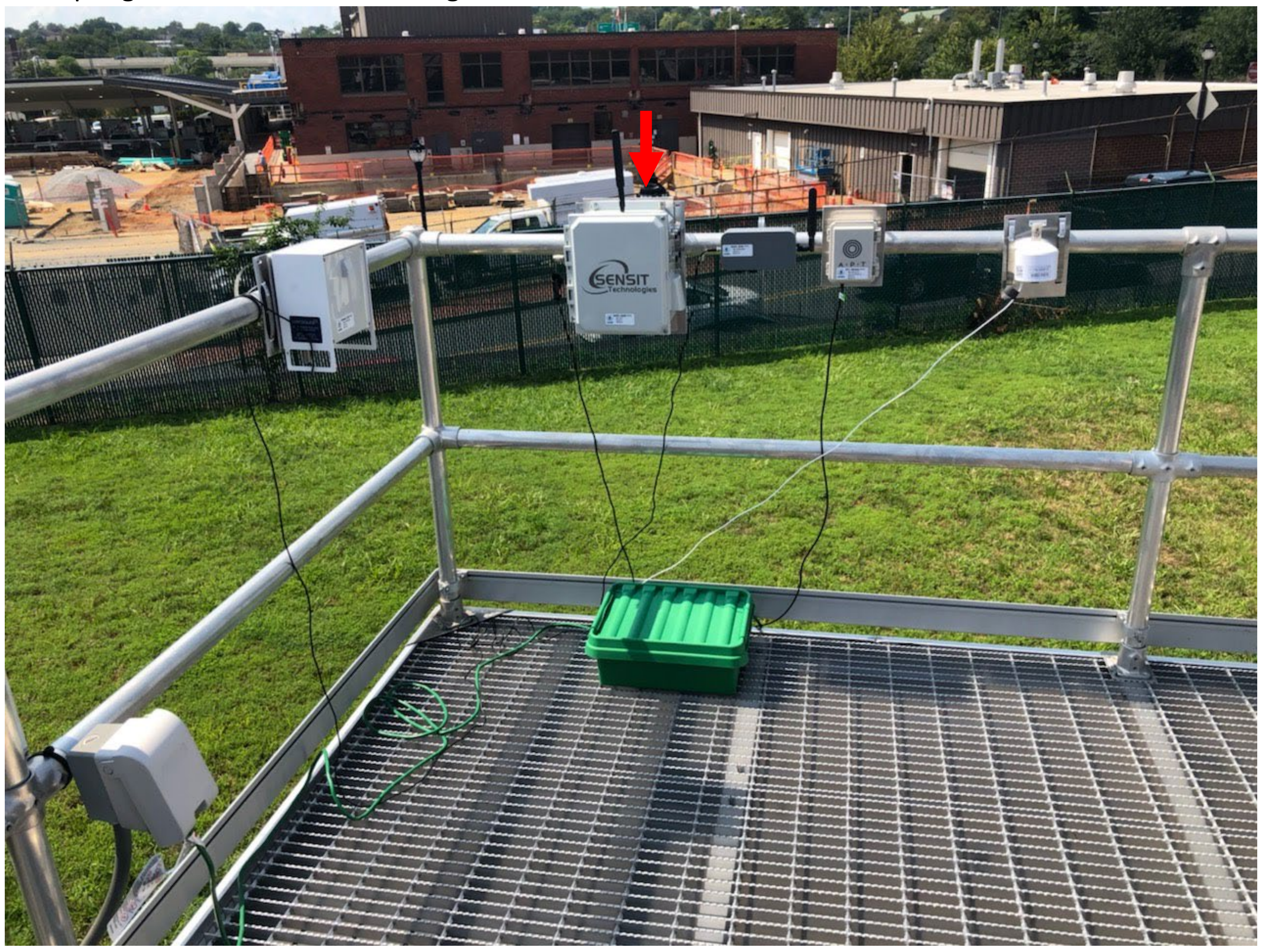

Figure 2: MLK Monitoring Station

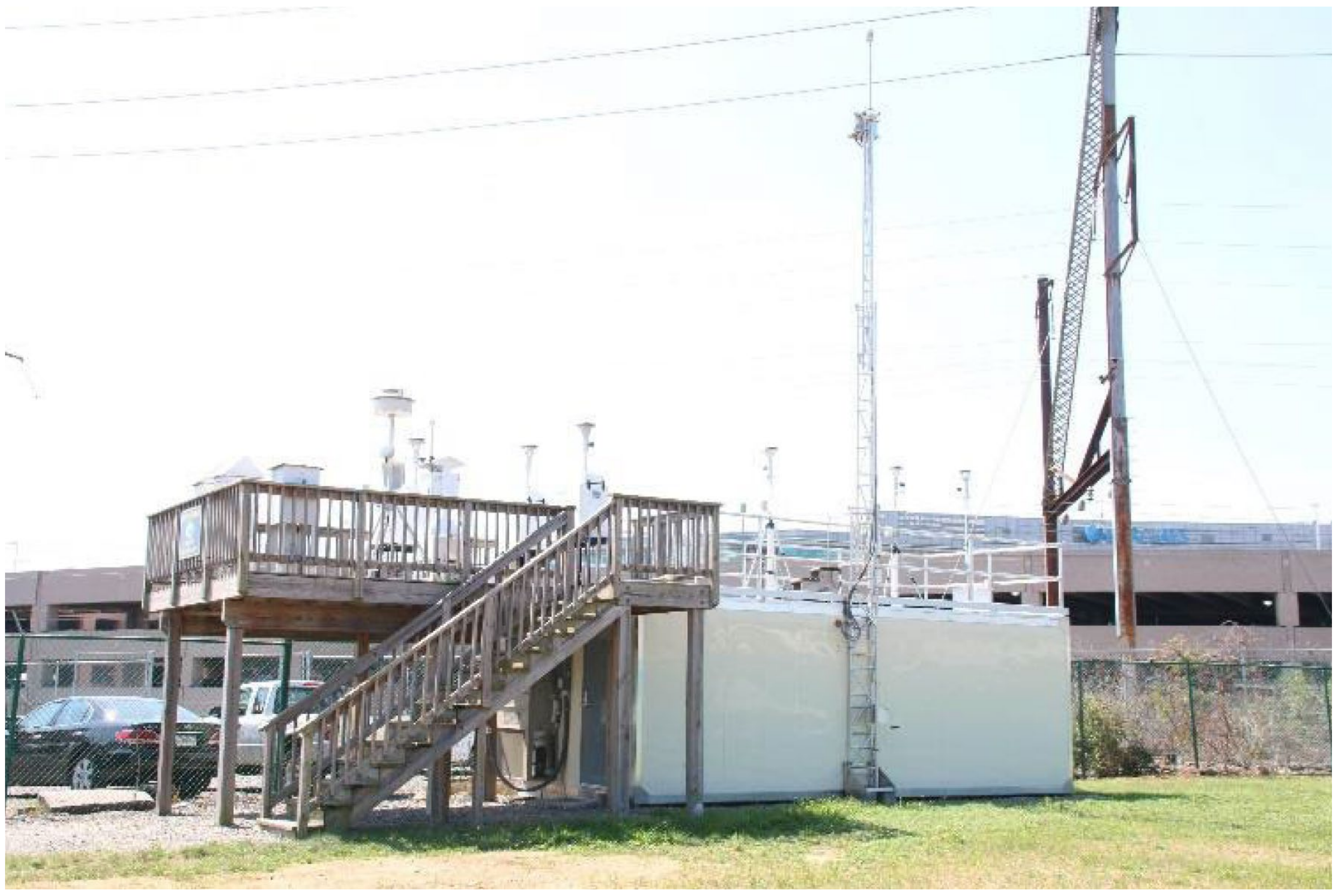

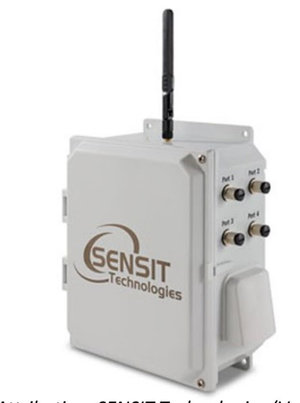

Data Storage and Transmission Method

The SENSIT RAMP was configured to record data at a 15-second sampling interval. Data are stored as daily text files (.txt format) on an onboard MicroSD card. Data files were obtained weekly via SD cards. Each field site operator was provided two labeled MicroSD cards for sensor units that they used to swap out each week. Data from the collected card was then read and processed off-site.

Data Correction Approach

SENSIT RAMP units were pre-configured by the manufacturer with a linear correction (i.e., concentration gain = 1.0 and offset = 0.0 µg m^{-3}). These presets reflect out-of-the box performance and were not modified by EPA prior or during testing.

After acquisition, the raw data was processed using the *sensortoolkit* python code library (v0.8.3b2). A continuous data set at the recorded sampling frequency was written to a .csv file. 1-hour and 24-hour averaged data sets were generated using a 75% completeness threshold and saved as separate .csv files. Outliers were NOT removed from data sets in order to assess "out-of-the-box" sensor performance.

Issues Encountered

<u>Pre-deployment observations</u>

- SENSIT RAMP units were received without documentation or manuals. After communicating the need to change default settings (logging interval and time zone) with the manufacturer, a draft user's manual and a USB cable were supplied. With the use of this USB cable, instrument settings could be changed, and real-time data could be logged using a serial communication software (CoolTerm, v.1.5.0). Because the sensor did not record data at the top of every minute, the RAMP was configured to record data at 15-second intervals so that the data could be averaged more closely to complete minutes. Prior to deployment, RAMP units were collocated in a bench-top evaluation to verify operational status and determine the extent of data invalidity (i.e., determine equilibration period) after an initial start-up event. The recorded response for parameters measured by the RAMP suggests that the gas sensors (CO, NO, NO_2, O_3) required approximately a 2-hour equilibration period, while the remaining sensors (temperature, relative humidity, particulate matter) did not require any equilibration period.

<u>Field observations and sensor data flags</u>

- The RAM_01 sensor unit recorded faulty CO2, temperature, and relative humidity measurements during the testing period, indicating flatline zeros for each parameter. This failure mode was due to a COZIR sensor failure, and this behavior was reported throughput the full duration of the July-August 2019 testing period, and the unit remained deployed until 3/11/2020, when the sensor unit was removed and a replacement unit was installed.

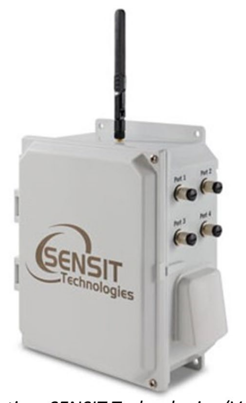

Image Attribution: SENSIT Technologies (Valparaiso, IN)

Description of Data Flags

AQS

The U.S. EPA's Air Quality System (AQS) is the Agency's primary ambient air monitoring data archive. A comprehensive list of data flags that are recorded alongside AQS data sets, referred to by U.S. EPA as 'qualifiers', can be found at the following link: https://aqs.epa.gov/aqsweb/documents/codetables/qualifiers.html

Invalidation of reference data: AQS qualifiers are organized by qualifier type, which indicates whether data logged alongside qualifier flags should be invalidated (set null). Qualifiers with type "Null Data Qualifier" are invalidated, and includes data logged during periods that coincide with QC checks (e.g., "BF-Precision/Zero/Span", "BJ- Operator Error", "BL - QA Audit", "AZ - QC Audit") among other events such as power outages. Data logged alongside qualifiers with type "Quality Assurance Qualifiers" are not invalidated and are included in this analysis (e.g., concentrations less than the federal MDL for the reference monitor "MD – Value less than MDL", QA reviewed values "Validated Value").

Data Flags Recorded During Testing

FRM/FEM Monitor	Timestamp (UTC)	Flag
Teledyne API T640 (Acquired via AQS)	2019-07-30 16:00:00+0000	AI - Insufficient Data (cannot calculate)
	2019-08-21 01:00:00+0000 to 2019-08-21 09:00:00+0000	AM - Miscellaneous Void
	2019-08-21 10:00:00+0000	AI - Insufficient Data (cannot calculate)
	2019-08-23 16:00:00+0000	AI - Insufficient Data (cannot calculate)
	2019-08-28 15:00:00+0000	AI - Insufficient Data (cannot calculate)

Meteorological Instrument	Timestamp (UTC)	Flag
Vaisala WXT-520 Temperature Monitor (Acquired via AQS)	2019-08-21 01:00:00+0000 to 2019-08-21 09:00:00+0000	AM - Miscellaneous Void
	2019-08-21 10:00:00+0000	AI - Insufficient Data (cannot calculate)
Vaisala WXT-520 Relative Humidity Monitor (Acquired via AQS)	2019-08-21 01:00:00+0000 to 2019-08-21 09:00:00+0000	AM - Miscellaneous Void
	2019-08-21 10:00:00+0000	AI - Insufficient Data (cannot calculate)

Testing Report - PM$_{2.5}$ Base Testing

QuantAQ ARISense

This report reflects out-of-the-box performance

Initial Base Testing - Wilmington, DE

U.S. Environmental Protection Agency
Office of Research and Development
PI: Clements.Andrea@epa.gov
919-541-1363

March 2020—April 2020

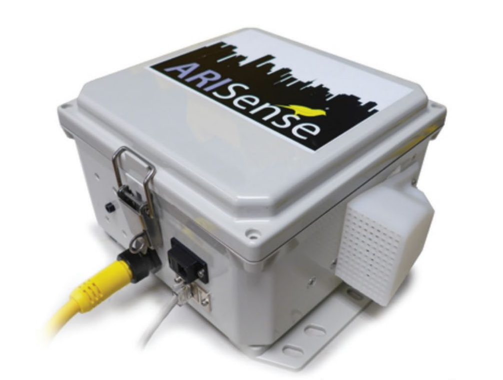

Image Attribution: QuantAQ, Inc (Somerville, MA)

Deployment Details

Testing Organization and Site Information	
Testing organization (Name, Organization type, Contact website)	U.S. Environmental Protection Agency - Office of Research and Development<br>Federal Government<br>Air Sensor Toolbox | U.S. EPA Website
Testing location (City, State, Latitude and Longitude)	MLK - Corner Of MLK Blvd And Justison St<br>Wilmington, DE<br>39.7395, -75.5575
AQS site ID	10 - 003 - 2004
Sampling timeframe (MM-DD-YY)	03-18-20 to 04-17-20
Sensor data source	QuantAQ Cloud download
Reference data source	AQS API download

Sensor Information			
Manufacturer, model	QuantAQ ARISense		
Device firmware version	Received by EPA on 4/30/2019		
Sampling time interval	1-minute		
Sensor serial numbers	ARS_01		
Issues encountered during deployment?	☑	See DE-ARS-Page 6 of this testing report	

FRM/FEM Information	
Manufacturer, model, designation	Teledyne Advanced Pollution Instrumentation T640 FEM
Sampling time interval	1-hour averaging
Date of calibration	As required by 40 CFR Part 58 and the DE Ambient Air Quality Monitoring Program QAPP maintained by DNREC
Date of flowrate verification check	Monthly as required by 40 CFR Part 58 Appendix A
Description, date(s) of maintenance activities	N/A

Time Series Plots

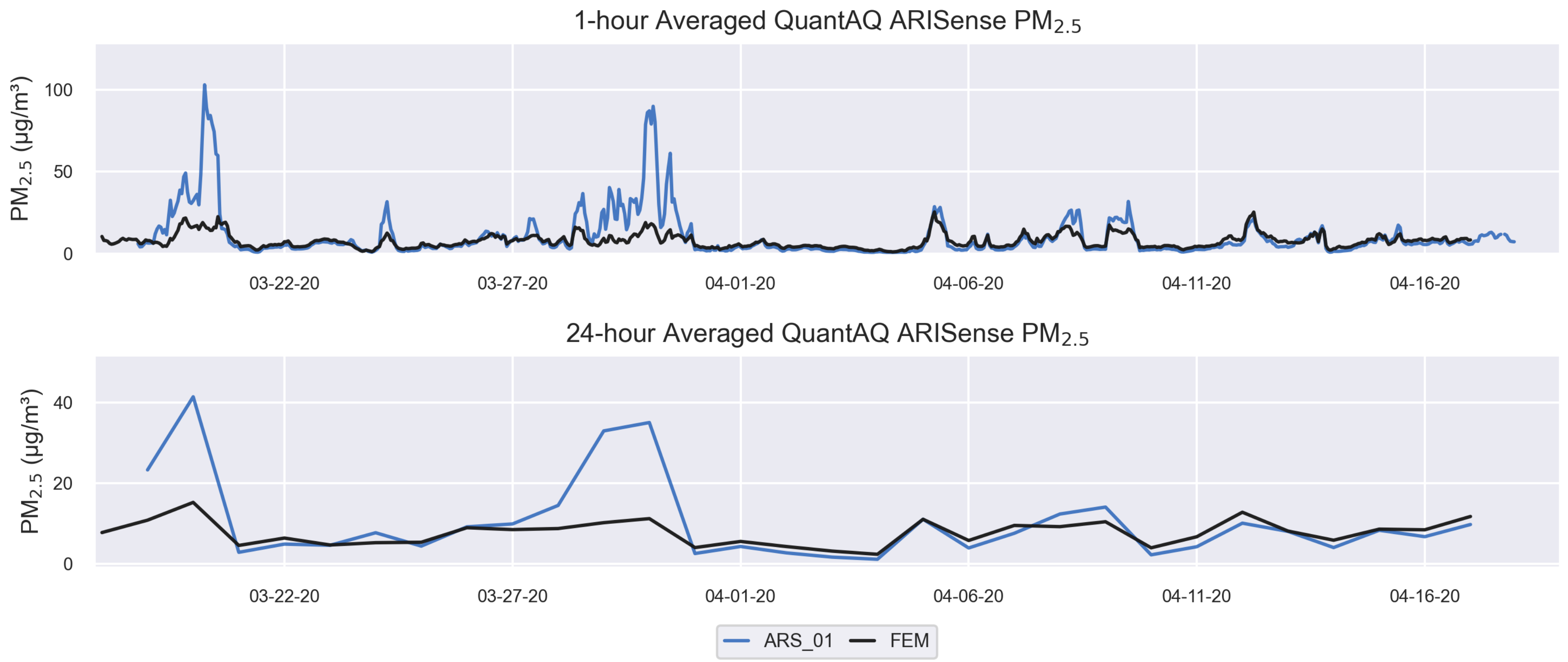

Scatter Plots: Comparison to FRM/FEM

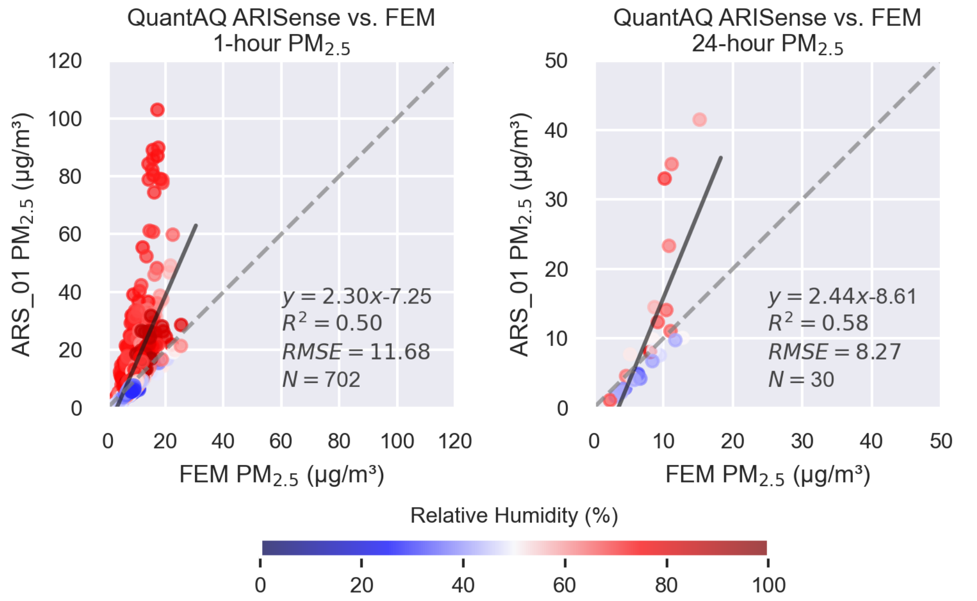

Range of FRM/FEM concentrations over duration of base test (µg/m³)	1.0-25.4 (1-hr),<br>2.3-15.2 (24-hr)
Number of 24-hr periods in FRM/FEM monitor measurements with a goal concentration ≥ 25 µg/m³	0

Performance Metrics

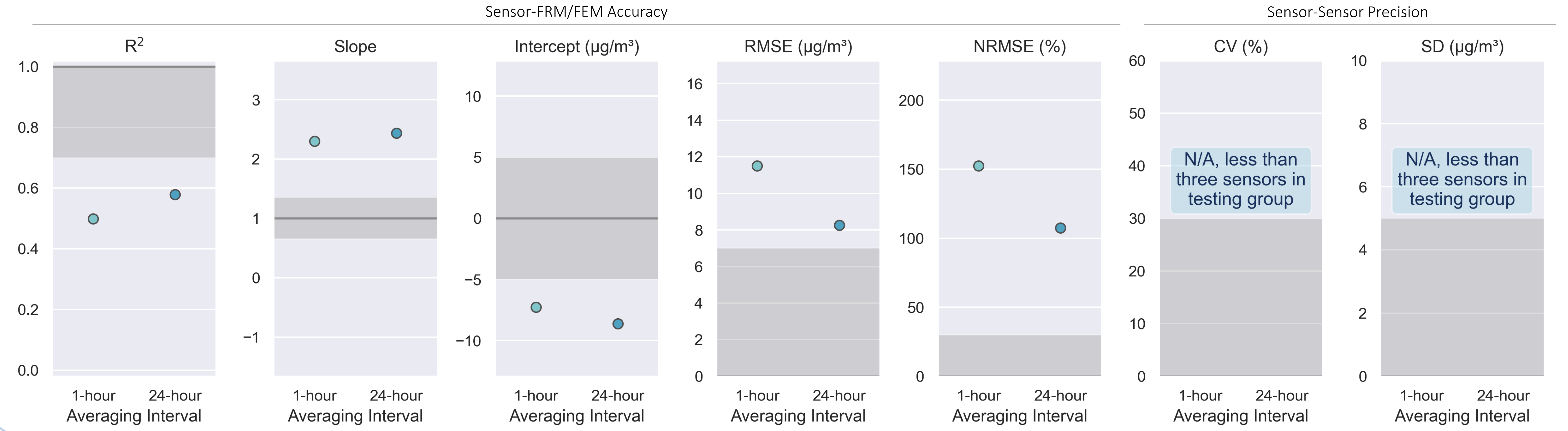

Meteorological Conditions During Deployment

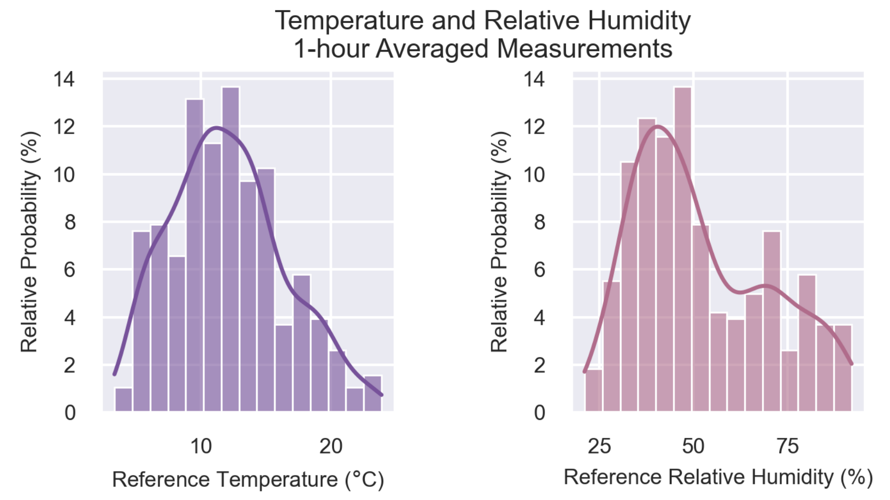

Number of 24-hr periods outside sensor manufacturer-listed temperature operational range (no operational range specified)	-
Number of 24-hr periods outside sensor manufacturer-listed relative humidity operational range (no operational range specified)	-

Meteorological Influence

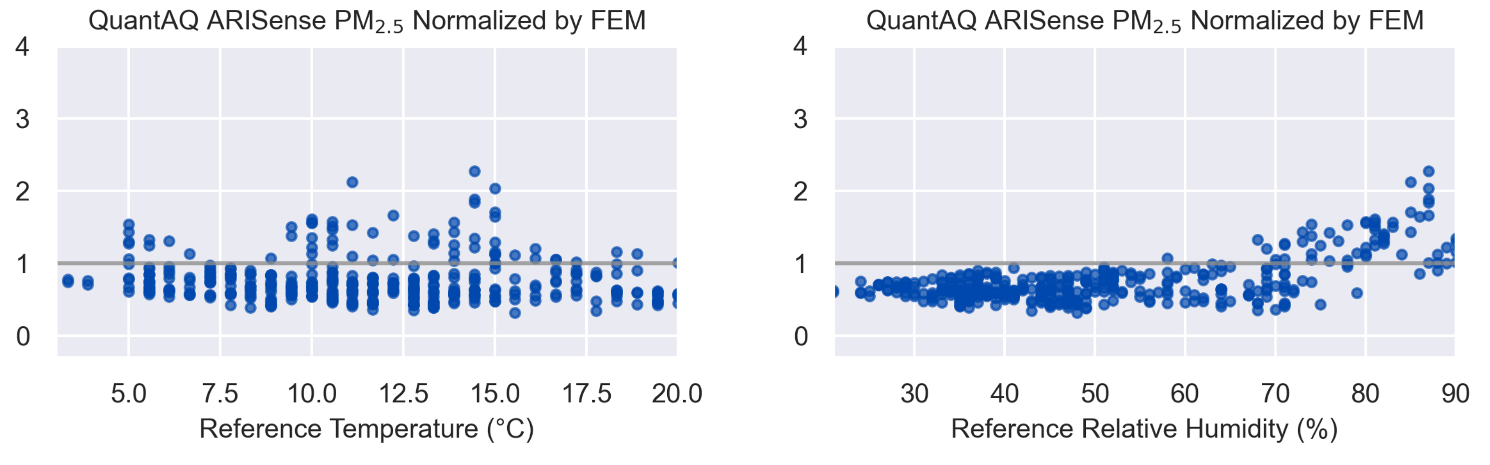

Mean number of paired, normalized concentration and temperature values (1-hr averages)	403
Mean number of paired, normalized concentration and relative humidity values (1-hr averages)	403

Testing Report - $PM_{2.5}$ Base Testing

QuantAQ ARISense

This report reflects out-of-the-box performance

Initial Base Testing - Wilmington, DE

U.S. Environmental Protection Agency
Office of Research and Development
PI: Clements.Andrea@epa.gov
919-541-1363

March 2020—April 2020

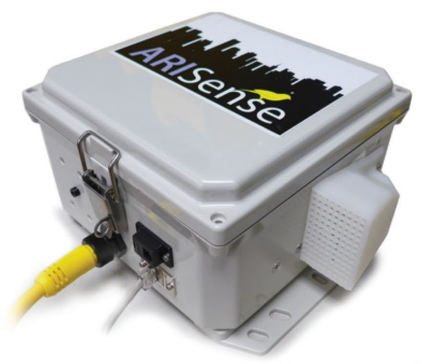

Image Attribution: QuantAQ, Inc (Somerville, MA)

Tabular Statistics

Sensor-FRM/FEM Correlation

	Bias and Linearity						Data Quality			
	R^2		Slope		Intercept (µg/m³)		Uptime (%)		Number of paired sensor and FRM/FEM concentration values	
	1-Hour ○	24-Hour ○	1-Hour ○	24-Hour ○	1-Hour ○	24-Hour ○	1-Hour ●	24-Hour ●	1-Hour	24-Hour
Metric Target Range	≥ 0.70	≥ 0.70	1.0 ± 0.35	1.0 ± 0.35	-5 ≤ b ≤ 5	-5 ≤ b ≤ 5	75%*	75%*	-	-
Sensor ARS_01	0.50	0.58	2.30	2.44	-7.25	-8.61	100	100	702	30

	Error			
	RMSE (µg/m³)		NRMSE (%)	
	1-Hour ☆	24-Hour ☆	1-Hour ☆	24-Hour ☆
Metric Target Range	≤ 7.0	≤ 7.0	≤ 30.0	≤ 30.0
Deployment Value	11.5	8.3	152.4	107.5

Device-specific metrics (computed for each sensor in evaluation)
○○○ Metric value for none of devices tested falls within the target range
●○○ Metric value for one of devices tested falls within the target range
●●○ Metric value for two of devices tested falls within the target range
●●● Metric value for three of devices tested falls within the target range

Single-valued metrics (computed via entire evaluation dataset)
☆ Indicates that the metric value is not within the target range
★ Indicates that the metric value is within the target range

Sensor-Sensor Precision[1]

	Precision (between collocated sensors)				Data Quality	
	CV (%)		SD (µg/m³)		Number of concurrent sensor concentration pairs	
	1-Hour ☆	24-Hour ☆	1-Hour ☆	24-Hour ☆	1-Hour	24-Hour
Metric Target Range	≤ 30.0	≤ 30.0	≤ 5.0	≤ 5.0	-	-
Deployment Value	-	-	-	-	-	-

[1]Precision statistics are computed for evaluations with at least three collocated sensor units. Metric values are left blank for evaluations with two or fewer sensor units.

*This value is only a recommendation for ensuring data quality and is not included in the list of target values discussed in Section 4 of the Performance Testing Protocols, Metrics, and Target Values for Fine Particulate Matter Air Sensors document.

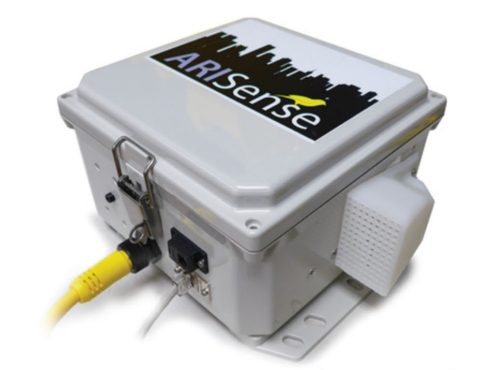

Image Attribution: QuantAQ, Inc (Somerville, MA)

Sensor-FRM/FEM Scatter Plots

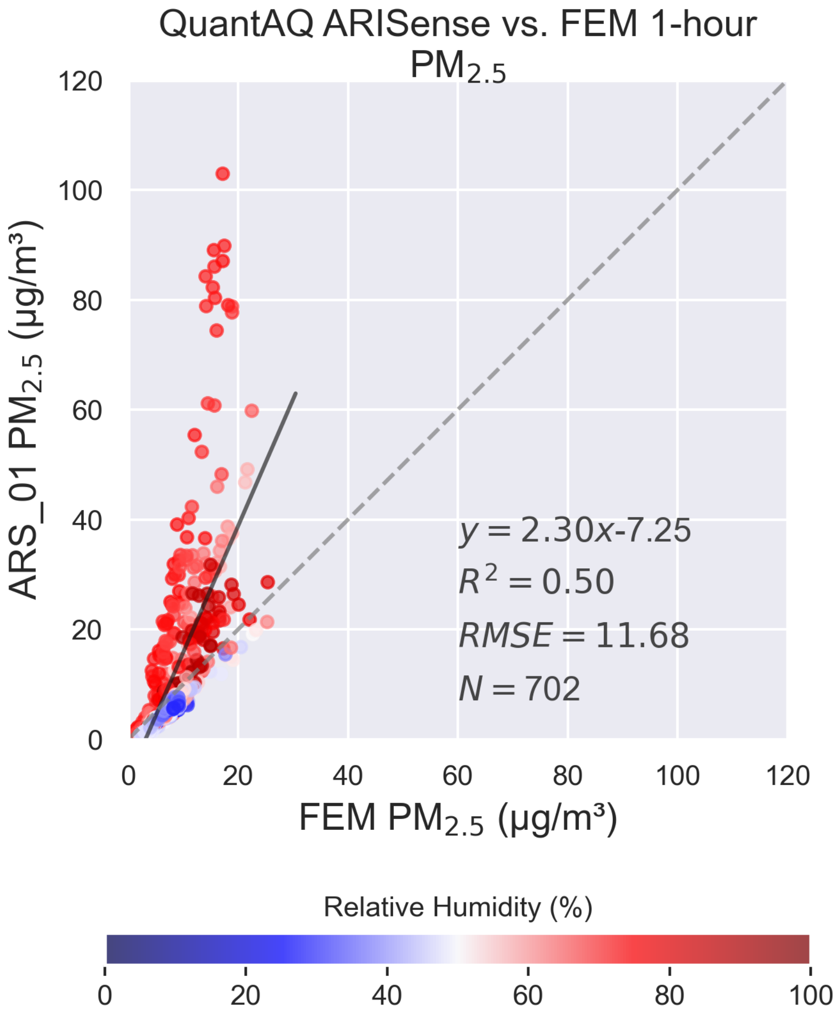

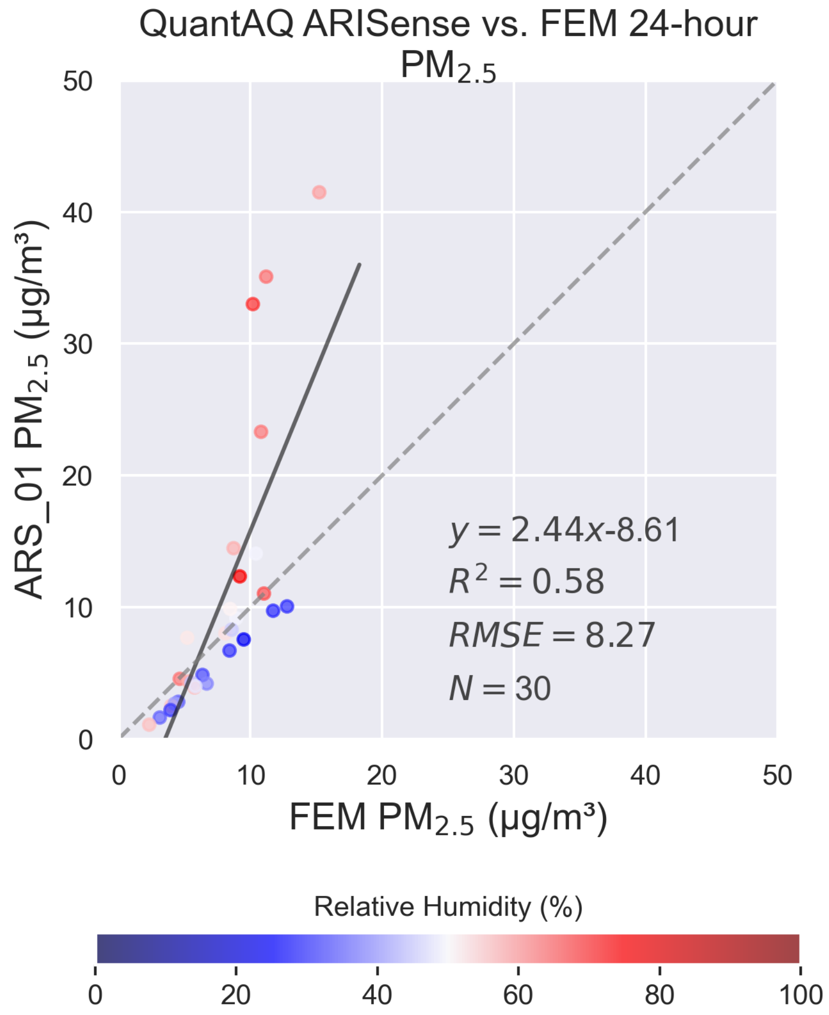

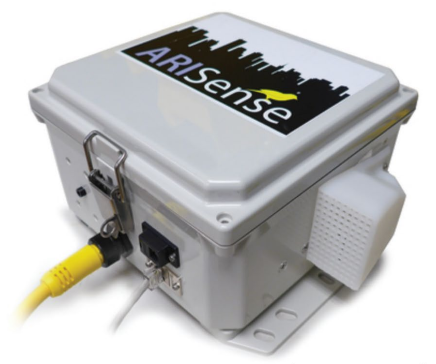

Image Attribution: QuantAQ, Inc (Somerville, MA)

Supplemental Information

Abbreviations used in Supplemental Information

FRM	Federal Reference Method
FEM	Federal Equivalent Method
SOP	Standard Operating Procedure
QAPP	Quality Assurance Project Plan
QC	Quality Control

Supplemental Documentation	Attached	Description & URL or file path to documentation
Field observations and sensor data flags	☑	See DE-ARS-Page 7 of this testing report
Maintenance logs	☐	No logs recorded during testing
Standard operating procedure(s)	☐	U.S. EPA Office Of Research and Development SOP available upon request
Photos of equipment setup and testing	☑	See DE-ARS-Page 5 of this testing report
Product specifications sheet(s)	☑	QuantAQ and Particles Plus: ARISense Specification Sheet
Product manual(s)	☐	N/A
Data storage and transmission method	☑	See DE-ARS-Page 6 of this testing report
Data correction approach	☑	See DE-ARS-Page 6 of this testing report
Issues encountered	☑	See DE-ARS-Page 6 of this testing report
Data analysis/correction scripts and version	☑	Averaging and processing of data, calculation of performance metrics, and generation of figures and other supplementary material for analysis were obtained using Python 3.9.7 with the packages sensortoolkit v0.8.3b2, pandas 1.3.5, NumPy 1.21.2, Matplotlib 3.5.0, statsmodels 0.13.0, and seaborn 0.11.2. All packages are available from the Python Package Index (PyPI) at https://pypi.org. The integrated development environment (IDE) Spyder 5.1.5 was used for scripting and data visualization. Version control for the Python base, packages, and IDE were all managed by conda 4.11.0.
Air Monitoring Station QAPP	☐	U.S. EPA Office Of Research and Development QAPP available upon request
Summary of FRM/FEM monitor QC checks	☑	See DE-ARS-Page 8 of this testing report
Manufacturer website for FRM/FEM monitor	☑	Teledyne API: T640 Product website
FRM/FEM monitor manual	☑	Teledyne API: T640 Product Manual
FRM/FEM monitor specifications sheet(s)	☑	Teledyne API: T640 Specification Sheet
Other documents	☐	

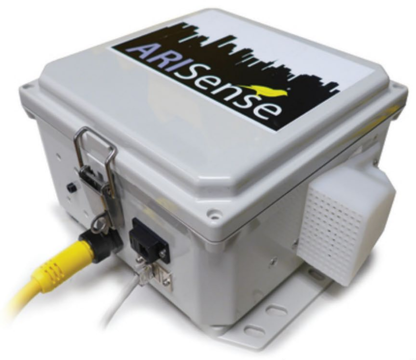

Image Attribution: QuantAQ, Inc (Somerville, MA)

Supplemental Information: Photos of Testing Site and Equipment Setup

Site Description:
The DE site is located in Wilmington, Delaware at the intersection of Justison St. and MLK Blvd and has been operated at this site since 1999. This site is the state NCore site and represents urban population exposure to multiple pollution sources. This site meets all EPA *40 CFR Part 58 App D and E* siting criteria.

Figure 1: MLK Monitoring Station

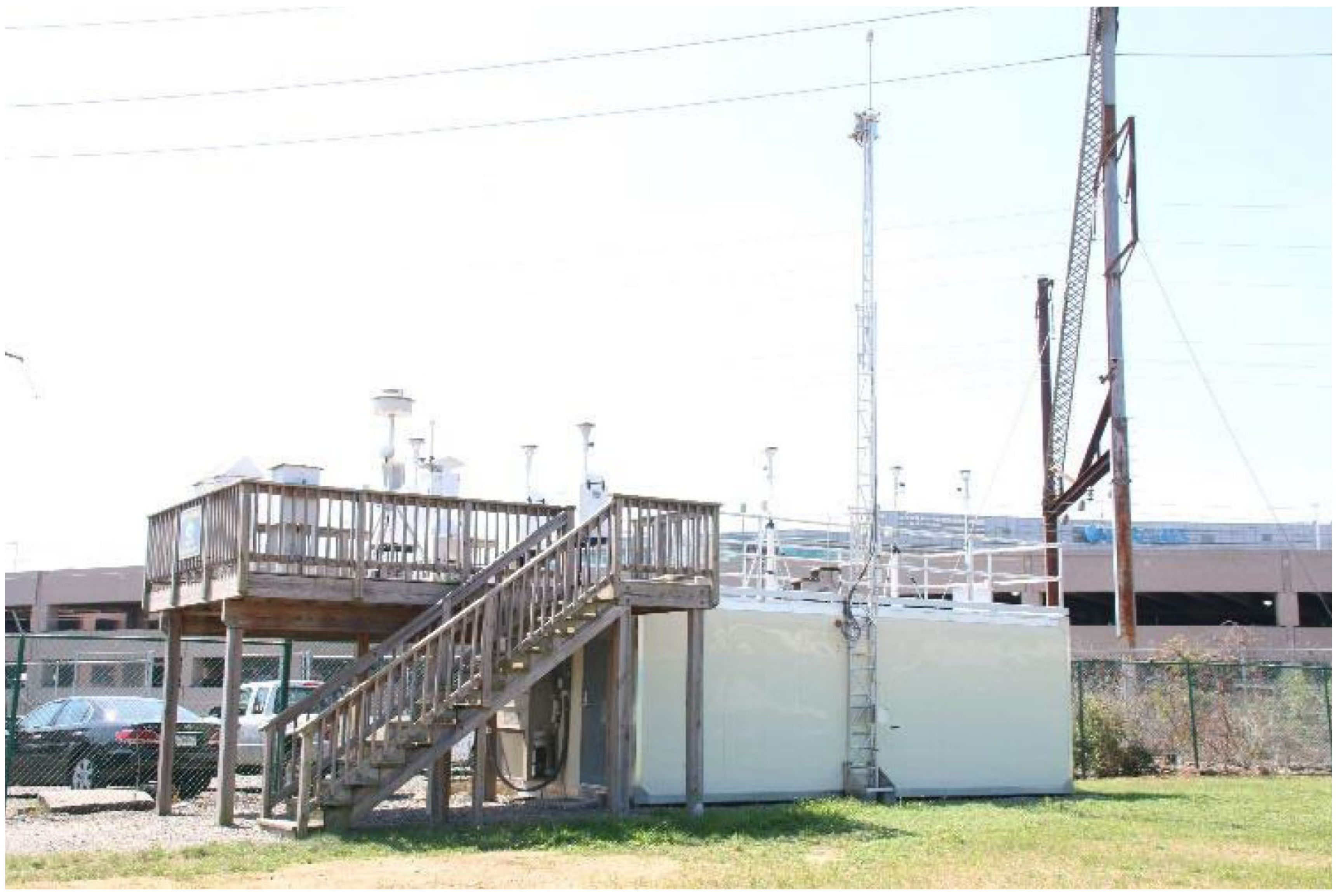

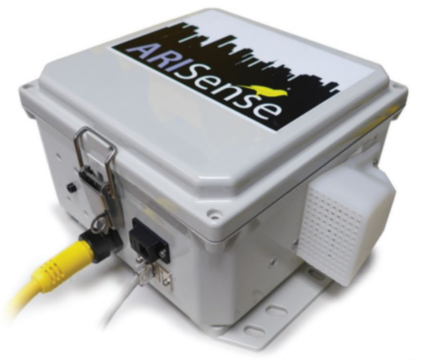

Image Attribution: QuantAQ, Inc (Somerville, MA)

Data Storage and Transmission Method

ARISense units upload data to an external cloud server at 2-minute averages via cellular signal. ARISense weekly data collections were performed by downloading data from the QuantAQ cloud server (last accessed 5/11/22).

Data Correction Approach

QuantAQ ARISense units measure PM2.5 concentrations via an optical particle counter (OPC). The OPC measures particle counts within particle size bins ranging from 0.3 microns to 10 microns, and mass concentrations for each bins are estimated from particle counts assuming constant values for density, spherical morphology, and average particle diameter. The manufacturer applies a general correction to account for the detection limitations of the OPC leading to concentrations that are closer to reality. These presets were configured by the sensor manufacturer and were not modified by EPA prior or during testing.

After acquisition, the raw data was processed using the *sensortoolkit* python code library (v0.8.3b2). A continuous data set at the recorded sampling frequency was written to a .csv file. 1-hour and 24-hour averaged data sets were generated using a 75% completeness threshold and saved as separate .csv files. Outliers were NOT removed from data sets in order to assess "out-of-the-box" sensor performance.

Issues Encountered

Pre-deployment observations

Numerous issues were encountered with the ARISense devices. Although previous models of the ARISense had used AlphaSense OPC-N2 these OPCs were unavailable at the time of manufacture. So instead, components from a particle plus OPC handheld were used.

- *Shipping damage:* On arrival to EPA many of the units were damaged due to the heavy pump breaking free during shipping and shaking around inside of the box breaking much of the plastic scaffolding and, in some cases, components. The manufacturer worked with us to fix these units.
- *Hardware and Firmware issues:* Multiple hardware and firmware issues were experienced that had to be fixed before starting the deployment and multiple manual firmware updates on both the gas sensor microcontrollers and the OPC microcontrollers were completed by researchers. The USB drives were frequently corrupted which required replacement and eventually a hardware update. In addition, the OPC microcontroller and the gas sensor microcontroller often miscommunicated. One firmware version reduced the number of bins reported by the OPC since the manufacturer determined the additional bins were not providing additional accuracy. New PCB boards were sent and installed before the tests could be run as well.
- *Initiation:* There was a high power draw when the cellular connectivity initiated and the pump was turned on. This meant that units occasionally failed to turn on at the same time and it meant these could not be powered by solar.
- *Water intake:* In addition, the OPC pump often drew in water during rainstorms leading the inline filter to saturate and the pump to fail due to overheating. Many pumps had to be replaced throughout the testing.
- *Cellular connectivity:* Some units could not consistently stay connected to the cellular, though other sensors at the same site did not experience issues. The manufacturer could not recreate this issue when they were sent back for service and the problems persisted.
- *Reset:* These units ended up being operated on timers so that they were power cycled every night which would typically get them back online if a software error had been encountered, leading to lower data loss than would have occurred with only weekly checks.

Field observations and sensor data flags are included on the following page

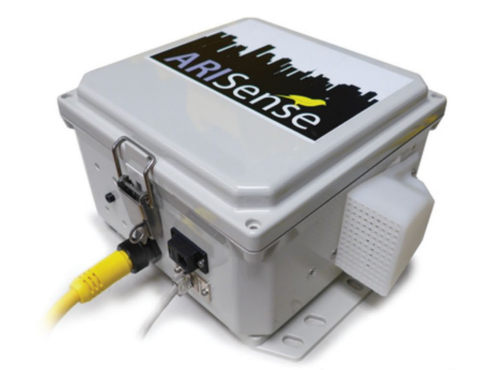

Image Attribution: QuantAQ, Inc (Somerville, MA)

Field observations and sensor data flags

The following table contains data flags describing events that were encountered during the testing period. The QuantAQ ARISense was configured to record data at regular 60-second sampling intervals, however, occasionally the unit recorded irregular sampling intervals ranging from 2-minute intervals to nearly 15 minutes at longest interval duration. The flags below note when these irregular sampling intervals occurred during the testing period. Sampling interval irregularities were not a result of modification to the device firmware configuration and are potentially associated power loading during periods where the ARISense unit initiated a cellular data upload.

Start Time (UTC)	End Time (UTC)	Sensor Serial ID	Parameters Impacted	Flag
2020-04-13 20:56:00+00:00	2020-04-13 21:04:00+00:00	ARS_01	ALL	6.0-Sampling interval abnormality
2020-04-13 21:45:00+00:00	2020-04-13 21:50:00+00:00	ARS_01	ALL	6.0-Sampling interval abnormality
2020-04-17 15:56:00+00:00	2020-04-17 16:16:00+00:00	ARS_01	ALL	6.0-Sampling interval abnormality
2020-04-17 16:51:00+00:00	2020-04-17 16:58:00+00:00	ARS_01	ALL	6.0-Sampling interval abnormality

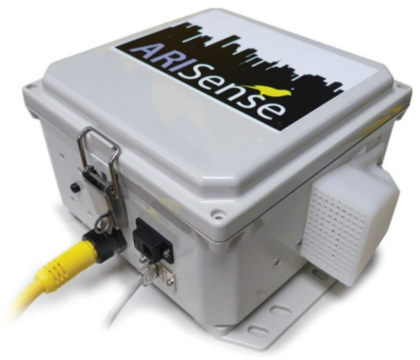

Image Attribution: QuantAQ, Inc (Somerville, MA)

Description of Data Flags

AQS

The U.S. EPA's Air Quality System (AQS) is the Agency's primary ambient air monitoring data archive. A comprehensive list of data flags that are recorded alongside AQS data sets, referred to by U.S. EPA as 'qualifiers', can be found at the following link: https://aqs.epa.gov/aqsweb/documents/codetables/qualifiers.html

Invalidation of reference data: AQS qualifiers are organized by qualifier type, which indicates whether data logged alongside qualifier flags should be invalidated (set null). Qualifiers with type "Null Data Qualifier" are invalidated, and includes data logged during periods that coincide with QC checks (e.g., "BF-Precision/Zero/Span", "BJ- Operator Error", "BL - QA Audit", "AZ - QC Audit") among other events such as power outages. Data logged alongside qualifiers with type "Quality Assurance Qualifiers" are not invalidated and are included in this analysis (e.g., concentrations less than the federal MDL for the reference monitor "MD – Value less than MDL", QA reviewed values "Validated Value").

Data Flags Recorded During Testing

FRM/FEM Monitor	Timestamp (UTC)	Flag
Teledyne API T640 (Acquired via AQS)	No data flags recorded during testing period	

Meteorological Instrument	Timestamp (UTC)	Flag
Vaisala WXT-520 Temperature Monitor (Acquired via AQS)	No data flags recorded during testing period	
Vaisala WXT-520 Relative Humidity Monitor (Acquired via AQS)	No data flags recorded during testing period	

Testing Report - $PM_{2.5}$ Base Testing

Aeroqual AQY

This report reflects out-of-the-box performance

Initial Base Testing - Decatur, GA

U.S. Environmental Protection Agency
Office of Research and Development
PI: Clements.Andrea@epa.gov
919-541-1363
September 2019—October 2019

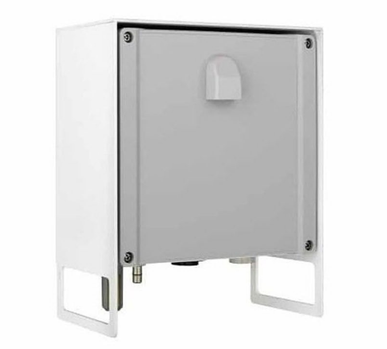

Image Attribution: Aeroqual Limited (Auckland, New Zealand)

Deployment Details

Testing Organization and Site Information	
Testing organization (Name, Organization type, Contact website)	U.S. Environmental Protection Agency - Office of Research and Development<br>Federal Government<br>Air Sensor Toolbox | U.S. EPA Website
Testing location (City, State, Latitude and Longitude)	South Dekalb<br>Decatur, GA<br>33.6877, -84.2905
AQS site ID	13 - 089 - 0002
Sampling timeframe (MM-DD-YY)	09-11-19 to 10-11-19
Sensor data source	Aeroqual Cloud download
Reference data source	AQS API download

Sensor Information			
Manufacturer, model	Aeroqual AQY		
Device firmware version	1.14.2		
Sampling time interval	1-minute		
Sensor serial numbers	AQY_01		
Issues encountered during deployment?	☑	See GA-AQY-Page 6 of this testing report	

FRM/FEM Information	
Manufacturer, model, designation	Teledyne Advanced Pollution Instrumentation T640X FEM
Sampling time interval	1-hour averaging
Date of calibration	As required by 40 CFR Part 58 and the Ambient Air Monitoring Plan maintained by GA DNR
Date of flowrate verification check	Monthly as required by 40 CFR Part 58 Appendix A
Description, date(s) of maintenance activities	See GA-AQY-Page 7 of this testing report

Time Series Plots

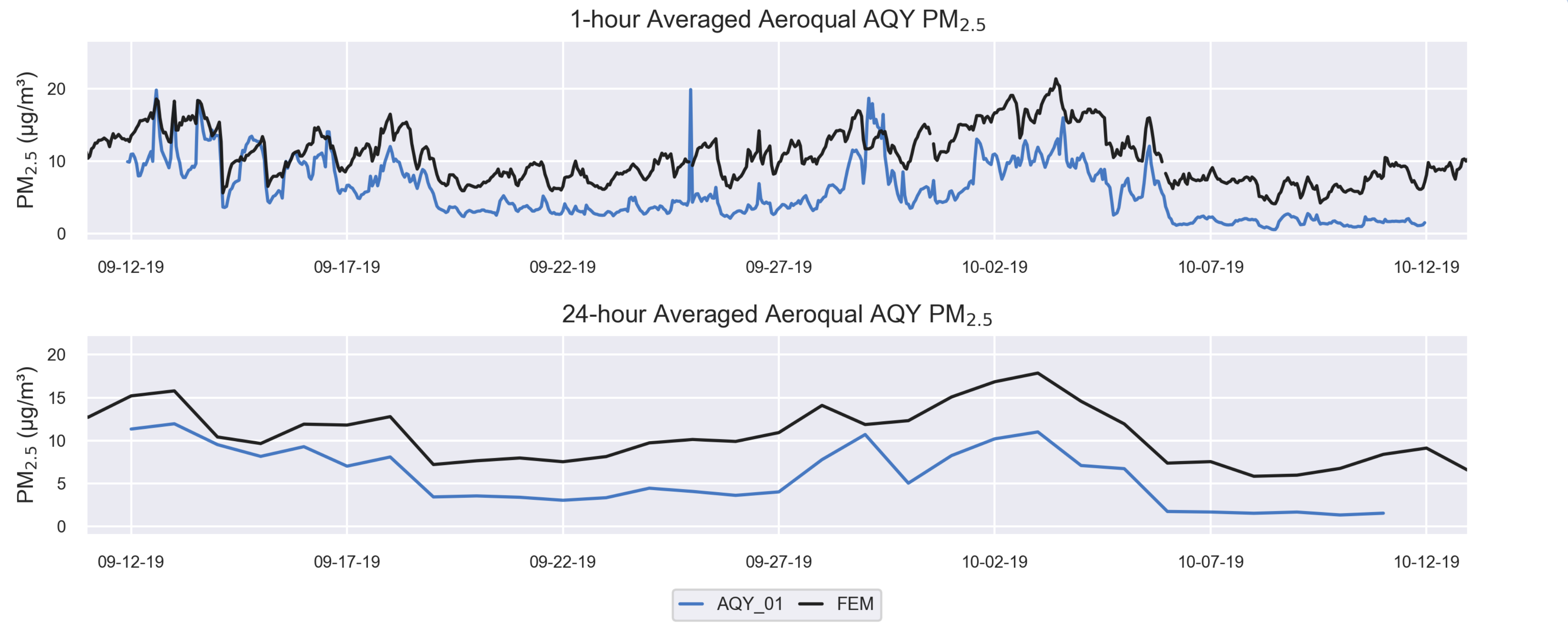

Scatter Plots: Comparison to FRM/FEM

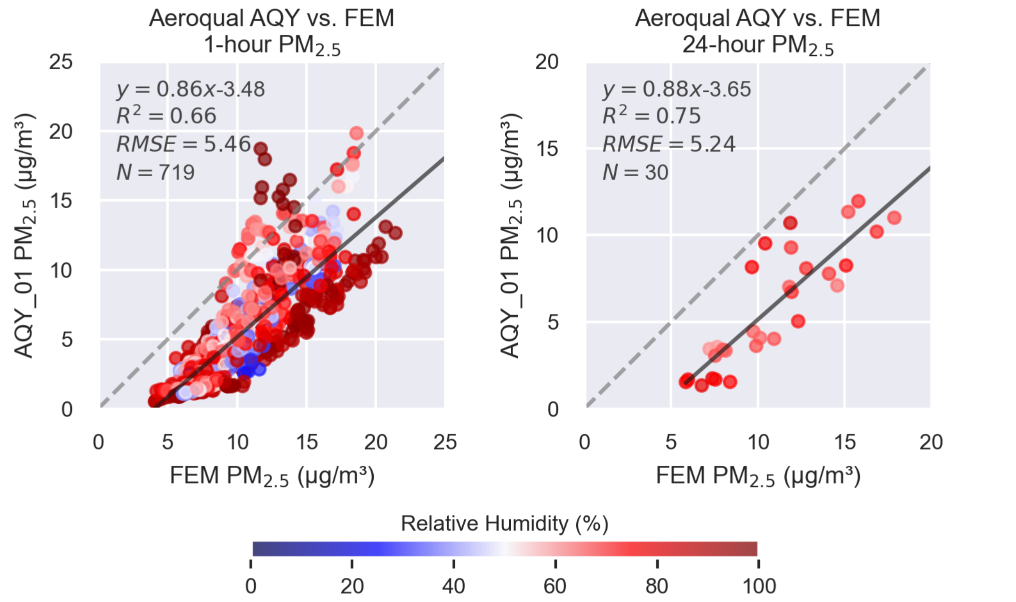

Range of FRM/FEM concentrations over duration of base test (µg/m³)	4.1-21.4 (1-hr), 5.8-17.9 (24-hr)
Number of 24-hr periods in FRM/FEM monitor measurements with a goal concentration ≥ 25 µg/m³	0

Performance Metrics

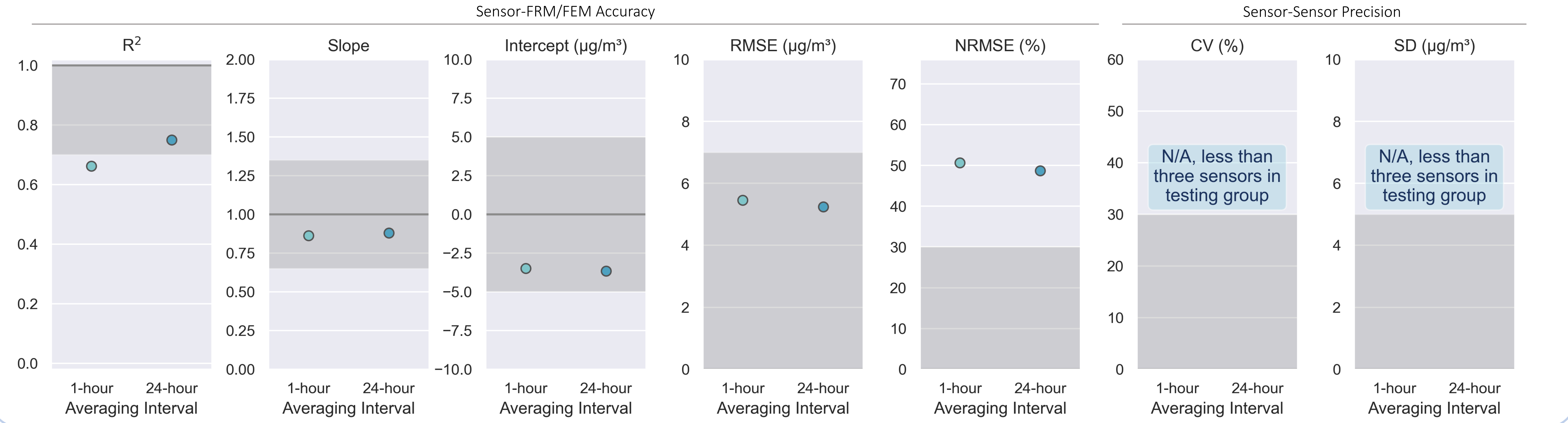

Meteorological Conditions During Deployment

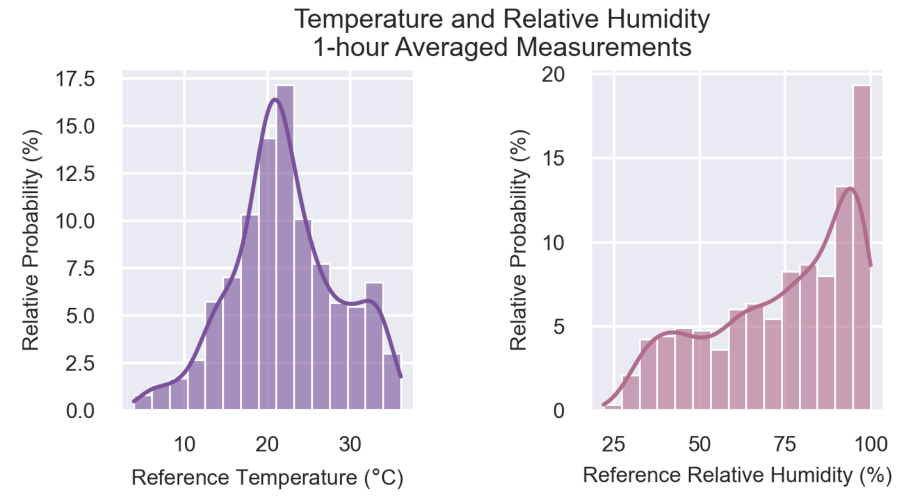

Number of 24-hr periods outside sensor manufacture-listed temperature operational range (-10 to 40 °C)	0
Number of 24-hr periods outside sensor manufacture-listed relative humidity operational range (no operational range specified)	-

Meteorological Influence

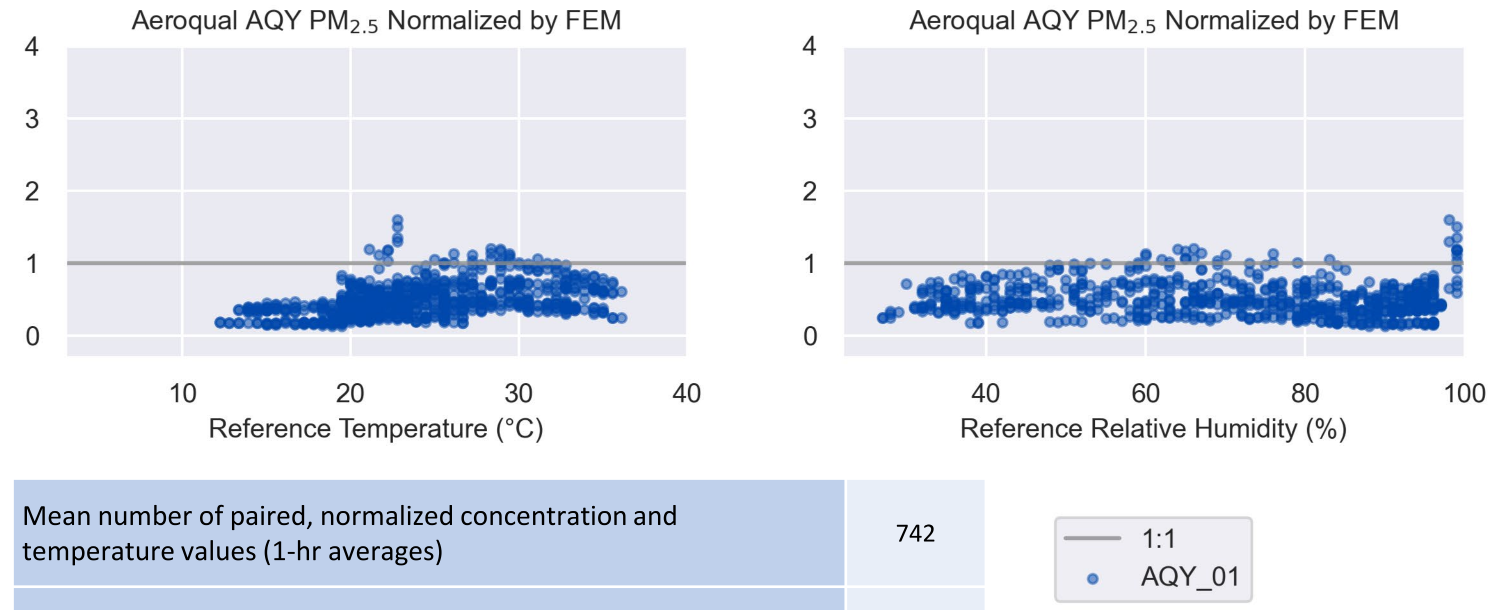

Mean number of paired, normalized concentration and temperature values (1-hr averages)	742
Mean number of paired, normalized concentration and relative humidity values (1-hr averages)	742

Testing Report - $PM_{2.5}$ Base Testing

Aeroqual AQY

This report reflects out-of-the-box performance

Initial Base Testing - Decatur, GA
U.S. Environmental Protection Agency
Office of Research and Development
PI: Clements.Andrea@epa.gov
919-541-1363
September 2019—October 2019

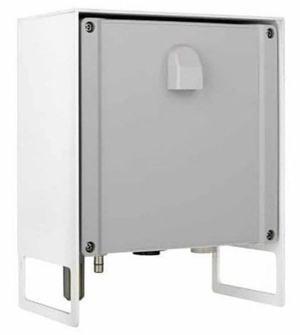

Image Attribution: Aeroqual Limited (Auckland, New Zealand)

Tabular Statistics

Sensor-FRM/FEM Correlation

	Bias and Linearity						Data Quality			
	R^2		Slope		Intercept (µg/m³)		Uptime (%)		Number of paired sensor and FRM/FEM concentration values	
	1-Hour ○	24-Hour ●	1-Hour ●	24-Hour ●	1-Hour ●	24-Hour ●	1-Hour ●	24-Hour ●	1-Hour	24-Hour
Metric Target Range	≥ 0.70	≥ 0.70	1.0 ± 0.35	1.0 ± 0.35	-5 ≤ b ≤ 5	-5 ≤ b ≤ 5	75%*	75%*	-	-
Sensor AQY_01	0.66	0.75	0.86	0.88	-3.48	-3.65	100	100	719	30

	Error			
	RMSE (µg/m³)		NRMSE (%)	
	1-Hour ★	24-Hour ★	1-Hour ☆	24-Hour ☆
Metric Target Range	≤ 7.0	≤ 7.0	≤ 30.0	≤ 30.0
Deployment Value	5.5	5.2	50.7	48.7

Device-specific metrics (computed for each sensor in evaluation)
○○○ Metric value for none of devices tested falls within the target range
●○○ Metric value for one of devices tested falls within the target range
●●○ Metric value for two of devices tested falls within the target range
●●● Metric value for three of devices tested falls within the target range

Single-valued metrics (computed via entire evaluation dataset)
☆ Indicates that the metric value is not within the target range
★ Indicates that the metric value is within the target range

Sensor-Sensor Precision[1]

	Precision (between collocated sensors)				Data Quality	
	CV (%)		SD (µg/m³)		Number of concurrent sensor concentration pairs	
	1-Hour ☆	24-Hour ☆	1-Hour ☆	24-Hour ☆	1-Hour	24-Hour
Metric Target Range	≤ 30.0	≤ 30.0	≤ 5.0	≤ 5.0	-	-
Deployment Value	-	-	-	-	-	-

[1]Precision statistics are computed for evaluations with at least three collocated sensor units. Metric values are left blank for evaluations with two or fewer sensor units.

*This value is only a recommendation for ensuring data quality and is not included in the list of target values discussed in Section 4 of the Performance Testing Protocols, Metrics, and Target Values for Fine Particulate Matter Air Sensors document.

U.S. Environmental Protection Agency
Office of Research and Development
PI: Clements.Andrea@epa.gov
919-541-1363

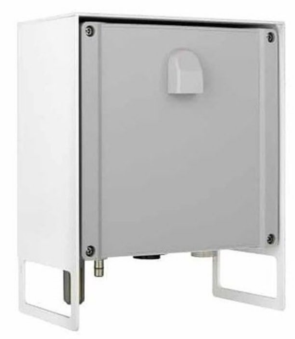

Image Attribution: Aeroqual Limited (Auckland, New Zealand)

Sensor-FRM/FEM Scatter Plots

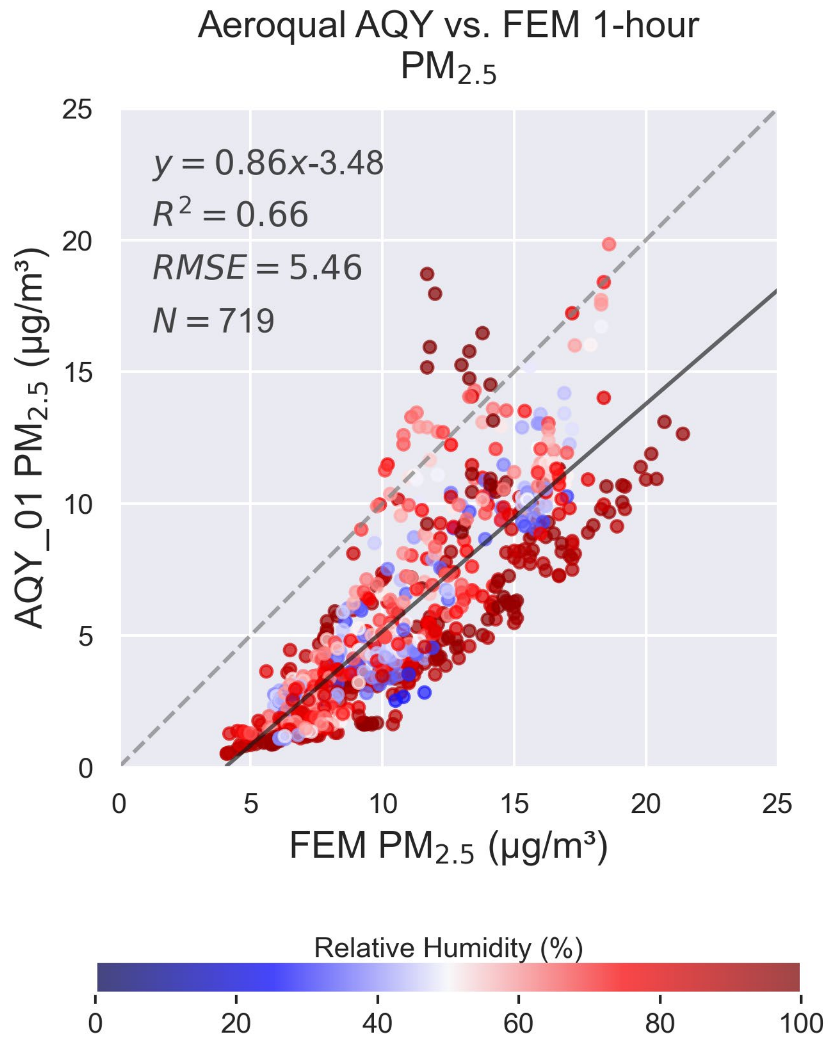

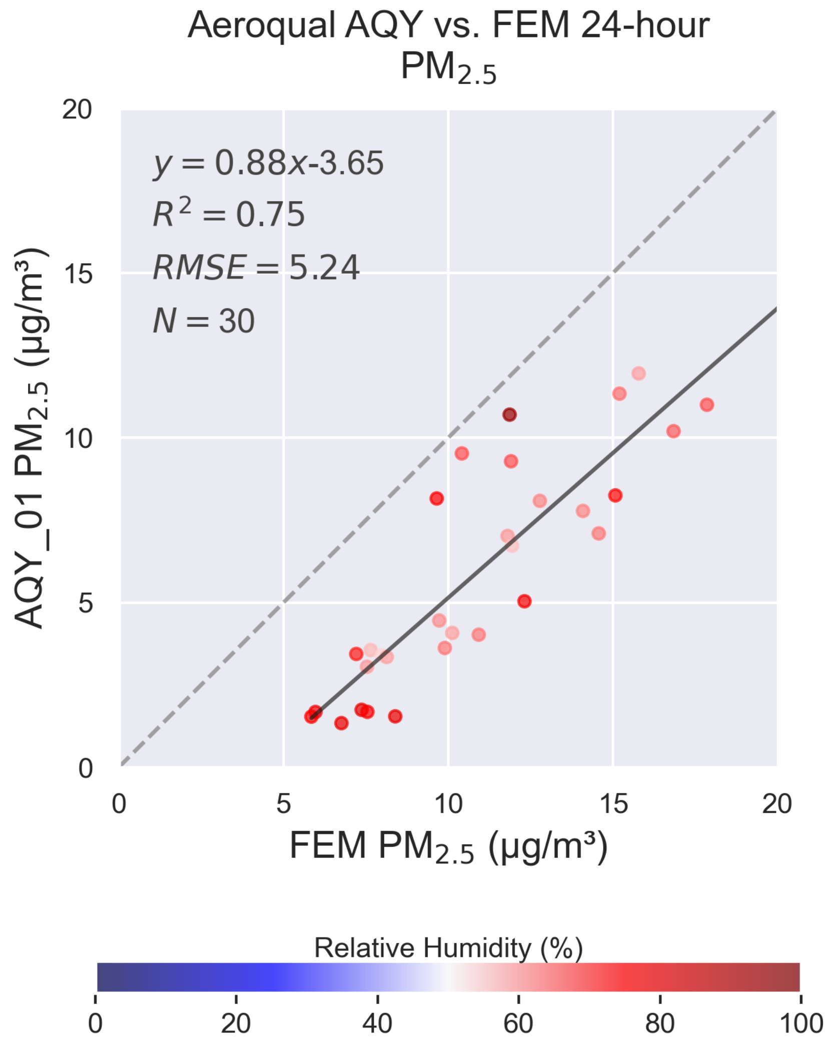

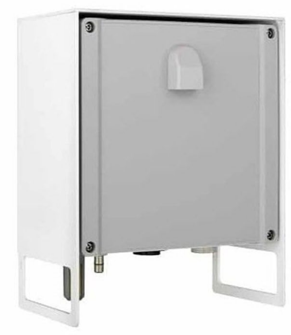

Image Attribution: Aeroqual Limited (Auckland, New Zealand)

Supplemental Information

Abbreviations used in Supplemental Information

FRM	Federal Reference Method
FEM	Federal Equivalent Method
SOP	Standard Operating Procedure
QAPP	Quality Assurance Project Plan
QC	Quality Control

Supplemental Documentation	Attached	Description & URL or file path to documentation
Field observations and sensor data flags	☑	See GA-AQY-Page 6 of this testing report
Maintenance logs	☐	No logs recorded during testing
Standard operating procedure(s)	☐	U.S. EPA Office Of Research and Development SOP available upon request
Photos of equipment setup and testing	☑	See GA-AQY-Page 5 of this testing report
Product specifications sheet(s)	☐	N/A*
Product manual(s)	☐	N/A*
Data storage and transmission method	☑	See GA-AQY-Page 6 of this testing report
Data correction approach	☑	See GA-AQY-Page 6 of this testing report
Issues encountered	☑	See GA-AQY-Page 6 of this testing report
Data analysis/correction scripts and version	☑	Averaging and processing of data, calculation of performance metrics, and generation of figures and other supplementary material for analysis were obtained using Python 3.9.7 with the packages sensortoolkit v0.8.3b2, pandas 1.3.5, NumPy 1.21.2, Matplotlib 3.5.0, statsmodels 0.13.0, and seaborn 0.11.2. All packages are available from the Python Package Index (PyPI) at https://pypi.org. The integrated development environment (IDE) Spyder 5.1.5 was used for scripting and data visualization. Version control for the Python base, packages, and IDE were all managed by conda 4.11.0.
Air Monitoring Station QAPP	☐	U.S. EPA Office Of Research and Development QAPP available upon request
Summary of FRM/FEM monitor QC checks	☑	See GA-AQY-Page 8 of this testing report
Manufacturer website for FRM/FEM monitor	☑	Teledyne API: T640/T640X Product website
FRM/FEM monitor manual	☑	Teledyne API: T640/T640X Product Manual
FRM/FEM monitor specifications sheet(s)	☑	Teledyne API: T640/T640X Specification Sheet
Other documents	☑	Manufacturer notice of AQY sales on hold

*As of 3/18/2021, the manufacturer of the AQY has placed sales of a similar unit on hold. Documentation for the AQY is currently unavailable from the manufacturer's website.

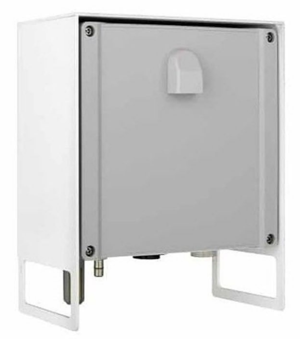

Image Attribution: Aeroqual Limited (Auckland, New Zealand)

Supplemental Information: Photos of Testing Site and Equipment Setup

Site Description:
The South DeKalb monitoring station was initially established as an O3 site in 1974 and is located in the Atlanta area to the southeast of the city. More measurements were added to the site in the 1990's and 2000's. This site is currently a multipollutant site that is designated as a National Core (Ncore), Photochemical Assessment Monitoring Station (PAMS), and National Air Toxics Trends Station (NATTS) site for the state of Georgia. Measurements at South DeKalb include continuous analyzers for ozone, carbon monoxide, nitrogen dioxide, sulfur dioxide, and particulate matter (PM) with an aerodynamic diameter less than 2.5 microns (PM2.5) and 10 microns (PM10). Volatile organic compounds (VOCs) are also measured during summer periods via AutoGC as well as year-round via canister sampling. Local meteorology is also measured at the site, including wind speed and direction, temperature, relative humidity, pressure, and precipitation.

Figure 1: Aeroqual AQY sensor (indicated by red arrow) attached to metal railing atop the sampling shelter at the monitoring site.

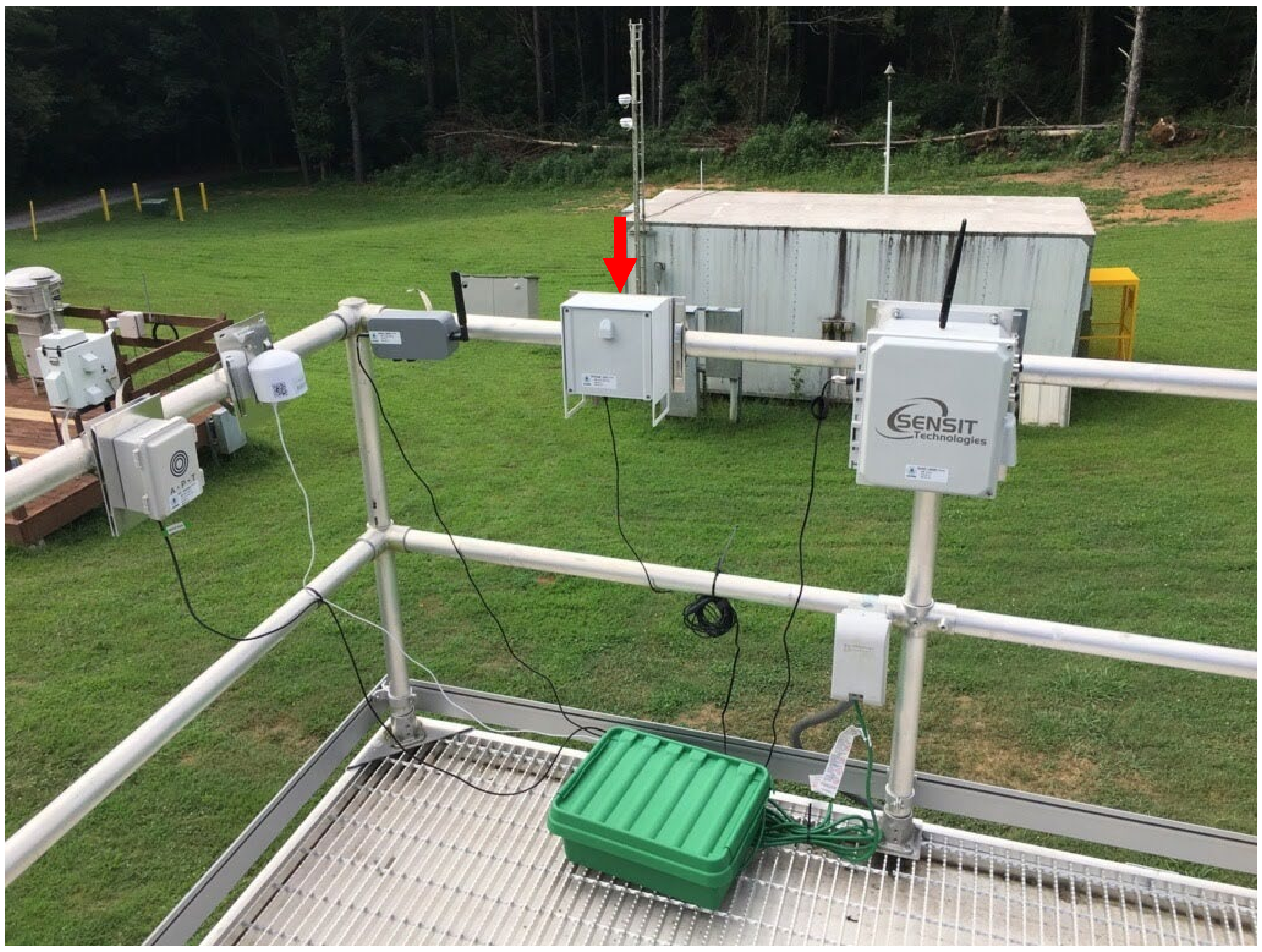

Figure 2: South Dekalb Monitoring Station

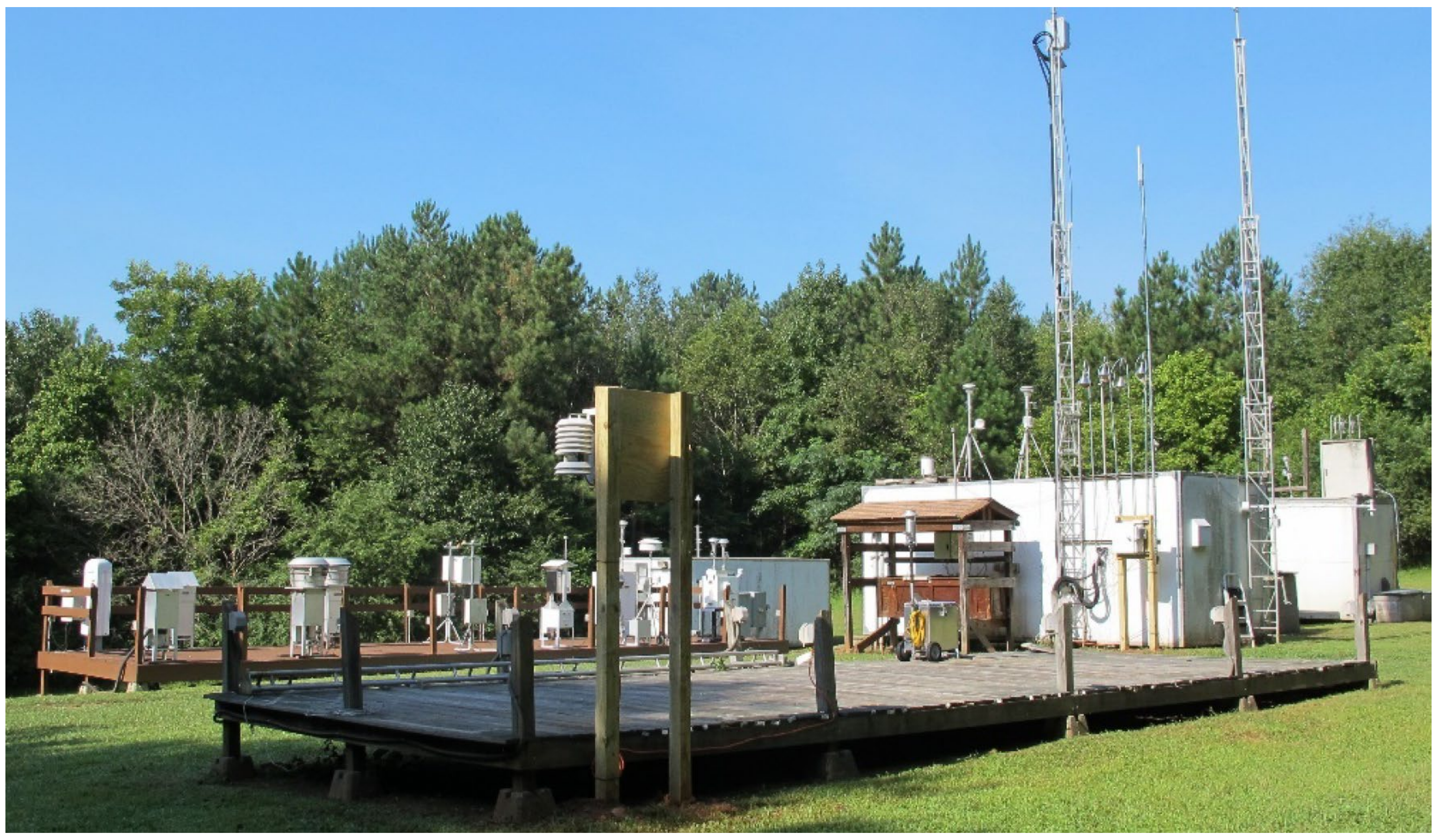

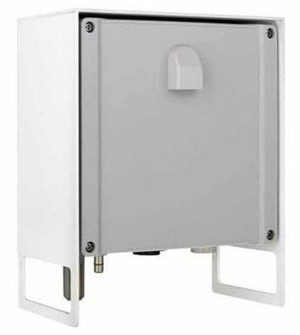

Image Attribution: Aeroqual Limited (Auckland, New Zealand)

Supplemental Information: Data Storage, Correction Approach, and Issues Encountered

Data Storage and Transmission Method

As part of CRADA #934-16 between Aeroqual and US EPA, Aeroqual supported data streaming. SIM cards were installed and data flowed to the Aeroqual Cloud. The 1-minute raw data was acquired weekly using the Aeroqual Cloud (*last accessed 5/11/22*) user interface (UI). The AQY has an internal data storage USB flash drive as a data backup, however the software on the drive is proprietary to Aeroqual and unreadable by outside systems.

Data Correction Approach

This evaluation report reflects "out-of-the-box" performance of the AQY. The manufacturer provides a procedure by which local collocation (sensor operated along side an FRM/FEM) data can be collected, a gain (slope) and offset (intercept) determined, and parameters entered into the Aeroqual Cloud user interface to be applied to all subsequently collected data. This procedure and feature was NOT used prior to this evaluation. Prospective consumers may get different performance from this device if they utilize this feature.

After acquisition, the raw data was processed using the *sensortoolkit* python code library (v0.8.3b2). A continuous data set at the recorded sampling frequency was written to a .csv file. 1-hour and 24-hour averaged data sets were generated using a 75% completeness threshold and saved as separate .csv files. Outliers were NOT removed from data sets in order to assess "out-of-the-box" sensor performance.

Issues Encountered

Pre-deployment observations

- *Timestamp inaccuracies*: During pre-deployment, the AQY devices did not properly sync timestamps with the onboard Real-Time Clock. Connecting the units to the internet by cellular or Wi-Fi allowed the unit to sync with internet time and resulted in proper timestamps.

Field observations and sensor data flags

The AQY unit was deployed at the South Dekalb monitoring site on 8/1/2019. Due to a miscommunication, the sensor unit was temporarily removed from the site on 8/28/2019 and was reinstalled in the previous configuration on 9/11/2019.

The following table contains data flags describing events that were encountered during the testing period. On 9/11/2019, a brief power outage was reported and lasted less than 10 minutes. The field technician was not at the site when the outage occurred, indicating that the cause of the outage was likely not attributed to operator error. The AQY unit was shut down temporarily during multiple occasions during the testing period, with the duration of shut down ranging from approximately 10-15 minutes. On 10/9/2019 during a routine site visit, the field technician noticed that the AQY unit's USB cable required reseating, as it had loosened since the prior site visit.

Start Time (UTC)	End Time (UTC)	Sensor Serial ID	Parameters Impacted	Flag
2019-09-11 21:08:00+00:00	2019-09-11 21:08:00+00:00	AQY_01	ALL	0-Sensor deployment
2019-09-16 19:38:00+00:00	2019-09-16 19:45:00+00:00	AQY_01	ALL	9-Probable power loss
2019-10-02 20:12:00+00:00	2019-10-02 20:12:00+00:00	AQY_01	ALL	3-Intentional shutdown for maintenance
2019-10-02 20:22:00+00:00	2019-10-02 20:22:00+00:00	AQY_01	ALL	3-Intentional shutdown for maintenance
2019-10-09 20:49:00+00:00	2019-10-09 20:49:00+00:00	AQY_01	ALL	3-Intentional shutdown for maintenance
2019-10-09 20:50:00+00:00	2019-10-09 21:03:00+00:00	AQY_01	ALL	4-Routine sensor maintenance - Sensor needed USB reformat

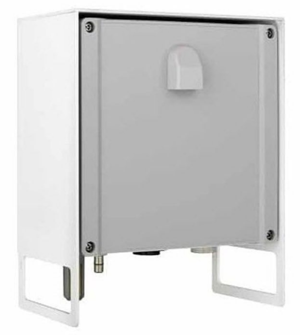

Image Attribution: Aeroqual Limited (Auckland, New Zealand)

Description of Data Flags

AQS

The U.S. EPA's Air Quality System (AQS) is the Agency's primary ambient air monitoring data archive. A comprehensive list of data flags that are recorded alongside AQS data sets, referred to by U.S. EPA as 'qualifiers', can be found at the following link: https://aqs.epa.gov/aqsweb/documents/codetables/qualifiers.html

Invalidation of reference data: AQS qualifiers are organized by qualifier type, which indicates whether data logged alongside qualifier flags should be invalidated (set null). Qualifiers with type "Null Data Qualifier" are invalidated, and includes data logged during periods that coincide with QC checks (e.g., "BF-Precision/Zero/Span", "BJ- Operator Error", "BL - QA Audit", "AZ - QC Audit") among other events such as power outages. Data logged alongside qualifiers with type "Quality Assurance Qualifiers" are not invalidated and are included in this analysis (e.g., concentrations less than the federal MDL for the reference monitor "MD – Value less than MDL", QA reviewed values "Validated Value").

Data Flags Recorded During Testing

FRM/FEM Monitor	Timestamp (UTC)	Flag
Teledyne API T640X (Acquired via AQS)	2019-09-24 23:00:00+0000	BE - Building/Site Repair.
	2019-09-30 13:00:00+0000	AZ - QC Audit
	2019-10-05 22:00:00+0000	BE - Building/Site Repair.

Meteorological Instrument	Timestamp (UTC)	Flag
RM Young 41375VC Temperature Monitor (Data acquired via AQS)	2019-09-27 13:00:00+0000 to 2019-09-27 14:00:00+0000	BA - Maintenance/Routine Repairs
[Make Model] Relative Humidity Monitor (Data acquired via AQS)	2019-09-27 13:00:00+0000 to 2019-09-27 14:00:00+0000	BA - Maintenance/Routine Repairs

Testing Report - $PM_{2.5}$ Base Testing

APT Maxima

This report reflects out-of-the-box performance

Initial Base Testing - Decatur, GA

U.S. Environmental Protection Agency
Office of Research and Development
PI: Clements.Andrea@epa.gov
919-541-1363

September 2019—October 2019

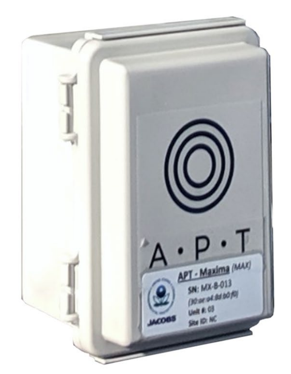

Deployment Details

Testing Organization and Site Information	
Testing organization (Name, Organization type, Contact website)	U.S. Environmental Protection Agency - Office of Research and Development<br>Federal Government<br>Air Sensor Toolbox | U.S. EPA Website
Testing location (City, State, Latitude and Longitude)	South Dekalb<br>Decatur, GA<br>33.6877, -84.2905
AQS site ID	13 - 089 - 0002
Sampling timeframe (MM-DD-YY)	09-18-19 to 10-18-19
Sensor data source	Onboard MicroSD card
Reference data source	AQS API download

Sensor Information			
Manufacturer, model	APT Maxima		
Device firmware version	v5.0 (installed 5/2/2019)		
Sampling time interval	30-seconds		
Sensor serial numbers	MAX_01		
Issues encountered during deployment?	☑	See GA-MAX-Page 6 of this testing report	

FRM/FEM Information	
Manufacturer, model, designation	Teledyne Advanced Pollution Instrumentation T640X FEM
Sampling time interval	1-hour averaging
Date of calibration	As required by 40 CFR Part 58 and the Ambient Air Monitoring Plan maintained by GA DNR
Date of flowrate verification check	Monthly as required by 40 CFR Part 58 Appendix A
Description, date(s) of maintenance activities	See GA-MAX-Page 7 of this testing report

Time Series Plots

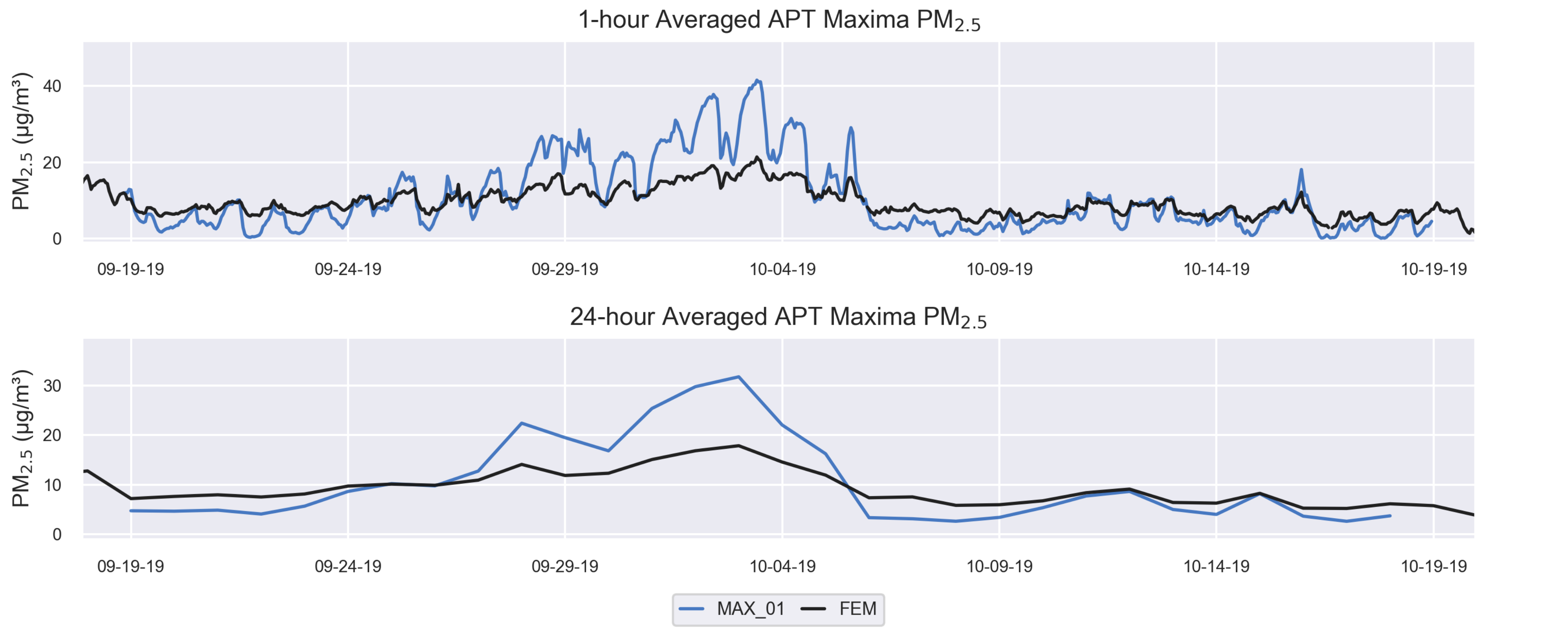

Scatter Plots: Comparison to FRM/FEM

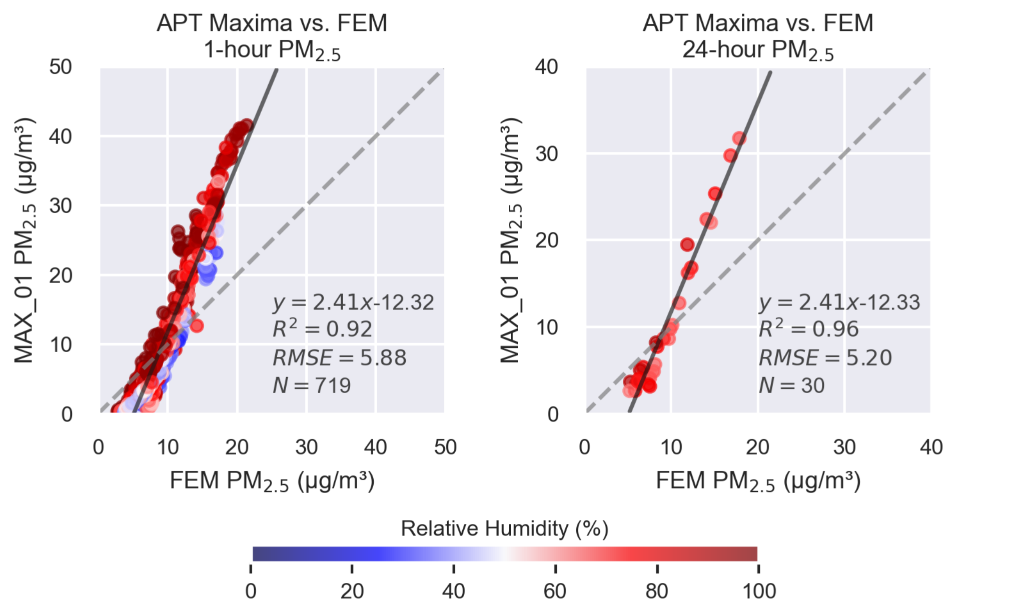

Range of FRM/FEM concentrations over duration of base test (µg/m³)	2.8-21.4 (1-hr), 5.2-17.9 (24-hr)
Number of 24-hr periods in FRM/FEM monitor measurements with a goal concentration ≥ 25 µg/m³	0

Performance Metrics

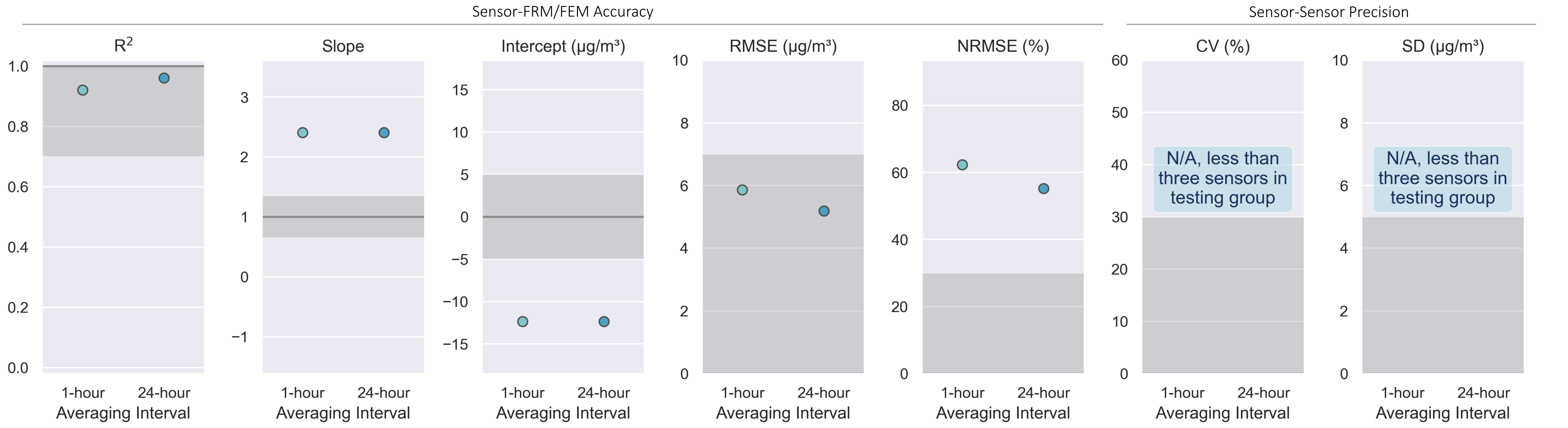

Meteorological Conditions During Deployment

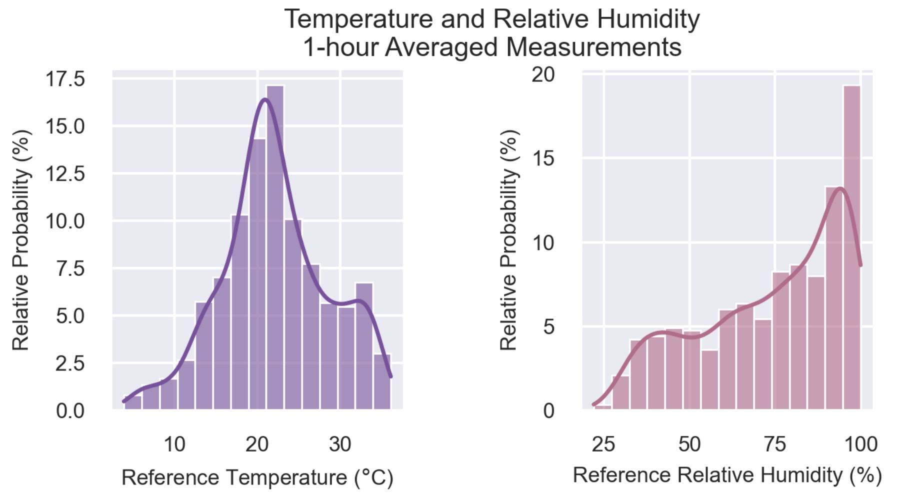

Number of 24-hr periods outside sensor manufacture-listed temperature operational range (no operational range specified)	-
Number of 24-hr periods outside sensor manufacture-listed relative humidity operational range (no operational range specified)	-

Meteorological Influence

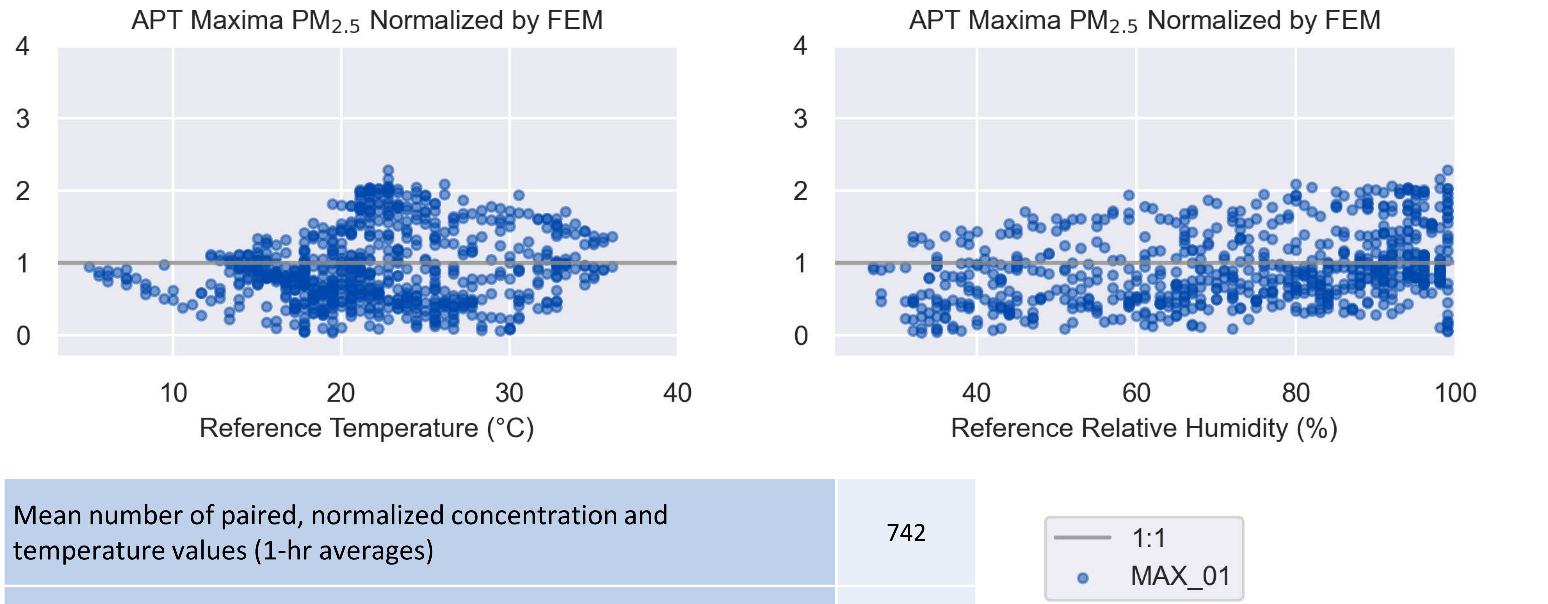

Mean number of paired, normalized concentration and temperature values (1-hr averages)	742
Mean number of paired, normalized concentration and relative humidity values (1-hr averages)	742

Testing Report - $PM_{2.5}$ Base Testing

APT Maxima

This report reflects out-of-the-box performance

Initial Base Testing - Decatur, GA
U.S. Environmental Protection Agency
Office of Research and Development
PI: Clements.Andrea@epa.gov
919-541-1363
September 2019—October 2019

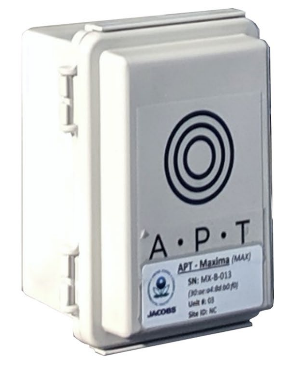

Tabular Statistics

Sensor-FRM/FEM Correlation

	Bias and Linearity						Data Quality			
	R^2		Slope		Intercept (µg/m³)		Uptime (%)		Number of paired sensor and FRM/FEM concentration values	
	1-Hour ●	24-Hour ●	1-Hour ○	24-Hour ○	1-Hour ○	24-Hour ○	1-Hour ●	24-Hour ●	1-Hour	24-Hour
Metric Target Range	≥ 0.70	≥ 0.70	1.0 ± 0.35	1.0 ± 0.35	-5 ≤ b ≤ 5	-5 ≤ b ≤ 5	75%*	75%*	-	-
Sensor MAX_01	0.92	0.96	2.41	2.41	-12.32	-12.33	100	100	719	30

	Error			
	RMSE (µg/m³)		NRMSE (%)	
	1-Hour ★	24-Hour ★	1-Hour ☆	24-Hour ☆
Metric Target Range	≤ 7.0	≤ 7.0	≤ 30.0	≤ 30.0
Deployment Value	5.9	5.2	62.3	55.2

Device-specific metrics (computed for each sensor in evaluation)
○○○ Metric value for none of devices tested falls within the target range
●○○ Metric value for one of devices tested falls within the target range
●●○ Metric value for two of devices tested falls within the target range
●●● Metric value for three of devices tested falls within the target range

Single-valued metrics (computed via entire evaluation dataset)
☆ Indicates that the metric value is not within the target range
★ Indicates that the metric value is within the target range

Sensor-Sensor Precision[1]

	Precision (between collocated sensors)				Data Quality	
	CV (%)		SD (µg/m³)		Number of concurrent sensor concentration pairs	
	1-Hour ☆	24-Hour ☆	1-Hour ☆	24-Hour ☆	1-Hour	24-Hour
Metric Target Range	≤ 30.0	≤ 30.0	≤ 5.0	≤ 5.0	-	-
Deployment Value	-	-	-	-	-	-

[1]Precision statistics are computed for evaluations with at least three collocated sensor units. Metric values are left blank for evaluations with two or fewer sensor units.

*This value is only a recommendation for ensuring data quality and is not included in the list of target values discussed in Section 4 of the Performance Testing Protocols, Metrics, and Target Values for Fine Particulate Matter Air Sensors document.

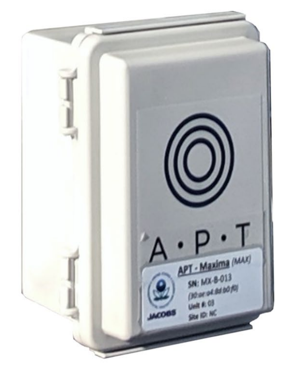

Sensor-FRM/FEM Scatter Plots

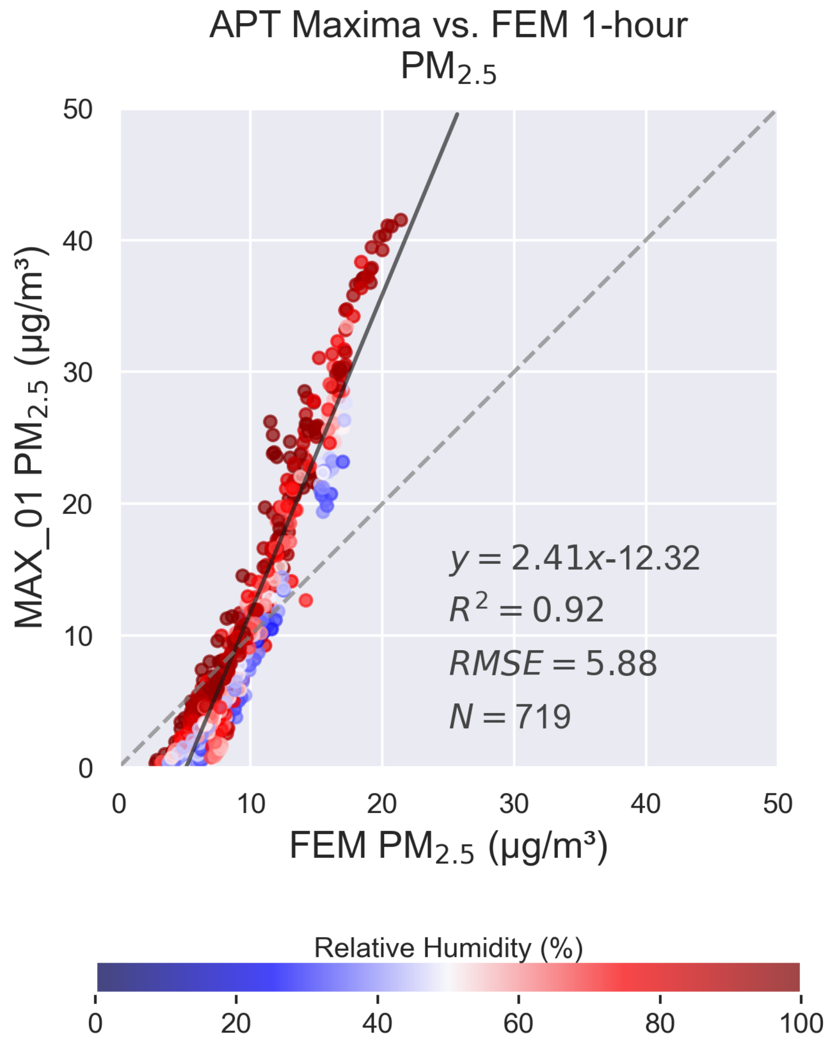

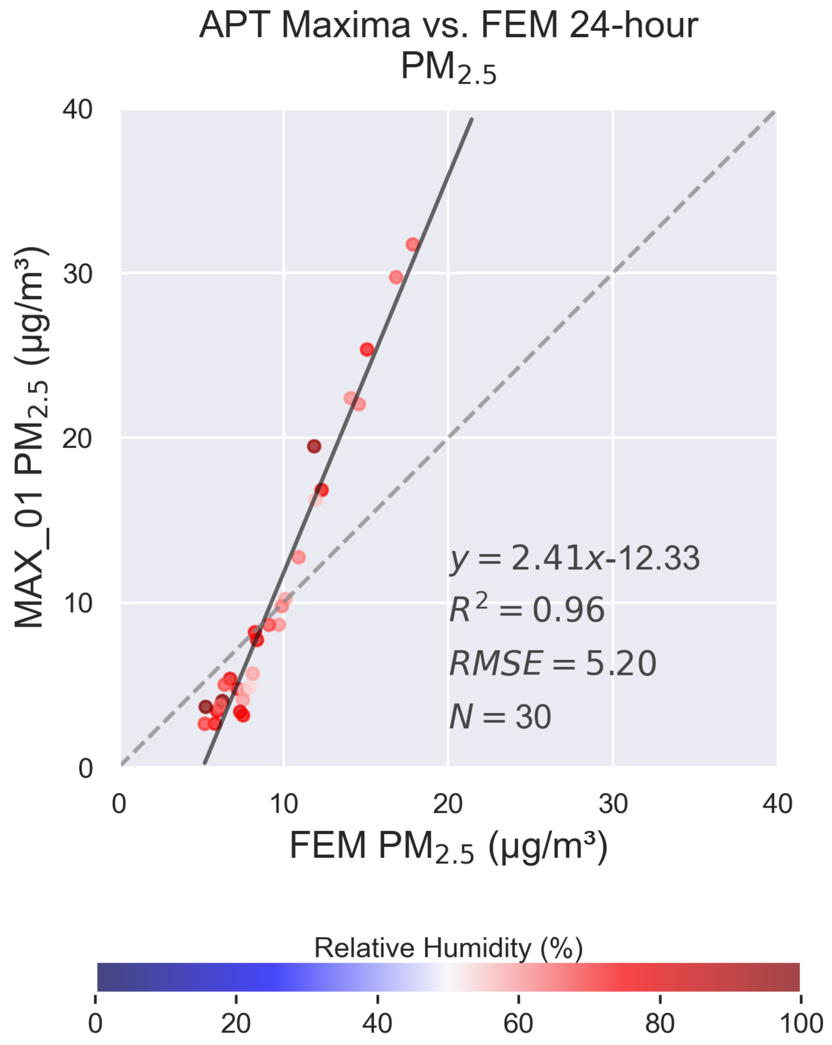

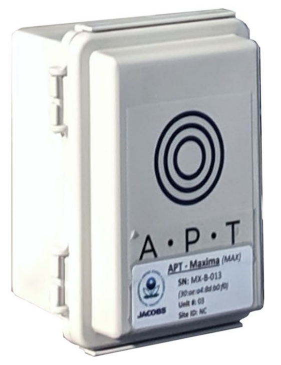

Supplemental Information

Abbreviations used in Supplemental Information

FRM	Federal Reference Method
FEM	Federal Equivalent Method
SOP	Standard Operating Procedure
QAPP	Quality Assurance Project Plan
QC	Quality Control

Supplemental Documentation	Attached	Description & URL or file path to documentation
Field observations and sensor data flags	☑	See GA-MAX-Page 6 of this testing report
Maintenance logs	☐	No logs recorded during testing
Standard operating procedure(s)	☐	U.S. EPA Office Of Research and Development SOP available upon request
Photos of equipment setup and testing	☑	See GA-MAX-Page 5 of this testing report
Product specifications sheet(s)	☐	N/A
Product manual(s)	☐	N/A
Data storage and transmission method	☑	See GA-MAX-Page 6 of this testing report
Data correction approach	☑	See GA-MAX-Page 6 of this testing report
Issues encountered	☑	See GA-MAX-Page 6 of this testing report
Data analysis/correction scripts and version	☑	Averaging and processing of data, calculation of performance metrics, and generation of figures and other supplementary material for analysis were obtained using Python 3.9.7 with the packages sensortoolkit v0.8.3b2, pandas 1.3.5, NumPy 1.21.2, Matplotlib 3.5.0, statsmodels 0.13.0, and seaborn 0.11.2. All packages are available from the Python Package Index (PyPI) at https://pypi.org. The integrated development environment (IDE) Spyder 5.1.5 was used for scripting and data visualization. Version control for the Python base, packages, and IDE were all managed by conda 4.11.0.
Air Monitoring Station QAPP	☐	U.S. EPA Office Of Research and Development QAPP available upon request
Summary of FRM/FEM monitor QC checks	☑	See GA-MAX-Page 7 of this testing report
Manufacturer website for FRM/FEM monitor	☑	Teledyne API: T640/T640X Product website
FRM/FEM monitor manual	☑	Teledyne API: T640/T640X Product Manual
FRM/FEM monitor specifications sheet(s)	☑	Teledyne API: T640/T640X Specification Sheet
Other documents	☐	

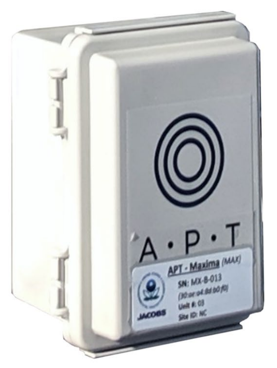

Supplemental Information: Photos of Testing Site and Equipment Setup

Site Description:

The South DeKalb monitoring station was initially established as an O3 site in 1974 and is located in the Atlanta area to the southeast of the city. More measurements were added to the site in the 1990's and 2000's. This site is currently a multipollutant site that is designated as a National Core (Ncore), Photochemical Assessment Monitoring Station (PAMS), and National Air Toxics Trends Station (NATTS) site for the state of Georgia. Measurements at South DeKalb include continuous analyzers for ozone, carbon monoxide, nitrogen dioxide, sulfur dioxide, and particulate matter (PM) with an aerodynamic diameter less than 2.5 microns (PM2.5) and 10 microns (PM10). Volatile organic compounds (VOCs) are also measured during summer periods via AutoGC as well as year-round via canister sampling. Local meteorology is also measured at the site, including wind speed and direction, temperature, relative humidity, pressure, and precipitation.

Figure 1: APT Maxima sensor (indicated by red arrow) attached to metal railing atop the sampling shelter at the monitoring site.

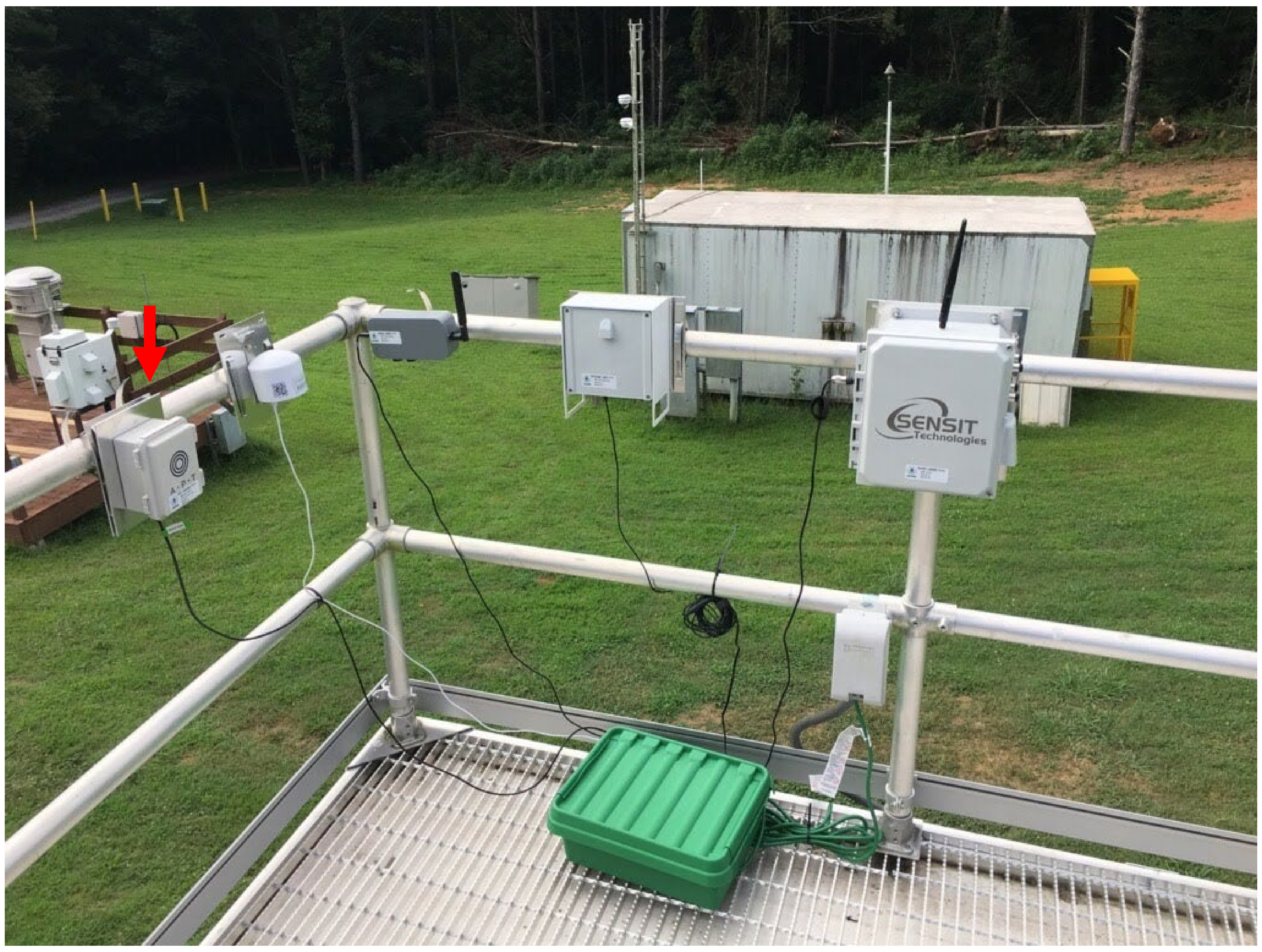

Figure 2: South Dekalb Monitoring Station

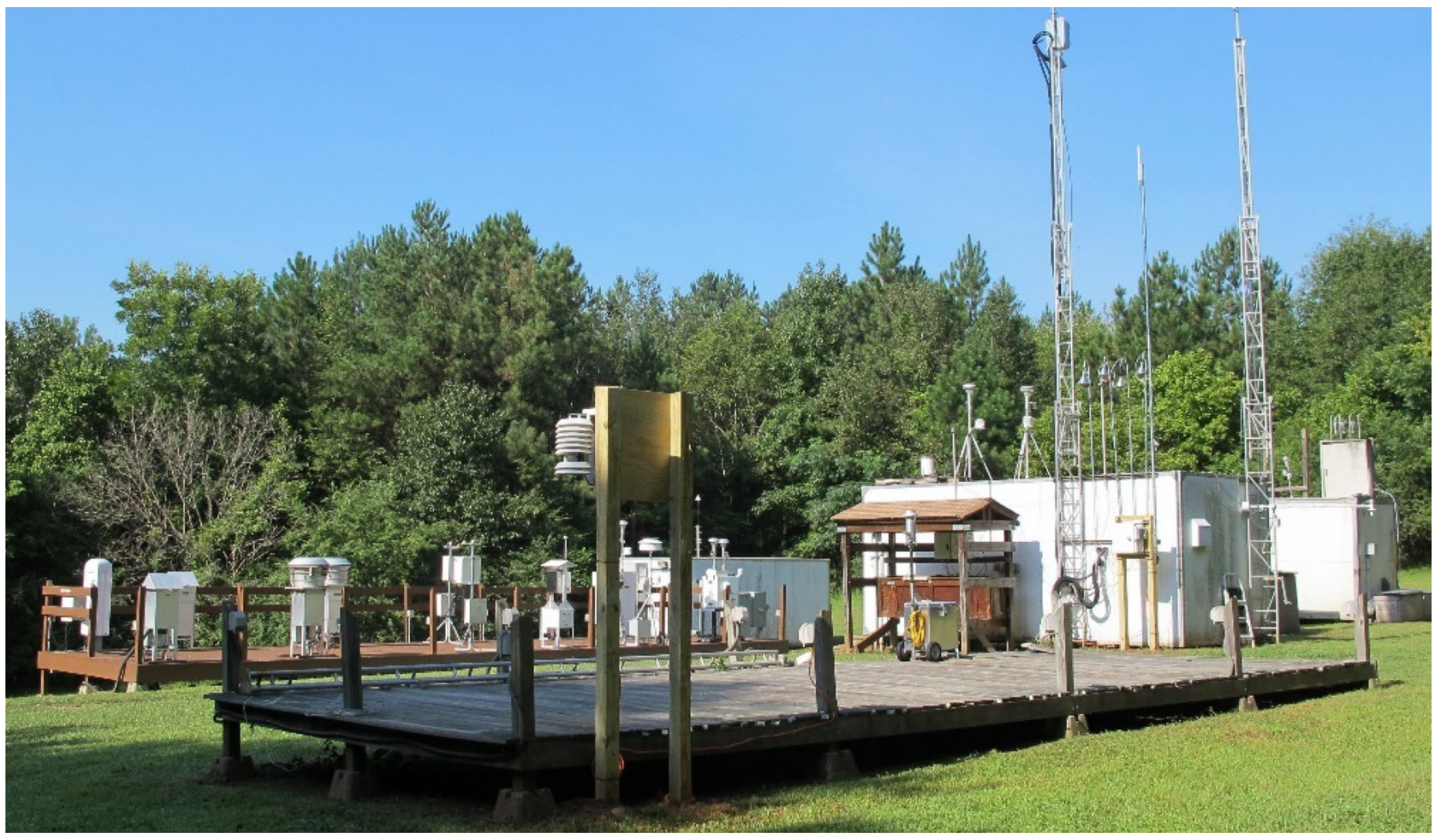

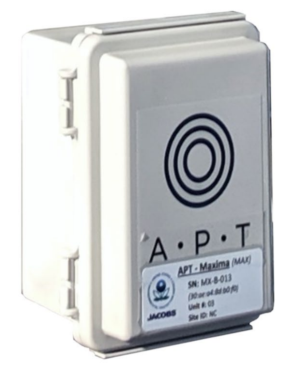

Data Storage and Transmission Method

The APT Maxima records data at 30 second intervals and stores the data on an internal microSD card and transmits data to the APT cloud server (last accessed 5/11/22) via Wi-Fi connection. The user interface for the Maxima sensors was under development during sensor testing, thus weekly raw data files were obtained from the internal MicroSD card. Each field site operator was provided two labeled MicroSD cards for the Maxima that they used to swap out each week. Data from the collected card was then read and processed off-site.

Data Correction Approach

This evaluation report reflects "out-of-the-box" performance of the APT Maxima. The Maxima data sets include two data columns contain $PM_{2.5}$ concentration measurements, "PM2.5(ug/m3)" and "PM2.5_Atm(ug/m3)". These columns correspond to separate correction factors provided by the onboard OEM PM sensor (Plantower PMSA003). For this report, $PM_{2.5}$ measurements for the column labeled "PM2.5(ug/m3)" were selected for analysis.

After acquisition, the raw data was processed using the *sensortoolkit* python code library (v0.8.3b2). A continuous data set at the recorded sampling frequency was written to a .csv file. 1-hour and 24-hour averaged data sets were generated using a 75% completeness threshold and saved as separate .csv files. Outliers were NOT removed from data sets in order to assess "out-of-the-box" sensor performance.

Issues Encountered

Pre-deployment observations

- *Sampling internal abnormality:* Maxima devices did not continuously sample at 30-second intervals as stated in the sensor user guide. Instead, Maxima units occasionally sampled at 31 seconds. This increased sample interval is due to the accumulation of processing time over a certain number of samples. Each 30-second sample is a collection of exactly 30 seconds of sampling followed by a brief fraction of a second used for processing the collected data. Once processed, the next 30-second data collection period begins. When the sum of the processing time reaches one full second, the sample interval then jumps to 31 seconds. Data from this pre-deployment testing indicated that the processing speed for each sensor varies such that 31-second sampling duration occur at random intervals across Maxima units.

Field observations and sensor data flags

The APT Maxima unit was deployed at the South Dekalb monitoring site on 8/1/2019. Due to a miscommunication, the sensor unit was temporarily removed from the site on 8/28/2019 and was reinstalled in the previous configuration on 9/11/2019.

The following table contains data flags describing events that were encountered during the testing period. In addition to the sampling interval abnormality discussed in the pre-deployment observations, during the testing period it was observed that the Maxima occasionally recorded longer-duration sampling intervals, ranging from approximately 40 seconds to over 120 seconds. These longer intervals occurred approximately daily or multiple times a day and at seemingly random intervals, without any discernible pattern regarding the timing of these events.

Start Time (UTC)	End Time (UTC)	Sensor Serial ID	Parameters Impacted	Flag
2019-10-17 07:41:00+00:00	2019-10-17 07:43:00+00:00	MAX_01	ALL	6-Sampling Interval Abnormality
2019-10-18 07:41:00+00:00	2019-10-18 07:43:00+00:00	MAX_01	ALL	6-Sampling Interval Abnormality
2019-10-18 10:58:00+00:00	2019-10-18 11:00:00+00:00	MAX_01	ALL	6-Sampling Interval Abnormality
2019-10-18 15:56:00+00:00	2019-10-18 15:58:00+00:00	MAX_01	ALL	6-Sampling Interval Abnormality

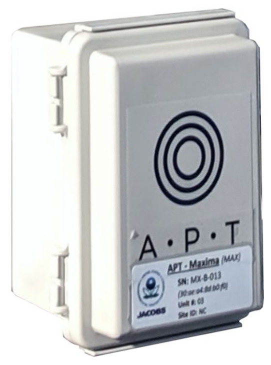

Supplemental Information: Description of FRM/FEM QC Checks and Data Flags

Description of Data Flags

AQS

The U.S. EPA's Air Quality System (AQS) is the Agency's primary ambient air monitoring data archive. A comprehensive list of data flags that are recorded alongside AQS data sets, referred to by U.S. EPA as 'qualifiers', can be found at the following link: https://aqs.epa.gov/aqsweb/documents/codetables/qualifiers.html

Invalidation of reference data: AQS qualifiers are organized by qualifier type, which indicates whether data logged alongside qualifier flags should be invalidated (set null). Qualifiers with type "Null Data Qualifier" are invalidated, and includes data logged during periods that coincide with QC checks (e.g., "BF-Precision/Zero/Span", "BJ- Operator Error", "BL - QA Audit", "AZ - QC Audit") among other events such as power outages. Data logged alongside qualifiers with type "Quality Assurance Qualifiers" are not invalidated and are included in this analysis (e.g., concentrations less than the federal MDL for the reference monitor "MD – Value less than MDL", QA reviewed values "Validated Value").

Data Flags Recorded During Testing

FRM/FEM Monitor	Timestamp (UTC)	Flag
Teledyne API T640X (Acquired via AQS)	2019-09-24 23:00:00+0000	BE - Building/Site Repair.
	2019-09-30 13:00:00+0000	AZ - QC Audit
	2019-10-05 22:00:00+0000	BE - Building/Site Repair.
	2019-10-16 15:00:00+0000	AZ - QC Audit

Meteorological Instrument	Timestamp (UTC)	Flag
RM Young 41375VC Temperature Monitor (Data acquired via AQS)	2019-09-27 13:00:00+0000 to 2019-09-27 14:00:00+0000	BA - Maintenance/Routine Repairs
[Make Model] Relative Humidity Monitor (Data acquired via AQS)	2019-09-27 13:00:00+0000 to 2019-09-27 14:00:00+0000	BA - Maintenance/Routine Repairs

Testing Report - $PM_{2.5}$ Base Testing

Clarity Node

This report reflects out-of-the-box performance

Initial Base Testing - Decatur, GA
U.S. Environmental Protection Agency
Office of Research and Development
PI: Clements.Andrea@epa.gov
919-541-1363
August 2019

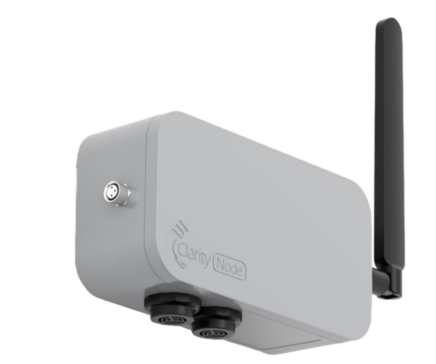

Image Attribution: Clarity Movement Co. (Berkeley, CA)

Deployment Details

Testing Organization and Site Information	
Testing organization (Name, Organization type, Contact website)	U.S. Environmental Protection Agency - Office of Research and Development<br>Federal Government<br>Air Sensor Toolbox | U.S. EPA Website
Testing location (City, State, Latitude and Longitude)	South Dekalb<br>Decatur, GA<br>33.6877, -84.2905
AQS site ID	13 - 089 - 0002
Sampling timeframe (MM-DD-YY)	08-01-19 to 08-28-19
Sensor data source	Clarity Dashboard download
Reference data source	AQS API download

Sensor Information			
Manufacturer, model	Clarity Node		
Device firmware version	Received by EPA May 2019		
Sampling time interval	3-minutes		
Sensor serial numbers	CNO_01		
Issues encountered during deployment?	☐	No Issues	

FRM/FEM Information	
Manufacturer, model, designation	Teledyne Advanced Pollution Instrumentation T640X FEM
Sampling time interval	1-hour averaging
Date of calibration	As required by 40 CFR Part 58 and the Ambient Air Monitoring Plan maintained by GA DNR
Date of flowrate verification check	Monthly as required by 40 CFR Part 58 Appendix A
Description, date(s) of maintenance activities	N/A

Time Series Plots

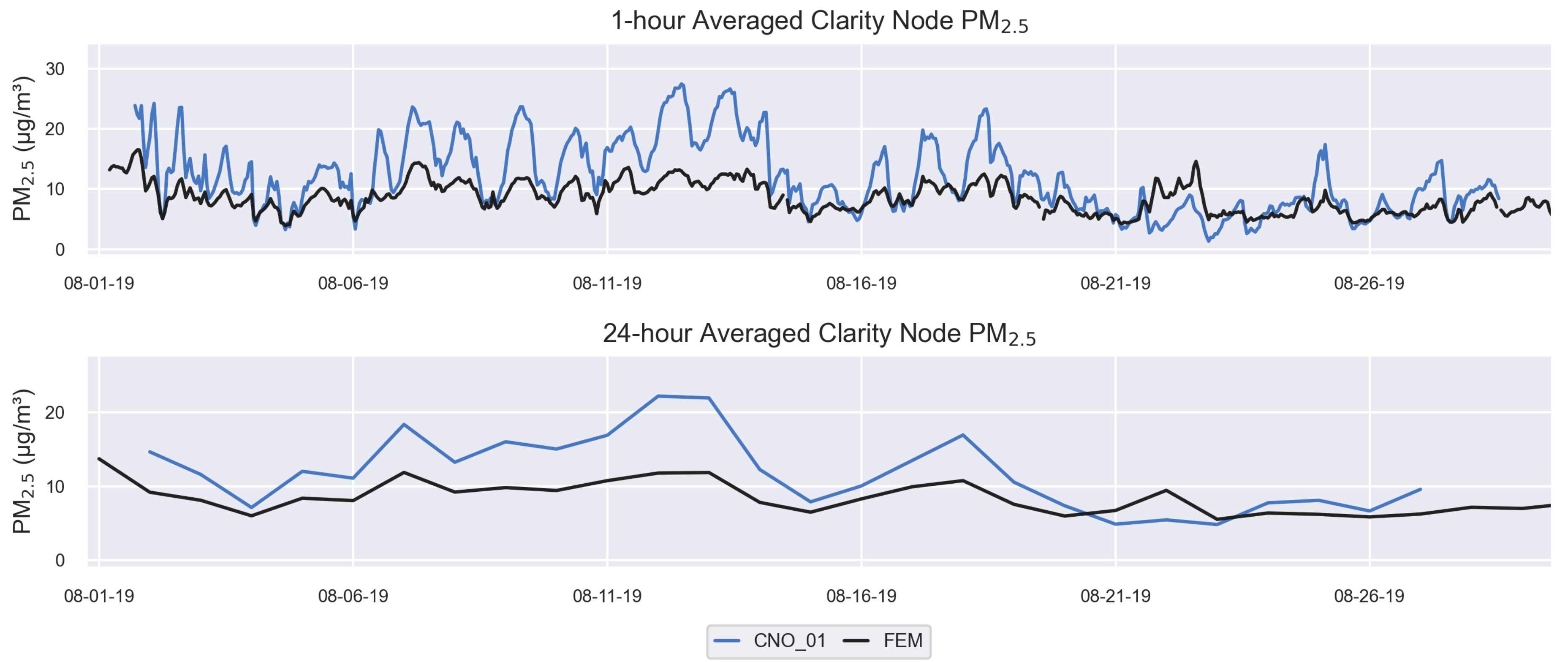

Scatter Plots: Comparison to FRM/FEM

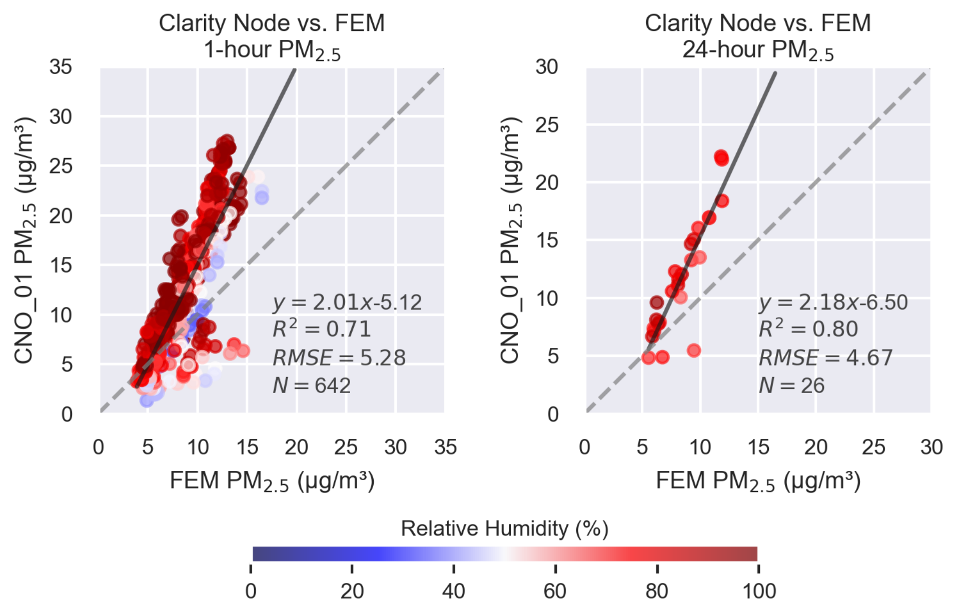

Range of FRM/FEM concentrations over duration of base test (µg/m³)	3.9-16.5 (1-hr), 5.5-11.9 (24-hr)
Number of 24-hr periods in FRM/FEM monitor measurements with a goal concentration ≥ 25 µg/m³	0

Performance Metrics

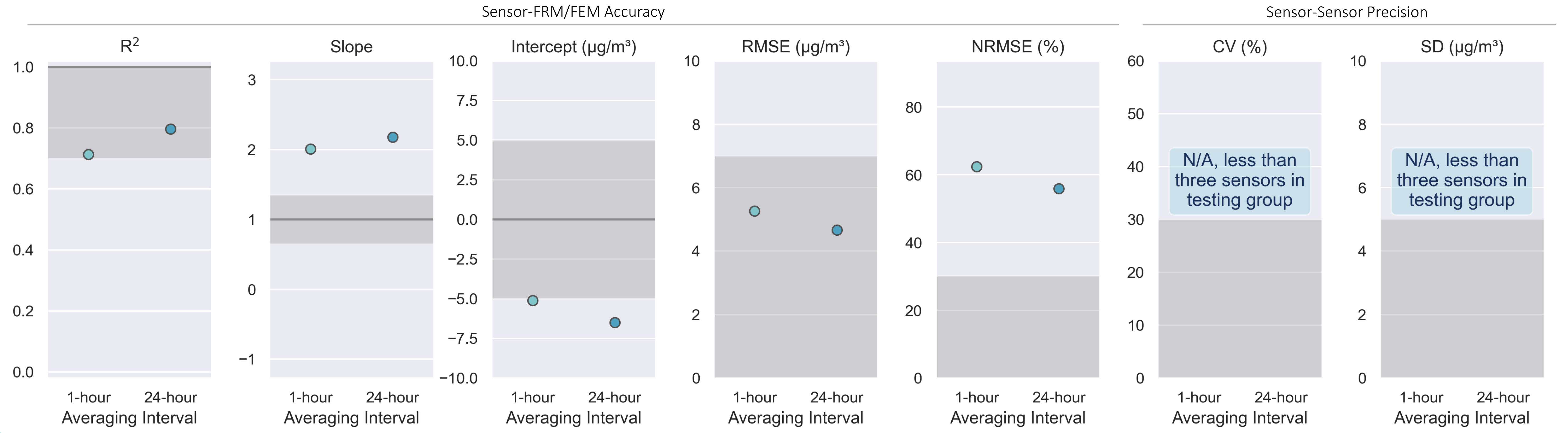

Meteorological Conditions During Deployment

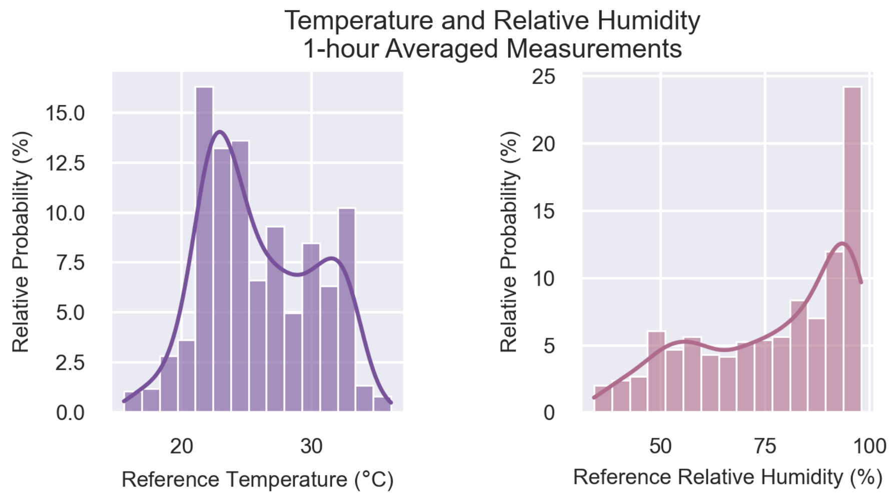

Number of 24-hr periods outside sensor manufacture-listed temperature operational range (-10 to 55 °C)	0
Number of 24-hr periods outside sensor manufacture-listed relative humidity operational range (10 to 98%)	0

Meteorological Influence

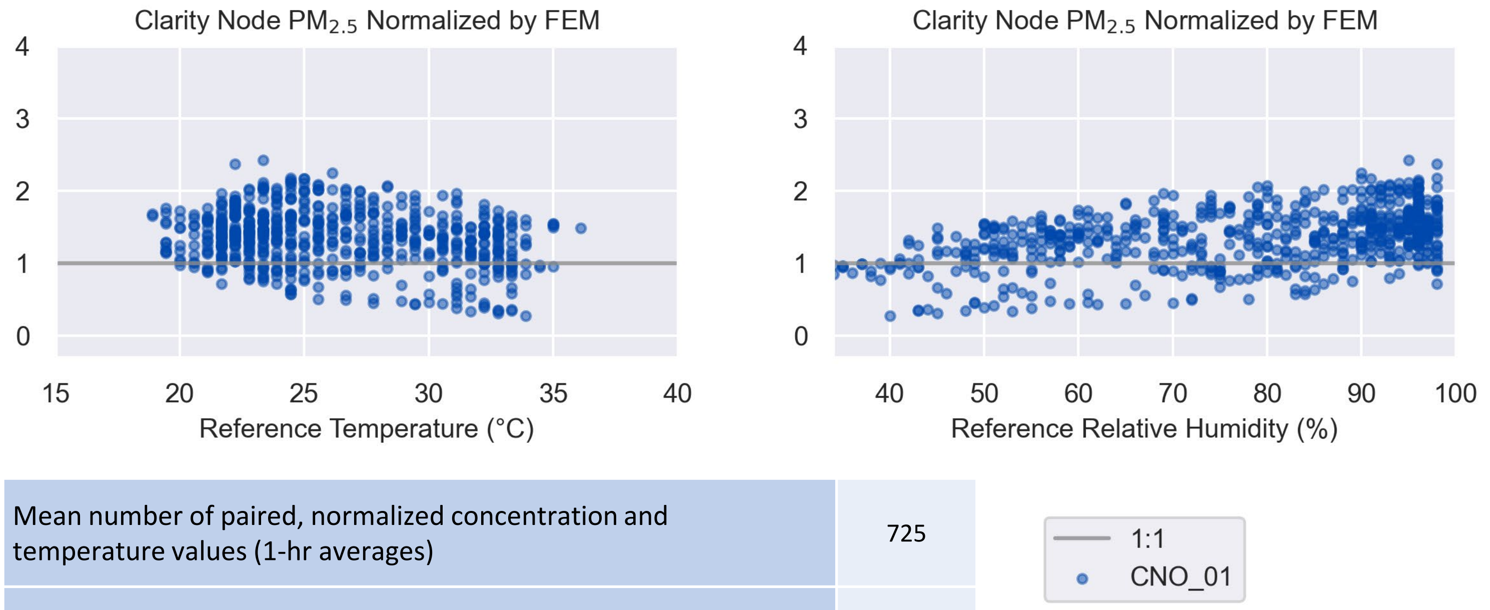

Mean number of paired, normalized concentration and temperature values (1-hr averages)	725
Mean number of paired, normalized concentration and relative humidity values (1-hr averages)	725

Testing Report - $PM_{2.5}$ Base Testing

Clarity Node

This report reflects out-of-the-box performance

Initial Base Testing - Decatur, GA

U.S. Environmental Protection Agency

Office of Research and Development

PI: Clements.Andrea@epa.gov

919-541-1363

August 2019

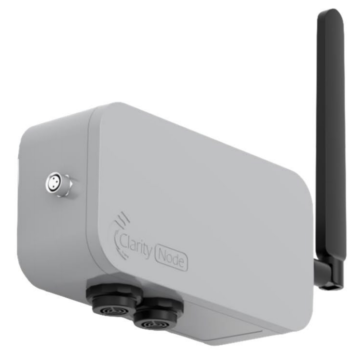

Image Attribution: Clarity Movement Co. (Berkeley, CA)

Tabular Statistics

Sensor-FRM/FEM Correlation

	Bias and Linearity						Data Quality			
	R^2		Slope		Intercept (µg/m³)		Uptime (%)		Number of paired sensor and FRM/FEM concentration values	
	1-Hour ●	24-Hour ●	1-Hour ○	24-Hour ○	1-Hour ○	24-Hour ○	1-Hour ●	24-Hour ●	1-Hour	24-Hour
Metric Target Range	≥ 0.70	≥ 0.70	1.0 ± 0.35	1.0 ± 0.35	-5 ≤ b ≤ 5	-5 ≤ b ≤ 5	75%*	75%*	-	-
Sensor CNO_01	0.71	0.80	2.01	2.18	-5.12	-6.50	100	96	642	26

	Error			
	RMSE (µg/m³)		NRMSE (%)	
	1-Hour ★	24-Hour ★	1-Hour ☆	24-Hour ☆
Metric Target Range	≤ 7.0	≤ 7.0	≤ 30.0	≤ 30.0
Deployment Value	5.3	4.7	62.4	55.9

Device-specific metrics (computed for each sensor in evaluation)

○○○ Metric value for none of devices tested falls within the target range

●○○ Metric value for one of devices tested falls within the target range

●●○ Metric value for two of devices tested falls within the target range

●●● Metric value for three of devices tested falls within the target range

Single-valued metrics (computed via entire evaluation dataset)

☆ Indicates that the metric value is not within the target range

★ Indicates that the metric value is within the target range

Sensor-Sensor Precision[1]

	Precision (between collocated sensors)				Data Quality	
	CV (%)		SD (µg/m³)		Number of concurrent sensor concentration pairs	
	1-Hour ☆	24-Hour ☆	1-Hour ☆	24-Hour ☆	1-Hour	24-Hour
Metric Target Range	≤ 30.0	≤ 30.0	≤ 5.0	≤ 5.0	-	-
Deployment Value	-	-	-	-	-	-

[1]Precision statistics are computed for evaluations with at least three collocated sensor units. Metric values are left blank for evaluations with two or fewer sensor units.

*This value is only a recommendation for ensuring data quality and is not included in the list of target values discussed in Section 4 of the Performance Testing Protocols, Metrics, and Target Values for Fine Particulate Matter Air Sensors document.

Testing Report - $PM_{2.5}$ Base Testing

Clarity Node

This report reflects out-of-the-box performance

Initial Base Testing - Decatur, GA
U.S. Environmental Protection Agency
Office of Research and Development
PI: Clements.Andrea@epa.gov
919-541-1363
August 2019

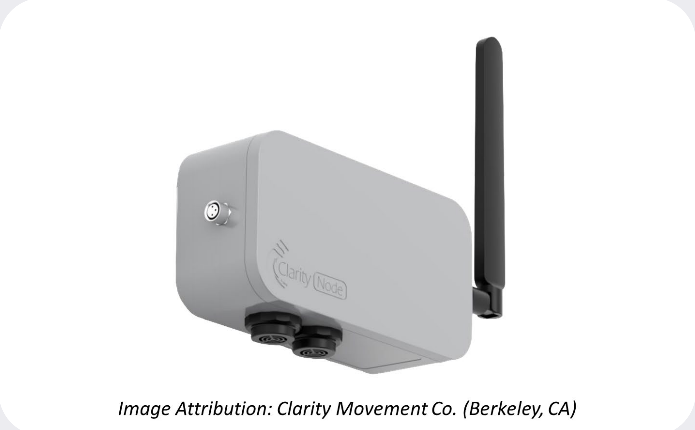
Image Attribution: Clarity Movement Co. (Berkeley, CA)

Sensor-FRM/FEM Scatter Plots

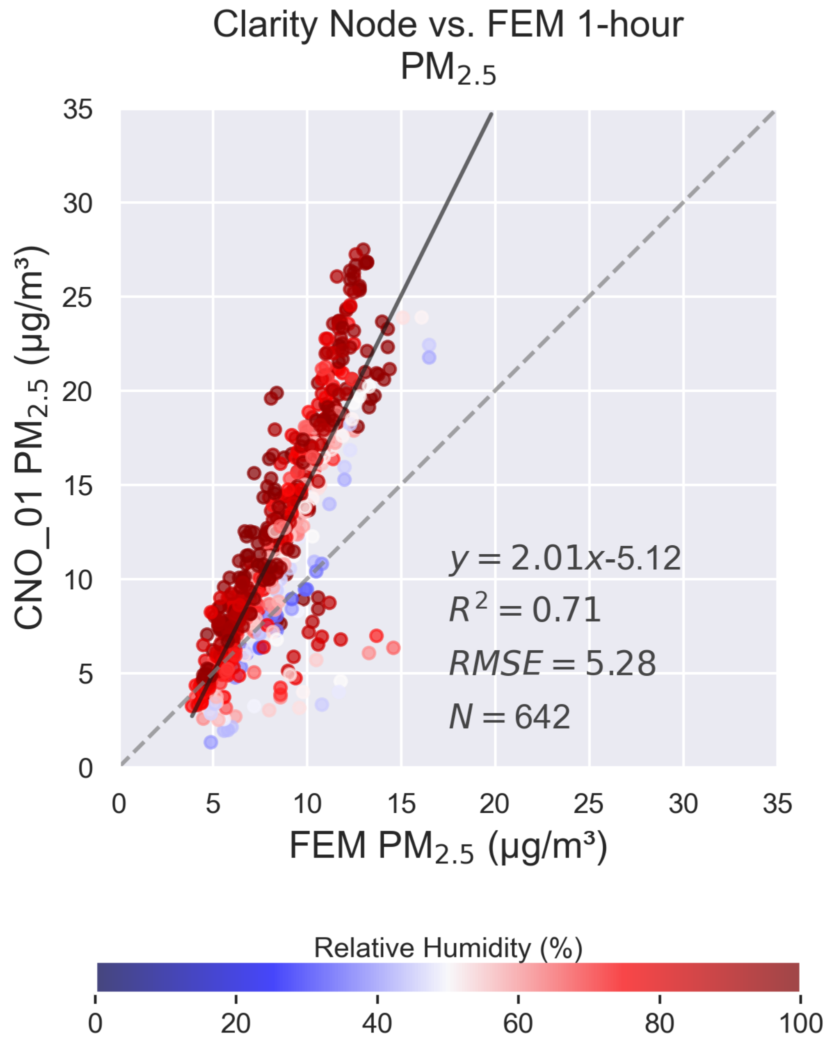

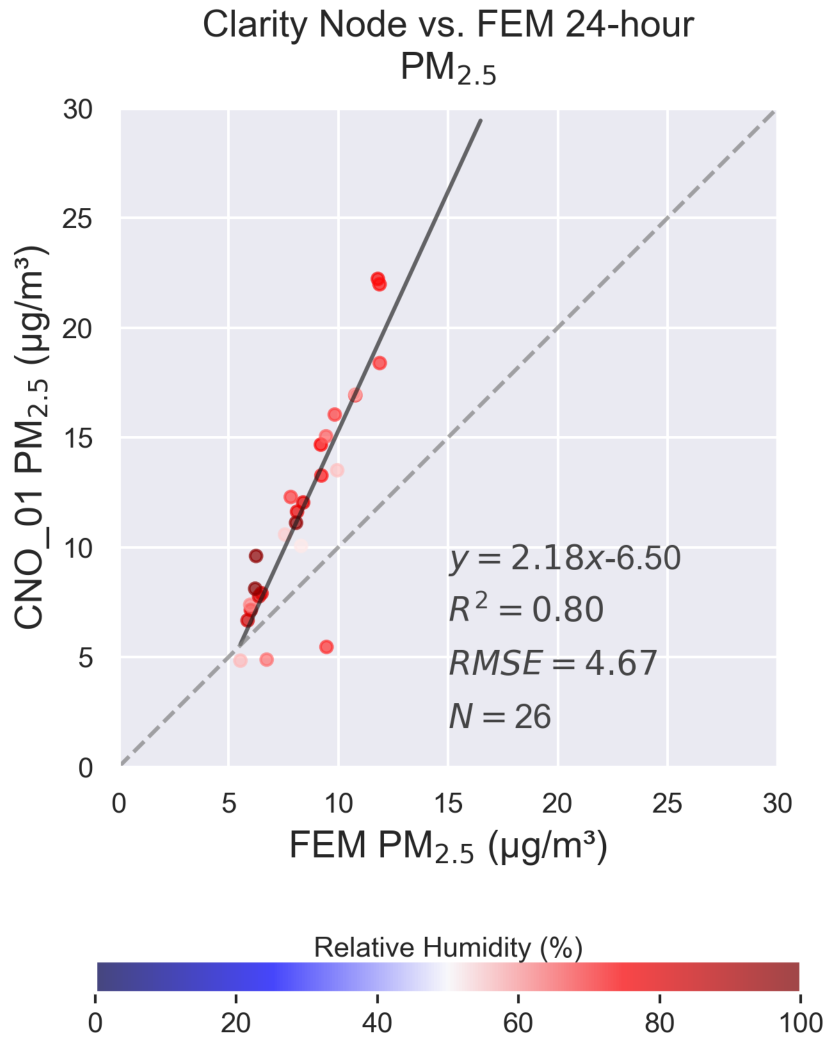

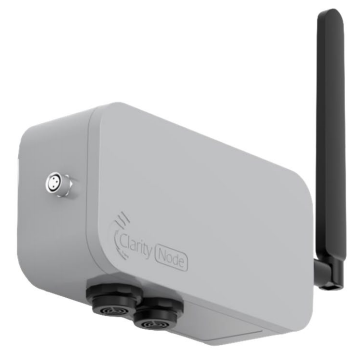

Image Attribution: Clarity Movement Co. (Berkeley, CA)

Supplemental Information

Abbreviations used in Supplemental Information

FRM	Federal Reference Method
FEM	Federal Equivalent Method
SOP	Standard Operating Procedure
QAPP	Quality Assurance Project Plan
QC	Quality Control

Supplemental Documentation	Attached	Description & URL or file path to documentation
Field observations and sensor data flags	☑	See GA-CNO-Page 6 of this testing report
Maintenance logs	☐	No logs recorded during testing
Standard operating procedure(s)	☐	U.S. EPA Office Of Research and Development SOP available upon request
Photos of equipment setup and testing	☑	See GA-CNO-Page 5 of this testing report
Product specifications sheet(s)	☐	N/A
Product manual(s)	☐	N/A
Data storage and transmission method	☑	See GA-CNO-Page 6 of this testing report
Data correction approach	☑	See GA-CNO-Page 6 of this testing report
Issues encountered	☑	See GA-CNO-Page 6 of this testing report
Data analysis/correction scripts and version	☑	Averaging and processing of data, calculation of performance metrics, and generation of figures and other supplementary material for analysis were obtained using Python 3.9.7 with the packages sensortoolkit v0.8.3b2, pandas 1.3.5, NumPy 1.21.2, Matplotlib 3.5.0, statsmodels 0.13.0, and seaborn 0.11.2. All packages are available from the Python Package Index (PyPI) at https://pypi.org. The integrated development environment (IDE) Spyder 5.1.5 was used for scripting and data visualization. Version control for the Python base, packages, and IDE were all managed by conda 4.11.0.
Air Monitoring Station QAPP	☐	U.S. EPA Office Of Research and Development QAPP available upon request
Summary of FRM/FEM monitor QC checks	☑	See GA-CNO-Page 7 of this testing report
Manufacturer website for FRM/FEM monitor	☑	Teledyne API: T640/T640X Product website
FRM/FEM monitor manual	☑	Teledyne API: T640/T640X Product Manual
FRM/FEM monitor specifications sheet(s)	☑	Teledyne API: T640/T640X Specification Sheet
Other documents	☐	

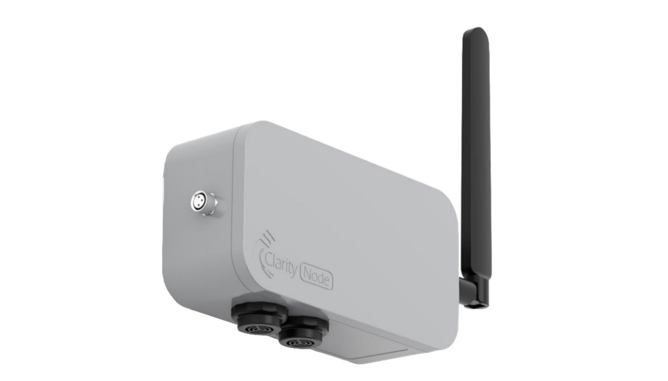

Image Attribution: Clarity Movement Co. (Berkeley, CA)

Supplemental Information: Photos of Testing Site and Equipment Setup

Site Description:
The South DeKalb monitoring station was initially established as an O3 site in 1974 and is located in the Atlanta area to the southeast of the city. More measurements were added to the site in the 1990's and 2000's. This site is currently a multipollutant site that is designated as a National Core (Ncore), Photochemical Assessment Monitoring Station (PAMS), and National Air Toxics Trends Station (NATTS) site for the state of Georgia. Measurements at South DeKalb include continuous analyzers for ozone, carbon monoxide, nitrogen dioxide, sulfur dioxide, and particulate matter (PM) with an aerodynamic diameter less than 2.5 microns (PM2.5) and 10 microns (PM10). Volatile organic compounds (VOCs) are also measured during summer periods via AutoGC as well as year-round via canister sampling. Local meteorology is also measured at the site, including wind speed and direction, temperature, relative humidity, pressure, and precipitation.

Figure 1: Clarity Node sensor (indicated by red arrow) attached to metal railing atop the sampling shelter at the monitoring site.

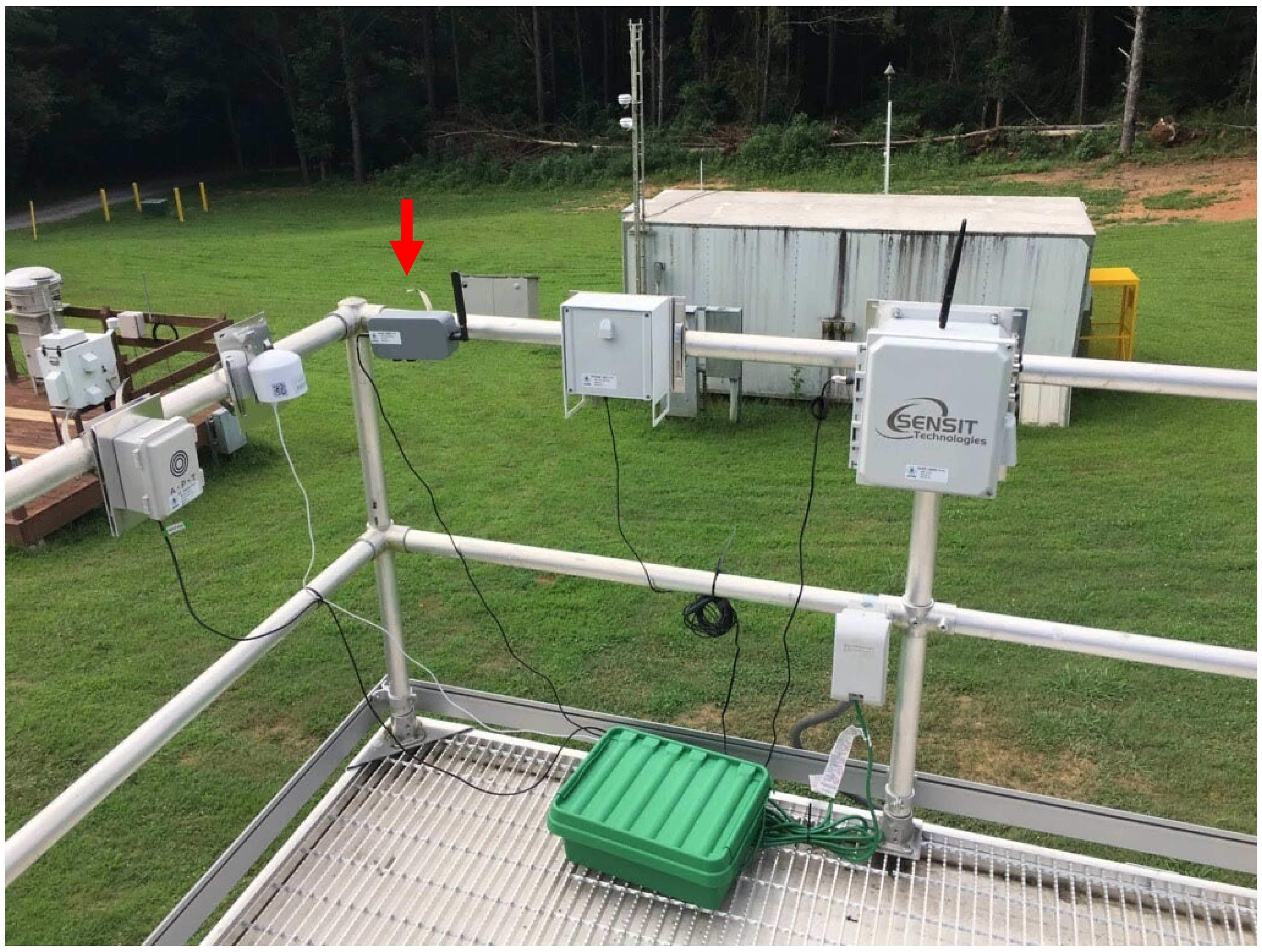

Figure 2: South Dekalb Monitoring Station

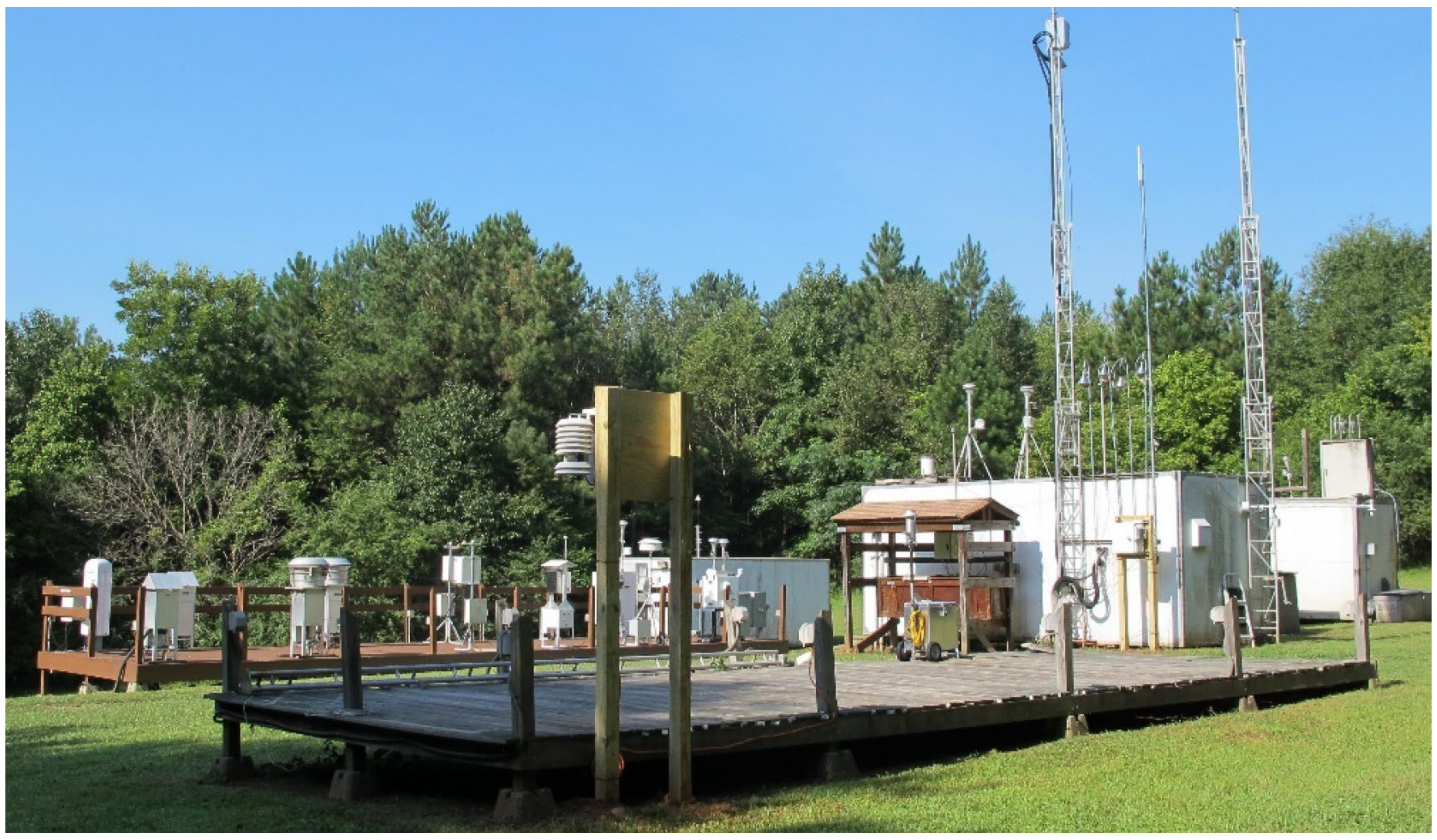

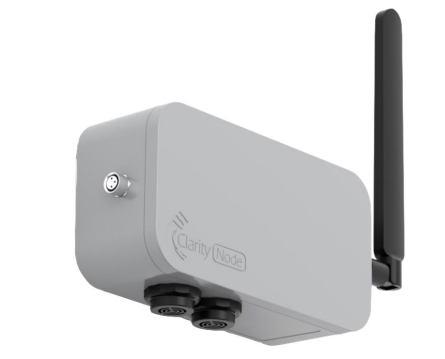

Supplemental Information: Data Storage, Correction Approach, and Issues Encountered

Data Storage and Transmission Method

The Clarity Node transmits data via cellular SIM card. Weekly raw data collections were performed over Wi-Fi using the Clarity dashboard (*last accessed 5/11/22*). The observed sampling interval fluctuated and is further discussed under pre-deployment observations below. On average, the Node models sampled every 2-3 minutes, and the Node-S sampled every 15-17 minutes.

Data Correction Approach

This evaluation report reflects “out-of-the-box” performance of the Clarity Node. The manufacturer provides a dashboard feature for calibrating sensor data against collocated reference monitors. Calibrated data were NOT used for this evaluation. Prospective consumers may get better performance from this device if they utilize this feature.

After acquisition, the raw data was processed using the *sensortoolkit* python code library (v0.8.3b2). A continuous data set at the recorded sampling frequency was written to a .csv file. 1-hour and 24-hour averaged data sets were generated using a 75% completeness threshold and saved as separate .csv files. Due to the variable sampling interval observed for Clarity Node units, 75% completeness was determined against the number of data points that would be recorded within 1-hour/24-hour periods if the sensor were to log measurements at the most commonly recorded sampling interval (i.e., the mode of recorded intervals). Outliers were NOT removed from data sets in order to assess “out-of-the-box” sensor performance.

Issues Encountered

Pre-deployment observations

- *Unsteady sampling interval:* Clarity Node sensors were received by EPA on May 28, 2019. A lab bench-top evaluation was performed from May 29, 2019 – May 31, 2019. During bench-top testing, it was observed that the sampling intervals noted by Clarity (5 minutes for non-solar Node, 15 minutes for the solar Node-S) were not reflective of recorded intervals. Communication with the vendor concluded that the sampling interval will fluctuate depending on the settings, battery charge, and strength of the cellular signal and that this sampling interval cannot be adjusted by the user at present. On average, the Node models sampled every 2-3 minutes, and the Node-S sampled every 15-17 minutes.

Field observations and sensor data flags

The Clarity Node unit was deployed at the South Dekalb monitoring site on 8/1/2019. Due to a miscommunication, the sensor unit was temporarily removed from the site on 8/28/2019 and was reinstalled in the previous configuration on 9/11/2019.

During the testing period, the Node operated nominally and did not require replacement or repair as a result of performance issues.

The following table contains data flags describing events that were encountered during the testing period.

Start Time (UTC)	End Time (UTC)	Sensor Serial ID	Parameters Impacted	Flag
2019-08-28 13:13:00+00:00	2019-08-28 13:13:00+00:00	CNO_01	ALL	0-Sensor retrieval

Description of Data Flags

AQS

The U.S. EPA's Air Quality System (AQS) is the Agency's primary ambient air monitoring data archive. A comprehensive list of data flags that are recorded alongside AQS data sets, referred to by U.S. EPA as 'qualifiers', can be found at the following link: https://aqs.epa.gov/aqsweb/documents/codetables/qualifiers.html

Invalidation of reference data: AQS qualifiers are organized by qualifier type, which indicates whether data logged alongside qualifier flags should be invalidated (set null). Qualifiers with type "Null Data Qualifier" are invalidated, and includes data logged during periods that coincide with QC checks (e.g., "BF-Precision/Zero/Span", "BJ- Operator Error", "BL - QA Audit", "AZ - QC Audit") among other events such as power outages. Data logged alongside qualifiers with type "Quality Assurance Qualifiers" are not invalidated and are included in this analysis (e.g., concentrations less than the federal MDL for the reference monitor "MD – Value less than MDL", QA reviewed values "Validated Value").

Data Flags Recorded During Testing

FRM/FEM Monitor	Timestamp (UTC)	Flag
Teledyne API T640X (Acquired via AQS)	2019-08-14 12:00:00+0000	AZ - QC Audit
	2019-08-19 13:00:00+0000	BL - QA Audit
	2019-08-28 13:00:00+0000	AZ - QC Audit

Meteorological Instrument	Timestamp (UTC)	Flag
RM Young 41375VC Temperature Monitor (Data acquired via AQS)	2019-08-08 14:00:00+0000 to 2019-08-08 15:00:00+0000	BL - QA Audit
[Make Model] Relative Humidity Monitor (Data acquired via AQS)	2019-08-08 14:00:00+0000 to 2019-08-08 15:00:00+0000	BL - QA Audit

Testing Report - $PM_{2.5}$ Base Testing

PurpleAir PA-II-SD

This report reflects out-of-the-box performance

Initial Base Testing - Decatur, GA
U.S. Environmental Protection Agency
Office of Research and Development
PI: Clements.Andrea@epa.gov
919-541-1363
September 2019—October 2019

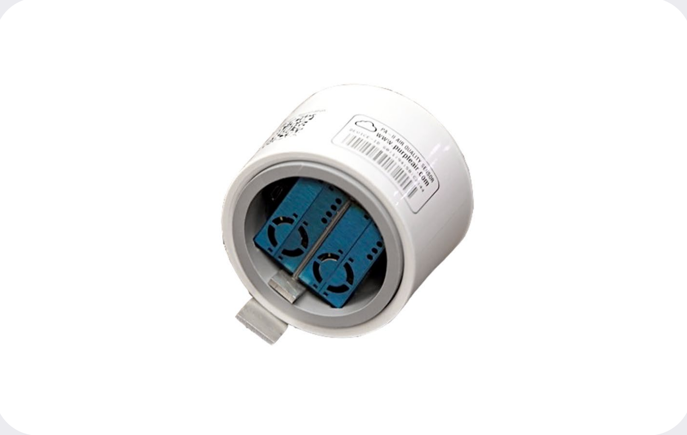

Deployment Details

Testing Organization and Site Information	
Testing organization (Name, Organization type, Contact website)	U.S. Environmental Protection Agency - Office of Research and Development<br>Federal Government<br>Air Sensor Toolbox | U.S. EPA Website
Testing location (City, State, Latitude and Longitude)	South Dekalb<br>Decatur, GA<br>33.6877, -84.2905
AQS site ID	13 - 089 - 0002
Sampling timeframe (MM-DD-YY)	09-12-19 to 10-12-19
Sensor data source	Onboard MicroSD card
Reference data source	AQS API download

Sensor Information			
Manufacturer, model	PurpleAir PA-II-SD		
Device firmware version	v4.02		
Sampling time interval	2-minutes		
Sensor serial numbers	PAR_01		
Issues encountered during deployment?	☑	See GA-PAR-Page 6 of this testing report	

FRM/FEM Information	
Manufacturer, model, designation	Teledyne Advanced Pollution Instrumentation T640X FEM
Sampling time interval	1-hour averaging
Date of calibration	As required by 40 CFR Part 58 and the Ambient Air Monitoring Plan maintained by GA DNR
Date of flowrate verification check	Monthly as required by 40 CFR Part 58 Appendix A
Description, date(s) of maintenance activities	See GA-PAR-Page 7 of this testing report

Time Series Plots

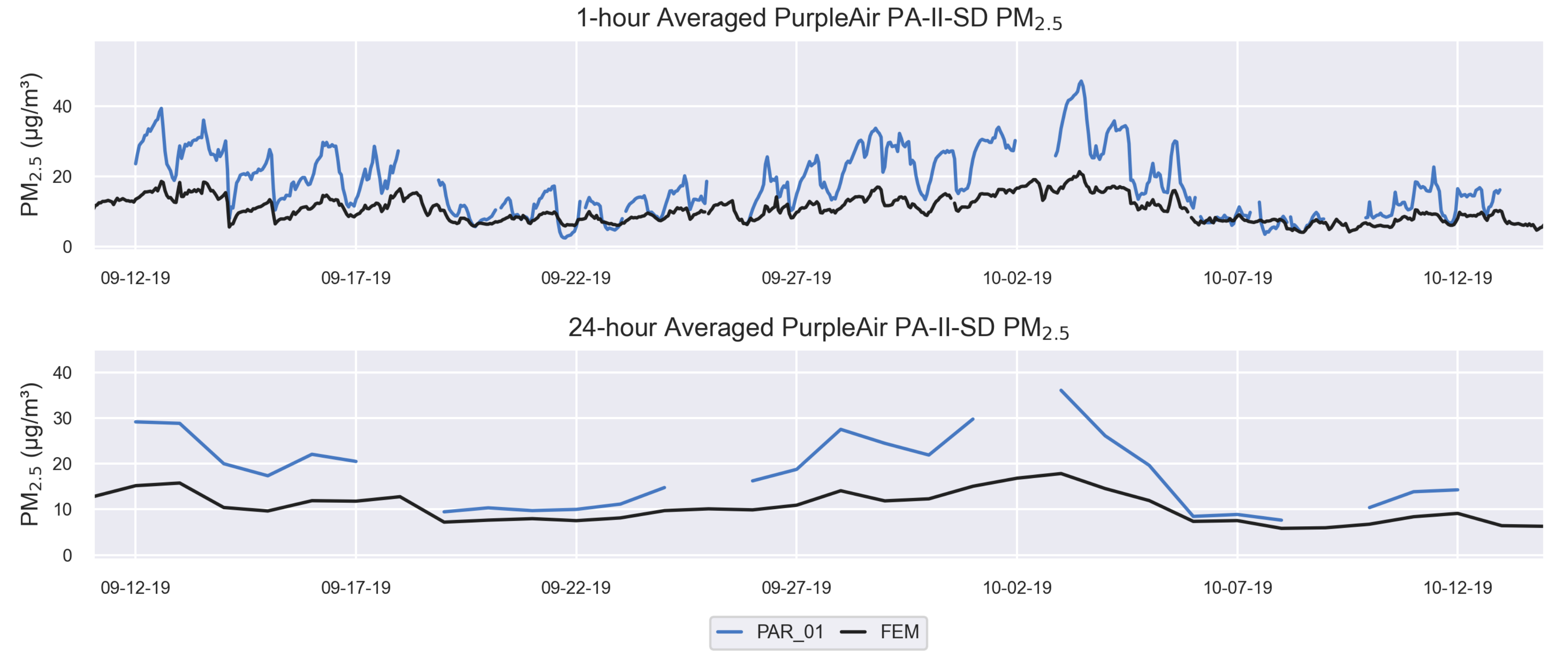

Scatter Plots: Comparison to FRM/FEM

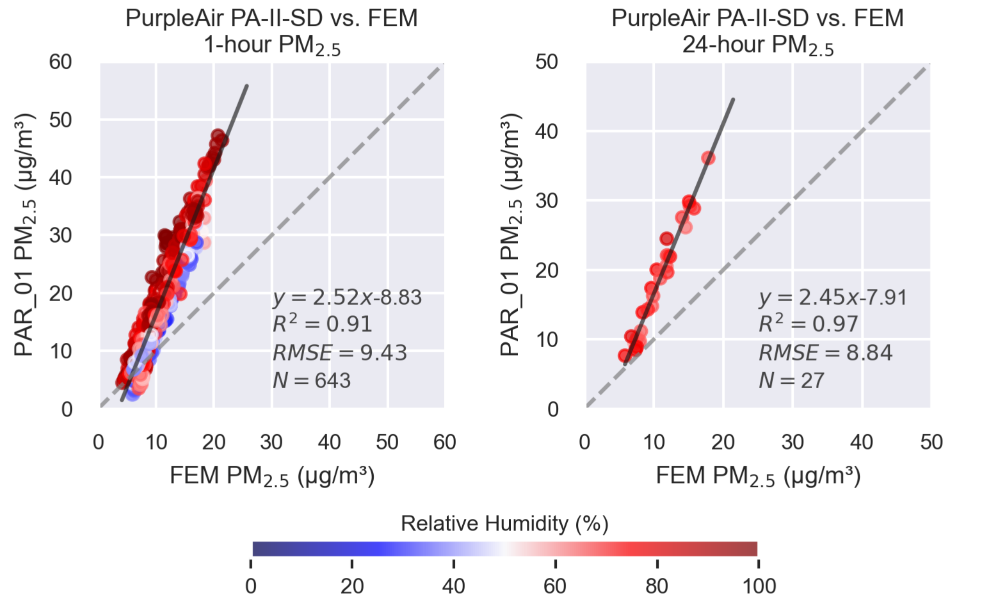

Range of FRM/FEM concentrations over duration of base test (µg/m³)	4.1-21.4 (1-hr), 5.8-17.9 (24-hr)
Number of 24-hr periods in FRM/FEM monitor measurements with a goal concentration ≥ 25 µg/m³	0

Performance Metrics

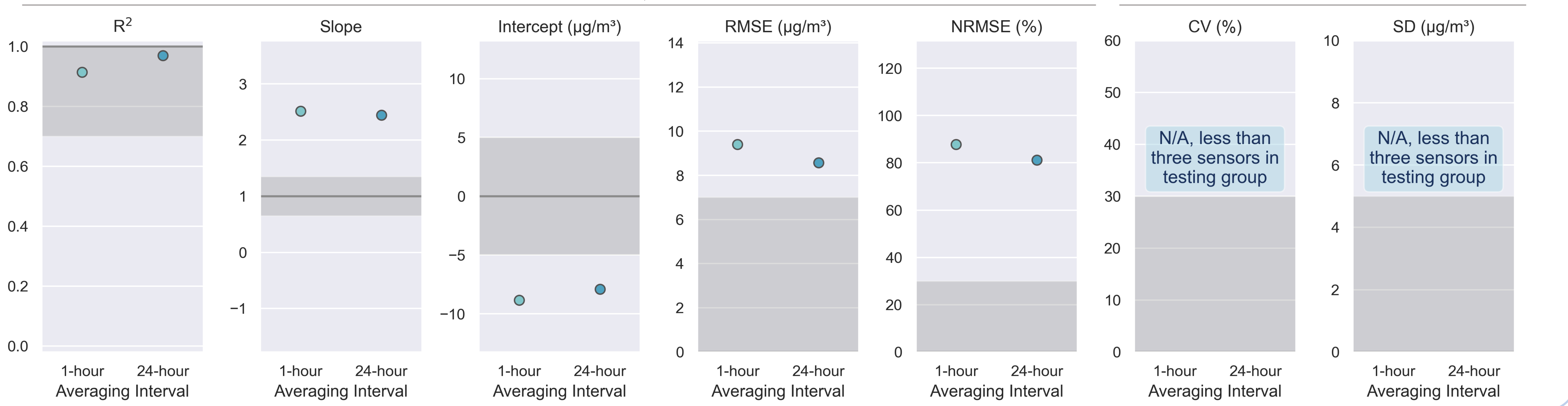

Meteorological Conditions During Deployment

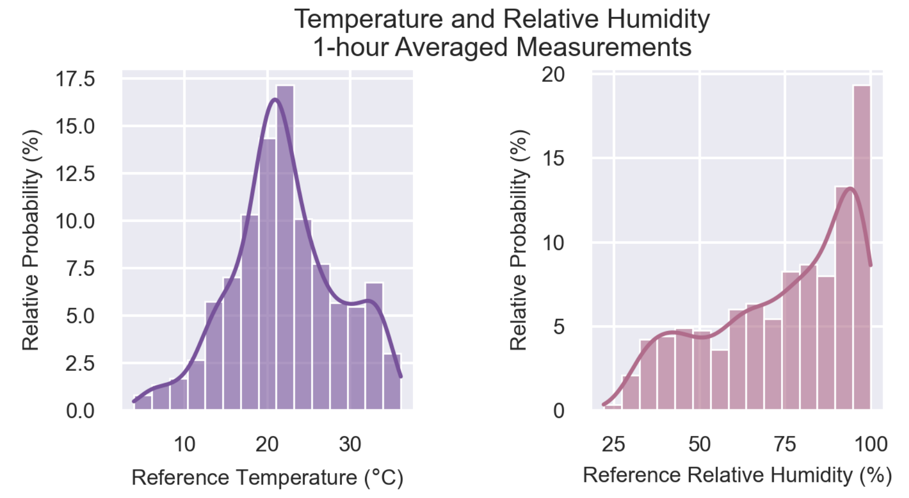

Number of 24-hr periods outside sensor manufacturer-listed temperature operational range (no operational range specified)	-
Number of 24-hr periods outside sensor manufacturer-listed relative humidity operational range (no operational range specified)	-

Meteorological Influence

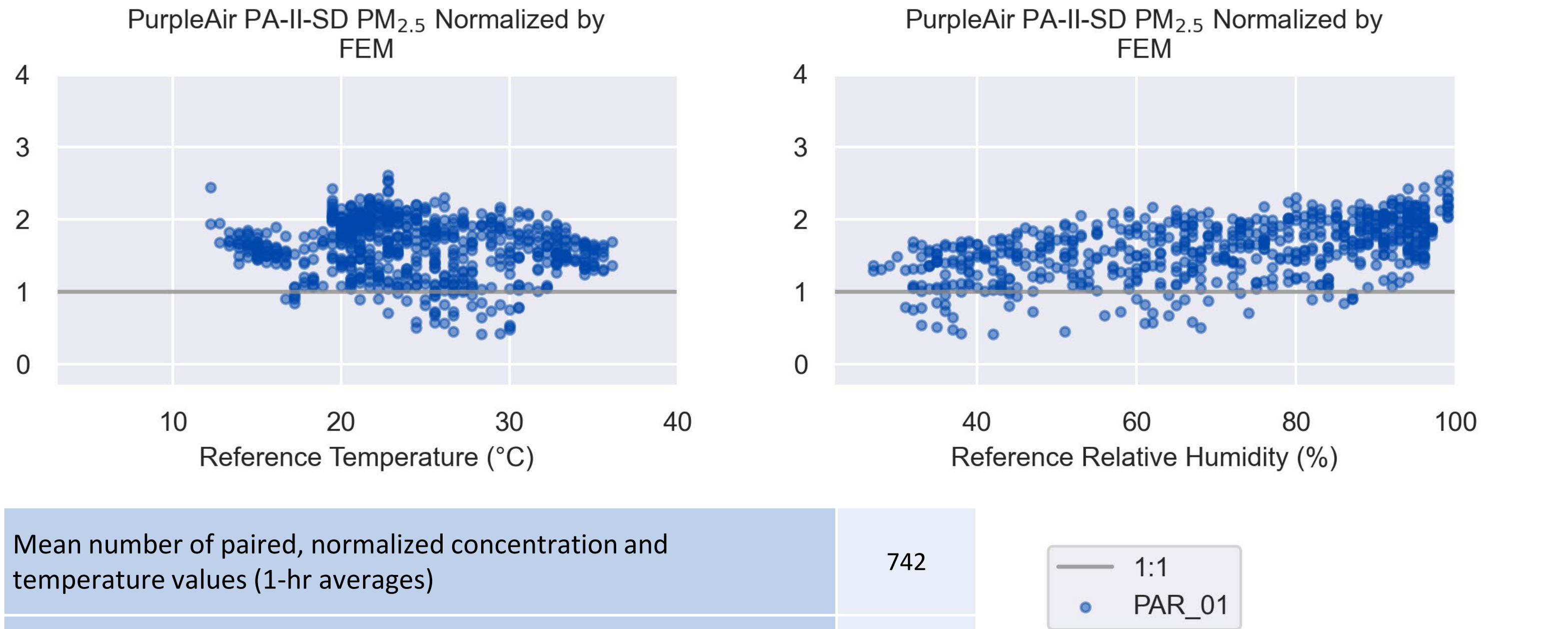

Mean number of paired, normalized concentration and temperature values (1-hr averages)	742
Mean number of paired, normalized concentration and relative humidity values (1-hr averages)	742

Testing Report - $PM_{2.5}$ Base Testing

PurpleAir PA-II-SD

This report reflects out-of-the-box performance

Initial Base Testing - Decatur, GA

U.S. Environmental Protection Agency
Office of Research and Development
PI: Clements.Andrea@epa.gov
919-541-1363
September 2019—October 2019

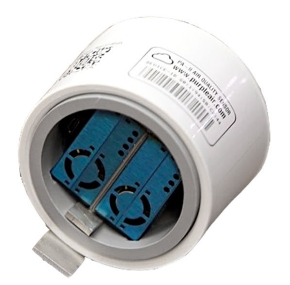

Tabular Statistics

Sensor-FRM/FEM Correlation

	Bias and Linearity						Data Quality			
	R^2		Slope		Intercept (µg/m³)		Uptime (%)		Number of paired sensor and FRM/FEM concentration values	
	1-Hour ●	24-Hour ●	1-Hour ○	24-Hour ○	1-Hour ○	24-Hour ○	1-Hour ●	24-Hour ●	1-Hour	24-Hour
Metric Target Range	≥ 0.70	≥ 0.70	1.0 ± 0.35	1.0 ± 0.35	-5 ≤ b ≤ 5	-5 ≤ b ≤ 5	75%*	75%*	-	-
Sensor PAR_01	0.91	0.97	2.52	2.45	-8.83	-7.91	89	87	643	27

	Error			
	RMSE (µg/m³)		NRMSE (%)	
	1-Hour ☆	24-Hour ☆	1-Hour ☆	24-Hour ☆
Metric Target Range	≤ 7.0	≤ 7.0	≤ 30.0	≤ 30.0
Deployment Value	9.4	8.6	87.8	81.2

Device-specific metrics (computed for each sensor in evaluation)
○○○ Metric value for none of devices tested falls within the target range
●○○ Metric value for one of devices tested falls within the target range
●●○ Metric value for two of devices tested falls within the target range
●●● Metric value for three of devices tested falls within the target range

Single-valued metrics (computed via entire evaluation dataset)
☆ Indicates that the metric value is not within the target range
★ Indicates that the metric value is within the target range

Sensor-Sensor Precision[1]

	Precision (between collocated sensors)				Data Quality	
	CV (%)		SD (µg/m³)		Number of concurrent sensor concentration pairs	
	1-Hour ☆	24-Hour ☆	1-Hour ☆	24-Hour ☆	1-Hour	24-Hour
Metric Target Range	≤ 30.0	≤ 30.0	≤ 5.0	≤ 5.0	-	-
Deployment Value	-	-	-	-	-	-

[1]Precision statistics are computed for evaluations with at least three collocated sensor units. Metric values are left blank for evaluations with two or fewer sensor units.

*This value is only a recommendation for ensuring data quality and is not included in the list of target values discussed in Section 4 of the Performance Testing Protocols, Metrics, and Target Values for Fine Particulate Matter Air Sensors document.

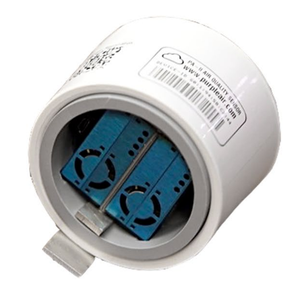

Sensor-FRM/FEM Scatter Plots

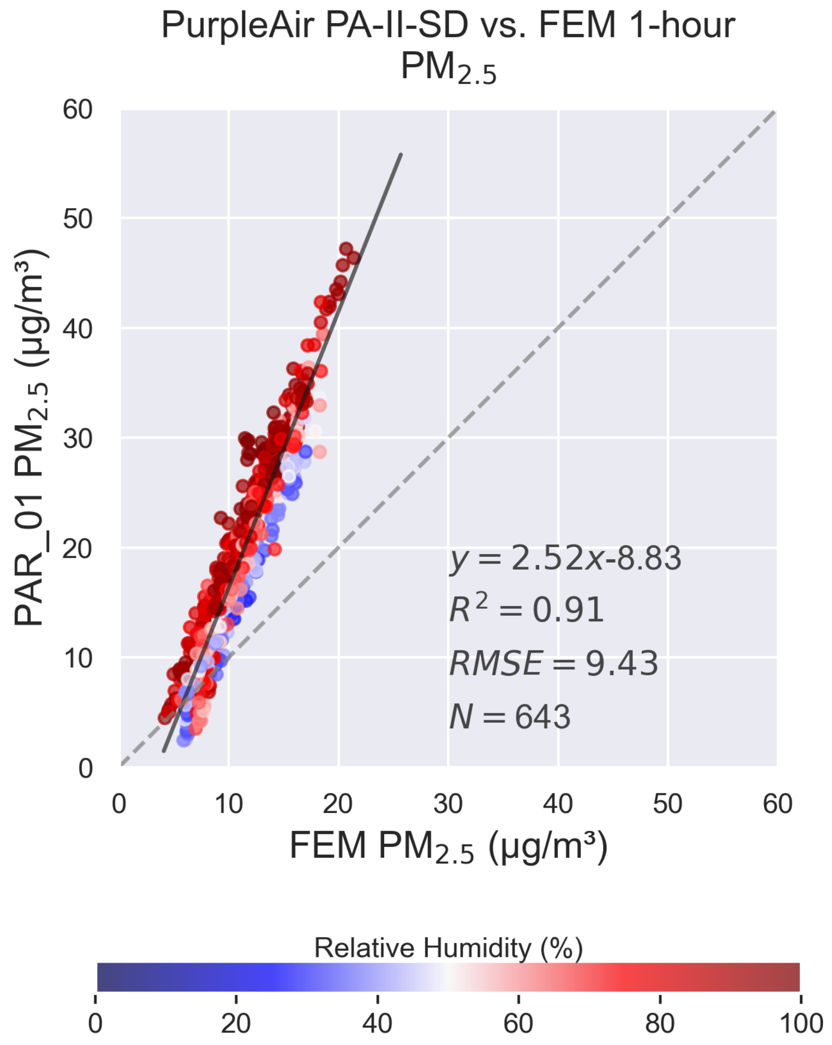

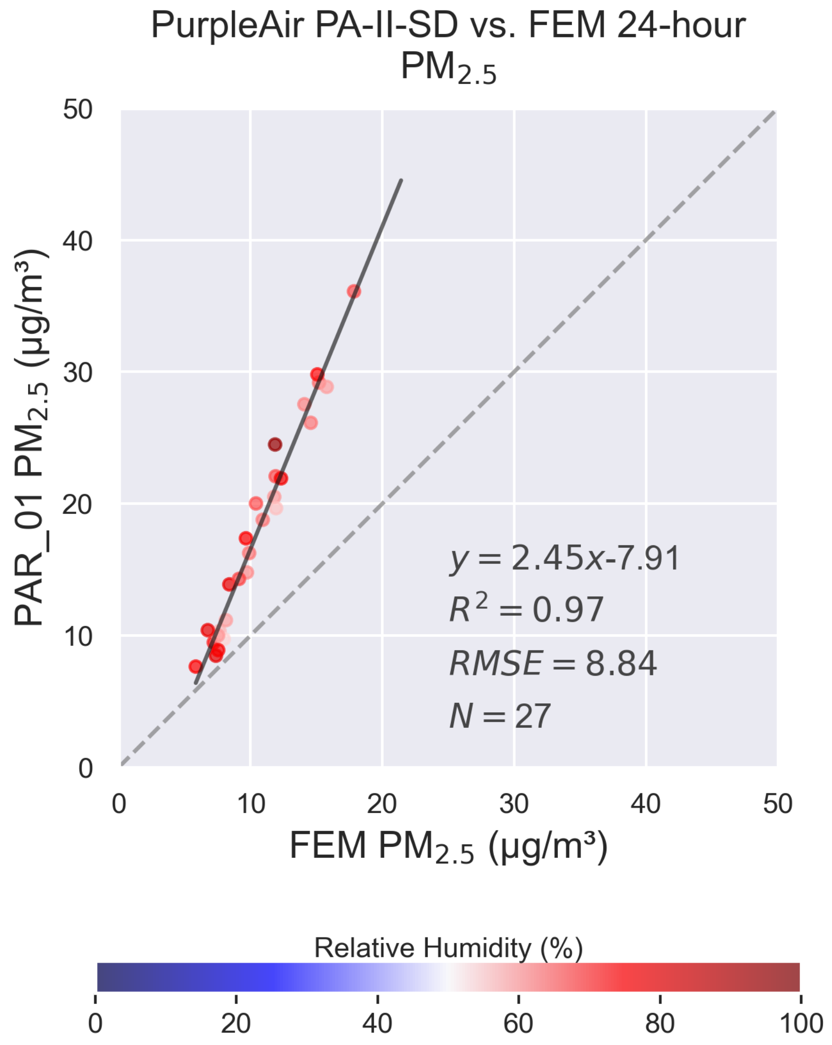

U.S. Environmental Protection Agency
Office of Research and Development
PI: Clements.Andrea@epa.gov
919-541-1363

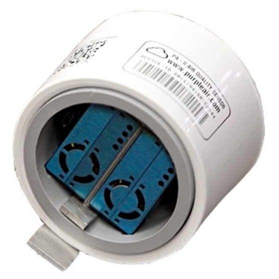

Supplemental Information

Abbreviations used in Supplemental Information

FRM	Federal Reference Method
FEM	Federal Equivalent Method
SOP	Standard Operating Procedure
QAPP	Quality Assurance Project Plan
QC	Quality Control

Supplemental Documentation	Attached	Description & URL or file path to documentation
Field observations and sensor data flags	☑	See GA-PAR-Page 6 of this testing report
Maintenance logs	☐	No logs recorded during testing
Standard operating procedure(s)	☐	U.S. EPA Office Of Research and Development SOP available upon request
Photos of equipment setup and testing	☑	See GA-PAR-Page 5 of this testing report
Product specifications sheet(s)	☑	PurpleAir: PA-II-SD Product website
Product manual(s)	☐	N/A
Data storage and transmission method	☑	See GA-PAR-Page 6 of this testing report
Data correction approach	☑	See GA-PAR-Page 6 of this testing report
Issues encountered	☑	See GA-PAR-Page 6 of this testing report
Data analysis/correction scripts and version	☑	Averaging and processing of data, calculation of performance metrics, and generation of figures and other supplementary material for analysis were obtained using Python 3.9.7 with the packages sensortoolkit v0.8.3b2, pandas 1.3.5, NumPy 1.21.2, Matplotlib 3.5.0, statsmodels 0.13.0, and seaborn 0.11.2. All packages are available from the Python Package Index (PyPI) at https://pypi.org. The integrated development environment (IDE) Spyder 5.1.5 was used for scripting and data visualization. Version control for the Python base, packages, and IDE were all managed by conda 4.11.0.
Air Monitoring Station QAPP	☐	U.S. EPA Office Of Research and Development QAPP available upon request
Summary of FRM/FEM monitor QC checks	☑	See GA-PAR-Page 7 of this testing report
Manufacturer website for FRM/FEM monitor	☑	Teledyne API: T640/T640X Product website
FRM/FEM monitor manual	☑	Teledyne API: T640/T640X Product Manual
FRM/FEM monitor specifications sheet(s)	☑	Teledyne API: T640/T640X Specification Sheet
Other documents	☐	

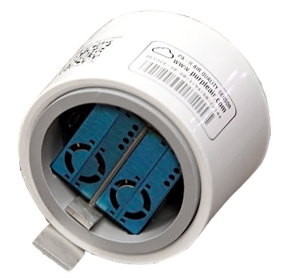

Supplemental Information: Photos of Testing Site and Equipment Setup

Site Description:
The South DeKalb monitoring station was initially established as an O3 site in 1974 and is located in the Atlanta area to the southeast of the city. More measurements were added to the site in the 1990's and 2000's. This site is currently a multipollutant site that is designated as a National Core (Ncore), Photochemical Assessment Monitoring Station (PAMS), and National Air Toxics Trends Station (NATTS) site for the state of Georgia. Measurements at South DeKalb include continuous analyzers for ozone, carbon monoxide, nitrogen dioxide, sulfur dioxide, and particulate matter (PM) with an aerodynamic diameter less than 2.5 microns (PM2.5) and 10 microns (PM10). Volatile organic compounds (VOCs) are also measured during summer periods via AutoGC as well as year-round via canister sampling. Local meteorology is also measured at the site, including wind speed and direction, temperature, relative humidity, pressure, and precipitation.

Figure 1: PurpleAir PA-II-SD sensor (indicated by red arrow) attached to metal railing atop the sampling shelter at the monitoring site.

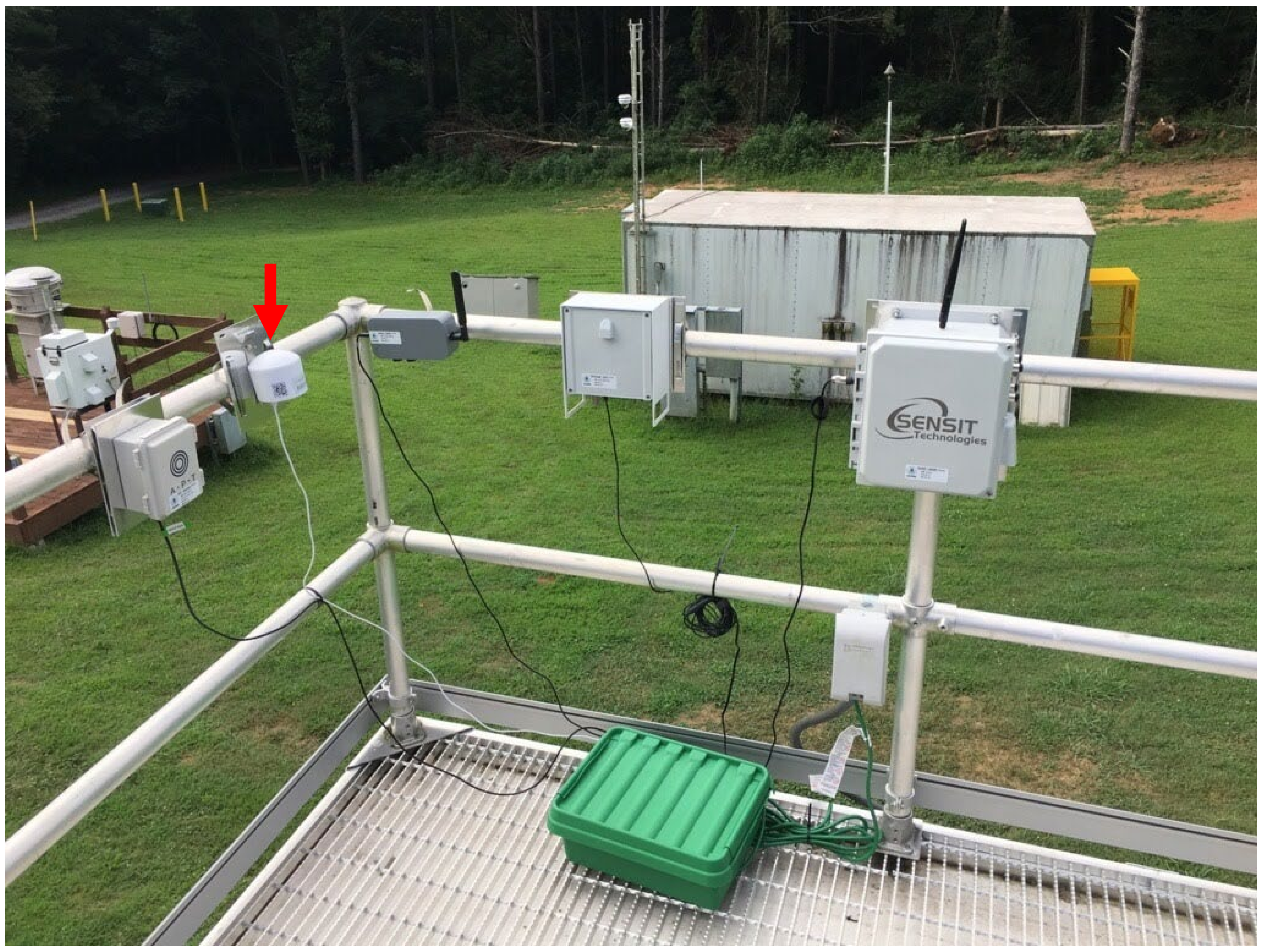

Figure 2: South Dekalb Monitoring Station

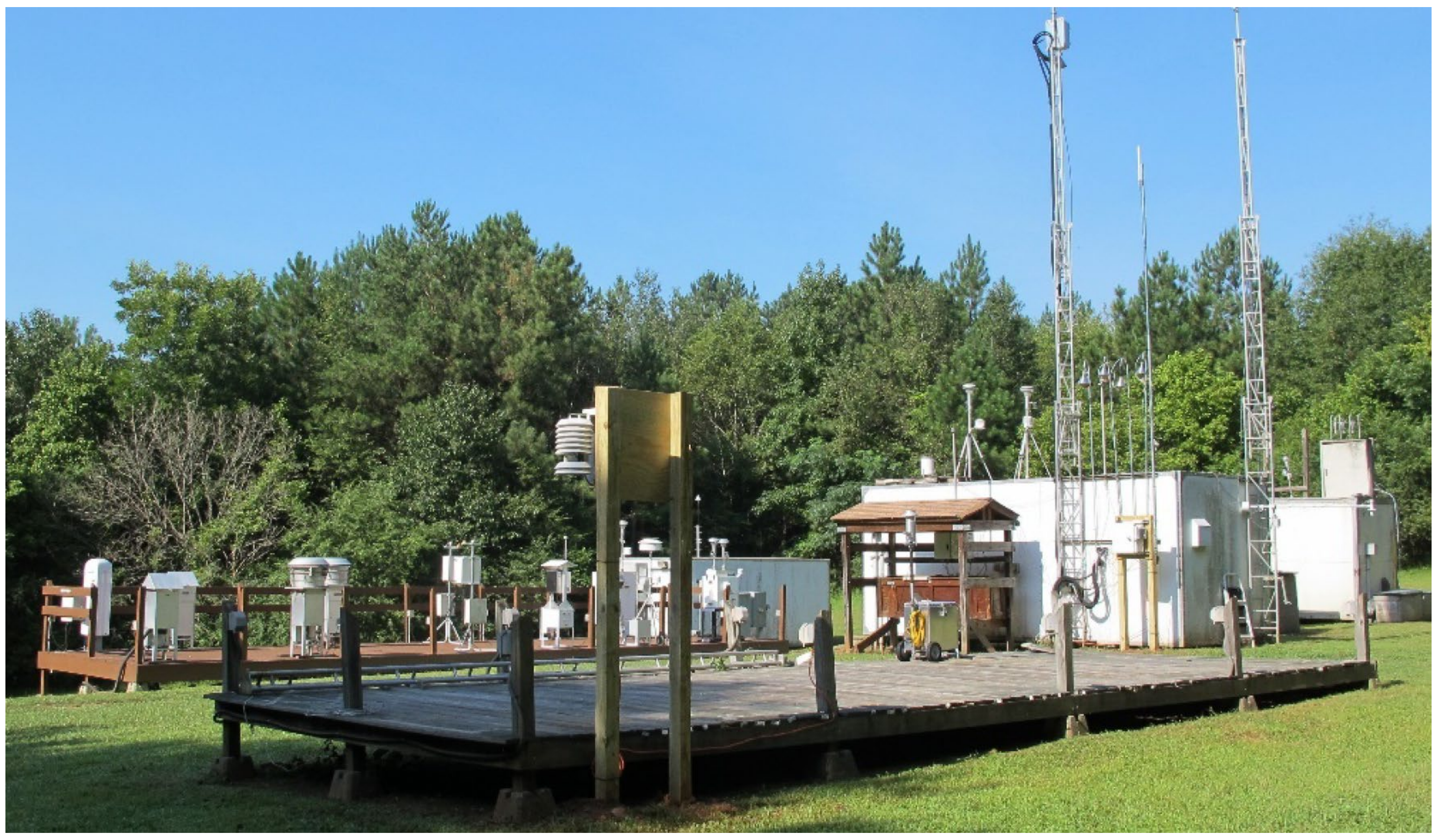

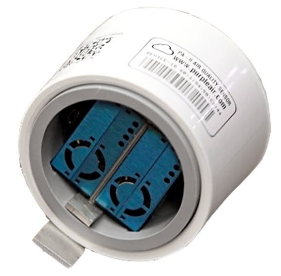

Data Storage and Transmission Method

The PurpleAir PA-II-SD records data at a 2-minute[1] sampling interval. Data is transmitted to the PurpleAir Cloud via Wi-Fi and stored on an internal MicroSD card. Due to the differing file structure between remote and direct download methods and the lag associated with cloud data download, weekly raw data were obtained primarily via onboard SD card. Each field site operator was provided two labeled MicroSD cards for sensor units that they used to swap out each week. Data from the collected card was then read and processed off-site.

Data Correction Approach

Data sets recorded by PurpleAir PA-II-SD contain two correction factors for PM concentrations, referred to internally as "CF=ATM" and "CF=1". $PM_{2.5}$ concentrations are recorded concurrently for each correction factor, and corresponding concentrations agree closely up to approximately 30 µg/m^3, above which one correction factor reflects concentrations roughly 1.5 times concentrations for the other correction factor. During a separate analysis by ORD, concentrations for the higher correction factor were determined to have slightly higher correlation with collocated reference measurements. As a result, data for the higher correction factor were used for this study. Prior to firmware version 5.00 (released on 11/30/2019), headers for concentrations corresponding to each correction factor were incorrectly labeled (i.e., CF=1 was labeled CF=ATM and vice versa). Testing indicated in this report was completed prior to this firmware update, and thus data sets acquired for PA-II-SD units had swapped $PM_{2.5}$ CF labels. Recorded PA-II-SD data sets indicate that the CF=ATM correction factor was the higher CF as labeled (following firmware update 5.00, this data channel corresponds to CF=1) and sensor data shown in this report correspond to this correction factor.

After acquisition, the raw data was processed using the *sensortoolkit* python code library (v0.8.3b2). A continuous data set at the recorded sampling frequency was written to a .csv file. 1-hour and 24-hour averaged data sets were generated using a 75% completeness threshold and saved as separate .csv files. Simultaneous measurement from the PurpleAir PA-II's dual PMS5003 sensors (labeled channels A and B) allows for identification of outliers resulting in large-scale disagreement between recorded values. The outlier removal method of Barkjohn et al. 2021[2] was applied to both 1-hour and 24-hour averaged A and B measurement pairs. This method includes thresholds for the absolute difference and the percent difference between hourly channel values. A-B channel averages were not included for hours where the absolute difference between channel values was greater than 5 µg/m^3 and the percent difference exceeded 70%. This outlier removal method is in use on the AirNow Fire and Smoke map and has been used for this evaluation since it requires no external information (e.g., data from the monitor) and no assumptions about typical PM2.5 concentrations (e.g., removing data over a certain threshold concentration).

Issues Encountered

Pre-deployment observations

- Prior to the testing period indicated in this report, the PurpleAir PA-II-SD unit was briefly collocated for a period of approximately one week at EPA's Ambient Monitoring Innovative Research Station (AIRS) in Research Triangle Park, NC. The unit was tested for proper operation, including powering on and data logging (either to the onboard SD card or via hotspot Wi-Fi connection to the PurpleAir Cloud), sampling, and acquisition of recorded data from either onboard memory or cloud storage. Units that did not pass this pre-deployment testing were returned to the manufacturer and constituted approximately 1 in 10 units tested for subsequent performance evaluation.

Field observations and sensor data flags

The PurpleAir PA-II-SD unit was deployed at the South Dekalb monitoring site on 8/1/2019. Due to a miscommunication, the sensor unit was temporarily removed from the site on 8/28/2019 and was reinstalled in the previous configuration on 9/11/2019. During the testing period, the PA-II-SD operated nominally and did not require replacement or repair.

During the testing period, no data flags logged by field technicians were recorded.

[1]As of version 4.02, updated across devices on 5/31/2019. Prior to this firmware version, devices recorded measurements at 80-second intervals
[2]Barkjohn, K. K., Gantt, B., and Clements, A. L.: Development and application of a United States-wide correction for PM2.5 data collected with the PurpleAir sensor, Atmos. Meas. Tech., 14, 4617–4637, https://doi.org/10.5194/amt-14-4617-2021, 2021.

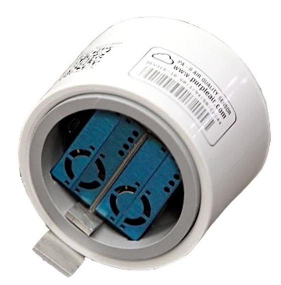

Supplemental Information: Description of FRM/FEM QC Checks and Data Flags

Description of Data Flags

AQS

The U.S. EPA's Air Quality System (AQS) is the Agency's primary ambient air monitoring data archive. A comprehensive list of data flags that are recorded alongside AQS data sets, referred to by U.S. EPA as 'qualifiers', can be found at the following link: https://aqs.epa.gov/aqsweb/documents/codetables/qualifiers.html

Invalidation of reference data: AQS qualifiers are organized by qualifier type, which indicates whether data logged alongside qualifier flags should be invalidated (set null). Qualifiers with type "Null Data Qualifier" are invalidated, and includes data logged during periods that coincide with QC checks (e.g., "BF-Precision/Zero/Span", "BJ- Operator Error", "BL - QA Audit", "AZ - QC Audit") among other events such as power outages. Data logged alongside qualifiers with type "Quality Assurance Qualifiers" are not invalidated and are included in this analysis (e.g., concentrations less than the federal MDL for the reference monitor "MD – Value less than MDL", QA reviewed values "Validated Value").

Data Flags Recorded During Testing

FRM/FEM Monitor	Timestamp (UTC)	Flag
Teledyne API T640X (Acquired via AQS)	2019-09-24 23:00:00+0000	BE - Building/Site Repair.
	2019-09-30 13:00:00+0000	AZ - QC Audit
	2019-10-05 22:00:00+0000	BE - Building/Site Repair.

Meteorological Instrument	Timestamp (UTC)	Flag
RM Young 41375VC Temperature Monitor (Data acquired via AQS)	2019-09-27 13:00:00+0000 to 2019-09-27 14:00:00+0000	BA - Maintenance/Routine Repairs
[Make Model] Relative Humidity Monitor (Data acquired via AQS)	2019-09-27 13:00:00+0000 to 2019-09-27 14:00:00+0000	BA - Maintenance/Routine Repairs

Testing Report - $PM_{2.5}$ Base Testing

SENSIT RAMP

This report reflects out-of-the-box performance

Initial Base Testing - Decatur, GA
U.S. Environmental Protection Agency
Office of Research and Development
PI: Clements.Andrea@epa.gov
919-541-1363
November 2019—December 2019

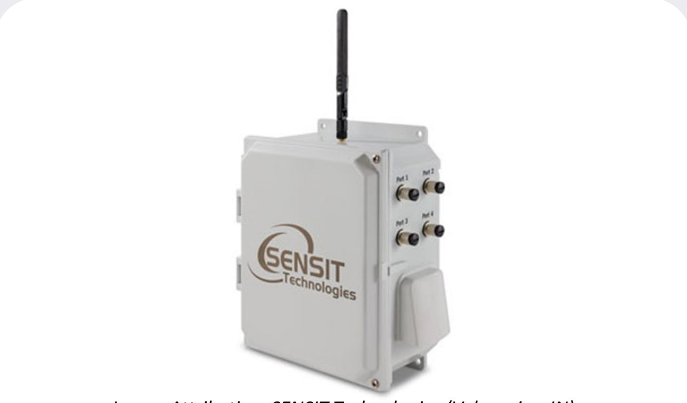

Image Attribution: SENSIT Technologies (Valparaiso, IN)

Deployment Details

Testing Organization and Site Information	
Testing organization (Name, Organization type, Contact website)	U.S. Environmental Protection Agency - Office of Research and Development<br>Federal Government<br>Air Sensor Toolbox | U.S. EPA Website
Testing location (City, State, Latitude and Longitude)	South Dekalb<br>Decatur, GA<br>33.6877, -84.2905
AQS site ID	13 - 089 - 0002
Sampling timeframe (MM-DD-YY)	11-28-19 to 12-28-19
Sensor data source	Onboard MicroSD card
Reference data source	AQS API download

Sensor Information			
Manufacturer, model	SENSIT RAMP		
Device firmware version	190313_AQ_v9.30		
Sampling time interval	15-seconds		
Sensor serial numbers	RAM_02		
Issues encountered during deployment?	☐	No Issues	

FRM/FEM Information	
Manufacturer, model, designation	Teledyne Advanced Pollution Instrumentation T640 FEM
Sampling time interval	1-hour averaging
Date of calibration	As required by 40 CFR Part 58 and the DE Ambient Air Quality Monitoring Program QAPP maintained by DNREC
Date of flowrate verification check	Monthly as required by 40 CFR Part 58 Appendix A
Description, date(s) of maintenance activities	See GA-RAM-Page 7 of this testing report

Time Series Plots

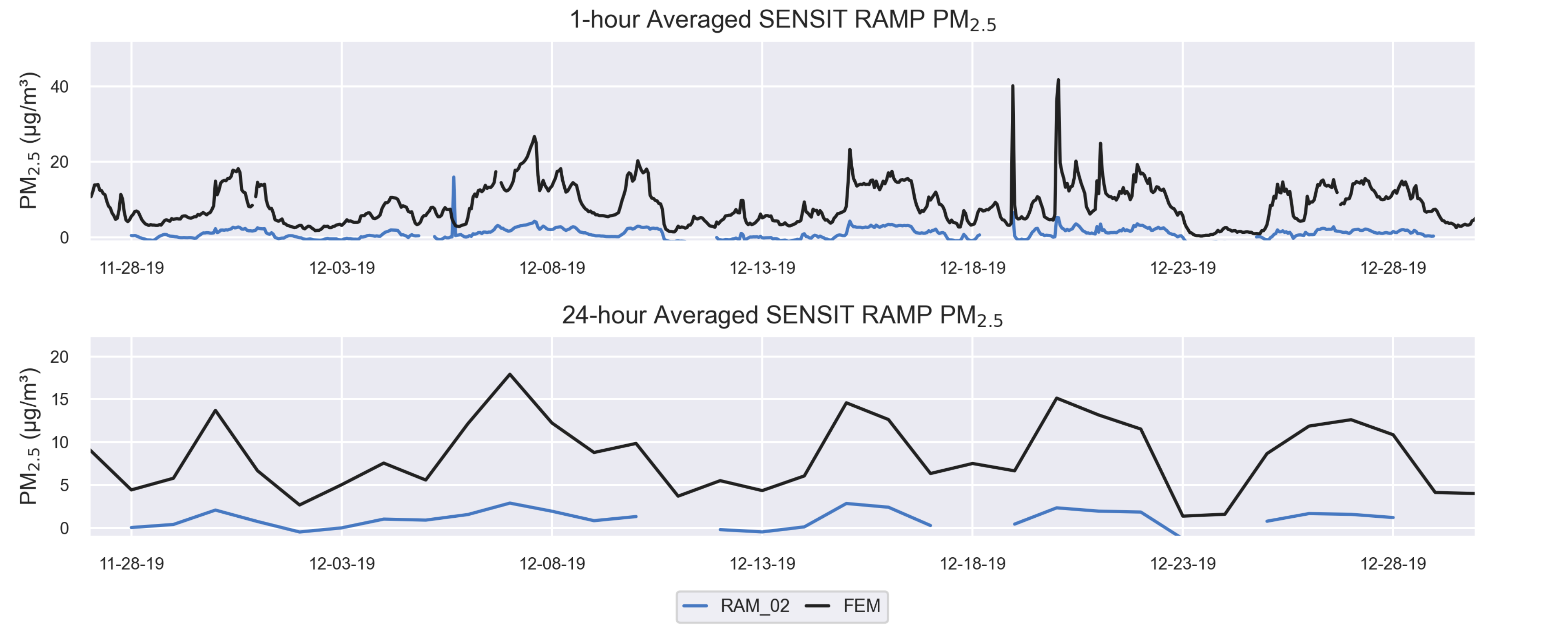

Scatter Plots: Comparison to FRM/FEM

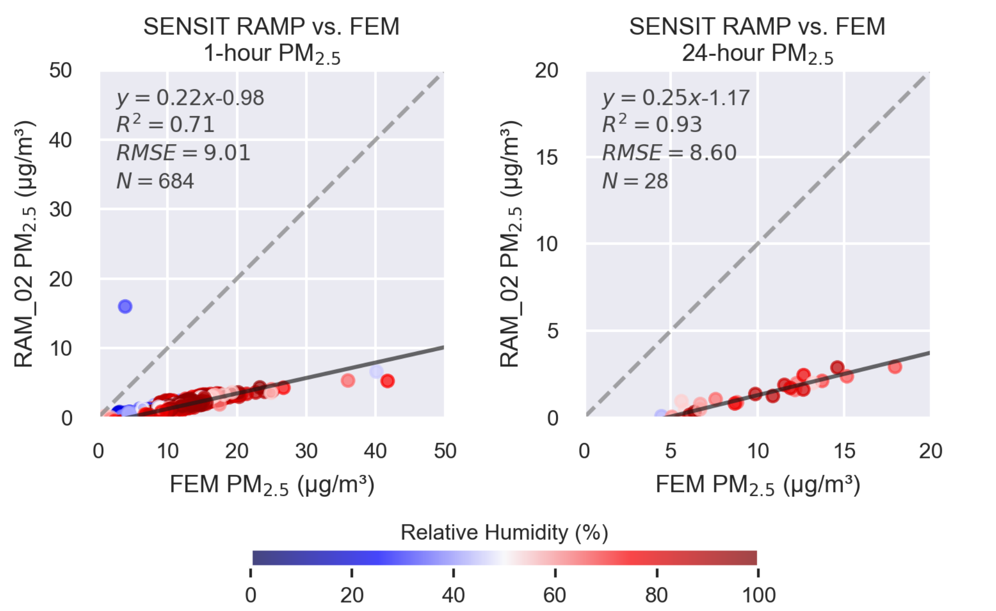

Range of FRM/FEM concentrations over duration of base test (µg/m³)	0.4-41.7 (1-hr),<br>1.4-17.9 (24-hr)
Number of 24-hr periods in FRM/FEM monitor measurements with a goal concentration ≥ 25 µg/m³	0

Performance Metrics

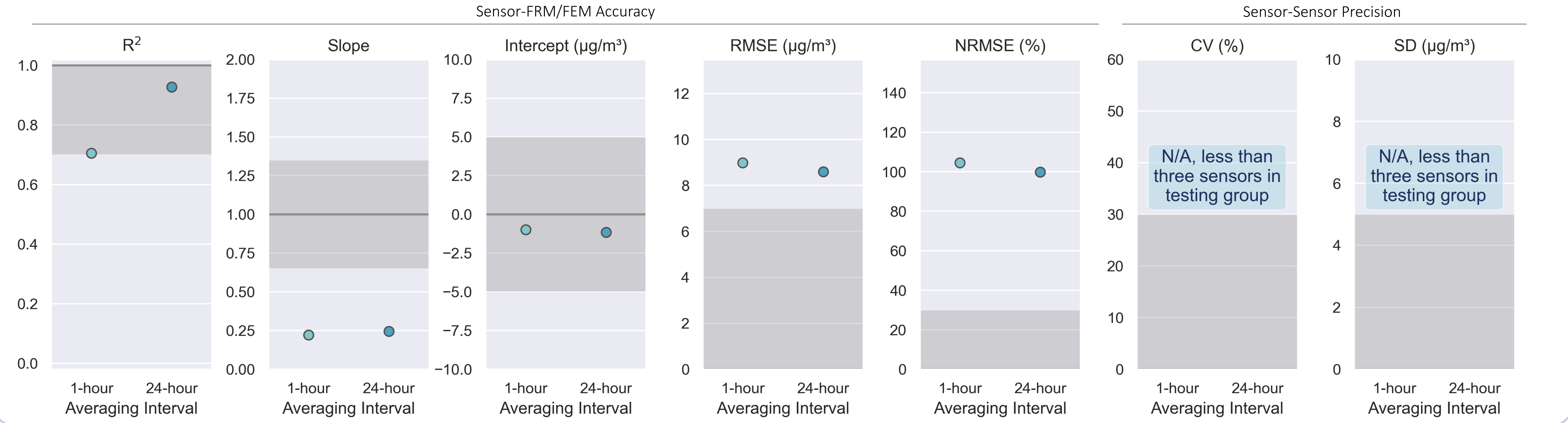

Meteorological Conditions During Deployment

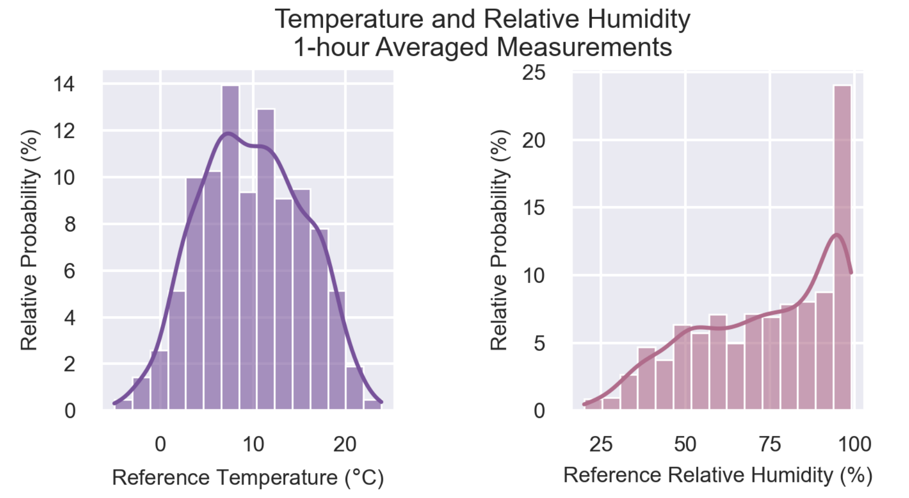

Number of 24-hr periods outside sensor manufacture-listed temperature operational range (-20 to 50 °C)	0
Number of 24-hr periods outside sensor manufacture-listed relative humidity operational range (no operational range specified)	-

Meteorological Influence

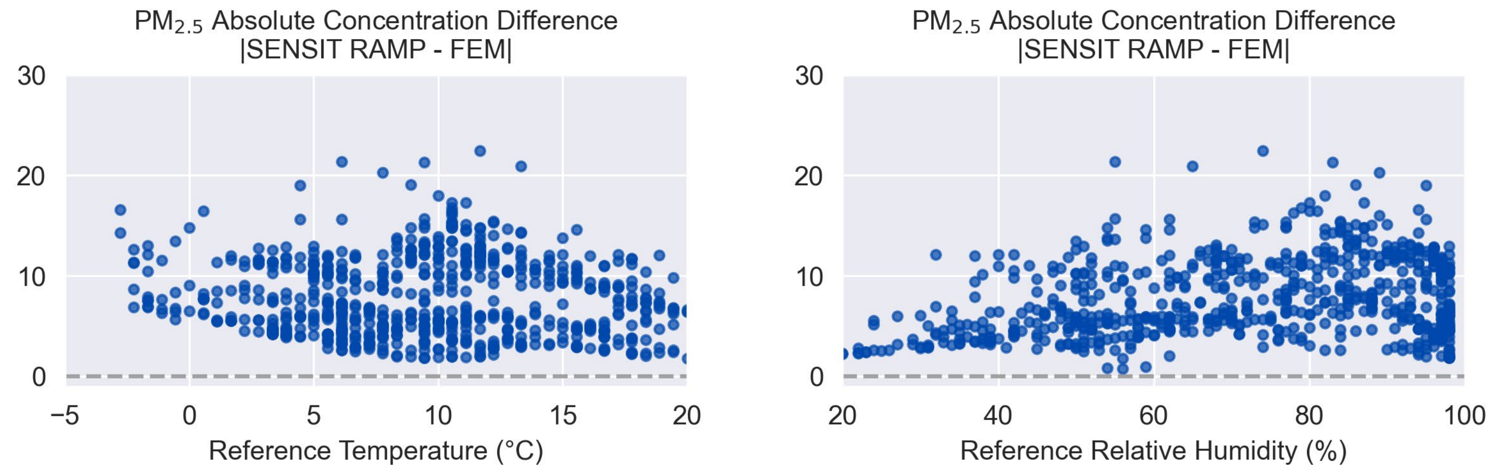

Mean number of paired, normalized concentration and temperature values (1-hr averages)	744
Mean number of paired, normalized concentration and relative humidity values (1-hr averages)	744

Testing Report - $PM_{2.5}$ Base Testing

SENSIT RAMP

This report reflects out-of-the-box performance

Initial Base Testing - Decatur, GA

U.S. Environmental Protection Agency
Office of Research and Development
PI: Clements.Andrea@epa.gov
919-541-1363

November 2019—December 2019

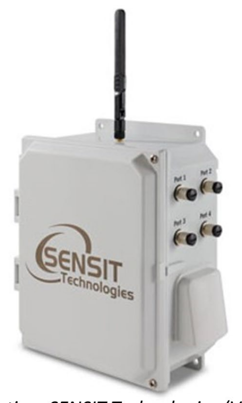

Image Attribution: SENSIT Technologies (Valparaiso, IN)

Tabular Statistics

Sensor-FRM/FEM Correlation

	Bias and Linearity						Data Quality			
	R^2		Slope		Intercept ($\mu g/m^3$)		Uptime (%)		Number of paired sensor and FRM/FEM concentration values	
	1-Hour ●	24-Hour ●	1-Hour ○	24-Hour ○	1-Hour ●	24-Hour ●	1-Hour ●	24-Hour ●	1-Hour	24-Hour
Metric Target Range	≥ 0.70	≥ 0.70	1.0 ± 0.35	1.0 ± 0.35	-5 ≤ b ≤ 5	-5 ≤ b ≤ 5	75%*	75%*	-	-
Sensor RAM_02	0.71	0.93	0.22	0.25	-0.98	-1.17	92	90	684	28

	Error			
	RMSE ($\mu g/m^3$)		NRMSE (%)	
	1-Hour ☆	24-Hour ☆	1-Hour ☆	24-Hour ☆
Metric Target Range	≤ 7.0	≤ 7.0	≤ 30.0	≤ 30.0
Deployment Value	9.0	8.6	104.5	99.8

Device-specific metrics (computed for each sensor in evaluation)
○○○ Metric value for none of devices tested falls within the target range
●○○ Metric value for one of devices tested falls within the target range
●●○ Metric value for two of devices tested falls within the target range
●●● Metric value for three of devices tested falls within the target range

Single-valued metrics (computed via entire evaluation dataset)
☆ Indicates that the metric value is not within the target range
★ Indicates that the metric value is within the target range

Sensor-Sensor Precision[1]

	Precision (between collocated sensors)				Data Quality	
	CV (%)		SD ($\mu g/m^3$)		Number of concurrent sensor concentration pairs	
	1-Hour ☆	24-Hour ☆	1-Hour ☆	24-Hour ☆	1-Hour	24-Hour
Metric Target Range	≤ 30.0	≤ 30.0	≤ 5.0	≤ 5.0	-	-
Deployment Value	-	-	-	-	-	-

[1]Precision statistics are computed for evaluations with at least three collocated sensor units. Metric values are left blank for evaluations with two or fewer sensor units.

*This value is only a recommendation for ensuring data quality and is not included in the list of target values discussed in Section 4 of the Performance Testing Protocols, Metrics, and Target Values for Fine Particulate Matter Air Sensors document.

PI: Clements.Andrea@epa.gov
919-541-1363

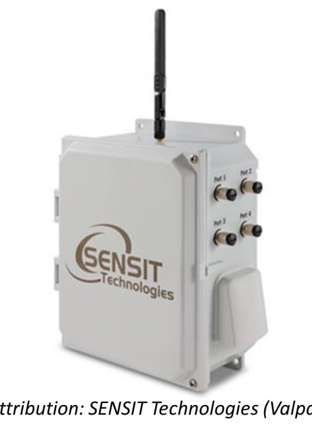
Image Attribution: SENSIT Technologies (Valparaiso, IN)

Sensor-FRM/FEM Scatter Plots

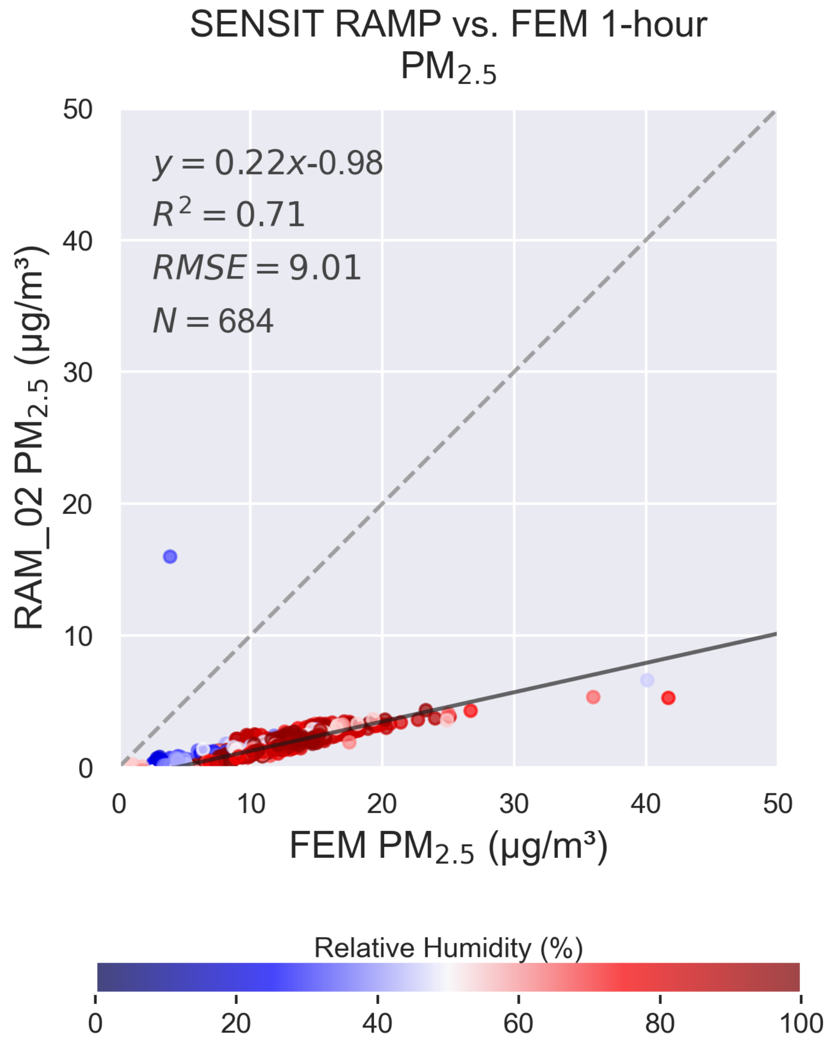

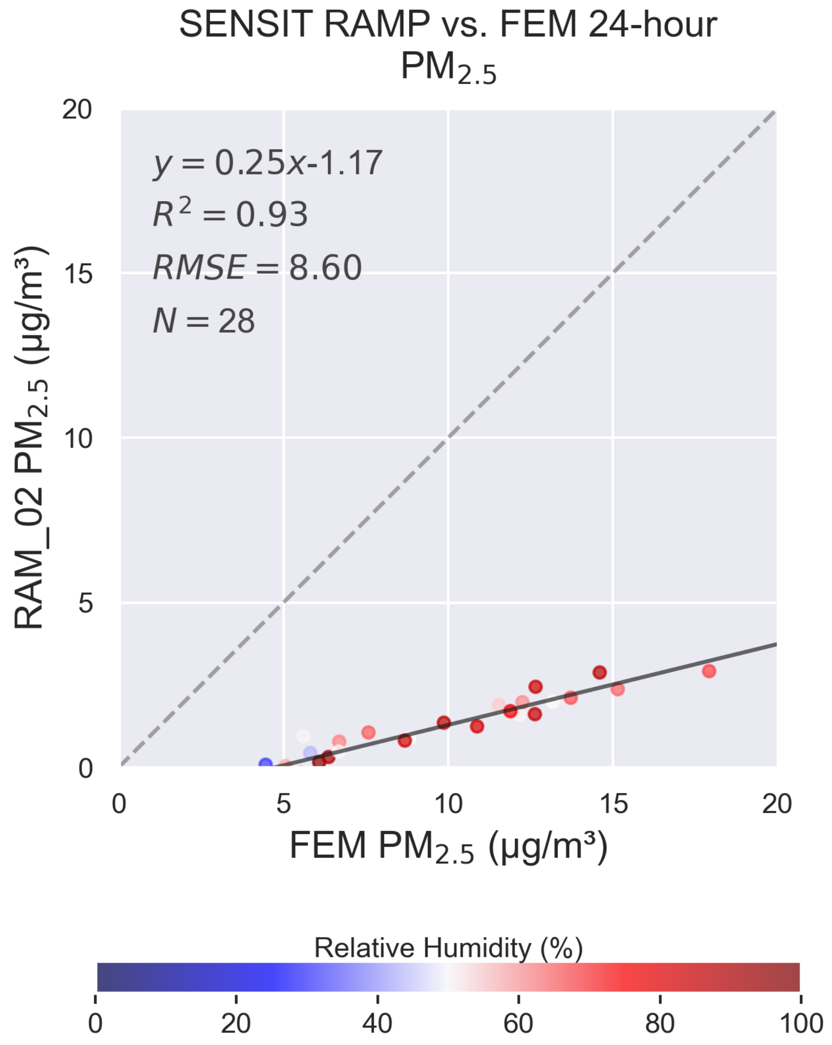

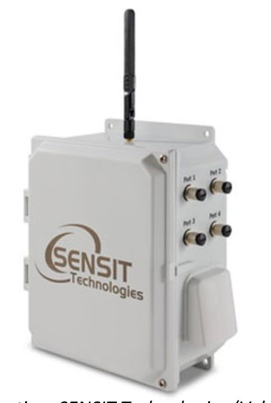

Image Attribution: SENSIT Technologies (Valparaiso, IN)

Supplemental Information

Abbreviations used in Supplemental Information

FRM	Federal Reference Method
FEM	Federal Equivalent Method
SOP	Standard Operating Procedure
QAPP	Quality Assurance Project Plan
QC	Quality Control

Supplemental Documentation	Attached	Description & URL or file path to documentation
Field observations and sensor data flags	☑	See GA-RAM-Page 6 of this testing report
Maintenance logs	☐	No logs recorded during testing
Standard operating procedure(s)	☐	U.S. EPA Office Of Research and Development SOP available upon request
Photos of equipment setup and testing	☑	See GA-RAM-Page 5 of this testing report
Product specifications sheet(s)	☑	SENSIT Technologies: RAMP Product Brochure
Product manual(s)	☑	SENSIT Technologies: RAMP Product Manual
Data storage and transmission method	☑	See GA-RAM-Page 6 of this testing report
Data correction approach	☑	See GA-RAM-Page 6 of this testing report
Issues encountered	☑	See GA-RAM-Page 6 of this testing report
Data analysis/correction scripts and version	☑	Averaging and processing of data, calculation of performance metrics, and generation of figures and other supplementary material for analysis were obtained using Python 3.9.7 with the packages sensortoolkit v0.8.3b2, pandas 1.3.5, NumPy 1.21.2, Matplotlib 3.5.0, statsmodels 0.13.0, and seaborn 0.11.2. All packages are available from the Python Package Index (PyPI) at https://pypi.org. The integrated development environment (IDE) Spyder 5.1.5 was used for scripting and data visualization. Version control for the Python base, packages, and IDE were all managed by conda 4.11.0.
Air Monitoring Station QAPP	☐	U.S. EPA Office Of Research and Development QAPP available upon request
Summary of FRM/FEM monitor QC checks	☑	See GA-RAM-Page 7 of this testing report
Manufacturer website for FRM/FEM monitor	☑	Teledyne API: T640/T640X Product website
FRM/FEM monitor manual	☑	Teledyne API: T640/T640X Product Manual
FRM/FEM monitor specifications sheet(s)	☑	Teledyne API: T640/T640X Specification Sheet
Other documents	☐	

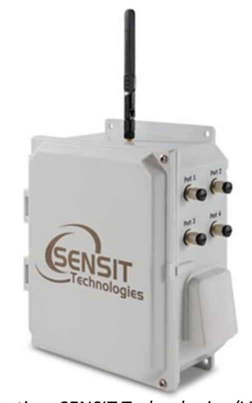

Image Attribution: SENSIT Technologies (Valparaiso, IN)

Supplemental Information: Photos of Testing Site and Equipment Setup

Site Description:
The South DeKalb monitoring station was initially established as an O3 site in 1974 and is located in the Atlanta area to the southeast of the city. More measurements were added to the site in the 1990's and 2000's. This site is currently a multipollutant site that is designated as a National Core (Ncore), Photochemical Assessment Monitoring Station (PAMS), and National Air Toxics Trends Station (NATTS) site for the state of Georgia. Measurements at South DeKalb include continuous analyzers for ozone, carbon monoxide, nitrogen dioxide, sulfur dioxide, and particulate matter (PM) with an aerodynamic diameter less than 2.5 microns (PM2.5) and 10 microns (PM10). Volatile organic compounds (VOCs) are also measured during summer periods via AutoGC as well as year-round via canister sampling. Local meteorology is also measured at the site, including wind speed and direction, temperature, relative humidity, pressure, and precipitation.

Figure 1: SENSIT RAMP sensor (indicated by red arrow) attached to metal railing atop the sampling shelter at the monitoring site.

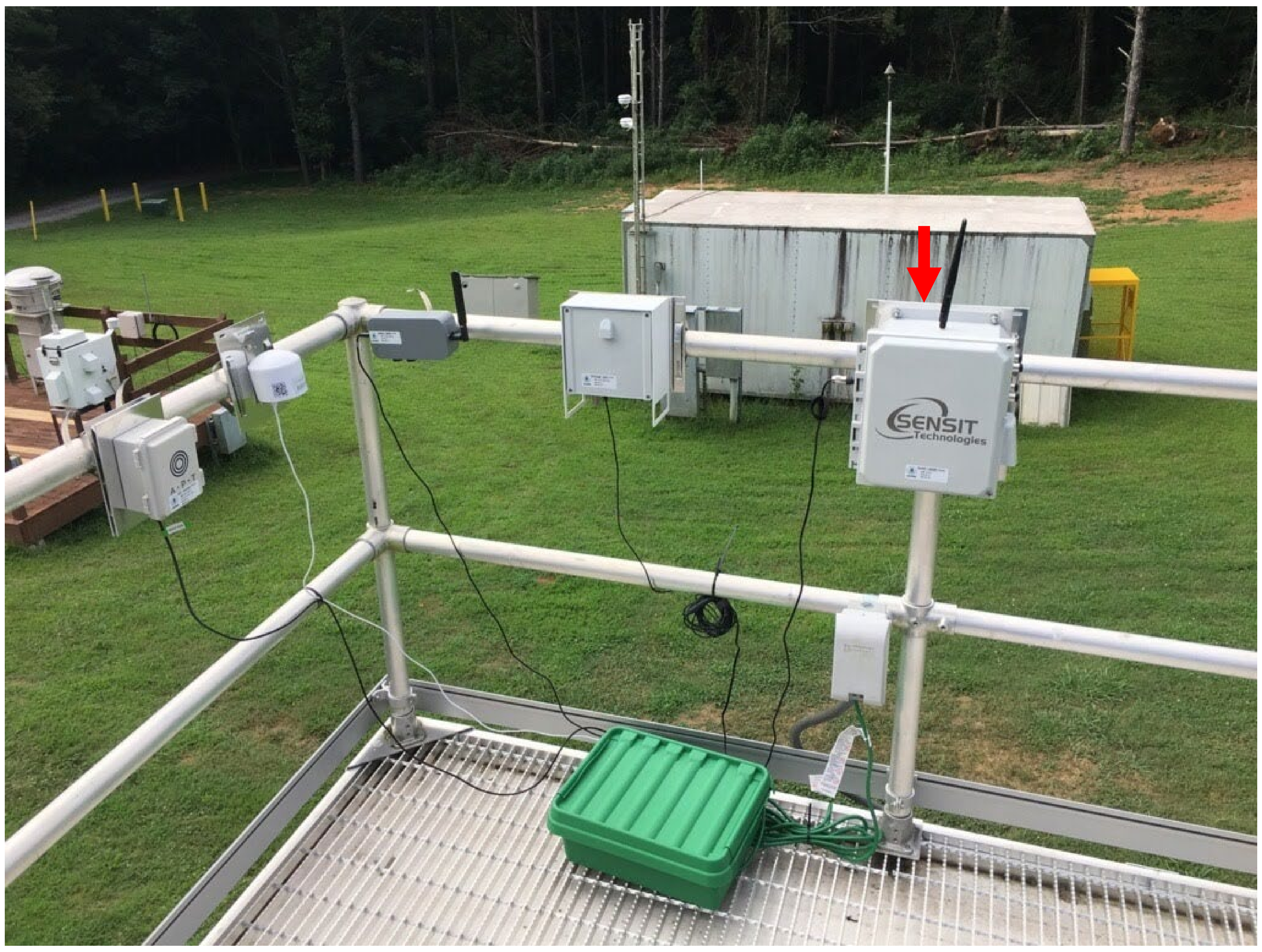

Figure 2: South Dekalb Monitoring Station

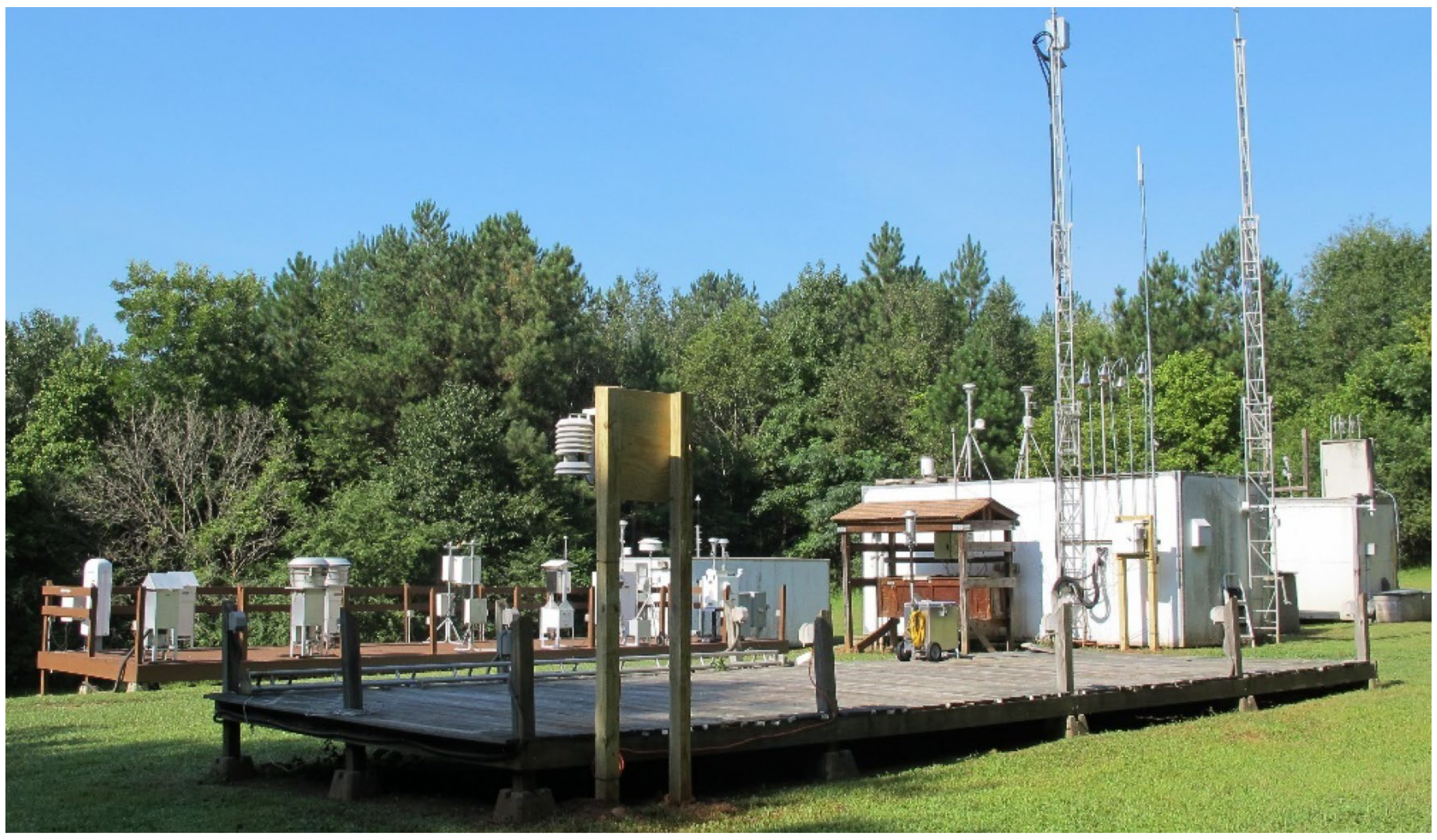

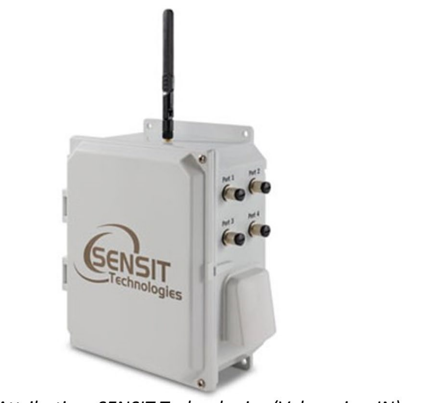

Image Attribution: SENSIT Technologies (Valparaiso, IN)

Supplemental Information: Data Storage, Correction Approach, and Issues Encountered

Data Storage and Transmission Method

The SENSIT RAMP was configured to record data at a 15-second sampling interval. Data are stored as daily text files (.txt format) on an onboard MicroSD card. Data files were obtained weekly via SD cards. Each field site operator was provided two labeled MicroSD cards for sensor units that they used to swap out each week. Data from the collected card was then read and processed off-site.

Data Correction Approach

SENSIT RAMP units were pre-configured by the manufacturer with a linear correction (i.e., concentration gain = 1.0 and offset = 0.0 µg m^{-3}). These presets reflect out-of-the box performance and were not modified by EPA prior or during testing.

After acquisition, the raw data was processed using the *sensortoolkit* python code library (v0.8.3b2). A continuous data set at the recorded sampling frequency was written to a .csv file. 1-hour and 24-hour averaged data sets were generated using a 75% completeness threshold and saved as separate .csv files. Outliers were NOT removed from data sets in order to assess "out-of-the-box" sensor performance.

Issues Encountered

<u>Pre-deployment observations</u>

- SENSIT RAMP units were received without documentation or manuals. After communicating the need to change default settings (logging interval and time zone) with the manufacturer, a draft user's manual and a USB cable were supplied. With the use of this USB cable, instrument settings could be changed, and real-time data could be logged using a serial communication software (CoolTerm, v.1.5.0). Because the sensor did not record data at the top of every minute, the RAMP was configured to record data at 15-second intervals so that the data could be averaged more closely to complete minutes. Prior to deployment, RAMP units were collocated in a bench-top evaluation to verify operational status and determine the extent of data invalidity (i.e., determine equilibration period) after an initial start-up event. The recorded response for parameters measured by the RAMP suggests that the gas sensors (CO, NO, NO_2, O_3) required approximately a 2-hour equilibration period, while the remaining sensors (temperature, relative humidity, particulate matter) did not require any equilibration period.

<u>Field observations and sensor data flags</u>

During shipment of the SENSIT RAMP to the South Dekalb site in August 2019, the initial unit was severely damaged due to a failure of the main bracket holding the unit's battery pack and main circuit board within the device enclosure. A replacement unit (referred to in this report as RAM_01) replaced the initial unit and was deployed on 8/15/2019. Due to a miscommunication, the replacement unit was temporarily removed from the site on 8/28/2019 and was reinstalled on 9/11/2019.

During the testing period described here, the RAMP operated nominally and did not require replacement or repair.

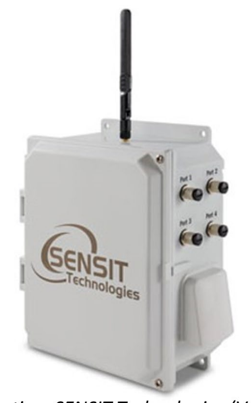

Image Attribution: SENSIT Technologies (Valparaiso, IN)

Description of Data Flags

AQS

The U.S. EPA's Air Quality System (AQS) is the Agency's primary ambient air monitoring data archive. A comprehensive list of data flags that are recorded alongside AQS data sets, referred to by U.S. EPA as 'qualifiers', can be found at the following link: https://aqs.epa.gov/aqsweb/documents/codetables/qualifiers.html

Invalidation of reference data: AQS qualifiers are organized by qualifier type, which indicates whether data logged alongside qualifier flags should be invalidated (set null). Qualifiers with type "Null Data Qualifier" are invalidated, and includes data logged during periods that coincide with QC checks (e.g., "BF-Precision/Zero/Span", "BJ- Operator Error", "BL - QA Audit", "AZ - QC Audit") among other events such as power outages. Data logged alongside qualifiers with type "Quality Assurance Qualifiers" are not invalidated and are included in this analysis (e.g., concentrations less than the federal MDL for the reference monitor "MD – Value less than MDL", QA reviewed values "Validated Value").

Data Flags Recorded During Testing

FRM/FEM Monitor	Timestamp (UTC)	Flag
Teledyne API T640X (Acquired via AQS)	2019-11-30 22:00:00+0000	BE - Building/Site Repair.
	2019-12-06 17:00:00+0000	BA - Maintenance/Routine Repairs
	2019-12-06 18:00:00+0000	AZ - QC Audit
	2019-12-07 12:00:00+0000	V - Validated Value.
	2019-12-20 00:00:00+0000	V - Validated Value.
	2019-12-26 17:00:00+0000	AT - Calibration.

Testing Report - PM$_{2.5}$ Base Testing

QuantAQ ARISense

This report reflects out-of-the-box performance

Initial Base Testing - Decatur, GA
U.S. Environmental Protection Agency
Office of Research and Development
PI: Clements.Andrea@epa.gov
919-541-1363
February 2020—March 2020

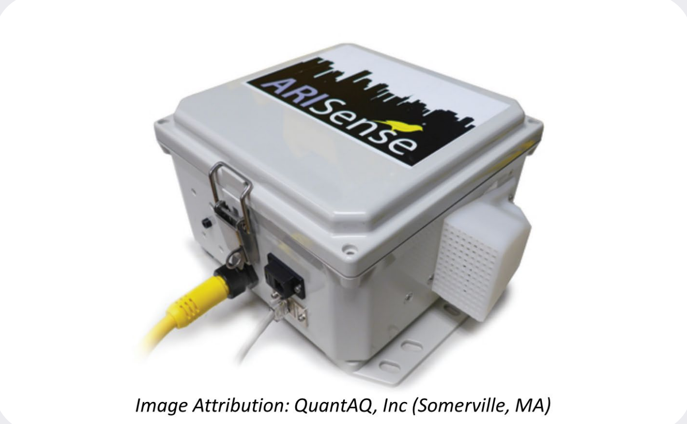

Image Attribution: QuantAQ, Inc (Somerville, MA)

Deployment Details

Testing Organization and Site Information	
Testing organization (Name, Organization type, Contact website)	U.S. Environmental Protection Agency - Office of Research and Development<br>Federal Government<br>Air Sensor Toolbox | U.S. EPA Website
Testing location (City, State, Latitude and Longitude)	South Dekalb<br>Decatur, GA<br>33.6877, -84.2905
AQS site ID	13 - 089 - 0002
Sampling timeframe (MM-DD-YY)	02-08-20 to 03-03-20
Sensor data source	QuantAQ Cloud download
Reference data source	AQS API download

Sensor Information			
Manufacturer, model	QuantAQ ARISense		
Device firmware version	Received by EPA on 4/30/2019		
Sampling time interval	1-minute		
Sensor serial numbers	ARS_01		
Issues encountered during deployment?	☑	See GA-ARS-Page 6 of this testing report	

FRM/FEM Information	
Manufacturer, model, designation	Teledyne Advanced Pollution Instrumentation T640 FEM
Sampling time interval	1-hour averaging
Date of calibration	As required by 40 CFR Part 58 and the DE Ambient Air Quality Monitoring Program QAPP maintained by DNREC
Date of flowrate verification check	Monthly as required by 40 CFR Part 58 Appendix A
Description, date(s) of maintenance activities	N/A

Time Series Plots

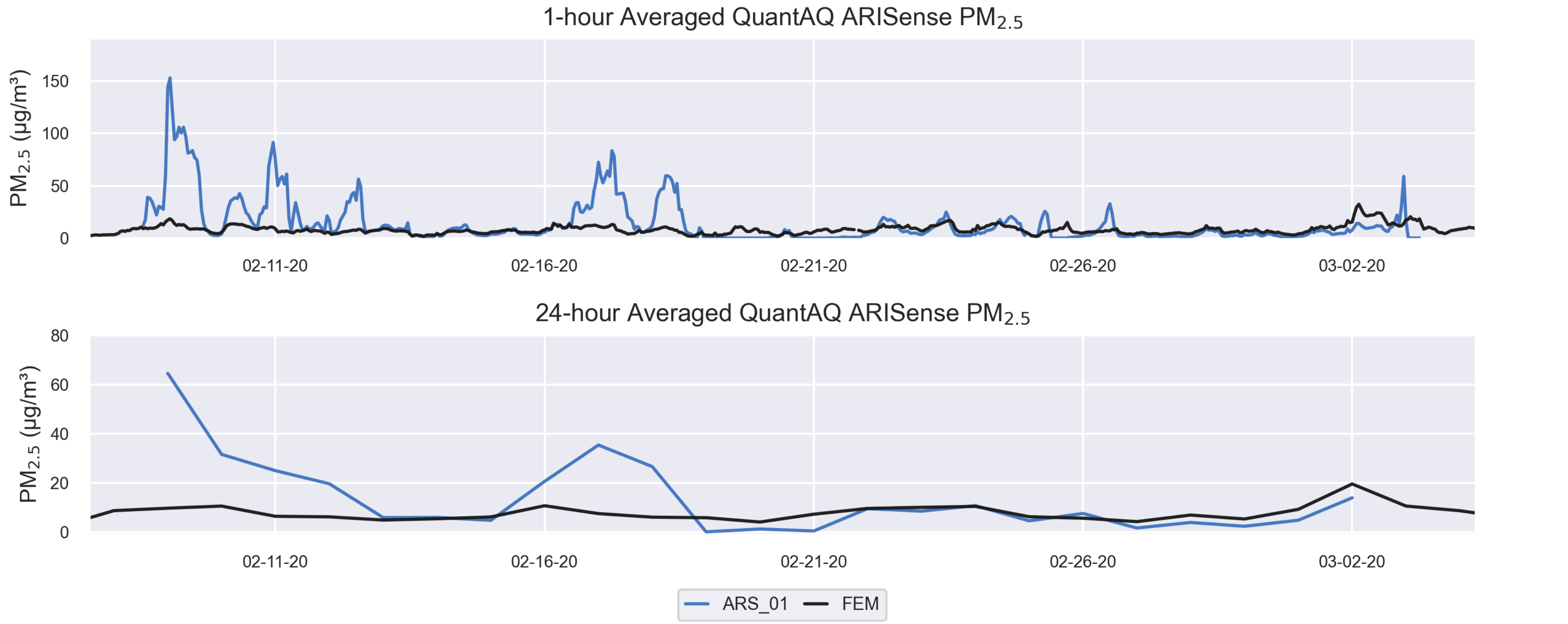

Scatter Plots: Comparison to FRM/FEM

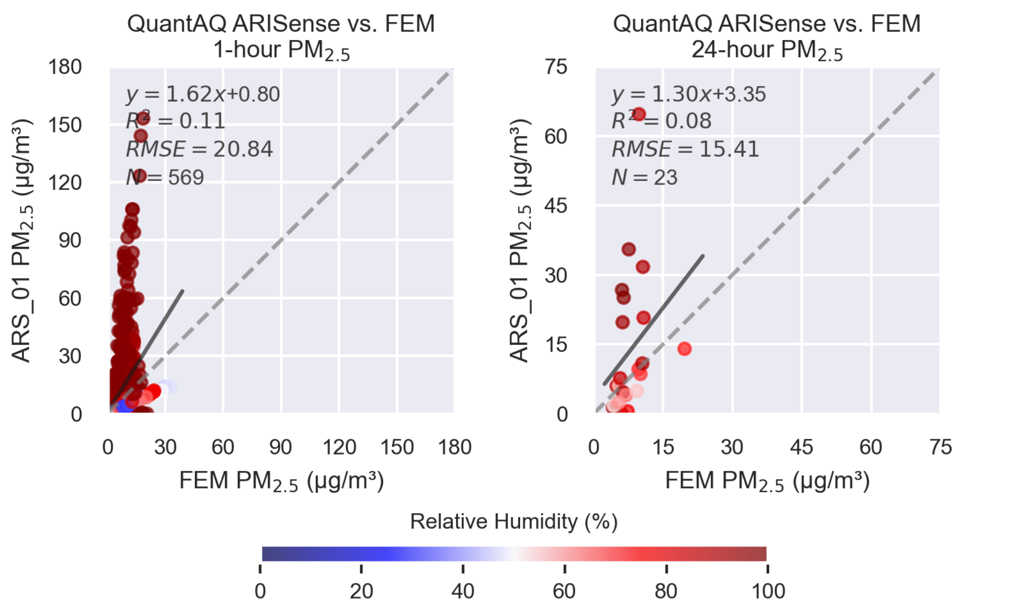

Range of FRM/FEM concentrations over duration of base test (µg/m³)	1.5-32.3 (1-hr),<br>4.2-19.7 (24-hr)
Number of 24-hr periods in FRM/FEM monitor measurements with a goal concentration ≥ 25 µg/m³	0

Performance Metrics

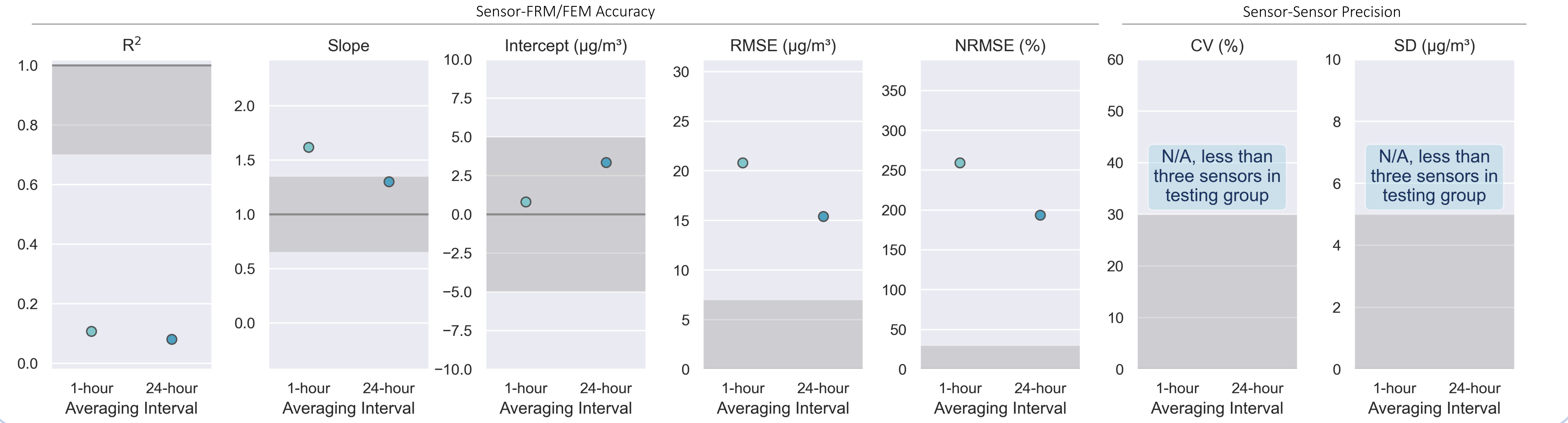

Meteorological Conditions During Deployment

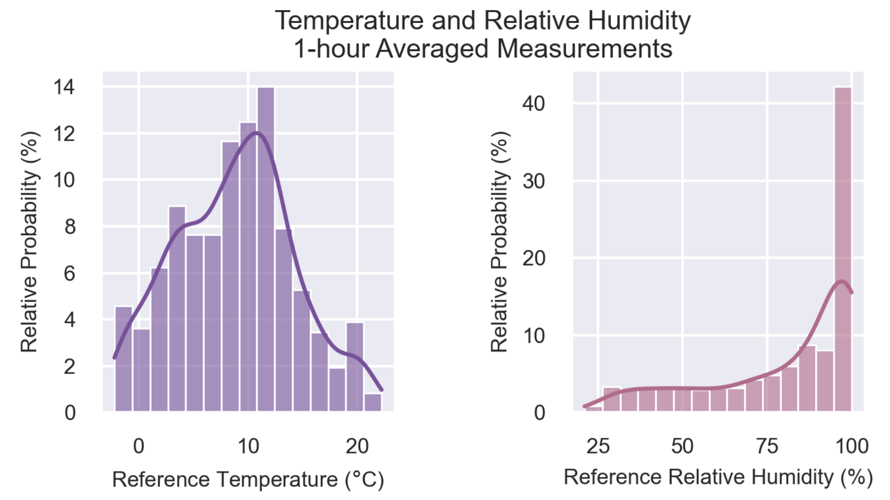

Number of 24-hr periods outside sensor manufacture-listed temperature operational range (no operational range specified)	-
Number of 24-hr periods outside sensor manufacture-listed relative humidity operational range (no operational range specified)	-

Meteorological Influence

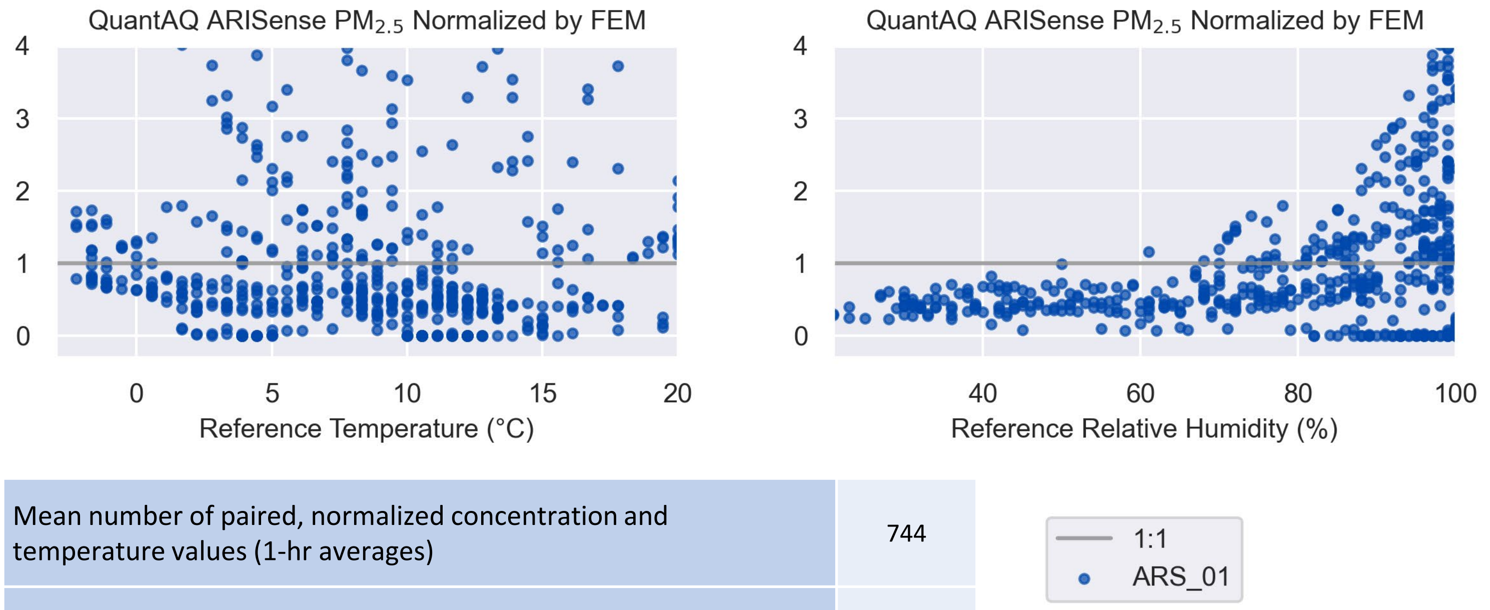

Mean number of paired, normalized concentration and temperature values (1-hr averages)	744
Mean number of paired, normalized concentration and relative humidity values (1-hr averages)	744

Testing Report - $PM_{2.5}$ Base Testing

QuantAQ ARISense

This report reflects out-of-the-box performance

Initial Base Testing - Decatur, GA

U.S. Environmental Protection Agency
Office of Research and Development
PI: Clements.Andrea@epa.gov
919-541-1363

February 2020—March 2020

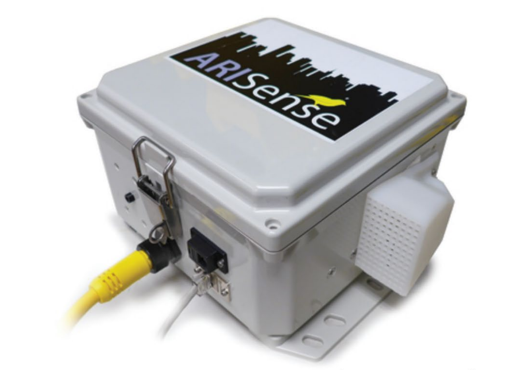

Image Attribution: QuantAQ, Inc (Somerville, MA)

Tabular Statistics

Sensor-FRM/FEM Correlation

	Bias and Linearity						Data Quality			
	R^2		Slope		Intercept (µg/m³)		Uptime (%)		Number of paired sensor and FRM/FEM concentration values	
	1-Hour ○	24-Hour ○	1-Hour ○	24-Hour ●	1-Hour ●	24-Hour ●	1-Hour ●	24-Hour ●	1-Hour	24-Hour
Metric Target Range	≥ 0.70	≥ 0.70	1.0 ± 0.35	1.0 ± 0.35	-5 ≤ b ≤ 5	-5 ≤ b ≤ 5	75%*	75%*	-	-
Sensor ARS_01	0.11	0.08	1.62	1.30	0.80	3.35	99	96	569	23

	Error			
	RMSE (µg/m³)		NRMSE (%)	
	1-Hour ☆	24-Hour ☆	1-Hour ☆	24-Hour ☆
Metric Target Range	≤ 7.0	≤ 7.0	≤ 30.0	≤ 30.0
Deployment Value	20.8	15.4	259.3	193.7

Device-specific metrics (computed for each sensor in evaluation)
○○○ Metric value for none of devices tested falls within the target range
●○○ Metric value for one of devices tested falls within the target range
●●○ Metric value for two of devices tested falls within the target range
●●● Metric value for three of devices tested falls within the target range

Single-valued metrics (computed via entire evaluation dataset)
☆ Indicates that the metric value is not within the target range
★ Indicates that the metric value is within the target range

Sensor-Sensor Precision[1]

	Precision (between collocated sensors)				Data Quality	
	CV (%)		SD (µg/m³)		Number of concurrent sensor concentration pairs	
	1-Hour ☆	24-Hour ☆	1-Hour ☆	24-Hour ☆	1-Hour	24-Hour
Metric Target Range	≤ 30.0	≤ 30.0	≤ 5.0	≤ 5.0	-	-
Deployment Value	-	-	-	-	-	-

[1]Precision statistics are computed for evaluations with at least three collocated sensor units. Metric values are left blank for evaluations with two or fewer sensor units.

*This value is only a recommendation for ensuring data quality and is not included in the list of target values discussed in Section 4 of the Performance Testing Protocols, Metrics, and Target Values for Fine Particulate Matter Air Sensors document.

Testing Report - PM$_{2.5}$ Base Testing

QuantAQ ARISense

This report reflects out-of-the-box performance

Initial Base Testing - Decatur, GA

U.S. Environmental Protection Agency
Office of Research and Development
PI: Clements.Andrea@epa.gov
919-541-1363

February 2020—March 2020

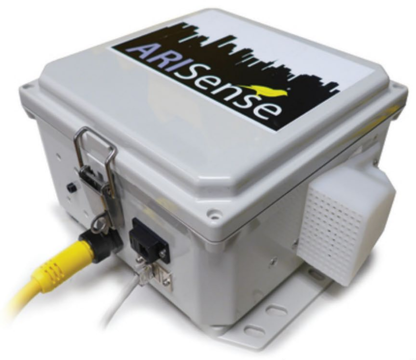

Image Attribution: QuantAQ, Inc (Somerville, MA)

Sensor-FRM/FEM Scatter Plots

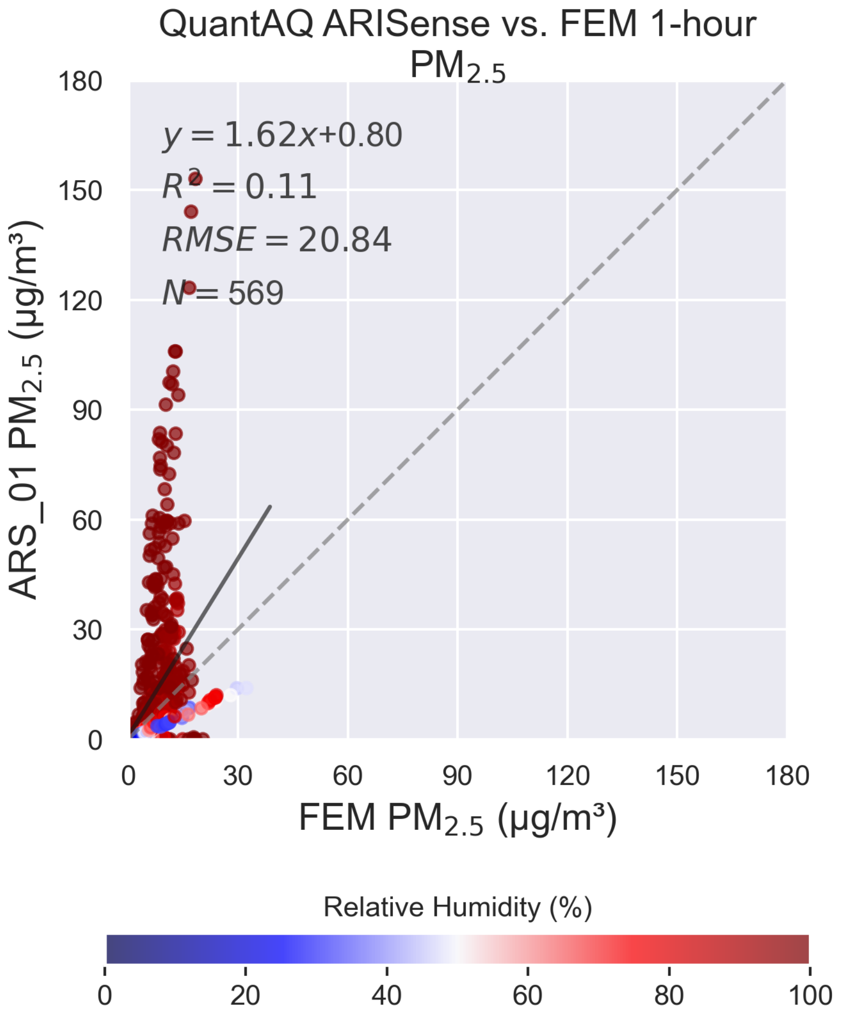

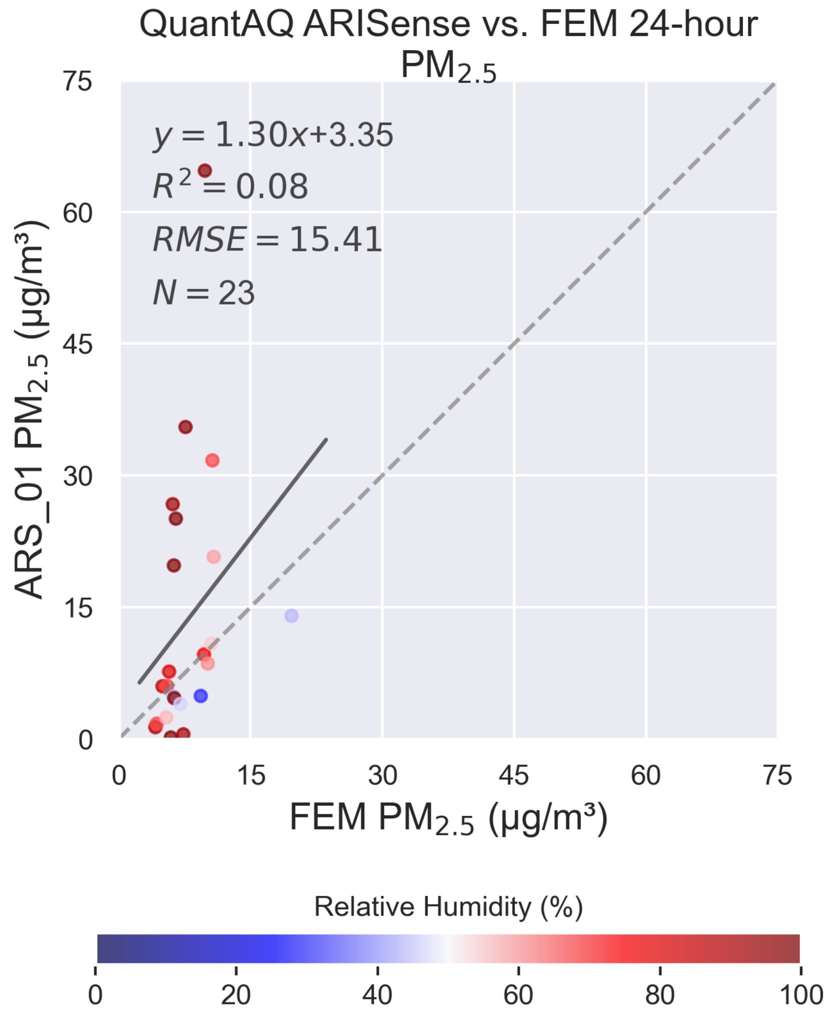

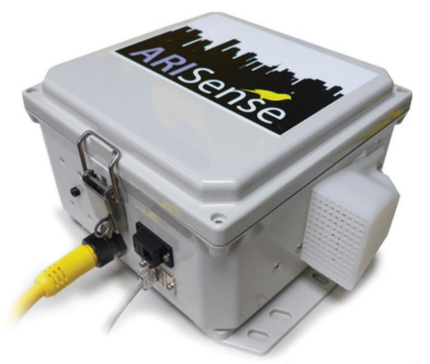

Image Attribution: QuantAQ, Inc (Somerville, MA)

Supplemental Information

Abbreviations used in Supplemental Information

FRM	Federal Reference Method
FEM	Federal Equivalent Method
SOP	Standard Operating Procedure
QAPP	Quality Assurance Project Plan
QC	Quality Control

Supplemental Documentation	Attached	Description & URL or file path to documentation
Field observations and sensor data flags	☑	See GA-ARS-Page 7 of this testing report
Maintenance logs	☐	No logs recorded during testing
Standard operating procedure(s)	☐	U.S. EPA Office Of Research and Development SOP available upon request
Photos of equipment setup and testing	☑	See GA-ARS-Page 5 of this testing report
Product specifications sheet(s)	☑	QuantAQ and Particles Plus: ARISense Specification Sheet
Product manual(s)	☐	N/A
Data storage and transmission method	☑	See GA-ARS-Page 6 of this testing report
Data correction approach	☑	See GA-ARS-Page 6 of this testing report
Issues encountered	☑	See GA-ARS-Page 6 of this testing report
Data analysis/correction scripts and version	☑	Averaging and processing of data, calculation of performance metrics, and generation of figures and other supplementary material for analysis were obtained using Python 3.9.7 with the packages sensortoolkit v0.8.3b2, pandas 1.3.5, NumPy 1.21.2, Matplotlib 3.5.0, statsmodels 0.13.0, and seaborn 0.11.2. All packages are available from the Python Package Index (PyPI) at https://pypi.org. The integrated development environment (IDE) Spyder 5.1.5 was used for scripting and data visualization. Version control for the Python base, packages, and IDE were all managed by conda 4.11.0.
Air Monitoring Station QAPP	☐	U.S. EPA Office Of Research and Development QAPP available upon request
Summary of FRM/FEM monitor QC checks	☑	See GA-ARS-Page 8 of this testing report
Manufacturer website for FRM/FEM monitor	☑	Teledyne API: T640/T640X Product website
FRM/FEM monitor manual	☑	Teledyne API: T640/T640X Product Manual
FRM/FEM monitor specifications sheet(s)	☑	Teledyne API: T640/T640X Specification Sheet
Other documents	☐	

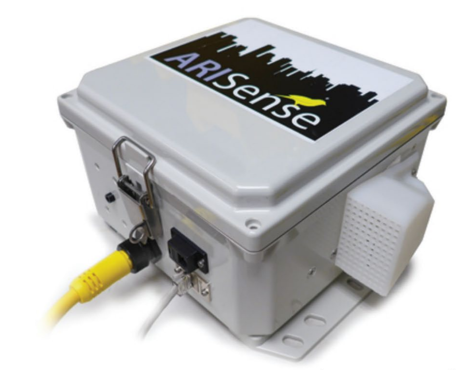

Image Attribution: QuantAQ, Inc (Somerville, MA)

Supplemental Information: Photos of Testing Site and Equipment Setup

Site Description:

The South DeKalb monitoring station was initially established as an O3 site in 1974 and is located in the Atlanta area to the southeast of the city. More measurements were added to the site in the 1990's and 2000's. This site is currently a multipollutant site that is designated as a National Core (Ncore), Photochemical Assessment Monitoring Station (PAMS), and National Air Toxics Trends Station (NATTS) site for the state of Georgia. Measurements at South DeKalb include continuous analyzers for ozone, carbon monoxide, nitrogen dioxide, sulfur dioxide, and particulate matter (PM) with an aerodynamic diameter less than 2.5 microns (PM2.5) and 10 microns (PM10). Volatile organic compounds (VOCs) are also measured during summer periods via AutoGC as well as year-round via canister sampling. Local meteorology is also measured at the site, including wind speed and direction, temperature, relative humidity, pressure, and precipitation.

Figure 1: South Dekalb Monitoring Station

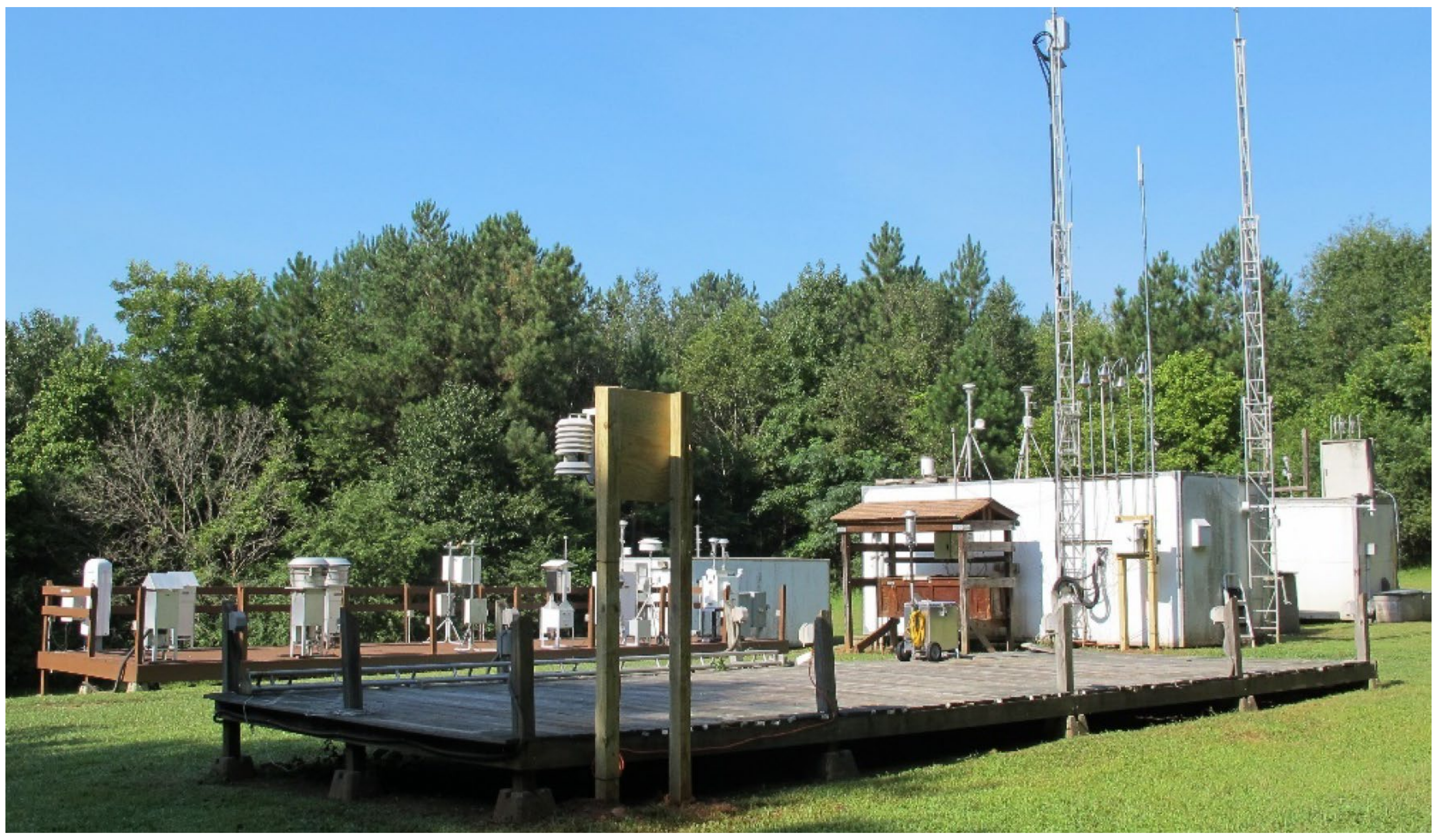

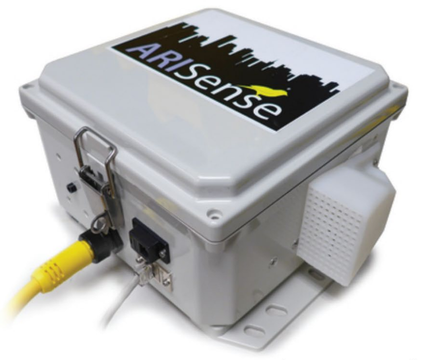

Image Attribution: QuantAQ, Inc (Somerville, MA)

Data Storage and Transmission Method

ARISense units upload data to an external cloud server at 2-minute averages via cellular signal. ARISense weekly data collections were performed by downloading data from the QuantAQ cloud server (last accessed 5/11/22).

Data Correction Approach

QuantAQ ARISense units measure PM2.5 concentrations via an optical particle counter (OPC). The OPC measures particle counts within particle size bins ranging from 0.3 microns to 10 microns, and mass concentrations for each bins are estimated from particle counts assuming constant values for density, spherical morphology, and average particle diameter. The manufacturer applies a general correction to account for the detection limitations of the OPC leading to concentrations that are closer to reality. These presets were configured by the sensor manufacturer and were not modified by EPA prior or during testing.

After acquisition, the raw data was processed using the *sensortoolkit* python code library (v0.8.3b2). A continuous data set at the recorded sampling frequency was written to a .csv file. 1-hour and 24-hour averaged data sets were generated using a 75% completeness threshold and saved as separate .csv files. Outliers were NOT removed from data sets in order to assess "out-of-the-box" sensor performance.

Issues Encountered

Pre-deployment observations

Numerous issues were encountered with the ARISense devices. Although previous models of the ARISense had used AlphaSense OPC-N2 these OPCs were unavailable at the time of manufacture. So instead, components from a particle plus OPC handheld were used.

- *Shipping damage:* On arrival to EPA many of the units were damaged due to the heavy pump breaking free during shipping and shaking around inside of the box breaking much of the plastic scaffolding and, in some cases, components. The manufacturer worked with us to fix these units.
- *Hardware and Firmware issues:* Multiple hardware and firmware issues were experienced that had to be fixed before starting the deployment and multiple manual firmware updates on both the gas sensor microcontrollers and the OPC microcontrollers were completed by researchers. The USB drives were frequently corrupted which required replacement and eventually a hardware update. In addition, the OPC microcontroller and the gas sensor microcontroller often miscommunicated. One firmware version reduced the number of bins reported by the OPC since the manufacturer determined the additional bins were not providing additional accuracy. New PCB boards were sent and installed before the tests could be run as well.
- *Initiation:* There was a high power draw when the cellular connectivity initiated and the pump was turned on. This meant that units occasionally failed to turn on at the same time and it meant these could not be powered by solar.
- *Water intake:* In addition, the OPC pump often drew in water during rainstorms leading the inline filter to saturate and the pump to fail due to overheating. Many pumps had to be replaced throughout the testing.
- *Cellular connectivity:* Some units could not consistently stay connected to the cellular, though other sensors at the same site did not experience issues. The manufacturer could not recreate this issue when they were sent back for service and the problems persisted.
- *Reset:* These units ended up being operated on timers so that they were power cycled every night which would typically get them back online if a software error had been encountered, leading to lower data loss than would have occurred with only weekly checks.

Field observations and sensor data flags are included on the following page

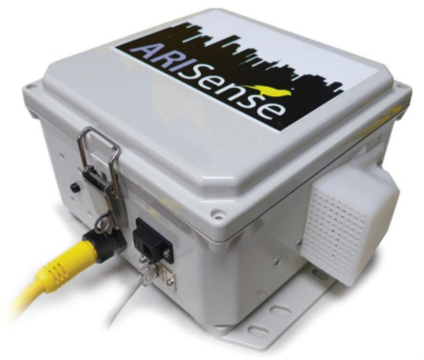

Image Attribution: QuantAQ, Inc (Somerville, MA)

Field observations and sensor data flags

The following table contains data flags describing events that were encountered during the testing period. The QuantAQ ARISense was configured to record data at regular 60-second sampling intervals, however, occasionally the unit recorded irregular sampling intervals ranging from 2-minute intervals to nearly 15 minutes at longest interval duration. The flags below note when these irregular sampling intervals occurred during the testing period. Sampling interval irregularities were not a result of modification to the device firmware configuration and are potentially associated power loading during periods where the ARISense unit initiated a cellular data upload.

Start Time (UTC)	End Time (UTC)	Sensor Serial ID	Parameters Impacted	Flag
2020-02-12 11:54:00+00:00	2020-02-12 12:00:00+00:00	ARS_01	ALL	6-Sampling interval abnormality
2020-02-14 01:26:00+00:00	2020-02-14 01:39:00+00:00	ARS_01	ALL	6-Sampling interval abnormality

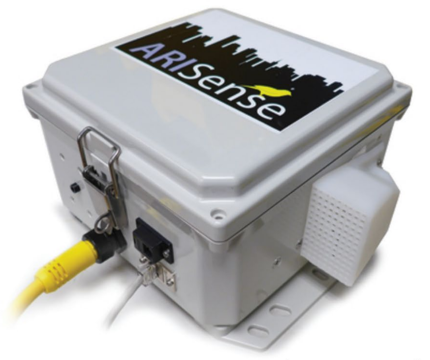

Image Attribution: QuantAQ, Inc (Somerville, MA)

Description of Data Flags

AQS

The U.S. EPA's Air Quality System (AQS) is the Agency's primary ambient air monitoring data archive. A comprehensive list of data flags that are recorded alongside AQS data sets, referred to by U.S. EPA as 'qualifiers', can be found at the following link: https://aqs.epa.gov/aqsweb/documents/codetables/qualifiers.html

Invalidation of reference data: AQS qualifiers are organized by qualifier type, which indicates whether data logged alongside qualifier flags should be invalidated (set null). Qualifiers with type "Null Data Qualifier" are invalidated, and includes data logged during periods that coincide with QC checks (e.g., "BF-Precision/Zero/Span", "BJ- Operator Error", "BL - QA Audit", "AZ - QC Audit") among other events such as power outages. Data logged alongside qualifiers with type "Quality Assurance Qualifiers" are not invalidated and are included in this analysis (e.g., concentrations less than the federal MDL for the reference monitor "MD – Value less than MDL", QA reviewed values "Validated Value").

Data Flags Recorded During Testing

FRM/FEM Monitor	Timestamp (UTC)	Flag
Teledyne API T640X (Acquired via AQS)	2020-02-21 19:00:00+0000	AZ - QC Audit
	2020-03-02 02:00:00+0000 to 2020-03-02 04:00:00+0000	V - Validated Value.
	2020-03-02 11:00:00+0000	V - Validated Value.

Testing Report - $PM_{2.5}$ Base Testing

Aeroqual AQY

This report reflects out-of-the-box performance

Initial Base Testing - RTP, NC
U.S. Environmental Protection Agency
Office of Research and Development
PI: Clements.Andrea@epa.gov
919-541-1363

August 2019—September 2019

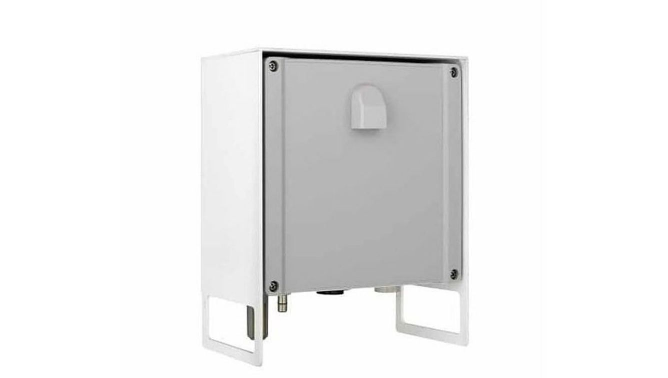

Image Attribution: Aeroqual Limited (Auckland, New Zealand)

Deployment Details

Testing Organization and Site Information	
Testing organization (Name, Organization type, Contact website)	U.S. Environmental Protection Agency - Office of Research and Development<br>Federal Government<br>Air Sensor Toolbox | U.S. EPA Website
Testing location (City, State, Latitude and Longitude)	Ambient Monitoring Innovative Research Station (AIRS)<br>RTP, NC<br>35.88951, -78.874572
AQS site ID	37 – 063 – 0099
Sampling timeframe (MM-DD-YY)	08-02-19 to 09-01-19
Sensor data source	Aeroqual Cloud download
Reference data source	OAQPS file transfer

Sensor Information			
Manufacturer, model	Aeroqual AQY		
Device firmware version	1.14.2		
Sampling time interval	1-minute		
Sensor serial numbers	AQY_01	AQY_02	AQY_03
Issues encountered during deployment?	☑	See NC-AQY-Page 6 of this testing report	

FRM/FEM Information	
Manufacturer, model, designation	Teledyne Advanced Pollution Instrumentation T640 FEM
Sampling time interval	1-hour averaging
Date of calibration	As required by 40 CFR Part 58 and the Burdens Creek QAPP maintained by OAQPS
Date of flowrate verification check	Monthly as required by 40 CFR Part 58 Appendix A
Description, date(s) of maintenance activities	N/A

Time Series Plots

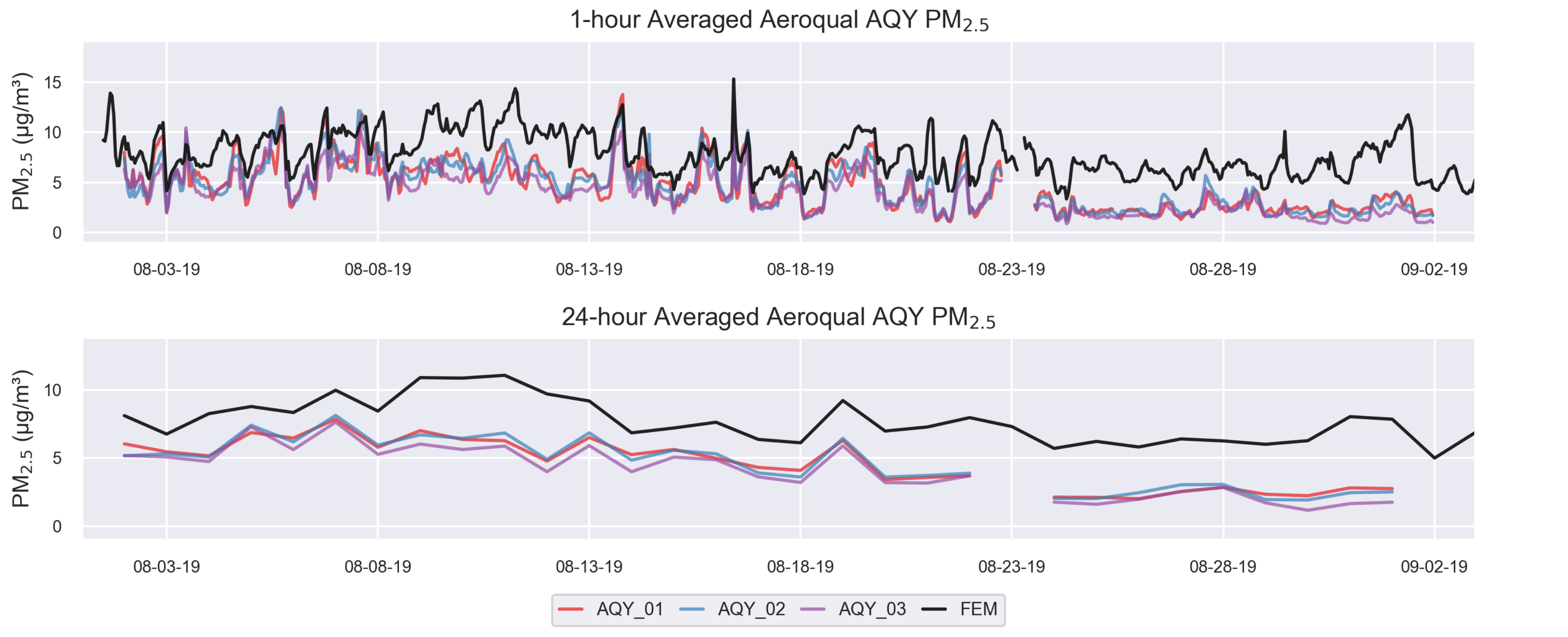

Scatter Plots: Comparison to FRM/FEM

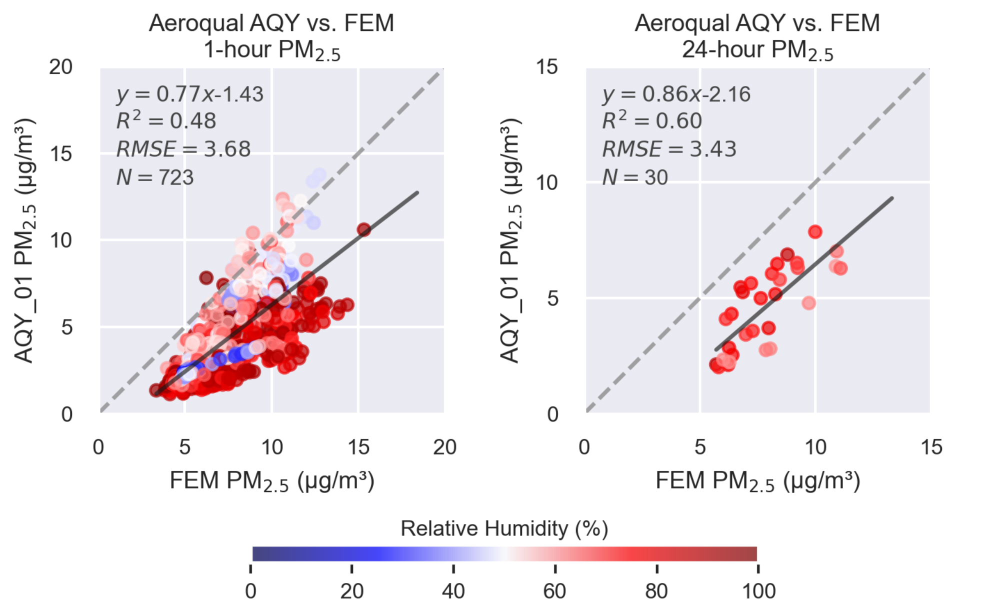

Range of FRM/FEM concentrations over duration of base test (μg/m³)	3.4-15.3 (1-hr), 5.7-11.1 (24-hr)
Number of 24-hr periods in FRM/FEM monitor measurements with a goal concentration ≥ 25 μg/m³	0

Performance Metrics★

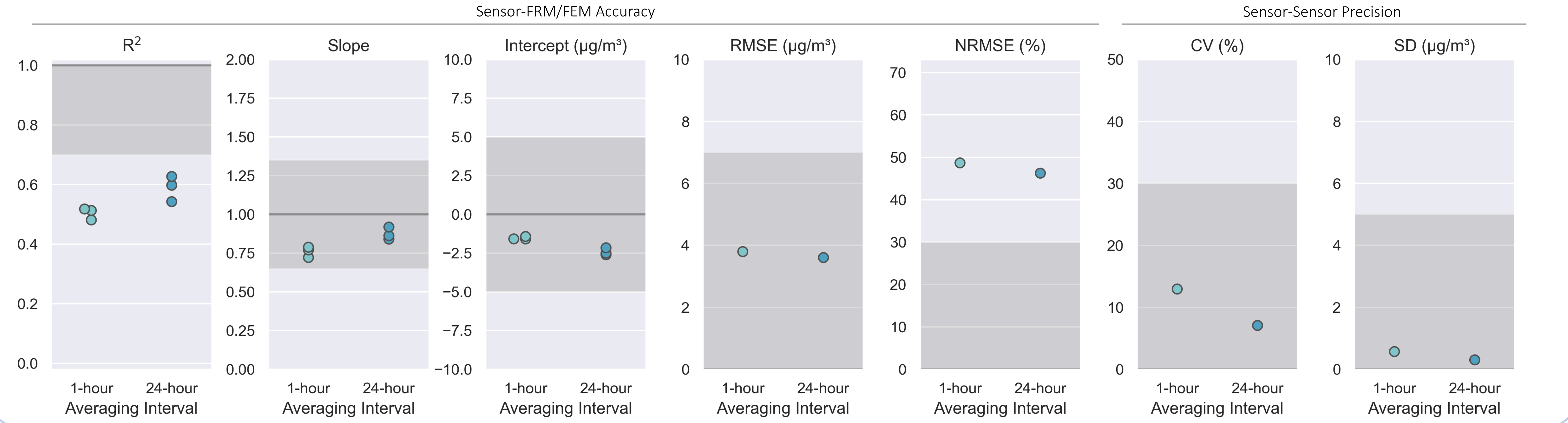

Meteorological Conditions During Deployment

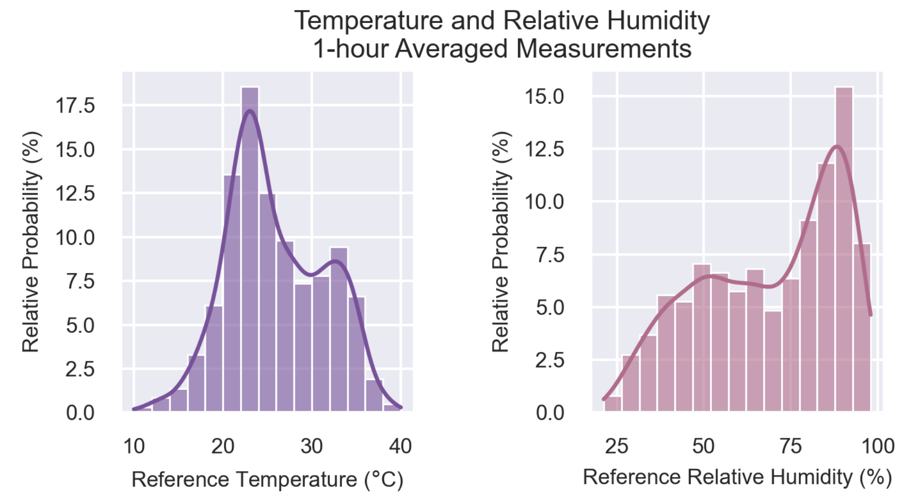

Number of 24-hr periods outside sensor manufacture-listed temperature operational range (-10 to 40 °C)	0
Number of 24-hr periods outside sensor manufacture-listed relative humidity operational range (no operational range specified)	-

Meteorological Influence

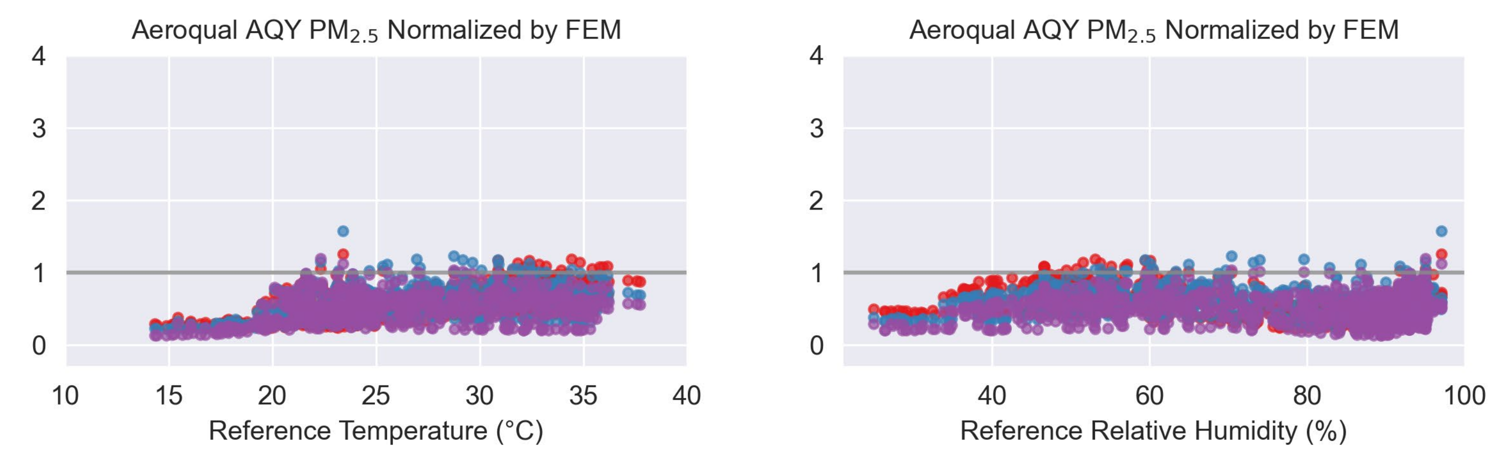

Mean number of paired, normalized concentration and temperature values (1-hr averages)	741
Mean number of paired, normalized concentration and relative humidity values (1-hr averages)	741

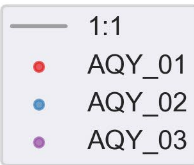

★For evaluations with greater than three sensors, grouping individual sensor metrics into boxplots is recommended for displaying results. Note that this recommendation does not apply to metrics computed as a single value for all sensors over the whole evaluation group, such as RMSE, NRMSE, CV, and standard deviation.

Testing Report - $PM_{2.5}$ Base Testing

Aeroqual AQY

This report reflects out-of-the-box performance

Initial Base Testing - RTP, NC

U.S. Environmental Protection Agency
Office of Research and Development
PI: Clements.Andrea@epa.gov
919-541-1363

August 2019—September 2019

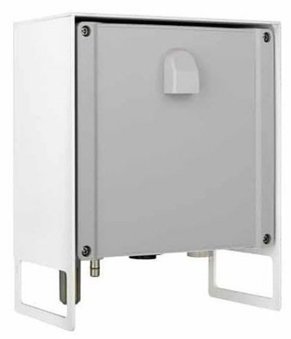

Image Attribution: Aeroqual Limited (Auckland, New Zealand)

Tabular Statistics

Sensor-FRM/FEM Correlation

	Bias and Linearity						Data Quality			
	R^2		Slope		Intercept (µg/m³)		Uptime (%)		Number of paired sensor and FRM/FEM concentration values	
	1-Hour ○○○	24-Hour ○○○	1-Hour ●●●	24-Hour ●●●	1-Hour ●●●	24-Hour ●●●	1-Hour ●●●	24-Hour ●●●	1-Hour	24-Hour
Metric Target Range	≥ 0.70	≥ 0.70	1.0 ± 0.35	1.0 ± 0.35	-5 ≤ b ≤ 5	-5 ≤ b ≤ 5	75%*	75%*	-	-
Sensor AQY_01	0.48	0.60	0.77	0.86	-1.43	-2.16	97	97	723	30
Sensor AQY_02	0.51	0.63	0.79	0.92	-1.59	-2.61	98	97	725	30
Sensor AQY_03	0.52	0.54	0.72	0.84	-1.58	-2.51	98	97	725	30
Mean	0.50	0.59	0.76	0.87	-1.53	-2.42	97	97	724	30

	Error			
	RMSE (µg/m³)		NRMSE (%)	
	1-Hour ★	24-Hour ★	1-Hour ☆	24-Hour ☆
Metric Target Range	≤ 7.0	≤ 7.0	≤ 30.0	≤ 30.0
Deployment Value	3.8	3.6	48.7	46.3

Device-specific metrics (computed for each sensor in evaluation)
○○○ Metric value for none of devices tested falls within the target range
●○○ Metric value for one of devices tested falls within the target range
●●○ Metric value for two of devices tested falls within the target range
●●● Metric value for three of devices tested falls within the target range

Single-valued metrics (computed via entire evaluation dataset)
☆ Indicates that the metric value is not within the target range
★ Indicates that the metric value is within the target range

Sensor-Sensor Precision

	Precision (between collocated sensors)				Data Quality	
	CV (%)		SD (µg/m³)		Number of concurrent sensor concentration pairs	
	1-Hour ★	24-Hour ★	1-Hour ★	24-Hour ★	1-Hour	24-Hour
Metric Target Range	≤ 30.0	≤ 30.0	≤ 5.0	≤ 5.0	-	-
Deployment Value	13.0	7.1	0.6	0.3	724	30

*This value is only a recommendation for ensuring data quality and is not included in the list of target values discussed in Section 4 of the Performance Testing Protocols, Metrics, and Target Values for Fine Particulate Matter Air Sensors document.

Testing Report - $PM_{2.5}$ Base Testing

Aeroqual AQY

This report reflects out-of-the-box performance

Initial Base Testing - RTP, NC
U.S. Environmental Protection Agency
Office of Research and Development
PI: Clements.Andrea@epa.gov
919-541-1363
August 2019—September 2019

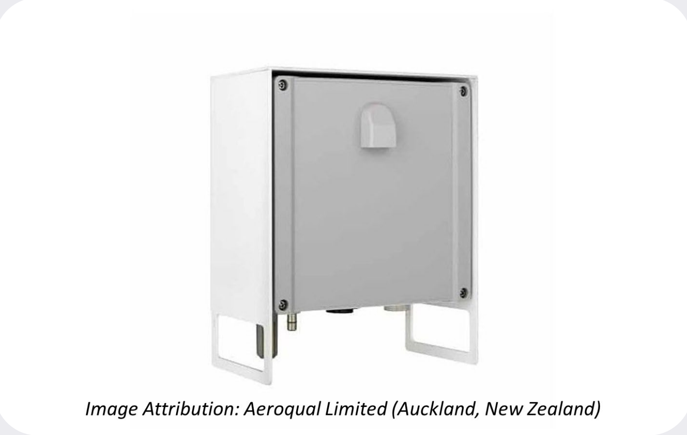

Sensor-FRM/FEM Scatter Plots

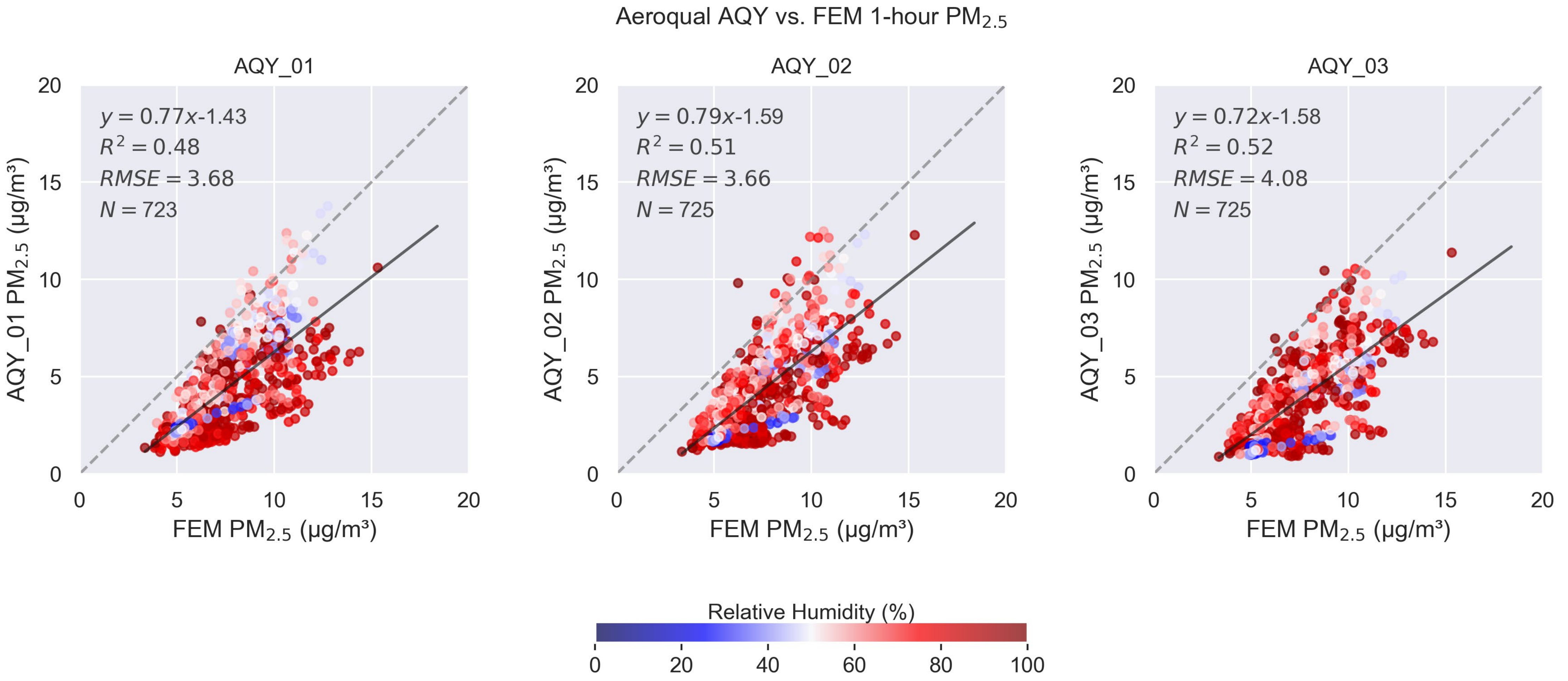

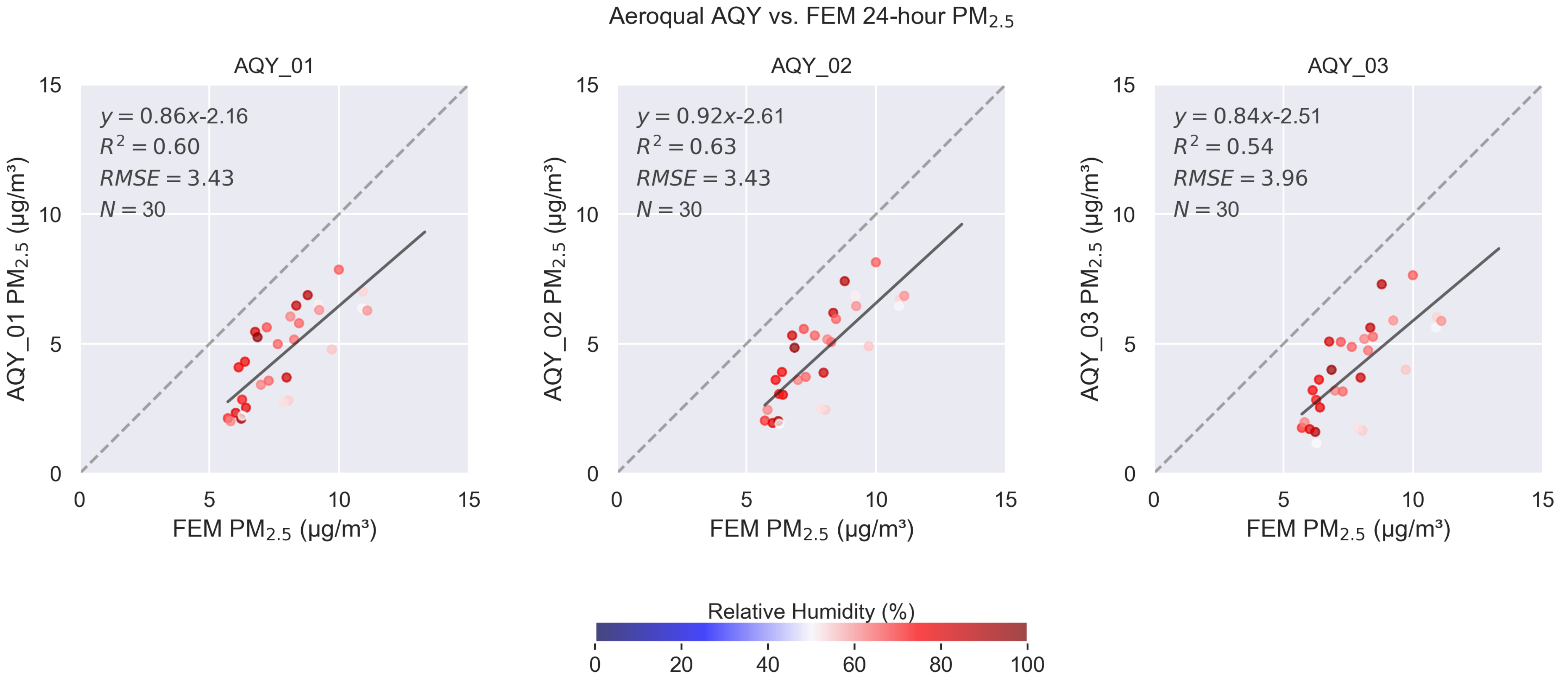

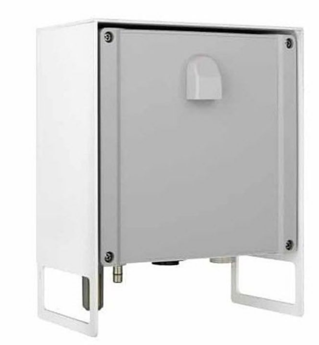

Image Attribution: Aeroqual Limited (Auckland, New Zealand)

Supplemental Information

Abbreviations used in Supplemental Information

FRM Federal Reference Method
FEM Federal Equivalent Method
SOP Standard Operating Procedure
QAPP Quality Assurance Project Plan
QC Quality Control

Supplemental Documentation	Attached	Description & URL or file path to documentation
Field observations and sensor data flags	☑	See NC-AQY-Page 6 of this testing report
Maintenance logs	☐	No logs recorded during testing
Standard operating procedure(s)	☐	U.S. EPA Office Of Research and Development SOP available upon request
Photos of equipment setup and testing	☑	See NC-AQY-Page 5 of this testing report
Product specifications sheet(s)	☐	N/A*
Product manual(s)	☐	N/A*
Data storage and transmission method	☑	See NC-AQY-Page 6 of this testing report
Data correction approach	☑	See NC-AQY-Page 6 of this testing report
Issues encountered	☑	See NC-AQY-Page 6 of this testing report
Data analysis/correction scripts and version	☑	Averaging and processing of data, calculation of performance metrics, and generation of figures and other supplementary material for analysis were obtained using Python 3.9.7 with the packages sensortoolkit v0.8.3b2, pandas 1.3.5, NumPy 1.21.2, Matplotlib 3.5.0, statsmodels 0.13.0, and seaborn 0.11.2. All packages are available from the Python Package Index (PyPI) at https://pypi.org. The integrated development environment (IDE) Spyder 5.1.5 was used for scripting and data visualization. Version control for the Python base, packages, and IDE were all managed by conda 4.11.0.
Air Monitoring Station QAPP	☐	U.S. EPA Office Of Research and Development QAPP available upon request
Summary of FRM/FEM monitor QC checks	☑	See NC-AQY-Page 7 of this testing report
Manufacturer website for FRM/FEM monitor	☑	Teledyne API: T640/T640X Product website
FRM/FEM monitor manual	☑	Teledyne API: T640/T640X Product Manual
FRM/FEM monitor specifications sheet(s)	☑	Teledyne API: T640/T640X Specification Sheet
Other documents	☑	Manufacturer notice of AQY sales on hold

*As of 3/18/2021, the manufacturer of the AQY has placed sales of a similar unit on hold. Documentation for the AQY is currently unavailable from the manufacturer's website.

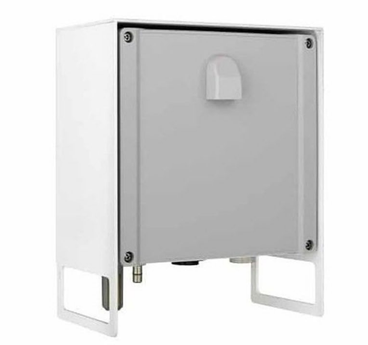

Image Attribution: Aeroqual Limited (Auckland, New Zealand)

Supplemental Information: Photos of Testing Site and Equipment Setup

Site Description:
The Burdens Creek Ambient Monitoring Innovation Research Station (AIRS) site is located on the U.S. EPA, RTP campus and is situated between Alexander Drive and Route 147. The site is intended to represent a neighborhood-scale site as defined in *40 CFR Part 58, Appendix D*. U.S. EPA's Office of Air Quality Planning and Standards (OAQPS) operates reference grade instruments in a free-standing shelter situated directly adjacent to the sensor testing platform.

Figure 1: Aeroqual AQY sensors (indicated by red arrow) attached to mounting assembly at evaluation site.

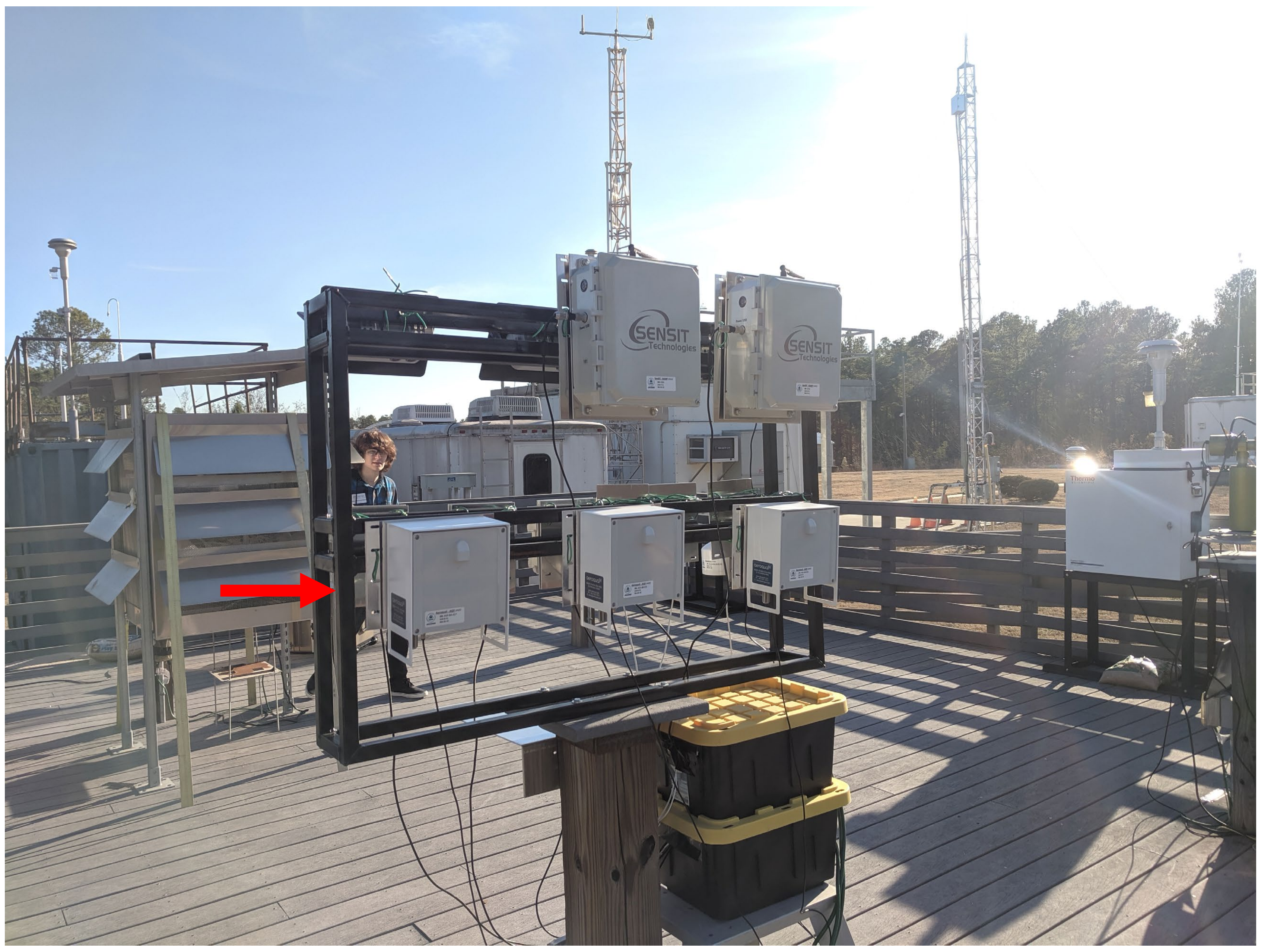

Figure 2: Observation deck at evaluation site. Approximate location of sensors indicated by the red arrow, the approximate location of T640x FEM (housed within sampling trailer) indicated by the black arrow. Sensors and FEM instrumentation are separated by approximately 15 meters.

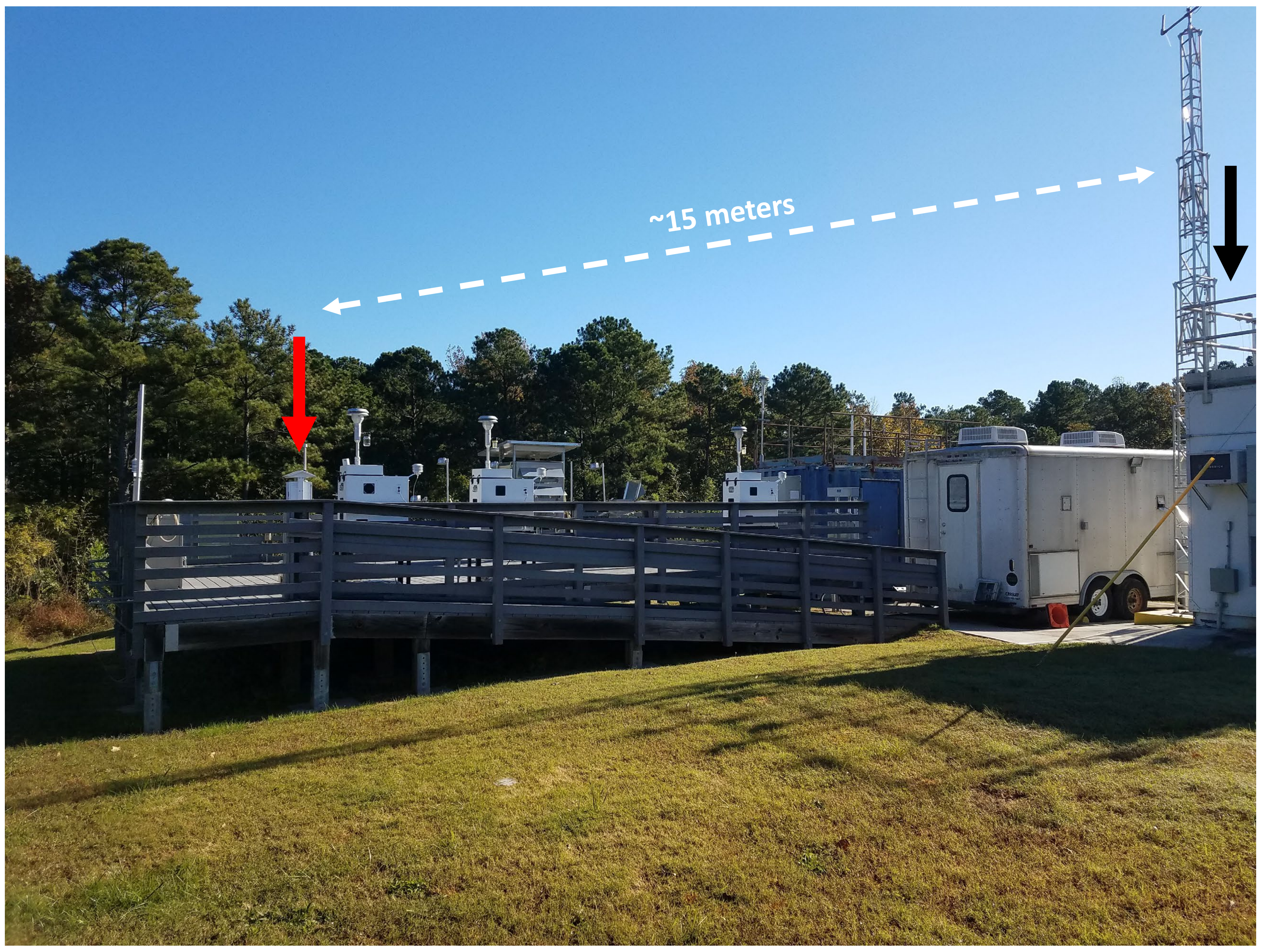

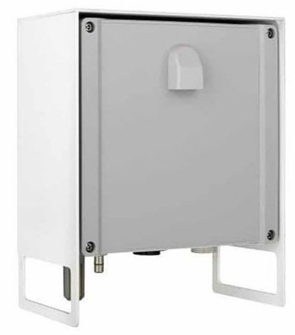

Image Attribution: Aeroqual Limited (Auckland, New Zealand)

Supplemental Information: Data Storage, Correction Approach, and Issues Encountered

Data Storage and Transmission Method

For this study, SIM cards were obtained from Aeroqual and installed in units with serials numbers beginning with BB. Units with serial numbers beginning with BA were connected to Wi-Fi via a mobile hotspot. Data from all units flowed to the Aeroqual Cloud. The 1-minute raw data was acquired weekly using the Aeroqual Cloud user interface (UI). The AQY possesses an internal data storage USB flash drive as a data backup, however the software on the drive is proprietary to Aeroqual and unreadable by outside systems.

Data Correction Approach

This evaluation report reflects "out-of-the-box" performance of the AQY. The manufacturer provides a procedure by which local collocation (sensor operated along side an FRM/FEM) data can be collected, a gain (slope) and offset (intercept) determined, and parameters entered into the Aeroqual Cloud user interface to be applied to all subsequently collected data. This procedure and feature has NOT been conducted prior to this evaluation. Prospective consumers may get better performance from this device if they utilize this feature.

After acquisition, the raw data was processed using the *sensortoolkit* python code library (v0.8.3b2). A continuous data set at the recorded sampling frequency was written to a .csv file. 1-hour and 24-hour averaged data sets were generated using a 75% completeness threshold and saved as separate .csv files. Outliers were NOT removed from data sets in order to assess "out-of-the-box" sensor performance.

Issues Encountered

Pre-deployment observations

- *Timestamp inaccuracies*: During the pre-deployment phase, it was observed that the AQY devices did not store the Real Time Clock's (RTC) time. For the units to maintain accurate time, they needed to be connected to the internet after each shutdown event. Since data could be downloaded over Wi-Fi while the sensor pods remained operational, the units were connected to a mobile hotspot at the beginning of the evaluation.
- *Hotspot connectivity*: The process of connecting the AQYs to a hotspot required their Wi-Fi adapter's operating mode to be changed from "Access Point" to "Client" and the hotspot's service set identifier (SSID) and password needed to be provided to the AQY's configuration. The repeatability and reproducibility of the processes to acquire internet time and subsequent data extraction were verified for instruments prior to deployment.

Field observations and sensor data flags

Aeroqual AQY units were deployed at the AIRS monitoring site on 8/1/2019. On 8/22/2019, a power outage was reported which extended into the following day at 11:52 AM. During this period, none of the AQY units were able to recorded data. A subsequent outage was reported for AQY unit AQY_01 on 8/25/2019 and lasted less than two hours. The field technician was not at the monitoring site when either outages were reported, indicating that the events were likely not caused by operator error.

The following table contains data flags describing events that were encountered during the testing period.

Start Time (UTC)	End Time (UTC)	Sensor Serial ID	Parameters Impacted	Flag
2019-08-22 18:26:00+00:00	2019-08-23 11:52:00+00:00	AQY_02	ALL	9.0-Data loss - Power connection error
2019-08-22 18:26:00+00:00	2019-08-23 11:52:00+00:00	AQY_03	ALL	9.0-Data loss - Power connection error
2019-08-22 18:26:00+00:00	2019-08-23 11:52:00+00:00	AQY_01	ALL	9.0-Data loss - Power connection error
2019-08-25 08:50:00+00:00	2019-08-25 10:17:00+00:00	AQY_01	ALL	9.0-Data loss - Power connection error

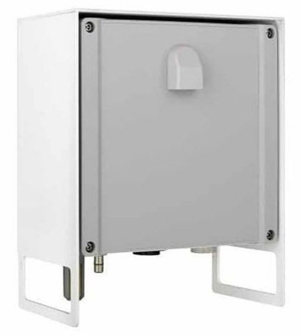

Image Attribution: Aeroqual Limited (Auckland, New Zealand)

Description of Data Flags

OAQPS manages data logged by reference monitors at the AIRS evaluation site using the Envidas data acquisition system software from DR DAS LTD[1]. Envidas contains over 100 data flags which are configurable by the monitoring agency and can be triggered based on instrument status. Appendix B Table 1 contains a description of Envidas data flags including the numeric code and status code name (a brief textual description, the table containing data flags below includes code names for events encountered by the reference monitor under the "Flag" column). Appendix B Table 1 also indicates whether reference data are invalidated for a given data flag. For this report, reference data were invalidated for periods where a data flag was logged and if the corresponding entry for the data flag in the "Data Status Invalidation" column in Appendix B Table 1 indicates data should be invalidated.

Data Flags Recorded During Testing

FRM/FEM Monitor	Timestamp (UTC)	Flag
Teledyne API T640X (Data acquired via local transfer from OAQPS)	2019-08-08 14:02:00+0000	<Samp
	2019-08-13 14:58:00+0000	<Samp
	2019-08-21 12:56:00+0000 to 2019-08-21 13:29:00+0000	Down
	2019-08-21 13:30:00+0000	<Samp
	2019-08-21 21:29:00+0000 to 2019-08-21 21:34:00+0000	<Samp
	2019-08-21 21:30:00+0000 to 2019-08-21 21:33:00+0000	Down
	2019-08-23 04:28:00+0000 to 2019-08-23 07:12:00+0000	NoData
	2019-08-23 07:13:00+0000	<Samp
	2019-08-26 21:18:00+0000	<Samp
	2019-08-28 15:07:00+0000	<Samp

Meteorological Instrument	Timestamp (UTC)	Flag
RM Young 41382 VC Temperature and Relative Humidity Monitor (Data acquired via local transfer from OAQPS)	2019-08-08 14:02:00+0000	<Samp
	2019-08-13 14:58:00+0000	<Samp
	2019-08-23 04:28:00+0000 to 2019-08-23 07:12:00+0000	NoData
	2019-08-23 07:13:00+0000	<Samp
	2019-08-26 21:18:00+0000	<Samp
	2019-08-28 15:07:00+0000	<Samp

[1] "Contact us" DR DAS LTD, 2022, https://dr-das.com/Home/Contact.html Accessed June 3 2022

Testing Report - $PM_{2.5}$ Base Testing

APT Maxima

This report reflects out-of-the-box performance

Initial Base Testing - RTP, NC

U.S. Environmental Protection Agency
Office of Research and Development
PI: Clements.Andrea@epa.gov
919-541-1363

May 2019—June 2019

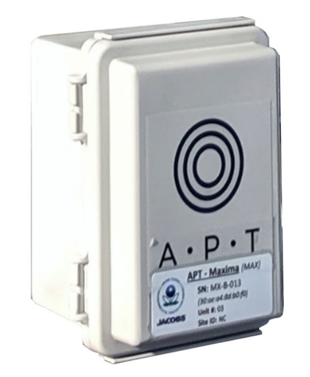

Deployment Details

Testing Organization and Site Information	
Testing organization (Name, Organization type, Contact website)	U.S. Environmental Protection Agency - Office of Research and Development<br>Federal Government<br>Air Sensor Toolbox | U.S. EPA Website
Testing location (City, State, Latitude and Longitude)	Ambient Monitoring Innovative Research Station (AIRS)<br>RTP, NC<br>35.88951, -78.874572
AQS site ID	37 – 063 – 0099
Sampling timeframe (MM-DD-YY)	08-02-19 to 09-01-19
Sensor data source	Onboard MicroSD card
Reference data source	OAQPS file transfer

Sensor Information			
Manufacturer, model	APT Maxima		
Device firmware version	v5.0 (installed 5/2/2019)		
Sampling time interval	30-seconds		
Sensor serial numbers	MAX_02	MAX_03	MAX_04
	MAX_05	MAX_06	MAX_07
	MAX_08	MAX_09	
Issues encountered during deployment?	☐	Issues with deployment	

FRM/FEM Information	
Manufacturer, model, designation	Teledyne Advanced Pollution Instrumentation T640 FEM
Sampling time interval	1-hour averaging
Date of calibration	As required by 40 CFR Part 58 and the Burdens Creek QAPP maintained by OAQPS
Date of flowrate verification check	Monthly as required by 40 CFR Part 58 Appendix A
Description, date(s) of maintenance activities	N/A

Time Series Plots

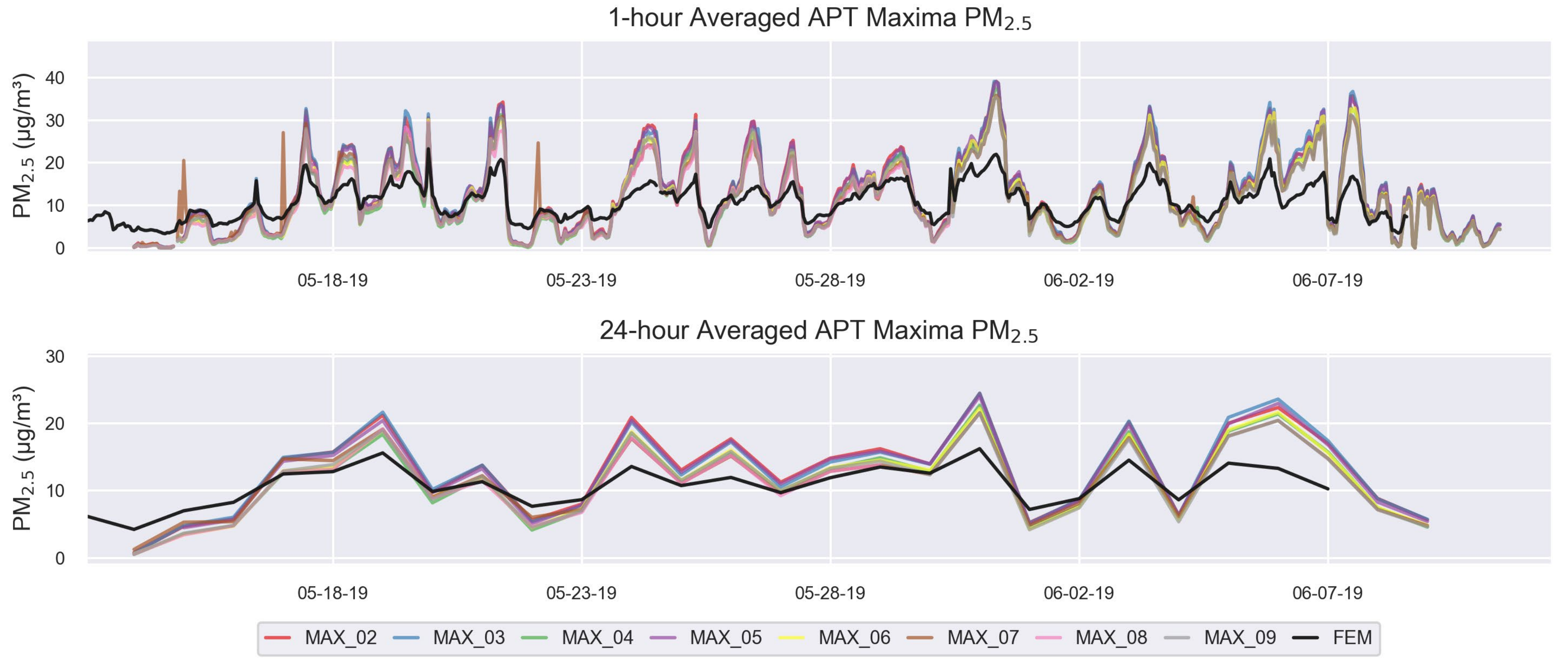

Scatter Plots: Comparison to FRM/FEM

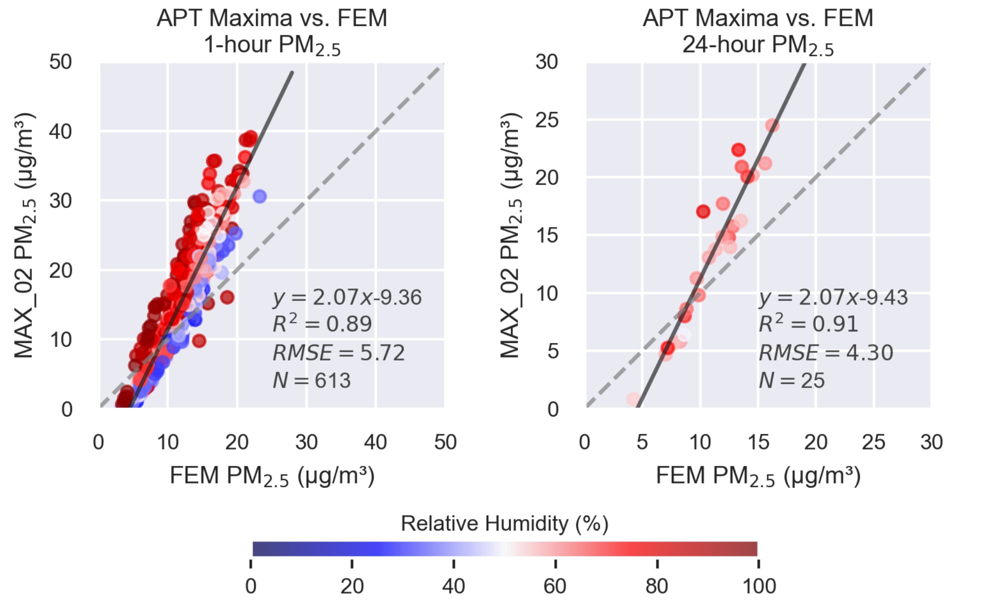

Range of FRM/FEM concentrations over duration of base test (µg/m³)	3.4-23.3 (1-hr), 7.0-16.2 (24-hr)
Number of 24-hr periods in FRM/FEM monitor measurements with a goal concentration ≥ 25 µg/m³	0

Performance Metrics★

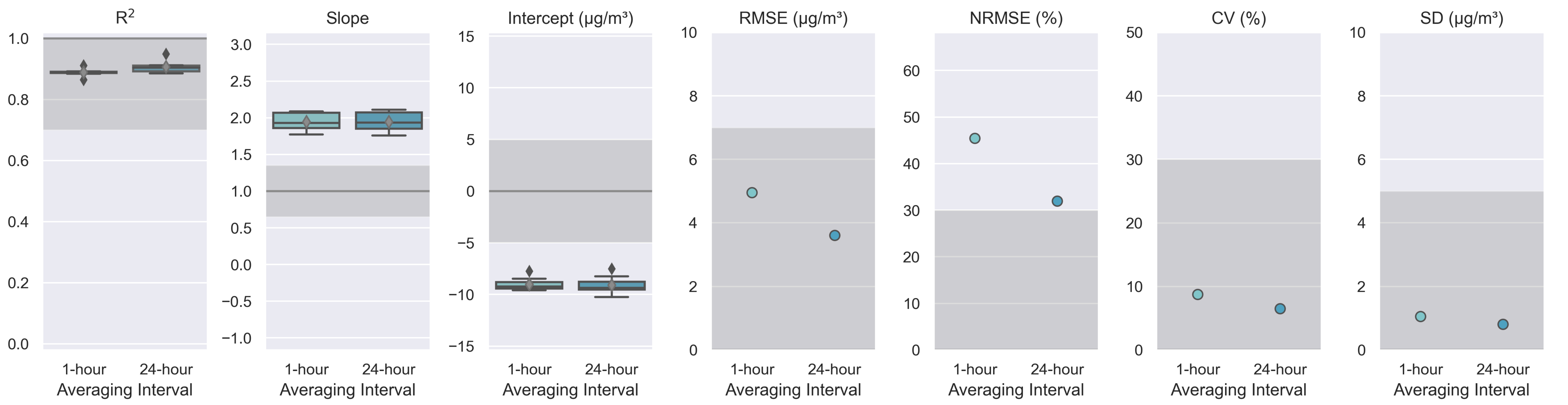

Meteorological Conditions During Deployment

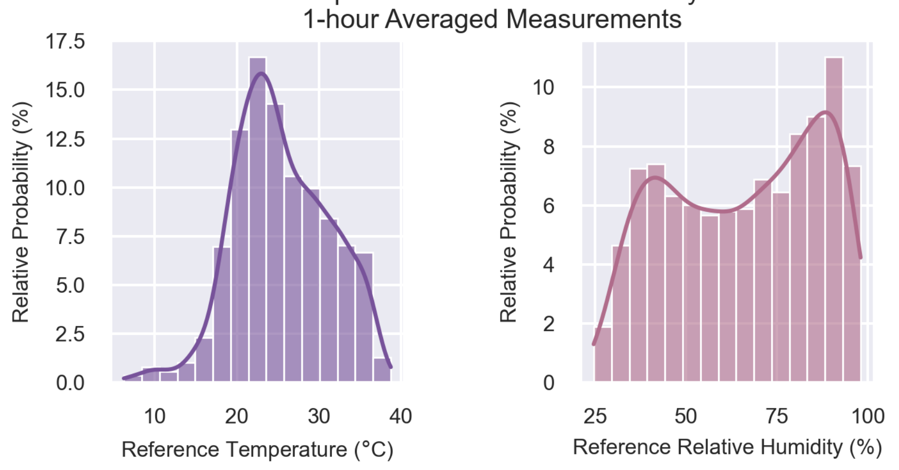

Number of 24-hr periods outside sensor manufacturer-listed temperature operational range (no operational range specified)	-
Number of 24-hr periods outside sensor manufacturer-listed relative humidity operational range (no operational range specified)	-

Meteorological Influence

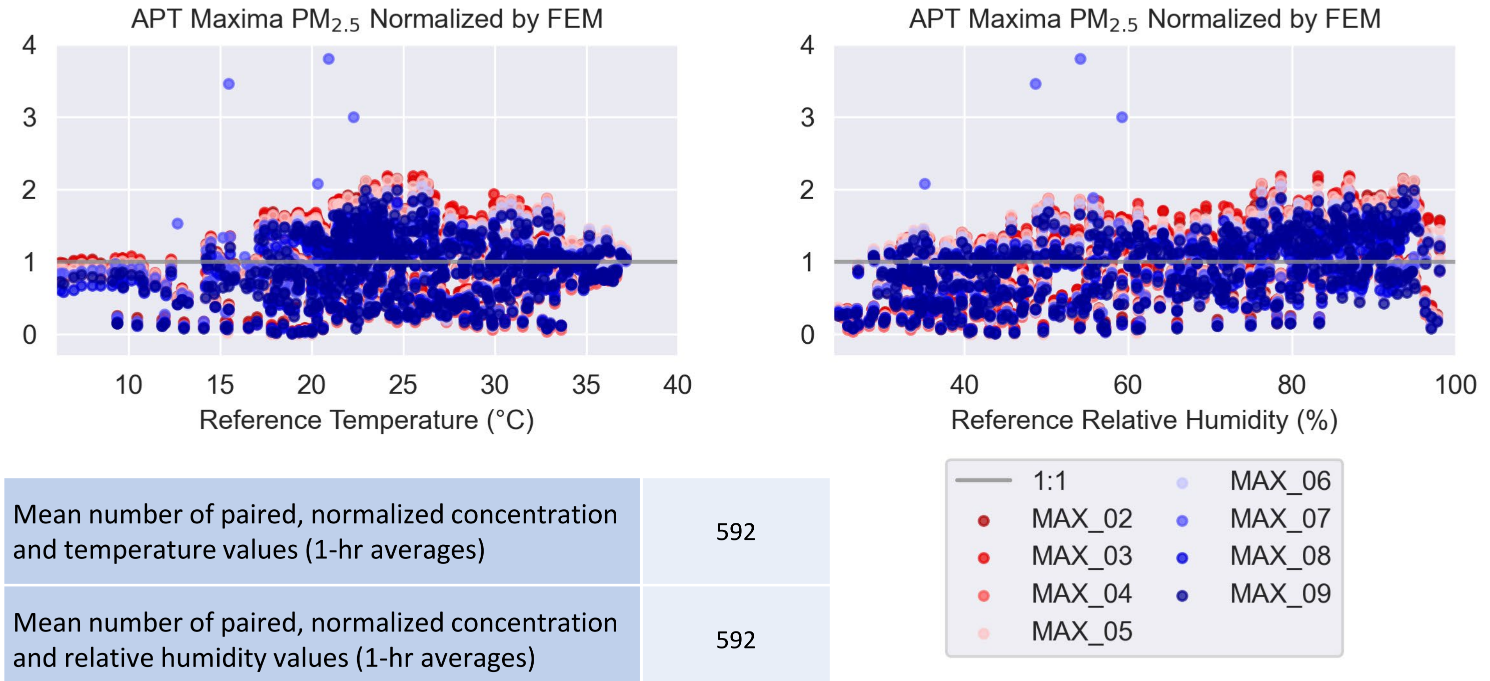

Mean number of paired, normalized concentration and temperature values (1-hr averages)	592
Mean number of paired, normalized concentration and relative humidity values (1-hr averages)	592

★For evaluations with greater than three sensors, grouping individual sensor metrics into boxplots is recommended for displaying results. Note that this recommendation does not apply to metrics computed as a single value for all sensors over the whole evaluation group, such as RMSE, NRMSE, CV, and standard deviation.

Testing Report - $PM_{2.5}$ Base Testing

APT Maxima

This report reflects out-of-the-box performance

Initial Base Testing - RTP, NC
U.S. Environmental Protection Agency
Office of Research and Development
PI: Clements.Andrea@epa.gov
919-541-1363
May 2019—June 2019

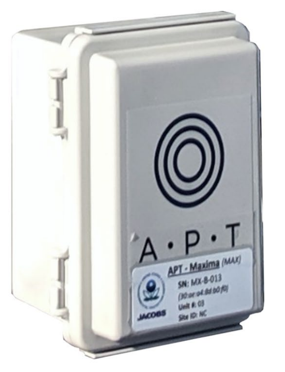

Tabular Statistics

Sensor-FRM/FEM Correlation

	Bias and Linearity						Data Quality			
	R^2		Slope		Intercept (μg/m³)		Uptime (%)		Number of paired sensor and FRM/FEM concentration values	
	1-Hour ●●●●●●●●	24-Hour ●●●●●●●●	1-Hour ○○○○○○○○	24-Hour ○○○○○○○○	1-Hour ○○○○○○○○	24-Hour ○○○○○○○○	1-Hour ●●●●●●●●	24-Hour ●●●●●●●●	1-Hour	24-Hour
Metric Target Range	≥ 0.70	≥ 0.70	1.0 ± 0.35	1.0 ± 0.35	-5 ≤ b ≤ 5	-5 ≤ b ≤ 5	75%*	75%*	-	-
Sensor MAX_02	0.89	0.91	2.07	2.07	-9.36	-9.43	100	96	613	25
Sensor MAX_03	0.89	0.90	2.09	2.09	-9.58	-9.76	100	96	613	25
Sensor MAX_04	0.88	0.89	1.94	1.93	-9.36	-9.40	100	96	613	25
Sensor MAX_05	0.89	0.88	2.06	2.11	-9.60	-10.24	98	93	600	24
Sensor MAX_06	0.89	0.89	1.92	1.93	-9.11	-9.31	100	96	613	25
Sensor MAX_07	0.86	0.91	1.80	1.77	-7.75	-7.51	100	96	613	25
Sensor MAX_08	0.91	0.95	1.77	1.76	-8.47	-8.24	99	94	393	16
Sensor MAX_09	0.89	0.91	1.88	1.88	-8.91	-8.96	100	96	613	25
Mean	0.89	0.91	1.94	1.94	-9.02	-9.10	99	96	584	24

	Error			
	RMSE (μg/m³)		NRMSE (%)	
	1-Hour ★	24-Hour ★	1-Hour ☆	24-Hour ☆
Metric Target Range	≤ 7.0	≤ 7.0	≤ 30.0	≤ 30.0
Deployment Value	5.0	3.6	45.5	32.0

Device-specific metrics (computed for each sensor in evaluation)
○○○ Metric value for none of devices tested falls within the target range
●○○ Metric value for one of devices tested falls within the target range
●●○ Metric value for two of devices tested falls within the target range
●●● Metric value for three of devices tested falls within the target range

Single-valued metrics (computed via entire evaluation dataset)
☆ Indicates that the metric value is not within the target range
★ Indicates that the metric value is within the target range

Sensor-Sensor Precision

	Precision (between collocated sensors)				Data Quality	
	CV (%)		SD (μg/m³)		Number of concurrent sensor concentration pairs	
	1-Hour ★	24-Hour ★	1-Hour ★	24-Hour ★	1-Hour	24-Hour
Metric Target Range	≤ 30.0	≤ 30.0	≤ 5.0	≤ 5.0	-	-
Deployment Value	8.8	6.5	1.1	0.8	646	26

*This value is only a recommendation for ensuring data quality and is not included in the list of target values discussed in Section 4 of the Performance Testing Protocols, Metrics, and Target Values for Fine Particulate Matter Air Sensors document.

Testing Report - $PM_{2.5}$ Base Testing

APT Maxima

This report reflects out-of-the-box performance

Initial Base Testing - RTP, NC
U.S. Environmental Protection Agency
Office of Research and Development
PI: Clements.Andrea@epa.gov
919-541-1363
May 2019—June 2019

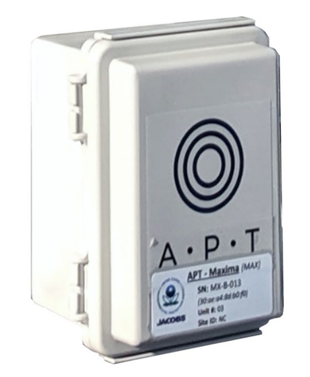

Sensor-FRM/FEM Scatter Plots

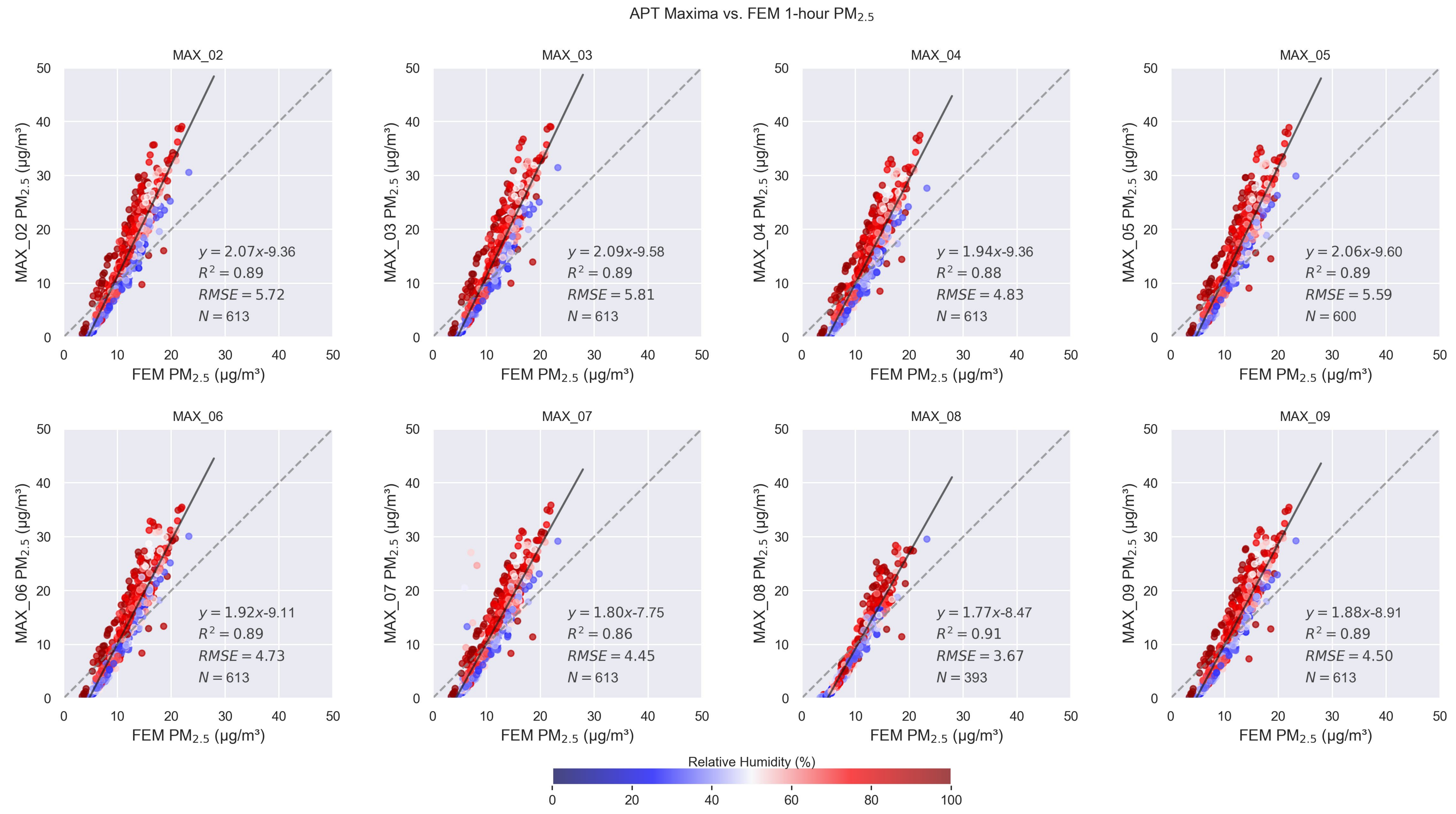

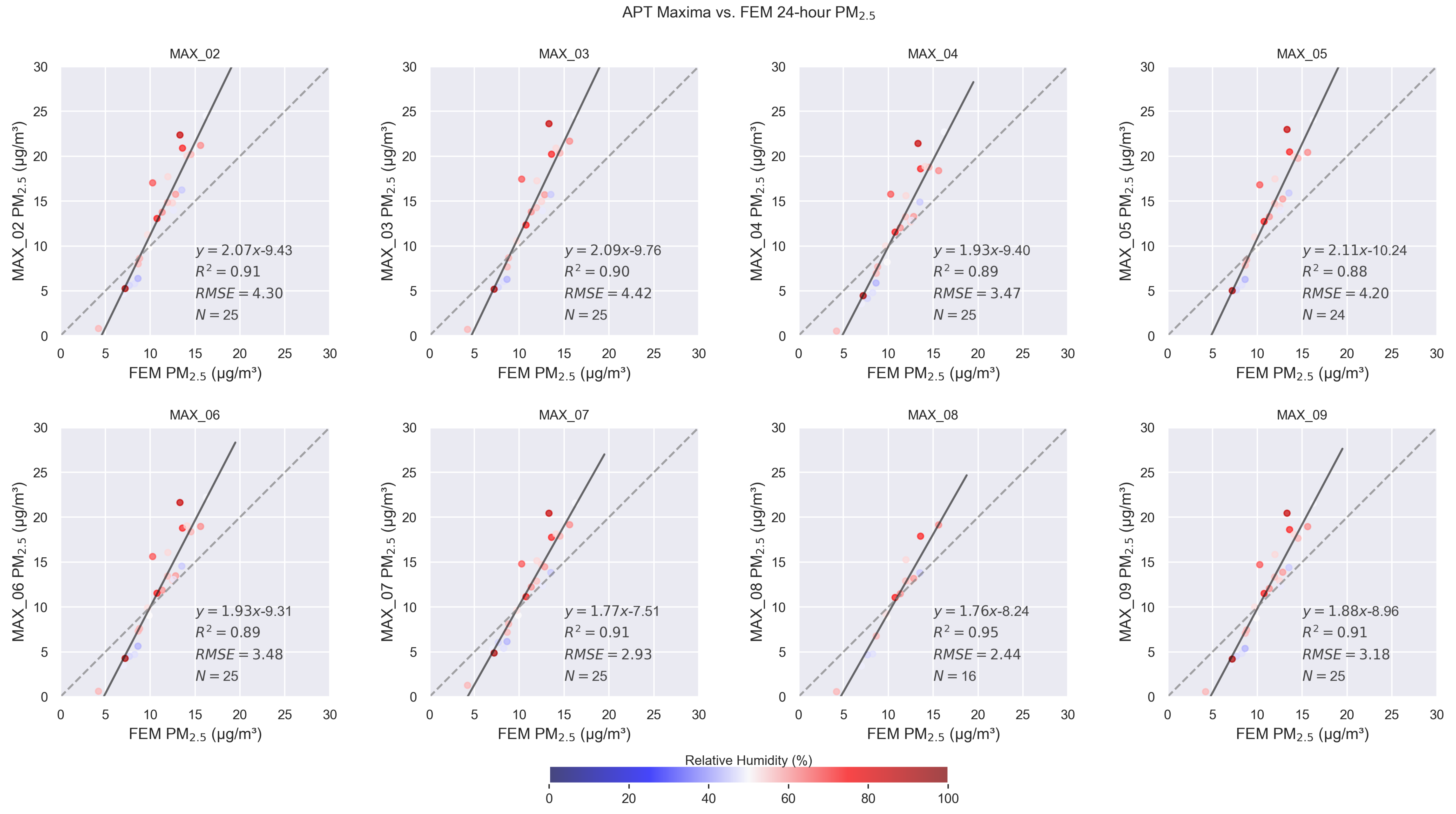

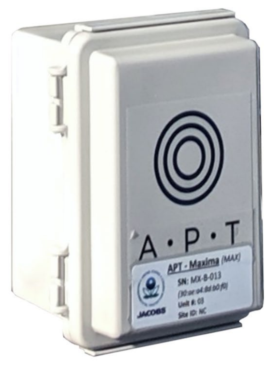

Supplemental Information

Abbreviations used in Supplemental Information

FRM	Federal Reference Method
FEM	Federal Equivalent Method
SOP	Standard Operating Procedure
QAPP	Quality Assurance Project Plan
QC	Quality Control

Supplemental Documentation	Attached	Description & URL or file path to documentation
Field observations and sensor data flags	☑	See NC-MAX-Page 6 of this testing report
Maintenance logs	☐	No logs recorded during testing
Standard operating procedure(s)	☐	U.S. EPA Office Of Research and Development SOP available upon request
Photos of equipment setup and testing	☑	See NC-MAX-Page 5 of this testing report
Product specifications sheet(s)	☐	N/A
Product manual(s)	☐	N/A
Data storage and transmission method	☑	See NC-MAX-Page 6 of this testing report
Data correction approach	☑	See NC-MAX-Page 6 of this testing report
Issues encountered	☑	See NC-MAX-Page 6 of this testing report. No issues were encountered during testing, however, an abnormality in the duration of sampling intervals was noted during the pre-deployment phase.
Data analysis/correction scripts and version	☑	Averaging and processing of data, calculation of performance metrics, and generation of figures and other supplementary material for analysis were obtained using Python 3.9.7 with the packages sensortoolkit v0.8.3b2, pandas 1.3.5, NumPy 1.21.2, Matplotlib 3.5.0, statsmodels 0.13.0, and seaborn 0.11.2. All packages are available from the Python Package Index (PyPI) at https://pypi.org. The integrated development environment (IDE) Spyder 5.1.5 was used for scripting and data visualization. Version control for the Python base, packages, and IDE were all managed by conda 4.11.0.
Air Monitoring Station QAPP	☐	U.S. EPA Office Of Research and Development QAPP available upon request
Summary of FRM/FEM monitor QC checks	☑	See NC-MAX-Page 7, 8 of this testing report
Manufacturer website for FRM/FEM monitor	☑	Teledyne API: T640/T640X Product website
FRM/FEM monitor manual	☑	Teledyne API: T640/T640X Product Manual
FRM/FEM monitor specifications sheet(s)	☑	Teledyne API: T640/T640X Specification Sheet
Other documents	☐	

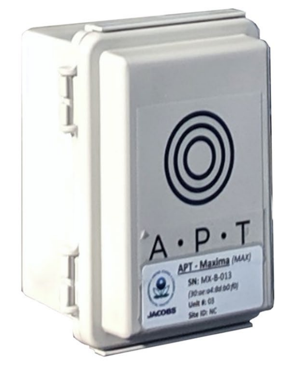

Site Description:

The Burdens Creek Ambient Monitoring Innovation Research Station (AIRS) site is located on the U.S. EPA, RTP campus and is situated between Alexander Drive and Route 147. The site is intended to represent a neighborhood-scale site as defined in *40 CFR Part 58, Appendix D*. U.S. EPA's Office of Air Quality Planning and Standards (OAQPS) operates reference grade instruments in a free-standing shelter situated directly adjacent to the sensor testing platform.

Figure 1: APT Maxima sensors (indicated by red arrow) attached to mounting assembly at evaluation site.

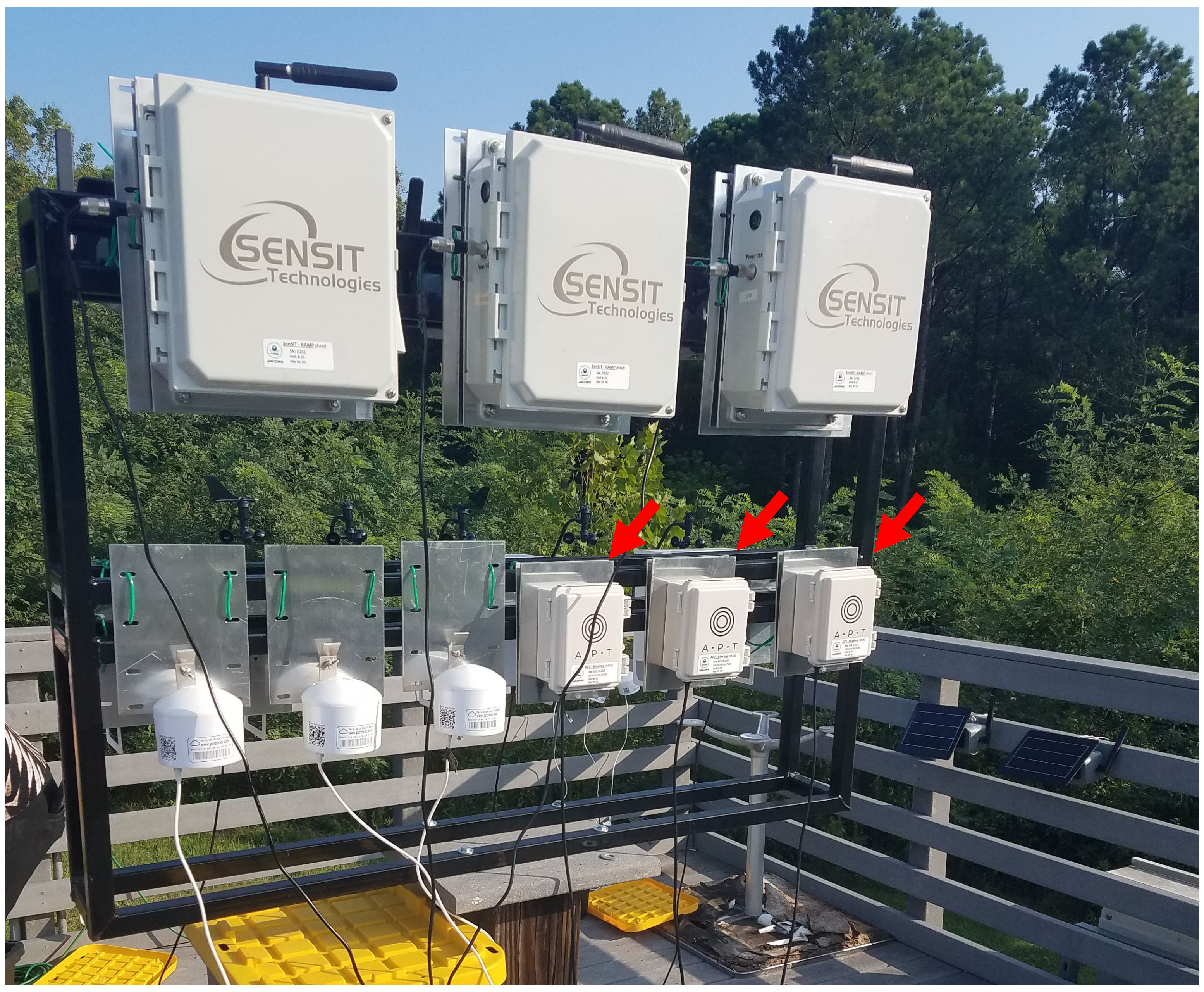

Figure 2: Observation deck at evaluation site. Approximate location of sensors indicated by the red arrow, the approximate location of T640x FEM (housed within sampling trailer) indicated by the black arrow. Sensors and FEM instrumentation are separated by approximately 15 meters.

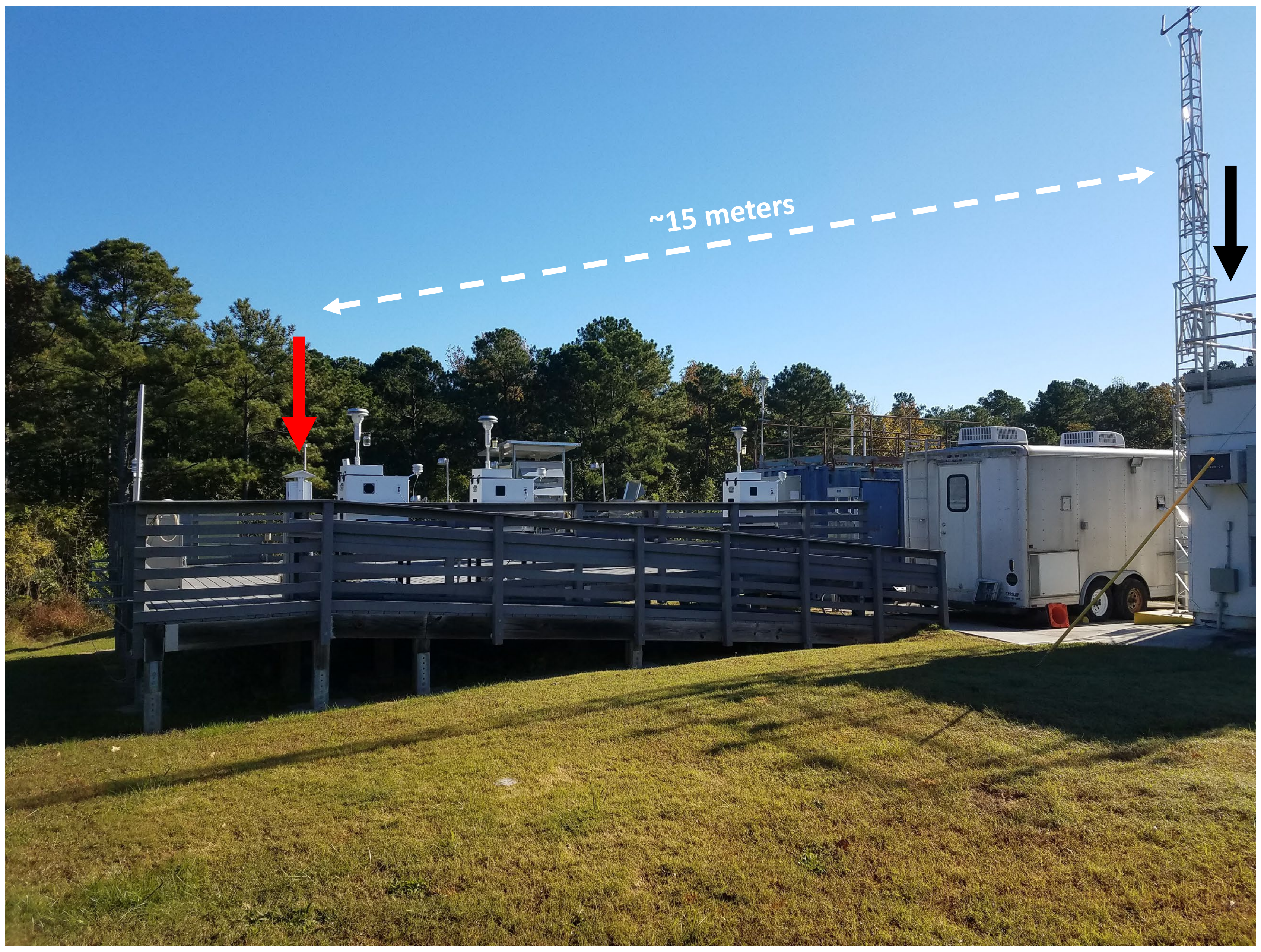

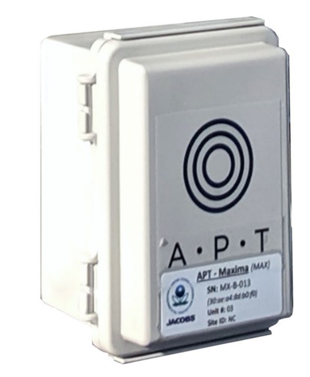

Data Storage and Transmission Method

The APT Maxima records data at 30 second intervals and stores the data on an internal microSD card and transmits data to the APT cloud server (last accessed 5/11/22) via Wi-Fi connection. The user interface for the Maxima sensors was under development during sensor testing, thus weekly raw data files were obtained from the internal MicroSD card. Each field site operator was provided two labeled MicroSD cards for the Maxima that they used to swap out each week. Data from the collected card was then read and processed off-site.

Data Correction Approach

This evaluation report reflects "out-of-the-box" performance of the APT Maxima. The Maxima data sets include two data columns contain $PM_{2.5}$ concentration measurements, "PM2.5(ug/m3)" and "PM2.5_Atm(ug/m3)". These columns correspond to separate correction factors provided by the onboard OEM PM sensor (Plantower PMSA003). For this report, $PM_{2.5}$ measurements for the column labeled "PM2.5(ug/m3)" were selected for analysis.

After acquisition, the raw data was processed using the *sensortoolkit* python code library (v0.8.3b2). A continuous data set at the recorded sampling frequency was written to a .csv file. 1-hour and 24-hour averaged data sets were generated using a 75% completeness threshold and saved as separate .csv files. Outliers were NOT removed from data sets in order to assess "out-of-the-box" sensor performance.

Issues Encountered

Pre-deployment observations

- *Sampling internal abnormality:* Maxima devices did not continuously sample at 30-second intervals as stated in the sensor user guide. Instead, Maxima units occasionally sampled at 31 seconds. This increased sample interval is due to the accumulation of processing time over a certain number of samples. Each 30-second sample is a collection of exactly 30 seconds of sampling followed by a brief fraction of a second used for processing the collected data. Once processed, the next 30-second data collection period begins. When the sum of the processing time reaches one full second, the sample interval then jumps to 31 seconds. Data from this pre-deployment testing indicated that the processing speed for each sensor varies such that 31-second sampling duration occur at random intervals across Maxima units.

Field observations and sensor data flags

APT Maxima units were deployed at the AIRS monitoring site on 5/13/2019. The Maxima units operated nominally during the testing period and did not require replacement or repair.

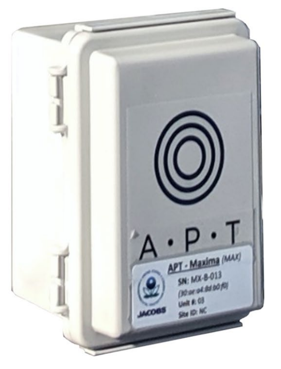

Description of Data Flags

OAQPS manages data logged by reference monitors at the AIRS evaluation site using the Envidas data acquisition system software from DR DAS LTD[1]. Envidas contains over 100 data flags which are configurable by the monitoring agency and can be triggered based on instrument status. Appendix B Table 1 contains a description of Envidas data flags including the numeric code and status code name (a brief textual description, the table containing data flags below includes code names for events encountered by the reference monitor under the “Flag” column). Appendix B Table 1 also indicates whether reference data are invalidated for a given data flag. For this report, reference data were invalidated for periods where a data flag was logged and if the corresponding entry for the data flag in the “Data Status Invalidation” column in Appendix B Table 1 indicates data should be invalidated.

Data Flags Recorded During Testing

FRM/FEM Monitor	Timestamp (UTC)	Flag
Teledyne API T640X (Data acquired via local transfer from OAQPS)	2019-05-14 17:32:00+0000	<Samp
	2019-05-14 17:40:00+0000	<Samp
	2019-05-14 17:54:00+0000	<Samp
	2019-05-14 21:47:00+0000	<Samp
	2019-05-14 21:58:00+0000	NoData
	2019-05-14 22:05:00+0000	<Samp
	2019-05-17 19:40:00+0000	<Samp
	2019-05-22 18:54:00+0000	NoData
	2019-05-22 18:55:00+0000	<Samp
	2019-05-24 13:28:00+0000 to 2019-05-24 14:12:00+0000	Down
	2019-05-24 15:30:00+0000	<Samp
	2019-05-24 17:17:00+0000	<Samp
	2019-05-24 17:24:00+0000	<Samp
	2019-06-03 14:23:00+0000	<Samp
	2019-06-03 14:24:00+0000	NoData
	2019-06-04 11:20:00+0000 to 2019-06-04 11:24:00+0000	NoData
	2019-06-04 12:13:00+0000	<Samp
	2019-06-04 17:02:00+0000 to 2019-06-05 04:59:00+0000	NoData
	2019-06-04 17:19:00+0000	<Samp
	2019-06-08 15:15:00+0000 to 2019-06-13 23:00:00+0000	NoData

Data flags continued on the followingNC-MAX-Page

[1]“Contact us” DR DAS LTD, 2022, https://dr-das.com/Home/Contact.html Accessed June 3 2022

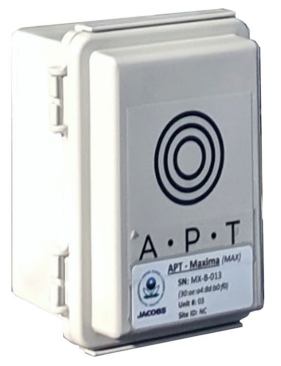

Supplemental Information: Description of FRM/FEM QC Checks and Data Flags

Flags Recorded During Testing (Cont.)

Meteorological Instrument	Timestamp (UTC)	Flag
RM Young 41382 VC Temperature and Relative Humidity Monitor (Data acquired via local transfer from OAQPS)	2019-05-14 17:32:00+0000	<Samp
	2019-05-14 17:40:00+0000	<Samp
	2019-05-14 17:54:00+0000	<Samp
	2019-05-14 21:47:00+0000	<Samp
	2019-05-14 21:58:00+0000	NoData
	2019-05-14 22:05:00+0000	<Samp
	2019-05-17 19:40:00+0000	<Samp
	2019-05-22 18:54:00+0000	NoData
	2019-05-22 18:55:00+0000	<Samp
	2019-05-24 15:30:00+0000	<Samp
	2019-05-24 17:17:00+0000	<Samp
	2019-05-24 17:24:00+0000	<Samp
	2019-06-04 11:20:00+0000 to 2019-06-04 11:24:00+0000	NoData
	2019-06-04 12:13:00+0000	<Samp
	2019-06-04 17:03:00+0000 to 2019-06-05 04:59:00+0000	NoData
	2019-06-04 17:19:00+0000	<Samp
	2019-06-08 19:56:00+0000 to 2019-06-13 23:00:00+0000	NoData

Testing Report - $PM_{2.5}$ Base Testing

Clarity Node-S

This report reflects out-of-the-box performance

Initial Base Testing - RTP, NC
U.S. Environmental Protection Agency
Office of Research and Development
PI: Clements.Andrea@epa.gov
919-541-1363
August 2019—September 2019

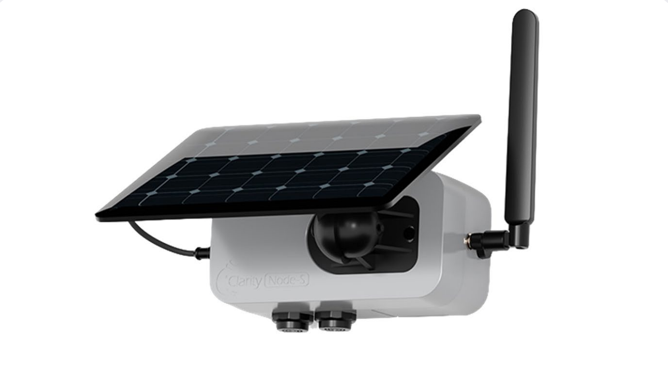
Image Attribution: Clarity Movement Co. (Berkeley, CA)

Deployment Details

Testing Organization and Site Information	
Testing organization (Name, Organization type, Contact website)	U.S. Environmental Protection Agency - Office of Research and Development<br>Federal Government<br>Air Sensor Toolbox | U.S. EPA Website
Testing location (City, State, Latitude and Longitude)	Ambient Monitoring Innovative Research Station (AIRS)<br>RTP, NC<br>35.88951, -78.874572
AQS site ID	37 – 063 – 0099
Sampling timeframe (MM-DD-YY)	08-02-19 to 09-01-19
Sensor data source	Clarity Dashboard download
Reference data source	OAQPS file transfer

Sensor Information			
Manufacturer, model	Clarity Node-S		
Device firmware version	Received by EPA May 2019		
Sampling time interval	17-minutes		
Sensor serial numbers	CNO_01	CNO_02	CNO_03
Issues encountered during deployment?	☐	No Issues	

FRM/FEM Information	
Manufacturer, model, designation	Teledyne Advanced Pollution Instrumentation T640 FEM
Sampling time interval	1-hour averaging
Date of calibration	As required by 40 CFR Part 58 and the Burdens Creek QAPP maintained by OAQPS
Date of flowrate verification check	Monthly as required by 40 CFR Part 58 Appendix A
Description, date(s) of maintenance activities	N/A

Time Series Plots

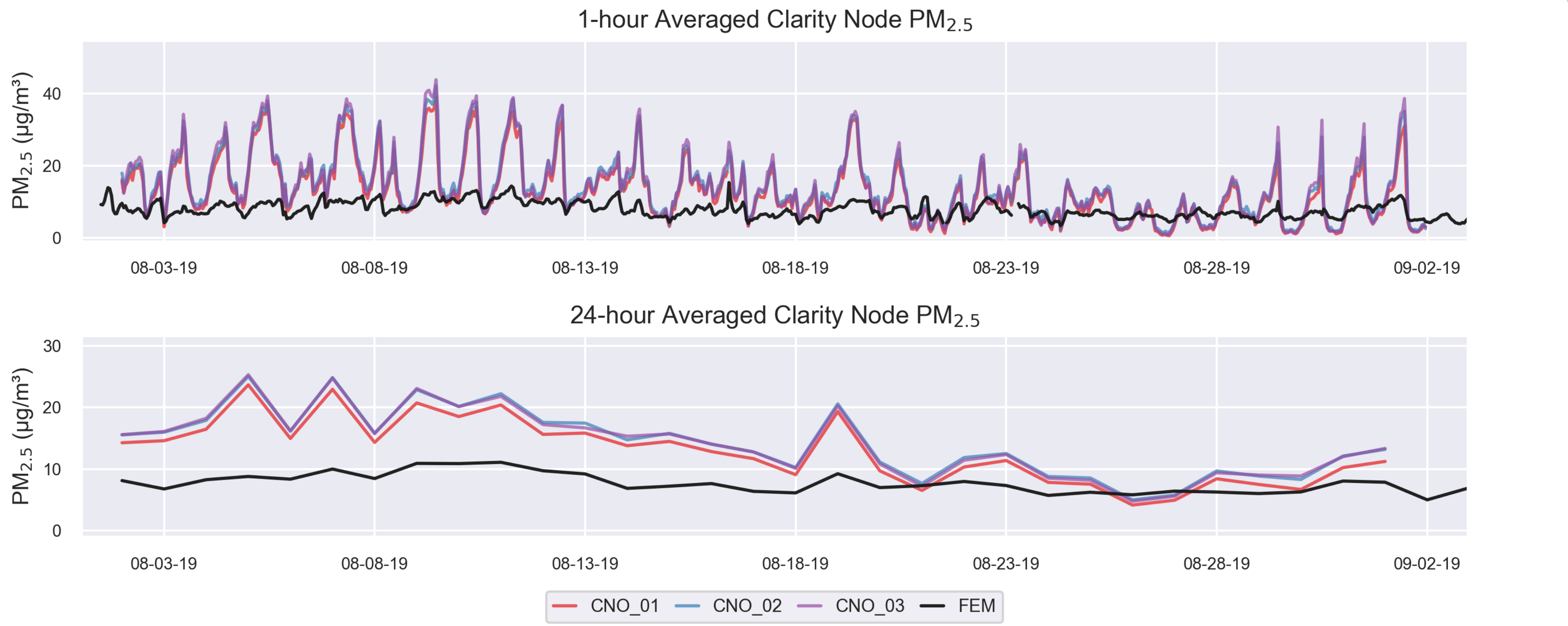

Scatter Plots: Comparison to FRM/FEM

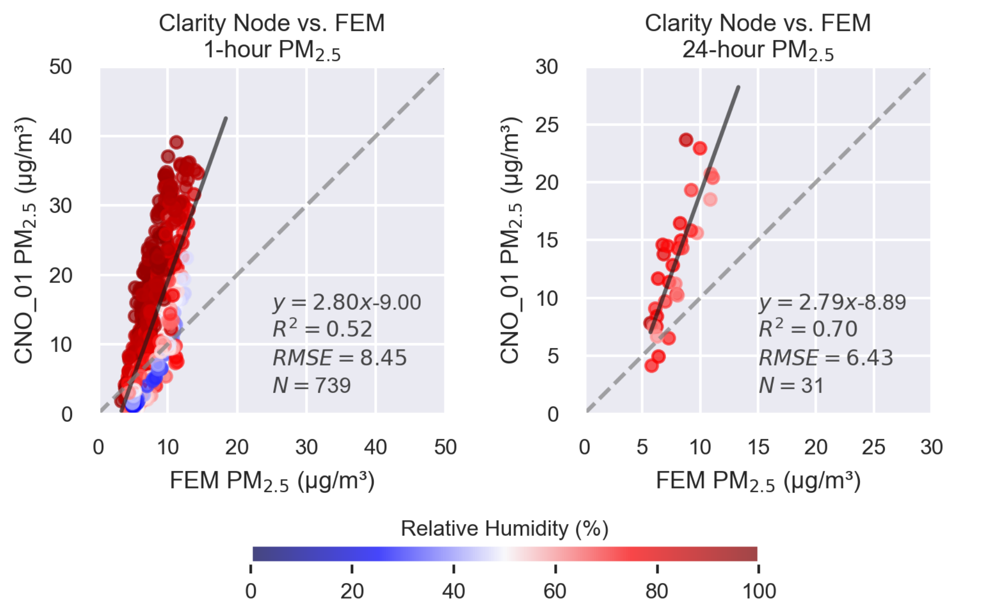

Range of FRM/FEM concentrations over duration of base test (µg/m³)	3.4-15.3 (1-hr), 5.7-11.1 (24-hr)
Number of 24-hr periods in FRM/FEM monitor measurements with a goal concentration ≥ 25 µg/m³	0

Performance Metrics★

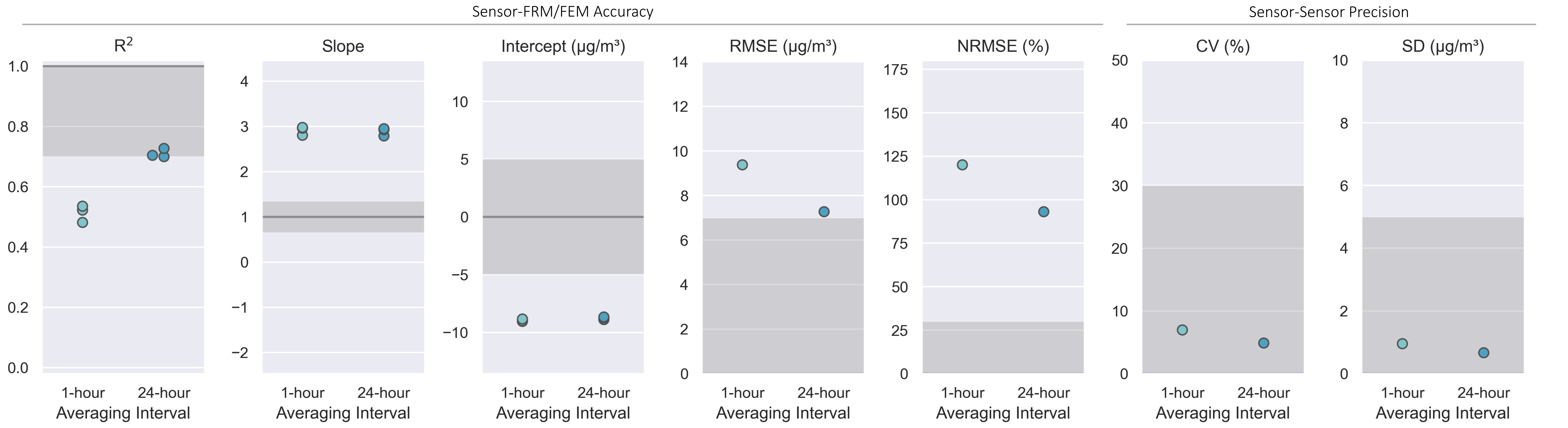

Meteorological Conditions During Deployment

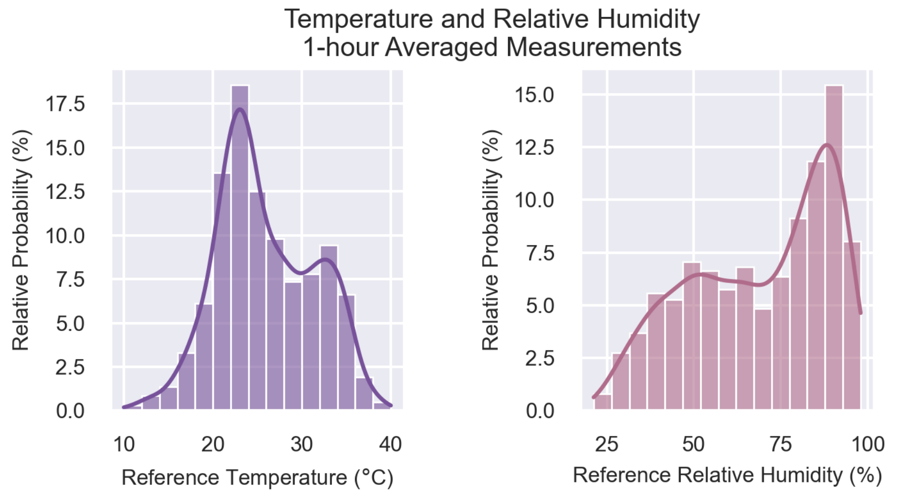

Number of 24-hr periods outside sensor manufacture-listed temperature operational range (-10 to 55 °C)	0
Number of 24-hr periods outside sensor manufacture-listed relative humidity operational range (10 to 98%)	0

Meteorological Influence

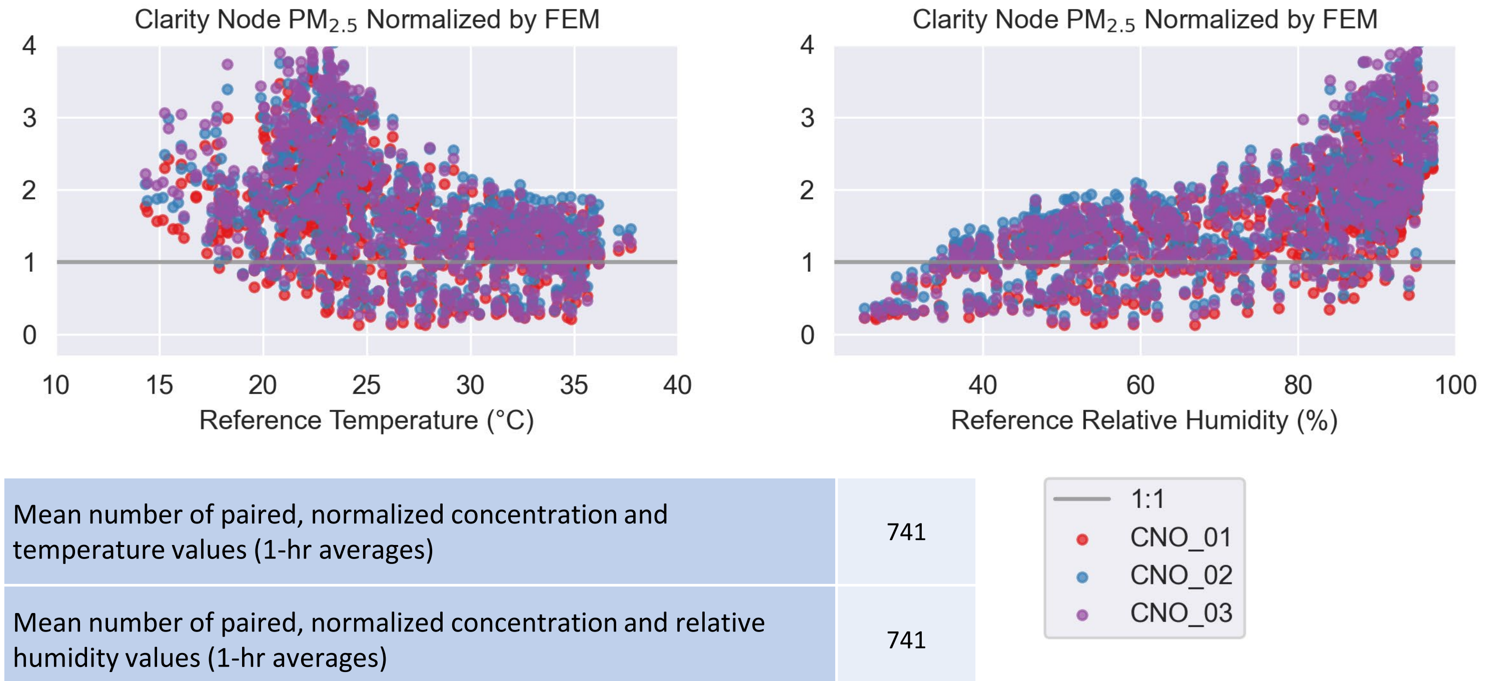

Mean number of paired, normalized concentration and temperature values (1-hr averages)	741
Mean number of paired, normalized concentration and relative humidity values (1-hr averages)	741

★For evaluations with greater than three sensors, grouping individual sensor metrics into boxplots is recommended for displaying results. Note that this recommendation does not apply to metrics computed as a single value for all sensors over the whole evaluation group, such as RMSE, NRMSE, CV, and standard deviation.

Testing Report - $PM_{2.5}$ Base Testing

Clarity Node-S

This report reflects out-of-the-box performance

Initial Base Testing - RTP, NC
U.S. Environmental Protection Agency
Office of Research and Development
PI: Clements.Andrea@epa.gov
919-541-1363
August 2019—September 2019

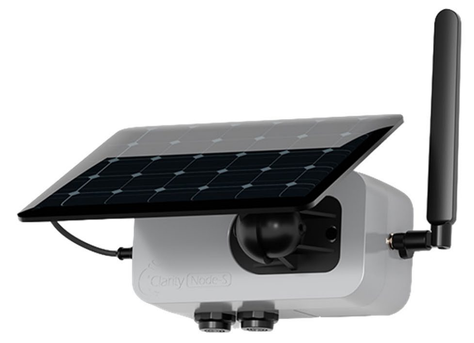

Image Attribution: Clarity Movement Co. (Berkeley, CA)

Tabular Statistics

Sensor-FRM/FEM Correlation

	Bias and Linearity						Data Quality			
	R^2		Slope		Intercept (µg/m³)		Uptime (%)		Number of paired sensor and FRM/FEM concentration values	
	1-Hour ○○○	24-Hour ●●○	1-Hour ○○○	24-Hour ○○○	1-Hour ○○○	24-Hour ○○○	1-Hour ●●●	24-Hour ●●●	1-Hour	24-Hour
Metric Target Range	≥ 0.70	≥ 0.70	1.0 ± 0.35	1.0 ± 0.35	-5 ≤ b ≤ 5	-5 ≤ b ≤ 5	75%*	75%*	-	-
Sensor CNO_01	0.52	0.70	2.80	2.79	-9.00	-8.89	100	100	739	31
Sensor CNO_02	0.54	0.73	2.96	2.95	-8.84	-8.74	100	100	738	31
Sensor CNO_03	0.48	0.70	2.98	2.93	-9.05	-8.67	100	100	737	31
Mean	0.51	0.71	2.91	2.89	-8.96	-8.77	100	100	738	31

	Error			
	RMSE (µg/m³)		NRMSE (%)	
	1-Hour ☆	24-Hour ☆	1-Hour ☆	24-Hour ☆
Metric Target Range	≤ 7.0	≤ 7.0	≤ 30.0	≤ 30.0
Deployment Value	9.4	7.3	120.2	93.2

Device-specific metrics (computed for each sensor in evaluation)
○○○ Metric value for none of devices tested falls within the target range
●○○ Metric value for one of devices tested falls within the target range
●●○ Metric value for two of devices tested falls within the target range
●●● Metric value for three of devices tested falls within the target range

Single-valued metrics (computed via entire evaluation dataset)
☆ Indicates that the metric value is not within the target range
★ Indicates that the metric value is within the target range

Sensor-Sensor Precision

	Precision (between collocated sensors)				Data Quality	
	CV (%)		SD (µg/m³)		Number of concurrent sensor concentration pairs	
	1-Hour ★	24-Hour ★	1-Hour ★	24-Hour ★	1-Hour	24-Hour
Metric Target Range	≤ 30.0	≤ 30.0	≤ 5.0	≤ 5.0	-	-
Deployment Value	7.0	4.9	1.0	0.7	740	31

*This value is only a recommendation for ensuring data quality and is not included in the list of target values discussed in Section 4 of the Performance Testing Protocols, Metrics, and Target Values for Fine Particulate Matter Air Sensors document.

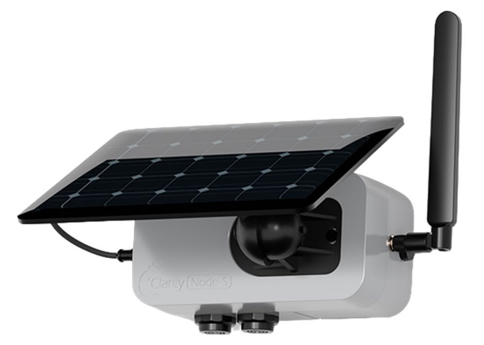

Image Attribution: Clarity Movement Co. (Berkeley, CA)

Sensor-FRM/FEM Scatter Plots

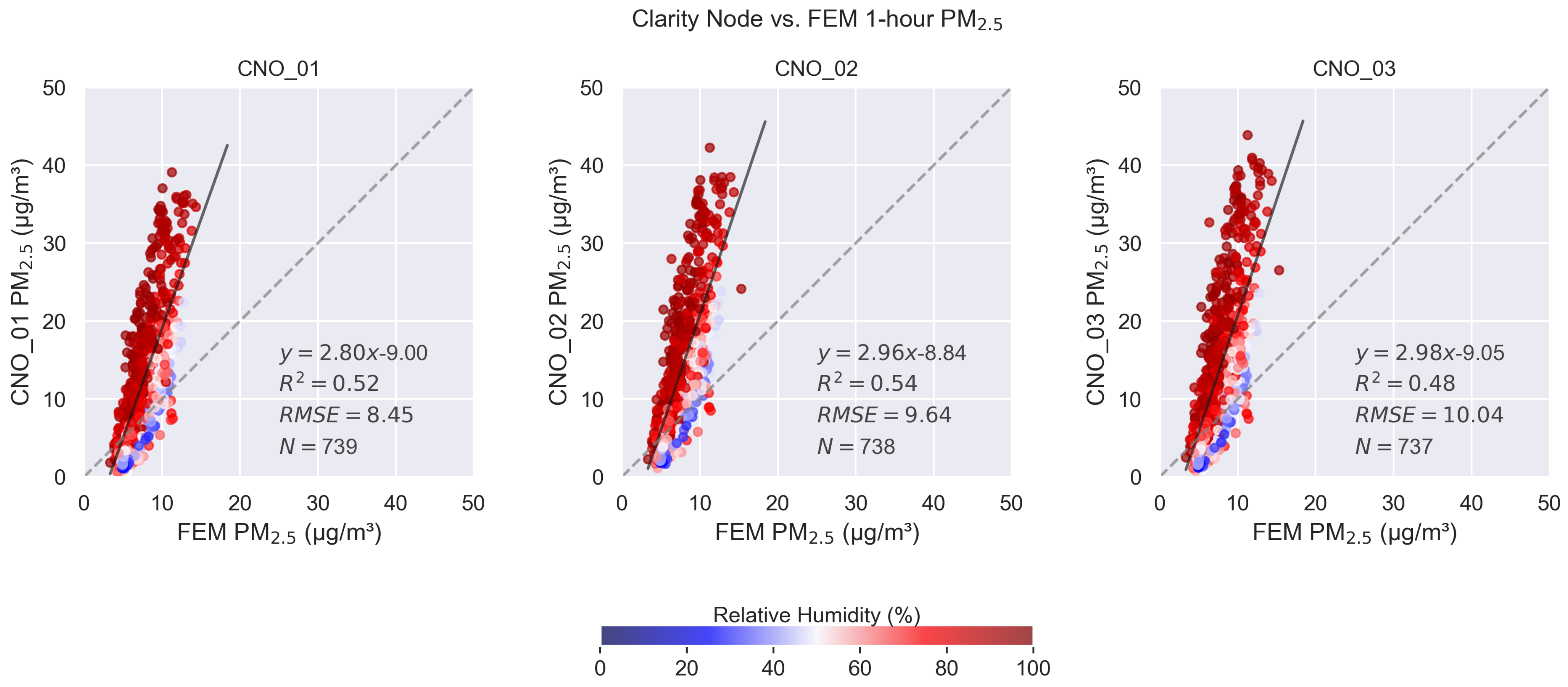

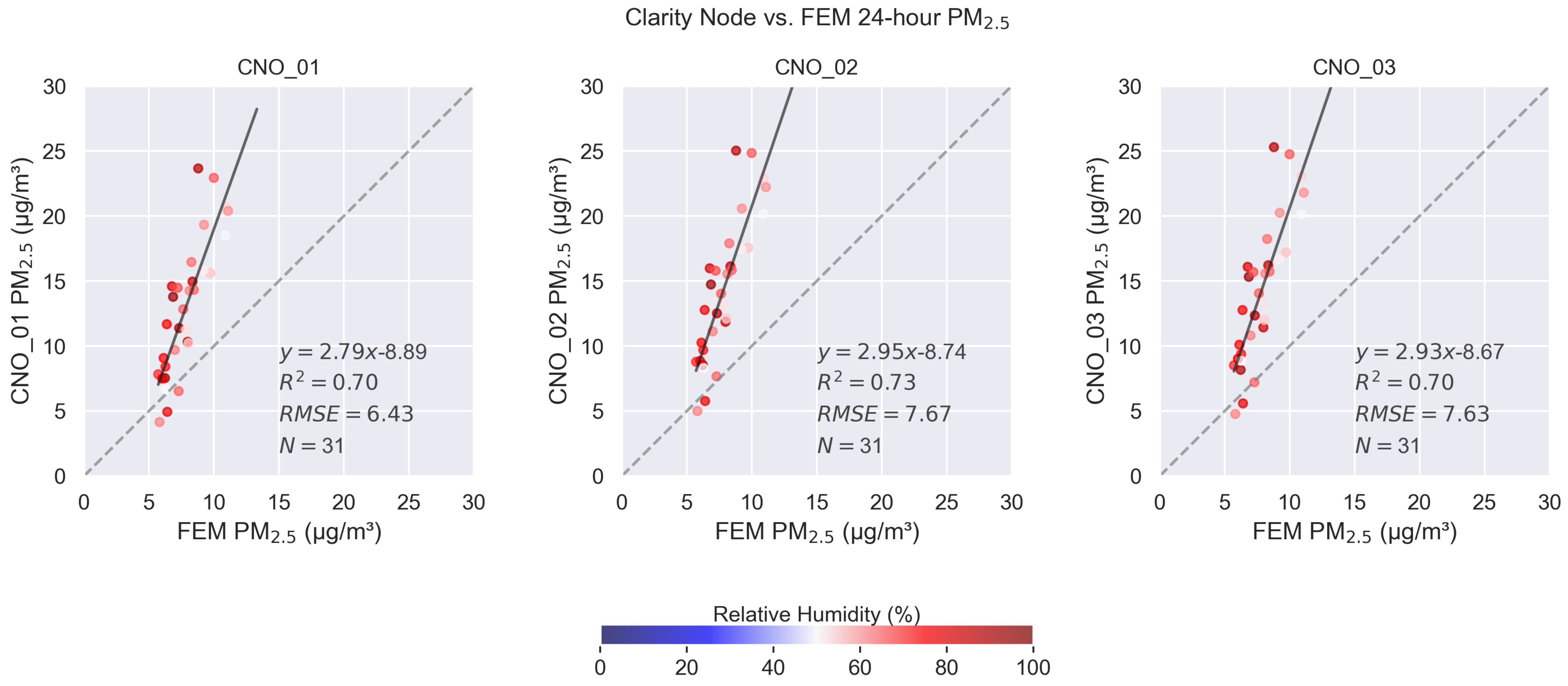

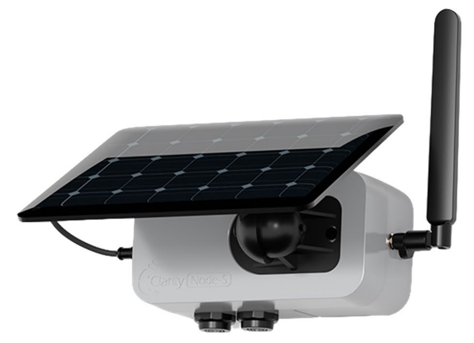

Image Attribution: Clarity Movement Co. (Berkeley, CA)

Supplemental Information

Abbreviations used in Supplemental Information

FRM	Federal Reference Method
FEM	Federal Equivalent Method
SOP	Standard Operating Procedure
QAPP	Quality Assurance Project Plan
QC	Quality Control

Supplemental Documentation	Attached	Description & URL or file path to documentation
Field observations and sensor data flags	☑	See NC-CNO-Page 6 of this testing report
Maintenance logs	☐	No logs recorded during testing
Standard operating procedure(s)	☐	U.S. EPA Office Of Research and Development SOP available upon request
Photos of equipment setup and testing	☑	See NC-CNO-Page 5 of this testing report
Product specifications sheet(s)	☐	N/A
Product manual(s)	☐	N/A
Data storage and transmission method	☑	See NC-CNO-Page 6 of this testing report
Data correction approach	☑	See NC-CNO-Page 6 of this testing report
Issues encountered	☑	See NC-CNO-Page 6 of this testing report
Data analysis/correction scripts and version	☑	Averaging and processing of data, calculation of performance metrics, and generation of figures and other supplementary material for analysis were obtained using Python 3.9.7 with the packages sensortoolkit v0.8.3b2, pandas 1.3.5, NumPy 1.21.2, Matplotlib 3.5.0, statsmodels 0.13.0, and seaborn 0.11.2. All packages are available from the Python Package Index (PyPI) at https://pypi.org. The integrated development environment (IDE) Spyder 5.1.5 was used for scripting and data visualization. Version control for the Python base, packages, and IDE were all managed by conda 4.11.0.
Air Monitoring Station QAPP	☐	U.S. EPA Office Of Research and Development QAPP available upon request
Summary of FRM/FEM monitor QC checks	☑	See NC-CNO-Page 7 of this testing report
Manufacturer website for FRM/FEM monitor	☑	Teledyne API: T640/T640X Product website
FRM/FEM monitor manual	☑	Teledyne API: T640/T640X Product Manual
FRM/FEM monitor specifications sheet(s)	☑	Teledyne API: T640/T640X Specification Sheet
Other documents	☐	

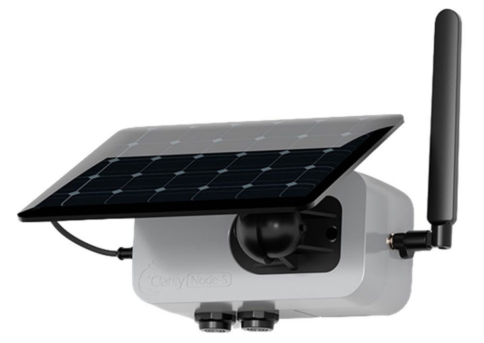
Image Attribution: Clarity Movement Co. (Berkeley, CA)

Supplemental Information: Photos of Testing Site and Equipment Setup

Site Description:
The Burdens Creek Ambient Monitoring Innovation Research Station (AIRS) site is located on the U.S. EPA, RTP campus and is situated between Alexander Drive and Route 147. The site is intended to represent a neighborhood-scale site as defined in *40 CFR Part 58, Appendix D*. U.S. EPA's Office of Air Quality Planning and Standards (OAQPS) operates reference grade instruments in a free-standing shelter situated directly adjacent to the sensor testing platform.

Figure 1: Clarity Node-S sensors (indicated by red arrow) attached to mounting assembly at evaluation site.

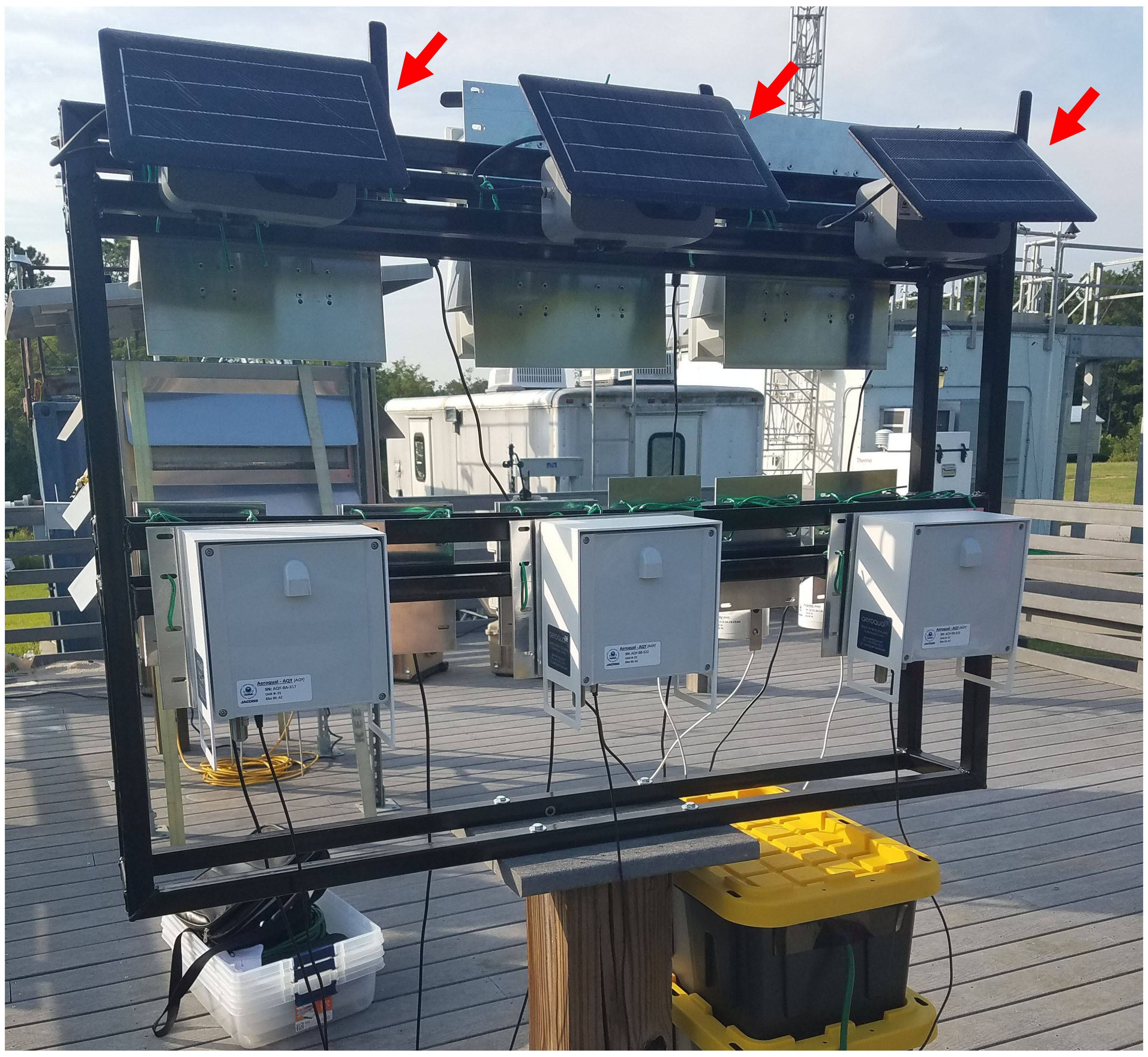

Figure 2: Observation deck at evaluation site. Approximate location of sensors indicated by the red arrow, the approximate location of T640x FEM (housed within sampling trailer) indicated by the black arrow. Sensors and FEM instrumentation are separated by approximately 15 meters.

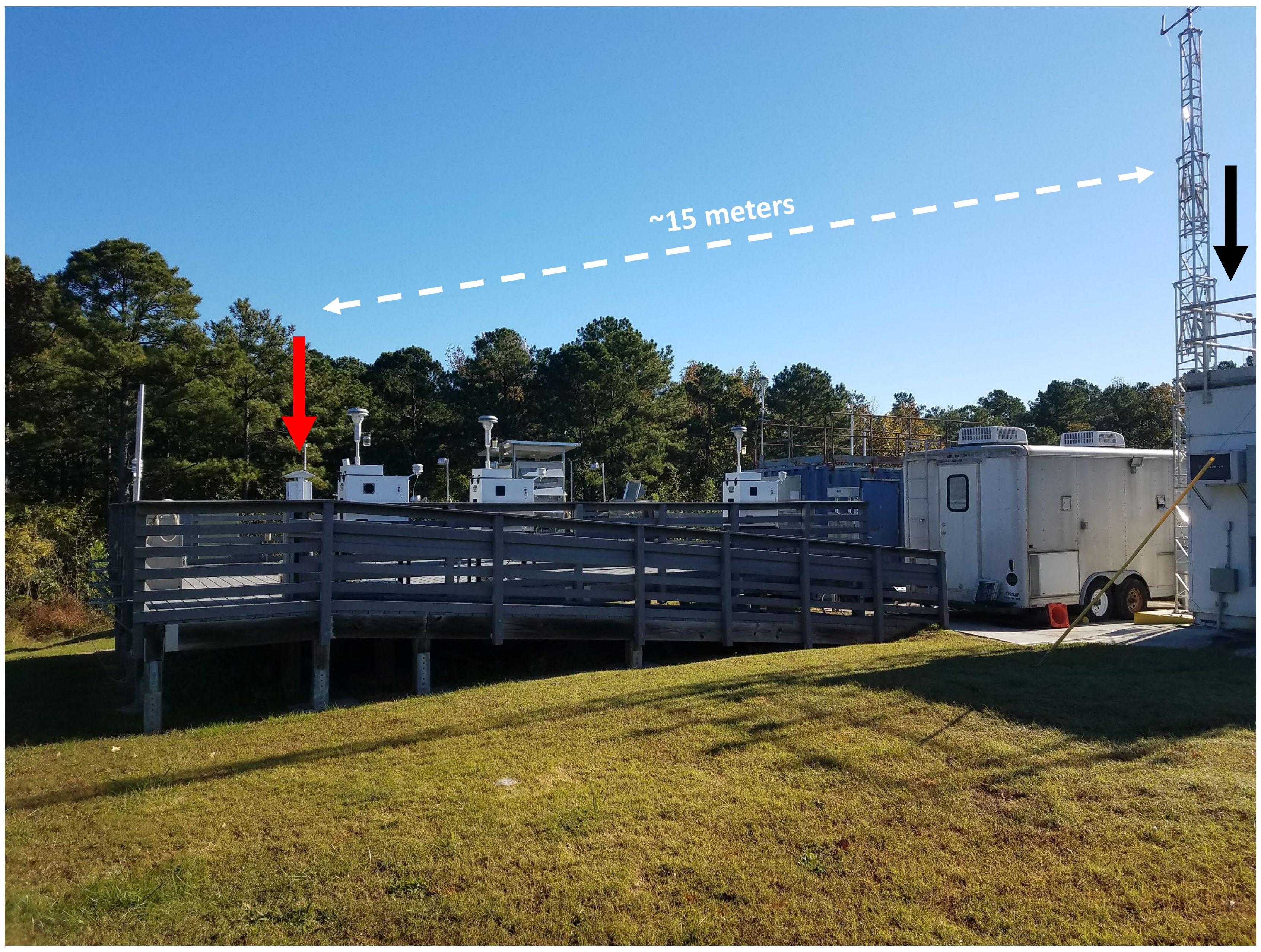

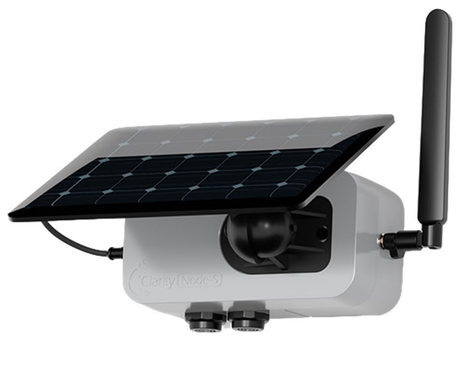

Image Attribution: Clarity Movement Co. (Berkeley, CA)

Data Storage and Transmission Method

The Clarity Node transmits data via cellular SIM card. Weekly raw data collections were performed over Wi-Fi using the Clarity dashboard (*last accessed 5/11/22*). The observed sampling interval fluctuated and is further discussed under pre-deployment observations below. On average, the Node models sampled every 2-3 minutes, and the Node-S sampled every 15-17 minutes.

Data Correction Approach

This evaluation report reflects "out-of-the-box" performance of the Clarity Node. The manufacturer provides a dashboard feature for calibrating sensor data against collocated reference monitors. Calibrated data were NOT used for this evaluation. Prospective consumers may get better performance from this device if they utilize this feature.

After acquisition, the raw data was processed using the *sensortoolkit* python code library (v0.8.3b2). A continuous data set at the recorded sampling frequency was written to a .csv file. 1-hour and 24-hour averaged data sets were generated using a 75% completeness threshold and saved as separate .csv files. Due to the variable sampling interval observed for Clarity Node units, 75% completeness was determined against the number of data points that would be recorded within 1-hour/24-hour periods if the sensor were to log measurements at the most commonly recorded sampling interval (i.e., the mode of recorded intervals). Outliers were NOT removed from data sets in order to assess "out-of-the-box" sensor performance.

Issues Encountered

Pre-deployment observations

- *Unsteady sampling interval:* Clarity Node sensors were received by EPA on May 28, 2019. A lab bench-top evaluation was performed from May 29, 2019 – May 31, 2019. During bench-top testing, it was observed that the sampling intervals noted by Clarity (5 minutes for non-solar Node, 15 minutes for the solar Node-S) were not reflective of recorded intervals. Communication with the vendor concluded that the sampling interval will fluctuate depending on the settings, battery charge, and strength of the cellular signal and that this sampling interval cannot be adjusted by the user at present. On average, the Node models sampled every 2-3 minutes, and the Node-S sampled every 15-17 minutes.

Field observations and sensor data flags

Clarity Node-S units were deployed at the AIRS monitoring site on 8/1/2019. The Node-S units operated nominally during the testing period and did not require replacement or repair.

Description of Data Flags

OAQPS manages data logged by reference monitors at the AIRS evaluation site using the Envidas data acquisition system software from DR DAS LTD[1]. Envidas contains over 100 data flags which are configurable by the monitoring agency and can be triggered based on instrument status. Appendix B Table 1 contains a description of Envidas data flags including the numeric code and status code name (a brief textual description, the table containing data flags below includes code names for events encountered by the reference monitor under the "Flag" column). Appendix B Table 1 also indicates whether reference data are invalidated for a given data flag. For this report, reference data were invalidated for periods where a data flag was logged and if the corresponding entry for the data flag in the "Data Status Invalidation" column in Appendix B Table 1 indicates data should be invalidated.

Data Flags Recorded During Testing

FRM/FEM Monitor	Timestamp (UTC)	Flag
Teledyne API T640X (Data acquired via local transfer from OAQPS)	2019-08-08 14:02:00+0000	<Samp
	2019-08-13 14:58:00+0000	<Samp
	2019-08-21 12:56:00+0000 to 2019-08-21 13:29:00+0000	Down
	2019-08-21 13:30:00+0000	<Samp
	2019-08-21 21:29:00+0000 to 2019-08-21 21:34:00+0000	<Samp
	2019-08-21 21:30:00+0000 to 2019-08-21 21:33:00+0000	Down
	2019-08-23 04:28:00+0000 to 2019-08-23 07:12:00+0000	NoData
	2019-08-23 07:13:00+0000	<Samp
	2019-08-26 21:18:00+0000	<Samp
	2019-08-28 15:07:00+0000	<Samp

Meteorological Instrument	Timestamp (UTC)	Flag
[RM Young 41382 VC Temperature and Relative Humidity Monitor (Data acquired via local transfer from OAQPS)	2019-08-08 14:02:00+0000	<Samp
	2019-08-13 14:58:00+0000	<Samp
	2019-08-23 04:28:00+0000 to 2019-08-23 07:12:00+0000	NoData
	2019-08-23 07:13:00+0000	<Samp
	2019-08-26 21:18:00+0000	<Samp
	2019-08-28 15:07:00+0000	<Samp

[1]"Contact us" DR DAS LTD, 2022, https://dr-das.com/Home/Contact.html Accessed June 3 2022

Testing Report - $PM_{2.5}$ Base Testing

PurpleAir PA-II-SD

This report reflects out-of-the-box performance

Initial Base Testing - RTP, NC
U.S. Environmental Protection Agency
Office of Research and Development
PI: Clements.Andrea@epa.gov
919-541-1363
August 2019—September 2019

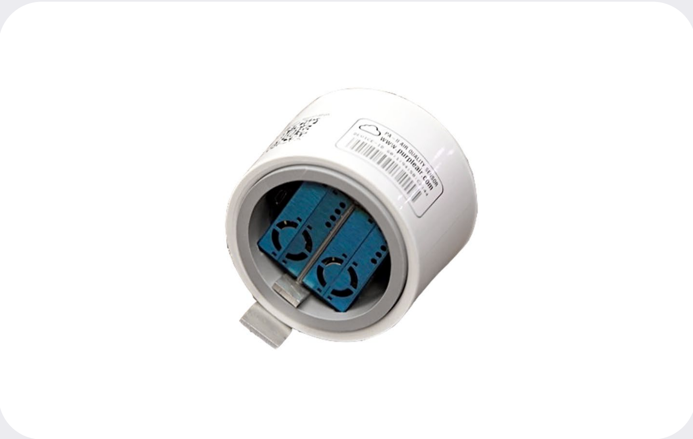

Deployment Details

Testing Organization and Site Information	
Testing organization (Name, Organization type, Contact website)	U.S. Environmental Protection Agency - Office of Research and Development<br>Federal Government<br>Air Sensor Toolbox | U.S. EPA Website
Testing location (City, State, Latitude and Longitude)	Ambient Monitoring Innovative Research Station (AIRS)<br>RTP, NC<br>35.88951, -78.874572
AQS site ID	37 – 063 – 0099
Sampling timeframe (MM-DD-YY)	08-02-19 to 09-01-19
Sensor data source	Onboard MicroSD card
Reference data source	OAQPS file transfer

Sensor Information			
Manufacturer, model	PurpleAir PA-II-SD		
Device firmware version	V4.02		
Sampling time interval	2-minutes		
Sensor serial numbers	PA01	PA02	PA03
Issues encountered during deployment?	☐	No Issues	

FRM/FEM Information	
Manufacturer, model, designation	Teledyne Advanced Pollution Instrumentation T640 FEM
Sampling time interval	1-hour averaging
Date of calibration	As required by 40 CFR Part 58 and the Burdens Creek QAPP maintained by OAQPS
Date of flowrate verification check	Monthly as required by 40 CFR Part 58 Appendix A
Description, date(s) of maintenance activities	N/A

Time Series Plots

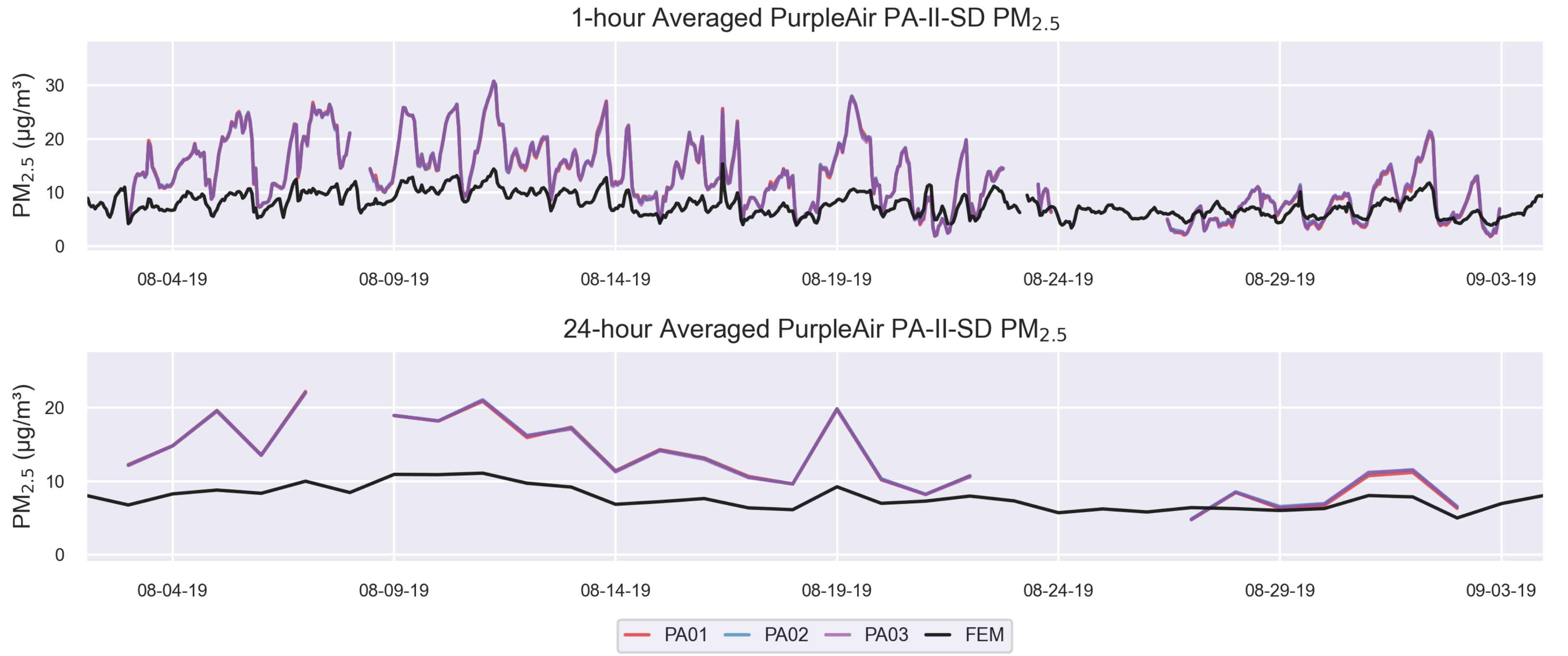

Scatter Plots: Comparison to FRM/FEM

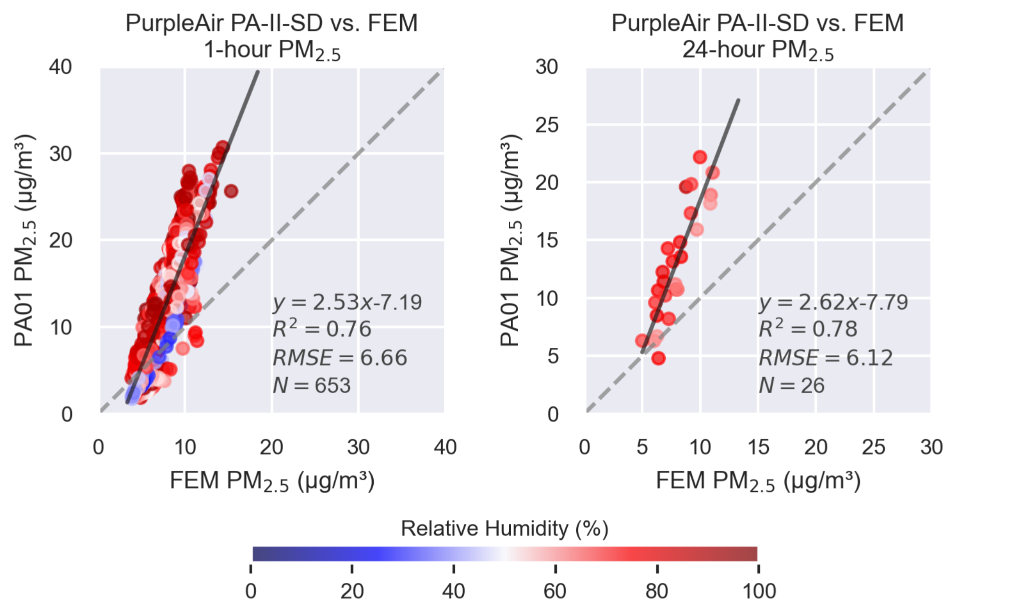

Range of FRM/FEM concentrations over duration of base test (μg/m³)	3.4-15.3 (1-hr), 5.0-11.1 (24-hr)
Number of 24-hr periods in FRM/FEM monitor measurements with a goal concentration ≥ 25 μg/m³	0

Performance Metrics★

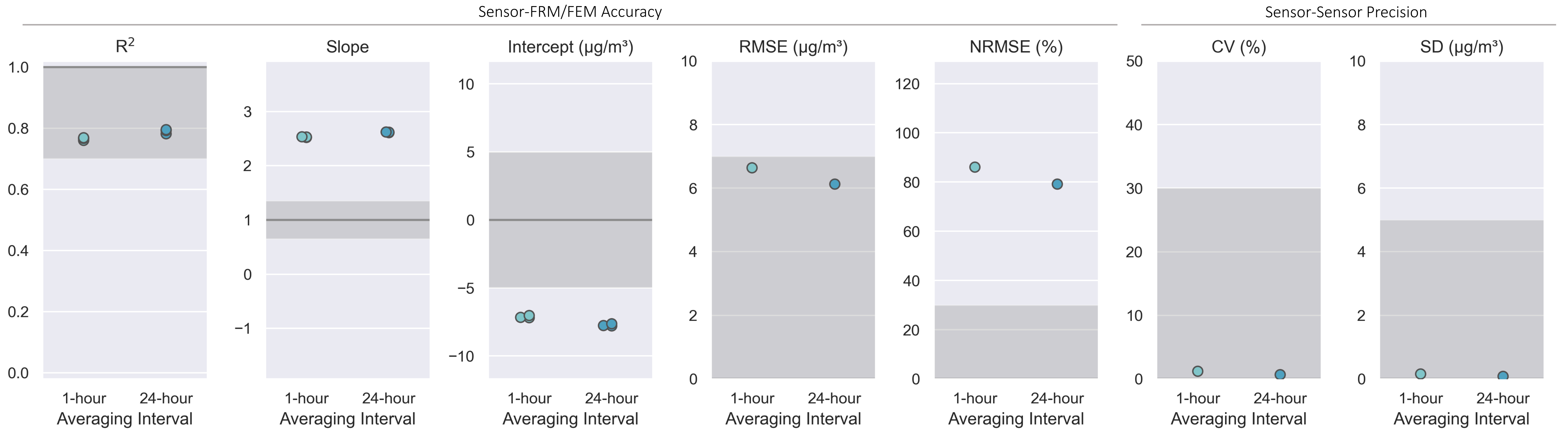

Meteorological Conditions During Deployment

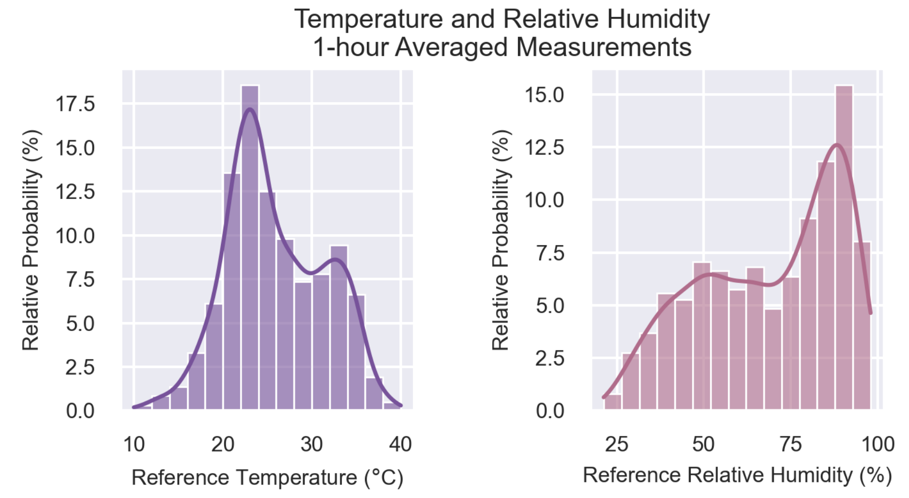

Number of 24-hr periods outside sensor manufacturer-listed temperature operational range (no operational range specified)	-
Number of 24-hr periods outside sensor manufacturer-listed relative humidity operational range (no operational range specified)	-

Meteorological Influence

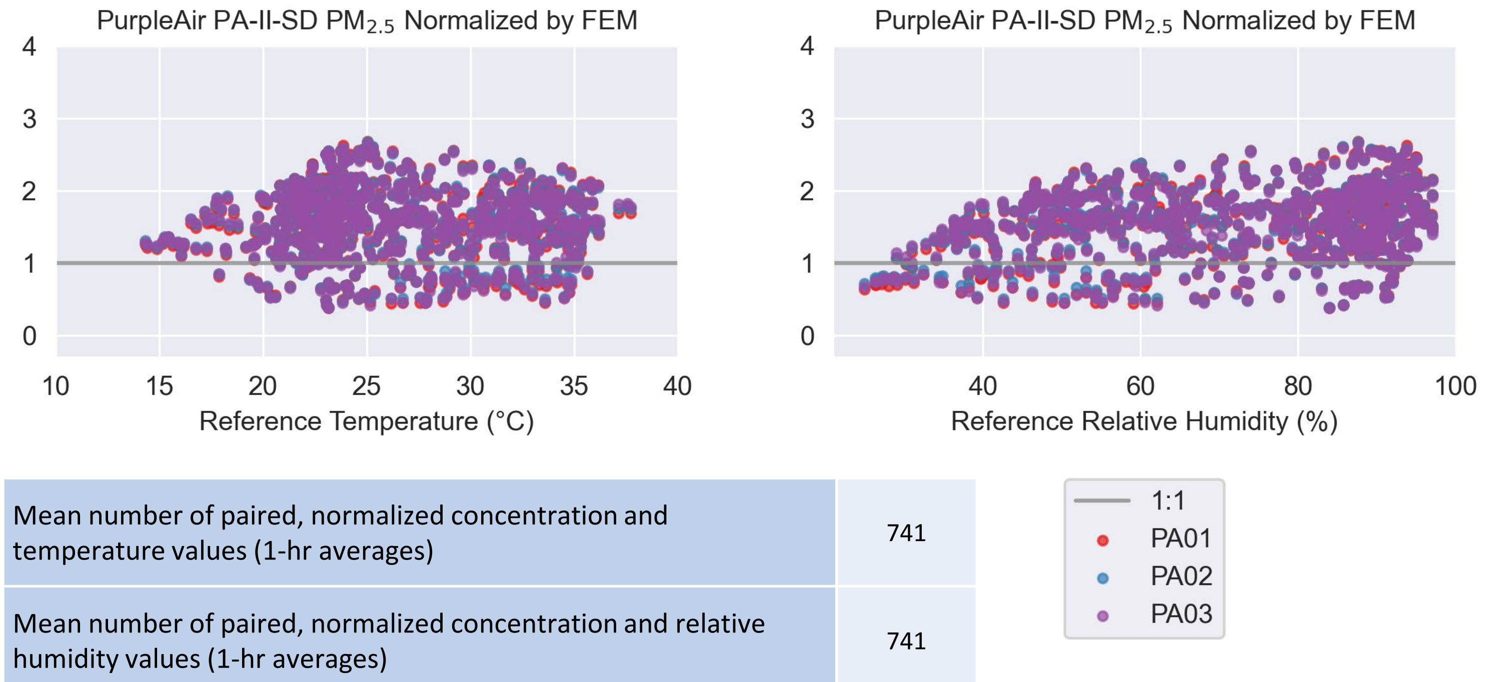

Mean number of paired, normalized concentration and temperature values (1-hr averages)	741
Mean number of paired, normalized concentration and relative humidity values (1-hr averages)	741

★For evaluations with greater than three sensors, grouping individual sensor metrics into boxplots is recommended for displaying results. Note that this recommendation does not apply to metrics computed as a single value for all sensors over the whole evaluation group, such as RMSE, NRMSE, CV, and standard deviation.

Testing Report - $PM_{2.5}$ Base Testing

PurpleAir PA-II-SD

This report reflects out-of-the-box performance

Initial Base Testing - RTP, NC
U.S. Environmental Protection Agency
Office of Research and Development
PI: Clements.Andrea@epa.gov
919-541-1363
August 2019—September 2019

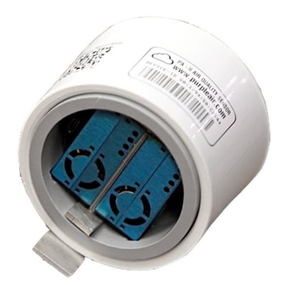

Tabular Statistics

Sensor-FRM/FEM Correlation

	Bias and Linearity						Data Quality			
	R^2		Slope		Intercept (µg/m³)		Uptime (%)		Number of paired sensor and FRM/FEM concentration values	
	1-Hour ●●●	24-Hour ●●●	1-Hour ○○○	24-Hour ○○○	1-Hour ○○○	24-Hour ○○○	1-Hour ●●●	24-Hour ●●●	1-Hour	24-Hour
Metric Target Range	≥ 0.70	≥ 0.70	1.0 ± 0.35	1.0 ± 0.35	-5 ≤ b ≤ 5	-5 ≤ b ≤ 5	75%*	75%*	-	-
Sensor PA01	0.76	0.78	2.53	2.62	-7.19	-7.79	88	84	653	26
Sensor PA02	0.77	0.79	2.52	2.61	-7.03	-7.64	88	84	653	26
Sensor PA03	0.77	0.80	2.53	2.62	-7.17	-7.77	88	84	653	26
Mean	0.77	0.79	2.53	2.62	-7.13	-7.73	88	84	653	26

	Error			
	RMSE (µg/m³)		NRMSE (%)	
	1-Hour ★	24-Hour ★	1-Hour ☆	24-Hour ☆
Metric Target Range	≤ 7.0	≤ 7.0	≤ 30.0	≤ 30.0
Deployment Value	6.6	6.1	86.1	79.2

Device-specific metrics (computed for each sensor in evaluation)
○○○ Metric value for none of devices tested falls within the target range
●○○ Metric value for one of devices tested falls within the target range
●●○ Metric value for two of devices tested falls within the target range
●●● Metric value for three of devices tested falls within the target range

Single-valued metrics (computed via entire evaluation dataset)
☆ Indicates that the metric value is not within the target range
★ Indicates that the metric value is within the target range

Sensor-Sensor Precision

	Precision (between collocated sensors)				Data Quality	
	CV (%)		SD (µg/m³)		Number of concurrent sensor concentration pairs	
	1-Hour ★	24-Hour ★	1-Hour ★	24-Hour ★	1-Hour	24-Hour
Metric Target Range	≤ 30.0	≤ 30.0	≤ 5.0	≤ 5.0	-	-
Deployment Value	1.2	0.7	0.2	0.1	654	26

*This value is only a recommendation for ensuring data quality and is not included in the list of target values discussed in Section 4 of the Performance Testing Protocols, Metrics, and Target Values for Fine Particulate Matter Air Sensors document.

U.S. Environmental Protection Agency
Office of Research and Development
PI: Clements.Andrea@epa.gov
919-541-1363

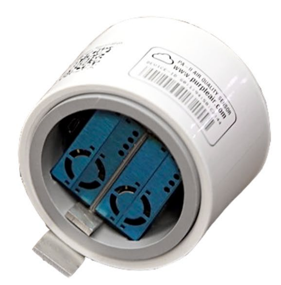

Sensor-FRM/FEM Scatter Plots

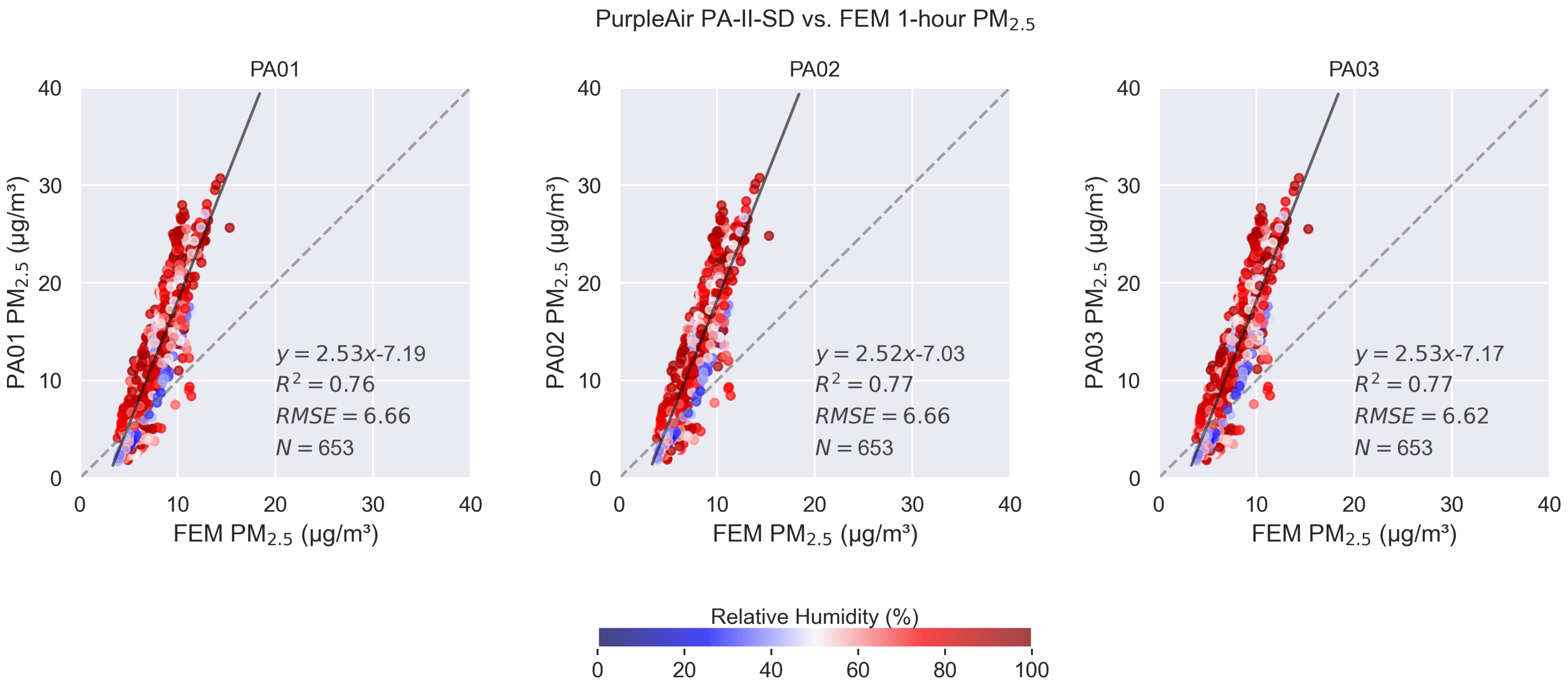

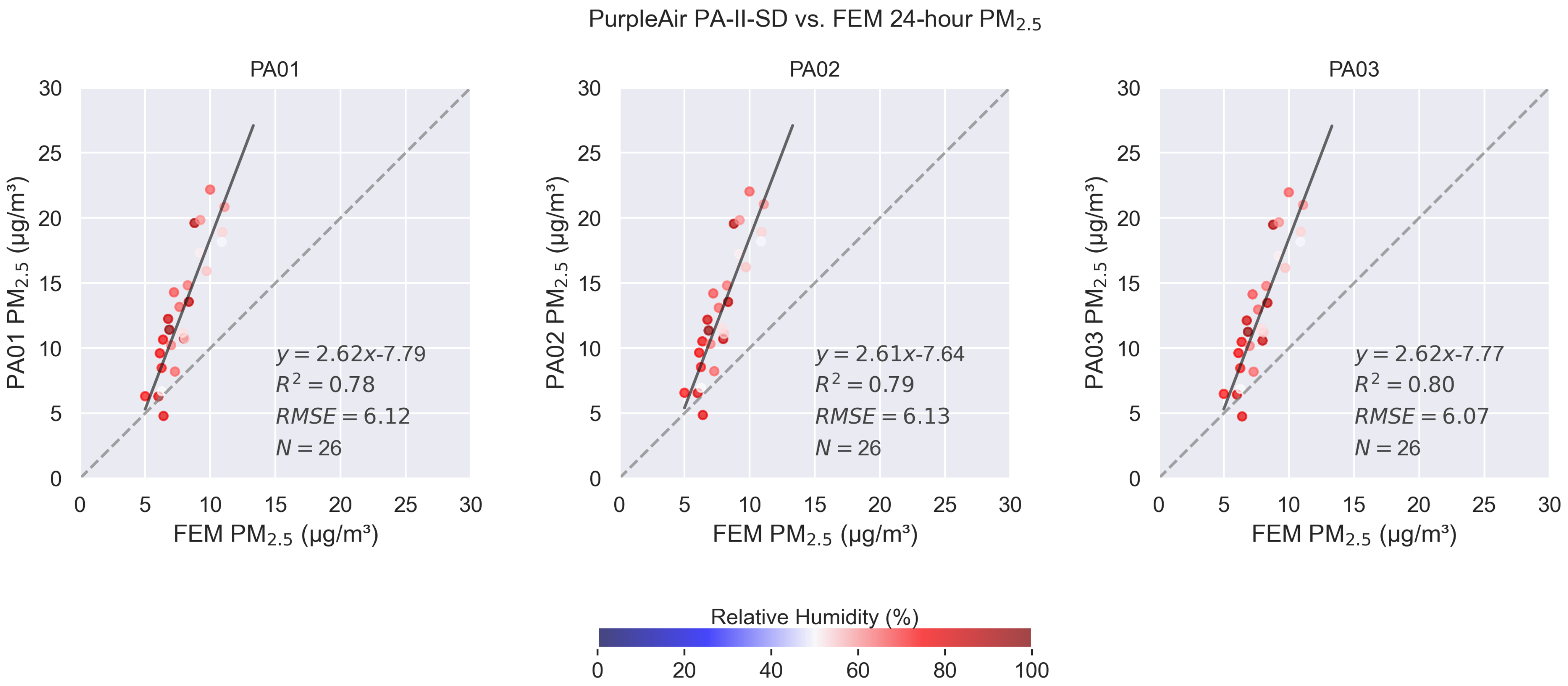

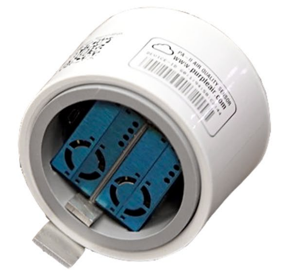

Supplemental Information

Abbreviations used in Supplemental Information

FRM	Federal Reference Method
FEM	Federal Equivalent Method
SOP	Standard Operating Procedure
QAPP	Quality Assurance Project Plan
QC	Quality Control

Supplemental Documentation	Attached	Description & URL or file path to documentation
Field observations and sensor data flags	☑	See NC-PAR-Page 6 of this testing report
Maintenance logs	☐	No logs recorded during testing
Standard operating procedure(s)	☐	U.S. EPA Office Of Research and Development SOP available upon request
Photos of equipment setup and testing	☑	See NC-PAR-Page 5 of this testing report
Product specifications sheet(s)	☑	PurpleAir: PA-II-SD Product website
Product manual(s)	☐	N/A
Data storage and transmission method	☑	See NC-PAR-Page 6 of this testing report
Data correction approach	☑	See NC-PAR-Page 6 of this testing report
Issues encountered	☑	See NC-PAR-Page 6 of this testing report
Data analysis/correction scripts and version	☑	Averaging and processing of data, calculation of performance metrics, and generation of figures and other supplementary material for analysis were obtained using Python 3.9.7 with the packages sensortoolkit v0.8.3b2, pandas 1.3.5, NumPy 1.21.2, Matplotlib 3.5.0, statsmodels 0.13.0, and seaborn 0.11.2. All packages are available from the Python Package Index (PyPI) at https://pypi.org. The integrated development environment (IDE) Spyder 5.1.5 was used for scripting and data visualization. Version control for the Python base, packages, and IDE were all managed by conda 4.11.0.
Air Monitoring Station QAPP	☐	U.S. EPA Office Of Research and Development QAPP available upon request
Summary of FRM/FEM monitor QC checks	☑	See NC-PAR-Page 7 of this testing report
Manufacturer website for FRM/FEM monitor	☑	Teledyne API: T640/T640X Product website
FRM/FEM monitor manual	☑	Teledyne API: T640/T640X Product Manual
FRM/FEM monitor specifications sheet(s)	☑	Teledyne API: T640/T640X Specification Sheet
Other documents	☐	

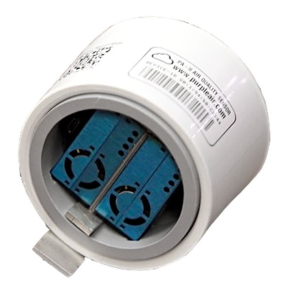

Supplemental Information: Photos of Testing Site and Equipment Setup

Site Description:

The Burdens Creek Ambient Monitoring Innovation Research Station (AIRS) site is located on the U.S. EPA, RTP campus and is situated between Alexander Drive and Route 147. The site is intended to represent a neighborhood-scale site as defined in *40 CFR Part 58, Appendix D*. U.S. EPA's Office of Air Quality Planning and Standards (OAQPS) operates reference grade instruments in a free-standing shelter situated directly adjacent to the sensor testing platform.

Figure 1: PurpleAir PA-II-SD sensors (indicated by red arrow) attached to mounting assembly at evaluation site.

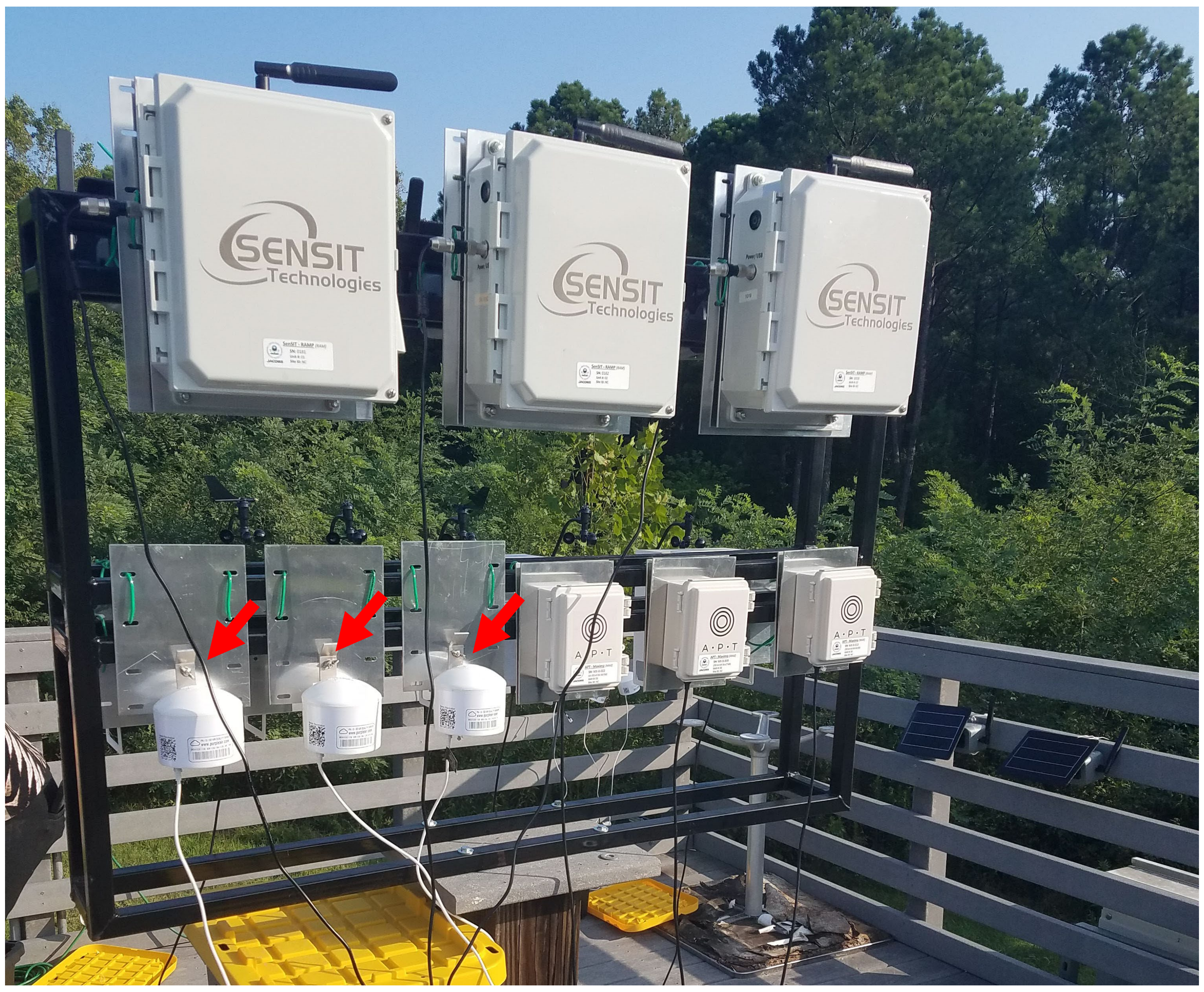

Figure 2: Observation deck at evaluation site. Approximate location of sensors indicated by the red arrow, the approximate location of T640x FEM (housed within sampling trailer) indicated by the black arrow. Sensors and FEM instrumentation are separated by approximately 15 meters.

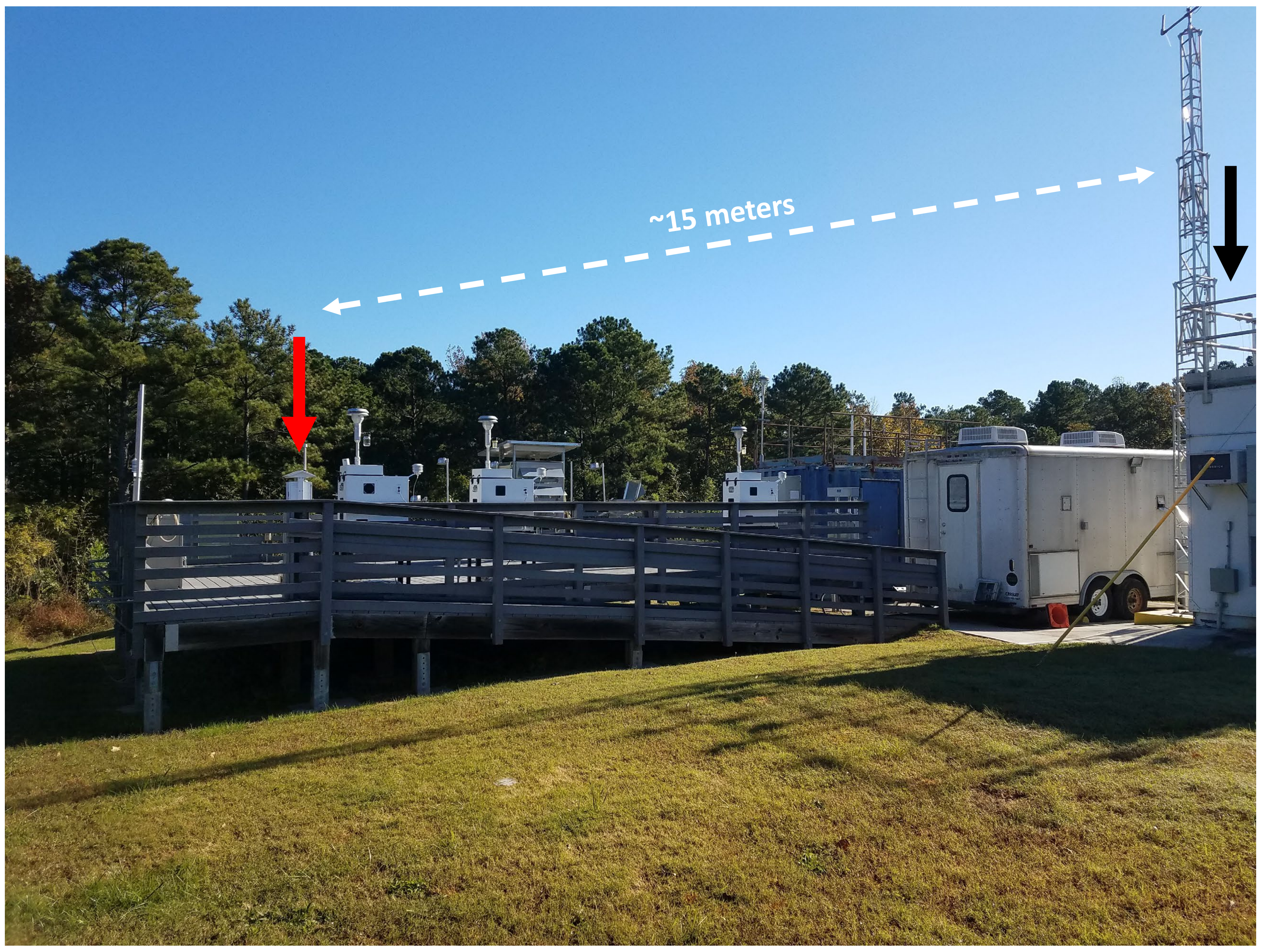

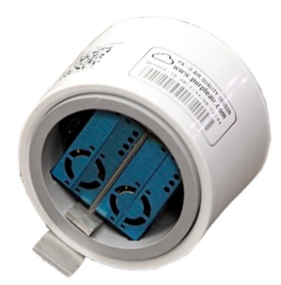

Data Storage and Transmission Method

The PurpleAir PA-II-SD records data at a 2-minute[1] sampling interval. Data is transmitted to the PurpleAir Cloud via Wi-Fi and stored on an internal MicroSD card. Due to the differing file structure between remote and direct download methods and the lag associated with cloud data download, weekly raw data were obtained primarily via onboard SD card. Each field site operator was provided two labeled MicroSD cards for sensor units that they used to swap out each week. Data from the collected card was then read and processed off-site.

Data Correction Approach

Data sets recorded by PurpleAir PA-II-SD contain two correction factors for PM concentrations, referred to internally as “CF=ATM” and “CF=1”. $PM_{2.5}$ concentrations are recorded concurrently for each correction factor, and corresponding concentrations agree closely up to approximately 30 µg/m^3, above which one correction factor reflects concentrations roughly 1.5 times concentrations for the other correction factor. During a separate analysis by ORD, concentrations for the higher correction factor were determined to have slightly higher correlation with collocated reference measurements. As a result, data for the higher correction factor were used for this study. Prior to firmware version 5.00 (released on 11/30/2019), headers for concentrations corresponding to each correction factor were incorrectly labeled (i.e., CF=1 was labeled CF=ATM and vice versa). Testing indicated in this report was completed prior to this firmware update, and thus data sets acquired for PA-II-SD units had swapped $PM_{2.5}$ CF labels. Recorded PA-II-SD data sets indicate that the CF=ATM correction factor was the higher CF as labeled (following firmware update 5.00, this data channel corresponds to CF=1) and sensor data shown in this report correspond to this correction factor.

After acquisition, the raw data was processed using the *sensortoolkit* python code library (v0.8.3b2). A continuous data set at the recorded sampling frequency was written to a .csv file. 1-hour and 24-hour averaged data sets were generated using a 75% completeness threshold and saved as separate .csv files. Simultaneous measurement from the PurpleAir PA-II’s dual PMS5003 sensors (labeled channels A and B) allows for identification of outliers resulting in large-scale disagreement between recorded values. The outlier removal method of Barkjohn et al. 2021[2] was applied to both 1-hour and 24-hour averaged A and B measurement pairs. This method includes thresholds for the absolute difference and the percent difference between hourly channel values. A-B channel averages were not included for hours where the absolute difference between channel values was greater than 5 µg/m^3 and the percent difference exceeded 70%. This outlier removal method is in use on the AirNow Fire and Smoke map and has been used for this evaluation since it requires no external information (e.g., data from the monitor) and no assumptions about typical PM2.5 concentrations (e.g., removing data over a certain threshold concentration).

Issues Encountered

Pre-deployment observations

- Prior to the testing period indicated in this report, the PurpleAir PA-II-SD unit was briefly collocated for a period of approximately one week at EPA’s Ambient Monitoring Innovative Research Station (AIRS) in Research Triangle Park, NC. The unit was tested for proper operation, including powering on and data logging (either to the onboard SD card or via hotspot Wi-Fi connection to the PurpleAir Cloud), sampling, and acquisition of recorded data from either onboard memory or cloud storage. Units that did not pass this pre-deployment testing were returned to the manufacturer and constituted approximately 1 in 10 units tested for subsequent performance evaluation.

Field observations and sensor data flags

PurpleAir PA-II-SD units were deployed at the AIRS monitoring site on 8/1/2019. The PA-II-SD units operated nominally during the testing period and did not require replacement or repair.

During the testing period, no data flags logged by field technicians were recorded.

[1]As of version 4.02, updated across devices on 5/31/2019. Prior to this firmware version, devices recorded measurements at 80-second intervals

[2]Barkjohn, K. K., Gantt, B., and Clements, A. L.: Development and application of a United States-wide correction for PM2.5 data collected with the PurpleAir sensor, Atmos. Meas. Tech., 14, 4617–4637, https://doi.org/10.5194/amt-14-4617-2021, 2021.

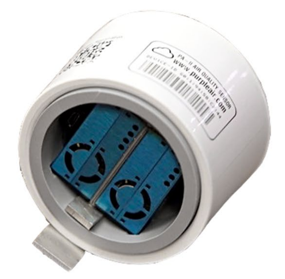

Description of Data Flags

OAQPS manages data logged by reference monitors at the AIRS evaluation site using the Envidas data acquisition system software from DR DAS LTD[1]. Envidas contains over 100 data flags which are configurable by the monitoring agency and can be triggered based on instrument status. Appendix B Table 1 contains a description of Envidas data flags including the numeric code and status code name (a brief textual description, the table containing data flags below includes code names for events encountered by the reference monitor under the "Flag" column). Appendix B Table 1 also indicates whether reference data are invalidated for a given data flag. For this report, reference data were invalidated for periods where a data flag was logged and if the corresponding entry for the data flag in the "Data Status Invalidation" column in Appendix B Table 1 indicates data should be invalidated.

Data Flags Recorded During Testing

FRM/FEM Monitor	Timestamp (UTC)	Flag
Teledyne API T640X (Data acquired via local transfer from OAQPS)	2019-08-08 14:02:00+0000	<Samp
	2019-08-13 14:58:00+0000	<Samp
	2019-08-21 12:56:00+0000 to 2019-08-21 13:29:00+0000	Down
	2019-08-21 13:30:00+0000	<Samp
	2019-08-21 21:29:00+0000 to 2019-08-21 21:34:00+0000	<Samp
	2019-08-21 21:30:00+0000 to 2019-08-21 21:33:00+0000	Down
	2019-08-23 04:28:00+0000 to 2019-08-23 07:12:00+0000	NoData
	2019-08-23 07:13:00+0000	<Samp
	2019-08-26 21:18:00+0000	<Samp
	2019-08-28 15:07:00+0000	<Samp

Meteorological Instrument	Timestamp (UTC)	Flag
[RM Young 41382 VC Temperature and Relative Humidity Monitor (Data acquired via local transfer from OAQPS)	2019-08-08 14:02:00+0000	<Samp
	2019-08-13 14:58:00+0000	<Samp
	2019-08-23 04:28:00+0000 to 2019-08-23 07:12:00+0000	NoData
	2019-08-23 07:13:00+0000	<Samp
	2019-08-26 21:18:00+0000	<Samp
	2019-08-28 15:07:00+0000	<Samp

[1] "Contact us" DR DAS LTD, 2022, https://dr-das.com/Home/Contact.html Accessed June 3 2022

Testing Report - $PM_{2.5}$ Base Testing

QuantAQ ARISense

This report reflects out-of-the-box performance

Initial Base Testing - RTP, NC

U.S. Environmental Protection Agency
Office of Research and Development
PI: Clements.Andrea@epa.gov
919-541-1363

March 2020—April 2020

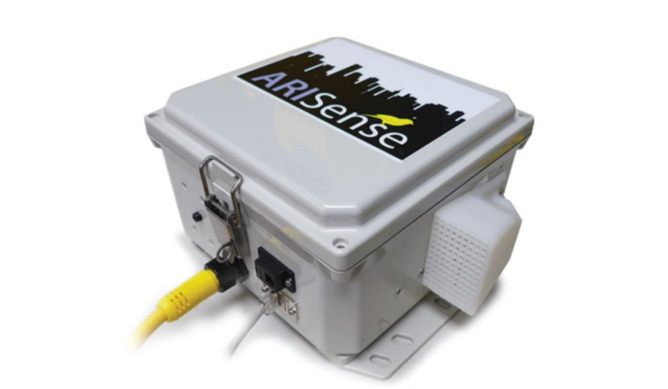

Image Attribution: QuantAQ, Inc (Somerville, MA)

Deployment Details

Testing Organization and Site Information	
Testing organization (Name, Organization type, Contact website)	U.S. Environmental Protection Agency - Office of Research and Development<br>Federal Government<br>Air Sensor Toolbox | U.S. EPA Website
Testing location (City, State, Latitude and Longitude)	Ambient Monitoring Innovative Research Station (AIRS)<br>RTP, NC<br>35.88951, -78.874572
AQS site ID	37 – 063 – 0099
Sampling timeframe (MM-DD-YY)	03-13-20 to 04-12-20
Sensor data source	QuantAQ Cloud download
Reference data source	OAQPS file transfer

Sensor Information			
Manufacturer, model	QuantAQ ARISense		
Device firmware version	Received by EPA on 4/30/2019		
Sampling time interval	1-minute		
Sensor serial numbers	ARS_01	ARS_02	ARS_03
Issues encountered during deployment?	☐	No Issues	

FRM/FEM Information	
Manufacturer, model, designation	Teledyne Advanced Pollution Instrumentation T640 FEM
Sampling time interval	1-hour averaging
Date of calibration	As required by 40 CFR Part 58 and the Burdens Creek QAPP maintained by OAQPS
Date of flowrate verification check	Monthly as required by 40 CFR Part 58 Appendix A
Description, date(s) of maintenance activities	See NC-ARS-Page 7 of this testing report

Time Series Plots

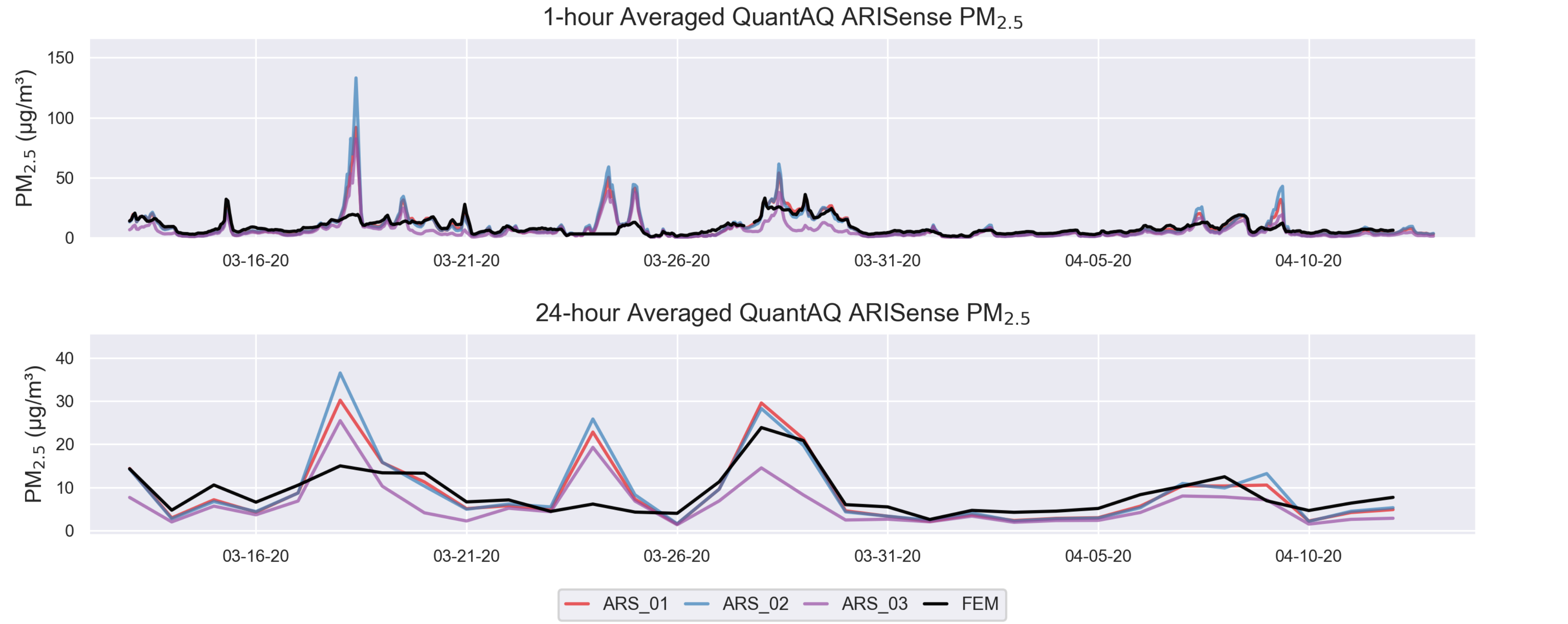

Scatter Plots: Comparison to FRM/FEM

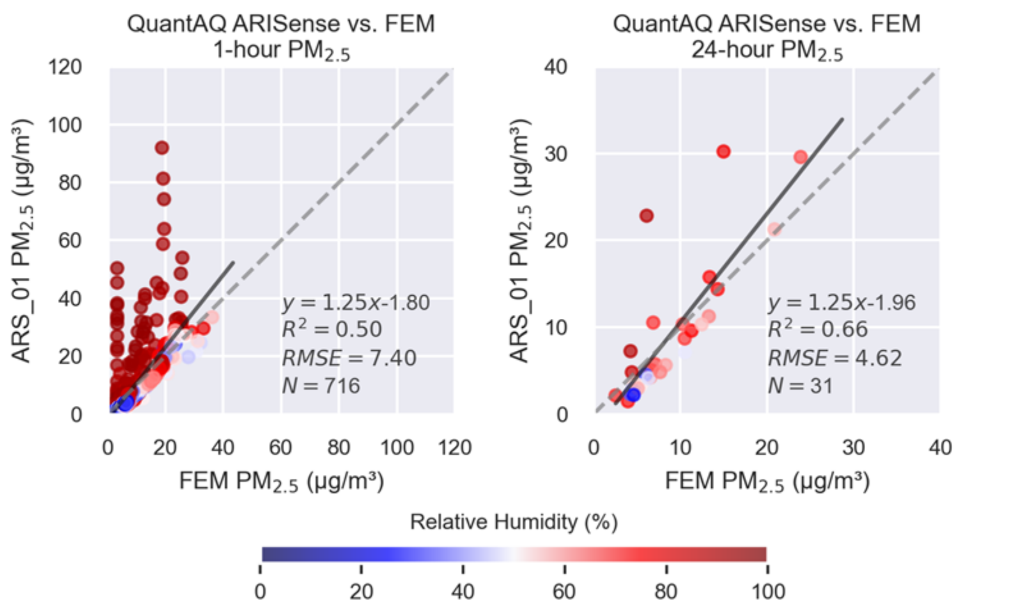

Range of FRM/FEM concentrations over duration of base test (µg/m³)	[1-hr] 1.0-36.1, avg: 8.6, [24-hr] 2.5-23.9, avg: 8.4
Number of 24-hr periods in FRM/FEM monitor measurements with a goal concentration ≥ 25 µg/m³	0

Performance Metrics★

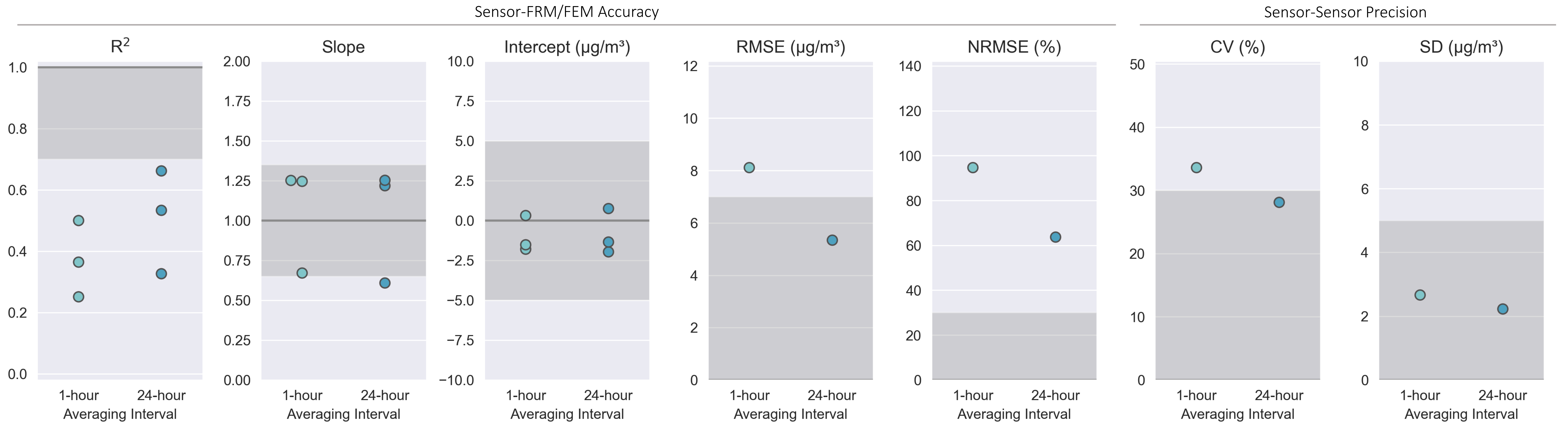

Meteorological Conditions During Deployment

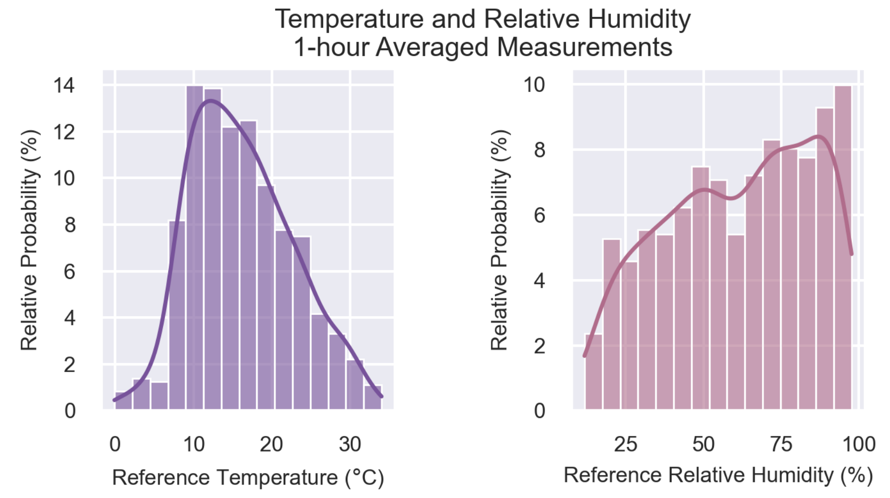

Number of 24-hr periods outside sensor manufacture-listed temperature operational range (no operational range specified)	-
Number of 24-hr periods outside sensor manufacture-listed relative humidity operational range (no operational range specified)	-

Meteorological Influence

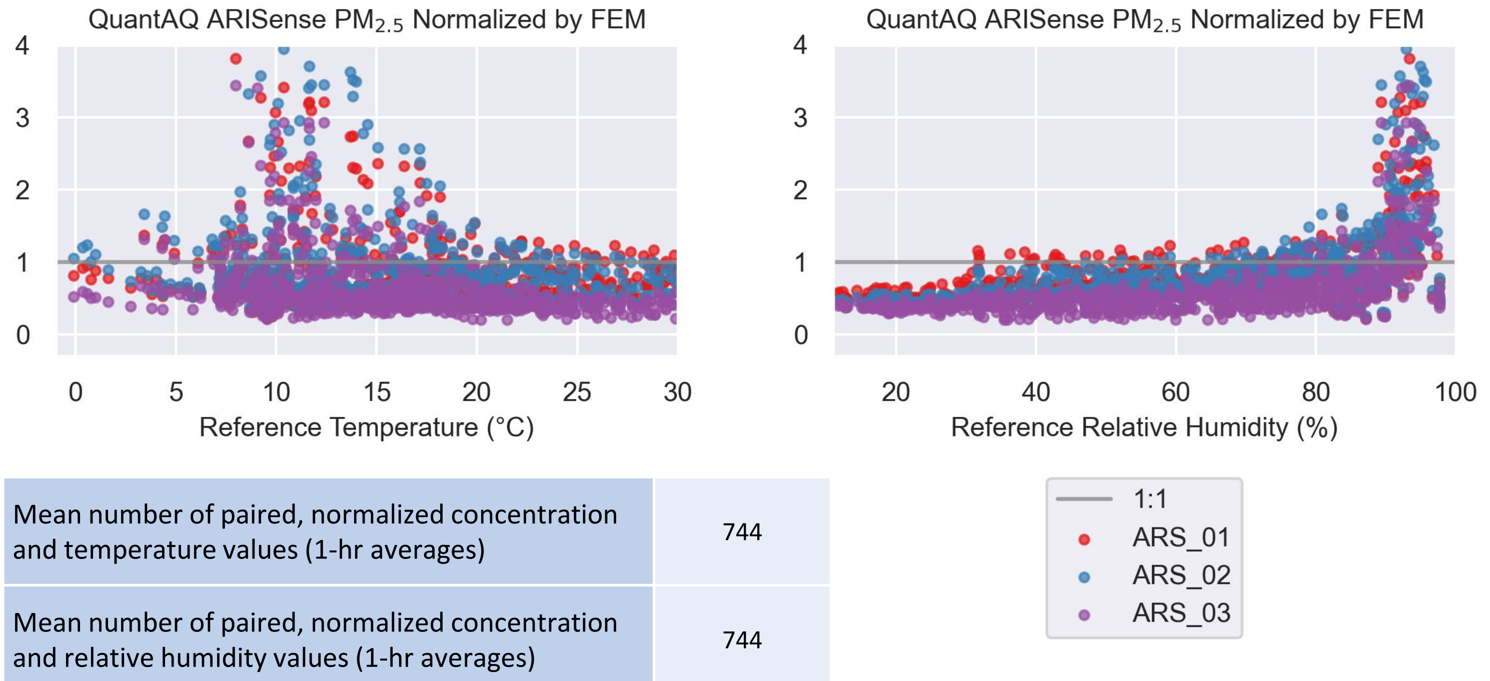

Mean number of paired, normalized concentration and temperature values (1-hr averages)	744
Mean number of paired, normalized concentration and relative humidity values (1-hr averages)	744

★For evaluations with greater than three sensors, grouping individual sensor metrics into boxplots is recommended for displaying results. Note that this recommendation does not apply to metrics computed as a single value for all sensors over the whole evaluation group, such as RMSE, NRMSE, CV, and standard deviation.

U.S. Environmental Protection Agency
Office of Research and Development
PI: Clements.Andrea@epa.gov
919-541-1363

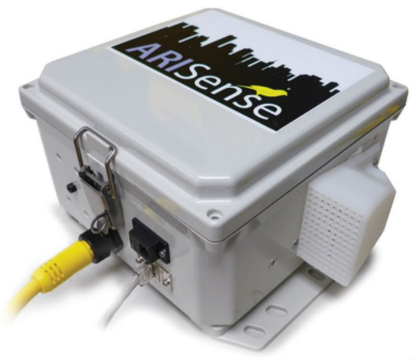

Image Attribution: QuantAQ, Inc (Somerville, MA)

Tabular Statistics

Sensor-FRM/FEM Correlation

	Bias and Linearity						Data Quality			
	R^2		Slope		Intercept (µg/m³)		Uptime (%)		Number of paired sensor and FRM/FEM concentration values	
	1-Hour ○○○	24-Hour ○○○	1-Hour ●●●	24-Hour ●●○	1-Hour ●●●	24-Hour ●●●	1-Hour ●●●	24-Hour ●●●	1-Hour	24-Hour
Metric Target Range	≥ 0.70	≥ 0.70	1.0 ± 0.35	1.0 ± 0.35	-5 ≤ b ≤ 5	-5 ≤ b ≤ 5	75%*	75%*	-	-
Sensor ARS_01	0.50	0.66	1.25	1.25	-1.80	-1.96	100	100	716	31
Sensor ARS_02	0.36	0.53	1.25	1.22	-1.52	-1.35	100	100	716	31
Sensor ARS_03	0.25	0.33	0.67	0.61	0.31	0.75	100	100	716	31
Mean	0.37	0.51	1.06	1.03	-1.00	-0.85	100	100	716	31

	Error			
	RMSE (µg/m³)		NRMSE (%)	
	1-Hour ☆	24-Hour ★	1-Hour ☆	24-Hour ☆
Metric Target Range	≤ 7.0	≤ 7.0	≤ 30.0	≤ 30.0
Deployment Value	8.1	5.3	94.8	63.8

Device-specific metrics (computed for each sensor in evaluation)
○○○ Metric value for none of devices tested falls within the target range
●○○ Metric value for one of devices tested falls within the target range
●●○ Metric value for two of devices tested falls within the target range
●●● Metric value for three of devices tested falls within the target range

Single-valued metrics (computed via entire evaluation dataset)
☆ Indicates that the metric value is not within the target range
★ Indicates that the metric value is within the target range

Sensor-Sensor Precision

	Precision (between collocated sensors)				Data Quality	
	CV (%)		SD (µg/m³)		Number of concurrent sensor concentration pairs	
	1-Hour ☆	24-Hour ★	1-Hour ★	24-Hour ★	1-Hour	24-Hour
Metric Target Range	≤ 30.0	≤ 30.0	≤ 5.0	≤ 5.0	-	-
Deployment Value	33.6	28.1	2.7	2.2	744	31

*This value is only a recommendation for ensuring data quality and is not included in the list of target values discussed in Section 4 of the Performance Testing Protocols, Metrics, and Target Values for Fine Particulate Matter Air Sensors document.

Testing Report - $PM_{2.5}$ Base Testing

QuantAQ ARISense

This report reflects out-of-the-box performance

Initial Base Testing - RTP, NC
U.S. Environmental Protection Agency
Office of Research and Development
PI: Clements.Andrea@epa.gov
919-541-1363
March 2020—April 2020

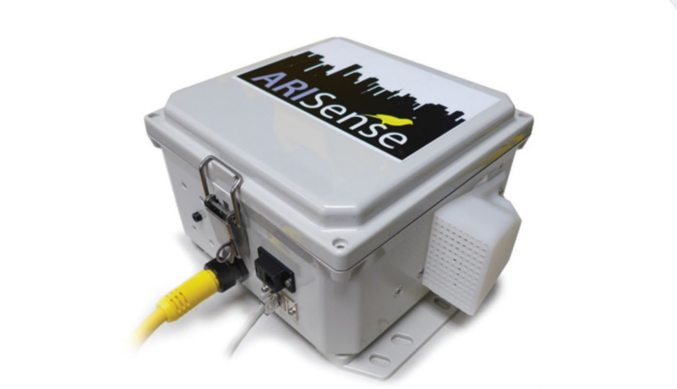

Image Attribution: QuantAQ, Inc (Somerville, MA)

Sensor-FRM/FEM Scatter Plots

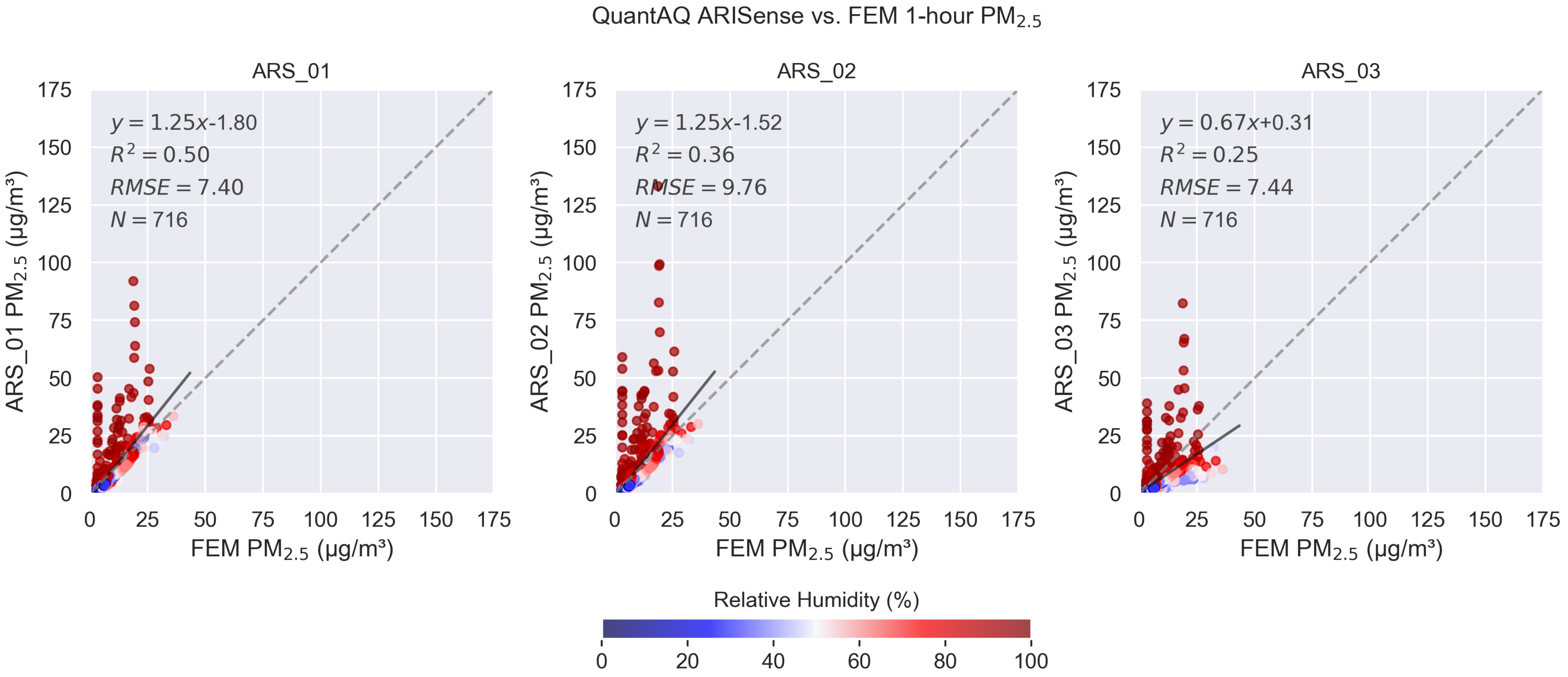

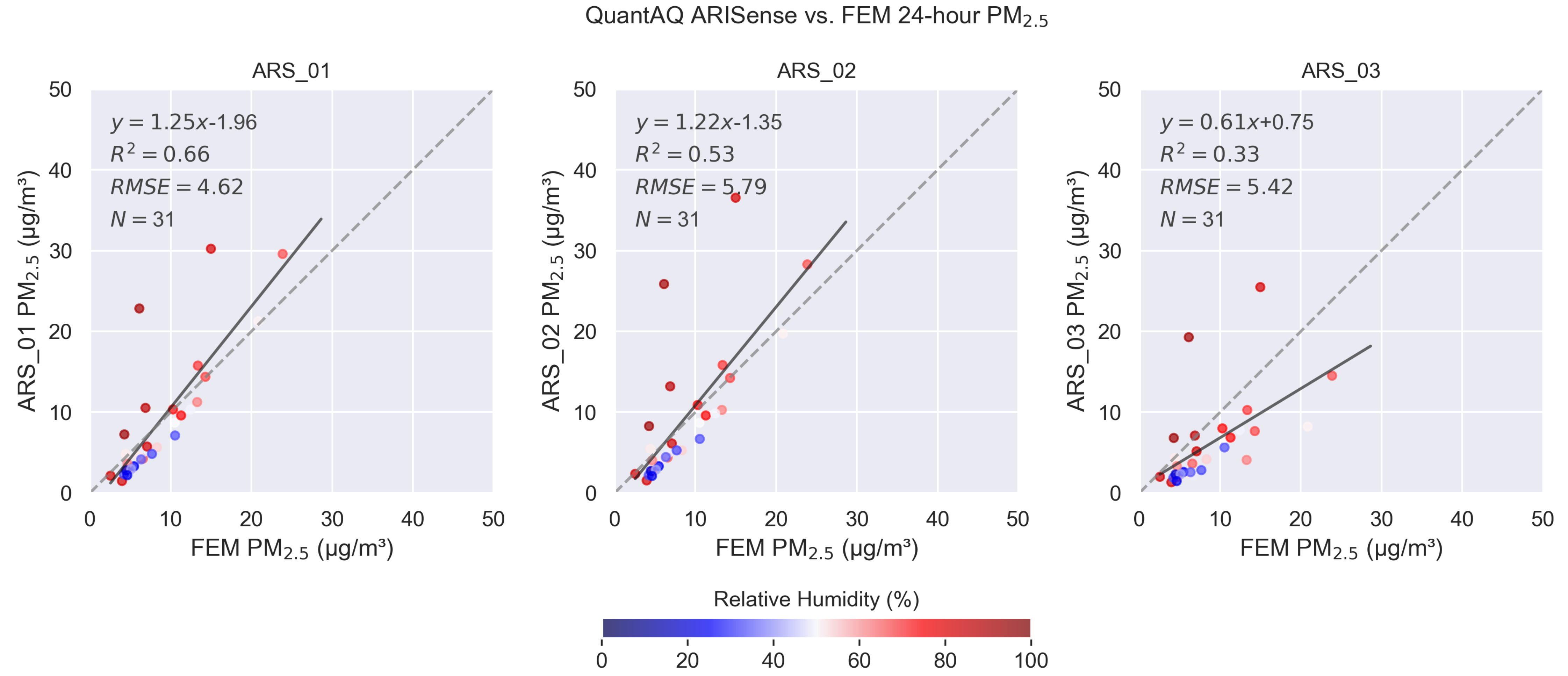

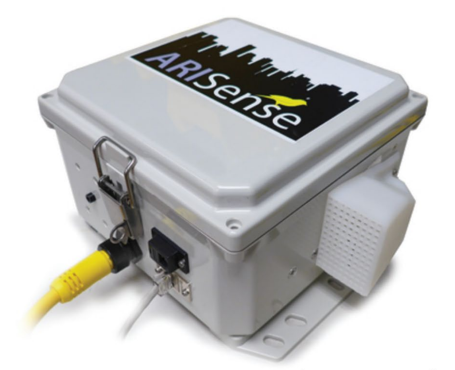

Image Attribution: QuantAQ, Inc (Somerville, MA)

Supplemental Information

Abbreviations used in Supplemental Information

FRM	Federal Reference Method
FEM	Federal Equivalent Method
SOP	Standard Operating Procedure
QAPP	Quality Assurance Project Plan
QC	Quality Control

Supplemental Documentation	Attached	Description & URL or file path to documentation
Field observations and sensor data flags	☑	See NC-ARS-Page 6 of this testing report
Maintenance logs	☐	No logs recorded during testing
Standard operating procedure(s)	☐	U.S. EPA Office Of Research and Development SOP available upon request
Photos of equipment setup and testing	☑	See NC-ARS-Page 5 of this testing report
Product specifications sheet(s)	☑	QuantAQ and Particles Plus: ARISense Specification Sheet
Product manual(s)	☐	N/A
Data storage and transmission method	☑	See NC-ARS-Page 6 of this testing report
Data correction approach	☑	See NC-ARS-Page 6 of this testing report
Issues encountered	☑	See NC-ARS-Page 6 of this testing report
Data analysis/correction scripts and version	☑	Averaging and processing of data, calculation of performance metrics, and generation of figures and other supplementary material for analysis were obtained using Python 3.9.7 with the packages sensortoolkit v0.8.3b2, pandas 1.3.5, NumPy 1.21.2, Matplotlib 3.5.0, statsmodels 0.13.0, and seaborn 0.11.2. All packages are available from the Python Package Index (PyPI) at https://pypi.org. The integrated development environment (IDE) Spyder 5.1.5 was used for scripting and data visualization. Version control for the Python base, packages, and IDE were all managed by conda 4.11.0.
Air Monitoring Station QAPP	☐	U.S. EPA Office Of Research and Development QAPP available upon request
Summary of FRM/FEM monitor QC checks	☑	See NC-ARS-Page 7 of this testing report
Manufacturer website for FRM/FEM monitor	☑	Teledyne API: T640/T640X Product website
FRM/FEM monitor manual	☑	Teledyne API: T640/T640X Product Manual
FRM/FEM monitor specifications sheet(s)	☑	Teledyne API: T640/T640X Specification Sheet
Other documents	☐	

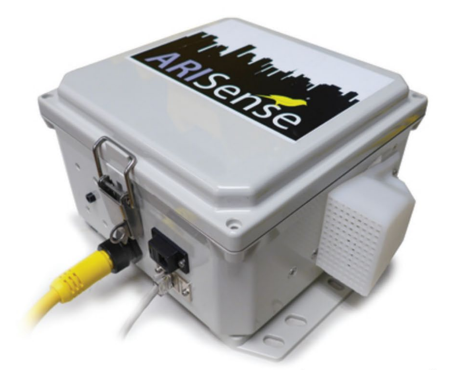

Image Attribution: QuantAQ, Inc (Somerville, MA)

Supplemental Information: Photos of Testing Site and Equipment Setup

Site Description:

The Burdens Creek Ambient Monitoring Innovation Research Station (AIRS) site is located on the U.S. EPA, RTP campus and is situated between Alexander Drive and Route 147. The site is intended to represent a neighborhood-scale site as defined in *40 CFR Part 58, Appendix D*. U.S. EPA's Office of Air Quality Planning and Standards (OAQPS) operates reference grade instruments in a free-standing shelter situated directly adjacent to the sensor testing platform.

Figure 1: Observation deck at evaluation site. Approximate location of AQY sensors indicated by the red arrow, the approximate location of T640x FEM (housed within sampling trailer) indicated by the black arrow. Sensors and FEM instrumentation are separated by approximately 15 meters.

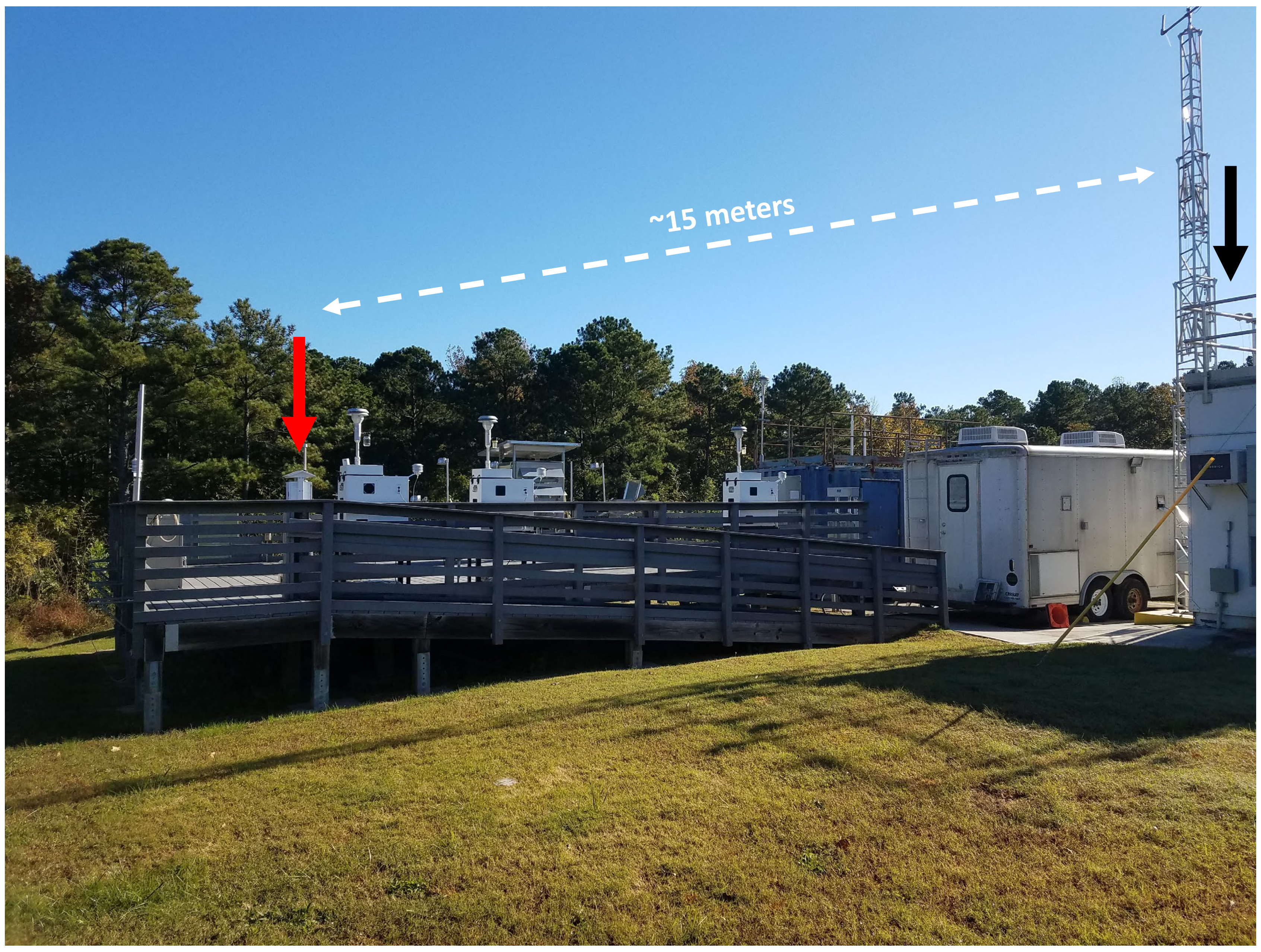

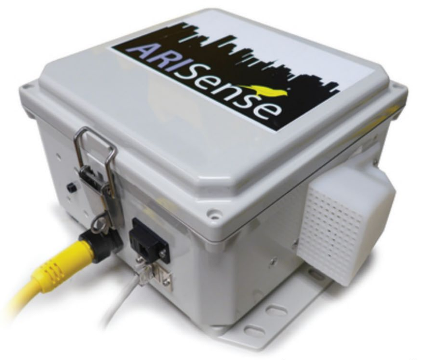

Image Attribution: QuantAQ, Inc (Somerville, MA)

Data Storage and Transmission Method

ARISense units upload data to an external cloud server at 2-minute averages via cellular signal. ARISense weekly data collections were performed by downloading data from the QuantAQ cloud server (last accessed 5/11/22).

Data Correction Approach

QuantAQ ARISense units measure PM2.5 concentrations via an optical particle counter (OPC). The OPC measures particle counts within particle size bins ranging from 0.3 microns to 10 microns, and mass concentrations for each bins are estimated from particle counts assuming constant values for density, spherical morphology, and average particle diameter. The manufacturer applies a general correction to account for the detection limitations of the OPC leading to concentrations that are closer to reality. These presets were configured by the sensor manufacturer and were not modified by EPA prior or during testing.

After acquisition, the raw data was processed using the *sensortoolkit* python code library (v0.8.3b2). A continuous data set at the recorded sampling frequency was written to a .csv file. 1-hour and 24-hour averaged data sets were generated using a 75% completeness threshold and saved as separate .csv files. Outliers were NOT removed from data sets in order to assess "out-of-the-box" sensor performance.

Issues Encountered

Pre-deployment observations

Numerous issues were encountered with the ARISense devices. Although previous models of the ARISense had used AlphaSense OPC-N2 these OPCs were unavailable at the time of manufacture. So instead, components from a particle plus OPC handheld were used.

- *Shipping damage:* On arrival to EPA many of the units were damaged due to the heavy pump breaking free during shipping and shaking around inside of the box breaking much of the plastic scaffolding and, in some cases, components. The manufacturer worked with us to fix these units.
- *Hardware and Firmware issues:* Multiple hardware and firmware issues were experienced that had to be fixed before starting the deployment and multiple manual firmware updates on both the gas sensor microcontrollers and the OPC microcontrollers were completed by researchers. The USB drives were frequently corrupted which required replacement and eventually a hardware update. In addition, the OPC microcontroller and the gas sensor microcontroller often miscommunicated. One firmware version reduced the number of bins reported by the OPC since the manufacturer determined the additional bins were not providing additional accuracy. New PCB boards were sent and installed before the tests could be run as well.
- *Initiation:* There was a high power draw when the cellular connectivity initiated and the pump was turned on. This meant that units occasionally failed to turn on at the same time and it meant these could not be powered by solar.
- *Water intake:* In addition, the OPC pump often drew in water during rainstorms leading the inline filter to saturate and the pump to fail due to overheating. Many pumps had to be replaced throughout the testing.
- *Cellular connectivity:* Some units could not consistently stay connected to the cellular, though other sensors at the same site did not experience issues. The manufacturer could not recreate this issue when they were sent back for service and the problems persisted.
- *Reset:* These units ended up being operated on timers so that they were power cycled every night which would typically get them back online if a software error had been encountered, leading to lower data loss than would have occurred with only weekly checks.

Field observations and sensor data flags

QuantAQ ARISense units were deployed at the AIRS monitoring site on 3/12/2020. The ARISense units operated nominally during the testing period and did not require replacement or repair.

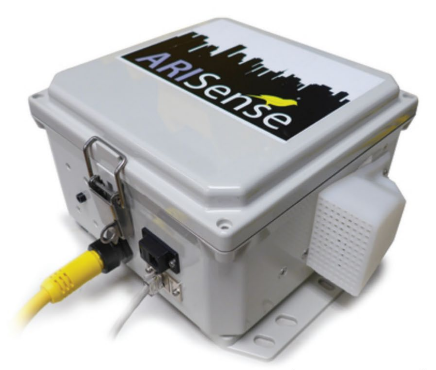

Image Attribution: QuantAQ, Inc (Somerville, MA)

Description of Data Flags

OAQPS manages data logged by reference monitors at the AIRS evaluation site using the Envidas data acquisition system software from DR DAS LTD[1]. Envidas contains over 100 data flags which are configurable by the monitoring agency and can be triggered based on instrument status. Appendix B Table 1 contains a description of Envidas data flags including the numeric code and status code name (a brief textual description, the table containing data flags below includes code names for events encountered by the reference monitor under the "Flag" column). Appendix B Table 1 also indicates whether reference data are invalidated for a given data flag. For this report, reference data were invalidated for periods where a data flag was logged and if the corresponding entry for the data flag in the "Data Status Invalidation" column in Appendix B Table 1 indicates data should be invalidated.

Data Flags Recorded During Testing

FRM/FEM Monitor	Timestamp (UTC)	Flag
Teledyne API T640X (Data acquired via local transfer from OAQPS)	2020-03-20 17:33:00+0000	<Samp
	2020-03-24 14:17:00+0000	<Samp
	2020-03-24 14:42:00+0000	OffScan
	2020-03-24 14:44:00+0000 to 2020-03-24 14:50:00+0000	OffScan
	2020-03-27 04:15:00+0000	<Samp
	2020-03-27 14:56:00+0000 to 2020-03-27 15:10:00+0000	OffScan
	2020-03-27 15:12:00+0000 to 2020-03-27 15:40:00+0000	OffScan
	2020-03-27 16:09:00+0000	OffScan
	2020-03-27 16:11:00+0000 to 2020-03-27 18:08:00+0000	OffScan
	2020-03-27 18:11:00+0000 to 2020-03-27 19:56:00+0000	OffScan
	2020-03-27 19:57:00+0000	<Samp
	2020-03-29 03:42:00+0000	<Samp
	2020-03-31 01:58:00+0000	<Samp
	2020-03-31 17:25:00+0000	<Samp
	2020-04-01 08:57:00+0000	<Samp
	2020-04-04 14:59:00+0000	<Samp
	2020-04-07 20:48:00+0000	<Samp
	2020-04-08 18:54:00+0000	<Samp
	2020-04-10 10:51:00+0000	<Samp
	2020-04-10 18:30:00+0000	<Samp
	2020-04-11 17:47:00+0000	<Samp
	2020-04-12 09:15:00+0000	<Samp

Meteorological Instrument	Timestamp (UTC)	Flag
RM Young 41382 VC Temperature and Relative Humidity Monitor (Data acquired via local transfer from OAQPS)	2019-08-08 14:02:00+0000	<Samp
	2019-08-13 14:58:00+0000	<Samp
	2019-08-23 04:28:00+0000 to 2019-08-23 07:12:00+0000	NoData [maintenance]
	2019-08-23 07:13:00+0000	<Samp
	2019-08-26 21:18:00+0000	<Samp
	2019-08-28 15:07:00+0000	<Samp

[1] "Contact us" DR DAS LTD, 2022, https://dr-das.com/Home/Contact.html Accessed June 3 2022

Testing Report - $PM_{2.5}$ Base Testing

SENSIT RAMP

This report reflects out-of-the-box performance

Initial Base Testing - RTP, NC

U.S. Environmental Protection Agency
Office of Research and Development
PI: Clements.Andrea@epa.gov
919-541-1363

October 2019—November 2019

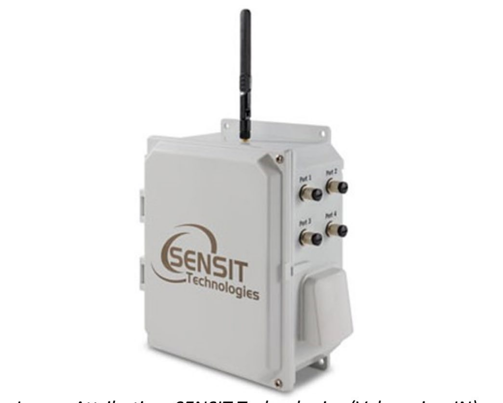

Image Attribution: SENSIT Technologies (Valparaiso, IN)

Deployment Details

Testing Organization and Site Information	
Testing organization (Name, Organization type, Contact website)	U.S. Environmental Protection Agency - Office of Research and Development<br>Federal Government<br>Air Sensor Toolbox | U.S. EPA Website
Testing location (City, State, Latitude and Longitude)	Ambient Monitoring Innovative Research Station (AIRS)<br>RTP, NC<br>35.88951, -78.874572
AQS site ID	37 – 063 – 0099
Sampling timeframe (MM-DD-YY)	08-02-19 to 09-01-19
Sensor data source	Onboard MicroSD card
Reference data source	OAQPS file transfer

Sensor Information			
Manufacturer, model	SENSIT RAMP		
Device firmware version	RAM01: 190313_AQ_v9.30,<br>RAM02: 190308_AQ_v9.30,<br>RAM04: 190730_AQ_v9.34		
Sampling time interval	15-seconds		
Sensor serial numbers	RAM_01	RAM_02	RAM_04
Issues encountered during deployment?	☑	See NC-RAM-Page 6 of this testing report	

FRM/FEM Information	
Manufacturer, model, designation	Teledyne Advanced Pollution Instrumentation T640 FEM
Sampling time interval	1-hour averaging
Date of calibration	As required by 40 CFR Part 58 and the Burdens Creek QAPP maintained by OAQPS
Date of flowrate verification check	Monthly as required by 40 CFR Part 58 Appendix A
Description, date(s) of maintenance activities	See NC-RAM-Page 7 of this testing report

Time Series Plots

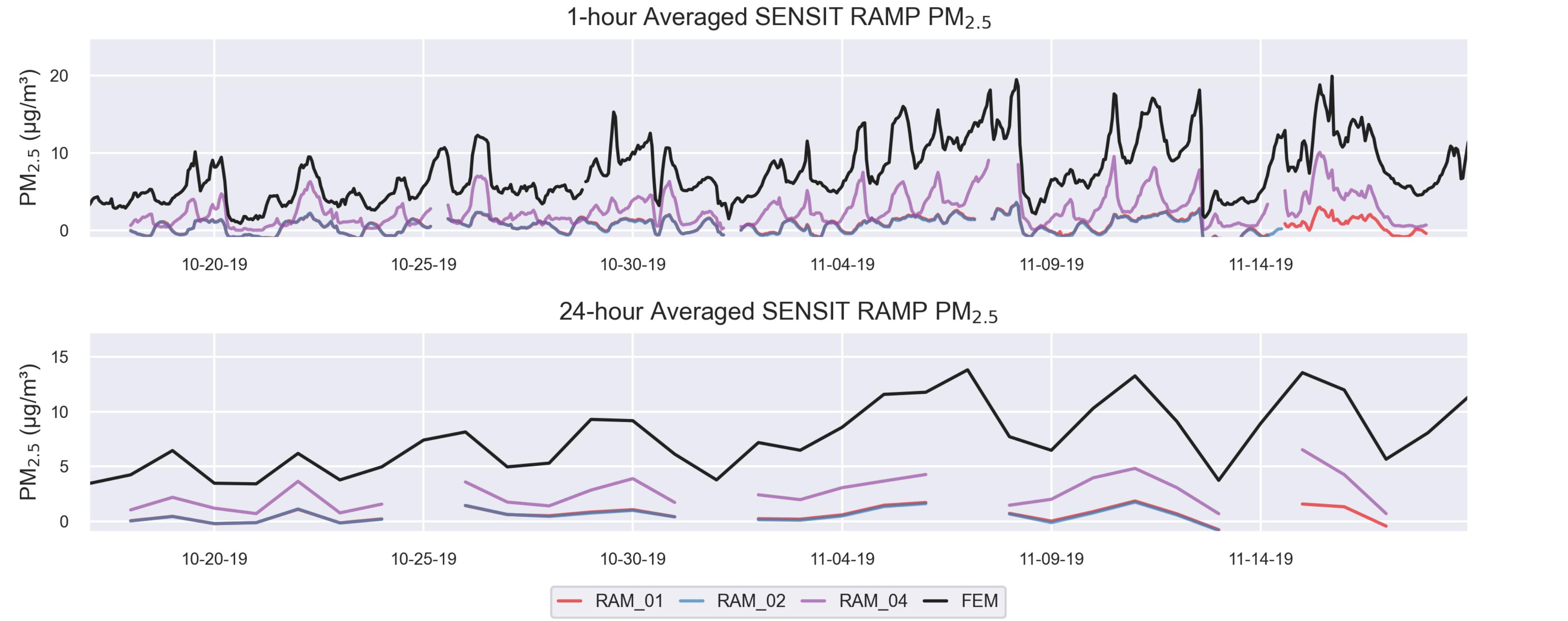

Scatter Plots: Comparison to FRM/FEM

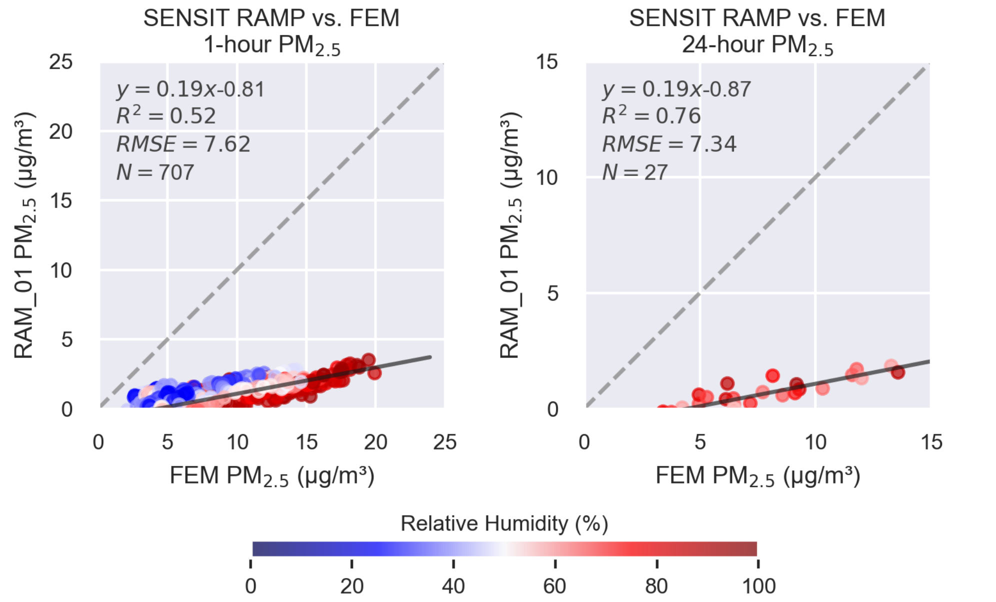

Range of FRM/FEM concentrations over duration of base test (µg/m³)	0.9-19.9 (1-hr),<br>3.4-13.8 (24-hr)
Number of 24-hr periods in FRM/FEM monitor measurements with a goal concentration ≥ 25 µg/m³	0

Performance Metrics★

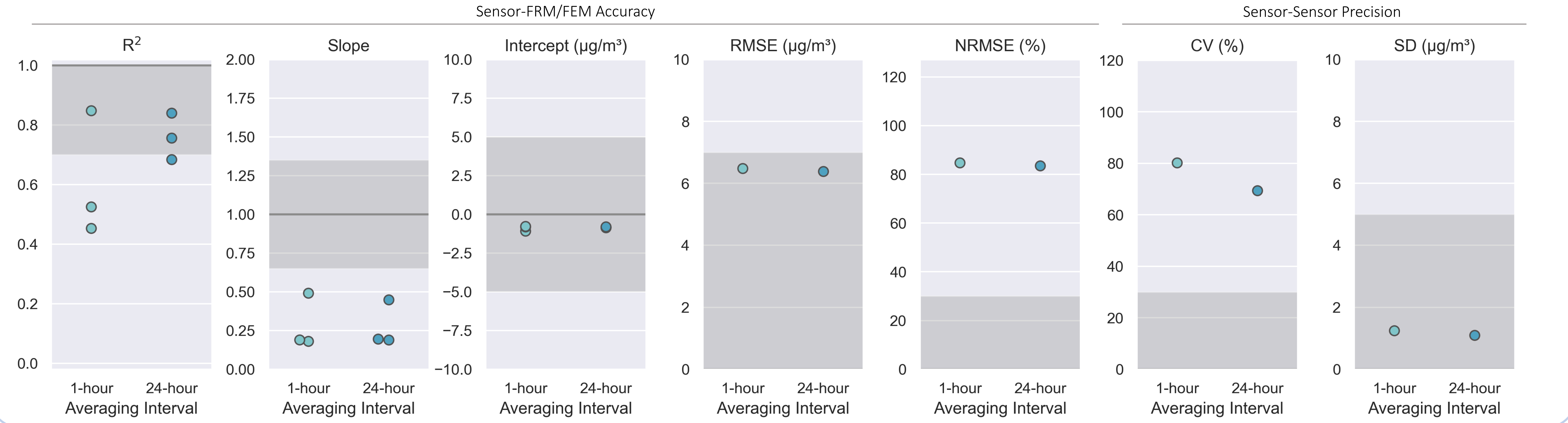

Meteorological Conditions During Deployment

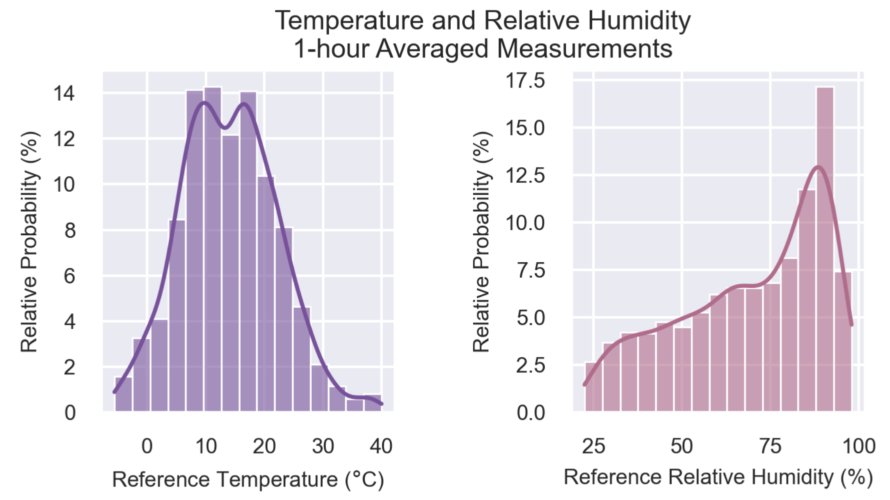

Number of 24-hr periods outside sensor manufacture-listed temperature operational range (-20 to 50 °C)	0
Number of 24-hr periods outside sensor manufacture-listed relative humidity operational range (no operational range specified)	-

Meteorological Influence

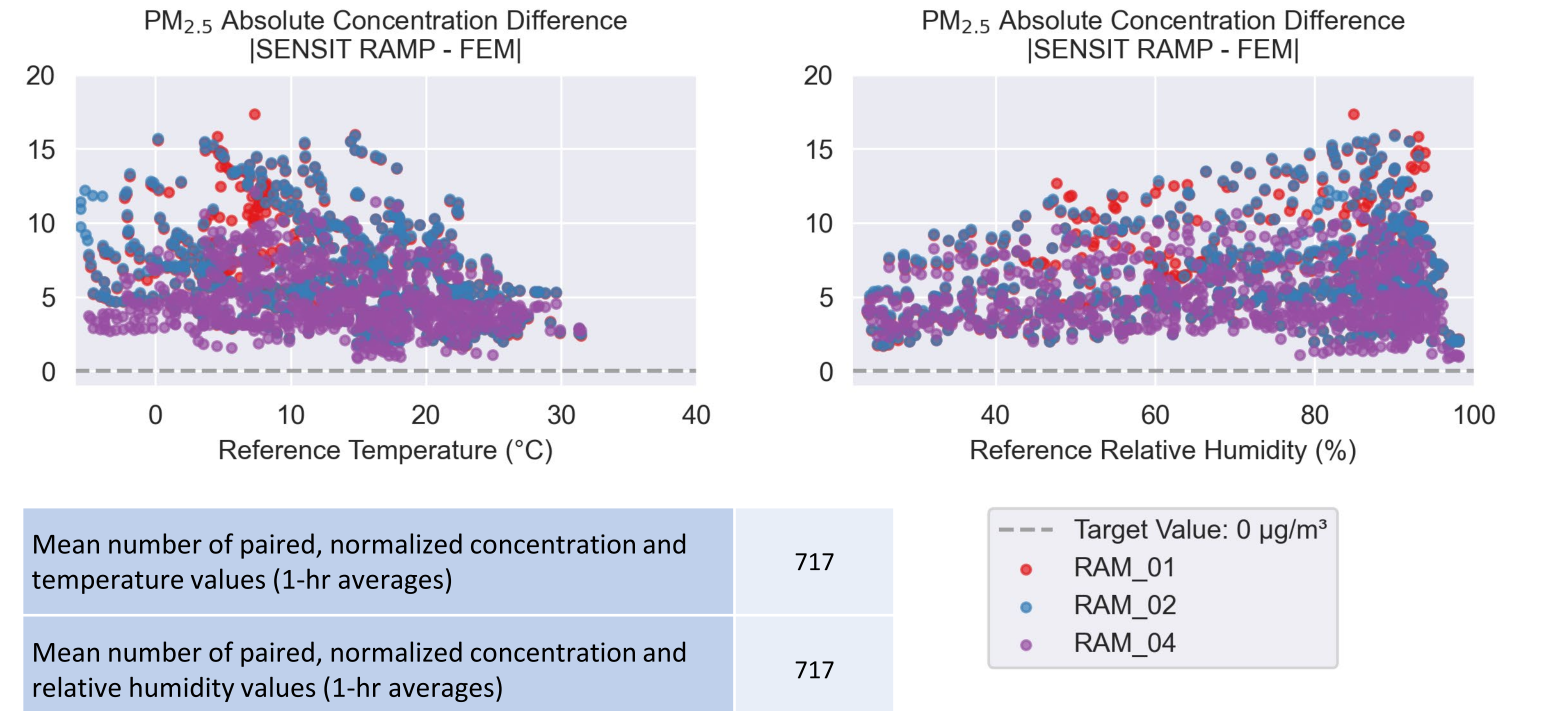

Mean number of paired, normalized concentration and temperature values (1-hr averages)	717
Mean number of paired, normalized concentration and relative humidity values (1-hr averages)	717

★For evaluations with greater than three sensors, grouping individual sensor metrics into boxplots is recommended for displaying results. Note that this recommendation does not apply to metrics computed as a single value for all sensors over the whole evaluation group, such as RMSE, NRMSE, CV, and standard deviation.

Testing Report - $PM_{2.5}$ Base Testing

SENSIT RAMP

This report reflects out-of-the-box performance

Initial Base Testing - RTP, NC
U.S. Environmental Protection Agency
Office of Research and Development
PI: Clements.Andrea@epa.gov
919-541-1363
October 2019—November 2019

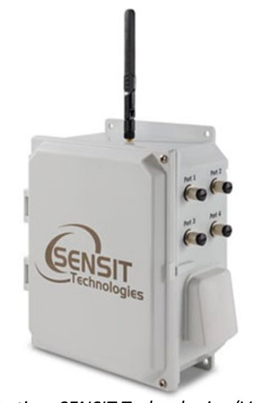

Image Attribution: SENSIT Technologies (Valparaiso, IN)

Tabular Statistics

Sensor-FRM/FEM Correlation

	Bias and Linearity						Data Quality			
	R^2		Slope		Intercept (µg/m³)		Uptime (%)		Number of paired sensor and FRM/FEM concentration values	
	1-Hour ●○○	24-Hour ●●○	1-Hour ○○○	24-Hour ○○○	1-Hour ●●●	24-Hour ●●●	1-Hour ●●●	24-Hour ●●●	1-Hour	24-Hour
Metric Target Range	≥ 0.70	≥ 0.70	1.0 ± 0.35	1.0 ± 0.35	-5 ≤ b ≤ 5	-5 ≤ b ≤ 5	75%*	75%*	-	-
Sensor RAM_01	0.52	0.76	0.19	0.19	-0.81	-0.87	95	87	707	27
Sensor RAM_02	0.45	0.68	0.18	0.19	-0.78	-0.84	96	86	633	24
Sensor RAM_04	0.85	0.84	0.49	0.45	-1.09	-0.81	94	87	700	27
Mean	0.61	0.76	0.29	0.28	-0.89	-0.84	95	87	680	26

	Error			
	RMSE (µg/m³)		NRMSE (%)	
	1-Hour ★	24-Hour ★	1-Hour ☆	24-Hour ☆
Metric Target Range	≤ 7.0	≤ 7.0	≤ 30.0	≤ 30.0
Deployment Value	6.5	6.4	84.8	83.5

Device-specific metrics (computed for each sensor in evaluation)
○○○ Metric value for none of devices tested falls within the target range
●○○ Metric value for one of devices tested falls within the target range
●●○ Metric value for two of devices tested falls within the target range
●●● Metric value for three of devices tested falls within the target range

Single-valued metrics (computed via entire evaluation dataset)
☆ Indicates that the metric value is not within the target range
★ Indicates that the metric value is within the target range

Sensor-Sensor Precision

	Precision (between collocated sensors)				Data Quality	
	CV (%)		SD (µg/m³)		Number of concurrent sensor concentration pairs	
	1-Hour ☆	24-Hour ☆	1-Hour ★	24-Hour ★	1-Hour	24-Hour
Metric Target Range	≤ 30.0	≤ 30.0	≤ 5.0	≤ 5.0	-	-
Deployment Value	80.3	69.4	1.2	1.1	693	27

*This value is only a recommendation for ensuring data quality and is not included in the list of target values discussed in Section 4 of the Performance Testing Protocols, Metrics, and Target Values for Fine Particulate Matter Air Sensors document.

Testing Report - $PM_{2.5}$ Base Testing

SENSIT RAMP

This report reflects out-of-the-box performance

Initial Base Testing - RTP, NC
U.S. Environmental Protection Agency
Office of Research and Development
PI: Clements.Andrea@epa.gov
919-541-1363
October 2019—November 2019

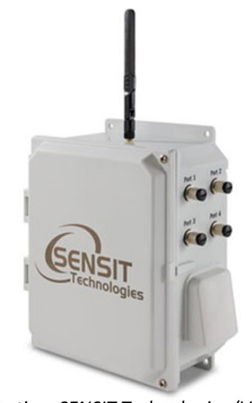

Image Attribution: SENSIT Technologies (Valparaiso, IN)

Sensor-FRM/FEM Scatter Plots

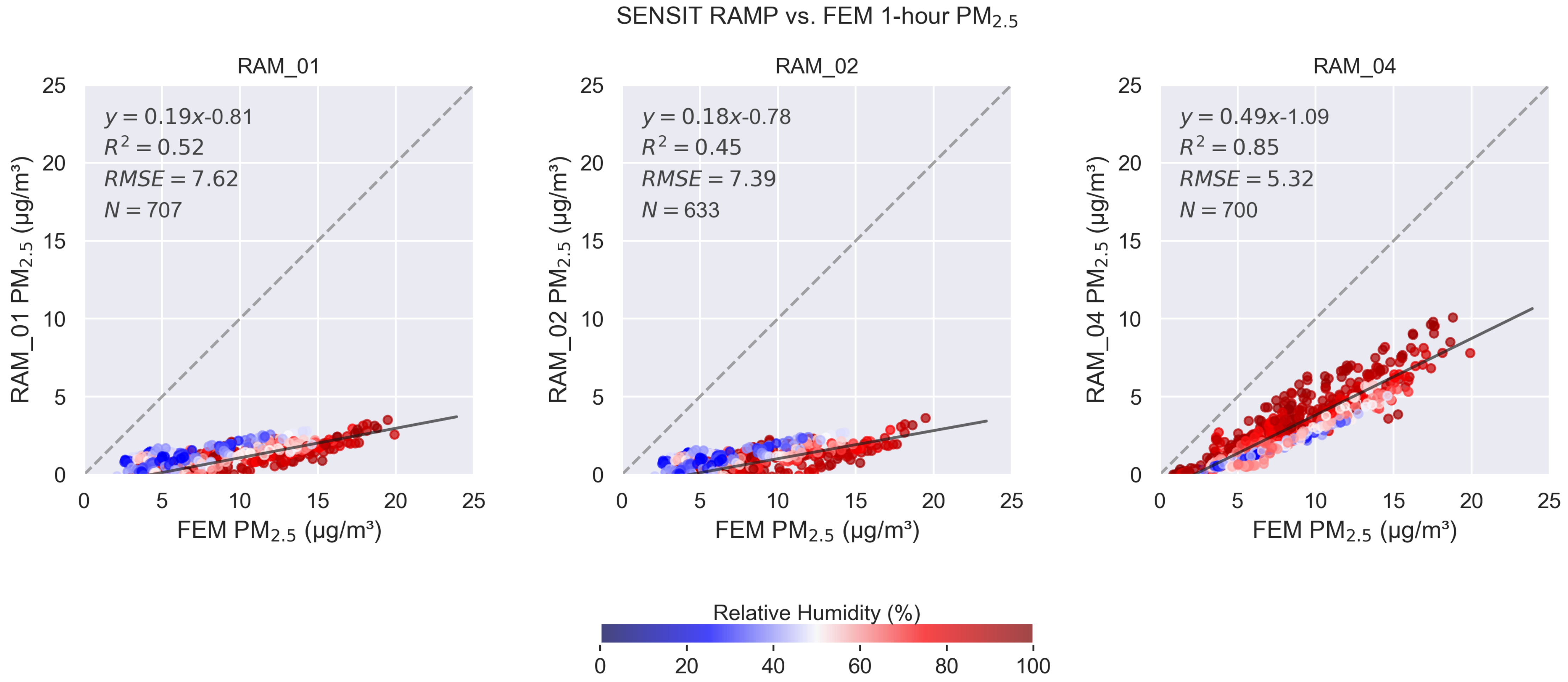

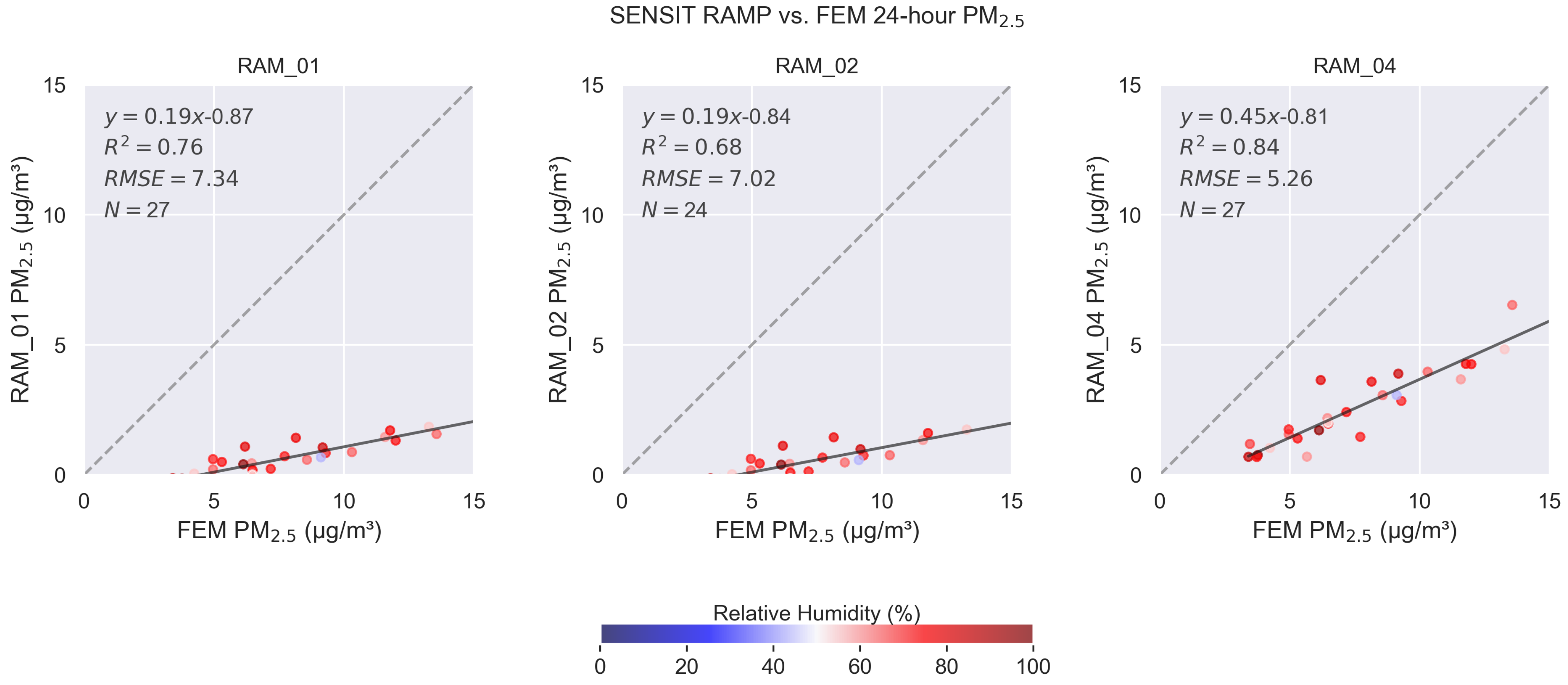

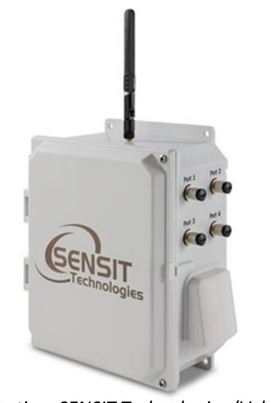

Image Attribution: SENSIT Technologies (Valparaiso, IN)

Supplemental Information

Abbreviations used in Supplemental Information

FRM	Federal Reference Method
FEM	Federal Equivalent Method
SOP	Standard Operating Procedure
QAPP	Quality Assurance Project Plan
QC	Quality Control

Supplemental Documentation	Attached	Description & URL or file path to documentation
Field observations and sensor data flags	☑	See NC-RAM-Page 6 of this testing report
Maintenance logs	☐	No logs recorded during testing
Standard operating procedure(s)	☐	U.S. EPA Office Of Research and Development SOP available upon request
Photos of equipment setup and testing	☑	See NC-RAM-Page 5 of this testing report
Product specifications sheet(s)	☑	SENSIT Technologies: RAMP Product Brochure
Product manual(s)	☑	SENSIT Technologies: RAMP Product Manual
Data storage and transmission method	☑	See NC-RAM-Page 6 of this testing report
Data correction approach	☑	See NC-RAM-Page 6 of this testing report
Issues encountered	☑	See NC-RAM-Page 6 of this testing report
Data analysis/correction scripts and version	☑	Averaging and processing of data, calculation of performance metrics, and generation of figures and other supplementary material for analysis were obtained using Python 3.9.7 with the packages sensortoolkit v0.8.3b2, pandas 1.3.5, NumPy 1.21.2, Matplotlib 3.5.0, statsmodels 0.13.0, and seaborn 0.11.2. All packages are available from the Python Package Index (PyPI) at https://pypi.org. The integrated development environment (IDE) Spyder 5.1.5 was used for scripting and data visualization. Version control for the Python base, packages, and IDE were all managed by conda 4.11.0.
Air Monitoring Station QAPP	☐	U.S. EPA Office Of Research and Development QAPP available upon request
Summary of FRM/FEM monitor QC checks	☑	See NC-RAM-Page 7, 8 of this testing report
Manufacturer website for FRM/FEM monitor	☑	Teledyne API: T640/T640X Product website
FRM/FEM monitor manual	☑	Teledyne API: T640/T640X Product Manual
FRM/FEM monitor specifications sheet(s)	☑	Teledyne API: T640/T640X Specification Sheet
Other documents	☐	

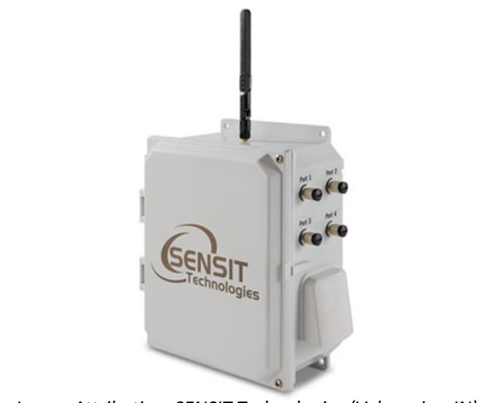

Image Attribution: SENSIT Technologies (Valparaiso, IN)

Supplemental Information: Photos of Testing Site and Equipment Setup

Site Description:
The Burdens Creek Ambient Monitoring Innovation Research Station (AIRS) site is located on the U.S. EPA, RTP campus and is situated between Alexander Drive and Route 147. The site is intended to represent a neighborhood-scale site as defined in *40 CFR Part 58, Appendix D*. U.S. EPA's Office of Air Quality Planning and Standards (OAQPS) operates reference grade instruments in a free-standing shelter situated directly adjacent to the sensor testing platform.

Figure 1: SENSIT RAMP sensors (indicated by red arrow) attached to mounting assembly at evaluation site.

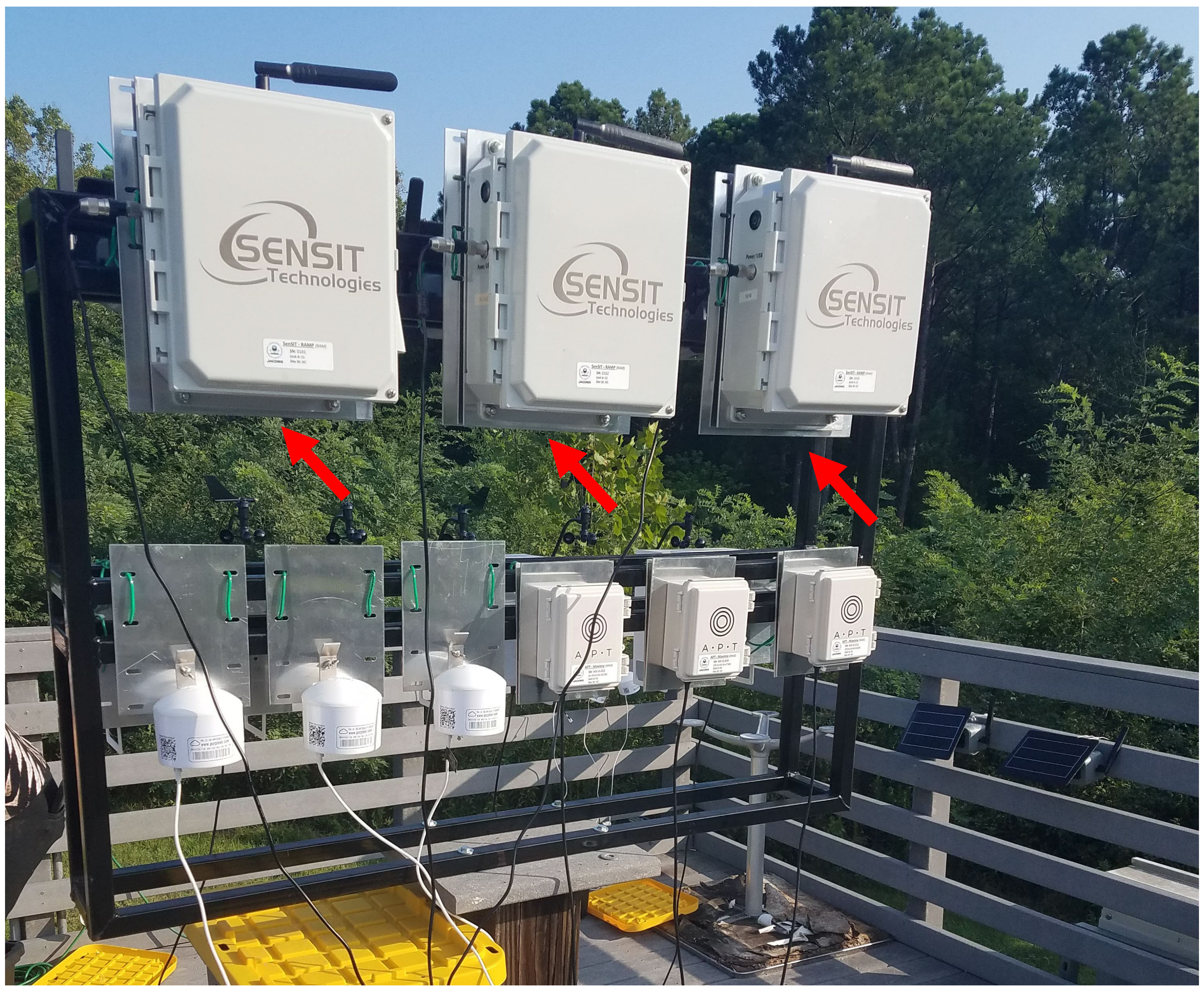

Figure 2: Observation deck at evaluation site. Approximate location of sensors indicated by the red arrow, the approximate location of T640x FEM (housed within sampling trailer) indicated by the black arrow. Sensors and FEM instrumentation are separated by approximately 15 meters.

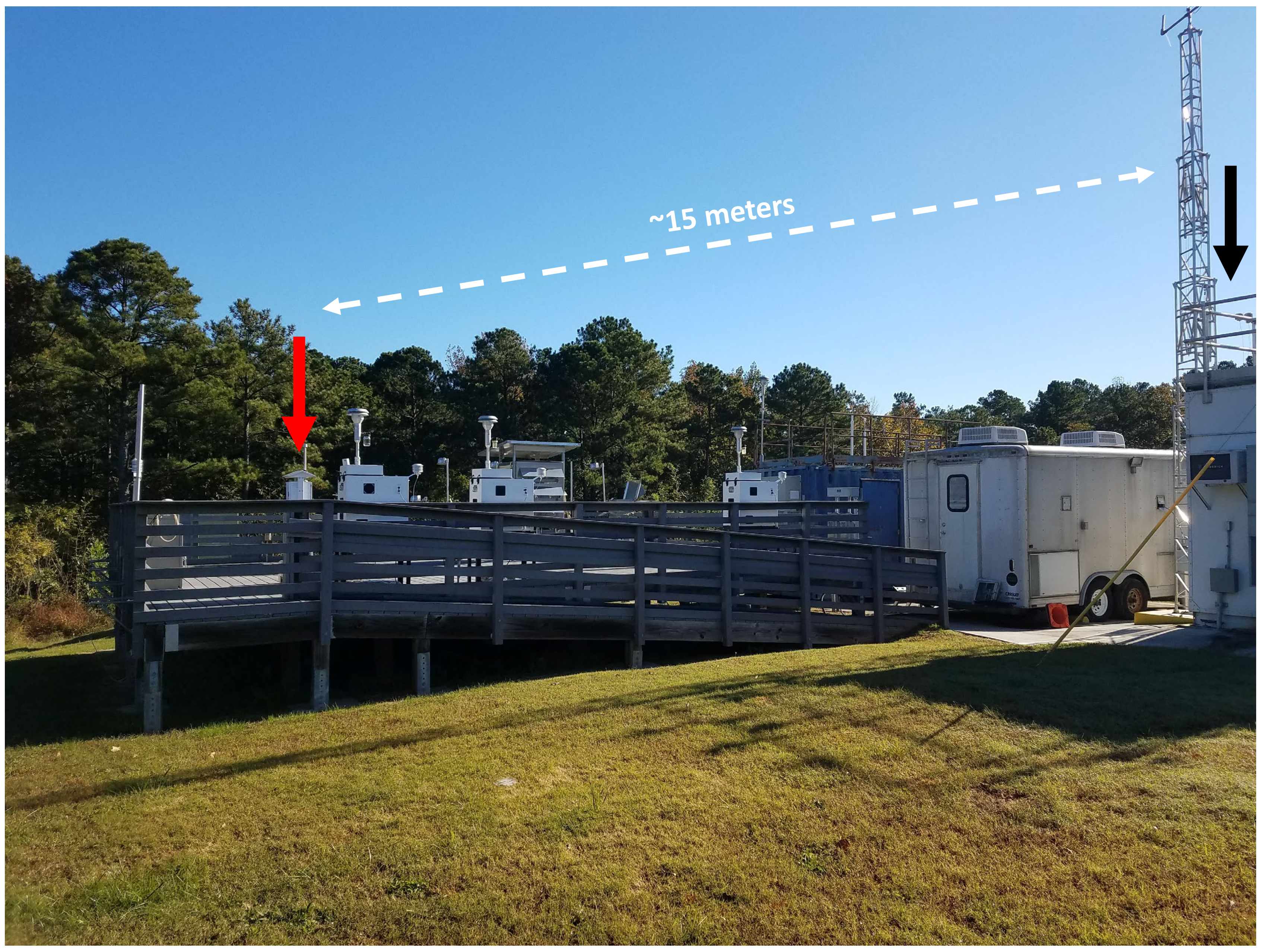

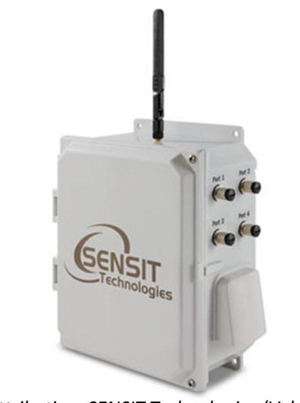

Image Attribution: SENSIT Technologies (Valparaiso, IN)

Supplemental Information: Data Storage, Correction Approach, and Issues Encountered

Data Storage and Transmission Method

The SENSIT RAMP was configured to record data at a 15-second sampling interval. Data are stored as daily text files (.txt format) on an onboard MicroSD card. Data files were obtained weekly via SD cards. Each field site operator was provided two labeled MicroSD cards for sensor units that they used to swap out each week. Data from the collected card was then read and processed off-site.

Data Correction Approach

SENSIT RAMP units were pre-configured by the manufacturer with a linear correction (i.e., concentration gain and offset). These presets (table below) reflect out-of-the box performance and were not modified by EPA prior or during testing.

Sensor ID	Preset Gain	Preset Offset (μg m^{-3})
RAM_01	1.0	0
RAM_02	1.0	0
RAM_04	1.0	0

After acquisition, the raw data was processed using the *sensortoolkit* python code library (v0.8.3b2). A continuous data set at the recorded sampling frequency was written to a .csv file. 1-hour and 24-hour averaged data sets were generated using a 75% completeness threshold and saved as separate .csv files. Outliers were NOT removed from data sets in order to assess “out-of-the-box” sensor performance.

Issues Encountered

<u>Pre-deployment observations</u>

- SENSIT RAMP units were received without documentation or manuals. After communicating the need to change default settings (logging interval and time zone) with the manufacturer, a draft user’s manual and a USB cable were supplied. With the use of this USB cable, instrument settings could be changed, and real-time data could be logged using a serial communication software (CoolTerm, v.1.5.0). Because the sensor did not record data at the top of every minute, the RAMP was configured to record data at 15-second intervals so that the data could be averaged more closely to complete minutes. Prior to deployment, RAMP units were collocated in a bench-top evaluation to verify operational status and determine the extent of data invalidity (i.e., determine equilibration period) after an initial start-up event. The recorded response for parameters measured by the RAMP suggests that the gas sensors (CO, NO, NO_2, O_3) required approximately a 2-hour equilibration period, while the remaining sensors (temperature, relative humidity, particulate matter) did not require any equilibration period.

The following table contains data flags describing events that were encountered during the testing period. Unit RAM_02 was retrieved slightly prior to the end of the testing period. RAM_02 was sent to the La Casa, CO monitoring site to replace a broken RAMP unit.

Start Time (UTC)	End Time (UTC)	Sensor Serial ID	Parameters Impacted	Flag
2019-11-14 13:01:00+00:00	2019-11-14 13:01:00+00:00	RAM_02	ALL	0.0-Sensor retrieval

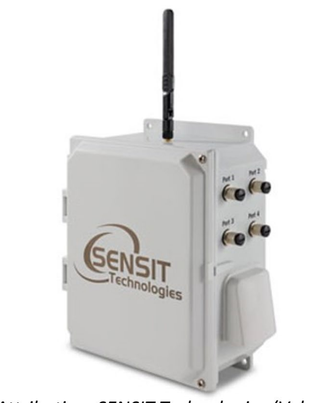

Image Attribution: SENSIT Technologies (Valparaiso, IN)

Description of Data Flags

OAQPS manages data logged by reference monitors at the AIRS evaluation site using the Envidas data acquisition system software from DR DAS LTD[1]. Envidas contains over 100 data flags which are configurable by the monitoring agency and can be triggered based on instrument status. Appendix B Table 1 contains a description of Envidas data flags including the numeric code and status code name (a brief textual description, the table containing data flags below includes code names for events encountered by the reference monitor under the "Flag" column). Appendix B Table 1 also indicates whether reference data are invalidated for a given data flag. For this report, reference data were invalidated for periods where a data flag was logged and if the corresponding entry for the data flag in the "Data Status Invalidation" column in Appendix B Table 1 indicates data should be invalidated.

Data Flags Recorded During Testing

FRM/FEM Monitor	Timestamp (UTC)	Flag
Teledyne API T640X (Data acquired via local transfer from OAQPS)	2019-10-19 03:44:00+0000	<Samp
	2019-10-28 18:03:00+0000	<Samp
	2019-10-28 20:11:00+0000 to 2019-10-28 21:04:00+0000	Down
	2019-10-28 21:05:00+0000	<Samp
	2019-11-07 22:05:00+0000	<Samp
	2019-11-08 15:52:00+0000	<Samp
	2019-11-08 15:59:00+0000	<Samp
	2019-11-08 16:09:00+0000	<Samp
	2019-11-12 18:31:00+0000 to 2019-11-12 18:32:00+0000	NoData [maintenance]
	2019-11-12 18:33:00+0000	<Samp
	2019-11-12 18:36:00+0000	<Samp
	2019-11-12 19:09:00+0000 to 2019-11-12 19:10:00+0000	<Samp
	2019-11-12 19:12:00+0000	<Samp
	2019-11-12 19:24:00+0000	<Samp
	2019-11-12 19:28:00+0000	<Samp
	2019-11-12 19:30:00+0000	NoData [maintenance]
	2019-11-12 19:31:00+0000	<Samp
	2019-11-12 19:33:00+0000	<Samp
	2019-11-12 19:34:00+0000	NoData [maintenance]
	2019-11-12 19:36:00+0000	<Samp
	2019-11-12 19:38:00+0000	<Samp
	2019-11-13 17:22:00+0000	NoData [maintenance]
	2019-11-13 17:26:00+0000	<Samp
	2019-11-13 17:29:00+0000	<Samp
	2019-11-13 17:31:00+0000	<Samp
	2019-11-13 17:36:00+0000	NoData [maintenance]
	2019-11-13 17:39:00+0000	NoData [maintenance]
	2019-11-13 17:50:00+0000	<Samp
	2019-11-13 17:55:00+0000 to 2019-11-13 17:56:00+0000	NoData [maintenance]
	2019-11-13 17:57:00+0000	<Samp
	2019-11-14 09:55:00+0000	<Samp
	2019-11-14 14:37:00+0000	NoData [maintenance]
	2019-11-15 07:02:00+0000 to 2019-11-15 07:03:00+0000	NoData [maintenance]
	2019-11-15 07:04:00+0000	<Samp

[1]"Contact us" DR DAS LTD, 2022, https://dr-das.com/Home/Contact.html Accessed June 3 2022

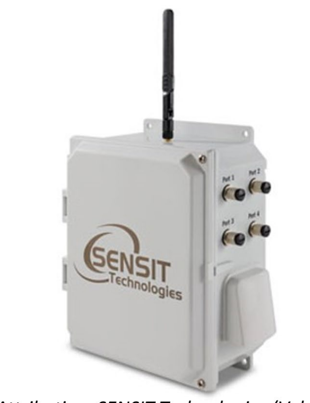

Image Attribution: SENSIT Technologies (Valparaiso, IN)

Data Flags Recorded During Testing (Cont.)

FRM/FEM Monitor	Timestamp (UTC)	Flag
RM Young 41382 VC Temperature and Relative Humidity Monitor (Data acquired via local transfer from OAQPS)	2019-11-07 22:05:00+0000	<Samp
	2019-11-08 15:52:00+0000	<Samp
	2019-11-08 15:59:00+0000	<Samp
	2019-11-08 16:09:00+0000	<Samp
	2019-11-12 18:31:00+0000 to 2019-11-12 18:32:00+0000	NoData [maintenance]
	2019-11-12 18:33:00+0000	<Samp
	2019-11-12 18:36:00+0000	<Samp
	2019-11-12 19:09:00+0000 to 2019-11-12 19:10:00+0000	<Samp
	2019-11-12 19:12:00+0000	<Samp
	2019-11-12 19:24:00+0000	<Samp
	2019-11-12 19:28:00+0000	<Samp
	2019-11-12 19:31:00+0000	<Samp
	2019-11-12 19:33:00+0000	<Samp
	2019-11-12 19:34:00+0000	NoData [maintenance]
	2019-11-12 19:36:00+0000	<Samp
	2019-11-12 19:38:00+0000	<Samp
	2019-11-13 17:22:00+0000	NoData [maintenance]
	2019-11-13 17:26:00+0000	<Samp
	2019-11-13 17:29:00+0000	<Samp
	2019-11-13 17:31:00+0000	<Samp
	2019-11-13 17:36:00+0000	NoData [maintenance]
	2019-11-13 17:39:00+0000	NoData [maintenance]
	2019-11-13 17:50:00+0000	<Samp
	2019-11-13 17:55:00+0000 to 2019-11-13 17:56:00+0000	NoData [maintenance]
	2019-11-13 17:57:00+0000	<Samp
	2019-11-14 14:37:00+0000	NoData [maintenance]
	2019-11-15 07:02:00+0000 to 2019-11-15 07:03:00+0000	NoData [maintenance]
	2019-11-15 07:04:00+0000	<Samp

Testing Report - $PM_{2.5}$ Base Testing

Aeroqual AQY

This report reflects out-of-the-box performance

Initial Base Testing - Edmond, OK

U.S. Environmental Protection Agency
Office of Research and Development
PI: Clements.Andrea@epa.gov
919-541-1363

August 2019—September 2019

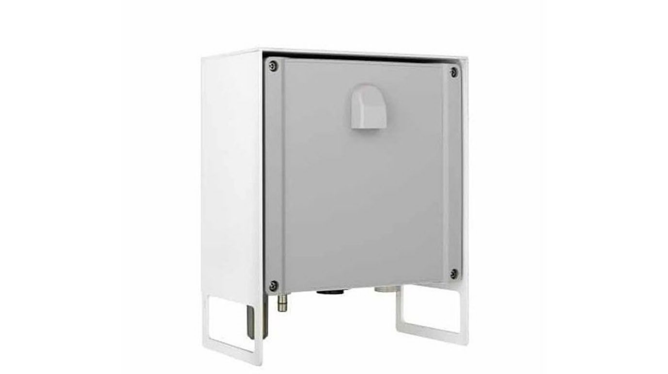

Image Attribution: Aeroqual Limited (Auckland, New Zealand)

Deployment Details

Testing Organization and Site Information	
Testing organization (Name, Organization type, Contact website)	U.S. Environmental Protection Agency - Office of Research and Development<br>Federal Government<br>Air Sensor Toolbox | U.S. EPA Website
Testing location (City, State, Latitude and Longitude)	OK Christian University<br>Edmond, OK<br>35.614131, -97.475083
AQS site ID	40 - 109 - 1037
Sampling timeframe (MM-DD-YY)	08-02-19 to 09-01-19
Sensor data source	Aeroqual Cloud download
Reference data source	AQS API download

Sensor Information			
Manufacturer, model	Aeroqual AQY		
Device firmware version	1.14.2		
Sampling time interval	1-minute		
Sensor serial numbers	AQY_01		
Issues encountered during deployment?	☐	No Issues	

FRM/FEM Information	
Manufacturer, model, designation	Teledyne Advanced Pollution Instrumentation T640 FEM
Sampling time interval	1-hour averaging
Date of calibration	As required by 40 CFR Part 58 and the QAPP for Ambient Air Monitoring in the State of Oklahoma maintained by ODEQ/AQD
Date of flowrate verification check	Monthly as required by 40 CFR Part 58 Appendix A
Description, date(s) of maintenance activities	N/A

Time Series Plots

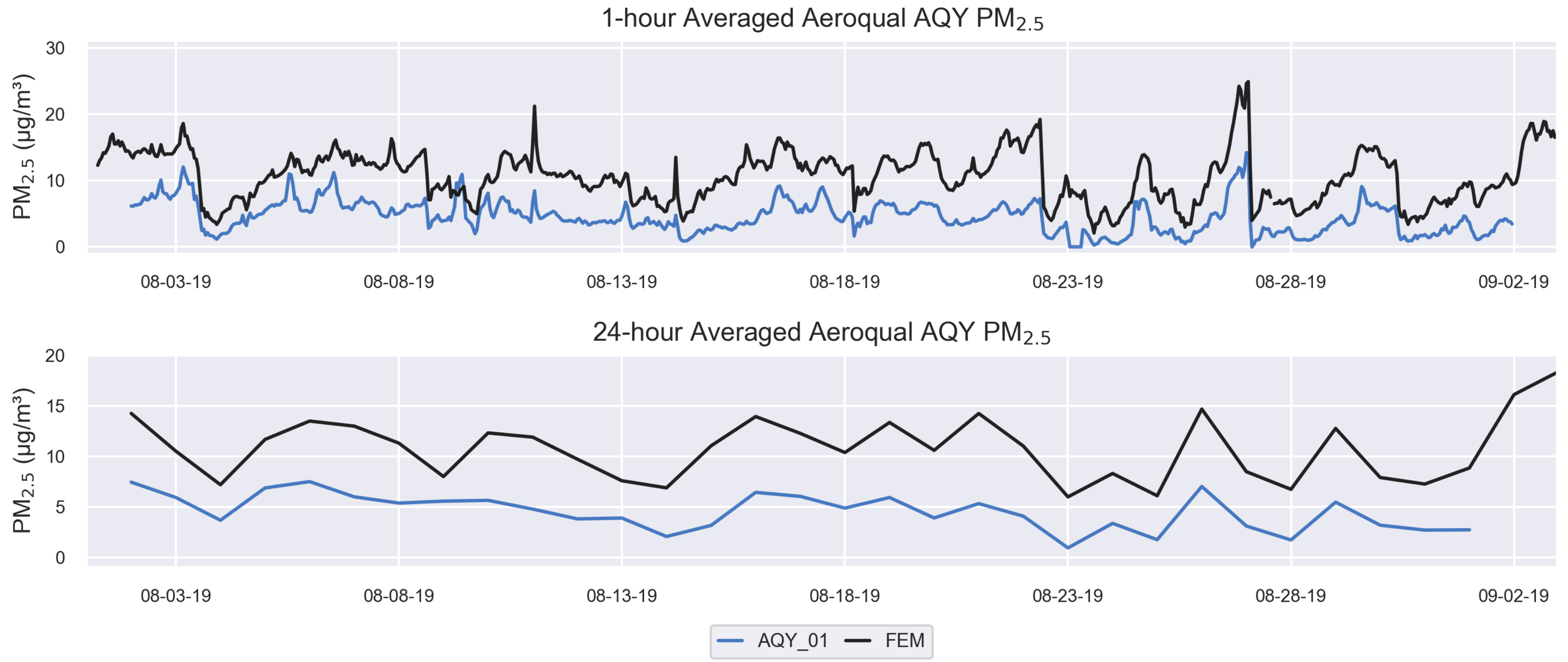

Scatter Plots: Comparison to FRM/FEM

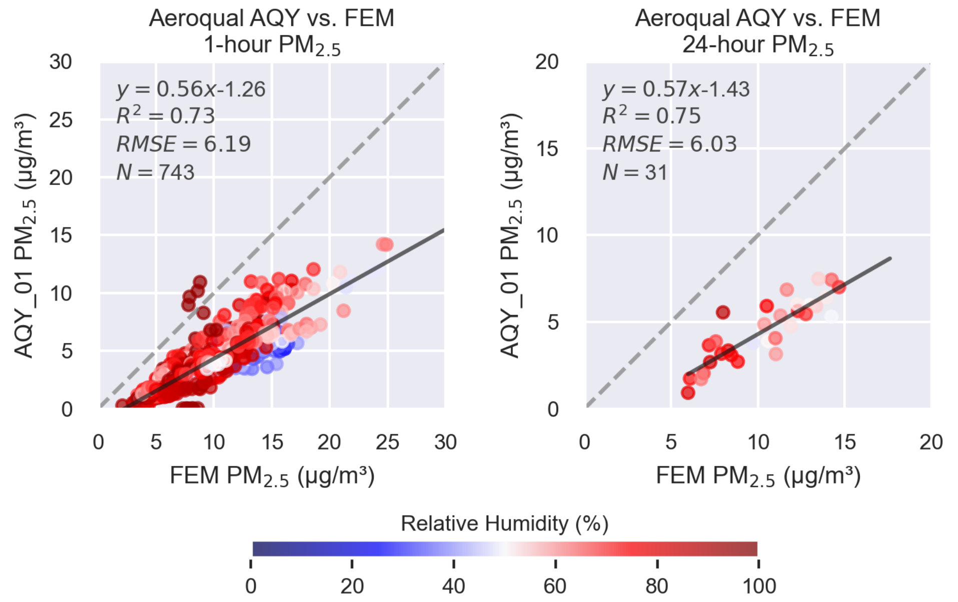

Range of FRM/FEM concentrations over duration of base test (µg/m³)	2.1-24.9 (1-hr), 6.0-14.7 (24-hr)
Number of 24-hr periods in FRM/FEM monitor measurements with a goal concentration ≥ 25 µg/m³	0

Performance Metrics

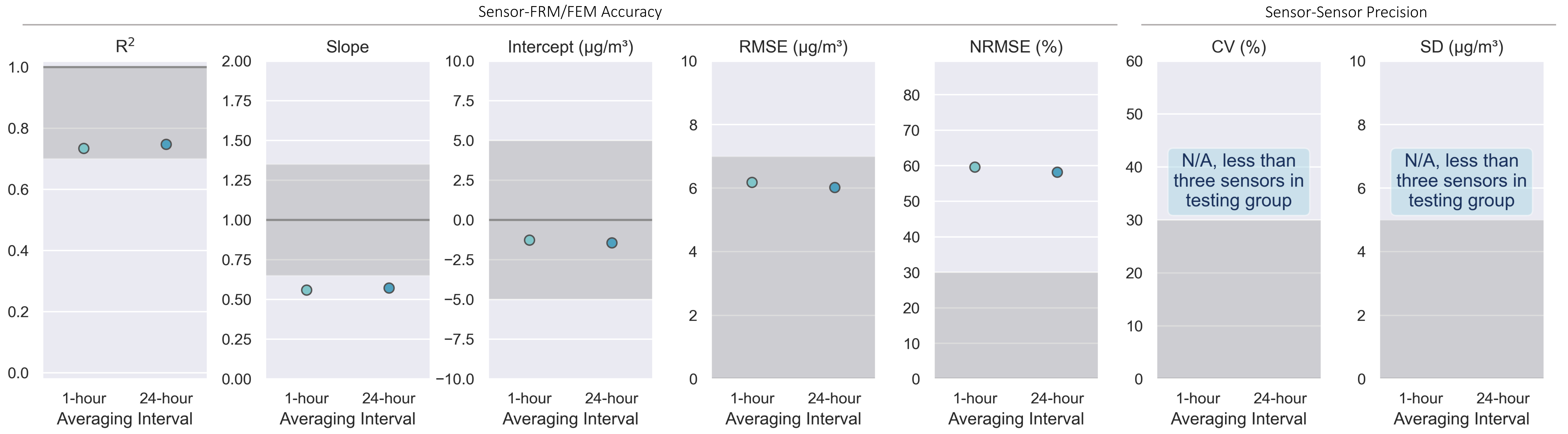

Meteorological Conditions During Deployment

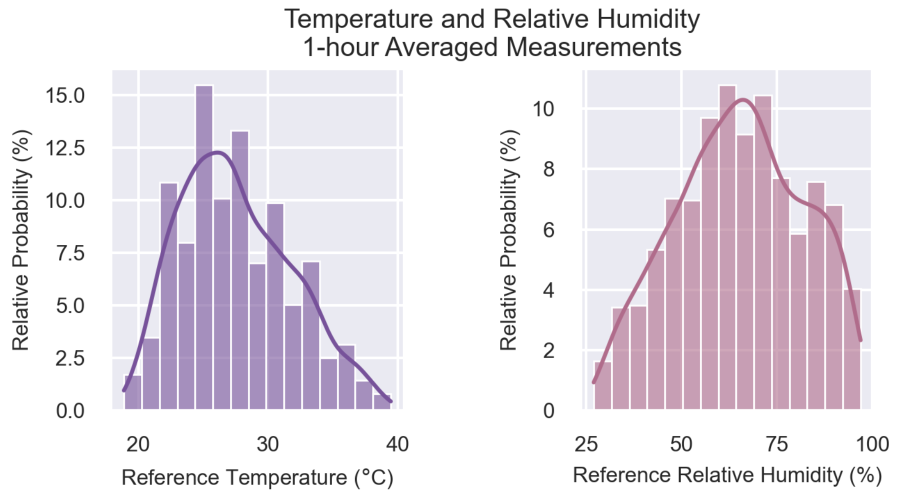

Number of 24-hr periods outside sensor manufacturer-listed temperature operational range (-10 to 40 °C)	0
Number of 24-hr periods outside sensor manufacturer-listed relative humidity operational range (no operational range specified)	-

Meteorological Influence

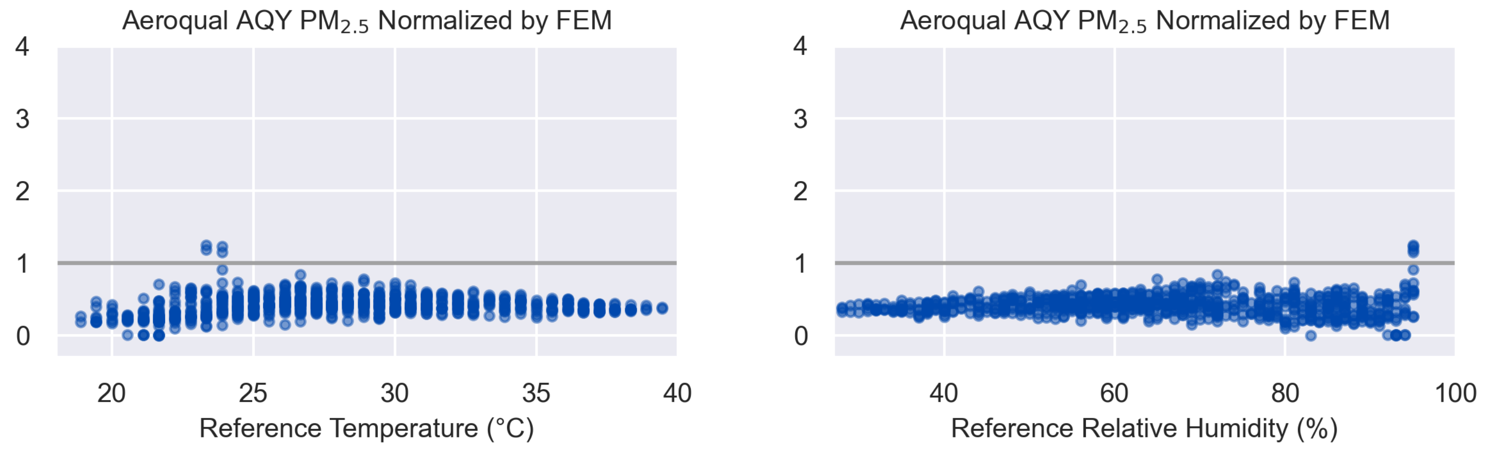

Mean number of paired, normalized concentration and temperature values (1-hr averages)	744
Mean number of paired, normalized concentration and relative humidity values (1-hr averages)	744

Testing Report - $PM_{2.5}$ Base Testing

Aeroqual AQY

This report reflects out-of-the-box performance

Initial Base Testing - Edmond, OK

U.S. Environmental Protection Agency
Office of Research and Development
PI: Clements.Andrea@epa.gov
919-541-1363

August 2019—September 2019

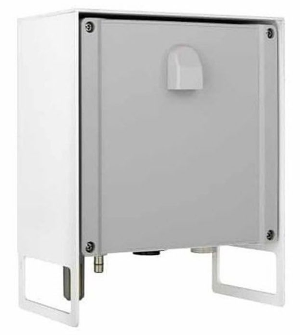

Image Attribution: Aeroqual Limited (Auckland, New Zealand)

Tabular Statistics

Sensor-FRM/FEM Correlation

	Bias and Linearity						Data Quality			
	R^2		Slope		Intercept (µg/m³)		Uptime (%)		Number of paired sensor and FRM/FEM concentration values	
	1-Hour ●	24-Hour ●	1-Hour ○	24-Hour ○	1-Hour ●	24-Hour ●	1-Hour ●	24-Hour ●	1-Hour	24-Hour
Metric Target Range	≥ 0.70	≥ 0.70	1.0 ± 0.35	1.0 ± 0.35	-5 ≤ b ≤ 5	-5 ≤ b ≤ 5	75%*	75%*	-	-
Sensor AQY_01	0.73	0.75	0.56	0.57	-1.26	-1.43	100	100	743	31

	Error			
	RMSE (µg/m³)		NRMSE (%)	
	1-Hour ★	24-Hour ★	1-Hour ☆	24-Hour ☆
Metric Target Range	≤ 7.0	≤ 7.0	≤ 30.0	≤ 30.0
Deployment Value	6.2	6.0	59.7	58.2

Device-specific metrics (computed for each sensor in evaluation)
○○○ Metric value for none of devices tested falls within the target range
●○○ Metric value for one of devices tested falls within the target range
●●○ Metric value for two of devices tested falls within the target range
●●● Metric value for three of devices tested falls within the target range

Single-valued metrics (computed via entire evaluation dataset)
☆ Indicates that the metric value is not within the target range
★ Indicates that the metric value is within the target range

Sensor-Sensor Precision[1]

	Precision (between collocated sensors)				Data Quality	
	CV (%)		SD (µg/m³)		Number of concurrent sensor concentration pairs	
	1-Hour ☆	24-Hour ☆	1-Hour ☆	24-Hour ☆	1-Hour	24-Hour
Metric Target Range	≤ 30.0	≤ 30.0	≤ 5.0	≤ 5.0	-	-
Deployment Value	-	-	-	-	-	-

[1]Precision statistics are computed for evaluations with at least three collocated sensor units. Metric values are left blank for evaluations with two or fewer sensor units.

*This value is only a recommendation for ensuring data quality and is not included in the list of target values discussed in Section 4 of the Performance Testing Protocols, Metrics, and Target Values for Fine Particulate Matter Air Sensors document.

Testing Report - $PM_{2.5}$ Base Testing

Aeroqual AQY

This report reflects out-of-the-box performance

Initial Base Testing - Edmond, OK
U.S. Environmental Protection Agency
Office of Research and Development
PI: Clements.Andrea@epa.gov
919-541-1363
August 2019—September 2019

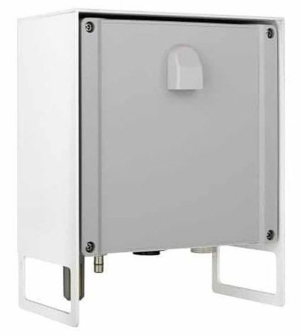

Image Attribution: Aeroqual Limited (Auckland, New Zealand)

Sensor-FRM/FEM Scatter Plots

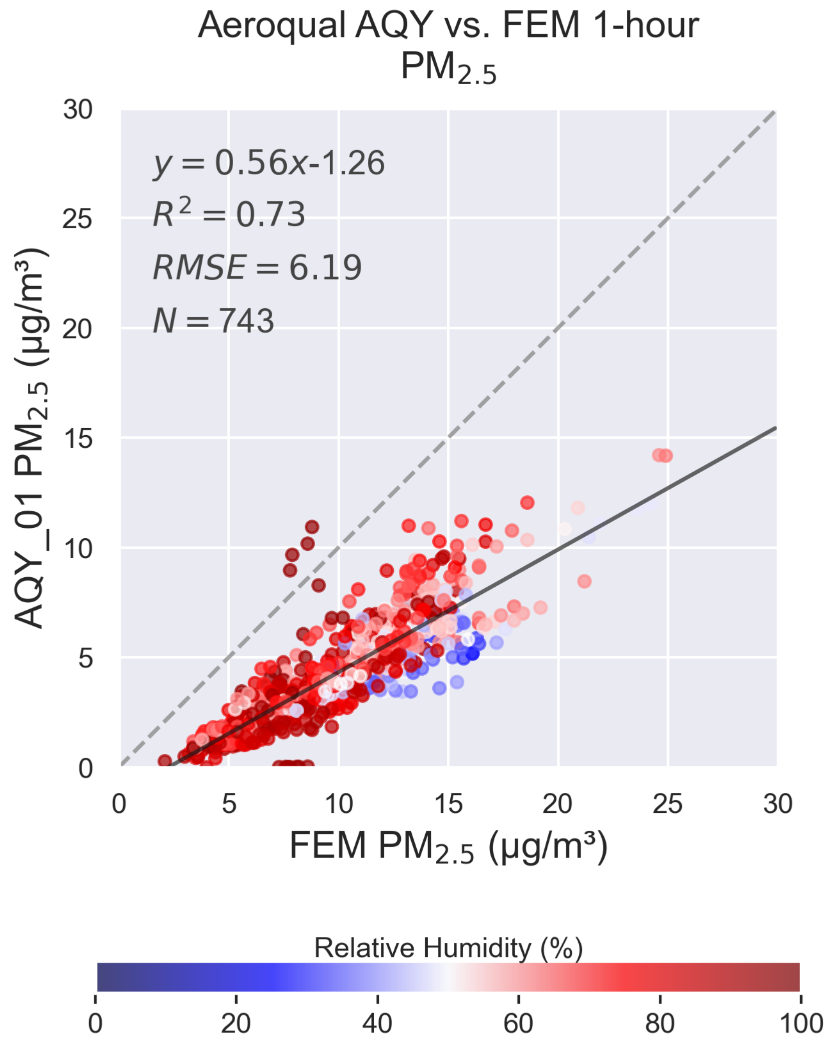

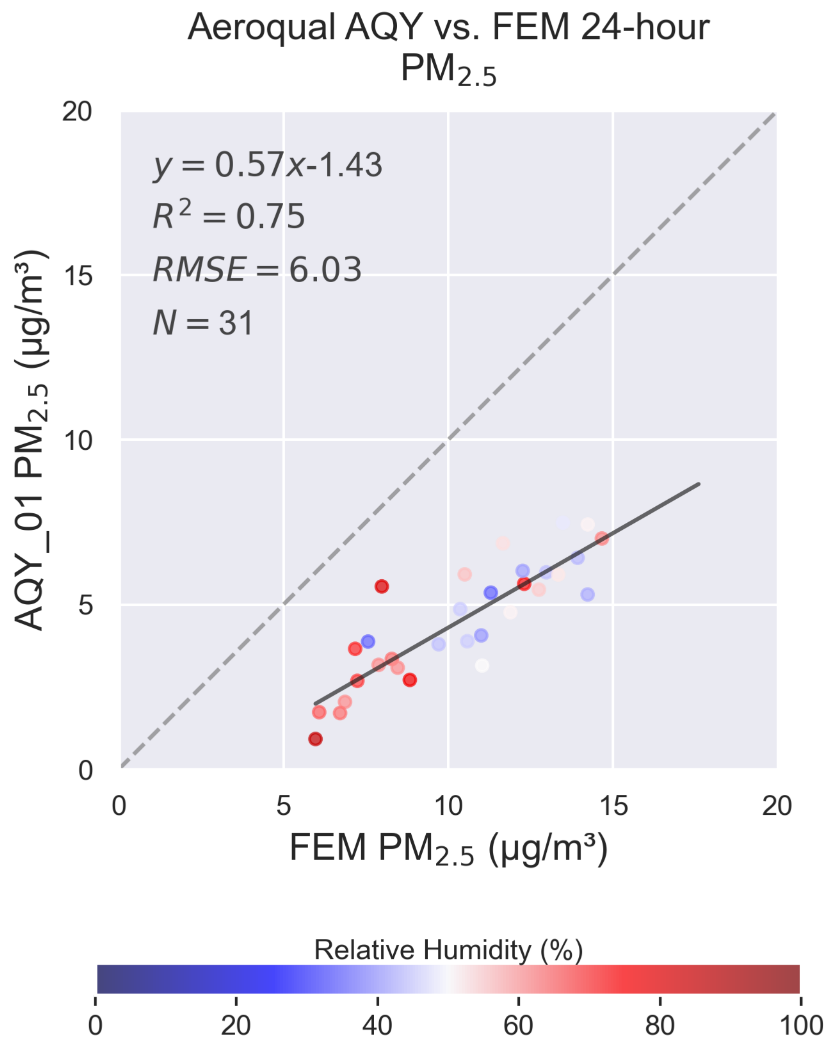

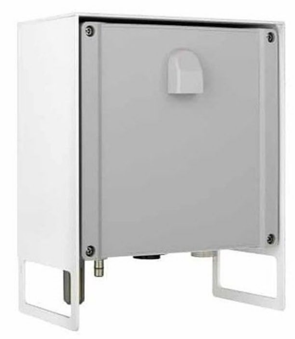

Image Attribution: Aeroqual Limited (Auckland, New Zealand)

Supplemental Information

Abbreviations used in Supplemental Information

FRM	Federal Reference Method
FEM	Federal Equivalent Method
SOP	Standard Operating Procedure
QAPP	Quality Assurance Project Plan
QC	Quality Control

Supplemental Documentation	Attached	Description & URL or file path to documentation
Field observations and sensor data flags	☑	See OK-AQY-Page 6 of this testing report
Maintenance logs	☐	No logs recorded during testing
Standard operating procedure(s)	☐	U.S. EPA Office Of Research and Development SOP available upon request
Photos of equipment setup and testing	☑	See OK-AQY-Page 5 of this testing report
Product specifications sheet(s)	☐	N/A*
Product manual(s)	☐	N/A*
Deployment issues	☐	No issues reported
Data storage and transmission method	☑	See OK-AQY-Page 6 of this testing report
Data correction approach	☑	See OK-AQY-Page 6 of this testing report
Data analysis/correction scripts and version	☑	Averaging and processing of data, calculation of performance metrics, and generation of figures and other supplementary material for analysis were obtained using Python 3.9.7 with the packages sensortoolkit v0.8.3b2, pandas 1.3.5, NumPy 1.21.2, Matplotlib 3.5.0, statsmodels 0.13.0, and seaborn 0.11.2. All packages are available from the Python Package Index (PyPI) at https://pypi.org. The integrated development environment (IDE) Spyder 5.1.5 was used for scripting and data visualization. Version control for the Python base, packages, and IDE were all managed by conda 4.11.0.
Air Monitoring Station QAPP	☐	U.S. EPA Office Of Research and Development QAPP available upon request
Summary of FRM/FEM monitor QC checks	☑	See OK-AQY-Page 7 of this testing report
Manufacturer website for FRM/FEM monitor	☑	Teledyne API: T640/T640X Product website
FRM/FEM monitor manual	☑	Teledyne API: T640/T640X Product Manual
FRM/FEM monitor specifications sheet(s)	☑	Teledyne API: T640/T640X Specification Sheet
Other documents	☑	Manufacturer notice of AQY sales on hold

*As of 3/18/2021, the manufacturer of the AQY has placed sales of a similar unit on hold. Documentation for the AQY is currently unavailable from the manufacturer's website.

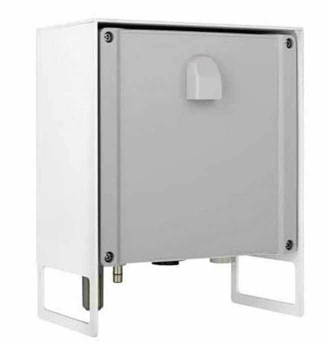

Image Attribution: Aeroqual Limited (Auckland, New Zealand)

Supplemental Information: Photos of Testing Site and Equipment Setup

Site Description:

The Oklahoma Christian University monitoring site was established in the late 1970s. There is a containerized building that houses the continuous instruments with a wooden platform directly adjacent for the filter-based instruments. Pollutants monitored include O_3, PM_{10} (filter based and continuous), $PM_{2.5}$ (filter based and continuous), $NO/NO_2/NO_x$, CO, SO_2 and meteorology. The site has solar panels to help defer the cost of power to the site.

Figure 1: Aeroqual AQY (indicated by red arrow) attached to metal railing atop the sampling shelter at the monitoring site.

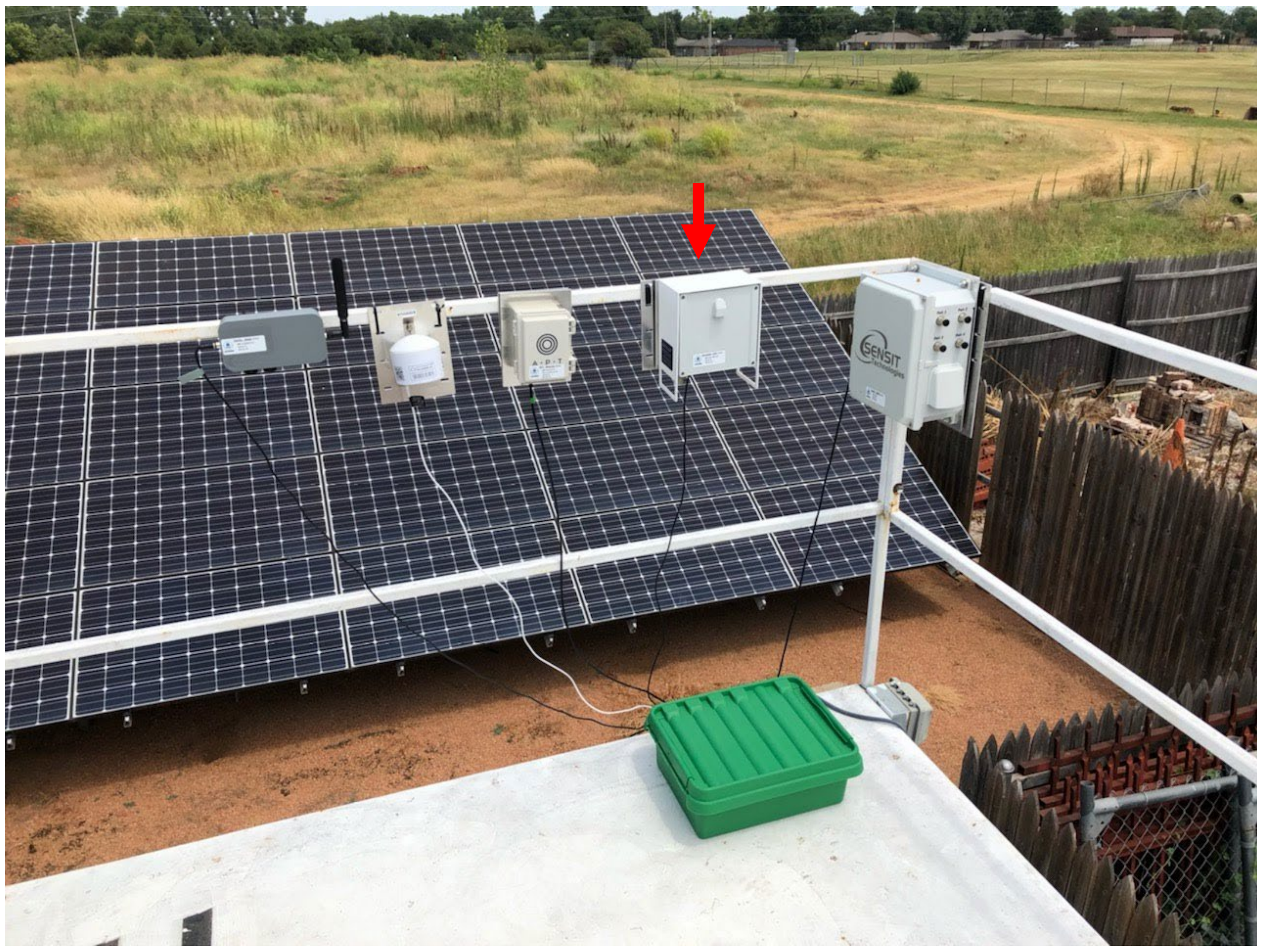

Figure 2: OK Monitoring Station sampling shelter

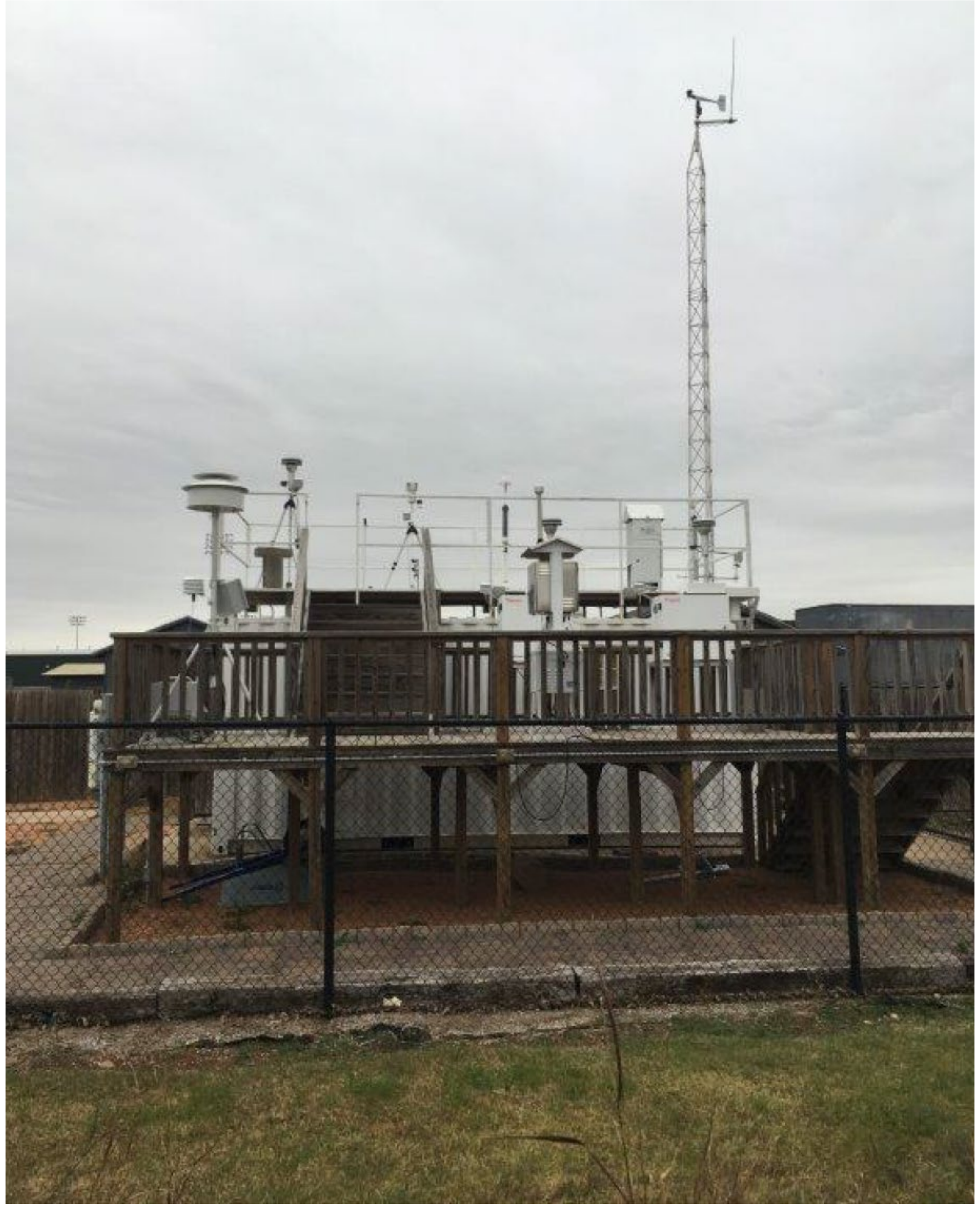

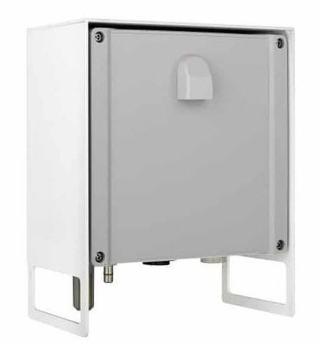

Image Attribution: Aeroqual Limited (Auckland, New Zealand)

Supplemental Information: Data Storage, Correction Approach, and Issues Encountered

Data Storage and Transmission Method

As part of CRADA #934-16 between Aeroqual and US EPA, Aeroqual supported data streaming. SIM cards were installed and data flowed to the Aeroqual Cloud. The 1-minute raw data was acquired weekly using the Aeroqual Cloud (*last accessed 5/11/22*) user interface (UI). The AQY has an internal data storage USB flash drive as a data backup, however the software on the drive is proprietary to Aeroqual and unreadable by outside systems.

Data Correction Approach

This evaluation report reflects “out-of-the-box” performance of the AQY. The manufacturer provides a procedure by which local collocation (sensor operated along side an FRM/FEM) data can be collected, a gain (slope) and offset (intercept) determined, and parameters entered into the Aeroqual Cloud user interface to be applied to all subsequently collected data. This procedure and feature was NOT used prior to this evaluation. Prospective consumers may get different performance from this device if they utilize this feature.

After acquisition, the raw data was processed using the *sensortoolkit* python code library (v0.8.3b2). A continuous data set at the recorded sampling frequency was written to a .csv file. 1-hour and 24-hour averaged data sets were generated using a 75% completeness threshold and saved as separate .csv files. Outliers were NOT removed from data sets in order to assess “out-of-the-box” sensor performance.

Issues Encountered

Pre-deployment observations

- *Timestamp inaccuracies*: During pre-deployment, the AQY devices did not properly sync timestamps with the onboard Real-Time Clock. Connecting the units to the internet by cellular or Wi-Fi allowed the unit to sync with internet time and resulted in proper timestamps.

Field observations and sensor data flags

The Aeroqual AQY was deployed at the OK monitoring site on 8/1/2019. The AQY unit operated nominally during the testing period and did not require replacement or repair.

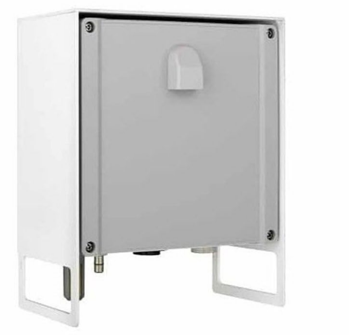

Description of Data Flags

AQS

The U.S. EPA's Air Quality System (AQS) is the Agency's primary ambient air monitoring data archive. A comprehensive list of data flags that are recorded alongside AQS data sets, referred to by U.S. EPA as 'qualifiers', can be found at the following link: https://aqs.epa.gov/aqsweb/documents/codetables/qualifiers.html

Invalidation of reference data: AQS qualifiers are organized by qualifier type, which indicates whether data logged alongside qualifier flags should be invalidated (set null). Qualifiers with type "Null Data Qualifier" are invalidated, and includes data logged during periods that coincide with QC checks (e.g., "BF-Precision/Zero/Span", "BJ- Operator Error", "BL - QA Audit", "AZ - QC Audit") among other events such as power outages. Data logged alongside qualifiers with type "Quality Assurance Qualifiers" are not invalidated and are included in this analysis (e.g., concentrations less than the federal MDL for the reference monitor "MD – Value less than MDL", QA reviewed values "Validated Value").

Data Flags Recorded During Testing

FRM/FEM Monitor	Timestamp (UTC)	Flag
Teledyne API T640 (Acquired via AQS)	2019-08-27 14:00:00+0000	AZ - QC Audit

Testing Report - $PM_{2.5}$ Base Testing

APT Maxima

This report reflects out-of-the-box performance

Initial Base Testing - Edmond, OK
U.S. Environmental Protection Agency
Office of Research and Development
PI: Clements.Andrea@epa.gov
919-541-1363
August 2019—September 2019

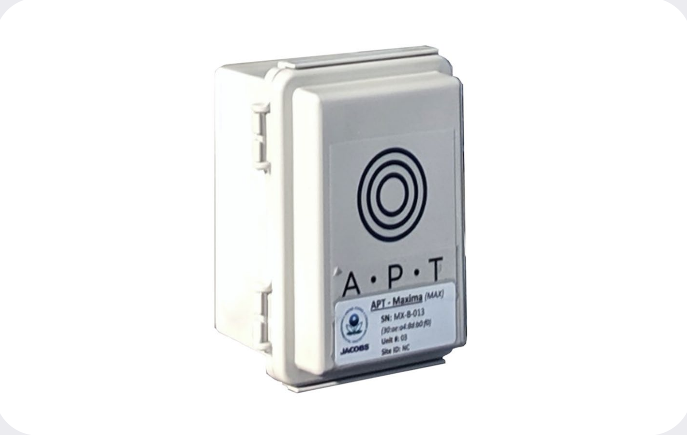

Deployment Details

Testing Organization and Site Information	
Testing organization (Name, Organization type, Contact website)	U.S. Environmental Protection Agency - Office of Research and Development<br>Federal Government<br>Air Sensor Toolbox | U.S. EPA Website
Testing location (City, State, Latitude and Longitude)	OK Christian University<br>Edmond, OK<br>35.614131, -97.475083
AQS site ID	40 - 109 - 1037
Sampling timeframe (MM-DD-YY)	08-02-19 to 09-01-19
Sensor data source	Aeroqual Cloud download
Reference data source	AQS API download

Sensor Information			
Manufacturer, model	APT Maxima		
Device firmware version	v5.0 (installed 5/2/2019)		
Sampling time interval	30-seconds		
Sensor serial numbers	MAX_01		
Issues encountered during deployment?	☐	No Issues	

FRM/FEM Information	
Manufacturer, model, designation	Teledyne Advanced Pollution Instrumentation T640 FEM
Sampling time interval	1-hour averaging
Date of calibration	As required by 40 CFR Part 58 and the QAPP for Ambient Air Monitoring in the State of Oklahoma maintained by ODEQ/AQD
Date of flowrate verification check	Monthly as required by 40 CFR Part 58 Appendix A
Description, date(s) of maintenance activities	N/A

Time Series Plots

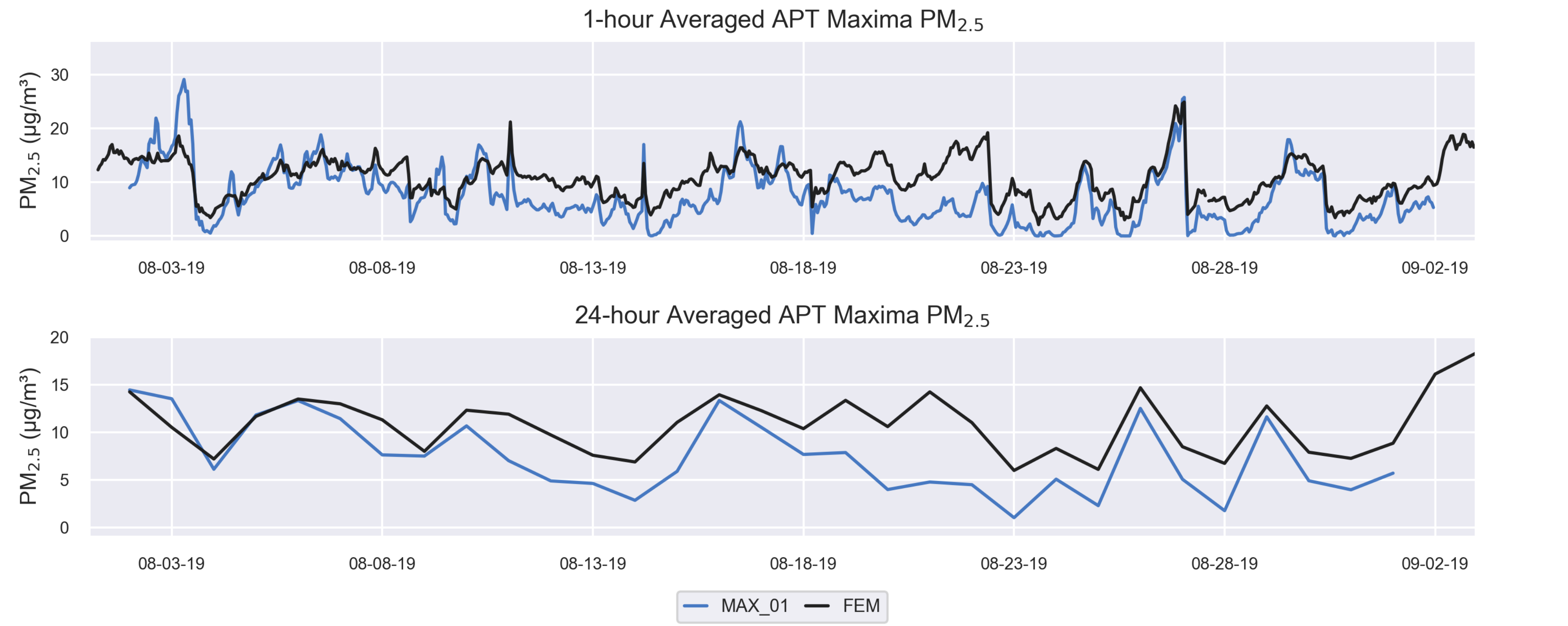

Scatter Plots: Comparison to FRM/FEM

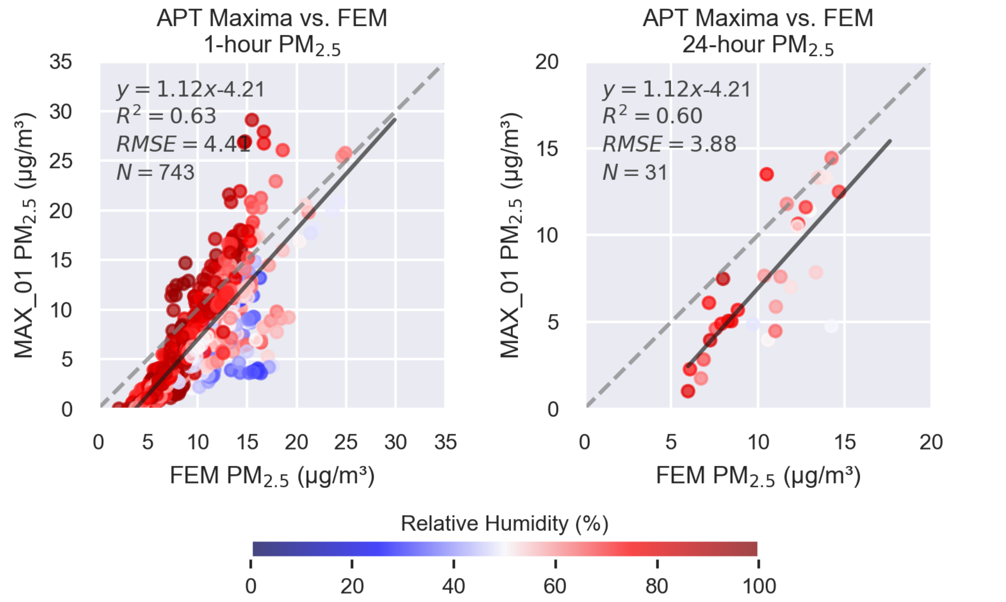

Range of FRM/FEM concentrations over duration of base test (μg/m³)	2.1-24.9 (1-hr), 6.0-14.7 (24-hr)
Number of 24-hr periods in FRM/FEM monitor measurements with a goal concentration ≥ 25 μg/m³	0

Performance Metrics

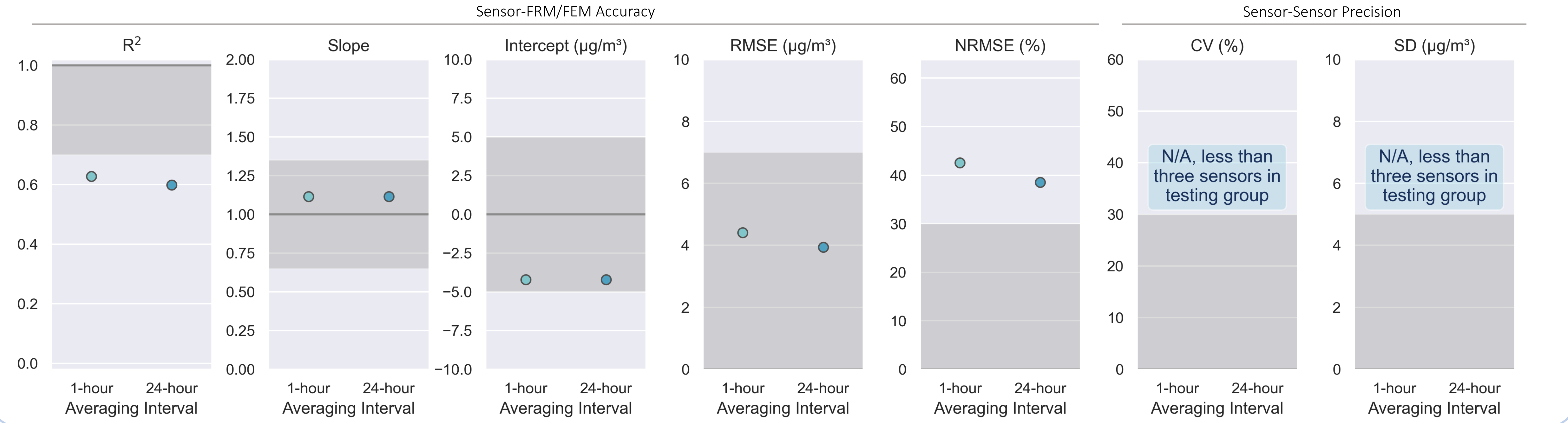

Meteorological Conditions During Deployment

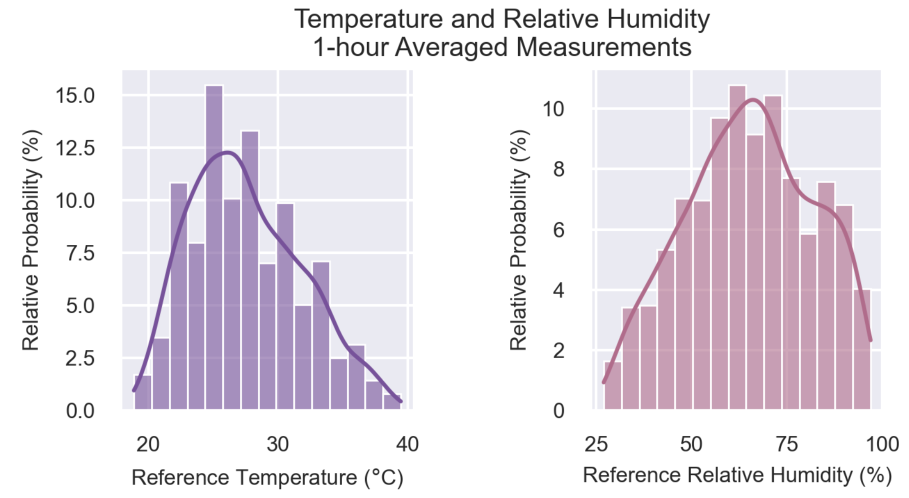

Number of 24-hr periods outside sensor manufacturer-listed temperature operational range (no operational range specified)	-
Number of 24-hr periods outside sensor manufacturer-listed relative humidity operational range (no operational range specified)	-

Meteorological Influence

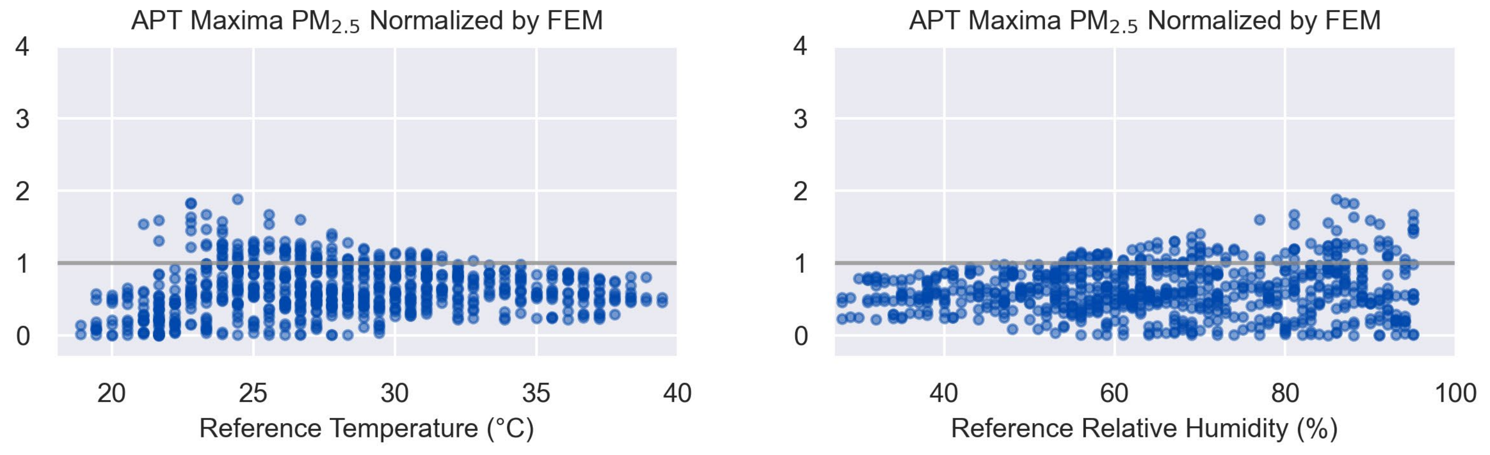

Mean number of paired, normalized concentration and temperature values (1-hr averages)	744
Mean number of paired, normalized concentration and relative humidity values (1-hr averages)	744

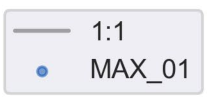

Testing Report - $PM_{2.5}$ Base Testing

APT Maxima

This report reflects out-of-the-box performance

Initial Base Testing - Edmond, OK
U.S. Environmental Protection Agency
Office of Research and Development
PI: Clements.Andrea@epa.gov
919-541-1363
August 2019—September 2019

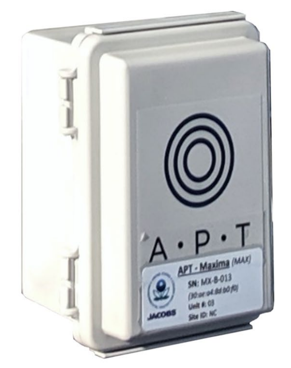

Tabular Statistics

Sensor-FRM/FEM Correlation

	Bias and Linearity						Data Quality			
	R^2		Slope		Intercept (µg/m³)		Uptime (%)		Number of paired sensor and FRM/FEM concentration values	
	1-Hour ○	24-Hour ○	1-Hour ●	24-Hour ●	1-Hour ●	24-Hour ●	1-Hour ●	24-Hour ●	1-Hour	24-Hour
Metric Target Range	≥ 0.70	≥ 0.70	1.0 ± 0.35	1.0 ± 0.35	-5 ≤ b ≤ 5	-5 ≤ b ≤ 5	75%*	75%*	-	-
Sensor MAX_01	0.63	0.60	1.12	1.12	-4.21	-4.21	100	100	743	31

	Error			
	RMSE (µg/m³)		NRMSE (%)	
	1-Hour ★	24-Hour ★	1-Hour ☆	24-Hour ☆
Metric Target Range	≤ 7.0	≤ 7.0	≤ 30.0	≤ 30.0
Deployment Value	4.4	3.9	42.6	38.5

Device-specific metrics (computed for each sensor in evaluation)
○○○ Metric value for none of devices tested falls within the target range
●○○ Metric value for one of devices tested falls within the target range
●●○ Metric value for two of devices tested falls within the target range
●●● Metric value for three of devices tested falls within the target range

Single-valued metrics (computed via entire evaluation dataset)
☆ Indicates that the metric value is not within the target range
★ Indicates that the metric value is within the target range

Sensor-Sensor Precision[1]

	Precision (between collocated sensors)				Data Quality	
	CV (%)		SD (µg/m³)		Number of concurrent sensor concentration pairs	
	1-Hour ☆	24-Hour ☆	1-Hour ☆	24-Hour ☆	1-Hour	24-Hour
Metric Target Range	≤ 30.0	≤ 30.0	≤ 5.0	≤ 5.0	-	-
Deployment Value	-	-	-	-	-	-

[1]Precision statistics are computed for evaluations with at least three collocated sensor units. Metric values are left blank for evaluations with two or fewer sensor units.

*This value is only a recommendation for ensuring data quality and is not included in the list of target values discussed in Section 4 of the Performance Testing Protocols, Metrics, and Target Values for Fine Particulate Matter Air Sensors document.

Testing Report - $PM_{2.5}$ Base Testing

APT Maxima

This report reflects out-of-the-box performance

Initial Base Testing - Edmond, OK
U.S. Environmental Protection Agency
Office of Research and Development
PI: Clements.Andrea@epa.gov
919-541-1363
August 2019—September 2019

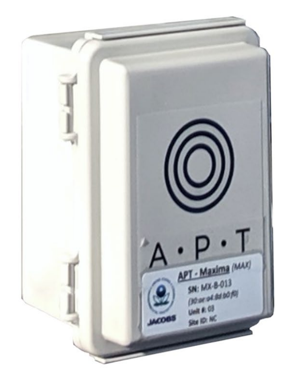

Sensor-FRM/FEM Scatter Plots

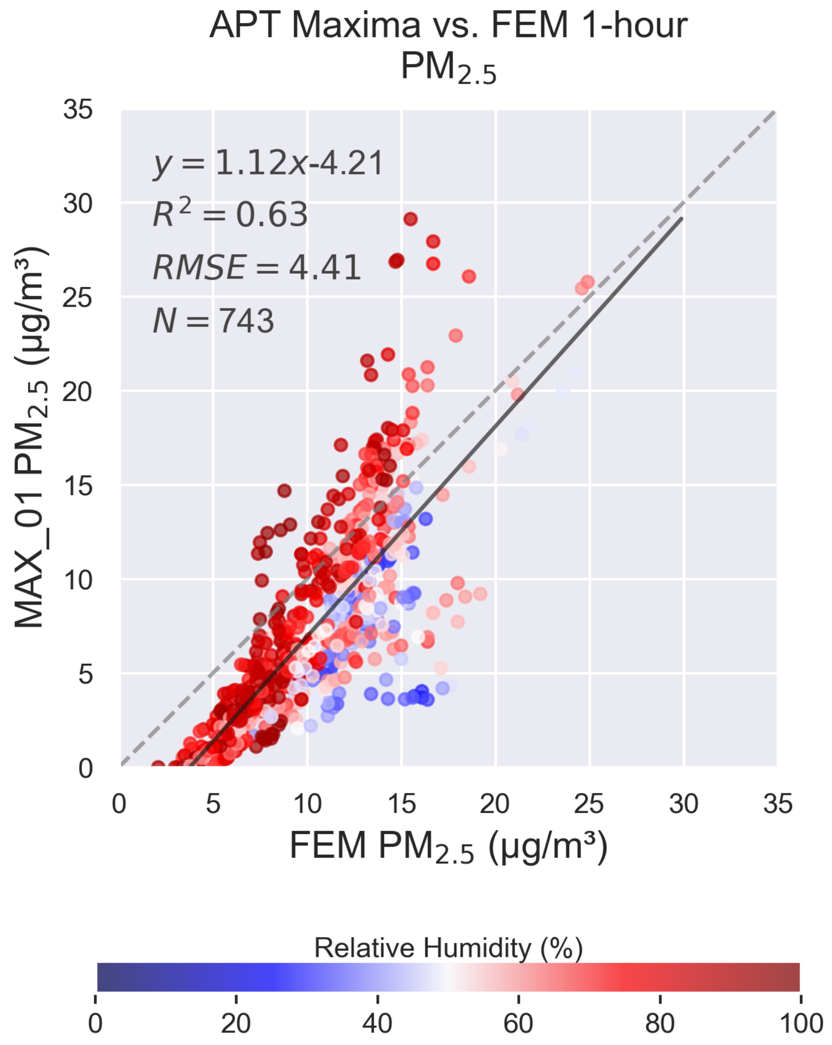

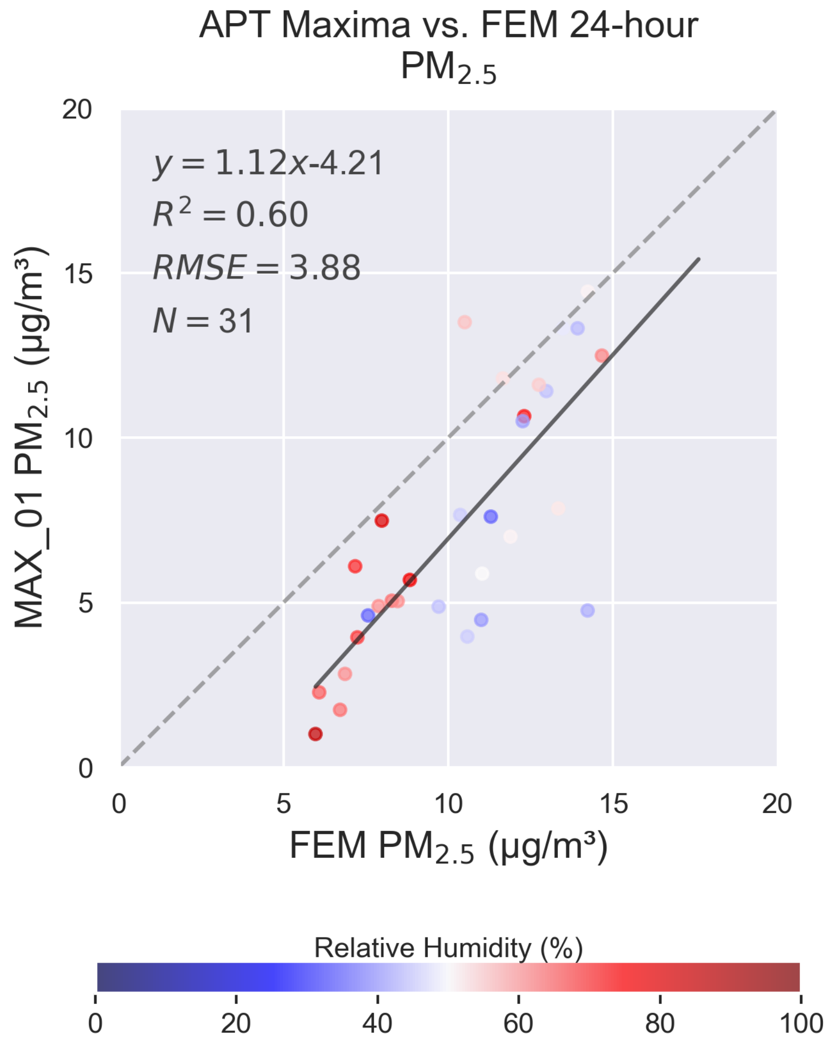

Testing Report - $PM_{2.5}$ Base Testing

APT Maxima

This report reflects out-of-the-box performance

Initial Base Testing - Edmond, OK
U.S. Environmental Protection Agency
Office of Research and Development
PI: Clements.Andrea@epa.gov
919-541-1363
August 2019—September 2019

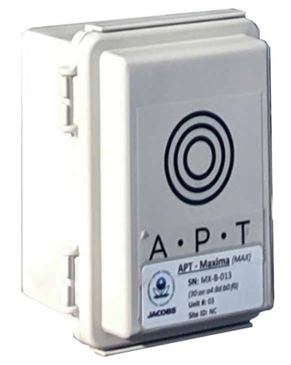

Supplemental Information

Abbreviations used in Supplemental Information

FRM	Federal Reference Method
FEM	Federal Equivalent Method
SOP	Standard Operating Procedure
QAPP	Quality Assurance Project Plan
QC	Quality Control

Supplemental Documentation	Attached	Description & URL or file path to documentation
Field observations and sensor data flags	☑	See OK-MAX-Page 6 of this testing report
Maintenance logs	☐	No logs recorded during testing
Standard operating procedure(s)	☐	U.S. EPA Office Of Research and Development SOP available upon request
Photos of equipment setup and testing	☑	See OK-MAX-Page 5 of this testing report
Product specifications sheet(s)	☐	N/A
Product manual(s)	☐	N/A
Data storage and transmission method	☑	See OK-MAX-Page 6 of this testing report
Data correction approach	☑	See OK-MAX-Page 6 of this testing report
Issues encountered	☑	See OK-MAX-Page 6 of this testing report. No issues were encountered during testing, however, an abnormality in the duration of sampling intervals was noted during the pre-deployment phase.
Data analysis/correction scripts and version	☑	Averaging and provcessing of data, calculation of performance metrics, and generation of figures and other supplementary material for analysis were obtained using Python 3.9.7 with the packages sensortoolkit v0.8.3b2, pandas 1.3.5, NumPy 1.21.2, Matplotlib 3.5.0, statsmodels 0.13.0, and seaborn 0.11.2. All packages are available from the Python Package Index (PyPI) at https://pypi.org. The integrated development environment (IDE) Spyder 5.1.5 was used for scripting and data visualization. Version control for the Python base, packages, and IDE were all managed by conda 4.11.0.
Air Monitoring Station QAPP	☐	U.S. EPA Office Of Research and Development QAPP available upon request
Summary of FRM/FEM monitor QC checks	☑	See OK-MAX-Page 7 of this testing report
Manufacturer website for FRM/FEM monitor	☑	Teledyne API: T640/T640X Product website
FRM/FEM monitor manual	☑	Teledyne API: T640/T640X Product Manual
FRM/FEM monitor specifications sheet(s)	☑	Teledyne API: T640/T640X Specification Sheet
Other documents	☐	

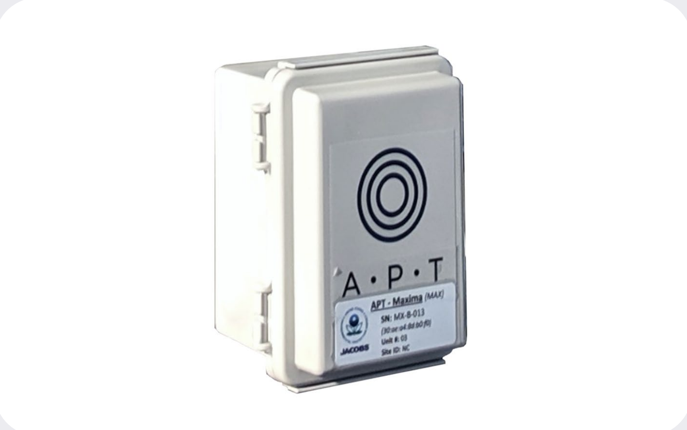

Supplemental Information: Photos of Testing Site and Equipment Setup

Site Description:
The Oklahoma Christian University monitoring site was established in the late 1970s. There is a containerized building that houses the continuous instruments with a wooden platform directly adjacent for the filter-based instruments. Pollutants monitored include O_3, PM_{10} (filter based and continuous), $PM_{2.5}$ (filter based and continuous), $NO/NO_2/NO_x$, CO, SO_2 and meteorology. The site has solar panels to help defer the cost of power to the site.

Figure 1: APT Maxima (indicated by red arrow) attached to metal railing atop the sampling shelter at the monitoring site.

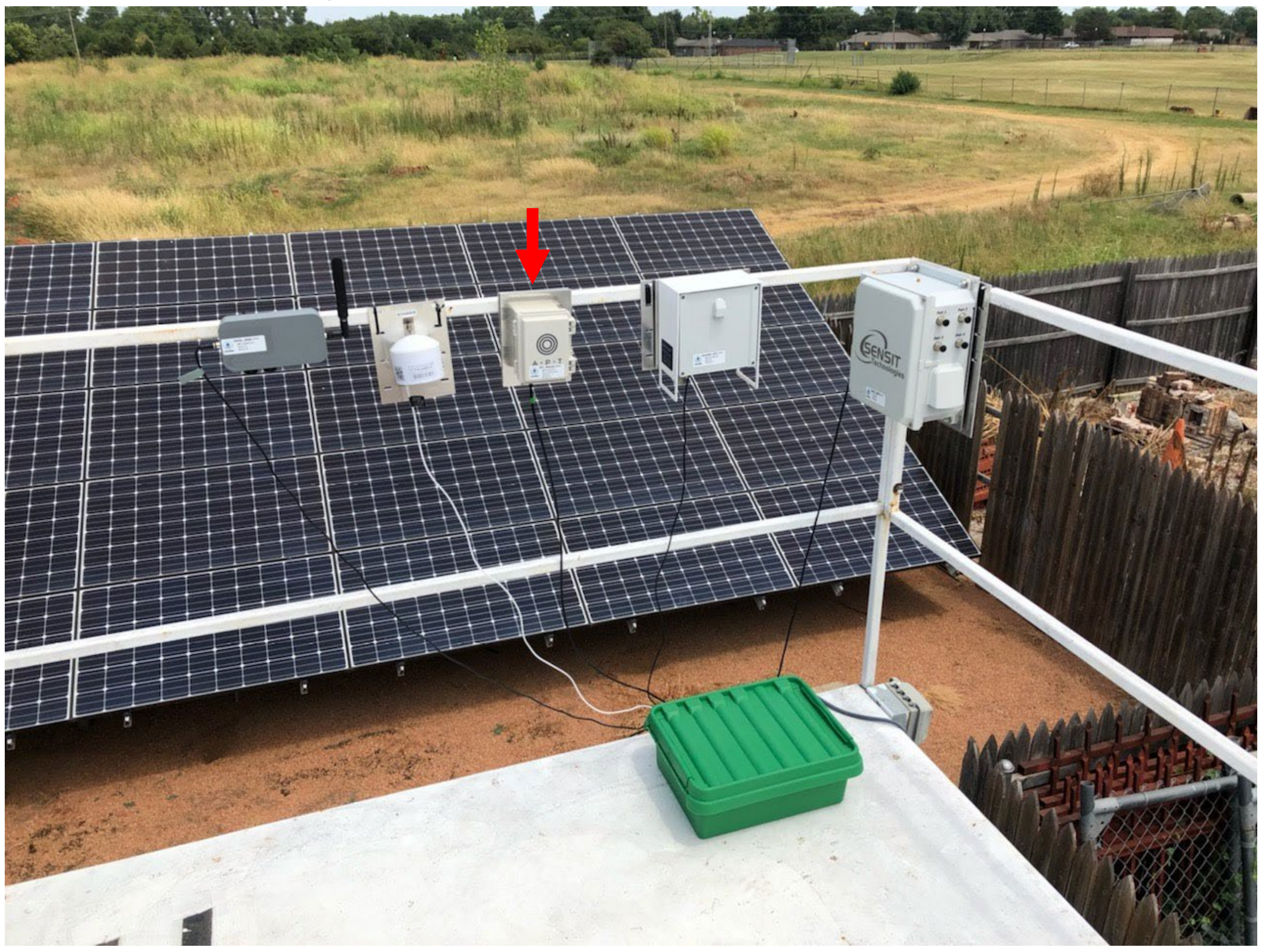

Figure 2: OK Monitoring Station sampling shelter

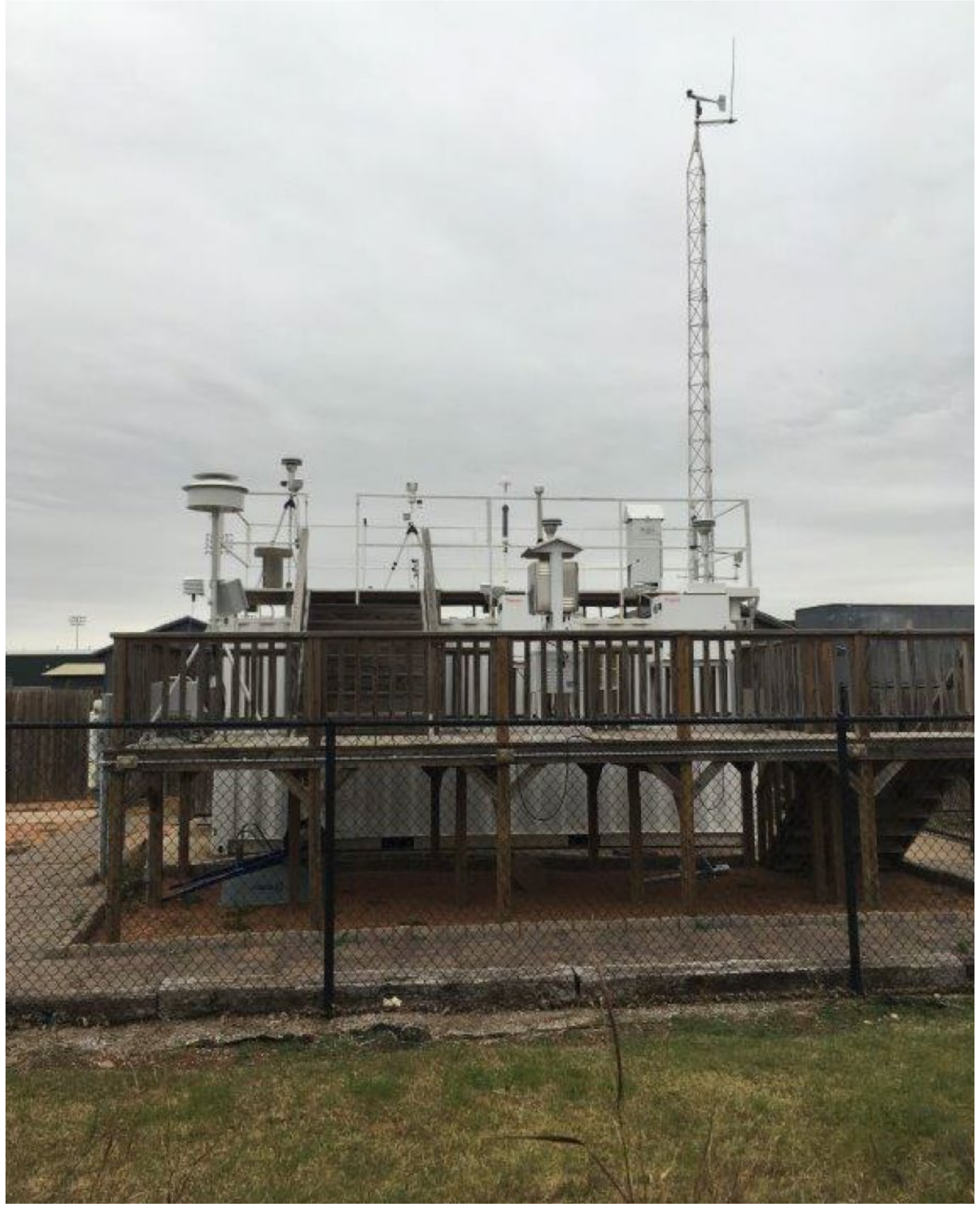

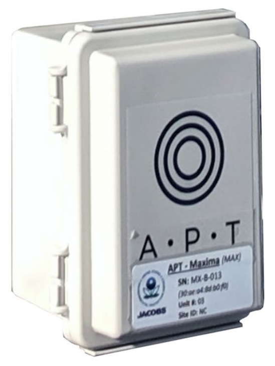

Supplemental Information: Data Storage, Correction Approach, and Issues Encountered

Data Storage and Transmission Method

The APT Maxima records data at 30 second intervals and stores the data on an internal microSD card and transmits data to the APT cloud server (last accessed 5/11/22) via Wi-Fi connection. The user interface for the Maxima sensors was under development during sensor testing, thus weekly raw data files were obtained from the internal MicroSD card. Each field site operator was provided two labeled MicroSD cards for the Maxima that they used to swap out each week. Data from the collected card was then read and processed off-site.

Data Correction Approach

This evaluation report reflects "out-of-the-box" performance of the APT Maxima. The Maxima data sets include two data columns contain $PM_{2.5}$ concentration measurements, "PM2.5(ug/m3)" and "PM2.5_Atm(ug/m3)". These columns correspond to separate correction factors provided by the onboard OEM PM sensor (Plantower PMSA003). For this report, $PM_{2.5}$ measurements for the column labeled "PM2.5(ug/m3)" were selected for analysis.

After acquisition, the raw data was processed using the *sensortoolkit* python code library (v0.8.3b2). A continuous data set at the recorded sampling frequency was written to a .csv file. 1-hour and 24-hour averaged data sets were generated using a 75% completeness threshold and saved as separate .csv files. Outliers were NOT removed from data sets in order to assess "out-of-the-box" sensor performance.

Issues Encountered

Pre-deployment observations

- *Sampling internal abnormality:* Maxima devices did not continuously sample at 30-second intervals as stated in the sensor user guide. Instead, Maxima units occasionally sampled at 31 seconds. This increased sample interval is due to the accumulation of processing time over a certain number of samples. Each 30-second sample is a collection of exactly 30 seconds of sampling followed by a brief fraction of a second used for processing the collected data. Once processed, the next 30-second data collection period begins. When the sum of the processing time reaches one full second, the sample interval then jumps to 31 seconds. Data from this pre-deployment testing indicated that the processing speed for each sensor varies such that 31-second sampling duration occur at random intervals across Maxima units.

Field observations and sensor data flags

The APT Maxima unit was deployed at the OK monitoring site on 8/1/2019. The Maxima unit operated nominally during the testing period and did not require replacement or repair.

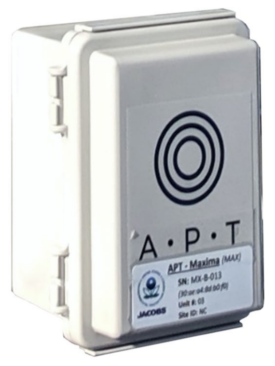

Supplemental Information: Description of FRM/FEM QC Checks and Data Flags

Description of Data Flags

AQS

The U.S. EPA's Air Quality System (AQS) is the Agency's primary ambient air monitoring data archive. A comprehensive list of data flags that are recorded alongside AQS data sets, referred to by U.S. EPA as 'qualifiers', can be found at the following link: https://aqs.epa.gov/aqsweb/documents/codetables/qualifiers.html

Invalidation of reference data: AQS qualifiers are organized by qualifier type, which indicates whether data logged alongside qualifier flags should be invalidated (set null). Qualifiers with type "Null Data Qualifier" are invalidated, and includes data logged during periods that coincide with QC checks (e.g., "BF-Precision/Zero/Span", "BJ- Operator Error", "BL - QA Audit", "AZ - QC Audit") among other events such as power outages. Data logged alongside qualifiers with type "Quality Assurance Qualifiers" are not invalidated and are included in this analysis (e.g., concentrations less than the federal MDL for the reference monitor "MD – Value less than MDL", QA reviewed values "Validated Value").

Data Flags Recorded During Testing

FRM/FEM Monitor	Timestamp (UTC)	Flag
Teledyne API T640 (Acquired via AQS)	2019-08-27 14:00:00+0000	AZ - QC Audit

Testing Report - $PM_{2.5}$ Base Testing

Clarity Node

This report reflects out-of-the-box performance

Initial Base Testing - Edmond, OK

U.S. Environmental Protection Agency
Office of Research and Development
PI: Clements.Andrea@epa.gov
919-541-1363
August 2019—September 2019

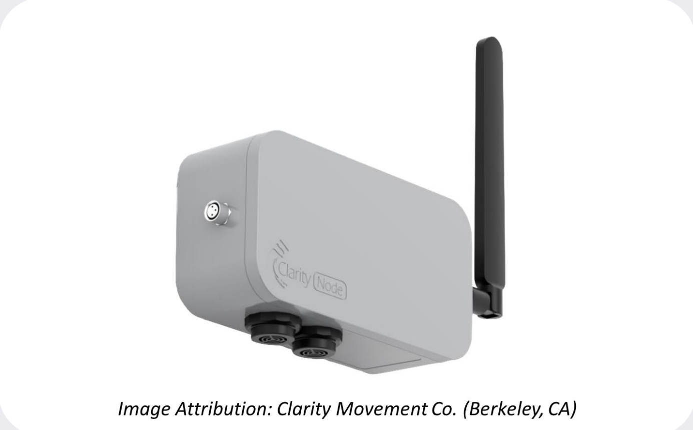

Deployment Details

Testing Organization and Site Information	
Testing organization (Name, Organization type, Contact website)	U.S. Environmental Protection Agency - Office of Research and Development<br>Federal Government<br>Air Sensor Toolbox | U.S. EPA Website
Testing location (City, State, Latitude and Longitude)	OK Christian University<br>Edmond, OK<br>35.614131, -97.475083
AQS site ID	40 - 109 - 1037
Sampling timeframe (MM-DD-YY)	08-21-19 to 09-20-19
Sensor data source	Clarity Dashboard download
Reference data source	AQS API download

Sensor Information			
Manufacturer, model	Clarity Node		
Device firmware version	Received by EPA May 2019		
Sampling time interval	3-minutes		
Sensor serial numbers	CNO_01		
Issues encountered during deployment?	☑	See OK-CNO-Page 6 of this testing report	

FRM/FEM Information	
Manufacturer, model, designation	Teledyne Advanced Pollution Instrumentation T640 FEM
Sampling time interval	1-hour averaging
Date of calibration	As required by 40 CFR Part 58 and the QAPP for Ambient Air Monitoring in the State of Oklahoma maintained by ODEQ/AQD
Date of flowrate verification check	Monthly as required by 40 CFR Part 58 Appendix A
Description, date(s) of maintenance activities	N/A

Time Series Plots

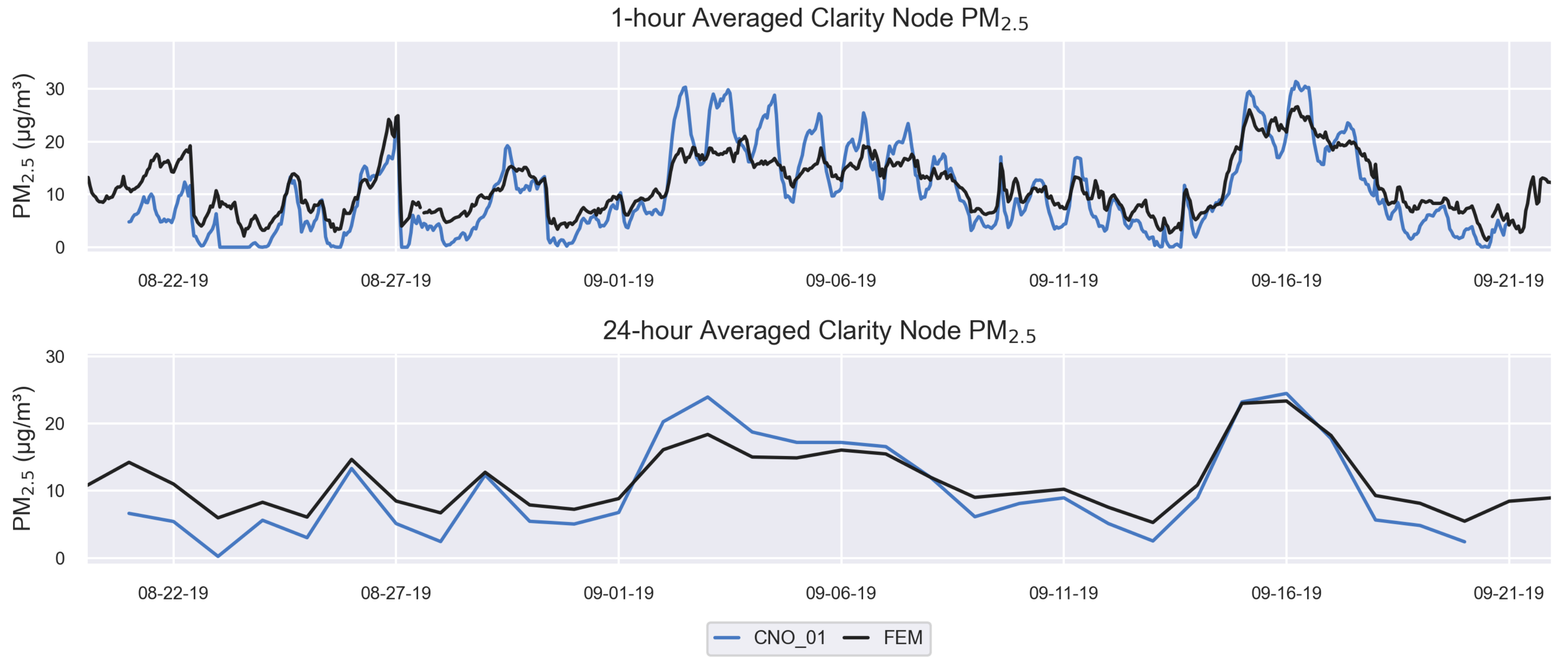

Scatter Plots: Comparison to FRM/FEM

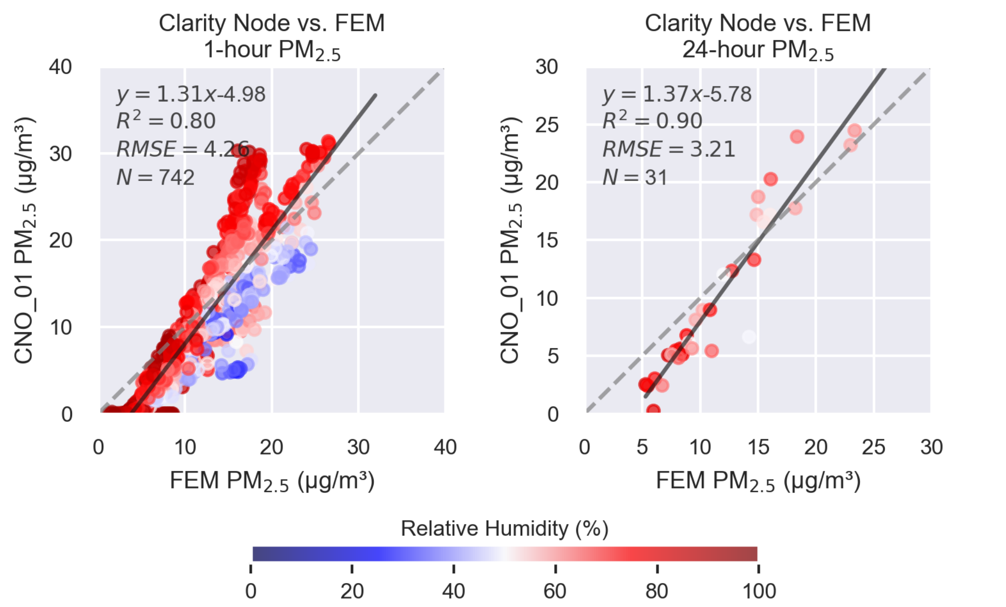

Range of FRM/FEM concentrations over duration of base test (µg/m³)	1.3-26.6 (1-hr), 5.3-23.4 (24-hr)
Number of 24-hr periods in FRM/FEM monitor measurements with a goal concentration ≥ 25 µg/m³	0

Performance Metrics

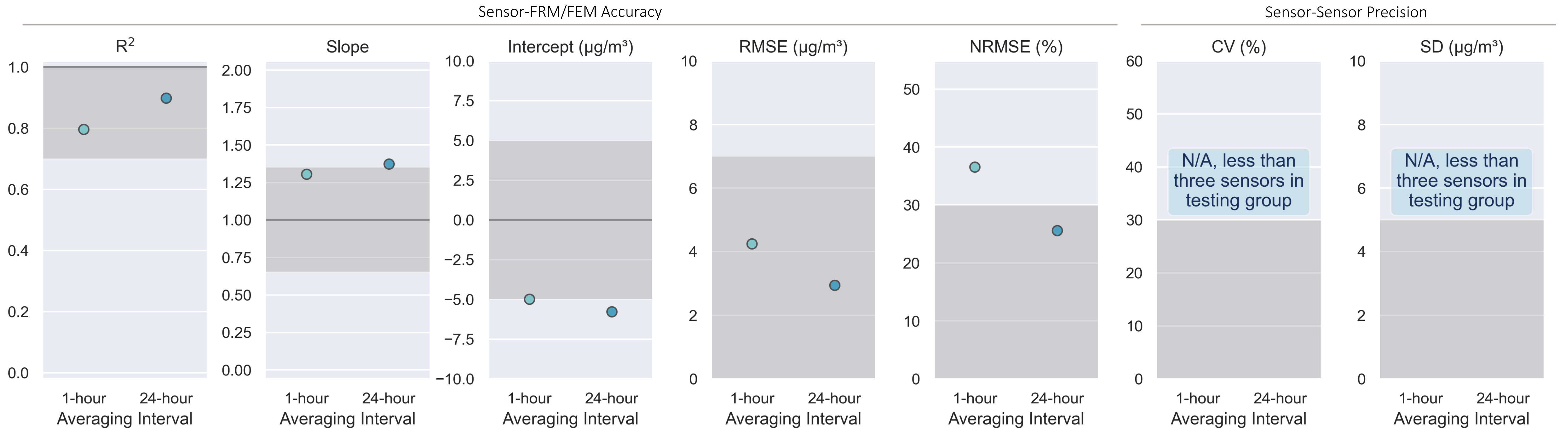

Meteorological Conditions During Deployment

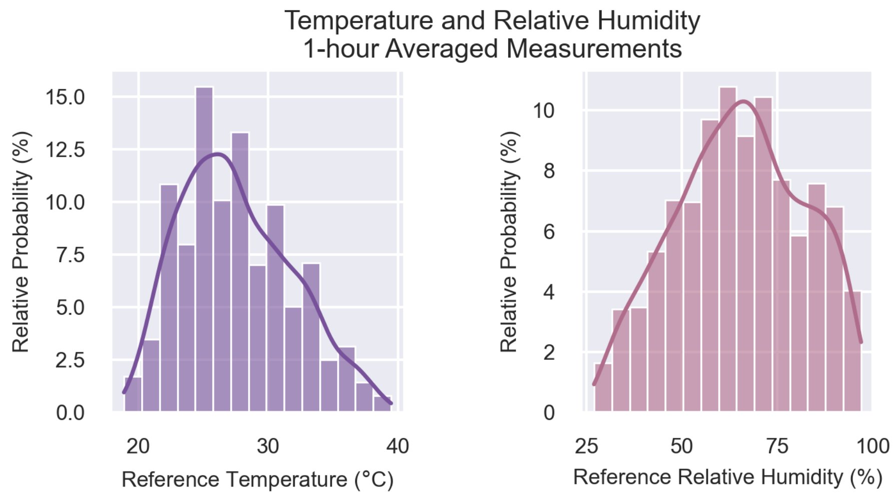

Number of 24-hr periods outside sensor manufacture-listed temperature operational range (-10 to 55 °C)	0
Number of 24-hr periods outside sensor manufacture-listed relative humidity operational range (10 to 98%)	0

Meteorological Influence

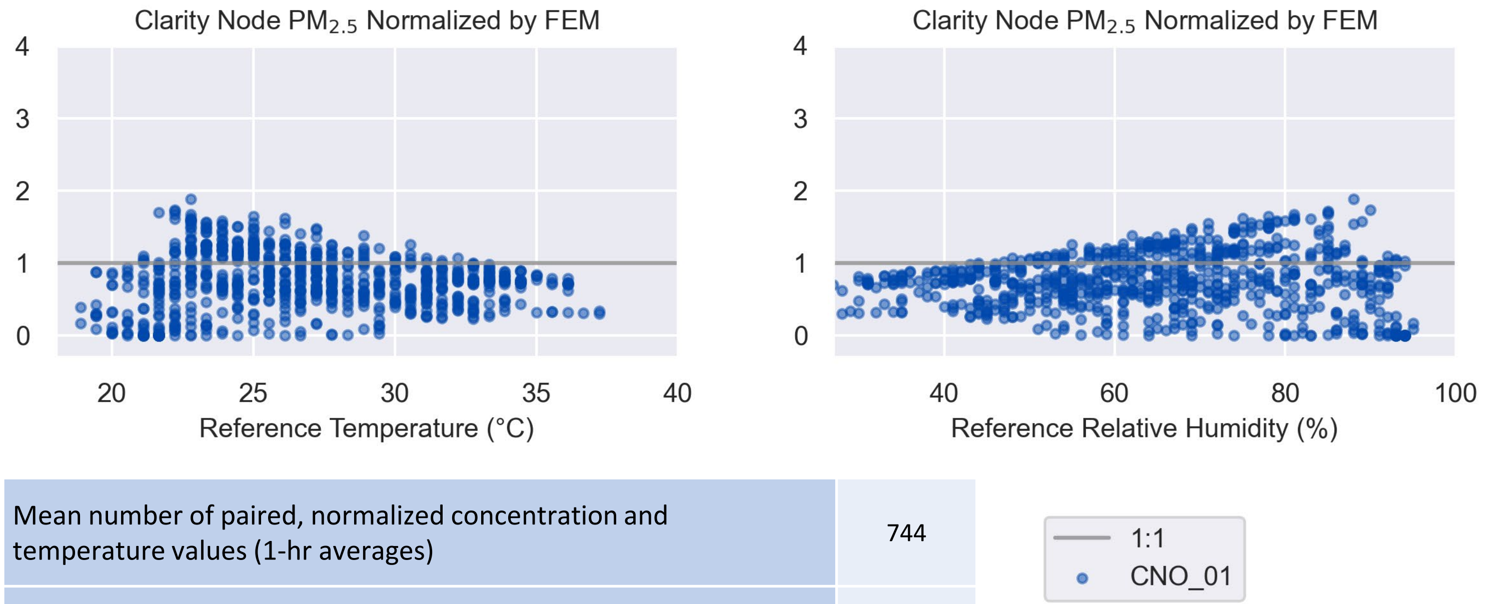

Mean number of paired, normalized concentration and temperature values (1-hr averages)	744
Mean number of paired, normalized concentration and relative humidity values (1-hr averages)	744

Testing Report - $PM_{2.5}$ Base Testing

Clarity Node

This report reflects out-of-the-box performance

Initial Base Testing - Edmond, OK

U.S. Environmental Protection Agency
Office of Research and Development
PI: Clements.Andrea@epa.gov
919-541-1363

August 2019—September 2019

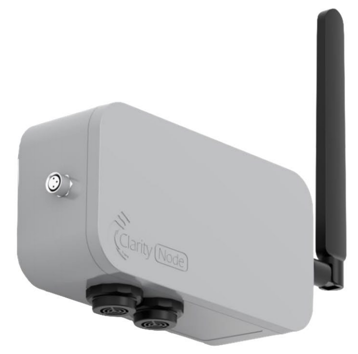

Image Attribution: Clarity Movement Co. (Berkeley, CA)

Tabular Statistics

Sensor-FRM/FEM Correlation

	Bias and Linearity						Data Quality			
	R^2		Slope		Intercept ($\mu g/m^3$)		Uptime (%)		Number of paired sensor and FRM/FEM concentration values	
	1-Hour ●	24-Hour ●	1-Hour ●	24-Hour ○	1-Hour ●	24-Hour ○	1-Hour ●	24-Hour ●	1-Hour	24-Hour
Metric Target Range	≥ 0.70	≥ 0.70	1.0 ± 0.35	1.0 ± 0.35	-5 ≤ b ≤ 5	-5 ≤ b ≤ 5	75%*	75%*	-	-
Sensor CNO_01	0.80	0.90	1.31	1.37	-4.98	-5.78	100	100	742	31

	Error			
	RMSE ($\mu g/m^3$)		NRMSE (%)	
	1-Hour ★	24-Hour ★	1-Hour ☆	24-Hour ★
Metric Target Range	≤ 7.0	≤ 7.0	≤ 30.0	≤ 30.0
Deployment Value	4.3	2.9	36.6	25.6

Device-specific metrics (computed for each sensor in evaluation)
○○○ Metric value for none of devices tested falls within the target range
●○○ Metric value for one of devices tested falls within the target range
●●○ Metric value for two of devices tested falls within the target range
●●● Metric value for three of devices tested falls within the target range

Single-valued metrics (computed via entire evaluation dataset)
☆ Indicates that the metric value is not within the target range
★ Indicates that the metric value is within the target range

Sensor-Sensor Precision[1]

	Precision (between collocated sensors)				Data Quality	
	CV (%)		SD ($\mu g/m^3$)		Number of concurrent sensor concentration pairs	
	1-Hour ☆	24-Hour ☆	1-Hour ☆	24-Hour ☆	1-Hour	24-Hour
Metric Target Range	≤ 30.0	≤ 30.0	≤ 5.0	≤ 5.0	-	-
Deployment Value	-	-	-	-	-	-

[1]Precision statistics are computed for evaluations with at least three collocated sensor units. Metric values are left blank for evaluations with two or fewer sensor units.

*This value is only a recommendation for ensuring data quality and is not included in the list of target values discussed in Section 4 of the Performance Testing Protocols, Metrics, and Target Values for Fine Particulate Matter Air Sensors document.

Testing Report - $PM_{2.5}$ Base Testing

Clarity Node

This report reflects out-of-the-box performance

Initial Base Testing - Edmond, OK
U.S. Environmental Protection Agency
Office of Research and Development
PI: Clements.Andrea@epa.gov
919-541-1363
August 2019—September 2019

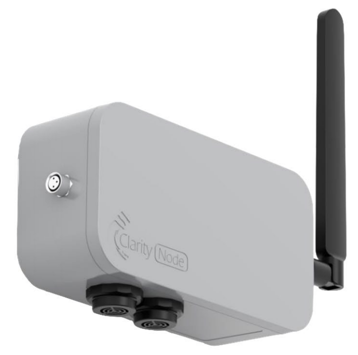

Image Attribution: Clarity Movement Co. (Berkeley, CA)

Sensor-FRM/FEM Scatter Plots

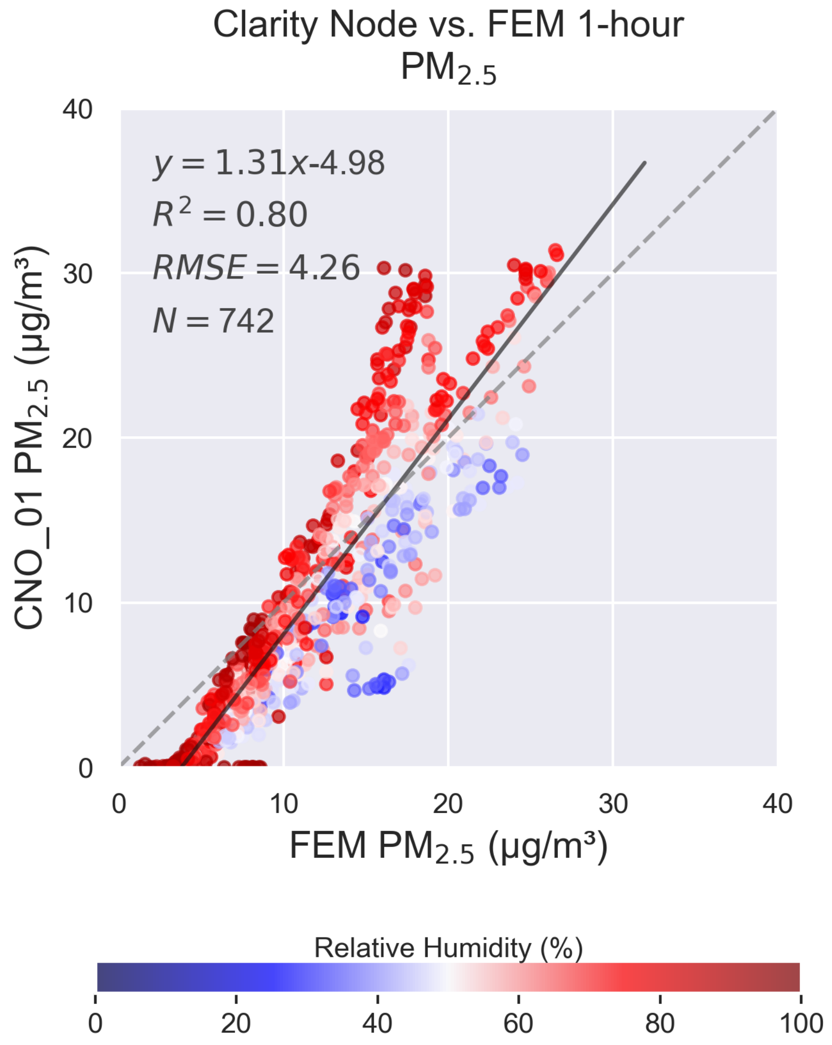

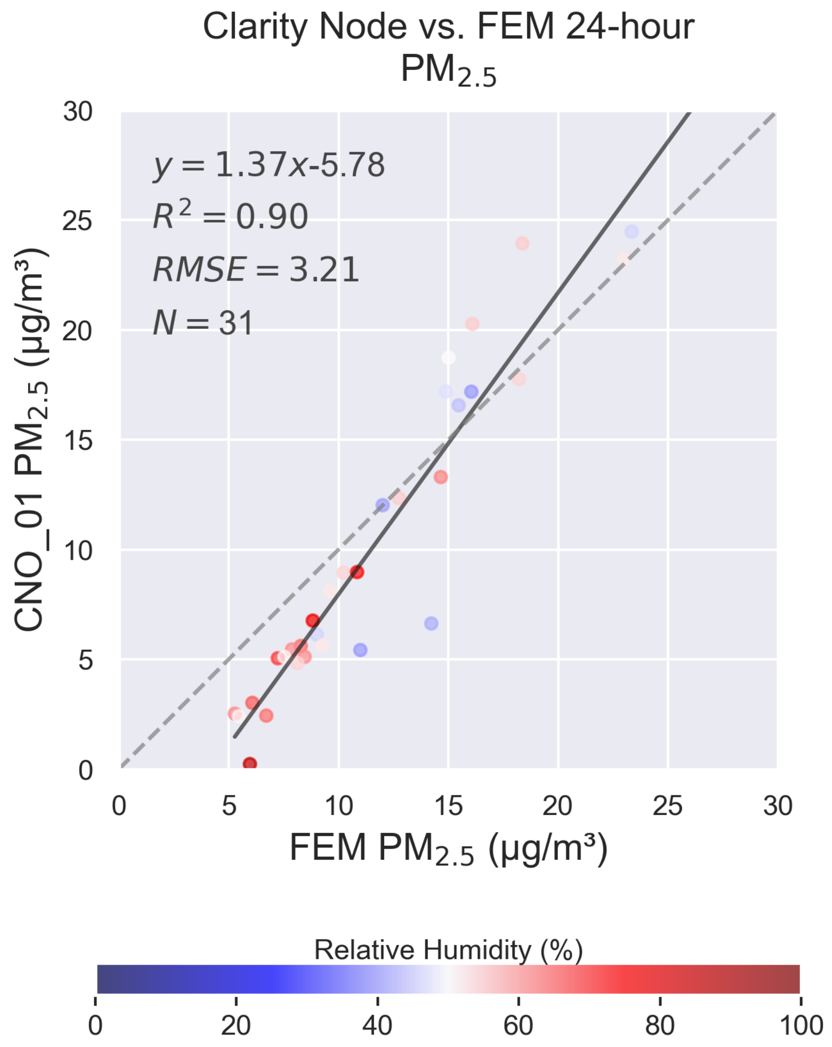

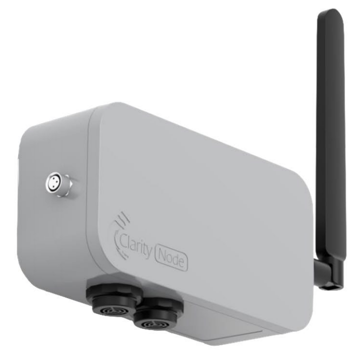

Image Attribution: Clarity Movement Co. (Berkeley, CA)

Supplemental Information

Abbreviations used in Supplemental Information

FRM	Federal Reference Method
FEM	Federal Equivalent Method
SOP	Standard Operating Procedure
QAPP	Quality Assurance Project Plan
QC	Quality Control

Supplemental Documentation	Attached	Description & URL or file path to documentation
Field observations and sensor data flags	☑	See OK-CNO-Page 6 of this testing report
Maintenance logs	☐	No logs recorded during testing
Standard operating procedure(s)	☐	U.S. EPA Office Of Research and Development SOP available upon request
Photos of equipment setup and testing	☑	See OK-CNO-Page 5 of this testing report
Product specifications sheet(s)	☐	N/A
Product manual(s)	☐	N/A
Data storage and transmission method	☑	See OK-CNO-Page 6 of this testing report
Data correction approach	☑	See OK-CNO-Page 6 of this testing report
Issues encountered	☑	See OK-CNO-Page 6 of this testing report
Data analysis/correction scripts and version	☑	Averaging and processing of data, calculation of performance metrics, and generation of figures and other supplementary material for analysis were obtained using Python 3.9.7 with the packages sensortoolkit v0.8.3b2, pandas 1.3.5, NumPy 1.21.2, Matplotlib 3.5.0, statsmodels 0.13.0, and seaborn 0.11.2. All packages are available from the Python Package Index (PyPI) at https://pypi.org. The integrated development environment (IDE) Spyder 5.1.5 was used for scripting and data visualization. Version control for the Python base, packages, and IDE were all managed by conda 4.11.0.
Air Monitoring Station QAPP	☐	U.S. EPA Office Of Research and Development QAPP available upon request
Summary of FRM/FEM monitor QC checks	☑	See OK-CNO-Page 7 of this testing report
Manufacturer website for FRM/FEM monitor	☑	Teledyne API: T640/T640X Product website
FRM/FEM monitor manual	☑	Teledyne API: T640/T640X Product Manual
FRM/FEM monitor specifications sheet(s)	☑	Teledyne API: T640/T640X Specification Sheet
Other documents	☐	

PI: Clements.Andrea@epa.gov

919-541-1363

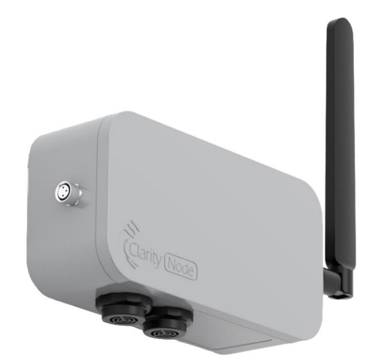

Image Attribution: Clarity Movement Co. (Berkeley, CA)

Supplemental Information: Photos of Testing Site and Equipment Setup

Site Description:

The Oklahoma Christian University monitoring site was established in the late 1970s. There is a containerized building that houses the continuous instruments with a wooden platform directly adjacent for the filter-based instruments. Pollutants monitored include O_3, PM_{10} (filter based and continuous), $PM_{2.5}$ (filter based and continuous), NO/NO_2/NO_x, CO, SO_2 and meteorology. The site has solar panels to help defer the cost of power to the site.

Figure 1: Clarity Node (indicated by red arrow) attached to metal railing atop the sampling shelter at the monitoring site.

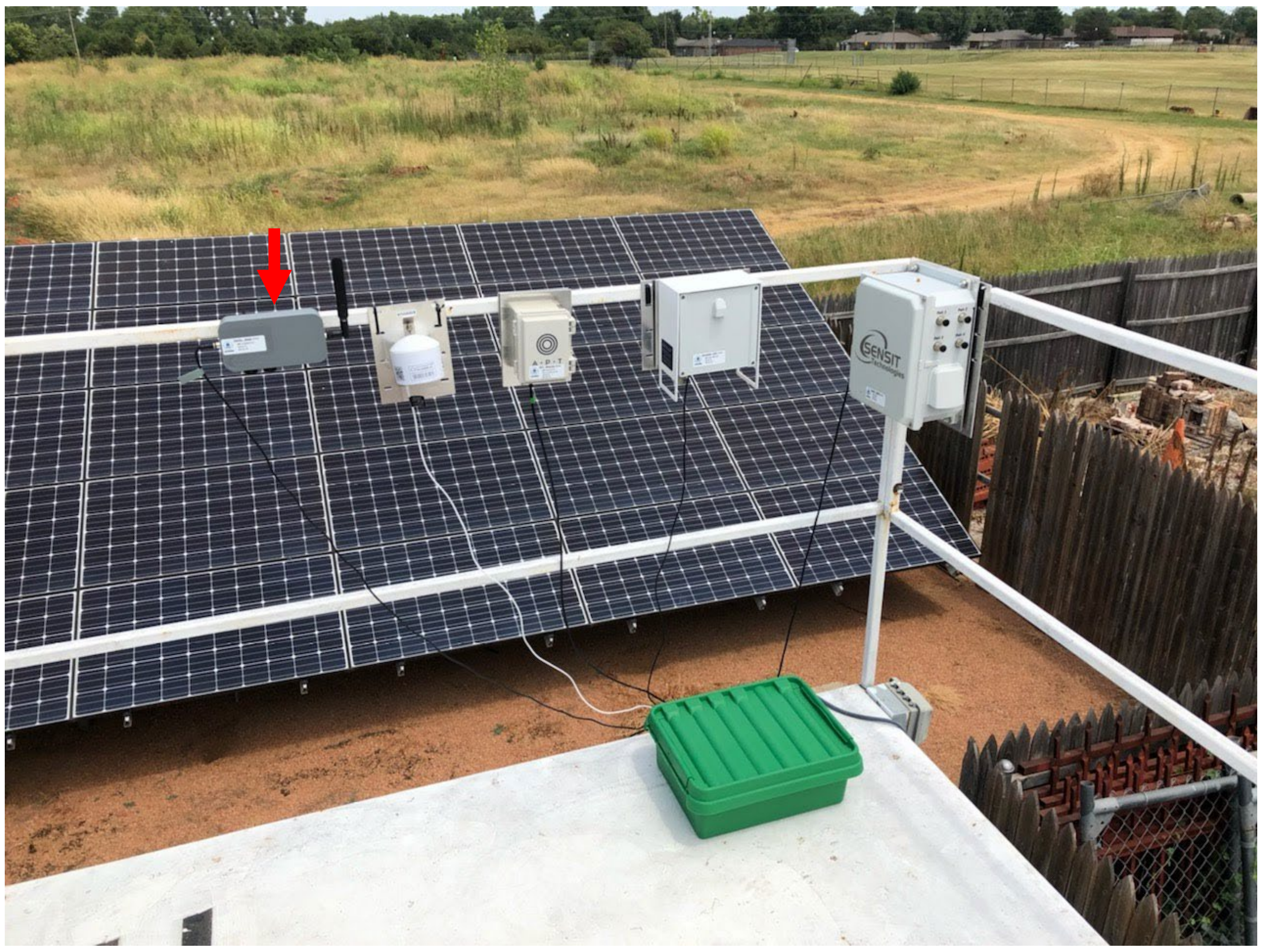

Figure 2: OK Monitoring Station sampling shelter

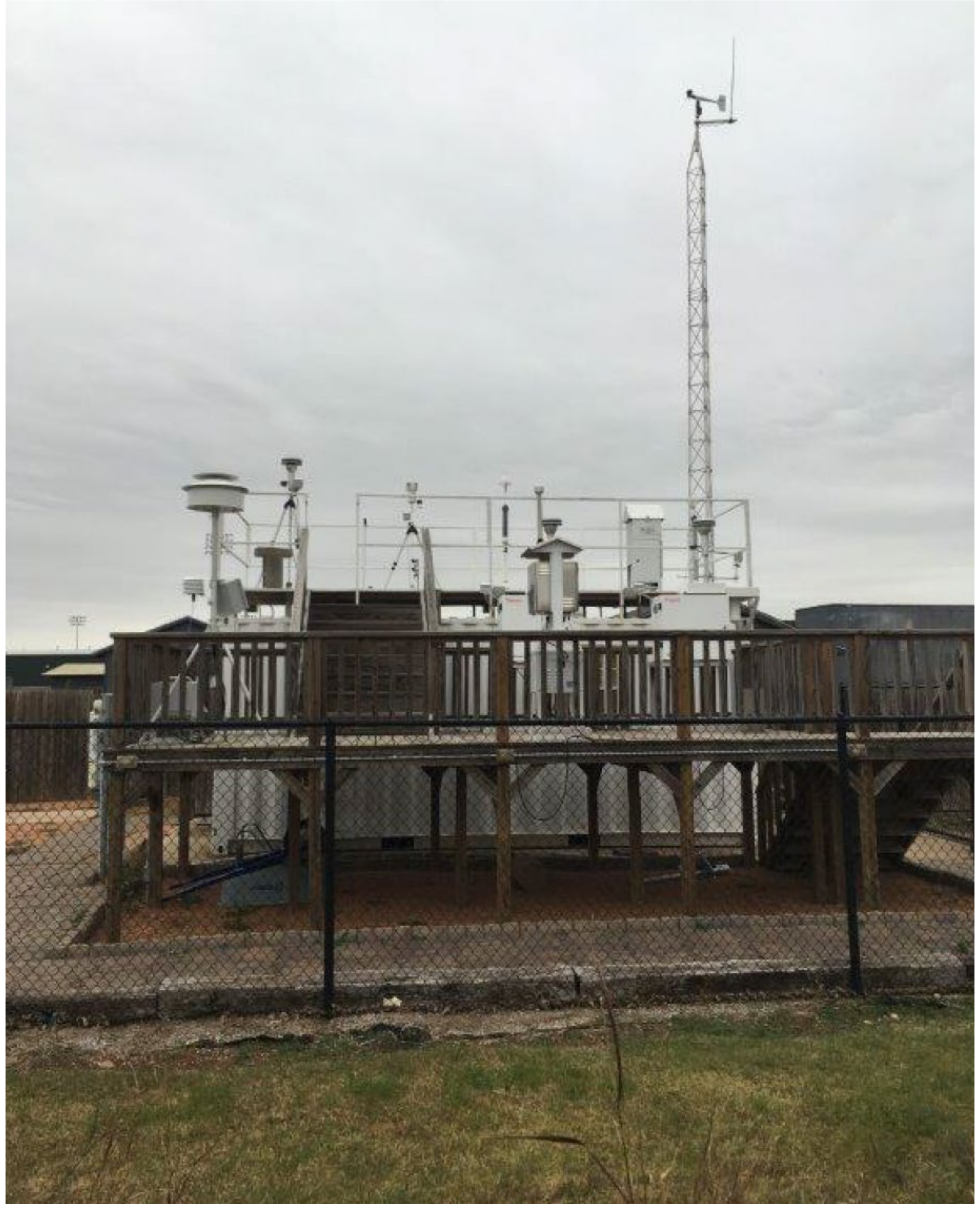

Initial Base Testing - Edmond, OK
U.S. Environmental Protection Agency
Office of Research and Development
PI: Clements.Andrea@epa.gov
919-541-1363
August 2019—September 2019

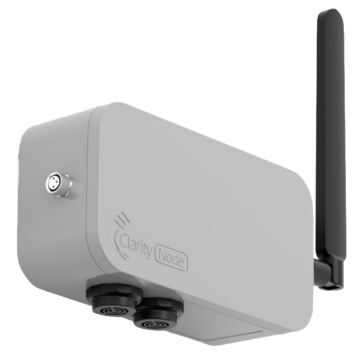
Image Attribution: Clarity Movement Co. (Berkeley, CA)

Supplemental Information: Data Storage, Correction Approach, and Issues Encountered

Data Storage and Transmission Method

The Clarity Node transmits data via cellular SIM card. Weekly raw data collections were performed over Wi-Fi using the Clarity dashboard (*last accessed 5/11/22*). The observed sampling interval fluctuated and is further discussed under pre-deployment observations below. On average, the Node models sampled every 2-3 minutes, and the Node-S sampled every 15-17 minutes.

Data Correction Approach

This evaluation report reflects "out-of-the-box" performance of the Clarity Node. The manufacturer provides a dashboard feature for calibrating sensor data against collocated reference monitors. Calibrated data were NOT used for this evaluation. Prospective consumers may get better performance from this device if they utilize this feature.

After acquisition, the raw data was processed using the *sensortoolkit* python code library (v0.8.3b2). A continuous data set at the recorded sampling frequency was written to a .csv file. 1-hour and 24-hour averaged data sets were generated using a 75% completeness threshold and saved as separate .csv files. Due to the variable sampling interval observed for Clarity Node units, 75% completeness was determined against the number of data points that would be recorded within 1-hour/24-hour periods if the sensor were to log measurements at the most commonly recorded sampling interval (i.e., the mode of recorded intervals). Outliers were NOT removed from data sets in order to assess "out-of-the-box" sensor performance.

Issues Encountered

Pre-deployment observations

- *Unsteady sampling interval:* Clarity Node sensors were received by EPA on May 28, 2019. A lab bench-top evaluation was performed from May 29, 2019 – May 31, 2019. During bench-top testing, it was observed that the sampling intervals noted by Clarity (5 minutes for non-solar Node, 15 minutes for the solar Node-S) were not reflective of recorded intervals. Communication with the vendor concluded that the sampling interval will fluctuate depending on the settings, battery charge, and strength of the cellular signal and that this sampling interval cannot be adjusted by the user at present. On average, the Node models sampled every 2-3 minutes, and the Node-S sampled every 15-17 minutes.

Field observations and sensor data flags

The Clarity Node unit was deployed at the OK monitoring site on 8/1/2019. Throughout the testing period, numerous sampling interval abnormality flags were logged, associated with an occasional change in the sampling frequency of the Clarity Node. As noted during pre-deployment testing, the Node typically samples at 2 to 3-minute intervals. Occasionally, the device recorded samples at longer intervals ranging from 4 to 6 minutes, followed by a period of alternating 2 to 3-minute sampling intervals. This longer-duration sampling interval repeatedly occurred at seemingly random intervals. This change in sampling frequency was not a result of unit configuration changes applied by a field technician and reflect the out-of-the-box dynamic sampling interval as programmed by the manufacturer.

The following table contains data flags describing events that were encountered during the testing period.

Start Time (UTC)	End Time (UTC)	Sensor Serial ID	Parameters Impacted	Flag
2019-09-05 20:40:00+00:00	2019-09-11 16:14:00+00:00	CNO_01	ALL	6.0-Sampling interval abnormality
2019-09-11 16:15:00+00:00	2019-09-18 16:05:00+00:00	CNO_01	ALL	6.0-Sampling interval abnormality
2019-09-18 16:06:00+00:00	2019-09-25 16:04:00+00:00	CNO_01	ALL	6.0-Sampling interval abnormality

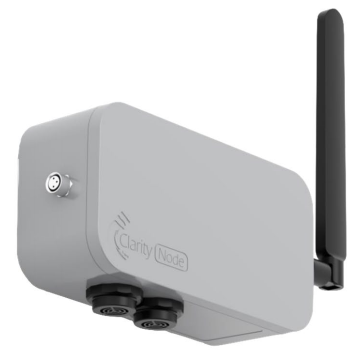

Image Attribution: Clarity Movement Co. (Berkeley, CA)

Description of Data Flags

AQS

The U.S. EPA's Air Quality System (AQS) is the Agency's primary ambient air monitoring data archive. A comprehensive list of data flags that are recorded alongside AQS data sets, referred to by U.S. EPA as 'qualifiers', can be found at the following link: https://aqs.epa.gov/aqsweb/documents/codetables/qualifiers.html

Invalidation of reference data: AQS qualifiers are organized by qualifier type, which indicates whether data logged alongside qualifier flags should be invalidated (set null). Qualifiers with type "Null Data Qualifier" are invalidated, and includes data logged during periods that coincide with QC checks (e.g., "BF-Precision/Zero/Span", "BJ- Operator Error", "BL - QA Audit", "AZ - QC Audit") among other events such as power outages. Data logged alongside qualifiers with type "Quality Assurance Qualifiers" are not invalidated and are included in this analysis (e.g., concentrations less than the federal MDL for the reference monitor "MD – Value less than MDL", QA reviewed values "Validated Value").

Data Flags Recorded During Testing

FRM/FEM Monitor	Timestamp (UTC)	Flag
Teledyne API T640 (Acquired via AQS)	2019-08-27 14:00:00+0000	AZ - QC Audit
	2019-09-20 14:00:00+0000	AZ - QC Audit

Testing Report - $PM_{2.5}$ Base Testing

PurpleAir PA-II-SD

This report reflects out-of-the-box performance

Initial Base Testing - Edmond, OK

U.S. Environmental Protection Agency
Office of Research and Development
PI: Clements.Andrea@epa.gov
919-541-1363
September 2019—October 2019

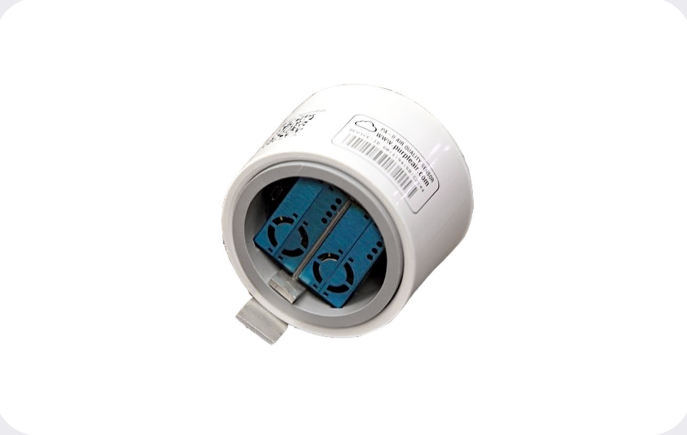

Deployment Details

Testing Organization and Site Information	
Testing organization (Name, Organization type, Contact website)	U.S. Environmental Protection Agency - Office of Research and Development<br>Federal Government<br>Air Sensor Toolbox | U.S. EPA Website
Testing location (City, State, Latitude and Longitude)	OK Christian University<br>Edmond, OK<br>35.614131, -97.475083
AQS site ID	40 - 109 - 1037
Sampling timeframe (MM-DD-YY)	09-19-19 to 10-19-19
Sensor data source	Onboard MicroSD card
Reference data source	AQS API download

Sensor Information		
Manufacturer, model	PurpleAir PA-II-SD	
Device firmware version	V4.02	
Sampling time interval	2-minutes	
Sensor serial numbers	PAR_01	
Issues encountered during deployment?	☑	See OK-PAR-Page 6 of this testing report

FRM/FEM Information	
Manufacturer, model, designation	Teledyne Advanced Pollution Instrumentation T640 FEM
Sampling time interval	1-hour averaging
Date of calibration	As required by 40 CFR Part 58 and the QAPP for Ambient Air Monitoring in the State of Oklahoma maintained by ODEQ/AQD
Date of flowrate verification check	Monthly as required by 40 CFR Part 58 Appendix A
Description, date(s) of maintenance activities	See OK-PAR-Page 7 of this testing report

Time Series Plots

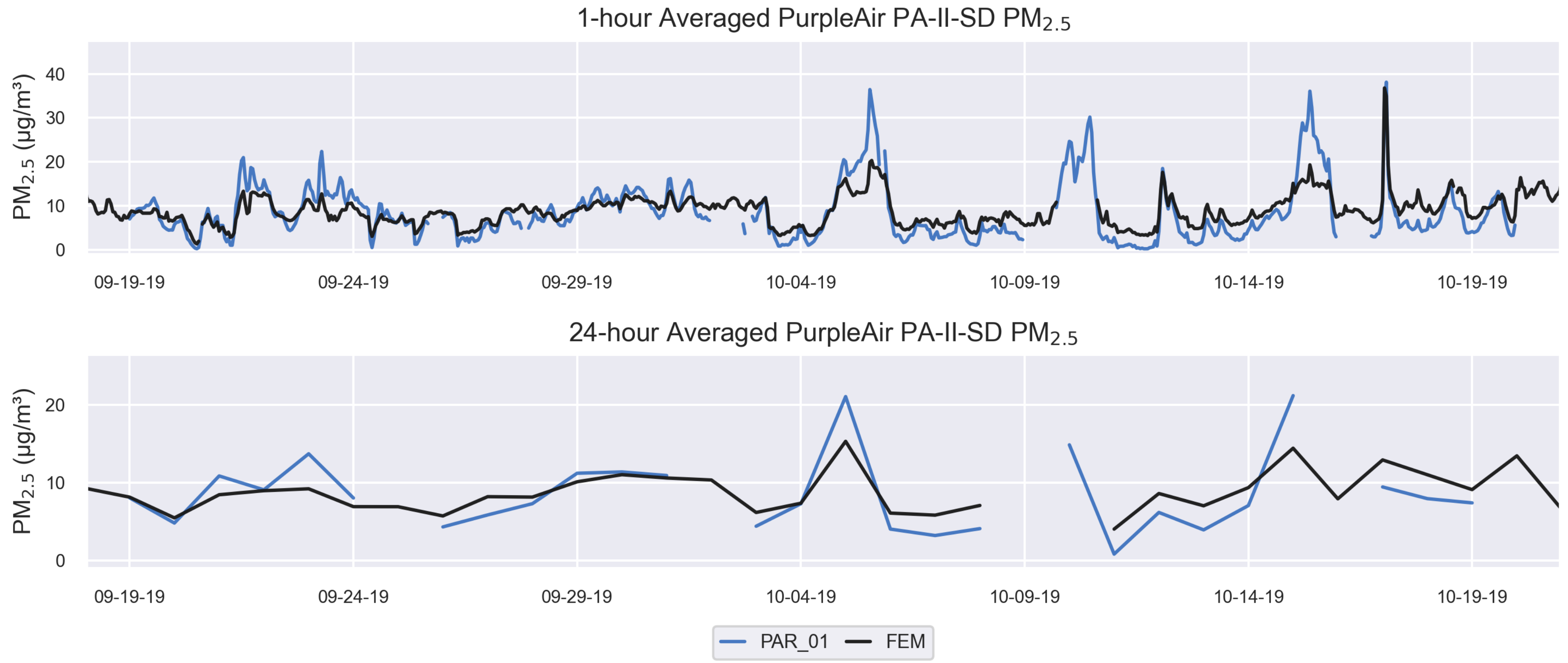

Scatter Plots: Comparison to FRM/FEM

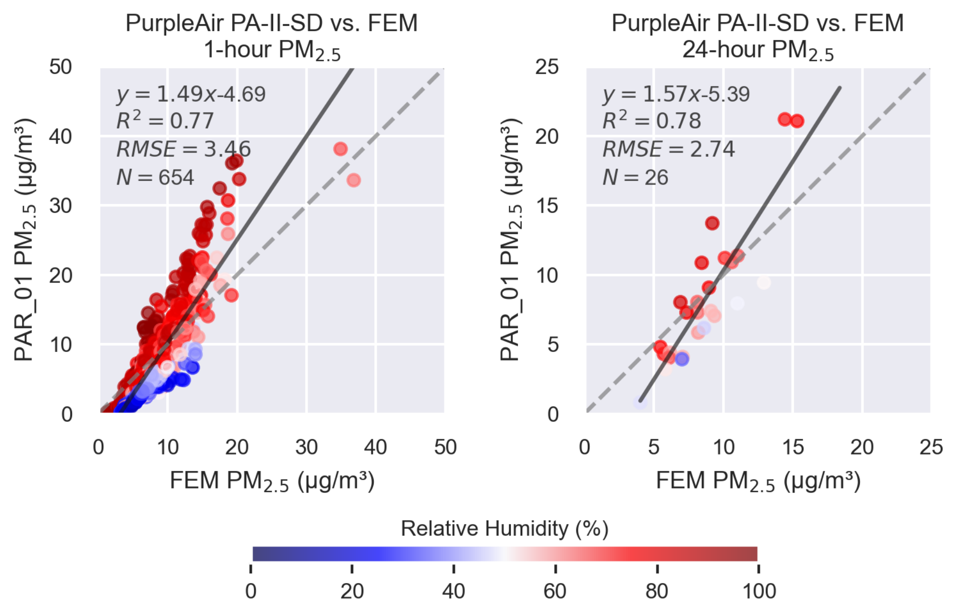

Range of FRM/FEM concentrations over duration of base test (µg/m³)	1.3-36.8 (1-hr), 4.0-15.3 (24-hr)
Number of 24-hr periods in FRM/FEM monitor measurements with a goal concentration ≥ 25 µg/m³	0

Performance Metrics

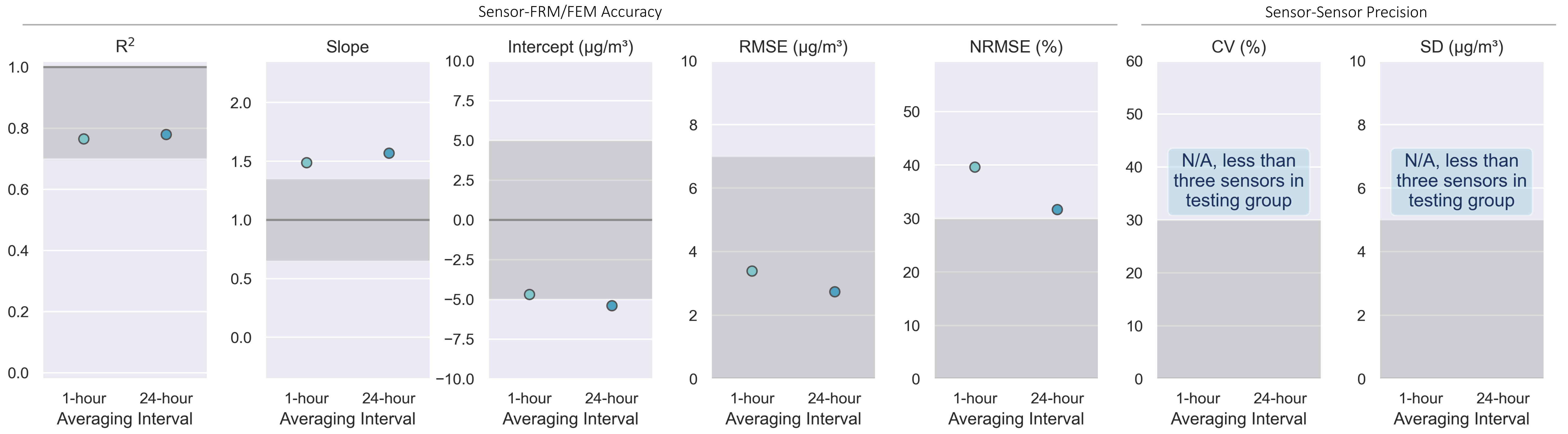

Meteorological Conditions During Deployment

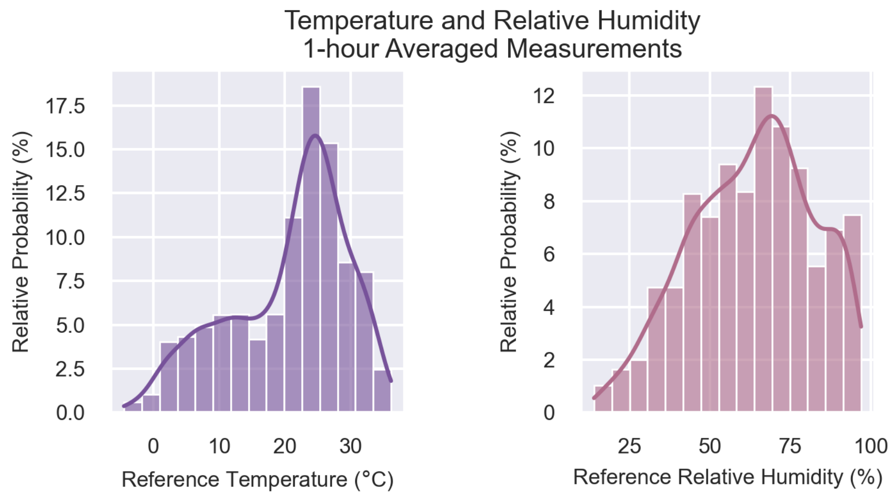

Number of 24-hr periods outside sensor manufacturer-listed temperature operational range (no operational range specified)	-
Number of 24-hr periods outside sensor manufacturer-listed relative humidity operational range (no operational range specified)	-

Meteorological Influence

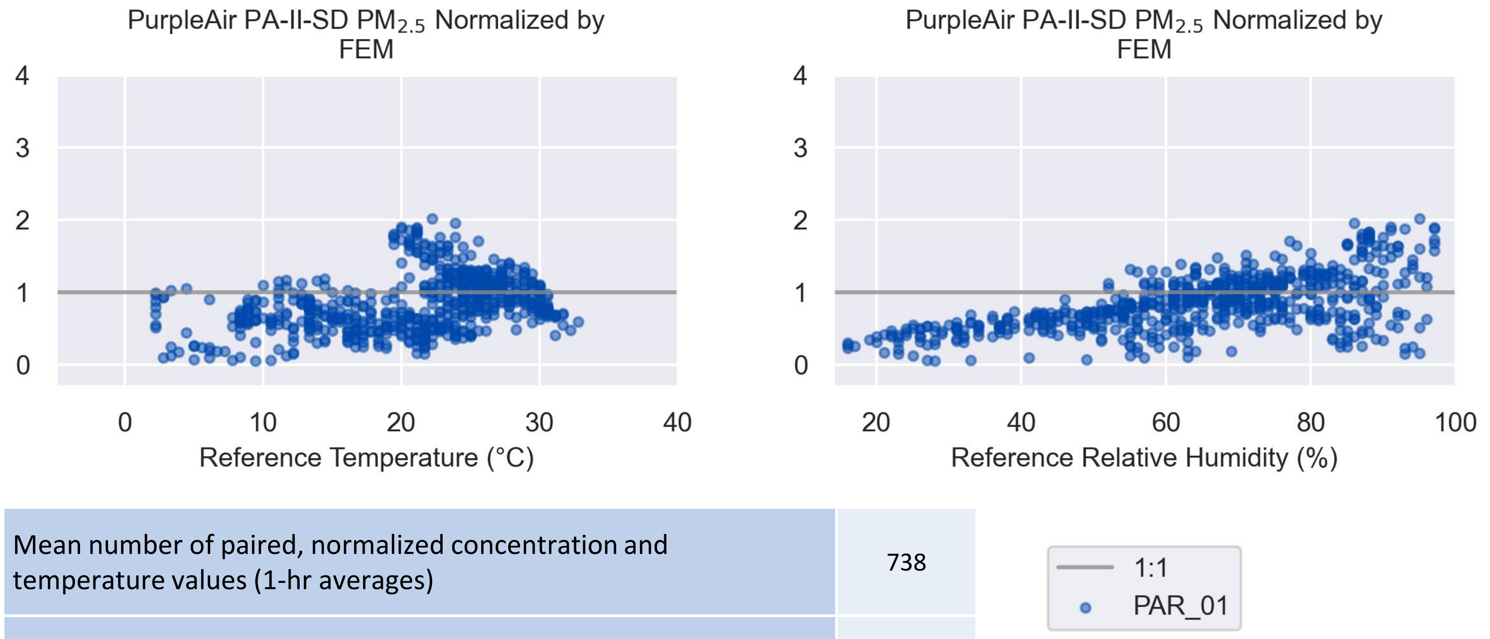

Mean number of paired, normalized concentration and temperature values (1-hr averages)	738
Mean number of paired, normalized concentration and relative humidity values (1-hr averages)	738

Testing Report - $PM_{2.5}$ Base Testing

PurpleAir PA-II-SD

This report reflects out-of-the-box performance

Initial Base Testing - Edmond, OK

U.S. Environmental Protection Agency
Office of Research and Development
PI: Clements.Andrea@epa.gov
919-541-1363
September 2019—October 2019

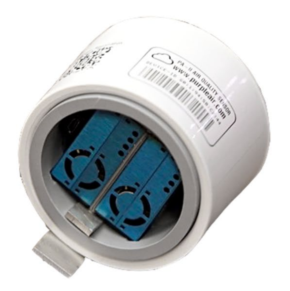

Tabular Statistics

Sensor-FRM/FEM Correlation

	Bias and Linearity						Data Quality			
	R^2		Slope		Intercept (µg/m³)		Uptime (%)		Number of paired sensor and FRM/FEM concentration values	
	1-Hour ●	24-Hour ●	1-Hour ○	24-Hour ○	1-Hour ●	24-Hour ○	1-Hour ●	24-Hour ●	1-Hour	24-Hour
Metric Target Range	≥ 0.70	≥ 0.70	1.0 ± 0.35	1.0 ± 0.35	-5 ≤ b ≤ 5	-5 ≤ b ≤ 5	75%*	75%*	-	-
Sensor PAR_01	0.77	0.78	1.49	1.57	-4.69	-5.39	92	87	654	26

	Error			
	RMSE (µg/m³)		NRMSE (%)	
	1-Hour ★	24-Hour ★	1-Hour ☆	24-Hour ☆
Metric Target Range	≤ 7.0	≤ 7.0	≤ 30.0	≤ 30.0
Deployment Value	3.4	2.7	39.6	31.7

Device-specific metrics (computed for each sensor in evaluation)
○○○ Metric value for none of devices tested falls within the target range
●○○ Metric value for one of devices tested falls within the target range
●●○ Metric value for two of devices tested falls within the target range
●●● Metric value for three of devices tested falls within the target range

Single-valued metrics (computed via entire evaluation dataset)
☆ Indicates that the metric value is not within the target range
★ Indicates that the metric value is within the target range

Sensor-Sensor Precision[1]

	Precision (between collocated sensors)				Data Quality	
	CV (%)		SD (µg/m³)		Number of concurrent sensor concentration pairs	
	1-Hour ☆	24-Hour ☆	1-Hour ☆	24-Hour ☆	1-Hour	24-Hour
Metric Target Range	≤ 30.0	≤ 30.0	≤ 5.0	≤ 5.0	-	-
Deployment Value	-	-	-	-	-	-

[1]Precision statistics are computed for evaluations with at least three collocated sensor units. Metric values are left blank for evaluations with two or fewer sensor units.

*This value is only a recommendation for ensuring data quality and is not included in the list of target values discussed in Section 4 of the Performance Testing Protocols, Metrics, and Target Values for Fine Particulate Matter Air Sensors document.

Testing Report - $PM_{2.5}$ Base Testing

PurpleAir PA-II-SD

This report reflects out-of-the-box performance

Initial Base Testing - Edmond, OK
U.S. Environmental Protection Agency
Office of Research and Development
PI: Clements.Andrea@epa.gov
919-541-1363
September 2019—October 2019

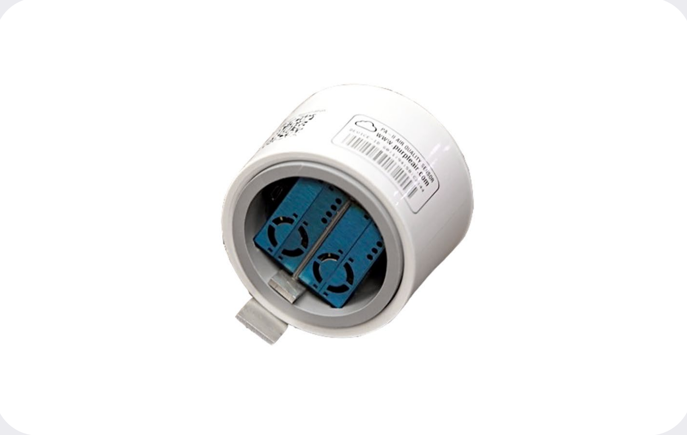

Sensor-FRM/FEM Scatter Plots

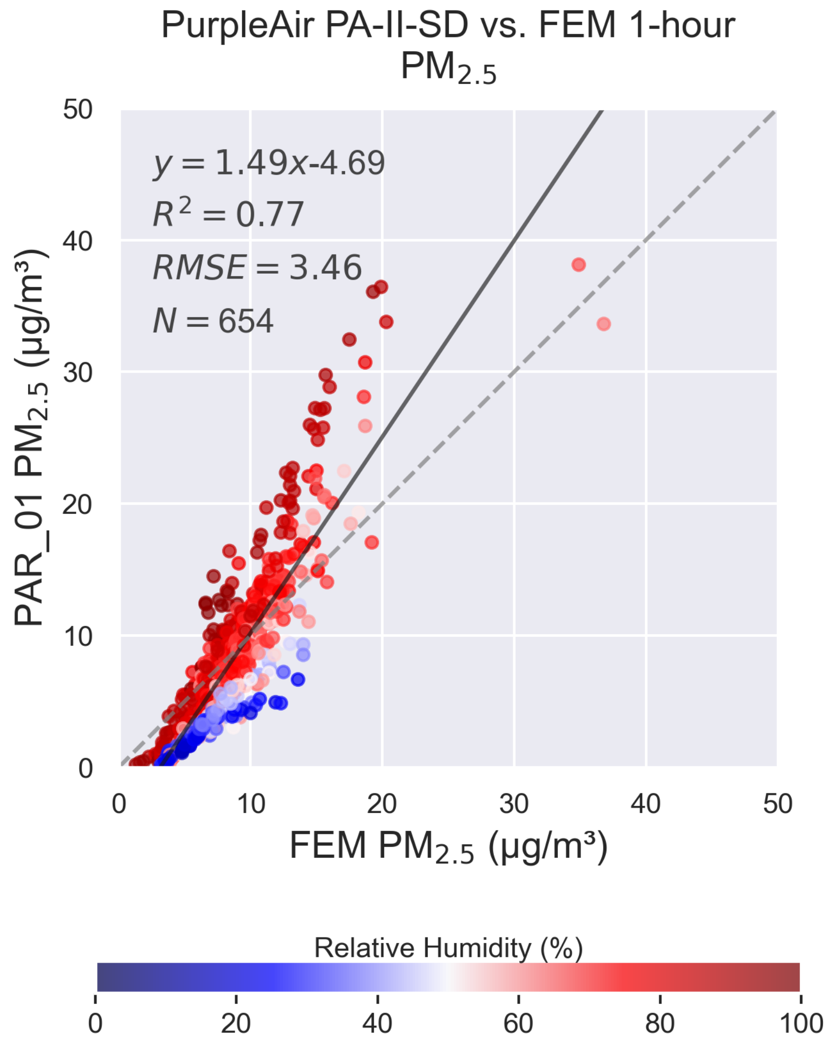

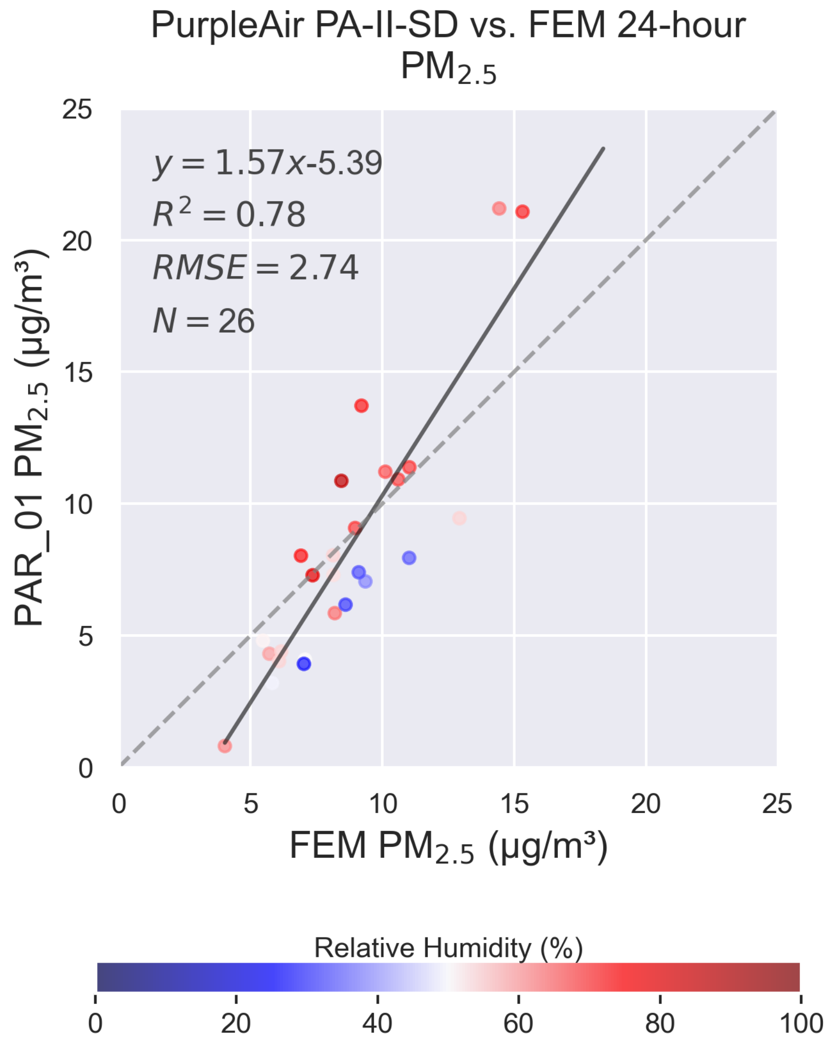

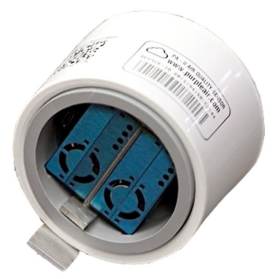

Supplemental Information

Abbreviations used in Supplemental Information

FRM	Federal Reference Method
FEM	Federal Equivalent Method
SOP	Standard Operating Procedure
QAPP	Quality Assurance Project Plan
QC	Quality Control

Supplemental Documentation	Attached	Description & URL or file path to documentation
Field observations and sensor data flags	☑	See OK-PAR-Page 7 of this testing report
Maintenance logs	☐	No logs recorded during testing
Standard operating procedure(s)	☐	U.S. EPA Office Of Research and Development SOP available upon request
Photos of equipment setup and testing	☑	See OK-PAR-Page 5 of this testing report
Product specifications sheet(s)	☑	PurpleAir: PA-II-SD Product website
Product manual(s)	☐	N/A
Data storage and transmission method	☑	See OK-PAR-Page 6 of this testing report
Data correction approach	☑	See OK-PAR-Page 6 of this testing report
Issues encountered	☑	See OK-PAR-Page 6 of this testing report
Data analysis/correction scripts and version	☑	Averaging and processing of data, calculation of performance metrics, and generation of figures and other supplementary material for analysis were obtained using Python 3.9.7 with the packages sensortoolkit v0.8.3b2, pandas 1.3.5, NumPy 1.21.2, Matplotlib 3.5.0, statsmodels 0.13.0, and seaborn 0.11.2. All packages are available from the Python Package Index (PyPI) at https://pypi.org. The integrated development environment (IDE) Spyder 5.1.5 was used for scripting and data visualization. Version control for the Python base, packages, and IDE were all managed by conda 4.11.0.
Air Monitoring Station QAPP	☐	U.S. EPA Office Of Research and Development QAPP available upon request
Summary of FRM/FEM monitor QC checks	☑	See OK-PAR-Page 8 of this testing report
Manufacturer website for FRM/FEM monitor	☑	Teledyne API: T640/T640X Product website
FRM/FEM monitor manual	☑	Teledyne API: T640/T640X Product Manual
FRM/FEM monitor specifications sheet(s)	☑	Teledyne API: T640/T640X Specification Sheet
Other documents	☐	

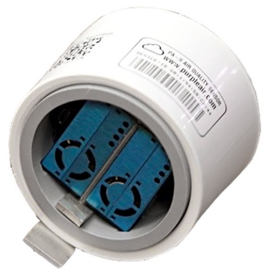

Supplemental Information: Photos of Testing Site and Equipment Setup

Site Description:

The Oklahoma Christian University monitoring site was established in the late 1970s. There is a containerized building that houses the continuous instruments with a wooden platform directly adjacent for the filter-based instruments. Pollutants monitored include O_3, PM_{10} (filter based and continuous), $PM_{2.5}$ (filter based and continuous), $NO/NO_2/NO_x$, CO, SO_2 and meteorology. The site has solar panels to help defer the cost of power to the site.

Figure 1: PurpleAir PA-II-SD (indicated by red arrow) attached to metal railing atop the sampling shelter at the monitoring site.

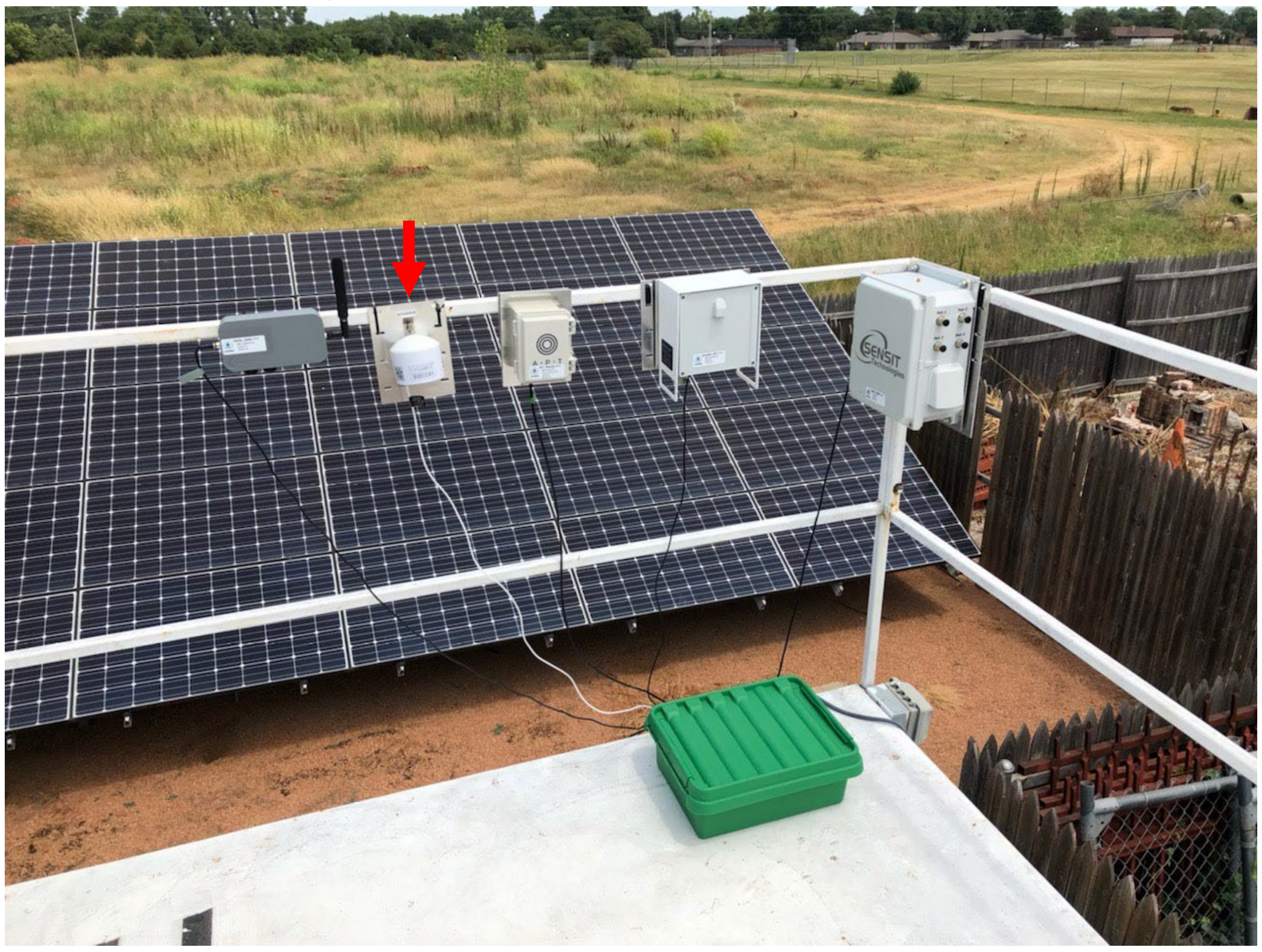

Figure 2: OK Monitoring Station sampling shelter

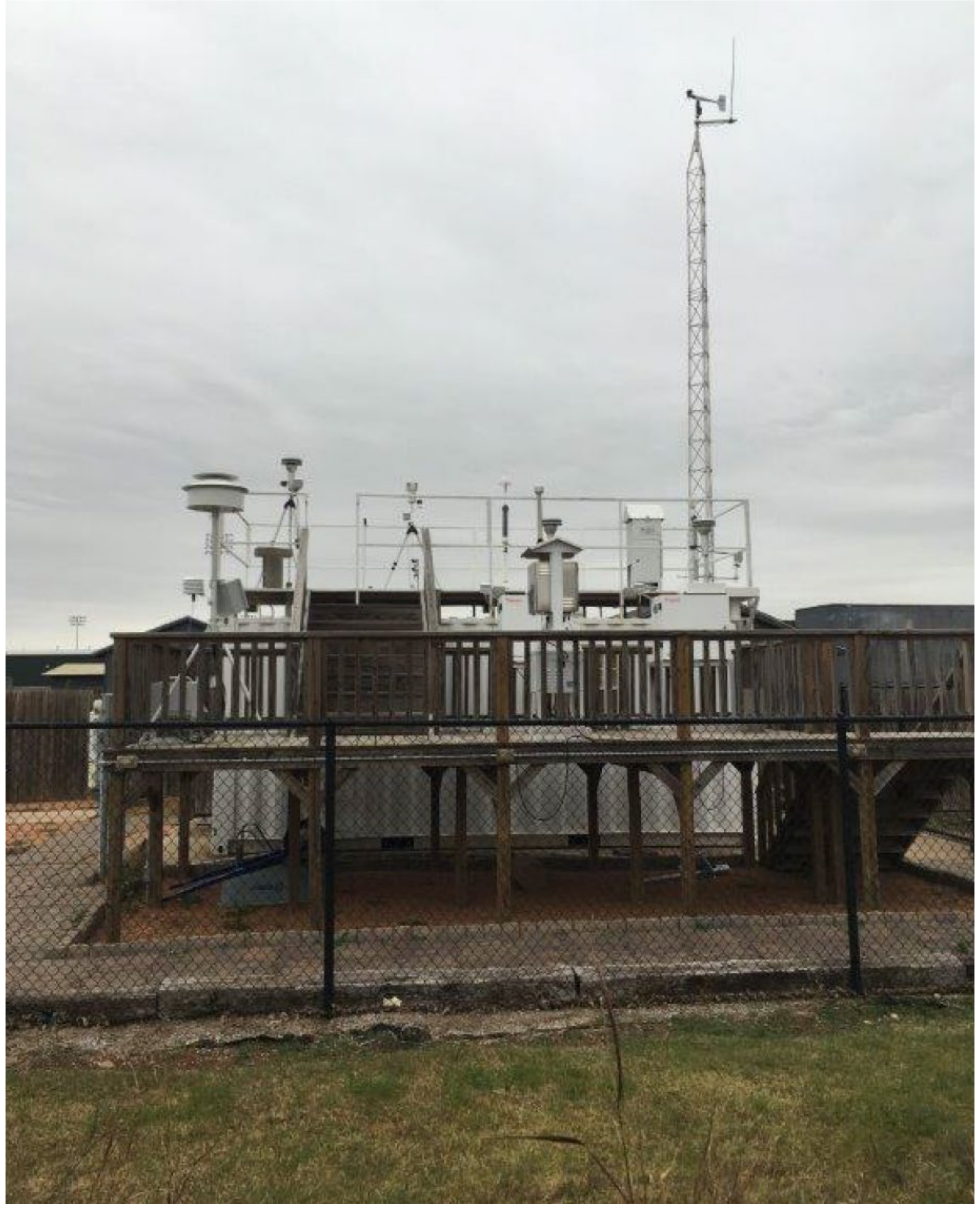

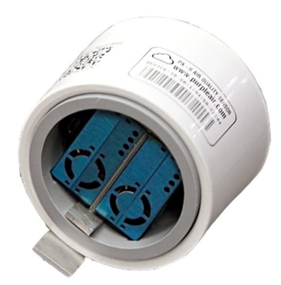

Supplemental Information: Data Storage, Correction Approach, and Issues Encountered

Data Storage and Transmission Method

The PurpleAir PA-II-SD records data at a 2-minute[1] sampling interval. Data is transmitted to the PurpleAir Cloud via Wi-Fi and stored on an internal MicroSD card. Due to the differing file structure between remote and direct download methods and the lag associated with cloud data download, weekly raw data were obtained primarily via onboard SD card. Each field site operator was provided two labeled MicroSD cards for sensor units that they used to swap out each week. Data from the collected card was then read and processed off-site.

Data Correction Approach

Data sets recorded by PurpleAir PA-II-SD contain two correction factors for PM concentrations, referred to internally as "CF=ATM" and "CF=1". $PM_{2.5}$ concentrations are recorded concurrently for each correction factor, and corresponding concentrations agree closely up to approximately 30 µg/m^3, above which one correction factor reflects concentrations roughly 1.5 times concentrations for the other correction factor. During a separate analysis by ORD, concentrations for the higher correction factor were determined to have slightly higher correlation with collocated reference measurements. As a result, data for the higher correction factor were used for this study. Prior to firmware version 5.00 (released on 11/30/2019), headers for concentrations corresponding to each correction factor were incorrectly labeled (i.e., CF=1 was labeled CF=ATM and vice versa). Testing indicated in this report was completed prior to this firmware update, and thus data sets acquired for PA-II-SD units had swapped $PM_{2.5}$ CF labels. Recorded PA-II-SD data sets indicate that the CF=ATM correction factor was the higher CF as labeled (following firmware update 5.00, this data channel corresponds to CF=1) and sensor data shown in this report correspond to this correction factor.

After acquisition, the raw data was processed using the *sensortoolkit* python code library (v0.8.3b2). A continuous data set at the recorded sampling frequency was written to a .csv file. 1-hour and 24-hour averaged data sets were generated using a 75% completeness threshold and saved as separate .csv files. Simultaneous measurement from the PurpleAir PA-II's dual PMS5003 sensors (labeled channels A and B) allows for identification of outliers resulting in large-scale disagreement between recorded values. The outlier removal method of Barkjohn et al. 2021[2] was applied to both 1-hour and 24-hour averaged A and B measurement pairs. This method includes thresholds for the absolute difference and the percent difference between hourly channel values. A-B channel averages were not included for hours where the absolute difference between channel values was greater than 5 µg/m^3 and the percent difference exceeded 70%. This outlier removal method is in use on the AirNow Fire and Smoke map and has been used for this evaluation since it requires no external information (e.g., data from the monitor) and no assumptions about typical PM2.5 concentrations (e.g., removing data over a certain threshold concentration).

Issues Encountered

Pre-deployment observations

- Prior to the testing period indicated in this report, the PurpleAir PA-II-SD unit was briefly collocated for a period of approximately one week at EPA's Ambient Monitoring Innovative Research Station (AIRS) in Research Triangle Park, NC. The unit was tested for proper operation, including powering on and data logging (either to the onboard SD card or via hotspot Wi-Fi connection to the PurpleAir Cloud), sampling, and acquisition of recorded data from either onboard memory or cloud storage. Units that did not pass this pre-deployment testing were returned to the manufacturer and constituted approximately 1 in 10 units tested for subsequent performance evaluation.

Field observations and sensor data flags are included on the following page

[1]As of version 4.02, updated across devices on 5/31/2019. Prior to this firmware version, devices recorded measurements at 80-second intervals
[2]Barkjohn, K. K., Gantt, B., and Clements, A. L.: Development and application of a United States-wide correction for PM2.5 data collected with the PurpleAir sensor, Atmos. Meas. Tech., 14, 4617–4637, https://doi.org/10.5194/amt-14-4617-2021, 2021.

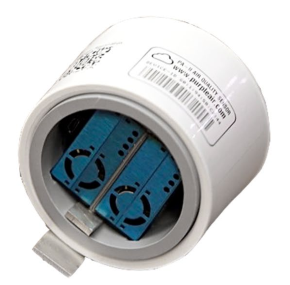

<u>Field observations and sensor data flags</u>

The following table contains data flags describing events that were encountered during the testing period. The PurpleAir PA-II-SD unit was deployed at the OK monitoring site on 8/1/2019. During the testing period, the PurpleAir was configured to record measurements at 120-second intervals. Occasionally, the sampling interval would be doubled to 240 seconds. These longer sampling intervals would occasionally occur on a roughly hourly basis; however, the occurrence of these longer intervals was highly unpredictable, and no discernible pattern was observed. Additionally, even longer sampling interval durations ranging from approximately 5 to 10 minutes would be logged and are indicated in the table below. As with sampling interval variation discussed subsequently, no discernible pattern in the frequency or occurrence of these longer sampling intervals is apparent from PA-II-SD data sets.

Start Time (UTC)	End Time (UTC)	Sensor Serial ID	Parameters Impacted	Flag
2019-09-28 00:03:00+00:00	2019-09-28 00:15:00+00:00	PAR_01	ALL	6.0-Sampling interval abnormality
2019-10-10 20:08:00+00:00	2019-10-10 20:18:00+00:00	PAR_01	ALL	6.0-Sampling interval abnormality

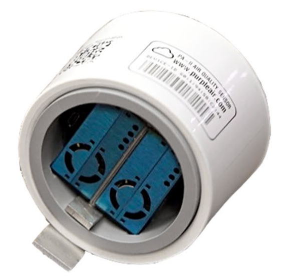

Description of Data Flags

AQS

The U.S. EPA's Air Quality System (AQS) is the Agency's primary ambient air monitoring data archive. A comprehensive list of data flags that are recorded alongside AQS data sets, referred to by U.S. EPA as 'qualifiers', can be found at the following link: https://aqs.epa.gov/aqsweb/documents/codetables/qualifiers.html

Invalidation of reference data: AQS qualifiers are organized by qualifier type, which indicates whether data logged alongside qualifier flags should be invalidated (set null). Qualifiers with type "Null Data Qualifier" are invalidated, and includes data logged during periods that coincide with QC checks (e.g., "BF-Precision/Zero/Span", "BJ- Operator Error", "BL - QA Audit", "AZ - QC Audit") among other events such as power outages. Data logged alongside qualifiers with type "Quality Assurance Qualifiers" are not invalidated and are included in this analysis (e.g., concentrations less than the federal MDL for the reference monitor "MD – Value less than MDL", QA reviewed values "Validated Value").

Data Flags Recorded During Testing

FRM/FEM Monitor	Timestamp (UTC)	Flag
Teledyne API T640 (Data acquired via AQS)	2019-09-20 14:00:00+0000	AZ - QC Audit
	2019-10-09 18:00:00+0000 to 2019-10-10 14:00:00+0000	AN - Machine Malfunction
	2019-10-18 15:00:00+0000	AZ - QC Audit

Meteorological Instrument	Timestamp (UTC)	Flag
RM Young 41382V Temperature Monitor (Data acquired via AQS)	2019-10-03 14:00:00+0000 to 2019-10-03 19:00:00+0000	BA - Maintenance/Routine Repairs
RM Young 41382V Relative Humidity Monitor (Data acquired via AQS)	2019-10-03 14:00:00+0000 to 2019-10-03 19:00:00+0000	BA - Maintenance/Routine Repairs

Testing Report - PM$_{2.5}$ Base Testing

SENSIT RAMP

This report reflects out-of-the-box performance

Initial Base Testing - Edmond, OK

U.S. Environmental Protection Agency
Office of Research and Development
PI: Clements.Andrea@epa.gov
919-541-1363

August 2019—September 2019

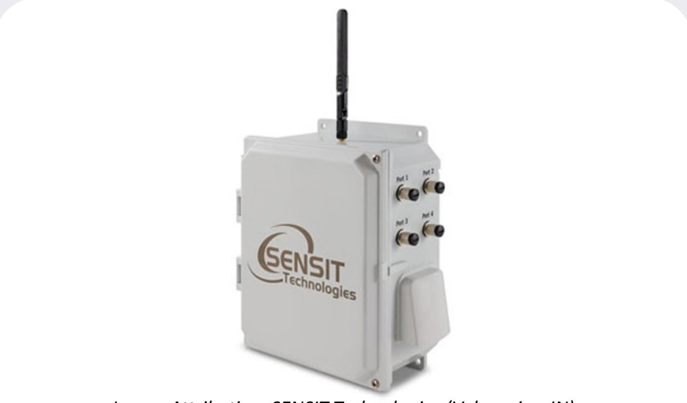

Image Attribution: SENSIT Technologies (Valparaiso, IN)

Deployment Details

Testing Organization and Site Information	
Testing organization (Name, Organization type, Contact website)	U.S. Environmental Protection Agency - Office of Research and Development<br>Federal Government<br>Air Sensor Toolbox | U.S. EPA Website
Testing location (City, State, Latitude and Longitude)	OK Christian University<br>Edmond, OK<br>35.614131, -97.475083
AQS site ID	40 - 109 - 1037
Sampling timeframe (MM-DD-YY)	08-15-19 to 09-14-19
Sensor data source	Onboard MicroSD card
Reference data source	AQS API download

Sensor Information			
Manufacturer, model	SENSIT RAMP		
Device firmware version	190313_AQ_v9.30		
Sampling time interval	15-seconds		
Sensor serial numbers	RAM_01		
Issues encountered during deployment?	☑	See OK-RAM-Page 6 of this testing report	

FRM/FEM Information	
Manufacturer, model, designation	Teledyne Advanced Pollution Instrumentation T640 FEM
Sampling time interval	1-hour averaging
Date of calibration	As required by 40 CFR Part 58 and the QAPP for Ambient Air Monitoring in the State of Oklahoma maintained by ODEQ/AQD
Date of flowrate verification check	Monthly as required by 40 CFR Part 58 Appendix A
Description, date(s) of maintenance activities	N/A

Time Series Plots

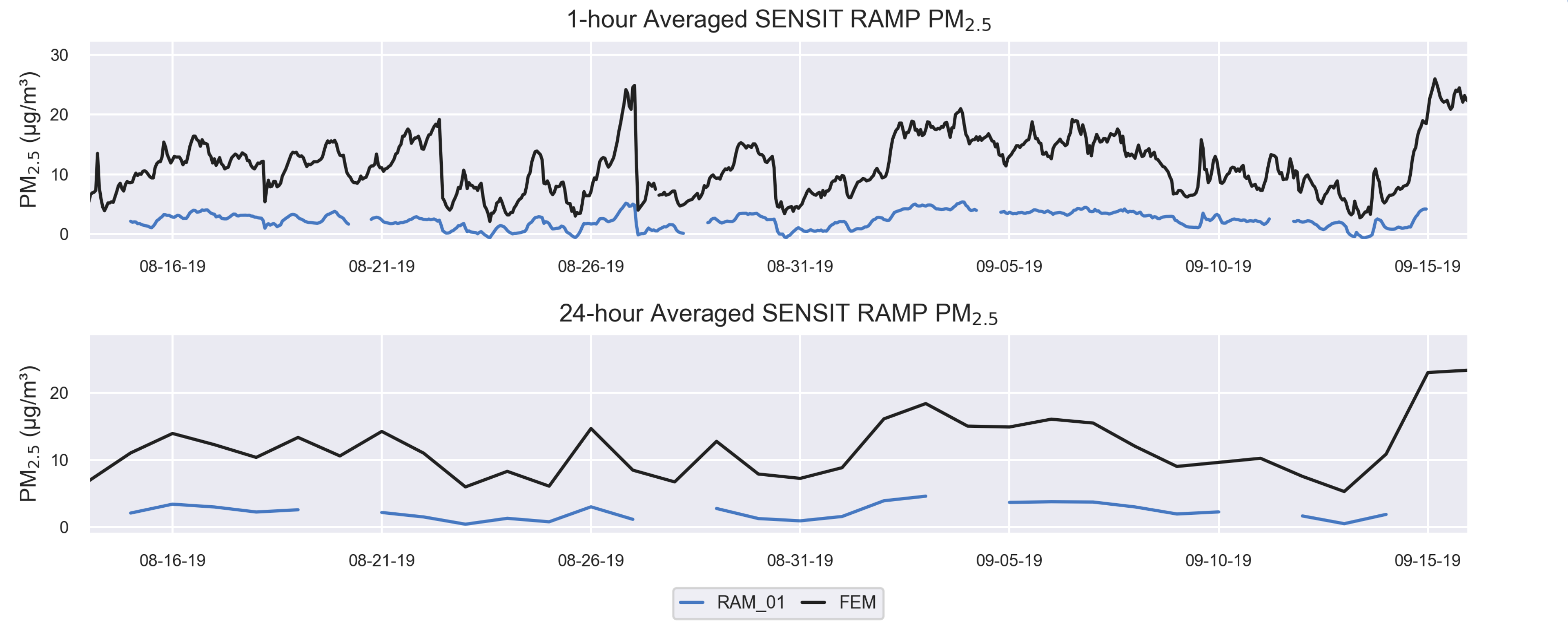

Scatter Plots: Comparison to FRM/FEM

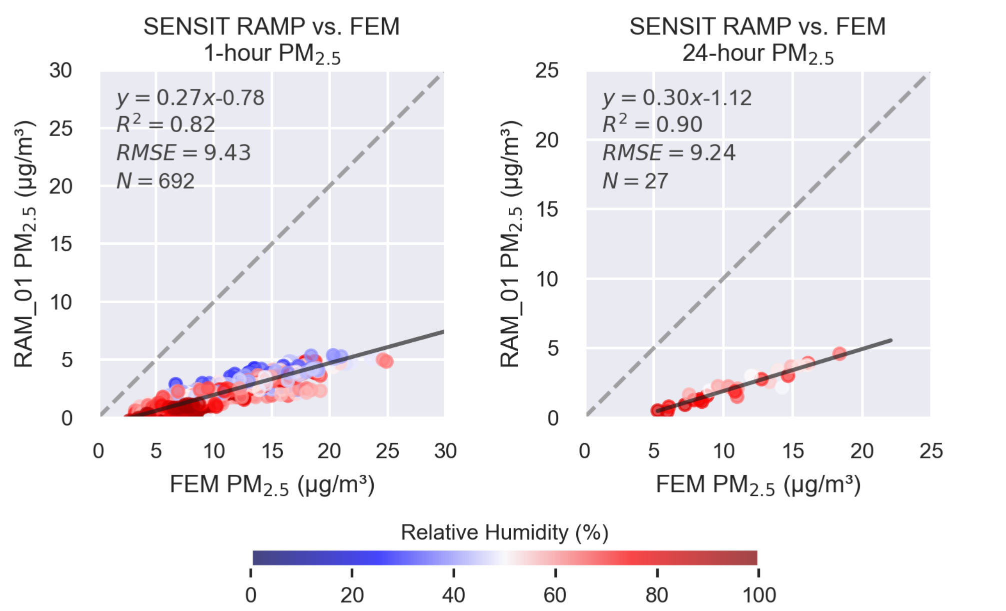

Range of FRM/FEM concentrations over duration of base test (µg/m³)	2.1-24.9 (1-hr),<br>5.3-18.4 (24-hr)
Number of 24-hr periods in FRM/FEM monitor measurements with a goal concentration ≥ 25 µg/m³	0

Performance Metrics

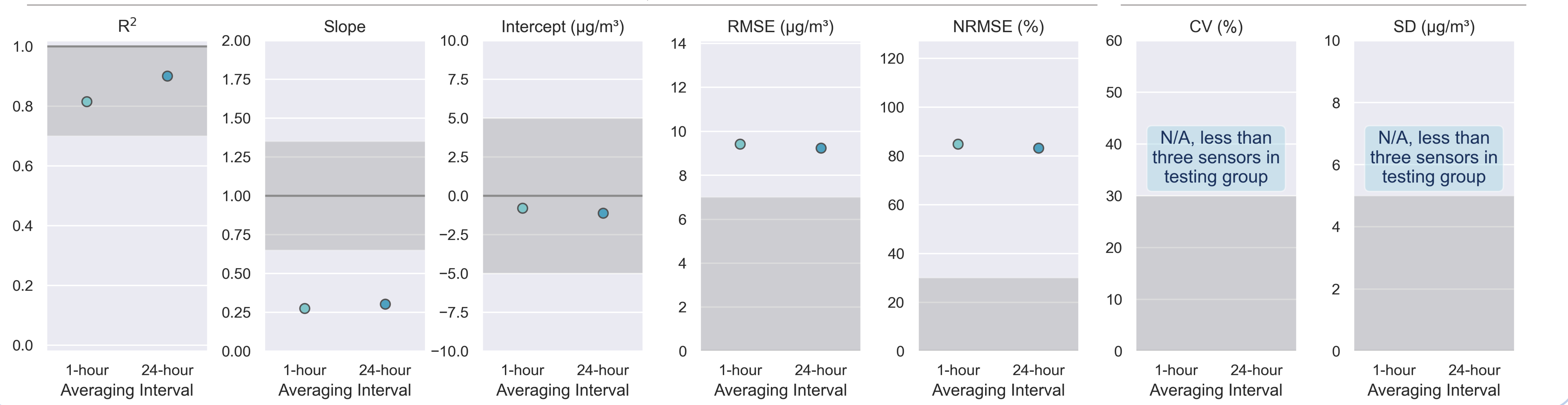

Meteorological Conditions During Deployment

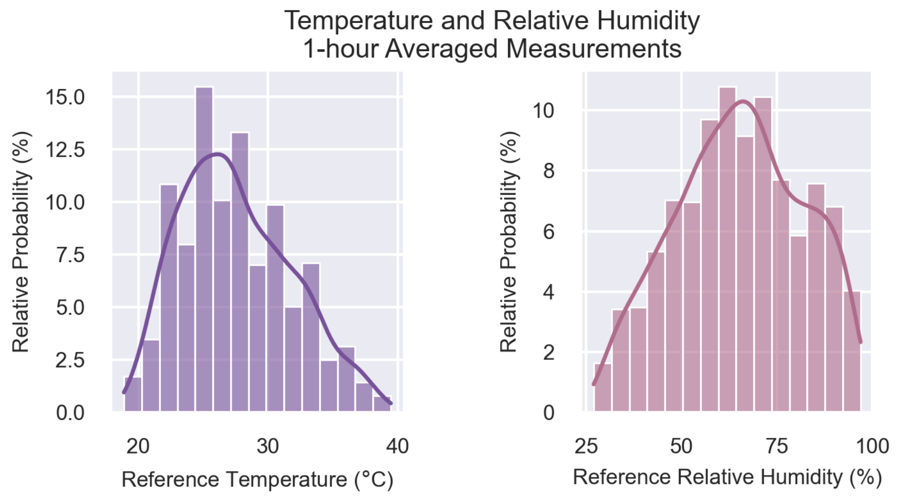

Number of 24-hr periods outside sensor manufacture-listed temperature operational range (-20 to 50 °C)	0
Number of 24-hr periods outside sensor manufacture-listed relative humidity operational range (no operational range specified)	-

Meteorological Influence

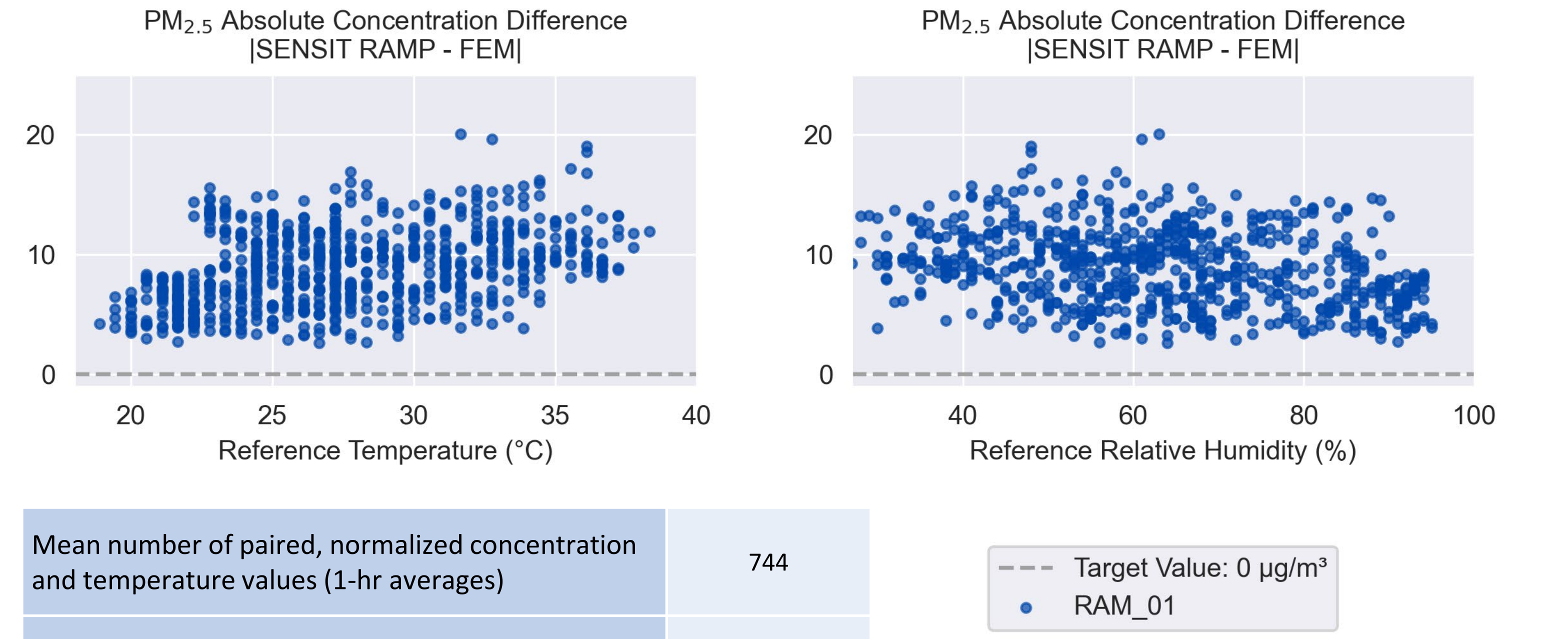

Mean number of paired, normalized concentration and temperature values (1-hr averages)	744
Mean number of paired, normalized concentration and relative humidity values (1-hr averages)	744

Testing Report - $PM_{2.5}$ Base Testing

SENSIT RAMP

This report reflects out-of-the-box performance

Initial Base Testing - Edmond, OK

U.S. Environmental Protection Agency
Office of Research and Development
PI: Clements.Andrea@epa.gov
919-541-1363

August 2019—September 2019

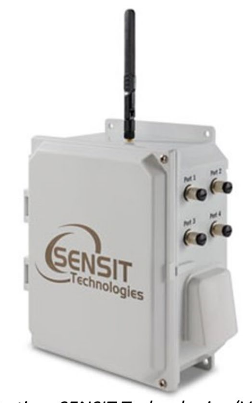

Image Attribution: SENSIT Technologies (Valparaiso, IN)

Tabular Statistics

Sensor-FRM/FEM Correlation

	Bias and Linearity						Data Quality			
	R^2		Slope		Intercept (µg/m³)		Uptime (%)		Number of paired sensor and FRM/FEM concentration values	
	1-Hour ●	24-Hour ●	1-Hour ○	24-Hour ○	1-Hour ●	24-Hour ●	1-Hour ●	24-Hour ●	1-Hour	24-Hour
Metric Target Range	≥ 0.70	≥ 0.70	1.0 ± 0.35	1.0 ± 0.35	-5 ≤ b ≤ 5	-5 ≤ b ≤ 5	75%*	75%*	-	-
Sensor RAM_01	0.82	0.90	0.27	0.30	-0.78	-1.12	93	87	692	27

	Error			
	RMSE (µg/m³)		NRMSE (%)	
	1-Hour ☆	24-Hour ☆	1-Hour ☆	24-Hour ☆
Metric Target Range	≤ 7.0	≤ 7.0	≤ 30.0	≤ 30.0
Deployment Value	9.4	9.2	84.9	83.3

Device-specific metrics (computed for each sensor in evaluation)
○○○ Metric value for none of devices tested falls within the target range
●○○ Metric value for one of devices tested falls within the target range
●●○ Metric value for two of devices tested falls within the target range
●●● Metric value for three of devices tested falls within the target range

Single-valued metrics (computed via entire evaluation dataset)
☆ Indicates that the metric value is not within the target range
★ Indicates that the metric value is within the target range

Sensor-Sensor Precision[1]

	Precision (between collocated sensors)				Data Quality	
	CV (%)		SD (µg/m³)		Number of concurrent sensor concentration pairs	
	1-Hour ☆	24-Hour ☆	1-Hour ☆	24-Hour ☆	1-Hour	24-Hour
Metric Target Range	≤ 30.0	≤ 30.0	≤ 5.0	≤ 5.0	-	-
Deployment Value	-	-	-	-	-	-

[1]Precision statistics are computed for evaluations with at least three collocated sensor units. Metric values are left blank for evaluations with two or fewer sensor units.

*This value is only a recommendation for ensuring data quality and is not included in the list of target values discussed in Section 4 of the Performance Testing Protocols, Metrics, and Target Values for Fine Particulate Matter Air Sensors document.

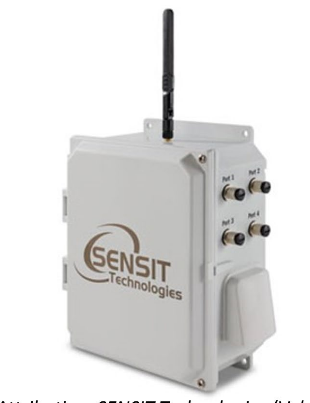

Image Attribution: SENSIT Technologies (Valparaiso, IN)

Sensor-FRM/FEM Scatter Plots

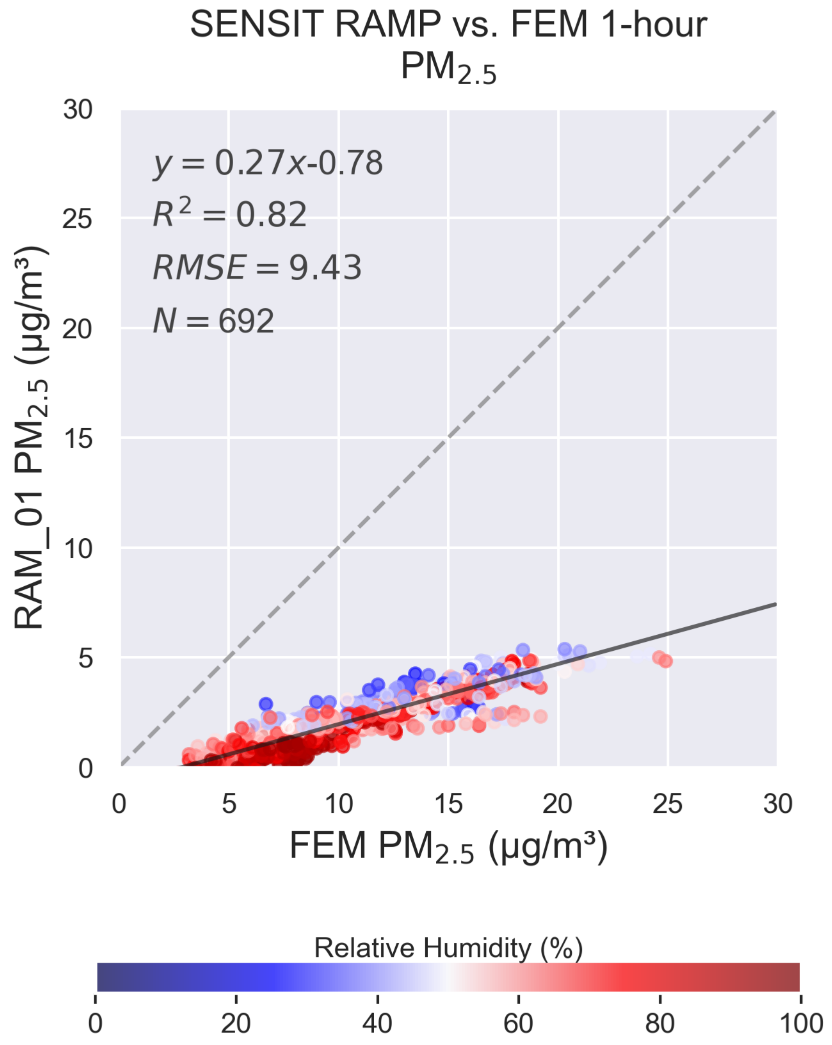

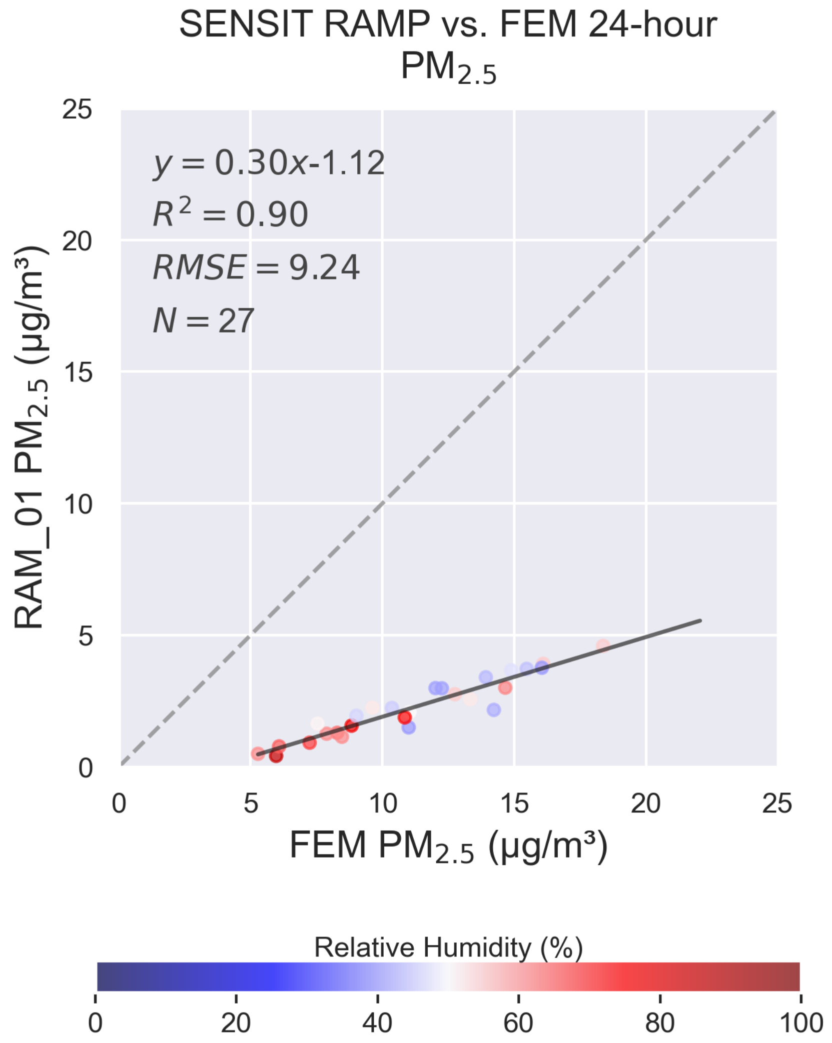

Testing Report - $PM_{2.5}$ Base Testing

SENSIT RAMP

This report reflects out-of-the-box performance

Initial Base Testing - Edmond, OK
U.S. Environmental Protection Agency
Office of Research and Development
PI: Clements.Andrea@epa.gov
919-541-1363
August 2019—September 2019

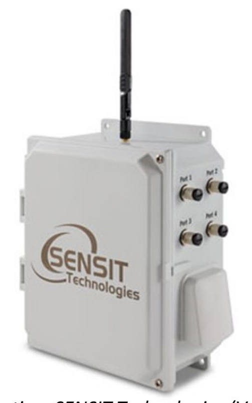

Image Attribution: SENSIT Technologies (Valparaiso, IN)

Supplemental Information

Abbreviations used in Supplemental Information

FRM	Federal Reference Method
FEM	Federal Equivalent Method
SOP	Standard Operating Procedure
QAPP	Quality Assurance Project Plan
QC	Quality Control

Supplemental Documentation	Attached	Description & URL or file path to documentation
Field observations and sensor data flags	☑	See OK-RAM-Page 6 of this testing report
Maintenance logs	☐	No logs recorded during testing
Standard operating procedure(s)	☐	U.S. EPA Office Of Research and Development SOP available upon request
Photos of equipment setup and testing	☑	See OK-RAM-Page 5 of this testing report
Product specifications sheet(s)	☑	SENSIT Technologies: RAMP Product Brochure
Product manual(s)	☑	SENSIT Technologies: RAMP Product Manual
Data storage and transmission method	☑	See OK-RAM-Page 6 of this testing report
Data correction approach	☑	See OK-RAM-Page 6 of this testing report
Issues encountered	☑	See OK-RAM-Page 6 of this testing report
Data analysis/correction scripts and version	☑	Averaging and processing of data, calculation of performance metrics, and generation of figures and other supplementary material for analysis were obtained using Python 3.9.7 with the packages sensortoolkit v0.8.3b2, pandas 1.3.5, NumPy 1.21.2, Matplotlib 3.5.0, statsmodels 0.13.0, and seaborn 0.11.2. All packages are available from the Python Package Index (PyPI) at https://pypi.org. The integrated development environment (IDE) Spyder 5.1.5 was used for scripting and data visualization. Version control for the Python base, packages, and IDE were all managed by conda 4.11.0.
Air Monitoring Station QAPP	☐	U.S. EPA Office Of Research and Development QAPP available upon request
Summary of FRM/FEM monitor QC checks	☑	See OK-RAM-Page 7 of this testing report
Manufacturer website for FRM/FEM monitor	☑	Teledyne API: T640/T640X Product website
FRM/FEM monitor manual	☑	Teledyne API: T640/T640X Product Manual
FRM/FEM monitor specifications sheet(s)	☑	Teledyne API: T640/T640X Specification Sheet
Other documents	☐	

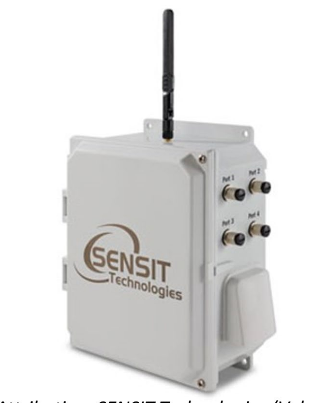

Image Attribution: SENSIT Technologies (Valparaiso, IN)

Supplemental Information: Photos of Testing Site and Equipment Setup

Site Description:

The Oklahoma Christian University monitoring site was established in the late 1970s. There is a containerized building that houses the continuous instruments with a wooden platform directly adjacent for the filter-based instruments. Pollutants monitored include O_3, PM_{10} (filter based and continuous), $PM_{2.5}$ (filter based and continuous), $NO/NO_2/NO_x$, CO, SO_2 and meteorology. The site has solar panels to help defer the cost of power to the site.

Figure 1: SENSIT RAMP (indicated by red arrow) attached to metal railing atop the sampling shelter at the monitoring site.

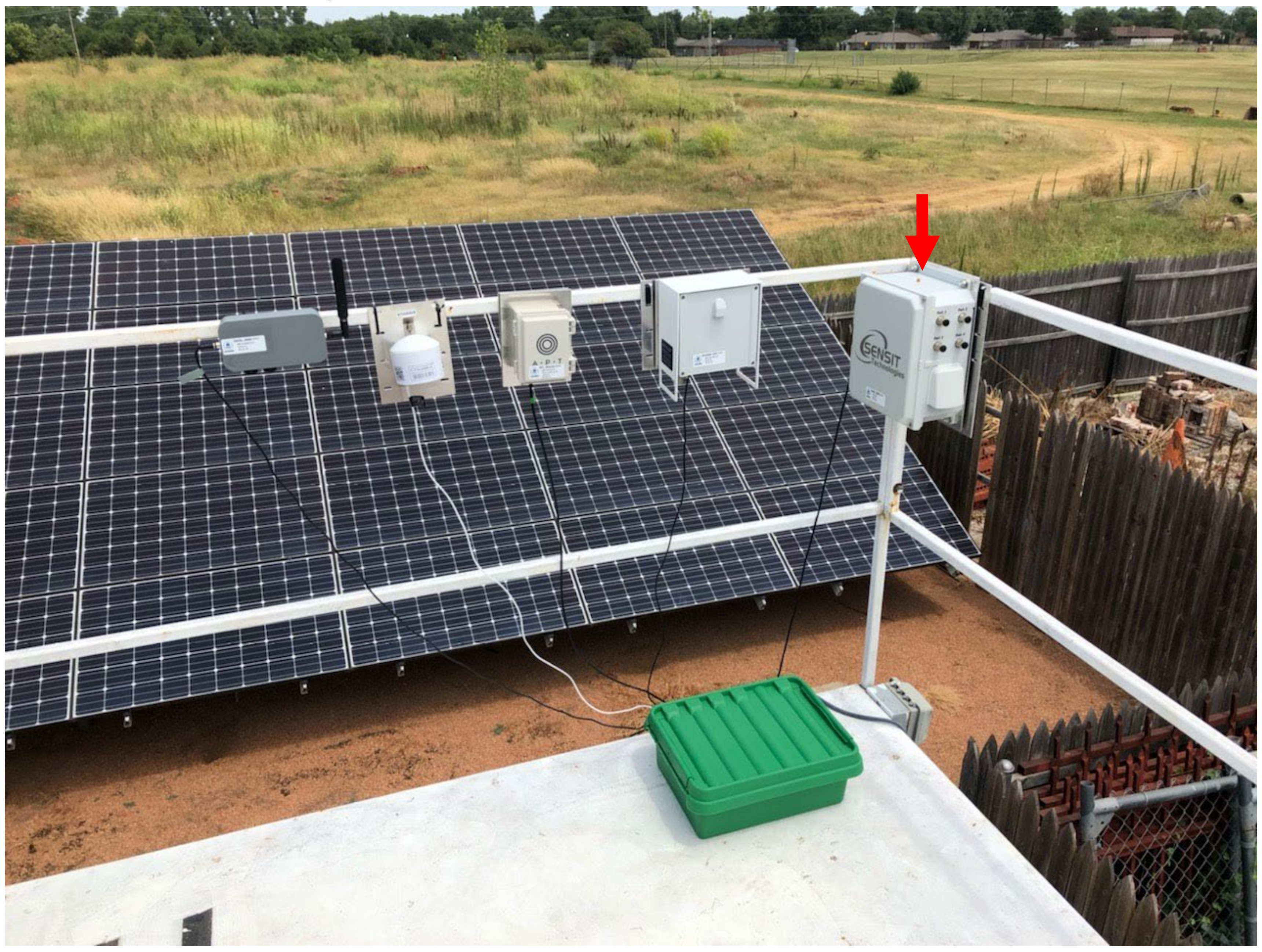

Figure 2: OK Monitoring Station sampling shelter

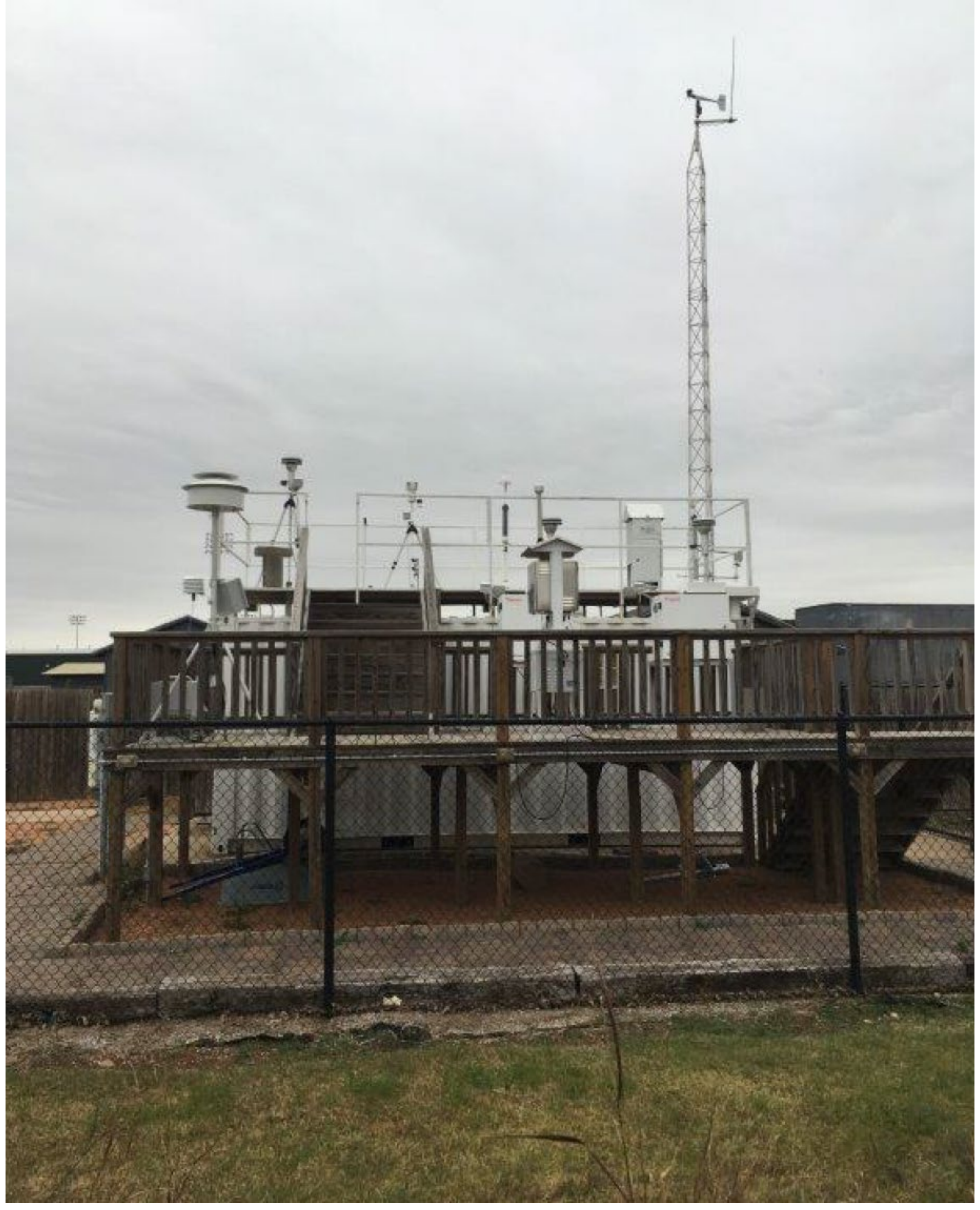

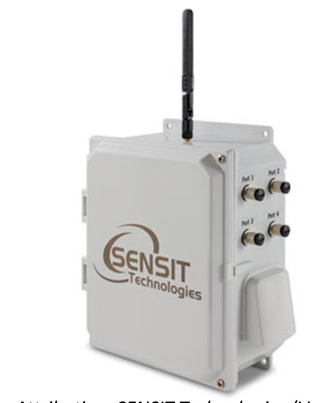

Image Attribution: SENSIT Technologies (Valparaiso, IN)

Supplemental Information: Data Storage, Correction Approach, and Issues Encountered

Data Storage and Transmission Method

The SENSIT RAMP was configured to record data at a 15-second sampling interval. Data are stored as daily text files (.txt format) on an onboard MicroSD card. Data files were obtained weekly via SD cards. Each field site operator was provided two labeled MicroSD cards for sensor units that they used to swap out each week. Data from the collected card was then read and processed off-site.

Data Correction Approach

SENSIT RAMP units were pre-configured by the manufacturer with a linear correction (i.e., concentration gain = 1.0 and offset = 0.0 μg m^{-3}). These presets reflect out-of-the box performance and were not modified by EPA prior or during testing.

After acquisition, the raw data was processed using the *sensortoolkit* python code library (v0.8.3b2). A continuous data set at the recorded sampling frequency was written to a .csv file. 1-hour and 24-hour averaged data sets were generated using a 75% completeness threshold and saved as separate .csv files. Outliers were NOT removed from data sets in order to assess "out-of-the-box" sensor performance.

Issues Encountered

Pre-deployment observations

- SENSIT RAMP units were received without documentation or manuals. After communicating the need to change default settings (logging interval and time zone) with the manufacturer, a draft user's manual and a USB cable were supplied. With the use of this USB cable, instrument settings could be changed, and real-time data could be logged using a serial communication software (CoolTerm, v.1.5.0). Because the sensor did not record data at the top of every minute, the RAMP was configured to record data at 15-second intervals so that the data could be averaged more closely to complete minutes. Prior to deployment, RAMP units were collocated in a bench-top evaluation to verify operational status and determine the extent of data invalidity (i.e., determine equilibration period) after an initial start-up event. The recorded response for parameters measured by the RAMP suggests that the gas sensors (CO, NO, NO_2, O_3) required approximately a 2-hour equilibration period, while the remaining sensors (temperature, relative humidity, particulate matter) did not require any equilibration period.

Field observations and sensor data flags

The SENSIT RAMP unit was deployed at the OK monitoring site on 8/1/2019. The RAMP unit operated nominally during the testing period and did not require replacement or repair.

The following table contains data flags describing events that were encountered during the testing period. Throughout the testing period, it was observed that data collected from the RAMP unit were recorded in Daylight Saving time (DST) instead of Central Standard Time (CST), which is the time zone in which the site is located. During each visit to the monitoring site, a flag was reported indicating this timestamp configuration. Timestamps were subsequently converted from Central Daylight Time to Coordinated Universal Time (UTC) for analysis.

Start Time (UTC)	End Time (UTC)	Sensor Serial ID	Parameters Impacted	Flag
2019-08-20 16:15:00+00:00	2019-08-28 16:10:00+00:00	RAM_01	DST	17.0-Date/time set in DST instead of LST
2019-08-28 16:10:00+00:00	2019-09-04 16:20:00+00:00	RAM_01	DST	17.0-Date/Time in Daylight Savings instead of Standard
2019-09-04 17:30:00+00:00	2019-09-11 17:25:00+00:00	RAM_01	DST	17.0-Date/Time in Daylight Savings instead of Standard
2019-09-11 17:31:00+00:00	2019-09-18 17:13:00+00:00	RAM_01	DST	17.0-Date/Time in Daylight Savings instead of Standard

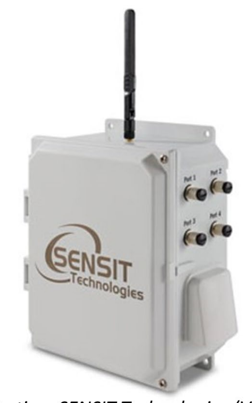

Image Attribution: SENSIT Technologies (Valparaiso, IN)

Description of Data Flags

AQS

The U.S. EPA's Air Quality System (AQS) is the Agency's primary ambient air monitoring data archive. A comprehensive list of data flags that are recorded alongside AQS data sets, referred to by U.S. EPA as 'qualifiers', can be found at the following link: https://aqs.epa.gov/aqsweb/documents/codetables/qualifiers.html

Invalidation of reference data: AQS qualifiers are organized by qualifier type, which indicates whether data logged alongside qualifier flags should be invalidated (set null). Qualifiers with type "Null Data Qualifier" are invalidated, and includes data logged during periods that coincide with QC checks (e.g., "BF-Precision/Zero/Span", "BJ- Operator Error", "BL - QA Audit", "AZ - QC Audit") among other events such as power outages. Data logged alongside qualifiers with type "Quality Assurance Qualifiers" are not invalidated and are included in this analysis (e.g., concentrations less than the federal MDL for the reference monitor "MD – Value less than MDL", QA reviewed values "Validated Value").

Data Flags Recorded During Testing

FRM/FEM Monitor	Timestamp (UTC)	Flag
Teledyne API T640<br>(Data acquired via AQS)	2019-08-27 14:00:00+0000	AZ - QC Audit

Testing Report - $PM_{2.5}$ Base Testing

QuantAQ ARISense

This report reflects out-of-the-box performance

Initial Base Testing - Edmond, OK
U.S. Environmental Protection Agency
Office of Research and Development
PI: Clements.Andrea@epa.gov
919-541-1363
April 2020—May 2020

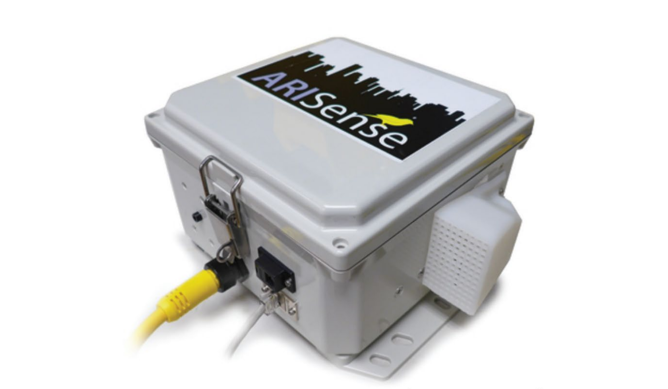
Image Attribution: QuantAQ, Inc (Somerville, MA)

Deployment Details

Testing Organization and Site Information	
Testing organization (Name, Organization type, Contact website)	U.S. Environmental Protection Agency - Office of Research and Development<br>Federal Government<br>Air Sensor Toolbox | U.S. EPA Website
Testing location (City, State, Latitude and Longitude)	OK Christian University<br>Edmond, OK<br>35.614131, -97.475083
AQS site ID	40 - 109 - 1037
Sampling timeframe (MM-DD-YY)	04-15-20 to 05-15-20
Sensor data source	QuantAQ Cloud download
Reference data source	AQS API download

Sensor Information			
Manufacturer, model	QuantAQ ARISense		
Device firmware version	Received by EPA on 4/30/2019		
Sampling time interval	1-minute		
Sensor serial numbers	ARS_01		
Issues encountered during deployment?	☑	See OK-ARS-Page 6 of this testing report	

FRM/FEM Information	
Manufacturer, model, designation	Teledyne Advanced Pollution Instrumentation T640 FEM
Sampling time interval	1-hour averaging
Date of calibration	As required by 40 CFR Part 58 and the QAPP for Ambient Air Monitoring in the State of Oklahoma maintained by ODEQ/AQD
Date of flowrate verification check	Monthly as required by 40 CFR Part 58 Appendix A
Description, date(s) of maintenance activities	N/A

Time Series Plots

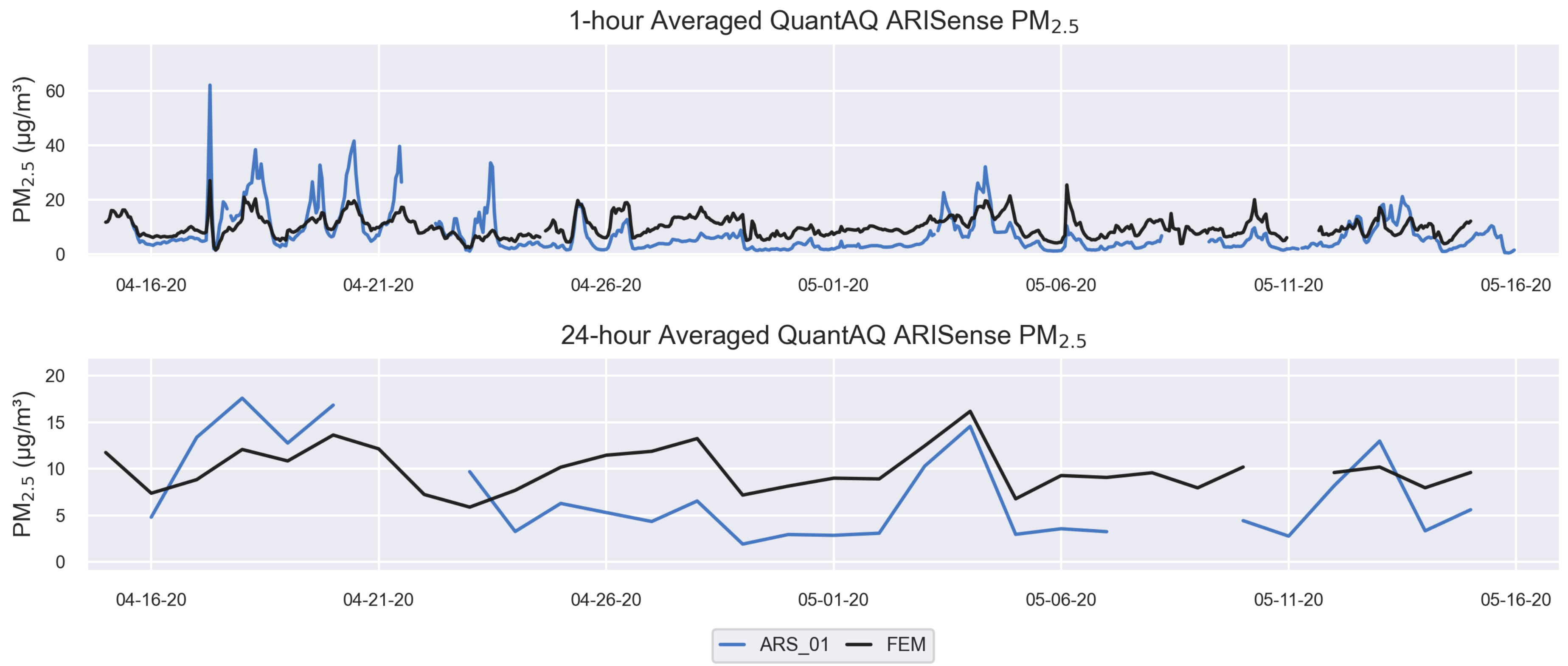

Scatter Plots: Comparison to FRM/FEM

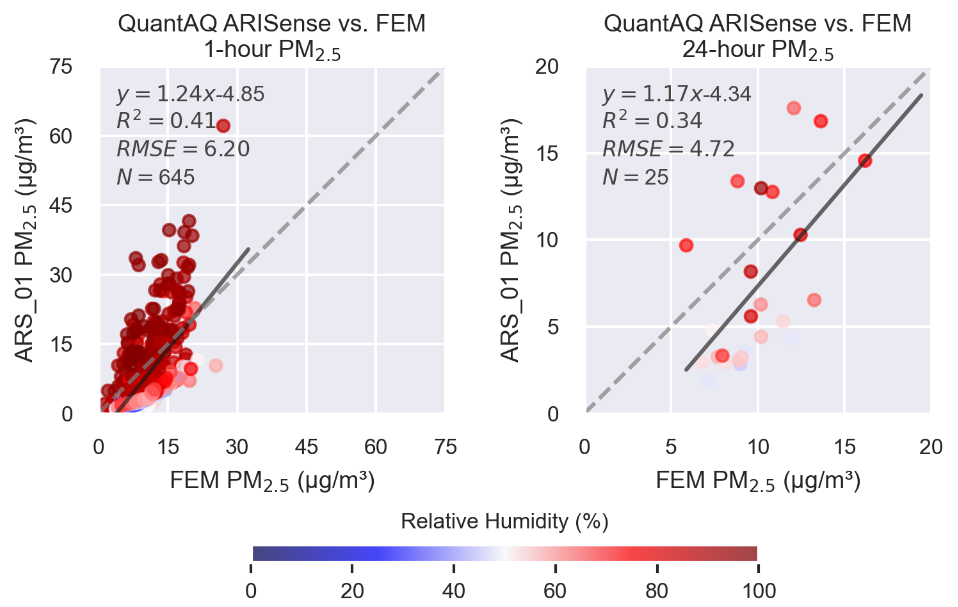

Range of FRM/FEM concentrations over duration of base test (µg/m³)	1.4-27.0 (1-hr), 5.9-16.2 (24-hr)
Number of 24-hr periods in FRM/FEM monitor measurements with a goal concentration ≥ 25 µg/m³	0

Performance Metrics

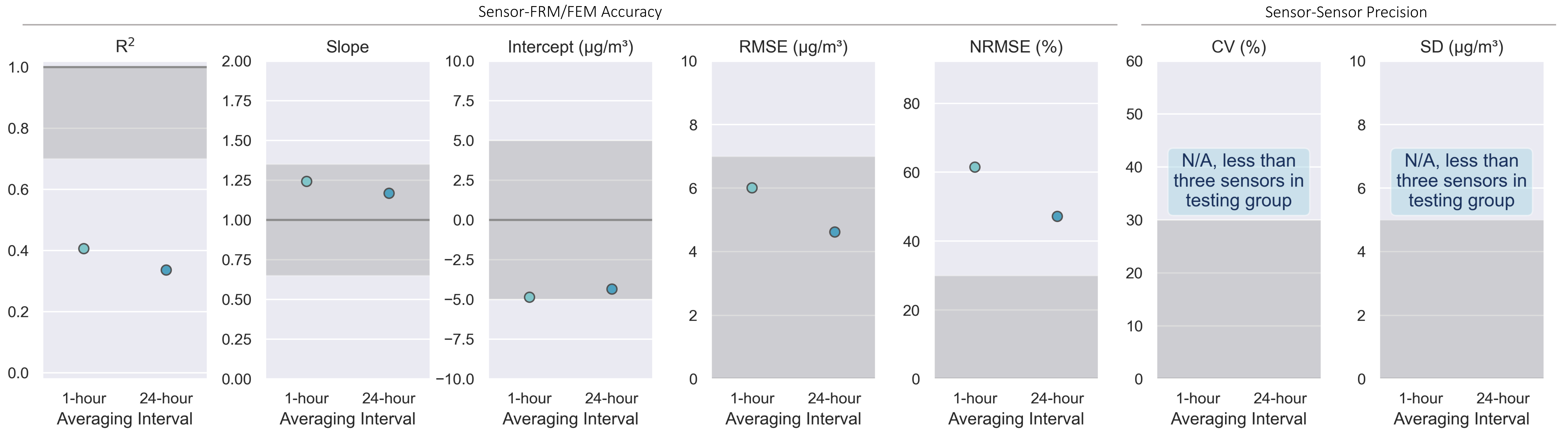

Meteorological Conditions During Deployment

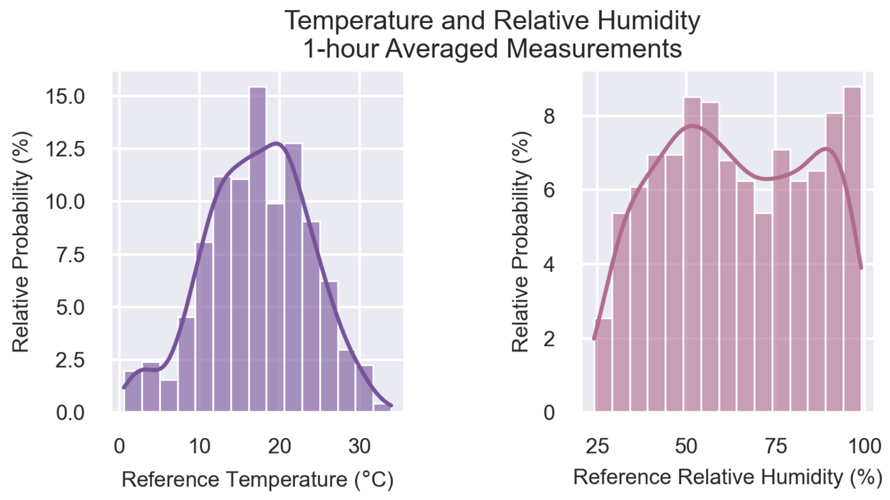

Number of 24-hr periods outside sensor manufacturer-listed temperature operational range (no operational range specified)	-
Number of 24-hr periods outside sensor manufacturer-listed relative humidity operational range (no operational range specified)	-

Meteorological Influence

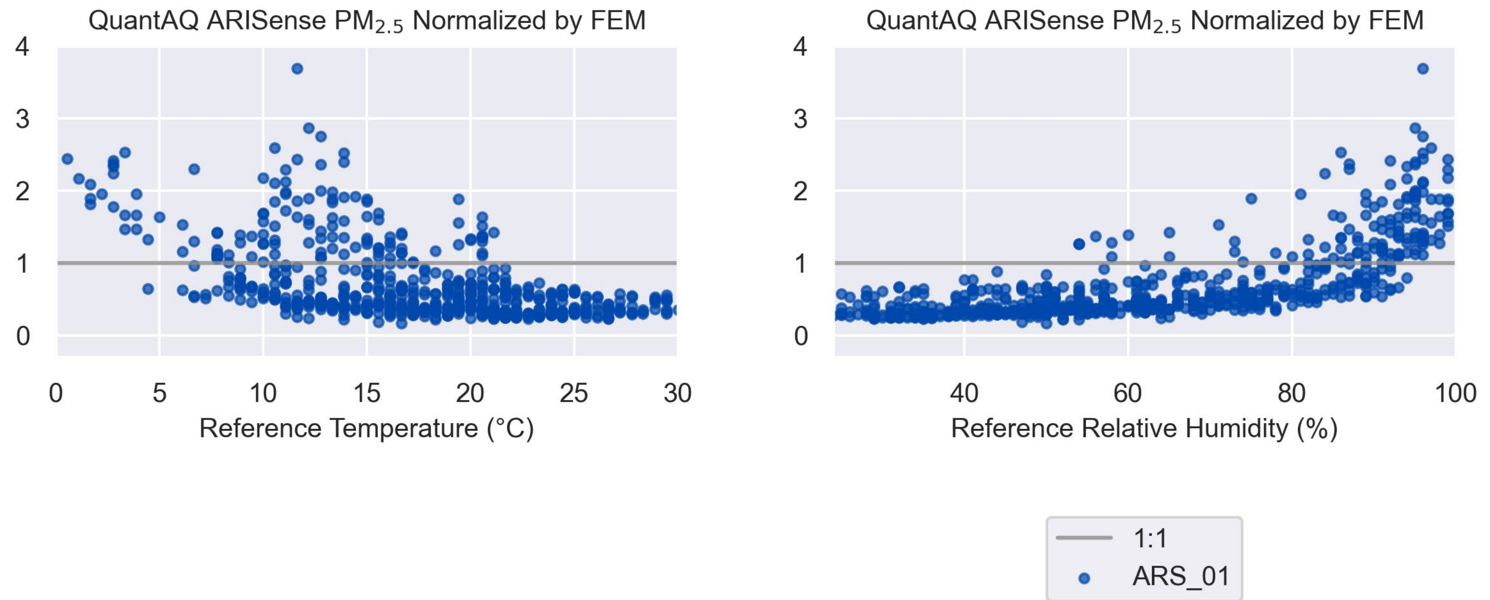

Testing Report - $PM_{2.5}$ Base Testing

QuantAQ ARISense

This report reflects out-of-the-box performance

Initial Base Testing - Edmond, OK

U.S. Environmental Protection Agency
Office of Research and Development
PI: Clements.Andrea@epa.gov
919-541-1363
April 2020—May 2020

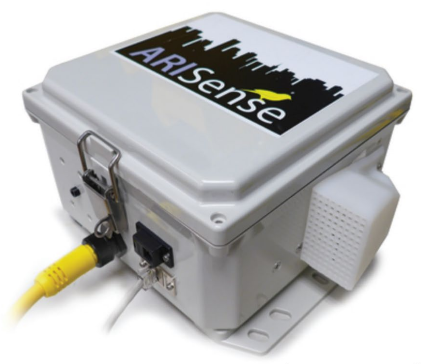

Image Attribution: QuantAQ, Inc (Somerville, MA)

Tabular Statistics

Sensor-FRM/FEM Correlation

	Bias and Linearity						Data Quality			
	R^2		Slope		Intercept (µg/m³)		Uptime (%)		Number of paired sensor and FRM/FEM concentration values	
	1-Hour ○	24-Hour ○	1-Hour ●	24-Hour ●	1-Hour ●	24-Hour ●	1-Hour ●	24-Hour ●	1-Hour	24-Hour
Metric Target Range	≥ 0.70	≥ 0.70	1.0 ± 0.35	1.0 ± 0.35	-5 ≤ b ≤ 5	-5 ≤ b ≤ 5	75%*	75%*	-	-
Sensor ARS_01	0.41	0.34	1.24	1.17	-4.85	-4.34	94	87	645	25

	Error			
	RMSE (µg/m³)		NRMSE (%)	
	1-Hour ★	24-Hour ★	1-Hour ☆	24-Hour ☆
Metric Target Range	≤ 7.0	≤ 7.0	≤ 30.0	≤ 30.0
Deployment Value	6.0	4.6	61.5	47.2

Device-specific metrics (computed for each sensor in evaluation)
○○○ Metric value for none of devices tested falls within the target range
●○○ Metric value for one of devices tested falls within the target range
●●○ Metric value for two of devices tested falls within the target range
●●● Metric value for three of devices tested falls within the target range

Single-valued metrics (computed via entire evaluation dataset)
☆ Indicates that the metric value is not within the target range
★ Indicates that the metric value is within the target range

Sensor-Sensor Precision[1]

	Precision (between collocated sensors)				Data Quality	
	CV (%)		SD (µg/m³)		Number of concurrent sensor concentration pairs	
	1-Hour ☆	24-Hour ☆	1-Hour ☆	24-Hour ☆	1-Hour	24-Hour
Metric Target Range	≤ 30.0	≤ 30.0	≤ 5.0	≤ 5.0	-	-
Deployment Value	-	-	-	-	-	-

[1]Precision statistics are computed for evaluations with at least three collocated sensor units. Metric values are left blank for evaluations with two or fewer sensor units.

*This value is only a recommendation for ensuring data quality and is not included in the list of target values discussed in Section 4 of the Performance Testing Protocols, Metrics, and Target Values for Fine Particulate Matter Air Sensors document.

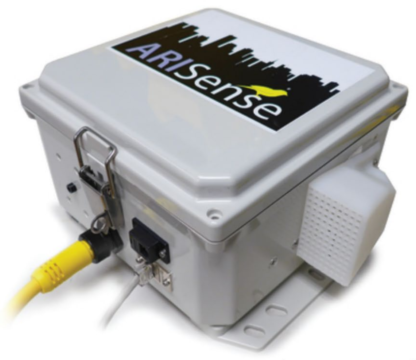

Image Attribution: QuantAQ, Inc (Somerville, MA)

Sensor-FRM/FEM Scatter Plots

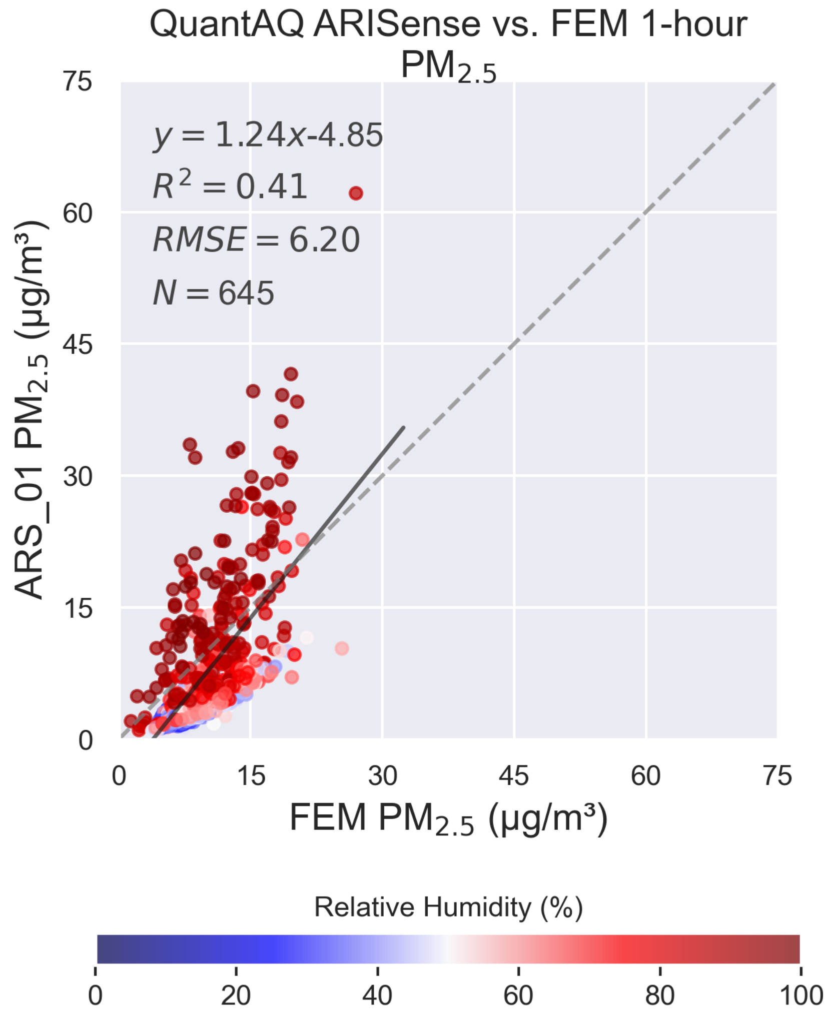

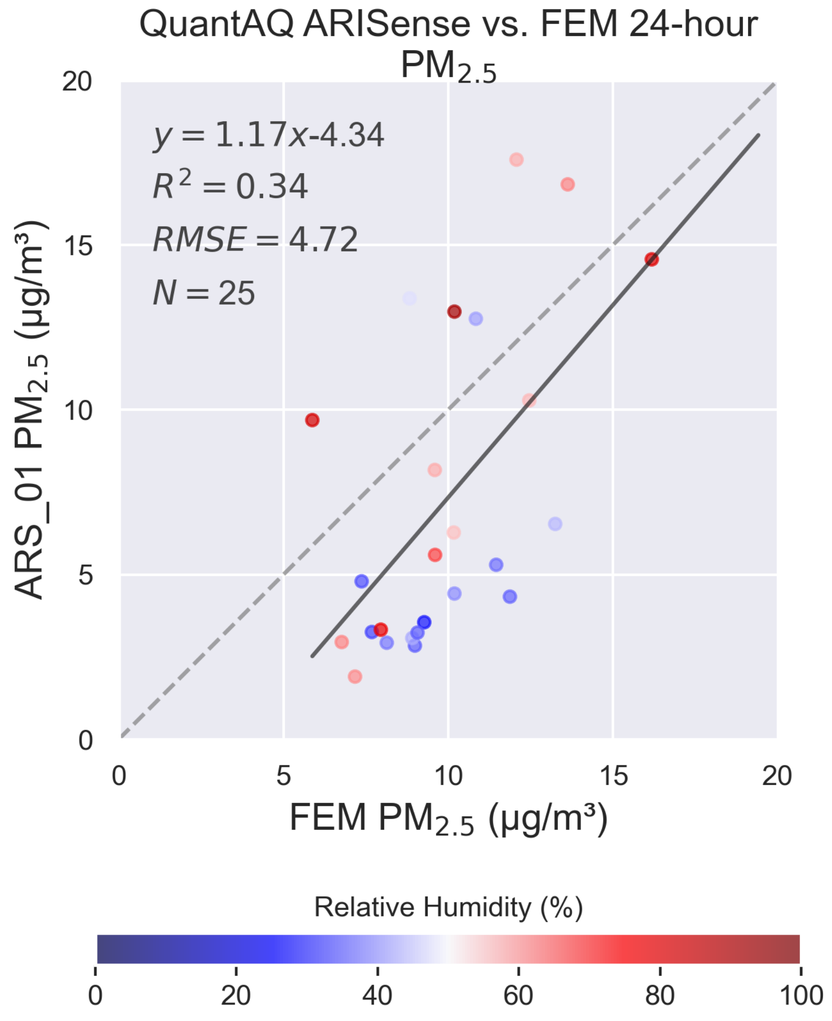

Initial Base Testing - Edmond, OK
U.S. Environmental Protection Agency
Office of Research and Development
PI: Clements.Andrea@epa.gov
919-541-1363
April 2020—May 2020

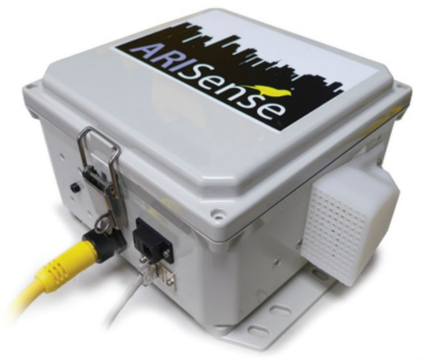

Image Attribution: QuantAQ, Inc (Somerville, MA)

Supplemental Information

Abbreviations used in Supplemental Information

FRM	Federal Reference Method
FEM	Federal Equivalent Method
SOP	Standard Operating Procedure
QAPP	Quality Assurance Project Plan
QC	Quality Control

Supplemental Documentation	Attached	Description & URL or file path to documentation
Field observations and sensor data flags	☑	See OK-ARS-Page 7 of this testing report
Maintenance logs	☐	No logs recorded during testing
Standard operating procedure(s)	☐	U.S. EPA Office Of Research and Development SOP available upon request
Photos of equipment setup and testing	☑	See OK-ARS-Page 5 of this testing report
Product specifications sheet(s)	☑	QuantAQ and Particles Plus: ARISense Specification Sheet
Product manual(s)	☐	N/A
Data storage and transmission method	☑	See OK-ARS-Page 6 of this testing report
Data correction approach	☑	See OK-ARS-Page 6 of this testing report
Issues encountered	☑	See OK-ARS-Page 6 of this testing report
Data analysis/correction scripts and version	☑	Averaging and processing of data, calculation of performance metrics, and generation of figures and other supplementary material for analysis were obtained using Python 3.9.7 with the packages sensortoolkit v0.8.3b2, pandas 1.3.5, NumPy 1.21.2, Matplotlib 3.5.0, statsmodels 0.13.0, and seaborn 0.11.2. All packages are available from the Python Package Index (PyPI) at https://pypi.org. The integrated development environment (IDE) Spyder 5.1.5 was used for scripting and data visualization. Version control for the Python base, packages, and IDE were all managed by conda 4.11.0.
Air Monitoring Station QAPP	☐	U.S. EPA Office Of Research and Development QAPP available upon request
Summary of FRM/FEM monitor QC checks	☑	See OK-ARS-Page 8 of this testing report
Manufacturer website for FRM/FEM monitor	☑	Teledyne API: T640/T640X Product website
FRM/FEM monitor manual	☑	Teledyne API: T640/T640X Product Manual
FRM/FEM monitor specifications sheet(s)	☑	Teledyne API: T640/T640X Specification Sheet
Other documents	☐	

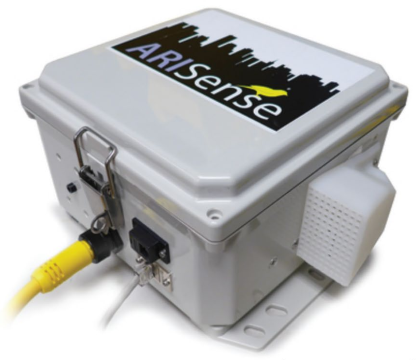

Image Attribution: QuantAQ, Inc (Somerville, MA)

Supplemental Information: Photos of Testing Site and Equipment Setup

Site Description:

The Oklahoma Christian University monitoring site was established in the late 1970s. There is a containerized building that houses the continuous instruments with a wooden platform directly adjacent for the filter-based instruments. Pollutants monitored include O_3, PM_{10} (filter based and continuous), $PM_{2.5}$ (filter based and continuous), $NO/NO_2/NO_x$, CO, SO_2 and meteorology. The site has solar panels to help defer the cost of power to the site.

Figure 1: OK Monitoring Station sampling shelter

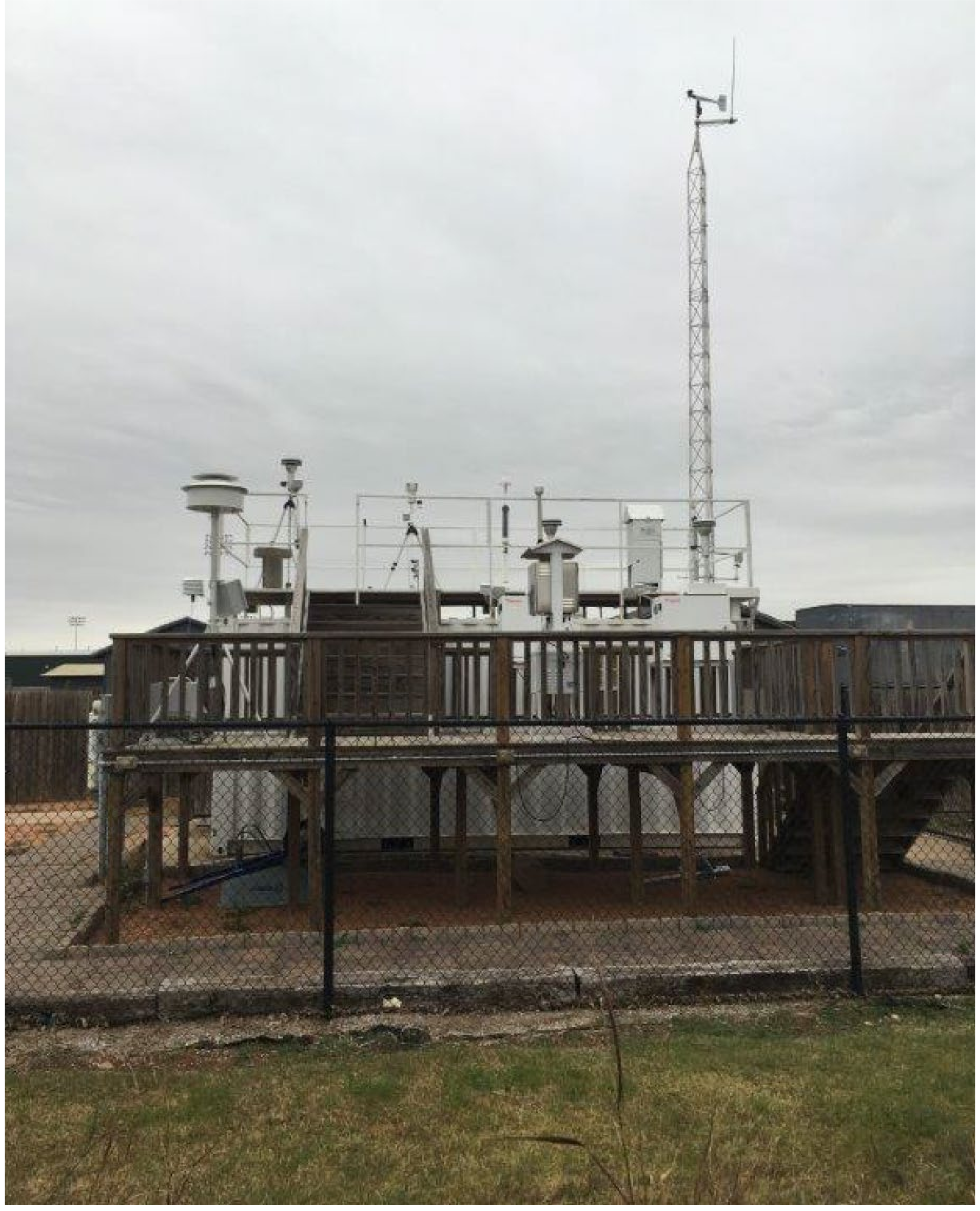

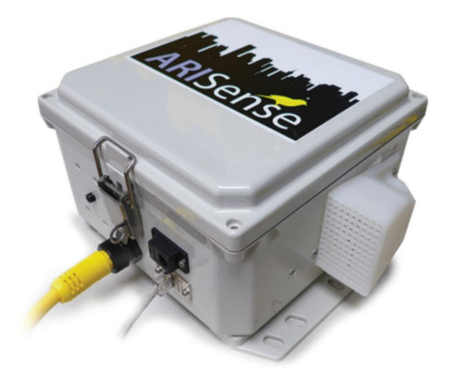

Image Attribution: QuantAQ, Inc (Somerville, MA)

Supplemental Information: Data Storage, Correction Approach, and Issues Encountered

Data Storage and Transmission Method

ARISense units upload data to an external cloud server at 2-minute averages via cellular signal. ARISense weekly data collections were performed by downloading data from the QuantAQ cloud server (last accessed 5/11/22).

Data Correction Approach

QuantAQ ARISense units measure PM2.5 concentrations via an optical particle counter (OPC). The OPC measures particle counts within particle size bins ranging from 0.3 microns to 10 microns, and mass concentrations for each bins are estimated from particle counts assuming constant values for density, spherical morphology, and average particle diameter. The manufacturer applies a general correction to account for the detection limitations of the OPC leading to concentrations that are closer to reality. These presets were configured by the sensor manufacturer and were not modified by EPA prior or during testing.

After acquisition, the raw data was processed using the *sensortoolkit* python code library (v0.8.3b2). A continuous data set at the recorded sampling frequency was written to a .csv file. 1-hour and 24-hour averaged data sets were generated using a 75% completeness threshold and saved as separate .csv files. Outliers were NOT removed from data sets in order to assess "out-of-the-box" sensor performance.

Issues Encountered

Pre-deployment observations

Numerous issues were encountered with the ARISense devices. Although previous models of the ARISense had used AlphaSense OPC-N2 these OPCs were unavailable at the time of manufacture. So instead, components from a particle plus OPC handheld were used.

- *Shipping damage:* On arrival to EPA many of the units were damaged due to the heavy pump breaking free during shipping and shaking around inside of the box breaking much of the plastic scaffolding and, in some cases, components. The manufacturer worked with us to fix these units.
- *Hardware and Firmware issues:* Multiple hardware and firmware issues were experienced that had to be fixed before starting the deployment and multiple manual firmware updates on both the gas sensor microcontrollers and the OPC microcontrollers were completed by researchers. The USB drives were frequently corrupted which required replacement and eventually a hardware update. In addition, the OPC microcontroller and the gas sensor microcontroller often miscommunicated. One firmware version reduced the number of bins reported by the OPC since the manufacturer determined the additional bins were not providing additional accuracy. New PCB boards were sent and installed before the tests could be run as well.
- *Initiation:* There was a high power draw when the cellular connectivity initiated and the pump was turned on. This meant that units occasionally failed to turn on at the same time and it meant these could not be powered by solar.
- *Water intake:* In addition, the OPC pump often drew in water during rainstorms leading the inline filter to saturate and the pump to fail due to overheating. Many pumps had to be replaced throughout the testing.
- *Cellular connectivity:* Some units could not consistently stay connected to the cellular, though other sensors at the same site did not experience issues. The manufacturer could not recreate this issue when they were sent back for service and the problems persisted.
- *Reset:* These units ended up being operated on timers so that they were power cycled every night which would typically get them back online if a software error had been encountered, leading to lower data loss than would have occurred with only weekly checks.

Field observations and sensor data flags are included on the following page

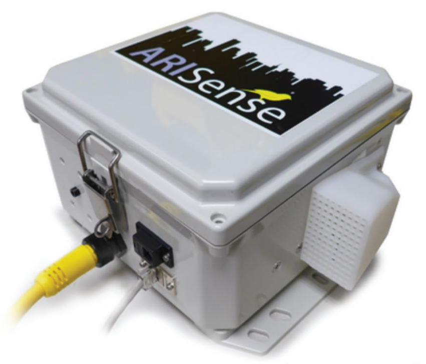

Image Attribution: QuantAQ, Inc (Somerville, MA)

Field observations and sensor data flags

The following table contains data flags describing events that were encountered during the testing period. The QuantAQ ARISense was configured to record data at regular 60-second sampling intervals, however, occasionally the unit recorded irregular sampling intervals ranging from 2-minute intervals to nearly 15 minutes at longest interval duration. The flags below note when these irregular sampling intervals occurred during the testing period. Sampling interval irregularities were not a result of modification to the device firmware configuration.

During the testing period, it was observed that the ARISense unit's pressure sensor would measure rapid shifts in recorded values as well as periods during which the measured pressure was a constant 24,000 Pa. On 5/8/2020 and again on 5/14/2020, power outages were reported which brought the ARISense offline and prevented the sensor from recording data. These outages occurred when the field technician was not at the monitoring site, indicating that the cause of the outage was likely not due to operator error.

Start Time (UTC)	End Time (UTC)	Sensor Serial ID	Parameters Impacted	Flag
2020-04-15 12:20:00+00:00	2020-04-27 22:59:00+00:00	ARS_01	ALL	6.0-Sampling interval abnormality
2020-04-20 23:00:00+00:00	2020-05-14 23:59:00+00:00	ARS_01	P	13.0-Sensor malfunction - Met data
2020-04-21 12:25:00+00:00	2020-04-22 05:13:00+00:00	ARS_01	ALL	9.0-Data loss - Power connection error
2020-04-26 23:00:00+00:00	2020-05-04 22:59:00+00:00	ARS_01	ALL	6.0-Sampling interval abnormality
2020-05-04 23:00:00+00:00	2020-05-25 22:59:00+00:00	ARS_01	ALL	6.0-Sampling interval abnormality
2020-05-08 05:25:00+00:00	2020-05-09 05:13:00+00:00	ARS_01	ALL	9.0-Data loss - Power connection error
2020-05-14 03:59:00+00:00	2020-05-14 04:30:00+00:00	ARS_01	ALL	9.0-Data loss - Power connection error
2020-05-14 04:31:00+00:00	2020-05-14 05:13:00+00:00	ARS_01	ALL	9.0-Data loss - Power connection error

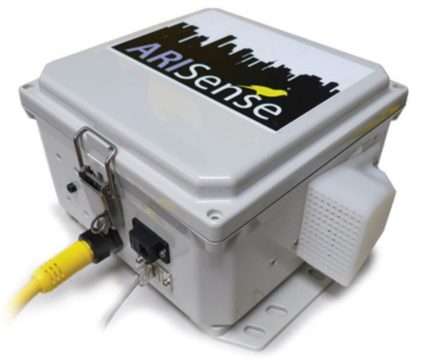

Image Attribution: QuantAQ, Inc (Somerville, MA)

Description of Data Flags

AQS

The U.S. EPA's Air Quality System (AQS) is the Agency's primary ambient air monitoring data archive. A comprehensive list of data flags that are recorded alongside AQS data sets, referred to by U.S. EPA as 'qualifiers', can be found at the following link: https://aqs.epa.gov/aqsweb/documents/codetables/qualifiers.html

Invalidation of reference data: AQS qualifiers are organized by qualifier type, which indicates whether data logged alongside qualifier flags should be invalidated (set null). Qualifiers with type "Null Data Qualifier" are invalidated, and includes data logged during periods that coincide with QC checks (e.g., "BF-Precision/Zero/Span", "BJ- Operator Error", "BL - QA Audit", "AZ - QC Audit") among other events such as power outages. Data logged alongside qualifiers with type "Quality Assurance Qualifiers" are not invalidated and are included in this analysis (e.g., concentrations less than the federal MDL for the reference monitor "MD – Value less than MDL", QA reviewed values "Validated Value").

Data Flags Recorded During Testing

FRM/FEM Monitor	Timestamp (UTC)	Flag
Teledyne API T640 (Data acquired via AQS)	2020-04-24 14:00:00+0000 to 2020-04-24 15:00:00+0000	AZ - QC Audit
	2020-05-11 00:00:00+0000 to 2020-05-11 15:00:00+0000	BK - Site computer/data logger down

Meteorological Instrument	Timestamp (UTC)	Flag
RM Young 41382V Temperature Monitor (Data acquired via AQS)	2020-05-11 00:00:00+0000 to 2020-05-11 15:00:00+0000	BK - Site computer/data logger down
RM Young 41382V Relative Humidity Monitor (Data acquired via AQS)	2020-05-11 00:00:00+0000 to 2020-05-11 15:00:00+0000	BK - Site computer/data logger down

Testing Report - $PM_{2.5}$ Base Testing

Aeroqual AQY

This report reflects out-of-the-box performance

Initial Base Testing - Milwaukee, WI

U.S. Environmental Protection Agency
Office of Research and Development
PI: Clements.Andrea@epa.gov
919-541-1363

July 2019—August 2019

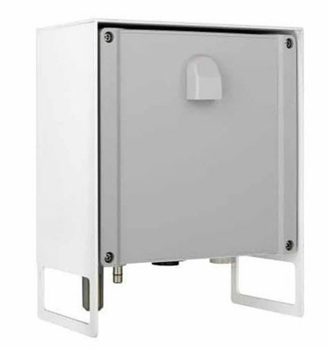
Image Attribution: Aeroqual Limited (Auckland, New Zealand)

Deployment Details

Testing Organization and Site Information	
Testing organization (Name, Organization type, Contact website)	U.S. Environmental Protection Agency - Office of Research and Development<br>Federal Government<br>Air Sensor Toolbox | U.S. EPA Website
Testing location (City, State, Latitude and Longitude)	WDNR Headquarters<br>Milwaukee, WI<br>43.061, -87.9135
AQS site ID	55 - 079 - 0026
Sampling timeframe (MM-DD-YY)	07-25-19 to 08-24-19
Sensor data source	Aeroqual Cloud download
Reference data source	AQS API download

Sensor Information			
Manufacturer, model	Aeroqual AQY		
Device firmware version	1.14.2		
Sampling time interval	1-minute		
Sensor serial numbers	AQY_01		
Issues encountered during deployment?	☐	No Issues	

FRM/FEM Information	
Manufacturer, model, designation	Teledyne Advanced Pollution Instrumentation T640X FEM
Sampling time interval	1-hour averaging
Date of calibration	As required by 40 CFR Part 58 and the Low Volume $PM_{2.5}$ and PM_{10} Networks QAPP maintained by WDNR
Date of flowrate verification check	Monthly as required by 40 CFR Part 58 Appendix A
Description, date(s) of maintenance activities	N/A

Time Series Plots

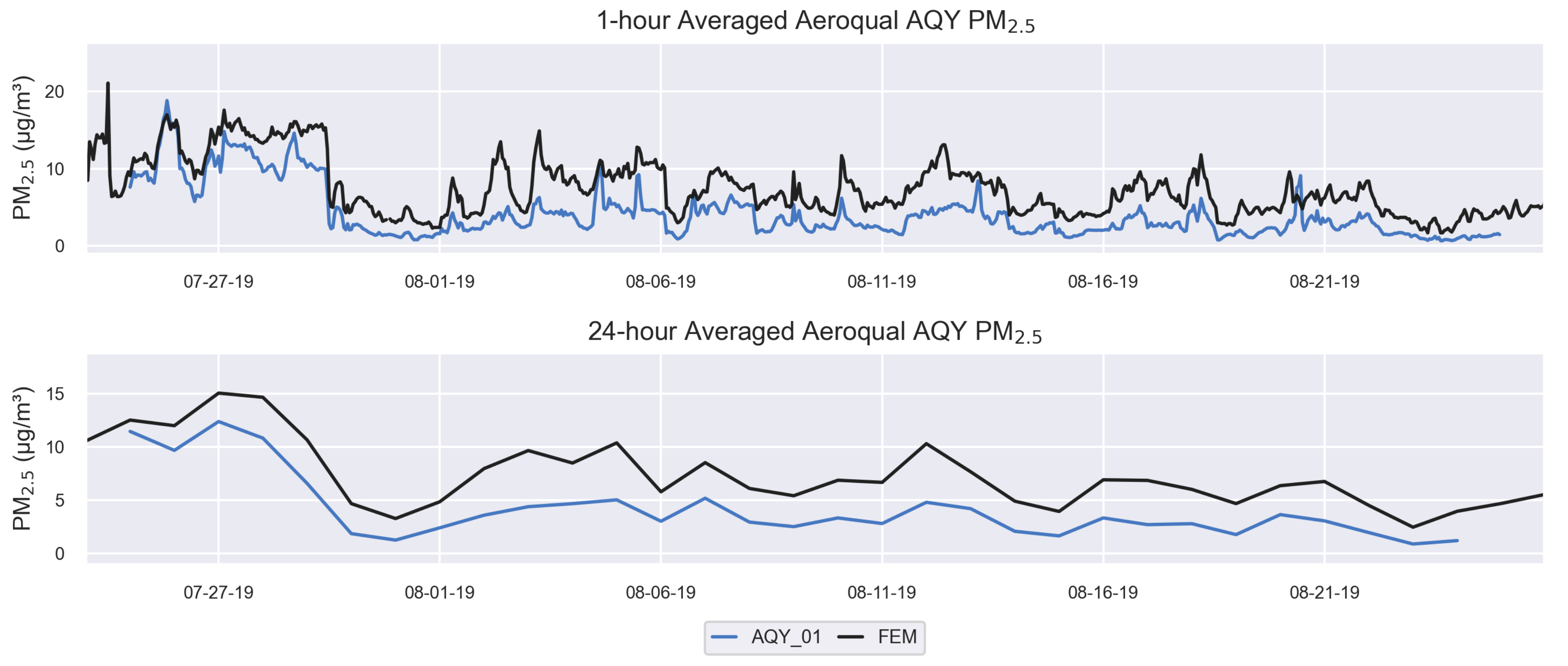

Scatter Plots: Comparison to FRM/FEM

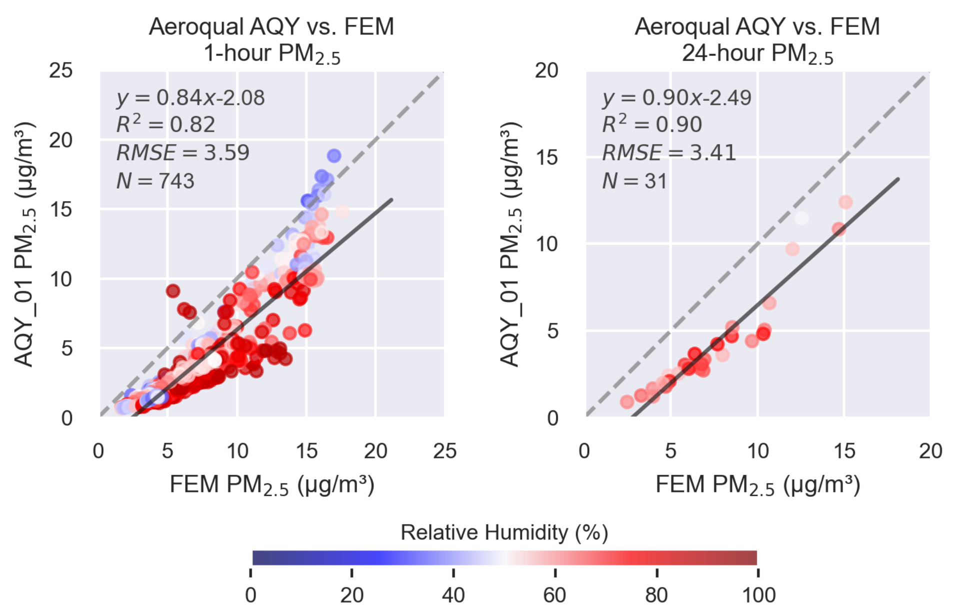

Range of FRM/FEM concentrations over duration of base test (µg/m³)	1.7-17.6 (1-hr), 2.5-15.1 (24-hr)
Number of 24-hr periods in FRM/FEM monitor measurements with a goal concentration ≥ 25 µg/m³	0

Performance Metrics

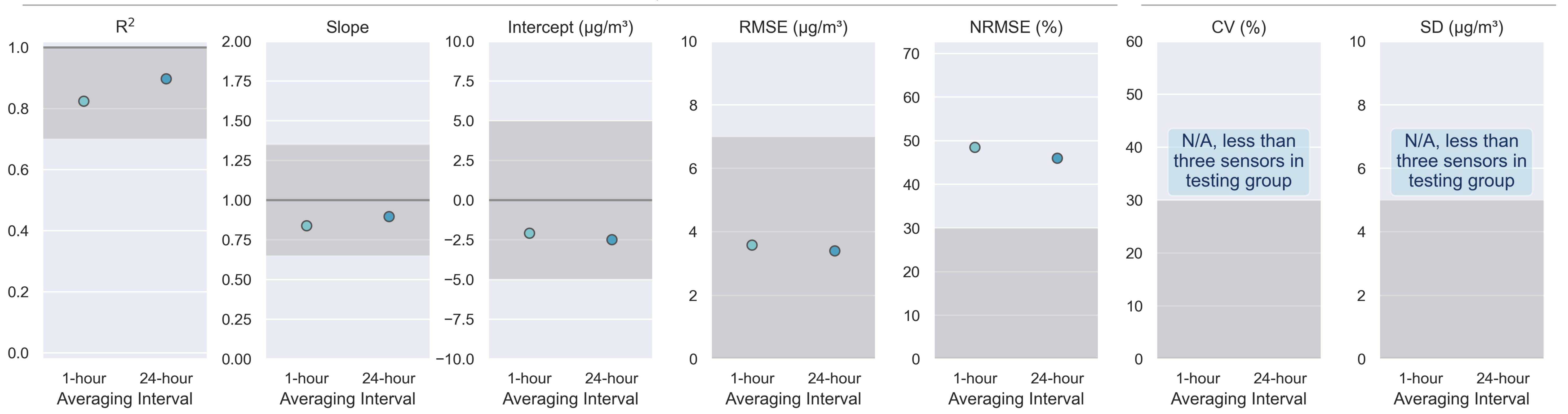

Meteorological Conditions During Deployment

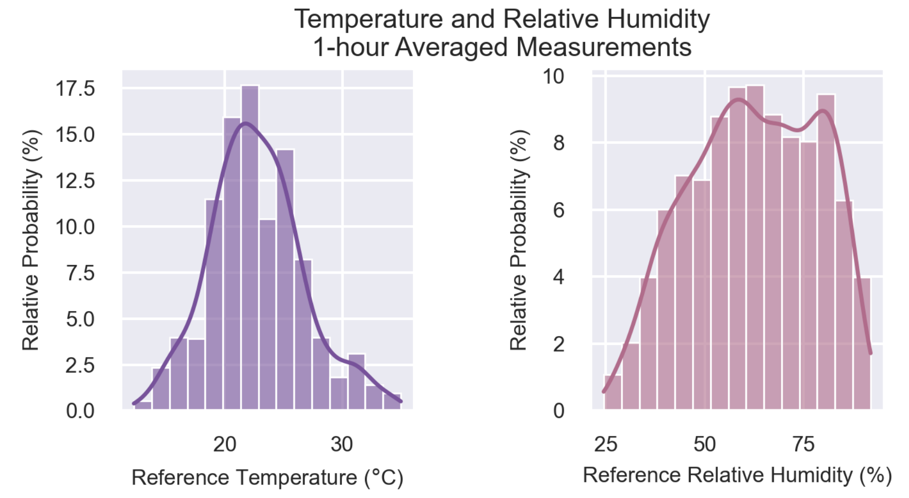

Number of 24-hr periods outside sensor manufacture-listed temperature operational range (-10 to 40 °C)	0
Number of 24-hr periods outside sensor manufacture-listed relative humidity operational range (no operational range specified)	-

Meteorological Influence

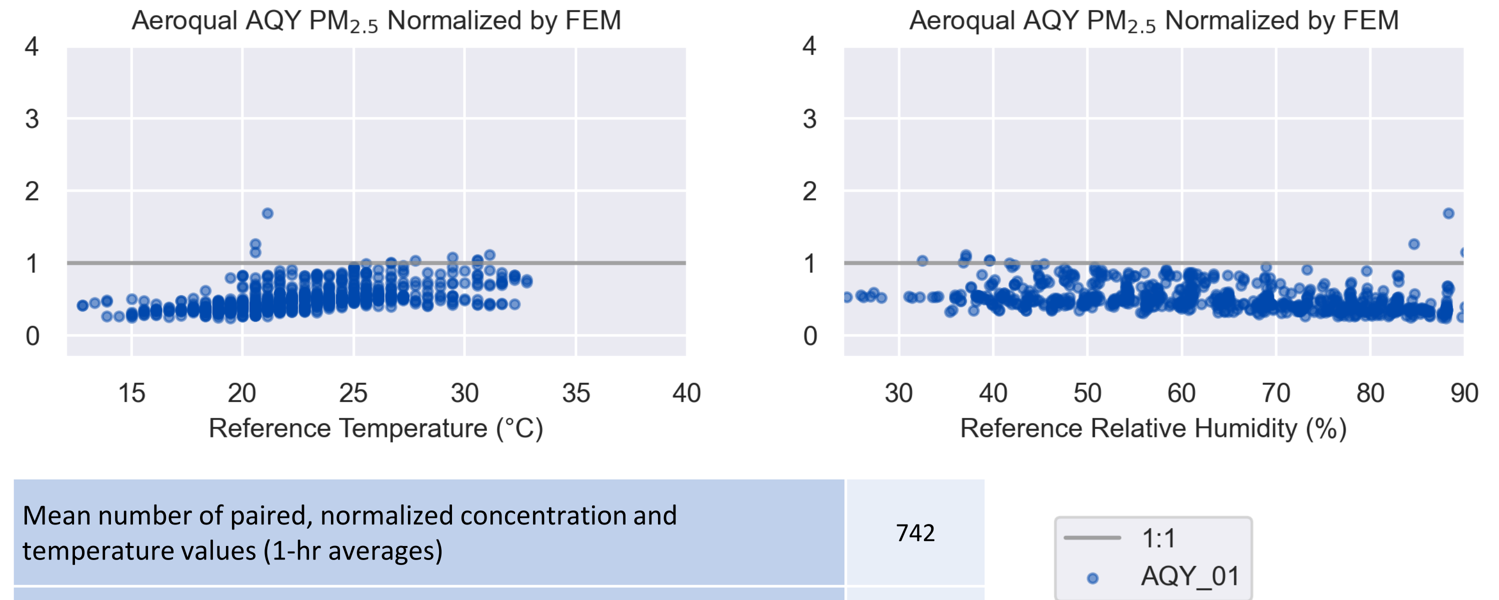

Mean number of paired, normalized concentration and temperature values (1-hr averages)	742
Mean number of paired, normalized concentration and relative humidity values (1-hr averages)	0

Testing Report - $PM_{2.5}$ Base Testing

Aeroqual AQY

This report reflects out-of-the-box performance

Initial Base Testing - Milwaukee, WI

U.S. Environmental Protection Agency
Office of Research and Development
PI: Clements.Andrea@epa.gov
919-541-1363

July 2019—August 2019

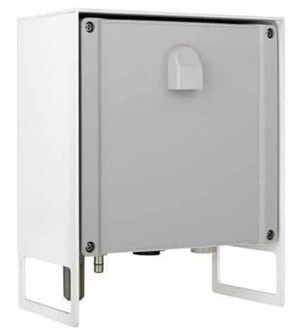

Image Attribution: Aeroqual Limited (Auckland, New Zealand)

Tabular Statistics

Sensor-FRM/FEM Correlation

	Bias and Linearity						Data Quality			
	R^2		Slope		Intercept ($\mu g/m^3$)		Uptime (%)		Number of paired sensor and FRM/FEM concentration values	
	1-Hour ●	24-Hour ●	1-Hour ●	24-Hour ●	1-Hour ●	24-Hour ●	1-Hour ●	24-Hour ●	1-Hour	24-Hour
Metric Target Range	≥ 0.70	≥ 0.70	1.0 ± 0.35	1.0 ± 0.35	-5 ≤ b ≤ 5	-5 ≤ b ≤ 5	75%*	75%*	-	-
Sensor AQY_01	0.82	0.90	0.84	0.90	-2.08	-2.49	100	100	743	31

	Error			
	RMSE ($\mu g/m^3$)		NRMSE (%)	
	1-Hour ★	24-Hour ★	1-Hour ☆	24-Hour ☆
Metric Target Range	≤ 7.0	≤ 7.0	≤ 30.0	≤ 30.0
Deployment Value	3.6	3.4	48.5	46.1

Device-specific metrics (computed for each sensor in evaluation)
○○○ Metric value for none of devices tested falls within the target range
●○○ Metric value for one of devices tested falls within the target range
●●○ Metric value for two of devices tested falls within the target range
●●● Metric value for three of devices tested falls within the target range

Single-valued metrics (computed via entire evaluation dataset)
☆ Indicates that the metric value is not within the target range
★ Indicates that the metric value is within the target range

Sensor-Sensor Precision[1]

	Precision (between collocated sensors)				Data Quality	
	CV (%)		SD ($\mu g/m^3$)		Number of concurrent sensor concentration pairs	
	1-Hour ☆	24-Hour ☆	1-Hour ☆	24-Hour ☆	1-Hour	24-Hour
Metric Target Range	≤ 30.0	≤ 30.0	≤ 5.0	≤ 5.0	-	-
Deployment Value	-	-	-	-	-	-

[1]Precision statistics are computed for evaluations with at least three collocated sensor units. Metric values are left blank for evaluations with two or fewer sensor units.

*This value is only a recommendation for ensuring data quality and is not included in the list of target values discussed in Section 4 of the Performance Testing Protocols, Metrics, and Target Values for Fine Particulate Matter Air Sensors document.

Testing Report - $PM_{2.5}$ Base Testing

Aeroqual AQY

This report reflects out-of-the-box performance

Initial Base Testing - Milwaukee, WI
U.S. Environmental Protection Agency
Office of Research and Development
PI: Clements.Andrea@epa.gov
919-541-1363
July 2019—August 2019

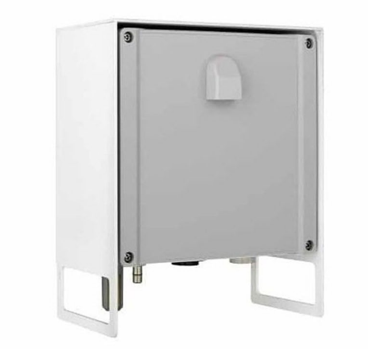

Image Attribution: Aeroqual Limited (Auckland, New Zealand)

Sensor-FRM/FEM Scatter Plots

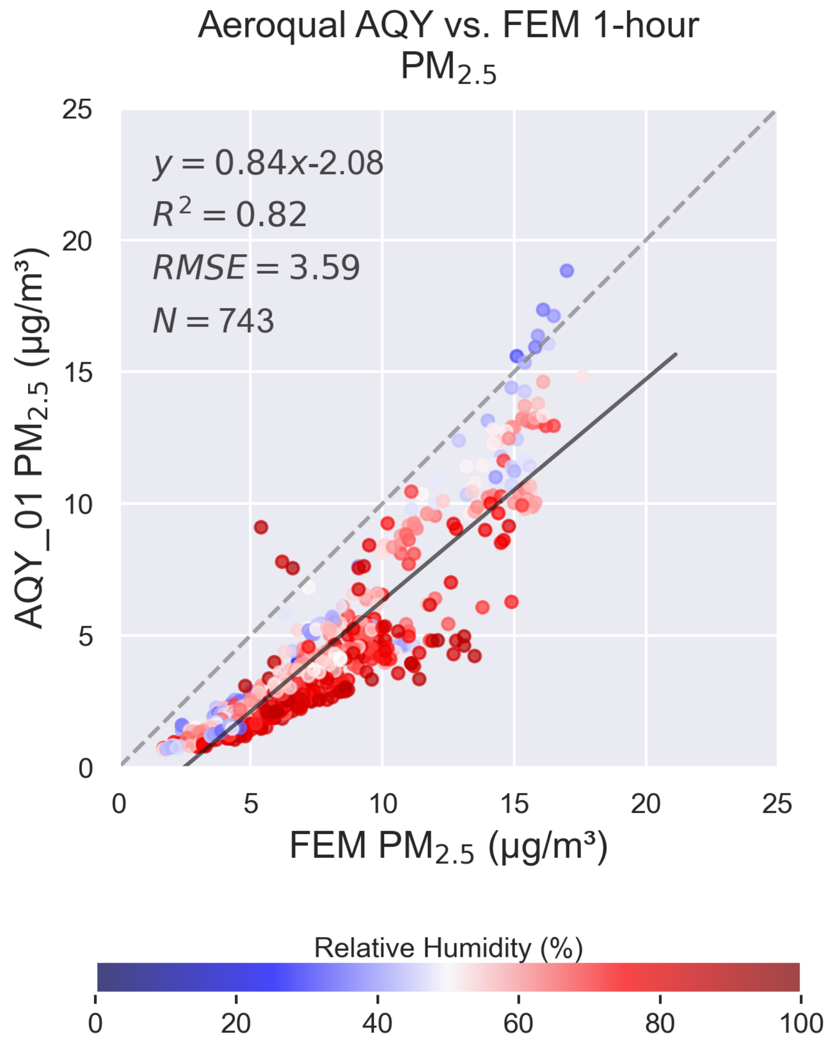

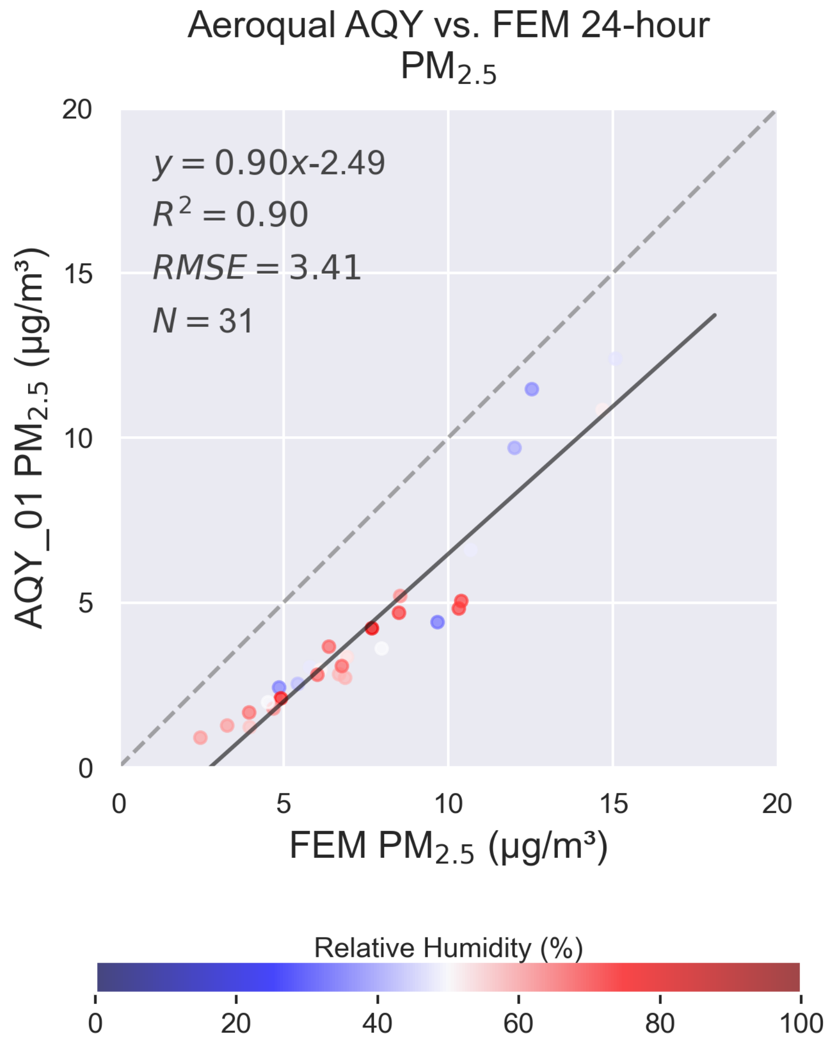

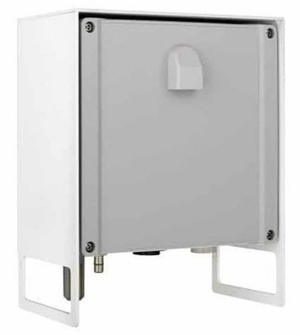

Image Attribution: Aeroqual Limited (Auckland, New Zealand)

Supplemental Information

Abbreviations used in Supplemental Information

FRM	Federal Reference Method
FEM	Federal Equivalent Method
SOP	Standard Operating Procedure
QAPP	Quality Assurance Project Plan
QC	Quality Control

Supplemental Documentation	Attached	Description & URL or file path to documentation
Field observations and sensor data flags	☑	See WI-AQY-Page 6 of this testing report
Maintenance logs	☐	No logs recorded during testing
Standard operating procedure(s)	☐	U.S. EPA Office Of Research and Development SOP available upon request
Photos of equipment setup and testing	☑	See WI-AQY-Page 5 of this testing report
Product specifications sheet(s)	☐	N/A*
Product manual(s)	☐	N/A*
Data storage and transmission method	☑	See WI-AQY-Page 6 of this testing report
Data correction approach	☑	See WI-AQY-Page 6 of this testing report
Issues encountered	☑	See WI-AQY-Page 6 of this testing report
Data analysis/correction scripts and version	☑	Averaging and processing of data, calculation of performance metrics, and generation of figures and other supplementary material for analysis were obtained using Python 3.9.7 with the packages sensortoolkit v0.8.3b2, pandas 1.3.5, NumPy 1.21.2, Matplotlib 3.5.0, statsmodels 0.13.0, and seaborn 0.11.2. All packages are available from the Python Package Index (PyPI) at https://pypi.org. The integrated development environment (IDE) Spyder 5.1.5 was used for scripting and data visualization. Version control for the Python base, packages, and IDE were all managed by conda 4.11.0.
Air Monitoring Station QAPP	☐	U.S. EPA Office Of Research and Development QAPP available upon request
Summary of FRM/FEM monitor QC checks	☑	See WI-AQY-Page 7 of this testing report
Manufacturer website for FRM/FEM monitor	☑	Teledyne API: T640/T640X Product website
FRM/FEM monitor manual	☑	Teledyne API: T640/T640X Product Manual
FRM/FEM monitor specifications sheet(s)	☑	Teledyne API: T640/T640X Specification Sheet
Other documents	☑	Manufacturer notice of AQY sales on hold

*As of 3/18/2021, the manufacturer of the AQY has placed sales of a similar unit on hold. Documentation for the AQY is currently unavailable from the manufacturer's website.

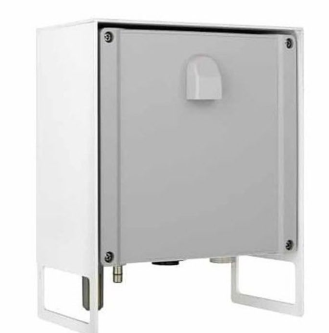

Image Attribution: Aeroqual Limited (Auckland, New Zealand)

Supplemental Information: Photos of Testing Site and Equipment Setup

Site Description:

The urban site was located in the parking lot at the former WDNR Southeast Regional Headquarters located at 2300 N. Martin Luther King Blvd. Sample inlets are roughly 5 meters above ground level and 50 meters from the nearest road. Verified through annual WDNR audits, the site meets the requirements of *40 CFT 58, Appendices C, D, E and G.*

Figure 1: Aeroqual AQY sensor (indicated by red arrow) attached to metal railing atop the sampling shelter at the monitoring site.

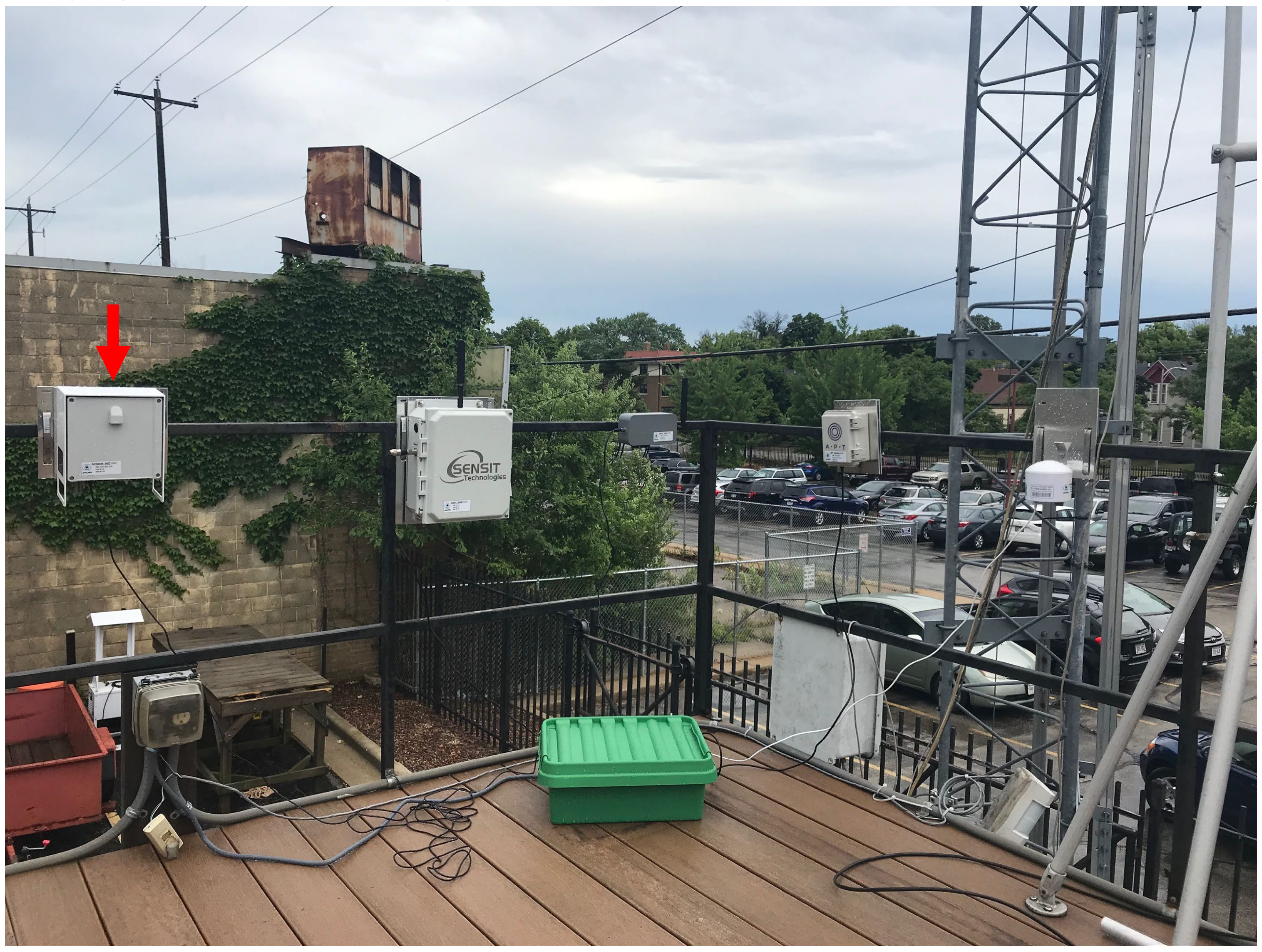

Figure 2: WDNR Monitoring Station

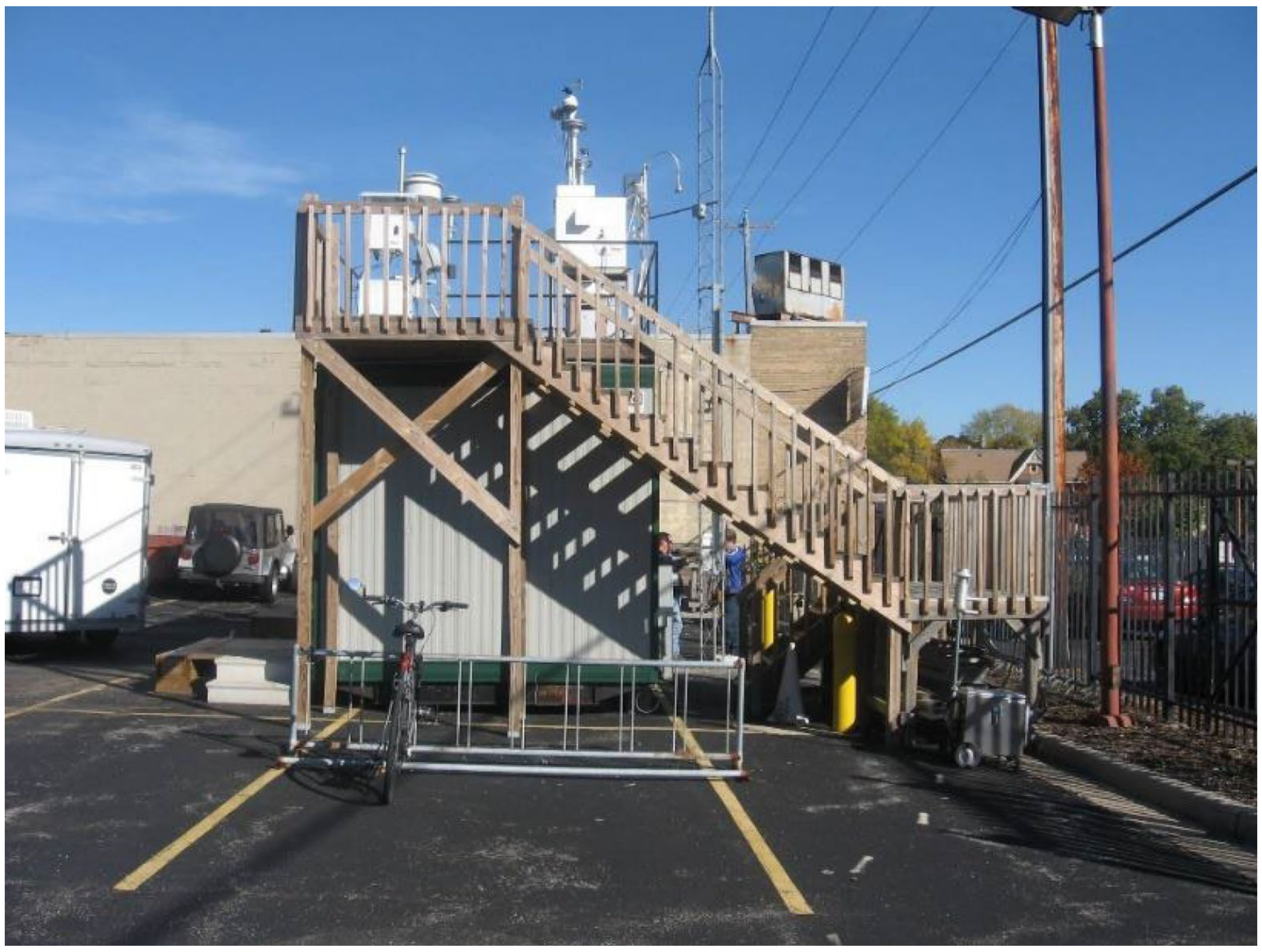

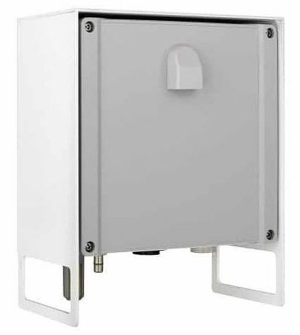

Image Attribution: Aeroqual Limited (Auckland, New Zealand)

Supplemental Information: Data Storage, Correction Approach, and Issues Encountered

Data Storage and Transmission Method

As part of CRADA #934-16 between Aeroqual and US EPA, Aeroqual supported data streaming. SIM cards were installed and data flowed to the Aeroqual Cloud. The 1-minute raw data was acquired weekly using the Aeroqual Cloud (*last accessed 5/11/22*) user interface (UI). The AQY has an internal data storage USB flash drive as a data backup, however the software on the drive is proprietary to Aeroqual and unreadable by outside systems.

Data Correction Approach

This evaluation report reflects "out-of-the-box" performance of the AQY. The manufacturer provides a procedure by which local collocation (sensor operated along side an FRM/FEM) data can be collected, a gain (slope) and offset (intercept) determined, and parameters entered into the Aeroqual Cloud user interface to be applied to all subsequently collected data. This procedure and feature was NOT used prior to this evaluation. Prospective consumers may get different performance from this device if they utilize this feature.

After acquisition, the raw data was processed using the *sensortoolkit* python code library (v0.8.3b2). A continuous data set at the recorded sampling frequency was written to a .csv file. 1-hour and 24-hour averaged data sets were generated using a 75% completeness threshold and saved as separate .csv files. Outliers were NOT removed from data sets in order to assess "out-of-the-box" sensor performance.

Issues Encountered

Pre-deployment observations

- *Timestamp inaccuracies*: During pre-deployment, the AQY devices did not properly sync timestamps with the onboard Real-Time Clock. Connecting the units to the internet by cellular or Wi-Fi allowed the unit to sync with internet time and resulted in proper timestamps.-

Field observations and sensor data flags

The Aeroqual AQY was deployed at the WDNR monitoring site on 7/22/2019. The AQY unit operated nominally during the testing period and did not require replacement or repair.

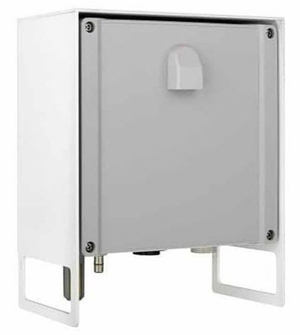

Image Attribution: Aeroqual Limited (Auckland, New Zealand)

Description of Data Flags

AQS

The U.S. EPA's Air Quality System (AQS) is the Agency's primary ambient air monitoring data archive. A comprehensive list of data flags that are recorded alongside AQS data sets, referred to by U.S. EPA as 'qualifiers', can be found at the following link: https://aqs.epa.gov/aqsweb/documents/codetables/qualifiers.html

Invalidation of reference data: AQS qualifiers are organized by qualifier type, which indicates whether data logged alongside qualifier flags should be invalidated (set null). Qualifiers with type "Null Data Qualifier" are invalidated, and includes data logged during periods that coincide with QC checks (e.g., "BF-Precision/Zero/Span", "BJ- Operator Error", "BL - QA Audit", "AZ - QC Audit") among other events such as power outages. Data logged alongside qualifiers with type "Quality Assurance Qualifiers" are not invalidated and are included in this analysis (e.g., concentrations less than the federal MDL for the reference monitor "MD – Value less than MDL", QA reviewed values "Validated Value").

Data Flags Recorded During Testing

FRM/FEM Monitor	Timestamp (UTC)	Flag
Teledyne API T640X<br>(Data acquired from AQS)	2019-07-30 20:00:00+0000	AX - Precision Check

Meteorological Instrument	Timestamp (UTC)	Flag
MetOne Temperature Monitor<br>(Data acquired from AQS)	2019-08-07 16:00:00+0000 to 2019-08-07 17:00:00+0000	AZ - QC Audit

Testing Report - $PM_{2.5}$ Base Testing

APT Maxima

This report reflects out-of-the-box performance

Initial Base Testing - Milwaukee, WI
U.S. Environmental Protection Agency
Office of Research and Development
PI: Clements.Andrea@epa.gov
919-541-1363
August 2019—September 2019

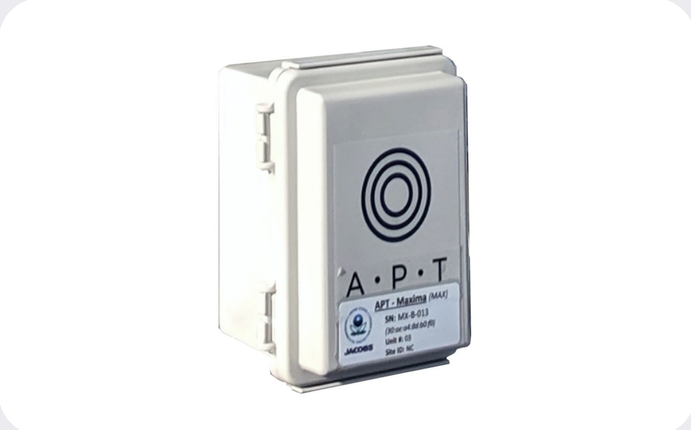

Deployment Details

Testing Organization and Site Information	
Testing organization (Name, Organization type, Contact website)	U.S. Environmental Protection Agency - Office of Research and Development<br>Federal Government<br>Air Sensor Toolbox | U.S. EPA Website
Testing location (City, State, Latitude and Longitude)	WDNR Headquarters<br>Milwaukee, WI<br>43.061, -87.9135
AQS site ID	55 - 079 - 0026
Sampling timeframe (MM-DD-YY)	08-22-19 to 09-21-19
Sensor data source	Aeroqual Cloud download
Reference data source	AQS API download

Sensor Information			
Manufacturer, model	APT Maxima		
Device firmware version	v5.0 (installed 5/2/2019)		
Sampling time interval	30-seconds		
Sensor serial numbers	MAX_02		
Issues encountered during deployment?	☐	No Issues	

FRM/FEM Information	
Manufacturer, model, designation	Teledyne Advanced Pollution Instrumentation T640X FEM
Sampling time interval	1-hour averaging
Date of calibration	As required by 40 CFR Part 58 and the Low Volume $PM_{2.5}$ and PM_{10} Networks QAPP maintained by WDNR
Date of flowrate verification check	Monthly as required by 40 CFR Part 58 Appendix A
Description, date(s) of maintenance activities	N/A

Time Series Plots

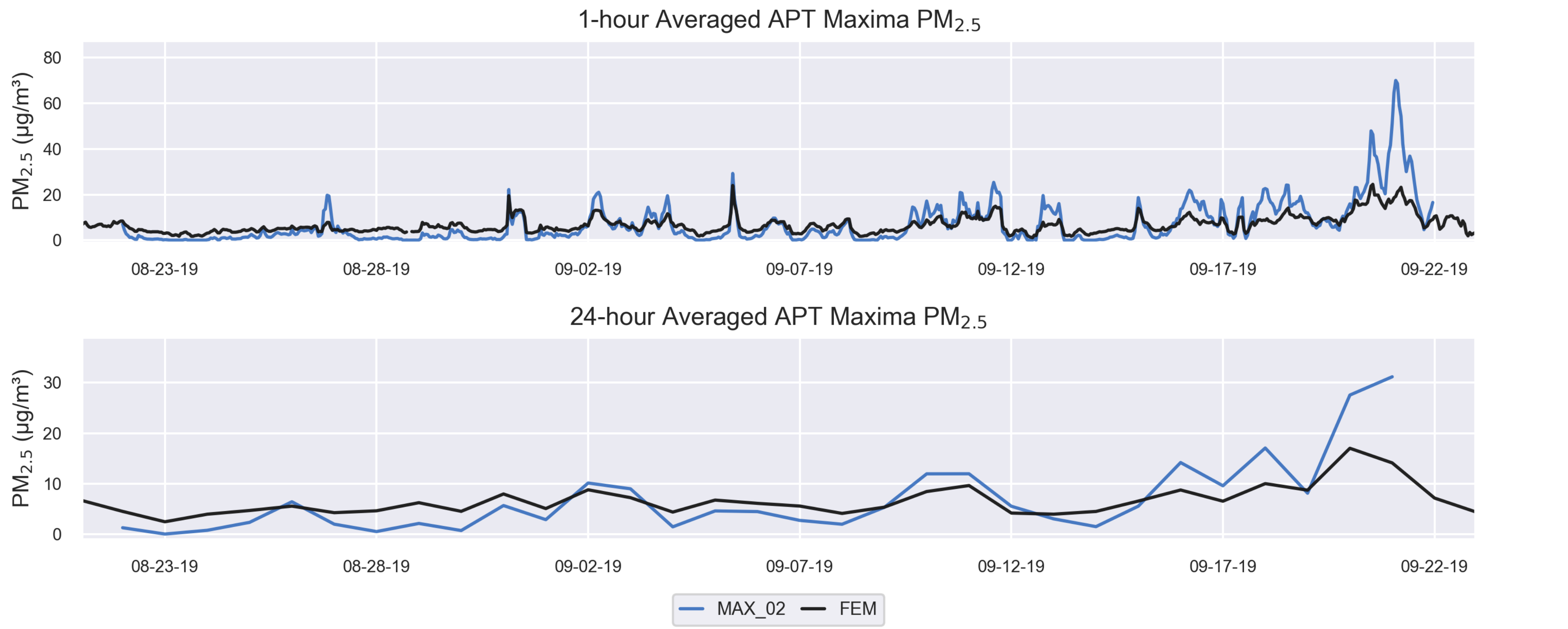

Scatter Plots: Comparison to FRM/FEM

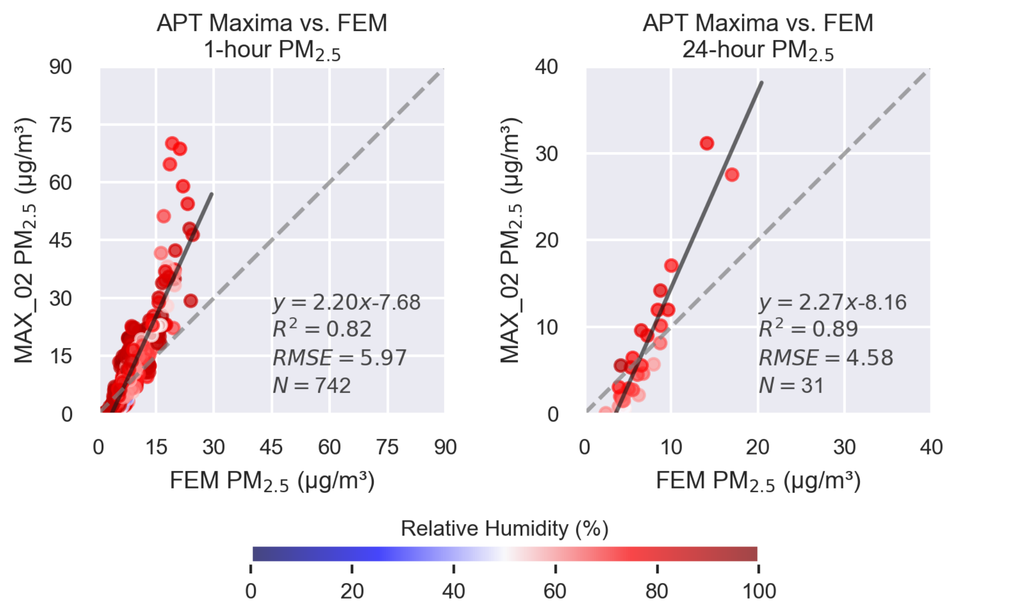

Range of FRM/FEM concentrations over duration of base test (µg/m³)	1.1-24.5 (1-hr), 2.5-17.0 (24-hr)
Number of 24-hr periods in FRM/FEM monitor measurements with a goal concentration ≥ 25 µg/m³	0

Performance Metrics

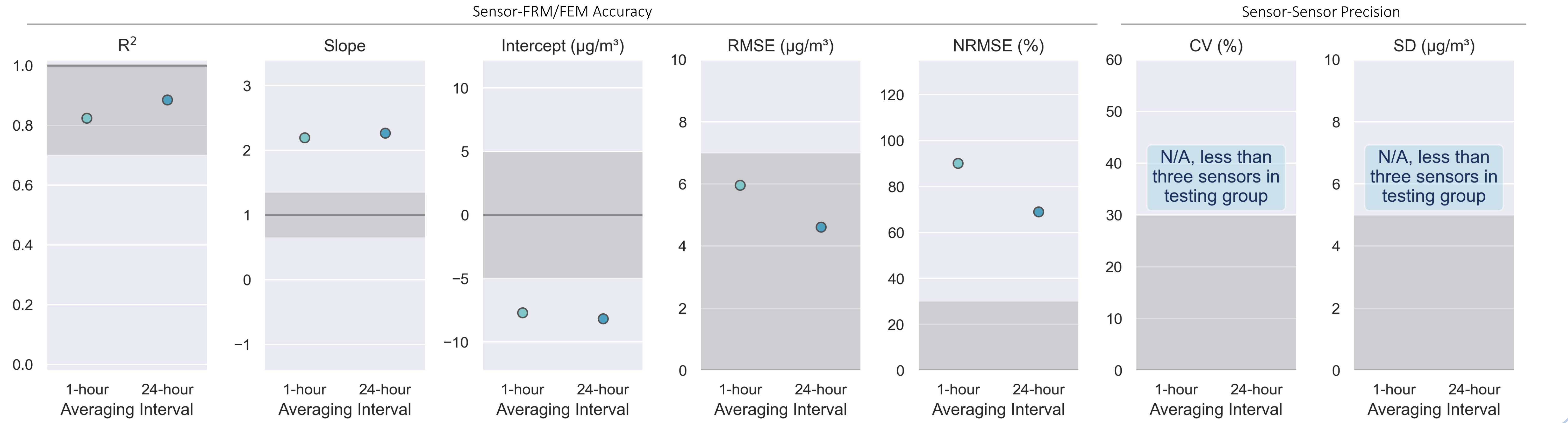

Meteorological Conditions During Deployment

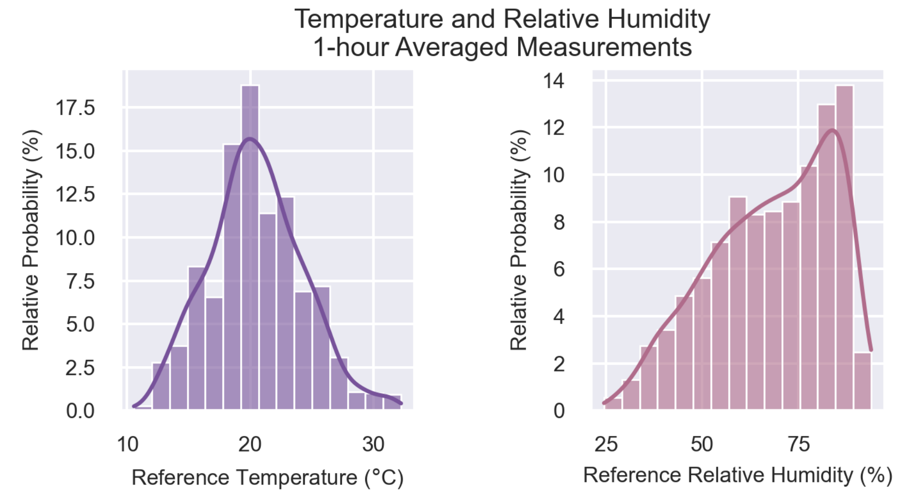

Number of 24-hr periods outside sensor manufacture-listed temperature operational range (no operational range specified)	-
Number of 24-hr periods outside sensor manufacture-listed relative humidity operational range (no operational range specified)	-

Meteorological Influence

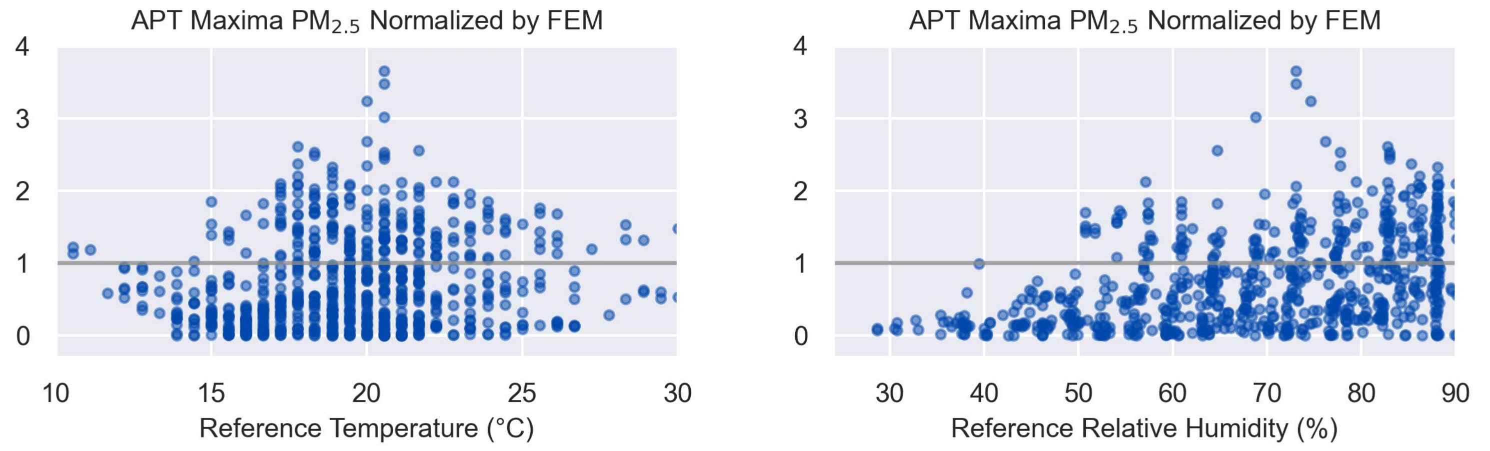

Mean number of paired, normalized concentration and temperature values (1-hr averages)	744
Mean number of paired, normalized concentration and relative humidity values (1-hr averages)	0

Testing Report - $PM_{2.5}$ Base Testing

APT Maxima

This report reflects out-of-the-box performance

Initial Base Testing - Milwaukee, WI

U.S. Environmental Protection Agency
Office of Research and Development
PI: Clements.Andrea@epa.gov
919-541-1363
August 2019—September 2019

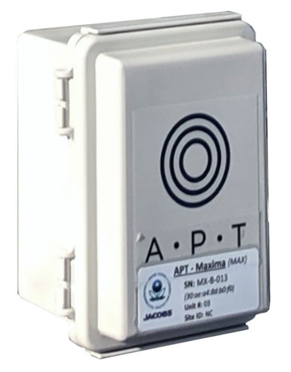

Tabular Statistics

Sensor-FRM/FEM Correlation

	Bias and Linearity						Data Quality			
	R^2		Slope		Intercept (µg/m³)		Uptime (%)		Number of paired sensor and FRM/FEM concentration values	
	1-Hour ●	24-Hour ●	1-Hour ○	24-Hour ○	1-Hour ○	24-Hour ○	1-Hour ●	24-Hour ●	1-Hour	24-Hour
Metric Target Range	≥ 0.70	≥ 0.70	1.0 ± 0.35	1.0 ± 0.35	-5 ≤ b ≤ 5	-5 ≤ b ≤ 5	75%*	75%*	-	-
Sensor MAX_02	0.82	0.89	2.20	2.27	-7.68	-8.16	100	100	742	31

	Error			
	RMSE (µg/m³)		NRMSE (%)	
	1-Hour ★	24-Hour ★	1-Hour ☆	24-Hour ☆
Metric Target Range	≤ 7.0	≤ 7.0	≤ 30.0	≤ 30.0
Deployment Value	6.0	4.6	90.1	69.0

Device-specific metrics (computed for each sensor in evaluation)
○○○ Metric value for none of devices tested falls within the target range
●○○ Metric value for one of devices tested falls within the target range
●●○ Metric value for two of devices tested falls within the target range
●●● Metric value for three of devices tested falls within the target range

Single-valued metrics (computed via entire evaluation dataset)
☆ Indicates that the metric value is not within the target range
★ Indicates that the metric value is within the target range

Sensor-Sensor Precision[1]

	Precision (between collocated sensors)				Data Quality	
	CV (%)		SD (µg/m³)		Number of concurrent sensor concentration pairs	
	1-Hour ☆	24-Hour ☆	1-Hour ☆	24-Hour ☆	1-Hour	24-Hour
Metric Target Range	≤ 30.0	≤ 30.0	≤ 5.0	≤ 5.0	-	-
Deployment Value	-	-	-	-	-	-

[1]Precision statistics are computed for evaluations with at least three collocated sensor units. Metric values are left blank for evaluations with two or fewer sensor units.

*This value is only a recommendation for ensuring data quality and is not included in the list of target values discussed in Section 4 of the Performance Testing Protocols, Metrics, and Target Values for Fine Particulate Matter Air Sensors document.

Testing Report - $PM_{2.5}$ Base Testing

APT Maxima

This report reflects out-of-the-box performance

Initial Base Testing - Milwaukee, WI
U.S. Environmental Protection Agency
Office of Research and Development
PI: Clements.Andrea@epa.gov
919-541-1363
August 2019—September 2019

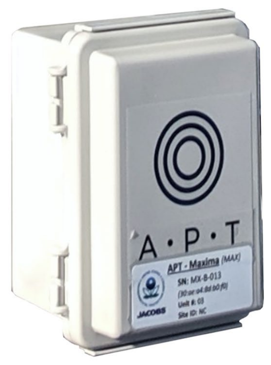

Sensor-FRM/FEM Scatter Plots

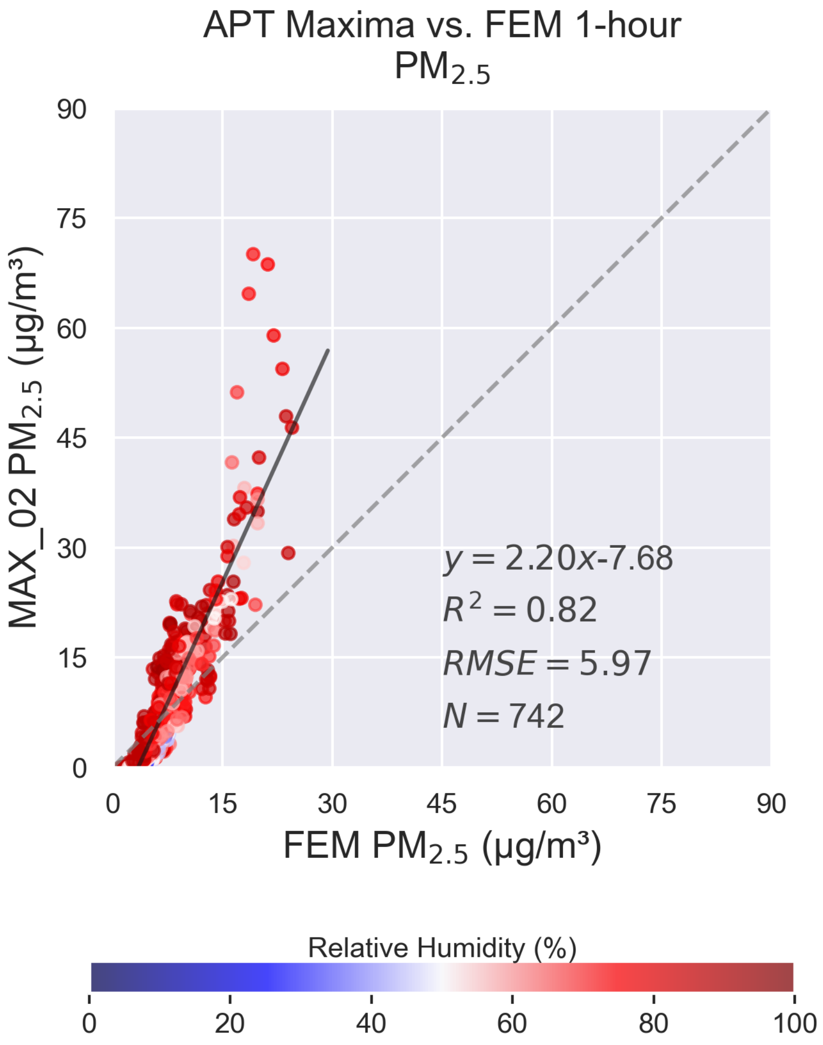

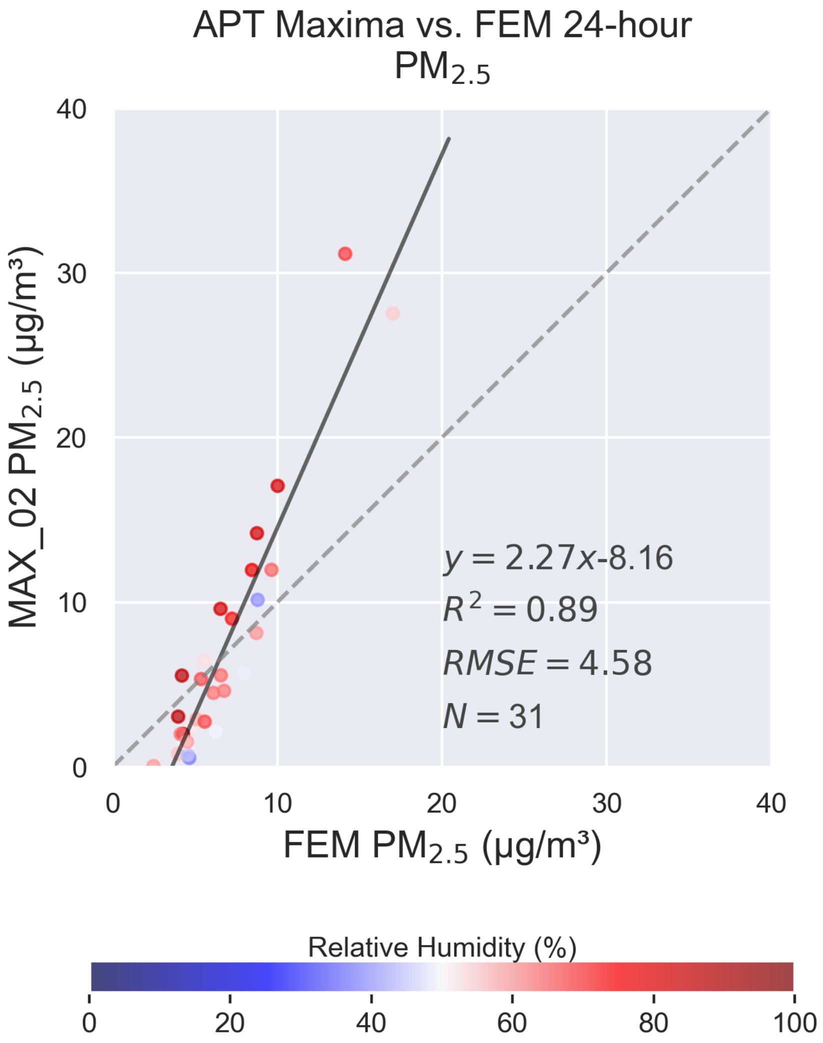

Initial Base Testing - Milwaukee, WI
U.S. Environmental Protection Agency
Office of Research and Development
PI: Clements.Andrea@epa.gov
919-541-1363
August 2019—September 2019

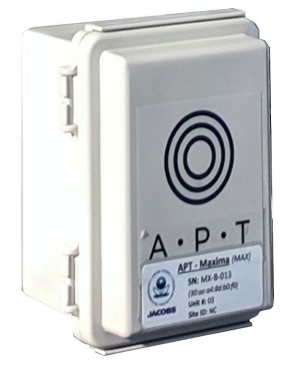

Supplemental Information

Abbreviations used in Supplemental Information

FRM	Federal Reference Method
FEM	Federal Equivalent Method
SOP	Standard Operating Procedure
QAPP	Quality Assurance Project Plan
QC	Quality Control

Supplemental Documentation	Attached	Description & URL or file path to documentation
Field observations and sensor data flags	☑	See WI-MAX-Page 6 of this testing report
Maintenance logs	☐	No logs recorded during testing
Standard operating procedure(s)	☐	U.S. EPA Office Of Research and Development SOP available upon request
Photos of equipment setup and testing	☑	See WI-MAX-Page 5 of this testing report
Product specifications sheet(s)	☐	N/A
Product manual(s)	☐	N/A
Data storage and transmission method	☑	See WI-MAX-Page 6 of this testing report
Data correction approach	☑	See WI-MAX-Page 6 of this testing report
Issues encountered	☑	See WI-MAX-Page 6 of this testing report
Data analysis/correction scripts and version	☑	Averaging and processing of data, calculation of performance metrics, and generation of figures and other supplementary material for analysis were obtained using Python 3.9.7 with the packages sensortoolkit v0.8.3b2, pandas 1.3.5, NumPy 1.21.2, Matplotlib 3.5.0, statsmodels 0.13.0, and seaborn 0.11.2. All packages are available from the Python Package Index (PyPI) at https://pypi.org. The integrated development environment (IDE) Spyder 5.1.5 was used for scripting and data visualization. Version control for the Python base, packages, and IDE were all managed by conda 4.11.0.
Air Monitoring Station QAPP	☐	U.S. EPA Office Of Research and Development QAPP available upon request
Summary of FRM/FEM monitor QC checks	☑	See WI-MAX-Page 7 of this testing report
Manufacturer website for FRM/FEM monitor	☑	Teledyne API: T640/T640X Product website
FRM/FEM monitor manual	☑	Teledyne API: T640/T640X Product Manual
FRM/FEM monitor specifications sheet(s)	☑	Teledyne API: T640/T640X Specification Sheet
Other documents	☐	

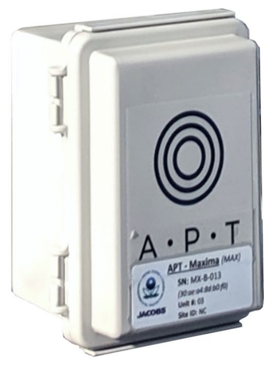

Supplemental Information: Photos of Testing Site and Equipment Setup

Site Description:

The urban site was located in the parking lot at the former WDNR Southeast Regional Headquarters located at 2300 N. Martin Luther King Blvd. Sample inlets are roughly 5 meters above ground level and 50 meters from the nearest road. Verified through annual WDNR audits, the site meets the requirements of *40 CFT 58, Appendices C, D, E and G.*

Figure 1: APT Maxima sensor (indicated by red arrow) attached to metal railing atop the sampling shelter at the monitoring site.

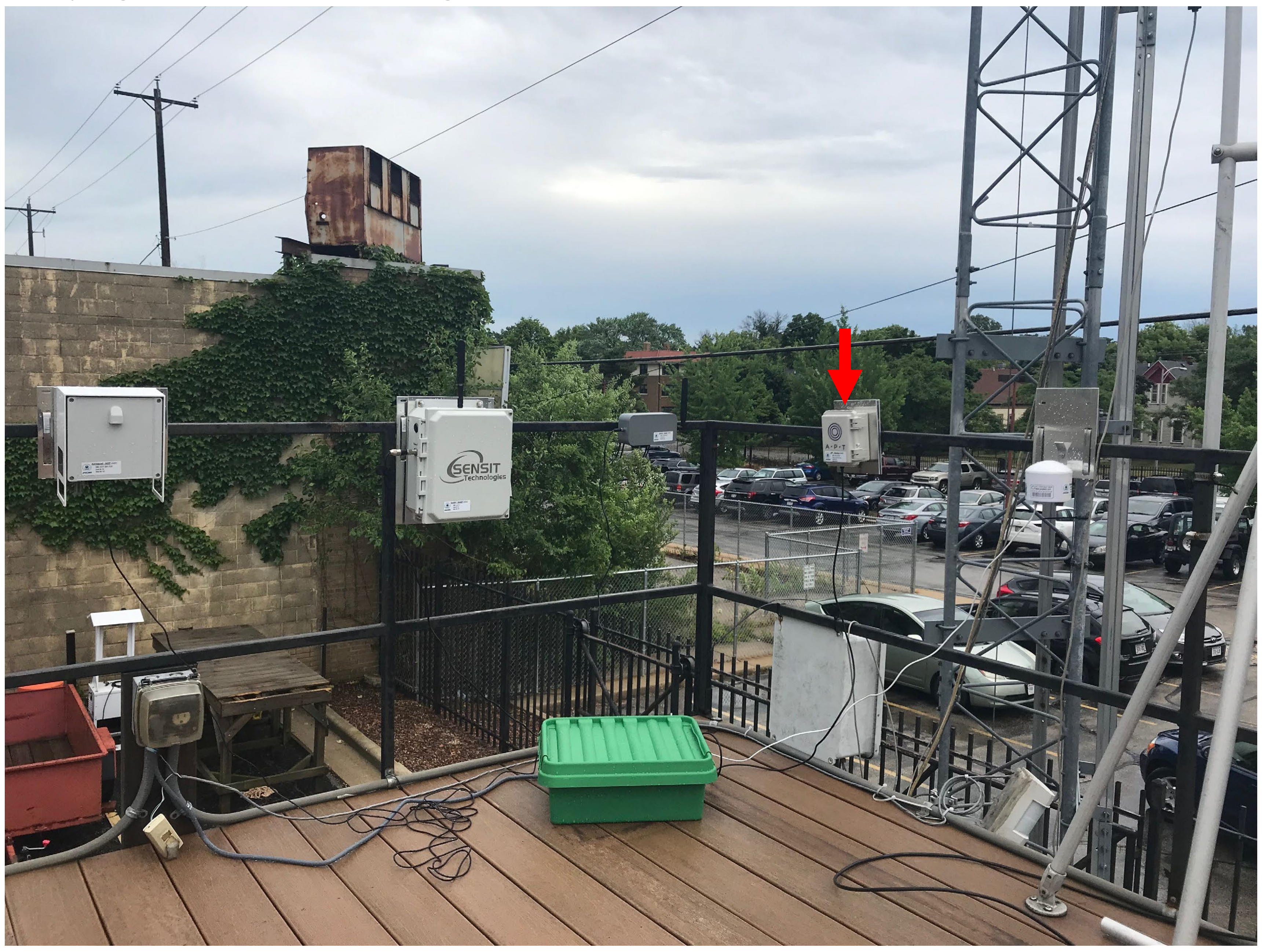

Figure 2: WDNR Monitoring Station

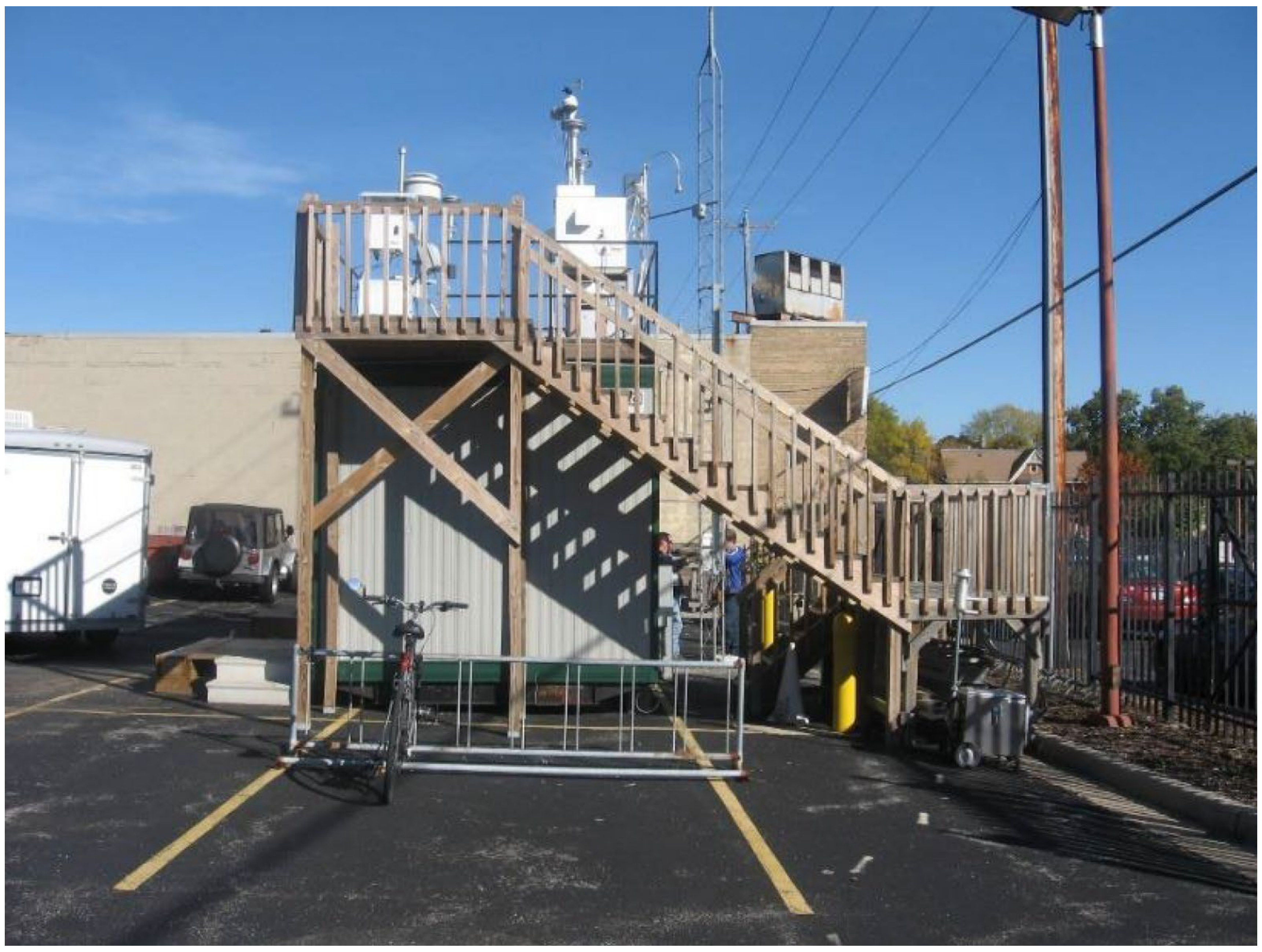

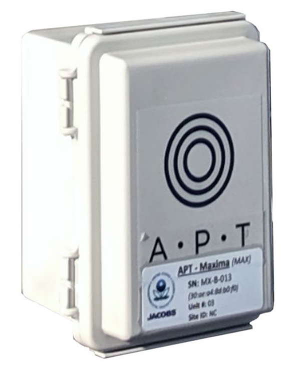

Data Storage and Transmission Method

The APT Maxima records data at 30 second intervals and stores the data on an internal microSD card and transmits data to the APT cloud server (last accessed 5/11/22) via Wi-Fi connection. The user interface for the Maxima sensors was under development during sensor testing, thus weekly raw data files were obtained from the internal MicroSD card. Each field site operator was provided two labeled MicroSD cards for the Maxima that they used to swap out each week. Data from the collected card was then read and processed off-site.

Data Correction Approach

This evaluation report reflects “out-of-the-box” performance of the APT Maxima. The Maxima data sets include two data columns contain $PM_{2.5}$ concentration measurements, “PM2.5(ug/m3)” and “PM2.5_Atm(ug/m3)”. These columns correspond to separate correction factors provided by the onboard OEM PM sensor (Plantower PMSA003). For this report, $PM_{2.5}$ measurements for the column labeled “PM2.5(ug/m3)” were selected for analysis.

After acquisition, the raw data was processed using the *sensortoolkit* python code library (v0.8.3b2). A continuous data set at the recorded sampling frequency was written to a .csv file. 1-hour and 24-hour averaged data sets were generated using a 75% completeness threshold and saved as separate .csv files. Outliers were NOT removed from data sets in order to assess “out-of-the-box” sensor performance.

Issues Encountered

<u>Pre-deployment observations</u>

- *Sampling internal abnormality:* Maxima devices did not continuously sample at 30-second intervals as stated in the sensor user guide. Instead, Maxima units occasionally sampled at 31 seconds. This increased sample interval is due to the accumulation of processing time over a certain number of samples. Each 30-second sample is a collection of exactly 30 seconds of sampling followed by a brief fraction of a second used for processing the collected data. Once processed, the next 30-second data collection period begins. When the sum of the processing time reaches one full second, the sample interval then jumps to 31 seconds. Data from this pre-deployment testing indicated that the processing speed for each sensor varies such that 31-second sampling duration occur at random intervals across Maxima units.
- *Faulty battery connection:* The initially deployed MAX sensor pod would not operate consistently (the battery connections on this device were faulty and required repair) and it was removed from sampling and replaced with a similar unit. The initial Maxima unit was removed from the monitoring site on 8/13/2019 following deployment on 7/22/2019. The replacement unit (referred to in this report as MAX_01) was deployed at the site on 8/9/2019.

<u>Field observations and sensor data flags</u>

During the testing period included in this report (08/22/19 to 09/21/19), the Maxima unit operated nominally and did not require replacement or repair.

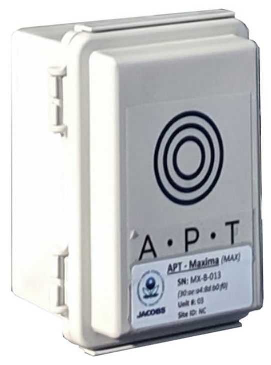

Supplemental Information: Description of FRM/FEM QC Checks and Data Flags

Description of Data Flags

AQS

The U.S. EPA's Air Quality System (AQS) is the Agency's primary ambient air monitoring data archive. A comprehensive list of data flags that are recorded alongside AQS data sets, referred to by U.S. EPA as 'qualifiers', can be found at the following link: https://aqs.epa.gov/aqsweb/documents/codetables/qualifiers.html

Invalidation of reference data: AQS qualifiers are organized by qualifier type, which indicates whether data logged alongside qualifier flags should be invalidated (set null). Qualifiers with type "Null Data Qualifier" are invalidated, and includes data logged during periods that coincide with QC checks (e.g., "BF-Precision/Zero/Span", "BJ- Operator Error", "BL - QA Audit", "AZ - QC Audit") among other events such as power outages. Data logged alongside qualifiers with type "Quality Assurance Qualifiers" are not invalidated and are included in this analysis (e.g., concentrations less than the federal MDL for the reference monitor "MD – Value less than MDL", QA reviewed values "Validated Value").

Data Flags Recorded During Testing

FRM/FEM Monitor	Timestamp (UTC)	Flag
Teledyne API T640X<br>(Data acquired from AQS)	2019-08-28 18:00:00+0000 to 2019-08-28 19:00:00+0000	AX - Precision Check

Testing Report - $PM_{2.5}$ Base Testing

Clarity Node

This report reflects out-of-the-box performance

Initial Base Testing - Milwaukee, WI

U.S. Environmental Protection Agency
Office of Research and Development
PI: Clements.Andrea@epa.gov
919-541-1363

July 2019—August 2019

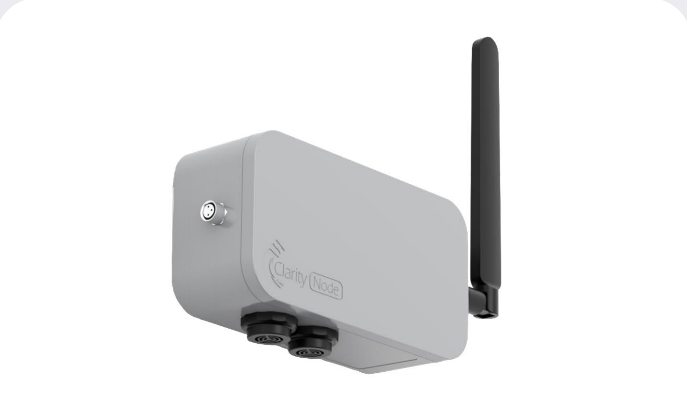

Image Attribution: Clarity Movement Co. (Berkeley, CA)

Deployment Details

Testing Organization and Site Information	
Testing organization (Name, Organization type, Contact website)	U.S. Environmental Protection Agency - Office of Research and Development<br>Federal Government<br>Air Sensor Toolbox | U.S. EPA Website
Testing location (City, State, Latitude and Longitude)	WDNR Headquarters<br>Milwaukee, WI<br>43.061, -87.9135
AQS site ID	55 - 079 - 0026
Sampling timeframe (MM-DD-YY)	07-26-19 to 08-25-19
Sensor data source	Clarity Dashboard download
Reference data source	AQS API download

Sensor Information			
Manufacturer, model	Clarity Node		
Device firmware version	Received by EPA May 2019		
Sampling time interval	3-minutes		
Sensor serial numbers	CNO_01		
Issues encountered during deployment?	☑	See WI-CNO-Page 6 of this testing report	

FRM/FEM Information	
Manufacturer, model, designation	Teledyne Advanced Pollution Instrumentation T640X FEM
Sampling time interval	1-hour averaging
Date of calibration	As required by 40 CFR Part 58 and the Low Volume $PM_{2.5}$ and PM_{10} Networks QAPP maintained by WDNR
Date of flowrate verification check	Monthly as required by 40 CFR Part 58 Appendix A
Description, date(s) of maintenance activities	N/A

Time Series Plots

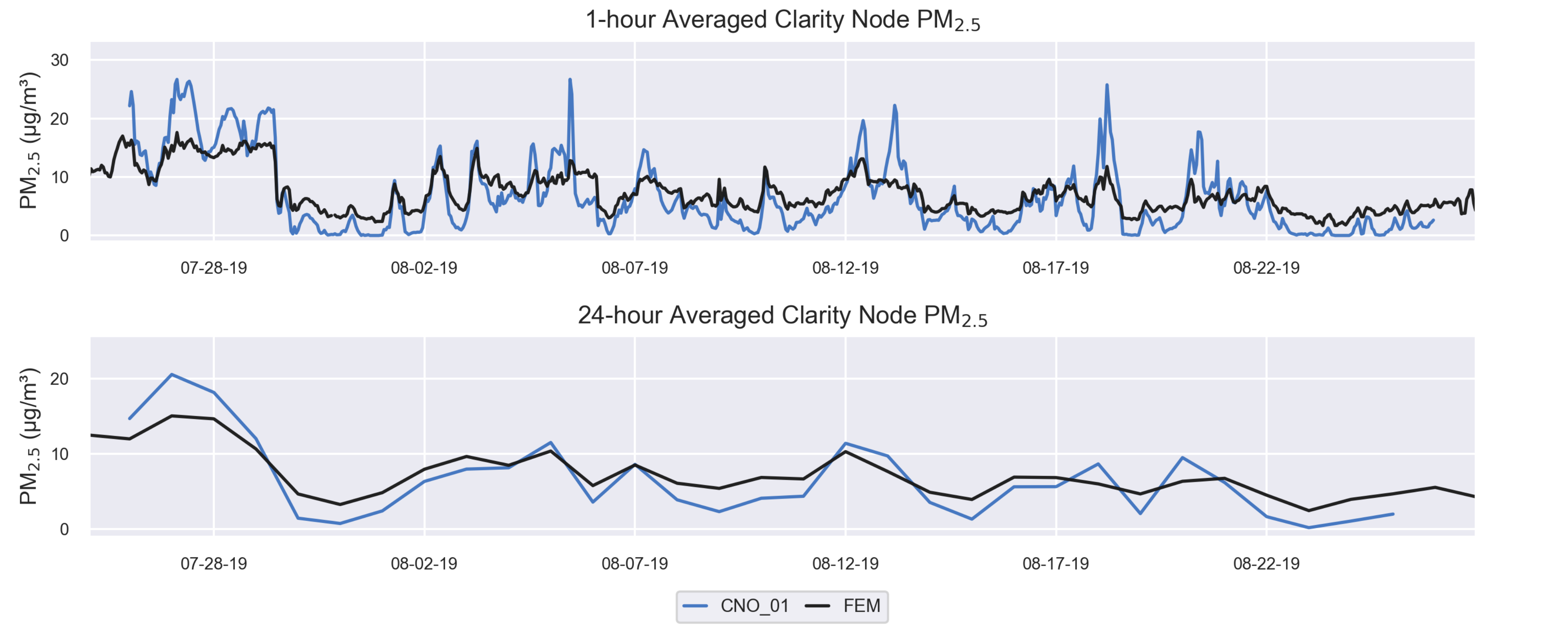

Scatter Plots: Comparison to FRM/FEM

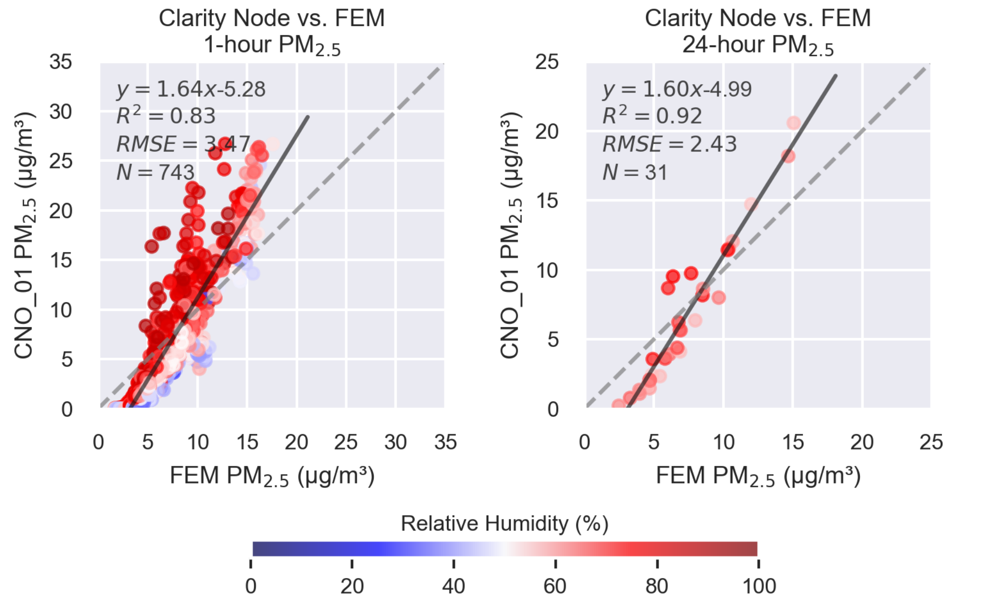

Range of FRM/FEM concentrations over duration of base test (µg/m³)	1.7-17.6 (1-hr),<br>2.5-15.1 (24-hr)
Number of 24-hr periods in FRM/FEM monitor measurements with a goal concentration ≥ 25 µg/m³	0

Performance Metrics

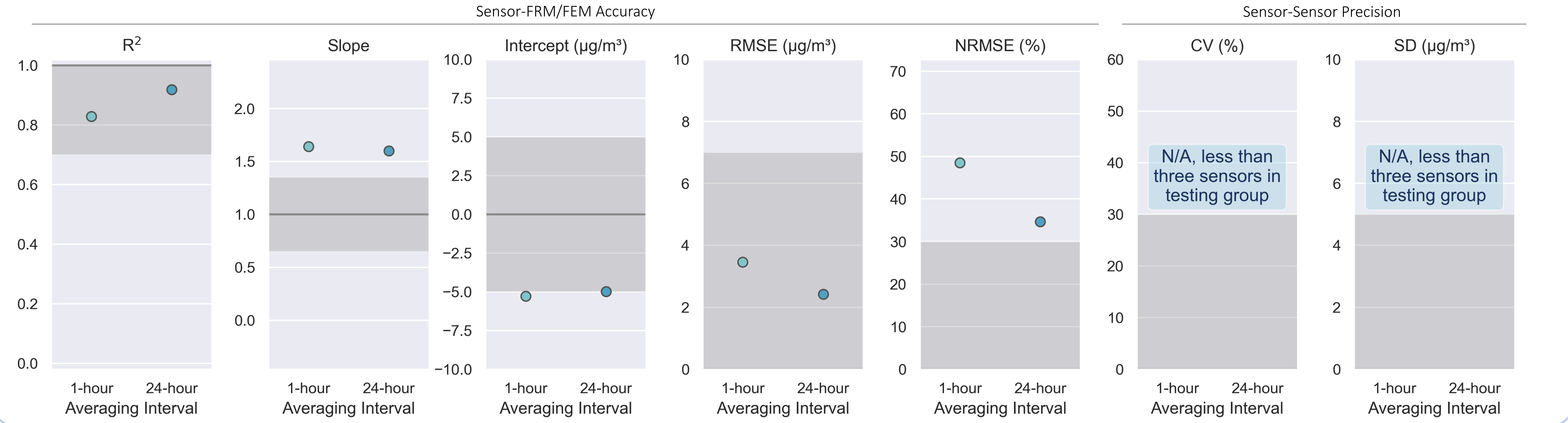

Meteorological Conditions During Deployment

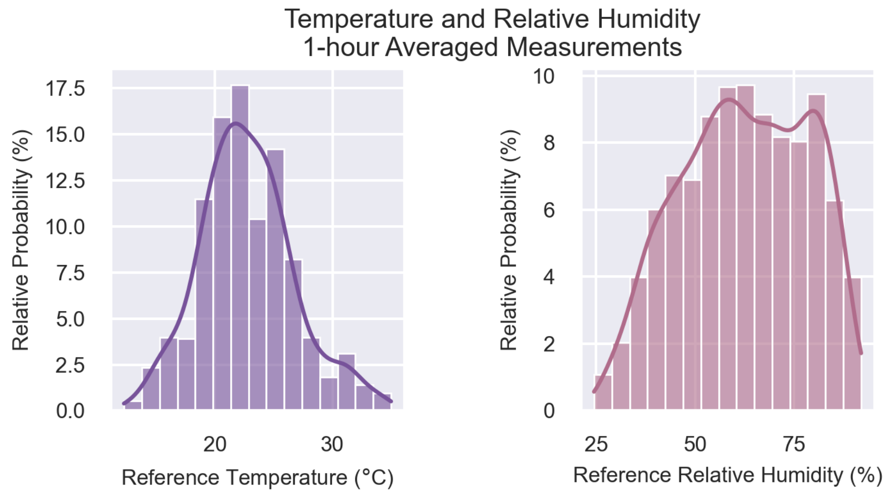

Number of 24-hr periods outside sensor manufacture-listed temperature operational range (-10 to 55 °C)	0
Number of 24-hr periods outside sensor manufacture-listed relative humidity operational range (no operational range specified)	-

Meteorological Influence

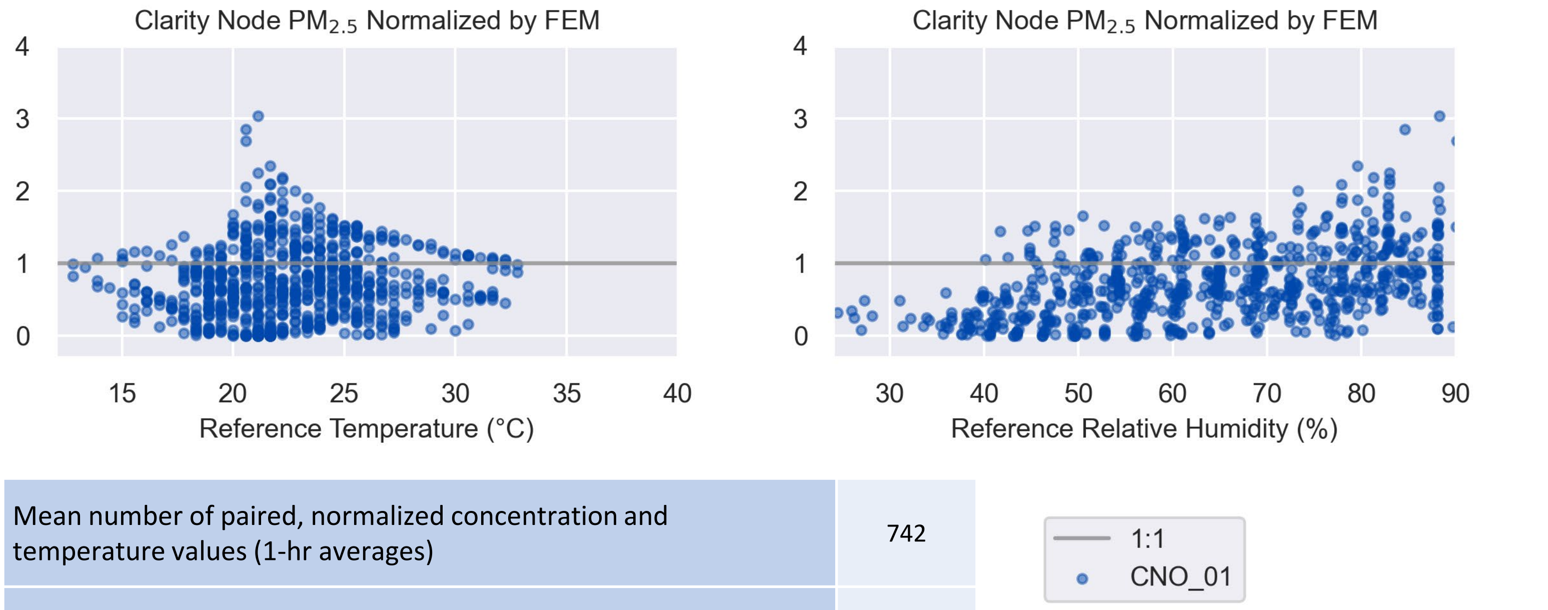

Mean number of paired, normalized concentration and temperature values (1-hr averages)	742
Mean number of paired, normalized concentration and relative humidity values (1-hr averages)	0

Testing Report - $PM_{2.5}$ Base Testing

Clarity Node

This report reflects out-of-the-box performance

Initial Base Testing - Milwaukee, WI
U.S. Environmental Protection Agency
Office of Research and Development
PI: Clements.Andrea@epa.gov
919-541-1363
July 2019—August 2019

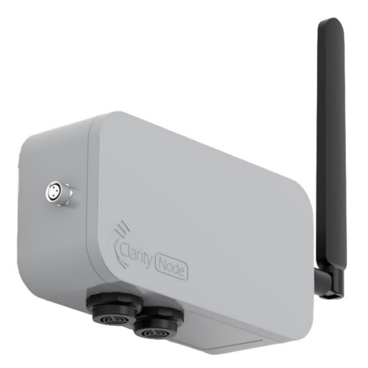

Image Attribution: Clarity Movement Co. (Berkeley, CA)

Tabular Statistics

Sensor-FRM/FEM Correlation

	Bias and Linearity						Data Quality			
	R^2		Slope		Intercept (µg/m³)		Uptime (%)		Number of paired sensor and FRM/FEM concentration values	
	1-Hour ●	24-Hour ●	1-Hour ○	24-Hour ○	1-Hour ○	24-Hour ●	1-Hour ●	24-Hour ●	1-Hour	24-Hour
Metric Target Range	≥ 0.70	≥ 0.70	1.0 ± 0.35	1.0 ± 0.35	-5 ≤ b ≤ 5	-5 ≤ b ≤ 5	75%*	75%*	-	-
Sensor CNO_01	0.83	0.92	1.64	1.60	-5.28	-4.99	100	100	743	31

	Error			
	RMSE (µg/m³)		NRMSE (%)	
	1-Hour ★	24-Hour ★	1-Hour ☆	24-Hour ☆
Metric Target Range	≤ 7.0	≤ 7.0	≤ 30.0	≤ 30.0
Deployment Value	3.5	2.4	48.5	34.7

Device-specific metrics (computed for each sensor in evaluation)
○○○ Metric value for none of devices tested falls within the target range
●○○ Metric value for one of devices tested falls within the target range
●●○ Metric value for two of devices tested falls within the target range
●●● Metric value for three of devices tested falls within the target range

Single-valued metrics (computed via entire evaluation dataset)
☆ Indicates that the metric value is not within the target range
★ Indicates that the metric value is within the target range

Sensor-Sensor Precision[1]

	Precision (between collocated sensors)				Data Quality	
	CV (%)		SD (µg/m³)		Number of concurrent sensor concentration pairs	
	1-Hour ☆	24-Hour ☆	1-Hour ☆	24-Hour ☆	1-Hour	24-Hour
Metric Target Range	≤ 30.0	≤ 30.0	≤ 5.0	≤ 5.0	-	-
Deployment Value	-	-	-	-	-	-

[1]Precision statistics are computed for evaluations with at least three collocated sensor units. Metric values are left blank for evaluations with two or fewer sensor units.

*This value is only a recommendation for ensuring data quality and is not included in the list of target values discussed in Section 4 of the Performance Testing Protocols, Metrics, and Target Values for Fine Particulate Matter Air Sensors document.

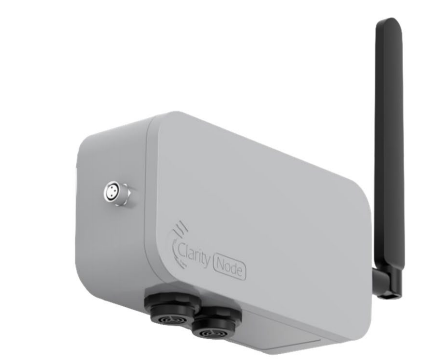

Image Attribution: Clarity Movement Co. (Berkeley, CA)

Sensor-FRM/FEM Scatter Plots

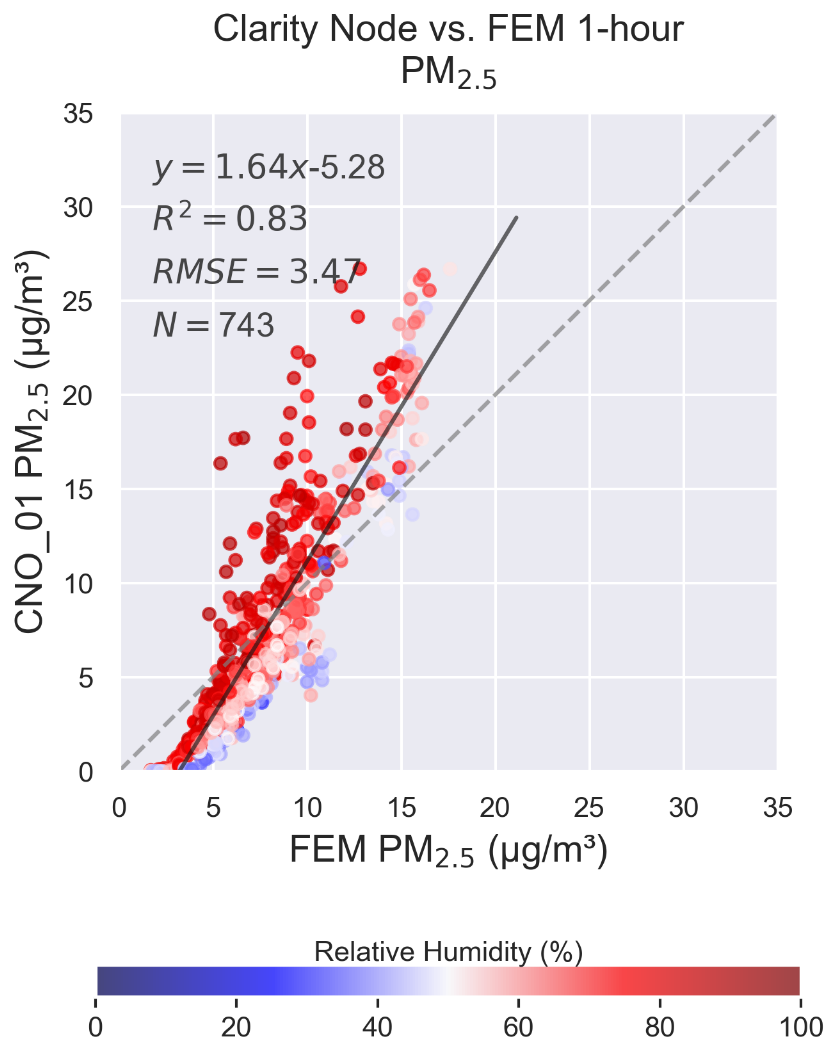

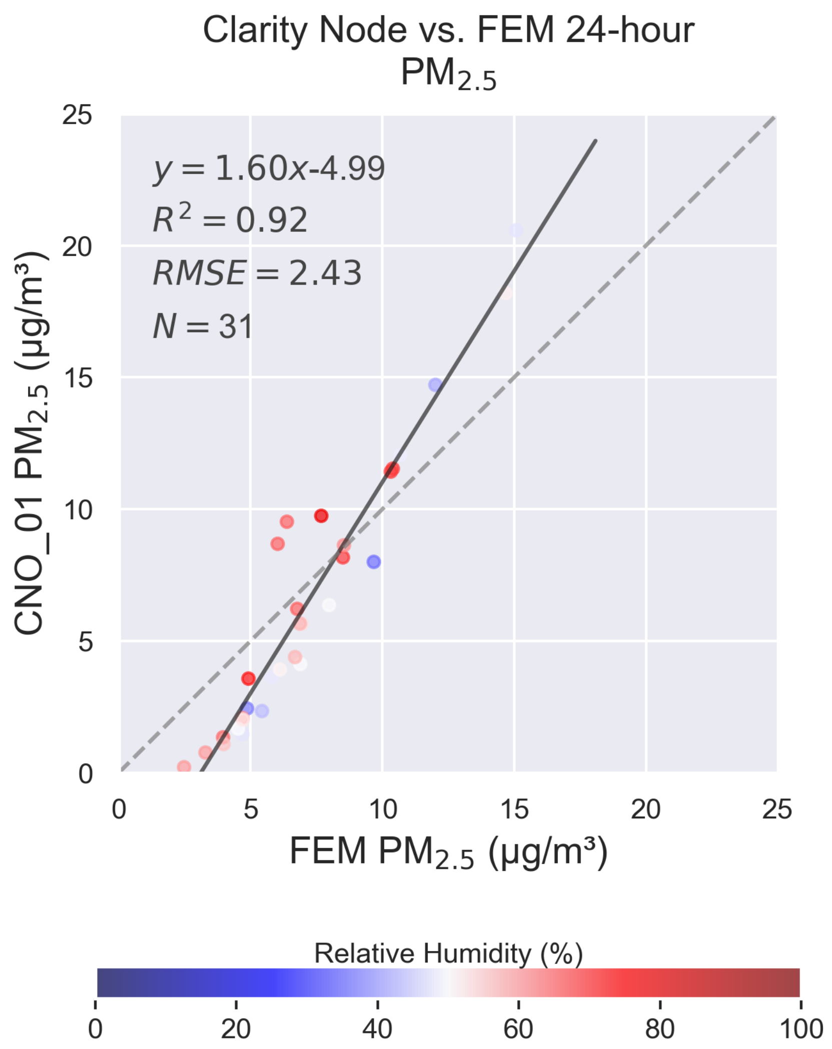

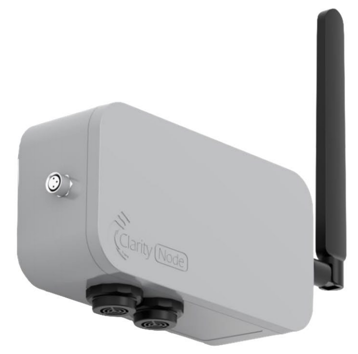

Image Attribution: Clarity Movement Co. (Berkeley, CA)

Supplemental Information

Abbreviations used in Supplemental Information

FRM	Federal Reference Method
FEM	Federal Equivalent Method
SOP	Standard Operating Procedure
QAPP	Quality Assurance Project Plan
QC	Quality Control

Supplemental Documentation	Attached	Description & URL or file path to documentation
Field observations and sensor data flags	☑	See WI-CNO-Page 6 of this testing report
Maintenance logs	☐	No logs recorded during testing
Standard operating procedure(s)	☐	U.S. EPA Office Of Research and Development SOP available upon request
Photos of equipment setup and testing	☑	See WI-CNO-Page 5 of this testing report
Product specifications sheet(s)	☐	N/A
Product manual(s)	☐	N/A
Data storage and transmission method	☑	See WI-CNO-Page 6 of this testing report
Data correction approach	☑	See WI-CNO-Page 6 of this testing report
Issues encountered	☑	See WI-CNO-Page 6 of this testing report
Data analysis/correction scripts and version	☑	Averaging and processing of data, calculation of performance metrics, and generation of figures and other supplementary material for analysis were obtained using Python 3.9.7 with the packages sensortoolkit v0.8.3b2, pandas 1.3.5, NumPy 1.21.2, Matplotlib 3.5.0, statsmodels 0.13.0, and seaborn 0.11.2. All packages are available from the Python Package Index (PyPI) at https://pypi.org. The integrated development environment (IDE) Spyder 5.1.5 was used for scripting and data visualization. Version control for the Python base, packages, and IDE were all managed by conda 4.11.0.
Air Monitoring Station QAPP	☐	U.S. EPA Office Of Research and Development QAPP available upon request
Summary of FRM/FEM monitor QC checks	☑	See WI-CNO-Page 7 of this testing report
Manufacturer website for FRM/FEM monitor	☑	Teledyne API: T640/T640X Product website
FRM/FEM monitor manual	☑	Teledyne API: T640/T640X Product Manual
FRM/FEM monitor specifications sheet(s)	☑	Teledyne API: T640/T640X Specification Sheet
Other documents	☐	

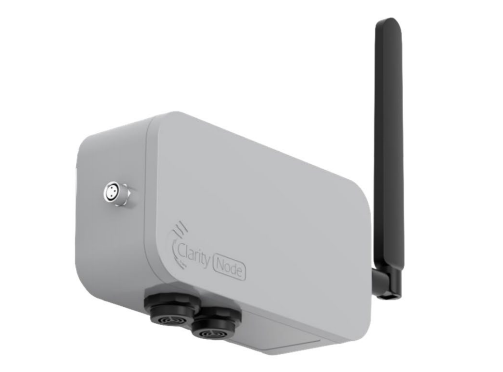

Image Attribution: Clarity Movement Co. (Berkeley, CA)

Supplemental Information: Photos of Testing Site and Equipment Setup

Site Description:

The urban site was located in the parking lot at the former WDNR Southeast Regional Headquarters located at 2300 N. Martin Luther King Blvd. Sample inlets are roughly 5 meters above ground level and 50 meters from the nearest road. Verified through annual WDNR audits, the site meets the requirements of *40 CFT 58, Appendices C, D, E and G.*

Figure 1: Clarity Node sensor (indicated by red arrow) attached to metal railing atop the sampling shelter at the monitoring site.

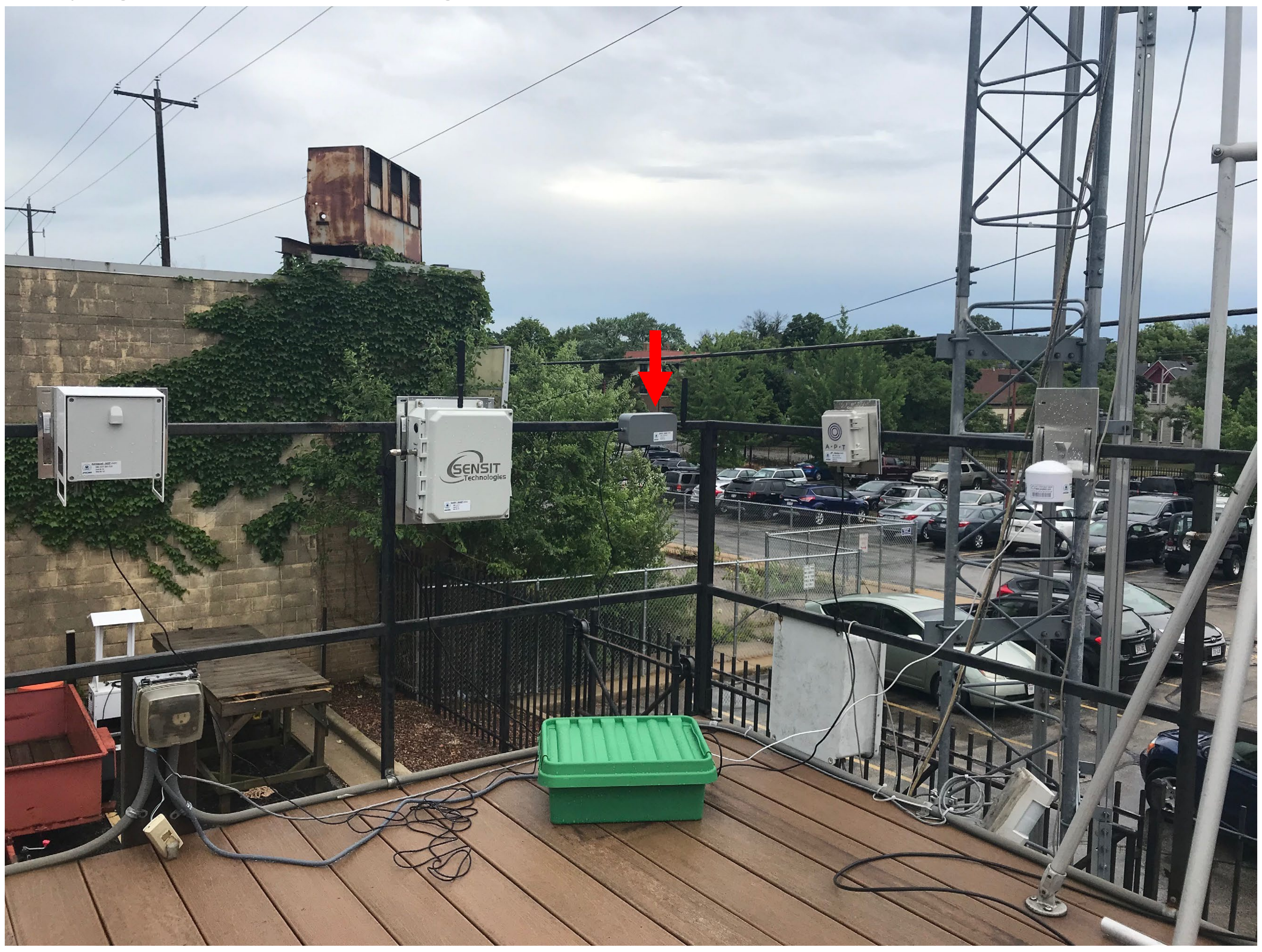

Figure 2: WDNR Monitoring Station

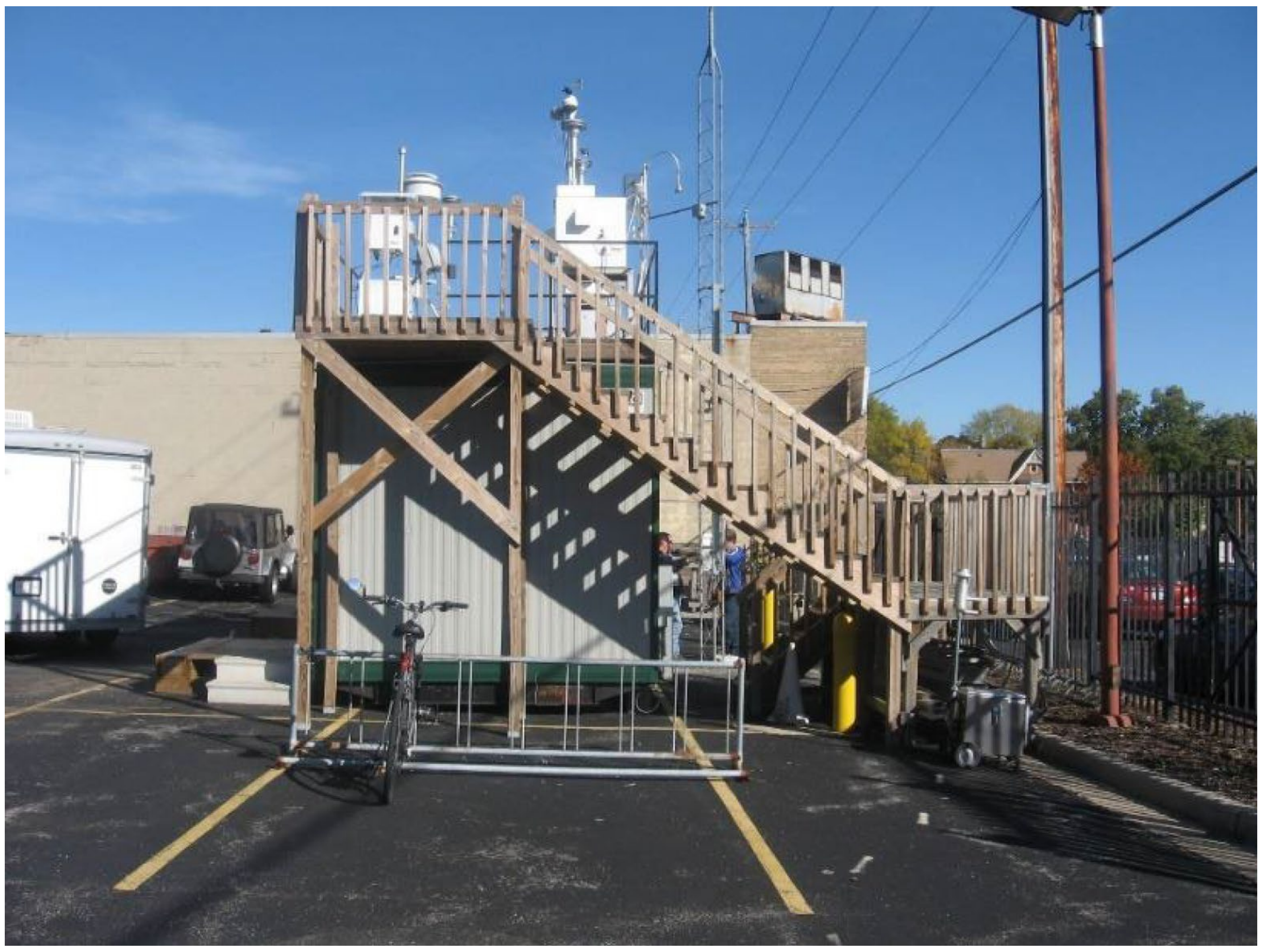

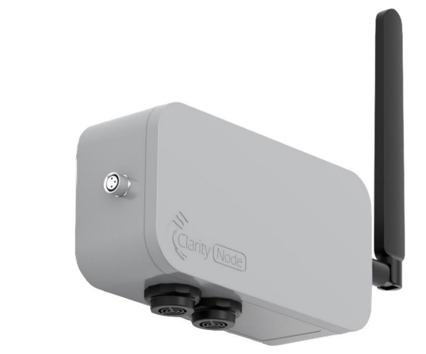
Image Attribution: Clarity Movement Co. (Berkeley, CA)

Supplemental Information: Data Storage, Correction Approach, and Issues Encountered

Data Storage and Transmission Method

The Clarity Node transmits data via cellular SIM card. Weekly raw data collections were performed over Wi-Fi using the Clarity dashboard (*last accessed 5/11/22*). The observed sampling interval fluctuated and is further discussed under pre-deployment observations below. On average, the Node models sampled every 2-3 minutes, and the Node-S sampled every 15-17 minutes.

Data Correction Approach

This evaluation report reflects "out-of-the-box" performance of the Clarity Node. The manufacturer provides a dashboard feature for calibrating sensor data against collocated reference monitors. Calibrated data were NOT used for this evaluation. Prospective consumers may get better performance from this device if they utilize this feature.

After acquisition, the raw data was processed using the *sensortoolkit* python code library (v0.8.3b2). A continuous data set at the recorded sampling frequency was written to a .csv file. 1-hour and 24-hour averaged data sets were generated using a 75% completeness threshold and saved as separate .csv files. Due to the variable sampling interval observed for Clarity Node units, 75% completeness was determined against the number of data points that would be recorded within 1-hour/24-hour periods if the sensor were to log measurements at the most commonly recorded sampling interval (i.e., the mode of recorded intervals). Outliers were NOT removed from data sets in order to assess "out-of-the-box" sensor performance.

Issues Encountered

Pre-deployment observations

- *Unsteady sampling interval:* Clarity Node sensors were received by EPA on May 28, 2019. A lab bench-top evaluation was performed from May 29, 2019 – May 31, 2019. During bench-top testing, it was observed that the sampling intervals noted by Clarity (5 minutes for non-solar Node, 15 minutes for the solar Node-S) were not reflective of recorded intervals. Communication with the vendor concluded that the sampling interval will fluctuate depending on the settings, battery charge, and strength of the cellular signal and that this sampling interval cannot be adjusted by the user at present. On average, the Node models sampled every 2-3 minutes, and the Node-S sampled every 15-17 minutes.

Field observations and sensor data flags

Throughout the testing period, numerous sampling interval abnormality flags were logged, associated with an occasional change in the sampling frequency of the Clarity Node. As noted during pre-deployment testing, the Node typically samples at 2 to 3-minute intervals. Occasionally, the device recorded samples at longer intervals ranging from 4 to 10 minutes, followed by a period of alternating 2 to 3-minute sampling intervals. This longer-duration sampling interval repeatedly occurred at seemingly random intervals. This change in sampling frequency was not a result of unit configuration changes applied by a field technician and reflect the out-of-the-box dynamic sampling interval as programmed by the manufacturer.

The following table contains data flags describing events that were encountered during the testing period.

Start Time (UTC)	End Time (UTC)	Sensor Serial ID	Parameters Impacted	Flag
2019-08-09 12:51:00+00:00	2019-08-16 11:46:00+00:00	CNO_01	ALL	6.0-Sampling interval abnormality
2019-08-16 11:41:00+00:00	2019-08-16 11:59:00+00:00	CNO_01	ALL	6.0-Sampling interval abnormality
2019-08-23 11:54:00+00:00	2019-08-23 12:10:00+00:00	CNO_01	ALL	6.0-Sampling interval abnormality

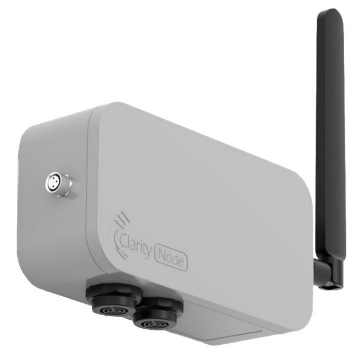

Image Attribution: Clarity Movement Co. (Berkeley, CA)

Description of Data Flags

AQS

The U.S. EPA's Air Quality System (AQS) is the Agency's primary ambient air monitoring data archive. A comprehensive list of data flags that are recorded alongside AQS data sets, referred to by U.S. EPA as 'qualifiers', can be found at the following link: https://aqs.epa.gov/aqsweb/documents/codetables/qualifiers.html

Invalidation of reference data: AQS qualifiers are organized by qualifier type, which indicates whether data logged alongside qualifier flags should be invalidated (set null). Qualifiers with type "Null Data Qualifier" are invalidated, and includes data logged during periods that coincide with QC checks (e.g., "BF-Precision/Zero/Span", "BJ- Operator Error", "BL - QA Audit", "AZ - QC Audit") among other events such as power outages. Data logged alongside qualifiers with type "Quality Assurance Qualifiers" are not invalidated and are included in this analysis (e.g., concentrations less than the federal MDL for the reference monitor "MD – Value less than MDL", QA reviewed values "Validated Value").

Data Flags Recorded During Testing

FRM/FEM Monitor	Timestamp (UTC)	Flag
Teledyne API T640X<br>(Data acquired from AQS)	2019-07-30 20:00:00+0000	AX - Precision Check

Meteorological Instrument	Timestamp (UTC)	Flag
MetOne Temperature Monitor<br>(Data acquired from AQS)	2019-08-07 16:00:00+0000 to 2019-08-07 17:00:00+0000	AZ - QC Audit

Testing Report - $PM_{2.5}$ Base Testing

PurpleAir PA-II-SD

This report reflects out-of-the-box performance

Initial Base Testing - Milwaukee, WI

U.S. Environmental Protection Agency
Office of Research and Development
PI: Clements.Andrea@epa.gov
919-541-1363

July 2019—August 2019

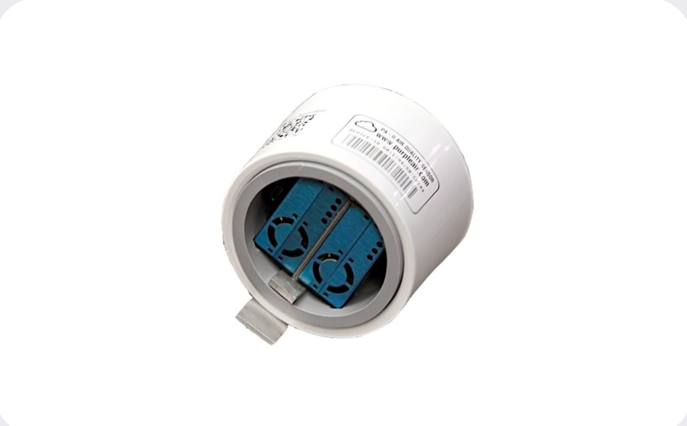

Deployment Details

Testing Organization and Site Information	
Testing organization (Name, Organization type, Contact website)	U.S. Environmental Protection Agency - Office of Research and Development<br>Federal Government<br>Air Sensor Toolbox | U.S. EPA Website
Testing location (City, State, Latitude and Longitude)	WDNR Headquarters<br>Milwaukee, WI<br>43.061, -87.9135
AQS site ID	55 - 079 - 0026
Sampling timeframe (MM-DD-YY)	07-24-19 to 08-23-19
Sensor data source	Onboard MicroSD card
Reference data source	AQS API download

Sensor Information			
Manufacturer, model	PurpleAir PA-II-SD		
Device firmware version	V4.02		
Sampling time interval	2-minutes		
Sensor serial numbers	PA01		
Issues encountered during deployment?	☐	No Issues	

FRM/FEM Information	
Manufacturer, model, designation	Teledyne Advanced Pollution Instrumentation T640X FEM
Sampling time interval	1-hour averaging
Date of calibration	As required by 40 CFR Part 58 and the Low Volume $PM_{2.5}$ and PM_{10} Networks QAPP maintained by WDNR
Date of flowrate verification check	Monthly as required by 40 CFR Part 58 Appendix A
Description, date(s) of maintenance activities	N/A

Time Series Plots

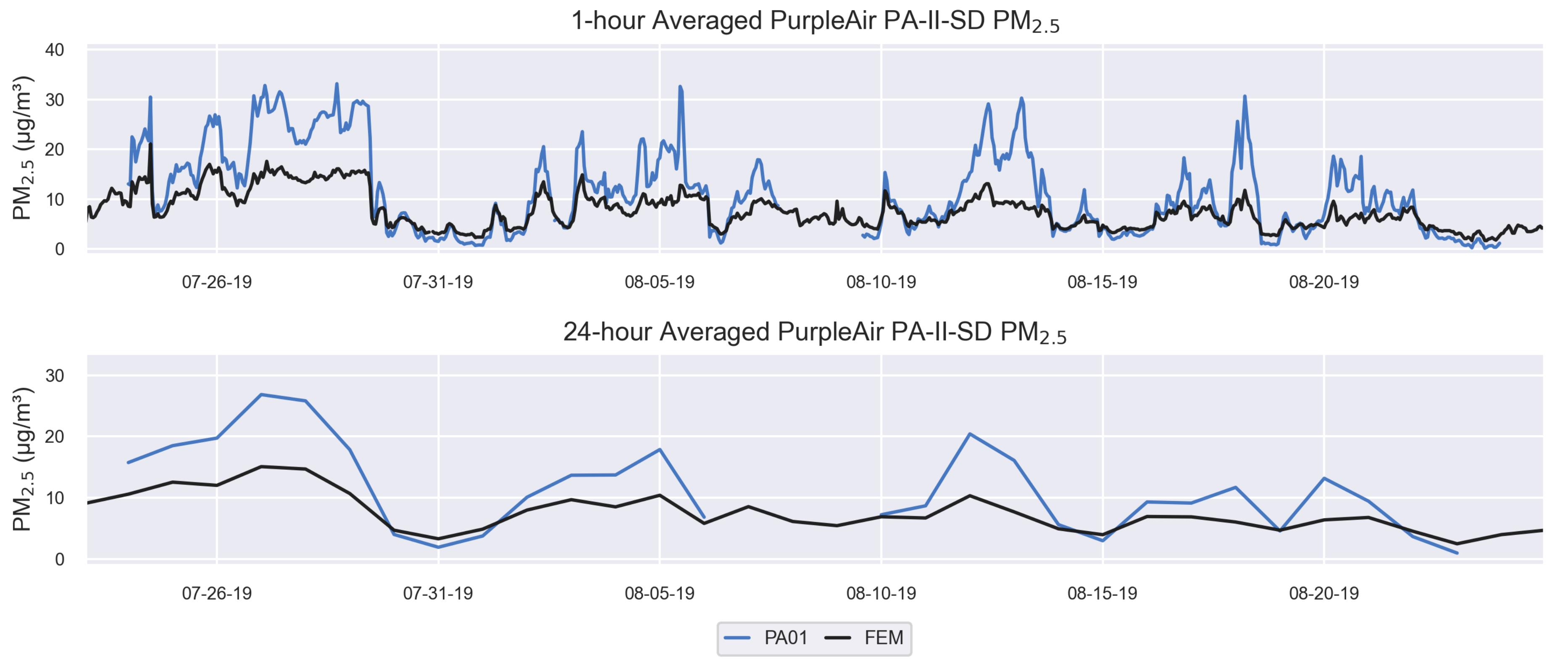

Scatter Plots: Comparison to FRM/FEM

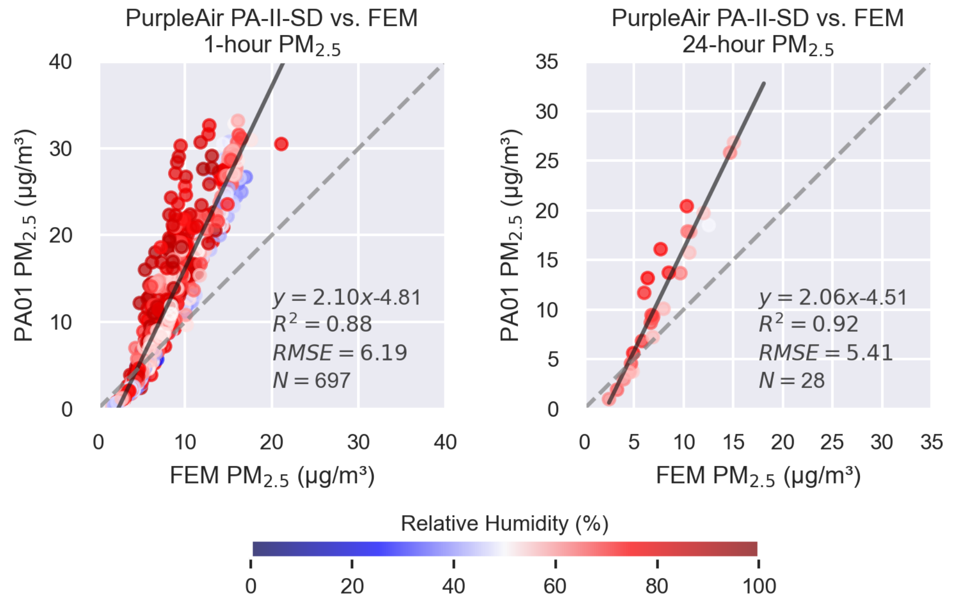

Range of FRM/FEM concentrations over duration of base test (µg/m³)	1.7-21.1 (1-hr), 2.5-15.1 (24-hr)
Number of 24-hr periods in FRM/FEM monitor measurements with a goal concentration ≥ 25 µg/m³	0

Performance Metrics

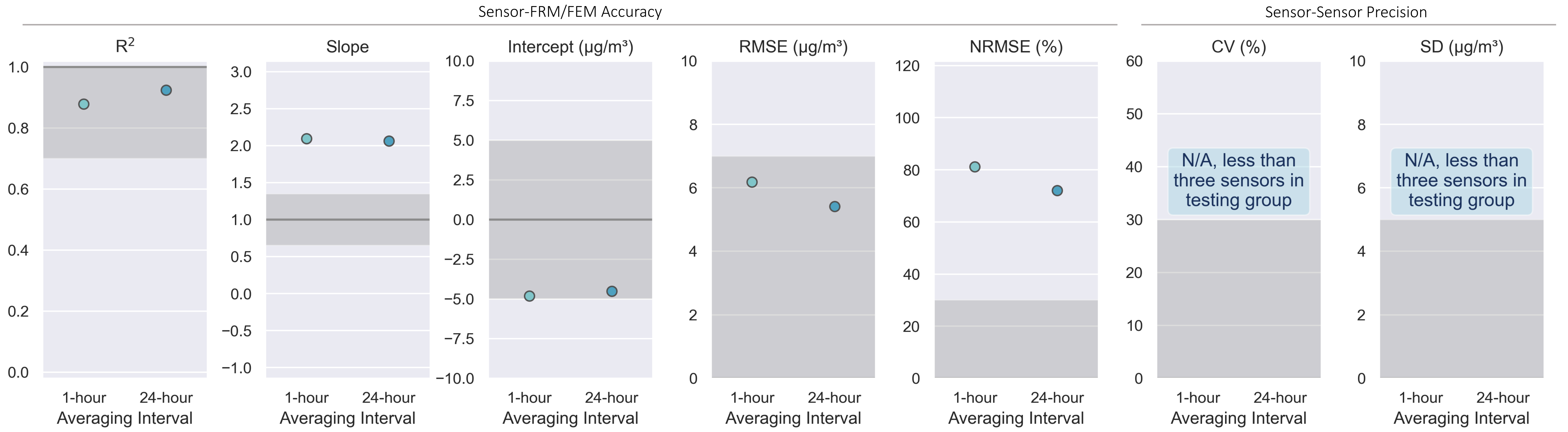

Meteorological Conditions During Deployment

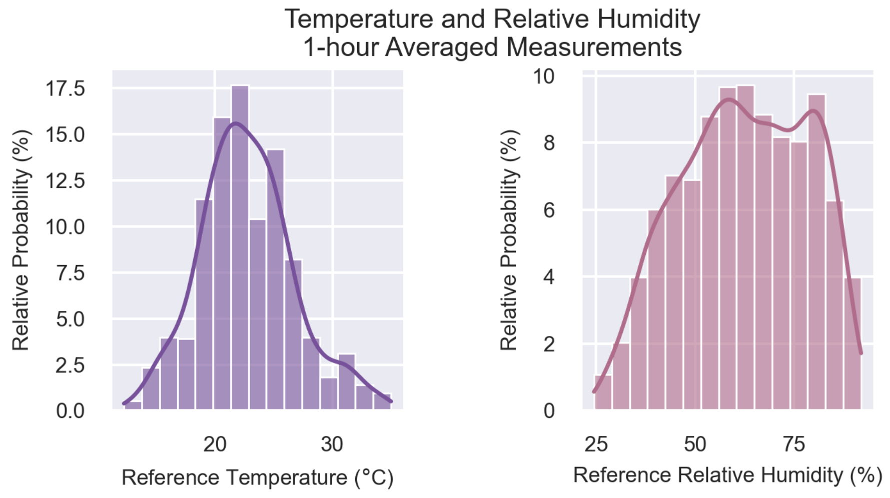

Number of 24-hr periods outside sensor manufacture-listed temperature operational range (no operational range specified)	-
Number of 24-hr periods outside sensor manufacture-listed relative humidity operational range (no operational range specified)	-

Meteorological Influence

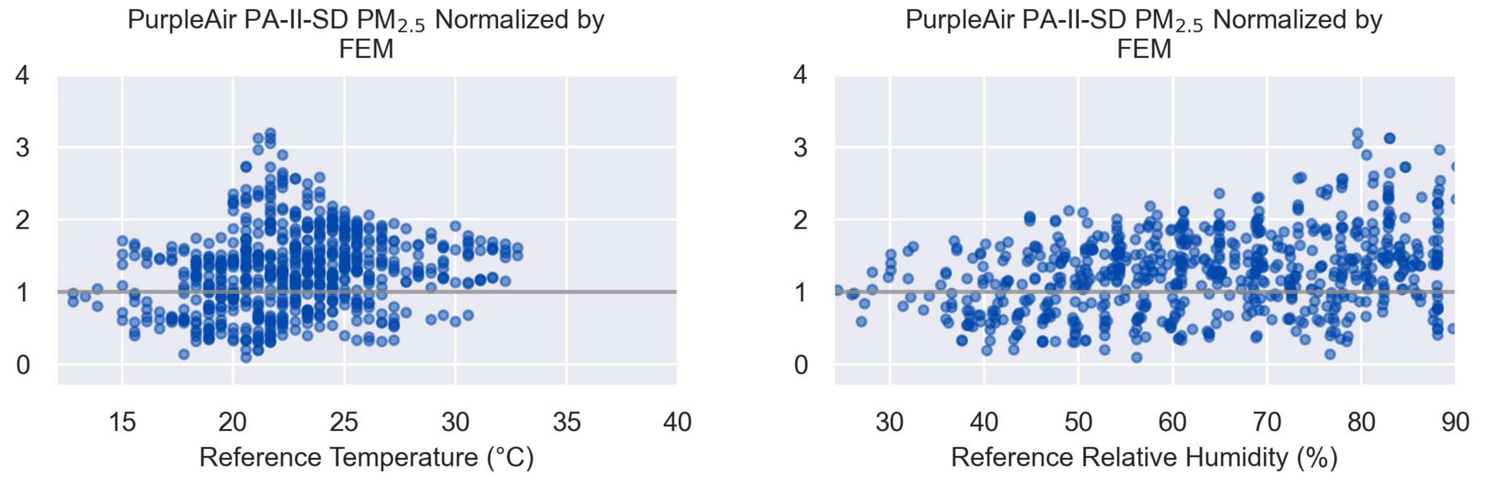

Mean number of paired, normalized concentration and temperature values (1-hr averages)	742
Mean number of paired, normalized concentration and relative humidity values (1-hr averages)	0

Testing Report - $PM_{2.5}$ Base Testing

PurpleAir PA-II-SD

This report reflects out-of-the-box performance

Initial Base Testing - Milwaukee, WI

U.S. Environmental Protection Agency
Office of Research and Development
PI: Clements.Andrea@epa.gov
919-541-1363

July 2019—August 2019

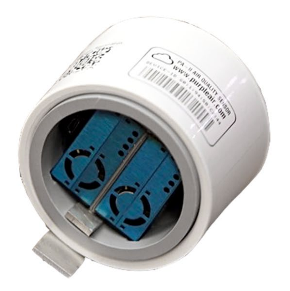

Tabular Statistics

Sensor-FRM/FEM Correlation

	Bias and Linearity						Data Quality			
	R^2		Slope		Intercept (µg/m³)		Uptime (%)		Number of paired sensor and FRM/FEM concentration values	
	1-Hour ●	24-Hour ●	1-Hour ○	24-Hour ○	1-Hour ●	24-Hour ●	1-Hour ●	24-Hour ●	1-Hour	24-Hour
Metric Target Range	≥ 0.70	≥ 0.70	1.0 ± 0.35	1.0 ± 0.35	-5 ≤ b ≤ 5	-5 ≤ b ≤ 5	75%*	75%*	-	-
Sensor PA01	0.88	0.92	2.10	2.06	-4.81	-4.51	94	90	697	28

	Error			
	RMSE (µg/m³)		NRMSE (%)	
	1-Hour ★	24-Hour ★	1-Hour ☆	24-Hour ☆
Metric Target Range	≤ 7.0	≤ 7.0	≤ 30.0	≤ 30.0
Deployment Value	6.2	5.4	81.2	72.1

Device-specific metrics (computed for each sensor in evaluation)
○○○ Metric value for none of devices tested falls within the target range
●○○ Metric value for one of devices tested falls within the target range
●●○ Metric value for two of devices tested falls within the target range
●●● Metric value for three of devices tested falls within the target range

Single-valued metrics (computed via entire evaluation dataset)
☆ Indicates that the metric value is not within the target range
★ Indicates that the metric value is within the target range

Sensor-Sensor Precision[1]

	Precision (between collocated sensors)				Data Quality	
	CV (%)		SD (µg/m³)		Number of concurrent sensor concentration pairs	
	1-Hour ☆	24-Hour ☆	1-Hour ☆	24-Hour ☆	1-Hour	24-Hour
Metric Target Range	≤ 30.0	≤ 30.0	≤ 5.0	≤ 5.0	-	-
Deployment Value	-	-	-	-	-	-

[1]Precision statistics are computed for evaluations with at least three collocated sensor units. Metric values are left blank for evaluations with two or fewer sensor units.

*This value is only a recommendation for ensuring data quality and is not included in the list of target values discussed in Section 4 of the Performance Testing Protocols, Metrics, and Target Values for Fine Particulate Matter Air Sensors document.

Testing Report - $PM_{2.5}$ Base Testing

PurpleAir PA-II-SD

This report reflects out-of-the-box performance

Initial Base Testing - Milwaukee, WI

U.S. Environmental Protection Agency
Office of Research and Development
PI: Clements.Andrea@epa.gov
919-541-1363
July 2019—August 2019

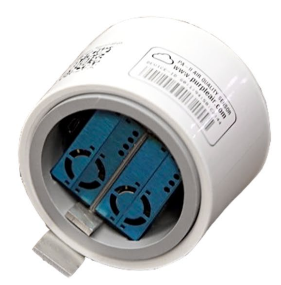

Sensor-FRM/FEM Scatter Plots

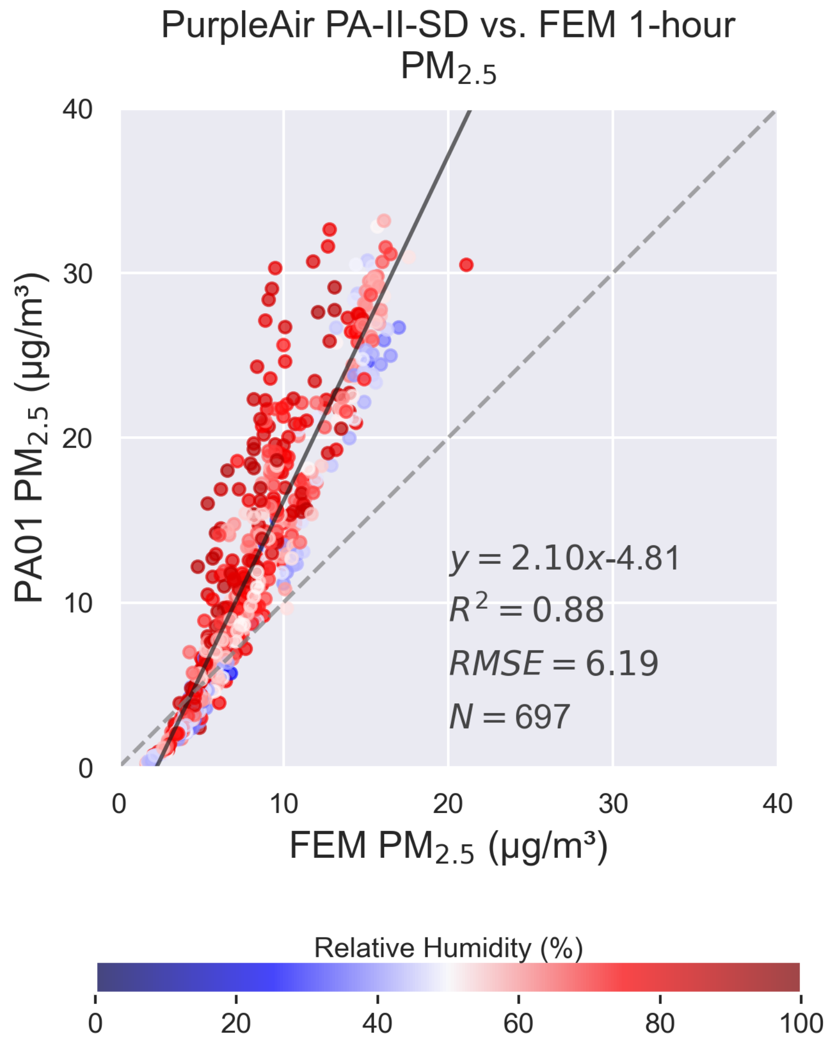

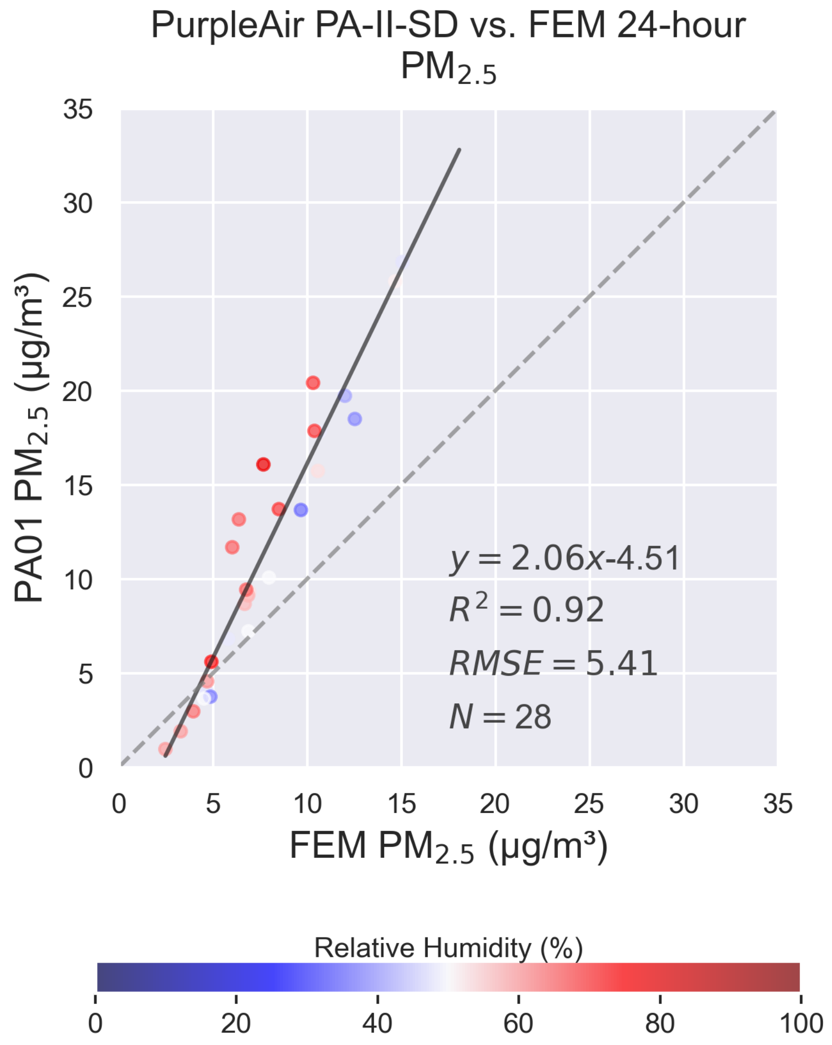

Testing Report - $PM_{2.5}$ Base Testing

PurpleAir PA-II-SD

This report reflects out-of-the-box performance

Initial Base Testing - Milwaukee, WI

U.S. Environmental Protection Agency
Office of Research and Development
PI: Clements.Andrea@epa.gov
919-541-1363

July 2019—August 2019

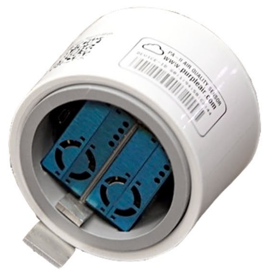

Supplemental Information

Abbreviations used in Supplemental Information

FRM	Federal Reference Method
FEM	Federal Equivalent Method
SOP	Standard Operating Procedure
QAPP	Quality Assurance Project Plan
QC	Quality Control

Supplemental Documentation	Attached	Description & URL or file path to documentation
Field observations and sensor data flags	☑	See WI-PAR-Page 6 of this testing report
Maintenance logs	☐	No logs recorded during testing
Standard operating procedure(s)	☐	U.S. EPA Office Of Research and Development SOP available upon request
Photos of equipment setup and testing	☑	See WI-PAR-Page 5 of this testing report
Product specifications sheet(s)	☑	PurpleAir: PA-II-SD Product website
Product manual(s)	☐	N/A
Data storage and transmission method	☑	See WI-PAR-Page 6 of this testing report
Data correction approach	☑	See WI-PAR-Page 6 of this testing report
Issues encountered	☑	See WI-PAR-Page 6 of this testing report
Data analysis/correction scripts and version	☑	Averaging and processing of data, calculation of performance metrics, and generation of figures and other supplementary material for analysis were obtained using Python 3.9.7 with the packages sensortoolkit v0.8.3b2, pandas 1.3.5, NumPy 1.21.2, Matplotlib 3.5.0, statsmodels 0.13.0, and seaborn 0.11.2. All packages are available from the Python Package Index (PyPI) at https://pypi.org. The integrated development environment (IDE) Spyder 5.1.5 was used for scripting and data visualization. Version control for the Python base, packages, and IDE were all managed by conda 4.11.0.
Air Monitoring Station QAPP	☐	U.S. EPA Office Of Research and Development QAPP available upon request
Summary of FRM/FEM monitor QC checks	☑	See WI-PAR-Page 7 of this testing report
Manufacturer website for FRM/FEM monitor	☑	Teledyne API: T640/T640X Product website
FRM/FEM monitor manual	☑	Teledyne API: T640/T640X Product Manual
FRM/FEM monitor specifications sheet(s)	☑	Teledyne API: T640/T640X Specification Sheet
Other documents	☐	

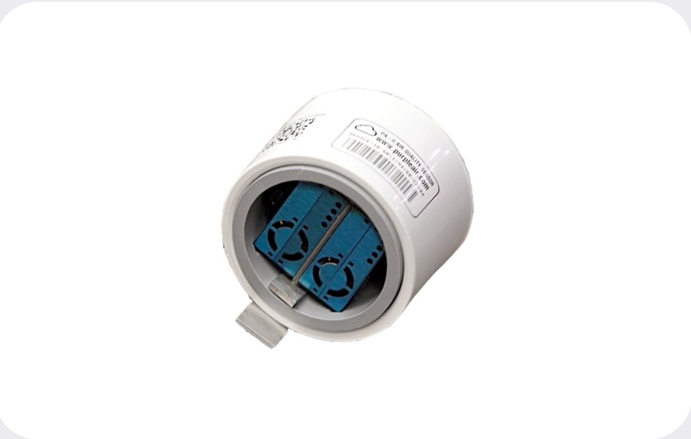

Supplemental Information: Photos of Testing Site and Equipment Setup

Site Description:

The urban site was located in the parking lot at the former WDNR Southeast Regional Headquarters located at 2300 N. Martin Luther King Blvd. Sample inlets are roughly 5 meters above ground level and 50 meters from the nearest road. Verified through annual WDNR audits, the site meets the requirements of *40 CFT 58, Appendices C, D, E and G.*

Figure 1: PurpleAir PA-II-SD sensor (indicated by red arrow) attached to metal railing atop the sampling shelter at the monitoring site.

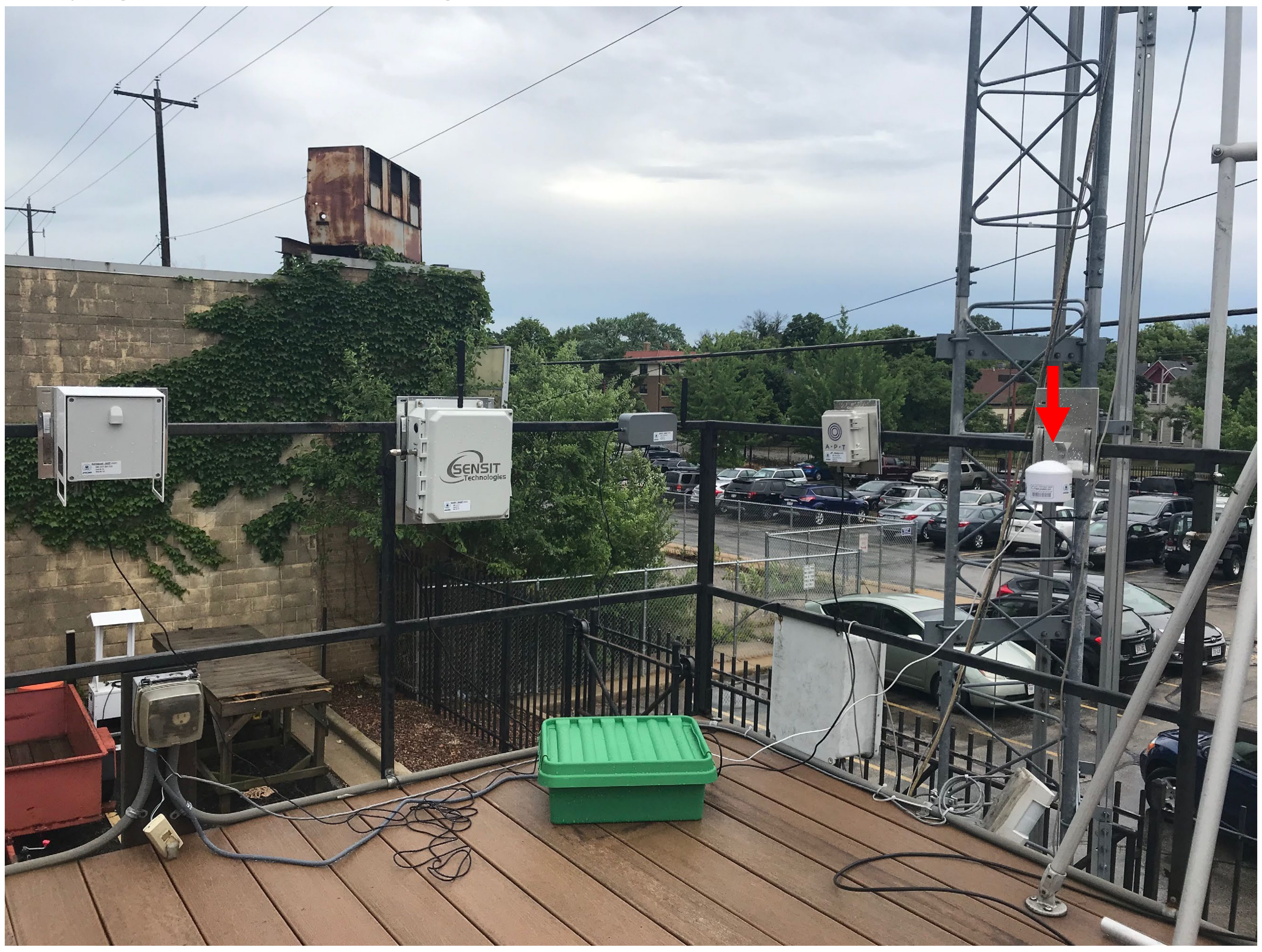

Figure 2: WDNR Monitoring Station

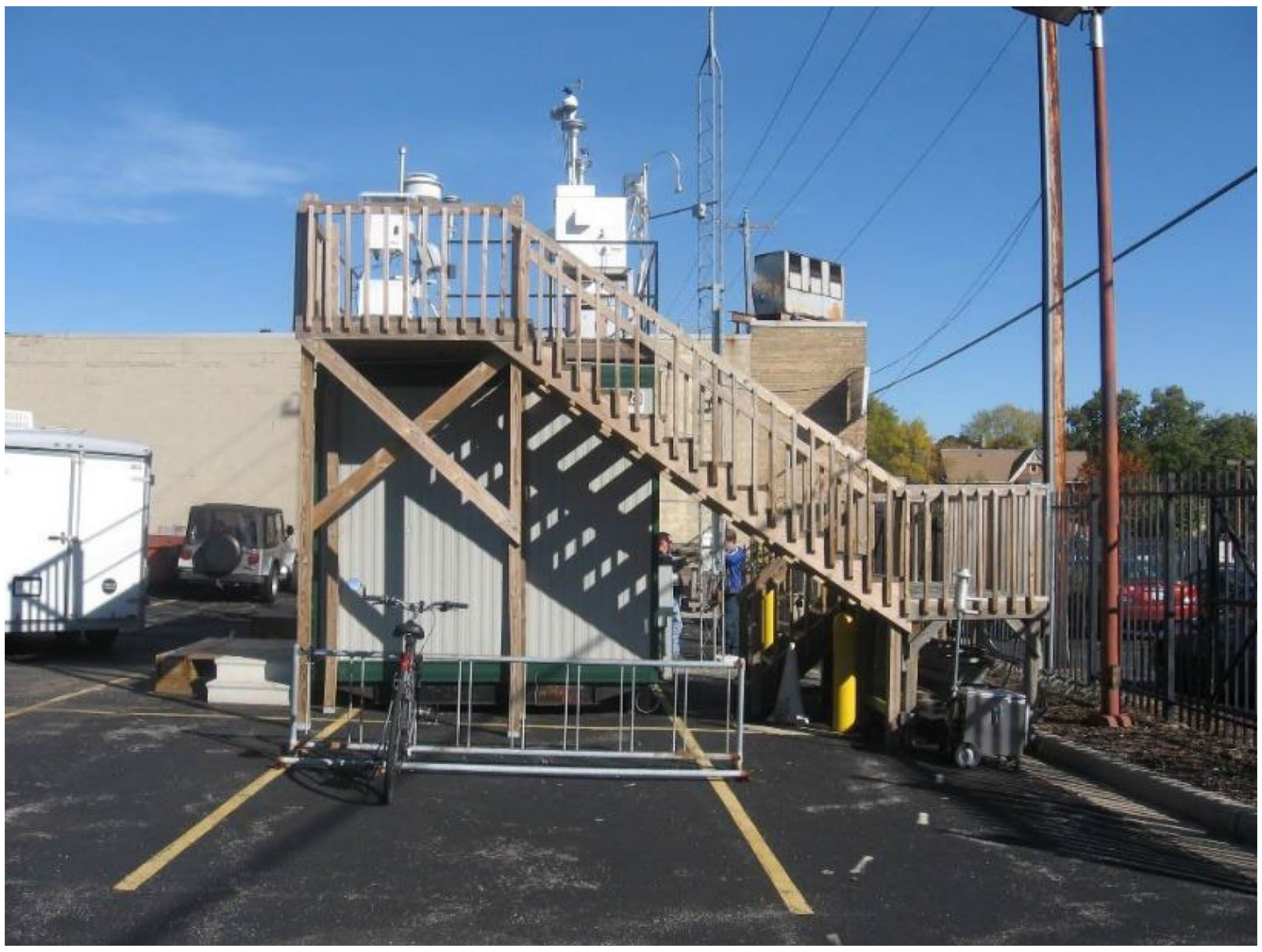

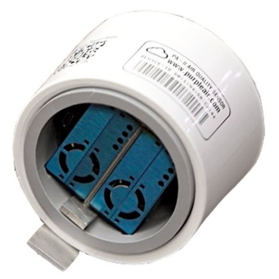

Data Storage and Transmission Method

The PurpleAir PA-II-SD records data at a 2-minute[1] sampling interval. Data is transmitted to the PurpleAir Cloud via Wi-Fi and stored on an internal MicroSD card. Due to the differing file structure between remote and direct download methods and the lag associated with cloud data download, weekly raw data were obtained primarily via onboard SD card. Each field site operator was provided two labeled MicroSD cards for sensor units that they used to swap out each week. Data from the collected card was then read and processed off-site.

Data Correction Approach

Data sets recorded by PurpleAir PA-II-SD contain two correction factors for PM concentrations, referred to internally as “CF=ATM” and “CF=1”. PM$_{2.5}$ concentrations are recorded concurrently for each correction factor, and corresponding concentrations agree closely up to approximately 30 µg/m^3, above which one correction factor reflects concentrations roughly 1.5 times concentrations for the other correction factor. During a separate analysis by ORD, concentrations for the higher correction factor were determined to have slightly higher correlation with collocated reference measurements. As a result, data for the higher correction factor were used for this study. Prior to firmware version 5.00 (released on 11/30/2019), headers for concentrations corresponding to each correction factor were incorrectly labeled (i.e., CF=1 was labeled CF=ATM and vice versa). Testing indicated in this report was completed prior to this firmware update, and thus data sets acquired for PA-II-SD units had swapped PM$_{2.5}$ CF labels. Recorded PA-II-SD data sets indicate that the CF=ATM correction factor was the higher CF as labeled (following firmware update 5.00, this data channel corresponds to CF=1) and sensor data shown in this report correspond to this correction factor.

After acquisition, the raw data was processed using the *sensortoolkit* python code library (v0.8.3b2). A continuous data set at the recorded sampling frequency was written to a .csv file. 1-hour and 24-hour averaged data sets were generated using a 75% completeness threshold and saved as separate .csv files. Simultaneous measurement from the PurpleAir PA-II’s dual PMS5003 sensors (labeled channels A and B) allows for identification of outliers resulting in large-scale disagreement between recorded values. The outlier removal method of Barkjohn et al. 2021[2] was applied to both 1-hour and 24-hour averaged A and B measurement pairs. This method includes thresholds for the absolute difference and the percent difference between hourly channel values. A-B channel averages were not included for hours where the absolute difference between channel values was greater than 5 µg/m^3 and the percent difference exceeded 70%. This outlier removal method is in use on the AirNow Fire and Smoke map and has been used for this evaluation since it requires no external information (e.g., data from the monitor) and no assumptions about typical PM2.5 concentrations (e.g., removing data over a certain threshold concentration).

Issues Encountered

Pre-deployment observations

- Prior to the testing period indicated in this report, the PurpleAir PA-II-SD unit was briefly collocated for a period of approximately one week at EPA’s Ambient Monitoring Innovative Research Station (AIRS) in Research Triangle Park, NC. The unit was tested for proper operation, including powering on and data logging (either to the onboard SD card or via hotspot Wi-Fi connection to the PurpleAir Cloud), sampling, and acquisition of recorded data from either onboard memory or cloud storage. Units that did not pass this pre-deployment testing were returned to the manufacturer and constituted approximately 1 in 10 units tested for subsequent performance evaluation.

Field observations and sensor data flags

The PurpleAir PA-II-SD was deployed at the WDNR monitoring site on 7/22/2019. The PA-II-SD unit operated nominally during the testing period and did not require replacement or repair.

During the testing period, no data flags logged by field technicians were recorded.

[1]As of version 4.02, updated across devices on 5/31/2019. Prior to this firmware version, devices recorded measurements at 80-second intervals
[2]Barkjohn, K. K., Gantt, B., and Clements, A. L.: Development and application of a United States-wide correction for PM2.5 data collected with the PurpleAir sensor, Atmos. Meas. Tech., 14, 4617–4637, https://doi.org/10.5194/amt-14-4617-2021, 2021.

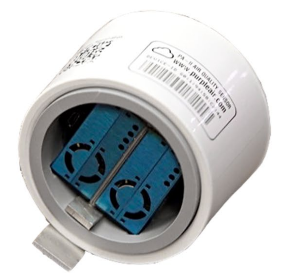

Supplemental Information: Description of FRM/FEM QC Checks and Data Flags

Description of Data Flags

AQS

The U.S. EPA's Air Quality System (AQS) is the Agency's primary ambient air monitoring data archive. A comprehensive list of data flags that are recorded alongside AQS data sets, referred to by U.S. EPA as 'qualifiers', can be found at the following link: https://aqs.epa.gov/aqsweb/documents/codetables/qualifiers.html

Invalidation of reference data: AQS qualifiers are organized by qualifier type, which indicates whether data logged alongside qualifier flags should be invalidated (set null). Qualifiers with type "Null Data Qualifier" are invalidated, and includes data logged during periods that coincide with QC checks (e.g., "BF-Precision/Zero/Span", "BJ- Operator Error", "BL - QA Audit", "AZ - QC Audit") among other events such as power outages. Data logged alongside qualifiers with type "Quality Assurance Qualifiers" are not invalidated and are included in this analysis (e.g., concentrations less than the federal MDL for the reference monitor "MD – Value less than MDL", QA reviewed values "Validated Value").

Data Flags Recorded During Testing

FRM/FEM Monitor	Timestamp (UTC)	Flag
Teledyne API T640X (Data acquired from AQS)	2019-07-30 20:00:00+0000	AX - Precision Check

Meteorological Instrument	Timestamp (UTC)	Flag
MetOne Temperature Monitor (Data acquired from AQS)	2019-08-07 16:00:00+0000 to 2019-08-07 17:00:00+0000	AZ - QC Audit

Testing Report - $PM_{2.5}$ Base Testing

QuantAQ ARISense

This report reflects out-of-the-box performance

Initial Base Testing - Milwaukee, WI

U.S. Environmental Protection Agency
Office of Research and Development
PI: Clements.Andrea@epa.gov
919-541-1363
February 2020—March 2020

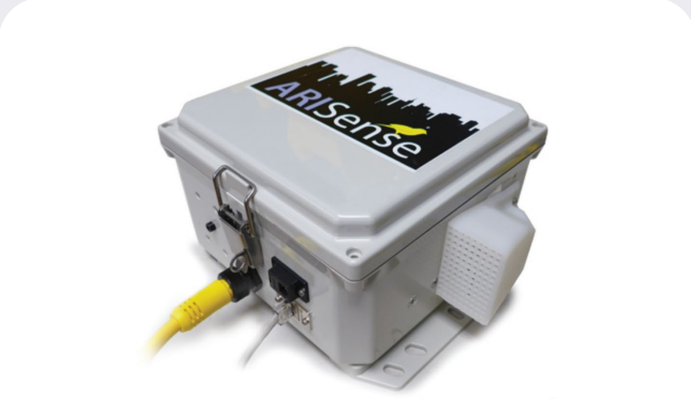

Image Attribution: QuantAQ, Inc (Somerville, MA)

Deployment Details

Testing Organization and Site Information	
Testing organization (Name, Organization type, Contact website)	U.S. Environmental Protection Agency - Office of Research and Development<br>Federal Government<br>Air Sensor Toolbox | U.S. EPA Website
Testing location (City, State, Latitude and Longitude)	WDNR Headquarters<br>Milwaukee, WI<br>43.061, -87.9135
AQS site ID	55 - 079 - 0026
Sampling timeframe (MM-DD-YY)	02-14-20 to 03-15-20
Sensor data source	QuantAQ Cloud download
Reference data source	AQS API download

Sensor Information			
Manufacturer, model	QuantAQ ARISense		
Device firmware version	Received by EPA on 4/30/2019		
Sampling time interval	1-minute		
Sensor serial numbers	ARS_01		
Issues encountered during deployment?	☑	See WI-ARS-Page 6 of this testing report	

FRM/FEM Information	
Manufacturer, model, designation	Teledyne Advanced Pollution Instrumentation T640X FEM
Sampling time interval	1-hour averaging
Date of calibration	As required by 40 CFR Part 58 and the Low Volume $PM_{2.5}$ and PM_{10} Networks QAPP maintained by WDNR
Date of flowrate verification check	Monthly as required by 40 CFR Part 58 Appendix A
Description, date(s) of maintenance activities	See WI-ARS-Page 7 of this testing report

Time Series Plots

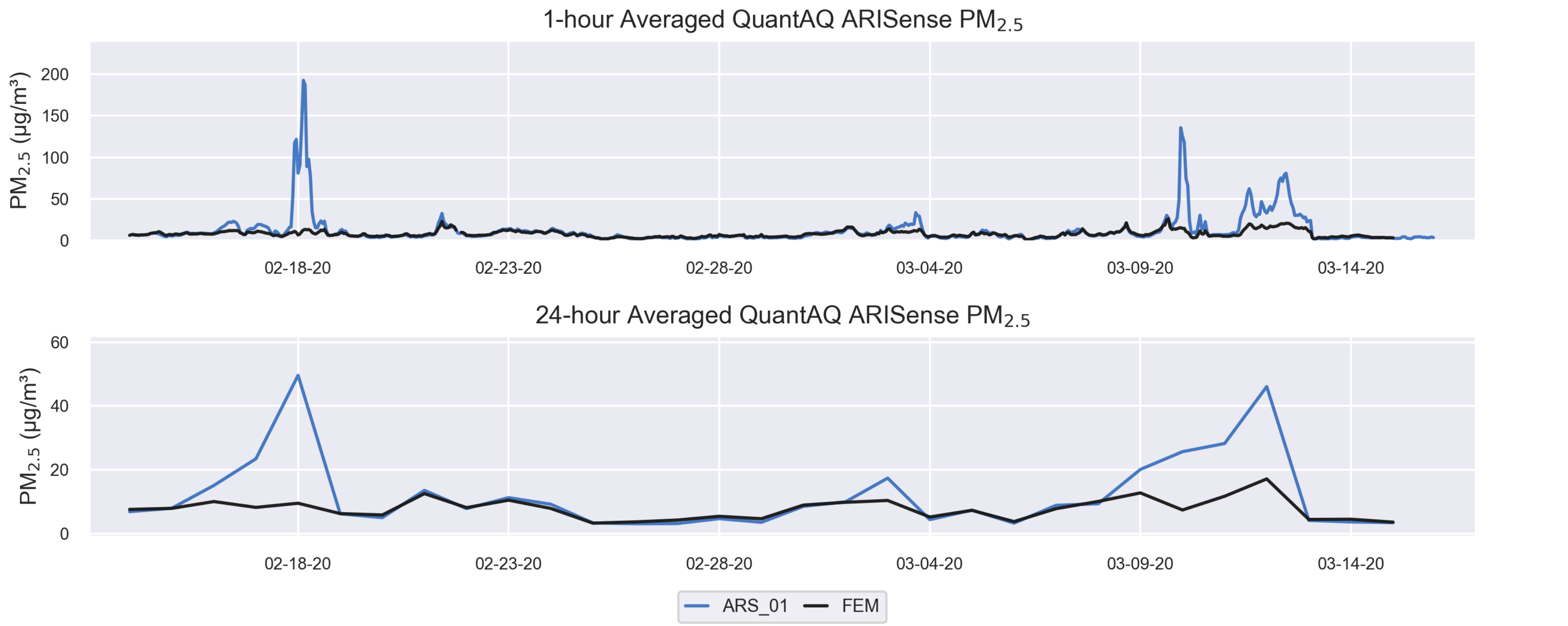

Scatter Plots: Comparison to FRM/FEM

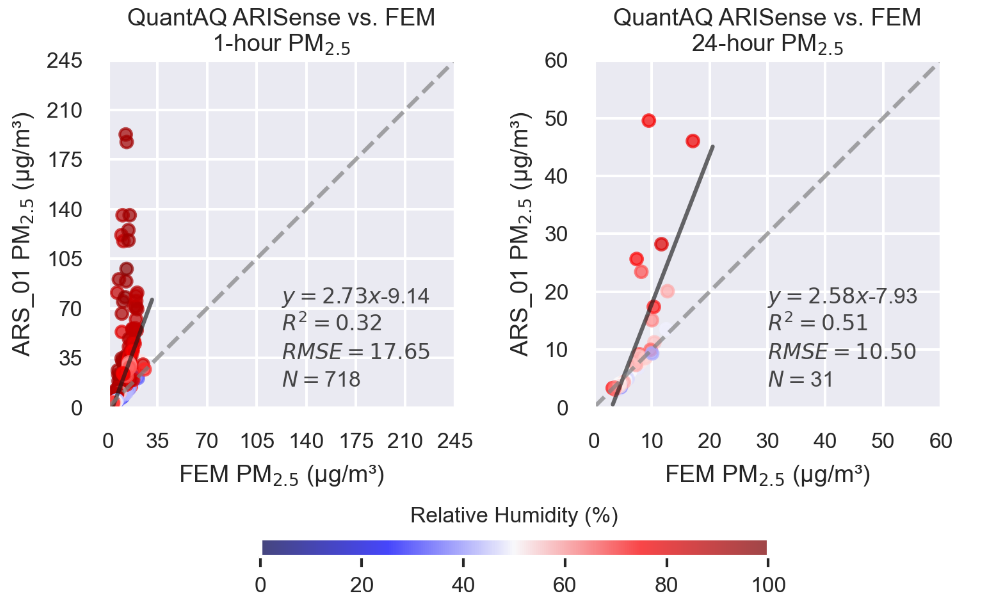

Range of FRM/FEM concentrations over duration of base test (µg/m³)	1.0-26.0 (1-hr), 3.3-17.1 (24-hr)
Number of 24-hr periods in FRM/FEM monitor measurements with a goal concentration ≥ 25 µg/m³	0

Performance Metrics

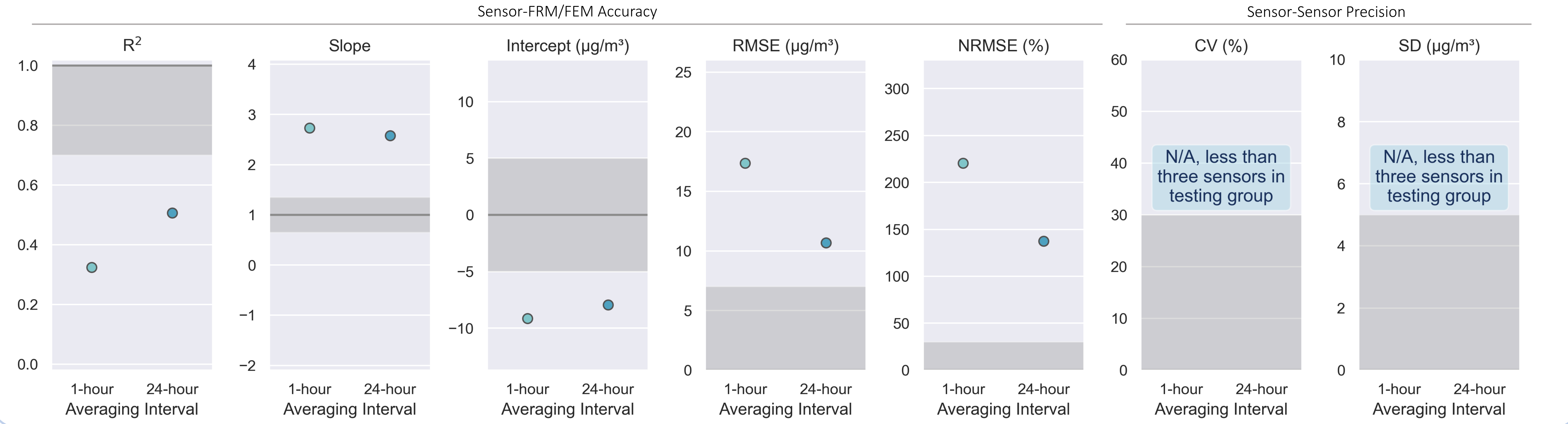

Meteorological Conditions During Deployment

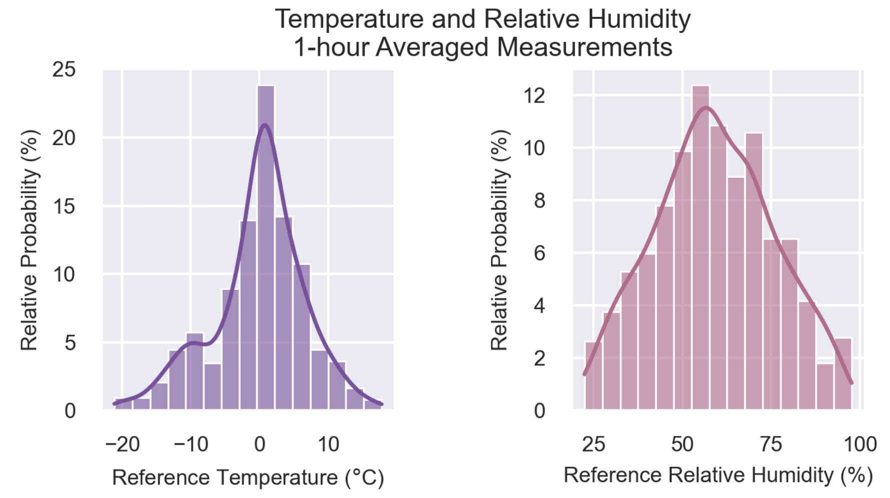

Number of 24-hr periods outside sensor manufacture-listed temperature operational range (no operational range specified)	-
Number of 24-hr periods outside sensor manufacture-listed relative humidity operational range (no operational range specified)	-

Meteorological Influence

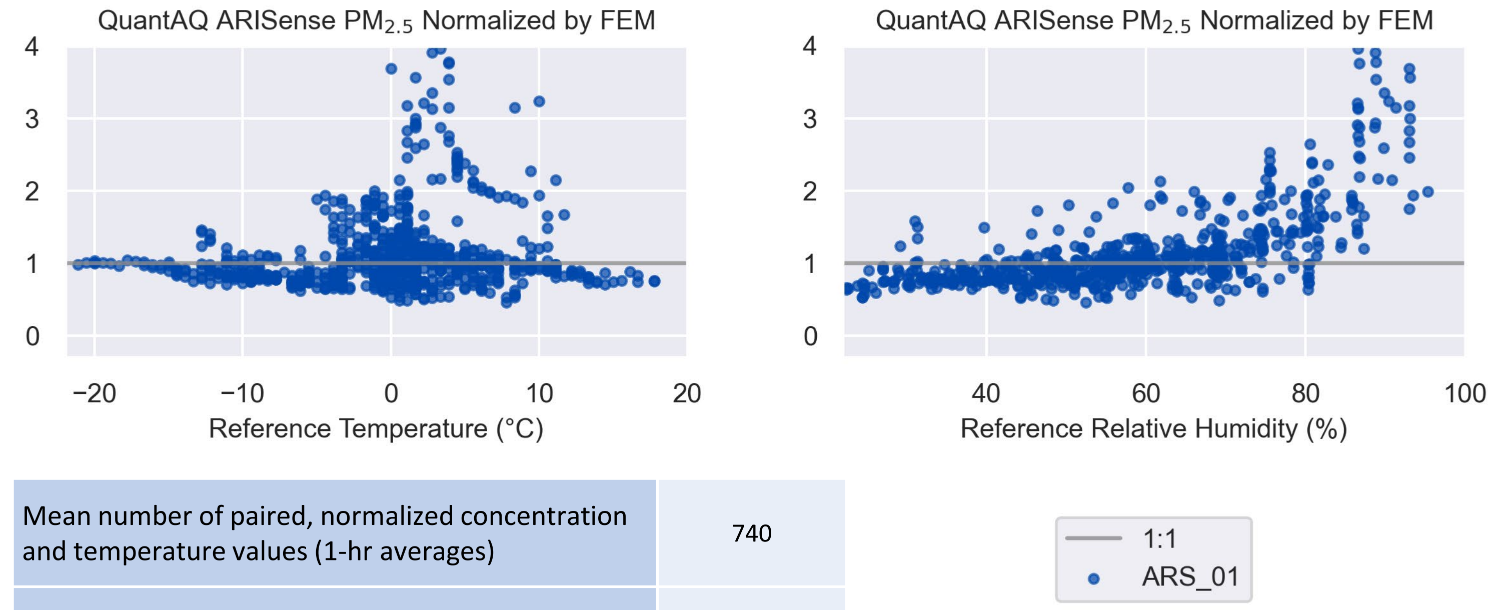

Mean number of paired, normalized concentration and temperature values (1-hr averages)	740
Mean number of paired, normalized concentration and relative humidity values (1-hr averages)	0

Testing Report - $PM_{2.5}$ Base Testing

QuantAQ ARISense

This report reflects out-of-the-box performance

Initial Base Testing - Milwaukee, WI
U.S. Environmental Protection Agency
Office of Research and Development
PI: Clements.Andrea@epa.gov
919-541-1363
February 2020—March 2020

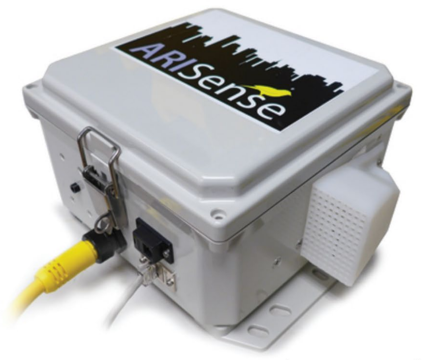

Image Attribution: QuantAQ, Inc (Somerville, MA)

Tabular Statistics

Sensor-FRM/FEM Correlation

	Bias and Linearity						Data Quality			
	R^2		Slope		Intercept (µg/m³)		Uptime (%)		Number of paired sensor and FRM/FEM concentration values	
	1-Hour ○	24-Hour ○	1-Hour ○	24-Hour ○	1-Hour ○	24-Hour ○	1-Hour ●	24-Hour ●	1-Hour	24-Hour
Metric Target Range	≥ 0.70	≥ 0.70	1.0 ± 0.35	1.0 ± 0.35	-5 ≤ b ≤ 5	-5 ≤ b ≤ 5	75%*	75%*	-	-
Sensor ARS_01	0.32	0.51	2.73	2.58	-9.14	-7.93	100	100	718	31

	Error			
	RMSE (µg/m³)		NRMSE (%)	
	1-Hour ☆	24-Hour ☆	1-Hour ☆	24-Hour ☆
Metric Target Range	≤ 7.0	≤ 7.0	≤ 30.0	≤ 30.0
Deployment Value	17.4	10.7	220.5	137.6

Device-specific metrics (computed for each sensor in evaluation)
○○○ Metric value for none of devices tested falls within the target range
●○○ Metric value for one of devices tested falls within the target range
●●○ Metric value for two of devices tested falls within the target range
●●● Metric value for three of devices tested falls within the target range

Single-valued metrics (computed via entire evaluation dataset)
☆ Indicates that the metric value is not within the target range
★ Indicates that the metric value is within the target range

Sensor-Sensor Precision[1]

	Precision (between collocated sensors)				Data Quality	
	CV (%)		SD (µg/m³)		Number of concurrent sensor concentration pairs	
	1-Hour ☆	24-Hour ☆	1-Hour ☆	24-Hour ☆	1-Hour	24-Hour
Metric Target Range	≤ 30.0	≤ 30.0	≤ 5.0	≤ 5.0	-	-
Deployment Value	-	-	-	-	-	-

[1]Precision statistics are computed for evaluations with at least three collocated sensor units. Metric values are left blank for evaluations with two or fewer sensor units.

*This value is only a recommendation for ensuring data quality and is not included in the list of target values discussed in Section 4 of the Performance Testing Protocols, Metrics, and Target Values for Fine Particulate Matter Air Sensors document.

Testing Report - $PM_{2.5}$ Base Testing

QuantAQ ARISense

This report reflects out-of-the-box performance

Initial Base Testing - Milwaukee, WI

U.S. Environmental Protection Agency

Office of Research and Development

PI: Clements.Andrea@epa.gov

919-541-1363

February 2020—March 2020

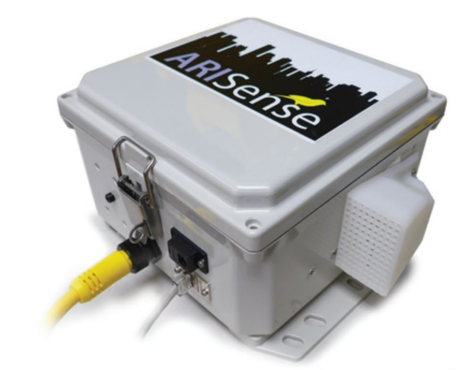

Image Attribution: QuantAQ, Inc (Somerville, MA)

Sensor-FRM/FEM Scatter Plots

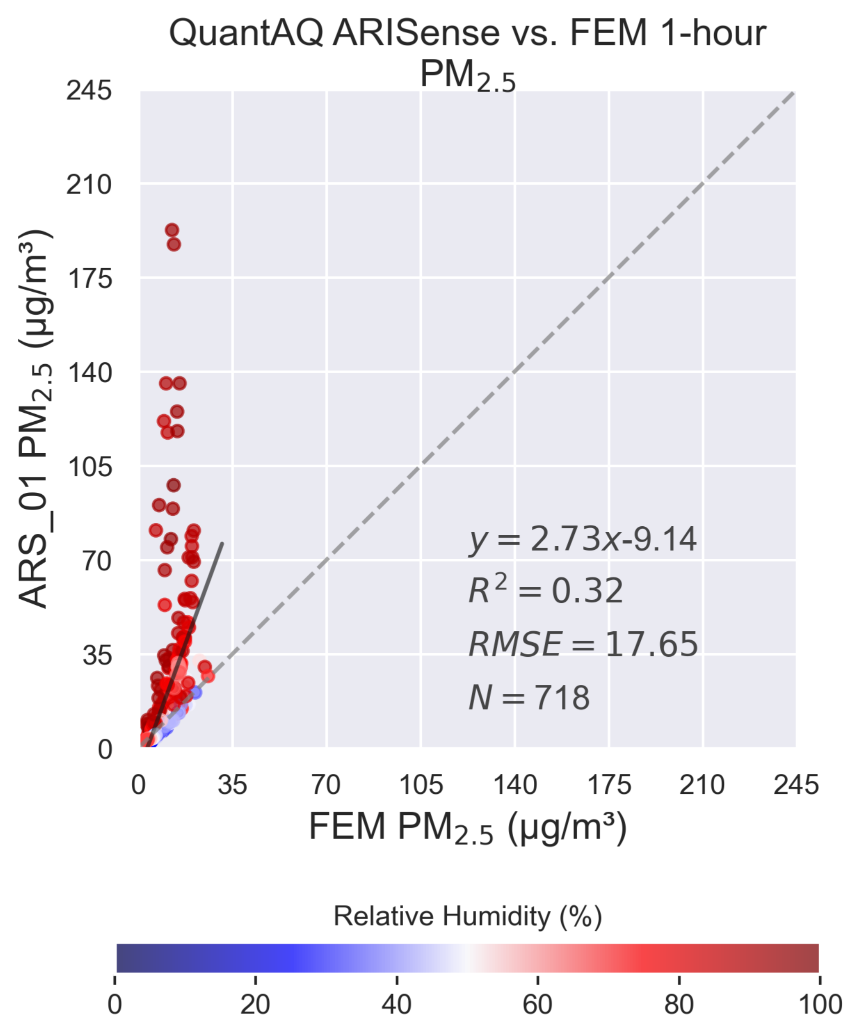

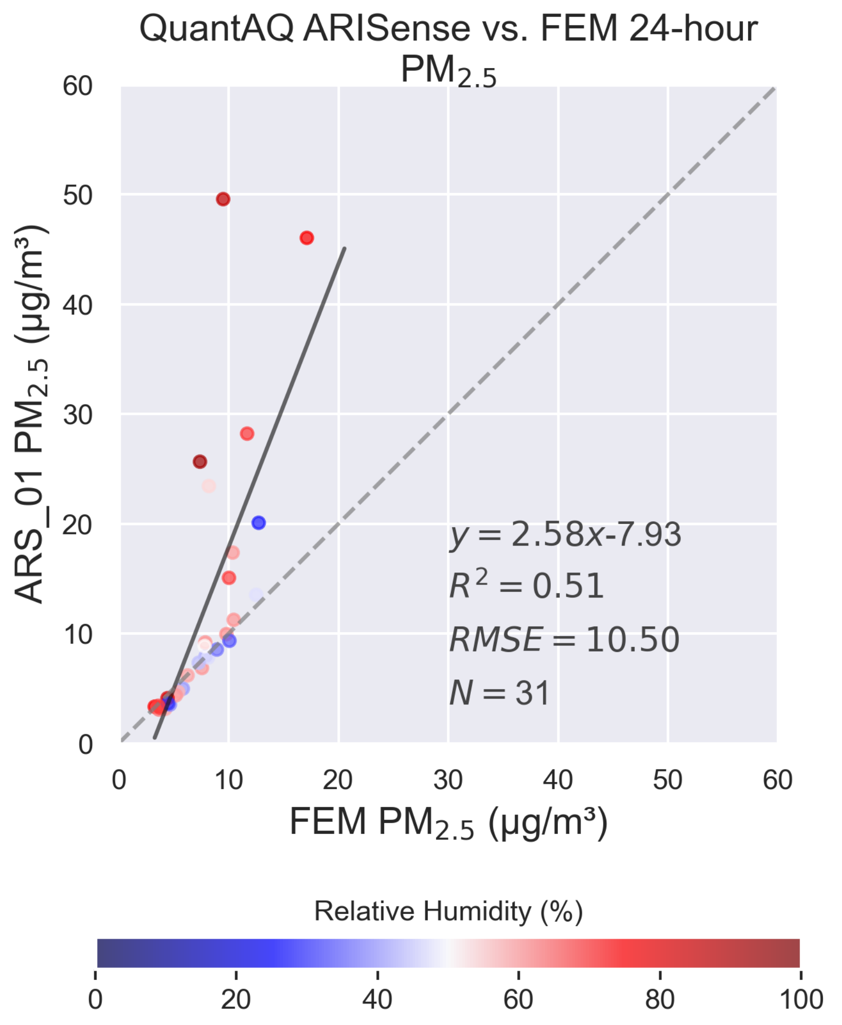

PI: Clements.Andrea@epa.gov
919-541-1363

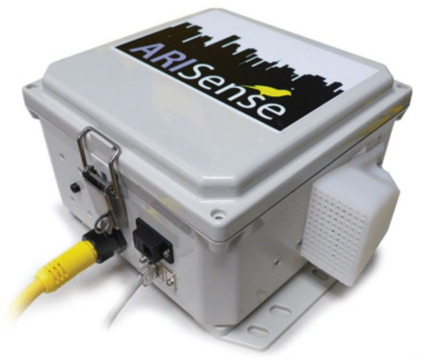

Image Attribution: QuantAQ, Inc (Somerville, MA)

Supplemental Information

Abbreviations used in Supplemental Information

FRM	Federal Reference Method
FEM	Federal Equivalent Method
SOP	Standard Operating Procedure
QAPP	Quality Assurance Project Plan
QC	Quality Control

Supplemental Documentation	Attached	Description & URL or file path to documentation
Field observations and sensor data flags	☑	See WI-ARS-Page 7 of this testing report
Maintenance logs	☐	No logs recorded during testing
Standard operating procedure(s)	☐	U.S. EPA Office Of Research and Development SOP available upon request
Photos of equipment setup and testing	☑	See WI-ARS-Page 5 of this testing report
Product specifications sheet(s)	☑	QuantAQ and Particles Plus: ARISense Specification Sheet
Product manual(s)	☐	N/A
Data storage and transmission method	☑	See WI-ARS-Page 6 of this testing report
Data correction approach	☑	See WI-ARS-Page 6 of this testing report
Issues encountered	☑	See WI-ARS-Page 6 of this testing report
Data analysis/correction scripts and version	☑	Averaging and processing of data, calculation of performance metrics, and generation of figures and other supplementary material for analysis were obtained using Python 3.9.7 with the packages sensortoolkit v0.8.3b2, pandas 1.3.5, NumPy 1.21.2, Matplotlib 3.5.0, statsmodels 0.13.0, and seaborn 0.11.2. All packages are available from the Python Package Index (PyPI) at https://pypi.org. The integrated development environment (IDE) Spyder 5.1.5 was used for scripting and data visualization. Version control for the Python base, packages, and IDE were all managed by conda 4.11.0.
Air Monitoring Station QAPP	☐	U.S. EPA Office Of Research and Development QAPP available upon request
Summary of FRM/FEM monitor QC checks	☑	See WI-ARS-Page 8 of this testing report
Manufacturer website for FRM/FEM monitor	☑	Teledyne API: T640/T640X Product website
FRM/FEM monitor manual	☑	Teledyne API: T640/T640X Product Manual
FRM/FEM monitor specifications sheet(s)	☑	Teledyne API: T640/T640X Specification Sheet
Other documents	☐	

PI: Clements.Andrea@epa.gov
919-541-1363

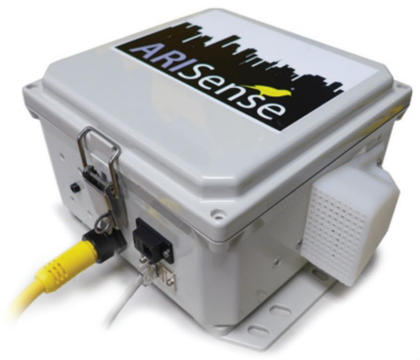

Image Attribution: QuantAQ, Inc (Somerville, MA)

Supplemental Information: Photos of Testing Site and Equipment Setup

Site Description:

The urban site was located in the parking lot at the former WDNR Southeast Regional Headquarters located at 2300 N. Martin Luther King Blvd. Sample inlets are roughly 5 meters above ground level and 50 meters from the nearest road. Verified through annual WDNR audits, the site meets the requirements of *40 CFT 58, Appendices C, D, E and G.*

Figure 1: WDNR Monitoring Station

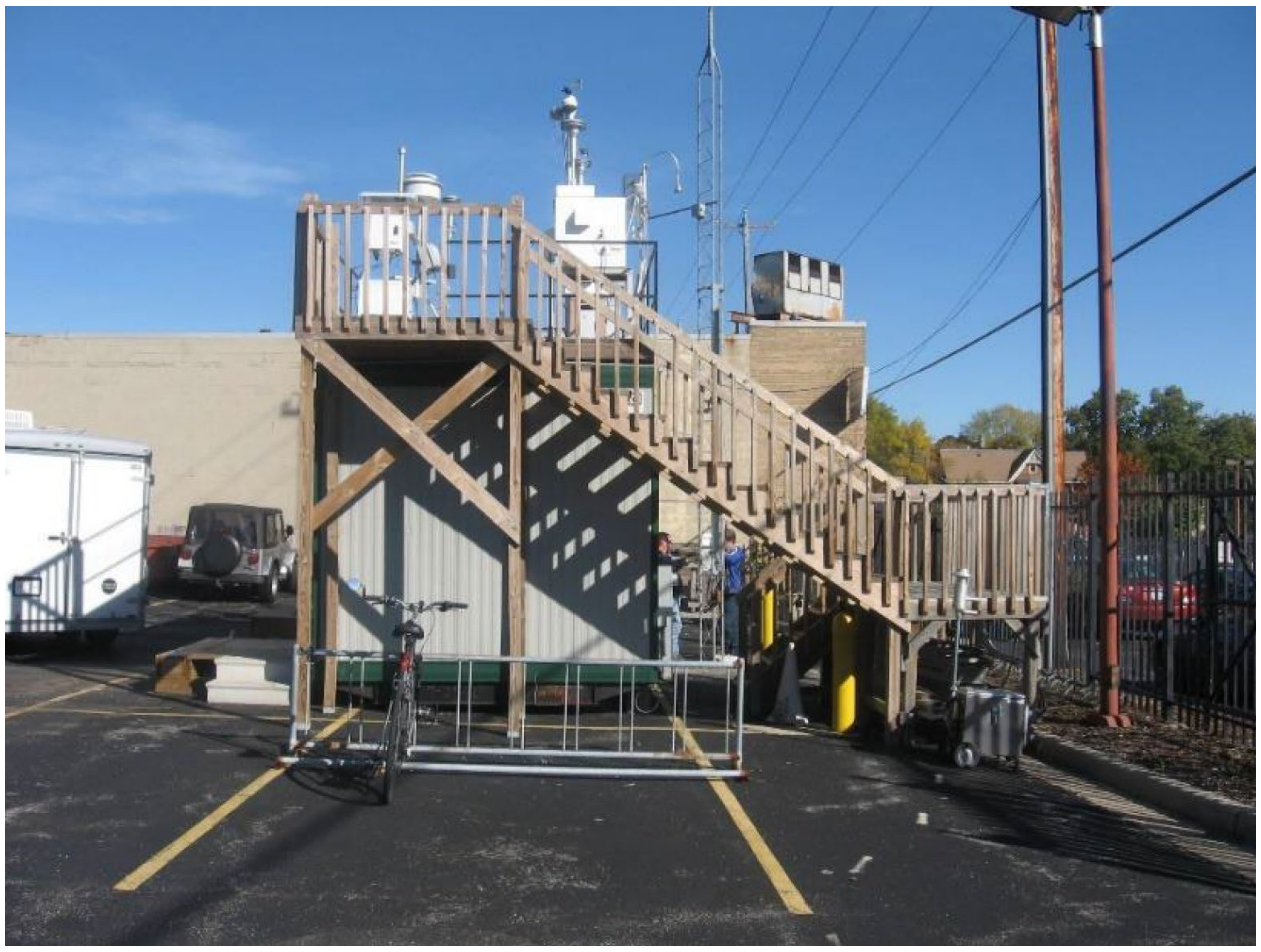

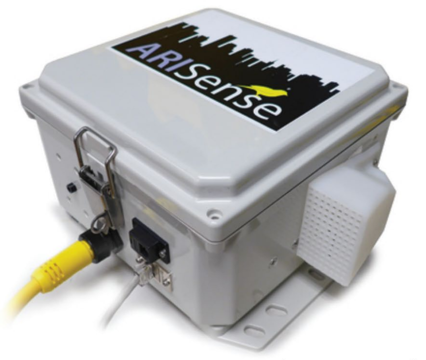

Image Attribution: QuantAQ, Inc (Somerville, MA)

Data Storage and Transmission Method

ARISense units upload data to an external cloud server at 2-minute averages via cellular signal. ARISense weekly data collections were performed by downloading data from the QuantAQ cloud server (last accessed 5/11/22).

Data Correction Approach

QuantAQ ARISense units measure PM2.5 concentrations via an optical particle counter (OPC). The OPC measures particle counts within particle size bins ranging from 0.3 microns to 10 microns, and mass concentrations for each bins are estimated from particle counts assuming constant values for density, spherical morphology, and average particle diameter. The manufacturer applies a general correction to account for the detection limitations of the OPC leading to concentrations that are closer to reality. These presets were configured by the sensor manufacturer and were not modified by EPA prior or during testing.

After acquisition, the raw data was processed using the *sensortoolkit* python code library (v0.8.3b2). A continuous data set at the recorded sampling frequency was written to a .csv file. 1-hour and 24-hour averaged data sets were generated using a 75% completeness threshold and saved as separate .csv files. Outliers were NOT removed from data sets in order to assess “out-of-the-box” sensor performance.

Issues Encountered

Pre-deployment observations

Numerous issues were encountered with the ARISense devices. Although previous models of the ARISense had used AlphaSense OPC-N2 these OPCs were unavailable at the time of manufacture. So instead, components from a particle plus OPC handheld were used.

- *Shipping damage:* On arrival to EPA many of the units were damaged due to the heavy pump breaking free during shipping and shaking around inside of the box breaking much of the plastic scaffolding and, in some cases, components. The manufacturer worked with us to fix these units.
- *Hardware and Firmware issues:* Multiple hardware and firmware issues were experienced that had to be fixed before starting the deployment and multiple manual firmware updates on both the gas sensor microcontrollers and the OPC microcontrollers were completed by researchers. The USB drives were frequently corrupted which required replacement and eventually a hardware update. In addition, the OPC microcontroller and the gas sensor microcontroller often miscommunicated. One firmware version reduced the number of bins reported by the OPC since the manufacturer determined the additional bins were not providing additional accuracy. New PCB boards were sent and installed before the tests could be run as well.
- *Initiation:* There was a high power draw when the cellular connectivity initiated and the pump was turned on. This meant that units occasionally failed to turn on at the same time and it meant these could not be powered by solar.
- *Water intake:* In addition, the OPC pump often drew in water during rainstorms leading the inline filter to saturate and the pump to fail due to overheating. Many pumps had to be replaced throughout the testing.
- *Cellular connectivity:* Some units could not consistently stay connected to the cellular, though other sensors at the same site did not experience issues. The manufacturer could not recreate this issue when they were sent back for service and the problems persisted.
- *Reset:* These units ended up being operated on timers so that they were power cycled every night which would typically get them back online if a software error had been encountered, leading to lower data loss than would have occurred with only weekly checks.

Field observations and sensor data flags are included on the following page

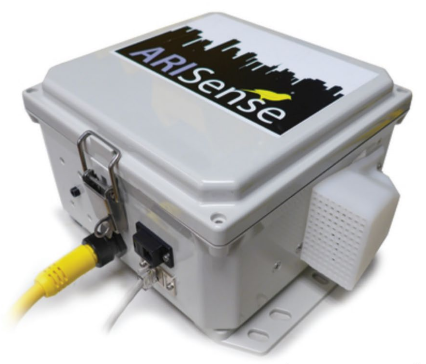

Image Attribution: QuantAQ, Inc (Somerville, MA)

Field observations and sensor data flags

The following table contains data flags describing events that were encountered during the testing period. The QuantAQ ARISense was configured to record data at regular 60-second sampling intervals, however, occasionally the unit recorded irregular sampling intervals ranging from 2-minute intervals to nearly 15 minutes at longest interval duration. The flags below note when these irregular sampling intervals occurred during the testing period. Sampling interval irregularities were not a result of modification to the device firmware configuration and are potentially associated power loading during periods where the ARISense unit initiated a cellular data upload.

Start Time (UTC)	End Time (UTC)	Sensor Serial ID	Parameters Impacted	Flag
2020-02-21 03:23:00+00:00	2020-02-21 03:32:00+00:00	ARS_01	ALL	6.0-Sampling interval abnormality
2020-02-21 20:52:00+00:00	2020-02-21 21:01:00+00:00	ARS_01	ALL	6.0-Sampling interval abnormality
2020-02-26 14:22:00+00:00	2020-02-26 14:28:00+00:00	ARS_01	ALL	6.0-Sampling interval abnormality
2020-03-14 08:32:00+00:00	2020-03-14 08:43:00+00:00	ARS_01	ALL	6.0-Sampling interval abnormality
2020-03-15 00:12:00+00:00	2020-03-15 00:23:00+00:00	ARS_01	ALL	6.0-Sampling interval abnormality
2020-03-15 22:21:00+00:00	2020-03-15 22:27:00+00:00	ARS_01	ALL	6.0-Sampling interval abnormality

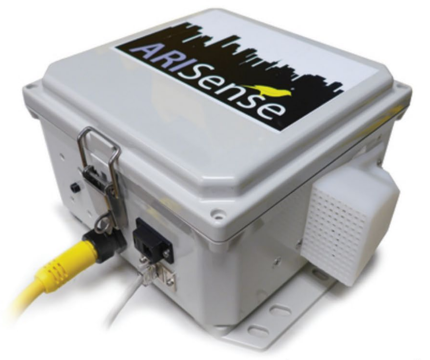

Image Attribution: QuantAQ, Inc (Somerville, MA)

Supplemental Information: Description of FRM/FEM QC Checks and Data Flags

Description of Data Flags

AQS

The U.S. EPA's Air Quality System (AQS) is the Agency's primary ambient air monitoring data archive. A comprehensive list of data flags that are recorded alongside AQS data sets, referred to by U.S. EPA as 'qualifiers', can be found at the following link: https://aqs.epa.gov/aqsweb/documents/codetables/qualifiers.html

Invalidation of reference data: AQS qualifiers are organized by qualifier type, which indicates whether data logged alongside qualifier flags should be invalidated (set null). Qualifiers with type "Null Data Qualifier" are invalidated, and includes data logged during periods that coincide with QC checks (e.g., "BF-Precision/Zero/Span", "BJ- Operator Error", "BL - QA Audit", "AZ - QC Audit") among other events such as power outages. Data logged alongside qualifiers with type "Quality Assurance Qualifiers" are not invalidated and are included in this analysis (e.g., concentrations less than the federal MDL for the reference monitor "MD – Value less than MDL", QA reviewed values "Validated Value").

Data Flags Recorded During Testing

FRM/FEM Monitor	Timestamp (UTC)	Flag
Teledyne API T640X (Data acquired from AQS)	2020-02-21 19:00:00+0000 to 2020-02-21 20:00:00+0000	AX - Precision Check
	2020-02-26 16:00:00+0000 to 2020-02-26 19:00:00+0000	2 - Operational Deviation
	2020-03-08 07:00:00+0000 to 2020-03-09 19:00:00+0000	2 - Operational Deviation

Meteorological Instrument	Timestamp (UTC)	Flag
MetOne Temperature Monitor (Data acquired from AQS)	2020-02-26 16:00:00+0000 to 2020-02-26 19:00:00+0000	BA - Maintenance/Routine Repairs
	2020-03-08 07:00:00+0000 to 2020-03-09 19:00:00+0000	2 - Operational Deviation

Testing Report - $PM_{2.5}$ Base Testing

SENSIT RAMP

This report reflects out-of-the-box performance

Initial Base Testing - Milwaukee, WI

U.S. Environmental Protection Agency
Office of Research and Development
PI: Clements.Andrea@epa.gov
919-541-1363

August 2019

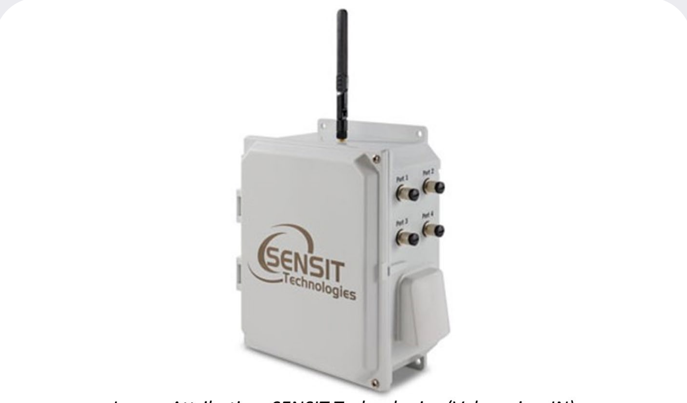

Image Attribution: SENSIT Technologies (Valparaiso, IN)

Deployment Details

Testing Organization and Site Information	
Testing organization (Name, Organization type, Contact website)	U.S. Environmental Protection Agency - Office of Research and Development<br>Federal Government<br>Air Sensor Toolbox | U.S. EPA Website
Testing location (City, State, Latitude and Longitude)	WDNR Headquarters<br>Milwaukee, WI<br>43.061, -87.9135
AQS site ID	55 - 079 - 0026
Sampling timeframe (MM-DD-YY)	08-01-19 to 08-31-19
Sensor data source	QuantAQ Cloud download
Reference data source	AQS API download

Sensor Information			
Manufacturer, model	SENSIT RAMP		
Device firmware version	190308_AQ_v9.30		
Sampling time interval	15-seconds		
Sensor serial numbers	RAM_01		
Issues encountered during deployment?	☑	See WI-RAM-Page 6 of this testing report	

FRM/FEM Information	
Manufacturer, model, designation	Teledyne Advanced Pollution Instrumentation T640X FEM
Sampling time interval	1-hour averaging
Date of calibration	As required by 40 CFR Part 58 and the Low Volume $PM_{2.5}$ and PM_{10} Networks QAPP maintained by WDNR
Date of flowrate verification check	Monthly as required by 40 CFR Part 58 Appendix A
Description, date(s) of maintenance activities	N/A

Time Series Plots

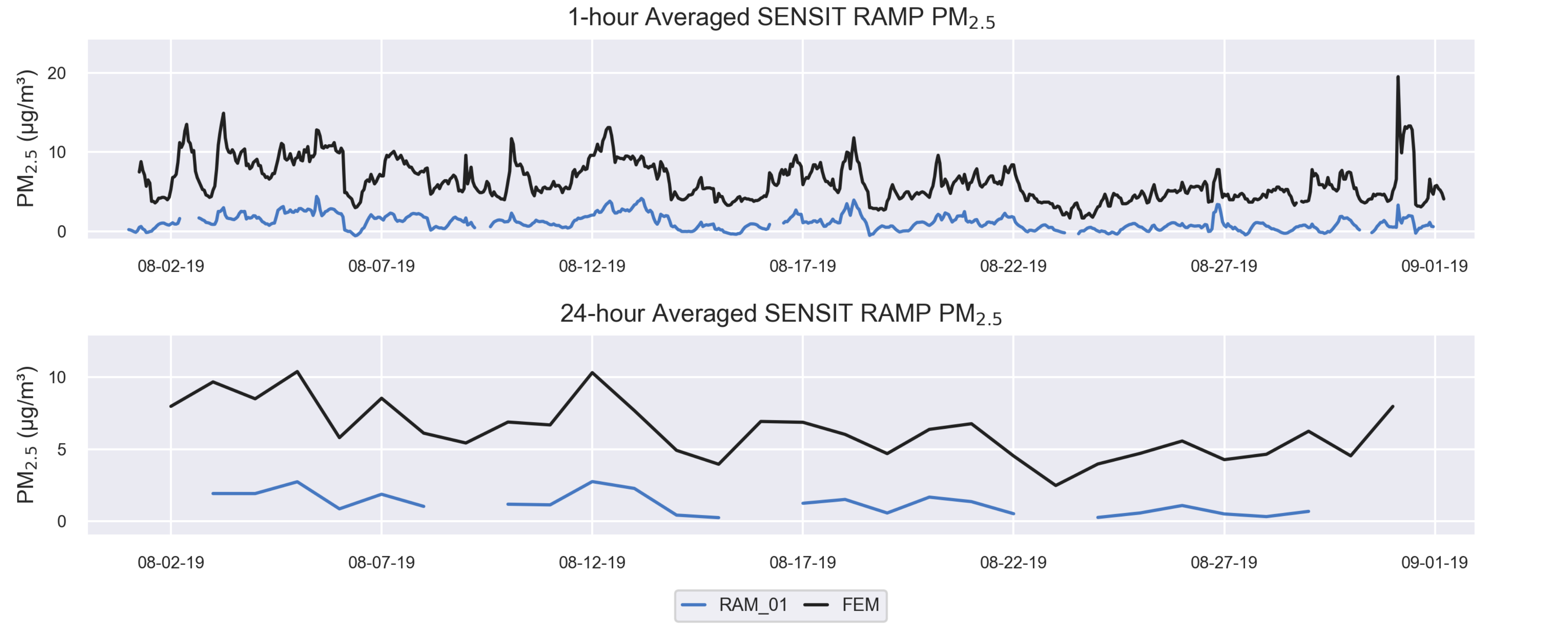

Scatter Plots: Comparison to FRM/FEM

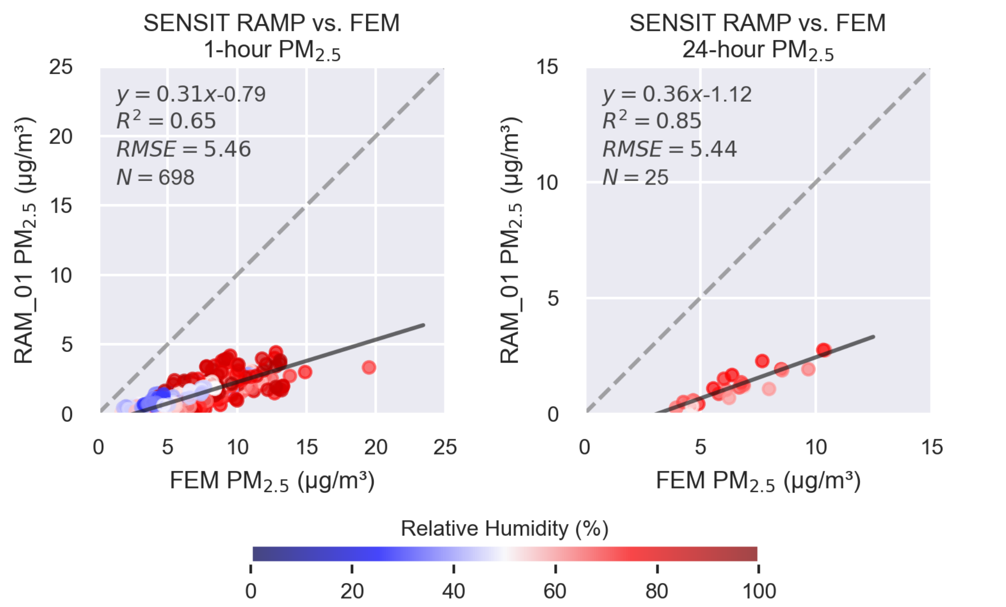

Range of FRM/FEM concentrations over duration of base test (µg/m³)	1.7-19.5 (1-hr),<br>2.5-10.4 (24-hr)
Number of 24-hr periods in FRM/FEM monitor measurements with a goal concentration ≥ 25 µg/m³	0

Performance Metrics

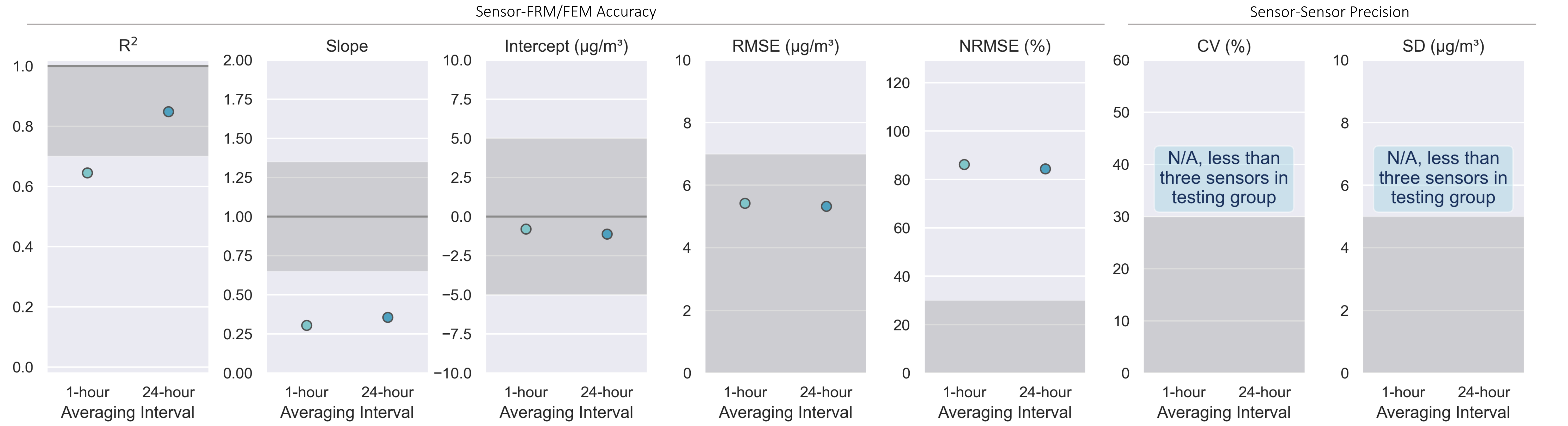

Meteorological Conditions During Deployment

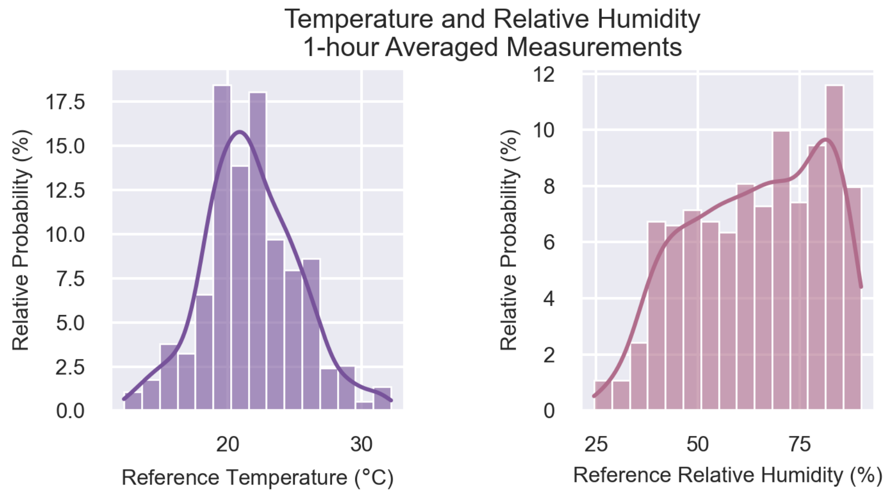

Number of 24-hr periods outside sensor manufacturer-listed temperature operational range (-20 to 50 °C)	0
Number of 24-hr periods outside sensor manufacturer-listed relative humidity operational range (no operational range specified)	-

Meteorological Influence

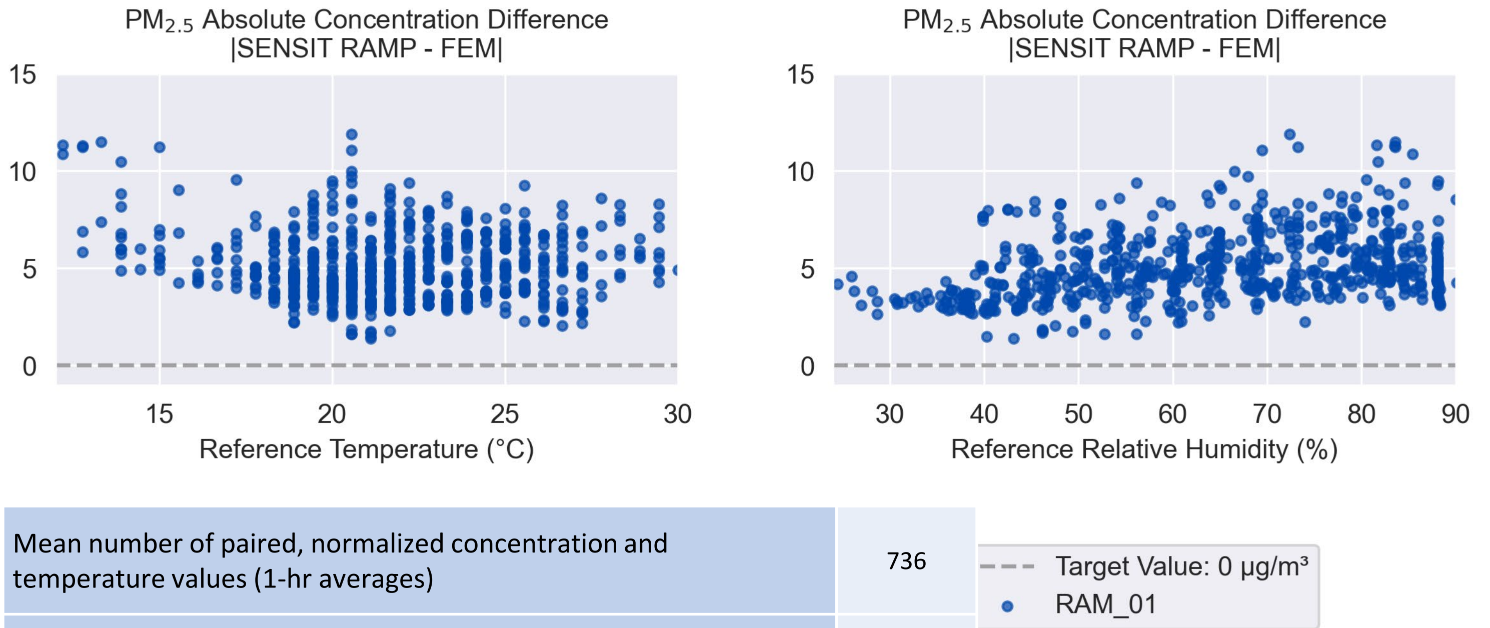

Mean number of paired, normalized concentration and temperature values (1-hr averages)	736
Mean number of paired, normalized concentration and relative humidity values (1-hr averages)	0

Testing Report - $PM_{2.5}$ Base Testing

SENSIT RAMP

This report reflects out-of-the-box performance

Initial Base Testing - Milwaukee, WI
U.S. Environmental Protection Agency
Office of Research and Development
PI: Clements.Andrea@epa.gov
919-541-1363
August 2019

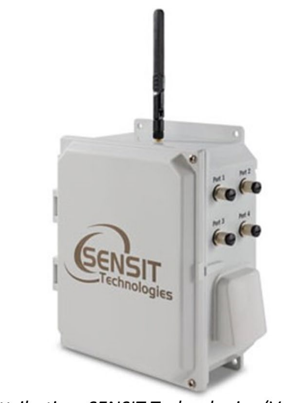

Image Attribution: SENSIT Technologies (Valparaiso, IN)

Tabular Statistics

Sensor-FRM/FEM Correlation

	Bias and Linearity						Data Quality			
	R^2		Slope		Intercept (µg/m³)		Uptime (%)		Number of paired sensor and FRM/FEM concentration values	
	1-Hour ○	24-Hour ●	1-Hour ○	24-Hour ○	1-Hour ●	24-Hour ●	1-Hour ●	24-Hour ●	1-Hour	24-Hour
Metric Target Range	≥ 0.70	≥ 0.70	1.0 ± 0.35	1.0 ± 0.35	-5 ≤ b ≤ 5	-5 ≤ b ≤ 5	75%*	75%*	-	-
Sensor RAM_01	0.65	0.85	0.31	0.36	-0.79	-1.12	95	84	698	25

	Error			
	RMSE (µg/m³)		NRMSE (%)	
	1-Hour ★	24-Hour ★	1-Hour ☆	24-Hour ☆
Metric Target Range	≤ 7.0	≤ 7.0	≤ 30.0	≤ 30.0
Deployment Value	5.4	5.3	86.2	84.4

Device-specific metrics (computed for each sensor in evaluation)
○○○ Metric value for none of devices tested falls within the target range
●○○ Metric value for one of devices tested falls within the target range
●●○ Metric value for two of devices tested falls within the target range
●●● Metric value for three of devices tested falls within the target range

Single-valued metrics (computed via entire evaluation dataset)
☆ Indicates that the metric value is not within the target range
★ Indicates that the metric value is within the target range

Sensor-Sensor Precision[1]

	Precision (between collocated sensors)				Data Quality	
	CV (%)		SD (µg/m³)		Number of concurrent sensor concentration pairs	
	1-Hour ☆	24-Hour ☆	1-Hour ☆	24-Hour ☆	1-Hour	24-Hour
Metric Target Range	≤ 30.0	≤ 30.0	≤ 5.0	≤ 5.0	-	-
Deployment Value	-	-	-	-	-	-

[1]Precision statistics are computed for evaluations with at least three collocated sensor units. Metric values are left blank for evaluations with two or fewer sensor units.

*This value is only a recommendation for ensuring data quality and is not included in the list of target values discussed in Section 4 of the Performance Testing Protocols, Metrics, and Target Values for Fine Particulate Matter Air Sensors document.

Testing Report - $PM_{2.5}$ Base Testing

SENSIT RAMP

This report reflects out-of-the-box performance

Initial Base Testing - Milwaukee, WI

U.S. Environmental Protection Agency
Office of Research and Development
PI: Clements.Andrea@epa.gov
919-541-1363

August 2019

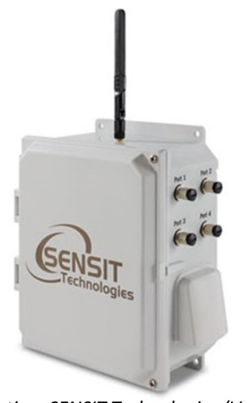

Image Attribution: SENSIT Technologies (Valparaiso, IN)

Sensor-FRM/FEM Scatter Plots

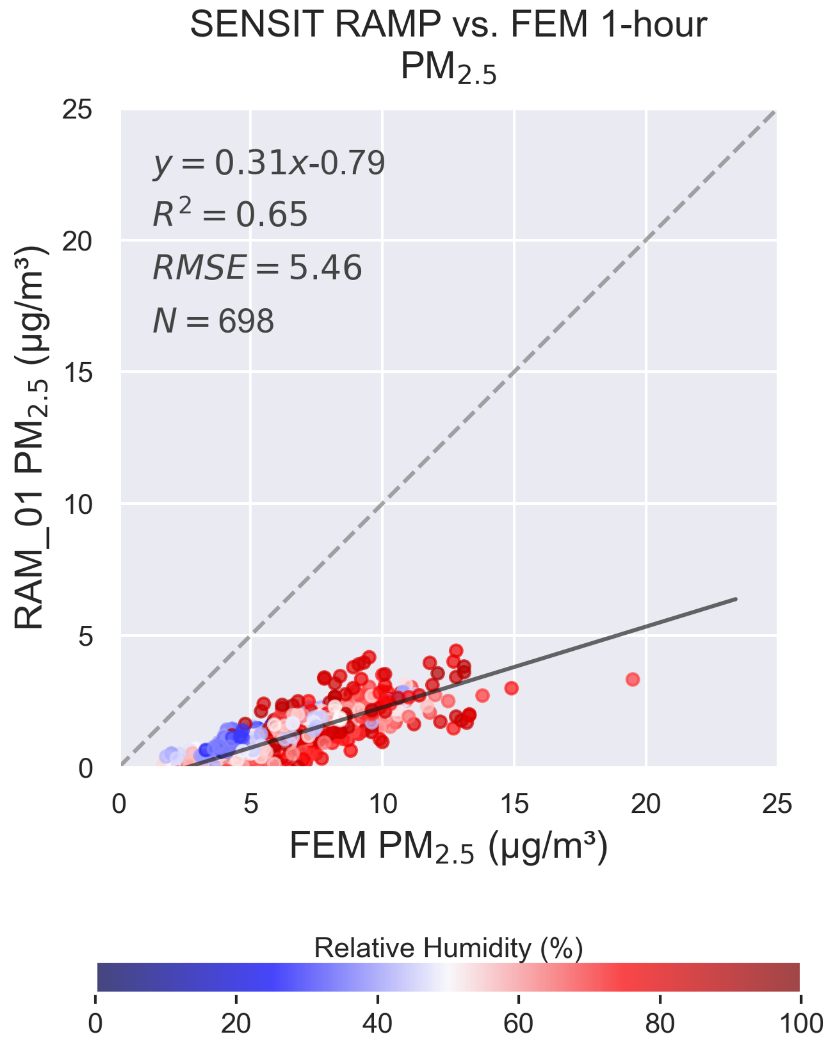

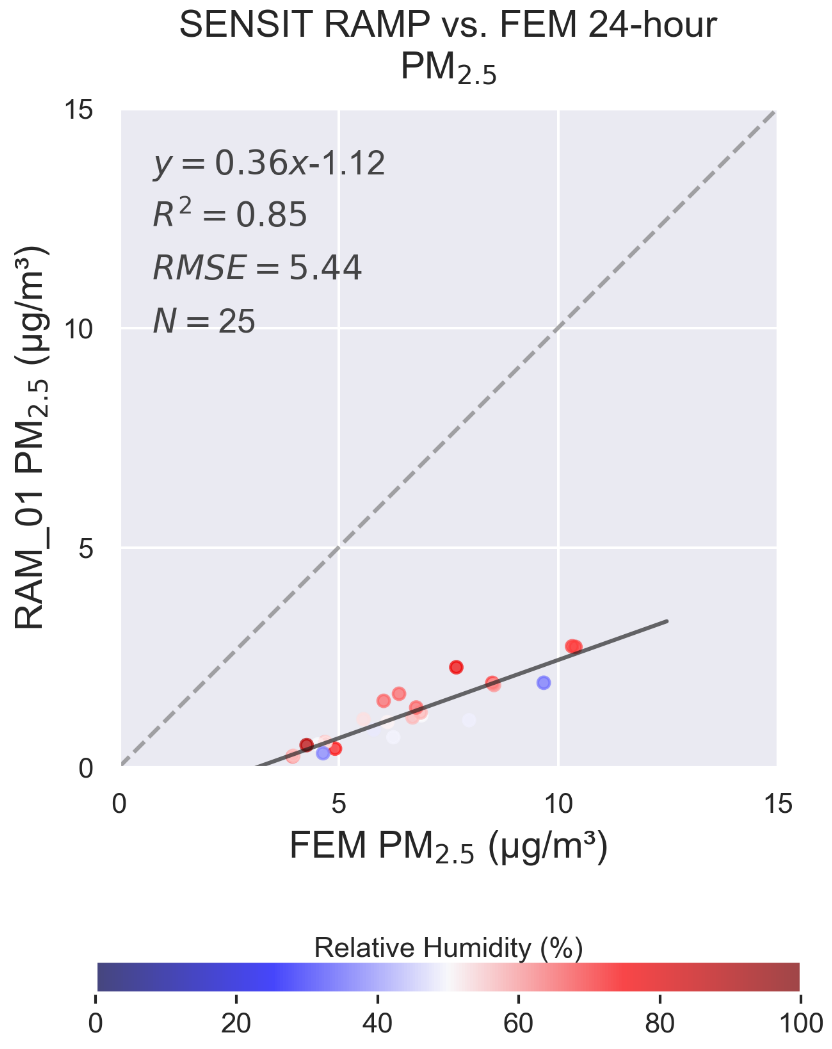

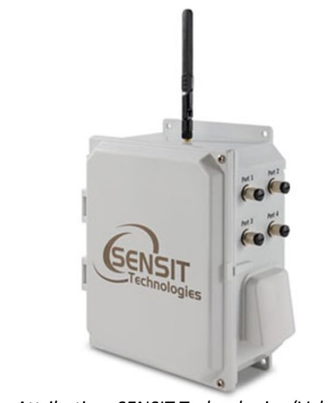

Image Attribution: SENSIT Technologies (Valparaiso, IN)

Supplemental Information

Abbreviations used in Supplemental Information

FRM	Federal Reference Method
FEM	Federal Equivalent Method
SOP	Standard Operating Procedure
QAPP	Quality Assurance Project Plan
QC	Quality Control

Supplemental Documentation	Attached	Description & URL or file path to documentation
Field observations and sensor data flags	☑	See WI-RAM-Page 6 of this testing report
Maintenance logs	☐	No logs recorded during testing
Standard operating procedure(s)	☐	U.S. EPA Office Of Research and Development SOP available upon request
Photos of equipment setup and testing	☑	See WI-RAM-Page 5 of this testing report
Product specifications sheet(s)	☑	SENSIT Technologies: RAMP Product Brochure
Product manual(s)	☑	SENSIT Technologies: RAMP Product Manual
Data storage and transmission method	☑	See WI-RAM-Page 6 of this testing report
Data correction approach	☑	See WI-RAM-Page 6 of this testing report
Issues encountered	☑	See WI-RAM-Page 6 of this testing report
Data analysis/correction scripts and version	☑	Averaging and processing of data, calculation of performance metrics, and generation of figures and other supplementary material for analysis were obtained using Python 3.9.7 with the packages sensortoolkit v0.8.3b2, pandas 1.3.5, NumPy 1.21.2, Matplotlib 3.5.0, statsmodels 0.13.0, and seaborn 0.11.2. All packages are available from the Python Package Index (PyPI) at https://pypi.org. The integrated development environment (IDE) Spyder 5.1.5 was used for scripting and data visualization. Version control for the Python base, packages, and IDE were all managed by conda 4.11.0.
Air Monitoring Station QAPP	☐	U.S. EPA Office Of Research and Development QAPP available upon request
Summary of FRM/FEM monitor QC checks	☑	See WI-RAM-Page 7 of this testing report
Manufacturer website for FRM/FEM monitor	☑	Teledyne API: T640/T640X Product website
FRM/FEM monitor manual	☑	Teledyne API: T640/T640X Product Manual
FRM/FEM monitor specifications sheet(s)	☑	Teledyne API: T640/T640X Specification Sheet
Other documents	☐	

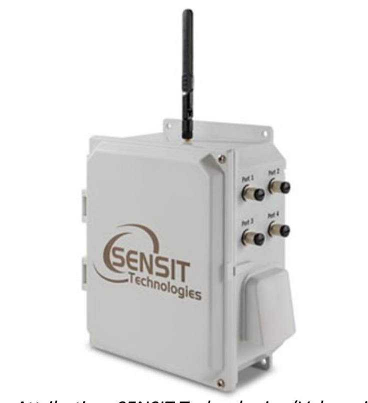

Image Attribution: SENSIT Technologies (Valparaiso, IN)

Supplemental Information: Photos of Testing Site and Equipment Setup

Site Description:

The urban site was located in the parking lot at the former WDNR Southeast Regional Headquarters located at 2300 N. Martin Luther King Blvd. Sample inlets are roughly 5 meters above ground level and 50 meters from the nearest road. Verified through annual WDNR audits, the site meets the requirements of *40 CFT 58, Appendices C, D, E and G.*

Figure 1: SENSIT RAMP sensor (indicated by red arrow) attached to metal railing atop the sampling shelter at the monitoring site.

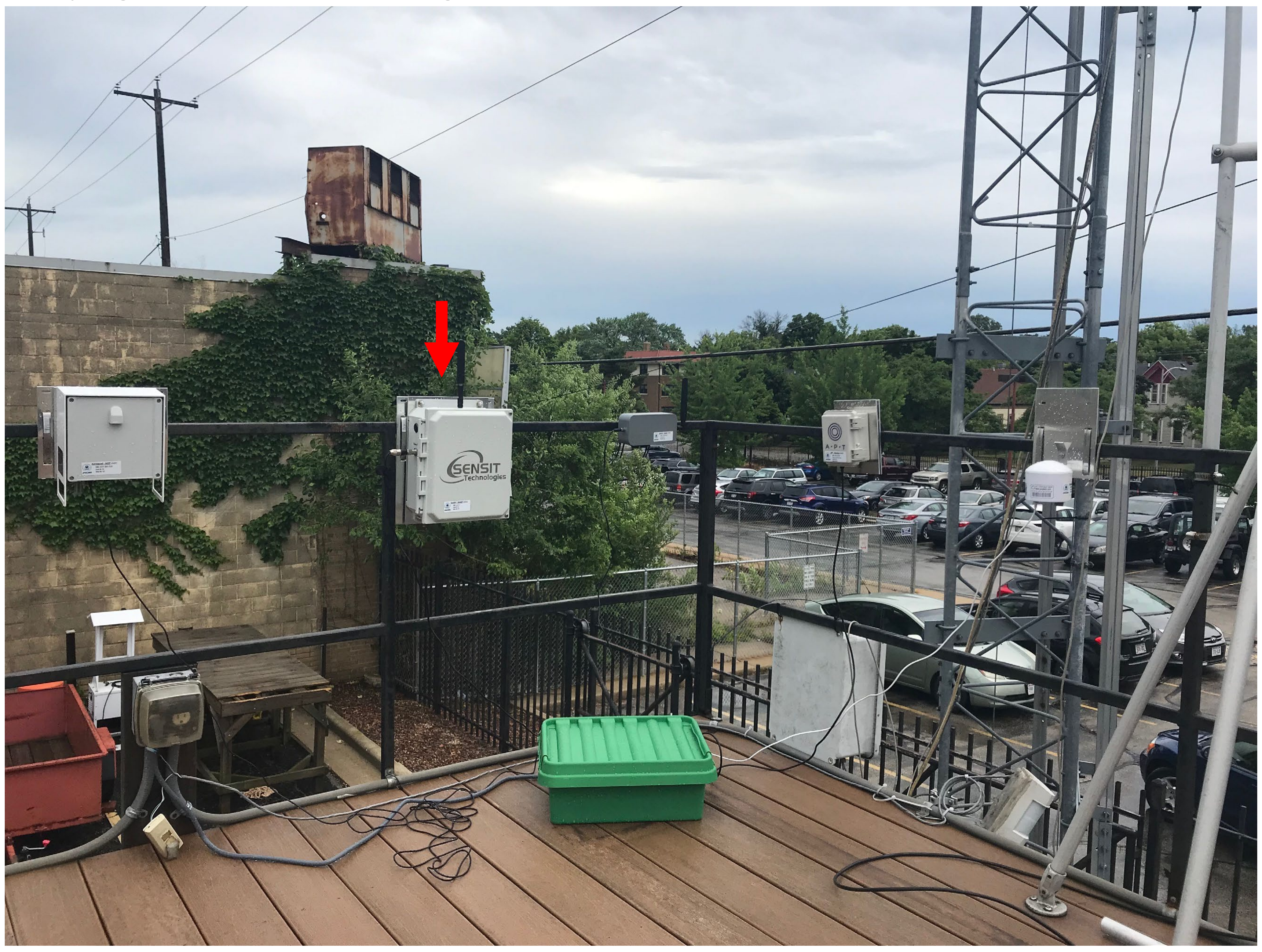

Figure 2: WDNR Monitoring Station

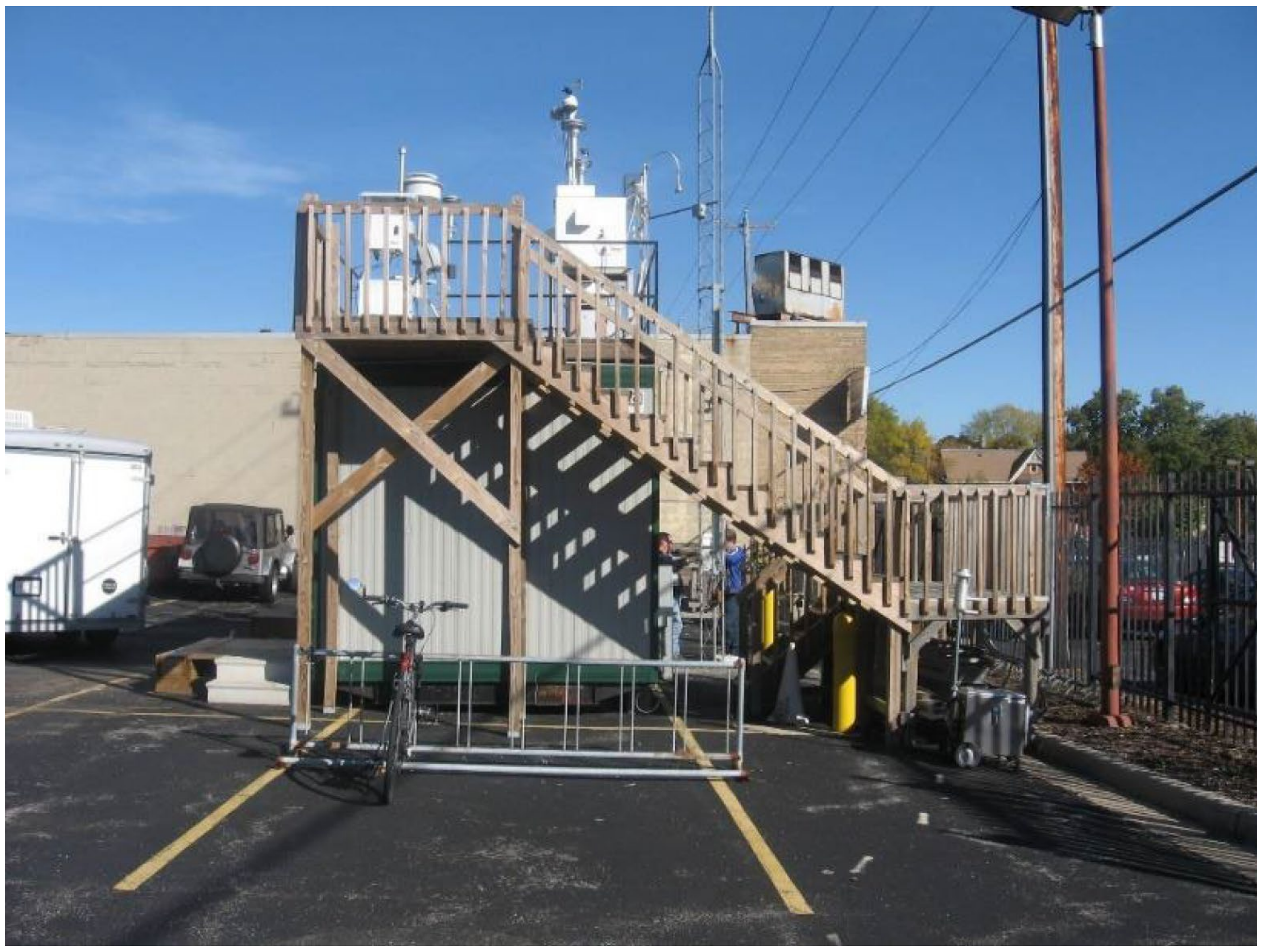

Initial Base Testing - Milwaukee, WI
U.S. Environmental Protection Agency
Office of Research and Development
PI: Clements.Andrea@epa.gov
919-541-1363
August 2019

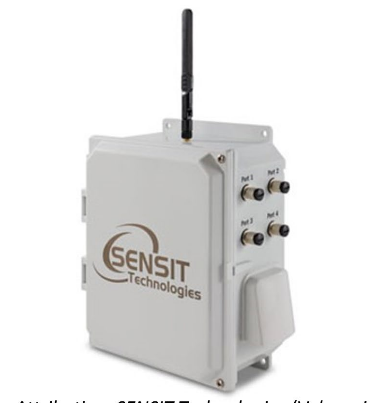

Supplemental Information: Data Storage, Correction Approach, and Issues Encountered

Data Storage and Transmission Method

The SENSIT RAMP was configured to record data at a 15-second sampling interval. Data are stored as daily text files (.txt format) on an onboard MicroSD card. Data files were obtained weekly via SD cards. Each field site operator was provided two labeled MicroSD cards for sensor units that they used to swap out each week. Data from the collected card was then read and processed off-site.

Data Correction Approach

SENSIT RAMP units were pre-configured by the manufacturer with a linear correction (i.e., concentration gain = 1.0 and offset = 0.0 µg m^{-3}). These presets reflect out-of-the box performance and were not modified by EPA prior or during testing.

After acquisition, the raw data was processed using the *sensortoolkit* python code library (v0.8.3b2). A continuous data set at the recorded sampling frequency was written to a .csv file. 1-hour and 24-hour averaged data sets were generated using a 75% completeness threshold and saved as separate .csv files. Outliers were NOT removed from data sets in order to assess "out-of-the-box" sensor performance.

Issues Encountered

Pre-deployment observations

- SENSIT RAMP units were received without documentation or manuals. After communicating the need to change default settings (logging interval and time zone) with the manufacturer, a draft user's manual and a USB cable were supplied. With the use of this USB cable, instrument settings could be changed, and real-time data could be logged using a serial communication software (CoolTerm, v.1.5.0). Because the sensor did not record data at the top of every minute, the RAMP was configured to record data at 15-second intervals so that the data could be averaged more closely to complete minutes. Prior to deployment, RAMP units were collocated in a bench-top evaluation to verify operational status and determine the extent of data invalidity (i.e., determine equilibration period) after an initial start-up event. The recorded response for parameters measured by the RAMP suggests that the gas sensors (CO, NO, NO_2, O_3) required approximately a 2-hour equilibration period, while the remaining sensors (temperature, relative humidity, particulate matter) did not require any equilibration period.

Field observations and sensor data flags

The following table contains data flags describing events that were encountered during the testing period. On 8/2/2019, a sampling interval abnormality was flagged and was associated with a brief period in which the sampling frequency changed from a constant, consecutive 15-second interval to a longer interval. For three samples, the interval between recorded values ranged from roughly 2 minutes to over 7 minutes. This event lasted less than 15 minutes, and following these abnormal intervals, the sampling frequency resumed the configured 15-second interval.

Start Time (UTC)	End Time (UTC)	Sensor Serial ID	Parameters Impacted	Flag
2019-08-02 17:27:00+00:00	2019-08-02 17:34:00+00:00	RAM_01	ALL	6.0-Sampling interval abnormality

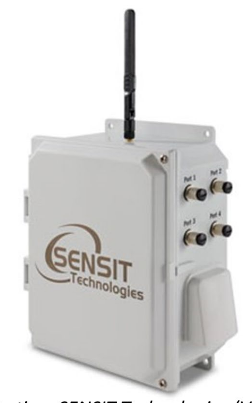

Image Attribution: SENSIT Technologies (Valparaiso, IN)

Description of Data Flags

AQS

The U.S. EPA's Air Quality System (AQS) is the Agency's primary ambient air monitoring data archive. A comprehensive list of data flags that are recorded alongside AQS data sets, referred to by U.S. EPA as 'qualifiers', can be found at the following link: https://aqs.epa.gov/aqsweb/documents/codetables/qualifiers.html

Invalidation of reference data: AQS qualifiers are organized by qualifier type, which indicates whether data logged alongside qualifier flags should be invalidated (set null). Qualifiers with type "Null Data Qualifier" are invalidated, and includes data logged during periods that coincide with QC checks (e.g., "BF-Precision/Zero/Span", "BJ- Operator Error", "BL - QA Audit", "AZ - QC Audit") among other events such as power outages. Data logged alongside qualifiers with type "Quality Assurance Qualifiers" are not invalidated and are included in this analysis (e.g., concentrations less than the federal MDL for the reference monitor "MD – Value less than MDL", QA reviewed values "Validated Value").

Data Flags Recorded During Testing

FRM/FEM Monitor	Timestamp (UTC)	Flag
Teledyne API T640X (Data acquired from AQS)	2019-08-28 18:00:00+0000 to 2019-08-28 19:00:00+0000	AX - Precision Check

Meteorological Instrument	Timestamp (UTC)	Flag
MetOne Temperature Monitor (Data acquired from AQS)	2019-08-07 16:00:00+0000 to 2019-08-07 17:00:00+0000	AZ - QC Audit
